# B‐DNA Structure and Stability: The Role of Nucleotide Composition and Order

**DOI:** 10.1002/open.202100231

**Published:** 2022-01-27

**Authors:** Celine Nieuwland, Trevor A. Hamlin, Célia Fonseca Guerra, Giampaolo Barone, F. Matthias Bickelhaupt

**Affiliations:** ^1^ Department of Theoretical Chemistry Amsterdam Institute of Molecular and Life Sciences (AIMMS) Amsterdam Center for Multiscale Modeling (ACMM) Vrije Universiteit Amsterdam De Boelelaan 1083 1081 HV Amsterdam (The Netherlands; ^2^ Leiden Institute of Chemistry Gorlaeus Laboratories Leiden University Einsteinweg 55 2300 CC Leiden (The Netherlands; ^3^ Dipartimento di Scienze e Tecnologie Biologiche, Chimiche e Farmaceutiche Università degli Studi di Palermo Viale delle Scienze, Edificio 17 90128 Palermo Italy; ^4^ Institute of Molecules and Materials Radboud University Nijmegen Heyendaalseweg 135 6525 AJ Nijmegen (The Netherlands

**Keywords:** activation strain model, density functional calculations, diagonal interactions, DNA structures, nucleotide composition

## Abstract

We have quantum chemically analyzed the influence of nucleotide composition and sequence (that is, order) on the stability of double‐stranded B‐DNA triplets in aqueous solution. To this end, we have investigated the structure and bonding of all 32 possible DNA duplexes with Watson–Crick base pairing, using dispersion‐corrected DFT at the BLYP‐D3(BJ)/TZ2P level and COSMO for simulating aqueous solvation. We find enhanced stabilities for duplexes possessing a higher GC base pair content. Our activation strain analyses unexpectedly identify the loss of stacking interactions within individual strands as a destabilizing factor in the duplex formation, in addition to the better‐known effects of partial desolvation. Furthermore, we show that the sequence‐dependent differences in the interaction energy for duplexes of the same overall base pair composition result from the so‐called “diagonal interactions” or “cross terms”. Whether cross terms are stabilizing or destabilizing depends on the nature of the electrostatic interaction between polar functional groups in the pertinent nucleobases.

## Graphical abstract

**All about that sequence**! Quantum chemical analyses reveal that the stability of B‐DNA triplets depends not only on the nucleotide composition, but also on the order in which the nucleotides occur. This sequence dependence in the duplex stability results primarily from attractive or repulsive cross terms between bases in adjacent Watson–Crick pairs.

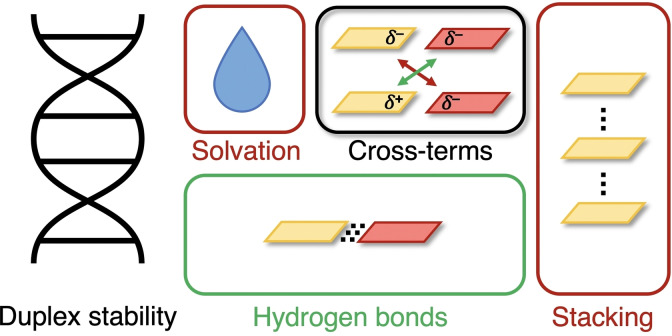

## Introduction

The genetic information encoded in the DNA of living organisms is the foundation of life.^[1]^ Therefore, understanding the dynamic geometry and stability of DNA represents a thriving field.^[2]^ By now it is well‐known that the structure of the double‐stranded helix is strongly affected by factors such as the nucleotide composition, solvation, the presence of counterions, and interaction with proteins and small molecules (e. g., drugs).^[3]^ Quantum chemical calculations are often employed to assess and quantify these variables individually and pinpoint their influence on the structure and stability of DNA molecules.^[4]^ Many of these studies report analyses of simplified DNA model systems. Naturally, one begins with the study of structure and stability of the Watson–Crick DNA base pairs[^4a^, ^4b^, ^4c^, ^4d^] adenine–thymine (A−T) and guanine−cytosine (G−C), mismatches,[^4a^, ^4c^, ^4d^] DNA single strands,[^4e^, ^4f^] or double‐stranded doublets (i. e., two stacked base pairs).[^4c^, ^4d^, ^4e^, ^4f^] The impact and importance of this work cannot be overstated, but quantum chemical computational studies of more realistic DNA models are necessary to see how these fundamental observations hold when all structural and environmental components are combined.^[5]^ Density Functional Theory (DFT) modeling of triplet DNA helices of all‐AT or all‐GC base pairs with a neutralized sugar‐phosphate backbone, immersed in a continuum type dielectric medium, provides geometries in agreement with the experimental B‐DNA geometry and with accurate binding energies.[^5a^, ^5f^] More recently, this work was extended towards the double‐stranded DNA pentamers d(A)_5_⋅d(T)_5_ and d(G)_5_⋅d(C)_5_ and the duplex Dickerson Dodecamer sequence d(CGCGAATTCGCG).^[5b]^ Different structural behavior and stabilities were observed for the d(A)_5_⋅d(T)_5_ and d(G)_5_⋅d(C)_5_ helices, which the authors ascribed to the presence of an extra hydrogen bond between the G−C base pairs. Nucleotide composition dependency was also observed in previous work of our group that showed that the stability of double‐stranded DNA doublets increases with the G−C base‐pair content, thus with the number of hydrogen bonds.^[4f]^ In addition, an effect of the nucleotide sequence (i. e., order) on the doublet stability was observed. This indicates that there are sequence‐dependent interactions that need to be understood to accurately predict the stabilities of nucleic acids. The reason for this is that the DNA double helix comprises not only hydrogen bonds between complementary bases, but also interactions between stacked bases and between bases that are diagonal to each other, that is, on opposite sides and in different layers.^[4d]^

**Figure 1:**
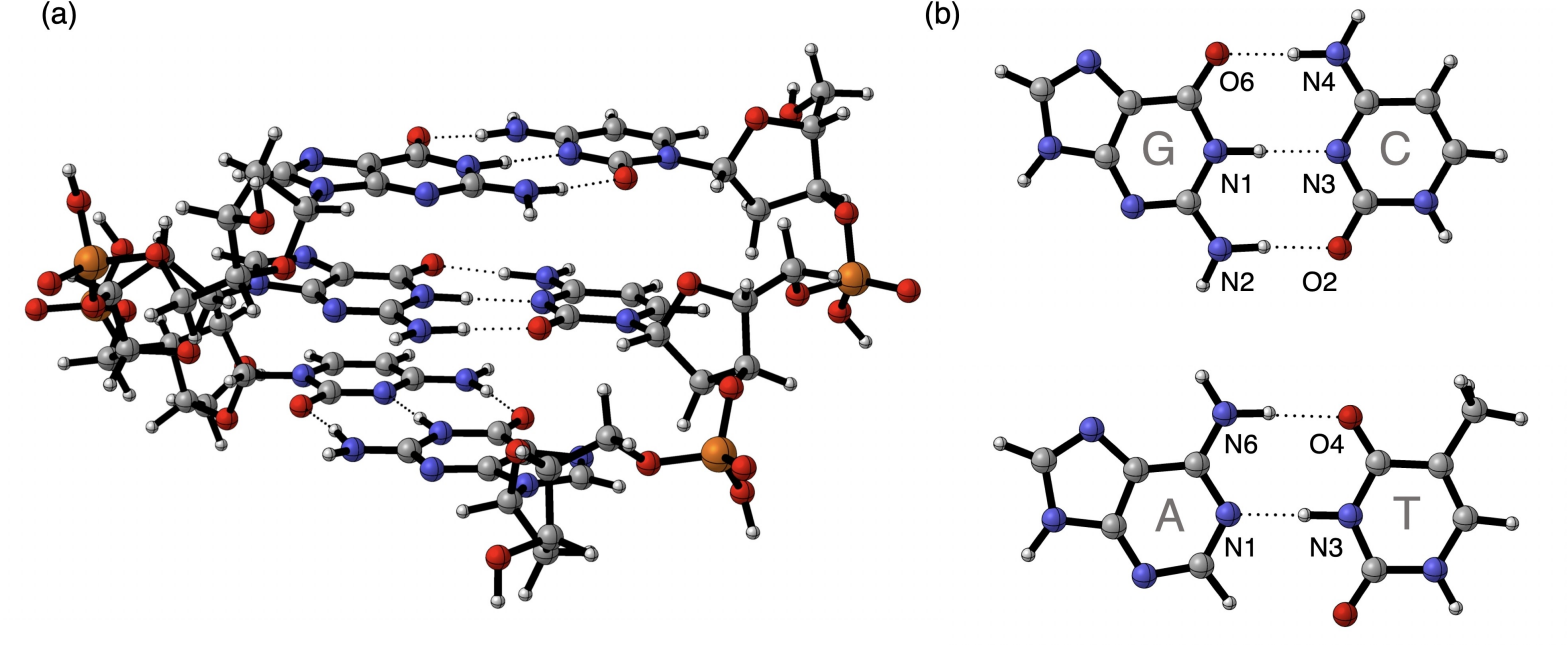
**(a)** Geometry of d(CGG)^H+^, as an example of the duplex B‐DNA triplets studied in this work, optimized at the BLYP‐D3(BJ)/TZ2P level using COSMO to simulate solvation in water. Hydrogen‐bond interactions are depicted by dotted lines. Color code in ball‐and‐stick structures: H – white; C – grey; N – blue; O – red; P – yellow. **(b)** Watson–Crick DNA base pairs: guanine (G) – cytosine (C) and adenine (A) – thymine (T) with labeling of the hydrogen‐bond front atoms.

In this work, we report an extensive analysis of intra‐DNA interactions using dispersion‐corrected DFT (DFT‐D) computations of more realistic DNA structures: trideoxyribonucleoside diphosphate double strands (i. e., triplets, see Figure 1a). We consider double‐stranded B‐DNA triplets with a charged backbone (d(DNA)^−4^) and a backbone neutralized by either H^+^ (d(DNA)^H+^) or Na^+^ (d(DNA)^Na+^) counterions. All 32 possible sequences of duplex DNA triplets were examined, considering Watson–Crick base pairing (Figure 1b). In the example of Figure 1a, the d(CGG)^H+^ duplex consists of the (CGG)^H+^ and (CCG)^H+^ complementary single strands with a CGG and CCG sequence in 5’‐to‐3’ notation, respectively. This definition for the single and double strands differs slightly from our previous work,^[4f]^ but we adjusted the notation to arrive at a more clear and compact notation for the triplets in this work.

First, we justify the accuracy of the DFT description of the DNA model systems by comparison of calculated geometrical parameters with available experimental X‐ray data, after which the effect of the nucleotide composition, backbone neutralization, and nucleotide order on the DNA stability is examined using the activation strain model (ASM)^[6]^ of reactivity and bonding, in combination with our canonical energy decomposition analysis (EDA).^[7]^ Finally, a structural decomposition of the DNA molecules allows us to delineate the importance of the interactions between structural components to the overall stability of the double helix.

Detailed analyses of the structure and stability of single‐ and double‐stranded DNA triplets is relevant, among others, because the 64 possible sequences of three bases in a single DNA (or RNA) strand constitute the codons, that is, the letters or units of the genetic code that direct protein synthesis (note that the 64 possible single strands can form 32 unique Watson–Crick duplexes).^[1]^ Furthermore, understanding the physical principles of DNA molecules might provide new insights into gene expression and genome stability. This work demonstrates that a detailed analysis of structural and stability properties of biological macromolecules of significant size is nowadays feasible using highly accurate quantum chemical methods and leads to new insights.

## Results and Discussion

### Validation of the Computational Method

All computations were performed at the BLYP‐D3(BJ)/TZ2P level of theory using the Conductor‐like Screening Model (COSMO) to simulate aqueous solvation (see also the Computational Details section). This level of theory has been proven to be accurate for our purpose, both in previous reports as well as in additional performance tests carried out in the present work.

Various computational studies on DNA‐based systems have demonstrated that BLYP‐D3(BJ) yields reliable geometries and (hydrogen) bond energy trends that are in line with experimental data.[^4c^, ^4f^, ^8^] BLYP‐D3(BJ) furthermore outperforms more recently developed hybrid functionals, such as M06‐2X, in terms of the accuracy/cost ratio in the description of non‐covalent interactions.^[9]^

**Scheme 1:**
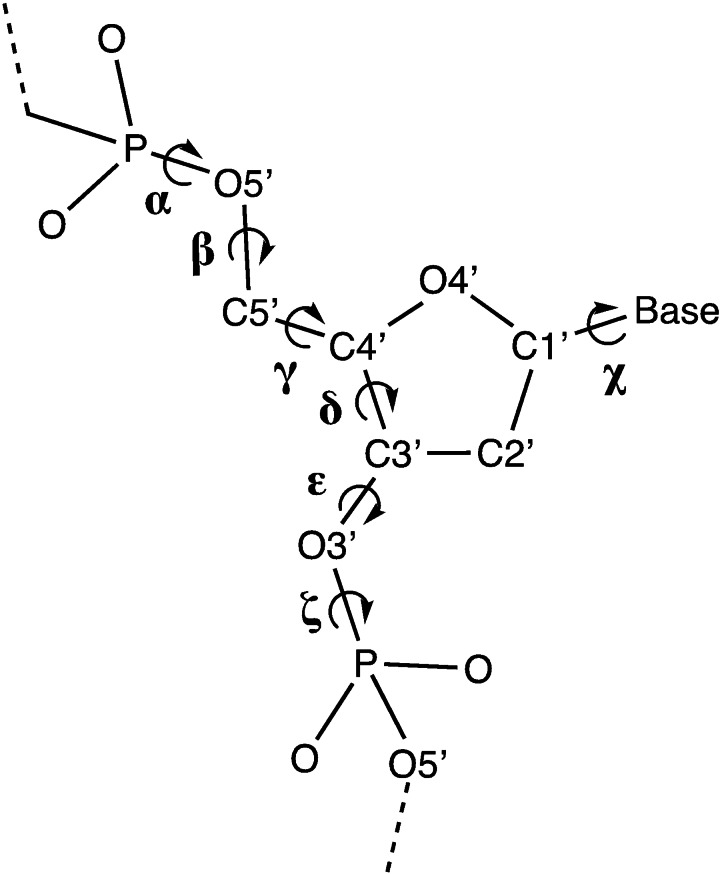
Definition of the sugar‐phosphate backbone torsion angles in B‐DNA structures.

Herein, we have confirmed the reliability of our COSMO(H_2_O)‐BLYP‐D3(BJ)/TZ2P description for studying the 32 d(DNA)^4−^ model double‐stranded triplets in the present work by comparing the computed geometries with available experimental X‐ray diffraction data.^[10]^ In our d(DNA)^4−^ duplex model systems, the backbone neutralization is omitted, so that the overall structure has a net charge of −4. The optimized geometries of the d(DNA)^4−^ triplets were verified by comparison of the mean calculated sugar‐phosphate backbone torsion angles (Scheme 1) to experimental mean values from B‐DNA crystal structures available in the literature.^[10]^ The mean calculated values are summarized in Table 1 (for details, including bond distances, see Tables S1 and S2 in the Supporting Information). In all cases, the obtained geometries are in very good agreement with the experimental data. Although in some cases the calculated mean backbone torsion angles of our relatively small dataset vary from the experimental mean values (up to 22° for the β angle), all calculated mean torsion angles fall well within the experimentally observed range of backbone torsion angles of B‐DNA.^[10]^ The use of the COSMO(H_2_O)‐BLYP‐D3(BJ)/TZ2P level of theory in our calculations is therefore justified since it is known from previous work that counterions, as are present under biological conditions and in our d(DNA)^H+^ and d(DNA)^Na+^ model systems, will further improve the agreement between the computed and experimental DNA geometries, in particular, backbone torsion angles.[^5b^, ^5f^]

**Table 1:** Mean backbone torsion angles (in °; standard deviations in parentheses) of our 32 d(DNA)^4−^ structures from computation^[a]^ compared to experiment.^[b]^

	Computation	Experiment
alpha (α)	−66 (2)	−62 (15)
beta (β)	−162 (9)	−184 (9)
gamma (γ)	50 (2)	48 (11)
delta (δ)	141 (3)	128 (13)
epsilon (ϵ)	166 (5)	184 (11)
zeta (ζ)	−92 (3)	−95 (10)
chi (χ)	−107 (4)	−102 (14)

[a] Our computations, performed at the COSMO(H_2_O)‐BLYP‐D3(BJ)/TZ2P level. See Scheme 1 for the definition of torsion angles; see the Supporting Information for Cartesian coordinates; [b] from X‐ray crystal structures (resolution better than 1.9 Å) of 10 to 12 nucleotides long B‐DNA oligodeoxynucleotides containing AT and GC base pair combinations.^[10]^

### B‐DNA Stability: Effect of Nucleotide Composition

We have explored how the stability of B‐DNA triplets depends on (i) the nucleotide composition, (ii) backbone neutralization by counterions, and (iii) the order in which the nucleotides occur in a strand by computing and analyzing the formation energy (▵*E*) of the double helix from the two complementary single strands which is defined in Equation 1.

(1) $$\Delta E=E{}_{\mathrm{ds},\mathrm{aq}}-E{}_{\mathrm{ss}1,\mathrm{aq}}-E{}_{\mathrm{ss}2,\mathrm{aq}}$$

Here, *E*_ds,aq_ denotes the energy of the solvated DNA double strand (ds) in its equilibrium geometry. *E*_ss1,aq_ and *E*_ss2,aq_ correspond to the energies of the two separate solvated single strands (ss), each one in its own equilibrium geometry. The dissociated single strands are not rigid and can adopt a range of flexible orientations in solution. However, our analysis involves the initial stage after dissociation of the DNA duplex into the two constituent single strands. These separated single strands present a somewhat condensed structure that maximizes the intramolecular interactions (see Figure S1 for a graphical example).

**Figure 2:**
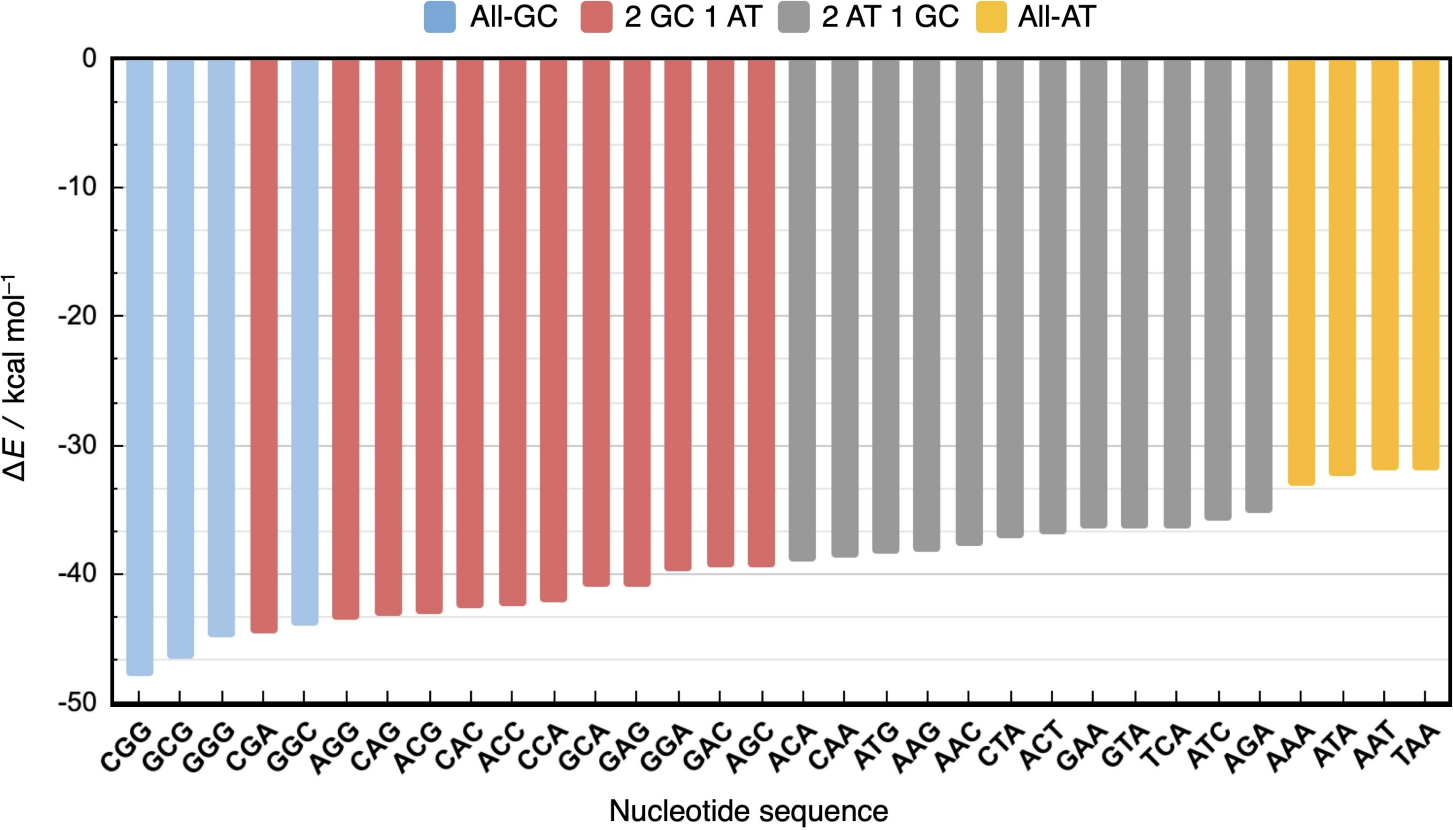
Formation energy ▵*E* (in kcal mol^−1^) of the 32 double‐stranded DNA triplets d(DNA)^H+^ (backbone neutralized by H^+^) from two complementary single strands, computed at the BLYP‐D3(BJ)/TZ2P level, using COSMO to simulate solvation in water. The colors indicate sequences with the same relative number of GC and AT base pairs.

Figure 2 shows our computed formation energies of the 32 d(DNA)^H+^ double strands involving non‐ionized (neutral) phosphate groups. Numerical data can be found in Table S3 in the Supporting Information.

First, we examine the effect of the nucleotide composition, that is, the number of GC versus AT base pairs in the duplexes. The formation of all 32 unique duplexes from their complementary single strands is net stabilizing with formation energies ▵*E* ranging from −32 kcal mol^−1^ for the weakest duplex, d(TAA)^H+^, to −48 kcal mol^−1^ for the most stable duplex, d(CGG)^H+^. The stabilization of ▵*E* increases nearly proportionally as a function of the number of GC pairs. This trend is caused by the fact that the triply hydrogen‐bonded G−C base pair is more stable than the only doubly hydrogen‐bonded A−T base pair, in line with previous work.[^4f^, ^5b^] Note, however, that for each nucleotide composition of the strands or, equivalently, Watson–Crick pair composition of the duplex (i. e., 0–3 G−C base pairs), there is still a substantial variation in the stabilities, depending on the order in which the base pairs occur, which can be as large as 5 kcal mol^−1^ in the case of duplexes containing two GC and one AT pair (see below).

The overall Gibbs free energy of formation, ▵*G*, is less negative than the electronic energy of formation, ▵*E*, mainly due to the destabilizing contributions of the zero‐point vibrational energy, ▵ZPE, and, predominantly, the entropy, T▵*S* (see below). The entropic penalty originates mainly from the increased overall rigidity when the relatively flexible single strands assemble to form the more rigid duplex. Importantly, the formation of duplexes remains spontaneous at room temperature (i. e., ▵*G*<0) in all 32 cases. Furthermore, the effect of the thermodynamic contributions (i. e., ▵*G*–▵*E*) is estimated to be relatively constant, which suggests that trends in ▵*E* are hardly affected and, by and large, carry over to ▵*G*.

We have estimated this by explicitly carrying out the extremely costly computation of ▵*G* for the most stable and for the least stable duplexes with a charged backbone, d(CGG)^4−^ (187 atoms) and d(TAA)^4−^ (190 atoms), respectively. The duplexes d(DNA)^4−^ with charged backbones have been chosen because they are less stable than d(DNA)^H+^ and d(DNA)^Na+^ (see below), and therefore are in theory more likely to become unbound due to thermodynamic corrections. Thus, we have performed frequency calculations for d(CGG)^4−^, d(TAA)^4−^, as well as for the respective single strands, to obtain the vibrational states in the pertinent partition functions. d(CGG)^4−^ is formed from the two complementary single strands (CGG)^2−^ and (CCG)^2−^, whereas d(TAA)^4−^ is constructed from the strands (TAA)^2−^ and (TTA)^2−^. The calculated ▵*G* values are −22.3 and −6.9 kcal mol^−1^ for d(CGG)^4−^ and d(TAA)^4−^, respectively. The corresponding ▵*E* values are found to be −44.8 and −28.8 kcal mol^−1^ (see Supporting Information Table S3). Thus, there is a destabilizing and relatively constant ▵ZPE and entropic T▵*S* contribution in the double‐helix formation of 22 to 23 kcal mol^−1^, that applies to all DNA triplets. Therefore, the sequence‐dependent trend in duplex stability primarily depends on the electronic energy of formation, ▵*E*.

### B‐DNA Stability: Effect of Counterions

Our analyses focus mainly on neutral DNA triplets d(DNA)^H+^ with non‐ionized phosphate groups (see for example, Figure 2) because, as further explorations show, they are computationally more stable than ionized (anionic) triplets or triplets with sodium counterions while displaying near‐, although not fully, identical trends in stability as a function of their sequences. Thus, DNA model duplexes with anionic sugar‐phosphate backbones (d(DNA)^4−^), those with neutral sugar‐phosphate backbones that we mainly focus on (d(DNA)^H+^, that is, neutralized with H^+^ counter ions), and those with sugar‐phosphate backbones neutralized by Na^+^ counter ions (d(DNA)^Na+^) show very similar trends in stability as function of the nucleotide composition, as can be seen by comparing Figure 2 and Supporting Information Figures S2 and S3.

Although backbone neutralization has little effect on stability trends as a function of base sequence, it has an important impact on the *absolute* B‐DNA stability. The formation energy ▵*E* becomes increasingly stabilizing along the order d(DNA)^4−^<d(DNA)^H+^<d(DNA)^Na+^. For example, along d(CGG)^4−^, d(CGG)^H+^, and d(CGG)^Na+^, the formation energy ▵*E* becomes increasingly stabilizing from −44.8 to −47.9 to −51.8 kcal mol^−1^, respectively (see Table S3). The reason for the enhanced stability upon backbone neutralization is, not unexpectedly, a more favorable electrostatic interaction (see the difference in ▵*V*_elstat_ of d(CGG)^H+^ versus d(CGG)^4−^ in Table S4).

### B‐DNA Stability: Effect of Nucleotide Order

As pointed out earlier, the stability of a duplex does not only depend on the base pair composition but also on the order in which the base pairs occur. In the following part, we show that sequence‐dependent variations in the stability of the duplex assembling from two complementary single strands arise mainly from three competing terms: (i) the desolvation; (ii) the deformation strain; and (iii) diagonal interactions, that is, cross terms. The meaning and implications of these factors will become clear in a moment, but the individual terms have a counteracting effect in such a way that the most stable DNA sequence is obtained at the optimum compromise of these terms. We first identify the general trends in these three terms, after which we explain these trends by an in‐depth analysis of each term. The nucleotide order effects are subtle and sometimes hard to foresee. Nevertheless, we can provide some useful guidelines for predicting the stabilities of oligonucleotides at the end of this work.

**Figure 3:**
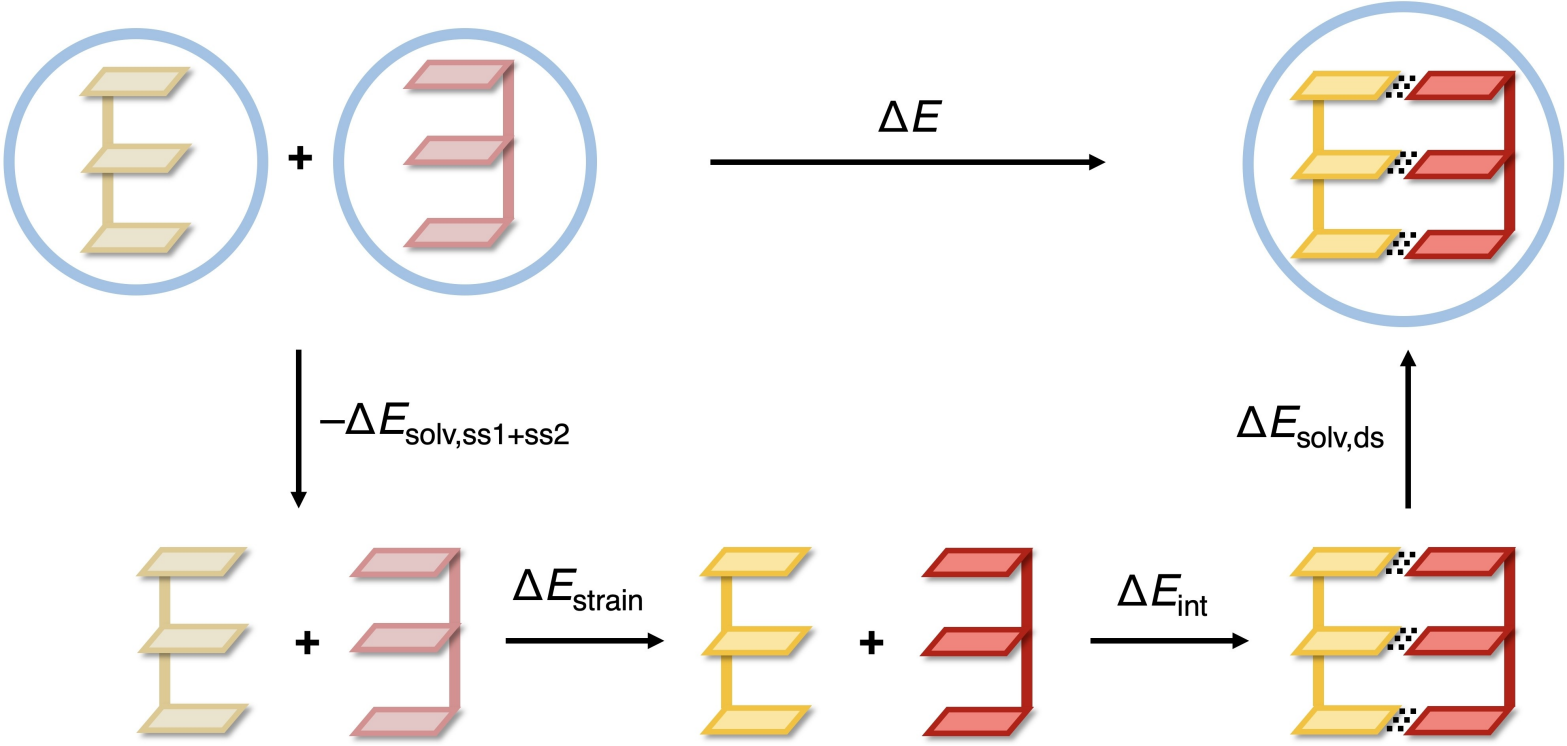
Partitioning of the formation energy (▵*E*) of the DNA duplex assembling from two complementary single strands (blue circles represent aqueous solvent medium).

To examine the effect of the nucleotide order on the duplex stability, let us focus on the two extreme cases in Figure 2, that is, the all‐GC (blue) and all‐AT (yellow) d(DNA)^H+^ duplexes. Among the former, d(CGG)^H+^ is the most stable duplex, followed by d(GCG)^H+^, d(GGG)^H+^, and d(GGC)^H+^. Among the latter, that is, the all‐AT duplexes, d(AAA)^H+^ is the most stable sequence, followed by d(ATA)^H+^, d(AAT)^H+^, and d(TAA)^H+^, respectively. To pinpoint the origin of the varying stability among these all‐GC and AT sequences, the duplex formation energy, ▵*E*, was partitioned using the activation strain model (ASM)^[6]^ as formulated by Equation (2) and illustrated by Figure 3.

(2) $$\Delta E=-\Delta E{}_{\mathrm{solv},\mathrm{ss}1+\mathrm{ss}2}+\Delta E{}_{\mathrm{strain}}+\Delta E{}_{\mathrm{int}}+\Delta E{}_{\mathrm{solv},\mathrm{ds}}$$

The desolvation energy (i. e., the negative of the solvation energy) of the two complementary single strands (−▵*E*_solv,ss1+ss2_) and the solvation energy of the duplex (▵*E*_solv,ds_) are computed by taking the energy difference going from the solvated species in their solution‐phase equilibrium geometry to the same species in the gas phase but still in their solution‐phase equilibrium geometry (see Equations (3) and 4).

(3) $$-\Delta E{}_{\mathrm{solv},\mathrm{ss}1+\mathrm{ss}2}=E{}_{\mathrm{ss}1,\mathrm{gas}}+E{}_{\mathrm{ss}2,\mathrm{gas}}-E{}_{\mathrm{ss}1,\mathrm{aq}}-E{}_{\mathrm{ss}2,\mathrm{aq}}$$

(4) $$\Delta E{}_{\mathrm{solv},\mathrm{ds}}=E{}_{\mathrm{ds},\mathrm{aq}}-E{}_{\mathrm{ds},\mathrm{gas}}$$

The subscript ‘aq’ refers to computations in aqueous solution whereas the subscript ‘gas’ refers to gas‐phase single‐point calculations on the solution‐phase equilibrium geometry. Thus, *E*_ss1,gas_, *E*_ss2,gas_, and *E*_ds,gas_ denote the gas‐phase energies of the two single DNA strands and the DNA duplex, each in their equilibrium geometry in aqueous solution.

The strain energy ▵*E*_strain_ is the energy required to deform the two complementary single strands, in the gas phase, from their equilibrium geometry in aqueous solution, with the energy *E*_ss1,gas_+*E*_ss2,gas_, to the structure they adopt in the DNA duplex in its equilibrium geometry in aqueous solution, with the energy *E*_ss1’,gas_+*E*_ss2’,gas_ (Equation 5).

(5) $$\Delta E{}_{\mathrm{strain}}=E{}_{\mathrm{ss}1{}^{'},\mathrm{gas}}+E{}_{\mathrm{ss}2{}^{'},\mathrm{gas}}-E{}_{\mathrm{ss}1,\mathrm{gas}}-E{}_{\mathrm{ss}2,\mathrm{gas}}$$

The interaction energy ▵*E*_int_ corresponds to the net stabilization associated with combining the two deformed single strands, again in the absence of any solvent, that is, with an energy of *E*_ss1’,gas_+*E*_ss2’,gas_, to form the double helix, with an energy of *E*_ds,gas_ (Equation 6).

(6) $$\Delta E{}_{\mathrm{int}}=E{}_{\mathrm{ds},\mathrm{gas}}-E{}_{\mathrm{ss}1{}^{'},\mathrm{gas}}-E{}_{\mathrm{ss}2{}^{'},\mathrm{gas}}$$

Note that in our energy decomposition as outlined in Figure 3 and Equation (2), in particular, the interaction energy ▵*E*_int_, is based on charge‐neutralized DNA duplexes with H^+^ counterions (d(DNA)^H+^) that provide stabilities, and trends therein, nicely match those occurring under physiological conditions, that is, in the presence of sodium counterions (see above). Importantly, our analysis is free of spurious Coulomb repulsion that would occur in the gas phase for non‐neutralized d(DNA)^4−^ model systems (compare ▵*V*_elstat_ for d(CGG)^H+^ and d(CGG)^4−^ in Table S4).

The partitioning of the formation energy ▵*E* is reported in Table 2 for the all‐GC and all‐AT d(DNA)^H+^ sequences (see Figure S4 for a graphical representation). The magnitudes of the energy terms are larger for the GC sequences than for the AT sequences, which can be attributed to the presence of additional hydrogen bonds in the former. Also, for some energy terms, we observe a more pronounced sequence‐dependent variation for the GC sequences (see below). From Table 2, it follows that, in all cases, ▵*E* is the result of a large, stabilizing interaction energy, counteracted by smaller, but still significant, destabilizing contributions from deformation strain and solvent effects.

**Table 2:** Partitioning of the formation energy ▵*E* (in kcal mol^−1^) for the d(DNA)^H+^ duplex assembling from two complementary single strands for the all‐GC and all‐AT sequences.^[a]^

d(DNA)^H+^	Δ*E*	−Δ*E*_solv,ss1+ss2_	Δ*E*_strain_	Δ*E*_int_	Δ*E*_solv,ds_	Δ*E*_solv,ds_–Δ*E*_solv,ss1+ss2_
d(CGG)^H+^	−47.9	161.8	19.1	−118.8	−110.0	51.8
d(GCG)^H+^	−46.6	146.4	16.6	−101.9	−107.7	38.7
d(GGG)^H+^	−44.9	168.7	21.9	−117.3	−118.2	50.5
d(GGC)^H+^	−44.0	153.3	17.8	−101.5	−113.6	39.7
						
d(AAA)^H+^	−33.2	122.9	7.8	−64.6	−99.3	23.6
d(ATA)^H+^	−32.4	121.5	5.3	−59.4	−99.8	21.7
d(AAT)^H+^	−32.0	121.0	7.5	−60.8	−99.7	21.3
d(TAA)^H+^	−32.0	123.1	7.2	−63.1	−99.1	24.0

[a] Computed at the BLYP‐D3(BJ)/TZ2P level using COSMO to simulate solvation in water. See also Equation (2).

The ▵*E*_strain_ term varies only slightly for the different d(DNA)^H+^ sequences. The least destabilizing strain energy is observed for the sequences d(GCG)^H+^ and d(ATA)^H+^. This can be ascribed to the fact that the associated single strands are characterized by an alternating arrangement of purine and the smaller pyrimidine bases which leads to less stabilizing internal stacking interactions than in the case of single strands involving stacks of two or three purines, for example, in the case of d(CGG)^H+^ (see Figure 1a) and d(AAA)^H+^, that go with larger mutual surface overlap and thus stronger stacking interactions. Consequently, the geometrical deformation required to form the double helix in the case of d(GCG)^H+^ and d(ATA)^H+^ also goes with a smaller loss of favorable stacking interaction and, thus, less strain ▵*E*_strain_, than in the case of d(CGG)^H+^ and d(AAA)^H+^.

The solvation of the two single strands (Δ*E*_solv,ss1+ss2_) is more stabilizing than the solvation of the corresponding double strand (Δ*E*_solv,ds_), as can be seen from the data in Table 2. This is because the hydrogen‐bond front atoms need to be partly desolvated to facilitate the complementary base pairing. This partial desolvation is the reason for the overall destabilizing effect of the solvent represented by Δ*E*_solv,ds_–Δ*E*_solv,ss1+ss2_ in Table 2. Furthermore, there is only little variation in Δ*E*_solv,ds_–Δ*E*_solv,ss1+ss2_ among the all‐AT sequences while there is a more pronounced sequence dependence in this term for the all‐GC duplexes (see below).

Finally, a trend opposite to Δ*E*_solv,ds_–Δ*E*_solv,ss1+ss2_ was found for the interaction energy Δ*E*_int_, meaning that when the overall desolvation is strong, the interaction energy is also strong as is the case for the CGG and GGG, and TAA and AAA sequences. This can be understood with the insight that the interaction with the aqueous solvent and between the complementary DNA bases relies both on hydrogen‐bond interactions. Therefore Δ*E*_solv,ds_–Δ*E*_solv,ss1+ss2_ and ▵*E*_int_ should yield the same trend, but with an opposite sign. A more detailed analysis of the interaction energy ▵*E*_int_ can be found in Table S4.^[7]^

### Interaction Analysis: Decomposition of the Solvation Energy

Next, we address the origin of the computed solvent effects Δ*E*_solv,ds_–Δ*E*_solv,ss1+ss2_ and the aforementioned trends therein. To this end, we decompose the solvation energy ▵*E*_solv_ (=*E*_aq_–*E*_gas_) into the components that are associated with two different physical phenomena, namely, the cavitation energy (*E*_solv,cav_) and the solute–solvent interaction (*E*_solv,int_) (see Equation 7).

(7) $$\Delta E{}_{\mathrm{solv}}=E{}_{\mathrm{solv},\mathrm{cav}}+E{}_{\mathrm{solv},\mathrm{int}}$$

The former, *E*_solv,cav_, is the energy associated with creating an empty cavity in the solvent to make room for the solute molecules, that is, the single and double DNA strands. Cavitation disrupts the local (fluxional) solvent structure and is accompanied by loss of intermolecular interactions between solvent molecules. In the COSMO model that we use in our computations, this is simulated by a destabilizing energy term, associated with creating a cavity in a dielectric medium of the right size and shape to host the solute molecules based on effective radii of the solvent and the atoms in the solute (for details, see Ref. [11]). The solute–solvent interaction *E*_solv,int_ results from the interaction of the solute molecule in the cavity with the solvent. In COSMO, this comes down to the interaction of the fully quantum chemically described solute molecules that generate, and electrostatically interact with, the mirror charges generated on the surface of the cavity in the dielectric continuum.^[11]^ Note that, also in our COSMO simulation, the solute–solvent interaction induces a change in the density of the solute, although no charge is transferred between solute and solvent. The results of the solvation energy decomposition of the all‐GC and all‐AT duplexes, based on Equation (7), are collected in Table 3.

**Table 3:** Partitioning of the solvation energy ▵*E*_solv_ (in kcal mol^−1^) of the double strand (ds) compared to the two complementary single strands (ss1+ss2) for the all‐GC and all‐AT d(DNA)^H+^ duplexes.^[a]^

	*ss1+ss2*				*ds*		
d(DNA)^H+^	Δ*E*_solv_	*E*_solv,cav_	*E*_solv,int_		Δ*E*_solv_	*E*_solv,cav_	*E*_solv,int_
d(CGG)^H+^	−161.8	7.8	−169.6		−110.0	6.8	−116.8
d(GCG)^H+^	−146.4	7.6	−154.0		−107.7	6.8	−114.5
d(GGG)^H+^	−168.7	7.5	−176.2		−118.2	6.8	−124.9
d(GGC)^H+^	−153.3	7.5	−160.7		−113.5	6.8	−120.3
							
d(AAA)^H+^	−122.9	7.6	−130.6		−99.3	6.9	−106.2
d(ATA)^H+^	−121.5	7.7	−129.3		−99.8	7.0	−106.8
d(AAT)^H+^	−121.0	7.6	−128.6		−99.7	6.9	−106.6
d(TAA)^H+^	−123.1	7.8	−130.9		−99.1	7.1	−106.2

[a] Computed at the BLYP‐D3(BJ)/TZ2P level using COSMO to simulate solvation in water.

Solute–solvent interactions are substantially stabilizing for all single and double DNA strands, whereas cavitation terms are smaller and destabilizing, as expected. The solute–solvent interaction term (*E*_solv,int_) becomes less stabilizing going from the single‐strands to the double‐strand as the duplex formation requires partial desolvation of the nucleobases, where stabilizing interactions with aqueous continuum are exchanged for the Watson–Crick hydrogen bonds. The cavitation term *E*_solv,cav_ becomes only slightly less destabilizing going from the two single strands to the double strand, which is associated with a decrease in cavity size within the continuum solvent. The contraction of the cavity is the result of the hydrogen‐bond interactions between the DNA base pairs which allows them to approach each other more closely than the sum of their van der Waals radii.^[1b]^ In Watson–Crick base pairs, this hydrogen‐bond contraction amounts to approximately 0.5 Å. Overall, the minor decrease of *E*_solv,cav_ does not make up for the significant loss in solute–solvent interactions, leaving the total solvent effect as a destabilizing factor in the duplex formation

The dependence of the overall solvent effect (Δ*E*_solv,ds_–Δ*E*_solv,ss1+ss2_) on the order in which the nucleotides occur results from the variation in solute–solvent interactions, as *E*_solv,cav_ hardly varies among the all‐GC and all‐AT sequences (Table 3). As the formation of the Watson–Crick hydrogen bonds shields the interior of the duplex, the sequence dependence of the solvent effect originates primarily from the difference in solute–solvent interactions of the single strands as supported by the more significant variation of *E*_solv,int_ for the two single strands compared to the double strands in Table 3. Remarkably, there is only little variation in Δ*E*_solv,ss1+ss2_ among the all‐AT sequences, while there is a clear sequence dependence in this term for the all‐GC duplexes. The reason for this is that the solvation energies of the nucleobases G and C differ more than the solvation energies of A and T, leading to more pronounced sequence‐dependent differences for the all‐GC single‐strands (see Figure S5a). In addition to the larger G versus C solvation energy difference, our computations identify that the NH_2_ group of G is positioned such that it can form an intra‐strand hydrogen bond with the deoxyribose oxygen atom of the backbone to which it is attached when located in the first or second position (5’‐to‐3’ notation) of a triplet single strand (see Figure S5b). The formation of internal hydrogen bonds reduces the desolvation energy (−Δ*E*_solv,ss1+ss2_) of all single‐strands with a G in position 1 or 2 upon forming the duplex (see Table S5). However, as the (CCC)^H+^ and (CCG)^H+^ single strands do not contain such guanines and therefore do not form intra‐strand hydrogen bonds, this leads to relatively higher desolvation energies of these strands. As the (CCC)^H+^ and (CCG)^H+^ single strands are part of the d(GGG)^H+^ and d(CGG)^H+^ duplexes, respectively, they lead to more destabilizing Δ*E*_solv,ds_–Δ*E*_solv,ss1+ss2_ values for these duplexes (Table 2). The position of the NH_2_ group in adenine cannot facilitate hydrogen bonds with the sugar‐phosphate backbone, which also explains the lack of sequence dependency in Δ*E*_solv,ds_–Δ*E*_solv,ss1+ss2_ for the all‐AT duplexes.

### Interaction Analysis: Decomposition of the Strain Energy

**Figure 4:**
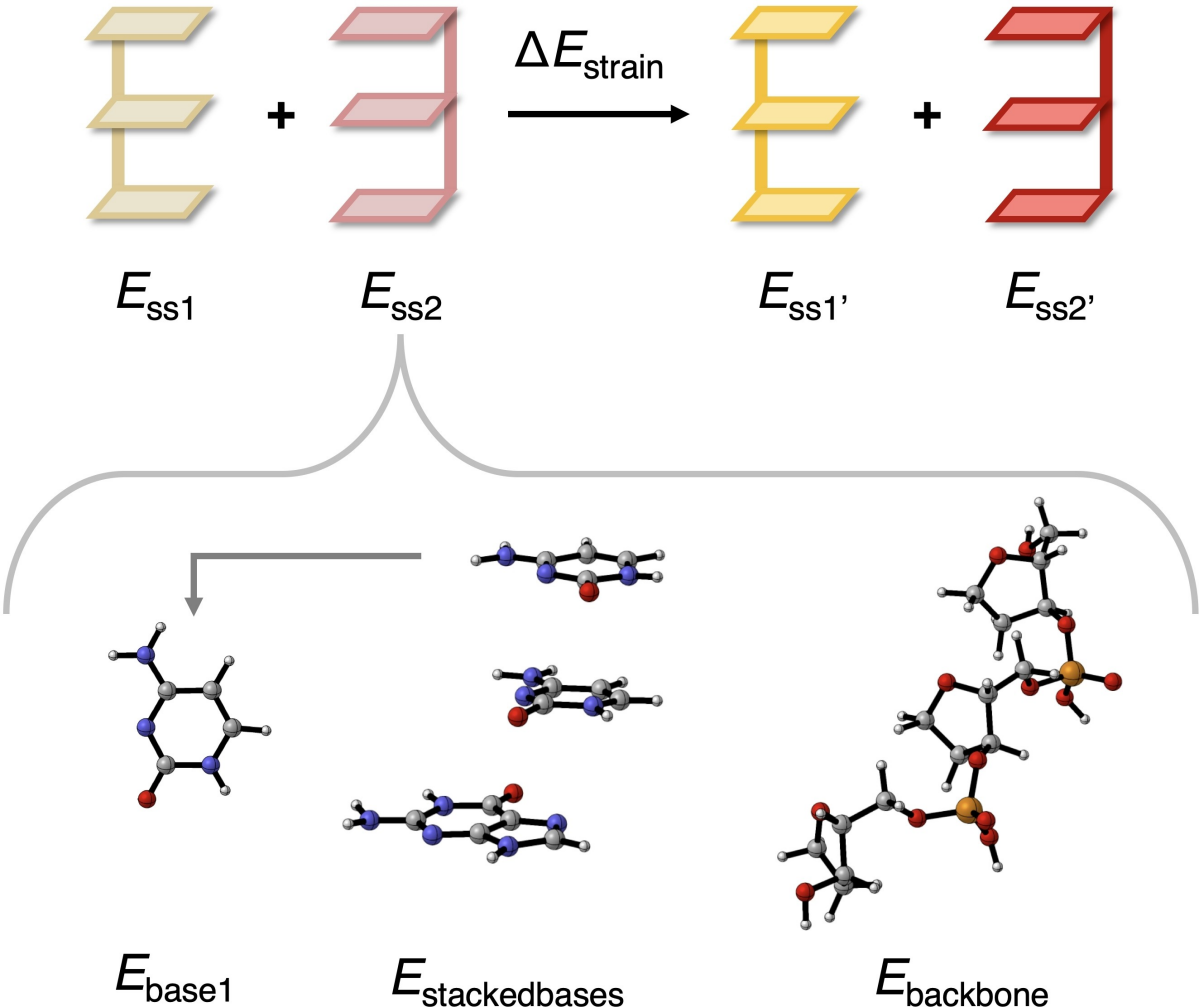
Structural decomposition of (DNA)^H+^ to evaluate the contributions of the nucleobases, base stacking, and sugar‐phosphate backbone to the total strain energy ▵*E*_strain_. Color code in ball‐and‐stick structures: H – white; C – grey; N – blue; O – red; P – yellow.

The strain energy ▵*E*_strain_ of the single strands consists of the deformation of the sugar‐phosphate backbone and the nucleobases due to the formation of the DNA double strand (see below). In this section, we quantify, for the various all‐GC and all‐AT sequences, which part of the deforming single strands contributes to what extent to the strain energy ▵*E*_strain_ (see Table 2) upon duplex formation: (i) the sugar‐phosphate backbone; (ii) the individual nucleobases; and (iii) the stacks of nucleobases. Using the COSMO‐optimized geometries of the DNA double and single strands, the contributions were examined by decomposing the single strands, in the gas phase, into smaller segments, as illustrated in Figure 4. The resulting smaller fragments were kept in the exact same geometry as they had in the larger strand and the dissociated glycosidic C−N bonds were terminated by hydrogen atoms, yielding C−H and N−H bonds in the backbone and the nucleobase fragments, respectively. These terminating C−H and N−H bonds have the same bond and dihedral angles as the original glycosidic bond and a fixed bond length of 1.03 (N−H) or 1.07 Å (C−H).

The contribution to ▵*E*_strain_ stemming from the deformation of the sugar‐phosphate backbone (Δ*E*_strain_backbone_, Equation (8)) is estimated by the difference between the energies of the backbone fragments in the separate single strands (*E*_backbone_ss1_ and *E*_backbone_ss2_, see Figure 4) and the energies of the backbone fragments in the duplex geometry (*E*_backbone_ss1’_ and *E*_backbone_ss2’_).

(8) $$\Delta E{}_{\mathrm{strain}\_\mathrm{backbone}}=E{}_{\mathrm{backbone}\_\mathrm{ss}1{}^{'}}+E{}_{\mathrm{backbone}\_\mathrm{ss}2{}^{'}}-E{}_{\mathrm{backbone}\_\mathrm{ss}1}-E{}_{\mathrm{backbone}\_\mathrm{ss}2}$$

The contribution to ▵*E*_strain_ stemming from the individual nucleobases upon forming the DNA duplex from the two complementary strands (Δ*E*_strain_bases_) is estimated using Equation (9). Here, *E*_base_ denotes the energy of a separate base in the geometry of the separate single strands (*E*_base_ss1_ and *E*_base_ss2_, see Figure 4) or in the deformed geometry within the DNA double strand (*E*_base_ss1’_ and *E*_base_ss2’_). So, Equation (9) sums up, for each strand, the deformation of the three individual bases within each strand.

(9) $$\Delta E_{\mathrm{strain},\mathrm{bases}}=\sum_{i=1}^{3}{\left(E_{\mathrm{base}\_\mathrm{ss}1^{'}}-E_{\mathrm{base}\_\mathrm{ss}2}\right)}_{i}\;+{\left(E_{{\mathrm{base}\_\mathrm{ss}2}^{'}}-E_{\mathrm{base}\_\mathrm{ss}2}\right)}_{i}\;$$

Finally, the contribution to ▵*E*_strain_ stemming from the change in base stacking interactions per strand is estimated by Δ*E*_strain_stacking_ as formulated in Equation (10). Here, Δ*E*_strain_stacking_ is the difference in stacking energy going from the DNA single strands (Δ*E*_stacking_ss1_ and Δ*E*_stacking_ss2_) to the respective strands in the duplex (Δ*E*_stacking_ss1’_ and Δ*E*_stacking_ss2’_). The stacking energy Δ*E*_stacking_ of an individual strand is formulated as the energy of the stack of bases (*E*_stackedbases_, see Figure 4) minus the energy of the three separate bases (*E*_base1‐3_) in the geometry within the respective stack (see Equation 11).

(10) $$\Delta E{}_{\mathrm{strain}\_\mathrm{stacking}}=\Delta E{}_{\mathrm{stacking}\_\mathrm{ss}1{}^{'}}+\Delta E{}_{\mathrm{stacking}\_\mathrm{ss}2{}^{'}}-\Delta E{}_{\mathrm{stacking}\_\mathrm{ss}1}-\Delta E{}_{\mathrm{stacking}\_\mathrm{ss}2}$$

(11) $$\Delta E{}_{\mathrm{stacking}}=E{}_{\mathrm{stackedbases}}-E{}_{\mathrm{base}1}-E{}_{\mathrm{base}2}-E{}_{\mathrm{base}3}$$

The estimates of the three different contributions to the strain energy ▵*E*_strain_ in the single strands upon duplex formation of the all‐GC and all‐AT sequences are collected in Table 4. Although the sum of Δ*E*_strain_backbone_, Δ*E*_strain_bases_, and Δ*E*_strain_stacking_ covers most of the components that give rise to the activation strain of duplex formation, we stress that these terms do not add up exactly to the exact ▵*E*_strain_ of the full duplexes, because of the fragmentation scheme which treats, in particular, the effects associated with the glycosidic bonds and the relative orientation of the backbone with regard to the nucleobases in an approximate manner.

**Table 4:** Energy contributions^[a]^ (in kcal mol^−1^) of the deformation of the sugar‐phosphate backbone, base geometry, and base stacking to the exact strain energy ▵*E*_strain_ for the d(DNA)^H+^ duplex assembling from the two complementary single strands for the all‐GC and all‐AT sequences.

d(DNA)^H+^	Δ*E*_strain_backbone_	Δ*E*_strain_bases_	Δ*E*_strain_stacking_	Total^[b]^	Δ*E*_strain_(exact)
d(CGG)^H+^	−1.2	9.2	6.8	14.8	19.1
d(GCG)^H+^	−2.5	9.0	4.6	11.1	16.6
d(GGG)^H+^	−3.3	8.9	8.9	14.5	21.9
d(GGC)^H+^	−3.4	8.8	3.5	8.9	17.8
					
d(AAA)^H+^	−2.3	5.1	3.1	5.9	7.8
d(ATA)^H+^	0.2	5.0	0.4	5.6	5.3
d(AAT)^H+^	0.6	5.1	1.7	7.4	7.5
d(TAA)^H+^	−1.0	4.9	2.4	6.3	7.2

[a] Computed at the BLYP‐D3(BJ)/TZ2P level; [b] the sum of Δ*E*_strain_backbone_, Δ*E*_strain_bases_, and Δ*E*_strain_stacking_.

Δ*E*_strain_ originates primarily from the deformation of the nucleobases and the loss of stacking interactions, while the backbone geometry deforms only slightly or even adopts a slightly more favorable geometry in the duplex. Interestingly, the values of Δ*E*_strain_backbone_ in Table 4 reflect that some of the backbone strain is relieved upon going from the geometry in the single strand to the hydrogen‐bonded geometry in the DNA duplex. This shows that in the single strands, the equilibrium geometry is mainly determined by optimizing the base stacking interactions, which leads to a suboptimal geometry for the sugar‐phosphate backbone. At variance, in the DNA duplex, the equilibrium geometry is dominated by optimizing the relatively strong Watson–Crick hydrogen bonds which happen to match better with the intrinsic optimum of the sugar‐phosphate backbone.

Thus, while base stacking is often regarded as the main contributor to duplex DNA stability,^[12]^ we demonstrate here that, in fact, the base stacking becomes *less* favorable in the duplex geometry compared to the two separate single strands, as a destabilizing Δ*E*_strain_stacking_ was observed for all DNA sequences in Table 4. Important to realize here is that DNA duplexes do not assemble directly from individual unstacked nucleosides. Instead, driven by the formation of hydrogen bonds, the double helix is obtained from the separately synthesized complementary strands where stacking interactions are already present.[^1a^, ^13^] Although base stacking contributes to the overall stability of DNA, based on the stacking interactions alone the formation of the duplex from two single‐strands would be unfavored. Nevertheless, the duplex formation is ultimately promoted by the presence of other stabilizing interactions between the complementary strands, including hydrogen bonding. Due to the formation of the hydrogen bonds, the nucleobases also deform upon forming the DNA double strand.^[14]^ Δ*E*_strain_bases_ is therefore destabilizing and amounts to approximately 9 kcal mol^−1^ for the all‐GC sequences, but only 5 kcal mol^−1^ for the AT sequences. This difference is again because the G−C base pair forms three hydrogen bonds, while there are only two hydrogen bonds in the A−T base pair, which gives rise to more deformation in the former and less in the latter.

### Interaction Analysis: Decomposition of the Interaction Energy

**Figure 5:**
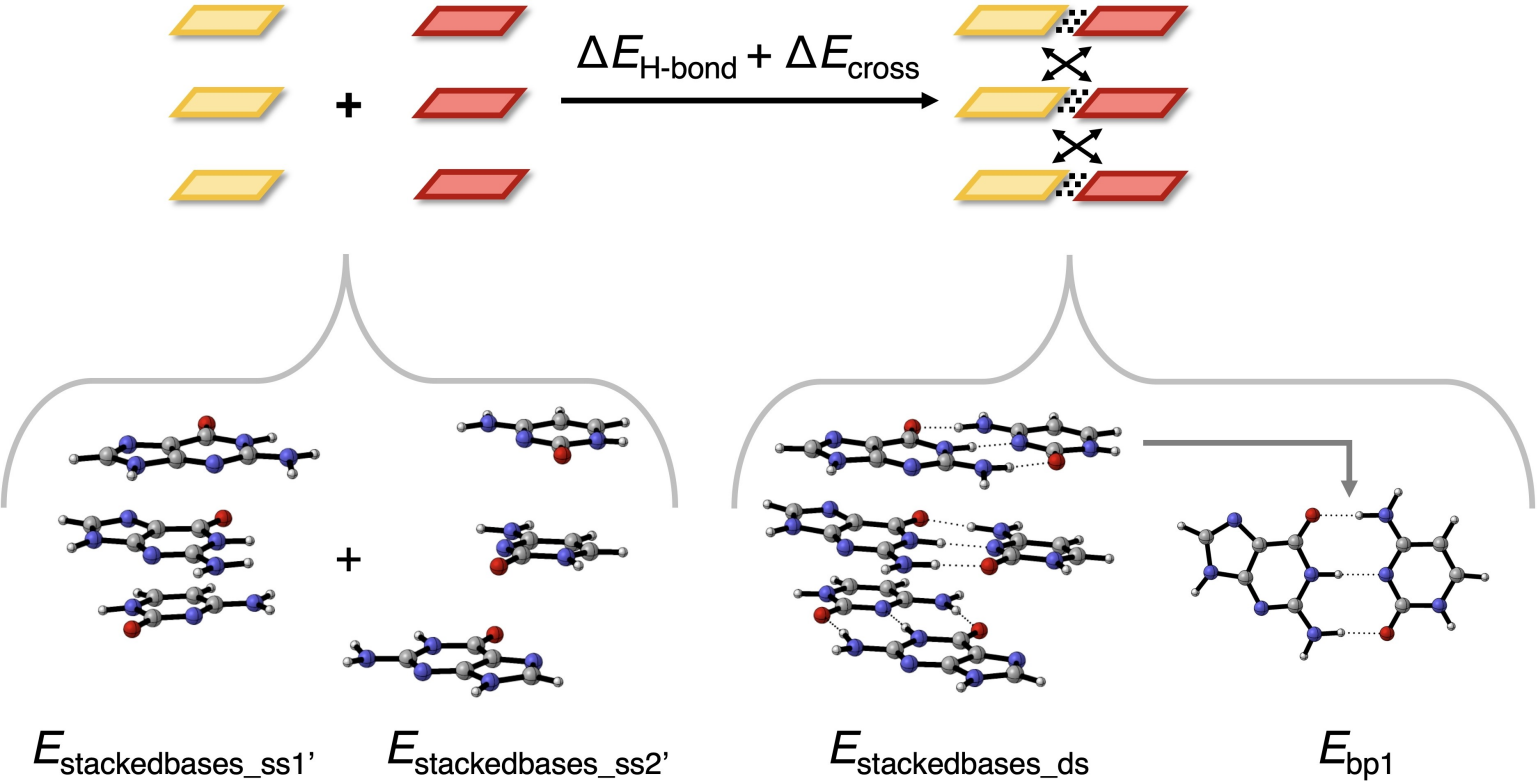
Structural decomposition to evaluate the contributions of the hydrogen‐bond interactions Δ*E*_H‐bond_ (dotted lines) and cross terms Δ*E*_cross_ (arrows) to the total interaction energy ▵*E*_int_ between two interacting stacks of nucleobases without backbone. Color code in ball‐and‐stick structures: H – white; C – grey; N – blue; O – red.

Lastly, we analyze the interaction energy (Δ*E*_int_) to identify the origin of the sequence dependence observed for the different all‐GC and all‐AT d(DNA)^H+^ sequences (recall Table 2). Our analyses reveal that Δ*E*_int_ consists of two major contributions: (i) the Watson–Crick hydrogen bonds; and (ii) the diagonal interactions between bases of two different, consecutively stacked Watson–Crick pairs. Interestingly, we find that the Watson–Crick hydrogen bonding interactions are sequence‐independent. Therefore, the sequence dependence originates nearly exclusively from the diagonal interactions. Using the COSMO‐optimized geometries of the d(DNA)^H+^ duplexes, both interaction energy components were examined in the gas phase by decomposing the double strands into smaller segments, as illustrated in Figure 5. For this analysis, we employ nucleobase fragments without the sugar‐phosphate backbone moiety. The consideration of only the nucleobases to describe the trends in total interaction energy is justified, as the interaction between the two sugar‐phosphate backbone fragments was found to be negligible for the all‐AT and all‐GC sequences (see Table S6). The resulting smaller nucleobase fragments were kept in the exact same geometry as they had in the strand with backbone and the dissociated glycosidic C−N bonds were terminated by hydrogen atoms, yielding N−H bonds that have the same bond and dihedral angles as the original glycosidic bond and a fixed bond length of 1.03 Å.

As defined by Equation 12 and illustrated in Figure 5, the total interaction energy between two complementary stacks of bases, with energy *E*_stackedbases_ss1’_ and *E*_stackedbases_ss2’_, upon forming the duplex with energy *E*_stackedbases_ds_, can be regarded as the sum of the Watson–Crick hydrogen‐bond interactions (Δ*E*_H‐bond_) and the diagonal interactions or so‐called “cross terms” (Δ*E*_cross_), which are the interactions between bases that are diagonal to each other (i. e., on opposite sides and in different layers).

(12) $$\Delta E{}_{H\text{-}\mathrm{bond}}+\Delta E{}_{\mathrm{cross}}=E{}_{\mathrm{stackedbases}\_\mathrm{ds}}-E{}_{\mathrm{stackedbases}\_\mathrm{ss}1{}^{'}}-E{}_{\mathrm{stackedbases}\_\mathrm{ss}2{}^{'}}$$

Δ*E*_H‐bond_ is computed as the sum of the energies of the three base pairs (*E*_bp_, see Figure 5) minus the sum of the energies of the six separate nucleobases in the geometry of the duplex (Equation (13)). Δ*E*_cross_ is obtained by subtracting Δ*E*_H‐bond_ from the total interaction energy between the two stacks (i. e., Δ*E*_H‐bond_+Δ*E*_cross_).

(13) $$\overset{3}{\underset{i=1}{\Delta E_{H-bond}=E_{bp1}+E_{bp2}+E_{bp3}-\sum }}\;{\left(E_{\mathrm{base}\_\mathrm{ss}1^{'}}+E_{\mathrm{base}\_\mathrm{ss}2}\right)}_{i}\;$$

**Figure 6:**
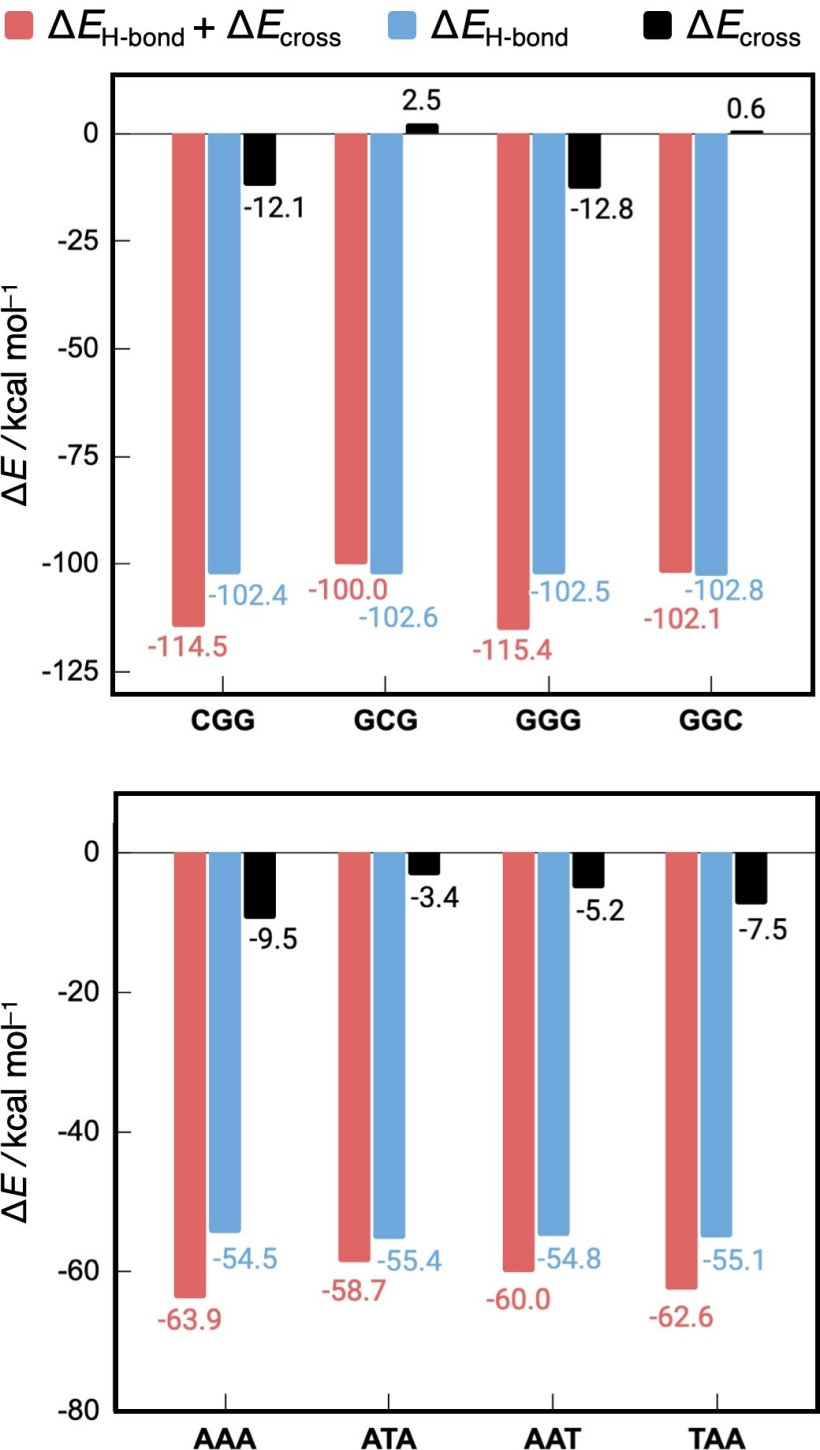
Relative contributions of the hydrogen bonds and cross terms to the total interaction energy (Δ*E*_H‐bond_+Δ*E*_cross_) between two complementary single strands without backbone in the DNA duplex for the all‐GC (top) and all‐AT (bottom) sequences. Energies are in kcal mol^−1^ and computed at the BLYP‐D3(BJ)/TZ2P level of theory.

The computed Δ*E*_H‐bond_+Δ*E*_cross_ and relative contributions are presented in Figure 6 for the all‐GC and all‐AT sequences. Note that Δ*E*_H‐bond_+Δ*E*_cross_ in Figure 6 for the DNA systems without the backbone shows the same trend but has not the same magnitude as Δ*E*_int_ of the complete DNA systems with backbone (Table 2) because the backbone of one strand also interacts weakly with the stacked DNA bases of the other strand. Our computations show that for d(CGG)^H+^, the interaction between the backbone of the CCG strand and the stacked bases of the complementary CGG strand is approximately −3 kcal mol^−1^. For the (CCG)^H+^ single strand with backbone interacting with the complementary CGG single strand without the backbone, a Δ*E*_int_ of −117.1 kcal mol^−1^ was reported. In this case, only one backbone interacts with the stacked DNA bases of the complementary strand, so that the value of −117.1 kcal mol^−1^ lies in between the interaction energy of the complete duplex d(CGG)^H+^ (−118.8 kcal mol^−1^) and the CGG duplex without backbone (−114.5 kcal mol^−1^). This confirms that the backbone of one strand interacts weakly with the bases of the complementary strand. The significant size of the backbone, and its bent orientation towards the nucleobases, makes this attractive backbone−complementary bases interaction of such significant size that it overcomes the slightly weakening effect of introducing alkyl substituents on the hydrogen bonding interaction in G−C and A–U/T base pairs.[^14a^, ^15^]

From Figure 6 follows that the sequence‐dependent variation in the total interaction energy (Δ*E*_H‐bond_+Δ*E*_cross_) originates from the cross‐term interactions (Δ*E*_cross_), as the hydrogen‐bond energy (Δ*E*_H‐bond_) varies barely for the different sequences. Among the GC sequences, the CGG and GGG duplexes experience a more stabilizing Δ*E*_H‐bond_+Δ*E*_cross_ than GCG and GGC by approximately −15 kcal mol^−1^. This is in line with the results in Table 2 for the DNA systems with backbone. The more stabilizing total interactions for CGG and GGG follow directly from the very stabilizing cross terms (>−12 kcal mol^−1^) for these sequences, while GGC and GCG experience small destabilizing diagonal interactions. The hydrogen‐bond interaction is almost constant for all GC sequences.

For the AT sequences, the total interaction strength also correlates with the differences in the diagonal interactions: Δ*E*_cross_ is stabilizing in all cases and the magnitude decreases going from AAA to TAA to AAT to ATA, explaining the trend of Δ*E*_H‐bond_+Δ*E*_cross_, as Δ*E*_H‐bond_ is nearly constant.

Whether the total cross terms are stabilizing or destabilizing depends on the net electrostatic attraction or repulsion between polar functional groups on diagonal nucleobases. We demonstrate this nucleotide order dependence of Δ*E*_cross_ by decomposing the diagonal interactions present in the interacting DNA stacks into physically meaningful terms based on Kohn–Sham molecular orbital theory using a quantitative energy decomposition analysis (EDA).^[7]^ This analysis decomposes the cross‐term interaction energy (Δ*E*_int_cross_) into Pauli repulsion (Δ*E*_Pauli_), electrostatic interaction (Δ*V*_elstat_), orbital interaction (Δ*E*_oi_), and dispersion (Δ*E*_disp_) components (Equation 14).

(14) $$\Delta E{}_{\mathrm{int}\_\mathrm{cross}}=\Delta E{}_{\mathrm{Pauli}}+\Delta V{}_{\mathrm{elstat}}+\Delta E{}_{\mathrm{oi}}+\Delta E{}_{\mathrm{disp}}$$

Here, Δ*E*_Pauli_ corresponds to the destabilizing interactions between the occupied orbitals on the fragments and accounts for any steric repulsion. Δ*V*_elstat_ accounts for the classical electrostatic interactions between the unperturbed charge distributions of the prepared (i. e., deformed) interacting molecular fragments and is usually attractive. The term Δ*E*_oi_ comprises charge transfer (i. e., donor–acceptor interactions between occupied orbitals on one of the interacting fragments and unoccupied orbitals on the other, including HOMO–LUMO interactions) and polarization (i. e., empty–occupied orbital mixing on one fragment due to the presence of the other fragment). The term Δ*E*_disp_ includes a dispersion energy correction.

**Figure 7:**
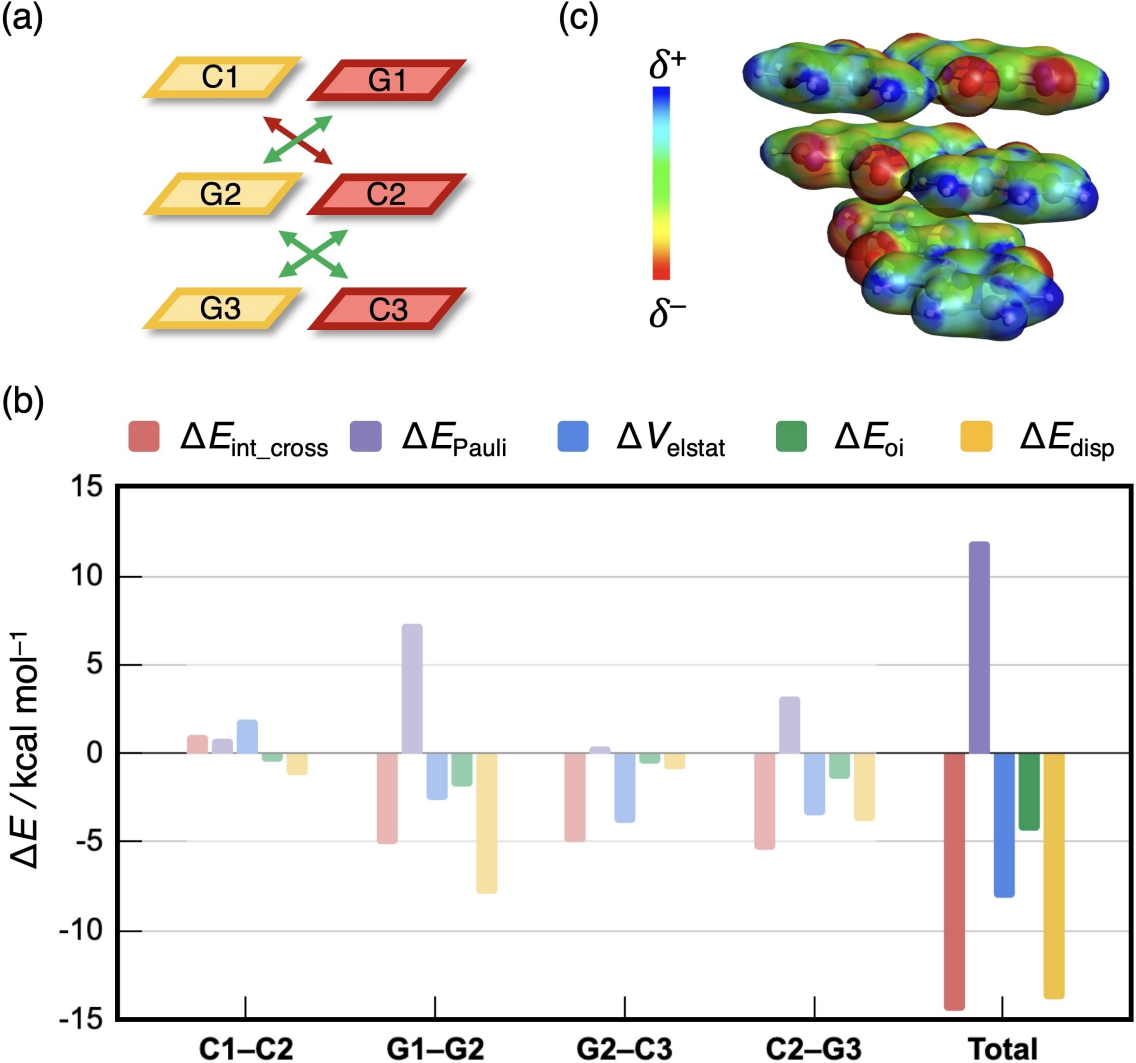
(a) Three stabilizing and one destabilizing cross‐terms are present in d(CGG)^H+^ without backbone. (b) Partitioning of the cross‐term interaction energy Δ*E*_int_cross_ (in kcal mol^−1^) between diagonal bases in the CGG duplex without backbone, computed at the BLYP‐D3(BJ)/TZ2P level. (c) Electrostatic potential surface (at 0.03 au) from −0.0 (red) to +0.2 (blue).

**Figure 8:**
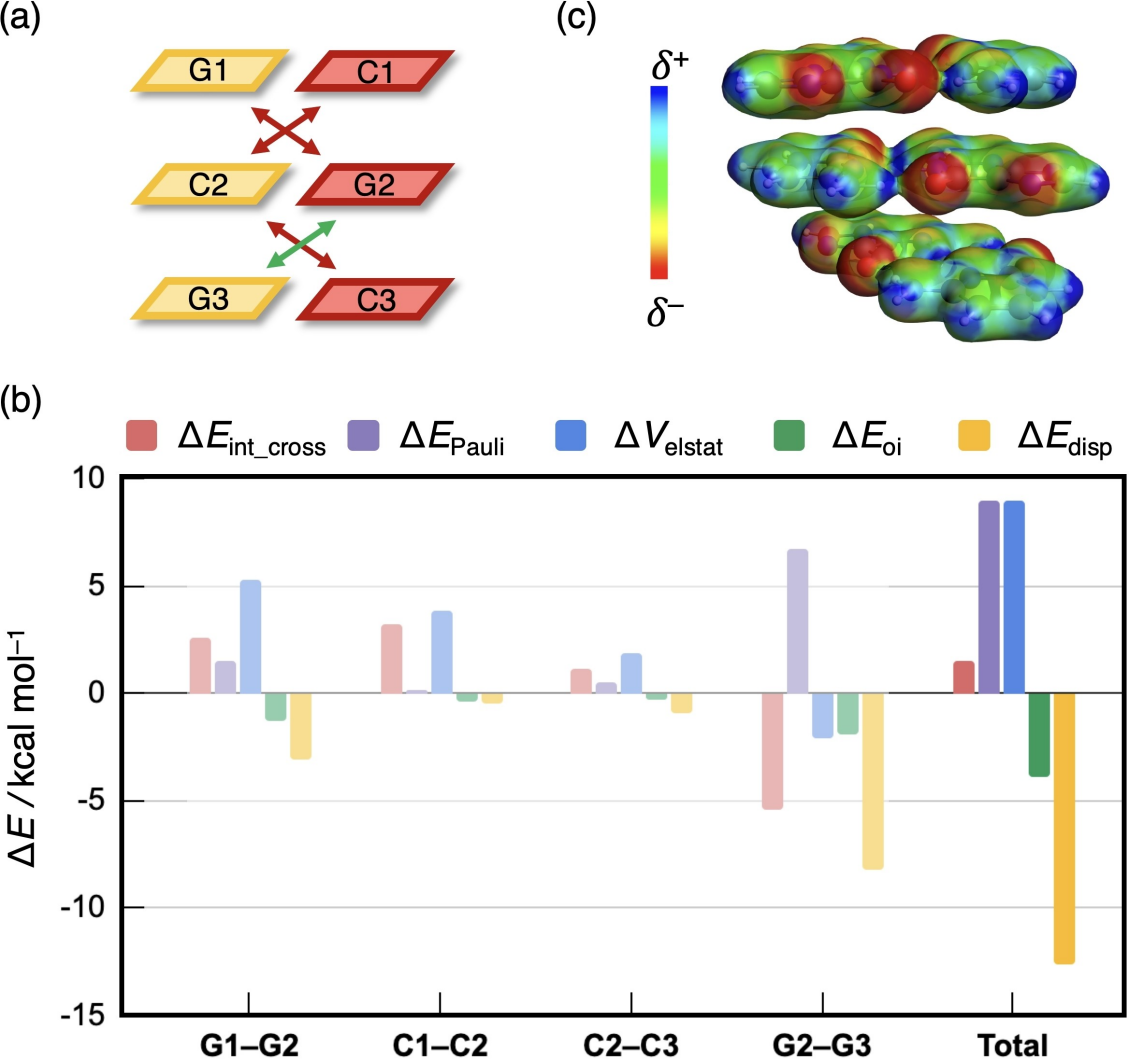
(a) One stabilizing and three destabilizing cross‐terms are present in d(GCG)^H+^ without backbone. (b) Partitioning of the cross‐term interaction energy Δ*E*_int_cross_ (in kcal mol^−1^) between diagonal bases in the GCG duplex without backbone, computed at the BLYP‐D3(BJ)/TZ2P level. (c) Electrostatic potential surface (at 0.03 au) from −0.0 (red) to +0.2 (blue).

To trace the origin of the sequence dependence of the cross terms and thus the total interaction energy between two complementary DNA strands, we focus on the two extreme cases in Figures 7 and 8, that is the CGG and the GCG sequence with stabilizing and destabilizing total cross terms, respectively. For the numerical values of the EDAs, see Supporting Information Figure S6, which also reports the interaction between the bases that are diagonal to each other but not in adjacent layers. This interaction was found to be negligibly small (<1 kcal mol^−1^) and is in line with the popular “nearest neighbor” approach to predict thermodynamic properties of DNA sequences.^[16]^

The decomposition of the diagonal interaction energy within the CGG duplex in Figure 7 shows that this sequence encounters three stabilizing (G1−G2, G2−C3, and C2−G3) and one destabilizing cross terms (C1−C2), and therefore a net stabilizing Δ*E*_int_cross_ (Figure 7a). The total Δ*E*_int_cross_ consists, in general, of a large stabilizing dispersion contribution Δ*E*_disp_, a significant amount of electrostatic interaction Δ*V*_elstat_, and a smaller contribution of orbital interactions Δ*E*_oi_, counteracted by a large destabilizing Pauli repulsion Δ*E*_Pauli_ (Figure 7b). Whether an individual diagonal interaction is stabilizing or destabilizing is dictated by the electrostatic interaction term. For example, the diagonal interaction between the bases C1 and C2 is destabilizing which results mainly from the destabilizing nature of Δ*V*_elstat_. This repulsive electrostatic interaction can be visualized by the electrostatic potential surface in Figure 7c. The hydrogen‐bond front atoms of the nucleobases guanine and cytosine are *δ* ^+^ NH_2_ groups or *δ* ^−^ carbonyl groups. Naturally, two diagonal *δ* ^+^ or two *δ* ^−^ functional groups will slightly repel each other, while opposite charges attract. Following this rationale, the C1−C2 interaction is destabilizing due to the electrostatic repulsion between two *δ* ^+^ NH_2_ groups. On the other hand, the stabilizing G2−C3 interaction results from the attractive interaction between a *δ* ^−^ carbonyl and *δ* ^+^ NH_2_ group.

The diagonal interaction energy was also decomposed for the GCG duplex without backbone (Figure 8). This DNA double‐strand gives rise to three destabilizing (G1−G2, C1−C2, and C2−C3), and only one stabilizing (G2−G3) cross‐term interaction (Figure 8a). This follows from the EDA in Figure 8b, which shows that the diagonal interactions between G1−G2, C1−C2, and C2−C3 are destabilizing due to a significant destabilizing Δ*V*_elstat_. The electrostatic potential plot in Figure 8c reveals that this repulsive Δ*V*_elstat_ is the result of the unfavorable interaction between two diagonal *δ* ^−^ carbonyl groups, in the case of G1−G2, or between two diagonal *δ* ^+^ amino groups, in the case of C1−C2 and C2−C3. As the absolute contributions of Δ*E*_Pauli_, Δ*E*_oi_, and Δ*E*_disp_ to the total Δ*E*_int_cross_ of GCG (Figure 8b) are nearly equal to the contributions for CGG (Figure 7b), we can conclude that Δ*V*_elstat_ determines whether the total cross‐term interaction energy is stabilizing or destabilizing.

The cross‐term interaction energy was also decomposed for two all‐AT d(DNA)^H+^ sequences without backbone, and the results of this analysis can be found in Supporting Information Figure S7. Here, the difference in the total Δ*E*_int_cross_ is also dictated by the difference in the electrostatic interaction Δ*V*_elstat_ resulting from combinations of attractive and repulsive interactions between polar functional groups involved in the Watson–Crick hydrogen bonding.

**Figure 9:**
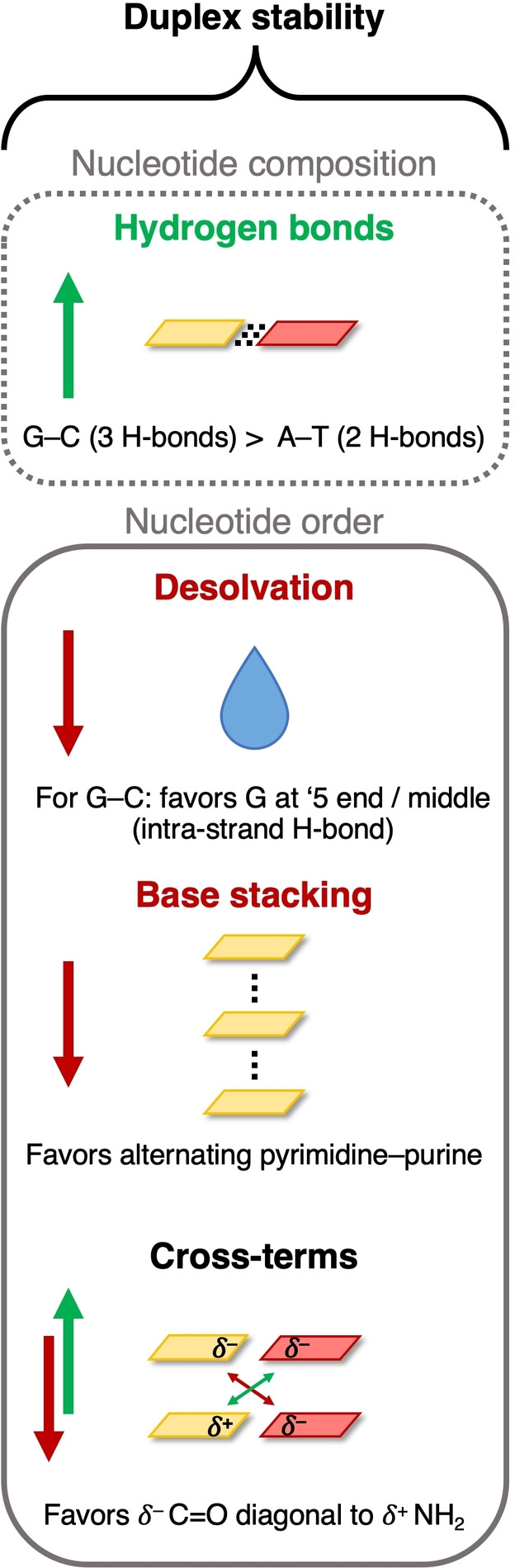
Schematic summary of the terms that contribute to the nucleotide composition and order trends in the stability of duplex DNA assembling from two complementary single strands, with stabilizing terms in green and destabilizing terms in red.

One of the main objectives of this work is to derive general rules for predicting duplex stability. In this last paragraph, summarized in Figure 9, we translate all findings and analyses into a set of rules to quantitatively predict stabilization or destabilization of DNA duplexes. The terms contributing to the duplex stability are counteracting and vary as a function of the nucleotide composition and order (see Figure 9). The subtle optimum of these terms determines which nucleotide sequence forms the most stable duplex. The hydrogen bonding between complementary bases is the most important stabilizing interaction within the DNA duplex and depends on the nucleotide composition as the triply hydrogen‐bonded G−C base pair is more stable than the only doubly hydrogen‐bonded A−T base pair. However, as we have shown, the hydrogen‐bond interactions are invariant with respect to the nucleotide order, and the sequence‐dependent variations in the duplex stability arise from the three other effects, and primarily the cross terms.

First of all, upon forming the duplex, the nucleobases need to be partially desolvated which works destabilizing. As the desolvation energy depends on the type of nucleobase, this term gives rise to sequence‐dependent solvent effects. This is particularly relevant for duplexes containing G−C base pairs, as G can form intra‐strand hydrogen bonds with the sugar‐phosphate backbone when at the 5’ end position or somewhere in the middle of the strand, which reduces the required desolvation energy. Furthermore, we identified the loss of stacking interactions within individual strands which is necessary to facilitate the Watson–Crick hydrogen bonding, as a destabilizing factor in the duplex formation. As pyrimidine and purine bases stack less well than bases of the same type, this term prefers an alternating sequence of pyrimidine and purine bases in the DNA double strand. Finally, we have shown that a strong dependence of the duplex stability on the nucleotide order is caused by the cross‐term interactions, which can be stabilizing or destabilizing depending on the net electrostatic attraction or repulsion between the hydrogen bonding functional groups on diagonal nucleobases. The positioning of a *δ* ^+^ NH_2_ group of one nucleobase opposite to a *δ* ^–^ carbonyl group of its diagonal nucleobase leads to attractive cross terms and, thus, in general, to more stable duplexes.

## Conclusions

The stability of double‐stranded DNA is not only proportional to the GC base pair content (i. e., more stable than the AT base pair due to an additional hydrogen bond), but also depends on the order of the Watson–Crick base pairs for a given GC versus AT content. This emerges from our quantum chemical analyses using dispersion‐corrected DFT computations of all 32 possible double‐stranded B‐DNA triplets in aqueous implicit solvation, in which we pinpoint the origin of these effects and provide guidelines for predicting the stability of oligonucleotides.

All possible duplex DNA triplets are stable in aqueous solution, also including an estimate of the destabilizing, but roughly constant, thermodynamic corrections (mainly from the zero‐point vibrational energy and loss of entropy). Therefore, the trend in duplex stability primarily depends on the electronic energy of formation, ▵*E*. The coordination of counterions to the negatively charged sugar‐phosphate backbone stabilizes the overall duplex but was found to not affect the nucleotide‐dependent trends significantly. Our activation strain analyses reveal that solvent effects are destabilizing, as duplex formation from complementary single strands requires partial desolvation of the nucleobases.

Although base stacking is often regarded as a crucial contributor to the double helix stability, we here identify the loss of stacking interactions within individual strands, to facilitate the Watson–Crick hydrogen bonding, as a destabilizing factor in the duplex formation. On the other hand, the deformation of the backbone was found to be negligible. The dependence of ▵*E* on the order of the nucleotides for duplexes of the same overall base pair composition follows primarily from the “diagonal interactions” or “cross terms”. We establish that, in general, more stabilizing cross terms lead to more stabilizing interaction energies and, in turn, more stable duplexes. The positioning of a *δ* ^+^ NH_2_ group of one nucleobase opposite to a *δ* ^−^ carbonyl group of its diagonal nucleobase manifests in attractive cross terms, while the interaction between two diagonal *δ* ^+^ NH_2_ or *δ* ^−^ carbonyl groups is repulsive. These insights have the potential to provide a more unified understanding of genome stability and also provide design principles for oligonucleotides with tailored stabilities.

## Computational Details

All calculations were performed using the Amsterdam Density Functional (version ADF2017.103) program developed by Baerends, Ziegler, and others,^[17]^ and the Quantum‐regions Interconnected by Local Descriptions (QUILD) program by Swart and Bickelhaupt.^[18]^ The QUILD program is a wrapper around ADF (and other programs) and is used for its superior geometry optimizer which is based on adapted delocalized coordinates.^[18a]^ The numerical integration was performed using the procedure developed by te Velde et al.[^17a^, ^17b^]

All computations are dispersion‐corrected density functional theory (DFT‐D3) based using the BLYP‐D3(BJ) functional in which the regular BLYP functional[^19^, ^20^] is augmented with an empirical correction for long‐range dispersion effects, described by a sum of damped interatomic potentials of the form C_6_R^−6^ added to the usual DFT energy.^[21]^ The basis set superposition error (BSSE) on the bond energy is effectively absorbed into the empirical dispersion‐correction potential.^[21a]^

Molecular orbitals were expanded in a large uncontracted set of Slater‐type orbitals (STOs) containing diffuse functions using a TZ2P basis set.^[17c]^ The basis set is of triple‐ζ quality for all atoms and has been augmented with two sets of polarization functions, that is, 2*p*, 3*d* on H, and 3*d* and 4*f* on C, N, O, Na, and P. The 1*s* core shells of the atoms C, N, O, Na, and P were treated by the frozen‐core approximation. The quality of the density fitting (ZlmFit) and the integration grid (BeckeGrid) was set to “good” for all computations.^[22]^ The reported Gibbs free energies in solution are calculated by adding thermal corrections computed at 298 K from vibrational frequencies obtained through numerical differentiation of the analytical gradient at COSMO(H_2_O)‐ZORA‐BLYP‐D3(BJ)/TZ2P to the total electronic energy computed at the same level. A more complete description of entropy effects can be obtained through MD simulations of model DNA systems in a box of explicit water molecules. This is, however, beyond the scope of this work in which we aim at estimating the existence of a constant entropic contribution to ▵*G* that does not affect the trends set by ▵*E*.

Solvation in water was simulated using the conductor‐like screening model (COSMO), as implemented in the ADF program^[11]^ with the solvent radius and dielectric constant being 1.9 Å and 78.4, respectively for water. Atomic radii to generate the COSMO cavities were set equal to the MM3 van der Waals radii divided by a factor 1.2 (for details, see Table S1 in the Supporting Information of Ref. [23]). The surface charges at the GEPOL93 solvent‐excluding surface were corrected for outlying charges. According to the work by Riley et al.,^[24]^ the dispersion correction does not need to be modified for the solvated systems.

The starting structures of the single and double‐stranded B‐DNA structures were build using the NUCLEIC routine of the TINKER molecular design program package.^[25]^ The analysis of the backbone torsion angles and the Watson–Crick hydrogen bonds in the double‐stranded structures was performed using the 3DNA software.^[26]^ The optimized structures were illustrated with CYLview20.^[27]^

## Conflict of interest

The authors declare no conflict of interest.

1

## Supporting information

As a service to our authors and readers, this journal provides supporting information supplied by the authors. Such materials are peer reviewed and may be re‐organized for online delivery, but are not copy‐edited or typeset. Technical support issues arising from supporting information (other than missing files) should be addressed to the authors.

Supporting Information

## Data Availability

The data that support the findings of this study are available in the supplementary material of this article.
