# Supplementary material for: B‐DNA Structure and Stability: The Role of Nucleotide Composition and Order
Source: ChemistryOpen. 2022 Jan 27;11(2):e202100231. doi: 10.1002/open.202100231 (PMC8805170; doi:10.1002/open.202100231)
Supplement: Supplementary file 1 — Supporting Information [file OPEN-11-e202100231-s001.pdf]

ChemistryOpen

Supporting Information

B-DNA Structure and Stability: The Role of Nucleotide Composition and Order

Celine Nieuwland, Trevor A. Hamlin, Célia Fonseca Guerra,* Giampaolo Barone,* and F. Matthias Bickelhaupt*

Contents

Figure S1. Visualization of the geometry of the $(GGG)^{H+}$ optimized single strand (ss) compared to its geometry within the duplex (ss'), computed at BLYP-D3(BJ)/TZ2P using COSMO to simulate solvation in water.

Figure S2. Formation energy ΔE (in kcal mol^{-1}) of the 32 double-stranded DNA triplets $d(DNA)^{4-}$ (no backbone neutralization) from two complementary single-strands, computed at BLYP-D3(BJ)/TZ2P using COSMO to simulate solvation in water. The colors indicate sequences with the same relative number of GC and AT base pairs.

Figure S3. Formation energy ΔE (in kcal mol^{-1}) of the 32 double-stranded DNA triplets $d(DNA)^{Na+}$ (backbone neutralized by Na^+) from two complementary single-strands, computed at BLYP-D3(BJ)/TZ2P using COSMO to simulate solvation in water. The colors indicate sequences with the same relative number of GC and AT base pairs.

Figure S4. Partitioning of the formation energy ΔE (in kcal mol^{-1}) for the $d(DNA)^{H+}$ duplex assembling from two complementary single-strands for the all-GC (left) and all-AT (right) sequences, computed at BLYP-D3(BJ)/TZ2P using COSMO to simulate solvation in water.

Figure S5. **(a)** Solvation energies (in kcal mol^{-1}) of isolated DNA bases guanine (G), cytosine (C), adenine (A), and thymine (T), calculated at BLYP-D3(BJ)/TZ2P using COSMO to simulated solvation in water. The DNA bases are substituted with a methyl group (CH_3) to simulate the sugar-phosphate backbone. **(b)** The NH_2 group of G can form intra-strand hydrogen bonds (green dotted lines) with the oxygen atom of the deoxyribose ring when in position 1 or 2 of a DNA triplet single-strand.

Figure S6. Partitioning of the interaction energy between diagonal bases ΔE_{int_cross} (in kcal mol^{-1}) in the DNA duplex for **(a)** the CGG and **(b)** the GCG sequence, computed at BLYP-D3(BJ)/TZ2P.

Figure S7. Partitioning of the interaction energy between diagonal bases ΔE_{int_cross} (in kcal mol^{-1}) in the DNA duplex for **(a)** the AAA and **(b)** the ATA sequence, computed at BLYP-D3(BJ)/TZ2P.

Table S1. Sugar-phosphate backbone torsion angles (in °) of the 32 optimized $d(DNA)^{4-}$ structures, computed at BLYP-D3(BJ)/TZ2P using COSMO to simulate solvation in water. See Scheme 1 for torsion angle definitions.

Table S2. Hydrogen-bond distances (in Å) of the 32 optimized $d(DNA)^{4-}$ structures, computed at BLYP-D3(BJ)/TZ2P using COSMO to simulate solvation in water. For atom labels, see Figure 1b.

Table S3. Formation energy ΔE (in kcal mol^{-1}) in decreasing order of the 32 double-stranded B-DNA structures $d(DNA)^{4-}$, $d(DNA)^{H+}$ and $d(DNA)^{Na+}$, computed at BLYP-D3(BJ)/TZ2P using COSMO to simulate solvation in water.

Table S4. Energy decomposition analysis (EDA) of the interaction energy ΔE_{int} (in kcal mol^{-1}) for the formation of the $d(DNA)^{H+}/d(DNA)^{4-}$ duplex from the two single-strands, computed at BLYP-D3(BJ)/TZ2P.

Table S5. Desolvation energy $-\Delta E_{solv}$ (in kcal mol^{-1}) of complementary all-GC and all-AT $(DNA)^{H+}$ single-strands (ss1 and ss2), computed at BLYP-D3(BJ)/TZ2P using COSMO to simulate solvation in water.

Table S6. Interaction energy for the interaction between two sugar-phosphate backbones $\Delta E_{int\_backbones}$ (in kcal mol^{-1}) in the $d(DNA)^{H+}$ geometry, in the absence of the nucleobases, computed at BLYP-D3(BJ)/TZ2P for the all-GC and all-AT sequences.

Table S7. Cartesian coordinates (in Å) and total bond energies E (in kcal mol^{-1}) of the optimized single- ($(DNA)^{2-}$, $(DNA)^{Na+}$, and $(DNA)^{H+}$) and double-stranded ($d(DNA)^{4-}$, $d(DNA)^{Na+}$, and $d(DNA)^{H+}$) trideoxyribonucleoside diphosphate DNA structures, computed at BLYP-D3(BJ)/TZ2P using COSMO to simulate solvation in water.

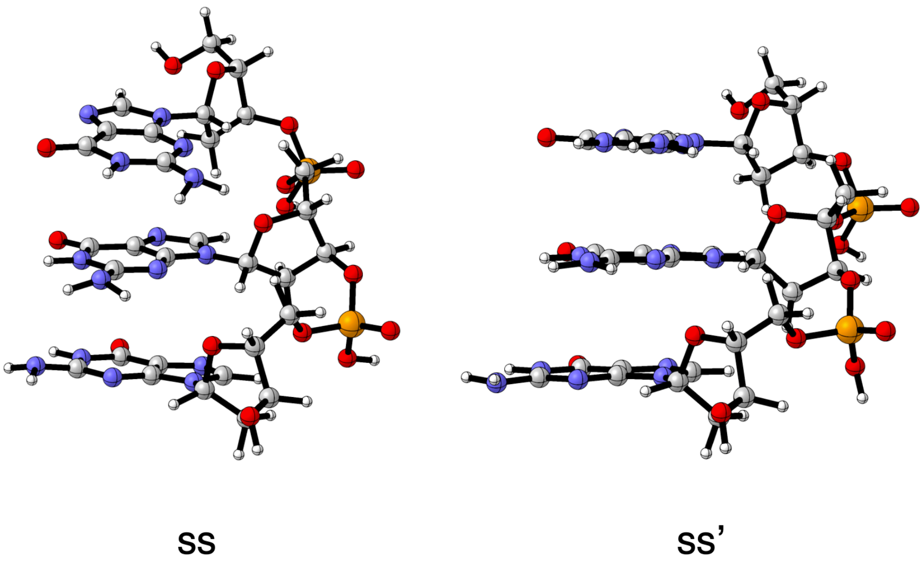

Figure S1. Visualization of the geometry of the (GGG)$^{H+}$ optimized single strand (ss) compared to its geometry within the duplex (ss'), computed at BLYP-D3(BJ)/TZ2P using COSMO to simulate solvation in water.

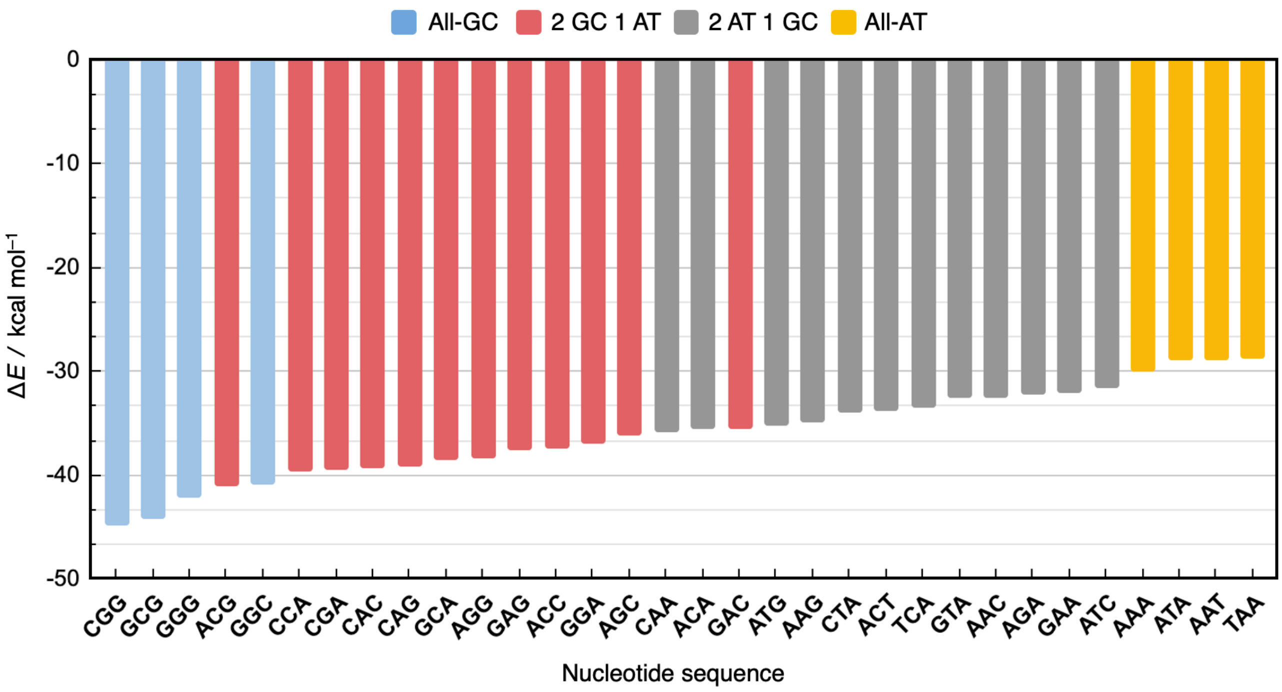

Figure S2. Formation energy Δ*E* (in kcal mol^{-1}) of the 32 double-stranded DNA triplets d(DNA)$^{4-}$ (no backbone neutralization) from two complementary single-strands, computed at BLYP-D3(BJ)/TZ2P using COSMO to simulate solvation in water. The colors indicate sequences with the same relative number of GC and AT base pairs.

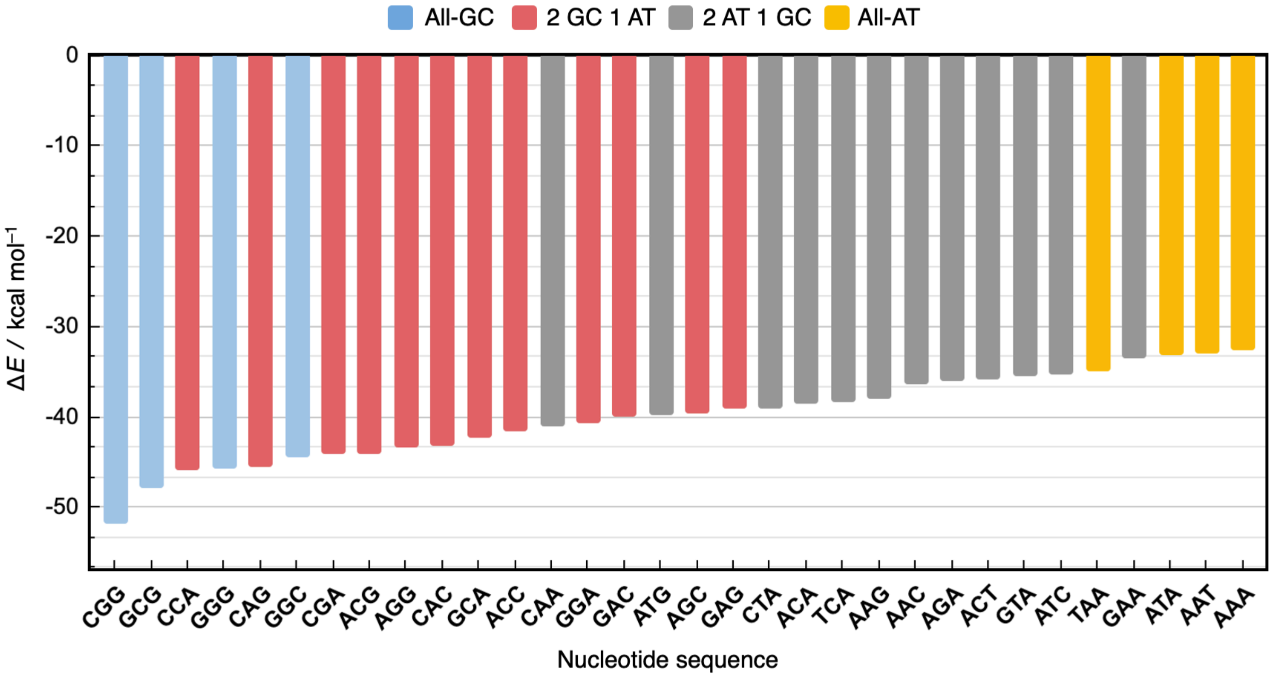

Figure S3. Formation energy ΔE (in kcal mol^{-1}) of the 32 double-stranded DNA triplets d(DNA)$^{Na+}$ (backbone neutralized by Na^+) from two complementary single-strands, computed at BLYP-D3(BJ)/TZ2P using COSMO to simulate solvation in water. The colors indicate sequences with the same relative number of GC and AT base pairs.

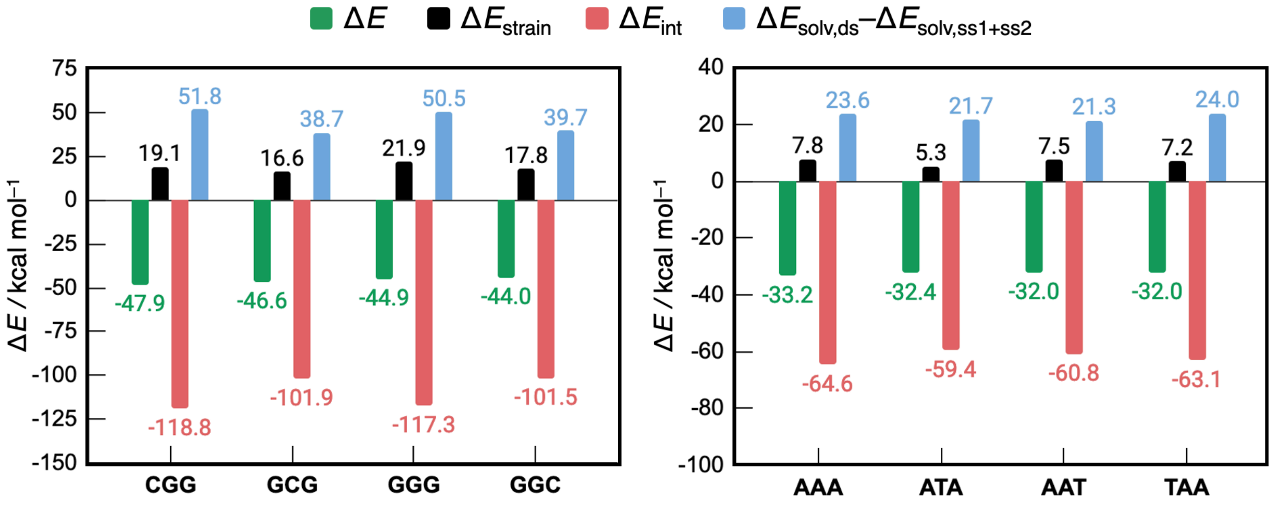

Figure S4. Partitioning of the formation energy ΔE (in kcal mol^{-1}) for the d(DNA)$^{H+}$ duplex assembling from two complementary single-strands for the all-GC (left) and all-AT (right) sequences, computed at BLYP-D3(BJ)/TZ2P using COSMO to simulate solvation in water.

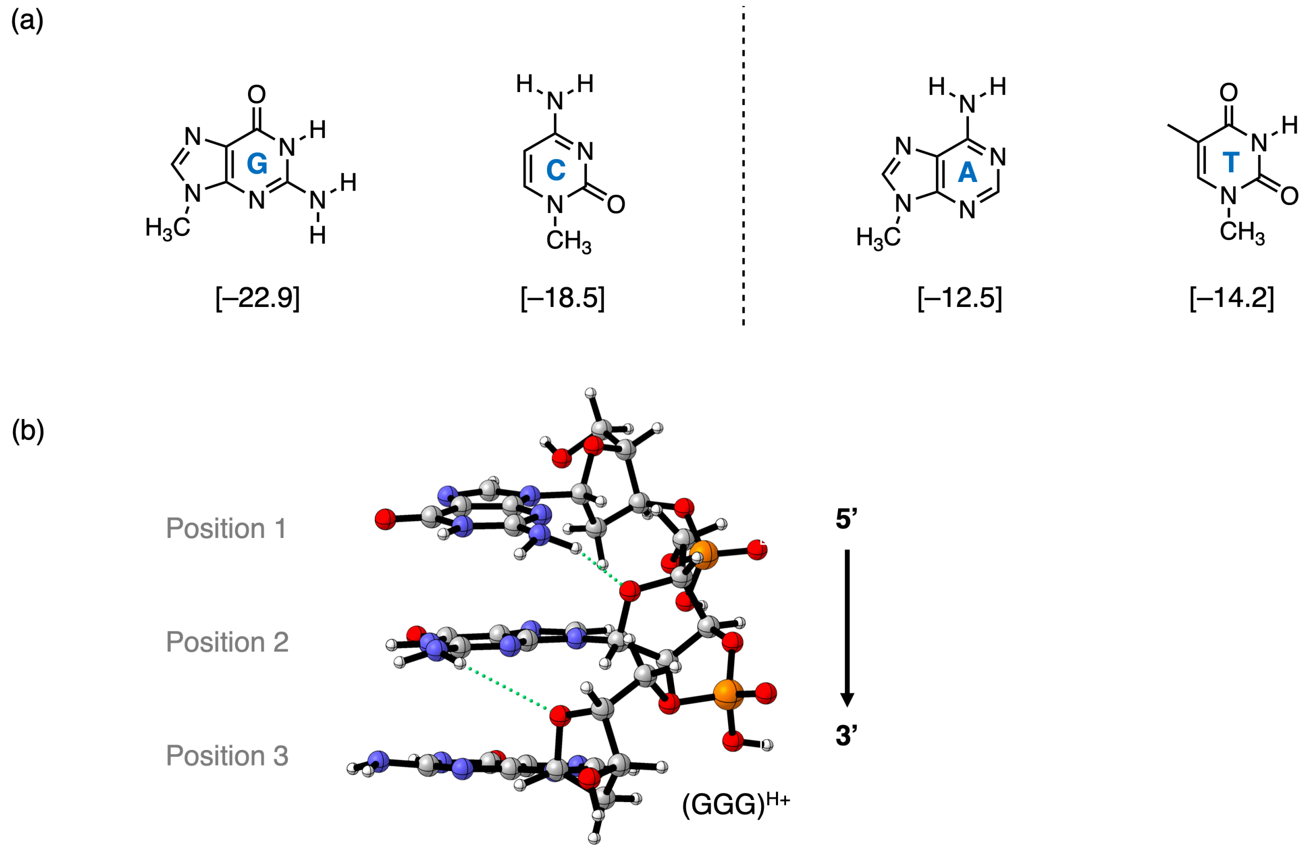

Figure S5. **(a)** Solvation energies (in kcal mol^{-1}) of isolated DNA bases guanine (G), cytosine (C), adenine (A), and thymine (T), calculated at BLYP-D3(BJ)/TZ2P using COSMO to simulated solvation in water. The DNA bases are substituted with a methyl group (CH_3) to simulate the sugar-phosphate backbone. **(b)** The NH_2 group of G can form intra-strand hydrogen bonds (green dotted lines) with the oxygen atom of the deoxyribose ring when in position 1 or 2 of a DNA triplet single-strand.

(a)

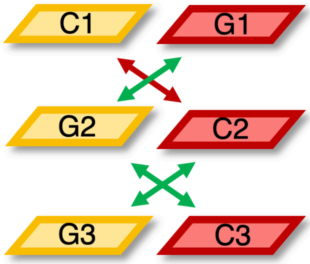

	ΔE_{int_cross}	ΔE_{Pauli}	ΔV_{elstat}	ΔE_{oi}	ΔE_{disp}
C1–C2	1.08	0.86	1.89	–0.42	–1.25
G1–G2	–5.10	7.36	–2.60	–1.89	–7.96
G2–C3	–5.03	0.44	–3.96	–0.59	–0.92
C2–G3	–5.43	3.26	–3.48	–1.45	–3.77
**Total**	**–14.48**	**11.92**	**–8.15**	**–4.35**	**–13.90**
C1–C3	0.54	0.01	0.66	–0.02	–0.11
G1–G3	–1.20	0.04	–0.76	–0.08	–0.40

(b)

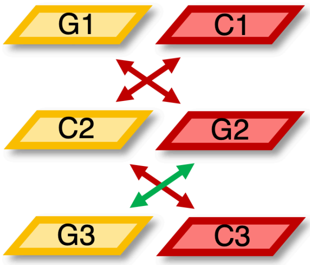

	ΔE_{int_cross}	ΔE_{Pauli}	ΔV_{elstat}	ΔE_{oi}	ΔE_{disp}
G1–G2	2.58	1.53	5.35	–1.26	–3.04
C1–C2	3.24	0.20	3.85	–0.34	–0.47
C2–C3	1.12	0.49	1.86	–0.31	–0.92
G2–G3	–5.42	6.75	–2.07	–1.93	–8.17
**Total**	**1.52**	**8.97**	**8.99**	**–3.84**	**–12.60**
G1–C3	–0.96	0.01	–0.82	–0.04	–0.11
C1–G3	0.29	0.02	0.55	–0.05	–0.23

Figure S6. Partitioning of the interaction energy between diagonal bases ΔE_{int_cross} (in kcal mol^{-1}) in the DNA duplex for **(a)** the CGG and **(b)** the GCG sequence, computed at BLYP-D3(BJ)/TZ2P.

(a)

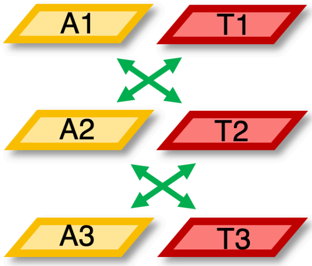

	ΔE_{int_cross}	ΔE_{Pauli}	ΔV_{elstat}	ΔE_{oi}	ΔE_{disp}
A1–T2	–2.04	0.99	–1.12	–0.41	–1.51
T1–A2	–2.40	2.57	–1.52	–0.72	–2.73
A2–T3	–1.65	0.81	–0.79	–0.33	–1.34
T2–A3	–2.17	2.27	–1.06	–0.62	–2.76
**Total**	**–8.26**	**6.64**	**–4.49**	**–2.08**	**–8.34**
A1–T3	0.01	0.02	0.15	–0.01	–0.15
T1–A3	–0.29	0.04	–0.06	–0.02	–0.24

(b)

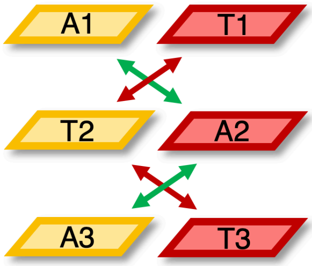

	ΔE_{int_cross}	ΔE_{Pauli}	ΔV_{elstat}	ΔE_{oi}	ΔE_{disp}
A1–A2	–1.02	2.68	0.08	–0.61	–3.18
T1–T2	0.86	0.20	1.31	–0.10	–0.56
T2–T3	0.71	0.17	1.10	–0.13	–0.43
A2–A3	–3.21	6.30	–1.62	–1.18	–6.70
**Total**	**–2.66**	**9.35**	**0.87**	**–2.02**	**–10.87**
A1–T3	0.06	0.01	0.18	–0.01	–0.12
T1–A3	–0.35	0.02	–0.14	–0.02	–0.22

Figure S7. Partitioning of the interaction energy between diagonal bases ΔE_{int_cross} (in kcal mol^{-1}) in the DNA duplex for **(a)** the AAA and **(b)** the ATA sequence, computed at BLYP-D3(BJ)/TZ2P.

Table S1. Sugar-phosphate backbone torsion angles (in °) of the 32 optimized d(DNA)$^{4-}$ structures, computed at BLYP-D3(BJ)/TZ2P using COSMO to simulate solvation in water. See Scheme 1 for torsion angle definitions.

	Base	α	β	γ	δ	ε	ζ	χ
**d(AAA)$^{4-}$**	1 A	–	–	50.1	143.2	162.9	−89.9	−100.9
	2 A	−66.7	−160.4	48.2	141.4	164.1	−92.9	−101.6
	3 A	−65.9	−162.1	50.1	144.2	–	–	−103.2
	4 T	–	–	49.5	140.4	164.6	−91.1	−109.4
	5 T	−64.1	−164.3	50.5	137.0	173.4	−94.2	−106.3
	6 T	−64.7	−175.6	52.2	135.0	–	–	−109.6
**d(AAC)$^{4-}$**	1 A	–	–	50.3	143.0	161.0	−89.7	−101.7
	2 A	−66.2	−157.2	47.6	141.9	170.7	−91.2	−102.5
	3 C	−64.3	−175.2	51.9	135.0	–	–	−111.6
	4 G	–	–	50.6	144.2	153.7	−86.4	−102.6
	5 T	−65.9	−149.9	49.0	139.3	172.2	−93.9	−104.2
	6 T	−63.7	−177.9	52.9	134.4	–	–	−107.9
**d(AAG)$^{4-}$**	1 A	–	–	50.3	142.9	161.0	−89.1	−100.3
	2 A	−66.2	−158.7	47.8	140.9	164.4	−90.7	−101.9
	3 G	−64.9	−166.3	50.1	141.6	–	–	−108.6
	4 C	–	–	50.4	142.0	161.2	−89.7	−109.4
	5 T	−65.1	−156.0	48.9	138.6	173.6	−94.1	−106.7
	6 T	−64.6	−177.1	52.5	134.6	–	–	−107.9
**d(AAT)$^{4-}$**	1 A	–	–	50.7	143.8	156.4	−86.7	−104.7
	2 A	−68.1	−149.6	47.6	142.5	172.2	−89.9	−102.4
	3 T	−62.9	−179.3	52.4	132.4	–	–	−110.7
	4 A	–	–	49.2	141.4	158.7	−87.7	−102.7
	5 T	−61.5	−165.7	51.8	136.3	172.4	−95.0	−108.5
	6 T	−64.5	−173.2	52.1	137.2	–	–	−108.3
**d(ACA)$^{4-}$**	1 A	–	–	49.0	142.3	168.4	−90.4	−103.6
	2 C	−63.4	−173.8	51.8	136.6	169.4	−98.8	−109.3
	3 A	−67.1	−156.6	47.4	146.0	–	–	−104.4
	4 T	–	–	50.1	143.1	166.2	−94.1	−110.2
	5 G	−68.3	−155.7	47.1	143.0	169.0	−91.0	−106.0
	6 T	−65.0	−169.5	51.1	135.6	–	–	−113.2

	Base	α	β	γ	δ	ε	ζ	χ
**d(ACC)$^{4-}$**	1 A	–	–	49.0	142.2	167.7	–94.5	–103.8
	2 C	–63.7	–172.3	51.9	139.5	168.2	–95.0	–107.9
	3 C	–67.1	–161.9	49.8	140.5	–	–	–117.5
	4 G	–	–	52.5	144.3	149.3	–81.7	–107.3
	5 G	–74.7	–133.2	46.3	144.3	168.2	–89.8	–98.4
	6 T	–62.9	–176.0	52.0	133.3	–	–	–111.4
**d(ACG)$^{4-}$**	1 A	–	–	49.3	142.4	167.4	–89.9	–102.7
	2 C	–63.7	–172.4	51.2	136.1	170.5	–100.4	–108.2
	3 G	–65.8	–160.5	47.9	145.7	–	–	–106.3
	4 C	–	–	50.7	143.5	165.0	–93.5	–109.1
	5 G	–68.2	–153.6	46.7	143.7	167.4	–90.4	–104.4
	6 T	–64.8	–169.2	51.3	136.5	–	–	–111.7
**d(ACT)$^{4-}$**	1 A	–	–	49.5	142.7	162.6	–89.7	–106.5
	2 C	–64.1	–163.2	50.3	139.5	175.6	–93.5	–109.4
	3 T	–67.0	–173.3	51.1	135.0	–	–	–111.0
	4 A	–	–	50.1	143.7	160.1	–89.2	–103.1
	5 G	–66.4	–157.9	48.4	141.5	170.5	–92.6	–106.3
	6 T	–65.1	–169.4	51.1	136.9	–	–	–111.1
**d(AGA)$^{4-}$**	1 A	–	–	49.6	142.8	165.3	–89.1	–100.1
	2 G	–64.3	–170.4	50.4	139.0	162.1	–92.0	–107.6
	3 A	–68.3	–149.0	47.2	146.6	–	–	–101.1
	4 T	–	–	49.7	142.7	167.8	–93.8	–111.5
	5 C	–67.2	–162.0	49.7	140.8	172.7	–92.1	–109.2
	6 T	–67.3	–168.6	50.3	135.8	–	–	–113.2
**d(AGC)$^{4-}$**	1 A	–	–	50.1	142.8	164.0	–90.2	–101.8
	2 G	–66.1	–165.1	49.3	139.7	167.7	–96.6	–107.2
	3 C	–64.9	–163.9	50.6	141.4	–	–	–115.2
	4 G	–	–	50.4	144.7	162.7	–90.6	–107.6
	5 C	–66.7	–156.8	49.0	140.1	174.6	–93.0	–108.9
	6 T	–65.8	–174.9	51.8	134.2	–	–	–109.4
**d(AGG)$^{4-}$**	1 A	–	–	49.6	142.6	166.4	–89.1	–99.9
	2 G	–63.9	–172.6	50.7	136.9	162.5	–92.9	–108.8

	Base	α	β	γ	δ	ε	ζ	χ
	3 G	−67.5	−150.7	47.0	146.0	–	–	−104.9
	4 C	–	–	50.7	143.7	167.5	−92.5	−112.5
	5 C	−69.2	−156.5	48.3	140.6	173.8	−92.1	−113.1
	6 T	−66.9	−169.7	50.3	136.3	–	–	−110.7
**d(ATA)$^{4-}$**	1 A	–	–	48.6	141.4	168.7	−91.2	−104.9
	2 T	−62.7	−173.7	51.8	135.0	166.9	−97.5	−108.2
	3 A	−66.4	−157.3	48.2	146.0	–	–	−104.3
	4 T	–	–	50.2	142.8	162.2	−90.4	−112.5
	5 A	−69.7	−146.9	45.6	142.9	173.5	−87.9	−102.3
	6 T	−62.9	−181.1	51.8	128.9	–	–	−111.3
**d(ATC)$^{4-}$**	1 A	–	–	48.6	141.5	166.8	−91.3	−103.2
	2 T	−63.6	−170.8	51.2	134.5	169.3	−98.2	−107.0
	3 C	−63.4	−170.0	51.5	140.1	–	–	−113.3
	4 G	–	–	52.4	144.4	150.0	−82.6	−107.6
	5 A	−74.2	−133.7	46.7	144.0	171.7	−88.0	−97.9
	6 T	−63.1	−180.9	51.7	129.8	–	–	−109.1
**d(ATG)$^{4-}$**	1 A	–	–	48.5	141.6	167.1	−90.2	−101.6
	2 T	−61.5	−176.1	52.7	133.8	164.8	−94.8	−109.4
	3 G	−66.9	−154.5	47.2	146.1	–	–	−105.1
	4 C	–	–	51.4	143.1	155.7	−86.4	−110.5
	5 A	−71.3	−139.9	45.9	143.7	169.1	−88.3	−101.2
	6 T	−62.6	−177.2	51.9	133.4	–	–	−109.2
**d(CAA)$^{4-}$**	1 C	–	–	50.2	143.0	166.8	−93.4	−110.4
	2 A	−69.5	−153.1	45.9	144.0	162.9	−89.7	−101.5
	3 A	−65.7	−164.0	50.3	142.6	–	–	−104.4
	4 T	–	–	49.0	140.1	170.8	−92.8	−110.4
	5 T	−65.1	−171.6	51.3	136.1	165.8	−95.5	−107.1
	6 G	−67.3	−156.9	47.9	145.4	–	–	−106.1
**d(CAC)$^{4-}$**	1 C	–	–	50.8	142.8	165.6	−93.3	−110.2
	2 A	−69.1	−151.6	45.6	143.5	169.2	−90.1	−103.5
	3 C	−64.3	−173.9	51.6	136.4	–	–	−110.3
	4 G	–	–	49.8	142.7	162.7	−90.9	−101.4

	Base	α	β	γ	δ	ε	ζ	χ
	5 T	−63.8	−165.4	50.9	138.1	165.3	−94.9	−107.0
	6 G	−66.7	−158.6	48.5	145.1	–	–	−104.1
**d(CAG)$^{4-}$**	1 C	–	–	50.6	142.7	165.3	−93.1	−110.2
	2 A	−67.5	−153.5	46.6	143.4	169.1	−88.5	−102.6
	3 G	−65.8	−172.7	49.8	137.2	–	–	−108.9
	4 C	–	–	49.4	140.8	167.7	−92.4	−110.6
	5 T	−65.3	−165.9	50.2	137.6	164.6	−94.5	−107.9
	6 G	−66.8	−156.6	48.1	145.6	–	–	−104.3
**d(CCA)$^{4-}$**	1 C	–	–	50.0	142.6	170.0	−95.3	−111.5
	2 C	−67.4	−164.7	50.2	142.0	166.1	−93.3	−109.8
	3 A	−70.8	−150.9	46.7	145.7	–	–	−102.8
	4 T	–	–	49.4	142.2	169.1	−93.3	−109.5
	5 G	−68.8	−162.9	48.5	141.4	157.3	−89.1	−104.5
	6 G	−68.3	−149.0	48.3	145.7	–	–	−103.3
**d(CGA)$^{4-}$**	1 C	–	–	49.9	141.6	168.4	−95.5	−108.2
	2 G	−65.5	−165.4	50.2	143.8	159.6	−89.6	−103.1
	3 A	−68.8	−150.5	47.6	145.8	–	–	−100.9
	4 T	–	–	49.3	141.8	170.1	−93.7	−110.5
	5 C	−66.7	−166.9	50.4	139.6	168.9	−96.1	−107.5
	6 G	−68.2	−156.1	47.4	145.5	–	–	−101.8
**d(CGG)$^{4-}$**	1 C	–	–	50.1	141.5	165.0	−92.7	−107.8
	2 G	−67.0	−159.9	48.6	143.3	158.6	−88.9	−102.9
	3 G	−69.1	−151.1	47.5	145.3	–	–	−103.3
	4 C	–	–	50.2	142.2	167.8	−92.8	−110.5
	5 C	−67.3	−162.3	49.3	140.2	166.1	−93.8	−108.8
	6 G	−68.2	−154.2	47.3	145.7	–	–	−101.5
**d(CTA)$^{4-}$**	1 C	–	–	49.7	141.0	167.7	−92.8	−110.3
	2 T	−65.5	−165.1	50.4	139.0	164.6	−95.1	−105.9
	3 A	−66.6	−156.8	48.7	146.0	–	–	−102.5
	4 T	–	–	49.9	142.6	165.6	−93.4	−111.4
	5 A	−67.7	−153.9	47.0	143.1	165.8	−88.5	−100.6
	6 G	−64.5	−171.5	50.5	138.7	–	–	−109.0

	Base	α	β	γ	δ	ε	ζ	χ
**d(GAA)$^{4-}$**	1 G	–	–	50.7	144.5	160.4	–88.8	–104.8
	2 A	–69.4	–150.0	46.2	143.3	162.2	–89.7	–100.6
	3 A	–65.3	–163.6	50.2	143.0	–	–	–103.7
	4 T	–	–	49.2	140.5	169.3	–93.2	–110.2
	5 T	–65.7	–168.8	50.8	136.1	169.9	–96.2	–106.9
	6 C	–64.9	–169.0	51.2	139.6	–	–	–113.6
**d(GAC)$^{4-}$**	1 G	–	–	51.2	144.5	157.9	–87.9	–106.1
	2 A	–70.0	–146.3	46.2	143.1	169.8	–89.6	–102.3
	3 C	–63.4	–176.3	51.7	134.9	–	–	–110.6
	4 G	–	–	49.8	142.6	161.5	–90.8	–101.7
	5 T	–64.2	–163.4	50.5	138.4	169.3	–96.3	–106.9
	6 C	–64.8	–169.6	51.5	139.8	–	–	–110.7
**d(GAG)$^{4-}$**	1 G	–	–	51.2	144.3	157.2	–87.5	–104.5
	2 A	–68.2	–148.7	47.3	142.9	169.9	–88.6	–101.1
	3 G	–65.2	–174.6	49.6	136.2	–	–	–109.3
	4 C	–	–	49.4	140.8	166.6	–92.5	–110.4
	5 T	–65.7	–163.9	49.8	137.6	169.3	–96.7	–107.6
	6 C	–64.6	–168.6	51.2	140.4	–	–	–111.3
**d(GCA)$^{4-}$**	1 G	–	–	49.7	143.6	166.0	–92.5	–105.7
	2 C	–65.9	–164.6	50.4	141.4	165.2	–93.4	–109.0
	3 A	–69.1	–152.3	47.0	145.6	–	–	–101.9
	4 T	–	–	49.7	142.5	167.7	–94.4	–109.7
	5 G	–69.0	–160.0	48.0	142.6	167.0	–92.8	–106.5
	6 C	–66.1	–164.0	50.5	140.0	–	–	–116.2
**d(GCG)$^{4-}$**	1 G	–	–	50.2	143.6	162.3	–89.7	–103.8
	2 C	–66.7	–159.6	48.8	140.5	165.0	–93.9	–107.0
	3 G	–67.8	–156.2	47.9	145.6	–	–	–102.9
	4 C	–	–	50.3	142.5	165.4	–93.8	–108.3
	5 G	–68.8	–157.1	47.6	143.4	163.3	–91.4	–104.6
	6 C	–65.8	–161.2	50.1	141.1	–	–	–113.9
**d(GGA)$^{4-}$**	1 G	–	–	50.3	143.9	162.6	–90.0	–102.7

	Base	α	β	γ	δ	ε	ζ	χ
	2 G	–66.5	–160.9	48.8	143.1	161.5	–90.6	–106.1
	3 A	–69.1	–150.1	47.5	146.0	–	–	–101.3
	4 T	–	–	49.6	142.2	170.2	–95.7	–110.8
	5 C	–67.5	–166.9	50.7	140.3	170.0	–93.0	–108.7
	6 C	–68.3	–162.8	49.5	139.4	–	–	–118.6
**d(GGC)$^{4-}$**	1 G	–	–	50.7	143.8	159.7	–88.9	–103.3
	2 G	–67.8	–154.5	46.9	142.9	165.3	–92.9	–106.6
	3 C	–65.7	–162.8	50.7	141.3	–	–	–114.6
	4 G	–	–	50.2	144.1	164.3	–92.5	–104.1
	5 C	–67.0	–161.8	49.6	140.4	168.7	–94.6	–108.4
	6 C	–66.3	–164.7	50.1	140.5	–	–	–114.4
**d(GGG)$^{4-}$**	1 G	–	–	50.5	143.7	159.1	–87.9	–100.8
	2 G	–67.4	–156.5	47.5	142.6	160.9	–90.6	–104.5
	3 G	–68.6	–152.8	47.7	145.2	–	–	–104.6
	4 C	–	–	50.1	142.3	167.5	–94.0	–110.8
	5 C	–67.9	–161.9	49.3	140.1	169.3	–93.6	–109.8
	6 C	–66.6	–163.9	49.8	140.2	–	–	–116.9
**d(GTA)$^{4-}$**	1 G	–	–	49.4	142.7	164.6	–91.3	–104.4
	2 T	–64.5	–164.7	50.7	138.7	164.0	–94.5	–105.9
	3 A	–66.4	–156.9	48.7	145.9	–	–	–101.5
	4 T	–	–	49.9	142.6	164.7	–92.4	–111.7
	5 A	–69.0	–150.6	45.7	143.0	168.3	–89.3	–101.7
	6 C	–63.0	–175.7	51.9	135.4	–	–	–111.2
**d(TAA)$^{4-}$**	1 T	–	–	50.2	142.4	165.7	–92.1	–111.1
	2 A	–68.9	–153.6	46.3	143.5	163.3	–89.8	–101.5
	3 A	–66.0	–163.5	50.1	143.0	–	–	–104.0
	4 T	–	–	49.1	140.4	170.8	–93.1	–110.5
	5 T	–65.1	–171.4	51.3	137.2	163.4	–93.3	–106.9
	6 A	–69.2	–151.5	47.5	145.7	–	–	–103.9
**d(TCA)$^{4-}$**	1 T	–	–	50.0	142.2	171.1	–95.4	–110.8
	2 C	–67.6	–167.3	50.6	141.4	167.0	–95.2	–106.9
	3 A	–70.9	–152.2	46.9	145.6	–	–	–105.7

Base	α	β	γ	δ	ε	ζ	χ
4 T	–	–	49.3	142.4	169.7	–93.1	–110.0
5 G	–68.9	–163.4	48.7	141.7	157.9	–89.6	–104.5
6 A	–69.0	–147.7	48.3	146.0	–	–	–101.1

Table S2. Hydrogen-bond distances (in Å) of the 32 optimized d(DNA)$^{4–}$ structures, computed at BLYP-D3(BJ)/TZ2P using COSMO to simulate solvation in water. For atom labels, see Figure 1b.

**d(AAA)$^{4–}$**	1 A–T	N6···O4	2.88	N1···N3	2.88		
	2 A–T	N6···O4	2.87	N1···N3	2.84		
	3 A–T	N6···O4	2.94	N1···N3	2.83		
**d(AAC)$^{4–}$**	1 A–T	N6···O4	2.87	N1···N3	2.88		
	2 A–T	N6···O4	2.89	N1···N3	2.82		
	3 C–G	O2···N2	2.84	N3···N1	2.93	N4···O6	2.86
**d(AAG)$^{4–}$**	1 A–T	N6···O4	2.87	N1···N3	2.88		
	2 A–T	N6···O4	2.88	N1···N3	2.83		
	3 G–C	O6···N4	2.88	N1···N3	2.92	N2···O2	2.81
**d(AAT)$^{4–}$**	1 A–T	N6···O4	2.87	N1···N3	2.87		
	2 A–T	N6···O4	2.93	N1···N3	2.82		
	3 T–A	N3···N1	2.84	O4···N6	2.92		
**d(ACA)$^{4–}$**	1 A–T	N6···O4	2.86	N1···N3	2.88		
	2 C–G	O2···N2	2.81	N3···N1	2.91	N4···O6	2.86
	3 A–T	N6···O4	2.91	N1···N3	2.83		
**d(ACC)$^{4–}$**	1 A–T	N6···O4	2.87	N1···N3	2.90		
	2 C–G	O2···N2	2.81	N3···N1	2.90	N4···O6	2.85
	3 C–G	O2···N2	2.88	N3···N1	2.92	N4···O6	2.83
**d(ACG)$^{4–}$**	1 A–T	N6···O4	2.86	N1···N3	2.88		
	2 C–G	O2···N2	2.8	N3···N1	2.91	N4···O6	2.86
	3 G–C	O6···N4	2.85	N1···N3	2.91	N2···O2	2.81
**d(ACT)$^{4–}$**	1 A–T	N6···O4	2.87	N1···N3	2.87		
	2 C–G	O2···N2	2.81	N3···N1	2.92	N4···O6	2.88

	3 T–A	N3···N1	2.86	O4···N6	2.86		
**d(AGA)$^{4-}$**	1 A–T	N6···O4	2.86	N1···N3	2.87		
	2 G–C	O6···N4	2.83	N1···N3	2.89	N2···O2	2.83
	3 A–T	N6···O4	2.94	N1···N3	2.83		
**d(AGC)$^{4-}$**	1 A–T	N6···O4	2.86	N1···N3	2.88		
	2 G–C	O6···N4	2.85	N1···N3	2.91	N2···O2	2.82
	3 C–G	O2···N2	2.86	N3···N1	2.92	N4···O6	2.85
**d(AGG)$^{4-}$**	1 A–T	N6···O4	2.85	N1···N3	2.88		
	2 G–C	O6···N4	2.83	N1···N3	2.89	N2···O2	2.82
	3 G–C	O6···N4	2.86	N1···N3	2.92	N2···O2	2.82
**d(ATA)$^{4-}$**	1 A–T	N6···O4	2.9	N1···N3	2.86		
	2 T–A	N3···N1	2.82	O4···N6	2.89		
	3 A–T	N6···O4	2.91	N1···N3	2.83		
**d(ATC)$^{4-}$**	1 A–T	N6···O4	2.9	N1···N3	2.86		
	2 T–A	N3···N1	2.82	O4···N6	2.9		
	3 C–G	O2···N2	2.87	N3···N1	2.92	N4···O6	2.84
**d(ATG)$^{4-}$**	1 A–T	N6···O4	2.9	N1···N3	2.86		
	2 T–A	N3···N1	2.82	O4···N6	2.89		
	3 G–C	O6···N4	2.86	N1···N3	2.91	N2···O2	2.80
**d(CAA)$^{4-}$**	1 C–G	O2···N2	2.82	N3···N1	2.91	N4···O6	2.86
	2 A–T	N6···O4	2.85	N1···N3	2.85		
	3 A–T	N6···O4	2.92	N1···N3	2.84		
**d(CAC)$^{4-}$**	1 C–G	O2···N2	2.82	N3···N1	2.92	N4···O6	2.86
	2 A–T	N6···O4	2.86	N1···N3	2.85		
	3 C–G	O2···N2	2.84	N3···N1	2.93	N4···O6	2.87
**d(CAG)$^{4-}$**	1 C–G	O2···N2	2.82	N3···N1	2.91	N4···O6	2.86
	2 A–T	N6···O4	2.86	N1···N3	2.84		
	3 G–C	O6···N4	2.86	N1···N3	2.92	N2···O2	2.82

**d(CCA)$^{4-}$**	1 C–G	O2···N2	2.83	N3···N1	2.92	N4···O6	2.86
	2 C–G	O2···N2	2.82	N3···N1	2.89	N4···O6	2.83
	3 A–T	N6···O4	2.9	N1···N3	2.84		
**d(CGA)$^{4-}$**	1 C–G	O2···N2	2.84	N3···N1	2.91	N4···O6	2.85
	2 G–C	O6···N4	2.84	N1···N3	2.90	N2···O2	2.82
	3 A–T	N6···O4	2.92	N1···N3	2.84		
**d(CGG)$^{4-}$**	1 C–G	O2···N2	2.83	N3···N1	2.91	N4···O6	2.85
	2 G–C	O6···N4	2.84	N1···N3	2.90	N2···O2	2.81
	3 G–C	O6···N4	2.86	N1···N3	2.92	N2···O2	2.82
**d(CTA)$^{4-}$**	1 C–G	O2···N2	2.82	N3···N1	2.92	N4···O6	2.87
	2 T–A	N3···N1	2.83	O4···N6	2.86		
	3 A–T	N6···O4	2.91	N1···N3	2.83		
**d(GAA)$^{4-}$**	1 G–C	O6···N4	2.85	N1···N3	2.92	N2···O2	2.86
	2 A–T	N6···O4	2.87	N1···N3	2.84		
	3 A–T	N6···O4	2.92	N1···N3	2.84		
**d(GAC)$^{4-}$**	1 G–C	O6···N4	2.85	N1···N3	2.92	N2···O2	2.86
	2 A–T	N6···O4	2.88	N1···N3	2.84		
	3 C–G	O2···N2	2.84	N3···N1	2.93	N4···O6	2.87
**d(GAG)$^{4-}$**	1 G–C	O6···N4	2.84	N1···N3	2.92	N2···O2	2.86
	2 A–T	N6···O4	2.88	N1···N3	2.83		
	3 G–C	O6···N4	2.86	N1···N3	2.92	N2···O2	2.82
**d(GCA)$^{4-}$**	1 G–C	O6···N4	2.86	N1···N3	2.92	N2···O2	2.86
	2 C–G	O2···N2	2.81	N3···N1	2.90	N4···O6	2.84
	3 A–T	N6···O4	2.91	N1···N3	2.84		
**d(GCG)$^{4-}$**	1 G–C	O6···N4	2.86	N1···N3	2.92	N2···O2	2.85
	2 C–G	O2···N2	2.81	N3···N1	2.90	N4···O6	2.85
	3 G–C	O6···N4	2.86	N1···N3	2.91	N2···O2	2.81
**d(GGA)$^{4-}$**	1 G–C	O6···N4	2.84	N1···N3	2.92	N2···O2	2.87
	2 G–C	O6···N4	2.83	N1···N3	2.90	N2···O2	2.84

	3 A–T	N6⋯O4	2.93	N1⋯N3	2.84		
**d(GGC)$^{4-}$**	1 G–C	O6⋯N4	2.84	N1⋯N3	2.92	N2⋯O2	2.85
	2 G–C	O6⋯N4	2.84	N1⋯N3	2.91	N2⋯O2	2.82
	3 C–G	O2⋯N2	2.85	N3⋯N1	2.92	N4⋯O6	2.86
**d(GGG)$^{4-}$**	1 G–C	O6⋯N4	2.84	N1⋯N3	2.91	N2⋯O2	2.86
	2 G–C	O6⋯N4	2.83	N1⋯N3	2.90	N2⋯O2	2.83
	3 G–C	O6⋯N4	2.86	N1⋯N3	2.91	N2⋯O2	2.82
**d(GTA)$^{4-}$**	1 G–C	O6⋯N4	2.87	N1⋯N3	2.93	N2⋯O2	2.84
	2 T–A	N3⋯N1	2.84	O4⋯N6	2.86		
	3 A–T	N6⋯O4	2.91	N1⋯N3	2.83		
**d(TAA)$^{4-}$**	1 T–A	N3⋯N1	2.85	O4⋯N6	2.90		
	2 A–T	N6⋯O4	2.87	N1⋯N3	2.83		
	3 A–T	N6⋯O4	2.93	N1⋯N3	2.84		
**d(TCA)$^{4-}$**	1 T–A	N3⋯N1	2.84	O4⋯N6	2.91		
	2 C–G	O2⋯N2	2.82	N3⋯N1	2.89	N4⋯O6	2.83
	3 A–T	N6⋯O4	2.89	N1⋯N3	2.85		

Table S3. Formation energy Δ*E* (in kcal mol^{-1}) in decreasing order of the 32 double-stranded B-DNA structures d(DNA)$^{4-}$, d(DNA)$^{H+}$ and d(DNA)$^{Na+}$, computed at BLYP-D3(BJ)/TZ2P using COSMO to simulate solvation in water.

**d(DNA)$^{4-}$**	**Δ*E***	**d(DNA)$^{H+}$**	**Δ*E***	**d(DNA)$^{Na+}$**	**Δ*E***
d(CGG)$^{4-}$	–44.84	d(CGG)$^{H+}$	–47.93	d(CGG)$^{Na+}$	–51.84
d(GCG)$^{4-}$	–44.16	d(GCG)$^{H+}$	–46.56	d(GCG)$^{Na+}$	–47.97
d(GGG)$^{4-}$	–42.23	d(GGG)$^{H+}$	–44.86	d(CCA)$^{Na+}$	–45.97
d(ACG)$^{4-}$	–41.00	d(CGA)$^{H+}$	–44.53	d(GGG)$^{Na+}$	–45.74
d(GGC)$^{4-}$	–40.94	d(GGC)$^{H+}$	–44.02	d(CAG)$^{Na+}$	–45.59
d(CCA)$^{4-}$	–39.59	d(AGG)$^{H+}$	–43.48	d(GGC)$^{Na+}$	–44.44
d(CGA)$^{4-}$	–39.48	d(CAG)$^{H+}$	–43.28	d(CGA)$^{Na+}$	–44.20
d(CAC)$^{4-}$	–39.34	d(ACG)$^{H+}$	–43.14	d(ACG)$^{Na+}$	–44.05
d(CAG)$^{4-}$	–39.10	d(CAC)$^{H+}$	–42.63	d(AGG)$^{Na+}$	–43.47
d(GCA)$^{4-}$	–38.56	d(ACC)$^{H+}$	–42.48	d(CAC)$^{Na+}$	–43.21
d(AGG)$^{4-}$	–38.43	d(CCA)$^{H+}$	–42.19	d(GCA)$^{Na+}$	–42.33
d(GAG)$^{4-}$	–37.54	d(GCA)$^{H+}$	–41.05	d(ACC)$^{Na+}$	–41.56
d(ACC)$^{4-}$	–37.37	d(GAG)$^{H+}$	–40.92	d(CAA)$^{Na+}$	–41.05
d(GGA)$^{4-}$	–36.96	d(GGA)$^{H+}$	–39.80	d(GGA)$^{Na+}$	–40.76
d(AGC)$^{4-}$	–36.18	d(GAC)$^{H+}$	–39.46	d(GAC)$^{Na+}$	–40.05
d(CAA)$^{4-}$	–35.87	d(AGC)$^{H+}$	–39.41	d(ATG)$^{Na+}$	–39.81
d(ACA)$^{4-}$	–35.60	d(ACA)$^{H+}$	–39.03	d(AGC)$^{Na+}$	–39.68
d(GAC)$^{4-}$	–35.48	d(CAA)$^{H+}$	–38.66	d(GAG)$^{Na+}$	–39.06
d(ATG)$^{4-}$	–35.20	d(ATG)$^{H+}$	–38.49	d(CTA)$^{Na+}$	–39.03
d(AAG)$^{4-}$	–34.85	d(AAG)$^{H+}$	–38.34	d(ACA)$^{Na+}$	–38.55
d(CTA)$^{4-}$	–34.03	d(AAC)$^{H+}$	–37.80	d(TCA)$^{Na+}$	–38.46
d(ACT)$^{4-}$	–33.76	d(CTA)$^{H+}$	–37.20	d(AAG)$^{Na+}$	–38.03
d(TCA)$^{4-}$	–33.54	d(ACT)$^{H+}$	–36.89	d(AAC)$^{Na+}$	–36.40
d(GTA)$^{4-}$	–32.58	d(GAA)$^{H+}$	–36.47	d(AGA)$^{Na+}$	–36.06
d(AAC)$^{4-}$	–32.48	d(GTA)$^{H+}$	–36.47	d(ACT)$^{Na+}$	–35.80
d(AGA)$^{4-}$	–32.19	d(TCA)$^{H+}$	–36.40	d(GTA)$^{Na+}$	–35.42
d(GAA)$^{4-}$	–32.05	d(ATC)$^{H+}$	–35.93	d(ATC)$^{Na+}$	–35.39

d(DNA)$^{4-}$	Δ*E*	d(DNA)$^{H+}$	Δ*E*	d(DNA)$^{Na+}$	Δ*E*
d(ATC)$^{4-}$	–31.54	d(AGA)$^{H+}$	–35.29	d(TAA)$^{Na+}$	–34.92
d(AAA)$^{4-}$	–30.02	d(AAA)$^{H+}$	–33.18	d(GAA)$^{Na+}$	–33.53
d(ATA)$^{4-}$	–28.96	d(ATA)$^{H+}$	–32.40	d(ATA)$^{Na+}$	–33.20
d(AAT)$^{4-}$	–28.93	d(AAT)$^{H+}$	–31.99	d(AAT)$^{Na+}$	–32.91
d(TAA)$^{4-}$	–28.83	d(TAA)$^{H+}$	–31.98	d(AAA)$^{Na+}$	–32.58

Table S4. Energy decomposition analysis (EDA) of the interaction energy ΔE_{int} (in kcal mol^{-1}) for the formation of the d(DNA)$^{H+}$/d(DNA)$^{4-}$ duplex from the two single-strands, computed at BLYP-D3(BJ)/TZ2P.

d(DNA)$^{H+}$	ΔE_{int}	ΔE_{Pauli}	ΔV_{elstat}	ΔE_{oi}	ΔE_{disp}
d(CGG)$^{H+}$	–118.7	155.4	–144.2	–94.7	–35.2
d(GCG)$^{H+}$	–101.9	149.6	–133.8	–84.5	–33.2
d(GGG)$^{H+}$	–117.4	147.4	–139.6	–94.8	–30.5
d(GGC)$^{H+}$	–101.8	141.5	–130.7	–84.8	–27.9
d(AAA)$^{H+}$	–64.7	112.3	–90.6	–60.7	-25.6
d(ATA)$^{H+}$	–59.6	116.5	–90.2	–57.8	-28.2
d(AAT)$^{H+}$	–60.8	111.4	–89.0	–58.4	-24.9
d(TAA)$^{H+}$	–63.2	115.8	–91.2	–59.2	-28.7
**d(DNA)$^{4-}$**					
d(CGG)$^{4-}$	–39.0	161.4	–57.9	–107.5	–35.1

Table S5. Desolvation energy $-\Delta E_{solv}$ (in kcal mol^{-1}) of complementary all-GC and all-AT (DNA)$^{H+}$ single-strands (ss1 and ss2), computed at BLYP-D3(BJ)/TZ2P using COSMO to simulate solvation in water.

ss1	$-\Delta E_{solv,ss1}$	ss2	$-\Delta E_{solv,ss2}$	$-\Delta E_{solv,ss1+ss2}$ (total)
(CGG)$^{H+}$	79.1	(CCG)$^{H+}$	82.8	161.8
(GCG)$^{H+}$	74.5	(CGC)$^{H+}$	71.9	146.4
(GGG)$^{H+}$	83.8	(CCC)$^{H+}$	84.9	168.7
(GGC)$^{H+}$	75.5	(GCC)$^{H+}$	77.8	153.3
(AAA)$^{H+}$	59.7	(TTT)$^{H+}$	63.3	122.9
(ATA)$^{H+}$	60.6	(TAT)$^{H+}$	60.9	121.5
(AAT)$^{H+}$	59.4	(ATT)$^{H+}$	61.5	121.0
(TAA)$^{H+}$	60.6	(TTA)$^{H+}$	62.6	123.1

Table S6. Interaction energy for the interaction between two sugar-phosphate backbones $\Delta E_{int\_backbones}$ (in kcal mol^{-1}) in the d(DNA)$^{H+}$ geometry, in the absence of the nucleobases, computed at BLYP-D3(BJ)/TZ2P for the all-GC and all-AT sequences.

d(DNA)$^{H+}$	$\Delta E_{int\_backbones}$
d(CGG)$^{H+}$	–0.1
d(GCG)$^{H+}$	–0.1
d(GGG)$^{H+}$	–0.1
d(GGC)$^{H+}$	–0.1
d(AAA)$^{H+}$	0.1
d(ATA)$^{H+}$	–0.2
d(AAT)$^{H+}$	0.2
d(TAA)$^{H+}$	–0.1

Table S7. Cartesian coordinates (in Å) and total bond energies E (in kcal mol^{-1}) of the optimized single- ((DNA)$^{2-}$, (DNA)$^{Na+}$, and (DNA)$^{H+}$) and double-stranded (d(DNA)$^{4-}$, d(DNA)$^{Na+}$, and d(DNA)$^{H+}$) trideoxyribonucleoside diphosphate DNA structures, computed at BLYP-D3(BJ)/TZ2P using COSMO to simulate solvation in water.

d(AAA)$^{4-}$

E: –27763.35 kcal mol^{-1}

```
O     -0.168849   -0.388899   -0.471706
C     -0.111135    0.050049    0.907282
C      1.317783   -0.020266    1.430593
O      1.760883   -1.407227    1.603483
C      2.793162   -1.738889    0.642664
C      2.382358    0.647025    0.539466
C      2.933255   -0.521930   -0.283544
O      3.350427    1.264382    1.437469
H     -1.107056   -0.465280   -0.718251
H     -0.747850   -0.582983    1.538308
H     -0.456116    1.090877    0.994058
H      1.326155    0.444112    2.422445
H      3.716994   -1.961915    1.185455
H      1.960293    1.427615   -0.098894
H      2.308190   -0.636802   -1.169836
H      3.958192   -0.360287   -0.607794
N      2.446569   -2.973363   -0.061371
C      1.548446   -3.132520   -1.110205
N      1.469087   -4.382716   -1.537562
C      2.354698   -5.091713   -0.727492
C      2.720281   -6.457716   -0.687320
N      2.212064   -7.389964   -1.514378
N      3.630133   -6.833432    0.255728
C      4.133084   -5.910494    1.099324
N      3.854903   -4.597890    1.147734
C      2.962289   -4.237904    0.205233
H      0.966975   -2.300694   -1.481217
H      2.584935   -8.347856   -1.501020
H      1.628814   -7.095651   -2.286821
H      4.861011   -6.286386    1.815559
P      4.858159    1.732958    0.953860
O      4.912098    1.961156   -0.537385
O      5.261886    2.818688    1.926205
O      5.746147    0.371605    1.230846
C      5.931319   -0.054039    2.607085
C      7.123426   -0.991432    2.719004
O      6.821980   -2.300890    2.138846
C      7.669468   -2.555900    0.993113
C      8.401707   -0.493921    2.015867
C      8.362216   -1.225315    0.670969
O      9.523036   -0.857827    2.872257
H      5.026189   -0.565930    2.956192
H      6.110045    0.822180    3.241087
H      7.323451   -1.144601    3.785366
H      8.383339   -3.347878    1.243042
H      8.404456    0.591321    1.885761
H      7.754519   -0.631694   -0.013784
H      9.348473   -1.354409    0.232532
N      6.865940   -3.085548   -0.103385
C      6.037549   -2.381408   -0.967858
N      5.449349   -3.155483   -1.865592
C      5.908708   -4.441520   -1.581198
C      5.656591   -5.699238   -2.179295
N      4.834382   -5.877941   -3.224993
N      6.298259   -6.780166   -1.651319
C      7.107480   -6.623497   -0.587906
N      7.400553   -5.484816    0.061730
C      6.782589   -4.420389   -0.483384
H      5.907712   -1.313045   -0.874875
H      4.679200   -6.819487   -3.607044
H      4.355379   -5.083007   -3.625107
H      7.572545   -7.534751   -0.221260
P     11.093085   -0.868718    2.360139
O     11.275911   -0.034558    1.115300
O     11.914752   -0.608086    3.603228
O     11.283231   -2.434209    1.881497
C     11.214840   -3.472025    2.895026
C     11.835027   -4.758087    2.372267
O     10.994881   -5.356436    1.335667
C     11.690596   -5.373359    0.067882
C     13.232002   -4.592092    1.748277
C     12.939694   -4.493664    0.245990
```

O	13.996735	−5.781001	2.083720
H	10.166540	−3.650166	3.163797
H	11.761101	−3.150186	3.789424
H	11.883585	−5.466572	3.207979
H	11.945120	−6.407070	−0.185437
H	13.741599	−3.700082	2.126954
H	12.719338	−3.454979	−0.008469
H	13.773877	−4.839033	−0.369417
H	14.872663	−5.694780	1.664701
N	10.787504	−4.921900	−0.985548
C	10.345454	−3.627587	−1.227243
N	9.493423	−3.551996	−2.236536
C	9.356178	−4.865518	−2.684758
C	8.595514	−5.448099	−3.725940
N	7.800165	−4.751090	−4.560624
N	8.693723	−6.797432	−3.890095
C	9.480287	−7.518646	−3.071059
N	10.241107	−7.069450	−2.059365
C	10.146066	−5.733251	−1.912400
H	10.682601	−2.801182	−0.618041
H	7.192957	−5.256177	−5.216839
H	7.621227	−3.773524	−4.370970
H	9.485406	−8.590072	−3.257352
O	6.231258	−11.573972	−10.696235
C	7.470828	−12.317134	−10.809895
C	7.904262	−12.832078	−9.443761
O	8.365456	−11.731386	−8.591327
C	7.521276	−11.603019	−7.421369
C	6.809967	−13.565374	−8.640241
C	6.299496	−12.492439	−7.676285
O	7.449686	−14.699083	−7.984073
H	6.069081	−11.136574	−11.550194
H	8.265299	−11.678015	−11.215307
H	7.339474	−13.180625	−11.477858
H	8.754808	−13.504930	−9.597827
H	8.078343	−11.909264	−6.534311
H	6.011973	−13.946670	−9.282262
H	5.502802	−11.935135	−8.171304
H	5.899949	−12.919467	−6.760909
N	7.197786	−10.181684	−7.203323
C	7.772299	−9.517654	−6.106676
O	8.562850	−10.053919	−5.324256
N	7.366350	−8.207259	−5.957522
C	6.528121	−7.485747	−6.799344
O	6.270057	−6.287696	−6.546597
C	6.023790	−8.208826	−7.955791
C	5.143956	−7.493376	−8.944637
C	6.394135	−9.507123	−8.105403
H	7.814679	−7.685354	−5.156188
H	5.661532	−6.626897	−9.374692
H	4.236156	−7.115805	−8.457888
H	4.849993	−8.163251	−9.757870
H	6.095563	−10.091168	−8.970465
P	6.739675	−15.529091	−6.742825
O	5.243349	−15.333869	−6.733707
O	7.350989	−16.911434	−6.796106
O	7.303329	−14.718016	−5.421090
C	8.734163	−14.752150	−5.170611
C	9.030103	−14.303776	−3.749900
O	8.773743	−12.873592	−3.603825
C	7.801924	−12.636577	−2.561129
C	8.200697	−15.017734	−2.661469
C	7.127858	−13.988212	−2.291678
O	9.113190	−15.337106	−1.572193
H	9.244528	−14.090536	−5.881060
H	9.107311	−15.774039	−5.306812
H	10.096648	−14.476120	−3.563008
H	8.297795	−12.246593	−1.669745
H	7.757416	−15.946888	−3.026739
H	6.265594	−14.141052	−2.942682
H	6.804255	−14.088361	−1.258275
N	6.890267	−11.562651	−2.993011
C	7.025143	−10.293609	−2.399427
O	7.842797	−10.043174	−1.511893
N	6.144488	−9.341400	−2.873095
C	5.181167	−9.510189	−3.859646
O	4.457392	−8.553586	−4.205309
C	5.102841	−10.839034	−4.444557
C	4.044788	−11.100229	−5.480261
C	5.975866	−11.782207	−4.004373
H	6.214484	−8.388921	−2.417719
H	3.056650	−10.843530	−5.080300
H	4.033769	−12.150576	−5.779606
H	4.197864	−10.480221	−6.371619
H	6.004058	−12.778427	−4.432120
P	8.575653	−15.967827	−0.141388
O	7.192877	−16.554044	−0.292377
O	9.727142	−16.796439	0.385269
O	8.392919	−14.617045	0.785627
C	9.590001	−13.861203	1.108825
C	9.246205	−12.723411	2.051283
O	8.435872	−11.736297	1.345097
C	7.277956	−11.405200	2.142498
C	8.457895	−13.129427	3.318800
C	7.034530	−12.626842	3.040714
O	9.074574	−12.446538	4.443627
H	10.026270	−13.455044	0.188170
H	10.324819	−14.518425	1.588802
H	10.187350	−12.248239	2.358307
H	7.469394	−10.505427	2.730862
H	8.485621	−14.211143	3.481774
H	6.476281	−13.402127	2.509933
H	6.493831	−12.367681	3.954505
H	8.594804	−12.715199	5.248581
N	6.177498	−11.059125	1.235960
C	5.754392	−9.722361	1.163786
O	6.237493	−8.816944	1.852418
N	4.735099	−9.493175	0.262050
C	4.100127	−10.436425	−0.539423
O	3.171709	−10.092335	−1.301929
C	4.594768	−11.798210	−0.429209
C	3.939811	−12.876561	−1.246987
C	5.614565	−12.032546	0.435630
H	4.368182	−8.506117	0.235702
H	2.870893	−12.939207	−1.010028
H	4.393901	−13.850156	−1.048845
H	4.016086	−12.660944	−2.318399
H	6.052796	−13.017244	0.546961

d(AAC)$^{4-}$

E: −27674.98 kcal mol^{-1}

O	−0.150331	−0.358262	−0.428478
C	−0.086570	0.065194	0.955120
C	1.342771	−0.024064	1.474550
O	1.773475	−1.417668	1.625318
C	2.804232	−1.742210	0.660682
C	2.411602	0.647550	0.590968
C	2.949313	−0.514374	−0.249767
O	3.384854	1.247120	1.496035
H	−1.089463	−0.421127	−0.675356
H	−0.727833	−0.569095	1.580312
H	−0.422232	1.108022	1.053979
H	1.357147	0.425300	2.473165
H	3.726853	−1.976343	1.200635
H	1.994291	1.440453	−0.035285

H	2.315971	−0.612697	−1.132187
H	3.972246	−0.356359	−0.581981
N	2.452980	−2.965361	−0.060293
C	1.556507	−3.103064	−1.113601
N	1.481763	−4.343120	−1.569987
C	2.369236	−5.067421	−0.775791
C	2.736930	−6.433483	−0.767472
N	2.236163	−7.342930	−1.621792
N	3.642848	−6.831105	0.170941
C	4.141653	−5.926930	1.036970
N	3.863244	−4.615387	1.114285
C	2.972553	−4.234467	0.177775
H	0.972600	−2.264841	−1.465955
H	2.575631	−8.313078	−1.605069
H	1.630191	−7.037008	−2.371566
H	4.865269	−6.319191	1.748925
P	4.913324	1.673837	1.041205
O	5.007716	1.893557	−0.449196
O	5.326290	2.751015	2.018659
O	5.758441	0.288690	1.341808
C	5.894807	−0.142284	2.722510
C	7.106430	−1.048011	2.884474
O	6.871239	−2.370107	2.303131
C	7.724286	−2.585963	1.155780
C	8.393208	−0.515652	2.228503
C	8.430018	−1.246737	0.881969
O	9.495988	−0.854475	3.117766
H	4.988526	−0.679162	3.027824
H	6.023014	0.734137	3.368095
H	7.267890	−1.192781	3.958273
H	8.430733	−3.391225	1.380655
H	8.370920	0.568975	2.097597
H	7.870673	−0.655024	0.156033
H	9.444643	−1.378546	0.513754
N	6.925652	−3.070847	0.031361
C	6.092036	−2.329026	−0.797741
N	5.511351	−3.061094	−1.734346
C	5.978747	−4.356263	−1.515565
C	5.734812	−5.583335	−2.178151
N	4.920947	−5.712476	−3.237824
N	6.370203	−6.688299	−1.696176
C	7.176694	−6.583571	−0.625022
N	7.472303	−5.475949	0.075583
C	6.851246	−4.386383	−0.416271
H	5.950900	−1.268808	−0.644633
H	4.749038	−6.640464	−3.643764
H	4.425158	−4.904816	−3.589146
H	7.633376	−7.514705	−0.298180
P	11.074575	−0.655681	2.670594
O	11.199559	0.340183	1.543123
O	11.832123	−0.472215	3.966945
O	11.436601	−2.124589	2.014376
C	11.407159	−3.276604	2.898021
C	11.876956	−4.512732	2.154498
O	10.888350	−4.878904	1.144423
C	11.555228	−5.106227	−0.118781
C	13.233405	−4.367133	1.425513
C	12.841878	−4.271031	−0.055176
O	14.005994	−5.563173	1.716958
H	10.384410	−3.432960	3.262145
H	12.064730	−3.100882	3.757714
H	11.952803	−5.331695	2.882124
H	11.767456	−6.168533	−0.248101
H	13.780199	−3.481683	1.763793
H	12.642724	−3.226251	−0.309036
H	13.618796	−4.653647	−0.721832
H	14.871175	−5.467537	1.278264
N	10.629726	−4.748965	−1.198703
C	10.100794	−5.760951	−2.046400
O	10.438737	−6.953028	−1.864079
N	9.248556	−5.389975	−3.036485
C	8.866253	−4.105540	−3.184647
N	8.024406	−3.813572	−4.186557
C	9.340964	−3.080668	−2.300222
C	10.215472	−3.450730	−1.332029
H	7.659012	−4.557724	−4.800482
H	7.668720	−2.871905	−4.285927
H	9.012534	−2.052995	−2.397480
H	10.617948	−2.754519	−0.606839
O	6.384697	−11.597794	−10.887297
C	7.563743	−12.439148	−10.916160
C	7.866575	−12.979824	−9.523855
O	8.328374	−11.915444	−8.627521
C	7.346891	−11.667773	−7.588208
C	6.677775	−13.642162	−8.800143
C	6.114190	−12.501217	−7.952403
O	7.219057	−14.760221	−8.032348
H	6.309864	−11.165506	−11.755860
H	8.432920	−11.868497	−11.267428
H	7.410065	−13.294197	−11.590936
H	8.687199	−13.698720	−9.620735
H	7.775062	−11.959426	−6.625217
H	5.935206	−14.039860	−9.497057
H	5.427392	−11.923618	−8.572955
H	5.576824	−12.859595	−7.078755
N	7.084489	−10.237216	−7.474064
C	6.324579	−9.419859	−8.313466
N	6.302793	−8.162120	−7.910946
C	7.089316	−8.134593	−6.755974
C	7.447626	−7.060989	−5.887031
O	7.102461	−5.856541	−5.971171
N	8.290746	−7.485115	−4.841741
C	8.760435	−8.774336	−4.677152
N	9.620448	−8.995701	−3.661347
N	8.415798	−9.783621	−5.482497
C	7.594432	−9.412974	−6.480652
H	5.853107	−9.814310	−9.202595
H	8.606398	−6.746693	−4.170610
H	9.830973	−8.265289	−2.965937
H	9.813333	−9.963692	−3.434608
P	6.532941	−15.375541	−6.663510
O	5.044033	−15.131424	−6.620804
O	7.099031	−16.772786	−6.545056
O	7.172107	−14.409495	−5.487373
C	8.603201	−14.479505	−5.243185
C	8.910811	−14.171424	−3.785804
O	8.710733	−12.754011	−3.490637
C	7.653788	−12.577360	−2.519989
C	8.049429	−14.953493	−2.774568
C	6.972783	−13.943562	−2.361333
O	8.930404	−15.358414	−1.688255
H	9.115961	−13.758960	−5.891316
H	8.966192	−15.487524	−5.472784
H	9.970354	−14.395371	−3.617128
H	8.078775	−12.223635	−1.579064
H	7.609585	−15.850193	−3.217619
H	6.123103	−14.051008	−3.036483
H	6.627663	−14.111207	−1.343755
N	6.767129	−11.485616	−2.969031
C	6.877804	−10.239379	−2.325127
O	7.619159	−10.036745	−1.361569
N	6.077423	−9.247305	−2.854536
C	5.174044	−9.370000	−3.902429
O	4.516245	−8.378638	−4.282940
C	5.079816	−10.690133	−4.501766
C	4.072885	−10.908646	−5.595892
C	5.905367	−11.663074	−4.035528
H	6.174887	−8.293295	−2.404347

H	3.074340	−10.611849	−5.253205
H	4.035908	−11.958827	−5.892855
H	4.301334	−10.299323	−6.477881
H	5.937753	−12.647782	−4.488917
P	8.351871	−16.059183	−0.307350
O	6.963081	−16.611985	−0.516803
O	9.478747	−16.936196	0.193519
O	8.173028	−14.758724	0.691403
C	9.374667	−14.032071	1.060699
C	9.029177	−12.906908	2.018122
O	8.244831	−11.897215	1.315888
C	7.086077	−11.551755	2.108006
C	8.215796	−13.321326	3.267051
C	6.807125	−12.782312	2.982589
O	8.834523	−12.674835	4.412133
H	9.840993	−13.614303	0.160096
H	10.086497	−14.712133	1.543973
H	9.972050	−12.451285	2.349201
H	7.294271	−10.667301	2.713664
H	8.217622	−14.406305	3.408735
H	6.238196	−13.536731	2.433558
H	6.262543	−12.526708	3.894935
H	8.342086	−12.950964	5.206847
N	6.002900	−11.163325	1.198301
C	5.637693	−9.809031	1.119678
O	6.149876	−8.923826	1.812574
N	4.635030	−9.539278	0.209917
C	3.984202	−10.453070	−0.612130
O	3.087493	−10.070405	−1.394668
C	4.425784	−11.832332	−0.501775
C	3.758948	−12.877977	−1.352258
C	5.417270	−12.109947	0.382702
H	4.313681	−8.536946	0.174818
H	2.680829	−12.904148	−1.153579
H	4.171582	−13.869318	−1.151116
H	3.880459	−12.653568	−2.418121
H	5.814932	−13.111597	0.494187

d(AAG)$^{4-}$

E: −27672.74 kcal mol^{-1}

O	−0.184026	−0.445203	−0.426506
C	−0.126990	−0.027774	0.959089
C	1.306403	−0.079963	1.472977
O	1.777353	−1.461338	1.613734
C	2.810358	−1.751681	0.640202
C	2.351754	0.627232	0.589541
C	2.920079	−0.513413	−0.260767
O	3.310776	1.249616	1.494125
H	−1.121900	−0.536300	−0.669259
H	−0.747977	−0.683127	1.583085
H	−0.490968	1.004847	1.065244
H	1.311781	0.363548	2.474323
H	3.741327	−1.967149	1.173723
H	1.909066	1.411280	−0.030497
H	2.289051	−0.621346	−1.143682
H	3.938706	−0.325136	−0.591093
N	2.486201	−2.977617	−0.088777
C	1.601496	−3.130827	−1.149895
N	1.549542	−4.372803	−1.604537
C	2.440179	−5.082460	−0.800671
C	2.826931	−6.443015	−0.783420
N	2.343850	−7.364828	−1.635562
N	3.730458	−6.823571	0.164008
C	4.208315	−5.909842	1.031679
N	3.908987	−4.602776	1.102619
C	3.022233	−4.238244	0.156358
H	1.008464	−2.302639	−1.510153
H	2.709579	−8.325415	−1.619904
H	1.754956	−7.067508	−2.402313
H	4.932201	−6.288872	1.750335
P	4.823772	1.720604	1.031110
O	4.896849	1.963552	−0.457035
O	5.217739	2.796353	2.018483
O	5.705899	0.354524	1.305231
C	5.867210	−0.088651	2.679163
C	7.081569	−0.995090	2.807053
O	6.831062	−2.306043	2.207552
C	7.696157	−2.520233	1.067164
C	8.358894	−0.451643	2.137454
C	8.376929	−1.172880	0.785852
O	9.473121	−0.782231	3.016400
H	4.967205	−0.629443	2.996286
H	6.006462	0.781511	3.331076
H	7.262833	−1.155046	3.875748
H	8.416007	−3.309919	1.306030
H	8.327591	0.633907	2.013964
H	7.787408	−0.582919	0.082398
H	9.381479	−1.284274	0.384898
N	6.915482	−3.030973	−0.056265
C	6.085686	−2.315040	−0.910033
N	5.524500	−3.071245	−1.839701
C	6.004377	−4.356663	−1.590123
C	5.781691	−5.599727	−2.229136
N	4.975984	−5.763863	−3.290122
N	6.428703	−6.686275	−1.718595
C	7.213871	−6.550271	−0.634195
N	7.480900	−5.426566	0.050242
C	6.860215	−4.354968	−0.477902
H	5.932095	−1.253139	−0.783080
H	4.827828	−6.700830	−3.686549
H	4.491176	−4.965842	−3.676600
H	7.675852	−7.468840	−0.280783
P	11.050616	−0.726238	2.528525
O	11.226361	0.171779	1.327830
O	11.845145	−0.499044	3.795912
O	11.291746	−2.260999	1.981219
C	11.217413	−3.344240	2.945175
C	11.788923	−4.616966	2.343069
O	10.898872	−5.127930	1.301559
C	11.609218	−5.225308	0.043960
C	13.177149	−4.455110	1.692644
C	12.856218	−4.340962	0.198357
O	13.939587	−5.653298	2.001682
H	10.171118	−3.508205	3.230902
H	11.791891	−3.081208	3.841279
H	11.843640	−5.367848	3.141343
H	11.869915	−6.271017	−0.149193
H	13.700954	−3.571601	2.071297
H	12.617704	−3.301543	−0.039487
H	13.679741	−4.672401	−0.439011
H	14.811866	−5.563924	1.575773
N	10.722928	−4.829463	−1.039143
C	10.251152	−3.548239	−1.322282
N	9.453114	−3.520065	−2.371747
C	9.385003	−4.844209	−2.811248
C	8.691204	−5.433457	−3.909079
O	7.946535	−4.865293	−4.744661
N	8.922814	−6.819984	−3.997771
C	9.728215	−7.547220	−3.145616
N	9.901263	−8.856248	−3.430116
N	10.359897	−7.001917	−2.098639
C	10.163939	−5.672792	−1.988036
H	10.532519	−2.702283	−0.712479
H	8.419029	−7.327113	−4.762549
H	9.353188	−9.319400	−4.173060

H	10.280810	−9.432289	−2.688874
O	6.239322	−11.719501	−10.861341
C	7.451329	−12.509965	−10.949828
C	7.878497	−12.977868	−9.564822
O	8.359363	−11.850756	−8.759562
C	7.513573	−11.664083	−7.595280
C	6.773436	−13.655571	−8.728301
C	6.268784	−12.522992	−7.833743
O	7.402581	−14.752424	−7.999815
H	6.083924	−11.318066	−11.734110
H	8.263850	−11.916457	−11.387589
H	7.285896	−13.396642	−11.578801
H	8.716326	−13.671876	−9.693504
H	8.056775	−11.967028	−6.700057
H	5.977698	−14.071318	−9.351864
H	5.503425	−11.968313	−8.379467
H	5.834018	−12.891316	−6.909017
N	7.238624	−10.230217	−7.410985
C	7.832517	−9.540000	−6.316458
O	8.609980	−10.157703	−5.556392
N	7.521470	−8.230872	−6.132573
C	6.705031	−7.581756	−6.986053
N	6.426684	−6.294914	−6.730583
C	6.160349	−8.239152	−8.139894
C	6.461727	−9.551026	−8.316079
H	6.900328	−5.799773	−5.960682
H	5.853514	−5.767749	−7.376420
H	5.544764	−7.705588	−8.854757
H	6.130275	−10.122158	−9.177191
P	6.728957	−15.458059	−6.665777
O	5.234704	−15.251327	−6.623153
O	7.330014	−16.844983	−6.614176
O	7.339436	−14.537429	−5.439777
C	8.774067	−14.573130	−5.210585
C	9.093493	−14.191154	−3.773996
O	8.875650	−12.764930	−3.549516
C	7.857908	−12.553817	−2.545867
C	8.257750	−14.935279	−2.713617
C	7.184011	−13.914578	−2.321422
O	9.162475	−15.286826	−1.627768
H	9.268764	−13.876118	−5.897368
H	9.149930	−15.585259	−5.398360
H	10.158159	−14.393305	−3.608434
H	8.314993	−12.171064	−1.631380
H	7.815719	−15.852875	−3.108686
H	6.324227	−14.048795	−2.979313
H	6.856437	−14.043758	−1.292578
N	6.954504	−11.477471	−2.996351
C	7.050768	−10.222539	−2.365836
O	7.794492	−10.000275	−1.408515
N	6.228512	−9.249176	−2.896919
C	5.305057	−9.402190	−3.922927
O	4.610435	−8.433966	−4.296016
C	5.224823	−10.731310	−4.505665
C	4.189179	−10.981540	−5.566501
C	6.077160	−11.683269	−4.044083
H	6.299507	−8.294982	−2.444073
H	3.198827	−10.687897	−5.198370
H	4.153241	−12.037104	−5.843588
H	4.382883	−10.385915	−6.466098
H	6.116420	−12.675424	−4.480735
P	8.614130	−15.962373	−0.222325
O	7.234613	−16.547414	−0.403737
O	9.763349	−16.804294	0.288210
O	8.419165	−14.642774	0.746337
C	9.610425	−13.893062	1.102213
C	9.248919	−12.763898	2.048555
O	8.449454	−11.773170	1.336628
C	7.282025	−11.442752	2.121624

C	8.441162	−13.180256	3.300492
C	7.023596	−12.668866	3.008919
O	9.046157	−12.512561	4.440962
H	10.066642	−13.477957	0.195326
H	10.334283	−14.556463	1.590427
H	10.184542	−12.290660	2.375355
H	7.470781	−10.547916	2.718145
H	8.462112	−14.263674	3.452227
H	6.468181	−13.438862	2.467566
H	6.473772	−12.412681	3.917959
H	8.558003	−12.792068	5.237085
N	6.194225	−11.085015	1.204775
C	5.803297	−9.739235	1.112224
O	6.297848	−8.837768	1.797560
N	4.797723	−9.497254	0.198599
C	4.162921	−10.431673	−0.612673
O	3.257810	−10.073970	−1.397209
C	4.629633	−11.801484	−0.487247
C	3.977477	−12.868851	−1.321803
C	5.626915	−12.051045	0.399233
H	4.450952	−8.503300	0.160016
H	2.901908	−12.914902	−1.112960
H	4.411505	−13.849681	−1.114563
H	4.084975	−12.652576	−2.390632
H	6.042929	−13.043800	0.522205

d(AAT)$^{4-}$

E: −27764.55 kcal mol^{-1}

O	−0.132799	−0.215885	−0.556979
C	−0.063758	0.239175	0.816524
C	1.354468	0.100274	1.355546
O	1.722686	−1.307998	1.535132
C	2.760133	−1.686394	0.596710
C	2.464877	0.708082	0.476256
C	2.944501	−0.490542	−0.346967
O	3.459130	1.264120	1.388375
H	−1.071827	−0.246609	−0.810453
H	−0.738374	−0.351080	1.449884
H	−0.355019	1.297575	0.886382
H	1.375000	0.566960	2.346132
H	3.670787	−1.922368	1.154822
H	2.096584	1.517688	−0.159491
H	2.290512	−0.583848	−1.215082
H	3.967255	−0.382161	−0.699607
N	2.393981	−2.924147	−0.087098
C	1.490733	−3.087776	−1.130241
N	1.425461	−4.336598	−1.565199
C	2.329988	−5.037852	−0.768527
C	2.723044	−6.396867	−0.744373
N	2.226054	−7.331839	−1.573677
N	3.654022	−6.761578	0.182901
C	4.148156	−5.836085	1.029314
N	3.841624	−4.530974	1.094755
C	2.932151	−4.181597	0.165011
H	0.897574	−2.259708	−1.492389
H	2.591383	−8.292232	−1.548523
H	1.603839	−7.051972	−2.320350
H	4.893635	−6.203443	1.731956
P	5.043122	1.514600	1.001579
O	5.232718	1.702092	−0.484147
O	5.523589	2.553068	1.989691
O	5.720626	0.051892	1.347988
C	5.798411	−0.378276	2.732666
C	7.017373	−1.266004	2.943597
O	6.839933	−2.588712	2.344492
C	7.703116	−2.754939	1.196803

C	8.316638	−0.700783	2.344563
C	8.418128	−1.408352	0.987861
O	9.392263	−1.033599	3.267951
H	4.886521	−0.928628	2.993340
H	5.885453	0.497714	3.385144
H	7.135036	−1.417669	4.022035
H	8.403793	−3.572034	1.392877
H	8.278998	0.385247	2.229511
H	7.898483	−0.803733	0.244297
H	9.450350	−1.540427	0.672649
N	6.916980	−3.189865	0.040116
C	6.048597	−2.427375	−0.733063
N	5.457638	−3.126671	−1.688088
C	5.949589	−4.422900	−1.539012
C	5.692390	−5.630832	−2.231844
N	4.846502	−5.739702	−3.269926
N	6.347591	−6.745674	−1.803153
C	7.181904	−6.672157	−0.751120
N	7.488224	−5.586929	−0.021875
C	6.848098	−4.486219	−0.460649
H	5.893316	−1.379034	−0.522668
H	4.660184	−6.661019	−3.681682
H	4.325716	−4.931279	−3.580915
H	7.658108	−7.608725	−0.473201
P	10.977257	−0.763061	2.886866
O	11.107205	0.299771	1.822971
O	11.683358	−0.632577	4.218443
O	11.408786	−2.178408	2.159596
C	11.359238	−3.387219	2.961593
C	11.805862	−4.573948	2.129744
O	10.813756	−4.837116	1.091799
C	11.499542	−5.070521	−0.159874
C	13.171616	−4.407033	1.417996
C	12.796591	−4.256718	−0.063229
O	13.936829	−5.615241	1.676166
H	10.334638	−3.551354	3.316556
H	12.021294	−3.285070	3.829864
H	11.862193	−5.447094	2.793670
H	11.704234	−6.135739	−0.286800
H	13.717879	−3.536194	1.792091
H	12.615285	−3.201339	−0.284496
H	13.574232	−4.630161	−0.734183
H	14.814695	−5.499598	1.268572
N	10.596310	−4.706545	−1.254133
C	10.077837	−5.725962	−2.068705
O	10.386167	−6.916693	−1.948197
N	9.193280	−5.291911	−3.032659
C	8.796109	−3.982810	−3.278842
O	8.002559	−3.734798	−4.212553
C	9.358962	−2.975063	−2.394802
C	8.983583	−1.533669	−2.598734
C	10.217525	−3.390243	−1.429067
H	8.810580	−6.044650	−3.667841
H	9.278873	−1.196230	−3.599869
H	9.472556	−0.893136	−1.860687
H	7.899079	−1.397767	−2.522523
H	10.659208	−2.697078	−0.723582
O	6.378349	−11.501724	−10.732150
C	7.570069	−12.322192	−10.803968
C	7.883229	−12.925625	−9.440669
O	8.372347	−11.912718	−8.499050
C	7.405693	−11.675367	−7.449320
C	6.693675	−13.609942	−8.739595
C	6.173159	−12.526339	−7.790108
O	7.215277	−14.801901	−8.082515
H	6.289328	−11.034335	−11.581006
H	8.429613	−11.723946	−11.131912
H	7.424547	−13.147585	−11.516229
H	8.694873	−13.647417	−9.579296
H	7.855381	−11.948083	−6.489587
H	5.922985	−13.924887	−9.448041
H	5.430654	−11.933855	−8.325561
H	5.699632	−12.945176	−6.906482
N	7.112571	−10.242964	−7.345618
C	6.313701	−9.474509	−8.185585
N	6.247646	−8.205012	−7.816414
C	7.049797	−8.122745	−6.679258
C	7.397960	−7.040653	−5.835090
N	6.956252	−5.782989	−6.006439
N	8.246384	−7.305087	−4.801608
C	8.717871	−8.553933	−4.626688
N	8.454323	−9.644549	−5.363757
C	7.609379	−9.373917	−6.377860
H	5.847142	−9.905249	−9.060313
H	7.240303	−5.045156	−5.351964
H	6.290316	−5.579525	−6.739567
H	9.382476	−8.680420	−3.774400
P	6.458407	−15.558930	−6.825492
O	4.973332	−15.289255	−6.821500
O	6.997005	−16.972135	−6.843715
O	7.063089	−14.742383	−5.526495
C	8.498128	−14.800633	−5.309621
C	8.828910	−14.308451	−3.912961
O	8.568953	−12.873346	−3.812115
C	7.653689	−12.606610	−2.724107
C	8.022707	−14.989518	−2.785404
C	6.969331	−13.942315	−2.413617
O	8.961033	−15.293124	−1.713959
H	9.005421	−14.176977	−6.055316
H	8.847413	−15.834458	−5.416739
H	9.899347	−14.473523	−3.742712
H	8.198423	−12.220002	−1.860203
H	7.562186	−15.922832	−3.117258
H	6.093786	−14.094919	−3.047199
H	6.664026	−14.020381	−1.373213
N	6.745788	−11.520054	−3.124875
C	6.905373	−10.259549	−2.520835
O	7.734320	−10.030418	−1.637932
N	6.042768	−9.288158	−2.984971
C	5.085288	−9.422752	−3.980842
O	4.397966	−8.438717	−4.326043
C	4.969597	−10.749692	−4.568102
C	3.916567	−10.982082	−5.615667
C	5.817938	−11.716290	−4.130267
H	6.157291	−8.330102	−2.546535
H	2.931198	−10.689770	−5.233685
H	3.875255	−12.033939	−5.908116
H	4.104277	−10.375160	−6.509436
H	5.819450	−12.713003	−4.558344
P	8.459722	−15.862764	−0.244420
O	7.053913	−16.406137	−0.321964
O	9.603598	−16.713346	0.263030
O	8.359455	−14.477729	0.644048
C	9.591634	−13.750384	0.891827
C	9.335276	−12.605967	1.854799
O	8.507935	−11.595274	1.204560
C	7.370260	−11.284838	2.038969
C	8.616645	−12.996405	3.165777
C	7.178781	−12.504448	2.952729
O	9.285529	−12.292077	4.246610
H	9.977681	−13.352902	−0.054708
H	10.339841	−14.425511	1.323870
H	10.305150	−12.154429	2.101534
H	7.565191	−10.377847	2.614383
H	8.659454	−14.075423	3.343580
H	6.601859	−13.286914	2.453938
H	6.679878	−12.243492	3.889382
H	8.839735	−12.538621	5.077698

N	6.231880	−10.962523	1.169660
C	5.800589	−9.628614	1.091859
O	6.296455	−8.712313	1.756560
N	4.757768	−9.414267	0.213800
C	4.105805	−10.369516	−0.557771
O	3.161918	−10.036849	−1.307130
C	4.600488	−11.729943	−0.434323
C	3.915194	−12.820260	−1.210576
C	5.647738	−11.949542	0.401943
H	4.390077	−8.427268	0.179237
H	2.850260	−12.859303	−0.951313
H	4.357487	−13.795243	−0.994817
H	3.973094	−12.635067	−2.289129
H	6.093309	−12.931302	0.513455

d(ACA)$^{4-}$

E: −27673.13 kcal mol^{-1}

O	−0.231767	−0.391251	−0.233790
C	−0.168664	0.029417	1.150806
C	1.269545	−0.002097	1.652926
O	1.753499	−1.375510	1.825219
C	2.779700	−1.687623	0.853220
C	2.298855	0.692279	0.742230
C	2.890046	−0.463266	−0.072569
O	3.254906	1.355229	1.618952
H	−1.170628	−0.485116	−0.471855
H	−0.776738	−0.631742	1.781218
H	−0.544197	1.057789	1.256156
H	1.280695	0.465766	2.642963
H	3.713856	−1.900335	1.382053
H	1.840611	1.446474	0.097639
H	2.283101	−0.599324	−0.968541
H	3.914277	−0.267568	−0.380134
N	2.440044	−2.924123	0.145738
C	1.502728	−3.083223	−0.869303
N	1.442278	−4.323416	−1.326828
C	2.380797	−5.025835	−0.573611
C	2.788548	−6.380552	−0.594133
N	2.278102	−7.294291	−1.437308
N	3.740690	−6.762324	0.304431
C	4.256179	−5.848397	1.149587
N	3.950929	−4.543603	1.244235
C	3.004632	−4.180635	0.355457
H	0.880847	−2.259071	−1.189199
H	2.653727	−8.251067	−1.449977
H	1.632750	−6.997919	−2.157447
H	5.018122	−6.224853	1.829242
P	4.678520	1.983168	1.063788
O	4.611611	2.265493	−0.417611
O	5.041539	3.068089	2.052988
O	5.707320	0.705093	1.230516
C	5.953103	0.216552	2.575994
C	7.029941	−0.852049	2.558203
O	6.530515	−2.057634	1.899102
C	7.454444	−2.459270	0.855517
C	8.337771	−0.459106	1.831199
C	8.224733	−1.187994	0.489157
O	9.429727	−0.925302	2.676214
H	5.027033	−0.201713	2.988772
H	6.281166	1.044755	3.215492
H	7.264598	−1.101649	3.600100
H	8.117202	−3.245346	1.221479
H	8.432408	0.621951	1.703970
H	7.642948	−0.560052	−0.190292
H	9.191073	−1.386059	0.031402
N	6.689546	−3.069399	−0.236352
C	6.785674	−4.472461	−0.462001
O	7.515586	−5.159996	0.281589
N	6.076916	−5.008305	−1.489753
C	5.256261	−4.252463	−2.243772
N	4.582495	−4.852086	−3.235803
C	5.101829	−2.847686	−1.993101
C	5.835806	−2.309389	−0.985803
H	4.701256	−5.860565	−3.425758
H	3.947209	−4.312635	−3.808496
H	4.421848	−2.240119	−2.578171
H	5.774268	−1.263249	−0.712421
P	11.010327	−0.958104	2.200791
O	11.223126	−0.157671	0.939024
O	11.812441	−0.675683	3.451945
O	11.201098	−2.536095	1.765418
C	11.134317	−3.560567	2.792523
C	11.873636	−4.815883	2.347224
O	11.133196	−5.541001	1.316318
C	11.811522	−5.452671	0.042985
C	13.271647	−4.565967	1.759197
C	13.014435	−4.510949	0.247234
O	14.103872	−5.696161	2.135449
H	10.084097	−3.805362	2.992009
H	11.595898	−3.185314	3.713186
H	11.944770	−5.480262	3.216306
H	12.114359	−6.458225	−0.262196
H	13.710497	−3.635840	2.135007
H	12.756229	−3.489433	−0.038513
H	13.881685	−4.829525	−0.336024
H	14.978900	−5.565819	1.726254
N	10.871948	−4.999918	−0.980704
C	10.311484	−3.736025	−1.109743
N	9.431691	−3.658332	−2.094751
C	9.396432	−4.940696	−2.639631
C	8.660712	−5.502708	−3.708559
N	7.790202	−4.813200	−4.467935
N	8.870385	−6.820936	−3.982576
C	9.755547	−7.526950	−3.254251
N	10.507235	−7.092445	−2.230300
C	10.282946	−5.791235	−1.958626
H	10.585566	−2.939937	−0.432486
H	7.242299	−5.297595	−5.190071
H	7.564387	−3.859090	−4.221345
H	9.854803	−8.573081	−3.537713
O	6.077549	−11.602997	−10.728389
C	7.289200	−12.383862	−10.882774
C	7.790094	−12.863101	−9.526206
O	8.314131	−11.744816	−8.736498
C	7.530913	−11.569095	−7.527309
C	6.737891	−13.550795	−8.631973
C	6.270083	−12.420293	−7.713214
O	7.429824	−14.629745	−7.935486
H	5.883702	−11.186515	−11.586181
H	8.075363	−11.780382	−11.353950
H	7.099355	−13.265540	−11.511917
H	8.620056	−13.554734	−9.707943
H	8.118559	−11.887289	−6.664796
H	5.915829	−13.980899	−9.209805
H	5.489376	−11.857976	−8.228894
H	5.863877	−12.784444	−6.772897
N	7.274160	−10.138064	−7.318190
C	7.912252	−9.487531	−6.249863
O	8.738544	−10.036886	−5.514663
N	7.537089	−8.170544	−6.082686
C	6.676330	−7.434086	−6.885707
O	6.446139	−6.231319	−6.621089
C	6.115910	−8.141769	−8.024964
C	5.217732	−7.404901	−8.981494
C	6.448482	−9.448997	−8.186051

H	8.006453	−7.668044	−5.282336
H	5.734950	−6.542436	−9.420131
H	4.329811	−7.018317	−8.465732
H	4.890184	−8.063459	−9.791227
H	6.099672	−10.028573	−9.035156
P	6.792450	−15.423566	−6.634828
O	5.299021	−15.222891	−6.548266
O	7.395171	−16.809990	−6.684525
O	7.427817	−14.584572	−5.363980
C	8.856488	−14.674894	−5.115200
C	9.177946	−14.374472	−3.656271
O	9.051331	−12.949672	−3.352122
C	7.927025	−12.709277	−2.472431
C	8.286279	−15.104134	−2.635795
C	7.215090	−14.061876	−2.302870
O	9.135596	−15.461899	−1.506376
H	9.382620	−13.960476	−5.760023
H	9.202771	−15.688288	−5.348474
H	10.227620	−14.643544	−3.493985
H	8.297563	−12.311525	−1.522644
H	7.849057	−16.017732	−3.046335
H	6.402676	−14.162135	−3.023711
H	6.810808	−14.192605	−1.301960
N	7.073059	−11.662215	−3.025805
C	6.218516	−11.755605	−4.124274
N	5.586098	−10.624764	−4.373521
C	6.041551	−9.730037	−3.402000
C	5.711986	−8.363340	−3.156226
O	4.915136	−7.635862	−3.795155
N	6.403875	−7.846067	−2.044456
C	7.325924	−8.542776	−1.287698
N	7.901497	−7.884922	−0.267176
N	7.644186	−9.822242	−1.523168
C	6.979011	−10.355422	−2.564844
H	6.137375	−12.675272	−4.684809
H	6.244227	−6.836136	−1.820659
H	7.735352	−6.882480	−0.085515
H	8.607651	−8.378214	0.261715
P	8.513673	−16.023874	−0.081449
O	7.129034	−16.592126	−0.277272
O	9.622185	−16.851515	0.530842
O	8.309085	−14.635473	0.784192
C	9.499279	−13.887451	1.148315
C	9.144837	−12.798897	2.144894
O	8.351202	−11.766193	1.484947
C	7.171291	−11.488136	2.271019
C	8.333013	−13.273079	3.372557
C	6.913842	−12.761186	3.089604
O	8.922597	−12.646747	4.543856
H	9.937385	−13.436836	0.249271
H	10.236047	−14.561864	1.600399
H	10.082868	−12.346659	2.492969
H	7.338976	−10.622140	2.914497
H	8.362818	−14.361656	3.479560
H	6.372590	−13.504055	2.497760
H	6.349759	−12.558766	4.003537
H	8.429453	−12.961332	5.323749
N	6.094855	−11.098327	1.352142
C	5.611508	−9.781534	1.392435
O	5.976623	−8.946392	2.227015
N	4.679772	−9.486747	0.417075
C	4.133696	−10.369886	−0.508868
O	3.262752	−9.976855	−1.315072
C	4.641833	−11.729439	−0.466945
C	4.059390	−12.744291	−1.410662
C	5.613796	−12.013521	0.436489
H	4.336968	−8.490680	0.399191
H	2.976321	−12.826813	−1.258940
H	4.504023	−13.728884	−1.252770
H	4.214230	−12.447581	−2.453634
H	6.077399	−12.991723	0.491813

d(ACC)$^{4-}$

E: −27585.68 kcal mol^{-1}

O	−0.256841	−0.365073	−0.260599
C	−0.166106	0.051126	1.123825
C	1.280697	0.004116	1.599499
O	1.751960	−1.375831	1.758274
C	2.756599	−1.695962	0.767456
C	2.298850	0.689956	0.669406
C	2.861203	−0.470369	−0.157311
O	3.278807	1.345538	1.524556
H	−1.200126	−0.458014	−0.480745
H	−0.769021	−0.606368	1.762874
H	−0.530318	1.082563	1.238353
H	1.315733	0.468418	2.590595
H	3.697136	−1.918962	1.281048
H	1.833295	1.447802	0.034265
H	2.234489	−0.599269	−1.040651
H	3.878390	−0.279964	−0.488674
N	2.392949	−2.927537	0.061555
C	1.436440	−3.071262	−0.938173
N	1.356733	−4.307348	−1.403298
C	2.302862	−5.023390	−0.672693
C	2.696668	−6.382131	−0.711342
N	2.154938	−7.284739	−1.546823
N	3.666288	−6.779327	0.161895
C	4.203311	−5.876972	1.006420
N	3.907455	−4.571041	1.121891
C	2.949736	−4.191378	0.252478
H	0.816784	−2.239302	−1.241674
H	2.512912	−8.247826	−1.573312
H	1.488846	−6.978047	−2.243480
H	4.978304	−6.263782	1.664896
P	4.707254	1.951711	0.956599
O	4.671971	2.134970	−0.541070
O	5.044501	3.101654	1.879093
O	5.741502	0.695376	1.230174
C	5.969042	0.304210	2.610713
C	7.078929	−0.728519	2.690694
O	6.637580	−1.990662	2.096616
C	7.526258	−2.372715	1.019380
C	8.392305	−0.343699	1.974814
C	8.307298	−1.105936	0.650149
O	9.482414	−0.778698	2.838702
H	5.046616	−0.119056	3.026433
H	6.252856	1.183386	3.201222
H	7.286252	−0.908025	3.752106
H	8.183551	−3.177815	1.351264
H	8.478799	0.734751	1.821225
H	7.751590	−0.491809	−0.061702
H	9.288418	−1.316900	0.229696
N	6.733291	−2.946524	−0.076109
C	6.789605	−4.348445	−0.323045
O	7.467839	−5.072456	0.434474
N	6.112195	−4.839666	−1.392201
C	5.336218	−4.046036	−2.154330
N	4.698002	−4.600048	−3.193736
C	5.193287	−2.647889	−1.862835
C	5.908867	−2.149493	−0.822874
H	4.788782	−5.609876	−3.394381
H	4.094006	−4.032501	−3.773341
H	4.538035	−2.013861	−2.447939
H	5.856870	−1.110790	−0.518450
P	11.058929	−0.824246	2.345183

O	11.269550	0.008343	1.104171
O	11.873794	−0.586919	3.596935
O	11.224156	−2.393883	1.863454
C	11.149869	−3.435310	2.874596
C	11.753279	−4.727530	2.346938
O	10.890307	−5.317843	1.326076
C	11.598173	−5.445912	0.073425
C	13.149276	−4.583211	1.709403
C	12.858738	−4.572844	0.203521
O	13.926759	−5.742315	2.113907
H	10.100805	−3.602501	3.147295
H	11.703617	−3.120277	3.766315
H	11.807634	−5.431551	3.186634
H	11.834970	−6.493617	−0.113576
H	13.650020	−3.666390	2.036759
H	12.653913	−3.548670	−0.118570
H	13.691046	−4.964129	−0.386924
H	14.806668	−5.663485	1.701924
N	10.694435	−5.042247	−1.018169
C	10.312673	−5.976147	−2.019359
O	10.796643	−7.131347	−2.003403
N	9.434950	−5.569484	−2.975332
C	8.973322	−4.302843	−3.005735
N	8.131395	−3.960743	−3.991933
C	9.365116	−3.344298	−2.015533
C	10.205159	−3.764033	−1.036889
H	7.809251	−4.662585	−4.677481
H	7.738510	−3.029123	−4.014871
H	8.985710	−2.330300	−2.035623
H	10.521552	−3.129912	−0.215837
O	5.905589	−11.644193	−10.837230
C	7.020945	−12.560618	−10.954728
C	7.469057	−13.039536	−9.579337
O	8.065793	−11.946055	−8.807282
C	7.242499	−11.641869	−7.649477
C	6.360904	−13.624102	−8.678882
C	5.935301	−12.415499	−7.841933
O	6.973473	−14.715516	−7.923312
H	5.753078	−11.253188	−11.715196
H	7.870084	−12.067760	−11.445847
H	6.736092	−13.441194	−11.549363
H	8.248352	−13.793716	−9.734265
H	7.772271	−11.953018	−6.746366
H	5.530343	−14.039957	−9.255490
H	5.229060	−11.830035	−8.433737
H	5.461500	−12.688662	−6.901050
N	7.072310	−10.203131	−7.519557
C	6.269585	−9.347919	−8.274421
N	6.320981	−8.098473	−7.848940
C	7.197731	−8.121201	−6.760782
C	7.634803	−7.085511	−5.886371
O	7.312497	−5.871165	−5.915464
N	8.526503	−7.561163	−4.904599
C	8.964130	−8.868398	−4.800667
N	9.870546	−9.147027	−3.835328
N	8.555485	−9.839717	−5.620275
C	7.681488	−9.419675	−6.551419
H	5.709274	−9.709841	−9.125508
H	8.869023	−6.855828	−4.210384
H	10.124818	−8.441999	−3.129897
H	9.990606	−10.128891	−3.611543
P	6.535689	−15.190080	−6.406218
O	5.069042	−14.942791	−6.146246
O	7.138538	−16.567206	−6.245962
O	7.328866	−14.110229	−5.447698
C	8.761131	−14.215139	−5.240548
C	9.100930	−14.077509	−3.759230
O	8.971885	−12.700367	−3.284997
C	7.787079	−12.550938	−2.465399
C	8.206898	−14.920645	−2.833409
C	7.112909	−13.933304	−2.407711
O	9.047779	−15.390190	−1.739847
H	9.259023	−13.419694	−5.806438
H	9.116051	−15.189225	−5.593774
H	10.152179	−14.356810	−3.631194
H	8.096311	−12.197000	−1.477701
H	7.787542	−15.790169	−3.345835
H	6.293585	−13.999127	−3.123433
H	6.725871	−14.156222	−1.416298
N	6.927068	−11.495472	−3.000955
C	6.052625	−11.553458	−4.089805
N	5.456633	−10.400801	−4.323762
C	5.962262	−9.524763	−3.360663
C	5.705873	−8.142136	−3.122948
O	4.926575	−7.384709	−3.749647
N	6.461248	−7.642436	−2.045420
C	7.350896	−8.380401	−1.287252
N	8.000858	−7.732935	−0.305167
N	7.585276	−9.680786	−1.503293
C	6.883813	−10.187490	−2.534265
H	5.932111	−12.462458	−4.659602
H	6.324582	−6.632018	−1.806044
H	7.780529	−6.759589	−0.045023
H	8.600579	−8.284352	0.293039
P	8.416632	−16.088290	−0.382938
O	7.044472	−16.659237	−0.647459
O	9.531034	−16.949820	0.169886
O	8.177564	−14.785533	0.600280
C	9.349599	−14.031447	1.006201
C	8.950176	−12.939040	1.979740
O	8.162015	−11.929017	1.280613
C	6.993381	−11.609406	2.068853
C	8.109696	−13.408734	3.192075
C	6.703087	−12.871644	2.892547
O	8.693753	−12.802466	4.376431
H	9.821008	−13.583470	0.122872
H	10.071920	−14.699500	1.490745
H	9.870785	−12.469868	2.352044
H	7.191120	−10.747384	2.709458
H	8.119104	−14.498320	3.291330
H	6.153560	−13.608179	2.300280
H	6.134473	−12.653703	3.800025
H	8.193864	−13.125626	5.148366
N	5.927276	−11.189172	1.153764
C	5.514179	−9.847623	1.164517
O	5.946250	−9.007739	1.960251
N	4.567734	−9.533536	0.208641
C	3.966014	−10.409223	−0.690646
O	3.085407	−9.996965	−1.475863
C	4.430289	−11.784968	−0.642788
C	3.813654	−12.792897	−1.572665
C	5.395671	−12.095785	0.258528
H	4.251054	−8.529637	0.199649
H	2.734550	−12.871920	−1.392550
H	4.259386	−13.780265	−1.431371
H	3.942896	−12.492024	−2.617998
H	5.813276	−13.093295	0.325354

d(ACG)$^{4-}$

E: −27582.61 kcal mol^{-1}

O	−0.253451	−0.466765	−0.259131
C	−0.198136	−0.063468	1.130781
C	1.239961	−0.079071	1.634278
O	1.747341	−1.446652	1.784840
C	2.777510	−1.725558	0.806207

C	2.258487	0.647004	0.736488
C	2.865966	−0.485139	−0.099158
O	3.203279	1.309063	1.625889
H	−1.190533	−0.571986	−0.499430
H	−0.796537	−0.742027	1.751852
H	−0.589591	0.957519	1.249417
H	1.242065	0.372737	2.631834
H	3.715535	−1.931098	1.331060
H	1.788799	1.405847	0.105554
H	2.257106	−0.615261	−0.994686
H	3.885995	−0.269432	−0.407935
N	2.458285	−2.955949	0.079451
C	1.531110	−3.115122	−0.944723
N	1.486757	−4.351756	−1.413722
C	2.426391	−5.051431	−0.659335
C	2.841761	−6.403772	−0.682340
N	2.342002	−7.317072	−1.531858
N	3.788640	−6.783792	0.222464
C	4.291177	−5.871199	1.076772
N	3.974720	−4.569664	1.177438
C	3.034113	−4.208412	0.282141
H	0.903331	−2.294906	−1.262818
H	2.708817	−8.277141	−1.532884
H	1.696104	−7.023772	−2.252615
H	5.050727	−6.246435	1.759690
P	4.629295	1.947636	1.090062
O	4.572932	2.251005	−0.387676
O	4.981927	3.019821	2.096956
O	5.658751	0.669386	1.246755
C	5.901892	0.166610	2.587596
C	7.006104	−0.873633	2.563058
O	6.540576	−2.084568	1.890234
C	7.473268	−2.450405	0.840104
C	8.302912	−0.435021	1.843440
C	8.219707	−1.158072	0.496002
O	9.407571	−0.866972	2.689654
H	4.982163	−0.280516	2.983853
H	6.202562	0.992883	3.242663
H	7.245209	−1.127871	3.602879
H	8.150241	−3.230087	1.193899
H	8.361284	0.649316	1.721971
H	7.633789	−0.537148	−0.186151
H	9.195577	−1.331357	0.048996
N	6.721668	−3.055261	−0.263616
C	6.826480	−4.455959	−0.501916
O	7.546718	−5.148176	0.247681
N	6.133564	−4.985512	−1.543120
C	5.323717	−4.225201	−2.303344
N	4.665385	−4.815079	−3.311196
C	5.150614	−2.825559	−2.033705
C	5.865884	−2.293761	−1.010567
H	4.780755	−5.822355	−3.506672
H	4.032421	−4.271072	−3.881885
H	4.469757	−2.217375	−2.617306
H	5.787073	−1.252901	−0.721554
P	10.990940	−0.817098	2.224485
O	11.170848	−0.012167	0.960391
O	11.770503	−0.486261	3.478677
O	11.264978	−2.385833	1.801162
C	11.215601	−3.402966	2.836577
C	11.943222	−4.662338	2.385325
O	11.182967	−5.383726	1.367130
C	11.859295	−5.326185	0.088554
C	13.334852	−4.419911	1.778481
C	13.058916	−4.376245	0.270456
O	14.169542	−5.549371	2.153190
H	10.168910	−3.644653	3.057986
H	11.694522	−3.022176	3.746352
H	12.024579	−5.323679	3.256072

H	12.167728	−6.337816	−0.190338
H	13.780356	−3.488363	2.142899
H	12.788825	−3.358417	−0.018032
H	13.920643	−4.692579	−0.322427
H	15.038732	−5.425316	1.729745
N	10.919822	−4.903590	−0.943006
C	10.346842	−3.642229	−1.100185
N	9.489077	−3.594398	−2.100932
C	9.478795	−4.886416	−2.631068
C	8.751787	−5.444185	−3.722234
O	7.932865	−4.866584	−4.477211
N	9.048277	−6.809839	−3.910249
C	9.957190	−7.535714	−3.166699
N	10.160366	−8.821652	−3.530716
N	10.642891	−7.013431	−2.144165
C	10.361604	−5.714437	−1.920117
H	10.601189	−2.834392	−0.429618
H	8.538302	−7.293351	−4.688047
H	9.565863	−9.280039	−4.240781
H	10.659458	−9.405306	−2.871260
O	6.052489	−11.549353	−10.781561
C	7.246361	−12.356766	−10.936556
C	7.742122	−12.842597	−9.580297
O	8.277981	−11.731452	−8.789945
C	7.492272	−11.547157	−7.580375
C	6.684089	−13.519818	−8.684927
C	6.221749	−12.381001	−7.773539
O	7.371459	−14.599057	−7.982839
H	5.871855	−11.122231	−11.637054
H	8.043901	−11.771706	−11.411865
H	7.036024	−13.235985	−11.562842
H	8.563942	−13.543628	−9.763980
H	8.072743	−11.877508	−6.718812
H	5.861038	−13.949985	−9.261554
H	5.452713	−11.811816	−8.299416
H	5.802841	−12.736036	−6.835036
N	7.260742	−10.113038	−7.367170
C	7.926422	−9.448702	−6.299770
O	8.743043	−10.087404	−5.599340
N	7.648712	−8.137097	−6.083329
C	6.796538	−7.464153	−6.882272
N	6.554017	−6.175665	−6.600923
C	6.185003	−8.095277	−8.016615
C	6.450194	−9.410575	−8.221260
H	7.001652	−5.718864	−5.791216
H	5.918624	−5.645838	−7.182698
H	5.544368	−7.540983	−8.692967
H	6.063152	−9.964752	−9.069859
P	6.752058	−15.372065	−6.662155
O	5.260398	−15.167326	−6.554710
O	7.351188	−16.760314	−6.701644
O	7.407192	−14.518750	−5.411797
C	8.838563	−14.603663	−5.179654
C	9.174147	−14.334521	−3.717108
O	9.050612	−12.918506	−3.376044
C	7.913533	−12.696461	−2.507836
C	8.293018	−15.086862	−2.704550
C	7.214485	−14.057859	−2.353785
O	9.151296	−15.454172	−1.584461
H	9.351665	−13.869723	−5.812811
H	9.189963	−15.608688	−5.440131
H	10.225846	−14.605314	−3.571884
H	8.271459	−12.300256	−1.552793
H	7.862123	−15.998635	−3.126096
H	6.400286	−14.156398	−3.072659
H	6.814843	−14.205452	−1.353588
N	7.053460	−11.654369	−3.061143
C	6.207906	−11.743501	−4.167328
N	5.587861	−10.607792	−4.425123

C	6.045457	−9.713031	−3.455050
C	5.735174	−8.339373	−3.224271
O	4.946293	−7.607599	−3.867694
N	6.439878	−7.818751	−2.121092
C	7.336635	−8.527838	−1.343903
N	7.927570	−7.865164	−0.335210
N	7.622709	−9.817893	−1.555312
C	6.965978	−10.345377	−2.604234
H	6.126491	−12.662677	−4.728431
H	6.291647	−6.805607	−1.900045
H	7.761117	−6.863554	−0.147056
H	8.601877	−8.373616	0.219948
P	8.546194	−16.005248	−0.148843
O	7.157770	−16.571771	−0.322282
O	9.660471	−16.831973	0.454323
O	8.355900	−14.611115	0.710835
C	9.551573	−13.861542	1.052509
C	9.214090	−12.772605	2.054910
O	8.402973	−11.743548	1.411199
C	7.227113	−11.483504	2.210210
C	8.429615	−13.247729	3.299216
C	6.999015	−12.758090	3.034853
O	9.028856	−12.601489	4.454909
H	9.972128	−13.410627	0.145288
H	10.297594	−14.534661	1.491183
H	10.157624	−12.316330	2.382047
H	7.389563	−10.614175	2.850372
H	8.477128	−14.334820	3.415493
H	6.461197	−13.511407	2.453421
H	6.444777	−12.561091	3.956010
H	8.547020	−12.907749	5.245074
N	6.134694	−11.109741	1.303835
C	5.655958	−9.790680	1.329604
O	6.031532	−8.945366	2.149211
N	4.716488	−9.505663	0.359245
C	4.162220	−10.398261	−0.552119
O	3.287940	−10.012217	−1.358363
C	4.665930	−11.758936	−0.496006
C	4.074635	−12.782872	−1.424273
C	5.644517	−12.034610	0.403018
H	4.378007	−8.507828	0.330362
H	2.992078	−12.859312	−1.266513
H	4.516456	−13.767152	−1.257189
H	4.225749	−12.499237	−2.471521
H	6.106990	−13.012934	0.465139

d(ACT)$^{4-}$

E: −27674.04 kcal mol^{-1}

O	−0.229897	−0.257200	−0.312567
C	−0.156847	0.166579	1.070595
C	1.275548	0.077496	1.581304
O	1.702705	−1.315351	1.752582
C	2.726102	−1.661708	0.790172
C	2.339551	0.730680	0.679288
C	2.872801	−0.447571	−0.141938
O	3.319507	1.343681	1.567071
H	−1.170543	−0.309651	−0.556214
H	−0.795237	−0.465955	1.700211
H	−0.490036	1.209986	1.169600
H	1.299350	0.542167	2.572573
H	3.651306	−1.895802	1.325774
H	1.920300	1.511595	0.039621
H	2.239638	−0.562430	−1.022986
H	3.895945	−0.291670	−0.475031
N	2.359633	−2.893712	0.089792
C	1.396812	−3.043831	−0.902281
N	1.323237	−4.281221	−1.366402
C	2.282118	−4.989473	−0.643893
C	2.690848	−6.343924	−0.684245
N	2.150117	−7.258215	−1.507481
N	3.677575	−6.725758	0.176298
C	4.216650	−5.816559	1.011973
N	3.905157	−4.515678	1.130620
C	2.930608	−4.150760	0.274016
H	0.770503	−2.214690	−1.201316
H	2.524471	−8.214890	−1.532561
H	1.472812	−6.965433	−2.199221
H	5.005045	−6.194724	1.660042
P	4.831052	1.792762	1.079008
O	4.888608	2.012283	−0.413152
O	5.251369	2.875104	2.047387
O	5.701344	0.419258	1.361540
C	5.832280	−0.032905	2.736490
C	6.972105	−1.030860	2.853195
O	6.620685	−2.289114	2.196637
C	7.561439	−2.587261	1.136824
C	8.300498	−0.564214	2.224778
C	8.328771	−1.285358	0.874275
O	9.368949	−0.975235	3.125506
H	4.894399	−0.502404	3.056582
H	6.039748	0.824167	3.387664
H	7.124098	−1.239052	3.918626
H	8.224179	−3.397717	1.443204
H	8.341432	0.521002	2.106403
H	7.814285	−0.664550	0.138767
H	9.344170	−1.468447	0.530773
N	6.819415	−3.111170	−0.022730
C	6.938420	−4.486587	−0.372019
O	7.700474	−5.219719	0.292742
N	6.223083	−4.949234	−1.430140
C	5.382758	−4.146925	−2.109967
N	4.698415	−4.677226	−3.133301
C	5.211138	−2.770655	−1.741584
C	5.936815	−2.306711	−0.692835
H	4.777757	−5.681104	−3.360472
H	4.034199	−4.108667	−3.641520
H	4.517138	−2.128905	−2.270620
H	5.849455	−1.292852	−0.318527
P	10.958957	−0.741223	2.733003
O	11.097595	0.301281	1.650422
O	11.676466	−0.599509	4.057449
O	11.365378	−2.175737	2.029748
C	11.386662	−3.357927	2.873072
C	11.937985	−4.540645	2.097924
O	10.981202	−4.941277	1.071962
C	11.661399	−5.082240	−0.194025
C	13.287020	−4.291579	1.382715
C	12.898879	−4.177981	−0.097552
O	14.130610	−5.445540	1.645641
H	10.368578	−3.584547	3.212672
H	12.019771	−3.174991	3.749211
H	12.061556	−5.373045	2.803521
H	11.936083	−6.125553	−0.362192
H	13.776344	−3.384032	1.748900
H	12.645288	−3.139218	−0.324099
H	13.699902	−4.498619	−0.768339
H	14.989841	−5.284832	1.214241
N	10.714434	−4.744370	−1.262854
C	10.258134	−5.764892	−2.113004
O	10.669379	−6.928576	−2.061328
N	9.308830	−5.361128	−3.030175
C	8.844453	−4.067184	−3.236065
O	8.017803	−3.830459	−4.143554
C	9.378329	−3.051713	−2.345441
C	8.959841	−1.621705	−2.547116

C	10.260224	−3.447450	−1.392626
H	8.958058	−6.113822	−3.680621
H	9.231631	−1.286138	−3.555533
H	9.446127	−0.963478	−1.824308
H	7.874220	−1.509341	−2.456640
H	10.669857	−2.752200	−0.669620
O	6.158316	−11.522972	−10.757958
C	7.338757	−12.350364	−10.899073
C	7.748512	−12.932439	−9.551704
O	8.284626	−11.898210	−8.661304
C	7.388373	−11.670242	−7.545768
C	6.622645	−13.619910	−8.755507
C	6.133930	−12.511644	−7.818937
O	7.227582	−14.762549	−8.080310
H	6.023366	−11.055415	−11.600580
H	8.175419	−11.760291	−11.294351
H	7.142740	−13.185443	−11.587754
H	8.557609	−13.647075	−9.736087
H	7.896901	−11.962736	−6.622110
H	5.821317	−13.989031	−9.401170
H	5.385188	−11.924587	−8.352318
H	5.679214	−12.899152	−6.910178
N	7.115935	−10.241823	−7.403639
C	6.242816	−9.461761	−8.151587
N	6.193604	−8.204087	−7.741281
C	7.081696	−8.146258	−6.668341
C	7.471155	−7.088763	−5.813693
N	6.999831	−5.834318	−5.918643
N	8.388953	−7.374567	−4.846194
C	8.885807	−8.623755	−4.748189
N	8.591724	−9.690376	−5.507501
C	7.675423	−9.397547	−6.450437
H	5.708211	−9.872102	−8.996612
H	7.292446	−5.112944	−5.247152
H	6.263215	−5.635182	−6.582242
H	9.607811	−8.771616	−3.947313
P	6.559769	−15.510941	−6.769369
O	5.069191	−15.281828	−6.700082
O	7.134875	−16.909744	−6.784023
O	7.201655	−14.652112	−5.516465
C	8.633860	−14.742788	−5.289401
C	8.969251	−14.371185	−3.853046
O	8.813098	−12.934442	−3.628261
C	7.756553	−12.679995	−2.670987
C	8.096847	−15.071494	−2.793921
C	7.034329	−14.019307	−2.467112
O	8.967725	−15.401714	−1.673229
H	9.155202	−14.066808	−5.978022
H	8.970087	−15.769093	−5.476432
H	10.024194	−14.616547	−3.686006
H	8.193443	−12.300680	−1.741535
H	7.650394	−15.994306	−3.172173
H	6.215293	−14.125835	−3.180335
H	6.639956	−14.129384	−1.459729
N	6.896699	−11.608641	−3.159215
C	5.976856	−11.654842	−4.208204
N	5.365956	−10.500774	−4.397749
C	5.903970	−9.638544	−3.438106
C	5.650660	−8.261017	−3.157613
O	4.848979	−7.488971	−3.734459
N	6.439499	−7.786013	−2.091398
C	7.373262	−8.531912	−1.397663
N	8.035927	−7.917898	−0.405227
N	7.620935	−9.820069	−1.668888
C	6.868528	−10.310019	−2.669794
H	5.826652	−12.560680	−4.777706
H	6.331932	−6.775687	−1.843307
H	7.893923	−6.923345	−0.171159
H	8.734402	−8.456154	0.088701
P	8.372750	−15.956813	−0.233913
O	6.979490	−16.513149	−0.398674
O	9.486670	−16.793034	0.356955
O	8.201011	−14.564288	0.631644
C	9.407232	−13.829753	0.969439
C	9.087370	−12.736920	1.973401
O	8.290034	−11.695577	1.332636
C	7.111765	−11.429727	2.125020
C	8.298776	−13.202306	3.218345
C	6.872661	−12.701287	2.951849
O	8.902702	−12.558497	4.372994
H	9.829733	−13.383361	0.060935
H	10.146259	−14.512740	1.404724
H	10.038104	−12.294432	2.298476
H	7.275377	−10.559607	2.763753
H	8.337026	−14.289501	3.337269
H	6.329431	−13.451892	2.372194
H	6.319230	−12.497760	3.872036
H	8.416009	−12.856843	5.163218
N	6.023512	−11.051637	1.214618
C	5.559388	−9.727228	1.230838
O	5.956312	−8.874389	2.032062
N	4.608205	−9.444415	0.271297
C	4.023355	−10.342708	−0.615081
O	3.129811	−9.958286	−1.400367
C	4.521444	−11.706018	−0.556945
C	3.905391	−12.736982	−1.461666
C	5.512588	−11.980497	0.329100
H	4.270933	−8.445972	0.246972
H	2.823049	−12.791623	−1.293559
H	4.331893	−13.725697	−1.279754
H	4.051567	−12.476007	−2.515492
H	5.968095	−12.962072	0.393184

d(AGA)$^{4-}$

E: −27673.44 kcal mol^{-1}

O	−0.190578	−0.387978	−0.360421
C	−0.161955	0.026279	1.027013
C	1.264566	0.002939	1.561867
O	1.759780	−1.367042	1.725224
C	2.804311	−1.654950	0.763017
C	2.308459	0.720165	0.686204
C	2.919061	−0.416658	−0.140478
O	3.242218	1.369586	1.597445
H	−1.122612	−0.501155	−0.616013
H	−0.778515	−0.643004	1.640356
H	−0.548211	1.051080	1.129637
H	1.247448	0.456553	2.558585
H	3.731038	−1.866419	1.305213
H	1.860400	1.486947	0.049012
H	2.324407	−0.539402	−1.046346
H	3.946503	−0.211854	−0.431372
N	2.490099	−2.884483	0.033746
C	1.609176	−3.043019	−1.029529
N	1.556699	−4.287724	−1.476804
C	2.443570	−4.993452	−0.666126
C	2.829535	−6.353638	−0.641678
N	2.341797	−7.281250	−1.486358
N	3.730885	−6.730738	0.309892
C	4.205477	−5.813061	1.175853
N	3.905404	−4.506325	1.239965
C	3.023193	−4.144685	0.288474
H	1.017795	−2.216153	−1.395391
H	2.730989	−8.233287	−1.482554
H	1.776367	−6.979830	−2.269296
H	4.923712	−6.190368	1.901344

P	4.707058	1.952588	1.106146
O	4.710577	2.250488	−0.373882
O	5.063467	3.018319	2.118995
O	5.684691	0.640383	1.302966
C	5.879381	0.144104	2.653525
C	6.978447	−0.903141	2.678547
O	6.533967	−2.129069	2.014723
C	7.452329	−2.469121	0.944253
C	8.296529	−0.485293	1.988997
C	8.201462	−1.173268	0.625235
O	9.375720	−0.971157	2.841776
H	4.944173	−0.295287	3.021502
H	6.162573	0.972859	3.313266
H	7.181951	−1.141582	3.729220
H	8.129810	−3.263113	1.275890
H	8.391631	0.599451	1.896245
H	7.598070	−0.539517	−0.029322
H	9.170540	−1.338183	0.158699
N	6.710149	−3.029681	−0.170195
C	5.875288	−2.366415	−1.068226
N	5.352970	−3.176702	−1.969471
C	5.864690	−4.440307	−1.661701
C	5.676084	−5.707884	−2.286051
O	4.945312	−5.978253	−3.268657
N	6.444013	−6.717425	−1.674344
C	7.264330	−6.536518	−0.577254
N	7.931787	−7.612766	−0.122283
N	7.404508	−5.356454	0.038785
C	6.710151	−4.364912	−0.544531
H	5.702047	−1.303040	−0.994705
H	6.395956	−7.667822	−2.113834
H	7.953487	−8.499014	−0.648546
H	8.626527	−7.443954	0.594170
P	10.939935	−1.150386	2.349377
O	11.234575	−0.321014	1.122943
O	11.766666	−1.007954	3.607911
O	10.964134	−2.717995	1.839412
C	10.822264	−3.782297	2.817578
C	11.585212	−5.025212	2.374905
O	10.916858	−5.707744	1.268430
C	11.675070	−5.556304	0.047001
C	13.019441	−4.754248	1.891457
C	12.859595	−4.626427	0.369788
O	13.824225	−5.904875	2.264728
H	9.759642	−4.026945	2.931980
H	11.220554	−3.448860	3.782439
H	11.596395	−5.723300	3.220099
H	12.000251	−6.546798	−0.283145
H	13.434322	−3.844943	2.338822
H	12.619784	−3.591914	0.117783
H	13.762837	−4.919099	−0.170972
H	14.722870	−5.760289	1.916075
N	10.801432	−5.061535	−1.015398
C	10.270274	−3.785345	−1.161206
N	9.437917	−3.685941	−2.184592
C	9.400681	−4.965279	−2.738418
C	8.685173	−5.518704	−3.825771
N	7.848976	−4.815708	−4.616243
N	8.868083	−6.843862	−4.083184
C	9.706222	−7.568569	−3.318536
N	10.435319	−7.144458	−2.274751
C	10.240024	−5.835386	−2.022092
H	10.524922	−2.996047	−0.468327
H	7.265338	−5.319495	−5.294517
H	7.580550	−3.881273	−4.334580
H	9.782828	−8.620538	−3.585745
O	5.934797	−11.737501	−10.686212
C	7.145035	−12.509542	−10.889103
C	7.703913	−12.979185	−9.551937

O	8.258925	−11.854104	−8.792039
C	7.528168	−11.666785	−7.555656
C	6.688593	−13.661255	−8.612627
C	6.264657	−12.529222	−7.674145
O	7.400689	−14.742582	−7.942464
H	5.701991	−11.324281	−11.535883
H	7.906308	−11.902471	−11.395131
H	6.934967	−13.395371	−11.505647
H	8.527260	−13.670285	−9.762181
H	8.153909	−11.965151	−6.712774
H	5.839861	−14.086078	−9.154446
H	5.452981	−11.972832	−8.147081
H	5.913244	−12.900068	−6.714393
N	7.266726	−10.231854	−7.357276
C	7.920985	−9.561932	−6.309587
O	8.753517	−10.100165	−5.573188
N	7.540851	−8.244775	−6.153744
C	6.648779	−7.528050	−6.942607
O	6.404937	−6.329113	−6.679316
C	6.065023	−8.257616	−8.056283
C	5.124675	−7.545531	−8.990414
C	6.409712	−9.562335	−8.211389
H	8.012886	−7.724802	−5.364272
H	5.609496	−6.671700	−9.443248
H	4.241495	−7.179247	−8.452159
H	4.791885	−8.214062	−9.789784
H	6.042813	−10.157792	−9.041878
P	6.781468	−15.547752	−6.639048
O	5.288942	−15.350761	−6.534479
O	7.389697	−16.930694	−6.702484
O	7.429367	−14.712460	−5.371234
C	8.865610	−14.783300	−5.160597
C	9.220547	−14.344624	−3.747757
O	9.027430	−12.904803	−3.585461
C	8.025814	−12.636219	−2.576153
C	8.397982	−15.020635	−2.632849
C	7.330939	−13.975796	−2.298452
O	9.311168	−15.292116	−1.529844
H	9.371535	−14.133303	−5.884785
H	9.206363	−15.814337	−5.308996
H	10.285616	−14.555548	−3.597128
H	8.500649	−12.226297	−1.683794
H	7.955359	−15.964323	−2.960245
H	6.480215	−14.125711	−2.965153
H	6.986495	−14.055539	−1.270022
N	7.126555	−11.573366	−3.057326
C	7.181171	−10.278939	−2.463660
O	7.994112	−10.063292	−1.540896
N	6.323491	−9.327515	−2.917102
C	5.473762	−9.576392	−3.931491
N	4.660717	−8.588221	−4.329441
C	5.451740	−10.853349	−4.583466
C	6.300096	−11.807184	−4.122543
H	4.738982	−7.642882	−3.917677
H	4.027568	−8.741426	−5.102983
H	4.792949	−11.044935	−5.421548
H	6.382042	−12.789961	−4.573033
P	8.761648	−15.852680	−0.073220
O	7.397556	−16.482448	−0.218971
O	9.923461	−16.621752	0.516457
O	8.522752	−14.461632	0.777708
C	9.691589	−13.678097	1.136547
C	9.311436	−12.604140	2.139913
O	8.498340	−11.584264	1.485065
C	7.305336	−11.341065	2.261432
C	8.507456	−13.101431	3.363513
C	7.077450	−12.621697	3.078435
O	9.079204	−12.464757	4.538253
H	10.108587	−13.209566	0.236501

H	10.451397	−14.331999	1.580269
H	10.239165	−12.134462	2.492513
H	7.442441	−10.470532	2.906124
H	8.562270	−14.189316	3.467566
H	6.554687	−13.377074	2.485939
H	6.507180	−12.433751	3.991640
H	8.588542	−12.789103	5.315648
N	6.225117	−10.981164	1.333953
C	5.702222	−9.678373	1.371310
O	6.031342	−8.836616	2.213311
N	4.773399	−9.409412	0.385674
C	4.236598	−10.315264	−0.523241
O	3.347764	−9.953915	−1.324566
C	4.771217	−11.664548	−0.465696
C	4.180686	−12.712351	−1.367838
C	5.759325	−11.915439	0.430442
H	4.392297	−8.427239	0.375843
H	3.106739	−12.817598	−1.171185
H	4.654244	−13.682734	−1.206443
H	4.284528	−12.435956	−2.422157
H	6.241735	−12.883826	0.494700

d(AGC)$^{4-}$

E: −27585.92 kcal mol^{-1}

O	−0.288401	−0.431941	−0.244570
C	−0.226679	−0.042625	1.149050
C	1.211531	−0.083750	1.650112
O	1.703480	−1.460414	1.764420
C	2.733649	−1.720682	0.779261
C	2.236466	0.653168	0.767903
C	2.820819	−0.464624	−0.100824
O	3.194715	1.279031	1.670393
H	−1.226812	−0.525191	−0.484279
H	−0.833293	−0.719693	1.763829
H	−0.604506	0.982207	1.278664
H	1.219865	0.343607	2.658477
H	3.671484	−1.933974	1.301562
H	1.774453	1.436204	0.161124
H	2.194517	−0.569329	−0.987376
H	3.836534	−0.252196	−0.424826
N	2.418070	−2.936610	0.028764
C	1.516439	−3.075119	−1.019969
N	1.481851	−4.302559	−1.513840
C	2.403846	−5.017137	−0.751366
C	2.819367	−6.368533	−0.789921
N	2.337307	−7.268385	−1.664236
N	3.750318	−6.762845	0.126244
C	4.223217	−5.868776	1.017540
N	3.897831	−4.571533	1.139197
C	2.986138	−4.192880	0.222228
H	0.899178	−2.248421	−1.341099
H	2.695850	−8.232084	−1.670517
H	1.707785	−6.961328	−2.393743
H	4.963919	−6.257988	1.713572
P	4.662955	1.843754	1.167330
O	4.663922	2.131104	−0.314641
O	5.037034	2.912069	2.170796
O	5.626497	0.521787	1.368485
C	5.855710	0.051859	2.723145
C	7.027966	−0.914068	2.753185
O	6.666014	−2.183300	2.121697
C	7.531476	−2.448630	0.992004
C	8.296318	−0.412198	2.031643
C	8.226053	−1.117670	0.675135
O	9.431146	−0.806443	2.855757
H	4.953935	−0.450286	3.094432
H	6.079809	0.904297	3.375208
H	7.262808	−1.118888	3.803994
H	8.245855	−3.235883	1.256028
H	8.306184	0.675232	1.923766
H	7.604145	−0.513062	0.011638
H	9.205537	−1.241255	0.217661
N	6.746986	−2.986575	−0.109849
C	5.877154	−2.298222	−0.956835
N	5.351263	−3.075825	−1.884040
C	5.891315	−4.342161	−1.647682
C	5.724619	−5.577632	−2.341057
O	4.998110	−5.807240	−3.336853
N	6.506610	−6.606217	−1.783647
C	7.332872	−6.469466	−0.684247
N	8.028097	−7.553399	−0.301693
N	7.475322	−5.314630	−0.020565
C	6.757072	−4.305485	−0.544211
H	5.681518	−1.244520	−0.824064
H	6.414188	−7.553496	−2.220270
H	7.887622	−8.480543	−0.732061
H	8.585430	−7.471962	0.537897
P	10.997224	−0.739442	2.335774
O	11.133291	0.113934	1.098509
O	11.818397	−0.459131	3.574690
O	11.253227	−2.292433	1.840867
C	11.215715	−3.343591	2.843603
C	11.850809	−4.612551	2.298914
O	11.005947	−5.204779	1.263647
C	11.722990	−5.283972	0.011259
C	13.244677	−4.423707	1.669431
C	12.955252	−4.376218	0.164103
O	14.042567	−5.580173	2.040739
H	10.173863	−3.542614	3.122289
H	11.766801	−3.021435	3.734856
H	11.918138	−5.329041	3.127048
H	11.995470	−6.319568	−0.192997
H	13.727873	−3.507799	2.024656
H	12.720018	−3.349520	−0.127373
H	13.798410	−4.726160	−0.436767
H	14.918660	−5.477953	1.625762
N	10.811275	−4.892824	−1.077301
C	10.433906	−5.839216	−2.066123
O	10.922138	−6.993158	−2.035269
N	9.551678	−5.451269	−3.024866
C	9.063090	−4.194829	−3.056590
N	8.214166	−3.875302	−4.045148
C	9.432722	−3.228467	−2.065867
C	10.290082	−3.626541	−1.092963
H	7.896595	−4.591713	−4.716791
H	7.762038	−2.970259	−4.042145
H	9.022523	−2.226303	−2.079547
H	10.593866	−2.985172	−0.272677
O	5.985935	−11.523679	−10.761624
C	7.129870	−12.393243	−10.944557
C	7.600660	−12.950287	−9.606276
O	8.212539	−11.907702	−8.776360
C	7.393454	−11.639113	−7.610082
C	6.506208	−13.584910	−8.727569
C	6.095115	−12.434438	−7.805234
O	7.131851	−14.709054	−8.039056
H	5.809795	−11.086152	−11.612774
H	7.959931	−11.842500	−11.405329
H	6.867875	−13.240777	−11.594816
H	8.378525	−13.691955	−9.816563
H	7.940803	−11.947751	−6.714490
H	5.664397	−13.962969	−9.313486
H	5.350355	−11.828205	−8.324160
H	5.666341	−12.784354	−6.868722
N	7.192452	−10.201541	−7.465512

C	6.398264	−9.362602	−8.249933
N	6.434019	−8.106749	−7.843275
C	7.291684	−8.104912	−6.740987
C	7.707772	−7.047375	−5.880434
O	7.381198	−5.835931	−5.942005
N	8.581872	−7.496303	−4.873444
C	9.022531	−8.798461	−4.730591
N	9.899065	−9.046284	−3.736751
N	8.630720	−9.792892	−5.534299
C	7.779439	−9.397075	−6.497945
H	5.863170	−9.743747	−9.108875
H	8.931270	−6.771249	−4.204341
H	10.195755	−8.312954	−3.077689
H	10.100998	−10.016412	−3.531348
P	6.492370	−15.433585	−6.701324
O	5.008261	−15.180223	−6.593669
O	7.050745	−16.838551	−6.710797
O	7.178024	−14.569152	−5.473986
C	8.613015	−14.669949	−5.268829
C	8.969448	−14.305599	−3.835110
O	8.813222	−12.870891	−3.600516
C	7.790314	−12.623282	−2.606882
C	8.116476	−15.016679	−2.766645
C	7.074046	−13.962361	−2.381871
O	9.010294	−15.380807	−1.675485
H	9.127565	−13.993982	−5.962263
H	8.939675	−15.697507	−5.465032
H	10.027218	−14.550569	−3.685771
H	8.248054	−12.243424	−1.692764
H	7.648528	−15.925458	−3.152117
H	6.213879	−14.067918	−3.044651
H	6.738099	−14.077385	−1.353981
N	6.917519	−11.532644	−3.081658
C	6.979833	−10.256777	−2.451156
O	7.716706	−10.103558	−1.455165
N	6.220945	−9.249181	−2.958357
C	5.412980	−9.446738	−4.017886
N	4.683183	−8.410928	−4.454684
C	5.345856	−10.720930	−4.670881
C	6.128301	−11.717441	−4.185230
H	4.782842	−7.475649	−4.027525
H	4.113706	−8.514482	−5.284070
H	4.715567	−10.874308	−5.538164
H	6.186858	−12.696632	−4.647319
P	8.433045	−16.049821	−0.277246
O	7.057914	−16.635892	−0.487464
O	9.572058	−16.889187	0.259372
O	8.214834	−14.728100	0.683334
C	9.396748	−13.987230	1.085422
C	9.014496	−12.888699	2.059709
O	8.243171	−11.866724	1.361447
C	7.060805	−11.548333	2.128390
C	8.167641	−13.342442	3.272671
C	6.764248	−12.803391	2.961510
O	8.748694	−12.724581	4.452751
H	9.869806	−13.543614	0.200713
H	10.112410	−14.664183	1.567064
H	9.943325	−12.434703	2.430381
H	7.244124	−10.678798	2.763022
H	8.172434	−14.430909	3.384367
H	6.215412	−13.545039	2.375536
H	6.192429	−12.575190	3.864463
H	8.239085	−13.030506	5.225386
N	6.005791	−11.141336	1.193852
C	5.615194	−9.792373	1.159952
O	6.051646	−8.937775	1.937631
N	4.684020	−9.491080	0.186239
C	4.081353	−10.380902	−0.696106
O	3.220862	−9.979218	−1.509327
C	4.517973	−11.762533	−0.601238
C	3.878903	−12.786144	−1.498529
C	5.469900	−12.063321	0.318393
H	4.370745	−8.486131	0.156418
H	2.795745	−12.818407	−1.330060
H	4.285495	−13.782257	−1.310269
H	4.029084	−12.535380	−2.554500
H	5.867790	−13.066060	0.418704

d(AGG)$^{4-}$

E: −27583.81 kcal mol^{-1}

O	−0.236105	−0.430175	−0.226402
C	−0.204945	−0.046971	1.169962
C	1.226497	−0.050602	1.692092
O	1.753086	−1.412469	1.821982
C	2.794910	−1.657688	0.844893
C	2.246280	0.708527	0.823427
C	2.880458	−0.396102	−0.029157
O	3.170118	1.363855	1.740520
H	−1.167597	−0.557589	−0.477329
H	−0.801521	−0.743639	1.772709
H	−0.612997	0.966353	1.298969
H	1.208470	0.381010	2.698520
H	3.729512	−1.865496	1.374670
H	1.774989	1.475476	0.203532
H	2.288883	−0.510917	−0.938045
H	3.903567	−0.162372	−0.314335
N	2.496913	−2.874960	0.087895
C	1.611041	−3.022866	−0.972841
N	1.574657	−4.257560	−1.448554
C	2.477724	−4.967818	−0.660016
C	2.882738	−6.322586	−0.669293
N	2.402931	−7.236077	−1.532923
N	3.794578	−6.709252	0.268497
C	4.260825	−5.804985	1.152764
N	3.943482	−4.503921	1.248170
C	3.051155	−4.132622	0.309883
H	1.004606	−2.197236	−1.315952
H	2.799729	−8.184992	−1.550138
H	1.825027	−6.924895	−2.302762
H	4.986752	−6.188813	1.866931
P	4.604145	2.014041	1.241553
O	4.569564	2.354085	−0.228883
O	4.940082	3.062247	2.279606
O	5.631791	0.733170	1.382151
C	5.859595	0.200504	2.713512
C	6.961372	−0.842803	2.682431
O	6.500089	−2.049431	1.995991
C	7.422559	−2.387770	0.928869
C	8.263626	−0.401089	1.974970
C	8.164990	−1.087817	0.610789
O	9.362390	−0.864984	2.813830
H	4.934737	−0.251961	3.091560
H	6.156010	1.011770	3.389254
H	7.192627	−1.108529	3.721079
H	8.104328	−3.177955	1.261792
H	8.336691	0.685269	1.881896
H	7.555840	−0.456638	−0.041221
H	9.133213	−1.247750	0.139945
N	6.685444	−2.950970	−0.186888
C	5.842803	−2.291318	−1.080853
N	5.338079	−3.100089	−1.993186
C	5.870328	−4.358026	−1.698397
C	5.708872	−5.619691	−2.341339
O	4.994076	−5.888311	−3.336604
N	6.478883	−6.627075	−1.728899

C	7.279206	−6.448324	−0.615863
N	7.944094	−7.522717	−0.157402
N	7.404610	−5.271049	0.008040
C	6.707233	−4.282231	−0.575221
H	5.649922	−1.232128	−0.996253
H	6.431434	−7.580205	−2.161669
H	7.919280	−8.434729	−0.637685
H	8.598206	−7.369192	0.598701
P	10.924589	−1.007143	2.303384
O	11.183049	−0.180950	1.066603
O	11.762964	−0.828455	3.550132
O	10.982599	−2.578504	1.810373
C	10.864103	−3.627994	2.808116
C	11.617569	−4.876978	2.367895
O	10.918155	−5.576328	1.292430
C	11.663021	−5.479343	0.055305
C	13.038915	−4.611714	1.844243
C	12.842660	−4.526380	0.324815
O	13.860296	−5.746791	2.230291
H	9.804490	−3.870787	2.951478
H	11.284523	−3.278658	3.757949
H	11.654161	−5.559026	3.225643
H	11.995979	−6.481263	−0.230344
H	13.458175	−3.688351	2.257393
H	12.580191	−3.502254	0.052298
H	13.736993	−4.820193	−0.230332
H	14.750603	−5.603896	1.860092
N	10.778769	−5.042016	−1.018734
C	10.226203	−3.774637	−1.210061
N	9.432679	−3.720617	−2.261982
C	9.442614	−5.013625	−2.791466
C	8.771434	−5.571917	−3.918208
O	8.002109	−4.991160	−4.722666
N	9.051339	−6.943800	−4.074760
C	9.908760	−7.676864	−3.278188
N	10.114375	−8.965900	−3.621619
N	10.543472	−7.154697	−2.222636
C	10.271830	−5.849067	−2.025895
H	10.441062	−2.966448	−0.525546
H	8.577546	−7.427198	−4.872739
H	9.601228	−9.412272	−4.397497
H	10.585133	−9.552911	−2.945261
O	5.861549	−11.856764	−10.787547
C	7.034869	−12.678504	−11.010429
C	7.639900	−13.110466	−9.680872
O	8.240842	−11.967035	−8.986899
C	7.563245	−11.730030	−7.727431
C	6.653702	−13.740015	−8.675367
C	6.275497	−12.560431	−7.777676
O	7.384426	−14.792049	−7.977947
H	5.611350	−11.461134	−11.640716
H	7.791447	−12.119233	−11.575039
H	6.768461	−13.581411	−11.578631
H	8.442815	−13.823030	−9.898939
H	8.212019	−12.026939	−6.902949
H	5.783457	−14.184345	−9.164995
H	5.468359	−12.004178	−8.258866
H	5.937688	−12.879818	−6.794283
N	7.348607	−10.285743	−7.551702
C	8.046141	−9.592109	−6.523976
O	8.896203	−10.205602	−5.841212
N	7.746017	−8.284861	−6.311734
C	6.852037	−7.638887	−7.087408
N	6.580303	−6.358465	−6.799021
C	6.213179	−8.296604	−8.191350
C	6.491478	−9.611139	−8.384374
H	7.051519	−5.881535	−6.014687
H	5.916944	−5.845309	−7.364631
H	5.536279	−7.764570	−8.849778
H	6.075044	−10.190151	−9.202582
P	6.809002	−15.523776	−6.612313
O	5.317957	−15.332594	−6.480656
O	7.429055	−16.902565	−6.611725
O	7.479914	−14.610956	−5.411739
C	8.915696	−14.680819	−5.197295
C	9.263922	−14.272828	−3.772210
O	9.099927	−12.832434	−3.580742
C	8.079280	−12.560424	−2.593105
C	8.412972	−14.954404	−2.682835
C	7.363063	−13.894808	−2.339975
O	9.303630	−15.270005	−1.572902
H	9.421125	−14.013094	−5.905268
H	9.259727	−15.707298	−5.366261
H	10.322803	−14.507002	−3.613475
H	8.535394	−12.157209	−1.688015
H	7.954757	−15.880657	−3.037274
H	6.513571	−14.017864	−3.013861
H	7.011405	−13.983462	−1.314924
N	7.199681	−11.487581	−3.093184
C	7.187074	−10.218145	−2.446027
O	7.896015	−10.040359	−1.434250
N	6.384874	−9.246165	−2.956107
C	5.604665	−9.472469	−4.030497
N	4.820670	−8.476007	−4.463412
C	5.617521	−10.739130	−4.701926
C	6.441277	−11.699874	−4.212897
H	4.869123	−7.537626	−4.031088
H	4.259623	−8.607368	−5.294454
H	5.009807	−10.913816	−5.581124
H	6.555895	−12.672289	−4.678955
P	8.718206	−15.874554	−0.148342
O	7.351831	−16.485399	−0.343913
O	9.860342	−16.673992	0.439680
O	8.473715	−14.510995	0.744408
C	9.640085	−13.745456	1.146650
C	9.243163	−12.684900	2.158274
O	8.456163	−11.646829	1.499972
C	7.240621	−11.412358	2.245036
C	8.402115	−13.195411	3.350802
C	6.982550	−12.707602	3.028580
O	8.939797	−12.575312	4.549968
H	10.084215	−13.265249	0.265917
H	10.382464	−14.414150	1.597998
H	10.165380	−12.229568	2.542724
H	7.365436	−10.557035	2.912088
H	8.450877	−14.284674	3.442729
H	6.479932	−13.451585	2.405423
H	6.382605	−12.535962	3.925849
H	8.423928	−12.906114	5.308066
N	6.190455	−11.023306	1.295772
C	5.715011	−9.701658	1.315955
O	6.062428	−8.866736	2.157663
N	4.809739	−9.405042	0.316616
C	4.274519	−10.290532	−0.612765
O	3.414381	−9.902663	−1.432942
C	4.775309	−11.652171	−0.553854
C	4.195518	−12.673987	−1.492410
C	5.726245	−11.937320	0.371785
H	4.451313	−8.414715	0.308312
H	3.110555	−12.747249	−1.349036
H	4.631088	−13.659931	−1.317833
H	4.359189	−12.392571	−2.538410
H	6.178331	−12.919512	0.442744

d(ATA)$^{4-}$

E: −27763.43 kcal mol^{-1}

O	−0.260246	−0.441634	−0.011015
C	−0.158397	−0.107968	1.395165
C	1.295558	−0.152516	1.848627
O	1.796509	−1.528851	1.932054
C	2.794861	−1.778454	0.915672
C	2.285931	0.601031	0.941371
C	2.874290	−0.504617	0.058185
O	3.254525	1.247025	1.815612
H	−1.204965	−0.532368	−0.226013
H	−0.739864	−0.814588	2.000745
H	−0.540701	0.907171	1.575413
H	1.336045	0.261188	2.861617
H	3.745404	−2.017615	1.402640
H	1.793810	1.371806	0.343099
H	2.254086	−0.600991	−0.833572
H	3.891887	−0.282778	−0.254250
N	2.437243	−2.974025	0.146337
C	1.428761	−3.091418	−0.804287
N	1.350739	−4.304638	−1.327104
C	2.350309	−5.033599	−0.685056
C	2.764649	−6.382191	−0.799614
N	2.211828	−7.263417	−1.650606
N	3.773508	−6.796225	0.018110
C	4.332802	−5.925511	0.879907
N	4.027224	−4.629592	1.058384
C	3.026857	−4.231674	0.247011
H	0.776507	−2.258866	−1.029133
H	2.570257	−8.224406	−1.705404
H	1.496410	−6.954553	−2.295068
H	5.135897	−6.331969	1.491096
P	4.643620	1.931043	1.237284
O	4.523695	2.256064	−0.231469
O	5.008732	2.994095	2.249533
O	5.708430	0.675738	1.334258
C	5.992589	0.146780	2.656250
C	7.097546	−0.888515	2.579953
O	6.616413	−2.081707	1.885495
C	7.546269	−2.436060	0.832486
C	8.382022	−0.432736	1.845981
C	8.282958	−1.138737	0.490351
O	9.500267	−0.865483	2.673772
H	5.086309	−0.312397	3.069479
H	6.309425	0.960221	3.319939
H	7.359742	−1.169766	3.607137
H	8.232561	−3.211319	1.180613
H	8.432103	0.653367	1.736763
H	7.684432	−0.511074	−0.174194
H	9.252653	−1.302775	0.026111
N	6.795289	−3.045575	−0.268489
C	6.927583	−4.429241	−0.483253
O	7.644312	−5.157152	0.205959
N	6.188540	−4.914137	−1.543011
C	5.315403	−4.199984	−2.352658
O	4.682665	−4.779481	−3.261877
C	5.202244	−2.780105	−2.061419
C	4.288560	−1.936684	−2.906948
C	5.937120	−2.283471	−1.032692
H	6.289593	−5.952312	−1.729052
H	4.573692	−2.000480	−3.963980
H	4.327122	−0.888270	−2.601391
H	3.252172	−2.287019	−2.835354
H	5.878202	−1.240520	−0.744242
P	11.071753	−0.884613	2.169315
O	11.258521	−0.079751	0.906257
O	11.892641	−0.599557	3.407555
O	11.263711	−2.460448	1.726126
C	11.206578	−3.485454	2.753300
C	11.931355	−4.743193	2.293582
O	11.176558	−5.448013	1.258866
C	11.857581	−5.367718	−0.013747
C	13.328804	−4.501417	1.700698
C	13.065394	−4.433173	0.190253
O	14.152485	−5.641463	2.065189
H	10.158137	−3.724469	2.968760
H	11.684078	−3.114782	3.667765
H	12.000715	−5.416728	3.155755
H	12.156544	−6.375729	−0.314920
H	13.777349	−3.577837	2.081157
H	12.810778	−3.408119	−0.085728
H	13.928306	−4.751664	−0.399442
H	15.027973	−5.514687	1.655835
N	10.922179	−4.914656	−1.041352
C	10.381702	−3.644643	−1.192042
N	9.512143	−3.567001	−2.186616
C	9.466248	−4.855115	−2.718464
C	8.737737	−5.420427	−3.791080
N	7.871966	−4.733643	−4.558308
N	8.941866	−6.741666	−4.054942
C	9.797027	−7.453032	−3.298760
N	10.531804	−7.019686	−2.261096
C	10.330095	−5.709831	−2.014152
H	10.661180	−2.842440	−0.524042
H	7.334768	−5.218834	−5.287318
H	7.676860	−3.764600	−4.346780
H	9.886372	−8.503901	−3.567439
O	5.854009	−11.700199	−10.565596
C	7.039635	−12.513623	−10.750658
C	7.601628	−12.950302	−9.403711
O	8.186407	−11.812730	−8.686104
C	7.472261	−11.577537	−7.446377
C	6.585063	−13.582317	−8.429703
C	6.186053	−12.405019	−7.536835
O	7.292675	−14.650691	−7.731839
H	5.624905	−11.312241	−11.428138
H	7.811956	−11.947451	−11.286489
H	6.798726	−13.415528	−11.331832
H	8.408985	−13.664049	−9.600435
H	8.095983	−11.877050	−6.603184
H	5.726108	−14.017685	−8.946810
H	5.397145	−11.844358	−8.042315
H	5.813851	−12.717596	−6.564421
N	7.254434	−10.134050	−7.276959
C	7.922476	−9.466649	−6.238002
O	8.744072	−10.012492	−5.495212
N	7.576707	−8.136568	−6.105555
C	6.725788	−7.404681	−6.924774
O	6.523835	−6.190094	−6.692591
C	6.132705	−8.134696	−8.032293
C	5.238018	−7.407218	−8.999469
C	6.430681	−9.454713	−8.153921
H	8.066639	−7.616714	−5.328062
H	5.765900	−6.564415	−9.462894
H	4.360212	−6.993667	−8.487072
H	4.893580	−8.080277	−9.790159
H	6.051793	−10.055665	−8.975220
P	6.770352	−15.327206	−6.319583
O	5.289601	−15.115496	−6.120740
O	7.377310	−16.711804	−6.300415
O	7.501348	−14.380365	−5.185151
C	8.940236	−14.454860	−5.010235
C	9.316871	−14.221997	−3.551755
O	9.207574	−12.814872	−3.168310
C	8.073912	−12.602789	−2.297646
C	8.451817	−14.998053	−2.544803
C	7.391511	−13.972676	−2.121026
O	9.330943	−15.419104	−1.462544
H	9.420866	−13.698005	−5.641342

H	9.293078	−15.448525	−5.308419
H	10.370437	−14.497475	−3.435503
H	8.429183	−12.189654	−1.349612
H	7.996241	−15.886005	−2.989513
H	6.531505	−14.069065	−2.784009
H	7.063150	−14.127336	−1.095531
N	7.191212	−11.577551	−2.861103
C	6.323394	−11.711157	−3.937860
N	5.684212	−10.590748	−4.229978
C	6.155796	−9.661031	−3.305138
C	5.861303	−8.292962	−3.097324
N	4.989746	−7.594172	−3.841947
N	6.512536	−7.664599	−2.078842
C	7.404985	−8.341896	−1.335501
N	7.762810	−9.631979	−1.452097
C	7.104358	−10.249559	−2.452895
H	6.235320	−12.650626	−4.463133
H	4.839125	−6.594435	−3.659417
H	4.498092	−8.052221	−4.596565
H	7.883926	−7.762329	−0.549535
P	8.730928	−16.147677	−0.106098
O	7.408610	−16.817694	−0.391058
O	9.896283	−16.919601	0.472045
O	8.377882	−14.858482	0.858552
C	9.477223	−14.009526	1.278571
C	8.947614	−12.872983	2.130110
O	8.162269	−11.970042	1.295089
C	6.989547	−11.567589	2.039349
C	8.041245	−13.298934	3.314706
C	6.650917	−12.774974	2.922877
O	8.564502	−12.652960	4.505895
H	9.988286	−13.603960	0.397057
H	10.196585	−14.594623	1.864231
H	9.809315	−12.323163	2.533050
H	7.206002	−10.681145	2.640043
H	8.043235	−14.383879	3.452318
H	6.122028	−13.541885	2.350327
H	6.045826	−12.501595	3.790811
H	8.045611	−12.974495	5.265851
N	5.956731	−11.160484	1.085033
C	5.594774	−9.805584	1.018110
O	6.072997	−8.936091	1.754995
N	4.647175	−9.514410	0.060490
C	4.029740	−10.407831	−0.808341
O	3.161821	−10.002556	−1.611384
C	4.463887	−11.791245	−0.704886
C	3.848163	−12.822707	−1.609254
C	5.401640	−12.090841	0.228907
H	4.350122	−8.502204	0.015913
H	2.768831	−12.901716	−1.429605
H	4.298080	−13.805024	−1.443686
H	3.978597	−12.545839	−2.660985
H	5.777716	−13.098853	0.351687

d(ATC)$^{4-}$

E: −27675.75 kcal mol^{-1}

O	−0.258557	−0.372855	0.038885
C	−0.140466	−0.066237	1.449887
C	1.318371	−0.125203	1.885772
O	1.814425	−1.505001	1.935918
C	2.797381	−1.738445	0.900834
C	2.300344	0.641848	0.980671
C	2.870451	−0.448957	0.068099
O	3.283594	1.268166	1.853192
H	−1.205733	−0.458186	−0.167413
H	−0.717976	−0.782070	2.048331
H	−0.516787	0.946700	1.653718
H	1.372959	0.267964	2.906241
H	3.752650	−1.991081	1.371717
H	1.803508	1.426177	0.404253
H	2.236350	−0.523956	−0.815742
H	3.883187	−0.224709	−0.256628
N	2.425018	−2.916602	0.112110
C	1.416775	−3.006753	−0.842065
N	1.316963	−4.213559	−1.375427
C	2.300109	−4.967042	−0.736789
C	2.682074	−6.324618	−0.856261
N	2.105236	−7.189091	−1.708117
N	3.681671	−6.765281	−0.040945
C	4.259990	−5.911483	0.825093
N	3.981506	−4.610516	1.012391
C	2.990459	−4.186250	0.202868
H	0.779768	−2.161293	−1.061473
H	2.439172	−8.158623	−1.766758
H	1.396368	−6.860634	−2.350205
H	5.054820	−6.338186	1.433378
P	4.690383	1.916505	1.277504
O	4.586763	2.236118	−0.193640
O	5.077102	2.975573	2.285743
O	5.723952	0.636015	1.386618
C	6.004742	0.120722	2.715071
C	7.132657	−0.890504	2.651113
O	6.676753	−2.100032	1.968322
C	7.583421	−2.431858	0.891888
C	8.401666	−0.407309	1.909979
C	8.329751	−1.133416	0.562485
O	9.538399	−0.785196	2.737276
H	5.103981	−0.355273	3.121248
H	6.298206	0.944387	3.376596
H	7.401937	−1.158235	3.679860
H	8.265959	−3.224894	1.206266
H	8.412908	0.677573	1.781850
H	7.756262	−0.512020	−0.128656
H	9.314611	−1.302791	0.133175
N	6.806076	−3.004073	−0.215647
C	6.900449	−4.387557	−0.457420
O	7.596586	−5.147117	0.219246
N	6.160333	−4.830383	−1.533747
C	5.327712	−4.073447	−2.346092
O	4.706667	−4.611690	−3.287336
C	5.238433	−2.659038	−2.017939
C	4.360702	−1.775583	−2.860351
C	5.967889	−2.204162	−0.966852
H	6.244129	−5.865242	−1.752494
H	4.662066	−1.828043	−3.913418
H	4.421549	−0.734465	−2.535207
H	3.314568	−2.099970	−2.813020
H	5.925102	−1.168339	−0.650200
P	11.105932	−0.697034	2.222184
O	11.228675	0.126032	0.963165
O	11.916017	−0.366836	3.456151
O	11.394990	−2.257981	1.770972
C	11.339480	−3.277182	2.805710
C	11.866349	−4.598580	2.272746
O	10.941962	−5.145751	1.282940
C	11.617151	−5.348921	0.021077
C	13.249389	−4.529508	1.593453
C	12.917643	−4.531494	0.096342
O	13.983102	−5.716932	1.997482
H	10.301185	−3.403427	3.135341
H	11.948381	−2.965710	3.662530
H	11.913124	−5.298607	3.116485
H	11.806590	−6.412023	−0.128972
H	13.801124	−3.632024	1.891093
H	12.753442	−3.504477	−0.239203

H	13.713352	−4.972687	−0.509192
H	14.853656	−5.687197	1.560120
N	10.711302	−4.949741	−1.067439
C	10.206215	−5.924007	−1.971488
O	10.541595	−7.123288	−1.836086
N	9.380805	−5.509724	−2.968387
C	9.019155	−4.216495	−3.082501
N	8.194387	−3.878977	−4.085440
C	9.482904	−3.228913	−2.152993
C	10.315278	−3.643156	−1.164745
H	7.835359	−4.596729	−4.735291
H	7.904048	−2.916466	−4.195027
H	9.170833	−2.194541	−2.229779
H	10.692682	−2.980602	−0.393424
O	5.860371	−11.680733	−10.759645
C	6.981410	−12.589235	−10.884142
C	7.452625	−13.050501	−9.510509
O	8.049810	−11.943397	−8.758078
C	7.239464	−11.634249	−7.593361
C	6.362082	−13.635346	−8.588820
C	5.936634	−12.421388	−7.759598
O	6.995190	−14.712238	−7.829423
H	5.688512	−11.304131	−11.640323
H	7.819382	−12.094321	−11.391951
H	6.695629	−13.477969	−11.465927
H	8.236830	−13.798906	−9.668171
H	7.784361	−11.930490	−6.693955
H	5.527785	−14.065173	−9.149600
H	5.217796	−11.848591	−8.348582
H	5.477184	−12.688101	−6.809925
N	7.057975	−10.195204	−7.476512
C	6.236764	−9.354623	−8.228054
N	6.279361	−8.101428	−7.812401
C	7.168529	−8.106116	−6.734159
C	7.602695	−7.059691	−5.869992
O	7.268105	−5.849327	−5.903853
N	8.504193	−7.521248	−4.890575
C	8.957309	−8.821879	−4.782851
N	9.876475	−9.084557	−3.822316
N	8.555072	−9.802409	−5.594342
C	7.669265	−9.397805	−6.521676
H	5.670261	−9.729308	−9.069458
H	8.827477	−6.811658	−4.191076
H	10.052531	−8.395966	−3.076413
H	9.998393	−10.067201	−3.599138
P	6.575409	−15.183947	−6.306614
O	5.111921	−14.938207	−6.028660
O	7.185151	−16.557986	−6.147645
O	7.375935	−14.098290	−5.359609
C	8.811853	−14.187167	−5.174456
C	9.173085	−14.036018	−3.699771
O	9.031611	−12.657972	−3.229103
C	7.857215	−12.520107	−2.397116
C	8.309548	−14.887035	−2.753212
C	7.210428	−13.914144	−2.302742
O	9.178993	−15.338507	−1.674970
H	9.293148	−13.391013	−5.753624
H	9.171601	−15.160053	−5.526083
H	10.230645	−14.297251	−3.587591
H	8.167996	−12.138977	−1.420622
H	7.890616	−15.763630	−3.253362
H	6.366286	−14.003711	−2.986404
H	6.866505	−14.128116	−1.292979
N	6.965957	−11.493188	−2.945034
C	6.091246	−11.596564	−4.022413
N	5.470352	−10.460993	−4.293390
C	5.964480	−9.551630	−3.360049
C	5.717244	−8.174690	−3.145639
N	4.860988	−7.444792	−3.877793

N	6.405556	−7.569254	−2.137667
C	7.276793	−8.278986	−1.397578
N	7.582412	−9.580941	−1.514854
C	6.900819	−10.171023	−2.516517
H	5.982568	−12.523893	−4.564407
H	4.769663	−6.436601	−3.705301
H	4.373825	−7.874087	−4.652398
H	7.784661	−7.716502	−0.617743
P	8.575564	−16.114390	−0.347221
O	7.259222	−16.783262	−0.661031
O	9.742138	−16.898076	0.212820
O	8.209935	−14.859316	0.657122
C	9.304398	−14.022143	1.112735
C	8.768029	−12.917533	2.001385
O	7.987187	−11.984976	1.194646
C	6.798599	−11.624537	1.936549
C	7.853637	−13.387305	3.162539
C	6.462087	−12.868144	2.768720
O	8.354433	−12.769165	4.377886
H	9.818209	−13.584231	0.248391
H	10.022544	−14.625788	1.680805
H	9.626556	−12.382186	2.429536
H	6.997780	−10.760327	2.574411
H	7.866920	−14.475687	3.269521
H	5.953967	−13.621906	2.161409
H	5.840036	−12.635016	3.636500
H	7.827340	−13.115006	5.121298
N	5.775518	−11.189105	0.984218
C	5.434852	−9.827125	0.936824
O	5.929155	−8.975647	1.683648
N	4.488204	−9.507781	−0.013105
C	3.853816	−10.379622	−0.892028
O	2.985870	−9.950957	−1.682566
C	4.273445	−11.769342	−0.815393
C	3.647069	−12.778195	−1.737856
C	5.210653	−12.096194	0.109653
H	4.210766	−8.489743	−0.046194
H	2.567042	−12.849351	−1.559461
H	4.087264	−13.767767	−1.589723
H	3.780023	−12.484372	−2.784639
H	5.576690	−13.110342	0.209940

d(ATG)$^{4-}$

E: −27673.11 kcal mol^{-1}

O	−0.260850	−0.539350	−0.048211
C	−0.174955	−0.200717	1.357477
C	1.279082	−0.188053	1.813732
O	1.834050	−1.543093	1.899757
C	2.831512	−1.760364	0.873758
C	2.243143	0.602093	0.909123
C	2.871394	−0.479888	0.023379
O	3.185481	1.281377	1.787712
H	−1.199176	−0.684876	−0.261140
H	−0.728742	−0.929409	1.962941
H	−0.598257	0.798374	1.536875
H	1.301231	0.227365	2.826627
H	3.790480	−1.975583	1.355172
H	1.725035	1.356783	0.312161
H	2.259688	−0.589430	−0.872478
H	3.882455	−0.222735	−0.283070
N	2.503805	−2.962544	0.100551
C	1.529947	−3.097438	−0.883518
N	1.472391	−4.318474	−1.390143
C	2.449759	−5.035583	−0.701963
C	2.865070	−6.386746	−0.775564
N	2.335120	−7.285442	−1.623021

N	3.847046	−6.785533	0.081896
C	4.377231	−5.898810	0.945725
N	4.062756	−4.600989	1.092904
C	3.091753	−4.217687	0.240433
H	0.882750	−2.272308	−1.144880
H	2.692567	−8.247968	−1.644843
H	1.649534	−6.986654	−2.303656
H	5.162112	−6.292294	1.588038
P	4.572195	1.985818	1.229298
O	4.469797	2.311820	−0.240757
O	4.906101	3.053007	2.248340
O	5.654108	0.746149	1.340118
C	5.908074	0.204499	2.663104
C	6.991346	−0.854170	2.596298
O	6.499764	−2.025404	1.873484
C	7.461380	−2.401537	0.855590
C	8.304146	−0.416263	1.901127
C	8.236035	−1.119344	0.542254
O	9.391170	−0.862064	2.763619
H	4.986428	−0.236637	3.061346
H	6.233355	1.007129	3.336016
H	7.223670	−1.154569	3.625420
H	8.117541	−3.190755	1.229043
H	8.372882	0.669033	1.794576
H	7.671887	−0.479496	−0.140821
H	9.216995	−1.302979	0.109403
N	6.743149	−2.992944	−0.274668
C	6.872228	−4.375568	−0.501773
O	7.556694	−5.117123	0.204678
N	6.164369	−4.841278	−1.590418
C	5.332826	−4.109866	−2.426254
O	4.727250	−4.672659	−3.364112
C	5.216381	−2.692650	−2.118566
C	4.334779	−1.831579	−2.980602
C	5.918486	−2.214351	−1.059565
H	6.270669	−5.875402	−1.788881
H	4.659526	−1.873106	−4.027273
H	4.360989	−0.789518	−2.652255
H	3.296650	−2.183307	−2.954849
H	5.857190	−1.174794	−0.759531
P	10.969871	−0.949954	2.290276
O	11.222584	−0.133040	1.046358
O	11.774173	−0.721473	3.551093
O	11.097852	−2.524880	1.824149
C	10.983223	−3.561530	2.835332
C	11.716151	−4.822144	2.395657
O	10.993378	−5.518931	1.334933
C	11.720325	−5.441299	0.085561
C	13.133359	−4.582383	1.850354
C	12.917335	−4.503978	0.332937
O	13.940646	−5.728521	2.233049
H	9.923441	−3.791768	2.998095
H	11.421256	−3.206056	3.775108
H	11.754554	−5.498378	3.257904
H	12.034775	−6.449613	−0.198414
H	13.573060	−3.663362	2.251615
H	12.666242	−3.478012	0.056920
H	13.799489	−4.815057	−0.231939
H	14.827694	−5.604711	1.848686
N	10.823983	−4.997823	−0.975922
C	10.278185	−3.727394	−1.154646
N	9.460170	−3.665988	−2.187926
C	9.451299	−4.957536	−2.720497
C	8.763880	−5.504224	−3.843429
O	7.968984	−4.918367	−4.617699
N	9.072357	−6.865437	−4.040450
C	9.925447	−7.609386	−3.252350
N	10.143243	−8.892897	−3.620340
N	10.552800	−7.105501	−2.183201

C	10.289105	−5.799446	−1.973579
H	10.517320	−2.922226	−0.475185
H	8.576702	−7.342963	−4.830295
H	9.556187	−9.346022	−4.340502
H	10.582482	−9.487267	−2.927800
O	5.861042	−11.767274	−10.732305
C	7.014291	−12.634123	−10.871890
C	7.534521	−13.055558	−9.503438
O	8.125988	−11.915532	−8.798206
C	7.386358	−11.642643	−7.577921
C	6.483965	−13.646289	−8.537720
C	6.082388	−12.436714	−7.689562
O	7.157208	−14.711731	−7.798376
H	5.672382	−11.384188	−11.606785
H	7.819457	−12.114678	−11.406855
H	6.747513	−13.540698	−11.434438
H	8.329555	−13.791342	−9.667691
H	7.979263	−11.952031	−6.718064
H	5.631700	−14.083816	−9.064482
H	5.326102	−11.873551	−8.240482
H	5.669512	−12.712106	−6.721429
N	7.211760	−10.193231	−7.426830
C	7.911320	−9.513906	−6.391996
O	8.719302	−10.152307	−5.681938
N	7.669762	−8.189145	−6.213057
C	6.835562	−7.516442	−7.031179
N	6.626717	−6.214915	−6.781874
C	6.200358	−8.164496	−8.142805
C	6.417250	−9.495954	−8.300017
H	7.076619	−5.754511	−5.975084
H	6.001462	−5.683397	−7.373391
H	5.573482	−7.613086	−8.834097
H	6.001033	−10.069651	−9.121767
P	6.699341	−15.258068	−6.310397
O	5.229729	−15.021805	−6.059215
O	7.302245	−16.640250	−6.202169
O	7.482143	−14.223771	−5.294789
C	8.922571	−14.299706	−5.137411
C	9.316174	−14.098004	−3.677506
O	9.201666	−12.702998	−3.254549
C	8.056268	−12.519333	−2.393016
C	8.468093	−14.904263	−2.679941
C	7.392838	−13.901345	−2.241855
O	9.361462	−15.328029	−1.610046
H	9.390714	−13.524804	−5.755144
H	9.276333	−15.284328	−5.462180
H	10.373227	−14.367355	−3.580438
H	8.399175	−12.113046	−1.437254
H	8.026208	−15.793513	−3.136520
H	6.538216	−13.998840	−2.911125
H	7.058536	−14.080988	−1.222940
N	7.163267	−11.497680	−2.948462
C	6.306428	−11.619622	−4.036187
N	5.676713	−10.493447	−4.327630
C	6.144994	−9.571945	−3.393422
C	5.862881	−8.201030	−3.186147
N	5.013204	−7.488902	−3.943353
N	6.498644	−7.585881	−2.150193
C	7.364635	−8.278644	−1.387859
N	7.713685	−9.569688	−1.507088
C	7.074627	−10.172583	−2.529170
H	6.220886	−12.552943	−4.572054
H	4.888630	−6.483065	−3.771323
H	4.575142	−7.924037	−4.743271
H	7.825062	−7.709875	−0.583075
P	8.794432	−15.991979	−0.208651
O	7.435083	−16.616564	−0.411105
O	9.953458	−16.793799	0.341904
O	8.537814	−14.661195	0.729994

C	9.689553	−13.845424	1.068411
C	9.263896	−12.715623	1.986053
O	8.424774	−11.778941	1.244174
C	7.264774	−11.453314	2.045633
C	8.458515	−13.151079	3.234804
C	7.027713	−12.686133	2.927096
O	9.032062	−12.458429	4.376312
H	10.129691	−13.432404	0.152704
H	10.443529	−14.458971	1.576275
H	10.169915	−12.190321	2.316742
H	7.459907	−10.563508	2.647885
H	8.513002	−14.232309	3.392251
H	6.504755	−13.470944	2.374192
H	6.458830	−12.449608	3.829756
H	8.555060	−12.758359	5.171793
N	6.169596	−11.089377	1.143188
C	5.763153	−9.746832	1.084831
O	6.239745	−8.859778	1.801767
N	4.773194	−9.488150	0.161083
C	4.165046	−10.401091	−0.692721
O	3.272573	−10.023036	−1.482839
C	4.637927	−11.771874	−0.593499
C	4.020971	−12.821113	−1.476120
C	5.616941	−12.039499	0.307341
H	4.452339	−8.483431	0.112482
H	2.943351	−12.899842	−1.287909
H	4.474947	−13.798897	−1.297726
H	4.142657	−12.562313	−2.534048
H	6.032702	−13.033674	0.416880

d(CAA)$^{4-}$

E: −27671.60 kcal mol^{-1}

O	−0.243575	−0.145914	−0.674626
C	−0.197374	0.278471	0.710587
C	1.194892	0.059264	1.289216
O	1.474083	−1.369968	1.455025
C	2.595151	−1.766988	0.621012
C	2.361561	0.620047	0.450247
C	2.837572	−0.600211	−0.342276
O	3.344708	1.129119	1.400946
H	−1.176997	−0.154943	−0.949663
H	−0.919831	−0.290887	1.309053
H	−0.440470	1.348006	0.792145
H	1.211394	0.516168	2.284944
H	3.462379	−1.967838	1.250551
H	2.051202	1.440666	−0.201508
H	2.217294	−0.693917	−1.236048
H	3.876330	−0.520646	−0.653059
N	2.282108	−3.046416	−0.032166
C	2.980649	−4.223556	0.356373
O	3.819771	−4.158913	1.281864
N	2.708336	−5.381874	−0.301051
C	1.768001	−5.431614	−1.265703
N	1.564740	−6.603171	−1.887662
C	0.992990	−4.274174	−1.608914
C	1.281552	−3.112814	−0.967071
H	2.142067	−7.428751	−1.663136
H	0.877141	−6.665316	−2.626953
H	0.201266	−4.323838	−2.347741
H	0.731639	−2.192979	−1.137877
P	4.889189	1.533499	0.977416
O	5.007817	1.752920	−0.511396
O	5.302313	2.603258	1.963425
O	5.715836	0.140165	1.287577
C	5.899169	−0.276658	2.667171
C	7.156875	−1.124749	2.820757
O	6.976861	−2.476603	2.292433
C	7.750605	−2.660466	1.083918
C	8.403792	−0.564253	2.113333
C	8.395869	−1.300751	0.769776
O	9.547739	−0.873575	2.963466
H	5.026170	−0.857087	2.989375
H	5.994968	0.609458	3.304873
H	7.349085	−1.228700	3.894203
H	8.490741	−3.447432	1.257981
H	8.353591	0.519762	1.981798
H	7.777835	−0.730371	0.075513
H	9.390120	−1.397502	0.341677
N	6.894754	−3.160676	0.011013
C	5.988136	−2.439081	−0.753209
N	5.355528	−3.183726	−1.645807
C	5.864415	−4.468067	−1.459570
C	5.603722	−5.696934	−2.109634
N	4.722103	−5.836971	−3.111383
N	6.307605	−6.785427	−1.688012
C	7.193523	−6.659487	−0.681814
N	7.506894	−5.549717	0.005489
C	6.815699	−4.478117	−0.427851
H	5.834050	−1.383510	−0.584552
H	4.581492	−6.753295	−3.557891
H	4.193382	−5.034135	−3.422784
H	7.712273	−7.575467	−0.407984
P	11.108389	−0.831770	2.425071
O	11.250069	0.054470	1.211104
O	11.943202	−0.598717	3.664973
O	11.329983	−2.372263	1.880197
C	11.300237	−3.450245	2.852617
C	11.901928	−4.713309	2.257419
O	11.021639	−5.266348	1.228587
C	11.700373	−5.301738	−0.048279
C	13.279037	−4.525143	1.593359
C	12.940773	−4.406650	0.102809
O	14.062844	−5.713247	1.886704
H	10.262930	−3.642311	3.152980
H	11.877999	−3.163204	3.739014
H	11.980535	−5.453490	3.063330
H	11.964183	−6.336175	−0.290088
H	13.793026	−3.635394	1.970996
H	12.694317	−3.367608	−0.126896
H	13.760807	−4.727677	−0.544059
H	14.926422	−5.611809	1.445946
N	10.777662	−4.873971	−1.092688
C	10.300655	−3.589941	−1.320836
N	9.429179	−3.530379	−2.313950
C	9.315191	−4.845090	−2.765068
C	8.543528	−5.440743	−3.790779
N	7.710330	−4.758524	−4.598256
N	8.669185	−6.787031	−3.964476
C	9.488911	−7.492670	−3.164845
N	10.260836	−7.030461	−2.166587
C	10.139622	−5.696981	−2.012097
H	10.631652	−2.756784	−0.717766
H	7.120854	−5.267755	−5.267524
H	7.528994	−3.780698	−4.413754
H	9.512311	−8.563566	−3.354390
O	6.162368	−11.520037	−10.792611
C	7.400521	−12.262665	−10.923551
C	7.840570	−12.798680	−9.567497
O	8.320401	−11.714411	−8.704880
C	7.490830	−11.591579	−7.524518
C	6.746894	−13.532634	−8.764513
C	6.260529	−12.474668	−7.770409
O	7.381010	−14.682774	−8.134541
H	5.997245	−11.068135	−11.638474
H	8.193510	−11.618574	−11.323934

H	7.264310	−13.116030	−11.603392
H	8.683400	−13.477276	−9.738097
H	8.056179	−11.904920	−6.645183
H	5.936177	−13.891118	−9.403392
H	5.453860	−11.904750	−8.234202
H	5.883987	−12.918469	−6.852259
N	7.175648	−10.168925	−7.298077
C	7.747088	−9.513372	−6.195154
O	8.529253	−10.057237	−5.409551
N	7.348387	−8.200440	−6.043853
C	6.516460	−7.472490	−6.886547
O	6.256656	−6.275857	−6.628463
C	6.019295	−8.186440	−8.051046
C	5.149552	−7.462296	−9.042599
C	6.383197	−9.486107	−8.203320
H	7.791170	−7.684723	−5.236122
H	5.673372	−6.595032	−9.463616
H	4.239074	−7.084617	−8.560898
H	4.860040	−8.126482	−9.862046
H	6.086550	−10.065520	−9.072278
P	6.596922	−15.603019	−7.004961
O	5.102544	−15.410824	−7.089655
O	7.220694	−16.976830	−7.115794
O	7.061622	−14.890268	−5.592165
C	8.469237	−14.957257	−5.241286
C	8.689670	−14.441899	−3.830752
O	8.464222	−12.999352	−3.782048
C	7.508292	−12.684846	−2.740505
C	7.784850	−15.069772	−2.744598
C	6.736894	−13.983723	−2.486945
O	8.649049	−15.357706	−1.606125
H	9.051090	−14.351345	−5.946762
H	8.814447	−15.996350	−5.300554
H	9.738013	−14.633883	−3.571502
H	8.030258	−12.342166	−1.844430
H	7.328505	−16.005390	−3.076918
H	5.932063	−14.112767	−3.214009
H	6.307202	−14.039143	−1.489497
N	6.690095	−11.547508	−3.173221
C	6.906650	−10.295217	−2.568415
O	7.761850	−10.100184	−1.703147
N	6.069614	−9.292689	−3.016762
C	5.095206	−9.394759	−4.000672
O	4.415544	−8.398246	−4.326896
C	4.953408	−10.703048	−4.618267
C	3.917429	−10.884744	−5.693270
C	5.763856	−11.701642	−4.182560
H	6.182457	−8.359474	−2.535299
H	2.926028	−10.599018	−5.322335
H	3.874487	−11.924109	−6.027157
H	4.129320	−10.245263	−6.558634
H	5.731392	−12.692245	−4.621083
P	8.090888	−15.718267	−0.095014
O	6.634043	−16.112217	−0.121984
O	9.136631	−16.631483	0.506471
O	8.141948	−14.248124	0.647825
C	9.437208	−13.643178	0.902636
C	9.344057	−12.638678	2.043056
O	8.638292	−11.427654	1.629932
C	7.372321	−11.312893	2.321892
C	8.609431	−13.149759	3.293781
C	7.179721	−12.628083	3.100655
O	9.262303	−12.555436	4.448250
H	9.785328	−13.135575	−0.005195
H	10.157868	−14.422954	1.174793
H	10.367148	−12.343475	2.305080
H	7.410703	−10.441781	2.982038
H	8.642653	−14.241886	3.366517
H	6.611202	−13.346599	2.506941
H	6.660846	−12.465857	4.048653
H	8.775127	−12.843459	5.241837
N	6.312683	−11.031543	1.359706
C	5.781444	−11.900552	0.407487
N	4.857117	−11.331234	−0.341605
C	4.770549	−10.017915	0.125448
C	3.939371	−8.933050	−0.281000
O	3.082907	−8.923150	−1.197478
N	4.165365	−7.777016	0.491787
C	5.085175	−7.671883	1.513328
N	5.140852	−6.489819	2.171930
N	5.878392	−8.682613	1.887152
C	5.675638	−9.812723	1.177952
H	6.140745	−12.915994	0.324356
H	3.628121	−6.920976	0.217320
H	4.686361	−5.647436	1.781821
H	5.953804	−6.350159	2.759997

d(CAC)$^{4–}$

E: −27583.66 kcal mol^{-1}

O	−0.234615	−0.122691	−0.701535
C	−0.195083	0.282748	0.689274
C	1.191740	0.046633	1.273981
O	1.464391	−1.386897	1.410839
C	2.593487	−1.768585	0.580616
C	2.367859	0.621680	0.457444
C	2.847370	−0.583095	−0.356248
O	3.341325	1.110196	1.428831
H	−1.165888	−0.119343	−0.983873
H	−0.925130	−0.289693	1.275324
H	−0.431584	1.352522	0.783809
H	1.201873	0.482071	2.279328
H	3.453605	−1.983208	1.215323
H	2.065240	1.456438	−0.179920
H	2.232912	−0.655938	−1.255891
H	3.888263	−0.499979	−0.659535
N	2.286487	−3.034279	−0.101461
C	2.977229	−4.220095	0.274301
O	3.800949	−4.175548	1.214775
N	2.714438	−5.364696	−0.410234
C	1.788056	−5.394046	−1.389413
N	1.591508	−6.552756	−2.036692
C	1.021821	−4.228062	−1.723251
C	1.301474	−3.080253	−1.053917
H	2.137431	−7.394687	−1.796008
H	0.904413	−6.603220	−2.777318
H	0.241553	−4.261811	−2.475061
H	0.754302	−2.156967	−1.214475
P	4.900403	1.496440	1.046001
O	5.060951	1.727142	−0.437068
O	5.305158	2.550270	2.052087
O	5.698299	0.087721	1.363371
C	5.835096	−0.350643	2.741741
C	7.096052	−1.187715	2.926178
O	6.951147	−2.535897	2.376246
C	7.757292	−2.695999	1.186742
C	8.355478	−0.600772	2.266121
C	8.414085	−1.328823	0.918334
O	9.475970	−0.897395	3.149723
H	4.956340	−0.943777	3.021925
H	5.899735	0.525444	3.396846
H	7.255248	−1.303724	4.003627
H	8.491816	−3.487795	1.361776
H	8.289628	0.482447	2.137578
H	7.834942	−0.756506	0.192640
H	9.431985	−1.423539	0.549138

N	6.926522	−3.171740	0.080514
C	6.010536	−2.433051	−0.657495
N	5.397970	−3.148495	−1.586799
C	5.928107	−4.430200	−1.454110
C	5.684114	−5.634644	−2.155371
N	4.803638	−5.742733	−3.162135
N	6.397778	−6.732621	−1.776948
C	7.286457	−6.634628	−0.770081
N	7.587745	−5.549741	−0.038698
C	6.876722	−4.470352	−0.420466
H	5.834109	−1.390356	−0.437241
H	4.641831	−6.652311	−3.614346
H	4.243345	−4.942032	−3.419208
H	7.814901	−7.555275	−0.531767
P	11.045764	−0.668508	2.687425
O	11.142043	0.333744	1.562639
O	11.811934	−0.475687	3.977380
O	11.431039	−2.127524	2.020951
C	11.434490	−3.285392	2.897778
C	11.952185	−4.500855	2.150787
O	10.982824	−4.899936	1.133962
C	11.654716	−5.060131	−0.137916
C	13.304357	−4.302472	1.427913
C	12.910982	−4.182382	−0.050064
O	14.107262	−5.485182	1.691087
H	10.414847	−3.478763	3.252721
H	12.079092	−3.091977	3.763537
H	12.053561	−5.319959	2.874856
H	11.903624	−6.109656	−0.301566
H	13.827727	−3.411293	1.787685
H	12.676857	−3.138762	−0.277040
H	13.700962	−4.521043	−0.725153
H	14.965065	−5.362772	1.244680
N	10.714137	−4.701437	−1.204705
C	10.199548	−5.708124	−2.066208
O	10.572907	−6.894306	−1.915541
N	9.321302	−5.340732	−3.034547
C	8.896241	−4.066142	−3.144235
N	8.022959	−3.777541	−4.120248
C	9.358740	−3.047631	−2.246197
C	10.260722	−3.413480	−1.301990
H	7.666033	−4.518804	−4.742599
H	7.640689	−2.843939	−4.193216
H	8.999355	−2.027959	−2.314362
H	10.655386	−2.722042	−0.568052
O	6.301542	−11.407949	−10.841371
C	7.472006	−12.258086	−10.914357
C	7.769590	−12.872328	−9.552072
O	8.267778	−11.868605	−8.606771
C	7.301012	−11.631968	−7.552795
C	6.567119	−13.539750	−8.856877
C	6.051922	−12.445123	−7.918645
O	7.075548	−14.725058	−8.177522
H	6.234417	−10.924818	−11.683352
H	8.346531	−11.680117	−11.239197
H	7.307787	−13.077227	−11.630003
H	8.570164	−13.606764	−9.690753
H	7.739335	−11.943717	−6.599904
H	5.800306	−13.856188	−9.568941
H	5.338132	−11.830797	−8.468805
H	5.551094	−12.853465	−7.044771
N	7.049868	−10.198471	−7.414734
C	6.280625	−9.374708	−8.239836
N	6.277563	−8.116610	−7.839978
C	7.088497	−8.093512	−6.703238
C	7.468454	−7.018412	−5.846519
O	7.118125	−5.814640	−5.922298
N	8.342012	−7.439163	−4.827060
C	8.813037	−8.727873	−4.670043
N	9.715928	−8.937607	−3.688536
N	8.435790	−9.743754	−5.454027
C	7.590114	−9.375563	−6.434517
H	5.792988	−9.765128	−9.121649
H	8.672264	−6.699910	−4.164169
H	9.940165	−8.199509	−3.005145
H	9.888394	−9.901945	−3.432800
P	6.247053	−15.509325	−6.982541
O	4.768780	−15.211247	−7.044943
O	6.761208	−16.931539	−7.020567
O	6.800505	−14.749703	−5.626896
C	8.212631	−14.884604	−5.313598
C	8.483912	−14.446504	−3.885353
O	8.321602	−13.000267	−3.755940
C	7.338998	−12.699404	−2.734445
C	7.571988	−15.089346	−2.816376
C	6.544104	−13.992063	−2.526180
O	8.432507	−15.424774	−1.688307
H	8.799552	−14.268135	−6.005469
H	8.516904	−15.931659	−5.428570
H	9.528538	−14.692059	−3.659384
H	7.841300	−12.379829	−1.819274
H	7.098681	−16.006891	−3.174915
H	5.738024	−14.083341	−3.257204
H	6.113194	−14.070615	−1.530925
N	6.547769	−11.541335	−3.166529
C	6.768838	−10.307211	−2.528030
O	7.579986	−10.152812	−1.614588
N	5.997184	−9.268729	−3.012985
C	5.055848	−9.330632	−4.032634
O	4.434712	−8.303634	−4.381213
C	4.878186	−10.633269	−4.651817
C	3.858697	−10.777624	−5.747503
C	5.647658	−11.659292	−4.205421
H	6.152009	−8.333143	−2.548593
H	2.873198	−10.447253	−5.398132
H	3.776708	−11.816758	−6.074204
H	4.115795	−10.155085	−6.612462
H	5.602237	−12.643623	−4.657885
P	7.872086	−15.807287	−0.183293
O	6.410156	−16.181668	−0.214543
O	8.907132	−16.746601	0.395931
O	7.944254	−14.353370	0.588449
C	9.248608	−13.766414	0.838670
C	9.169271	−12.752538	1.971230
O	8.470206	−11.541035	1.549847
C	7.214804	−11.403909	2.257537
C	8.438044	−13.247465	3.230616
C	7.012604	−12.712778	3.043603
O	9.104771	−12.651336	4.376347
H	9.604387	−13.270362	−0.072638
H	9.958065	−14.554815	1.115853
H	10.196179	−12.463698	2.225890
H	7.275932	−10.531534	2.914478
H	8.461122	−14.339313	3.311334
H	6.434416	−13.429221	2.456963
H	6.500557	−12.540832	3.993623
H	8.619512	−12.927952	5.175172
N	6.147538	−11.108778	1.308962
C	5.570835	−11.978245	0.384277
N	4.658712	−11.391402	−0.366280
C	4.628431	−10.065237	0.070899
C	3.847666	−8.954695	−0.363932
O	2.990566	−8.928662	−1.280339
N	4.132071	−7.788773	0.373555
C	5.059716	−7.698689	1.390254
N	5.166052	−6.507674	2.021528
N	5.814861	−8.732195	1.781841
C	5.556429	−9.870530	1.105469

H	5.888675	−13.008697	0.319133
H	3.617835	−6.920879	0.092866
H	4.701974	−5.661607	1.652530
H	5.977787	−6.384378	2.614114

d(CAG)$^{4-}$

E: −27581.40 kcal mol^{-1}

O	−0.238905	−0.147920	−0.646862
C	−0.190146	0.259385	0.743243
C	1.204140	0.037047	1.315655
O	1.490190	−1.393470	1.455346
C	2.614403	−1.769420	0.615703
C	2.368392	0.619242	0.486867
C	2.852448	−0.584458	−0.326048
O	3.345057	1.121065	1.448161
H	−1.173085	−0.156793	−0.919397
H	−0.909167	−0.319614	1.336542
H	−0.436332	1.326889	0.838920
H	1.219462	0.476165	2.319380
H	3.481599	−1.977401	1.242895
H	2.052727	1.448600	−0.151361
H	2.232858	−0.665183	−1.221660
H	3.891095	−0.494838	−0.635365
N	2.309939	−3.038265	−0.061335
C	3.013303	−4.218327	0.310132
O	3.845164	−4.165268	1.242567
N	2.752053	−5.366033	−0.369598
C	1.815114	−5.404820	−1.337975
N	1.621801	−6.566265	−1.981396
C	1.033257	−4.246493	−1.662934
C	1.312515	−3.094597	−1.000302
H	2.190579	−7.397510	−1.756289
H	0.931057	−6.622686	−2.718182
H	0.243518	−4.288468	−2.404391
H	0.756568	−2.175753	−1.156266
P	4.899871	1.513885	1.053289
O	5.050108	1.742390	−0.431265
O	5.304637	2.573301	2.053892
O	5.707225	0.110537	1.370085
C	5.822067	−0.334307	2.748150
C	7.062803	−1.197575	2.944929
O	6.903671	−2.534281	2.374331
C	7.743240	−2.698753	1.207030
C	8.348934	−0.627950	2.322076
C	8.427205	−1.341077	0.967266
O	9.439343	−0.961268	3.230752
H	4.928573	−0.909930	3.018461
H	5.897323	0.538303	3.407081
H	7.196900	−1.328782	4.024337
H	8.461986	−3.501182	1.397860
H	8.309912	0.458166	2.207134
H	7.874638	−0.752413	0.233699
H	9.451907	−1.454632	0.621298
N	6.939149	−3.158154	0.076313
C	6.040552	−2.414477	−0.676339
N	5.430912	−3.129428	−1.608765
C	5.947129	−4.415889	−1.463235
C	5.697551	−5.624705	−2.154805
N	4.819231	−5.742507	−3.162194
N	6.402073	−6.723886	−1.760463
C	7.273255	−6.627770	−0.737974
N	7.569498	−5.541372	−0.008427
C	6.880210	−4.458255	−0.415681
H	5.872962	−1.368082	−0.466325
H	4.681580	−6.649850	−3.627292
H	4.292048	−4.933469	−3.459420
H	7.788519	−7.552073	−0.485151
P	11.023688	−0.761866	2.808015
O	11.169597	0.280903	1.725935
O	11.766360	−0.638041	4.120214
O	11.383125	−2.202083	2.092430
C	11.370823	−3.395138	2.919611
C	11.898996	−4.579594	2.129245
O	10.930859	−4.964507	1.103537
C	11.598797	−5.060829	−0.177403
C	13.241275	−4.328360	1.405115
C	12.826097	−4.146162	−0.059422
O	14.064371	−5.510033	1.601805
H	10.346599	−3.600761	3.254151
H	12.005663	−3.240332	3.800230
H	12.018427	−5.420060	2.825350
H	11.883569	−6.100347	−0.373523
H	13.755054	−3.447203	1.801064
H	12.538707	−3.104418	−0.224954
H	13.618197	−4.415263	−0.762604
H	14.915337	−5.352761	1.153099
N	10.665610	−4.695196	−1.228416
C	10.144567	−3.429408	−1.494191
N	9.303527	−3.427267	−2.509214
C	9.256111	−4.753648	−2.942689
C	8.532935	−5.362610	−4.009832
O	7.739620	−4.812333	−4.811730
N	8.798584	−6.741387	−4.109108
C	9.654654	−7.445421	−3.287623
N	9.839932	−8.753029	−3.571774
N	10.318953	−6.880960	−2.270884
C	10.092993	−5.557194	−2.153223
H	10.429005	−2.570038	−0.905866
H	8.285092	−7.262985	−4.858007
H	9.253168	−9.233733	−4.272217
H	10.265085	−9.316528	−2.845876
O	6.160110	−11.585870	−10.995459
C	7.374420	−12.372794	−11.085041
C	7.774966	−12.886145	−9.707961
O	8.268912	−11.794226	−8.864224
C	7.416632	−11.617017	−7.704293
C	6.647539	−13.568206	−8.905650
C	6.165071	−12.464597	−7.961412
O	7.243763	−14.709697	−8.222802
H	6.025615	−11.149897	−11.855078
H	8.195002	−11.765907	−11.487991
H	7.220971	−13.238707	−11.745203
H	8.601293	−13.591886	−9.847127
H	7.951685	−11.929967	−6.807027
H	5.843440	−13.932948	−9.549700
H	5.394229	−11.883768	−8.471067
H	5.742206	−12.865395	−7.043606
N	7.146234	−10.181097	−7.514669
C	7.710636	−9.496550	−6.401048
O	8.452734	−10.120899	−5.611512
N	7.410283	−8.182131	−6.232047
C	6.629583	−7.526044	−7.113179
N	6.353455	−6.236428	−6.870490
C	6.117739	−8.178894	−8.284077
C	6.409209	−9.494293	−8.446830
H	6.790440	−5.744644	−6.076040
H	5.805801	−5.707762	−7.536960
H	5.532590	−7.639973	−9.020233
H	6.100087	−10.062968	−9.317973
P	6.455783	−15.537032	−7.027606
O	4.966278	−15.301926	−7.089168
O	7.030977	−16.935600	−7.074070
O	6.976215	−14.763304	−5.667461
C	8.387672	−14.862438	−5.338187
C	8.638034	−14.404583	−3.911846

O	8.462324	−12.959411	−3.801526
C	7.480679	−12.652488	−2.781573
C	7.723732	−15.042668	−2.841605
C	6.690199	−13.947026	−2.565461
O	8.582888	−15.365038	−1.708111
H	8.967666	−14.238860	−6.029741
H	8.715980	−15.903494	−5.440703
H	9.682592	−14.638689	−3.672908
H	7.982496	−12.325454	−1.868664
H	7.256083	−15.965596	−3.193614
H	5.888879	−14.047063	−3.300915
H	6.252972	−14.018803	−1.572361
N	6.684081	−11.500361	−3.218485
C	6.887452	−10.264092	−2.576210
O	7.693375	−10.102609	−1.659144
N	6.099548	−9.236425	−3.056747
C	5.157505	−9.308777	−4.074179
O	4.507580	−8.295379	−4.409492
C	5.006991	−10.609409	−4.705358
C	3.992919	−10.762522	−5.805311
C	5.790915	−11.625767	−4.261648
H	6.223250	−8.305399	−2.574465
H	3.001863	−10.447194	−5.457646
H	3.926104	−11.800872	−6.137611
H	4.242634	−10.132267	−6.667127
H	5.761977	−12.608677	−4.718447
P	8.025628	−15.721802	−0.196406
O	6.564103	−16.098763	−0.217812
O	9.063242	−16.649032	0.397721
O	8.096282	−14.255054	0.550582
C	9.397677	−13.659212	0.794147
C	9.322873	−12.663775	1.944178
O	8.613853	−11.448514	1.551823
C	7.352691	−11.343163	2.254660
C	8.604569	−13.184268	3.200429
C	7.171911	−12.662871	3.028565
O	9.271280	−12.597094	4.350776
H	9.736619	−13.145307	−0.113573
H	10.118184	−14.444598	1.050392
H	10.350188	−12.372646	2.194144
H	7.394798	−10.475426	2.918964
H	8.639732	−14.276853	3.265408
H	6.596663	−13.379492	2.439333
H	6.665535	−12.506004	3.984207
H	8.793297	−12.889662	5.148258
N	6.282995	−11.059128	1.304592
C	5.730683	−11.926913	0.363287
N	4.811176	−11.348238	−0.384570
C	4.750578	−10.029488	0.071090
C	3.946331	−8.930294	−0.349433
O	3.093348	−8.909419	−1.269581
N	4.198128	−7.771112	0.410614
C	5.117186	−7.676703	1.434096
N	5.189999	−6.494166	2.088988
N	5.893671	−8.698631	1.813096
C	5.666467	−9.831029	1.115501
H	6.071775	−12.948905	0.285070
H	3.671604	−6.908934	0.134214
H	4.733162	−5.649007	1.708014
H	6.005583	−6.361393	2.674714

d(CCA)$^{4-}$

E: −27581.15 kcal mol^{-1}

O	−0.367222	−0.124198	−0.465065
C	−0.296576	0.295896	0.920389
C	1.113500	0.101286	1.462578
O	1.421700	−1.323205	1.620452
C	2.525931	−1.703769	0.760210
C	2.245263	0.683713	0.591866
C	2.736239	−0.528419	−0.203562
O	3.241971	1.222149	1.509602
H	−1.305864	−0.141716	−0.721425
H	−0.993417	−0.289405	1.533615
H	−0.557612	1.360122	1.011704
H	1.148884	0.557894	2.457840
H	3.409360	−1.903018	1.368006
H	1.898044	1.490005	−0.058857
H	2.114254	−0.635305	−1.094671
H	3.771591	−0.420906	−0.517916
N	2.209572	−2.981477	0.101313
C	2.941387	−4.154170	0.444918
O	3.809101	−4.092067	1.343510
N	2.675463	−5.301110	−0.233369
C	1.718237	−5.345548	−1.181039
N	1.537863	−6.500157	−1.839074
C	0.907823	−4.198349	−1.474189
C	1.185962	−3.047563	−0.809823
H	2.121740	−7.325268	−1.630674
H	0.826217	−6.564404	−2.555151
H	0.100574	−4.246946	−2.195958
H	0.613242	−2.135530	−0.944853
P	4.733757	1.727277	1.008357
O	4.760115	1.948527	−0.484445
O	5.125584	2.825828	1.971330
O	5.665966	0.393189	1.279003
C	5.897277	−0.015412	2.654236
C	7.051199	−1.002979	2.737820
O	6.675277	−2.292985	2.161958
C	7.531901	−2.610632	1.036323
C	8.345820	−0.573916	2.016862
C	8.251054	−1.305294	0.676159
O	9.452891	−1.017135	2.857732
H	4.988637	−0.484484	3.051488
H	6.137025	0.864999	3.261792
H	7.263579	−1.162095	3.801565
H	8.228981	−3.401131	1.316447
H	8.415102	0.509758	1.892457
H	7.647409	−0.697150	−0.000748
H	9.221857	−1.468019	0.214644
N	6.715114	−3.176965	−0.044645
C	6.835007	−4.557565	−0.371915
O	7.624385	−5.273498	0.277734
N	6.082784	−5.041363	−1.396922
C	5.224869	−4.250102	−2.068895
N	4.526400	−4.787054	−3.079410
C	5.053353	−2.871184	−1.715287
C	5.809452	−2.388112	−0.697111
H	4.649036	−5.779508	−3.342861
H	3.869044	−4.215271	−3.592608
H	4.337956	−2.242937	−2.231759
H	5.726817	−1.369799	−0.335277
P	11.019569	−1.073893	2.339109
O	11.224020	−0.208265	1.119386
O	11.854820	−0.882131	3.585656
O	11.160751	−2.627352	1.806295
C	11.173774	−3.711650	2.771843
C	11.984707	−4.890458	2.246886
O	11.269692	−5.611427	1.195430
C	11.914699	−5.422097	−0.084726
C	13.349247	−4.517345	1.644287
C	13.055869	−4.411396	0.141443
O	14.265156	−5.602676	1.951337
H	10.143901	−4.034149	2.966659
H	11.623583	−3.362003	3.707842
H	12.120622	−5.591299	3.078943

H	12.278027	−6.390729	−0.439266
H	13.731392	−3.576875	2.054683
H	12.725511	−3.397264	−0.090751
H	13.929929	−4.643966	−0.471666
H	15.119387	−5.393940	1.530853
N	10.925010	−4.990860	−1.069387
C	10.288848	−3.758794	−1.139757
N	9.371409	−3.703051	−2.090923
C	9.388453	−4.969493	−2.673010
C	8.645447	−5.542286	−3.731300
N	7.703824	−4.884348	−4.430182
N	8.921044	−6.836901	−4.057016
C	9.871794	−7.510341	−3.382687
N	10.636169	−7.063004	−2.373462
C	10.346694	−5.786322	−2.051212
H	10.540679	−2.967941	−0.448259
H	7.184618	−5.364263	−5.175744
H	7.435501	−3.951680	−4.147749
H	10.018874	−8.541181	−3.699709
O	6.089630	−11.536801	−10.819441
C	7.302204	−12.316682	−10.972303
C	7.770319	−12.843121	−9.621617
O	8.297289	−11.758380	−8.788976
C	7.496097	−11.600917	−7.589723
C	6.690347	−13.541524	−8.770238
C	6.232576	−12.441760	−7.808917
O	7.343700	−14.664839	−8.108886
H	5.917257	−11.089930	−11.666486
H	8.100679	−11.701260	−11.405827
H	7.123254	−13.176313	−11.634364
H	8.593213	−13.541700	−9.809075
H	8.068851	−11.935145	−6.723202
H	5.866473	−13.926106	−9.376489
H	5.449779	−11.857035	−8.295610
H	5.833522	−12.842212	−6.880380
N	7.242624	−10.170136	−7.362718
C	7.878662	−9.532973	−6.284486
O	8.678949	−10.099634	−5.534762
N	7.532732	−8.205875	−6.125546
C	6.695353	−7.453683	−6.939070
O	6.484430	−6.246688	−6.677791
C	6.134883	−8.150542	−8.084240
C	5.264027	−7.396124	−9.052514
C	6.444079	−9.463682	−8.241826
H	8.016207	−7.707614	−5.332042
H	5.803948	−6.543781	−9.483585
H	4.376814	−6.992428	−8.548960
H	4.934823	−8.048045	−9.866735
H	6.097027	−10.034328	−9.097637
P	6.619872	−15.527031	−6.898401
O	5.123717	−15.325964	−6.908615
O	7.224844	−16.911058	−6.984791
O	7.165825	−14.761284	−5.544621
C	8.573811	−14.883545	−5.208293
C	8.821397	−14.492846	−3.758522
O	8.698494	−13.048411	−3.571966
C	7.600036	−12.746011	−2.674309
C	7.868046	−15.140685	−2.732923
C	6.814172	−14.052575	−2.516308
O	8.672885	−15.461875	−1.557977
H	9.163442	−14.232975	−5.866118
H	8.895191	−15.921406	−5.354752
H	9.855435	−14.768608	−3.520808
H	8.002931	−12.387260	−1.722228
H	7.425840	−16.067783	−3.107011
H	6.072102	−14.148543	−3.311249
H	6.307481	−14.132211	−1.556679
N	6.812810	−11.644635	−3.204755
C	5.886730	−11.678606	−4.244859
N	5.321053	−10.505783	−4.461024
C	5.896979	−9.647270	−3.520767
C	5.663174	−8.268973	−3.245060
O	4.874795	−7.491005	−3.834971
N	6.440354	−7.807889	−2.166521
C	7.359058	−8.568296	−1.467656
N	8.033552	−7.956036	−0.478451
N	7.595849	−9.856772	−1.740389
C	6.837001	−10.337188	−2.741187
H	5.694103	−12.588755	−4.793174
H	6.309739	−6.805646	−1.888676
H	7.866531	−6.972233	−0.215189
H	8.650085	−8.529145	0.081536
P	8.067339	−15.662423	−0.036370
O	6.604522	−16.035272	−0.061882
O	9.080767	−16.532133	0.674220
O	8.122237	−14.129955	0.565384
C	9.414497	−13.520582	0.824118
C	9.321155	−12.549188	1.993939
O	8.591534	−11.337754	1.627369
C	7.322513	−11.278395	2.320923
C	8.607638	−13.112808	3.234185
C	7.165032	−12.617125	3.066507
O	9.256096	−12.539625	4.401685
H	9.746291	−12.984915	−0.073179
H	10.145819	−14.299203	1.068130
H	10.342379	−12.245406	2.253507
H	7.338588	−10.423641	3.002948
H	8.664929	−14.205752	3.273584
H	6.606459	−13.333116	2.460840
H	6.652425	−12.490007	4.023230
H	8.779934	−12.861111	5.189138
N	6.254096	−10.997108	1.367999
C	5.718579	−11.853985	0.404479
N	4.798474	−11.270826	−0.338413
C	4.724379	−9.959667	0.137034
C	3.926011	−8.855271	−0.280107
O	3.063473	−8.829222	−1.192015
N	4.204209	−7.691155	0.462744
C	5.101779	−7.611648	1.508275
N	5.184086	−6.428580	2.158298
N	5.845646	−8.648717	1.908790
C	5.628259	−9.771555	1.194976
H	6.070654	−12.871024	0.308068
H	3.671831	−6.829638	0.198243
H	4.725876	−5.583050	1.783461
H	5.989448	−6.302193	2.758857

d(CGA)$^{4-}$

E: −27581.08 kcal mol^{-1}

O	−0.339523	−0.389595	−0.495817
C	−0.252056	0.011689	0.894234
C	1.180349	−0.116802	1.395863
O	1.567650	−1.523236	1.529551
C	2.665973	−1.839875	0.631397
C	2.255714	0.535318	0.503170
C	2.796939	−0.639957	−0.315121
O	3.235041	1.132237	1.403547
H	−1.282698	−0.449825	−0.727626
H	−0.901134	−0.617890	1.515887
H	−0.564644	1.059661	1.010137
H	1.218371	0.331218	2.395082
H	3.572957	−2.008131	1.213420
H	1.845872	1.323398	−0.133659
H	2.168660	−0.764517	−1.199311
H	3.819719	−0.479410	−0.644828

N	2.387987	−3.119998	−0.038337
C	3.133930	−4.280512	0.317150
O	3.991958	−4.202614	1.223364
N	2.880709	−5.438771	−0.348757
C	1.921460	−5.505151	−1.293106
N	1.738843	−6.673375	−1.926623
C	1.097866	−4.370403	−1.597351
C	1.362200	−3.210248	−0.944130
H	2.337372	−7.487676	−1.717366
H	1.032316	−6.747595	−2.646522
H	0.287066	−4.437282	−2.313784
H	0.770155	−2.311572	−1.081178
P	4.701531	1.697509	0.895477
O	4.723904	1.900700	−0.600231
O	5.039118	2.828616	1.842372
O	5.690566	0.412316	1.188344
C	5.902074	0.012293	2.567827
C	7.091239	−0.930399	2.683397
O	6.792415	−2.236580	2.101844
C	7.619219	−2.471108	0.933300
C	8.385306	−0.450444	1.997388
C	8.320101	−1.140469	0.633110
O	9.488119	−0.887694	2.848429
H	5.002674	−0.492489	2.941724
H	6.091774	0.900144	3.182857
H	7.277795	−1.082984	3.752670
H	8.329526	−3.272318	1.158035
H	8.431917	0.638115	1.906066
H	7.704596	−0.523556	−0.024135
H	9.297537	−1.269075	0.173305
N	6.804963	−2.971323	−0.162269
C	5.932948	−2.260904	−0.984013
N	5.344013	−3.028226	−1.882940
C	5.846337	−4.311629	−1.650654
C	5.596995	−5.551421	−2.308096
O	4.826496	−5.771316	−3.273238
N	6.358295	−6.600548	−1.757639
C	7.231922	−6.475694	−0.693831
N	7.862711	−7.592242	−0.284288
N	7.447489	−5.318305	−0.061130
C	6.749503	−4.292551	−0.577355
H	5.780829	−1.199939	−0.852414
H	6.274483	−7.537371	−2.221014
H	7.824670	−8.474079	−0.819501
H	8.578850	−7.474041	0.420342
P	11.048296	−1.048477	2.337861
O	11.323685	−0.201022	1.119022
O	11.884561	−0.910798	3.590789
O	11.087290	−2.609436	1.807549
C	10.979432	−3.684935	2.777599
C	11.723274	−4.922352	2.291408
O	11.012808	−5.581301	1.197271
C	11.748587	−5.447660	−0.040190
C	13.143203	−4.650915	1.766883
C	12.940178	−4.517189	0.251311
O	13.957378	−5.803823	2.112652
H	9.921302	−3.929464	2.929263
H	11.413442	−3.363562	3.731029
H	11.760268	−5.633277	3.125344
H	12.066735	−6.442143	−0.365946
H	13.571942	−3.743811	2.205514
H	12.690638	−3.481893	0.011656
H	13.828105	−4.805169	−0.316693
H	14.846765	−5.657112	1.741730
N	10.855359	−4.961289	−1.088994
C	10.330867	−3.682498	−1.234524
N	9.476950	−3.585154	−2.239849
C	9.416610	−4.869214	−2.780374
C	8.668799	−5.425854	−3.844118

N	7.820875	−4.721260	−4.619172
N	8.831405	−6.755758	−4.092654
C	9.681660	−7.480548	−3.341879
N	10.441391	−7.053415	−2.320577
C	10.264176	−5.740213	−2.075140
H	10.608736	−2.887752	−0.556947
H	7.229280	−5.218505	−5.295601
H	7.577949	−3.777103	−4.348017
H	9.739570	−8.536471	−3.598688
O	5.945965	−11.505681	−10.796348
C	7.148410	−12.297430	−10.966420
C	7.636911	−12.815301	−9.619618
O	8.188901	−11.726711	−8.807618
C	7.414501	−11.549240	−7.596145
C	6.565099	−13.495263	−8.743321
C	6.140374	−12.385802	−7.777306
O	7.213666	−14.624795	−8.089457
H	5.757598	−11.068206	−11.644863
H	7.943539	−11.693356	−11.421291
H	6.948803	−13.161729	−11.616195
H	8.450211	−13.522891	−9.814046
H	8.002891	−11.873469	−6.736063
H	5.723853	−13.869644	−9.331773
H	5.348376	−11.799413	−8.247061
H	5.762754	−12.781091	−6.837402
N	7.170889	−10.112779	−7.382679
C	7.814285	−9.466832	−6.313414
O	8.619149	−10.028729	−5.564509
N	7.459639	−8.142470	−6.154416
C	6.601335	−7.400269	−6.956868
O	6.376407	−6.198487	−6.689065
C	6.030593	−8.106247	−8.092056
C	5.133404	−7.364734	−9.045699
C	6.351875	−9.416173	−8.252004
H	7.934444	−7.636933	−5.358391
H	5.657470	−6.511357	−9.493598
H	4.254664	−6.964166	−8.525026
H	4.791242	−8.024421	−9.848365
H	5.996606	−9.993444	−9.100027
P	6.489703	−15.482989	−6.876227
O	4.996756	−15.260531	−6.868956
O	7.075832	−16.874050	−6.972038
O	7.061575	−14.728888	−5.524593
C	8.481998	−14.839377	−5.238120
C	8.783531	−14.374524	−3.822451
O	8.620885	−12.927213	−3.710065
C	7.630674	−12.607930	−2.699467
C	7.903145	−14.998644	−2.718414
C	6.862898	−13.909941	−2.442681
O	8.788281	−15.276251	−1.592958
H	9.045944	−14.225419	−5.951166
H	8.796556	−15.884082	−5.344602
H	9.834963	−14.611778	−3.620790
H	8.126401	−12.240330	−1.799898
H	7.440193	−15.936038	−3.036800
H	6.044615	−14.033006	−3.155533
H	6.450510	−13.966509	−1.438493
N	6.806964	−11.489400	−3.175325
C	6.964696	−10.197709	−2.594551
O	7.820680	−10.031285	−1.699844
N	6.163711	−9.192545	−3.035606
C	5.276396	−9.390007	−4.028050
N	4.517617	−8.354682	−4.416352
C	5.160503	−10.664045	−4.677838
C	5.946743	−11.672148	−4.222005
H	4.619126	−7.424641	−3.977092
H	3.847441	−8.478144	−5.163251
H	4.481182	−10.813449	−5.508589
H	5.951031	−12.659593	−4.668472

P	8.243375	−15.701375	−0.092580
O	6.801305	−16.144952	−0.138516
O	9.319917	−16.595459	0.482735
O	8.239599	−14.256406	0.698943
C	9.509378	−13.617798	0.995572
C	9.370788	−12.669582	2.179796
O	8.638451	−11.458912	1.816534
C	7.350779	−11.421058	2.479173
C	8.633440	−13.258622	3.393725
C	7.190527	−12.772281	3.201309
O	9.250300	−12.697881	4.584068
H	9.847413	−13.057476	0.115421
H	10.254618	−14.383246	1.240474
H	10.381778	−12.359583	2.470261
H	7.340569	−10.574820	3.171707
H	8.699116	−14.351422	3.418005
H	6.651378	−13.484002	2.573276
H	6.655945	−12.664693	4.148259
H	8.758782	−13.034285	5.355634
N	6.309179	−11.140673	1.498244
C	5.835485	−11.991964	0.499889
N	4.963681	−11.406267	−0.297153
C	4.859422	−10.097861	0.179202
C	4.083438	−8.995088	−0.281167
O	3.274122	−8.969473	−1.239067
N	4.311803	−7.834278	0.485869
C	5.147270	−7.757367	1.580802
N	5.203254	−6.567489	2.225753
N	5.858091	−8.797004	2.029901
C	5.692705	−9.914159	1.293821
H	6.195762	−13.007220	0.422453
H	3.802652	−6.969437	0.183918
H	4.810925	−5.714412	1.795211
H	5.964812	−6.453188	2.883667

d(CGG)$^{4-}$

E: −27490.47 kcal mol^{-1}

H: −26569.42 kcal mol^{-1}

G: −26716.5 kcal mol^{-1}

O	−0.405267	−0.291866	−0.288853
C	−0.290845	0.065607	1.111120
C	1.151282	−0.078903	1.579341
O	1.540739	−1.488774	1.655837
C	2.620591	−1.771864	0.724749
C	2.208408	0.604974	0.687707
C	2.729228	−0.541709	−0.183577
O	3.204294	1.173755	1.588356
H	−1.352810	−0.338032	−0.505722
H	−0.927652	−0.583576	1.725367
H	−0.600592	1.109324	1.266241
H	1.209966	0.333876	2.592591
H	3.539935	−1.955618	1.281981
H	1.785245	1.414597	0.087549
H	2.076492	−0.634873	−1.053693
H	3.743583	−0.370933	−0.535317
N	2.334446	−3.030446	0.019823
C	3.089227	−4.197753	0.331418
O	3.956498	−4.141920	1.230150
N	2.833099	−5.336699	−0.365050
C	1.863089	−5.379065	−1.299340
N	1.677621	−6.529259	−1.964699
C	1.031499	−4.239200	−1.560492
C	1.298617	−3.097647	−0.876301
H	2.281330	−7.346650	−1.785554
H	0.961165	−6.586579	−2.676283
H	0.213117	−4.288104	−2.269695
H	0.701598	−2.197850	−0.980068
P	4.692867	1.685585	1.088703
O	4.716573	1.942261	−0.398802
O	5.088111	2.764560	2.073079
O	5.619455	0.344780	1.326485
C	5.847642	−0.101996	2.689164
C	7.072092	−1.002706	2.763863
O	6.811994	−2.308048	2.163362
C	7.610925	−2.484304	0.965955
C	8.329290	−0.456108	2.059208
C	8.264415	−1.125241	0.684029
O	9.469320	−0.851606	2.880370
H	4.967864	−0.653622	3.042779
H	6.006971	0.767220	3.338005
H	7.287764	−1.169401	3.825477
H	8.349523	−3.270494	1.148460
H	8.323290	0.634627	1.983529
H	7.620106	−0.515783	0.048026
H	9.238389	−1.213214	0.207045
N	6.782135	−2.976170	−0.123238
C	5.877783	−2.265315	−0.909487
N	5.293731	−3.021545	−1.820730
C	5.830943	−4.297879	−1.633000
C	5.602126	−5.523457	−2.323228
O	4.825555	−5.733277	−3.286152
N	6.387278	−6.572461	−1.806344
C	7.267848	−6.458677	−0.746061
N	7.923276	−7.572850	−0.373253
N	7.471795	−5.312508	−0.089643
C	6.749422	−4.287349	−0.572896
H	5.696860	−1.213847	−0.742239
H	6.299253	−7.505343	−2.276629
H	7.833012	−8.471334	−0.874492
H	8.617220	−7.473533	0.355337
P	11.019637	−0.957340	2.328077
O	11.237543	−0.088292	1.112544
O	11.882524	−0.802665	3.561199
O	11.094155	−2.510174	1.781671
C	11.051692	−3.594314	2.747083
C	11.806832	−4.806959	2.219797
O	11.064642	−5.470285	1.150403
C	11.763637	−5.338711	−0.110272
C	13.196333	−4.490899	1.640193
C	12.929459	−4.363476	0.135081
O	14.060501	−5.617168	1.952464
H	10.006904	−3.867048	2.938520
H	11.514960	−3.268350	3.685118
H	11.899466	−5.523266	3.045057
H	12.110803	−6.327860	−0.423139
H	13.613012	−3.571093	2.063625
H	12.629172	−3.338551	−0.090968
H	13.804586	−4.616066	−0.468739
H	14.930257	−5.439528	1.549827
N	10.831949	−4.906767	−1.144696
C	10.259840	−3.644562	−1.309835
N	9.417601	−3.597219	−2.323458
C	9.411910	−4.890366	−2.851844
C	8.685779	−5.456618	−3.940024
O	7.874358	−4.881938	−4.706610
N	8.961834	−6.828552	−4.103242
C	9.861566	−7.553515	−3.347895
N	10.057148	−8.844288	−3.697128
N	10.550615	−7.023140	−2.331511
C	10.282575	−5.718544	−2.125690
H	10.501531	−2.832374	−0.639402
H	8.448966	−7.318869	−4.873134
H	9.454182	−9.305528	−4.396721
H	10.532749	−9.427647	−3.020033

O	6.018264	−11.570197	−10.989141
C	7.192981	−12.408663	−11.124056
C	7.646166	−12.910589	−9.759298
O	8.208661	−11.818515	−8.960708
C	7.416258	−11.601594	−7.764809
C	6.545521	−13.548441	−8.886438
C	6.124207	−12.405152	−7.959150
O	7.164607	−14.671464	−8.192338
H	5.864153	−11.136773	−11.846678
H	8.015281	−11.844293	−11.581650
H	6.971032	−13.280375	−11.756604
H	8.445520	−13.640903	−9.926631
H	7.979694	−11.930577	−6.891045
H	5.709100	−13.925497	−9.480339
H	5.363078	−11.809707	−8.467463
H	5.708721	−12.764980	−7.020263
N	7.210956	−10.157337	−7.574149
C	7.853558	−9.492788	−6.491386
O	8.625055	−10.139863	−5.749915
N	7.591917	−8.173962	−6.298862
C	6.777080	−7.495497	−7.131106
N	6.535026	−6.205185	−6.858657
C	6.192473	−8.127307	−8.279059
C	6.441227	−9.449161	−8.462001
H	6.974549	−5.745734	−6.045849
H	5.937681	−5.665260	−7.470972
H	5.582276	−7.569932	−8.980399
H	6.070700	−10.006475	−9.316242
P	6.438123	−15.457475	−6.933671
O	4.949146	−15.209899	−6.921693
O	6.999018	−16.861699	−6.970280
O	7.035942	−14.652015	−5.624341
C	8.452989	−14.783389	−5.329929
C	8.746040	−14.361480	−3.898688
O	8.621937	−12.914678	−3.746597
C	7.599855	−12.594056	−2.769417
C	7.830028	−14.990383	−2.827516
C	6.803161	−13.887012	−2.558016
O	8.685004	−15.307473	−1.688925
H	9.027627	−14.155032	−6.021501
H	8.758090	−15.827802	−5.462053
H	9.787558	−14.631736	−3.687590
H	8.067813	−12.243814	−1.848664
H	7.358216	−15.913235	−3.174528
H	6.003390	−13.981270	−3.295932
H	6.361828	−13.954095	−1.566691
N	6.808115	−11.457754	−3.258600
C	6.944449	−10.183148	−2.635395
O	7.743815	−10.051530	−1.685902
N	6.187437	−9.155201	−3.102745
C	5.345378	−9.322389	−4.139787
N	4.614446	−8.271909	−4.538920
C	5.242102	−10.583079	−4.817289
C	5.997166	−11.608888	−4.349035
H	4.693224	−7.355961	−4.066578
H	3.991143	−8.367856	−5.329239
H	4.598463	−10.707545	−5.679980
H	6.014123	−12.586347	−4.817254
P	8.106350	−15.688421	−0.190882
O	6.657097	−16.106628	−0.249615
O	9.156338	−16.590044	0.420438
O	8.116632	−14.226699	0.568460
C	9.392873	−13.605357	0.873152
C	9.259712	−12.663610	2.063045
O	8.543491	−11.442744	1.703823
C	7.250206	−11.398247	2.354500
C	8.507362	−13.253561	3.267160
C	7.070597	−12.752970	3.065230
O	9.119022	−12.705744	4.466123
H	9.739546	−13.042898	−0.002245
H	10.128687	−14.380687	1.115401
H	10.272362	−12.367239	2.362014
H	7.242837	−10.557781	3.054096
H	8.563080	−14.347060	3.285074
H	6.531212	−13.455526	2.427294
H	6.528513	−12.646912	4.008135
H	8.617150	−13.041413	5.231319
N	6.218440	−11.097357	1.369332
C	5.726297	−11.933581	0.367308
N	4.862108	−11.327193	−0.422770
C	4.782220	−10.020025	0.062417
C	4.026192	−8.899073	−0.387990
O	3.220354	−8.848267	−1.348049
N	4.270242	−7.751108	0.393952
C	5.108811	−7.698303	1.488195
N	5.178726	−6.519780	2.151377
N	5.810214	−8.751630	1.919710
C	5.623154	−9.859151	1.174563
H	6.067124	−12.954806	0.281275
H	3.765338	−6.877657	0.109904
H	4.784965	−5.656754	1.741933
H	5.944215	−6.422465	2.807377

d(CTA)$^{4-}$

E: −27671.19 kcal mol^{-1}

O	−0.379882	−0.207458	−0.283369
C	−0.274603	0.133469	1.121678
C	1.161783	−0.035792	1.599475
O	1.527812	−1.453844	1.666602
C	2.610420	−1.746614	0.746743
C	2.231770	0.639396	0.716906
C	2.755505	−0.509840	−0.147081
O	3.230968	1.203305	1.615733
H	−1.325617	−0.238524	−0.510785
H	−0.925953	−0.513578	1.722490
H	−0.571554	1.179562	1.284485
H	1.221132	0.366684	2.616669
H	3.520494	−1.959333	1.309063
H	1.817543	1.448413	0.110157
H	2.126582	−0.590078	−1.035345
H	3.781825	−0.348754	−0.465816
N	2.305490	−2.989671	0.016262
C	3.063275	−4.166556	0.277314
O	3.972054	−4.130551	1.135909
N	2.775301	−5.290622	−0.429939
C	1.779594	−5.306801	−1.338298
N	1.568351	−6.442200	−2.020172
C	0.958631	−4.151840	−1.563265
C	1.248859	−3.028591	−0.859245
H	2.101027	−7.297462	−1.800325
H	0.801237	−6.495702	−2.677598
H	0.126939	−4.177557	−2.257797
H	0.659561	−2.120155	−0.930613
P	4.694151	1.753972	1.076462
O	4.658315	2.041036	−0.404711
O	5.105105	2.816660	2.071621
O	5.652577	0.424036	1.252698
C	5.901105	−0.052994	2.601843
C	7.066385	−1.027448	2.616312
O	6.691313	−2.282842	1.970896
C	7.582302	−2.561952	0.864257
C	8.346861	−0.536685	1.905814
C	8.280224	−1.235862	0.544958
O	9.465140	−0.950137	2.745693
H	5.001885	−0.552423	2.982942

H	6.137923	0.795856	3.254014
H	7.298907	−1.243897	3.665812
H	8.295528	−3.337029	1.151339
H	8.374624	0.551292	1.804876
H	7.673101	−0.617630	−0.119353
H	9.258541	−1.367278	0.089323
N	6.805414	−3.144731	−0.237506
C	6.938152	−4.524020	−0.486558
O	7.691240	−5.258241	0.154308
N	6.156962	−4.993957	−1.524501
C	5.292984	−4.255407	−2.322219
O	4.646939	−4.808650	−3.238334
C	5.204384	−2.838511	−2.015328
C	4.316678	−1.973367	−2.867193
C	5.941942	−2.365359	−0.976728
H	6.256082	−6.024782	−1.740491
H	4.591237	−2.070431	−3.924322
H	4.402587	−0.921897	−2.584832
H	3.266544	−2.276846	−2.783467
H	5.884719	−1.328854	−0.664457
P	11.037514	−0.982254	2.246831
O	11.240992	−0.160140	0.997232
O	11.854328	−0.725208	3.493891
O	11.216327	−2.552279	1.778156
C	11.149383	−3.595881	2.785190
C	11.875465	−4.845556	2.306193
O	11.132615	−5.522331	1.245146
C	11.830749	−5.413498	−0.016379
C	13.282143	−4.596343	1.738578
C	13.040692	−4.491622	0.226362
O	14.095240	−5.748951	2.087442
H	10.098973	−3.837134	2.988061
H	11.620331	−3.244240	3.710463
H	11.930494	−5.539044	3.153573
H	12.128019	−6.415695	−0.337681
H	13.729681	−3.684288	2.147056
H	12.796986	−3.458832	−0.029452
H	13.909990	−4.802211	−0.358097
H	14.975431	−5.619325	1.689330
N	10.911889	−4.930063	−1.044869
C	10.391754	−3.649969	−1.181101
N	9.521438	−3.547770	−2.172706
C	9.452981	−4.829287	−2.717353
C	8.704525	−5.373281	−3.786866
N	7.840637	−4.666001	−4.538188
N	8.885187	−6.694919	−4.064243
C	9.735396	−7.426784	−3.322130
N	10.483427	−7.015688	−2.284866
C	10.304616	−5.705173	−2.024261
H	10.685139	−2.858700	−0.506398
H	7.285900	−5.137894	−5.262783
H	7.657325	−3.698253	−4.310382
H	9.805891	−8.476263	−3.601471
O	5.852389	−11.459056	−10.698096
C	7.042906	−12.268116	−10.870387
C	7.550538	−12.764413	−9.522416
O	8.118382	−11.664661	−8.736622
C	7.360560	−11.470311	−7.515380
C	6.494224	−13.426946	−8.613630
C	6.076095	−12.290113	−7.677895
O	7.166375	−14.532338	−7.939691
H	5.656443	−11.036885	−11.552673
H	7.836832	−11.682581	−11.351049
H	6.823182	−13.143225	−11.498925
H	8.358577	−13.477450	−9.719229
H	7.954534	−11.798838	−6.661234
H	5.649321	−13.827595	−9.179528
H	5.298654	−11.704350	−8.172589
H	5.683801	−12.647293	−6.728933
N	7.138972	−10.031806	−7.307788
C	7.789911	−9.394017	−6.239723
O	8.583312	−9.966280	−5.486338
N	7.462741	−8.060699	−6.092910
C	6.647127	−7.300057	−6.921836
O	6.463258	−6.085223	−6.676429
C	6.069546	−8.001079	−8.055768
C	5.214655	−7.242650	−9.035059
C	6.350029	−9.323190	−8.193808
H	7.960321	−7.555951	−5.310331
H	5.774765	−6.411551	−9.481553
H	4.338416	−6.808507	−8.537585
H	4.868402	−7.900477	−9.837566
H	5.986331	−9.901093	−9.038006
P	6.537041	−15.311112	−6.626446
O	5.051003	−15.073656	−6.507784
O	7.104604	−16.711485	−6.689762
O	7.218590	−14.489605	−5.369485
C	8.650755	−14.606522	−5.159508
C	9.011608	−14.365286	−3.699571
O	8.915961	−12.952634	−3.336200
C	7.794411	−12.723399	−2.451659
C	8.136428	−15.118149	−2.681710
C	7.080569	−14.077057	−2.291226
O	9.015930	−15.521827	−1.591251
H	9.169946	−13.874463	−5.789900
H	8.978499	−15.614435	−5.438971
H	10.060887	−14.653690	−3.573633
H	8.171378	−12.334161	−1.501262
H	7.680140	−16.014727	−3.109221
H	6.244013	−14.161122	−2.986085
H	6.705606	−14.227564	−1.281581
N	6.937693	−11.667444	−2.990457
C	6.011575	−11.764965	−4.020404
N	5.403336	−10.618039	−4.279336
C	5.956947	−9.711047	−3.376887
C	5.724123	−8.334878	−3.146620
N	4.850535	−7.595635	−3.848692
N	6.441537	−7.741169	−2.150708
C	7.348889	−8.457459	−1.462094
N	7.659393	−9.754739	−1.612538
C	6.923403	−10.339512	−2.576938
H	5.854206	−12.697242	−4.542974
H	4.737572	−6.594081	−3.644025
H	4.300951	−8.031385	−4.576137
H	7.887149	−7.903770	−0.695958
P	8.447589	−16.050969	−0.133757
O	7.048641	−16.604948	−0.259764
O	9.569459	−16.885179	0.444662
O	8.298095	−14.645551	0.713483
C	9.508312	−13.899537	1.007407
C	9.197982	−12.761247	1.963739
O	8.404268	−11.740153	1.282149
C	7.211825	−11.456879	2.053206
C	8.405888	−13.171152	3.224561
C	6.978164	−12.706881	2.913149
O	8.984625	−12.452649	4.347815
H	9.925744	−13.496309	0.076599
H	10.249481	−14.563558	1.468074
H	10.151912	−12.311061	2.267618
H	7.369648	−10.564496	2.668427
H	8.460467	−14.249355	3.403800
H	6.466780	−13.478071	2.331007
H	6.395618	−12.491516	3.812435
H	8.485883	−12.705312	5.146387
N	6.126218	−11.129246	1.145246
C	5.467807	−11.988235	0.266131
N	4.537741	−11.378120	−0.441678
C	4.575715	−10.047531	−0.018875

C	3.801300	−8.917410	−0.413552
O	2.886394	−8.873963	−1.271873
N	4.166793	−7.753938	0.289936
C	5.153208	−7.685103	1.251382
N	5.328198	−6.495470	1.868546
N	5.895233	−8.740042	1.611250
C	5.563893	−9.874051	0.962545
H	5.727706	−13.034639	0.204823
H	3.668517	−6.871500	0.026525
H	4.864020	−5.641987	1.518622
H	6.193635	−6.380599	2.381654

d(GAA)$^{4-}$

E: −27674.20 kcal mol^{-1}

O	−0.260171	−0.270211	−0.480739
C	−0.184086	0.185351	0.892086
C	1.238114	0.053868	1.422565
O	1.614535	−1.351757	1.598825
C	2.657325	−1.720211	0.659292
C	2.340818	0.667298	0.537989
C	2.820562	−0.527419	−0.290636
O	3.342150	1.212601	1.448933
H	−1.201136	−0.314711	−0.724564
H	−0.851345	−0.408272	1.530048
H	−0.480592	1.242225	0.963858
H	1.261433	0.521488	2.412826
H	3.573597	−1.935618	1.216698
H	1.969161	1.480537	−0.091003
H	2.158745	−0.630487	−1.152016
H	3.840321	−0.412290	−0.650715
N	2.307243	−2.967357	−0.010148
C	1.371624	−3.167196	−1.025466
N	1.314349	−4.425521	−1.421940
C	2.252341	−5.097244	−0.634552
C	2.650500	−6.465206	−0.615982
O	2.217664	−7.401414	−1.333199
N	3.652630	−6.710266	0.342096
C	4.176028	−5.763640	1.199776
N	5.079797	−6.191974	2.114425
N	3.806059	−4.480308	1.183659
C	2.869299	−4.206946	0.256443
H	0.757414	−2.355041	−1.388410
H	4.041535	−7.681974	0.363505
H	5.519056	−7.118584	2.007214
H	5.631388	−5.463647	2.553951
P	4.897702	1.546869	1.011853
O	5.016741	1.774706	−0.475819
O	5.366380	2.588020	2.003241
O	5.659087	0.112482	1.302121
C	5.832033	−0.323065	2.677441
C	7.091197	−1.168825	2.823424
O	6.918017	−2.509495	2.263097
C	7.712934	−2.667697	1.065289
C	8.336881	−0.583089	2.133336
C	8.346418	−1.297753	0.777712
O	9.478586	−0.887664	2.987479
H	4.957670	−0.909307	2.984629
H	5.921706	0.553319	3.328989
H	7.279319	−1.294968	3.895128
H	8.460241	−3.447725	1.240482
H	8.272604	0.501943	2.017395
H	7.726626	−0.721686	0.089793
H	9.344399	−1.377219	0.354516
N	6.879744	−3.167086	−0.026807
C	5.984941	−2.450956	−0.812181
N	5.377728	−3.200395	−1.718080
C	5.888965	−4.482360	−1.519184
C	5.641142	−5.718698	−2.161341
N	4.779137	−5.871288	−3.178844
N	6.326873	−6.805947	−1.706759
C	7.187662	−6.672132	−0.680125
N	7.493229	−5.553435	−0.003986
C	6.817510	−4.484659	−0.466096
H	5.819979	−1.395623	−0.650628
H	4.639421	−6.796844	−3.603095
H	4.258545	−5.072987	−3.515282
H	7.690121	−7.587272	−0.376308
P	11.042288	−0.832700	2.459243
O	11.187578	0.059149	1.249983
O	11.867943	−0.600033	3.705383
O	11.274383	−2.369724	1.910174
C	11.236471	−3.451560	2.877838
C	11.854766	−4.707357	2.285197
O	10.993341	−5.259831	1.240239
C	11.689359	−5.277388	−0.027531
C	13.239235	−4.504625	1.641789
C	12.919855	−4.373527	0.148002
O	14.026255	−5.690837	1.933935
H	10.195954	−3.651066	3.161884
H	11.799269	−3.165005	3.774045
H	11.926718	−5.452010	3.087520
H	11.965558	−6.307300	−0.274666
H	13.742424	−3.615465	2.035044
H	12.667866	−3.334233	−0.073950
H	13.750763	−4.681467	−0.491260
H	14.894001	−5.581157	1.503607
N	10.776709	−4.849665	−1.080785
C	10.297375	−3.567406	−1.314618
N	9.436215	−3.511824	−2.317062
C	9.331714	−4.827175	−2.768617
C	8.572474	−5.426995	−3.801234
N	7.745844	−4.748884	−4.619128
N	8.703406	−6.773442	−3.970518
C	9.518554	−7.474844	−3.162333
N	10.278892	−7.008457	−2.157604
C	10.151213	−5.675318	−2.006161
H	10.618015	−2.732329	−0.708461
H	7.156085	−5.263450	−5.283997
H	7.557118	−3.772134	−4.435644
H	9.548914	−8.545721	−3.350307
O	6.251271	−11.548681	−10.791976
C	7.492000	−12.288351	−10.914864
C	7.930954	−12.811772	−9.553593
O	8.397763	−11.716578	−8.696813
C	7.561567	−11.594347	−7.521278
C	6.839343	−13.548073	−8.749896
C	6.337654	−12.483848	−7.771292
O	7.479136	−14.685964	−8.102683
H	6.085065	−11.105937	−11.642438
H	8.283647	−11.644780	−11.318774
H	7.359992	−13.147556	−11.588163
H	8.780093	−13.484548	−9.715518
H	8.124355	−11.902842	−6.638546
H	6.036406	−13.921114	−9.390349
H	5.534075	−11.922043	−8.249971
H	5.951272	−12.921147	−6.854503
N	7.237072	−10.173424	−7.298409
C	7.801153	−9.513483	−6.194566
O	8.584958	−10.052265	−5.406595
N	7.391897	−8.203945	−6.043728
C	6.551976	−7.483617	−6.885797
O	6.281138	−6.289913	−6.626780
C	6.060217	−8.202276	−8.049719
C	5.181450	−7.486647	−9.039386
C	6.437364	−9.498006	−8.203218

H	7.832924	−7.682254	−5.238770
H	5.690033	−6.605534	−9.449623
H	4.261204	−7.131381	−8.559174
H	4.909219	−8.149121	−9.866198
H	6.146411	−10.079742	−9.072492
P	6.711884	−15.580797	−6.941765
O	5.217393	−15.379770	−6.997946
O	7.324388	−16.960524	−7.038689
O	7.210454	−14.844906	−5.551701
C	8.622114	−14.926705	−5.219648
C	8.857960	−14.447778	−3.798525
O	8.647514	−13.004119	−3.714573
C	7.674748	−12.698482	−2.691528
C	7.949937	−15.093927	−2.727459
C	6.913251	−14.005418	−2.432743
O	8.805316	−15.425075	−1.595535
H	9.198134	−14.305420	−5.916413
H	8.961502	−15.965405	−5.308186
H	9.905741	−14.653688	−3.549141
H	8.178550	−12.335957	−1.792639
H	7.479889	−16.012187	−3.087139
H	6.083736	−14.126514	−3.132398
H	6.519541	−14.075077	−1.421277
N	6.840434	−11.575452	−3.146119
C	7.031422	−10.312821	−2.552010
O	7.873170	−10.096135	−1.678998
N	6.173467	−9.330777	−3.007603
C	5.190954	−9.465919	−3.979786
O	4.473735	−8.493121	−4.295016
C	5.075537	−10.781884	−4.586949
C	4.026286	−10.995153	−5.643052
C	5.915122	−11.757663	−4.153117
H	6.254957	−8.390974	−2.527821
H	3.035491	−10.729521	−5.256226
H	4.000878	−12.037094	−5.969195
H	4.207365	−10.356029	−6.515343
H	5.905647	−12.751420	−4.586393
P	8.210707	−15.911545	−0.131464
O	6.767495	−16.339190	−0.239443
O	9.262563	−16.837647	0.437828
O	8.198538	−14.496152	0.716242
C	9.478390	−13.867761	0.996155
C	9.302500	−12.742206	2.000332
O	8.572837	−11.633683	1.389437
C	7.390667	−11.326281	2.162505
C	8.534743	−13.122586	3.283387
C	7.129075	−12.558014	3.044409
O	9.208589	−12.471720	4.394332
H	9.898270	−13.466971	0.065562
H	10.171498	−14.611129	1.407026
H	10.303077	−12.382479	2.271902
H	7.557007	−10.425139	2.753613
H	8.521532	−14.206037	3.439275
H	6.527429	−13.298846	2.511564
H	6.617719	−12.295815	3.974079
H	8.720781	−12.698983	5.207141
N	6.297841	−10.996590	1.235036
C	5.766536	−9.678819	1.196010
O	6.234843	−8.803543	1.960678
N	4.751452	−9.419046	0.331285
C	4.280461	−10.371903	−0.497296
N	3.282408	−10.042735	−1.331281
C	4.836122	−11.693699	−0.504530
C	5.841315	−11.953106	0.369262
H	2.907844	−9.080961	−1.350272
H	2.909567	−10.734748	−1.967401
H	4.475519	−12.451856	−1.189104
H	6.348588	−12.910079	0.419772

d(GAC)$^{4-}$

E: −27586.17 kcal mol^{-1}

O	−0.230564	−0.224703	−0.517743
C	−0.162692	0.215712	0.860233
C	1.250501	0.053729	1.406550
O	1.602347	−1.360829	1.562579
C	2.654954	−1.727056	0.633250
C	2.375426	0.665020	0.547865
C	2.844213	−0.522692	−0.296478
O	3.372255	1.178607	1.482824
H	−1.169309	−0.249981	−0.772881
H	−0.847734	−0.372715	1.484056
H	−0.441790	1.276630	0.939734
H	1.267746	0.503722	2.405059
H	3.560713	−1.959889	1.200355
H	2.025739	1.496031	−0.070418
H	2.185208	−0.602233	−1.162664
H	3.867531	−0.417299	−0.649855
N	2.303188	−2.960347	−0.059852
C	1.376716	−3.136754	−1.088020
N	1.326864	−4.384775	−1.516810
C	2.261316	−5.073236	−0.739505
C	2.666447	−6.439193	−0.755042
O	2.242807	−7.357673	−1.500068
N	3.664161	−6.704862	0.202224
C	4.175370	−5.779321	1.089991
N	5.075407	−6.226933	1.998383
N	3.798359	−4.497991	1.106367
C	2.867661	−4.204299	0.179328
H	0.763605	−2.316877	−1.435373
H	4.044872	−7.680524	0.212607
H	5.517803	−7.150059	1.874372
H	5.612112	−5.508516	2.471179
P	4.955212	1.446199	1.104623
O	5.141792	1.673423	−0.376174
O	5.431134	2.461497	2.118730
O	5.643668	−0.020654	1.413298
C	5.765039	−0.473521	2.788121
C	7.032895	−1.299061	2.973880
O	6.913516	−2.634573	2.386828
C	7.737923	−2.751645	1.205215
C	8.290627	−0.674147	2.344776
C	8.386765	−1.372987	0.983005
O	9.403863	−0.961231	3.239953
H	4.888410	−1.078633	3.047704
H	5.812601	0.393874	3.455714
H	7.179541	−1.440094	4.050069
H	8.477350	−3.541309	1.368634
H	8.202524	0.409549	2.235564
H	7.821461	−0.789845	0.255216
H	9.414335	−1.453610	0.637634
N	6.928979	−3.206054	0.072426
C	6.029349	−2.457868	−0.678822
N	5.441248	−3.162248	−1.631898
C	5.968547	−4.445746	−1.502732
C	5.731824	−5.647559	−2.211663
N	4.868384	−5.751600	−3.234210
N	6.423403	−6.753076	−1.815060
C	7.285419	−6.666994	−0.784432
N	7.580842	−5.584546	−0.047263
C	6.891739	−4.497355	−0.446009
H	5.845848	−1.417645	−0.452146
H	4.702378	−6.664380	−3.675371
H	4.320203	−4.947679	−3.507923
H	7.792936	−7.594444	−0.529577
P	10.972673	−0.676135	2.805977

O	11.056897	0.363350	1.714386
O	11.716327	−0.504122	4.111906
O	11.403868	−2.102650	2.099317
C	11.406762	−3.291530	2.933364
C	11.915177	−4.478782	2.137236
O	10.938396	−4.831101	1.110303
C	11.618053	−4.994229	−0.157402
C	13.266236	−4.256827	1.416117
C	12.870537	−4.113074	−0.059535
O	14.079478	−5.436782	1.658252
H	10.388584	−3.493757	3.287335
H	12.057734	−3.133851	3.801697
H	12.018009	−5.326299	2.827727
H	11.872967	−6.043218	−0.316267
H	13.782290	−3.367839	1.790996
H	12.630408	−3.066861	−0.267660
H	13.661125	−4.436457	−0.741345
H	14.941085	−5.293197	1.225656
N	10.683329	−4.643050	−1.230644
C	10.181932	−5.655336	−2.094683
O	10.562408	−6.838338	−1.939008
N	9.308934	−5.295331	−3.070374
C	8.880896	−4.022436	−3.189974
N	8.015877	−3.741180	−4.175040
C	9.333231	−2.997908	−2.293570
C	10.228652	−3.356316	−1.340380
H	7.666931	−4.486232	−4.797317
H	7.632233	−2.808800	−4.255991
H	8.972948	−1.979160	−2.370575
H	10.617613	−2.657845	−0.610518
O	6.339051	−11.406261	−10.875540
C	7.511868	−12.252913	−10.951928
C	7.820641	−12.858051	−9.588024
O	8.320735	−11.845726	−8.652595
C	7.360557	−11.607427	−7.593710
C	6.624625	−13.525372	−8.881404
C	6.112273	−12.426728	−7.947133
O	7.138731	−14.706549	−8.198593
H	6.264443	−10.927852	−11.719570
H	8.382205	−11.673816	−11.285764
H	7.345452	−13.076583	−11.661760
H	8.623262	−13.590158	−9.726869
H	7.806616	−11.912767	−6.642345
H	5.854385	−13.847670	−9.587094
H	5.393321	−11.818133	−8.496821
H	5.617175	−12.831832	−7.068912
N	7.102986	−10.174479	−7.459861
C	6.319300	−9.359913	−8.280722
N	6.305421	−8.101571	−7.882211
C	7.123186	−8.068690	−6.750789
C	7.494713	−6.988554	−5.896675
O	7.131323	−5.788945	−5.972914
N	8.374837	−7.399272	−4.878719
C	8.861314	−8.682352	−4.722909
N	9.767443	−8.881153	−3.742154
N	8.494246	−9.702830	−5.506101
C	7.640598	−9.344820	−6.483826
H	5.829551	−9.756690	−9.158553
H	8.694060	−6.657012	−4.213377
H	9.974351	−8.144131	−3.051861
H	9.957947	−9.843617	−3.492156
P	6.334984	−15.471938	−6.975055
O	4.857637	−15.164170	−7.001458
O	6.838806	−16.897650	−7.009321
O	6.927782	−14.700652	−5.642592
C	8.344408	−14.847437	−5.355736
C	8.635542	−14.438114	−3.922810
O	8.484471	−12.992708	−3.765821
C	7.493115	−12.697643	−2.755939
C	7.725670	−15.094699	−2.861968
C	6.707448	−13.997146	−2.538462
O	8.581790	−15.460870	−1.741720
H	8.922451	−14.219263	−6.044405
H	8.642266	−15.893196	−5.495438
H	9.680508	−14.692602	−3.710430
H	7.985515	−12.361900	−1.841064
H	7.240774	−15.998431	−3.238915
H	5.877231	−14.083428	−3.241964
H	6.312089	−14.088852	−1.529622
N	6.687241	−11.549883	−3.201947
C	6.888908	−10.308198	−2.569638
O	7.689940	−10.139624	−1.649663
N	6.100286	−9.284188	−3.056898
C	5.144519	−9.370405	−4.061114
O	4.489474	−8.360421	−4.395135
C	4.981962	−10.679932	−4.670728
C	3.938978	−10.849175	−5.740496
C	5.777733	−11.689347	−4.231583
H	6.225146	−8.345211	−2.584124
H	2.959465	−10.522269	−5.371718
H	3.858638	−11.893082	−6.051192
H	4.168226	−10.235064	−6.619146
H	5.744071	−12.677111	−4.677811
P	7.990000	−15.958315	−0.280611
O	6.537019	−16.353374	−0.381235
O	9.027196	−16.915962	0.262812
O	8.015002	−14.558284	0.591251
C	9.309811	−13.960989	0.870210
C	9.162899	−12.840202	1.884410
O	8.451821	−11.713505	1.287052
C	7.273093	−11.395349	2.062139
C	8.397176	−13.213894	3.170430
C	6.999684	−12.624371	2.944383
O	9.090099	−12.580480	4.279862
H	9.733598	−13.560813	−0.058986
H	9.988534	−14.723356	1.270384
H	10.172445	−12.502622	2.151459
H	7.452155	−10.497010	2.653667
H	8.366063	−14.297683	3.321295
H	6.380566	−13.355413	2.418650
H	6.501775	−12.354335	3.879085
H	8.602865	−12.801369	5.094698
N	6.182207	−11.050089	1.138720
C	5.709611	−9.711088	1.063072
O	6.220458	−8.835664	1.799087
N	4.700014	−9.432467	0.197510
C	4.184033	−10.386594	−0.601952
N	3.195985	−10.037912	−1.440418
C	4.684772	−11.729374	−0.577540
C	5.684412	−12.007022	0.297134
H	2.861647	−9.061883	−1.481995
H	2.791896	−10.730158	−2.056990
H	4.291873	−12.488567	−1.242838
H	6.155431	−12.981172	0.363897

d(GAG)$^{4-}$

E: −27584.20 kcal mol^{-1}

O	−0.231513	−0.279187	−0.485744
C	−0.158932	0.161908	0.891865
C	1.262339	0.026165	1.424043
O	1.641826	−1.381406	1.574820
C	2.688955	−1.728481	0.631800
C	2.367163	0.659603	0.555188
C	2.851879	−0.517932	−0.294701
O	3.361695	1.194881	1.480259

H	-1.172074	-0.327084	-0.730613
H	-0.826264	-0.440022	1.521942
H	-0.457749	1.217256	0.974781
H	1.280935	0.475324	2.422931
H	3.603551	-1.950872	1.188711
H	1.994829	1.483297	-0.059681
H	2.190516	-0.605086	-1.158167
H	3.871888	-0.394242	-0.651839
N	2.348657	-2.964451	-0.062450
C	1.434428	-3.151694	-1.099490
N	1.395401	-4.402574	-1.521392
C	2.323982	-5.082028	-0.729427
C	2.734907	-6.446323	-0.730077
O	2.324664	-7.371578	-1.474125
N	3.720477	-6.701406	0.242751
C	4.214799	-5.768070	1.131705
N	5.100657	-6.205813	2.059814
N	3.833412	-4.488223	1.132994
C	2.915244	-4.204696	0.190884
H	0.820670	-2.337869	-1.459171
H	4.108933	-7.673835	0.261195
H	5.553448	-7.124643	1.942529
H	5.634103	-5.479235	2.524421
P	4.939858	1.478825	1.093608
O	5.117870	1.708021	-0.387983
O	5.408496	2.501084	2.104433
O	5.645086	0.019922	1.401422
C	5.737678	-0.438664	2.776568
C	6.981631	-1.294979	2.977872
O	6.846220	-2.617557	2.368160
C	7.713058	-2.742329	1.216614
C	8.268769	-0.690160	2.390478
C	8.386893	-1.373192	1.022724
O	9.348773	-1.018171	3.312467
H	4.842810	-1.021635	3.024610
H	5.797943	0.425840	3.447195
H	7.099581	-1.452590	4.055615
H	8.436820	-3.540985	1.403930
H	8.209677	0.396961	2.295477
H	7.846678	-0.773932	0.288765
H	9.421056	-1.470460	0.700039
N	6.940149	-3.185902	0.056707
C	6.064265	-2.436810	-0.719595
N	5.484136	-3.146770	-1.674175
C	5.993712	-4.434987	-1.519782
C	5.757280	-5.644962	-2.214710
N	4.908241	-5.763504	-3.247648
N	6.434507	-6.749746	-1.789495
C	7.270418	-6.659560	-0.737672
N	7.557298	-5.570806	-0.008241
C	6.894174	-4.482626	-0.443430
H	5.890481	-1.390758	-0.512197
H	4.766258	-6.678185	-3.694093
H	4.394243	-4.954260	-3.567340
H	7.760736	-7.589151	-0.458017
P	10.933668	-0.761719	2.924930
O	11.069120	0.311634	1.871777
O	11.648423	-0.650062	4.253627
O	11.346379	-2.172195	2.180300
C	11.340002	-3.387980	2.973182
C	11.871778	-4.544571	2.146036
O	10.899918	-4.902122	1.113593
C	11.574648	-4.992725	-0.164258
C	13.209704	-4.261288	1.423607
C	12.790234	-4.064062	-0.037916
O	14.053136	-5.431390	1.601967
H	10.317371	-3.607939	3.303439
H	11.975999	-3.256162	3.856738
H	12.000935	-5.405159	2.815394
H	11.873879	-6.028858	-0.357626
H	13.709151	-3.377731	1.831980
H	12.488184	-3.024087	-0.188728
H	13.584740	-4.313018	-0.745738
H	14.904152	-5.248570	1.163198
N	10.643243	-4.640441	-1.220720
C	10.116983	-3.379935	-1.502429
N	9.292309	-3.390151	-2.530674
C	9.262721	-4.719103	-2.957825
C	8.564981	-5.338477	-4.035848
O	7.782772	-4.798350	-4.855126
N	8.842824	-6.715705	-4.123225
C	9.691479	-7.408473	-3.284549
N	9.892281	-8.715454	-3.559559
N	10.333372	-6.833750	-2.259239
C	10.092989	-5.511838	-2.150742
H	10.384578	-2.514221	-0.915374
H	8.342711	-7.246700	-4.874432
H	9.324948	-9.204462	-4.270036
H	10.314901	-9.271884	-2.826694
O	6.272935	-11.638897	-10.982451
C	7.492591	-12.418702	-11.061631
C	7.891423	-12.917430	-9.678757
O	8.373568	-11.813692	-8.842954
C	7.515506	-11.633596	-7.688174
C	6.764965	-13.600134	-8.875574
C	6.271048	-12.491456	-7.944023
O	7.363865	-14.732847	-8.179963
H	6.138542	-11.210781	-11.846039
H	8.310566	-11.809872	-11.466907
H	7.346931	-13.291088	-11.714929
H	8.723593	-13.618122	-9.807811
H	8.049404	-11.936441	-6.786714
H	5.966366	-13.975388	-9.520444
H	5.499111	-11.920735	-8.463152
H	5.845073	-12.887214	-7.025858
N	7.233104	-10.198454	-7.508870
C	7.791409	-9.501011	-6.400420
O	8.542137	-10.112243	-5.608143
N	7.474528	-8.189728	-6.237203
C	6.681444	-7.548661	-7.118695
N	6.385888	-6.262615	-6.880075
C	6.175605	-8.213825	-8.285129
C	6.484921	-9.525686	-8.442789
H	6.825842	-5.757680	-6.095657
H	5.830806	-5.744817	-7.549030
H	5.581663	-7.686160	-9.022355
H	6.181917	-10.102161	-9.310858
P	6.587847	-15.539302	-6.962965
O	5.098586	-15.299311	-7.007282
O	7.158101	-16.940045	-6.994285
O	7.129005	-14.745689	-5.621666
C	8.542347	-14.851060	-5.302541
C	8.796755	-14.414745	-3.870218
O	8.626854	-12.969671	-3.741054
C	7.630413	-12.666905	-2.739216
C	7.875777	-15.064411	-2.814068
C	6.848682	-13.968091	-2.515168
O	8.721260	-15.413935	-1.680019
H	9.118871	-14.217218	-5.987454
H	8.869047	-15.890663	-5.421646
H	9.840129	-14.654281	-3.632820
H	8.116153	-12.320765	-1.824456
H	7.400046	-15.974782	-3.186544
H	6.027560	-14.065701	-3.228205
H	6.440615	-14.050771	-1.510526
N	6.822458	-11.526932	-3.199882
C	7.004778	-10.280174	-2.570608
O	7.790853	-10.101761	-1.639008

N	6.210322	−9.267091	−3.069695
C	5.261143	−9.368438	−4.078160
O	4.582628	−8.373526	−4.410005
C	5.127467	−10.679116	−4.692917
C	4.101997	−10.860816	−5.777810
C	5.930150	−11.677945	−4.242103
H	6.304357	−8.329823	−2.586968
H	3.113366	−10.545068	−5.423884
H	4.038179	−11.905469	−6.089594
H	4.336042	−10.244260	−6.653673
H	5.914926	−12.666793	−4.686823
P	8.120737	−15.897951	−0.218632
O	6.667619	−16.291762	−0.323122
O	9.153316	−16.852093	0.339664
O	8.143363	−14.490266	0.640962
C	9.436401	−13.889073	0.919405
C	9.288062	−12.774565	1.940648
O	8.574127	−11.644178	1.352886
C	7.384312	−11.346562	2.119409
C	8.523578	−13.158470	3.223798
C	7.120791	−12.582681	2.994912
O	9.206927	−12.519482	4.335933
H	9.855620	−13.482049	−0.008850
H	10.119311	−14.650788	1.313532
H	10.296990	−12.436081	2.208453
H	7.545509	−10.447890	2.715349
H	8.503444	−14.242816	3.372744
H	6.511190	−13.317332	2.463198
H	6.616629	−12.322045	3.928936
H	8.717968	−12.743793	5.148800
N	6.295564	−11.013683	1.188340
C	5.793708	−9.685046	1.124452
O	6.275480	−8.808120	1.878675
N	4.789912	−9.416878	0.249113
C	4.305490	−10.371634	−0.569308
N	3.323366	−10.033064	−1.418796
C	4.831390	−11.704826	−0.551318
C	5.826628	−11.971952	0.331447
H	2.974018	−9.062203	−1.458704
H	2.945733	−10.725139	−2.052082
H	4.460953	−12.464478	−1.228867
H	6.316650	−12.937160	0.393953

d(GCA)$^{4-}$

E: −27584.38 kcal mol^{-1}

O	−0.334608	−0.319351	−0.308108
C	−0.239029	0.131570	1.065129
C	1.200014	0.042669	1.557563
O	1.620190	−1.349719	1.742338
C	2.637238	−1.711489	0.774972
C	2.255185	0.674991	0.630696
C	2.763025	−0.517702	−0.184402
O	3.261147	1.278818	1.495465
H	−1.279254	−0.382116	−0.533081
H	−0.872002	−0.484610	1.716327
H	−0.564979	1.178714	1.148611
H	1.239633	0.523270	2.540900
H	3.570569	−1.921539	1.306416
H	1.834537	1.455478	−0.008479
H	2.107258	−0.648188	−1.046683
H	3.778607	−0.370475	−0.544065
N	2.273893	−2.962321	0.113845
C	1.292043	−3.157614	−0.859857
N	1.223310	−4.411605	−1.266230
C	2.197446	−5.087005	−0.528219
C	2.604784	−6.452438	−0.550637
O	2.152661	−7.377128	−1.271769
N	3.639336	−6.711117	0.366051
C	4.201056	−5.773729	1.210972
N	5.140217	−6.212058	2.074032
N	3.838603	−4.485666	1.216581
C	2.854213	−4.203909	0.342139
H	0.655206	−2.345144	−1.180788
H	3.994061	−7.694921	0.401372
H	5.470166	−7.187158	2.064505
H	5.686102	−5.509750	2.556363
P	4.739041	1.775790	0.951265
O	4.734410	1.980040	−0.544149
O	5.153477	2.884482	1.892604
O	5.671511	0.440775	1.217028
C	5.901730	0.033990	2.592768
C	7.057926	−0.949502	2.672734
O	6.685577	−2.237115	2.087508
C	7.555405	−2.549412	0.969910
C	8.348715	−0.507012	1.952608
C	8.263225	−1.238706	0.610738
O	9.458507	−0.933757	2.797552
H	4.993459	−0.435215	2.990389
H	6.140411	0.914667	3.200368
H	7.272612	−1.114735	3.734942
H	8.259602	−3.330632	1.258233
H	8.404663	0.577395	1.827139
H	7.654880	−0.634804	−0.065917
H	9.236134	−1.392854	0.150642
N	6.750111	−3.132070	−0.111191
C	6.878511	−4.516880	−0.414126
O	7.678915	−5.213445	0.242707
N	6.117464	−5.028166	−1.419182
C	5.246864	−4.256643	−2.098620
N	4.538738	−4.823593	−3.085683
C	5.072487	−2.870757	−1.774167
C	5.835153	−2.361553	−0.773678
H	4.666238	−5.820346	−3.325604
H	3.853764	−4.276468	−3.589802
H	4.347924	−2.257807	−2.296277
H	5.750158	−1.335899	−0.433902
P	11.025834	−0.997393	2.283307
O	11.234922	−0.154639	1.048430
O	11.858201	−0.783209	3.528070
O	11.166251	−2.560711	1.780062
C	11.139384	−3.625679	2.766629
C	11.922725	−4.835435	2.272312
O	11.201479	−5.552430	1.223092
C	11.865894	−5.398970	−0.051364
C	13.305099	−4.510556	1.683250
C	13.036212	−4.422001	0.174584
O	14.186839	−5.614758	2.021640
H	10.099193	−3.916222	2.956628
H	11.588878	−3.270376	3.700731
H	12.027434	−5.526042	3.117346
H	12.201900	−6.383363	−0.389090
H	13.707319	−3.573999	2.083271
H	12.742990	−3.401866	−0.080161
H	13.910744	−4.693976	−0.421196
H	15.053913	−5.432949	1.615209
N	10.903260	−4.949603	−1.055253
C	10.322980	−3.693320	−1.167092
N	9.421201	−3.623402	−2.132604
C	9.390302	−4.904326	−2.681338
C	8.635857	−5.473389	−3.733738
N	7.733743	−4.795139	−4.465403
N	8.858308	−6.787329	−4.019460
C	9.768709	−7.483493	−3.313071
N	10.535793	−7.043079	−2.302936
C	10.302006	−5.745228	−2.022570

H	10.599274	−2.895029	−0.493671
H	7.192795	−5.279372	−5.192458
H	7.503425	−3.843299	−4.214812
H	9.876153	−8.527329	−3.602120
O	6.089728	−11.506257	−10.806404
C	7.302948	−12.284906	−10.958951
C	7.780720	−12.796300	−9.605833
O	8.302736	−11.699834	−8.784989
C	7.504476	−11.539530	−7.584088
C	6.709876	−13.494838	−8.742922
C	6.243972	−12.386083	−7.796563
O	7.377689	−14.602782	−8.069504
H	5.909944	−11.069955	−11.657372
H	8.097381	−11.672069	−11.403347
H	7.122170	−13.151705	−11.611048
H	8.607949	−13.490420	−9.790354
H	8.081243	−11.868757	−6.718212
H	5.888844	−13.896429	−9.342157
H	5.462857	−11.809871	−8.295949
H	5.838041	−12.775767	−6.866402
N	7.245928	−10.109469	−7.360859
C	7.877976	−9.468205	−6.283234
O	8.691097	−10.027107	−5.540697
N	7.511745	−8.148333	−6.114672
C	6.660108	−7.403697	−6.920519
O	6.431906	−6.201821	−6.650265
C	6.106167	−8.102364	−8.068061
C	5.219978	−7.356299	−9.028647
C	6.433313	−9.410406	−8.233159
H	7.981961	−7.648974	−5.313581
H	5.746674	−6.496602	−9.461607
H	4.331541	−6.964374	−8.517913
H	4.892902	−8.010430	−9.842182
H	6.090039	−9.982458	−9.089532
P	6.690509	−15.442181	−6.823254
O	5.196934	−15.228076	−6.779390
O	7.280269	−16.832585	−6.910056
O	7.293039	−14.662993	−5.500158
C	8.709850	−14.797258	−5.208284
C	8.999648	−14.457373	−3.753104
O	8.894638	−13.019201	−3.508813
C	7.792724	−12.731853	−2.615545
C	8.067856	−15.131978	−2.727962
C	7.020193	−14.050197	−2.451406
O	8.891293	−15.475372	−1.574842
H	9.281113	−14.125308	−5.860620
H	9.024701	−15.829838	−5.398142
H	10.037872	−14.745141	−3.553073
H	8.190457	−12.360659	−1.665495
H	7.614983	−16.047006	−3.117961
H	6.239671	−14.132981	−3.209934
H	6.561216	−14.154097	−1.470267
N	6.985982	−11.643505	−3.152916
C	6.078952	−11.701419	−4.209982
N	5.473162	−10.548441	−4.423207
C	6.001556	−9.677847	−3.466979
C	5.703026	−8.313846	−3.176375
O	4.894947	−7.561902	−3.771583
N	6.432897	−7.837143	−2.071894
C	7.358807	−8.571074	−1.355503
N	7.953683	−7.957733	−0.316324
N	7.657431	−9.844310	−1.641746
C	6.951141	−10.340002	−2.674787
H	5.937466	−12.612491	−4.772489
H	6.292402	−6.830813	−1.816534
H	7.839597	−6.947904	−0.132320
H	8.681352	−8.470590	0.163317
P	8.257200	−15.930538	−0.118888
O	6.829999	−16.399708	−0.263408
O	9.311524	−16.811791	0.513111
O	8.177636	−14.491957	0.687346
C	9.429044	−13.832549	1.020803
C	9.199260	−12.762059	2.074735
O	8.458425	−11.638747	1.504625
C	7.247037	−11.405738	2.257427
C	8.409351	−13.226087	3.315632
C	6.994388	−12.691581	3.060661
O	9.031051	−12.607647	4.474727
H	9.850776	−13.375119	0.117433
H	10.140377	−14.570183	1.410024
H	10.183637	−12.393051	2.389470
H	7.369209	−10.535191	2.902647
H	8.425778	−14.315560	3.421136
H	6.434544	−13.417182	2.464571
H	6.445027	−12.497431	3.985373
H	8.528995	−12.888513	5.261803
N	6.175695	−11.052750	1.312345
C	5.573208	−9.768073	1.363761
O	5.914370	−8.960841	2.259896
N	4.633343	−9.460723	0.431560
C	4.263310	−10.354280	−0.508233
N	3.321824	−9.984697	−1.389278
C	4.858285	−11.656485	−0.571749
C	5.813656	−11.951669	0.344723
H	2.921843	−9.034218	−1.365575
H	3.076206	−10.604318	−2.150315
H	4.573165	−12.367782	−1.337203
H	6.354026	−12.891757	0.354594

d(GCG)$^{4-}$

E: −27493.59 kcal mol^{-1}

O	−0.375351	−0.335164	−0.215527
C	−0.264126	0.081567	1.167072
C	1.183367	−0.001746	1.635025
O	1.625518	−1.393028	1.764686
C	2.630510	−1.705439	0.767306
C	2.217509	0.677860	0.717048
C	2.724647	−0.478286	−0.150770
O	3.226146	1.264100	1.591395
H	−1.322361	−0.416035	−0.424167
H	−0.878079	−0.560306	1.811665
H	−0.602857	1.121565	1.283692
H	1.230451	0.445059	2.633889
H	3.574823	−1.919690	1.276909
H	1.778417	1.476907	0.113999
H	2.051861	−0.583940	−1.003198
H	3.731582	−0.308597	−0.525752
N	2.276586	−2.938285	0.069784
C	1.303213	−3.119984	−0.915159
N	1.245090	−4.366346	−1.346276
C	2.217435	−5.050722	−0.613757
C	2.627813	−6.414923	−0.650958
O	2.181879	−7.331258	−1.386516
N	3.655491	−6.683885	0.270882
C	4.210122	−5.756225	1.131212
N	5.148525	−6.200066	1.991803
N	3.840928	−4.470604	1.154396
C	2.862272	−4.180396	0.277450
H	0.663990	−2.306043	−1.226748
H	4.006684	−7.669221	0.301033
H	5.479958	−7.174577	1.975739
H	5.686314	−5.499958	2.486518
P	4.726013	1.721715	1.075211
O	4.742171	1.983008	−0.411350
O	5.173337	2.777443	2.060948

O	5.602653	0.342316	1.294838
C	5.825431	−0.121580	2.653809
C	7.019972	−1.061209	2.703364
O	6.699976	−2.344121	2.081378
C	7.566293	−2.583313	0.943038
C	8.284498	−0.537093	1.991321
C	8.233023	−1.238677	0.630931
O	9.419065	−0.917343	2.824728
H	4.929814	−0.644138	3.011524
H	6.020015	0.736969	3.306751
H	7.246750	−1.249961	3.759137
H	8.295203	−3.355061	1.193008
H	8.280081	0.551186	1.889211
H	7.613544	−0.635711	−0.035964
H	9.214927	−1.348207	0.177581
N	6.767515	−3.144948	−0.154753
C	6.905445	−4.522358	−0.489492
O	7.705524	−5.229450	0.157042
N	6.151072	−5.016107	−1.507587
C	5.281569	−4.234216	−2.175608
N	4.581073	−4.781467	−3.178956
C	5.089245	−2.859653	−1.813337
C	5.842095	−2.368570	−0.796960
H	4.712627	−5.771390	−3.441835
H	3.893456	−4.226491	−3.670565
H	4.357329	−2.241479	−2.319172
H	5.739442	−1.354413	−0.428794
P	10.984668	−0.888200	2.302254
O	11.141510	−0.023880	1.074490
O	11.811191	−0.637877	3.544667
O	11.205031	−2.437372	1.787612
C	11.211616	−3.505250	2.771487
C	11.978589	−4.710135	2.244190
O	11.226585	−5.403521	1.201752
C	11.883411	−5.262388	−0.081008
C	13.348993	−4.380375	1.628661
C	13.045844	−4.274136	0.128755
O	14.235998	−5.490712	1.933637
H	10.178651	−3.797975	2.995679
H	11.691109	−3.152820	3.691955
H	12.101552	−5.412372	3.077455
H	12.229714	−6.248079	−0.404753
H	13.762085	−3.449977	2.032078
H	12.732420	−3.254676	−0.103584
H	13.909186	−4.526906	−0.491583
H	15.092849	−5.307063	1.506798
N	10.915672	−4.838526	−1.085104
C	10.326131	−3.583173	−1.226827
N	9.443943	−3.542963	−2.206647
C	9.432632	−4.834879	−2.737526
C	8.680458	−5.401822	−3.807253
O	7.834384	−4.834708	−4.540090
N	8.985648	−6.764744	−4.001501
C	9.915494	−7.482576	−3.276576
N	10.118366	−8.768576	−3.640647
N	10.620743	−6.952911	−2.271535
C	10.338574	−5.654344	−2.046915
H	10.587535	−2.770747	−0.564901
H	8.466690	−7.255162	−4.768743
H	9.503049	−9.238245	−4.325299
H	10.642177	−9.343879	−2.992972
O	6.182471	−11.459091	−10.951157
C	7.376060	−12.274137	−11.059834
C	7.793750	−12.791843	−9.689496
O	8.307743	−11.704985	−8.852853
C	7.467210	−11.524636	−7.681113
C	6.678872	−13.466924	−8.864022
C	6.198858	−12.344634	−7.940430
O	7.302937	−14.585910	−8.166399

H	6.046930	−11.017791	−11.807818
H	8.202821	−11.687372	−11.479981
H	7.191701	−13.138620	−11.714285
H	8.612192	−13.503774	−9.843859
H	8.004358	−11.866050	−6.795814
H	5.873110	−13.856771	−9.491466
H	5.450661	−11.756195	−8.475064
H	5.747012	−12.720545	−7.025519
N	7.235803	−10.088973	−7.467411
C	7.865738	−9.432978	−6.372972
O	8.647769	−10.080743	−5.642426
N	7.592222	−8.118796	−6.164183
C	6.778026	−7.436281	−6.993706
N	6.531346	−6.147621	−6.715302
C	6.208311	−8.058022	−8.154575
C	6.469065	−9.375262	−8.352408
H	6.951014	−5.695165	−5.888433
H	5.930606	−5.608611	−7.324924
H	5.601242	−7.495883	−8.854965
H	6.113279	−9.921860	−9.219355
P	6.594443	−15.384230	−6.906709
O	5.105484	−15.138628	−6.868242
O	7.153823	−16.788644	−6.965465
O	7.210679	−14.592922	−5.598475
C	8.623497	−14.746953	−5.298632
C	8.902947	−14.450987	−3.831479
O	8.814149	−13.020647	−3.544117
C	7.679544	−12.740812	−2.690093
C	7.951440	−15.140854	−2.835486
C	6.902020	−14.059801	−2.561145
O	8.755854	−15.509865	−1.676529
H	9.204786	−14.060340	−5.926192
H	8.932009	−15.776037	−5.515498
H	9.935657	−14.756457	−3.629410
H	8.046212	−12.375329	−1.725835
H	7.502859	−16.047805	−3.249061
H	6.136597	−14.129809	−3.335855
H	6.423544	−14.177825	−1.590979
N	6.890022	−11.647820	−3.243205
C	6.004893	−11.687650	−4.319938
N	5.427801	−10.522039	−4.545347
C	5.956394	−9.660898	−3.580820
C	5.691919	−8.287143	−3.304055
O	4.905586	−7.518489	−3.906860
N	6.432772	−7.818916	−2.201401
C	7.318841	−8.578078	−1.459408
N	7.932096	−7.969608	−0.428256
N	7.569185	−9.865496	−1.720578
C	6.872919	−10.343500	−2.767218
H	5.859024	−12.594558	−4.888149
H	6.311123	−6.809533	−1.945545
H	7.835737	−6.958342	−0.240234
H	8.617510	−8.510149	0.081926
P	8.114365	−15.907038	−0.208744
O	6.672427	−16.336086	−0.331952
O	9.144590	−16.803931	0.440792
O	8.081964	−14.447592	0.560629
C	9.351108	−13.811751	0.871583
C	9.161986	−12.758810	1.951321
O	8.425869	−11.613329	1.421620
C	7.211394	−11.403492	2.177074
C	8.390954	−13.236856	3.197916
C	6.972495	−12.698358	2.970608
O	9.030756	−12.633307	4.354961
H	9.753440	−13.342330	−0.034433
H	10.062540	−14.566000	1.227146
H	10.158089	−12.409524	2.250943
H	7.326194	−10.537427	2.829179
H	8.407593	−14.327602	3.289835

H	6.402049	−13.418788	2.379050
H	6.439247	−12.512131	3.906294
H	8.538214	−12.920484	5.145662
N	6.134881	−11.050917	1.237405
C	5.554020	−9.755904	1.276175
O	5.911184	−8.945539	2.163365
N	4.617810	−9.441777	0.342778
C	4.232023	−10.338098	−0.587928
N	3.298138	−9.960164	−1.473619
C	4.803401	−11.651169	−0.637815
C	5.757419	−11.952662	0.278037
H	2.918436	−9.000988	−1.461722
H	3.042719	−10.582423	−2.229181
H	4.505807	−12.364589	−1.396514
H	6.284909	−12.900081	0.291972

d(GGA)$^{4-}$

E: −27584.73 kcal mol^{-1}

O	−0.307332	−0.297676	−0.395344
C	−0.229950	0.154719	0.978436
C	1.203019	0.071643	1.489640
O	1.630348	−1.318925	1.666599
C	2.661313	−1.663324	0.704771
C	2.268767	0.719775	0.585447
C	2.786667	−0.460829	−0.241072
O	3.259916	1.314933	1.474394
H	−1.248986	−0.374910	−0.627983
H	−0.867879	−0.464151	1.622226
H	−0.561773	1.200435	1.057900
H	1.224814	0.543529	2.477884
H	3.590475	−1.868062	1.245237
H	1.855567	1.509930	−0.046991
H	2.133815	−0.582133	−1.106675
H	3.803291	−0.310084	−0.596321
N	2.318823	−2.911646	0.030717
C	1.389349	−3.109271	−0.991117
N	1.330810	−4.366809	−1.388651
C	2.260426	−5.041522	−0.594570
C	2.650066	−6.410917	−0.573264
O	2.218924	−7.344532	−1.295941
N	3.639202	−6.664147	0.395935
C	4.157005	−5.721887	1.262849
N	5.043136	−6.157979	2.186926
N	3.798182	−4.434924	1.239716
C	2.874422	−4.154227	0.301831
H	0.777712	−2.296786	−1.356680
H	4.004091	−7.644519	0.439996
H	5.412191	−7.119043	2.156650
H	5.587438	−5.445858	2.658175
P	4.774822	1.752398	0.984492
O	4.826555	1.982504	−0.506714
O	5.204090	2.829087	1.956233
O	5.639463	0.374600	1.255853
C	5.822297	−0.058207	2.630428
C	6.990171	−1.027100	2.742515
O	6.660877	−2.326149	2.155840
C	7.518010	−2.594592	1.017656
C	8.289456	−0.566458	2.052396
C	8.217379	−1.269295	0.695098
O	9.388575	−1.007686	2.904727
H	4.906316	−0.548051	2.983143
H	6.024535	0.812861	3.264669
H	7.177489	−1.187029	3.810499
H	8.229080	−3.384425	1.279158
H	8.345239	0.520524	1.950394
H	7.594873	−0.661186	0.035608
H	9.191664	−1.401730	0.230324
N	6.726030	−3.130828	−0.077494
C	5.834502	−2.448142	−0.903136
N	5.274756	−3.232301	−1.804918
C	5.814698	−4.499477	−1.569141
C	5.609547	−5.745087	−2.229991
O	4.850381	−5.985060	−3.199488
N	6.393844	−6.774127	−1.672908
C	7.262505	−6.623793	−0.608060
N	7.923793	−7.724020	−0.194205
N	7.444223	−5.458774	0.022049
C	6.716700	−4.452986	−0.494125
H	5.647712	−1.392423	−0.771652
H	6.351488	−7.703339	−2.155825
H	7.956016	−8.577056	−0.772380
H	8.661321	−7.571068	0.482327
P	10.952410	−1.143005	2.396112
O	11.213920	−0.291037	1.177507
O	11.785621	−0.993472	3.649530
O	11.015275	−2.702374	1.864825
C	10.929799	−3.782537	2.831376
C	11.701013	−5.001681	2.341213
O	11.005685	−5.674849	1.245654
C	11.729596	−5.508435	0.005439
C	13.113499	−4.696107	1.816067
C	12.905617	−4.559046	0.301263
O	13.953687	−5.831915	2.155404
H	9.877157	−4.050980	2.980963
H	11.355716	−3.454569	3.786247
H	11.754615	−5.714012	3.172882
H	12.063479	−6.492051	−0.336710
H	13.522709	−3.781733	2.258194
H	12.638771	−3.527094	0.066055
H	13.797728	−4.829538	−0.268667
H	14.839657	−5.662476	1.785879
N	10.820328	−5.024299	−1.031412
C	10.270674	−3.754101	−1.156726
N	9.413010	−3.658265	−2.159162
C	9.378325	−4.934273	−2.720243
C	8.646865	−5.487231	−3.797335
N	7.784550	−4.788394	−4.561186
N	8.842171	−6.807453	−4.071824
C	9.705176	−7.527441	−3.331066
N	10.450210	−7.103381	−2.297859
C	10.244263	−5.799021	−2.029518
H	10.535266	−2.965003	−0.467555
H	7.213284	−5.284400	−5.255390
H	7.524903	−3.851613	−4.280412
H	9.789158	−8.576057	−3.608131
O	6.016675	−11.571034	−10.808395
C	7.224857	−12.351110	−10.991893
C	7.736344	−12.858440	−9.649604
O	8.281235	−11.758528	−8.847399
C	7.517449	−11.589935	−7.628511
C	6.684085	−13.549823	−8.758868
C	6.250747	−12.439897	−7.798382
O	7.357678	−14.662009	−8.099968
H	5.811259	−11.140191	−11.656340
H	8.007524	−11.740463	−11.459347
H	7.025578	−13.219768	−11.635771
H	8.557415	−13.554973	−9.850745
H	8.117973	−11.909469	−6.774951
H	5.844251	−13.943343	−9.336684
H	5.451364	−11.864352	−8.269006
H	5.879070	−12.835385	−6.856500
N	7.260349	−10.156523	−7.409912
C	7.901258	−9.507529	−6.341322
O	8.721516	−10.061711	−5.602698
N	7.522595	−8.192072	−6.166808

C	6.640667	−7.460169	−6.953373
O	6.388604	−6.268122	−6.667166
C	6.077548	−8.165311	−8.092949
C	5.157858	−7.432156	−9.031288
C	6.421518	−9.467707	−8.266934
H	7.985668	−7.686298	−5.363444
H	5.660240	−6.562273	−9.472253
H	4.274556	−7.057213	−8.499426
H	4.824360	−8.089279	−9.839561
H	6.069289	−10.045055	−9.116215
P	6.658969	−15.525740	−6.875714
O	5.168307	−15.293967	−6.828514
O	7.234674	−16.919309	−6.992875
O	7.269126	−14.784461	−5.532674
C	8.692452	−14.917559	−5.270918
C	9.022759	−14.463450	−3.858179
O	8.876160	−13.013892	−3.737513
C	7.886630	−12.687211	−2.734254
C	8.153419	−15.082103	−2.743110
C	7.122352	−13.988317	−2.452949
O	9.043328	−15.361226	−1.622738
H	9.253231	−14.307481	−5.989788
H	8.989755	−15.966089	−5.387799
H	10.075118	−14.709454	−3.674458
H	8.380661	−12.298406	−1.842579
H	7.680744	−16.016423	−3.055414
H	6.286285	−14.113562	−3.144067
H	6.737797	−14.040818	−1.436512
N	7.045699	−11.584381	−3.223361
C	7.172057	−10.287497	−2.644199
O	8.033849	−10.092946	−1.762738
N	6.320204	−9.314554	−3.061611
C	5.426178	−9.540018	−4.042672
N	4.614636	−8.535034	−4.399736
C	5.346327	−10.814220	−4.696345
C	6.175810	−11.795133	−4.257389
H	4.693688	−7.606380	−3.950975
H	3.934578	−8.675256	−5.135017
H	4.658332	−10.984845	−5.515474
H	6.206599	−12.782013	−4.704672
P	8.478907	−15.805042	−0.133339
O	7.065249	−16.326486	−0.222485
O	9.585344	−16.634632	0.478293
O	8.372501	−14.354803	0.647840
C	9.607742	−13.659448	0.968769
C	9.358552	−12.597274	2.027371
O	8.598599	−11.484478	1.461622
C	7.388691	−11.266206	2.219859
C	8.576802	−13.079444	3.266983
C	7.155172	−12.557828	3.020710
O	9.195689	−12.465200	4.429702
H	10.006343	−13.188398	0.061911
H	10.343645	−14.376998	1.349258
H	10.336078	−12.212096	2.343859
H	7.500911	−10.394521	2.865583
H	8.604335	−14.169507	3.363065
H	6.599704	−13.286259	2.423691
H	6.608022	−12.373710	3.948780
H	8.702080	−12.762692	5.215989
N	6.309618	−10.927955	1.277430
C	5.654302	−9.670097	1.357365
O	5.950323	−8.877459	2.281109
N	4.710081	−9.377526	0.424255
C	4.366303	−10.270822	−0.525534
N	3.407780	−9.925323	−1.397523
C	5.001787	−11.553084	−0.605890
C	5.973760	−11.825788	0.300113
H	2.987691	−8.982766	−1.374293
H	3.159837	−10.557898	−2.146581
H	4.731467	−12.266854	−1.374245
H	6.544769	−12.747423	0.297594

d(GGC)$^{4-}$

E: −27497.01 kcal mol^{-1}

O	−0.396548	−0.280231	−0.184240
C	−0.278544	0.138776	1.197077
C	1.168462	0.039057	1.663394
O	1.595533	−1.357609	1.782141
C	2.600730	−1.669538	0.783120
C	2.209629	0.714283	0.749316
C	2.696885	−0.440529	−0.130278
O	3.227133	1.281039	1.627666
H	−1.344628	−0.357909	−0.388964
H	−0.898639	−0.494815	1.844063
H	−0.605500	1.182801	1.312048
H	1.220641	0.478981	2.665141
H	3.544312	−1.885136	1.293128
H	1.779741	1.525582	0.155854
H	2.011918	−0.534588	−0.974121
H	3.700021	−0.280845	−0.519175
N	2.247888	−2.899005	0.081728
C	1.305284	−3.073022	−0.932742
N	1.260591	−4.315546	−1.376521
C	2.213760	−5.004233	−0.622980
C	2.632023	−6.364706	−0.665468
O	2.208935	−7.275349	−1.421316
N	3.641011	−6.637417	0.277790
C	4.157431	−5.719010	1.171250
N	5.071526	−6.171168	2.058793
N	3.773299	−4.439464	1.206088
C	2.827401	−4.141304	0.296543
H	0.676032	−2.257343	−1.259201
H	4.017374	−7.614824	0.283887
H	5.460607	−7.122535	1.983333
H	5.605013	−5.466115	2.552635
P	4.753705	1.671809	1.137780
O	4.811729	1.921655	−0.349985
O	5.223957	2.716726	2.124553
O	5.569516	0.259286	1.384487
C	5.770957	−0.191510	2.751142
C	7.000865	−1.083567	2.848541
O	6.754136	−2.404131	2.269427
C	7.559672	−2.597737	1.083294
C	8.249589	−0.532132	2.134550
C	8.207948	−1.238903	0.776975
O	9.398502	−0.884104	2.959599
H	4.886222	−0.747250	3.084792
H	5.913646	0.676646	3.404575
H	7.215363	−1.232499	3.912564
H	8.300627	−3.379372	1.278939
H	8.218301	0.555177	2.027630
H	7.571549	−0.653608	0.111073
H	9.192841	−1.328608	0.323890
N	6.732091	−3.109440	−0.002212
C	5.799198	−2.407529	−0.767261
N	5.237137	−3.158252	−1.695153
C	5.812384	−4.421447	−1.540184
C	5.627494	−5.631690	−2.270691
O	4.861503	−5.831943	−3.243723
N	6.437812	−6.673702	−1.782087
C	7.332980	−6.560023	−0.733733
N	8.044446	−7.654809	−0.417044
N	7.511289	−5.423026	−0.050450
C	6.742565	−4.409829	−0.488141
H	5.584508	−1.367678	−0.568161

H	6.361249	−7.596365	−2.271921
H	7.932794	−8.553881	−0.910381
H	8.696532	−7.578729	0.351603
P	10.956068	−0.803842	2.417299
O	11.073274	0.089728	1.206483
O	11.793485	−0.562354	3.653492
O	11.206997	−2.338220	1.864987
C	11.203970	−3.423057	2.831462
C	11.854225	−4.660496	2.234873
O	11.004229	−5.230735	1.191753
C	11.711948	−5.270328	−0.068266
C	13.235697	−4.425454	1.592571
C	12.924818	−4.339367	0.093246
O	14.059953	−5.577575	1.917945
H	10.170241	−3.651653	3.118047
H	11.761595	−3.120915	3.725400
H	11.947019	−5.403521	3.036893
H	12.006506	−6.295198	−0.294882
H	13.706707	−3.511772	1.969123
H	12.662546	−3.310059	−0.164437
H	13.766944	−4.652685	−0.528926
H	14.927708	−5.447243	1.493301
N	10.780678	−4.880205	−1.139277
C	10.406651	−5.820885	−2.134632
O	10.924345	−6.962311	−2.129710
N	9.496076	−5.441897	−3.069483
C	8.969550	−4.200247	−3.067475
N	8.085127	−3.891597	−4.027313
C	9.335815	−3.239000	−2.070167
C	10.226647	−3.628087	−1.124252
H	7.793041	−4.597503	−4.720744
H	7.621172	−2.992781	−4.012060
H	8.897897	−2.248528	−2.058195
H	10.533190	−2.991115	−0.301910
O	6.191485	−11.425085	−10.929954
C	7.336390	−12.301435	−11.066957
C	7.722240	−12.892325	−9.716438
O	8.297432	−11.874093	−8.832372
C	7.414773	−11.612143	−7.712666
C	6.569743	−13.530032	−8.917171
C	6.124784	−12.398730	−7.986265
O	7.132018	−14.685439	−8.227905
H	6.081201	−10.949815	−11.771870
H	8.196794	−11.748557	−11.465054
H	7.105950	−13.131069	−11.751442
H	8.502238	−13.639673	−9.896536
H	7.912472	−11.932310	−6.792149
H	5.755504	−13.873684	−9.560498
H	5.403310	−11.779420	−8.521593
H	5.653672	−12.769097	−7.078399
N	7.208575	−10.174664	−7.564309
C	6.400104	−9.337264	−8.336219
N	6.423668	−8.085010	−7.919038
C	7.289947	−8.082224	−6.824142
C	7.693173	−7.027596	−5.954210
O	7.334629	−5.823991	−5.990903
N	8.593683	−7.469536	−4.968687
C	9.057517	−8.764679	−4.843800
N	9.968895	−9.000513	−3.877294
N	8.662821	−9.761457	−5.643868
C	7.795021	−9.370486	−6.595652
H	5.864858	−9.713673	−9.196578
H	8.926593	−6.748420	−4.286797
H	10.234608	−8.271048	−3.200249
H	10.156425	−9.969671	−3.653784
P	6.380345	−15.452496	−6.973972
O	4.901462	−15.151295	−6.950404
O	6.890352	−16.875298	−7.020224
O	7.013931	−14.675798	−5.662919
C	8.428378	−14.854399	−5.380711
C	8.735491	−14.462415	−3.944542
O	8.639961	−13.013716	−3.772679
C	7.616344	−12.685241	−2.804344
C	7.803728	−15.088542	−2.885692
C	6.800806	−13.968249	−2.594037
O	8.641109	−15.445854	−1.747797
H	9.017474	−14.232725	−6.066050
H	8.701878	−15.905147	−5.530000
H	9.771333	−14.754779	−3.737310
H	8.081753	−12.336554	−1.881455
H	7.309799	−15.991730	−3.252491
H	5.985978	−14.042086	−3.317243
H	6.378897	−14.042611	−1.594222
N	6.836893	−11.539803	−3.300519
C	6.986321	−10.265347	−2.680203
O	7.784627	−10.139628	−1.729354
N	6.234625	−9.232913	−3.145295
C	5.383860	−9.395366	−4.177123
N	4.650419	−8.341565	−4.560104
C	5.266576	−10.655838	−4.851068
C	6.016492	−11.686832	−4.386218
H	4.737102	−7.431258	−4.078890
H	4.030799	−8.423227	−5.355403
H	4.614983	−10.776803	−5.707895
H	6.020416	−12.666136	−4.851219
P	8.020828	−15.896996	−0.283805
O	6.572051	−16.302561	−0.403500
O	9.050562	−16.830956	0.312008
O	8.019596	−14.469893	0.543763
C	9.303122	−13.868134	0.862815
C	9.133201	−12.794271	1.924486
O	8.442624	−11.634144	1.367022
C	7.232197	−11.363091	2.109238
C	8.331725	−13.225814	3.169163
C	6.938236	−12.634630	2.922335
O	8.984895	−12.633894	4.324867
H	9.731371	−13.423468	−0.043876
H	9.986420	−14.638867	1.237568
H	10.136490	−12.473493	2.232203
H	7.375676	−10.490430	2.746804
H	8.303436	−14.315083	3.274542
H	6.343573	−13.341682	2.338401
H	6.405989	−12.413147	3.850833
H	8.474141	−12.891442	5.114289
N	6.175046	−10.988409	1.156800
C	5.632965	−9.674978	1.171217
O	6.025782	−8.856517	2.034623
N	4.688899	−9.357794	0.246496
C	4.261580	−10.266019	−0.654015
N	3.312775	−9.891888	−1.524480
C	4.805297	−11.591728	−0.688993
C	5.764993	−11.897686	0.219496
H	2.936463	−8.930880	−1.513925
H	2.989474	−10.543692	−2.226876
H	4.476374	−12.314363	−1.425787
H	6.269164	−12.857270	0.247142

d(GGG)$^{4-}$

E: −27493.98 kcal mol^{-1}

O	−0.383675	−0.334767	−0.144807
C	−0.263466	0.080469	1.237346
C	1.189939	0.014630	1.690332
O	1.653293	−1.370417	1.804333
C	2.647320	−1.661475	0.787387
C	2.205237	0.716146	0.767569

C	2.713705	−0.425218	−0.118804
O	3.214649	1.310151	1.636917
H	−1.330901	−0.443094	−0.339352
H	−0.861840	−0.571142	1.886774
H	−0.614918	1.115579	1.360365
H	1.240436	0.454566	2.692150
H	3.600607	−1.867923	1.282811
H	1.749133	1.516009	0.178142
H	2.028948	−0.527818	−0.961655
H	3.712665	−0.243116	−0.508622
N	2.299820	−2.891700	0.083552
C	1.357458	−3.070808	−0.930168
N	1.302349	−4.318475	−1.358054
C	2.246968	−5.006104	−0.593169
C	2.646559	−6.372582	−0.610986
O	2.212557	−7.290985	−1.351437
N	3.648941	−6.642665	0.339697
C	4.173232	−5.717431	1.221140
N	5.075341	−6.168286	2.121571
N	3.806029	−4.432524	1.233535
C	2.867396	−4.136663	0.315773
H	0.735188	−2.254924	−1.268524
H	4.015899	−7.623329	0.359844
H	5.440749	−7.130166	2.072726
H	5.622347	−5.464209	2.601620
P	4.730789	1.727470	1.136134
O	4.770430	1.991987	−0.349731
O	5.193155	2.772314	2.127213
O	5.568955	0.326418	1.362799
C	5.779982	−0.137283	2.723205
C	6.995316	−1.050357	2.796630
O	6.719010	−2.354750	2.196630
C	7.541330	−2.553601	1.019248
C	8.250715	−0.510001	2.083652
C	8.185472	−1.195205	0.716393
O	9.394116	−0.893642	2.904011
H	4.890636	−0.681304	3.064472
H	5.944343	0.723390	3.381796
H	7.215491	−1.218382	3.856985
H	8.285571	−3.328010	1.229567
H	8.240379	0.579562	1.994733
H	7.534141	−0.598605	0.075230
H	9.158445	−1.281631	0.237634
N	6.729744	−3.078240	−0.068324
C	5.813127	−2.391461	−0.863980
N	5.249961	−3.164950	−1.772555
C	5.811387	−4.428742	−1.572832
C	5.613082	−5.662019	−2.257971
O	4.847801	−5.890476	−3.225872
N	6.407461	−6.696039	−1.724889
C	7.284074	−6.558353	−0.663777
N	7.952748	−7.660587	−0.275444
N	7.468723	−5.401477	−0.019355
C	6.728767	−4.392475	−0.510742
H	5.609361	−1.342524	−0.705343
H	6.353451	−7.623832	−2.208368
H	7.918481	−8.541520	−0.811100
H	8.673181	−7.533319	0.423025
P	10.947203	−0.945223	2.350083
O	11.129478	−0.077384	1.128105
O	11.807162	−0.751600	3.579729
O	11.075025	−2.498560	1.814972
C	11.061841	−3.575763	2.788975
C	11.825427	−4.781744	2.259109
O	11.076418	−5.461591	1.204782
C	11.755734	−5.330029	−0.066261
C	13.202464	−4.451844	1.657719
C	12.912900	−4.338772	0.155848
O	14.085697	−5.564244	1.965837
H	10.024021	−3.861509	2.998582
H	11.534265	−3.236206	3.717632
H	11.938914	−5.491159	3.087628
H	12.110194	−6.316765	−0.378419
H	13.612678	−3.523545	2.068723
H	12.596609	−3.319414	−0.073577
H	13.782413	−4.584993	−0.458618
H	14.947708	−5.377414	1.550965
N	10.804475	−4.915296	−1.090024
C	10.209255	−3.663418	−1.248821
N	9.363385	−3.627505	−2.260007
C	9.381772	−4.917941	−2.794890
C	8.674396	−5.489788	−3.892559
O	7.853533	−4.926659	−4.657516
N	8.985609	−6.852467	−4.069755
C	9.900098	−7.563248	−3.318841
N	10.136499	−8.842030	−3.686491
N	10.567210	−7.029092	−2.289881
C	10.269537	−5.732542	−2.074029
H	10.439659	−2.849910	−0.576179
H	8.494066	−7.345214	−4.851908
H	9.547067	−9.311075	−4.392499
H	10.620875	−9.421477	−3.012141
O	6.201496	−11.614114	−11.011125
C	7.382960	−12.445341	−11.130754
C	7.822453	−12.942283	−9.759843
O	8.368484	−11.844471	−8.957276
C	7.560783	−11.630364	−7.772002
C	6.715028	−13.584575	−8.898793
C	6.275652	−12.441361	−7.980431
O	7.331859	−14.701959	−8.193545
H	6.054372	−11.183494	−11.871318
H	8.207350	−11.876081	−11.578587
H	7.174431	−13.318974	−11.765188
H	8.628389	−13.667879	−9.915407
H	8.115829	−11.954720	−6.891174
H	5.888413	−13.968263	−9.502042
H	5.518217	−11.851250	−8.500326
H	5.848638	−12.801986	−7.047081
N	7.343627	−10.187050	−7.585568
C	7.963448	−9.518280	−6.492954
O	8.733432	−10.158388	−5.743093
N	7.679848	−8.204644	−6.297442
C	6.866733	−7.533595	−7.137779
N	6.597786	−6.249934	−6.859268
C	6.310041	−8.167292	−8.298425
C	6.578054	−9.485203	−8.482377
H	7.013103	−5.789943	−6.033924
H	6.000838	−5.715777	−7.477134
H	5.702503	−7.615021	−9.006073
H	6.226202	−10.044478	−9.343279
P	6.600719	−15.487682	−6.937791
O	5.114462	−15.225098	−6.919035
O	7.148078	−16.896846	−6.980738
O	7.210720	−14.694224	−5.625645
C	8.625228	−14.848155	−5.329401
C	8.917753	−14.440297	−3.894186
O	8.809880	−12.991964	−3.735774
C	7.783977	−12.660468	−2.772132
C	7.987487	−15.064992	−2.833197
C	6.977972	−13.948600	−2.550191
O	8.825195	−15.409213	−1.691059
H	9.210303	−14.222286	−6.014367
H	8.915933	−15.895658	−5.468289
H	9.954931	−14.722461	−3.678649
H	8.245461	−12.297359	−1.852757
H	7.499556	−15.973624	−3.194285
H	6.163147	−14.032573	−3.272446
H	6.556556	−14.016847	−1.549680

N	6.991807	−11.528805	−3.279309
C	7.104354	−10.250943	−2.656076
O	7.893818	−10.104576	−1.701159
N	6.320745	−9.241273	−3.118784
C	5.480830	−9.425441	−4.155391
N	4.713922	−8.395327	−4.537166
C	5.405811	−10.685775	−4.836188
C	6.184004	−11.695688	−4.370802
H	4.772369	−7.482293	−4.055281
H	4.084430	−8.501681	−5.321544
H	4.762427	−10.822702	−5.696979
H	6.220381	−12.672931	−4.838931
P	8.200844	−15.867085	−0.231217
O	6.765787	−16.314477	−0.365811
O	9.249214	−16.767573	0.383029
O	8.149309	−14.434807	0.587465
C	9.411807	−13.797663	0.921847
C	9.203554	−12.734437	1.987744
O	8.493184	−11.586341	1.429208
C	7.276064	−11.339489	2.167770
C	8.397671	−13.192209	3.220376
C	6.995818	−12.625041	2.963464
O	9.027383	−12.598046	4.387812
H	9.835829	−13.336593	0.021405
H	10.113348	−14.550261	1.299644
H	10.195473	−12.392670	2.309436
H	7.401938	−10.472129	2.816516
H	8.389310	−14.282739	3.316031
H	6.422282	−13.335345	2.361972
H	6.447726	−12.424893	3.887641
H	8.515837	−12.875225	5.170068
N	6.220228	−10.970057	1.211036
C	5.621993	−9.682855	1.266808
O	5.951505	−8.889149	2.178473
N	4.695000	−9.363193	0.325769
C	4.323519	−10.251357	−0.618462
N	3.386775	−9.876478	−1.501771
C	4.909267	−11.557914	−0.682026
C	5.859157	−11.861807	0.237450
H	2.982514	−8.927449	−1.471703
H	3.111267	−10.509185	−2.240965
H	4.619828	−12.266585	−1.448220
H	6.393089	−12.805424	0.247955

d(GTA)$^{4-}$

E: −27673.45 kcal mol^{-1}

O	−0.352657	−0.405335	−0.120865
C	−0.227337	−0.031220	1.272983
C	1.229868	−0.100963	1.713491
O	1.697331	−1.487640	1.809257
C	2.688747	−1.766450	0.789386
C	2.228638	0.614053	0.784056
C	2.756111	−0.517923	−0.100817
O	3.239153	1.226939	1.636712
H	−1.301735	−0.478809	−0.322800
H	−0.819673	−0.704359	1.905742
H	−0.582343	0.998341	1.426888
H	1.289971	0.327553	2.719523
H	3.644076	−1.985283	1.276311
H	1.753572	1.401479	0.193257
H	2.091128	−0.621446	−0.959133
H	3.760900	−0.322027	−0.465098
N	2.332056	−2.984935	0.064114
C	1.309452	−3.152027	−0.872978
N	1.238638	−4.387783	−1.331333
C	2.251726	−5.081890	−0.666370
C	2.657038	−6.446926	−0.745835
O	2.181633	−7.349148	−1.479313
N	3.716892	−6.735042	0.133474
C	4.295854	−5.828324	0.999518
N	5.246752	−6.297728	1.834222
N	3.938569	−4.540179	1.054756
C	2.931128	−4.229167	0.216941
H	0.649511	−2.335136	−1.129566
H	4.062608	−7.722350	0.136529
H	5.607406	−7.259164	1.747930
H	5.811366	−5.607873	2.313751
P	4.687111	1.774293	1.058092
O	4.624614	2.036636	−0.426775
O	5.112742	2.854639	2.027330
O	5.651856	0.448990	1.238762
C	5.909317	−0.015532	2.590560
C	7.083459	−0.978418	2.604149
O	6.718756	−2.237316	1.957214
C	7.615584	−2.507636	0.852973
C	8.356158	−0.471797	1.890074
C	8.297876	−1.174827	0.530701
O	9.482166	−0.864086	2.729302
H	5.015764	−0.519151	2.979390
H	6.142191	0.839508	3.236113
H	7.321067	−1.194020	3.652518
H	8.337917	−3.273378	1.142146
H	8.366586	0.616130	1.785661
H	7.683990	−0.565135	−0.135192
H	9.277787	−1.296496	0.075784
N	6.844050	−3.101703	−0.246616
C	6.990640	−4.479643	−0.490725
O	7.754922	−5.203209	0.148717
N	6.203526	−4.963559	−1.518197
C	5.317105	−4.238934	−2.305174
O	4.655565	−4.809397	−3.199285
C	5.221525	−2.820376	−2.009264
C	4.313197	−1.969294	−2.852990
C	5.966434	−2.333700	−0.982552
H	6.296313	−5.998559	−1.717830
H	4.574771	−2.067704	−3.913264
H	4.391192	−0.915758	−2.575747
H	3.268190	−2.284683	−2.752630
H	5.902814	−1.295886	−0.675775
P	11.052046	−0.888362	2.221700
O	11.244510	−0.067206	0.969735
O	11.873652	−0.624639	3.464186
O	11.235986	−2.458342	1.754566
C	11.175295	−3.498865	2.765224
C	11.903028	−4.747840	2.287693
O	11.158779	−5.427639	1.229624
C	11.856908	−5.325360	−0.032286
C	13.308122	−4.496838	1.716713
C	13.064012	−4.398483	0.204563
O	14.125094	−5.645731	2.068675
H	10.126225	−3.742358	2.972710
H	11.648219	−3.143073	3.687952
H	11.961508	−5.439394	3.136432
H	12.157469	−6.328839	−0.346604
H	13.753711	−3.581969	2.120994
H	12.816633	−3.367305	−0.054134
H	13.933392	−4.708180	−0.380224
H	15.005114	−5.513797	1.670842
N	10.936641	−4.852320	−1.064335
C	10.422085	−3.572123	−1.219137
N	9.547153	−3.482146	−2.208033
C	9.468985	−4.772473	−2.730455
C	8.709381	−5.331471	−3.784570
N	7.843759	−4.633413	−4.541861
N	8.877787	−6.660080	−4.036638

C	9.728320	−7.383224	−3.286282
N	10.486589	−6.957553	−2.262591
C	10.318963	−5.640858	−2.026372
H	10.722439	−2.770306	−0.560130
H	7.290334	−5.112309	−5.262859
H	7.680722	−3.655731	−4.343037
H	9.791623	−8.437981	−3.547242
O	5.931035	−11.448978	−10.703343
C	7.128623	−12.247874	−10.874435
C	7.639895	−12.737385	−9.525490
O	8.196424	−11.630361	−8.741153
C	7.436571	−11.442643	−7.520563
C	6.588953	−13.408242	−8.616589
C	6.158999	−12.272924	−7.684533
O	7.270437	−14.506643	−7.940103
H	5.730264	−11.031330	−11.559009
H	7.917630	−11.656185	−11.355678
H	6.916608	−13.125808	−11.501681
H	8.454955	−13.442953	−9.720117
H	8.032684	−11.766747	−6.666267
H	5.748574	−13.818096	−9.182713
H	5.379022	−11.694546	−8.183793
H	5.765116	−12.630678	−6.736443
N	7.202194	−10.006302	−7.311815
C	7.840526	−9.364881	−6.238612
O	8.639794	−9.929938	−5.485597
N	7.493278	−8.037409	−6.085446
C	6.667212	−7.284940	−6.912095
O	6.462903	−6.074913	−6.659670
C	6.102680	−7.988460	−8.050968
C	5.236831	−7.237971	−9.026726
C	6.404010	−9.305224	−8.195598
H	7.977470	−7.529748	−5.296218
H	5.784865	−6.396993	−9.469872
H	4.355009	−6.818413	−8.526606
H	4.899318	−7.897230	−9.831768
H	6.050210	−9.884550	−9.043074
P	6.657967	−15.274766	−6.613007
O	5.169682	−15.055375	−6.489394
O	7.245258	−16.667338	−6.657835
O	7.333474	−14.423811	−5.371747
C	8.763827	−14.532537	−5.145241
C	9.099181	−14.331635	−3.672555
O	9.009928	−12.927689	−3.272367
C	7.864105	−12.705788	−2.420027
C	8.192394	−15.101991	−2.697005
C	7.139914	−14.059903	−2.297684
O	9.037860	−15.550000	−1.598374
H	9.284037	−13.777185	−5.746379
H	9.105283	−15.528842	−5.448352
H	10.142910	−14.632068	−3.531560
H	8.213227	−12.328892	−1.454006
H	7.733687	−15.978196	−3.162090
H	6.309374	−14.123474	−3.001614
H	6.754569	−14.231530	−1.295679
N	7.024800	−11.640987	−2.972803
C	6.127564	−11.733365	−4.029402
N	5.511901	−10.590554	−4.286453
C	6.030048	−9.691196	−3.356433
C	5.768017	−8.322701	−3.111211
N	4.894026	−7.593785	−3.822942
N	6.450163	−7.730370	−2.090877
C	7.342484	−8.445469	−1.382345
N	7.672739	−9.738360	−1.537517
C	6.980408	−10.319346	−2.537321
H	5.999333	−12.659129	−4.570954
H	4.770500	−6.593320	−3.621346
H	4.391081	−8.024765	−4.586178
H	7.850247	−7.896343	−0.592395
P	8.415100	−16.167875	−0.198788
O	7.024760	−16.715931	−0.413209
O	9.517959	−17.029779	0.374697
O	8.224528	−14.814246	0.724067
C	9.419825	−14.069873	1.078486
C	9.059927	−12.926882	2.008988
O	8.281375	−11.929919	1.278515
C	7.108259	−11.581222	2.052091
C	8.226811	−13.325439	3.249847
C	6.818470	−12.811756	2.921704
O	8.810011	−12.642692	4.392770
H	9.886760	−13.671575	0.169458
H	10.134790	−14.732841	1.580510
H	9.996008	−12.461916	2.345653
H	7.306274	−10.695993	2.658444
H	8.242357	−14.406896	3.416020
H	6.278764	−13.574441	2.353641
H	6.241705	−12.562416	3.815999
H	8.300086	−12.902870	5.181856
N	6.040030	−11.198239	1.123975
C	5.603758	−9.845937	1.063922
O	6.127269	−8.998207	1.823043
N	4.621704	−9.523834	0.183704
C	4.108045	−10.444331	−0.656587
N	3.144660	−10.051941	−1.502600
C	4.574770	−11.800661	−0.650189
C	5.532585	−12.125105	0.253550
H	2.828730	−9.070330	−1.521303
H	2.772035	−10.704898	−2.179215
H	4.182525	−12.536895	−1.341413
H	5.958303	−13.117910	0.325598

d(TAA)$^{4-}$

E: −27761.58 kcal mol^{-1}

H: −26819.01 kcal mol^{-1}

G: −26970.96 kcal mol^{-1}

O	−0.241339	−0.127634	−0.567268
C	−0.181924	0.274222	0.824077
C	1.216578	0.048568	1.384050
O	1.500243	−1.383247	1.524409
C	2.614229	−1.764446	0.678097
C	2.373145	0.625993	0.542059
C	2.852860	−0.581752	−0.267051
O	3.359118	1.135360	1.488932
H	−1.176691	−0.124630	−0.835639
H	−0.897500	−0.305639	1.420564
H	−0.425064	1.341823	0.925237
H	1.243129	0.488600	2.386945
H	3.485864	−1.979659	1.298073
H	2.050950	1.449845	−0.099708
H	2.233650	−0.665841	−1.162295
H	3.891155	−0.494653	−0.576747
N	2.296505	−3.028997	−0.001949
C	3.006800	−4.186616	0.354688
O	3.853725	−4.216369	1.252396
N	2.685283	−5.302183	−0.393228
C	1.704335	−5.400870	−1.373104
O	1.508912	−6.491275	−1.957716
C	0.948421	−4.188513	−1.634106
C	−0.169464	−4.220006	−2.641191
C	1.277494	−3.071839	−0.934005
H	3.201198	−6.184738	−0.135998
H	−0.918507	−4.974437	−2.370868
H	0.206844	−4.483776	−3.637635
H	−0.662499	−3.245421	−2.702770

H	0.730948	−2.140927	−1.050558
P	4.904217	1.531411	1.059116
O	5.013943	1.773775	−0.426769
O	5.334718	2.580663	2.059287
O	5.717464	0.124142	1.340744
C	5.893276	−0.319076	2.713049
C	7.134617	−1.192165	2.849092
O	6.929150	−2.527123	2.287434
C	7.714903	−2.703735	1.085840
C	8.392443	−0.636787	2.156716
C	8.380589	−1.348099	0.799578
O	9.527307	−0.976253	3.006389
H	5.009697	−0.888015	3.026735
H	6.005935	0.553806	3.366056
H	7.323820	−1.324625	3.919896
H	8.443900	−3.501884	1.256563
H	8.357031	0.449945	2.044013
H	7.773535	−0.756189	0.113661
H	9.375287	−1.450984	0.374065
N	6.864815	−3.178908	−0.003641
C	5.980092	−2.434764	−0.771778
N	5.352180	−3.158092	−1.685167
C	5.843949	−4.451148	−1.511798
C	5.582114	−5.666268	−2.186077
N	4.703023	−5.785276	−3.194287
N	6.273481	−6.766743	−1.776776
C	7.134951	−6.669942	−0.747594
N	7.440695	−5.576784	−0.030003
C	6.775069	−4.488764	−0.462159
H	5.838007	−1.379267	−0.592088
H	4.576539	−6.688414	−3.669356
H	4.214677	−4.965620	−3.527338
H	7.640017	−7.595656	−0.481146
P	11.091865	−0.936781	2.479152
O	11.248887	−0.026920	1.284935
O	11.920349	−0.736917	3.728939
O	11.302264	−2.467307	1.904255
C	11.263336	−3.564482	2.854386
C	11.866618	−4.815621	2.236478
O	10.990347	−5.346172	1.192181
C	11.673419	−5.350396	−0.082788
C	13.245945	−4.613273	1.581707
C	12.912097	−4.457833	0.093451
O	14.027118	−5.809404	1.848179
H	10.223599	−3.760524	3.143487
H	11.835274	−3.296593	3.750572
H	11.941221	−5.573045	3.026448
H	11.940034	−6.378531	−0.347619
H	13.759817	−3.733693	1.982486
H	12.665277	−3.413566	−0.110326
H	13.734352	−4.761968	−0.558752
H	14.892130	−5.698680	1.412594
N	10.753048	−4.900888	−1.120155
C	10.280184	−3.611871	−1.327761
N	9.411839	−3.533000	−2.322325
C	9.296015	−4.839561	−2.796635
C	8.530539	−5.415482	−3.838381
N	7.703524	−4.718637	−4.639285
N	8.654936	−6.758770	−4.034563
C	9.468410	−7.480177	−3.242672
N	10.235301	−7.037125	−2.232211
C	10.115656	−5.706262	−2.055362
H	10.611871	−2.790009	−0.709754
H	7.123373	−5.213968	−5.326711
H	7.530346	−3.740830	−4.447316
H	9.491720	−8.547334	−3.451772
O	6.117782	−11.433995	−10.898277
C	7.351040	−12.183053	−11.038897
C	7.795592	−12.726373	−9.687153
O	8.284550	−11.647054	−8.823303
C	7.460768	−11.524644	−7.639266
C	6.702445	−13.457482	−8.881047
C	6.224085	−12.398674	−7.884636
O	7.334778	−14.609807	−8.253043
H	5.950117	−10.978510	−11.741710
H	8.145451	−11.541746	−11.441048
H	7.206405	−13.033121	−11.721132
H	8.634419	−13.408211	−9.864339
H	8.027295	−11.845869	−6.763511
H	5.888260	−13.812951	−9.517120
H	5.420740	−11.822634	−8.346430
H	5.845751	−12.842306	−6.967338
N	7.155862	−10.101101	−7.404084
C	7.733679	−9.456781	−6.298215
O	8.512774	−10.011514	−5.516948
N	7.344276	−8.142216	−6.137494
C	6.512691	−7.403983	−6.971405
O	6.260362	−6.207977	−6.703039
C	6.006286	−8.107507	−8.138260
C	5.133302	−7.372951	−9.119152
C	6.362997	−9.407998	−8.301204
H	7.788690	−7.635485	−5.324643
H	5.655986	−6.501728	−9.533119
H	4.225104	−6.999419	−8.629762
H	4.839747	−8.028573	−9.944056
H	6.059169	−9.980211	−9.172461
P	6.550894	−15.528248	−7.121734
O	5.057511	−15.325798	−7.197209
O	7.165349	−16.905620	−7.239435
O	7.027858	−14.822762	−5.709166
C	8.436488	−14.901314	−5.364655
C	8.668092	−14.394509	−3.952401
O	8.450558	−12.950404	−3.893934
C	7.485963	−12.639720	−2.860425
C	7.767373	−15.020512	−2.862503
C	6.712884	−13.938521	−2.614931
O	8.633360	−15.292953	−1.721011
H	9.019227	−14.295216	−6.069309
H	8.774357	−15.942413	−5.431209
H	9.717282	−14.592143	−3.701498
H	8.000882	−12.299150	−1.959622
H	7.317905	−15.962346	−3.186816
H	5.917237	−14.070587	−3.351313
H	6.272223	−13.994903	−1.622093
N	6.670358	−11.500605	−3.295401
C	6.886616	−10.250733	−2.685341
O	7.733718	−10.060550	−1.811675
N	6.053827	−9.244902	−3.135879
C	5.085063	−9.342585	−4.125857
O	4.404897	−8.343922	−4.447523
C	4.936140	−10.650467	−4.740596
C	3.893219	−10.830707	−5.809312
C	5.743430	−11.652056	−4.303416
H	6.163229	−8.312746	−2.647210
H	2.905285	−10.539181	−5.433821
H	3.842986	−11.870931	−6.138977
H	4.102941	−10.194940	−6.677752
H	5.705296	−12.643460	−4.739600
P	8.080228	−15.590193	−0.194685
O	6.627422	−15.999441	−0.202737
O	9.137012	−16.463582	0.444024
O	8.112902	−14.088365	0.483638
C	9.396634	−13.465260	0.752862
C	9.291409	−12.509248	1.934326
O	8.564620	−11.292117	1.577946
C	7.286978	−11.243436	2.251783
C	8.568527	−13.083678	3.163539
C	7.126442	−12.585992	2.990801

O	9.209158	−12.521440	4.340043
H	9.724412	−12.914162	−0.136802
H	10.136341	−14.238579	0.988635
H	10.309918	−12.206568	2.204604
H	7.283327	−10.388790	2.934087
H	8.624342	−14.176914	3.193184
H	6.569768	−13.299089	2.379975
H	6.609611	−12.463252	3.945634
H	8.728946	−12.850707	5.121760
N	6.231738	−10.969619	1.277625
C	5.742545	−11.824449	0.298478
N	4.831366	−11.257472	−0.475242
C	4.715889	−9.955173	0.009053
C	3.908239	−8.860827	−0.379584
N	3.029216	−8.901983	−1.396740
N	4.033958	−7.706444	0.334163
C	4.914479	−7.640958	1.349156
N	5.727580	−8.612313	1.797612
C	5.584833	−9.753658	1.093502
H	6.117881	−12.833774	0.207387
H	2.512433	−8.055210	−1.664300
H	2.966887	−9.732879	−1.968861
H	4.971499	−6.676668	1.850554

d(TCA)$^{4−}$

E: −27671.16 kcal mol$^{−1}$

O	−0.358795	−0.192979	−0.401704
C	−0.312848	0.167974	1.001526
C	1.094027	−0.025144	1.552273
O	1.425696	−1.450167	1.651836
C	2.546390	−1.773571	0.792714
C	2.224059	0.614682	0.720480
C	2.754528	−0.553157	−0.114618
O	3.198374	1.139154	1.669369
H	−1.293568	−0.213403	−0.671254
H	−1.006658	−0.455202	1.579849
H	−0.593631	1.222662	1.134805
H	1.110627	0.387132	2.567042
H	3.424665	−1.987929	1.404294
H	1.866640	1.436977	0.096067
H	2.157065	−0.632849	−1.024682
H	3.794403	−0.413790	−0.398477
N	2.259619	−3.023201	0.068931
C	2.995367	−4.177138	0.387354
O	3.837639	−4.219726	1.289347
N	2.713524	−5.268334	−0.409881
C	1.766388	−5.344713	−1.424026
O	1.623438	−6.407203	−2.069888
C	0.981964	−4.142027	−1.646967
C	−0.105416	−4.155447	−2.686538
C	1.260839	−3.051808	−0.887053
H	3.260748	−6.144929	−0.194273
H	−0.855012	−4.923683	−2.459555
H	0.303753	−4.388896	−3.677159
H	−0.605595	−3.184006	−2.736653
H	0.690214	−2.132253	−0.973504
P	4.672880	1.716798	1.193594
O	4.689749	2.005991	−0.287702
O	5.027410	2.782707	2.206442
O	5.649438	0.404011	1.403823
C	5.892710	−0.055598	2.760614
C	7.037958	−1.055307	2.791176
O	6.638855	−2.312787	2.161500
C	7.497956	−2.602176	1.030476
C	8.330885	−0.607449	2.077118
C	8.227415	−1.292103	0.712394
O	9.440591	−1.081765	2.896356
H	4.985148	−0.531692	3.151927
H	6.146991	0.799855	3.397283
H	7.259917	−1.263388	3.844499
H	8.190163	−3.403636	1.292176
H	8.402781	0.479507	1.988092
H	7.629611	−0.654076	0.058451
H	9.196441	−1.446740	0.243589
N	6.683607	−3.134571	−0.069076
C	6.756032	−4.519473	−0.392307
O	7.472881	−5.271992	0.299835
N	6.045419	−4.966750	−1.461275
C	5.236672	−4.144666	−2.155921
N	4.565584	−4.652300	−3.200478
C	5.082746	−2.767682	−1.786948
C	5.822859	−2.314711	−0.742961
H	4.675836	−5.645134	−3.468136
H	3.955966	−4.055214	−3.742954
H	4.400435	−2.114654	−2.317593
H	5.759663	−1.296468	−0.378160
P	11.007709	−1.108287	2.375293
O	11.197980	−0.223431	1.167344
O	11.843439	−0.923202	3.622556
O	11.167301	−2.653365	1.822347
C	11.196340	−3.747224	2.777355
C	11.994415	−4.922318	2.226219
O	11.257024	−5.628144	1.178907
C	11.890707	−5.441315	−0.107090
C	13.350034	−4.547440	1.604340
C	13.032041	−4.428685	0.107930
O	14.268120	−5.637490	1.887064
H	10.169782	−4.069382	2.989434
H	11.664841	−3.406973	3.707580
H	12.142155	−5.632640	3.048147
H	12.252343	−6.409198	−0.465751
H	13.740870	−3.611309	2.016399
H	12.692240	−3.414201	−0.109352
H	13.896220	−4.651675	−0.522506
H	15.115881	−5.427276	1.454470
N	10.890925	−5.009806	−1.080809
C	10.230282	−3.788937	−1.117868
N	9.320191	−3.722069	−2.075290
C	9.369571	−4.968562	−2.697413
C	8.651810	−5.520083	−3.784042
N	7.709542	−4.854772	−4.474013
N	8.953676	−6.799317	−4.146676
C	9.909246	−7.475718	−3.481858
N	10.656618	−7.045337	−2.452637
C	10.338747	−5.785387	−2.092828
H	10.461528	−3.015788	−0.399261
H	7.207177	−5.316632	−5.242597
H	7.424553	−3.935512	−4.165778
H	10.078366	−8.494098	−3.826810
O	5.972081	−11.421512	−10.920299
C	7.170918	−12.216819	−11.099740
C	7.657161	−12.756816	−9.760542
O	8.215279	−11.684179	−8.932132
C	7.437970	−11.521001	−7.717910
C	6.583095	−13.443371	−8.892530
C	6.157479	−12.340586	−7.920030
O	7.233125	−14.576220	−8.244018
H	5.788233	−10.970306	−11.762593
H	7.969348	−11.610015	−11.545158
H	6.967791	−13.070601	−11.762299
H	8.466375	−13.465845	−9.966960
H	8.020045	−11.868965	−6.863046
H	5.743772	−13.815612	−9.485125
H	5.376537	−11.740893	−8.391438
H	5.767776	−12.739196	−6.986806

N	7.211103	−10.087588	−7.479238
C	7.865936	−9.468425	−6.401929
O	8.660220	−10.053606	−5.660352
N	7.545701	−8.135760	−6.233687
C	6.713864	−7.365065	−7.035814
O	6.525666	−6.156078	−6.766555
C	6.130923	−8.045839	−8.179041
C	5.262591	−7.271944	−9.133861
C	6.416168	−9.363077	−8.346673
H	8.041805	−7.651824	−5.439873
H	5.809134	−6.420927	−9.559194
H	4.382519	−6.864307	−8.620751
H	4.921567	−7.911901	−9.952951
H	6.050697	−9.923291	−9.201718
P	6.511077	−15.437998	−7.032050
O	5.016269	−15.226895	−7.033605
O	7.106832	−16.825462	−7.126674
O	7.068707	−14.681157	−5.677813
C	8.476973	−14.815031	−5.347604
C	8.735655	−14.419291	−3.901176
O	8.620666	−12.973229	−3.721202
C	7.535145	−12.662929	−2.811220
C	7.786397	−15.056340	−2.865057
C	6.738758	−13.962168	−2.649729
O	8.598747	−15.368322	−1.692498
H	9.069735	−14.173387	−6.011274
H	8.788063	−15.856496	−5.490667
H	9.769826	−14.699289	−3.669112
H	7.950673	−12.311875	−1.861584
H	7.338934	−15.985217	−3.228128
H	5.996121	−14.054350	−3.444754
H	6.231926	−14.036552	−1.689892
N	6.755657	−11.550996	−3.330759
C	5.825218	−11.568839	−4.367065
N	5.276060	−10.387317	−4.578782
C	5.868406	−9.539465	−3.638839
C	5.654607	−8.158917	−3.356765
O	4.875930	−7.367823	−3.941613
N	6.434305	−7.715629	−2.272681
C	7.340632	−8.492159	−1.576181
N	8.024856	−7.894574	−0.583337
N	7.562709	−9.781379	−1.857789
C	6.799974	−10.245933	−2.863183
H	5.615669	−12.475298	−4.915107
H	6.294621	−6.720282	−1.974710
H	7.807901	−6.936660	−0.268071
H	8.600461	−8.490968	−0.003596
P	8.002617	−15.568320	−0.167376
O	6.538313	−15.935770	−0.184492
O	9.018484	−16.439264	0.537641
O	8.063318	−14.036203	0.436104
C	9.353856	−13.427797	0.703967
C	9.261082	−12.480913	1.894329
O	8.524659	−11.263501	1.559808
C	7.247437	−11.239083	2.236569
C	8.555494	−13.071644	3.126022
C	7.107328	−12.585913	2.971527
O	9.202111	−12.512996	4.301049
H	9.680698	−12.872126	−0.182957
H	10.088749	−14.208468	0.930119
H	10.281691	−12.176489	2.154564
H	7.234359	−10.386810	2.921595
H	8.621565	−14.164576	3.146139
H	6.549478	−13.302011	2.365748
H	6.601088	−12.470916	3.932986
H	8.730033	−12.850248	5.084330
N	6.184084	−10.975161	1.268559
C	5.675924	−11.836750	0.302790
N	4.772808	−11.264475	−0.475711
C	4.686931	−9.951503	−0.015286
C	3.923563	−8.838583	−0.438346
N	3.035892	−8.878118	−1.450101
N	4.105865	−7.665129	0.230395
C	4.980616	−7.604897	1.252145
N	5.741657	−8.597395	1.740705
C	5.560949	−9.750489	1.065613
H	6.030972	−12.854366	0.223711
H	2.581742	−8.010216	−1.758277
H	2.989392	−9.702299	−2.034818
H	5.078187	−6.627155	1.719831

(AAA)$^{2-}$

E: −14078.25 kcal mol^{-1}

O	−0.278001	−0.456206	−0.153483
C	−0.175086	−0.021114	1.223939
C	1.276246	−0.055892	1.687258
O	1.766169	−1.432468	1.803107
C	2.767239	−1.700987	0.788977
C	2.284889	0.662732	0.771170
C	2.805332	−0.467719	−0.121114
O	3.291131	1.254893	1.645926
H	−1.221073	−0.591661	−0.350704
H	−0.765641	−0.675785	1.877440
H	−0.546156	1.008879	1.332148
H	1.311326	0.384106	2.689623
H	3.724633	−1.882415	1.286992
H	1.822854	1.463688	0.187944
H	2.114608	−0.581205	−0.957424
H	3.797050	−0.267187	−0.519033
N	2.454420	−2.945317	0.090616
C	1.570016	−3.156213	−0.959829
N	1.546808	−4.414348	−1.372446
C	2.457406	−5.076008	−0.547935
C	2.887780	−6.420648	−0.474545
N	2.420235	−7.389259	−1.295737
N	3.801185	−6.749453	0.475854
C	4.259326	−5.786607	1.299189
N	3.926792	−4.484069	1.321823
C	3.023207	−4.181865	0.374345
H	0.958589	−2.355764	−1.350733
H	2.842554	−8.309066	−1.272498
H	1.818486	−7.152109	−2.073260
H	4.992873	−6.107697	2.036728
P	4.797684	1.706031	1.143393
O	4.829709	1.967171	−0.343245
O	5.238173	2.761798	2.132620
O	5.669986	0.325832	1.370655
C	5.889849	−0.137962	2.729159
C	7.086333	−1.076201	2.787444
O	6.785757	−2.357478	2.148758
C	7.600653	−2.535309	0.966318

C	8.361086	−0.551180	2.098908
C	8.281106	−1.185024	0.708643
O	9.486278	−1.008678	2.905760
H	4.994975	−0.664186	3.083631
H	6.079555	0.719977	3.384454
H	7.292389	−1.279334	3.844283
H	8.322489	−3.338401	1.146079
H	8.389027	0.540592	2.049792
H	7.648489	−0.549090	0.087293
H	9.251629	−1.279740	0.229303
N	6.769319	−3.000223	−0.138564
C	5.897971	−2.256369	−0.921719
N	5.284854	−2.979786	−1.845578
C	5.772553	−4.274251	−1.663349
C	5.519695	−5.496902	−2.324085
N	4.666295	−5.601487	−3.372589
N	6.187488	−6.601248	−1.906443
C	7.045228	−6.490695	−0.873795
N	7.352514	−5.392028	−0.162013
C	6.692404	−4.307787	−0.601730
H	5.756657	−1.198825	−0.752025
H	4.410096	−6.525740	−3.698539
H	4.033943	−4.837707	−3.576143
H	7.552411	−7.408540	−0.581823
P	11.049886	−1.032158	2.376685
O	11.243872	−0.127693	1.183721
O	11.889681	−0.874531	3.624918
O	11.198460	−2.566748	1.794203
C	11.149058	−3.679317	2.725174
C	11.829963	−4.901665	2.126356
O	11.039836	−5.468556	1.034668
C	11.718438	−5.297451	−0.230474
C	13.229093	−4.642113	1.543238
C	12.958991	−4.428267	0.047988
O	14.025439	−5.831120	1.795171
H	10.102693	−3.916604	2.952773
H	11.662342	−3.405244	3.654177
H	11.888817	−5.663619	2.912605
H	11.979560	−6.283832	−0.623969
H	13.704295	−3.768884	2.002414
H	12.741137	−3.373790	−0.130808
H	13.806853	−4.721238	−0.575691
H	14.902239	−5.688390	1.393904
N	10.794748	−4.718806	−1.204838
C	10.326260	−3.412164	−1.252218
N	9.434549	−3.214267	−2.209037
C	9.297835	−4.456413	−2.827677
C	8.491043	−4.911239	−3.893234
N	7.635728	−4.100107	−4.569126
N	8.596538	−6.211990	−4.268750
C	9.441611	−7.016031	−3.595815
N	10.236998	−6.703600	−2.555647
C	10.130797	−5.407031	−2.212287
H	10.673411	−2.673708	−0.543824
H	6.932212	−4.539805	−5.152559
H	7.393753	−3.205539	−4.159491
H	9.481272	−8.050042	−3.935014

(AAC)$^{2-}$

E: −13679.68 kcal mol^{-1}

O	−0.299780	−0.398510	−0.151006
C	−0.183975	0.048142	1.221850
C	1.266434	−0.018932	1.683936
O	1.721348	−1.407432	1.803093
C	2.728333	−1.696467	0.800686
C	2.291976	0.671608	0.764264
C	2.780420	−0.474823	−0.123989
O	3.314791	1.238228	1.637468
H	−1.246918	−0.500957	−0.348771
H	−0.789928	−0.584934	1.882670
H	−0.528913	1.088219	1.320088
H	1.313295	0.424180	2.684451
H	3.681563	−1.877087	1.306994
H	1.850416	1.483517	0.180376
H	2.075107	−0.584904	−0.948690
H	3.769103	−0.297026	−0.539316
N	2.413482	−2.946883	0.116434
C	1.526870	−3.165550	−0.930333
N	1.524566	−4.420744	−1.352679
C	2.454320	−5.071103	−0.540537
C	2.918451	−6.405725	−0.487982
N	2.468520	−7.374243	−1.317681
N	3.851821	−6.722452	0.447474
C	4.293405	−5.758539	1.278615
N	3.928481	−4.465088	1.321020
C	3.008708	−4.174099	0.385568
H	0.899582	−2.372539	−1.311824
H	2.903203	−8.288420	−1.302897
H	1.838083	−7.147627	−2.075207
H	5.042711	−6.069944	2.004350
P	4.833804	1.652752	1.142995
O	4.890039	1.879520	−0.348509
O	5.284084	2.719560	2.115330
O	5.680622	0.261221	1.409638
C	5.866191	−0.187921	2.778307
C	7.073893	−1.108710	2.886427
O	6.818335	−2.409403	2.267403
C	7.622435	−2.577275	1.079293
C	8.359047	−0.576308	2.227067
C	8.336946	−1.235265	0.845072
O	9.469265	−1.001083	3.070177
H	4.967857	−0.722401	3.110308
H	6.024985	0.678741	3.430245
H	7.250159	−1.290401	3.952251
H	8.322272	−3.404131	1.235523
H	8.370102	0.514387	2.156493
H	7.755280	−0.601344	0.174185
H	9.334015	−1.354360	0.428940
N	6.775772	−2.991573	−0.039081
C	5.889735	−2.204490	−0.763241
N	5.263484	−2.873843	−1.718237
C	5.757685	−4.174187	−1.622176
C	5.501098	−5.352010	−2.358412
N	4.622629	−5.394429	−3.391276
N	6.187203	−6.475707	−2.031029
C	7.060886	−6.426741	−1.005994
N	7.369321	−5.377356	−0.224708
C	6.695602	−4.269934	−0.580141
H	5.747873	−1.161274	−0.520616
H	4.358863	−6.299662	−3.762512
H	3.973789	−4.627950	−3.519711
H	7.581539	−7.357175	−0.785937
P	11.046761	−0.902040	2.588514
O	11.213032	0.094416	1.467122
O	11.843365	−0.783728	3.868486
O	11.297588	−2.385749	1.910020
C	11.245453	−3.547510	2.781963
C	11.790156	−4.770886	2.063974
O	10.876260	−5.177852	1.000002
C	11.573634	−5.231092	−0.267375
C	13.175893	−4.587310	1.410535
C	12.853467	−4.401076	−0.077398
O	13.930990	−5.803297	1.663802
H	10.206526	−3.730448	3.082624
H	11.845819	−3.361028	3.680028

H	11.843726	−5.586149	2.796836
H	11.790335	−6.267115	−0.530986
H	13.707971	−3.725927	1.826731
H	12.665215	−3.342879	−0.277114
H	13.663435	−4.740888	−0.727799
H	14.803514	−5.700815	1.241413
N	10.667013	−4.737020	−1.313045
C	10.194200	−5.622476	−2.329829
O	10.602879	−6.801884	−2.358858
N	9.307860	−5.132749	−3.241338
C	8.883720	−3.864124	−3.174346
N	8.001282	−3.446287	−4.108312
C	9.336461	−2.959934	−2.159681
C	10.216416	−3.448226	−1.248410
H	7.612073	−4.117698	−4.759354
H	7.563145	−2.537748	−4.037857
H	8.978950	−1.939032	−2.107327
H	10.589780	−2.862988	−0.415929

AAG)$^{2-}$

E: −14242.31 kcal mol^{-1}

O	−0.260193	−0.512363	−0.167224
C	−0.162566	−0.064760	1.206562
C	1.287076	−0.094175	1.675511
O	1.778623	−1.469400	1.800432
C	2.787686	−1.739431	0.794254
C	2.297962	0.621125	0.759257
C	2.822264	−0.513352	−0.125211
O	3.300636	1.218225	1.634838
H	−1.202450	−0.650809	−0.366565
H	−0.755042	−0.713931	1.863732
H	−0.534684	0.965878	1.304179
H	1.317622	0.351738	2.675445
H	3.743663	−1.909676	1.298700
H	1.837258	1.418894	0.170666
H	2.131407	−0.635474	−0.960232
H	3.813250	−0.313289	−0.524899
N	2.487925	−2.991409	0.105143
C	1.609160	−3.218362	−0.946546
N	1.602349	−4.478880	−1.352542
C	2.518687	−5.125074	−0.522123
C	2.966242	−6.463560	−0.441186
N	2.513271	−7.441818	−1.259371
N	3.881025	−6.776201	0.513180
C	4.325157	−5.803530	1.332669
N	3.976379	−4.505143	1.347590
C	3.071187	−4.219237	0.396684
H	0.989720	−2.427124	−1.343709
H	2.950889	−8.354484	−1.233994
H	1.912812	−7.214606	−2.040883
H	5.060632	−6.111668	2.073737
P	4.810423	1.663537	1.137191
O	4.848465	1.922803	−0.349599
O	5.250405	2.718649	2.127142
O	5.677634	0.280637	1.368187
C	5.900639	−0.180417	2.727095
C	7.121521	−1.086798	2.792456
O	6.859541	−2.382650	2.167205
C	7.659717	−2.540664	0.971631
C	8.380007	−0.531042	2.098919
C	8.321694	−1.180267	0.714318
O	9.518661	−0.938544	2.913634
H	5.017257	−0.730066	3.074634
H	6.064006	0.681021	3.384894
H	7.333027	−1.275183	3.850879
H	8.391700	−3.338028	1.135313
H	8.372216	0.560403	2.036976
H	7.687754	−0.558304	0.080597
H	9.297892	−1.265540	0.245355
N	6.819758	−3.004390	−0.128565
C	5.935449	−2.260601	−0.898433
N	5.315109	−2.982067	−1.818277
C	5.811699	−4.274364	−1.649838
C	5.556464	−5.494805	−2.313728
N	4.689914	−5.600865	−3.350397
N	6.228731	−6.600613	−1.904666
C	7.087010	−6.493990	−0.871782
N	7.397781	−5.397649	−0.158609
C	6.740096	−4.310643	−0.595616
H	5.790513	−1.204953	−0.720480
H	4.428696	−6.525148	−3.671787
H	4.061950	−4.833888	−3.554584
H	7.593118	−7.413389	−0.583138
P	11.080093	−0.952643	2.379770
O	11.264825	−0.064039	1.173309
O	11.920042	−0.765908	3.624329
O	11.242818	−2.494066	1.821327
C	11.146739	−3.589371	2.768328
C	11.746278	−4.853336	2.170164
O	10.918205	−5.358411	1.077439
C	11.623805	−5.266886	−0.182014
C	13.161801	−4.687498	1.591142
C	12.912836	−4.471020	0.093308
O	13.881583	−5.921343	1.857254
H	10.092838	−3.764063	3.016920
H	11.690380	−3.334746	3.685951
H	11.754532	−5.618303	2.956051
H	11.828839	−6.278424	−0.543599
H	13.688992	−3.842059	2.045425
H	12.758209	−3.406982	−0.094678
H	13.744478	−4.818598	−0.524299
H	14.767494	−5.836058	1.459525
N	10.754824	−4.658019	−1.185220
C	10.384546	−3.317857	−1.285679
N	9.531631	−3.098325	−2.267994
C	9.316022	−4.349084	−2.846435
C	8.493073	−4.734561	−3.947277
O	7.765048	−4.032883	−4.672947
N	8.579400	−6.135670	−4.176456
C	9.336383	−7.029589	−3.450613
N	9.232708	−8.346056	−3.779780
N	10.109494	−6.657871	−2.434353
C	10.062084	−5.331545	−2.177884
H	10.771122	−2.580562	−0.597187
H	7.993291	−6.485051	−4.931551
H	8.877690	−8.605434	−4.692530
H	9.928017	−8.966287	−3.380286

(AAT)$^{2-}$

E: −13938.86 kcal mol^{-1}

O	−0.200469	−0.369391	−0.290611
C	−0.135438	0.106966	1.075633
C	1.289312	0.014275	1.608401
O	1.703446	−1.383071	1.768456
C	2.756404	−1.706245	0.824527
C	2.376830	0.664169	0.731558
C	2.868302	−0.506044	−0.121943
O	3.379791	1.198968	1.648346
H	−1.140051	−0.456355	−0.527937
H	−0.787774	−0.494558	1.721385
H	−0.457510	1.157165	1.134381
H	1.297729	0.471959	2.603439

H	3.681895	−1.883678	1.380512
H	1.990920	1.486502	0.123454
H	2.187887	−0.617200	−0.967264
H	3.874206	−0.358398	−0.505712
N	2.462873	−2.968846	0.156753
C	1.620795	−3.208905	−0.921358
N	1.642436	−4.470542	−1.323344
C	2.542455	−5.102665	−0.464124
C	3.021778	−6.429682	−0.378041
N	2.616107	−7.414480	−1.214149
N	3.919121	−6.724838	0.598183
C	4.317634	−5.746056	1.433632
N	3.939544	−4.455744	1.442446
C	3.053625	−4.187518	0.468925
H	1.006776	−2.424871	−1.341426
H	3.105418	−8.301217	−1.203980
H	2.051419	−7.188143	−2.022277
H	5.039967	−6.039937	2.193213
P	4.927067	1.574250	1.213170
O	5.042409	1.810979	−0.273142
O	5.371652	2.618454	2.212129
O	5.727654	0.157679	1.494745
C	5.874047	−0.306638	2.863129
C	7.087557	−1.217658	2.998716
O	6.861173	−2.526148	2.385651
C	7.675119	−2.691962	1.205595
C	8.377471	−0.669416	2.362767
C	8.422078	−1.362057	0.995996
O	9.477334	−1.034285	3.245692
H	4.970188	−0.851849	3.160945
H	6.005833	0.553889	3.528964
H	7.244589	−1.392018	4.068759
H	8.355471	−3.535683	1.354427
H	8.358135	0.418454	2.261511
H	7.900881	−0.734160	0.272915
H	9.441231	−1.518358	0.650648
N	6.833793	−3.067753	0.065258
C	5.963473	−2.247068	−0.641501
N	5.322011	−2.881441	−1.609132
C	5.784410	−4.194566	−1.539229
C	5.490994	−5.351996	−2.294512
N	4.617967	−5.349434	−3.329812
N	6.130172	−6.506313	−1.974043
C	6.989698	−6.504706	−0.936454
N	7.340911	−5.474107	−0.146998
C	6.715674	−4.335386	−0.495354
H	5.844610	−1.206941	−0.374224
H	4.331966	−6.236129	−3.727181
H	4.011467	−4.550803	−3.466993
H	7.464798	−7.458762	−0.716651
P	11.055107	−0.813392	2.802841
O	11.177976	0.260044	1.748803
O	11.818659	−0.727656	4.105831
O	11.407028	−2.231159	2.038504
C	11.346691	−3.450769	2.823200
C	11.737616	−4.633757	1.959464
O	10.713034	−4.844522	0.940551
C	11.361297	−5.099533	−0.323782
C	13.092047	−4.496551	1.218401
C	12.691358	−4.337212	−0.255969
O	13.836269	−5.719686	1.462837
H	10.328276	−3.592712	3.204564
H	12.035046	−3.381156	3.673991
H	11.781684	−5.521834	2.604036
H	11.519096	−6.171133	−0.462558
H	13.663614	−3.636907	1.580268
H	12.546088	−3.277025	−0.480532
H	13.439416	−4.743809	−0.940949
H	14.710447	−5.620941	1.042992
N	10.448909	−4.687341	−1.396567
C	9.896653	−5.670642	−2.222095
O	10.153781	−6.874997	−2.132406
N	9.022288	−5.175021	−3.172649
C	8.641530	−3.843267	−3.388024
O	7.855023	−3.567555	−4.306884
C	9.236912	−2.887279	−2.466533
C	8.889399	−1.432320	−2.613912
C	10.096699	−3.356235	−1.527596
H	8.610299	−5.872214	−3.789909
H	9.180279	−1.066166	−3.605862
H	9.399432	−0.829120	−1.859493
H	7.808678	−1.278270	−2.520003
H	10.562663	−2.698578	−0.804075

(ACA)$^{2-}$

E: −13676.57 kcal mol^{-1}

O	−0.281265	−0.409928	−0.168792
C	−0.200206	0.020346	1.212092
C	1.238738	−0.046906	1.708939
O	1.690110	−1.434593	1.854444
C	2.707404	−1.744666	0.871912
C	2.284123	0.633888	0.805846
C	2.806819	−0.521402	−0.051680
O	3.286184	1.215791	1.690269
H	−1.223350	−0.498584	−0.395717
H	−0.821196	−0.621262	1.850100
H	−0.549855	1.058361	1.312346
H	1.262850	0.404708	2.706360
H	3.647617	−1.953285	1.391697
H	1.852938	1.432239	0.196431
H	2.147665	−0.633810	−0.913542
H	3.818148	−0.348420	−0.410655
N	2.370476	−2.982884	0.171522
C	1.440076	−3.165846	−0.845361
N	1.417386	−4.406103	−1.307708
C	2.377360	−5.083537	−0.556160
C	2.852626	−6.414528	−0.585605
N	2.417158	−7.327598	−1.484793
N	3.811811	−6.772439	0.307228
C	4.283858	−5.841988	1.160545
N	3.928865	−4.549537	1.262537
C	2.972328	−4.220452	0.376860
H	0.797688	−2.358051	−1.167066
H	2.707552	−8.293632	−1.393244
H	1.622104	−7.118066	−2.074352
H	5.054818	−6.182806	1.849243
P	4.760024	1.736810	1.154151
O	4.732281	2.025886	−0.327208
O	5.189203	2.790283	2.150724
O	5.692589	0.389003	1.328218
C	5.945841	−0.098236	2.673320
C	7.106453	−1.079899	2.672107
O	6.728531	−2.324941	2.009188
C	7.589368	−2.568135	0.865065
C	8.384935	−0.583015	1.964637
C	8.296964	−1.238483	0.584553
O	9.506491	−1.038449	2.778483
H	5.045601	−0.594326	3.056402
H	6.191490	0.744816	3.329547
H	7.338455	−1.313144	3.718049
H	8.295179	−3.366063	1.100880
H	8.426021	0.507371	1.898356
H	7.691746	−0.593834	−0.056286
H	9.267821	−1.370329	0.116372
N	6.772796	−3.090210	−0.238151

C	6.829554	−4.491542	−0.548272
O	7.591165	−5.228812	0.099058
N	6.036872	−4.950539	−1.558555
C	5.204035	−4.132041	−2.205660
N	4.465741	−4.654241	−3.214419
C	5.085480	−2.742760	−1.876064
C	5.882938	−2.276411	−0.878864
H	4.463433	−5.658687	−3.347472
H	3.712829	−4.120448	−3.628372
H	4.381772	−2.093610	−2.382874
H	5.840085	−1.251686	−0.529189
P	11.077146	−1.045049	2.273642
O	11.273434	−0.184102	1.048931
O	11.896073	−0.820955	3.526384
O	11.268435	−2.594485	1.753785
C	11.178842	−3.682478	2.707038
C	11.850393	−4.926661	2.142800
O	11.092271	−5.485412	1.026481
C	11.786907	−5.269172	−0.223591
C	13.274290	−4.706526	1.607314
C	13.061025	−4.467426	0.105262
O	14.022937	−5.923600	1.870801
H	10.123717	−3.897404	2.915452
H	11.677240	−3.399884	3.642159
H	11.860288	−5.684248	2.935228
H	12.009579	−6.246952	−0.658954
H	13.763469	−3.854510	2.091252
H	12.911131	−3.401460	−0.070067
H	13.912082	−4.802098	−0.492447
H	14.913114	−5.809779	1.490492
N	10.895657	−4.612065	−1.181226
C	10.567718	−3.264792	−1.273230
N	9.647870	−3.015855	−2.191364
C	9.340223	−4.262716	−2.732671
C	8.420412	−4.673394	−3.720389
N	7.633630	−3.798262	−4.400820
N	8.344113	−5.995438	−4.017159
C	9.135450	−6.856467	−3.347757
N	10.032771	−6.584675	−2.381792
C	10.098029	−5.268458	−2.111494
H	11.024797	−2.529632	−0.627443
H	6.835460	−4.172749	−4.901978
H	7.542913	−2.855199	−4.041922
H	9.031618	−7.904877	−3.623604

$(ACC)^{2-}$

E: −13278.41 kcal mol^{-1}

O	−0.298223	−0.341201	−0.125220
C	−0.207836	0.082414	1.257142
C	1.233336	0.005040	1.745561
O	1.678362	−1.386076	1.878514
C	2.690305	−1.693599	0.890255
C	2.276819	0.687613	0.841269
C	2.789790	−0.464666	−0.025752
O	3.283550	1.263937	1.724708
H	−1.241986	−0.419319	−0.349152
H	−0.828751	−0.558835	1.895583
H	−0.551206	1.121800	1.363980
H	1.265544	0.449359	2.745947
H	3.631634	−1.909273	1.405198
H	1.845082	1.490720	0.238474
H	2.123964	−0.569101	−0.883553
H	3.798436	−0.291664	−0.391896
N	2.347317	−2.926402	0.182638
C	1.411387	−3.100668	−0.830783
N	1.390401	−4.335398	−1.307851
C	2.358281	−5.018024	−0.571041
C	2.838051	−6.347257	−0.618731
N	2.396531	−7.253217	−1.521290
N	3.806166	−6.710961	0.262565
C	4.279099	−5.788887	1.124471
N	3.918511	−4.499608	1.244884
C	2.955256	−4.164149	0.368961
H	0.764356	−2.291325	−1.139163
H	2.695579	−8.218017	−1.447008
H	1.601376	−7.038403	−2.108715
H	5.056570	−6.134347	1.803494
P	4.775662	1.746918	1.205386
O	4.782527	2.012754	−0.280533
O	5.211306	2.805235	2.193507
O	5.676224	0.381366	1.416208
C	5.882935	−0.102936	2.770624
C	7.048203	−1.078887	2.811525
O	6.700856	−2.328941	2.140599
C	7.575530	−2.559844	1.007731
C	8.343114	−0.574098	2.143953
C	8.306244	−1.231989	0.762047
O	9.447397	−1.022653	2.983434
H	4.971836	−0.602908	3.121144
H	6.101212	0.742165	3.433697
H	7.246485	−1.309602	3.864640
H	8.265201	−3.373962	1.235851
H	8.377768	0.516101	2.075680
H	7.739147	−0.585619	0.089152
H	9.302093	−1.368213	0.348967
N	6.771101	−3.044211	−0.124394
C	6.853213	−4.425563	−0.507580
O	7.617325	−5.186139	0.109746
N	6.083441	−4.841307	−1.553672
C	5.246747	−4.001268	−2.169658
N	4.520649	−4.480984	−3.205310
C	5.108622	−2.631565	−1.772068
C	5.883850	−2.207453	−0.739787
H	4.540366	−5.474310	−3.403307
H	3.780865	−3.927755	−3.616879
H	4.405939	−1.965845	−2.258149
H	5.824972	−1.202168	−0.338636
P	11.028492	−0.916287	2.516365
O	11.198909	0.063361	1.380977
O	11.812982	−0.770574	3.801550
O	11.299851	−2.408175	1.865703
C	11.244283	−3.558099	2.752741
C	11.820145	−4.786345	2.066144
O	10.934058	−5.233224	0.996095
C	11.631029	−5.236798	−0.272399
C	13.211487	−4.594433	1.430914
C	12.906163	−4.403595	−0.060427
O	13.967491	−5.809282	1.687817
H	10.201487	−3.751015	3.032404
H	11.822189	−3.349669	3.660632
H	11.872351	−5.585696	2.816331
H	11.854435	−6.262919	−0.566345
H	13.735768	−3.732877	1.856762
H	12.722410	−3.344825	−0.259109
H	13.724795	−4.740983	−0.701194
H	14.840642	−5.706887	1.266766
N	10.720822	−4.720535	−1.306199
C	10.203466	−5.599048	−2.306865
O	10.577787	−6.791212	−2.333907
N	9.315387	−5.092888	−3.207849
C	8.920277	−3.814308	−3.137147
N	8.032161	−3.376601	−4.055407
C	9.404983	−2.921292	−2.128816
C	10.288697	−3.425976	−1.230121
H	7.621509	−4.033286	−4.707926

H	7.642473	−2.445508	−3.999054
H	9.064845	−1.895076	−2.069528
H	10.677655	−2.849126	−0.398921

(ACG)$^{2-}$

E: −13840.38 kcal mol^{-1}

O	−0.267804	−0.448811	−0.153787
C	−0.185276	−0.024718	1.228882
C	1.254754	−0.090541	1.722556
O	1.710624	−1.477733	1.859013
C	2.730282	−1.777442	0.875655
C	2.296493	0.599307	0.821898
C	2.822789	−0.549618	−0.042203
O	3.295768	1.182124	1.709026
H	−1.210234	−0.531974	−0.381380
H	−0.803550	−0.670714	1.865129
H	−0.537163	1.011976	1.334597
H	1.279237	0.355328	2.722566
H	3.671648	−1.983249	1.394462
H	1.861277	1.398848	0.216897
H	2.161348	−0.660950	−0.902535
H	3.832581	−0.371812	−0.403497
N	2.400649	−3.014043	0.169328
C	1.464523	−3.198606	−0.841859
N	1.449867	−4.435839	−1.312690
C	2.422135	−5.109265	−0.573252
C	2.909282	−6.435485	−0.614899
N	2.475431	−7.347049	−1.517082
N	3.878974	−6.790186	0.267802
C	4.348538	−5.861069	1.124177
N	3.981714	−4.573046	1.237900
C	3.015816	−4.247042	0.361385
H	0.812416	−2.394333	−1.152956
H	2.767807	−8.312591	−1.426065
H	1.668561	−7.142691	−2.092366
H	5.128230	−6.199036	1.804340
P	4.774553	1.695431	1.179481
O	4.751728	1.998159	−0.299232
O	5.210112	2.736565	2.186107
O	5.694944	0.338218	1.342781
C	5.947332	−0.160674	2.684012
C	7.132787	−1.112209	2.680148
O	6.787919	−2.365580	2.016463
C	7.648835	−2.585549	0.867382
C	8.395119	−0.575986	1.971890
C	8.335380	−1.242386	0.594967
O	9.530873	−0.976993	2.794630
H	5.056048	−0.683886	3.051601
H	6.165805	0.679657	3.353040
H	7.372972	−1.340956	3.725267
H	8.366619	−3.375563	1.094151
H	8.394420	0.514528	1.896331
H	7.728013	−0.611867	−0.057702
H	9.314229	−1.359148	0.139612
N	6.837649	−3.108641	−0.239562
C	6.909298	−4.506311	−0.561332
O	7.669036	−5.243168	0.089410
N	6.131113	−4.964241	−1.583125
C	5.282177	−4.151279	−2.218075
N	4.538418	−4.675408	−3.218746
C	5.139319	−2.768994	−1.867635
C	5.931986	−2.301412	−0.868339
H	4.560363	−5.675263	−3.377667
H	3.793818	−4.140347	−3.644807
H	4.421637	−2.126183	−2.362946
H	5.871633	−1.282445	−0.504586
P	11.100140	−0.959977	2.286962
O	11.282810	−0.106659	1.054726
O	11.915395	−0.709313	3.537275
O	11.315729	−2.511699	1.783610
C	11.198808	−3.585749	2.750068
C	11.816678	−4.858492	2.189393
O	11.027803	−5.386956	1.080578
C	11.733731	−5.226100	−0.172017
C	13.246064	−4.698632	1.646765
C	13.035826	−4.465604	0.143988
O	13.949228	−5.940668	1.919107
H	10.139407	−3.759790	2.974256
H	11.718531	−3.311920	3.676422
H	11.801116	−5.611794	2.986070
H	11.922735	−6.221250	−0.583286
H	13.769436	−3.861764	2.121213
H	12.920165	−3.396702	−0.038995
H	13.872573	−4.833245	−0.454731
H	14.841605	−5.863426	1.534709
N	10.874862	−4.556948	−1.148833
C	10.619571	−3.192996	−1.289240
N	9.740489	−2.935807	−2.239596
C	9.382766	−4.182785	−2.750401
C	8.468207	−4.533185	−3.788652
O	7.773801	−3.793690	−4.510350
N	8.411909	−5.943431	−3.957753
C	9.129328	−6.872892	−3.236024
N	8.898065	−8.188054	−3.509172
N	9.985721	−6.535579	−2.277462
C	10.070533	−5.201027	−2.075810
H	11.095150	−2.461597	−0.652916
H	7.762400	−6.266474	−4.671300
H	8.490211	−8.444741	−4.400724
H	9.565621	−8.847068	−3.124156

(ACT)$^{2-}$

E: −13537.30 kcal mol^{-1}

O	−0.242841	−0.331197	−0.145787
C	−0.168351	0.092959	1.237336
C	1.264031	0.000627	1.748672
O	1.692912	−1.394925	1.888761
C	2.715755	−1.713127	0.914851
C	2.329784	0.671549	0.862011
C	2.839298	−0.485615	−0.000184
O	3.331454	1.230202	1.762979
H	−1.183950	−0.406783	−0.381240
H	−0.805996	−0.540603	1.866748
H	−0.502013	1.136156	1.337788
H	1.284478	0.444555	2.749523
H	3.648037	−1.936844	1.442434
H	1.919179	1.482967	0.255603
H	2.181683	−0.583048	−0.865134
H	3.853806	−0.323326	−0.354529
N	2.371108	−2.944000	0.205359
C	1.438020	−3.115040	−0.811175
N	1.412095	−4.350623	−1.285831
C	2.374229	−5.036850	−0.544782
C	2.848951	−6.367954	−0.589450
N	2.405713	−7.273952	−1.491487
N	3.813862	−6.734316	0.294307
C	4.288014	−5.813066	1.156531
N	3.931437	−4.522632	1.274905
C	2.972244	−4.184409	0.395739
H	0.797051	−2.302266	−1.123394
H	2.698760	−8.240177	−1.412133
H	1.610561	−7.057936	−2.078399

H	5.062712	−6.160580	1.837744
P	4.843443	1.680259	1.273782
O	4.883527	1.957916	−0.209143
O	5.287336	2.718353	2.279424
O	5.707227	0.290717	1.487628
C	5.883596	−0.211347	2.839966
C	7.054036	−1.181204	2.894598
O	6.724062	−2.433535	2.221591
C	7.611971	−2.670198	1.101897
C	8.352142	−0.659862	2.245641
C	8.384573	−1.360383	0.882661
O	9.449734	−1.033762	3.127540
H	4.966674	−0.719655	3.161895
H	6.082403	0.625788	3.518934
H	7.241187	−1.410679	3.950175
H	8.274425	−3.507630	1.325453
H	8.349061	0.427366	2.138581
H	7.884282	−0.722836	0.151949
H	9.402240	−1.541020	0.544505
N	6.812927	−3.109460	−0.055843
C	6.868748	−4.479708	−0.477726
O	7.626537	−5.270794	0.110571
N	6.087249	−4.854706	−1.529391
C	5.265526	−3.983025	−2.122575
N	4.519349	−4.425408	−3.157368
C	5.155878	−2.621647	−1.689070
C	5.938219	−2.240776	−0.646264
H	4.541472	−5.407737	−3.401879
H	3.810742	−3.839585	−3.577505
H	4.468719	−1.929107	−2.159264
H	5.896362	−1.246541	−0.215714
P	11.026755	−0.767914	2.708037
O	11.132048	0.281596	1.628291
O	11.764158	−0.619895	4.021232
O	11.444468	−2.193008	1.991905
C	11.429520	−3.385787	2.820091
C	11.851952	−4.589498	1.999562
O	10.826316	−4.872312	1.002019
C	11.457665	−5.106077	−0.274454
C	13.193929	−4.444070	1.240755
C	12.773487	−4.316388	−0.230930
O	13.960076	−5.652033	1.495696
H	10.417855	−3.547841	3.211292
H	12.119066	−3.261597	3.663645
H	11.927839	−5.448067	2.680303
H	11.636216	−6.173771	−0.418179
H	13.757472	−3.570155	1.580896
H	12.609997	−3.262311	−0.469123
H	13.521492	−4.719903	−0.917811
H	14.822881	−5.551675	1.053174
N	10.519534	−4.713432	−1.332589
C	9.901636	−5.716648	−2.085561
O	10.106805	−6.925264	−1.928048
N	9.040663	−5.238274	−3.055254
C	8.693267	−3.908155	−3.322825
O	7.905274	−3.646915	−4.245392
C	9.319522	−2.931553	−2.445184
C	8.992520	−1.476833	−2.636437
C	10.186438	−3.382570	−1.503692
H	8.579806	−5.950310	−3.618395
H	9.255703	−1.153591	−3.650716
H	9.537792	−0.854935	−1.922608
H	7.918103	−1.298846	−2.512539
H	10.674986	−2.706479	−0.812823

(AGA)$^{2-}$

E: −14244.96 kcal mol^{-1}

O	−0.164781	−0.610462	−0.169286
C	−0.149339	−0.158399	1.206383
C	1.279869	−0.095154	1.731304
O	1.847589	−1.435073	1.908656
C	2.897356	−1.678857	0.939354
C	2.280240	0.661397	0.838561
C	2.927492	−0.453982	0.012052
O	3.202958	1.347723	1.734702
H	−1.089384	−0.797216	−0.408239
H	−0.725458	−0.843185	1.841543
H	−0.589115	0.846776	1.285819
H	1.245225	0.371245	2.721659
H	3.841554	−1.821222	1.473705
H	1.793426	1.407111	0.204870
H	2.319128	−0.621824	−0.877425
H	3.936495	−0.198286	−0.302542
N	2.654191	−2.942763	0.242489
C	1.791955	−3.193494	−0.818099
N	1.824368	−4.452435	−1.226510
C	2.751358	−5.073963	−0.390528
C	3.241032	−6.397363	−0.316801
N	2.811656	−7.390168	−1.136301
N	4.160614	−6.688705	0.639366
C	4.557208	−5.711383	1.478668
N	4.164119	−4.426327	1.502385
C	3.266953	−4.156395	0.538972
H	1.151944	−2.418995	−1.215792
H	3.334205	−8.258486	−1.149980
H	2.296644	−7.146628	−1.973354
H	5.291990	−6.002865	2.227049
P	4.613758	2.032299	1.214034
O	4.562876	2.333765	−0.264404
O	4.918159	3.116497	2.224447
O	5.688293	0.793468	1.380877
C	5.934989	0.291530	2.720355
C	7.002540	−0.788334	2.696706
O	6.506379	−1.974127	1.996019
C	7.426976	−2.313129	0.928850
C	8.331963	−0.405935	2.005772
C	8.170716	−1.017209	0.613522
O	9.392712	−1.011094	2.811496
H	5.007644	−0.126320	3.131077
H	6.268200	1.111325	3.368240
H	7.207991	−1.066946	3.737198
H	8.114946	−3.094442	1.263443
H	8.496009	0.674125	1.976015
H	7.538240	−0.351018	0.020711
H	9.114532	−1.172942	0.095582
N	6.698154	−2.899126	−0.180002
C	5.834117	−2.296177	−1.088867
N	5.361861	−3.148267	−1.981845
C	5.943757	−4.375453	−1.656427
C	5.840456	−5.660150	−2.272738
O	5.152413	−6.016671	−3.243076
N	6.698017	−6.601636	−1.626654
C	7.515187	−6.344327	−0.544824
N	8.287112	−7.358438	−0.054787
N	7.560549	−5.157485	0.045668
C	6.782027	−4.230938	−0.542937
H	5.604842	−1.242622	−1.035265
H	6.693571	−7.533702	−2.034634
H	8.469920	−8.146828	−0.666893
H	9.106167	−7.024223	0.458493
P	10.896209	−1.416872	2.268808
O	11.297522	−0.574759	1.080991
O	11.752513	−1.482896	3.512974
O	10.673877	−2.931200	1.659707
C	10.547641	−4.089005	2.522758

C	11.371570	−5.254425	1.981578
O	10.787242	−5.832702	0.771393
C	11.544758	−5.446649	−0.401922
C	12.814084	−4.894190	1.596107
C	12.718453	−4.579819	0.094468
O	13.632753	−6.063872	1.862858
H	9.492651	−4.381991	2.568869
H	10.903215	−3.843434	3.529272
H	11.364741	−6.046212	2.738891
H	11.875166	−6.362424	−0.897323
H	13.184670	−4.037745	2.169035
H	12.507815	−3.518415	−0.039908
H	13.643345	−4.819560	−0.435431
H	14.539962	−5.861679	1.569409
N	10.670969	−4.776424	−1.368410
C	10.220042	−3.460455	−1.361722
N	9.344590	−3.206506	−2.319968
C	9.193994	−4.416787	−2.994796
C	8.381799	−4.811918	−4.080790
N	7.535361	−3.960055	−4.716186
N	8.468423	−6.097118	−4.511356
C	9.304000	−6.941383	−3.875278
N	10.099739	−6.686707	−2.819864
C	10.009598	−5.405277	−2.418408
H	10.560554	−2.756995	−0.617228
H	6.825770	−4.370391	−5.313891
H	7.281271	−3.100297	−4.242757
H	9.331012	−7.959276	−4.261149

(AGC)$^{2-}$

E: −13845.39 kcal mol^{-1}

O	−0.271950	−0.631214	−0.069134
C	−0.198169	−0.139338	1.291113
C	1.251391	−0.081690	1.758435
O	1.811397	−1.427937	1.916777
C	2.833690	−1.671779	0.917535
C	2.221483	0.659482	0.819136
C	2.802288	−0.468523	−0.034352
O	3.201425	1.326640	1.668713
H	−1.207280	−0.812344	−0.267133
H	−0.757075	−0.798754	1.967353
H	−0.622790	0.873164	1.358991
H	1.260732	0.390781	2.746458
H	3.797642	−1.778221	1.424930
H	1.717654	1.415522	0.211271
H	2.122123	−0.649291	−0.867315
H	3.780299	−0.220130	−0.436864
N	2.600666	−2.955068	0.256208
C	1.759573	−3.241577	−0.812607
N	1.843086	−4.499671	−1.215349
C	2.784512	−5.083351	−0.367950
C	3.337798	−6.382242	−0.294594
N	2.982228	−7.384755	−1.134242
N	4.253034	−6.635326	0.676796
C	4.592726	−5.643987	1.524230
N	4.147735	−4.375866	1.540338
C	3.253019	−4.144713	0.564643
H	1.098304	−2.493526	−1.225503
H	3.522125	−8.241968	−1.124787
H	2.444045	−7.174075	−1.965004
H	5.325355	−5.905990	2.285432
P	4.629966	1.942934	1.111541
O	4.603126	2.132471	−0.385799
O	4.950288	3.093948	2.039657
O	5.678453	0.699676	1.384606
C	5.917930	0.302714	2.760215
C	7.006424	−0.756957	2.834692
O	6.565657	−1.987180	2.176557
C	7.441374	−2.284154	1.063997
C	8.356483	−0.395570	2.181353
C	8.218554	−0.996301	0.783121
O	9.383811	−1.042060	2.996789
H	4.993310	−0.106284	3.185843
H	6.222610	1.174722	3.351274
H	7.166421	−0.982124	3.895561
H	8.109291	−3.106741	1.334279
H	8.546351	0.680680	2.164189
H	7.622653	−0.313541	0.173206
H	9.176865	−1.169635	0.296959
N	6.666901	−2.780842	−0.061266
C	5.824692	−2.087802	−0.927109
N	5.330356	−2.860660	−1.878077
C	5.873979	−4.125390	−1.639629
C	5.776358	−5.348148	−2.372824
O	5.115822	−5.603753	−3.394658
N	6.602355	−6.356325	−1.793437
C	7.421107	−6.197927	−0.693662
N	8.195786	−7.248257	−0.298605
N	7.471483	−5.067825	−0.002335
C	6.709088	−4.086107	−0.515416
H	5.629020	−1.034363	−0.793153
H	6.609141	−7.243283	−2.292586
H	8.385907	−7.984044	−0.970484
H	8.993809	−6.967894	0.274568
P	10.923141	−1.347610	2.481876
O	11.323270	−0.399251	1.377806
O	11.738432	−1.473642	3.748534
O	10.786253	−2.824888	1.757650
C	10.633871	−4.026494	2.559619
C	11.348283	−5.207431	1.910183
O	10.644177	−5.678145	0.718547
C	11.434986	−5.442944	−0.475160
C	12.789844	−4.919658	1.455538
C	12.638047	−4.590842	−0.036833
O	13.555913	−6.134555	1.674579
H	9.567191	−4.258137	2.660249
H	11.062670	−3.859604	3.553506
H	11.342525	−6.030853	2.633879
H	11.737239	−6.403653	−0.890333
H	13.233739	−4.090109	2.015802
H	12.435645	−3.524264	−0.153391
H	13.535373	−4.838633	−0.609397
H	14.457145	−5.979421	1.337111
N	10.576641	−4.829959	−1.504341
C	10.153442	−5.612976	−2.625803
O	10.586110	−6.775740	−2.764639
N	9.271859	−5.052810	−3.500293
C	8.826966	−3.800995	−3.323666
N	7.953657	−3.310808	−4.231706
C	9.244504	−2.994260	−2.218055
C	10.096947	−3.560885	−1.325588
H	7.565740	−3.937817	−4.927039
H	7.461609	−2.445364	−4.051024
H	8.868321	−1.988783	−2.075625
H	10.413904	−3.057981	−0.419450

(AGG)$^{2-}$

E: −14408.83 kcal mol^{-1}

O	−0.156855	−0.644615	−0.200941
C	−0.153946	−0.188863	1.173614
C	1.270535	−0.123114	1.710902
O	1.837429	−1.462352	1.896273

C	2.898609	−1.706313	0.939872
C	2.278419	0.632270	0.825529
C	2.935920	−0.484480	0.008575
O	3.191853	1.321525	1.728768
H	−1.079546	−0.829770	−0.448472
H	−0.735184	−0.872444	1.805477
H	−0.595239	0.816133	1.246583
H	1.226777	0.345623	2.699827
H	3.837512	−1.844467	1.484572
H	1.796769	1.375564	0.185106
H	2.336706	−0.656695	−0.886366
H	3.948036	−0.228507	−0.296434
N	2.665736	−2.973126	0.244910
C	1.808050	−3.230339	−0.817801
N	1.845813	−4.490641	−1.221431
C	2.771570	−5.106235	−0.379586
C	3.264402	−6.427947	−0.298813
N	2.841288	−7.424831	−1.116559
N	4.180290	−6.713463	0.662664
C	4.570891	−5.731873	1.499770
N	4.174920	−4.447537	1.516513
C	3.280919	−4.183688	0.548451
H	1.167113	−2.459087	−1.220412
H	3.365774	−8.291924	−1.124909
H	2.327336	−7.186320	−1.955630
H	5.302898	−6.018758	2.252565
P	4.597890	2.020201	1.213665
O	4.544457	2.330985	−0.262766
O	4.893155	3.099932	2.231623
O	5.682195	0.788886	1.372414
C	5.936706	0.283682	2.709159
C	7.012639	−0.787375	2.678154
O	6.519034	−1.979221	1.985859
C	7.442890	−2.324510	0.923473
C	8.332808	−0.395194	1.973806
C	8.172982	−1.025617	0.589873
O	9.406323	−0.974907	2.780660
H	5.013894	−0.142626	3.121583
H	6.265781	1.103838	3.358786
H	7.231004	−1.060890	3.717429
H	8.139578	−3.094171	1.268588
H	8.480887	0.686712	1.929784
H	7.531749	−0.373294	−0.009297
H	9.115898	−1.180699	0.070395
N	6.719391	−2.933737	−0.176398
C	5.847092	−2.354718	−1.094377
N	5.386245	−3.224857	−1.974818
C	5.983755	−4.439401	−1.633432
C	5.903638	−5.732339	−2.235357
O	5.242194	−6.108937	−3.217523
N	6.754674	−6.659613	−1.559717
C	7.548383	−6.382419	−0.464408
N	8.307392	−7.388405	0.062360
N	7.585084	−5.184016	0.100596
C	6.813806	−4.270566	−0.516582
H	5.603236	−1.303661	−1.055292
H	6.751957	−7.601679	−1.945186
H	8.503870	−8.185362	−0.534209
H	9.121290	−7.039723	0.574753
P	10.920641	−1.345636	2.243315
O	11.292549	−0.526913	1.029900
O	11.782644	−1.346768	3.485617
O	10.747795	−2.883448	1.680799
C	10.607306	−4.009194	2.581116
C	11.371536	−5.216989	2.046774
O	10.751902	−5.776165	0.846449
C	11.519740	−5.440062	−0.335205
C	12.828551	−4.933599	1.650493
C	12.743240	−4.635061	0.145423
O	13.589599	−6.138854	1.930071
H	9.544920	−4.263670	2.670730
H	11.003538	−3.749594	3.569161
H	11.331305	−5.999204	2.813122
H	11.795600	−6.377389	−0.823324
H	13.244055	−4.089138	2.210633
H	12.593318	−3.565379	−0.002647
H	13.650332	−4.933805	−0.385240
H	14.503185	−5.987185	1.626040
N	10.683166	−4.728398	−1.305595
C	10.350050	−3.372930	−1.342334
N	9.514638	−3.080955	−2.320591
C	9.265910	−4.293533	−2.961251
C	8.446397	−4.595212	−4.091222
O	7.762042	−3.832115	−4.795930
N	8.473672	−5.989067	−4.376514
C	9.198967	−6.939938	−3.691214
N	9.028933	−8.240622	−4.061409
N	9.988097	−6.642173	−2.664488
C	9.979890	−5.328487	−2.340097
H	10.738852	−2.677124	−0.615029
H	7.879221	−6.282485	−5.148977
H	8.666095	−8.450113	−4.984353
H	9.705229	−8.900930	−3.694120

(AGT)$^{2-}$

E: −14102.98 kcal mol^{-1}

O	−0.300330	−0.415117	−0.083831
C	−0.195744	0.048614	1.284184
C	1.251047	−0.009612	1.759943
O	1.710634	−1.395943	1.889554
C	2.733486	−1.680920	0.901105
C	2.282725	0.679218	0.846306
C	2.773888	−0.468762	−0.036384
O	3.304667	1.238051	1.726852
H	−1.245950	−0.518483	−0.288695
H	−0.805897	−0.577534	1.947589
H	−0.543404	1.088939	1.366850
H	1.286120	0.439032	2.758545
H	3.685268	−1.839039	1.417412
H	1.848017	1.493828	0.261146
H	2.064515	−0.592131	−0.855859
H	3.758800	−0.289257	−0.458322
N	2.447108	−2.944006	0.229857
C	1.564680	−3.192107	−0.814139
N	1.605778	−4.445202	−1.239312
C	2.563402	−5.062823	−0.435090
C	3.092141	−6.373241	−0.401093
N	2.693362	−7.351244	−1.248839
N	4.035361	−6.660550	0.533683
C	4.430229	−5.688402	1.379598
N	4.014383	−4.411394	1.429485
C	3.085550	−4.149865	0.494437
H	0.908198	−2.420308	−1.190640
H	3.195764	−8.230493	−1.258308
H	2.115133	−7.121550	−2.046593
H	5.186891	−5.976376	2.107331
P	4.817264	1.676931	1.233934
O	4.867902	1.918059	−0.255368
O	5.255647	2.739375	2.216445
O	5.686354	0.295879	1.483945
C	5.866439	−0.180677	2.843202
C	7.067287	−1.112512	2.934970
O	6.807223	−2.399315	2.291020
C	7.615666	−2.558857	1.107347
C	8.361746	−0.572806	2.298902

C	8.385018	−1.239054	0.918402
O	9.457508	−0.971325	3.172327
H	4.963640	−0.714068	3.164784
H	6.031322	0.671904	3.512393
H	7.236203	−1.316913	3.997970
H	8.280250	−3.417894	1.241187
H	8.360177	0.517110	2.218393
H	7.866088	−0.588971	0.212770
H	9.398447	−1.401515	0.557238
N	6.764830	−2.897232	−0.037677
C	5.888615	−2.047944	−0.713075
N	5.242544	−2.656355	−1.689700
C	5.701728	−3.972629	−1.662646
C	5.370972	−5.086762	−2.491918
O	4.585190	−5.150538	−3.452461
N	6.089208	−6.248426	−2.087470
C	6.979154	−6.322246	−1.035249
N	7.520275	−7.538439	−0.759446
N	7.295336	−5.272808	−0.285467
C	6.639402	−4.143011	−0.631544
H	5.776880	−1.016194	−0.414340
H	5.878793	−7.089777	−2.620076
H	7.504333	−8.269244	−1.460437
H	8.313117	−7.545110	−0.128166
P	11.040564	−0.821391	2.722142
O	11.201551	0.212642	1.634401
O	11.810646	−0.721663	4.020223
O	11.335629	−2.277130	2.004958
C	11.269431	−3.462394	2.841371
C	11.756629	−4.670628	2.065519
O	10.791775	−5.001035	1.017969
C	11.503502	−5.231109	−0.215663
C	13.130180	−4.496260	1.372555
C	12.773637	−4.377458	−0.115855
O	13.908434	−5.688630	1.660599
H	10.234755	−3.623964	3.167711
H	11.901944	−3.326916	3.726629
H	11.817509	−5.516953	2.762071
H	11.740491	−6.290960	−0.329270
H	13.657060	−3.610603	1.739533
H	12.560436	−3.332199	−0.356185
H	13.570772	−4.737318	−0.770926
H	14.787253	−5.571264	1.255447
N	10.604743	−4.900907	−1.328427
C	10.190711	−5.926116	−2.181237
O	10.553193	−7.102452	−2.077848
N	9.316397	−5.510335	−3.170228
C	8.842049	−4.212610	−3.415179
O	8.072713	−4.008113	−4.366454
C	9.317120	−3.206334	−2.477766
C	8.873612	−1.783023	−2.667668
C	10.155387	−3.602055	−1.486869
H	9.018183	−6.234472	−3.821127
H	9.175288	−1.418113	−3.656808
H	9.313932	−1.130858	−1.910530
H	7.782700	−1.701014	−2.615074
H	10.524613	−2.909205	−0.739786

$(ATA)^{2-}$

E: −13936.92 kcal mol^{-1}

O	−0.294834	−0.361758	−0.023155
C	−0.184634	0.036687	1.365239
C	1.264879	−0.042425	1.827731
O	1.713859	−1.433655	1.952385
C	2.710912	−1.741140	0.950943
C	2.287222	0.644373	0.902861
C	2.809270	−0.507916	0.040793
O	3.297450	1.249037	1.761003
H	−1.241809	−0.421497	−0.239278
H	−0.793873	−0.617460	2.001698
H	−0.529224	1.072997	1.495012
H	1.315740	0.400329	2.827979
H	3.657933	−1.970574	1.449476
H	1.833424	1.431210	0.295018
H	2.153165	−0.615139	−0.823864
H	3.822444	−0.335018	−0.312608
N	2.344195	−2.964204	0.234718
C	1.335518	−3.125682	−0.709118
N	1.276221	−4.355098	−1.195994
C	2.291276	−5.048372	−0.537042
C	2.748860	−6.384379	−0.619933
N	2.206760	−7.295286	−1.457407
N	3.769374	−6.762962	0.195701
C	4.298693	−5.854075	1.038978
N	3.964293	−4.560478	1.186252
C	2.955093	−4.206737	0.369298
H	0.671026	−2.309442	−0.958213
H	2.625147	−8.213638	−1.538600
H	1.514019	−7.014830	−2.138975
H	5.108749	−6.214262	1.670573
P	4.743295	1.796507	1.175379
O	4.660548	2.088710	−0.302816
O	5.189337	2.852931	2.161542
O	5.700578	0.460960	1.314605
C	5.967908	−0.035065	2.653067
C	7.105867	−1.038372	2.625330
O	6.682664	−2.265489	1.950568
C	7.582660	−2.559096	0.857345
C	8.387492	−0.562867	1.904565
C	8.292755	−1.242419	0.535806
O	9.506663	−1.011114	2.723740
H	5.065056	−0.513045	3.052506
H	6.244084	0.799693	3.307951
H	7.350796	−1.290302	3.663867
H	8.290898	−3.335693	1.153679
H	8.434972	0.525449	1.817487
H	7.683295	−0.609630	−0.112902
H	9.260238	−1.388969	0.064638
N	6.804071	−3.146739	−0.244185
C	6.900795	−4.527803	−0.450267
O	7.622896	−5.275858	0.208991
N	6.108580	−4.994960	−1.486558
C	5.227039	−4.269255	−2.298013
O	4.583741	−4.849981	−3.184502
C	5.159052	−2.847976	−1.993085
C	4.256794	−1.983022	−2.828281
C	5.929643	−2.369812	−0.982323
H	6.169499	−5.995779	−1.662897
H	4.522123	−2.067507	−3.888956
H	4.334880	−0.933995	−2.535214
H	3.211537	−2.299782	−2.736663
H	5.897299	−1.327155	−0.688434
P	11.077095	−1.042707	2.216412
O	11.277967	−0.209619	0.973266
O	11.900237	−0.800782	3.462640
O	11.250074	−2.603709	1.727554
C	11.158503	−3.673890	2.700186
C	11.827698	−4.927422	2.154439
O	11.081410	−5.485745	1.030026
C	11.788924	−5.266667	−0.211457
C	13.260513	−4.722308	1.637058
C	13.069383	−4.481784	0.131723
O	13.992902	−5.946818	1.910252
H	10.102986	−3.883548	2.911936
H	11.657239	−3.377047	3.630564

H	11.819692	−5.680686	2.950874
H	12.003607	−6.244158	−0.651350
H	13.752369	−3.874987	2.126697
H	12.938253	−3.414070	−0.045932
H	13.922872	−4.830048	−0.454534
H	14.889050	−5.842298	1.541403
N	10.911346	−4.594250	−1.173642
C	10.639797	−3.237729	−1.308620
N	9.719208	−2.982224	−2.225061
C	9.351440	−4.232926	−2.717818
C	8.404929	−4.638782	−3.685132
N	7.642338	−3.762234	−4.376251
N	8.256929	−5.969798	−3.918133
C	9.021935	−6.838784	−3.226876
N	9.949335	−6.570053	−2.289960
C	10.074750	−5.247539	−2.071059
H	11.131443	−2.497657	−0.694512
H	6.911951	−4.104211	−4.987930
H	7.689409	−2.772907	−4.173455
H	8.863517	−7.891400	−3.456534

(ATC)$^{2-}$

E: −13538.22 kcal mol^{-1}

O	−0.272590	−0.333412	−0.019377
C	−0.180637	0.052309	1.373926
C	1.265367	−0.014573	1.849471
O	1.730473	−1.401791	1.960053
C	2.736177	−1.684968	0.959827
C	2.286935	0.696676	0.942379
C	2.828037	−0.437766	0.069169
O	3.282648	1.304631	1.815355
H	−1.216379	−0.411280	−0.243166
H	−0.787857	−0.615688	1.997752
H	−0.538862	1.083105	1.511064
H	1.301721	0.415474	2.855860
H	3.681600	−1.912162	1.462599
H	1.828053	1.485731	0.341142
H	2.180258	−0.537699	−0.802279
H	3.840762	−0.246568	−0.274754
N	2.388080	−2.901150	0.221959
C	1.416995	−3.052257	−0.762564
N	1.369904	−4.278543	−1.258006
C	2.353227	−4.981640	−0.562308
C	2.804265	−6.320882	−0.633023
N	2.295728	−7.221745	−1.500195
N	3.790026	−6.709796	0.220281
C	4.290715	−5.808379	1.088490
N	3.958198	−4.513370	1.229338
C	2.985383	−4.148804	0.374071
H	0.766797	−2.232353	−1.034829
H	2.678145	−8.158086	−1.539550
H	1.595746	−6.948252	−2.176726
H	5.072271	−6.176886	1.750332
P	4.746124	1.838805	1.262655
O	4.707170	2.114609	−0.220542
O	5.172229	2.903179	2.248779
O	5.690473	0.498826	1.442304
C	5.918340	0.010653	2.790888
C	7.047747	−1.003206	2.792822
O	6.627914	−2.222956	2.102419
C	7.539547	−2.517916	1.022264
C	8.347345	−0.533474	2.103446
C	8.284857	−1.210416	0.731301
O	9.452321	−0.991809	2.934062
H	5.001675	−0.456943	3.170341
H	6.186441	0.846903	3.447148
H	7.264356	−1.261702	3.835816
H	8.224533	−3.315545	1.317628
H	8.400700	0.554433	2.018362
H	7.716114	−0.567193	0.057116
H	9.272695	−1.370053	0.307438
N	6.771183	−3.068585	−0.106961
C	6.869139	−4.442042	−0.360716
O	7.559584	−5.219067	0.298468
N	6.120997	−4.865011	−1.447120
C	5.295166	−4.100176	−2.279981
O	4.698613	−4.639349	−3.223650
C	5.222331	−2.690022	−1.926389
C	4.374882	−1.786777	−2.778409
C	5.942974	−2.256221	−0.860378
H	6.187298	−5.859196	−1.657034
H	4.684588	−1.853815	−3.828116
H	4.461142	−0.746459	−2.458021
H	3.320206	−2.083393	−2.739612
H	5.905604	−1.224222	−0.531140
P	11.031695	−0.887240	2.454866
O	11.186921	0.065479	1.294766
O	11.823240	−0.706893	3.731096
O	11.305490	−2.392945	1.839312
C	11.264358	−3.518768	2.757491
C	11.787778	−4.774090	2.079783
O	10.857687	−5.212672	1.044505
C	11.522788	−5.279516	−0.238727
C	13.166548	−4.634731	1.403079
C	12.828497	−4.481663	−0.085772
O	13.901671	−5.857742	1.678893
H	10.230275	−3.684446	3.083259
H	11.881990	−3.299499	3.636217
H	11.840085	−5.560307	2.843891
H	11.706132	−6.321419	−0.502943
H	13.721297	−3.773185	1.788375
H	12.675110	−3.424285	−0.314555
H	13.619022	−4.866157	−0.735053
H	14.770774	−5.782564	1.243727
N	10.604088	−4.769342	−1.268221
C	10.008115	−5.673542	−2.202207
O	10.297210	−6.887183	−2.155159
N	9.143115	−5.163234	−3.124002
C	8.837779	−3.858370	−3.130431
N	7.969690	−3.418607	−4.065484
C	9.375538	−2.944614	−2.167861
C	10.245036	−3.450735	−1.255460
H	7.601586	−4.062149	−4.755304
H	7.742151	−2.437213	−4.145944
H	9.094341	−1.898712	−2.159177
H	10.679829	−2.851933	−0.463362

(ATG)$^{2-}$

E: −14100.39 kcal mol^{-1}

O	−0.299016	−0.373491	0.003812
C	−0.185914	0.004587	1.397683
C	1.265161	−0.076870	1.854889
O	1.718980	−1.468362	1.957428
C	2.716376	−1.756337	0.950571
C	2.283113	0.627059	0.937936
C	2.806020	−0.510727	0.057008
O	3.293058	1.221560	1.803320
H	−1.246496	−0.428194	−0.211596
H	−0.791378	−0.660869	2.026043
H	−0.533274	1.037732	1.543846
H	1.317303	0.350866	2.861570
H	3.665626	−1.986976	1.444209

H	1.825528	1.421813	0.343366
H	2.145787	−0.608859	−0.805679
H	3.816930	−0.329584	−0.299212
N	2.355021	−2.971589	0.218870
C	1.339309	−3.127357	−0.718082
N	1.286189	−4.349845	−1.223191
C	2.313202	−5.043993	−0.583894
C	2.780696	−6.374815	−0.690109
N	2.237235	−7.278009	−1.535191
N	3.811222	−6.756946	0.110963
C	4.340187	−5.856129	0.963373
N	3.996757	−4.567671	1.132369
C	2.977959	−4.210356	0.329034
H	0.666043	−2.312861	−0.948892
H	2.663691	−8.190780	−1.635699
H	1.537138	−6.992309	−2.207008
H	5.158373	−6.218907	1.582901
P	4.741814	1.769776	1.226228
O	4.660441	2.086345	−0.246958
O	5.193332	2.808349	2.228745
O	5.691019	0.427178	1.343991
C	5.963498	−0.087033	2.674590
C	7.123833	−1.064008	2.633078
O	6.728425	−2.291978	1.944553
C	7.632543	−2.553667	0.846460
C	8.391981	−0.547906	1.915823
C	8.316454	−1.218968	0.541317
O	9.522751	−0.966717	2.734446
H	5.069501	−0.591141	3.061644
H	6.219272	0.741694	3.345069
H	7.377170	−1.322674	3.667952
H	8.356012	−3.320311	1.131928
H	8.407404	0.541897	1.835834
H	7.696822	−0.592877	−0.104152
H	9.288909	−1.340165	0.073085
N	6.863131	−3.142295	−0.261196
C	6.972611	−4.520680	−0.473089
O	7.692334	−5.268056	0.190249
N	6.193039	−4.989411	−1.518722
C	5.295298	−4.270687	−2.320632
O	4.647858	−4.858501	−3.198707
C	5.209173	−2.853202	−2.004435
C	4.285877	−1.995182	−2.823424
C	5.975308	−2.372343	−0.991869
H	6.239623	−5.993704	−1.677589
H	4.547869	−2.061944	−3.886028
H	4.346320	−0.948465	−2.517513
H	3.246985	−2.331912	−2.728801
H	5.925513	−1.333339	−0.687693
P	11.095016	−0.960569	2.232948
O	11.280909	−0.131695	0.984802
O	11.907080	−0.689022	3.480481
O	11.308127	−2.521672	1.759198
C	11.199593	−3.581494	2.742034
C	11.835006	−4.855236	2.203345
O	11.062570	−5.406410	1.093514
C	11.774689	−5.244510	−0.154996
C	13.267400	−4.688414	1.671091
C	13.068263	−4.471303	0.164016
O	13.977779	−5.922270	1.960553
H	10.141355	−3.764668	2.964435
H	11.711911	−3.287557	3.666135
H	11.818978	−5.598734	3.009031
H	11.973391	−6.239389	−0.561929
H	13.780033	−3.842850	2.141851
H	12.947094	−3.405167	−0.030976
H	13.912617	−4.839333	−0.423595
H	14.873175	−5.841351	1.583890
N	10.912651	−4.585987	−1.138011
C	10.642765	−3.224642	−1.282889
N	9.751001	−2.980807	−2.224572
C	9.399883	−4.233936	−2.725359
C	8.474434	−4.597650	−3.749238
O	7.757168	−3.869822	−4.458707
N	8.417910	−6.012541	−3.899717
C	9.168411	−6.932154	−3.194222
N	8.955725	−8.247901	−3.464845
N	10.033283	−6.579143	−2.248129
C	10.106759	−5.242719	−2.054383
H	11.116107	−2.485058	−0.654342
H	7.776354	−6.344809	−4.616893
H	8.518172	−8.516531	−4.338195
H	9.626459	−8.902889	−3.079183

(ATT)$^{2-}$

E: −13796.76 kcal mol^{-1}

O	−0.224907	−0.339630	0.030763
C	−0.116691	0.065123	1.417466
C	1.333421	−0.007350	1.879022
O	1.788298	−1.396920	1.999369
C	2.778848	−1.699951	0.989911
C	2.350498	0.686886	0.953301
C	2.875748	−0.461139	0.088503
O	3.356669	1.304718	1.808066
H	−1.171255	−0.420389	−0.181032
H	−0.723040	−0.589182	2.056347
H	−0.465345	1.100562	1.543242
H	1.383984	0.433135	2.880246
H	3.726374	−1.934641	1.485028
H	1.889562	1.470322	0.346335
H	2.221179	−0.561904	−0.777508
H	3.887230	−0.283022	−0.264448
N	2.411131	−2.917966	0.264179
C	1.446360	−3.064330	−0.727374
N	1.384858	−4.294577	−1.211212
C	2.351677	−5.005368	−0.500857
C	2.789161	−6.349596	−0.560613
N	2.280021	−7.249279	−1.428639
N	3.763826	−6.744372	0.302772
C	4.267639	−5.843329	1.169742
N	3.950087	−4.543410	1.298158
C	2.988432	−4.173180	0.432783
H	0.810439	−2.238254	−1.013612
H	2.653088	−8.189727	−1.459726
H	1.590658	−6.971374	−2.114219
H	5.038767	−6.216836	1.840888
P	4.840036	1.777850	1.252759
O	4.821320	2.027680	−0.235253
O	5.296567	2.844201	2.223113
O	5.735312	0.408010	1.462469
C	5.916077	−0.075065	2.820103
C	7.031374	−1.102742	2.856510
O	6.613370	−2.319290	2.162707
C	7.554100	−2.655223	1.123359
C	8.352457	−0.643824	2.201924
C	8.378556	−1.388071	0.860997
O	9.429582	−1.022036	3.103871
H	4.981741	−0.525521	3.175774
H	6.178530	0.760773	3.479208
H	7.219530	−1.358600	3.905807
H	8.183728	−3.490419	1.436701
H	8.383202	0.438257	2.058356
H	7.911407	−0.756987	0.103222
H	9.392960	−1.621983	0.545315
N	6.799843	−3.156598	−0.043235

C	6.837486	−4.528169	−0.316620
O	7.503438	−5.344342	0.320948
N	6.059431	−4.905025	−1.400212
C	5.256228	−4.094726	−2.215243
O	4.610465	−4.600055	−3.143267
C	5.265211	−2.684431	−1.854986
C	4.478390	−1.730155	−2.708575
C	6.004613	−2.298279	−0.783722
H	6.056357	−5.902669	−1.603735
H	4.804189	−1.805624	−3.752706
H	4.614766	−0.698484	−2.377421
H	3.409150	−1.970019	−2.689409
H	6.011476	−1.270462	−0.438006
P	11.013265	−0.751029	2.709688
O	11.129694	0.305114	1.637914
O	11.730660	−0.611447	4.034272
O	11.440976	−2.170814	1.989660
C	11.413764	−3.370150	2.807768
C	11.804082	−4.572127	1.970032
O	10.764342	−4.818867	0.977354
C	11.388345	−5.113960	−0.289159
C	13.144827	−4.447430	1.201922
C	12.721705	−4.353103	−0.272699
O	13.910902	−5.648339	1.485245
H	10.404583	−3.517556	3.210610
H	12.116017	−3.266932	3.643616
H	11.867994	−5.440883	2.639141
H	11.541623	−6.189751	−0.398249
H	13.709740	−3.566031	1.518927
H	12.580104	−3.303493	−0.542784
H	13.458785	−4.792856	−0.948937
H	14.780499	−5.549950	1.055847
N	10.452258	−4.733491	−1.354628
C	9.828563	−5.744897	−2.092351
O	10.036844	−6.950742	−1.927901
N	8.941499	−5.274386	−3.045292
C	8.607715	−3.942352	−3.336288
O	7.798181	−3.691588	−4.241447
C	9.265805	−2.956822	−2.492835
C	8.970573	−1.499850	−2.718255
C	10.138917	−3.401684	−1.553769
H	8.496152	−5.990189	−3.616832
H	9.245305	−1.205387	−3.738389
H	9.526496	−0.874499	−2.015831
H	7.900099	−1.295280	−2.604583
H	10.650736	−2.717360	−0.888559

(CAA)$^{2-}$

E: −13676.70 kcal mol^{-1}

O	−0.279017	−0.314654	−0.265878
C	−0.164910	0.043187	1.134115
C	1.275255	−0.108359	1.606849
O	1.656021	−1.519141	1.692289
C	2.730305	−1.817271	0.757070
C	2.340398	0.563544	0.715929
C	2.852054	−0.589593	−0.152403
O	3.339210	1.124461	1.619279
H	−1.226474	−0.376522	−0.478906
H	−0.805916	−0.602484	1.747630
H	−0.470264	1.088520	1.287743
H	1.333006	0.309556	2.618119
H	3.647658	−2.010810	1.314424
H	1.927184	1.376791	0.113793
H	2.200928	−0.676214	−1.024295
H	3.868842	−0.430782	−0.501730
N	2.429080	−3.075423	0.060391
C	3.147679	−4.264982	0.419136
O	4.003707	−4.211505	1.320820
N	2.856208	−5.413608	−0.255648
C	1.891026	−5.438683	−1.183381
N	1.666844	−6.605715	−1.826394
C	1.099569	−4.285165	−1.496236
C	1.400608	−3.132979	−0.841103
H	2.215092	−7.423477	−1.590528
H	0.938001	−6.690332	−2.521686
H	0.285413	−4.327573	−2.210697
H	0.832013	−2.219055	−0.975399
P	4.837571	1.613814	1.129433
O	4.878130	1.863712	−0.358910
O	5.241190	2.689606	2.113703
O	5.744619	0.261447	1.376959
C	5.961642	−0.193076	2.737991
C	7.195750	−1.080907	2.818161
O	6.967682	−2.379620	2.187683
C	7.733170	−2.490606	0.964393
C	8.456176	−0.510432	2.141801
C	8.398574	−1.126023	0.741087
O	9.591768	−0.941967	2.949338
H	5.084429	−0.757900	3.076487
H	6.104410	0.671812	3.396405
H	7.393822	−1.269767	3.878940
H	8.462968	−3.296961	1.086096
H	8.452635	0.582309	2.106396
H	7.770783	−0.484386	0.122260
H	9.376743	−1.202133	0.273973
N	6.867326	−2.915465	−0.132688
C	6.039028	−2.142119	−0.933759
N	5.382323	−2.852386	−1.838339
C	5.791234	−4.167828	−1.621489
C	5.454224	−5.389851	−2.241717
N	4.611699	−5.465031	−3.302995
N	6.035256	−6.525125	−1.778803
C	6.903575	−6.438233	−0.750961
N	7.294109	−5.338457	−0.082986
C	6.706469	−4.227654	−0.559020
H	5.955402	−1.074747	−0.792492
H	4.264173	−6.375992	−3.578731
H	4.037616	−4.662603	−3.529379
H	7.342486	−7.379431	−0.423982
P	11.150656	−0.990253	2.411144
O	11.356562	−0.083110	1.221878
O	11.997793	−0.851894	3.656744
O	11.271912	−2.524169	1.819849
C	11.200977	−3.637838	2.748294
C	11.839063	−4.876633	2.136679
O	11.020433	−5.410053	1.049328
C	11.700459	−5.268141	−0.219228
C	13.240525	−4.656858	1.542370
C	12.965245	−4.432549	0.049735
O	14.004330	−5.868739	1.785921
H	10.150920	−3.845378	2.987721
H	11.732785	−3.382098	3.672136
H	11.882233	−5.645015	2.917751
H	11.933402	−6.264536	−0.605361
H	13.744474	−3.798628	1.999027
H	12.774366	−3.372113	−0.124163
H	13.800209	−4.746088	−0.581380
H	14.881886	−5.750186	1.378406
N	10.788433	−4.672747	−1.193682
C	10.369164	−3.350415	−1.263480
N	9.464817	−3.140904	−2.205847
C	9.266735	−4.391266	−2.790772
C	8.419521	−4.840324	−3.827099
N	7.580237	−4.014087	−4.506703
N	8.467687	−6.152545	−4.172196

C	9.298359	−6.971883	−3.499639
N	10.127584	−6.665243	−2.484593
C	10.076633	−5.358360	−2.170235
H	10.759245	−2.607729	−0.582369
H	6.842584	−4.448403	−5.051376
H	7.367659	−3.109268	−4.102098
H	9.292785	−8.013928	−3.815722

(CAC)$^{2-}$

E: −13277.97 kcal mol^{-1}

O	−0.297132	−0.279667	−0.243459
C	−0.175824	0.083447	1.154531
C	1.264823	−0.075476	1.623722
O	1.638879	−1.488605	1.706018
C	2.708804	−1.788321	0.765831
C	2.331634	0.590287	0.730488
C	2.820317	−0.563540	−0.148584
O	3.344736	1.127952	1.633143
H	−1.245780	−0.334048	−0.453298
H	−0.818769	−0.555570	1.772939
H	−0.474014	1.131339	1.304836
H	1.327224	0.341034	2.635252
H	3.630107	−1.975583	1.318936
H	1.925699	1.414775	0.138654
H	2.149069	−0.647562	−1.005414
H	3.828852	−0.408197	−0.521542
N	2.408707	−3.050412	0.077211
C	3.141516	−4.232733	0.430227
O	3.996339	−4.173461	1.332863
N	2.865901	−5.380746	−0.251832
C	1.897502	−5.414259	−1.176192
N	1.688828	−6.579976	−1.826379
C	1.086525	−4.270840	−1.477072
C	1.376367	−3.117254	−0.819232
H	2.255790	−7.388904	−1.604385
H	0.962080	−6.669589	−2.523293
H	0.268703	−4.321497	−2.186723
H	0.796774	−2.209354	−0.947839
P	4.848243	1.606889	1.151125
O	4.910396	1.821469	−0.342125
O	5.243396	2.703151	2.115921
O	5.753461	0.259772	1.440594
C	5.936610	−0.179856	2.811440
C	7.156256	−1.084123	2.929366
O	6.926336	−2.389882	2.313169
C	7.698719	−2.525547	1.099889
C	8.438798	−0.538854	2.275781
C	8.421173	−1.184125	0.886633
O	9.550801	−0.965724	3.115898
H	5.044482	−0.726838	3.139730
H	6.079555	0.691709	3.461051
H	7.327413	−1.261890	3.996570
H	8.393773	−3.361969	1.221922
H	8.444994	0.552547	2.215751
H	7.851596	−0.537691	0.218245
H	9.420660	−1.306767	0.476920
N	6.829835	−2.905254	−0.014527
C	5.998348	−2.087869	−0.769193
N	5.349571	−2.744582	−1.718358
C	5.768380	−4.066745	−1.584544
C	5.451029	−5.244470	−2.294168
N	4.606872	−5.252510	−3.356820
N	6.049758	−6.402064	−1.916498
C	6.910870	−6.377842	−0.878509
N	7.279300	−5.325768	−0.126900
C	6.680421	−4.189835	−0.524567
H	5.905966	−1.033522	−0.554250
H	4.271055	−6.144751	−3.700329
H	4.025534	−4.441017	−3.525631
H	7.363927	−7.333720	−0.620219
P	11.127320	−0.870282	2.631083
O	11.295886	0.135615	1.518289
O	11.927212	−0.766169	3.910386
O	11.370876	−2.348913	1.938934
C	11.319589	−3.516202	2.803354
C	11.831279	−4.741999	2.066208
O	10.888742	−5.125354	1.018801
C	11.567756	−5.208762	−0.257311
C	13.207129	−4.574953	1.386216
C	12.859801	−4.392314	−0.096652
O	13.955397	−5.796603	1.631960
H	10.284662	−3.686389	3.124358
H	11.941461	−3.345425	3.689767
H	11.888193	−5.563336	2.792211
H	11.769352	−6.251424	−0.506530
H	13.754958	−3.716772	1.788184
H	12.680054	−3.332920	−0.297877
H	13.653854	−4.744601	−0.759922
H	14.823228	−5.701997	1.198022
N	10.651125	−4.721796	−1.296500
C	10.140336	−5.622881	−2.281511
O	10.525595	−6.810259	−2.288878
N	9.245207	−5.138129	−3.187067
C	8.848862	−3.859587	−3.144644
N	7.963986	−3.444145	−4.078354
C	9.336386	−2.940670	−2.159674
C	10.225154	−3.423467	−1.254229
H	7.538088	−4.128226	−4.692611
H	7.527043	−2.535042	−4.003750
H	8.999275	−1.912092	−2.125067
H	10.625195	−2.825053	−0.443902

(CAG)$^{2-}$

E: −13840.87 kcal mol^{-1}

O	−0.247855	−0.394601	−0.294895
C	−0.146375	−0.022245	1.102243
C	1.290357	−0.163114	1.588692
O	1.677541	−1.571406	1.687082
C	2.762184	−1.868823	0.762891
C	2.360393	0.506983	0.702651
C	2.878255	−0.649067	−0.157531
O	3.353917	1.069373	1.611512
H	−1.193352	−0.460357	−0.515319
H	−0.789813	−0.664369	1.716943
H	−0.457145	1.023305	1.243018
H	1.336726	0.262324	2.597462
H	3.677311	−2.046726	1.328823
H	1.951290	1.318767	0.095712
H	2.226831	−0.746134	−1.028142
H	3.894016	−0.488764	−0.508359
N	2.478690	−3.137485	0.078944
C	3.208053	−4.314917	0.455704
O	4.061644	−4.240863	1.358288
N	2.928559	−5.475541	−0.203374
C	1.967745	−5.522336	−1.134542
N	1.755238	−6.701143	−1.760064
C	1.168456	−4.380047	−1.468219
C	1.455781	−3.216417	−0.827143
H	2.312619	−7.509510	−1.513193
H	1.033363	−6.801153	−2.460579
H	0.358852	−4.439318	−2.186629
H	0.880296	−2.309270	−0.977161

P	4.853968	1.560569	1.128616
O	4.898586	1.817418	−0.358462
O	5.254421	2.631547	2.119358
O	5.761417	0.207727	1.371407
C	5.981887	−0.253308	2.729665
C	7.241806	−1.104649	2.811242
O	7.058370	−2.411899	2.184533
C	7.808218	−2.495186	0.949422
C	8.483683	−0.493850	2.135933
C	8.457853	−1.119862	0.738118
O	9.630860	−0.867831	2.955913
H	5.118869	−0.845906	3.056622
H	6.095570	0.609358	3.396492
H	7.444298	−1.286470	3.872345
H	8.545390	−3.297640	1.049557
H	8.436796	0.597544	2.090588
H	7.835292	−0.488904	0.103435
H	9.444906	−1.185770	0.289037
N	6.932225	−2.908724	−0.146168
C	6.087942	−2.126539	−0.923550
N	5.414696	−2.826110	−1.823006
C	5.828638	−4.143053	−1.630918
C	5.474985	−5.358263	−2.255031
N	4.607801	−5.423845	−3.296711
N	6.055915	−6.501309	−1.809623
C	6.933438	−6.428069	−0.788486
N	7.339670	−5.336188	−0.117356
C	6.759449	−4.216618	−0.582238
H	6.004638	−1.061562	−0.765951
H	4.236544	−6.329649	−3.557579
H	4.044703	−4.611536	−3.515259
H	7.368075	−7.375012	−0.472643
P	11.191602	−0.900825	2.423637
O	11.393207	−0.009757	1.221461
O	12.029480	−0.728605	3.671701
O	11.337217	−2.442063	1.859537
C	11.213957	−3.536915	2.804184
C	11.760455	−4.817935	2.192515
O	10.902111	−5.279432	1.103449
C	11.608358	−5.228996	−0.158306
C	13.177028	−4.702310	1.602645
C	12.925526	−4.477795	0.106583
O	13.855653	−5.959970	1.865124
H	10.157639	−3.675308	3.065259
H	11.777310	−3.305343	3.716058
H	11.746797	−5.588944	2.972482
H	11.776329	−6.251483	−0.508496
H	13.736812	−3.875518	2.052271
H	12.806059	−3.408993	−0.079478
H	13.740408	−4.853222	−0.517036
H	14.742238	−5.904526	1.463650
N	10.759024	−4.600498	−1.165561
C	10.459610	−3.245224	−1.299946
N	9.605401	−3.007870	−2.276819
C	9.314113	−4.260781	−2.815824
C	8.457159	−4.631965	−3.895771
O	7.760569	−3.912764	−4.634495
N	8.462648	−6.042031	−4.083620
C	9.189554	−6.953974	−3.349035
N	9.022930	−8.270677	−3.647893
N	9.994013	−6.595891	−2.352546
C	10.016758	−5.263037	−2.129916
H	10.894196	−2.508824	−0.639889
H	7.850658	−6.379948	−4.823277
H	8.641568	−8.535075	−4.548454
H	9.693017	−8.913476	−3.241362

$(CAT)^{2-}$

E: −13537.52 kcal mol^{-1}

O	−0.181071	−0.307628	−0.361913
C	−0.112530	0.079439	1.033286
C	1.305342	−0.086488	1.566145
O	1.662441	−1.501698	1.680941
C	2.765026	−1.822218	0.785289
C	2.417109	0.556673	0.712975
C	2.920883	−0.610257	−0.139217
O	3.405084	1.077885	1.653588
H	−1.121063	−0.371023	−0.605092
H	−0.787882	−0.541179	1.635532
H	−0.404578	1.132944	1.152627
H	1.327301	0.341956	2.574459
H	3.662988	−2.006791	1.376019
H	2.050333	1.385953	0.102645
H	2.277830	−0.695136	−1.017155
H	3.942807	−0.471506	−0.479228
N	2.481791	−3.093873	0.107752
C	3.175098	−4.278409	0.528231
O	3.995569	−4.205866	1.461312
N	2.902278	−5.443027	−0.125912
C	1.980349	−5.486447	−1.095993
N	1.775867	−6.669333	−1.715817
C	1.215546	−4.335448	−1.476525
C	1.496084	−3.168220	−0.838639
H	2.314639	−7.481368	−1.440894
H	1.083320	−6.767113	−2.445714
H	0.435601	−4.390623	−2.227341
H	0.943520	−2.253792	−1.024761
P	4.927005	1.547627	1.222718
O	5.034333	1.781743	−0.264834
O	5.304773	2.624908	2.215264
O	5.812410	0.187706	1.516540
C	5.961189	−0.276722	2.882922
C	7.189094	−1.167867	3.018171
O	6.990888	−2.476596	2.398118
C	7.773775	−2.600153	1.191397
C	8.474136	−0.598532	2.390632
C	8.526276	−1.268989	1.011648
O	9.574490	−0.967182	3.271787
H	5.066519	−0.839437	3.175761
H	6.076306	0.583769	3.552447
H	7.344061	−1.346797	4.087633
H	8.450973	−3.451929	1.301213
H	8.446332	0.490655	2.306305
H	8.018244	−0.624395	0.294562
H	9.548056	−1.424706	0.672946
N	6.908439	−2.935982	0.055972
C	6.094791	−2.082210	−0.679299
N	5.411929	−2.703967	−1.626435
C	5.783701	−4.041662	−1.512055
C	5.397203	−5.201904	−2.217068
N	4.533018	−5.170434	−3.261276
N	5.939989	−6.389247	−1.844392
C	6.803601	−6.410759	−0.809192
N	7.238450	−5.377670	−0.065307
C	6.699884	−4.210427	−0.460736
H	6.040002	−1.028467	−0.449541
H	4.141306	−6.044927	−3.590518
H	4.000550	−4.326649	−3.432369
H	7.203489	−7.389260	−0.549637
P	11.154672	−0.769304	2.830795
O	11.295719	0.298727	1.773499
O	11.917379	−0.689265	4.134826
O	11.488451	−2.194641	2.072302
C	11.411927	−3.409558	2.862856
C	11.755710	−4.605434	1.997249

O	10.706444	−4.794166	0.999754
C	11.323725	−5.097740	−0.269741
C	13.099329	−4.506857	1.229310
C	12.677328	−4.374376	−0.242240
O	13.830933	−5.733652	1.490768
H	10.397931	−3.524043	3.264464
H	12.118236	−3.354740	3.699965
H	11.790706	−5.491651	2.645129
H	11.446784	−6.176607	−0.385786
H	13.688954	−3.647556	1.561383
H	12.561169	−3.316670	−0.493191
H	13.401617	−4.820859	−0.927862
H	14.704343	−5.650794	1.065967
N	10.404475	−4.681964	−1.334953
C	9.791663	−5.666896	−2.115455
O	9.988114	−6.878241	−1.981619
N	8.931750	−5.163086	−3.075289
C	8.626544	−3.821567	−3.346858
O	7.856830	−3.540279	−4.277852
C	9.273699	−2.863704	−2.462646
C	8.999037	−1.398671	−2.656964
C	10.115821	−3.340832	−1.512125
H	8.481151	−5.860970	−3.664210
H	9.310723	−1.077673	−3.658353
H	9.536346	−0.798521	−1.918682
H	7.927174	−1.188637	−2.572207
H	10.617952	−2.679902	−0.816383

(CCA)$^{2-}$

E: −13275.76 kcal mol^{-1}

O	−0.313524	−0.205592	−0.242445
C	−0.207842	0.150479	1.158747
C	1.224157	−0.029249	1.646648
O	1.574549	−1.448250	1.737087
C	2.649352	−1.770598	0.813188
C	2.311061	0.618543	0.764897
C	2.807314	−0.545880	−0.096800
O	3.319430	1.153081	1.672793
H	−1.259592	−0.252929	−0.465294
H	−0.867894	−0.482488	1.765159
H	−0.494520	1.201532	1.308551
H	1.280531	0.388005	2.658175
H	3.557385	−1.986204	1.377792
H	1.921228	1.439865	0.158453
H	2.166329	−0.621836	−0.977219
H	3.832176	−0.403967	−0.430207
N	2.326581	−3.020482	0.110063
C	3.038269	−4.220933	0.445160
O	3.897935	−4.190352	1.344685
N	2.741376	−5.352658	−0.254599
C	1.782743	−5.351368	−1.189644
N	1.568091	−6.496231	−1.872781
C	0.991930	−4.189540	−1.472767
C	1.296689	−3.053756	−0.792324
H	2.113204	−7.322086	−1.657545
H	0.840625	−6.560860	−2.571848
H	0.179446	−4.212888	−2.189921
H	0.734301	−2.133169	−0.907837
P	4.805084	1.663146	1.160681
O	4.802907	1.956266	−0.320146
O	5.229602	2.710871	2.165804
O	5.724094	0.308279	1.345620
C	5.966230	−0.181850	2.691588
C	7.159517	−1.124963	2.710049
O	6.836067	−2.392318	2.062468
C	7.658055	−2.584510	0.880931
C	8.423404	−0.594950	2.003404
C	8.342248	−1.238507	0.616419
O	9.557829	−1.033393	2.808820
H	5.075759	−0.712081	3.050781
H	6.169655	0.664122	3.358373
H	7.389930	−1.338990	3.760161
H	8.380007	−3.379561	1.072609
H	8.441570	0.496662	1.946610
H	7.730105	−0.593390	−0.016118
H	9.314947	−1.349640	0.146880
N	6.813514	−3.089685	−0.211686
C	6.814641	−4.497098	−0.493639
O	7.544611	−5.251550	0.174320
N	6.012737	−4.947729	−1.499770
C	5.205574	−4.112222	−2.159793
N	4.454261	−4.620460	−3.163731
C	5.124067	−2.718327	−1.841903
C	5.937355	−2.261719	−0.853097
H	4.433832	−5.622510	−3.308273
H	3.750203	−4.056371	−3.620136
H	4.434315	−2.056809	−2.351802
H	5.916898	−1.233604	−0.512594
P	11.124011	−1.028105	2.291709
O	11.305757	−0.162251	1.068123
O	11.950923	−0.802122	3.538869
O	11.321864	−2.575053	1.765780
C	11.245606	−3.664717	2.718701
C	11.908893	−4.908042	2.143223
O	11.134663	−5.463054	1.035886
C	11.816004	−5.250728	−0.222224
C	13.325935	−4.688459	1.589474
C	13.093922	−4.448987	0.090416
O	14.077628	−5.905721	1.843246
H	10.193588	−3.879835	2.941870
H	11.757023	−3.383290	3.647058
H	11.928810	−5.667360	2.933787
H	12.033336	−6.229502	−0.658157
H	13.821461	−3.836740	2.067342
H	12.941651	−3.382967	−0.082625
H	13.937357	−4.783315	−0.518193
H	14.963979	−5.790622	1.454405
N	10.913634	−4.594943	−1.169993
C	10.585982	−3.247432	−1.260235
N	9.654595	−2.999296	−2.166813
C	9.338953	−4.246817	−2.702040
C	8.411645	−4.657807	−3.682991
N	7.621159	−3.782977	−4.358426
N	8.332785	−5.980115	−3.978392
C	9.130421	−6.840922	−3.316002
N	10.036908	−6.568603	−2.358874
C	10.103934	−5.252235	−2.089209
H	11.051890	−2.511706	−0.621359
H	6.824194	−4.156876	−4.861768
H	7.533312	−2.838900	−4.001814
H	9.024366	−7.889390	−3.590765

(CCC)$^{2-}$

E: −12877.58 kcal mol^{-1}

O	−0.343792	−0.155864	−0.142674
C	−0.216990	0.179367	1.261972
C	1.225670	0.008747	1.721142
O	1.592997	−1.407273	1.786470
C	2.650809	−1.707249	0.836493
C	2.288708	0.680683	0.827946
C	2.782038	−0.467270	−0.057167
O	3.303907	1.221429	1.724662

H	−1.293060	−0.215274	−0.348382
H	−0.858394	−0.470955	1.869988
H	−0.513073	1.224471	1.433579
H	1.296767	0.412846	2.736991
H	3.571059	−1.925591	1.380100
H	1.877233	1.503523	0.237955
H	2.126625	−0.536356	−0.927440
H	3.798808	−0.308504	−0.407580
N	2.324205	−2.948247	0.118963
C	3.048442	−4.149234	0.424293
O	3.911818	−4.130293	1.320596
N	2.759247	−5.267658	−0.299222
C	1.789101	−5.257552	−1.222586
N	1.579040	−6.390066	−1.926981
C	0.981087	−4.099324	−1.470139
C	1.283323	−2.974172	−0.771259
H	2.137314	−7.213210	−1.736646
H	0.842569	−6.449539	−2.616997
H	0.159513	−4.116934	−2.176988
H	0.710382	−2.057341	−0.861546
P	4.796020	1.713007	1.216413
O	4.812581	1.984389	−0.268327
O	5.222403	2.770189	2.210394
O	5.700974	0.351411	1.429368
C	5.906509	−0.134633	2.782824
C	7.091071	−1.087477	2.832153
O	6.773995	−2.351746	2.174514
C	7.618952	−2.551392	1.013859
C	8.375590	−0.564686	2.159619
C	8.334968	−1.214403	0.773199
O	9.489258	−1.005919	2.990849
H	5.003256	−0.655206	3.123008
H	6.102191	0.712233	3.450580
H	7.292260	−1.305292	3.887337
H	8.319769	−3.363401	1.213135
H	8.398634	0.526214	2.097458
H	7.759752	−0.566192	0.109711
H	9.329124	−1.338970	0.351849
N	6.793854	−3.026274	−0.108265
C	6.832181	−4.415549	−0.465365
O	7.561990	−5.191119	0.178717
N	6.068094	−4.825723	−1.516829
C	5.257914	−3.971830	−2.151381
N	4.533634	−4.440872	−3.191035
C	5.142912	−2.598018	−1.763015
C	5.921533	−2.179442	−0.731029
H	4.546564	−5.431098	−3.402101
H	3.845610	−3.859506	−3.649865
H	4.453536	−1.923693	−2.256170
H	5.876144	−1.170635	−0.337919
P	11.065635	−0.915945	2.506702
O	11.233661	0.056266	1.364340
O	11.864961	−0.771351	3.782837
O	11.315880	−2.413292	1.860587
C	11.263654	−3.559400	2.752817
C	11.858400	−4.784631	2.076329
O	10.980609	−5.252612	1.007771
C	11.675245	−5.241352	−0.262242
C	13.246612	−4.574329	1.440557
C	12.937254	−4.388412	−0.050675
O	14.019785	−5.778338	1.697477
H	10.220131	−3.761509	3.023489
H	11.831065	−3.340677	3.664926
H	11.921262	−5.578420	2.831460
H	11.914091	−6.263022	−0.559374
H	13.758885	−3.705090	1.865440
H	12.736508	−3.332759	−0.248940
H	13.760667	−4.712896	−0.691926
H	14.890799	−5.664174	1.275106
N	10.753906	−4.736767	−1.292269
C	10.253709	−5.618897	−2.298687
O	10.650537	−6.803415	−2.332943
N	9.354301	−5.124411	−3.195193
C	8.941735	−3.851491	−3.121757
N	8.049334	−3.423275	−4.040346
C	9.413997	−2.953896	−2.111502
C	10.303388	−3.448410	−1.212899
H	7.645738	−4.084788	−4.692471
H	7.642233	−2.500157	−3.977216
H	9.060260	−1.932438	−2.050785
H	10.683670	−2.868165	−0.379966

(CCG)$^{2-}$

E: −13439.57 kcal mol^{-1}

H: −12986.73 kcal mol^{-1}

G: −13068.88 kcal mol^{-1}

O	−0.291983	−0.247294	−0.246268
C	−0.190109	0.105369	1.156107
C	1.241325	−0.071887	1.646457
O	1.595618	−1.490146	1.732820
C	2.676174	−1.805045	0.812776
C	2.328460	0.582294	0.769475
C	2.830299	−0.578116	−0.094556
O	3.331864	1.118123	1.682418
H	−1.237455	−0.293178	−0.471939
H	−0.849860	−0.530853	1.759462
H	−0.479738	1.155319	1.308102
H	1.294239	0.342260	2.659471
H	3.583369	−2.016323	1.380146
H	1.937538	1.404306	0.164597
H	2.189220	−0.654903	−0.974919
H	3.854805	−0.432589	−0.427804
N	2.362927	−3.055281	0.106667
C	3.086917	−4.250295	0.434802
O	3.950761	−4.214620	1.330158
N	2.795125	−5.383009	−0.265589
C	1.831529	−5.387399	−1.195440
N	1.619563	−6.533659	−1.877172
C	1.031032	−4.230850	−1.473435
C	1.329982	−3.094199	−0.791998
H	2.173437	−7.355096	−1.667732
H	0.889736	−6.602142	−2.573333
H	0.215862	−4.258905	−2.187375
H	0.760518	−2.177354	−0.903379
P	4.824766	1.616810	1.180779
O	4.832110	1.922611	−0.297502
O	5.254790	2.651898	2.196587
O	5.728805	0.251177	1.358045
C	5.967849	−0.249581	2.700880
C	7.184328	−1.162848	2.721454
O	6.897333	−2.438872	2.074891
C	7.716603	−2.607630	0.887389
C	8.433174	−0.596079	2.016895
C	8.381427	−1.249292	0.632605
O	9.579215	−0.983636	2.832471
H	5.086398	−0.804998	3.043614
H	6.143888	0.593248	3.379221
H	7.418759	−1.370608	3.771954
H	8.449391	−3.395191	1.069147
H	8.412207	0.494963	1.951889
H	7.769871	−0.617080	−0.013200
H	9.362487	−1.347072	0.177928
N	6.876461	−3.112151	−0.209229
C	6.887606	−4.516987	−0.501357

O	7.611644	−5.273275	0.172062
N	6.100785	−4.965426	−1.519924
C	5.282336	−4.132216	−2.171279
N	4.525719	−4.640703	−3.167616
C	5.180676	−2.743999	−1.833790
C	5.988849	−2.287856	−0.841891
H	4.539143	−5.636375	−3.348100
H	3.846363	−4.069988	−3.651272
H	4.480695	−2.086190	−2.334595
H	5.954421	−1.264257	−0.489326
P	11.144564	−0.953954	2.314745
O	11.314426	−0.091709	1.086814
O	11.966328	−0.705866	3.561356
O	11.367541	−2.501075	1.799808
C	11.267315	−3.581286	2.761321
C	11.879941	−4.848911	2.183768
O	11.077750	−5.369571	1.080608
C	11.769592	−5.201356	−0.179043
C	13.302642	−4.684036	1.625074
C	13.074782	−4.442122	0.126311
O	14.010576	−5.926629	1.881806
H	10.211817	−3.759390	3.000058
H	11.799892	−3.311737	3.681565
H	11.874788	−5.608351	2.974683
H	11.954247	−6.193999	−0.598283
H	13.830591	−3.849367	2.098277
H	12.956909	−3.372251	−0.048903
H	13.904594	−4.805883	−0.484265
H	14.898634	−5.845516	1.488277
N	10.897955	−4.527184	−1.141002
C	10.645730	−3.161910	−1.276384
N	9.751862	−2.900663	−2.211608
C	9.380804	−4.145888	−2.717370
C	8.454525	−4.491730	−3.747087
O	7.754162	−3.749167	−4.459384
N	8.393574	−5.901519	−3.919249
C	9.120227	−6.834396	−3.211000
N	8.887017	−8.148008	−3.488433
N	9.988083	−6.501248	−2.261188
C	10.076795	−5.167405	−2.056168
H	11.134453	−2.432692	−0.647484
H	7.737021	−6.221087	−4.627987
H	8.467118	−8.400332	−4.375609
H	9.558736	−8.809052	−3.114516

(CCT)²⁻

E: −13136.55 kcal mol⁻¹

O	−0.265512	−0.144442	−0.236891
C	−0.180889	0.202234	1.167972
C	1.239743	0.002366	1.681521
O	1.574227	−1.421246	1.761873
C	2.661020	−1.743461	0.851898
C	2.350361	0.648280	0.828547
C	2.843485	−0.510293	−0.041798
O	3.350441	1.155055	1.762634
H	−1.208056	−0.185383	−0.475532
H	−0.859363	−0.426229	1.758422
H	−0.457659	1.255791	1.318893
H	1.280678	0.407344	2.698717
H	3.557807	−1.971764	1.429158
H	1.983029	1.484883	0.228912
H	2.211419	−0.568110	−0.929911
H	3.872869	−0.373510	−0.362282
N	2.341581	−2.984248	0.132048
C	3.039162	−4.192343	0.469802
O	3.879964	−4.175377	1.387441
N	2.751478	−5.314965	−0.247718
C	1.811915	−5.298966	−1.201970
N	1.604939	−6.435485	−1.900562
C	1.031875	−4.130399	−1.487593
C	1.329646	−3.002816	−0.790951
H	2.145797	−7.264609	−1.687031
H	0.893425	−6.488802	−2.616779
H	0.233850	−4.142406	−2.221152
H	0.775579	−2.077644	−0.909182
P	4.869998	1.610116	1.303368
O	4.933487	1.906436	−0.175285
O	5.300191	2.635488	2.328319
O	5.730576	0.218986	1.514244
C	5.900275	−0.294298	2.863296
C	7.099510	−1.229190	2.930893
O	6.817861	−2.501787	2.274537
C	7.671078	−2.692882	1.119704
C	8.381442	−0.676223	2.278345
C	8.420561	−1.367452	0.909886
O	9.492682	−1.031477	3.151419
H	4.994231	−0.834490	3.163140
H	6.062623	0.541319	3.553703
H	7.288613	−1.441977	3.989382
H	8.351237	−3.524782	1.306161
H	8.354442	0.411291	2.177950
H	7.914157	−0.730196	0.184058
H	9.440785	−1.530482	0.570887
N	6.846553	−3.122187	−0.025749
C	6.848647	−4.502429	−0.414888
O	7.573765	−5.309288	0.197428
N	6.061065	−4.874017	−1.462828
C	5.267759	−3.987464	−2.074398
N	4.509901	−4.420434	−3.103227
C	5.195224	−2.618752	−1.659972
C	5.988704	−2.241915	−0.624523
H	4.512914	−5.401778	−3.351332
H	3.854330	−3.805225	−3.564766
H	4.524376	−1.916291	−2.138524
H	5.967384	−1.241786	−0.207637
P	11.062756	−0.748132	2.718278
O	11.147643	0.299689	1.635092
O	11.809427	−0.589012	4.024941
O	11.490958	−2.170599	2.001945
C	11.486500	−3.362987	2.830833
C	11.895336	−4.567259	2.004243
O	10.858745	−4.843295	1.015706
C	11.476405	−5.079780	−0.267064
C	13.231004	−4.427892	1.233013
C	12.799025	−4.300736	−0.235536
O	13.995390	−5.637738	1.483485
H	10.480623	−3.523634	3.236895
H	12.188429	−3.239912	3.664409
H	11.973290	−5.427412	2.682733
H	11.644508	−6.148700	−0.414261
H	13.800367	−3.555301	1.566657
H	12.643383	−3.245982	−0.475603
H	13.538041	−4.712619	−0.927159
H	14.854940	−5.540591	1.034121
N	10.529617	−4.677406	−1.314176
C	9.890726	−5.674401	−2.058311
O	10.088183	−6.884745	−1.905728
N	9.015370	−5.186973	−3.010634
C	8.680548	−3.852795	−3.275714
O	7.879211	−3.583807	−4.184240
C	9.335221	−2.882271	−2.412236
C	9.026549	−1.423616	−2.603280
C	10.211470	−3.342369	−1.484036
H	8.538498	−5.894105	−3.566544
H	9.287019	−1.105097	−3.619809

H	9.585384	−0.808164	−1.894262
H	7.955522	−1.231156	−2.472197
H	10.720617	−2.670623	−0.804010

(CGA)$^{2-}$

E: −13842.21 kcal mol^{-1}

O	−0.211237	−0.498331	−0.265606
C	−0.156419	−0.111596	1.130314
C	1.275437	−0.180625	1.646846
O	1.722547	−1.567948	1.779493
C	2.841546	−1.837678	0.889996
C	2.328261	0.524441	0.768084
C	2.946651	−0.620053	−0.039724
O	3.263370	1.174624	1.678980
H	−1.147785	−0.608059	−0.506032
H	−0.782418	−0.778241	1.736719
H	−0.517940	0.919537	1.255146
H	1.282927	0.264439	2.648137
H	3.746097	−1.985160	1.481717
H	1.886621	1.287385	0.122300
H	2.361675	−0.764707	−0.950364
H	3.973744	−0.408644	−0.324182
N	2.613324	−3.118381	0.203149
C	3.386730	−4.268532	0.574261
O	4.220109	−4.174537	1.493886
N	3.172915	−5.428464	−0.110540
C	2.222602	−5.505674	−1.049600
N	2.060318	−6.689363	−1.685609
C	1.364084	−4.401258	−1.361687
C	1.597045	−3.233043	−0.707558
H	2.763268	−7.408587	−1.562782
H	1.451813	−6.756452	−2.491193
H	0.559382	−4.489750	−2.082648
H	0.983008	−2.350039	−0.850178
P	4.655661	1.897857	1.159612
O	4.616761	2.150800	−0.328109
O	4.905986	3.024904	2.138488
O	5.774318	0.709763	1.383564
C	6.041696	0.276882	2.742879
C	7.170916	−0.741399	2.772243
O	6.760333	−1.980562	2.115099
C	7.613312	−2.228609	0.968018
C	8.483631	−0.318544	2.080634
C	8.321873	−0.906464	0.678334
O	9.562409	−0.920602	2.864977
H	5.138031	−0.181668	3.163051
H	6.319243	1.140708	3.359104
H	7.373181	−0.969698	3.825331
H	8.327284	−3.017991	1.216116
H	8.627761	0.764934	2.070972
H	7.674184	−0.238840	0.105866
H	9.265601	−1.031414	0.151404
N	6.825008	−2.763524	−0.126234
C	5.987982	−2.115452	−1.027268
N	5.441998	−2.946484	−1.899557
C	5.945513	−4.205974	−1.566347
C	5.750056	−5.490555	−2.160662
O	5.030401	−5.818081	−3.118065
N	6.560833	−6.475446	−1.514845
C	7.417147	−6.252131	−0.455797
N	8.150687	−7.300493	0.026525
N	7.538885	−5.065630	0.120865
C	6.806414	−4.101805	−0.467977
H	5.836036	−1.047252	−0.987187
H	6.496903	−7.409718	−1.912983
H	8.296793	−8.094108	−0.588498
H	8.985918	−7.000068	0.532201
P	11.065250	−1.300251	2.303526
O	11.441810	−0.447847	1.115134
O	11.935376	−1.355680	3.538772
O	10.865290	−2.819355	1.693272
C	10.706039	−3.962428	2.572213
C	11.426706	−5.184307	2.009154
O	10.750836	−5.739056	0.836008
C	11.492760	−5.442264	−0.371429
C	12.872675	−4.926103	1.557356
C	12.732235	−4.632653	0.055886
O	13.624294	−6.142696	1.811400
H	9.638236	−4.187480	2.676191
H	11.125599	−3.731843	3.557723
H	11.402995	−5.962694	2.780106
H	11.752360	−6.391345	−0.846521
H	13.322746	−4.086359	2.097340
H	12.571358	−3.563621	−0.089585
H	13.618970	−4.930682	−0.508437
H	14.528201	−6.006390	1.473141
N	10.630268	−4.750908	−1.331934
C	10.231636	−3.418797	−1.327604
N	9.352436	−3.136909	−2.274765
C	9.143421	−4.345223	−2.937419
C	8.298676	−4.717079	−4.006563
N	7.473966	−3.838166	−4.634850
N	8.329765	−6.007225	−4.429172
C	9.141939	−6.878846	−3.799827
N	9.962285	−6.647968	−2.758054
C	9.927995	−5.361165	−2.365205
H	10.609861	−2.726069	−0.590624
H	6.738474	−4.229729	−5.213930
H	7.249454	−2.973505	−4.154434
H	9.124519	−7.899500	−4.178907

(CGC)$^{2-}$

E: −13442.49 kcal mol^{-1}

O	−0.336247	−0.324034	−0.137595
C	−0.203496	0.047129	1.257249
C	1.247033	−0.079814	1.706274
O	1.651534	−1.484671	1.787521
C	2.717061	−1.764125	0.835690
C	2.284049	0.605644	0.793138
C	2.787071	−0.541081	−0.085970
O	3.300627	1.171351	1.673793
H	−1.286513	−0.389994	−0.336691
H	−0.824451	−0.602810	1.886726
H	−0.521297	1.089168	1.407803
H	1.315632	0.342124	2.715161
H	3.649460	−1.926759	1.378359
H	1.848683	1.416247	0.203127
H	2.108061	−0.646525	−0.934261
H	3.786588	−0.362140	−0.471656
N	2.439305	−3.034461	0.153301
C	3.216325	−4.194916	0.482399
O	4.069915	−4.122140	1.385447
N	2.983687	−5.338256	−0.222982
C	2.007054	−5.392820	−1.136872
N	1.828369	−6.558726	−1.798476
C	1.140128	−4.281371	−1.398693
C	1.395647	−3.127294	−0.728390
H	2.511209	−7.298592	−1.688270
H	1.179116	−6.618392	−2.571717
H	0.313661	−4.353898	−2.096343
H	0.781492	−2.239435	−0.835447
P	4.765148	1.723510	1.149363

O	4.778758	1.924660	−0.346759
O	5.125587	2.850997	2.092169
O	5.746893	0.429583	1.432144
C	5.968301	0.017239	2.805893
C	7.157738	−0.927604	2.901669
O	6.860976	−2.221370	2.289368
C	7.642701	−2.403075	1.086795
C	8.452318	−0.437691	2.225896
C	8.372907	−1.075335	0.836795
O	9.555442	−0.926939	3.045700
H	5.071989	−0.491882	3.181088
H	6.162976	0.898610	3.428532
H	7.342227	−1.108622	3.966393
H	8.334751	−3.236506	1.240216
H	8.512819	0.652653	2.174259
H	7.776038	−0.421720	0.198946
H	9.350929	−1.208945	0.379243
N	6.782572	−2.814954	−0.017758
C	5.931317	−2.028809	−0.791163
N	5.325336	−2.721058	−1.739333
C	5.790902	−4.026843	−1.590217
C	5.527877	−5.204279	−2.353320
O	4.786894	−5.355907	−3.340717
N	6.281151	−6.306821	−1.857945
C	7.164244	−6.271140	−0.799136
N	7.806769	−7.429414	−0.469689
N	7.383763	−5.175444	−0.085016
C	6.690927	−4.101624	−0.519236
H	5.803967	−0.976125	−0.587021
H	6.156188	−7.178834	−2.367783
H	7.899190	−8.153361	−1.173372
H	8.612529	−7.312335	0.136756
P	11.119382	−0.998250	2.521063
O	11.359807	−0.036580	1.382544
O	11.957013	−0.948665	3.779019
O	11.201273	−2.505377	1.854240
C	11.105051	−3.657663	2.734568
C	11.688490	−4.888642	2.057735
O	10.821010	−5.336028	0.970847
C	11.541105	−5.329318	−0.284021
C	13.089153	−4.689086	1.445610
C	12.803774	−4.485081	−0.047788
O	13.845217	−5.903922	1.701265
H	10.052090	−3.838066	2.983227
H	11.659641	−3.460276	3.658980
H	11.728530	−5.689619	2.806551
H	11.779497	−6.351988	−0.578195
H	13.604292	−3.829717	1.886759
H	12.608958	−3.426504	−0.236998
H	13.634627	−4.806468	−0.680947
H	14.724428	−5.793570	1.294886
N	10.642952	−4.818795	−1.331216
C	10.189587	−5.691503	−2.368252
O	10.615871	−6.863534	−2.418760
N	9.293372	−5.199598	−3.269866
C	8.865939	−3.931856	−3.192673
N	7.989062	−3.507168	−4.129303
C	9.307597	−3.038689	−2.164252
C	10.178742	−3.535110	−1.248617
H	7.599229	−4.177903	−4.781052
H	7.525038	−2.613489	−4.032395
H	8.941402	−2.021545	−2.100393
H	10.532900	−2.960452	−0.400049

(CGG)$^{2-}$

E: −14006.06 kcal mol^{-1}

H: −13540.59 kcal mol^{-1}

G: −13625.33 kcal mol^{-1}

O	−0.183603	−0.599973	−0.290783
C	−0.127295	−0.191796	1.098904
C	1.306349	−0.244147	1.612221
O	1.761936	−1.626558	1.765964
C	2.882211	−1.903421	0.880473
C	2.352752	0.453492	0.719708
C	2.979655	−0.700252	−0.068606
O	3.282056	1.128517	1.618359
H	−1.120022	−0.721084	−0.526165
H	−0.747615	−0.852796	1.717211
H	−0.494613	0.838977	1.209130
H	1.313885	0.217025	2.606217
H	3.787958	−2.036560	1.473725
H	1.904273	1.201070	0.060781
H	2.396213	−0.864114	−0.977007
H	4.005613	−0.488141	−0.356303
N	2.660912	−3.195960	0.213776
C	3.445205	−4.334551	0.597121
O	4.285401	−4.220206	1.508010
N	3.233417	−5.507027	−0.067358
C	2.275856	−5.605512	−0.997063
N	2.114563	−6.800720	−1.610658
C	1.409597	−4.510962	−1.322482
C	1.639650	−3.331173	−0.688467
H	2.812007	−7.521773	−1.469802
H	1.492798	−6.890128	−2.403649
H	0.600603	−4.615945	−2.036349
H	1.019253	−2.454173	−0.840766
P	4.675088	1.843070	1.089200
O	4.638239	2.074622	−0.402015
O	4.924897	2.984089	2.051774
O	5.791378	0.657128	1.331571
C	6.052841	0.237422	2.695993
C	7.205571	−0.753755	2.742551
O	6.830289	−2.010133	2.098036
C	7.676730	−2.239516	0.941843
C	8.509909	−0.305663	2.051898
C	8.367545	−0.907511	0.653151
O	9.600733	−0.868826	2.848026
H	5.155045	−0.239913	3.108033
H	6.303131	1.110585	3.310922
H	7.407288	−0.966206	3.798970
H	8.400448	−3.023277	1.179354
H	8.626287	0.781128	2.032192
H	7.715116	−0.252064	0.072298
H	9.315748	−1.022853	0.133196
N	6.884710	−2.776298	−0.150512
C	6.046594	−2.131072	−1.054613
N	5.487540	−2.966769	−1.912740
C	5.980561	−4.226739	−1.568211
C	5.774524	−5.515793	−2.148265
O	5.070566	−5.846624	−3.117497
N	6.555729	−6.505922	−1.474663
C	7.398927	−6.282511	−0.404533
N	8.093815	−7.340328	0.114430
N	7.546058	−5.087511	0.145085
C	6.842827	−4.119147	−0.470266
H	5.903904	−1.061078	−1.025635
H	6.475853	−7.446195	−1.856714
H	8.227043	−8.151090	−0.480765
H	8.932824	−7.048464	0.618135
P	11.108468	−1.245181	2.299187
O	11.480888	−0.425215	1.086518
O	11.977009	−1.252709	3.537211
O	10.927997	−2.781879	1.733650
C	10.733105	−3.895178	2.640543

C	11.386594	-5.156746	2.083449
O	10.694059	-5.664576	0.900652
C	11.462540	-5.395419	-0.296428
C	12.851383	-4.983317	1.651281
C	12.748745	-4.674214	0.150318
O	13.525573	-6.244100	1.908056
H	9.658489	-4.068679	2.771381
H	11.182586	-3.665952	3.613586
H	11.307926	-5.933957	2.852132
H	11.664035	-6.353779	-0.780885
H	13.344932	-4.175473	2.202021
H	12.664154	-3.596318	0.010026
H	13.619862	-5.029057	-0.405550
H	14.438632	-6.162151	1.577131
N	10.661554	-4.635690	-1.259951
C	10.425582	-3.260543	-1.308844
N	9.598237	-2.921216	-2.279073
C	9.254988	-4.120526	-2.901017
C	8.392899	-4.379318	-4.009579
O	7.749265	-3.580754	-4.713521
N	8.314829	-5.775931	-4.273055
C	8.985220	-6.766084	-3.588180
N	8.714924	-8.056792	-3.933338
N	9.813461	-6.510949	-2.581809
C	9.902866	-5.195965	-2.276048
H	10.873534	-2.585510	-0.595722
H	7.682136	-6.036910	-5.026472
H	8.321762	-8.254256	-4.846533
H	9.351560	-8.756827	-3.568713

(CGT)$^{2-}$

E: −13701.23 kcal mol^{-1}

O	-0.288298	-0.336431	-0.143227
C	-0.173944	0.028201	1.254935
C	1.268408	-0.108581	1.726515
O	1.667702	-1.515438	1.800765
C	2.746874	-1.787244	0.860906
C	2.324521	0.580011	0.838581
C	2.825310	-0.558593	-0.051449
O	3.335392	1.116412	1.745603
H	-1.235901	-0.397555	-0.355937
H	-0.808184	-0.620570	1.872184
H	-0.487962	1.071457	1.405005
H	1.321057	0.303343	2.740510
H	3.672918	-1.949558	1.414049
H	1.908531	1.407122	0.257567
H	2.146032	-0.653507	-0.900848
H	3.825982	-0.383727	-0.434686
N	2.481044	-3.056645	0.173425
C	3.257785	-4.214079	0.512771
O	4.116775	-4.131208	1.409660
N	3.019586	-5.366096	-0.176830
C	2.043315	-5.428411	-1.091027
N	1.858158	-6.603254	-1.733044
C	1.187251	-4.314152	-1.374683
C	1.443472	-3.154242	-0.714801
H	2.514606	-7.359887	-1.584867
H	1.197977	-6.680694	-2.495088
H	0.365575	-4.390168	-2.077524
H	0.833816	-2.265052	-0.835043
P	4.823258	1.640610	1.263786
O	4.871127	1.886920	-0.224894
O	5.198560	2.725744	2.248930
O	5.765404	0.311604	1.518569
C	5.945878	-0.172017	2.874063
C	7.163528	-1.082034	2.963178
O	6.932283	-2.370067	2.313180
C	7.709562	-2.486192	1.102981
C	8.449076	-0.516556	2.331300
C	8.477410	-1.161800	0.940063
O	9.551597	-0.911852	3.198139
H	5.052127	-0.726510	3.185031
H	6.091023	0.678021	3.551518
H	7.334043	-1.289820	4.025165
H	8.375521	-3.348884	1.198262
H	8.433452	0.574400	2.266590
H	7.964718	-0.499005	0.242287
H	9.492651	-1.318837	0.581271
N	6.837068	-2.790232	-0.035444
C	6.001828	-1.912439	-0.725564
N	5.325787	-2.507012	-1.690482
C	5.719216	-3.842692	-1.638646
C	5.322057	-4.955466	-2.438723
O	4.532738	-4.996055	-3.398002
N	5.979043	-6.144288	-2.011144
C	6.872591	-6.240295	-0.964122
N	7.359550	-7.477813	-0.670469
N	7.243377	-5.194342	-0.234664
C	6.644713	-4.039438	-0.603553
H	5.934780	-0.871755	-0.447285
H	5.730101	-6.984353	-2.529028
H	7.310649	-8.211022	-1.367792
H	8.170309	-7.503808	-0.062260
P	11.133085	-0.768005	2.743404
O	11.297101	0.264699	1.654703
O	11.907146	-0.670326	4.039389
O	11.421034	-2.225598	2.026567
C	11.345047	-3.408970	2.864761
C	11.788277	-4.629054	2.081377
O	10.796826	-4.934649	1.051896
C	11.483238	-5.206297	-0.188111
C	13.154657	-4.492876	1.364656
C	12.778838	-4.388015	-0.120407
O	13.912689	-5.696335	1.659671
H	10.313814	-3.547056	3.211964
H	11.998115	-3.287430	3.737008
H	11.840069	-5.475962	2.778294
H	11.688390	-6.274475	-0.285770
H	13.705237	-3.613192	1.710440
H	12.592954	-3.340883	-0.375103
H	13.555633	-4.781280	-0.780749
H	14.789735	-5.600563	1.245144
N	10.578193	-4.870409	-1.293181
C	10.109003	-5.900859	-2.110557
O	10.420975	-7.088428	-1.976439
N	9.243926	-5.475790	-3.103697
C	8.822745	-4.166317	-3.381334
O	8.061110	-3.954728	-4.337160
C	9.340258	-3.157248	-2.469596
C	8.943109	-1.723843	-2.684058
C	10.173838	-3.560057	-1.477796
H	8.910675	-6.204111	-3.732590
H	9.269361	-1.380100	-3.673148
H	9.391805	-1.075676	-1.928124
H	7.854770	-1.610148	-2.647739
H	10.575731	-2.863223	-0.751711

(CTA)$^{2-}$

E: −13535.76 kcal mol^{-1}

O	-0.336692	-0.208074	-0.019461
C	-0.189269	0.097535	1.390016
C	1.262900	-0.072086	1.818822

O	1.638013	−1.487711	1.858803
C	2.688630	−1.768983	0.896831
C	2.299569	0.620365	0.909932
C	2.809204	−0.516325	0.020357
O	3.317006	1.191749	1.783309
H	−1.288929	−0.251818	−0.215532
H	−0.816704	−0.569597	1.994315
H	−0.488474	1.136862	1.588322
H	1.353207	0.319345	2.838105
H	3.614763	−1.996865	1.426500
H	1.858528	1.428925	0.321732
H	2.161858	−0.586300	−0.855559
H	3.828032	−0.347809	−0.319082
N	2.356281	−2.994251	0.152957
C	3.117969	−4.189309	0.374158
O	4.026865	−4.187890	1.225515
N	2.818594	−5.283061	−0.381427
C	1.807411	−5.256331	−1.259812
N	1.579555	−6.367432	−1.990870
C	0.965024	−4.107321	−1.421676
C	1.276314	−3.005750	−0.691268
H	2.213781	−7.153662	−1.917842
H	0.862428	−6.385831	−2.703311
H	0.115068	−4.113254	−2.094165
H	0.685965	−2.095637	−0.720577
P	4.761251	1.751943	1.207014
O	4.681345	2.060155	−0.268191
O	5.201137	2.801272	2.204080
O	5.724823	0.420547	1.336021
C	6.004327	−0.075539	2.672101
C	7.160953	−1.057676	2.639314
O	6.753001	−2.301842	1.990114
C	7.612744	−2.580912	0.862661
C	8.419882	−0.565968	1.891592
C	8.316353	−1.261704	0.530785
O	9.562183	−0.976735	2.697493
H	5.111547	−0.572140	3.071252
H	6.266450	0.762628	3.328272
H	7.426952	−1.290710	3.677022
H	8.327978	−3.362599	1.127862
H	8.441672	0.521840	1.789586
H	7.704622	−0.634466	−0.120406
H	9.281672	−1.409268	0.055388
N	6.800775	−3.156520	−0.223325
C	6.836624	−4.545190	−0.399903
O	7.511438	−5.310045	0.292465
N	6.061320	−4.997349	−1.453657
C	5.226053	−4.252097	−2.294272
O	4.607556	−4.812534	−3.211168
C	5.175707	−2.830819	−1.985465
C	4.292285	−1.951298	−2.825995
C	5.945803	−2.365126	−0.968426
H	6.090619	−6.001684	−1.616564
H	4.559891	−2.041227	−3.885514
H	4.386615	−0.903372	−2.533598
H	3.241243	−2.250018	−2.737173
H	5.928805	−1.321352	−0.677744
P	11.125253	−0.961706	2.168200
O	11.280433	−0.146069	0.907129
O	11.958455	−0.668642	3.396780
O	11.342520	−2.527097	1.709136
C	11.248768	−3.572604	2.709026
C	11.866153	−4.856856	2.175503
O	11.070002	−5.415297	1.085483
C	11.760143	−5.270216	−0.176675
C	13.289857	−4.707243	1.616076
C	13.066340	−4.506396	0.109933
O	13.996401	−5.942286	1.908323
H	10.194439	−3.746710	2.955700
H	11.781616	−3.268510	3.618156
H	11.858359	−5.591120	2.989470
H	11.940931	−6.270585	−0.578815
H	13.816644	−3.859673	2.067119
H	12.953465	−3.441502	−0.095669
H	13.896401	−4.890246	−0.487717
H	14.886438	−5.871674	1.517183
N	10.883446	−4.614012	−1.149706
C	10.638114	−3.257147	−1.326115
N	9.719484	−3.012359	−2.247383
C	9.326319	−4.270484	−2.700547
C	8.373460	−4.688260	−3.656273
N	7.634071	−3.818103	−4.383471
N	8.201817	−6.022338	−3.849893
C	8.949524	−6.884365	−3.131461
N	9.881848	−6.604377	−2.202812
C	10.031739	−5.278571	−2.024107
H	11.146643	−2.508610	−0.736144
H	6.858368	−4.168177	−4.932681
H	7.665397	−2.829898	−4.169405
H	8.770919	−7.940318	−3.328368

(CTC)$^{2-}$

E: −13137.13 kcal mol^{-1}

O	−0.324637	−0.154107	−0.027728
C	−0.195409	0.143605	1.385216
C	1.252823	−0.019431	1.829889
O	1.635655	−1.432888	1.865275
C	2.698568	−1.702482	0.914083
C	2.295610	0.685192	0.937202
C	2.823499	−0.443118	0.047935
O	3.297568	1.260672	1.825925
H	−1.273920	−0.216873	−0.232111
H	−0.825236	−0.531479	1.977996
H	−0.503874	1.179765	1.586573
H	1.329327	0.366264	2.852449
H	3.619207	−1.929605	1.453775
H	1.855793	1.493486	0.347671
H	2.189256	−0.510354	−0.837635
H	3.844898	−0.264046	−0.277304
N	2.381254	−2.924448	0.157531
C	3.136591	−4.121432	0.390884
O	4.025772	−4.124476	1.262794
N	2.854021	−5.211524	−0.376303
C	1.858734	−5.182250	−1.272900
N	1.645013	−6.290856	−2.011777
C	1.019582	−4.032702	−1.446437
C	1.317359	−2.933453	−0.706861
H	2.269249	−7.083035	−1.920310
H	0.933903	−6.311955	−2.730101
H	0.181582	−4.036630	−2.133764
H	0.726795	−2.023836	−0.743651
P	4.756182	1.814961	1.281591
O	4.721269	2.094254	−0.201070
O	5.165356	2.883860	2.270881
O	5.717681	0.488320	1.464904
C	5.960328	0.013353	2.815732
C	7.112047	−0.976273	2.824449
O	6.712570	−2.223858	2.174499
C	7.578645	−2.500536	1.052399
C	8.388458	−0.498302	2.100926
C	8.293739	−1.182092	0.734081
O	9.518281	−0.943298	2.905601
H	5.055356	−0.473195	3.199728
H	6.210894	0.860437	3.465010
H	7.349705	−1.202760	3.870223

H	8.287296	−3.287383	1.320001
H	8.430802	0.589717	2.009806
H	7.695548	−0.546983	0.078682
H	9.270051	−1.327089	0.278937
N	6.779816	−3.064386	−0.048658
C	6.834433	−4.448692	−0.252170
O	7.477188	−5.223819	0.458679
N	6.124102	−4.882160	−1.357760
C	5.328143	−4.124350	−2.224065
O	4.760216	−4.668687	−3.182606
C	5.250004	−2.710275	−1.887758
C	4.404362	−1.819716	−2.754629
C	5.961961	−2.261538	−0.822036
H	6.166677	−5.883503	−1.536387
H	4.708782	−1.908264	−3.804268
H	4.495854	−0.773882	−2.454012
H	3.348235	−2.110582	−2.705030
H	5.924650	−1.223952	−0.511613
P	11.082253	−0.867659	2.376279
O	11.213393	0.052000	1.186717
O	11.914434	−0.663142	3.622825
O	11.321674	−2.391389	1.791635
C	11.287655	−3.496665	2.734157
C	11.804063	−4.765923	2.077149
O	10.866155	−5.222782	1.056190
C	11.518906	−5.302968	−0.232539
C	13.177838	−4.640331	1.387822
C	12.828407	−4.507102	−0.100198
O	13.911531	−5.861467	1.674645
H	10.256431	−3.653894	3.072821
H	11.913885	−3.259966	3.602230
H	11.860059	−5.537967	2.855091
H	11.695382	−6.347607	−0.490256
H	13.737894	−3.774945	1.756746
H	12.675080	−3.452839	−0.343408
H	13.612738	−4.902829	−0.750352
H	14.775822	−5.796540	1.228527
N	10.592686	−4.796065	−1.257440
C	10.012597	−5.696761	−2.204312
O	10.319629	−6.906818	−2.171862
N	9.140044	−5.187984	−3.119600
C	8.820911	−3.886162	−3.114051
N	7.952626	−3.445852	−4.048017
C	9.350564	−2.974942	−2.144642
C	10.223320	−3.479965	−1.234939
H	7.566217	−4.092063	−4.725073
H	7.689561	−2.471451	−4.098836
H	9.060282	−1.931727	−2.128989
H	10.652633	−2.884225	−0.437146

(CTG)$^{2-}$

E: −13701.43 kcal mol^{-1}

O	−0.135515	−0.459121	−0.357717
C	−0.057435	−0.053757	1.031844
C	1.371805	−0.182380	1.543078
O	1.747104	−1.588153	1.705759
C	2.846649	−1.936157	0.823732
C	2.454768	0.449410	0.643749
C	3.027475	−0.744443	−0.126238
O	3.413975	1.096881	1.529570
H	−1.076728	−0.543090	−0.589991
H	−0.711084	−0.681067	1.650795
H	−0.370400	0.994686	1.141687
H	1.407860	0.283379	2.534285
H	3.739767	−2.132081	1.418751
H	2.042952	1.205528	−0.029207
H	2.448997	−0.878069	−1.042133
H	4.068885	−0.593369	−0.399672
N	2.543395	−3.208794	0.148879
C	3.257282	−4.395892	0.520724
O	4.119930	−4.335505	1.417384
N	2.968262	−5.548993	−0.145857
C	2.011756	−5.579870	−1.083443
N	1.791175	−6.749878	−1.719227
C	1.217673	−4.429651	−1.402865
C	1.516306	−3.273125	−0.756344
H	2.386325	−7.546139	−1.526139
H	1.112200	−6.819832	−2.465113
H	0.408762	−4.476738	−2.122929
H	0.951600	−2.357875	−0.901315
P	4.860181	1.685126	0.985882
O	4.842718	1.882571	−0.510129
O	5.199360	2.823542	1.923205
O	5.875514	0.415077	1.260937
C	6.096746	0.023761	2.641893
C	7.216648	−0.996976	2.727138
O	6.779243	−2.268950	2.154565
C	7.691208	−2.686995	1.115002
C	8.520665	−0.606093	1.995439
C	8.475436	−1.435158	0.709146
O	9.624248	−0.950072	2.883627
H	5.175738	−0.408998	3.051307
H	6.368784	0.904434	3.235805
H	7.436091	−1.159574	3.789235
H	8.352066	−3.470849	1.491471
H	8.568954	0.464582	1.784163
H	7.949473	−0.856494	−0.051978
H	9.464019	−1.677749	0.330376
N	6.923565	−3.324672	0.030840
C	6.868736	−4.725470	0.005570
O	7.406615	−5.450236	0.842523
N	6.173404	−5.237353	−1.077557
C	5.439337	−4.535309	−2.042270
O	4.858875	−5.151996	−2.948223
C	5.446471	−3.091520	−1.871785
C	4.677301	−2.253364	−2.853185
C	6.173838	−2.568578	−0.851373
H	6.130562	−6.253431	−1.127967
H	5.052710	−2.424932	−3.868508
H	4.774999	−1.189852	−2.622736
H	3.613989	−2.520405	−2.847717
H	6.201626	−1.501112	−0.669231
P	11.202737	−0.899786	2.393946
O	11.377273	−0.013378	1.185080
O	11.997302	−0.666306	3.660613
O	11.451058	−2.434930	1.854799
C	11.263155	−3.534219	2.780124
C	11.643320	−4.842251	2.104250
O	10.758962	−5.113212	0.975405
C	11.490704	−5.035630	−0.269445
C	13.074365	−4.898286	1.543827
C	12.895908	−4.511611	0.069596
O	13.540938	−6.264923	1.702890
H	10.211777	−3.576356	3.088768
H	11.886639	−3.388219	3.671115
H	11.502637	−5.646637	2.836462
H	11.517969	−6.036394	−0.710396
H	13.745511	−4.211368	2.070251
H	12.943736	−3.425495	−0.016907
H	13.660647	−4.950572	−0.575467
H	14.431543	−6.319871	1.310568
N	10.752285	−4.207874	−1.224456
C	10.821046	−2.830793	−1.438696
N	9.979250	−2.418668	−2.368527
C	9.309206	−3.566889	−2.787746

C	8.303847	−3.745310	−3.787510
O	7.770292	−2.902737	−4.531081
N	7.915926	−5.111190	−3.873520
C	8.417898	−6.145591	−3.112460
N	7.849459	−7.370411	−3.275020
N	9.373322	−5.972458	−2.204526
C	9.774692	−4.686958	−2.083701
H	11.483249	−2.195644	−0.868643
H	7.155567	−5.301953	−4.522870
H	7.321402	−7.571372	−4.115826
H	8.335510	−8.154107	−2.853972

(CTT)$^{2-}$

E: −13395.58 kcal mol^{-1}

O	−0.295048	−0.172136	0.000285
C	−0.147452	0.141108	1.408153
C	1.304537	−0.028739	1.837766
O	1.676658	−1.445169	1.882188
C	2.725663	−1.731428	0.919284
C	2.341499	0.659198	0.924986
C	2.846819	−0.481979	0.040018
O	3.359072	1.239582	1.793462
H	−1.247270	−0.221093	−0.194469
H	−0.775869	−0.521781	2.015997
H	−0.445155	1.182001	1.600617
H	1.396043	0.365974	2.855687
H	3.651377	−1.958596	1.449949
H	1.899497	1.464889	0.333359
H	2.197465	−0.551531	−0.834252
H	3.863478	−0.314920	−0.303535
N	2.393471	−2.959432	0.179598
C	3.139549	−4.159932	0.426113
O	4.040744	−4.154502	1.285615
N	2.834596	−5.262849	−0.313711
C	1.834005	−5.238112	−1.204419
N	1.598646	−6.358896	−1.917815
C	1.011956	−4.079496	−1.399194
C	1.325959	−2.970975	−0.680511
H	2.195002	−7.168184	−1.796147
H	0.867693	−6.392265	−2.615365
H	0.170968	−4.085293	−2.082922
H	0.744585	−2.056156	−0.729659
P	4.844549	1.713635	1.245758
O	4.836287	1.959457	−0.243030
O	5.294597	2.784394	2.214673
O	5.741825	0.347297	1.466461
C	5.931435	−0.121144	2.828218
C	7.094715	−1.095128	2.889002
O	6.741421	−2.354159	2.238638
C	7.625813	−2.623920	1.132075
C	8.389109	−0.597958	2.213940
C	8.397942	−1.324740	0.863109
O	9.493453	−0.965264	3.088711
H	5.016268	−0.616258	3.174290
H	6.144637	0.730524	3.484622
H	7.294511	−1.310195	3.945037
H	8.297231	−3.445420	1.389300
H	8.394197	0.486667	2.084751
H	7.891381	−0.695952	0.130367
H	9.409220	−1.518542	0.513407
N	6.832122	−3.124817	−0.009805
C	6.819306	−4.505438	−0.240504
O	7.439225	−5.325239	0.440971
N	6.069891	−4.886036	−1.340247
C	5.296669	−4.076555	−2.182275
O	4.669499	−4.581644	−3.123939
C	5.309266	−2.663883	−1.833615
C	4.528565	−1.715935	−2.699591
C	6.045245	−2.269915	−0.762528
H	6.035733	−5.888215	−1.515771
H	4.861888	−1.798601	−3.740879
H	4.661196	−0.681663	−2.374706
H	3.459232	−1.957061	−2.685875
H	6.053788	−1.238270	−0.429578
P	11.068920	−0.736132	2.640732
O	11.174936	0.285894	1.535265
O	11.828930	−0.572817	3.938686
O	11.447396	−2.185742	1.951187
C	11.415326	−3.358531	2.806822
C	11.805213	−4.588709	2.010400
O	10.766246	−4.869589	1.024392
C	11.383849	−5.140457	−0.250745
C	13.144642	−4.488673	1.239878
C	12.717525	−4.380097	−0.231672
O	13.885849	−5.707162	1.513309
H	10.404143	−3.490352	3.210523
H	12.114563	−3.229643	3.641671
H	11.865635	−5.434791	2.707689
H	11.536118	−6.214269	−0.378267
H	13.728917	−3.620530	1.558932
H	12.576992	−3.327333	−0.489349
H	13.451417	−4.813974	−0.915209
H	14.747376	−5.634103	1.063251
N	10.442930	−4.743082	−1.306716
C	9.805550	−5.743817	−2.047120
O	9.992176	−6.953216	−1.881358
N	8.931529	−5.258603	−3.004611
C	8.625126	−3.921057	−3.300284
O	7.832134	−3.656460	−4.215882
C	9.289961	−2.946728	−2.448660
C	9.018537	−1.485488	−2.675363
C	10.148872	−3.405932	−1.503606
H	8.477516	−5.967020	−3.578609
H	9.300329	−1.197313	−3.695234
H	9.582929	−0.867907	−1.972709
H	7.951136	−1.263844	−2.564889
H	10.664211	−2.731129	−0.831206

(GAA)$^{2-}$

E: −14245.43 kcal mol^{-1}

O	−0.382655	−0.191817	−0.397387
C	−0.255430	0.303825	0.957591
C	1.169113	0.118568	1.464399
O	1.479222	−1.306463	1.621288
C	2.569236	−1.668421	0.733667
C	2.291727	0.684354	0.567791
C	2.684966	−0.529106	−0.277185
O	3.340625	1.143046	1.479424
H	−1.331684	−0.223520	−0.610275
H	−0.934343	−0.239120	1.627953
H	−0.502782	1.374821	1.003647
H	1.227854	0.582903	2.455091
H	3.487325	−1.776688	1.315404
H	1.963470	1.533755	−0.036687
H	1.948510	−0.646447	−1.075410
H	3.678012	−0.447611	−0.714334
N	2.327952	−2.983884	0.166379
C	1.408542	−3.390053	−0.798045
N	1.512901	−4.676811	−1.084816
C	2.550138	−5.148342	−0.275787
C	3.133410	−6.446267	−0.143741
O	2.835767	−7.508891	−0.719723

N	4.208753	−6.426943	0.793140
C	4.639611	−5.330243	1.511327
N	5.676558	−5.489040	2.386660
N	4.065625	−4.140214	1.401546
C	3.058657	−4.106588	0.509405
H	0.693605	−2.698572	−1.223004
H	4.679688	−7.319827	0.921871
H	6.305205	−6.269058	2.222897
H	6.151849	−4.607334	2.596796
P	4.940800	1.291404	1.102709
O	5.138907	1.523305	−0.376284
O	5.490649	2.252735	2.131707
O	5.531145	−0.223534	1.378917
C	5.729085	−0.719641	2.727476
C	7.048122	−1.479619	2.849949
O	7.015766	−2.783903	2.186749
C	7.748905	−2.741231	0.937103
C	8.268674	−0.764888	2.246634
C	8.321022	−1.320505	0.817576
O	9.410260	−1.116999	3.083414
H	4.902631	−1.392619	2.982863
H	5.745321	0.119497	3.431390
H	7.211147	−1.679597	3.913845
H	8.524830	−3.510359	0.979590
H	8.158564	0.322991	2.253302
H	7.686788	−0.694870	0.189665
H	9.325730	−1.308481	0.404005
N	6.879900	−3.129615	−0.176187
C	5.963640	−2.354078	−0.876794
N	5.292512	−3.041073	−1.787930
C	5.778726	−4.343497	−1.682063
C	5.464278	−5.542881	−2.359948
N	4.543926	−5.604430	−3.353523
N	6.136901	−6.669630	−2.013638
C	7.067307	−6.599081	−1.041261
N	7.439573	−5.524809	−0.323326
C	6.765084	−4.419562	−0.684254
H	5.827455	−1.306858	−0.653739
H	4.236463	−6.516755	−3.669864
H	3.907312	−4.827621	−3.481910
H	7.579445	−7.531340	−0.809786
P	10.983404	−1.057413	2.589963
O	11.177296	−0.061111	1.472399
O	11.785892	−0.964766	3.868464
O	11.193622	−2.541650	1.900959
C	11.113281	−3.713803	2.754630
C	11.748851	−4.914033	2.068920
O	10.936725	−5.356171	0.937033
C	11.669494	−5.207502	−0.302093
C	13.163975	−4.673373	1.513013
C	12.917963	−4.373006	0.029285
O	13.914279	−5.902336	1.708265
H	10.060837	−3.931718	2.974779
H	11.639524	−3.520870	3.696838
H	11.773753	−5.733792	2.797294
H	11.923239	−6.199288	−0.688155
H	13.664876	−3.843793	2.022680
H	12.714159	−3.307034	−0.090924
H	13.768364	−4.642462	−0.601607
H	14.799732	−5.771377	1.322223
N	10.793060	−4.605829	−1.301980
C	10.347477	−3.291443	−1.348218
N	9.452391	−3.079167	−2.298566
C	9.289105	−4.319914	−2.914326
C	8.451335	−4.767218	−3.959887
N	7.583155	−3.951393	−4.610360
N	8.535180	−6.069523	−4.336144
C	9.382389	−6.883183	−3.677941
N	10.195221	−6.581189	−2.648389
C	10.112062	−5.283175	−2.305913
H	10.714477	−2.554425	−0.648327
H	6.874567	−4.379978	−5.195357
H	7.363991	−3.049177	−4.205646
H	9.405032	−7.917113	−4.018933

$(GAC)^{2-}$

E: −13846.59 kcal mol^{-1}

O	−0.394673	−0.186640	−0.332768
C	−0.273487	0.298518	1.026366
C	1.151365	0.118732	1.534642
O	1.473388	−1.305485	1.673913
C	2.560158	−1.650496	0.776053
C	2.271095	0.704983	0.648279
C	2.673024	−0.492433	−0.214785
O	3.316501	1.155381	1.567980
H	−1.343334	−0.235216	−0.543599
H	−0.949620	−0.254699	1.690836
H	−0.528486	1.367258	1.080861
H	1.203778	0.570371	2.531481
H	3.479313	−1.770800	1.354368
H	1.938428	1.561794	0.056605
H	1.940022	−0.600140	−1.017390
H	3.666292	−0.397417	−0.648578
N	2.317797	−2.954220	0.181680
C	1.411681	−3.335167	−0.805638
N	1.524269	−4.612362	−1.128795
C	2.553876	−5.104005	−0.321910
C	3.146514	−6.400982	−0.227499
O	2.862520	−7.446159	−0.840744
N	4.214868	−6.404146	0.717795
C	4.627616	−5.329335	1.478529
N	5.656154	−5.509256	2.359004
N	4.044620	−4.140984	1.402773
C	3.047973	−4.084401	0.500477
H	0.699368	−2.633823	−1.218504
H	4.689668	−7.298000	0.824007
H	6.296971	−6.275193	2.177602
H	6.113299	−4.632002	2.618539
P	4.912670	1.335455	1.191241
O	5.106033	1.581617	−0.286006
O	5.447524	2.297186	2.227538
O	5.530913	−0.171386	1.455761
C	5.717435	−0.679989	2.801562
C	7.022459	−1.464185	2.919087
O	6.964649	−2.765275	2.250005
C	7.706932	−2.737404	1.007893
C	8.253945	−0.765216	2.319159
C	8.313150	−1.328488	0.893644
O	9.389555	−1.126736	3.158084
H	4.877935	−1.337994	3.054066
H	5.749537	0.155122	3.509902
H	7.182971	−1.671979	3.981816
H	8.464386	−3.525039	1.050357
H	8.154015	0.323511	2.318685
H	7.704526	−0.692558	0.250450
H	9.326386	−1.340905	0.500283
N	6.837917	−3.098222	−0.116476
C	5.916456	−2.301072	−0.787000
N	5.259306	−2.952620	−1.733094
C	5.763197	−4.251345	−1.686994
C	5.476168	−5.416154	−2.433839
N	4.560603	−5.438544	−3.433213
N	6.167996	−6.548276	−2.146210
C	7.085771	−6.515661	−1.159053
N	7.431009	−5.477598	−0.377878

C	6.743617	−4.364265	−0.687040
H	5.766701	−1.268210	−0.511695
H	4.273001	−6.336829	−3.804003
H	3.910978	−4.666811	−3.522504
H	7.611353	−7.450761	−0.973010
P	10.963468	−0.967620	2.683552
O	11.108170	0.067337	1.594103
O	11.750418	−0.865048	3.971020
O	11.259314	−2.421492	1.960307
C	11.206977	−3.612451	2.791761
C	11.734716	−4.811579	2.023212
O	10.821966	−5.145749	0.934124
C	11.540780	−5.188068	−0.323123
C	13.131605	−4.627110	1.392554
C	12.832314	−4.388063	−0.092210
O	13.866147	−5.861313	1.616232
H	10.170080	−3.797679	3.097135
H	11.818520	−3.463794	3.689479
H	11.766380	−5.660687	2.718158
H	11.743251	−6.222832	−0.602682
H	13.670634	−3.787667	1.843324
H	12.666145	−3.320562	−0.259817
H	13.644205	−4.721870	−0.743344
H	14.746809	−5.756650	1.211526
N	10.661796	−4.658258	−1.372767
C	10.169574	−5.523626	−2.399035
O	10.556765	−6.709375	−2.442157
N	9.290936	−5.007089	−3.302798
C	8.891132	−3.731299	−3.220786
N	8.005647	−3.289984	−4.139957
C	9.370656	−2.844121	−2.202900
C	10.242641	−3.359090	−1.298899
H	7.609533	−3.943011	−4.805178
H	7.601095	−2.366077	−4.072038
H	9.036260	−1.815890	−2.140503
H	10.631017	−2.787028	−0.464274

(GAG)$^{2-}$

E: −14409.53 kcal mol^{-1}

O	−0.403045	−0.229570	−0.325443
C	−0.261195	0.260014	1.030147
C	1.171469	0.083112	1.517011
O	1.495453	−1.340283	1.659192
C	2.575092	−1.686829	0.752679
C	2.277794	0.664439	0.610193
C	2.667439	−0.538891	−0.250867
O	3.336113	1.121982	1.511470
H	−1.354888	−0.281383	−0.520540
H	−0.926749	−0.291380	1.706719
H	−0.516035	1.328713	1.084950
H	1.239372	0.540619	2.510308
H	3.502262	−1.791642	1.320139
H	1.935875	1.517129	0.018030
H	1.919783	−0.654856	−1.038822
H	3.653518	−0.446707	−0.701840
N	2.336520	−3.000174	0.179885
C	1.409704	−3.407313	−0.776894
N	1.517659	−4.692472	−1.069599
C	2.565040	−5.161806	−0.272319
C	3.155553	−6.457382	−0.150368
O	2.857452	−7.519436	−0.727306
N	4.239809	−6.436184	0.776110
C	4.674171	−5.339331	1.492070
N	5.721759	−5.495954	2.355313
N	4.093488	−4.151689	1.392263
C	3.076533	−4.120294	0.511160
H	0.687316	−2.717816	−1.192289
H	4.716505	−7.327156	0.896586
H	6.354305	−6.269962	2.179044
H	6.194826	−4.612304	2.563087
P	4.930910	1.278159	1.116482
O	5.109530	1.531903	−0.361307
O	5.491542	2.225533	2.152484
O	5.528180	−0.239482	1.364065
C	5.743532	−0.750188	2.705050
C	7.079967	−1.481412	2.814402
O	7.070325	−2.790304	2.159057
C	7.787843	−2.740368	0.900818
C	8.277154	−0.740321	2.195405
C	8.340141	−1.311945	0.772657
O	9.431364	−1.040699	3.035447
H	4.933239	−1.444738	2.953962
H	5.743998	0.079729	3.419895
H	7.259374	−1.673004	3.877190
H	8.572884	−3.500591	0.934259
H	8.133784	0.343537	2.187095
H	7.699502	−0.700978	0.136690
H	9.345098	−1.290446	0.360233
N	6.909222	−3.138029	−0.203238
C	5.970711	−2.371005	−0.884654
N	5.289985	−3.062353	−1.784503
C	5.792867	−4.359146	−1.692458
C	5.471727	−5.561557	−2.361628
N	4.523900	−5.634016	−3.328979
N	6.158453	−6.684229	−2.028086
C	7.100335	−6.608231	−1.066505
N	7.477278	−5.531677	−0.355196
C	6.796563	−4.428449	−0.711174
H	5.825264	−1.326865	−0.653186
H	4.203213	−6.551177	−3.617468
H	3.878147	−4.861627	−3.438536
H	7.619178	−7.538168	−0.840694
P	11.001736	−0.984085	2.535405
O	11.188385	−0.029707	1.380478
O	11.805442	−0.835215	3.808386
O	11.222633	−2.490595	1.903576
C	11.105770	−3.631438	2.793505
C	11.699624	−4.869088	2.138619
O	10.883076	−5.292862	1.003702
C	11.639541	−5.201391	−0.227243
C	13.130912	−4.696111	1.598809
C	12.918868	−4.415673	0.106239
O	13.829976	−5.949009	1.828420
H	10.047299	−3.807315	3.021629
H	11.640584	−3.428733	3.729133
H	11.681547	−5.676577	2.880912
H	11.855248	−6.211512	−0.587368
H	13.657095	−3.877363	2.100396
H	12.763789	−3.344732	−0.038221
H	13.765347	−4.734836	−0.506504
H	14.724719	−5.859507	1.452050
N	10.808603	−4.582549	−1.254033
C	10.441091	−3.241196	−1.352142
N	9.587831	−3.018272	−2.333151
C	9.369162	−4.267799	−2.913595
C	8.525145	−4.653920	−3.998294
O	7.778879	−3.953482	−4.706581
N	8.606666	−6.055213	−4.227993
C	9.378705	−6.949005	−3.517069
N	9.276489	−8.263556	−3.850056
N	10.162047	−6.577849	−2.508272
C	10.115658	−5.252548	−2.249084
H	10.829950	−2.503217	−0.665563
H	8.010298	−6.404527	−4.975191
H	8.906563	−8.524859	−4.756272

H	9.971922	−8.885843	−3.454275

(GAT)$^{2-}$

E: −14105.99 kcal mol^{-1}

O	−0.297239	−0.114147	−0.432574
C	−0.215289	0.370990	0.929381
C	1.180246	0.142855	1.496621
O	1.446816	−1.291878	1.650823
C	2.569142	−1.670127	0.812430
C	2.353249	0.688991	0.654583
C	2.753255	−0.526192	−0.183429
O	3.375138	1.112240	1.613306
H	−1.234284	−0.095591	−0.694509
H	−0.938534	−0.155195	1.566118
H	−0.432588	1.448631	0.971539
H	1.207317	0.595642	2.493915
H	3.457291	−1.801821	1.434477
H	2.071343	1.551421	0.045141
H	2.048546	−0.622620	−1.012475
H	3.764316	−0.464137	−0.579456
N	2.327886	−2.974765	0.220599
C	1.423835	−3.351794	−0.769764
N	1.529896	−4.630347	−1.089943
C	2.553814	−5.126134	−0.278523
C	3.135722	−6.427411	−0.178339
O	2.846184	−7.471941	−0.790162
N	4.199511	−6.436640	0.771916
C	4.620245	−5.362340	1.529170
N	5.645387	−5.548498	2.411991
N	4.048239	−4.168641	1.446878
C	3.052347	−4.107621	0.543209
H	0.717819	−2.645982	−1.186395
H	4.667036	−7.333846	0.882059
H	6.274070	−6.326201	2.238591
H	6.117861	−4.675741	2.660906
P	4.987792	1.244300	1.286762
O	5.231552	1.504335	−0.180409
O	5.523345	2.170612	2.354216
O	5.546070	−0.286236	1.542252
C	5.720833	−0.817573	2.880070
C	7.047318	−1.563658	3.001875
O	7.032897	−2.862513	2.325391
C	7.765674	−2.804346	1.079497
C	8.256771	−0.819488	2.410983
C	8.362405	−1.388934	0.988684
O	9.395720	−1.117771	3.267866
H	4.897291	−1.506702	3.098493
H	5.714087	0.001717	3.606975
H	7.210401	−1.774765	4.063429
H	8.528887	−3.587048	1.101249
H	8.107732	0.263260	2.397249
H	7.775944	−0.756225	0.322991
H	9.389023	−1.403066	0.631046
N	6.891337	−3.146363	−0.050261
C	5.973412	−2.334628	−0.709851
N	5.301135	−2.971716	−1.653974
C	5.786660	−4.277288	−1.617753
C	5.471626	−5.434752	−2.365436
N	4.550900	−5.437440	−3.357682
N	6.139427	−6.583027	−2.079674
C	7.054603	−6.572882	−1.090081
N	7.430239	−5.540826	−0.313596
C	6.769893	−4.410155	−0.622587
H	5.839629	−1.301101	−0.429721
H	4.243717	−6.327022	−3.733384
H	3.928722	−4.644903	−3.456283
H	7.555289	−7.520660	−0.900902
P	10.960587	−0.860862	2.805865
O	11.054385	0.216247	1.752569
O	11.735092	−0.761019	4.101155
O	11.330294	−2.271275	2.036069
C	11.281012	−3.494548	2.816456
C	11.726091	−4.664896	1.961384
O	10.738141	−4.899650	0.910653
C	11.424958	−5.073154	−0.348565
C	13.095304	−4.488176	1.260840
C	12.726798	−4.273236	−0.213779
O	13.842640	−5.715777	1.471618
H	10.256683	−3.663066	3.170009
H	11.944775	−3.409535	3.685103
H	11.772066	−5.553675	2.604405
H	11.622011	−6.131650	−0.529212
H	13.651486	−3.639940	1.670839
H	12.554219	−3.208322	−0.390955
H	13.503346	−4.624459	−0.897658
H	14.719640	−5.600608	1.061926
N	10.524662	−4.646028	−1.426210
C	10.002249	−5.618275	−2.284592
O	10.281115	−6.819181	−2.222052
N	9.129559	−5.115901	−3.233525
C	8.724545	−3.786998	−3.418540
O	7.937612	−3.503493	−4.334821
C	9.293700	−2.842770	−2.468720
C	8.919793	−1.391522	−2.585430
C	10.149191	−3.318696	−1.529143
H	8.742023	−5.804344	−3.876040
H	9.211284	−0.997189	−3.566096
H	9.411900	−0.796040	−1.813145
H	7.835612	−1.259311	−2.497077
H	10.590666	−2.670241	−0.781976

(GCA)$^{2-}$

E: −13843.21 kcal mol^{-1}

O	−0.420279	−0.109491	−0.346296
C	−0.310855	0.411742	1.000684
C	1.100887	0.214095	1.539021
O	1.390705	−1.212521	1.720273
C	2.461556	−1.611420	0.829241
C	2.247351	0.751684	0.657810
C	2.626951	−0.471942	−0.177704
O	3.299131	1.178055	1.579641
H	−1.365721	−0.134959	−0.575257
H	−1.010719	−0.107342	1.668189
H	−0.542023	1.486980	1.020537
H	1.145527	0.688440	2.525519
H	3.374577	−1.766868	1.409590
H	1.947984	1.607505	0.047212
H	1.909638	−0.572569	−0.994926
H	3.633292	−0.411729	−0.588135
N	2.165579	−2.908660	0.241875
C	1.224111	−3.257217	−0.725868
N	1.311381	−4.528488	−1.078393
C	2.357858	−5.049949	−0.312870
C	2.962441	−6.344875	−0.292706
O	2.672840	−7.360783	−0.953579
N	4.050294	−6.385051	0.625300
C	4.509400	−5.328398	1.384343
N	5.590260	−5.527133	2.195840
N	3.927063	−4.137076	1.369268
C	2.890520	−4.054440	0.514917
H	0.507366	−2.536884	−1.097111
H	4.546149	−7.273103	0.664756

H	6.230950	−6.271269	1.936890
H	6.057630	−4.650999	2.443475
P	4.884719	1.367134	1.156684
O	5.022096	1.664096	−0.316965
O	5.456091	2.294044	2.205327
O	5.506785	−0.149276	1.353679
C	5.745380	−0.681500	2.684119
C	7.039225	−1.489958	2.736903
O	6.916358	−2.784651	2.068395
C	7.701903	−2.809377	0.842808
C	8.257959	−0.811596	2.085159
C	8.261626	−1.393177	0.666861
O	9.413824	−1.175673	2.897855
H	4.905671	−1.327148	2.965925
H	5.822469	0.144806	3.398885
H	7.246519	−1.697468	3.792257
H	8.487667	−3.556870	0.951221
H	8.171426	0.278410	2.077903
H	7.608850	−0.775408	0.048549
H	9.250510	−1.392603	0.217809
N	6.860241	−3.298765	−0.262616
C	6.971778	−4.677720	−0.654068
O	7.807574	−5.404726	−0.091650
N	6.133110	−5.132343	−1.627818
C	5.221893	−4.327542	−2.184620
N	4.437808	−4.844710	−3.158131
C	5.061410	−2.963356	−1.781767
C	5.888596	−2.504959	−0.805506
H	4.484561	−5.837580	−3.353569
H	3.646062	−4.325588	−3.513468
H	4.294991	−2.329363	−2.210961
H	5.799241	−1.508060	−0.392056
P	10.982026	−1.109332	2.391550
O	11.151544	−0.179666	1.213923
O	11.791041	−0.923555	3.656482
O	11.223906	−2.623631	1.791127
C	11.162770	−3.758210	2.692136
C	11.862144	−4.960898	2.074162
O	11.122021	−5.477292	0.926732
C	11.836221	−5.215530	−0.303796
C	13.288102	−4.694505	1.564345
C	13.086386	−4.397768	0.071576
O	14.053388	−5.909754	1.785785
H	10.113337	−4.007681	2.890744
H	11.654443	−3.507047	3.639629
H	11.878996	−5.754467	2.830780
H	12.087920	−6.176067	−0.761551
H	13.758281	−3.855881	2.088769
H	12.910927	−3.329391	−0.060739
H	13.951719	−4.686042	−0.529805
H	14.945427	−5.766983	1.419828
N	10.948577	−4.550324	−1.257732
C	10.577496	−3.211850	−1.298215
N	9.654692	−2.956265	−2.211394
C	9.390063	−4.190390	−2.803598
C	8.477672	−4.594647	−3.801326
N	7.650412	−3.725179	−4.437649
N	8.446605	−5.906368	−4.149705
C	9.268038	−6.765056	−3.514985
N	10.156080	−6.500961	−2.538505
C	10.177611	−5.194743	−2.218001
H	11.007597	−2.489091	−0.620480
H	6.877177	−4.106285	−4.971112
H	7.531154	−2.796297	−4.052193
H	9.199396	−7.804966	−3.831403

(GCC)$^{2-}$

E: −13445.08 kcal mol^{-1}

O	−0.413255	−0.033782	−0.325646
C	−0.315013	0.468922	1.029353
C	1.090079	0.256200	1.579131
O	1.368652	−1.174489	1.746051
C	2.446456	−1.570210	0.862833
C	2.247432	0.797446	0.714662
C	2.630642	−0.419328	−0.128588
O	3.291419	1.213390	1.650418
H	−1.356907	−0.059489	−0.561653
H	−1.024387	−0.055185	1.682684
H	−0.540360	1.545212	1.061030
H	1.128638	0.718557	2.571494
H	3.351549	−1.739748	1.451402
H	1.957012	1.659528	0.108651
H	1.922875	−0.507235	−0.955602
H	3.641701	−0.358475	−0.526554
N	2.148555	−2.857808	0.254762
C	1.216942	−3.184483	−0.730137
N	1.306449	−4.447915	−1.108731
C	2.344987	−4.986664	−0.344633
C	2.951904	−6.280670	−0.351971
O	2.670315	−7.279691	−1.041254
N	4.032555	−6.341966	0.573437
C	4.480101	−5.305092	1.366149
N	5.550139	−5.524412	2.185130
N	3.896477	−4.113955	1.374257
C	2.869464	−4.010432	0.510854
H	0.504873	−2.455461	−1.093382
H	4.527956	−7.230985	0.597225
H	6.187243	−6.272572	1.930572
H	6.014677	−4.657960	2.467993
P	4.882865	1.397292	1.249550
O	5.043246	1.697231	−0.221151
O	5.445112	2.316485	2.309367
O	5.494857	−0.122908	1.449128
C	5.695471	−0.673746	2.777987
C	6.972007	−1.507846	2.841830
O	6.841174	−2.783855	2.139403
C	7.653874	−2.799785	0.934289
C	8.213824	−0.833488	2.233349
C	8.253027	−1.391911	0.805978
O	9.347425	−1.229562	3.059570
H	4.837406	−1.304308	3.037445
H	5.777260	0.142593	3.503765
H	7.152054	−1.744347	3.895957
H	8.417639	−3.570566	1.039249
H	8.139615	0.257182	2.241292
H	7.641297	−0.750576	0.169438
H	9.263239	−1.404394	0.405675
N	6.826422	−3.238487	−0.205575
C	6.950823	−4.592982	−0.673859
O	7.792614	−5.343083	−0.152397
N	6.119158	−4.999149	−1.675173
C	5.203189	−4.171084	−2.190765
N	4.413955	−4.643417	−3.181635
C	5.042250	−2.827521	−1.723756
C	5.861073	−2.417771	−0.719779
H	4.459353	−5.625848	−3.424693
H	3.622307	−4.107041	−3.511001
H	4.280611	−2.172701	−2.129291
H	5.770499	−1.441798	−0.258928
P	10.922305	−1.023717	2.607685
O	11.049630	0.004611	1.510029
O	11.688771	−0.884276	3.904378
O	11.277286	−2.473487	1.904985
C	11.266440	−3.658816	2.745735

C	11.881243	−4.836595	2.006350
O	11.017508	−5.247751	0.905041
C	11.729569	−5.170614	−0.354249
C	13.274870	−4.581278	1.398478
C	12.981956	−4.320851	−0.084306
O	14.054551	−5.791486	1.601812
H	10.232187	−3.899859	3.020155
H	11.840206	−3.467327	3.660088
H	11.945581	−5.671074	2.716286
H	11.981537	−6.176056	−0.692830
H	13.775631	−3.731808	1.874487
H	12.778229	−3.257410	−0.229794
H	13.814590	−4.607192	−0.731764
H	14.926623	−5.652777	1.188936
N	10.820053	−4.628673	−1.374128
C	10.296320	−5.487746	−2.389336
O	10.679344	−6.675804	−2.448874
N	9.394029	−4.968040	−3.267639
C	8.985599	−3.696627	−3.159274
N	8.065893	−3.252217	−4.042148
C	9.482363	−2.820573	−2.141716
C	10.380890	−3.338604	−1.265708
H	7.663275	−3.894267	−4.713772
H	7.681109	−2.320126	−3.972048
H	9.136006	−1.798293	−2.054605
H	10.772946	−2.775769	−0.426543

(GCG)$^{2-}$

E: −14006.94 kcal mol^{-1}

O	−0.424297	−0.132170	−0.305341
C	−0.311130	0.374212	1.047074
C	1.104140	0.177783	1.576387
O	1.399821	−1.249346	1.742965
C	2.467966	−1.636015	0.843570
C	2.244308	0.727707	0.694319
C	2.624894	−0.486899	−0.153774
O	3.298973	1.150454	1.614485
H	−1.371114	−0.168383	−0.527148
H	−1.004805	−0.156407	1.711825
H	−0.548137	1.447853	1.080254
H	1.151978	0.643207	2.567020
H	3.384086	−1.793682	1.418278
H	1.938457	1.587549	0.092775
H	1.904351	−0.583021	−0.968674
H	3.629312	−0.419973	−0.568076
N	2.173927	−2.928147	0.244457
C	1.229558	−3.269830	−0.722744
N	1.322838	−4.535901	−1.092167
C	2.376561	−5.060533	−0.339073
C	2.990682	−6.351111	−0.340623
O	2.704720	−7.360005	−1.013802
N	4.084005	−6.395872	0.570559
C	4.537856	−5.347540	1.344247
N	5.622786	−5.551720	2.149565
N	3.947214	−4.160443	1.348785
C	2.906732	−4.072828	0.499530
H	0.506816	−2.548916	−1.081196
H	4.585021	−7.281618	0.596721
H	6.274167	−6.277976	1.866137
H	6.081693	−4.675368	2.411702
P	4.881562	1.348715	1.184322
O	5.008989	1.660469	−0.287146
O	5.455131	2.268197	2.238238
O	5.510242	−0.166783	1.364484
C	5.759836	−0.706170	2.690360
C	7.063465	−1.499373	2.732137
O	6.949578	−2.795226	2.064632
C	7.733763	−2.816636	0.837707
C	8.268403	−0.802745	2.072404
C	8.285175	−1.396993	0.659478
O	9.432318	−1.128240	2.890081
H	4.928264	−1.362995	2.970438
H	5.830326	0.116149	3.410447
H	7.282158	−1.704312	3.785803
H	8.522878	−3.560780	0.945285
H	8.157083	0.284836	2.053258
H	7.633049	−0.788360	0.031253
H	9.277009	−1.394006	0.216792
N	6.893973	−3.307085	−0.268530
C	7.012323	−4.682960	−0.668007
O	7.838534	−5.413856	−0.096250
N	6.188447	−5.131991	−1.656890
C	5.269805	−4.329334	−2.205913
N	4.486303	−4.844796	−3.178702
C	5.090894	−2.973206	−1.782824
C	5.913513	−2.516909	−0.802681
H	4.553688	−5.830465	−3.400678
H	3.705538	−4.320642	−3.549806
H	4.316060	−2.343375	−2.203224
H	5.813091	−1.526115	−0.377039
P	10.998409	−1.042041	2.379915
O	11.151371	−0.135557	1.182029
O	11.804949	−0.812228	3.639630
O	11.267275	−2.564543	1.814993
C	11.192432	−3.678874	2.739470
C	11.857016	−4.906533	2.133452
O	11.097696	−5.410097	0.993445
C	11.821241	−5.190312	−0.240479
C	13.288661	−4.685535	1.617670
C	13.090838	−4.397888	0.123098
O	14.019720	−5.920138	1.848642
H	10.140358	−3.901468	2.955501
H	11.700820	−3.420845	3.676481
H	11.854465	−5.692510	2.898353
H	12.051002	−6.166102	−0.676846
H	13.784307	−3.855961	2.133036
H	12.938586	−3.326995	−0.017343
H	13.947786	−4.710703	−0.478191
H	14.912519	−5.808346	1.473963
N	10.956771	−4.521922	−1.212000
C	10.644398	−3.165686	−1.306259
N	9.752949	−2.913918	−2.246423
C	9.445683	−4.157238	−2.798291
C	8.523749	−4.514607	−3.827765
O	7.780809	−3.784203	−4.509965
N	8.519259	−5.920455	−4.036178
C	9.281676	−6.841315	−3.350719
N	9.092407	−8.156656	−3.654017
N	10.140991	−6.497574	−2.397073
C	10.177230	−5.167690	−2.158527
H	11.090488	−2.436522	−0.646304
H	7.862773	−6.250036	−4.740372
H	8.679268	−8.406015	−4.545126
H	9.786380	−8.802036	−3.293458

(GCT)$^{2-}$

E: −13704.35 kcal mol^{-1}

O	−0.323457	0.090508	−0.363853
C	−0.248651	0.581689	0.996653
C	1.124709	0.299579	1.593618
O	1.330582	−1.144811	1.752947
C	2.436812	−1.573223	0.922336

C	2.334017	0.797391	0.774512
C	2.694854	−0.430411	−0.061434
O	3.362874	1.165201	1.747022
H	−1.256498	0.125769	−0.637884
H	−1.005426	0.090245	1.621353
H	−0.421676	1.667463	1.027105
H	1.149284	0.750350	2.591796
H	3.310399	−1.763560	1.549963
H	2.100450	1.673692	0.164327
H	2.006781	−0.491648	−0.907457
H	3.718235	−0.408902	−0.430255
N	2.132623	−2.852907	0.302216
C	1.202689	−3.157261	−0.690981
N	1.284698	−4.415257	−1.088756
C	2.318165	−4.971920	−0.331249
C	2.914937	−6.270227	−0.358832
O	2.626230	−7.256087	−1.064052
N	3.993978	−6.353875	0.565749
C	4.448636	−5.333552	1.376322
N	5.515937	−5.577315	2.191319
N	3.874811	−4.137333	1.403194
C	2.848078	−4.012806	0.540884
H	0.498406	−2.416459	−1.045898
H	4.484174	−7.246032	0.573987
H	6.136669	−6.337677	1.932526
H	6.000049	−4.723447	2.481215
P	4.973673	1.268036	1.394979
O	5.190852	1.597037	−0.061815
O	5.557479	2.122769	2.495744
O	5.489641	−0.290070	1.564132
C	5.688991	−0.881840	2.874913
C	7.007178	−1.649500	2.931596
O	6.938517	−2.929302	2.226493
C	7.718985	−2.897904	1.002023
C	8.204642	−0.903189	2.317607
C	8.296087	−1.477932	0.896305
O	9.357711	−1.190006	3.159323
H	4.860844	−1.568326	3.082654
H	5.710317	−0.093370	3.634275
H	7.207185	−1.883284	3.982490
H	8.494724	−3.661008	1.067816
H	8.049472	0.178404	2.301524
H	7.702805	−0.848507	0.232535
H	9.319941	−1.484742	0.530965
N	6.873410	−3.314504	−0.136699
C	6.983825	−4.662229	−0.626375
O	7.816981	−5.430328	−0.114670
N	6.156290	−5.044192	−1.639149
C	5.245146	−4.201322	−2.141422
N	4.456956	−4.649247	−3.141654
C	5.084920	−2.869657	−1.641757
C	5.906848	−2.482265	−0.632145
H	4.516201	−5.619168	−3.427245
H	3.684055	−4.093908	−3.483066
H	4.325141	−2.204045	−2.032876
H	5.818359	−1.515749	−0.151794
P	10.906388	−0.845663	2.699190
O	10.938709	0.212765	1.623738
O	11.670425	−0.671720	3.993664
O	11.364518	−2.248669	1.964142
C	11.390348	−3.451986	2.777138
C	11.913911	−4.617352	1.959218
O	10.942880	−4.959807	0.923598
C	11.613558	−5.044984	−0.353782
C	13.261852	−4.368834	1.243697
C	12.860901	−4.161418	−0.223173
O	14.070728	−5.561068	1.431057
H	10.376494	−3.677204	3.129345
H	12.041148	−3.299388	3.646514
H	12.022504	−5.477217	2.633037
H	11.879613	−6.082001	−0.567622
H	13.782496	−3.498189	1.653771
H	12.620156	−3.107496	−0.381846
H	13.649386	−4.452895	−0.921349
H	14.925765	−5.409061	0.988365
N	10.666155	−4.654497	−1.405191
C	10.118683	−5.657287	−2.212023
O	10.403418	−6.855779	−2.113294
N	9.230792	−5.190085	−3.162938
C	8.793257	−3.875391	−3.363023
O	7.981914	−3.622663	−4.267853
C	9.358628	−2.902276	−2.441134
C	8.940790	−1.464292	−2.571001
C	10.249469	−3.340710	−1.515783
H	8.822884	−5.902181	−3.765575
H	9.168804	−1.087938	−3.575298
H	9.456789	−0.837226	−1.840366
H	7.859164	−1.356513	−2.427796
H	10.689633	−2.668044	−0.789846

(GGA)$^{2-}$

E: −14410.31 kcal mol^{-1}

O	−0.445520	−0.382512	−0.365665
C	−0.358145	0.157570	0.975423
C	1.068537	0.059586	1.500251
O	1.451760	−1.342602	1.699692
C	2.569540	−1.670337	0.834176
C	2.165476	0.658581	0.595280
C	2.633065	−0.555196	−0.208879
O	3.184715	1.192409	1.497036
H	−1.389148	−0.455199	−0.591754
H	−1.016822	−0.398577	1.654925
H	−0.658099	1.215817	0.987512
H	1.093811	0.553838	2.477851
H	3.486631	−1.714229	1.428346
H	1.797100	1.470494	−0.036904
H	1.915562	−0.737566	−1.011856
H	3.624589	−0.426461	−0.636803
N	2.403932	−3.012099	0.297780
C	1.527869	−3.485198	−0.677106
N	1.720731	−4.764157	−0.952595
C	2.777182	−5.160414	−0.127918
C	3.456945	−6.410299	0.008612
O	3.249499	−7.492293	−0.570675
N	4.522207	−6.310168	0.954848
C	4.855535	−5.184959	1.681871
N	5.894799	−5.260767	2.567832
N	4.193148	−4.044024	1.565924
C	3.201125	−4.082644	0.658944
H	0.775496	−2.845234	−1.117740
H	5.042688	−7.171565	1.106520
H	6.593535	−5.977296	2.397727
H	6.289877	−4.339513	2.774267
P	4.737749	1.540279	1.055440
O	4.850336	1.729991	−0.438066
O	5.185599	2.614856	2.020958
O	5.537853	0.135137	1.376963
C	5.789158	−0.266345	2.748016
C	7.037563	−1.139760	2.853276
O	6.859137	−2.425976	2.186344
C	7.636333	−2.461487	0.959770
C	8.327454	−0.567705	2.235215
C	8.246751	−1.069806	0.790550
O	9.429260	−1.134934	3.015747
H	4.928442	−0.836973	3.117643

H	5.924735	0.621904	3.375544
H	7.193199	−1.348822	3.917075
H	8.406341	−3.224942	1.069667
H	8.387476	0.521949	2.300833
H	7.574070	−0.410100	0.240487
H	9.213564	−1.092760	0.291241
N	6.813725	−2.922949	−0.148027
C	5.878481	−2.254854	−0.933412
N	5.327773	−3.042785	−1.842053
C	5.918622	−4.294924	−1.648634
C	5.758928	−5.538458	−2.335562
O	5.020887	−5.824261	−3.292355
N	6.622195	−6.538477	−1.784774
C	7.509776	−6.358731	−0.742487
N	8.259014	−7.419068	−0.323293
N	7.633062	−5.201185	−0.109562
C	6.841852	−4.228591	−0.598240
H	5.654219	−1.210470	−0.772406
H	6.565327	−7.447783	−2.238653
H	8.393147	−8.195362	−0.962261
H	9.091421	−7.141374	0.199592
P	10.924460	−1.541507	2.452633
O	11.352052	−0.632765	1.324318
O	11.768036	−1.702180	3.696611
O	10.686037	−3.011096	1.741781
C	10.548047	−4.224872	2.524152
C	11.384845	−5.353961	1.925954
O	10.814512	−5.871649	0.681454
C	11.593283	−5.436295	−0.459986
C	12.828278	−4.960947	1.575619
C	12.744811	−4.573995	0.090310
O	13.657322	−6.132963	1.793363
H	9.492973	−4.521641	2.531752
H	10.882473	−4.044766	3.551244
H	11.378179	−6.184466	2.640627
H	11.946523	−6.329056	−0.981551
H	13.182927	−4.129926	2.194133
H	12.514985	−3.511244	0.006695
H	13.680120	−4.769872	−0.439343
H	14.567536	−5.902209	1.532163
N	10.729627	−4.741906	−1.418155
C	10.259199	−3.434062	−1.366940
N	9.382183	−3.160007	−2.318496
C	9.248072	−4.349834	−3.032555
C	8.431727	−4.725828	−4.123024
N	7.571310	−3.869682	−4.730188
N	8.525919	−5.999825	−4.585195
C	9.378352	−6.850150	−3.980185
N	10.180854	−6.613473	−2.926049
C	10.077219	−5.345025	−2.488105
H	10.586971	−2.751174	−0.597325
H	6.869783	−4.264728	−5.346734
H	7.330531	−3.007546	−4.255398
H	9.413454	−7.857191	−4.392990

(GGC)$^{2-}$

E: −14010.99 kcal mol^{-1}

O	−0.449072	−0.239498	−0.308803
C	−0.344044	0.261490	1.045833
C	1.078839	0.103775	1.565870
O	1.417087	−1.315535	1.723539
C	2.526577	−1.651547	0.851086
C	2.195363	0.693765	0.678791
C	2.624051	−0.510100	−0.160733
O	3.228999	1.168519	1.598496
H	−1.394888	−0.281024	−0.533797
H	−1.019995	−0.291070	1.711153
H	−0.609937	1.328176	1.086531
H	1.119687	0.568017	2.557492
H	3.440611	−1.737950	1.444903
H	1.854583	1.536112	0.071518
H	1.898597	−0.647887	−0.965611
H	3.617211	−0.402713	−0.590393
N	2.324658	−2.972294	0.277349
C	1.433130	−3.391615	−0.708085
N	1.597623	−4.663161	−1.030712
C	2.650199	−5.110549	−0.227891
C	3.307215	−6.377038	−0.146139
O	3.078531	−7.429794	−0.769846
N	4.377440	−6.335599	0.797774
C	4.737617	−5.246856	1.566439
N	5.775060	−5.382561	2.445330
N	4.100854	−4.087024	1.495903
C	3.101690	−4.071302	0.594939
H	0.691497	−2.720564	−1.120024
H	4.882054	−7.212150	0.912002
H	6.453668	−6.112874	2.254544
H	6.190025	−4.483495	2.702216
P	4.801113	1.457131	1.187847
O	4.946475	1.691958	−0.296413
O	5.286424	2.471531	2.198416
O	5.531022	0.003761	1.465161
C	5.748500	−0.468713	2.818844
C	7.024666	−1.300747	2.916601
O	6.901956	−2.596698	2.248871
C	7.645097	−2.603208	1.006811
C	8.281367	−0.661171	2.300009
C	8.275449	−1.209751	0.868495
O	9.408214	−1.105243	3.112989
H	4.894605	−1.083811	3.126476
H	5.838414	0.386991	3.497373
H	7.191415	−1.512088	3.977806
H	8.390200	−3.401088	1.062779
H	8.248087	0.431556	2.318971
H	7.647161	−0.558228	0.260155
H	9.270262	−1.239365	0.429130
N	6.775089	−2.971999	−0.110308
C	5.852238	−2.184992	−0.799495
N	5.230404	−2.852624	−1.754365
C	5.753526	−4.144355	−1.696509
C	5.505959	−5.290836	−2.512708
O	4.733598	−5.418679	−3.478398
N	6.308644	−6.392080	−2.096575
C	7.230936	−6.376662	−1.070636
N	7.893277	−7.535563	−0.793267
N	7.457895	−5.302653	−0.326567
C	6.712998	−4.234183	−0.678184
H	5.683840	−1.154379	−0.526608
H	6.179950	−7.246592	−2.634701
H	7.964773	−8.244740	−1.514251
H	8.716537	−7.427231	−0.209217
P	10.974910	−1.137044	2.594759
O	11.210362	−0.135968	1.489588
O	11.802197	−1.116069	3.860168
O	11.084417	−2.620070	1.878407
C	10.981490	−3.805064	2.713662
C	11.602668	−5.003905	2.011815
O	10.786205	−5.412917	0.872838
C	11.553209	−5.334140	−0.352856
C	13.026258	−4.774073	1.467206
C	12.801038	−4.498809	−0.024948
O	13.772237	−6.000990	1.693191
H	9.924627	−4.009196	2.924620
H	11.505740	−3.633842	3.660712
H	11.617065	−5.833486	2.729955

H	11.808870	−6.338986	−0.690185
H	13.522573	−3.937737	1.970167
H	12.611237	−3.432725	−0.170883
H	13.657887	−4.788382	−0.638463
H	14.661590	−5.878564	1.313462
N	10.689918	−4.775396	−1.404635
C	10.252142	−5.608230	−2.481257
O	10.691469	−6.772481	−2.577527
N	9.355124	−5.089344	−3.366558
C	8.917321	−3.828857	−3.242012
N	8.026641	−3.380919	−4.153016
C	9.357191	−2.969134	−2.184674
C	10.223612	−3.495784	−1.281492
H	7.656809	−4.020350	−4.846101
H	7.582746	−2.478689	−4.046365
H	8.990215	−1.954740	−2.088002
H	10.568883	−2.949567	−0.410824

(GGG)$^{2-}$

E: −14574.17 kcal mol^{-1}

O	−0.428116	−0.424058	−0.423526
C	−0.342354	0.134595	0.909934
C	1.079245	0.024258	1.445664
O	1.441369	−1.380805	1.668914
C	2.572448	−1.729071	0.828894
C	2.190099	0.594476	0.537989
C	2.648944	−0.639708	−0.239320
O	3.208097	1.135864	1.437427
H	−1.369095	−0.448533	−0.670716
H	−1.014309	−0.401522	1.592919
H	−0.626404	1.197373	0.904170
H	1.105449	0.533164	2.415704
H	3.482346	−1.756188	1.434485
H	1.835236	1.398712	−0.111597
H	1.930125	−0.834013	−1.038497
H	3.642401	−0.531621	−0.668099
N	2.415362	−3.082948	0.323299
C	1.527439	−3.583721	−0.626436
N	1.727719	−4.866260	−0.879004
C	2.802119	−5.235189	−0.065189
C	3.491540	−6.477501	0.088037
O	3.284364	−7.572216	−0.467518
N	4.565573	−6.351877	1.020119
C	4.905475	−5.208270	1.715628
N	5.956036	−5.262400	2.589484
N	4.238595	−4.071462	1.580338
C	3.230772	−4.137262	0.691627
H	0.761496	−2.959361	−1.066376
H	5.092496	−7.207124	1.184253
H	6.647919	−5.987605	2.427318
H	6.365252	−4.337279	2.752279
P	4.785016	1.402077	1.025612
O	4.937916	1.570960	−0.467171
O	5.262387	2.463722	1.990970
O	5.510017	−0.035468	1.371105
C	5.792956	−0.422281	2.739342
C	7.098565	−1.209121	2.831376
O	7.003724	−2.505441	2.168010
C	7.725324	−2.477279	0.907768
C	8.335652	−0.549784	2.194080
C	8.273744	−1.057788	0.749183
O	9.487530	−1.016947	2.967939
H	4.974659	−1.053247	3.106459
H	5.871221	0.470249	3.370085
H	7.282790	−1.407482	3.892517
H	8.525048	−3.215087	0.967446
H	8.312582	0.541660	2.253400
H	7.574850	−0.424901	0.201233
H	9.239764	−1.038905	0.248736
N	6.871935	−2.942208	−0.178526
C	5.928528	−2.278783	−0.959958
N	5.354155	−3.076896	−1.844311
C	5.935106	−4.331276	−1.639235
C	5.757548	−5.583976	−2.304303
O	5.018249	−5.877347	−3.258771
N	6.604002	−6.588799	−1.734026
C	7.488452	−6.404947	−0.689066
N	8.197022	−7.476763	−0.227485
N	7.644555	−5.233934	−0.092964
C	6.872698	−4.256879	−0.601952
H	5.716041	−1.229670	−0.815556
H	6.518160	−7.510369	−2.158310
H	8.317225	−8.273490	−0.843869
H	9.033474	−7.205768	0.291428
P	10.977270	−1.426770	2.396484
O	11.373128	−0.574560	1.212993
O	11.844999	−1.503493	3.632040
O	10.756623	−2.933009	1.767663
C	10.586209	−4.100026	2.609373
C	11.338074	−5.291760	2.020625
O	10.733246	−5.772407	0.779282
C	11.542746	−5.400457	−0.362739
C	12.807655	−5.008532	1.671916
C	12.758994	−4.628874	0.183691
O	13.546783	−6.237475	1.902328
H	9.518093	−4.337126	2.676250
H	10.974420	−3.897578	3.613676
H	11.270830	−6.113941	2.742247
H	11.825004	−6.320878	−0.879223
H	13.221842	−4.201176	2.285353
H	12.616840	−3.551938	0.090890
H	13.676566	−4.903530	−0.342103
H	14.468850	−6.082370	1.627049
N	10.741542	−4.642949	−1.328934
C	10.423056	−3.283075	−1.325194
N	9.588055	−2.954093	−2.292424
C	9.323282	−4.145535	−2.966470
C	8.479130	−4.411931	−4.087362
O	7.785182	−3.626775	−4.757987
N	8.487658	−5.799133	−4.408025
C	9.215246	−6.773796	−3.758966
N	9.029249	−8.063268	−4.158902
N	10.018951	−6.509388	−2.734996
C	10.028961	−5.204606	−2.377466
H	10.822679	−2.609982	−0.581737
H	7.886499	−6.066113	−5.184962
H	8.650373	−8.248263	−5.080732
H	9.704812	−8.737857	−3.817189

(GGT)$^{2-}$

E: −14269.90 kcal mol^{-1}

O	−0.416198	−0.196829	−0.245135
C	−0.306740	0.276132	1.119020
C	1.112608	0.086600	1.638085
O	1.430175	−1.340785	1.765010
C	2.540181	−1.671064	0.890745
C	2.239576	0.679809	0.766152
C	2.653729	−0.511684	−0.098160
O	3.276439	1.121768	1.699619
H	−1.360221	−0.193761	−0.481582
H	−0.992583	−0.278934	1.772235
H	−0.556240	1.345765	1.180115

H	1.157991	0.528155	2.639745
H	3.450949	−1.777304	1.485217
H	1.912338	1.540080	0.176791
H	1.926316	−0.624026	−0.905370
H	3.648601	−0.412331	−0.525420
N	2.327590	−2.978465	0.293103
C	1.414364	−3.376761	−0.680770
N	1.560274	−4.645667	−1.021481
C	2.621641	−5.113196	−0.242451
C	3.262022	−6.389028	−0.182749
O	3.014494	−7.430710	−0.818259
N	4.341218	−6.373684	0.750752
C	4.728809	−5.297985	1.524938
N	5.771622	−5.461002	2.391107
N	4.112103	−4.125909	1.469893
C	3.099473	−4.088147	0.584063
H	0.672136	−2.693106	−1.070351
H	4.835821	−7.258167	0.848168
H	6.421290	−6.217584	2.203348
H	6.218258	−4.575390	2.642820
P	4.861718	1.362202	1.311863
O	5.031184	1.643967	−0.161966
O	5.376821	2.318421	2.363341
O	5.532958	−0.127372	1.536974
C	5.734469	−0.670484	2.865379
C	7.046528	−1.445542	2.945215
O	6.993946	−2.728386	2.242518
C	7.720210	−2.668603	0.995146
C	8.267289	−0.716491	2.357505
C	8.359493	−1.270077	0.926836
O	9.398678	−1.041387	3.215084
H	4.902839	−1.341584	3.109147
H	5.768512	0.144872	3.596562
H	7.220808	−1.682682	3.999555
H	8.458455	−3.475308	0.998792
H	8.137733	0.368988	2.355644
H	7.792244	−0.612255	0.268362
H	9.385767	−1.307107	0.567933
N	6.831056	−2.960351	−0.138151
C	5.921682	−2.105212	−0.765545
N	5.237970	−2.702670	−1.722143
C	5.698314	−4.017822	−1.731524
C	5.332344	−5.120469	−2.562264
O	4.515326	−5.167123	−3.496764
N	6.055169	−6.291549	−2.193208
C	6.990684	−6.376749	−1.182922
N	7.519623	−7.603804	−0.922305
N	7.350639	−5.333980	−0.443338
C	6.682037	−4.198361	−0.747450
H	5.808269	−1.080123	−0.450035
H	5.816080	−7.129069	−2.720420
H	7.459217	−8.332724	−1.623871
H	8.357019	−7.615239	−0.350689
P	10.974019	−0.873108	2.751382
O	11.123276	0.169188	1.669713
O	11.751246	−0.775238	4.045089
O	11.272564	−2.321679	2.022504
C	11.198668	−3.515015	2.846990
C	11.720278	−4.708310	2.070376
O	10.788155	−5.035270	0.991916
C	11.531808	−5.203588	−0.234856
C	13.107817	−4.505256	1.416188
C	12.783662	−4.333300	−0.074210
O	13.883003	−5.706111	1.676307
H	10.157869	−3.690851	3.144780
H	11.806523	−3.380885	3.749423
H	11.772068	−5.563370	2.756679
H	11.791430	−6.254060	−0.379907
H	13.623503	−3.632487	1.827469
H	12.560405	−3.282221	−0.276008
H	13.600970	−4.654867	−0.724366
H	14.765913	−5.576969	1.283700
N	10.651364	−4.852246	−1.355382
C	10.224789	−5.870407	−2.211314
O	10.565454	−7.052838	−2.104303
N	9.366027	−5.440795	−3.207816
C	8.892333	−4.140584	−3.438738
O	8.120230	−3.925856	−4.385548
C	9.370398	−3.143988	−2.492330
C	8.927762	−1.718475	−2.666499
C	10.207125	−3.550546	−1.504369
H	9.062397	−6.159642	−3.862096
H	9.238196	−1.338356	−3.647292
H	9.358927	−1.076877	−1.894843
H	7.836064	−1.638702	−2.623450
H	10.577660	−2.863157	−0.752938

$(GTA)^{2-}$

E: −14102.73 kcal mol^{-1}

O	−0.506590	0.142767	−0.079291
C	−0.305907	0.622301	1.273062
C	1.127462	0.366274	1.723687
O	1.375271	−1.074603	1.854922
C	2.385195	−1.482358	0.900033
C	2.233820	0.886158	0.782286
C	2.518751	−0.330606	−0.095987
O	3.359990	1.265049	1.636013
H	−1.464537	0.135630	−0.249744
H	−0.983483	0.105792	1.964896
H	−0.500831	1.703256	1.333762
H	1.248253	0.814787	2.715824
H	3.325839	−1.666248	1.423615
H	1.922842	1.762453	0.207685
H	1.748244	−0.395902	−0.866950
H	3.498749	−0.290801	−0.565836
N	2.030996	−2.763128	0.308365
C	1.003734	−3.082633	−0.578064
N	1.041317	−4.349174	−0.956099
C	2.143530	−4.896258	−0.293901
C	2.702890	−6.211953	−0.313842
O	2.328418	−7.220910	−0.939361
N	3.857079	−6.286099	0.521426
C	4.377626	−5.256089	1.280862
N	5.472273	−5.504293	2.051362
N	3.839590	−4.043437	1.296806
C	2.757770	−3.923238	0.504584
H	0.270841	−2.345204	−0.877100
H	4.300030	−7.201876	0.559952
H	6.039933	−6.319712	1.850057
H	5.984480	−4.665312	2.330430
P	4.931195	1.335356	1.127050
O	5.014397	1.620342	−0.352879
O	5.624067	2.218044	2.139687
O	5.447403	−0.225360	1.299835
C	5.704696	−0.754603	2.628907
C	7.017549	−1.528138	2.678442
O	6.910153	−2.829932	2.013773
C	7.726788	−2.858578	0.817148
C	8.214771	−0.827871	2.008687
C	8.218136	−1.425916	0.596891
O	9.385149	−1.154905	2.815321
H	4.883606	−1.424498	2.907201
H	5.761377	0.069253	3.347844
H	7.241924	−1.727660	3.731523
H	8.553112	−3.553454	0.969152

H	8.100241	0.259093	1.989981
H	7.520727	−0.848318	−0.011297
H	9.196110	−1.389698	0.125387
N	6.938423	−3.441584	−0.287482
C	7.160161	−4.791749	−0.587841
O	7.984994	−5.500028	−0.010489
N	6.353513	−5.279910	−1.602828
C	5.363382	−4.598126	−2.325720
O	4.728860	−5.191429	−3.210704
C	5.170159	−3.215113	−1.924005
C	4.124738	−2.407805	−2.641936
C	5.941317	−2.718886	−0.921472
H	6.518510	−6.251138	−1.860569
H	4.299207	−2.433999	−3.723974
H	4.142327	−1.366302	−2.316210
H	3.121700	−2.815625	−2.471211
H	5.801807	−1.712311	−0.544085
P	10.949279	−1.099293	2.294275
O	11.104813	−0.217480	1.078400
O	11.766384	−0.860700	3.544577
O	11.194155	−2.633070	1.750045
C	11.159987	−3.741438	2.682923
C	11.893594	−4.938802	2.094556
O	11.190543	−5.476738	0.934021
C	11.918202	−5.191572	−0.282562
C	13.326113	−4.654017	1.614540
C	13.153008	−4.362083	0.116953
O	14.101205	−5.859607	1.854009
H	10.116876	−4.013884	2.884922
H	11.641300	−3.452964	3.625014
H	11.904991	−5.725694	2.858109
H	12.186734	−6.144875	−0.745434
H	13.774798	−3.808960	2.147514
H	12.973010	−3.295418	−0.021550
H	14.033083	−4.645231	−0.465168
H	14.996571	−5.709920	1.499151
N	11.036502	−4.528392	−1.246378
C	10.677063	−3.187336	−1.314465
N	9.759836	−2.943046	−2.237048
C	9.487982	−4.186061	−2.806078
C	8.577654	−4.601451	−3.803797
N	7.745837	−3.746925	−4.438276
N	8.525787	−5.924367	−4.113285
C	9.340147	−6.776327	−3.458245
N	10.232574	−6.498220	−2.490666
C	10.265471	−5.184815	−2.198397
H	11.105281	−2.452018	−0.649246
H	7.093811	−4.094375	−5.129764
H	7.764576	−2.758110	−4.229464
H	9.257860	−7.822479	−3.749027

(GTC)$^{2-}$

E: −13704.10 kcal mol^{-1}

O	−0.507569	0.182278	−0.043925
C	−0.317149	0.635336	1.318840
C	1.113656	0.372273	1.773704
O	1.364482	−1.070752	1.871371
C	2.381173	−1.452963	0.913428
C	2.224320	0.918649	0.852358
C	2.520985	−0.275650	−0.051615
O	3.342826	1.286377	1.721334
H	−1.464211	0.178210	−0.221623
H	−0.998636	0.104477	1.995599
H	−0.513984	1.714629	1.399473
H	1.226530	0.796570	2.777280
H	3.317721	−1.649870	1.439487
H	1.912367	1.805940	0.295416
H	1.758849	−0.323487	−0.832085
H	3.505063	−0.220473	−0.510345
N	2.034299	−2.717966	0.284202
C	1.027585	−3.011233	−0.634828
N	1.072972	−4.266429	−1.047373
C	2.158739	−4.833695	−0.375071
C	2.720153	−6.147727	−0.423072
O	2.363531	−7.135633	−1.091249
N	3.852508	−6.248687	0.438731
C	4.352671	−5.243114	1.243135
N	5.424308	−5.517090	2.036328
N	3.815780	−4.030190	1.280644
C	2.754465	−3.884818	0.465308
H	0.302003	−2.265092	−0.929712
H	4.294775	−7.165437	0.460072
H	5.995495	−6.328567	1.830102
H	5.926403	−4.689433	2.361541
P	4.921591	1.352835	1.237281
O	5.032451	1.646100	−0.239239
O	5.603355	2.223669	2.267383
O	5.426623	−0.212381	1.407490
C	5.643271	−0.758224	2.737180
C	6.947694	−1.543648	2.808740
O	6.847162	−2.832178	2.117201
C	7.687792	−2.847554	0.940437
C	8.164100	−0.838153	2.182838
C	8.211169	−1.417398	0.763890
O	9.315049	−1.185123	3.005881
H	4.809075	−1.423211	2.986696
H	5.689159	0.056981	3.466716
H	7.143024	−1.764918	3.863155
H	8.496800	−3.563363	1.091443
H	8.052348	0.249047	2.174541
H	7.553721	−0.820921	0.130018
H	9.213534	−1.387214	0.344731
N	6.914509	−3.384771	−0.200338
C	7.155695	−4.713491	−0.575270
O	7.970678	−5.450338	−0.019934
N	6.383448	−5.144330	−1.641153
C	5.423896	−4.420680	−2.362288
O	4.829644	−4.958157	−3.309502
C	5.211904	−3.062598	−1.889468
C	4.200762	−2.212705	−2.607743
C	5.941391	−2.625666	−0.829791
H	6.566151	−6.097484	−1.950331
H	4.406658	−2.205999	−3.684343
H	4.226988	−1.183421	−2.246828
H	3.185887	−2.607433	−2.480058
H	5.788171	−1.641483	−0.401330
P	10.882131	−0.957164	2.533999
O	10.976542	0.039532	1.404509
O	11.655264	−0.762913	3.819151
O	11.262425	−2.419496	1.871440
C	11.282387	−3.579738	2.746057
C	11.916393	−4.765493	2.036319
O	11.061349	−5.214336	0.943176
C	11.774435	−5.155082	−0.315841
C	13.308140	−4.506874	1.425861
C	13.016387	−4.285285	−0.063473
O	14.101669	−5.702430	1.659553
H	10.255251	−3.834494	3.034946
H	11.857316	−3.350191	3.650850
H	11.989811	−5.581923	2.766199
H	12.037672	−6.164582	−0.633047
H	13.797280	−3.640297	1.882662
H	12.801982	−3.227885	−0.235081
H	13.853684	−4.578082	−0.701894
H	14.972620	−5.563837	1.244277

N	10.860769	−4.644988	−1.349622
C	10.339547	−5.537113	−2.338744
O	10.720414	−6.725644	−2.358402
N	9.440137	−5.043988	−3.236830
C	9.040589	−3.766399	−3.176696
N	8.125141	−3.348996	−4.077004
C	9.523336	−2.862700	−2.176392
C	10.418332	−3.353695	−1.280488
H	7.803034	−3.981922	−4.799055
H	7.827100	−2.383641	−4.109248
H	9.176354	−1.838266	−2.122078
H	10.803806	−2.765117	−0.455959

(GTG)$^{2-}$

E: −14266.35 kcal mol^{-1}

O	−0.499618	0.161323	−0.074814
C	−0.291538	0.633222	1.278979
C	1.140237	0.361190	1.724911
O	1.374603	−1.082879	1.846169
C	2.388831	−1.490125	0.895627
C	2.248731	0.877776	0.783779
C	2.525491	−0.337757	−0.098717
O	3.378163	1.247974	1.637287
H	−1.457574	0.169918	−0.245252
H	−0.973078	0.121055	1.970239
H	−0.474937	1.715878	1.344468
H	1.267590	0.802708	2.719373
H	3.327745	−1.673162	1.422306
H	1.941973	1.757725	0.212618
H	1.751889	−0.397729	−0.867148
H	3.504337	−0.302070	−0.571352
N	2.038110	−2.770713	0.302250
C	1.012973	−3.090883	−0.586250
N	1.054539	−4.356406	−0.967268
C	2.157453	−4.901939	−0.305097
C	2.720104	−6.216130	−0.327522
O	2.348981	−7.224571	−0.955880
N	3.873449	−6.289333	0.508928
C	4.390509	−5.259854	1.271674
N	5.484160	−5.508148	2.043700
N	3.849952	−4.048355	1.289221
C	2.768418	−3.929059	0.496193
H	0.278371	−2.354895	−0.884750
H	4.317603	−7.204584	0.547103
H	6.053217	−6.322123	1.840411
H	5.997078	−4.669170	2.322302
P	4.950292	1.303802	1.129750
O	5.037747	1.590867	−0.349483
O	5.650908	2.177414	2.144836
O	5.450915	−0.262123	1.300180
C	5.704180	−0.795984	2.628285
C	7.028449	−1.549836	2.685717
O	6.945046	−2.853399	2.021129
C	7.760133	−2.867702	0.822923
C	8.217971	−0.831249	2.022231
C	8.239118	−1.429322	0.610324
O	9.389096	−1.137090	2.836065
H	4.891030	−1.480749	2.893003
H	5.740937	0.023399	3.353517
H	7.249789	−1.746420	3.739959
H	8.592014	−3.556819	0.970619
H	8.085374	0.253586	2.001932
H	7.542826	−0.857325	−0.004157
H	9.220924	−1.383727	0.147915
N	6.975470	−3.451525	−0.284876
C	7.192429	−4.803705	−0.576461
O	8.006938	−5.515610	0.011376
N	6.390492	−5.293102	−1.595188
C	5.400001	−4.613660	−2.321796
O	4.762832	−5.213593	−3.200043
C	5.208442	−3.229487	−1.924899
C	4.160973	−2.423735	−2.641676
C	5.979192	−2.730913	−0.923286
H	6.531016	−6.274891	−1.824567
H	4.338691	−2.444037	−3.723214
H	4.173117	−1.383562	−2.310954
H	3.159528	−2.837005	−2.474453
H	5.837713	−1.723744	−0.548552
P	10.954837	−1.056559	2.323414
O	11.103385	−0.174948	1.106595
O	11.761323	−0.801121	3.577594
O	11.227616	−2.587976	1.785830
C	11.184521	−3.692759	2.722434
C	11.897717	−4.901495	2.132772
O	11.179307	−5.429470	0.977260
C	11.910998	−5.168597	−0.242873
C	13.332088	−4.638950	1.645193
C	13.155734	−4.350000	0.147713
O	14.091321	−5.854664	1.885157
H	10.139242	−3.950832	2.932228
H	11.675231	−3.407976	3.660945
H	11.901891	−5.686896	2.898019
H	12.169059	−6.130467	−0.693575
H	13.795356	−3.798674	2.173267
H	12.984673	−3.282106	0.007153
H	14.030396	−4.643172	−0.437645
H	14.987547	−5.717906	1.527291
N	11.039127	−4.505273	−1.214356
C	10.699866	−3.154262	−1.298352
N	9.800059	−2.913476	−2.233081
C	9.516077	−4.158524	−2.793556
C	8.599506	−4.524458	−3.824519
O	7.831483	−3.805676	−4.489344
N	8.617451	−5.931642	−4.040497
C	9.414194	−6.842420	−3.374957
N	9.260070	−8.154971	−3.696877
N	10.265076	−6.487163	−2.417430
C	10.272380	−5.159296	−2.165541
H	11.130638	−2.419915	−0.634129
H	7.984507	−6.265057	−4.764753
H	8.834121	−8.409381	−4.580241
H	9.958665	−8.794203	−3.335295

(GTT)$^{2-}$

E: −13962.82 kcal mol^{-1}

O	−0.496238	0.396778	0.066197
C	−0.250535	0.789689	1.438230
C	1.171385	0.427550	1.848010
O	1.334522	−1.031520	1.899863
C	2.346971	−1.435793	0.945589
C	2.287109	0.935486	0.908857
C	2.510048	−0.259506	−0.015263
O	3.439597	1.245985	1.756498
H	−1.448405	0.503151	−0.103761
H	−0.948736	0.277955	2.113268
H	−0.378666	1.875514	1.559630
H	1.336663	0.817187	2.858429
H	3.279714	−1.649089	1.470845
H	2.003030	1.842862	0.369724
H	1.722356	−0.269211	−0.772006
H	3.481960	−0.240623	−0.502094
N	1.977440	−2.692988	0.316602

C	0.957981	−2.966257	−0.594066
N	0.985219	−4.217734	−1.019476
C	2.072982	−4.802326	−0.366280
C	2.616849	−6.123056	−0.433771
O	2.237851	−7.100144	−1.105184
N	3.759154	−6.245827	0.411054
C	4.283349	−5.253654	1.217494
N	5.362852	−5.549975	1.990363
N	3.762563	−4.033910	1.273756
C	2.690277	−3.868401	0.475478
H	0.237627	−2.210232	−0.876221
H	4.188635	−7.168842	0.419939
H	5.909258	−6.377628	1.782392
H	5.885979	−4.737142	2.321176
P	5.013403	1.208092	1.256975
O	5.133965	1.489952	−0.220528
O	5.761071	2.031488	2.280213
O	5.415175	−0.387424	1.424356
C	5.616677	−0.948178	2.750182
C	6.953537	−1.674736	2.847455
O	6.922798	−2.971201	2.162883
C	7.750431	−2.950941	0.978389
C	8.142785	−0.913451	2.235680
C	8.263553	−1.513146	0.827005
O	9.291725	−1.164785	3.092839
H	4.806749	−1.656304	2.954840
H	5.601562	−0.148433	3.497393
H	7.143430	−1.885149	3.904844
H	8.564139	−3.666163	1.104409
H	7.962771	0.163519	2.198349
H	7.640829	−0.927235	0.150849
H	9.287083	−1.483650	0.461810
N	6.968247	−3.466055	−0.172270
C	7.182240	−4.797399	−0.552757
O	8.000722	−5.547028	−0.018224
N	6.382599	−5.217741	−1.602758
C	5.412467	−4.482873	−2.302386
O	4.780591	−5.018826	−3.224318
C	5.237312	−3.119904	−1.830788
C	4.227022	−2.251641	−2.527741
C	5.991528	−2.695114	−0.783602
H	6.524124	−6.182860	−1.895896
H	4.395570	−2.269913	−3.610565
H	4.298698	−1.217736	−2.185424
H	3.205429	−2.611361	−2.357870
H	5.852637	−1.711747	−0.348556
P	10.837888	−0.785505	2.646939
O	10.853079	0.251017	1.550216
O	11.579166	−0.565455	3.947204
O	11.345243	−2.190158	1.945797
C	11.408652	−3.372364	2.787479
C	11.978657	−4.537980	2.002083
O	11.036375	−4.924926	0.955900
C	11.735209	−5.033116	−0.302958
C	13.334604	−4.271683	1.307454
C	12.962514	−4.121688	−0.174015
O	14.172988	−5.432800	1.550638
H	10.401122	−3.625157	3.139455
H	12.047501	−3.176782	3.657011
H	12.095968	−5.381897	2.694512
H	12.022581	−6.070394	−0.485479
H	13.820422	−3.373588	1.700615
H	12.705174	−3.078990	−0.375693
H	13.771765	−4.421262	−0.844303
H	15.034529	−5.268064	1.125436
N	10.800043	−4.684674	−1.381580
C	10.309449	−5.710834	−2.194883
O	10.644369	−6.894806	−2.092775
N	9.403275	−5.283402	−3.150255
C	8.927043	−3.982812	−3.369744
O	8.105936	−3.769315	−4.275019
C	9.456514	−2.982458	−2.455964
C	9.008077	−1.556537	−2.615379
C	10.346865	−3.385174	−1.513239
H	9.052829	−6.008524	−3.773786
H	9.258336	−1.187589	−3.617023
H	9.487542	−0.908347	−1.878740
H	7.920559	−1.472177	−2.507668
H	10.756514	−2.692328	−0.787936

(TAA)$^{2-}$

E: −13937.22 kcal mol^{-1}

H: −13467.52 kcal mol^{-1}

G: −13553.57 kcal mol^{-1}

O	−0.225163	−0.384552	−0.344546
C	−0.142728	0.014377	1.046618
C	1.282847	−0.137259	1.559125
O	1.645766	−1.552959	1.689566
C	2.748338	−1.876054	0.809459
C	2.379719	0.498540	0.680086
C	2.901727	−0.683441	−0.140895
O	3.364261	1.065168	1.594418
H	−1.167173	−0.419617	−0.586216
H	−0.806463	−0.605519	1.662445
H	−0.439357	1.066893	1.161083
H	1.320195	0.303034	2.561446
H	3.650132	−2.052495	1.397985
H	1.992878	1.300315	0.046023
H	2.267310	−0.801573	−1.021858
H	3.927140	−0.547363	−0.472860
N	2.466379	−3.155482	0.133473
C	3.259165	−4.265426	0.441277
O	4.173892	−4.254764	1.267184
N	2.936400	−5.398303	−0.290035
C	1.903229	−5.560774	−1.223077
O	1.748384	−6.657629	−1.787065
C	1.084648	−4.377568	−1.431736
C	−0.079049	−4.452408	−2.382889
C	1.400350	−3.249562	−0.744315
H	3.517466	−6.214318	−0.106937
H	−0.789079	−5.228774	−2.072174
H	0.258031	−4.711258	−3.394374
H	−0.604923	−3.494389	−2.425952
H	0.806585	−2.344249	−0.828060
P	4.863461	1.563552	1.110329
O	4.909182	1.796299	−0.380521
O	5.250462	2.651851	2.086995
O	5.782099	0.222604	1.374266
C	6.003363	−0.225112	2.735929
C	7.247776	−1.098844	2.815687
O	7.048173	−2.384603	2.150864
C	7.812467	−2.445357	0.924424
C	8.512777	−0.497460	2.174850
C	8.487974	−1.077355	0.757388
O	9.640142	−0.930532	2.992977
H	5.132990	−0.799941	3.075037
H	6.135594	0.641895	3.393688
H	7.430681	−1.311497	3.874595
H	8.534387	−3.262673	1.013518
H	8.495276	0.595814	2.165908
H	7.881818	−0.415126	0.139396
H	9.477949	−1.146336	0.314663
N	6.943290	−2.816910	−0.192748
C	6.158310	−2.001346	−0.996943

N	5.470249	−2.677028	−1.904980
C	5.810331	−4.011501	−1.687143
C	5.402785	−5.215186	−2.301809
N	4.554509	−5.248049	−3.357157
N	5.899699	−6.382192	−1.816504
C	6.774907	−6.339205	−0.790189
N	7.237966	−5.259675	−0.137312
C	6.717844	−4.118744	−0.621113
H	6.122619	−0.931273	−0.856172
H	4.177398	−6.137354	−3.661609
H	4.078980	−4.402871	−3.645133
H	7.149073	−7.303693	−0.451124
P	11.205855	−0.987648	2.474668
O	11.440053	−0.066296	1.301580
O	12.036424	−0.875020	3.734134
O	11.317928	−2.514986	1.864782
C	11.185749	−3.635094	2.778782
C	11.755780	−4.897986	2.149993
O	10.916096	−5.357769	1.045293
C	11.626308	−5.258138	−0.210820
C	13.174939	−4.752797	1.574122
C	12.931612	−4.496522	0.081251
O	13.864842	−6.008829	1.812041
H	10.125697	−3.787919	3.015403
H	11.729444	−3.420944	3.706463
H	11.745339	−5.682065	2.916674
H	11.809270	−6.266816	−0.592227
H	13.721985	−3.930372	2.046755
H	12.800991	−3.425164	−0.081390
H	13.755545	−4.848057	−0.544247
H	14.752228	−5.936644	1.415157
N	10.766485	−4.612988	−1.200295
C	10.433046	−3.267100	−1.285182
N	9.547787	−3.010033	−2.234017
C	9.272452	−4.251141	−2.807512
C	8.401628	−4.656185	−3.842880
N	7.617699	−3.786651	−4.532157
N	8.365842	−5.972434	−4.173189
C	9.140055	−6.835911	−3.488778
N	9.984047	−6.572125	−2.474143
C	10.016497	−5.261430	−2.173904
H	10.866793	−2.542037	−0.611467
H	6.870462	−4.176550	−5.096430
H	7.461553	−2.863580	−4.144476
H	9.067016	−7.878942	−3.792805

(TAC)$^{2-}$

E: −13538.14 kcal mol^{-1}

O	−0.230380	−0.317580	−0.327015
C	−0.152919	0.073706	1.066530
C	1.269740	−0.085143	1.585200
O	1.630907	−1.502793	1.700086
C	2.737230	−1.814296	0.819681
C	2.371927	0.558729	0.719099
C	2.884319	−0.613167	−0.120715
O	3.359854	1.099748	1.646139
H	−1.171369	−0.346825	−0.573494
H	−0.821131	−0.547646	1.675924
H	−0.446910	1.126439	1.185537
H	1.302237	0.343207	2.592790
H	3.638675	−1.988691	1.409316
H	1.992737	1.375522	0.099833
H	2.239441	−0.720788	−0.995281
H	3.904949	−0.472037	−0.463822
N	2.465238	−3.090707	0.135148
C	3.261356	−4.198452	0.442624
O	4.162833	−4.190676	1.283112
N	2.959073	−5.325050	−0.306891
C	1.932377	−5.488195	−1.247671
O	1.788480	−6.582015	−1.819788
C	1.105817	−4.309647	−1.451774
C	−0.052926	−4.387299	−2.408736
C	1.407192	−3.185249	−0.752181
H	3.536649	−6.141815	−0.116773
H	−0.761062	−5.167244	−2.102663
H	0.289900	−4.642576	−3.419022
H	−0.583028	−3.431689	−2.451880
H	0.806550	−2.284212	−0.832161
P	4.869682	1.587948	1.188585
O	4.947682	1.813104	−0.302130
O	5.246792	2.676860	2.168018
O	5.776832	0.241953	1.475689
C	5.959624	−0.209363	2.842189
C	7.188222	−1.102482	2.952103
O	6.981411	−2.393762	2.298824
C	7.764005	−2.485222	1.088239
C	8.472981	−0.525442	2.329941
C	8.484667	−1.136311	0.924731
O	9.578317	−0.953974	3.178013
H	5.072391	−0.769375	3.161900
H	6.091288	0.656082	3.502278
H	7.348219	−1.306753	4.016266
H	8.459106	−3.324199	1.188767
H	8.464553	0.567109	2.295443
H	7.927850	−0.473358	0.262262
H	9.492426	−1.246022	0.531579
N	6.904950	−2.830101	−0.047312
C	6.109146	−1.987336	−0.812673
N	5.452569	−2.623682	−1.770650
C	5.829777	−3.958068	−1.632812
C	5.473810	−5.128518	−2.338244
N	4.636816	−5.115629	−3.402019
N	6.007776	−6.309085	−1.931007
C	6.862041	−6.309161	−0.886152
N	7.272738	−5.264443	−0.147131
C	6.723926	−4.109042	−0.560631
H	6.043134	−0.930568	−0.600443
H	4.306112	−5.992521	−3.785748
H	4.146450	−4.266140	−3.649255
H	7.265894	−7.281553	−0.609631
P	11.159813	−0.864699	2.708438
O	11.349867	0.158460	1.614880
O	11.948138	−0.789167	3.997143
O	11.394903	−2.333961	1.993780
C	11.291418	−3.514181	2.835246
C	11.718927	−4.749169	2.062338
O	10.746948	−5.034860	1.011317
C	11.425776	−5.192263	−0.257778
C	13.102712	−4.654443	1.380662
C	12.766030	−4.457498	−0.102805
O	13.789189	−5.910430	1.631305
H	10.254196	−3.632168	3.171870
H	11.937156	−3.399381	3.713825
H	11.726966	−5.592578	2.765384
H	11.561608	−6.251083	−0.483119
H	13.692992	−3.824004	1.780548
H	12.650161	−3.389867	−0.308662
H	13.536255	−4.860439	−0.765336
H	14.668820	−5.852424	1.215171
N	10.550664	−4.669565	−1.313875
C	10.002350	−5.554924	−2.293690
O	10.319831	−6.761636	−2.278951
N	9.149215	−5.032672	−3.218602
C	8.828657	−3.732516	−3.202301
N	7.981364	−3.281826	−4.154104

C	9.358553	−2.827173	−2.226381
C	10.205409	−3.347132	−1.301451
H	7.526711	−3.949154	−4.766113
H	7.600715	−2.346275	−4.102741
H	9.083405	−1.779657	−2.212151
H	10.633196	−2.758184	−0.498689

$(TAG)^{2-}$

E: −14101.40 kcal mol^{-1}

O	−0.198418	−0.480095	−0.351424
C	−0.119696	−0.070667	1.036766
C	1.307156	−0.204270	1.550732
O	1.685402	−1.615221	1.687445
C	2.793326	−1.928729	0.810340
C	2.398446	0.439620	0.670491
C	2.931820	−0.739798	−0.146544
O	3.376923	1.017401	1.584573
H	−1.140046	−0.525854	−0.592781
H	−0.776920	−0.693139	1.656976
H	−0.427174	0.979482	1.144238
H	1.338524	0.240876	2.551147
H	3.696800	−2.089368	1.400622
H	2.004815	1.236386	0.034317
H	2.295888	−0.869289	−1.024926
H	3.955040	−0.595479	−0.481937
N	2.529569	−3.215736	0.142145
C	3.342279	−4.310510	0.452195
O	4.260259	−4.279784	1.273904
N	3.034451	−5.452761	−0.270816
C	2.001171	−5.636611	−1.199682
O	1.862575	−6.738743	−1.757797
C	1.162479	−4.468364	−1.411358
C	−0.002875	−4.567248	−2.358036
C	1.462388	−3.331452	−0.731596
H	3.632375	−6.256849	−0.089402
H	−0.702195	−5.349090	−2.037104
H	0.334258	−4.831671	−3.367940
H	−0.541191	−3.616477	−2.408405
H	0.854346	−2.435980	−0.818649
P	4.875023	1.521165	1.103146
O	4.921310	1.759866	−0.386748
O	5.257304	2.607127	2.084116
O	5.797854	0.182475	1.362611
C	6.020906	−0.270114	2.722388
C	7.289983	−1.107604	2.806019
O	7.133920	−2.400857	2.144942
C	7.889609	−2.437463	0.911966
C	8.537335	−0.467236	2.168394
C	8.544604	−1.057495	0.754366
O	9.673857	−0.843272	3.002895
H	5.164431	−0.872035	3.049796
H	6.123484	0.594980	3.388064
H	7.476032	−1.312891	3.865837
H	8.622362	−3.246279	0.988331
H	8.478230	0.624409	2.148565
H	7.939999	−0.407301	0.122337
H	9.542422	−1.112992	0.328219
N	7.018404	−2.808452	−0.205872
C	6.225508	−1.993881	−1.005217
N	5.534941	−2.669723	−1.910045
C	5.881329	−4.002842	−1.698421
C	5.469564	−5.207118	−2.309397
N	4.611057	−5.243911	−3.354995
N	5.965369	−6.375096	−1.822772
C	6.837182	−6.331671	−0.793209
N	7.302215	−5.252319	−0.142649
C	6.790802	−4.110315	−0.633521
H	6.185012	−0.924521	−0.861382
H	4.233298	−6.133005	−3.658232
H	4.159596	−4.394944	−3.668470
H	7.207417	−7.296599	−0.451171
P	11.240519	−0.915293	2.492168
O	11.487438	−0.018324	1.302603
O	12.063450	−0.778550	3.754592
O	11.350804	−2.453434	1.913579
C	11.165131	−3.554324	2.840564
C	11.651518	−4.850167	2.210244
O	10.791766	−5.232716	1.091970
C	11.533811	−5.205630	−0.150619
C	13.085223	−4.802984	1.652648
C	12.878942	−4.526347	0.158547
O	13.681489	−6.105856	1.894442
H	10.101173	−3.643642	3.091754
H	11.731802	−3.365619	3.760274
H	11.577278	−5.637499	2.970288
H	11.659395	−6.231819	−0.507875
H	13.683331	−4.023388	2.135999
H	12.813805	−3.448255	0.001143
H	13.687969	−4.924016	−0.458994
H	14.576235	−6.096174	1.507716
N	10.743558	−4.524476	−1.171064
C	10.526949	−3.153405	−1.307827
N	9.698267	−2.865997	−2.293207
C	9.338438	−4.099684	−2.835827
C	8.463113	−4.420148	−3.917547
O	7.808742	−3.661526	−4.655290
N	8.384576	−5.828250	−4.104107
C	9.060917	−6.781319	−3.371898
N	8.826885	−8.084536	−3.678231
N	9.879204	−6.470799	−2.370975
C	9.974781	−5.141746	−2.144774
H	10.996713	−2.442508	−0.643910
H	7.757625	−6.129525	−4.846961
H	8.426702	−8.327560	−4.576398
H	9.450912	−8.767350	−3.264230

$(TAT)^{2-}$

E: −13797.55 kcal mol^{-1}

O	−0.182388	−0.320461	−0.332848
C	−0.115084	0.074899	1.060045
C	1.302378	−0.087828	1.592356
O	1.657615	−1.506274	1.714543
C	2.767925	−1.825000	0.841071
C	2.415417	0.549134	0.735300
C	2.926993	−0.626175	−0.100126
O	3.399450	1.083781	1.670856
H	−1.121784	−0.356710	−0.584215
H	−0.791046	−0.541793	1.665573
H	−0.405854	1.129226	1.173054
H	1.326706	0.343090	2.599079
H	3.664446	−2.002072	1.437227
H	2.046756	1.368501	0.113062
H	2.286728	−0.730961	−0.978337
H	3.950206	−0.491358	−0.437647
N	2.494761	−3.102515	0.159481
C	3.274561	−4.215847	0.488391
O	4.167093	−4.208245	1.338512
N	2.967530	−5.347695	−0.250773
C	1.953585	−5.508897	−1.205371
O	1.804513	−6.607403	−1.766957
C	1.143996	−4.322965	−1.433871
C	−0.001137	−4.397516	−2.407261

C	1.447788	−3.194887	−0.741370
H	3.535523	−6.167856	−0.046997
H	−0.714354	−5.177095	−2.112481
H	0.355511	−4.651375	−3.413293
H	−0.529462	−3.441050	−2.456226
H	0.857499	−2.288725	−0.838006
P	4.918350	1.557819	1.228650
O	5.012842	1.790091	−0.259956
O	5.299113	2.636219	2.218179
O	5.808662	0.199935	1.515783
C	5.963297	−0.268248	2.879965
C	7.189167	−1.162999	3.003682
O	6.992882	−2.454345	2.347956
C	7.791761	−2.549899	1.149412
C	8.481494	−0.581173	2.402349
C	8.543426	−1.214881	1.006964
O	9.571373	−0.976591	3.284457
H	5.068885	−0.829904	3.175768
H	6.083551	0.589098	3.552639
H	7.334885	−1.368815	4.069634
H	8.468607	−3.403004	1.250077
H	8.457987	0.510136	2.346867
H	8.038198	−0.551560	0.305193
H	9.566964	−1.359108	0.668553
N	6.941791	−2.863418	−0.004539
C	6.153835	−1.995285	−0.750894
N	5.476537	−2.605221	−1.710229
C	5.825377	−3.948858	−1.591757
C	5.424417	−5.104174	−2.299597
N	4.571820	−5.061726	−3.345826
N	5.918204	−6.305221	−1.896195
C	6.768389	−6.339147	−0.848866
N	7.224789	−5.310590	−0.113127
C	6.720411	−4.133394	−0.525155
H	6.109165	−0.940936	−0.521678
H	4.244146	−5.922099	−3.766289
H	4.160748	−4.185202	−3.636843
H	7.131443	−7.326758	−0.570563
P	11.158461	−0.807785	2.856244
O	11.329163	0.261572	1.804875
O	11.912499	−0.748714	4.166412
O	11.469673	−2.236662	2.094616
C	11.364225	−3.452639	2.880550
C	11.699276	−4.651773	2.016174
O	10.661877	−4.818392	1.002332
C	11.290973	−5.124374	−0.260536
C	13.056163	−4.576121	1.269601
C	12.660192	−4.432703	−0.208095
O	13.761755	−5.816026	1.540293
H	10.343795	−3.551796	3.269860
H	12.061352	−3.411490	3.726007
H	11.707588	−5.540446	2.661516
H	11.389335	−6.204780	−0.386033
H	13.655960	−3.727963	1.612018
H	12.572265	−3.372376	−0.459292
H	13.385022	−4.894420	−0.882887
H	14.644149	−5.746829	1.131724
N	10.398333	−4.674956	−1.335341
C	9.775932	−5.635212	−2.138224
O	9.940918	−6.852319	−2.016083
N	8.944002	−5.098981	−3.105727
C	8.681333	−3.746685	−3.369725
O	7.935900	−3.435406	−4.310886
C	9.337205	−2.815564	−2.463495
C	9.106970	−1.341321	−2.646464
C	10.147133	−3.325053	−1.501825
H	8.490028	−5.778578	−3.713210
H	9.453101	−1.017070	−3.635452
H	9.641261	−0.765130	−1.887244
H	8.039496	−1.102334	−2.586780
H	10.650522	−2.685307	−0.787257

(TCA)$^{2-}$

E: −13535.69 kcal mol^{-1}

O	−0.305962	−0.297497	−0.190603
C	−0.202365	0.065000	1.209135
C	1.234691	−0.085007	1.689718
O	1.613050	−1.500015	1.774539
C	2.696445	−1.790525	0.860947
C	2.304931	0.584735	0.803423
C	2.820163	−0.569974	−0.059464
O	3.308635	1.136488	1.705039
H	−1.251710	−0.335921	−0.416690
H	−0.847394	−0.579059	1.820022
H	−0.508181	1.110631	1.357336
H	1.288957	0.328582	2.702482
H	3.612732	−1.976901	1.423620
H	1.895692	1.397692	0.198633
H	2.171247	−0.669509	−0.931920
H	3.838381	−0.408230	−0.402949
N	2.409867	−3.052601	0.155083
C	3.215840	−4.165431	0.419169
O	4.134828	−4.174137	1.240305
N	2.902386	−5.273633	−0.350943
C	1.877576	−5.408054	−1.295684
O	1.744462	−6.478211	−1.914297
C	1.038290	−4.230932	−1.451278
C	−0.124071	−4.284107	−2.405360
C	1.339823	−3.127165	−0.720241
H	3.509120	−6.079935	−0.214481
H	−0.824021	−5.081055	−2.125772
H	0.217570	−4.499501	−3.425240
H	−0.662388	−3.332228	−2.412525
H	0.733525	−2.227625	−0.766001
P	4.782666	1.671053	1.180459
O	4.759796	1.970893	−0.298679
O	5.201270	2.718839	2.187559
O	5.722857	0.328435	1.347501
C	5.983440	−0.168399	2.687181
C	7.184466	−1.101595	2.687270
O	6.872607	−2.359104	2.016710
C	7.688268	−2.519539	0.826932
C	8.445919	−0.548990	1.993492
C	8.375006	−1.168615	0.594751
O	9.580099	−0.986354	2.799729
H	5.101390	−0.708553	3.052355
H	6.186397	0.674369	3.358084
H	7.415805	−1.333272	3.733438
H	8.408401	−3.321402	0.995926
H	8.454059	0.543572	1.954224
H	7.769810	−0.509480	−0.029596
H	9.351462	−1.270169	0.130364
N	6.839685	−2.996477	−0.277284
C	6.802155	−4.405143	−0.555937
O	7.500364	−5.177766	0.122564
N	6.000992	−4.834722	−1.573036
C	5.232988	−3.975515	−2.250264
N	4.472923	−4.465416	−3.255025
C	5.181344	−2.580786	−1.929384
C	5.994542	−2.146315	−0.929657
H	4.493152	−5.455546	−3.464800
H	3.865402	−3.864870	−3.794916
H	4.518571	−1.899808	−2.449718
H	5.994980	−1.117540	−0.591336
P	11.147127	−1.014270	2.288286

O	11.352748	−0.164878	1.056836
O	11.972467	−0.791335	3.536974
O	11.317370	−2.570215	1.778871
C	11.190336	−3.647774	2.740286
C	11.805351	−4.921944	2.178688
O	11.017229	−5.450186	1.068038
C	11.721056	−5.282325	−0.184750
C	13.235420	−4.766038	1.636879
C	13.027377	−4.530059	0.133857
O	13.933662	−6.010728	1.907818
H	10.128886	−3.816901	2.958505
H	11.707289	−3.380000	3.669835
H	11.786395	−5.676025	2.974338
H	11.902679	−6.274160	−0.607286
H	13.761385	−3.931380	2.112422
H	12.918494	−3.460391	−0.049447
H	13.862310	−4.902520	−0.464357
H	14.826114	−5.937260	1.522929
N	10.859287	−4.599752	−1.150687
C	10.579397	−3.241716	−1.246049
N	9.681385	−2.961156	−2.176455
C	9.339665	−4.196530	−2.723902
C	8.426347	−4.574370	−3.731247
N	7.680416	−3.673245	−4.420362
N	8.315200	−5.892467	−4.036069
C	9.067506	−6.780166	−3.356514
N	9.954089	−6.540716	−2.372283
C	10.054495	−5.228053	−2.094059
H	11.052309	−2.523588	−0.592426
H	6.900394	−4.017092	−4.968745
H	7.618478	−2.724251	−4.072435
H	8.937643	−7.823550	−3.640159

(TCC)$^{2-}$

E: −13137.46 kcal mol^{-1}

O	−0.318599	−0.229109	−0.190003
C	−0.222374	0.126043	1.212193
C	1.210348	−0.034579	1.701876
O	1.581792	−1.452278	1.776963
C	2.672319	−1.738604	0.870994
C	2.290086	0.637716	0.829235
C	2.804402	−0.511749	−0.039903
O	3.290421	1.175131	1.743691
H	−1.263005	−0.259878	−0.422898
H	−0.875273	−0.517221	1.815382
H	−0.523222	1.172680	1.363363
H	1.259336	0.369994	2.718487
H	3.583657	−1.930306	1.439963
H	1.889609	1.458758	0.229486
H	2.158286	−0.603776	−0.915362
H	3.823516	−0.347818	−0.378897
N	2.389889	−2.994870	0.152650
C	3.203795	−4.105887	0.399139
O	4.120328	−4.122598	1.222780
N	2.901982	−5.202058	−0.392813
C	1.873724	−5.331145	−1.334617
O	1.748197	−6.392074	−1.970547
C	1.021472	−4.160258	−1.465896
C	−0.147022	−4.211038	−2.412566
C	1.317194	−3.064763	−0.720151
H	3.512744	−6.007089	−0.267054
H	−0.835762	−5.020375	−2.140293
H	0.189140	−4.406155	−3.438278
H	−0.696305	−3.265424	−2.400238
H	0.704443	−2.168784	−0.750557
P	4.777495	1.699699	1.247975
O	4.796143	1.972306	−0.236384
O	5.173458	2.763359	2.247253
O	5.711808	0.358546	1.463467
C	5.923402	−0.130021	2.814686
C	7.108323	−1.083603	2.858308
O	6.799642	−2.334848	2.173240
C	7.645374	−2.503768	1.008155
C	8.403264	−0.552252	2.213223
C	8.374999	−1.166937	0.810639
O	9.504775	−1.022428	3.044981
H	5.022099	−0.653106	3.156844
H	6.119620	0.715101	3.484845
H	7.295422	−1.321766	3.911727
H	8.338122	−3.327305	1.186796
H	8.434620	0.539800	2.178999
H	7.817458	−0.495068	0.156251
H	9.374049	−1.290494	0.400427
N	6.822402	−2.941147	−0.132411
C	6.811857	−4.332977	−0.486664
O	7.498894	−5.134027	0.170709
N	6.052345	−4.715462	−1.552806
C	5.296175	−3.829628	−2.210385
N	4.564558	−4.274851	−3.254577
C	5.225908	−2.452394	−1.824319
C	5.998990	−2.062765	−0.776172
H	4.608452	−5.250277	−3.522485
H	3.986438	−3.647162	−3.796077
H	4.579508	−1.750354	−2.336854
H	5.983846	−1.050921	−0.389430
P	11.087811	−0.930972	2.581505
O	11.274254	0.065766	1.463244
O	11.872338	−0.818198	3.870025
O	11.339089	−2.416076	1.906170
C	11.264293	−3.577949	2.776558
C	11.793252	−4.811699	2.063433
O	10.882010	−5.209075	0.994569
C	11.574602	−5.239861	−0.275814
C	13.185785	−4.655941	1.419291
C	12.876767	−4.448365	−0.068728
O	13.909249	−5.892275	1.665105
H	10.220906	−3.744820	3.070434
H	11.862495	−3.400905	3.677963
H	11.826426	−5.625196	2.799491
H	11.762969	−6.273132	−0.569325
H	13.737022	−3.811719	1.846049
H	12.723250	−3.383478	−0.261127
H	13.680135	−4.807176	−0.717168
H	14.783756	−5.810252	1.242165
N	10.678823	−4.693920	−1.306343
C	10.149947	−5.548207	−2.322174
O	10.497642	−6.747855	−2.361363
N	9.280107	−5.010382	−3.222391
C	8.917850	−3.722533	−3.142057
N	8.047633	−3.254034	−4.061487
C	9.418489	−2.851882	−2.121337
C	10.281692	−3.388455	−1.221334
H	7.638329	−3.889217	−4.735597
H	7.691937	−2.309345	−4.008961
H	9.105758	−1.817400	−2.054564
H	10.682069	−2.829489	−0.383216

(TCG)$^{2-}$

E: −13699.39 kcal mol^{-1}

O	−0.300578	−0.345682	−0.147320
C	−0.188200	−0.005797	1.257470
C	1.254151	−0.152025	1.723096

O	1.645218	−1.564972	1.780150
C	2.725965	−1.829295	0.856113
C	2.310743	0.542773	0.839252
C	2.831471	−0.593095	−0.045153
O	3.313831	1.092787	1.742646
H	−1.247764	−0.382544	−0.367655
H	−0.822941	−0.665144	1.862829
H	−0.500942	1.034702	1.425634
H	1.313979	0.244562	2.742355
H	3.647680	−2.015860	1.409716
H	1.888142	1.360283	0.249914
H	2.176535	−0.685902	−0.913973
H	3.845571	−0.416845	−0.394124
N	2.447281	−3.081634	0.130122
C	3.276332	−4.185963	0.355436
O	4.206616	−4.198962	1.163582
N	2.970337	−5.280400	−0.437161
C	1.929453	−5.412513	−1.364146
O	1.801096	−6.472183	−2.002099
C	1.067269	−4.247212	−1.477928
C	−0.110904	−4.298735	−2.412522
C	1.363629	−3.154640	−0.728282
H	3.592999	−6.078927	−0.329319
H	−0.796072	−5.109304	−2.135312
H	0.215774	−4.491842	−3.441658
H	−0.661239	−3.353847	−2.393558
H	0.742635	−2.263949	−0.744380
P	4.784780	1.637738	1.221554
O	4.758103	1.958333	−0.253191
O	5.203323	2.672504	2.242119
O	5.727886	0.294886	1.368436
C	5.990863	−0.218731	2.701477
C	7.210899	−1.126600	2.693191
O	6.925990	−2.385590	2.014634
C	7.742419	−2.523734	0.822259
C	8.458553	−0.537667	2.003567
C	8.417959	−1.163793	0.605960
O	9.602554	−0.924775	2.822004
H	5.117654	−0.781779	3.052878
H	6.173360	0.617156	3.386712
H	7.448800	−1.360934	3.737394
H	8.468046	−3.322673	0.981236
H	8.428097	0.554220	1.957495
H	7.818490	−0.512784	−0.032216
H	9.403544	−1.256065	0.159008
N	6.899772	−2.989904	−0.291100
C	6.873655	−4.393228	−0.594808
O	7.560339	−5.175221	0.085378
N	6.096350	−4.808331	−1.636096
C	5.322731	−3.943058	−2.300486
N	4.566104	−4.420721	−3.312548
C	5.244744	−2.558570	−1.942247
C	6.046415	−2.135911	−0.929267
H	4.644558	−5.393552	−3.581024
H	4.011674	−3.802208	−3.888088
H	4.574063	−1.875471	−2.449597
H	6.030146	−1.115490	−0.566827
P	11.169641	−0.942658	2.311373
O	11.371735	−0.100810	1.074133
O	11.990808	−0.701142	3.559653
O	11.352117	−2.501892	1.816768
C	11.202553	−3.565748	2.790614
C	11.775525	−4.861277	2.235198
O	10.966136	−5.363479	1.128452
C	11.682047	−5.244150	−0.123467
C	13.209612	−4.753483	1.691366
C	13.006720	−4.522205	0.187641
O	13.870219	−6.017115	1.971065
H	10.138640	−3.702792	3.018921
H	11.733976	−3.303567	3.713698
H	11.734508	−5.610981	3.034383
H	11.840053	−6.250279	−0.520894
H	13.761423	−3.932161	2.160822
H	12.922151	−3.451284	−0.000852
H	13.831636	−4.917857	−0.409751
H	14.765185	−5.972121	1.587325
N	10.850012	−4.559352	−1.111947
C	10.634214	−3.189426	−1.263210
N	9.779975	−2.913708	−2.230674
C	9.399364	−4.153433	−2.743414
C	8.501060	−4.484625	−3.802247
O	7.842670	−3.731112	−4.542759
N	8.412999	−5.893780	−3.967926
C	9.093253	−6.838595	−3.229736
N	8.840702	−8.148115	−3.508201
N	9.934697	−6.519195	−2.251627
C	10.046966	−5.186398	−2.051503
H	11.117889	−2.468449	−0.621065
H	7.778147	−6.203213	−4.700575
H	8.440918	−8.397298	−4.405404
H	9.483460	−8.822328	−3.107701

$(TCT)^{2-}$

E: −13396.29 kcal mol^{-1}

O	−0.286610	−0.229018	−0.138005
C	−0.186191	0.108029	1.268360
C	1.248083	−0.059370	1.751767
O	1.618922	−1.478013	1.809132
C	2.702905	−1.755484	0.892247
C	2.325300	0.622272	0.883182
C	2.833561	−0.517070	−0.002775
O	3.329570	1.149031	1.800336
H	−1.231763	−0.262356	−0.367227
H	−0.837519	−0.542654	1.865200
H	−0.486065	1.152765	1.433774
H	1.300744	0.332932	2.772975
H	3.616651	−1.956929	1.453616
H	1.923692	1.451339	0.295275
H	2.183111	−0.595037	−0.876266
H	3.851402	−0.351134	−0.344481
N	2.413991	−3.002392	0.160660
C	3.215380	−4.123170	0.405347
O	4.125695	−4.154124	1.235573
N	2.908153	−5.211211	−0.395277
C	1.888600	−5.322377	−1.348682
O	1.758778	−6.377245	−1.993988
C	1.048699	−4.142423	−1.478498
C	−0.110205	−4.173673	−2.437835
C	1.347362	−3.056081	−0.720772
H	3.510876	−6.022533	−0.271432
H	−0.803824	−4.985695	−2.187526
H	0.236559	−4.351614	−3.463375
H	−0.656863	−3.226726	−2.415233
H	0.741971	−2.155128	−0.748261
P	4.829949	1.642786	1.314250
O	4.860157	1.939183	−0.165161
O	5.251099	2.678505	2.332322
O	5.728669	0.273799	1.508983
C	5.917688	−0.245458	2.852464
C	7.113434	−1.184960	2.896064
O	6.826810	−2.438301	2.207424
C	7.681496	−2.606153	1.050662
C	8.401850	−0.623560	2.262570
C	8.447571	−1.284704	0.879301
O	9.501397	−1.001957	3.140157

H	5.014705	−0.783998	3.164544
H	6.093065	0.585767	3.545236
H	7.300208	−1.424821	3.949247
H	8.350692	−3.450892	1.218257
H	8.381297	0.466223	2.185082
H	7.957290	−0.623855	0.163535
H	9.469264	−1.450143	0.545265
N	6.858278	−2.995837	−0.111120
C	6.815272	−4.375158	−0.506466
O	7.497839	−5.209703	0.114539
N	6.036197	−4.711542	−1.573637
C	5.291739	−3.791586	−2.197904
N	4.532219	−4.192987	−3.238981
C	5.251685	−2.426420	−1.768712
C	6.040334	−2.084202	−0.716565
H	4.581786	−5.153092	−3.556521
H	3.989859	−3.531089	−3.776775
H	4.614849	−1.697045	−2.253917
H	6.042555	−1.086584	−0.293734
P	11.082543	−0.773630	2.719066
O	11.213510	0.263329	1.630260
O	11.822507	−0.631197	4.031304
O	11.467141	−2.215450	2.016685
C	11.407322	−3.399445	2.855573
C	11.794745	−4.624661	2.050282
O	10.769333	−4.881335	1.044538
C	11.403416	−5.145424	−0.224926
C	13.148579	−4.532818	1.304330
C	12.749378	−4.408232	−0.173467
O	13.872738	−5.760953	1.582251
H	10.388229	−3.523611	3.241378
H	12.095212	−3.290071	3.702609
H	11.833633	−5.479084	2.739308
H	11.539213	−6.220045	−0.363185
H	13.736137	−3.673425	1.640700
H	12.631524	−3.351907	−0.428009
H	13.488429	−4.850534	−0.846036
H	14.747196	−5.688697	1.157442
N	10.491751	−4.717779	−1.293080
C	9.842679	−5.696117	−2.052656
O	9.995338	−6.911533	−1.890407
N	9.013455	−5.183718	−3.032971
C	8.731239	−3.840273	−3.311893
O	7.967069	−3.548631	−4.245242
C	9.389032	−2.888544	−2.429738
C	9.132074	−1.421008	−2.630052
C	10.220297	−3.373924	−1.473348
H	8.528582	−5.876973	−3.599339
H	9.436150	−1.111273	−3.637129
H	9.685653	−0.823075	−1.902375
H	8.063964	−1.194840	−2.534921
H	10.726309	−2.717161	−0.776308

(TGA)$^{2-}$

E: −14101.93 kcal mol^{-1}

O	−0.249714	−0.346925	−0.248178
C	−0.183754	0.006422	1.156265
C	1.247311	−0.101569	1.665300
O	1.666801	−1.502609	1.769225
C	2.779241	−1.773152	0.883856
C	2.314881	0.597636	0.799243
C	2.910704	−0.545426	−0.028932
O	3.261337	1.209219	1.722685
H	−1.189192	−0.420719	−0.490816
H	−0.820925	−0.661468	1.749411
H	−0.524068	1.041144	1.305880
H	1.268182	0.320046	2.676092
H	3.682605	−1.950950	1.469895
H	1.889504	1.380222	0.166320
H	2.320160	−0.668285	−0.939394
H	3.941840	−0.349726	−0.312421
N	2.521530	−3.037190	0.166719
C	3.319762	−4.149569	0.452012
O	4.230570	−4.154715	1.282654
N	3.002472	−5.269795	−0.300665
C	1.950974	−5.429048	−1.213148
O	1.779303	−6.525621	−1.773113
C	1.128332	−4.245282	−1.400054
C	−0.050452	−4.316264	−2.332711
C	1.447706	−3.124749	−0.702701
H	3.571411	−6.092759	−0.113692
H	−0.754400	−5.094989	−2.013777
H	0.270560	−4.570348	−3.350393
H	−0.579023	−3.359175	−2.362254
H	0.846340	−2.222951	−0.766500
P	4.671424	1.910140	1.219508
O	4.638273	2.194555	−0.262481
O	4.947248	3.008956	2.222791
O	5.759045	0.689025	1.418383
C	6.017614	0.223516	2.768741
C	7.143095	−0.798619	2.779805
O	6.729038	−2.022426	2.094477
C	7.610331	−2.266459	0.968699
C	8.459233	−0.363509	2.100520
C	8.307344	−0.936416	0.690574
O	9.535723	−0.969284	2.885687
H	5.109680	−0.239682	3.174354
H	6.296178	1.071506	3.405926
H	7.342720	−1.049842	3.828107
H	8.331193	−3.043643	1.232953
H	8.599206	0.720515	2.104303
H	7.653068	−0.270019	0.123452
H	9.253262	−1.047813	0.164510
N	6.851073	−2.817177	−0.138017
C	5.971491	−2.193501	−1.015445
N	5.454097	−3.032430	−1.897138
C	6.021054	−4.272986	−1.593682
C	5.868319	−5.556297	−2.201688
O	5.146022	−5.900286	−3.152320
N	6.713479	−6.520759	−1.568007
C	7.571014	−6.279250	−0.512595
N	8.335224	−7.307277	−0.041499
N	7.659416	−5.091951	0.071614
C	6.889909	−4.149003	−0.501712
H	5.767369	−1.135082	−0.950496
H	6.678660	−7.452886	−1.975218
H	8.486031	−8.102911	−0.652583
H	9.168626	−6.989749	0.458417
P	11.033251	−1.372040	2.324506
O	11.427743	−0.516781	1.143793
O	11.899024	−1.453742	3.561192
O	10.806193	−2.880166	1.698927
C	10.669391	−4.043246	2.554174
C	11.466245	−5.219380	1.995718
O	10.860339	−5.778917	0.787131
C	11.620004	−5.406802	−0.389141
C	12.911890	−4.883286	1.600097
C	12.808879	−4.558205	0.101369
O	13.710503	−6.069750	1.852331
H	9.610071	−4.318232	2.612079
H	11.041139	−3.812043	3.558246
H	11.452055	−6.015661	2.748195
H	11.934471	−6.327961	−0.885122
H	13.303157	−4.037391	2.175065
H	12.610479	−3.493346	−0.024520

H	13.726152	−4.806416	−0.437775
H	14.618743	−5.883150	1.551786
N	10.752255	−4.724317	−1.352323
C	10.317882	−3.402887	−1.342310
N	9.435307	−3.139260	−2.291606
C	9.260918	−4.348920	−2.961990
C	8.429323	−4.736985	−4.036079
N	7.586055	−3.876717	−4.663821
N	8.493147	−6.024721	−4.463193
C	9.326926	−6.877634	−3.836346
N	10.139865	−6.629920	−2.792599
C	10.070465	−5.346541	−2.393054
H	10.674545	−2.702246	−0.602314
H	6.866516	−4.278286	−5.255519
H	7.350905	−3.009895	−4.193647
H	9.336261	−7.896809	−4.219725

(TGC)$^{2–}$

E: −13702.61 kcal mol$^{−1}$

O	−0.302516	−0.427635	−0.174669
C	−0.195100	−0.029243	1.215035
C	1.252195	−0.115437	1.681925
O	1.684935	−1.512816	1.793556
C	2.768858	−1.782227	0.871798
C	2.285441	0.573392	0.766959
C	2.826182	−0.580587	−0.079251
O	3.282065	1.176073	1.643521
H	−1.248860	−0.506205	−0.387204
H	−0.809235	−0.681703	1.848387
H	−0.536918	1.007890	1.343159
H	1.301777	0.323537	2.684198
H	3.699265	−1.914234	1.427243
H	1.838071	1.359195	0.153155
H	2.163744	−0.726518	−0.934862
H	3.827405	−0.386343	−0.451325
N	2.525102	−3.073565	0.202989
C	3.365517	−4.151832	0.498715
O	4.278135	−4.112043	1.325992
N	3.091108	−5.290891	−0.243008
C	2.040629	−5.500682	−1.147377
O	1.918094	−6.604793	−1.705135
C	1.161237	−4.357588	−1.328824
C	−0.025104	−4.488987	−2.245001
C	1.442374	−3.216461	−0.648293
H	3.691005	−6.090013	−0.048455
H	−0.690332	−5.294851	−1.910448
H	0.294293	−4.738445	−3.264419
H	−0.594753	−3.555883	−2.276293
H	0.803754	−2.340861	−0.713755
P	4.700584	1.829019	1.100360
O	4.695057	1.982735	−0.401416
O	4.972677	3.009202	2.007396
O	5.780451	0.626558	1.419776
C	6.014055	0.254598	2.802573
C	7.147403	−0.757136	2.905015
O	6.784672	−2.007567	2.241235
C	7.624663	−2.208082	1.079125
C	8.493424	−0.342164	2.280013
C	8.382003	−0.895997	0.859465
O	9.527239	−0.990855	3.085588
H	5.101761	−0.193279	3.215506
H	6.268769	1.145016	3.390274
H	7.289948	−0.976096	3.969293
H	8.305976	−3.039914	1.274613
H	8.658332	0.738236	2.302273
H	7.786427	−0.199118	0.266714
H	9.349622	−1.037280	0.381212
N	6.821437	−2.639394	−0.053893
C	5.988465	−1.900993	−0.890342
N	5.445636	−2.640256	−1.842570
C	5.945416	−3.928673	−1.634500
C	5.774475	−5.139765	−2.372040
O	5.082069	−5.358646	−3.381816
N	6.557065	−6.193829	−1.807491
C	7.407191	−6.079394	−0.724754
N	8.140522	−7.165734	−0.349460
N	7.520657	−4.957662	−0.027346
C	6.797286	−3.938226	−0.522890
H	5.832273	−0.843588	−0.733667
H	6.513192	−7.077678	−2.311013
H	8.297614	−7.900757	−1.030377
H	8.947374	−6.928615	0.228812
P	11.063886	−1.302702	2.568861
O	11.477826	−0.344332	1.478508
O	11.874202	−1.451778	3.836320
O	10.917793	−2.770122	1.825137
C	10.727005	−3.973694	2.616816
C	11.390586	−5.174845	1.950484
O	10.663587	−5.602447	0.756733
C	11.465733	−5.399050	−0.434705
C	12.842822	−4.941566	1.496278
C	12.703598	−4.599701	0.005795
O	13.561444	−6.186112	1.708721
H	9.653279	−4.165479	2.726947
H	11.170543	−3.832928	3.608386
H	11.353858	−6.004748	2.666121
H	11.728228	−6.370588	−0.851951
H	13.319086	−4.132813	2.060391
H	12.545644	−3.525173	−0.105648
H	13.589242	−4.881639	−0.569056
H	14.468134	−6.063634	1.372471
N	10.635476	−4.748066	−1.463080
C	10.177014	−5.510493	−2.584510
O	10.558983	−6.690360	−2.725574
N	9.320106	−4.911235	−3.458020
C	8.933797	−3.639962	−3.281638
N	8.088799	−3.107601	−4.192283
C	9.386343	−2.854190	−2.174541
C	10.212488	−3.459220	−1.282644
H	7.678037	−3.711670	−4.894707
H	7.646186	−2.214627	−4.018848
H	9.055645	−1.833010	−2.031221
H	10.552093	−2.971340	−0.376383

(TGG)$^{2–}$

E: −14265.80 kcal mol$^{−1}$

O	−0.245924	−0.388199	−0.252776
C	−0.181947	−0.028872	1.150135
C	1.248943	−0.131384	1.660714
O	1.671789	−1.531026	1.769633
C	2.788263	−1.800848	0.889638
C	2.315791	0.568233	0.793899
C	2.919436	−0.576023	−0.027226
O	3.256728	1.188830	1.716734
H	−1.185072	−0.461900	−0.496748
H	−0.818037	−0.695749	1.745653
H	−0.524864	1.005663	1.295306
H	1.267624	0.293700	2.670117
H	3.690086	−1.974093	1.479403
H	1.888419	1.345595	0.155972
H	2.333159	−0.704973	−0.939627
H	3.951598	−0.378552	−0.306660

N	2.536726	−3.067750	0.175055
C	3.344253	−4.174004	0.457326
O	4.258934	−4.172336	1.283587
N	3.031405	−5.296962	−0.293416
C	1.978497	−5.463318	−1.203007
O	1.814122	−6.560622	−1.763918
C	1.146212	−4.285813	−1.386306
C	−0.035159	−4.364922	−2.314952
C	1.460543	−3.162789	−0.690625
H	3.606909	−6.115738	−0.108449
H	−0.732297	−5.149007	−1.993983
H	0.284257	−4.616137	−3.333969
H	−0.570800	−3.411573	−2.342128
H	0.853370	−2.264726	−0.753258
P	4.662565	1.896603	1.210739
O	4.622057	2.186440	−0.270031
O	4.937125	2.992901	2.217067
O	5.755301	0.678818	1.401181
C	6.020695	0.211603	2.749649
C	7.157119	−0.798023	2.755940
O	6.751133	−2.029584	2.079726
C	7.633695	−2.276072	0.955439
C	8.463051	−0.350073	2.064696
C	8.314951	−0.941390	0.661978
O	9.552456	−0.927064	2.853193
H	5.118115	−0.262035	3.155377
H	6.291449	1.060658	3.388906
H	7.368223	−1.042047	3.803736
H	8.363897	−3.042700	1.226290
H	8.584716	0.736109	2.054401
H	7.651779	−0.288894	0.088766
H	9.260455	−1.049684	0.134974
N	6.878734	−2.847305	−0.144355
C	5.992149	−2.244283	−1.030631
N	5.484334	−3.099487	−1.901227
C	6.063640	−4.329747	−1.582260
C	5.929813	−5.621291	−2.176453
O	5.232244	−5.983478	−3.139698
N	6.765146	−6.574459	−1.512177
C	7.599605	−6.315708	−0.441652
N	8.346585	−7.338176	0.068804
N	7.683508	−5.116864	0.116865
C	6.924524	−4.184370	−0.485996
H	5.775878	−1.187439	−0.979218
H	6.729278	−7.516387	−1.897024
H	8.511017	−8.143120	−0.526450
H	9.174501	−7.008025	0.570282
P	11.058442	−1.304851	2.298422
O	11.428827	−0.474922	1.091860
O	11.929495	−1.327101	3.534230
O	10.871036	−2.835646	1.719107
C	10.712853	−3.966974	2.610610
C	11.451985	−5.185160	2.064363
O	10.818256	−5.725559	0.862809
C	11.591578	−5.400034	−0.318213
C	12.912839	−4.926013	1.665271
C	12.827209	−4.614825	0.162963
O	13.651784	−6.147548	1.932604
H	9.646215	−4.202141	2.703751
H	11.117833	−3.722903	3.599028
H	11.400113	−5.971099	2.826214
H	11.853510	−6.340712	−0.807770
H	13.346079	−4.093850	2.230341
H	12.690463	−3.542137	0.024031
H	13.728346	−4.921450	−0.373282
H	14.567221	−6.010651	1.627350
N	10.765306	−4.674860	−1.287077
C	10.450223	−3.314775	−1.321179
N	9.613284	−3.011204	−2.294642
C	9.343527	−4.220360	−2.933609
C	8.507049	−4.512827	−4.053660
O	7.825016	−3.742201	−4.752361
N	8.510512	−5.907931	−4.335701
C	9.231404	−6.867641	−3.657866
N	9.042476	−8.166084	−4.025688
N	10.034169	−6.579051	−2.639218
C	10.047186	−5.264984	−2.316716
H	10.851615	−2.624539	−0.595111
H	7.906368	−6.193890	−5.103432
H	8.666004	−8.373245	−4.943786
H	9.712344	−8.835021	−3.662579

$(TGT)^{2-}$

E: −13960.96 kcal mol^{-1}

O	−0.274967	−0.353234	−0.165348
C	−0.178861	0.002631	1.236595
C	1.257367	−0.134570	1.724107
O	1.656478	−1.544564	1.795219
C	2.746910	−1.806192	0.877458
C	2.325853	0.557085	0.853283
C	2.837747	−0.578432	−0.034698
O	3.327036	1.084491	1.774842
H	−1.219727	−0.401838	−0.393334
H	−0.819624	−0.651058	1.841713
H	−0.495857	1.044345	1.389415
H	1.298967	0.268627	2.741700
H	3.668053	−1.969444	1.439240
H	1.918332	1.386741	0.270121
H	2.168735	−0.676375	−0.891861
H	3.843270	−0.404417	−0.404555
N	2.489439	−3.075433	0.175358
C	3.299608	−4.176435	0.468898
O	4.213956	−4.162674	1.295533
N	2.993962	−5.306073	−0.274440
C	1.949557	−5.480175	−1.193756
O	1.797115	−6.580142	−1.751290
C	1.113026	−4.307220	−1.387365
C	−0.060960	−4.395836	−2.324298
C	1.416596	−3.180069	−0.693546
H	3.565573	−6.124397	−0.075118
H	−0.767358	−5.167670	−1.994139
H	0.265802	−4.670136	−3.334723
H	−0.588215	−3.439027	−2.375425
H	0.805362	−2.285547	−0.762114
P	4.817180	1.618143	1.305090
O	4.865822	1.886360	−0.179723
O	5.188585	2.687258	2.308604
O	5.759724	0.286191	1.539396
C	5.948225	−0.218188	2.885833
C	7.174623	−1.117721	2.953900
O	6.961241	−2.386408	2.260907
C	7.748623	−2.455230	1.053423
C	8.458588	−0.518628	2.349442
C	8.506823	−1.120895	0.939389
O	9.557206	−0.924511	3.215582
H	5.060430	−0.785842	3.190131
H	6.087624	0.620998	3.577800
H	7.341085	−1.358215	4.009599
H	8.419687	−3.316172	1.125581
H	8.429433	0.573574	2.316664
H	7.997494	−0.440147	0.256726
H	9.526976	−1.258213	0.586644
N	6.884022	−2.728684	−0.099894
C	6.054529	−1.832920	−0.775035
N	5.364125	−2.408374	−1.740946

C	5.741962	−3.749389	−1.706929
C	5.312988	−4.849835	−2.507200
O	4.503264	−4.872371	−3.449552
N	5.945723	−6.055818	−2.085658
C	6.864191	−6.170899	−1.059745
N	7.340065	−7.414664	−0.787140
N	7.261889	−5.134200	−0.329798
C	6.675168	−3.968624	−0.682001
H	5.999212	−0.794593	−0.485677
H	5.679128	−6.888261	−2.607768
H	7.250972	−8.150115	−1.478093
H	8.157815	−7.463122	−0.190161
P	11.142883	−0.784077	2.774432
O	11.321644	0.254263	1.693637
O	11.905847	−0.697759	4.077683
O	11.429444	−2.239224	2.052370
C	11.333740	−3.426371	2.883268
C	11.780219	−4.645050	2.099577
O	10.804922	−4.934641	1.050140
C	11.508747	−5.185334	−0.185024
C	13.159535	−4.514860	1.408115
C	12.810425	−4.380912	−0.080986
O	13.897615	−5.732425	1.695230
H	10.296799	−3.559366	3.214871
H	11.975081	−3.314098	3.765390
H	11.811985	−5.497034	2.791424
H	11.706062	−6.253012	−0.300900
H	13.715047	−3.647913	1.777468
H	12.641143	−3.327491	−0.320091
H	13.594505	−4.771181	−0.734417
H	14.781535	−5.641756	1.294175
N	10.622962	−4.820169	−1.296544
C	10.131354	−5.835947	−2.119828
O	10.412792	−7.031242	−1.988194
N	9.280403	−5.386493	−3.114589
C	8.886846	−4.067041	−3.384359
O	8.129368	−3.833789	−4.338484
C	9.424334	−3.074564	−2.465929
C	9.056215	−1.632049	−2.670597
C	10.247038	−3.500613	−1.474601
H	8.934231	−6.103787	−3.749189
H	9.372545	−1.293043	−3.664515
H	9.532162	−0.995977	−1.920592
H	7.971237	−1.493243	−2.615536
H	10.660651	−2.816071	−0.743367

(TTA)$^{2-}$

E: −13795.53 kcal mol^{-1}

H: −13323.66 kcal mol^{-1}

G: −13410.46 kcal mol^{-1}

O	−0.303695	−0.247523	−0.062709
C	−0.181537	0.056746	1.349399
C	1.264004	−0.104678	1.800136
O	1.646622	−1.520919	1.840484
C	2.713994	−1.788474	0.904508
C	2.312622	0.595007	0.910807
C	2.843138	−0.537443	0.027413
O	3.313422	1.166070	1.801948
H	−1.252182	−0.275239	−0.278881
H	−0.814824	−0.614796	1.942459
H	−0.489129	1.093926	1.544456
H	1.337409	0.280513	2.822718
H	3.634330	−2.011414	1.447190
H	1.877844	1.402132	0.316386
H	2.209726	−0.612763	−0.857953
H	3.866384	−0.363804	−0.295105
N	2.399273	−3.017618	0.148380
C	3.183300	−4.156624	0.354923
O	4.127076	−4.215282	1.146701
N	2.821063	−5.232138	−0.440306
C	1.766453	−5.313360	−1.360507
O	1.578369	−6.366137	−1.992324
C	0.963918	−4.105452	−1.468170
C	−0.214143	−4.092822	−2.404271
C	1.308731	−3.036540	−0.705869
H	3.380526	−6.073143	−0.313415
H	−0.919399	−4.895691	−2.157100
H	0.109923	−4.257006	−3.439661
H	−0.739900	−3.135125	−2.352306
H	0.723791	−2.121726	−0.707246
P	4.760088	1.741866	1.243528
O	4.688572	2.060753	−0.229539
O	5.184605	2.784032	2.254182
O	5.730212	0.414907	1.367787
C	6.006810	−0.092449	2.700123
C	7.172342	−1.063846	2.662963
O	6.784324	−2.300236	1.985799
C	7.654832	−2.543103	0.858027
C	8.435708	−0.549211	1.938857
C	8.343357	−1.208780	0.559525
O	9.571216	−0.980018	2.743902
H	5.116331	−0.601004	3.089446
H	6.257919	0.740807	3.366727
H	7.427874	−1.314290	3.699103
H	8.378213	−3.321326	1.110349
H	8.456619	0.541129	1.866398
H	7.724594	−0.570471	−0.073892
H	9.310915	−1.331347	0.081713
N	6.854590	−3.106969	−0.244078
C	6.902327	−4.493037	−0.438433
O	7.586965	−5.260818	0.238094
N	6.116071	−4.939617	−1.489737
C	5.270644	−4.188073	−2.317293
O	4.627548	−4.747550	−3.217678
C	5.226326	−2.768847	−2.002014
C	4.344606	−1.882181	−2.837134
C	6.000212	−2.310901	−0.983434
H	6.170551	−5.938390	−1.680102
H	4.617520	−1.961846	−3.896115
H	4.436627	−0.837069	−2.534265
H	3.293121	−2.182558	−2.757255
H	5.984052	−1.268948	−0.686666
P	11.136737	−0.998826	2.223229
O	11.322739	−0.169080	0.975560
O	11.967870	−0.746263	3.461892
O	11.316387	−2.561683	1.740041
C	11.197463	−3.623804	2.719364
C	11.805576	−4.905703	2.168836
O	11.017649	−5.433080	1.057757
C	11.725711	−5.271071	−0.192526
C	13.238676	−4.763781	1.631345
C	13.037708	−4.530073	0.126761
O	13.924416	−6.013830	1.907832
H	10.138055	−3.789078	2.950132
H	11.722713	−3.343372	3.640432
H	11.777778	−5.655224	2.968438
H	11.898961	−6.264995	−0.613467
H	13.770594	−3.932571	2.106297
H	12.940813	−3.460010	−0.059190
H	13.870658	−4.912701	−0.467669
H	14.819189	−5.949510	1.526568
N	10.871011	−4.582888	−1.162945
C	10.635891	−3.220008	−1.300847
N	9.733948	−2.941108	−2.228845

C	9.342680	−4.182087	−2.728324
C	8.403952	−4.563953	−3.713343
N	7.672052	−3.669167	−4.413413
N	8.230622	−5.890603	−3.953825
C	8.962289	−6.778812	−3.251152
N	9.878780	−6.533856	−2.296974
C	10.031704	−5.215072	−2.072834
H	11.137789	−2.493887	−0.678187
H	6.950741	−3.993196	−5.045270
H	7.743108	−2.680674	−4.213717
H	8.783928	−7.826874	−3.487008

$(TTC)^{2-}$

E: −13396.72 kcal mol^{-1}

O	−0.317025	−0.200098	−0.047135
C	−0.209563	0.078195	1.371671
C	1.233244	−0.079817	1.832384
O	1.625680	−1.493983	1.851940
C	2.702123	−1.737934	0.920261
C	2.284389	0.642076	0.963969
C	2.828577	−0.471388	0.065517
O	3.274638	1.203298	1.872879
H	−1.263391	−0.241715	−0.269818
H	−0.841911	−0.610303	1.946157
H	−0.528098	1.108856	1.583818
H	1.295085	0.288420	2.861929
H	3.619576	−1.962691	1.467247
H	1.849333	1.456972	0.380304
H	2.202502	−0.535706	−0.825913
H	3.851949	−0.282515	−0.247086
N	2.403786	−2.956586	0.140519
C	3.189033	−4.095577	0.342714
O	4.114626	−4.165229	1.154718
N	2.852447	−5.156669	−0.482629
C	1.809679	−5.230331	−1.417368
O	1.639458	−6.272750	−2.070834
C	0.997687	−4.027595	−1.512871
C	−0.172748	−4.011594	−2.458373
C	1.324801	−2.968929	−0.728621
H	3.412446	−5.997734	−0.357551
H	−0.880222	−4.814469	−2.217699
H	0.159071	−4.174272	−3.491354
H	−0.698825	−3.054038	−2.408115
H	0.732673	−2.058770	−0.722035
P	4.727969	1.789697	1.344218
O	4.688158	2.100782	−0.131779
O	5.121852	2.839155	2.359459
O	5.706090	0.470989	1.496543
C	5.960521	−0.029141	2.836214
C	7.122818	−1.005947	2.819122
O	6.737510	−2.238413	2.132618
C	7.612520	−2.477730	1.010102
C	8.397882	−0.495356	2.114404
C	8.319794	−1.146756	0.730557
O	9.526864	−0.944180	2.917536
H	5.062786	−0.533192	3.214453
H	6.204750	0.806797	3.501910
H	7.360020	−1.260131	3.858617
H	8.324359	−3.267794	1.258744
H	8.426790	0.595036	2.049052
H	7.721953	−0.500257	0.085997
H	9.300743	−1.272218	0.279652
N	6.819938	−3.016214	−0.110407
C	6.887069	−4.394079	−0.352865
O	7.549101	−5.181603	0.323294
N	6.154781	−4.806231	−1.454949
C	5.349899	−4.028603	−2.297282
O	4.761282	−4.556131	−3.252884
C	5.275886	−2.622516	−1.930667
C	4.428177	−1.712185	−2.775381
C	5.996224	−2.197591	−0.859950
H	6.229513	−5.796829	−1.679185
H	4.741615	−1.765741	−3.824609
H	4.509370	−0.675470	−2.442432
H	3.373944	−2.012531	−2.743941
H	5.959530	−1.167258	−0.526144
P	11.094987	−0.867640	2.400486
O	11.237778	0.053535	1.213532
O	11.916753	−0.666242	3.654271
O	11.337113	−2.390988	1.815875
C	11.281411	−3.497569	2.755993
C	11.785326	−4.772494	2.100201
O	10.852228	−5.210724	1.067275
C	11.515963	−5.291010	−0.215796
C	13.168739	−4.667477	1.427233
C	12.838893	−4.521302	−0.064017
O	13.877553	−5.902599	1.716996
H	10.245528	−3.641611	3.086267
H	11.903839	−3.270920	3.629580
H	11.819833	−5.547865	2.876093
H	11.675196	−6.336635	−0.480448
H	13.739658	−3.813798	1.806610
H	12.709516	−3.463083	−0.303940
H	13.622917	−4.929283	−0.707007
H	14.748155	−5.850963	1.281499
N	10.610894	−4.759083	−1.247083
C	10.012316	−5.645594	−2.195939
O	10.284179	−6.863650	−2.158364
N	9.163134	−5.114318	−3.120762
C	8.879036	−3.804703	−3.119527
N	8.025917	−3.343703	−4.058196
C	9.423205	−2.907275	−2.145458
C	10.274447	−3.434412	−1.227696
H	7.651656	−3.976058	−4.754993
H	7.812228	−2.358595	−4.130557
H	9.159238	−1.856983	−2.131940
H	10.710627	−2.848922	−0.426045

$(TTG)^{2-}$

E: −13959.03 kcal mol^{-1}

O	−0.297690	−0.281672	−0.058653
C	−0.182475	−0.009090	1.360456
C	1.263738	−0.161864	1.811855
O	1.663989	−1.573410	1.828270
C	2.736577	−1.812421	0.890668
C	2.306957	0.566804	0.939757
C	2.857533	−0.542914	0.038602
O	3.293956	1.134797	1.847582
H	−1.245296	−0.320825	−0.276856
H	−0.808368	−0.702401	1.936168
H	−0.503948	1.019212	1.578869
H	1.329345	0.205517	2.841519
H	3.657251	−2.038225	1.431568
H	1.864699	1.378420	0.357018
H	2.232059	−0.608669	−0.853293
H	3.881613	−0.351584	−0.272497
N	2.433223	−3.029816	0.110843
C	3.222570	−4.168049	0.301426
O	4.167605	−4.233369	1.091128
N	2.863191	−5.234796	−0.506860
C	1.805340	−5.309770	−1.423901
O	1.619090	−6.356015	−2.067104

C	0.997611	−4.103871	−1.514775
C	−0.184312	−4.085301	−2.445900
C	1.340513	−3.042860	−0.740634
H	3.426945	−6.074721	−0.392515
H	−0.891970	−4.885298	−2.196293
H	0.134468	−4.249553	−3.482723
H	−0.705989	−3.125705	−2.390394
H	0.751662	−2.130564	−0.729599
P	4.729941	1.753201	1.307844
O	4.653321	2.110563	−0.156207
O	5.132180	2.776111	2.347067
O	5.725278	0.442480	1.398687
C	6.005547	−0.094485	2.718743
C	7.174416	−1.060204	2.658303
O	6.787433	−2.283898	1.958559
C	7.678841	−2.520182	0.845003
C	8.434365	−0.525272	1.941048
C	8.355573	−1.177708	0.557234
O	9.571715	−0.944060	2.749893
H	5.117366	−0.614600	3.098046
H	6.254902	0.724433	3.403520
H	7.435332	−1.328610	3.688775
H	8.407983	−3.289415	1.108964
H	8.440721	0.565614	1.874629
H	7.731478	−0.542658	−0.074689
H	9.326491	−1.286673	0.082592
N	6.900466	−3.093587	−0.266447
C	6.968541	−4.477839	−0.461879
O	7.652263	−5.239489	0.222879
N	6.200905	−4.932516	−1.523768
C	5.343718	−4.192904	−2.352059
O	4.707102	−4.764286	−3.248866
C	5.274488	−2.775957	−2.031514
C	4.373556	−1.901259	−2.858572
C	6.036348	−2.309467	−1.008236
H	6.247595	−5.934837	−1.695154
H	4.650672	−1.962666	−3.917393
H	4.440395	−0.857815	−2.542981
H	3.329121	−2.226583	−2.782367
H	6.000316	−1.269323	−0.706987
P	11.141689	−0.945015	2.242705
O	11.328292	−0.123960	0.989577
O	11.959039	−0.670425	3.485994
O	11.345809	−2.509819	1.776557
C	11.218758	−3.563602	2.763710
C	11.819478	−4.853512	2.223921
O	11.027220	−5.384645	1.117985
C	11.739870	−5.247480	−0.133149
C	13.253554	−4.723769	1.685501
C	13.054013	−4.506588	0.178684
O	13.934766	−5.973446	1.976930
H	10.158017	−3.720829	2.994009
H	11.744039	−3.279116	3.683585
H	11.788224	−5.595838	3.030275
H	11.911254	−6.248991	−0.536389
H	13.788662	−3.889393	2.151248
H	12.958434	−3.438501	−0.019597
H	13.886403	−4.897368	−0.411364
H	14.829950	−5.916461	1.595381
N	10.894184	−4.569870	−1.117426
C	10.658244	−3.202445	−1.263723
N	9.782806	−2.936830	−2.214858
C	9.408553	−4.180984	−2.721494
C	8.492282	−4.521307	−3.761631
O	7.804615	−3.775357	−4.481274
N	8.406621	−5.934064	−3.917477
C	9.125492	−6.871977	−3.202927
N	8.887963	−8.181799	−3.480165
N	9.983672	−6.540678	−2.242792
C	10.084269	−5.206767	−2.044251
H	11.140939	−2.474881	−0.628226
H	7.769805	−6.249728	−4.646302
H	8.454014	−8.438511	−4.358879
H	9.539514	−8.852457	−3.088859

(TTT)$^{2-}$

E: −13655.08 kcal mol^{-1}

O	−0.302897	−0.204626	−0.001813
C	−0.161598	0.111176	1.406178
C	1.288645	−0.055719	1.840161
O	1.661974	−1.474981	1.882566
C	2.715067	−1.752458	0.933395
C	2.329613	0.632668	0.932211
C	2.835583	−0.508261	0.047423
O	3.347314	1.206428	1.803239
H	−1.254271	−0.239989	−0.203330
H	−0.791222	−0.551734	2.012716
H	−0.460847	1.151868	1.596058
H	1.377692	0.334009	2.859744
H	3.641727	−1.974408	1.465622
H	1.890446	1.439495	0.340279
H	2.183778	−0.583678	−0.824446
H	3.851425	−0.341741	−0.297650
N	2.391179	−2.984915	0.186559
C	3.175248	−4.123657	0.394685
O	4.114513	−4.182667	1.191663
N	2.820281	−5.197403	−0.406202
C	1.767485	−5.279267	−1.328456
O	1.585589	−6.330236	−1.965395
C	0.959433	−4.074662	−1.432014
C	−0.221264	−4.067636	−2.364665
C	1.300992	−3.005519	−0.668530
H	3.378184	−6.038862	−0.275369
H	−0.934363	−4.857552	−2.098565
H	0.097664	−4.256223	−3.397153
H	−0.737124	−3.103933	−2.329940
H	0.713436	−2.092463	−0.668355
P	4.834104	1.680026	1.254604
O	4.821847	1.928951	−0.233557
O	5.288335	2.744666	2.227465
O	5.728122	0.309894	1.467282
C	5.921062	−0.169195	2.824512
C	7.095887	−1.129939	2.877744
O	6.766828	−2.381164	2.198522
C	7.652089	−2.605622	1.083325
C	8.391415	−0.604903	2.226331
C	8.414847	−1.293553	0.855883
O	9.490821	−0.988845	3.099586
H	5.011243	−0.678455	3.164246
H	6.122744	0.678094	3.490111
H	7.286596	−1.365005	3.931014
H	8.328168	−3.431617	1.311997
H	8.389615	0.483104	2.127000
H	7.908576	−0.647580	0.138182
H	9.429570	−1.468240	0.506547
N	6.859201	−3.074250	−0.075306
C	6.853204	−4.447810	−0.345689
O	7.487388	−5.282686	0.300913
N	6.081968	−4.802355	−1.441997
C	5.302269	−3.969675	−2.257593
O	4.651028	−4.454718	−3.193809
C	5.328137	−2.565303	−1.880606
C	4.552373	−1.592104	−2.723639
C	6.072752	−2.199666	−0.804615
H	6.076261	−5.795919	−1.664797

H	4.889117	−1.648043	−3.765595
H	4.687278	−0.567384	−2.370386
H	3.482290	−1.830073	−2.721543
H	6.085944	−1.176022	−0.448033
P	11.070934	−0.772397	2.661378
O	11.193250	0.252665	1.560705
O	11.824670	−0.621745	3.964470
O	11.438867	−2.222837	1.968101
C	11.390200	−3.399058	2.818542
C	11.797009	−4.626688	2.026403
O	10.779964	−4.905619	1.016731
C	11.420738	−5.140117	−0.254388
C	13.151759	−4.523065	1.285440
C	12.755017	−4.383736	−0.191685
O	13.877709	−5.752717	1.550291
H	10.370905	−3.532396	3.200879
H	12.072268	−3.272860	3.667874
H	11.841608	−5.474909	2.722282
H	11.572929	−6.210274	−0.408764
H	13.736049	−3.665662	1.632464
H	12.623473	−3.325385	−0.430269
H	13.501430	−4.806085	−0.868788
H	14.746637	−5.680618	1.114380
N	10.499068	−4.712172	−1.316186
C	9.858185	−5.693038	−2.079796
O	10.034563	−6.906669	−1.937097
N	8.992006	−5.182560	−3.031717
C	8.694489	−3.837414	−3.299954
O	7.898971	−3.549101	−4.206485
C	9.368139	−2.884985	−2.431024
C	9.112281	−1.417634	−2.634343
C	10.217656	−3.369208	−1.489628
H	8.535712	−5.876114	−3.621829
H	9.402117	−1.115770	−3.648026
H	9.679079	−0.817088	−1.919119
H	8.046673	−1.186307	−2.526092
H	10.733225	−2.711183	−0.800917

d(AAA)$^{Na+}$

E: −27689.90 kcal mol^{-1}

O	−0.096421	−0.363074	−0.801150
C	0.012452	0.143750	0.550980
C	1.447756	0.019927	1.048575
O	1.821489	−1.380422	1.257224
C	2.822758	−1.791432	0.291497
C	2.521604	0.601773	0.107344
C	2.980912	−0.622088	−0.689467
O	3.541238	1.241657	0.949002
H	−1.041747	−0.394290	−1.029976
H	−0.642129	−0.421157	1.226932
H	−0.275330	1.204665	0.590067
H	1.502415	0.518700	2.022021
H	3.750333	−2.009395	0.824674
H	2.123019	1.383753	−0.542463
H	2.298401	−0.732636	−1.533033
H	3.992457	−0.533522	−1.079104
N	2.433986	−3.047342	−0.342504
C	1.549487	−3.250324	−1.394333
N	1.462211	−4.520519	−1.757779
C	2.327394	−5.196626	−0.897996
C	2.688111	−6.560526	−0.786737
N	2.194033	−7.529091	−1.580343
N	3.578193	−6.893896	0.190329
C	4.075236	−5.932034	0.994122
N	3.808190	−4.617245	0.969403
C	2.931294	−4.301742	−0.003279
H	0.987704	−2.432879	−1.823434
H	2.574194	−8.481937	−1.521683
H	1.638652	−7.268181	−2.384693
H	4.789622	−6.275407	1.739866
P	5.156718	1.126396	0.769980
O	5.597083	1.167468	−0.682773
O	5.761774	2.173803	1.684759
O	5.496746	−0.382371	1.287557
C	5.715423	−0.687261	2.690462
C	7.082745	−1.337063	2.863584
O	7.127507	−2.665658	2.263666
C	7.857077	−2.641609	1.013840
C	8.241735	−0.556133	2.215834
C	8.380558	−1.206131	0.831459
O	9.398040	−0.714465	3.095183
H	4.930619	−1.378568	3.013652
H	5.667163	0.227438	3.289570
H	7.263874	−1.463771	3.936099
H	8.662926	−3.376531	1.095184
H	8.027992	0.513110	2.142045
H	7.763456	−0.643786	0.131907
H	9.403366	−1.185485	0.462043
N	7.014418	−3.109935	−0.091687
C	6.231044	−2.381652	−0.982588
N	5.620490	−3.144582	−1.875454
C	6.014250	−4.445522	−1.565213
C	5.714430	−5.699609	−2.148137
N	4.889950	−5.861493	−3.195408
N	6.312782	−6.797718	−1.604847
C	7.120363	−6.660061	−0.537965
N	7.447185	−5.526647	0.104525
C	6.876138	−4.445167	−0.458655
H	6.147715	−1.306706	−0.924573
H	4.694131	−6.801799	−3.559861
H	4.445747	−5.054898	−3.611589
H	7.552457	−7.582540	−0.160467
P	10.954767	−0.598830	2.604082
O	11.130296	0.331990	1.421841
O	11.759851	−0.278246	3.850285
O	11.280268	−2.109140	2.066776
C	11.184747	−3.207041	3.019668
C	11.725375	−4.478649	2.390470
O	10.830864	−4.924381	1.323732
C	11.576934	−5.088962	0.093198
C	13.130657	−4.349674	1.765463
C	12.845453	−4.240979	0.262560
O	13.860072	−5.559666	2.101036
H	10.136267	−3.348682	3.305512
H	11.769870	−2.968915	3.915542
H	11.744769	−5.251664	3.169084
H	11.807903	−6.148359	−0.059203
H	13.664520	−3.473809	2.147487
H	12.644439	−3.197262	0.008371
H	13.672412	−4.603463	−0.352611
H	14.748399	−5.486747	1.706299
N	10.729766	−4.698223	−1.023316
C	10.312208	−3.414605	−1.353081
N	9.481828	−3.388568	−2.381338
C	9.337395	−4.724474	−2.754431
C	8.575218	−5.354837	−3.766046
N	7.783140	−4.690609	−4.629464
N	8.666287	−6.711198	−3.864002
C	9.440179	−7.392765	−2.999365
N	10.186822	−6.897944	−1.997654
C	10.101344	−5.555530	−1.919774
H	10.652049	−2.552986	−0.796528
H	7.148335	−5.221880	−5.238958
H	7.602220	−3.707868	−4.468080
H	9.442272	−8.472587	−3.128348
O	5.928709	−11.544814	−10.530852
C	7.186691	−12.237304	−10.725965
C	7.707922	−12.767060	−9.396109
O	8.188230	−11.675755	−8.544013
C	7.401330	−11.582399	−7.331675
C	6.676024	−13.542195	−8.553547
C	6.193975	−12.508535	−7.533756
O	7.381948	−14.675199	−7.956562
H	5.707320	−11.091235	−11.362797
H	7.935921	−11.560330	−11.154857
H	7.052310	−13.090062	−11.406846
H	8.564364	−13.415265	−9.610735
H	8.011208	−11.878203	−6.476068
H	5.855182	−13.934393	−9.157678
H	5.349250	−11.968520	−7.963772
H	5.867149	−12.966471	−6.603934
N	7.050071	−10.171627	−7.087826
C	7.671794	−9.492565	−6.026067
O	8.499353	−10.017112	−5.274901
N	7.268155	−8.181391	−5.872760
C	6.382542	−7.475323	−6.680099
O	6.135891	−6.273795	−6.436721
C	5.815989	−8.218674	−7.793416
C	4.869318	−7.523706	−8.733484
C	6.190635	−9.514228	−7.950563
H	7.749201	−7.649416	−5.099765
H	5.344940	−6.648120	−9.192372
H	3.982763	−7.162954	−8.197026
H	4.543603	−8.201304	−9.527946
H	5.851686	−10.108671	−8.793130
P	6.721614	−15.589118	−6.765954
O	5.210364	−15.498634	−6.742852
O	7.326954	−16.973608	−6.905739
O	7.258604	−14.853273	−5.404780
C	8.695860	−14.843722	−5.163961
C	8.981183	−14.328101	−3.765141
O	8.671362	−12.904031	−3.686825

C	7.763134	−12.651242	−2.589377
C	8.178612	−15.022716	−2.642477
C	7.099518	−13.999072	−2.284457
O	9.114735	−15.292686	−1.554434
H	9.184082	−14.198815	−5.903316
H	9.089769	−15.861831	−5.261309
H	10.053138	−14.459710	−3.575441
H	8.310929	−12.265103	−1.726759
H	7.748857	−15.972424	−2.967228
H	6.236676	−14.173237	−2.929595
H	6.777930	−14.080290	−1.249158
N	6.840054	−11.575309	−2.974774
C	7.008370	−10.304918	−2.389876
O	7.865974	−10.059694	−1.540512
N	6.112351	−9.349582	−2.826514
C	5.110869	−9.512490	−3.776118
O	4.387034	−8.550026	−4.101106
C	4.995378	−10.844593	−4.349759
C	3.895503	−11.102486	−5.340689
C	5.876624	−11.792301	−3.940366
H	6.201968	−8.397060	−2.371906
H	2.923379	−10.861379	−4.894340
H	3.881267	−12.148760	−5.654332
H	4.004543	−10.467807	−6.227974
H	5.872424	−12.792367	−4.358282
P	8.609822	−15.843378	−0.093012
O	7.224411	−16.450899	−0.161011
O	9.723095	−16.726132	0.441152
O	8.482401	−14.471911	0.788181
C	9.699151	−13.711848	1.043710
C	9.399303	−12.570500	1.997251
O	8.545134	−11.594033	1.332257
C	7.396983	−11.304585	2.161181
C	8.685363	−12.973809	3.307710
C	7.235893	−12.519472	3.087227
O	9.332975	−12.248088	4.386396
H	10.081450	−13.311694	0.097655
H	10.456297	−14.368850	1.486863
H	10.353668	−12.088306	2.246461
H	7.565866	−10.386135	2.726491
H	8.754441	−14.050651	3.490787
H	6.678164	−13.320708	2.596281
H	6.729578	−12.261884	4.020738
H	8.891393	−12.501600	5.217650
N	6.253605	−11.023731	1.284120
C	5.776491	−9.705560	1.197958
O	6.233758	−8.771692	1.865413
N	4.738207	−9.529806	0.305383
C	4.121377	−10.512101	−0.462833
O	3.175213	−10.215608	−1.223228
C	4.655645	−11.856296	−0.321356
C	4.005316	−12.976537	−1.084872
C	5.703769	−12.035375	0.522864
H	4.343096	−8.554483	0.253035
H	2.941998	−13.045061	−0.825673
H	4.476734	−13.934855	−0.856284
H	4.058133	−12.803321	−2.165396
H	6.176148	−13.002608	0.648893
Na	5.071890	−17.911698	−7.006189
Na	8.103044	−18.533490	0.727347
Na	12.716408	1.650037	2.735466
Na	7.053324	3.020513	−0.190475

d(AAC)$^{Na+}$

E: −27601.88 kcal mol^{-1}

O	0.031648	−0.256986	−0.677940
C	0.031144	0.180395	0.702421
C	1.427612	0.061031	1.301313
O	1.815823	−1.339707	1.490462
C	2.881542	−1.703855	0.577939
C	2.558192	0.690971	0.467606
C	3.094325	−0.492051	−0.341650
O	3.513528	1.255164	1.422690
H	−0.894670	−0.313224	−0.970590
H	−0.658175	−0.430701	1.298704
H	−0.282775	1.232197	0.772835
H	1.399344	0.523126	2.293662
H	3.773304	−1.947955	1.163484
H	2.205566	1.501919	−0.173894
H	2.488109	−0.581110	−1.243716
H	4.131308	−0.369229	−0.643059
N	2.536408	−2.930754	−0.136343
C	1.694713	−3.070330	−1.234149
N	1.602910	−4.320036	−1.658931
C	2.418277	−5.049691	−0.795411
C	2.741541	−6.425863	−0.731614
N	2.263847	−7.339798	−1.594030
N	3.579509	−6.827156	0.266904
C	4.060254	−5.913995	1.133939
N	3.824242	−4.591913	1.158949
C	2.996973	−4.209530	0.166387
H	1.161806	−2.224443	−1.644122
H	2.573302	−8.318180	−1.537849
H	1.721539	−7.032525	−2.390516
H	4.730756	−6.306882	1.895816
P	5.067598	1.613382	1.057117
O	5.279556	1.862140	−0.421423
O	5.482421	2.723061	2.005385
O	5.854864	0.222921	1.412322
C	5.930114	−0.201249	2.803610
C	7.120103	−1.127815	3.002567
O	6.885035	−2.438841	2.403983
C	7.749049	−2.646313	1.261765
C	8.434939	−0.611731	2.391784
C	8.502388	−1.323755	1.036082
O	9.506025	−0.992217	3.308870
H	5.003868	−0.720853	3.073918
H	6.050767	0.676901	3.447603
H	7.242096	−1.280439	4.080389
H	8.425666	−3.478602	1.475739
H	8.445262	0.475023	2.282518
H	7.994388	−0.703092	0.297594
H	9.525446	−1.489748	0.704594
N	6.958296	−3.075631	0.110548
C	6.198499	−2.290472	−0.750162
N	5.604118	−2.993226	−1.699920
C	5.984433	−4.312388	−1.459462
C	5.694026	−5.527379	−2.124710
N	4.911774	−5.622585	−3.211030
N	6.251583	−6.660111	−1.614434
C	7.028402	−6.597794	−0.519405
N	7.361955	−5.504619	0.185388
C	6.819194	−4.386427	−0.332662
H	6.120102	−1.222371	−0.612403
H	4.718318	−6.541913	−3.626187
H	4.482373	−4.791929	−3.594455
H	7.420455	−7.551956	−0.176678
P	11.084711	−0.788435	2.912313
O	11.273833	0.314214	1.890812
O	11.834483	−0.648594	4.224030
O	11.463351	−2.191629	2.164043
C	11.383290	−3.422831	2.937958
C	11.738754	−4.599812	2.052215
O	10.690957	−4.784434	1.056806
C	11.316720	−5.140910	−0.198774

C	13.081805	−4.474131	1.283257
C	12.655446	−4.388521	−0.190854
O	13.851754	−5.670296	1.574926
H	10.366660	−3.544106	3.328911
H	12.084517	−3.374087	3.779019
H	11.789001	−5.493912	2.689014
H	11.459250	−6.221423	−0.262039
H	13.643450	−3.589208	1.596104
H	12.508746	−3.340541	−0.469241
H	13.390794	−4.832664	−0.866284
H	14.727086	−5.564518	1.159367
N	10.401638	−4.791650	−1.286489
C	9.858184	−5.814186	−2.117211
O	10.172261	−7.007315	−1.907245
N	9.019983	−5.446936	−3.120222
C	8.674343	−4.156943	−3.307237
N	7.843130	−3.871009	−4.318655
C	9.177719	−3.118866	−2.454449
C	10.034723	−3.484830	−1.469683
H	7.471123	−4.620156	−4.922321
H	7.522340	−2.921164	−4.452663
H	8.884331	−2.084816	−2.588292
H	10.460454	−2.775209	−0.771827
O	6.312584	−11.886213	−11.016777
C	7.480699	−12.742156	−10.994144
C	7.810340	−13.151534	−9.562785
O	8.249802	−11.995643	−8.776894
C	7.288176	−11.709000	−7.728277
C	6.642258	−13.771773	−8.769835
C	6.058137	−12.568458	−8.032677
O	7.211272	−14.812936	−7.901382
H	6.229663	−11.524400	−11.916279
H	8.347984	−12.216928	−11.414205
H	7.304071	−13.654283	−11.582891
H	8.645492	−13.858978	−9.604348
H	7.730198	−11.964422	−6.764204
H	5.910362	−14.260815	−9.416295
H	5.385308	−12.048923	−8.717519
H	5.505032	−12.844684	−7.138316
N	7.025298	−10.280579	−7.651778
C	6.274498	−9.468432	−8.503110
N	6.224061	−8.214673	−8.089170
C	6.977759	−8.186895	−6.911282
C	7.292289	−7.121845	−6.014295
O	6.931436	−5.921889	−6.090926
N	8.106360	−7.550901	−4.946052
C	8.580714	−8.839132	−4.778768
N	9.391941	−9.077232	−3.726837
N	8.280686	−9.837505	−5.614048
C	7.492221	−9.460211	−6.634820
H	5.825625	−9.863535	−9.404150
H	8.394730	−6.815769	−4.259472
H	9.577926	−8.354823	−3.017876
H	9.567531	−10.052157	−3.507541
P	6.748031	−15.144618	−6.370754
O	5.254832	−15.002952	−6.153041
O	7.359168	−16.490269	−6.027981
O	7.437021	−13.950492	−5.490354
C	8.861794	−13.968257	−5.189056
C	9.075785	−13.837421	−3.686161
O	8.788516	−12.484648	−3.220092
C	7.582194	−12.461316	−2.418525
C	8.184034	−14.773682	−2.848088
C	7.000827	−13.881657	−2.446952
O	8.993291	−15.239647	−1.727229
H	9.331719	−13.125044	−5.704743
H	9.306897	−14.905586	−5.536485
H	10.132441	−14.034767	−3.476351
H	7.850992	−12.152832	−1.407357
H	7.859900	−15.648012	−3.417205
H	6.219004	−13.993261	−3.197966
H	6.581962	−14.167448	−1.484753
N	6.683274	−11.396283	−2.908440
C	6.761564	−10.150631	−2.254985
O	7.480196	−9.947554	−1.273948
N	5.963605	−9.166850	−2.795210
C	5.101099	−9.284634	−3.876664
O	4.454398	−8.291391	−4.272542
C	5.038172	−10.599598	−4.488564
C	4.082984	−10.816001	−5.628632
C	5.854668	−11.570400	−4.001983
H	6.047241	−8.211892	−2.336065
H	3.070720	−10.511401	−5.337357
H	4.053492	−11.867515	−5.921984
H	4.358098	−10.212428	−6.501351
H	5.905021	−12.546590	−4.467102
P	8.355692	−15.962274	−0.401101
O	6.969106	−16.516458	−0.654621
O	9.403870	−16.941729	0.094594
O	8.187496	−14.709244	0.638662
C	9.395261	−13.985717	1.016585
C	9.052363	−12.887865	2.005226
O	8.247797	−11.871049	1.338511
C	7.093997	−11.557698	2.153545
C	8.263582	−13.339707	3.257008
C	6.845013	−12.809229	3.007379
O	8.894936	−12.714569	4.405985
H	9.847966	−13.545970	0.120764
H	10.110593	−14.678304	1.475302
H	9.996904	−12.432067	2.330934
H	7.297811	−10.681237	2.771785
H	8.278817	−14.427889	3.372757
H	6.272720	−13.557396	2.452994
H	6.314109	−12.578313	3.934136
H	8.419973	−13.015829	5.202229
N	5.992754	−11.171568	1.264926
C	5.596369	−9.824322	1.221743
O	6.095925	−8.944471	1.929997
N	4.578089	−9.556096	0.328057
C	3.946804	−10.464205	−0.515558
O	3.038834	−10.084056	−1.285930
C	4.424556	−11.834475	−0.444617
C	3.784802	−12.872473	−1.324221
C	5.428056	−12.110683	0.426294
H	4.244954	−8.558739	0.306088
H	2.710536	−12.944029	−1.116119
H	4.233188	−13.855427	−1.161452
H	3.886576	−12.607184	−2.382656
H	5.855101	−13.103574	0.504867
Na	5.195570	−17.275151	−5.297635
Na	7.716616	−18.722417	−0.044556
Na	12.738280	1.490182	3.429754
Na	6.205384	4.070267	0.141400

d(AAG)$^{Na+}$

E: −27600.43 kcal mol^{-1}

O	−0.010057	−0.379819	−0.744041
C	0.004440	0.074495	0.630574
C	1.415910	0.005726	1.201173
O	1.853136	−1.379281	1.395509
C	2.908338	−1.720059	0.462081
C	2.509203	0.665017	0.340038
C	3.072669	−0.507732	−0.466559
O	3.455407	1.282573	1.271213
H	−0.939981	−0.470857	−1.015601

H	−0.652937	−0.550139	1.248518
H	−0.340433	1.117021	0.695547
H	1.392640	0.476441	2.189620
H	3.817032	−1.939577	1.030870
H	2.115759	1.453736	−0.305504
H	2.457462	−0.619754	−1.359937
H	4.102004	−0.356727	−0.782588
N	2.581705	−2.959294	−0.240024
C	1.745427	−3.129705	−1.337474
N	1.678675	−4.386700	−1.745869
C	2.505408	−5.089276	−0.870568
C	2.854721	−6.457816	−0.785048
N	2.393699	−7.395629	−1.631483
N	3.698941	−6.827006	0.220469
C	4.159149	−5.892476	1.075997
N	3.895249	−4.575963	1.081891
C	3.065187	−4.224827	0.080807
H	1.197844	−2.300586	−1.761714
H	2.732057	−8.363732	−1.565134
H	1.857387	−7.109798	−2.439975
H	4.836317	−6.260960	1.844065
P	5.013930	1.624906	0.912851
O	5.241506	1.858630	−0.566138
O	5.426883	2.742031	1.852944
O	5.787177	0.232151	1.287001
C	5.821754	−0.192472	2.679691
C	7.009147	−1.113400	2.912674
O	6.804629	−2.418697	2.291939
C	7.723887	−2.613944	1.189160
C	8.342169	−0.582763	2.356420
C	8.461664	−1.279013	0.996468
O	9.376759	−0.969068	3.312621
H	4.889777	−0.715815	2.921483
H	5.919102	0.685077	3.328589
H	7.093447	−1.275100	3.992966
H	8.405419	−3.433332	1.436347
H	8.351821	0.505402	2.260632
H	7.962137	−0.659456	0.250831
H	9.496144	−1.423580	0.690751
N	6.991351	−3.062799	0.008057
C	6.230989	−2.304399	−0.874544
N	5.662951	−3.031515	−1.822913
C	6.063471	−4.340305	−1.558088
C	5.794596	−5.573106	−2.199787
N	5.006855	−5.708238	−3.278667
N	6.376328	−6.686156	−1.669933
C	7.134170	−6.590539	−0.562771
N	7.429939	−5.481415	0.131630
C	6.880906	−4.382059	−0.417288
H	6.132142	−1.235563	−0.755319
H	4.820835	−6.641387	−3.666633
H	4.567683	−4.893113	−3.683543
H	7.541472	−7.530327	−0.199090
P	10.971837	−0.801904	2.974577
O	11.223365	0.276715	1.940693
O	11.673989	−0.650425	4.311334
O	11.350515	−2.227196	2.269479
C	11.235386	−3.438557	3.069696
C	11.671015	−4.635853	2.246608
O	10.684780	−4.894644	1.201305
C	11.379906	−5.130791	−0.046698
C	13.043734	−4.482541	1.546134
C	12.673007	−4.312124	0.067621
O	13.788081	−5.705337	1.793236
H	10.195974	−3.566481	3.393085
H	11.876529	−3.354528	3.954757
H	11.711361	−5.504401	2.917519
H	11.586824	−6.200090	−0.167429
H	13.601288	−3.624224	1.932389
H	12.468208	−3.257080	−0.136682
H	13.455905	−4.662607	−0.609456
H	14.667788	−5.600919	1.386612
N	10.514381	−4.761837	−1.151138
C	10.087268	−3.481072	−1.500973
N	9.319301	−3.474982	−2.572145
C	9.229128	−4.812753	−2.961116
C	8.552779	−5.423448	−4.058158
O	7.841754	−4.866979	−4.929791
N	8.757066	−6.815543	−4.095595
C	9.526969	−7.530015	−3.200418
N	9.682558	−8.849438	−3.442310
N	10.137873	−6.963743	−2.151407
C	9.963898	−5.628715	−2.086708
H	10.377115	−2.614184	−0.926056
H	8.264108	−7.337107	−4.858086
H	9.175676	−9.318842	−4.209442
H	10.022328	−9.416601	−2.675738
O	5.933564	−11.886050	−10.824711
C	7.170355	−12.625541	−10.980016
C	7.712794	−13.040997	−9.617776
O	8.191199	−11.875306	−8.868164
C	7.408822	−11.700058	−7.658031
C	6.699402	−13.745206	−8.693966
C	6.183166	−12.604397	−7.818087
O	7.441720	−14.773131	−7.957362
H	5.707606	−11.502066	−11.689945
H	7.924592	−12.008744	−11.484488
H	7.002376	−13.533599	−11.576850
H	8.571471	−13.699003	−9.789542
H	8.008537	−11.977249	−6.791864
H	5.899568	−14.241985	−9.247754
H	5.384327	−12.094837	−8.360047
H	5.789928	−12.947717	−6.865121
N	7.102096	−10.275346	−7.472410
C	7.701596	−9.567387	−6.391390
O	8.499500	−10.166231	−5.637431
N	7.371709	−8.261909	−6.214208
C	6.527985	−7.634593	−7.057450
N	6.237086	−6.348387	−6.811453
C	5.964597	−8.313812	−8.189662
C	6.287772	−9.620794	−8.362823
H	6.740330	−5.831731	−6.075355
H	5.652845	−5.835032	−7.458719
H	5.320076	−7.798838	−8.892514
H	5.943400	−10.208331	−9.207964
P	6.952900	−15.380146	−6.518006
O	5.446454	−15.366847	−6.361902
O	7.645202	−16.721447	−6.366036
O	7.535454	−14.299171	−5.436470
C	8.967738	−14.253357	−5.170321
C	9.211093	−13.982975	−3.692869
O	8.927962	−12.593126	−3.355802
C	7.799620	−12.499534	−2.456759
C	8.349239	−14.841209	−2.746459
C	7.193278	−13.907043	−2.368354
O	9.203960	−15.220367	−1.625763
H	9.416753	−13.456663	−5.772791
H	9.422139	−15.212849	−5.437633
H	10.273701	−14.159403	−3.492115
H	8.148696	−12.146583	−1.484755
H	7.991933	−15.755084	−3.226569
H	6.390128	−14.052664	−3.091161
H	6.799411	−14.121924	−1.377727
N	6.884541	−11.442997	−2.930655
C	6.963829	−10.187439	−2.296122
O	7.697571	−9.964266	−1.331035
N	6.143852	−9.218933	−2.833501
C	5.235356	−9.370483	−3.872337

O	4.544360	−8.401646	−4.249887
C	5.169834	−10.698189	−4.460624
C	4.150855	−10.949869	−5.536986
C	6.023419	−11.646496	−3.993286
H	6.213852	−8.260987	−2.382026
H	3.152066	−10.673243	−5.178636
H	4.131028	−12.002630	−5.825722
H	4.346964	−10.341561	−6.427185
H	6.073764	−12.633126	−4.438330
P	8.620087	−15.877223	−0.242391
O	7.233681	−16.460563	−0.420734
O	9.696614	−16.815561	0.272404
O	8.466534	−14.573647	0.734570
C	9.675145	−13.823111	1.051591
C	9.345661	−12.699709	2.016057
O	8.512643	−11.711772	1.341600
C	7.362628	−11.405819	2.163733
C	8.593138	−13.124365	3.298658
C	7.157458	−12.638034	3.056946
O	9.228645	−12.440090	4.411152
H	10.093705	−13.405438	0.128995
H	10.413833	−14.491610	1.508892
H	10.292562	−12.223611	2.303369
H	7.551968	−10.507211	2.754048
H	8.638831	−14.206731	3.454271
H	6.596680	−13.416460	2.533410
H	6.634725	−12.393440	3.984893
H	8.768959	−12.713845	5.225996
N	6.239455	−11.071734	1.281218
C	5.809225	−9.736575	1.210032
O	6.291078	−8.828626	1.895288
N	4.780857	−9.513165	0.316135
C	4.160898	−10.456955	−0.495750
O	3.239674	−10.116061	−1.268875
C	4.664111	−11.815648	−0.387940
C	4.027677	−12.891797	−1.222939
C	5.684714	−12.046059	0.476876
H	4.419381	−8.525659	0.276555
H	2.955024	−12.960854	−1.006442
H	4.482566	−13.864763	−1.023181
H	4.122556	−12.669321	−2.291851
H	6.133412	−13.027169	0.579408
Na	5.505707	−17.752165	−5.890578
Na	8.040162	−18.621659	0.301552
Na	12.665487	1.442646	3.501963
Na	6.171701	4.056222	−0.041542

d(AAT)$^{Na+}$

E: −27694.88 kcal mol^{-1}

O	0.260430	0.095790	−0.500476
C	0.380451	0.536787	0.873983
C	1.758524	0.184108	1.422514
O	1.910876	−1.265305	1.565222
C	2.923001	−1.762561	0.653003
C	2.946090	0.651874	0.554378
C	3.270062	−0.591037	−0.276435
O	4.004915	1.123931	1.458560
H	−0.665871	0.220851	−0.771018
H	−0.380189	0.052833	1.499888
H	0.250257	1.626748	0.943053
H	1.839185	0.623201	2.422622
H	3.786119	−2.093631	1.231355
H	2.683836	1.504536	−0.075154
H	2.606966	−0.584978	−1.143278
H	4.297720	−0.618487	−0.630985
N	2.444026	−2.952048	−0.041547
C	1.544157	−3.035580	−1.096053
N	1.406060	−4.268594	−1.559598
C	2.259803	−5.042584	−0.772203
C	2.584919	−6.420588	−0.784908
N	2.048059	−7.308759	−1.641709
N	3.492913	−6.856283	0.134030
C	4.027743	−5.980927	1.009541
N	3.787902	−4.664921	1.110039
C	2.903855	−4.244998	0.184663
H	1.013140	−2.162291	−1.450054
H	2.389602	−8.277804	−1.656732
H	1.451713	−6.978318	−2.388982
H	4.749479	−6.405901	1.704747
P	5.584530	0.723909	1.427304
O	6.171268	0.711665	0.030147
O	6.262812	1.609656	2.457810
O	5.584683	−0.842355	1.910273
C	5.854487	−1.288997	3.263590
C	7.239722	−1.936095	3.329415
O	7.273546	−3.212707	2.627646
C	7.970311	−3.094031	1.365406
C	8.308667	−1.055083	2.670856
C	8.495224	−1.651511	1.267065
O	9.527773	−1.066848	3.475271
H	5.084902	−2.021552	3.523013
H	5.810785	−0.445927	3.960495
H	7.484989	−2.133936	4.378396
H	8.779435	−3.828122	1.367049
H	7.963052	−0.024749	2.639897
H	7.924175	−1.064636	0.547978
H	9.540973	−1.645789	0.966238
N	7.102910	−3.470753	0.246803
C	6.243527	−2.672625	−0.505538
N	5.607806	−3.347838	−1.446814
C	6.058431	−4.659657	−1.316509
C	5.769934	−5.844989	−2.031762
N	4.911999	−5.911069	−3.061678
N	6.414731	−6.979498	−1.638468
C	7.280932	−6.941011	−0.611393
N	7.625008	−5.875064	0.131269
C	6.984050	−4.757895	−0.265551
H	6.152906	−1.612237	−0.323377
H	4.718035	−6.811170	−3.515286
H	4.424297	−5.077939	−3.360512
H	7.748674	−7.891212	−0.365825
P	10.729368	−0.053105	3.008921
O	10.167402	1.140760	2.248632
O	11.560870	0.252235	4.235375
O	11.566013	−0.936849	1.920685
C	12.215389	−2.158040	2.390395
C	12.527839	−3.080596	1.231100
O	11.308923	−3.646667	0.680572
C	11.646419	−4.223141	−0.607229
C	13.234800	−2.463411	0.013976
C	12.910918	−3.474037	−1.108818
O	14.637496	−2.324310	0.308818
H	11.563497	−2.674723	3.102709
H	13.151244	−1.891464	2.892545
H	13.161891	−3.889120	1.630907
H	11.819437	−5.294505	−0.502401
H	12.801506	−1.481311	−0.202345
H	12.739300	−2.994410	−2.074577
H	13.741524	−4.177515	−1.213573
H	15.026899	−1.722799	−0.350601
N	10.476188	−4.091928	−1.486889
C	9.996902	−5.228979	−2.157105
O	10.457491	−6.362662	−1.988026
N	8.966750	−4.975516	−3.040769
C	8.420242	−3.733185	−3.356696

O	7.536065	−3.641335	−4.232907
C	8.950540	−2.599037	−2.614319
C	8.397835	−1.224596	−2.872121
C	9.956909	−2.836052	−1.736963
H	8.627457	−5.810196	−3.586259
H	8.594461	−0.912366	−3.905101
H	8.848265	−0.492103	−2.196317
H	7.312077	−1.211284	−2.731123
H	10.413494	−2.038060	−1.162799
O	6.540758	−11.543677	−10.573634
C	7.806935	−12.241495	−10.658661
C	8.164141	−12.857492	−9.311706
O	8.594219	−11.845224	−8.342319
C	7.624435	−11.691681	−7.280829
C	7.021573	−13.633864	−8.632407
C	6.453431	−12.633267	−7.618726
O	7.618215	−14.826011	−8.035351
H	6.416764	−11.051064	−11.403732
H	8.607866	−11.553579	−10.957232
H	7.746541	−13.053177	−11.398215
H	9.017900	−13.523993	−9.471051
H	8.102774	−11.938474	−6.328115
H	6.260053	−13.959262	−9.344449
H	5.640998	−12.084473	−8.095825
H	6.055662	−13.123261	−6.733117
N	7.224628	−10.285662	−7.167049
C	6.399447	−9.578702	−8.036106
N	6.251228	−8.310513	−7.690594
C	7.024145	−8.162768	−6.540982
C	7.283745	−7.042824	−5.715456
N	6.757389	−5.826826	−5.933904
N	8.126246	−7.224977	−4.658274
C	8.667195	−8.440585	−4.447511
N	8.485806	−9.564857	−5.162622
C	7.650597	−9.371530	−6.203888
H	5.980808	−10.050554	−8.913989
H	6.965345	−5.048446	−5.296844
H	6.102903	−5.695501	−6.693688
H	9.324609	−8.510887	−3.583064
P	6.809312	−15.774624	−6.971631
O	5.307036	−15.650208	−7.113485
O	7.400884	−17.165613	−7.105907
O	7.204351	−15.107534	−5.530796
C	8.610931	−15.107403	−5.152771
C	8.751405	−14.558249	−3.747954
O	8.442012	−13.132206	−3.750462
C	7.540344	−12.828570	−2.660941
C	7.823258	−15.224543	−2.702584
C	6.795199	−14.137695	−2.372632
O	8.661793	−15.608405	−1.572469
H	9.179066	−14.488501	−5.856235
H	8.999830	−16.131758	−5.182889
H	9.793585	−14.692348	−3.433417
H	8.095432	−12.477952	−1.787621
H	7.345612	−16.124541	−3.093583
H	5.937645	−14.257919	−3.037800
H	6.446555	−14.198244	−1.343830
N	6.691582	−11.702515	−3.065073
C	6.881334	−10.456621	−2.440002
O	7.712354	−10.265566	−1.549599
N	6.042939	−9.458929	−2.888804
C	5.101539	−9.545724	−3.905943
O	4.440247	−8.540177	−4.235231
C	4.970721	−10.853819	−4.532402
C	3.943422	−11.038894	−5.613255
C	5.779062	−11.851279	−4.092830
H	6.176268	−8.513342	−2.423070
H	2.943929	−10.792427	−5.235086
H	3.931843	−12.070355	−5.973746
H	4.136948	−10.370207	−6.459858
H	5.753742	−12.840903	−4.533799
P	8.018328	−16.251528	−0.205769
O	6.623541	−16.794677	−0.434912
O	9.052654	−17.218679	0.340867
O	7.866894	−14.946277	0.768797
C	9.085254	−14.243391	1.149909
C	8.742442	−13.068866	2.045265
O	8.015504	−12.071460	1.269703
C	6.881076	−11.610226	2.035736
C	7.875950	−13.401694	3.285060
C	6.510675	−12.782652	2.955972
O	8.510845	−12.769580	4.428406
H	9.593384	−13.882417	0.248490
H	9.752232	−14.928560	1.685889
H	9.688305	−12.629535	2.389995
H	7.144005	−10.716192	2.605137
H	7.805704	−14.481022	3.450707
H	5.898232	−13.516504	2.425084
H	5.971080	−12.456343	3.848432
H	7.991735	−13.004076	5.219510
N	5.832091	−11.193879	1.099598
C	5.475257	−9.837198	1.034755
O	5.983877	−8.964790	1.746212
N	4.489936	−9.550430	0.111794
C	3.827269	−10.453103	−0.713837
O	2.933742	−10.055617	−1.491507
C	4.251327	−11.839322	−0.606319
C	3.565041	−12.878105	−1.449529
C	5.234306	−12.131830	0.282582
H	4.166024	−8.547603	0.091469
H	2.491038	−12.902046	−1.228997
H	3.977066	−13.872116	−1.260220
H	3.666201	−12.649561	−2.516062
H	5.613623	−13.139750	0.402291
Na	5.153865	−18.046950	−7.493768
Na	7.353477	−18.980891	0.320577
Na	11.275497	2.616111	3.925428
Na	8.291921	2.066209	1.306730

d(ACA)$^{Na+}$

E: −27599.43 kcal mol^{-1}

O	−0.042304	−0.371118	−0.375630
C	−0.104883	−0.034043	1.031406
C	1.290686	−0.046798	1.643558
O	1.807719	−1.408732	1.800803
C	2.904640	−1.658962	0.889296
C	2.357922	0.714735	0.838453
C	3.064132	−0.385077	0.037661
O	3.220263	1.383767	1.810898
H	−0.954630	−0.488982	−0.693022
H	−0.733950	−0.752097	1.572458
H	−0.526396	0.972880	1.165077
H	1.211808	0.380194	2.648730
H	3.802707	−1.887238	1.470395
H	1.925224	1.476861	0.186969
H	2.562784	−0.492638	−0.924816
H	4.109999	−0.150133	−0.145225
N	2.633430	−2.862782	0.099667
C	1.758916	−2.986984	−0.975028
N	1.720391	−4.212954	−1.470807
C	2.608288	−4.943075	−0.683277
C	2.995856	−6.304058	−0.707417
N	2.517932	−7.192625	−1.594094
N	3.889212	−6.719378	0.236054
C	4.361114	−5.832131	1.132977

N	4.061733	−4.526792	1.243584
C	3.178438	−4.129327	0.306239
H	1.161519	−2.150060	−1.307677
H	2.849166	−8.164851	−1.582569
H	1.916670	−6.874545	−2.342336
H	5.077438	−6.233259	1.847166
P	4.599424	2.150087	1.365930
O	4.560058	2.607497	−0.077294
O	4.850144	3.213442	2.419205
O	5.722450	0.962711	1.452535
C	5.942172	0.336133	2.748296
C	6.964329	−0.774935	2.616064
O	6.403750	−1.873566	1.834829
C	7.386278	−2.293936	0.850325
C	8.297542	−0.373333	1.931224
C	8.194759	−1.031064	0.552292
O	9.367793	−0.888102	2.788586
H	4.997466	−0.074593	3.121822
H	6.310645	1.084461	3.459746
H	7.188080	−1.138388	3.626604
H	8.016336	−3.085831	1.257102
H	8.422713	0.709233	1.863531
H	7.634094	−0.352513	−0.096393
H	9.160248	−1.229851	0.090481
N	6.691918	−2.894382	−0.285764
C	6.795078	−4.302318	−0.497024
O	7.485911	−4.983563	0.286322
N	6.134673	−4.841506	−1.553588
C	5.367046	−4.085752	−2.361967
N	4.733671	−4.691887	−3.375078
C	5.232179	−2.672323	−2.150705
C	5.912654	−2.130316	−1.109540
H	4.842314	−5.707591	−3.534410
H	4.132448	−4.153942	−3.984820
H	4.611062	−2.060454	−2.793828
H	5.865916	−1.075486	−0.872416
P	10.862950	−1.305264	2.279179
O	11.311525	−0.505859	1.072299
O	11.757350	−1.253374	3.503374
O	10.668555	−2.842513	1.762123
C	10.551943	−3.941266	2.711397
C	11.472673	−5.089496	2.309294
O	10.952364	−5.828118	1.163344
C	11.718121	−5.531607	−0.027023
C	12.892544	−4.654478	1.911362
C	12.808365	−4.519955	0.381897
O	13.797194	−5.712726	2.323487
H	9.512819	−4.285897	2.712895
H	10.827697	−3.595446	3.712675
H	11.510420	−5.793095	3.148916
H	12.136208	−6.469520	−0.401241
H	13.176370	−3.711657	2.390917
H	12.516122	−3.501710	0.121140
H	13.762043	−4.740948	−0.103381
H	14.693392	−5.463218	2.032878
N	10.826528	−5.056778	−1.086766
C	10.234789	−3.805521	−1.217288
N	9.386734	−3.736215	−2.229700
C	9.400905	−5.011710	−2.791621
C	8.706835	−5.577949	−3.885832
N	7.837940	−4.897748	−4.654807
N	8.953715	−6.887437	−4.171445
C	9.836454	−7.580102	−3.426716
N	10.550997	−7.139695	−2.378783
C	10.288093	−5.847807	−2.096215
H	10.457017	−3.009529	−0.523319
H	7.312488	−5.384572	−5.392209
H	7.578029	−3.956577	−4.392785
H	9.966392	−8.621438	−3.715549
O	5.765585	−11.860686	−10.664322
C	6.931672	−12.703356	−10.838174
C	7.493601	−13.120315	−9.484355
O	8.096894	−11.977900	−8.792555
C	7.386611	−11.704787	−7.556464
C	6.468188	−13.713596	−8.495834
C	6.084013	−12.507569	−7.637000
O	7.160633	−14.784730	−7.770204
H	5.537851	−11.488288	−11.534029
H	7.712891	−12.166747	−11.391019
H	6.667983	−13.613225	−11.396541
H	8.288079	−13.851294	−9.669264
H	8.000762	−12.007989	−6.708635
H	5.606965	−14.158501	−8.999393
H	5.313540	−11.948728	−8.171666
H	5.690458	−12.778715	−6.660114
N	7.202305	−10.257913	−7.405079
C	7.899562	−9.595235	−6.380608
O	8.724516	−10.151477	−5.650085
N	7.581234	−8.258517	−6.252815
C	6.729365	−7.518409	−7.062557
O	6.549284	−6.299421	−6.836525
C	6.112851	−8.242768	−8.161691
C	5.223152	−7.502838	−9.123848
C	6.382061	−9.568789	−8.278203
H	8.077920	−7.750818	−5.473829
H	5.768144	−6.685292	−9.612134
H	4.370085	−7.052624	−8.601022
H	4.841533	−8.177234	−9.896031
H	5.982215	−10.167303	−9.091164
P	6.770114	−15.282440	−6.263418
O	5.294319	−15.121655	−5.958997
O	7.352251	−16.674348	−6.107376
O	7.547110	−14.215303	−5.299509
C	8.981085	−14.314560	−5.073764
C	9.291050	−14.142909	−3.590355
O	9.166530	−12.755024	−3.158134
C	7.973636	−12.564217	−2.358930
C	8.368389	−14.953130	−2.663548
C	7.287526	−13.941009	−2.264192
O	9.189731	−15.423899	−1.552050
H	9.482043	−13.530316	−5.650625
H	9.338983	−15.295941	−5.401452
H	10.335855	−14.431374	−3.433927
H	8.275957	−12.180682	−1.380156
H	7.941837	−15.826236	−3.163080
H	6.465250	−14.018799	−2.975073
H	6.895262	−14.130972	−1.267973
N	7.126026	−11.523388	−2.939086
C	6.259926	−11.627947	−4.029638
N	5.654434	−10.491503	−4.310434
C	6.139900	−9.578240	−3.372675
C	5.836538	−8.199873	−3.168682
O	5.057163	−7.478977	−3.835686
N	6.528921	−7.665261	−2.066629
C	7.440166	−8.355145	−1.289833
N	8.031889	−7.673219	−0.296013
N	7.739567	−9.646699	−1.491484
C	7.064417	−10.198457	−2.518152
H	6.150733	−12.557796	−4.564637
H	6.362287	−6.652360	−1.858163
H	7.822522	−6.682525	−0.098061
H	8.702450	−8.166036	0.277167
P	8.536335	−16.064800	−0.192312
O	7.153531	−16.634327	−0.431948
O	9.579795	−17.011247	0.372500
O	8.348240	−14.755833	0.771707
C	9.542925	−14.006041	1.136770
C	9.173292	−12.885636	2.089177

O	8.381639	−11.886691	1.379472
C	7.234099	−11.531580	2.185440
C	8.352952	−13.315171	3.330090
C	6.946230	−12.770829	3.043651
O	8.967201	−12.683766	4.484801
H	10.000235	−13.589006	0.232427
H	10.262130	−14.674429	1.624357
H	10.106896	−12.418325	2.429833
H	7.457686	−10.657842	2.801055
H	8.353335	−14.401876	3.457759
H	6.377922	−13.513960	2.477473
H	6.396470	−12.526660	3.955996
H	8.478748	−12.978159	5.275366
N	6.156051	−11.118753	1.281658
C	5.730904	−9.780409	1.297734
O	6.148411	−8.943938	2.104516
N	4.792460	−9.468548	0.333646
C	4.202927	−10.345344	−0.572356
O	3.339085	−9.932565	−1.375434
C	4.657210	−11.723885	−0.509543
C	4.033613	−12.734920	−1.431203
C	5.620486	−12.031464	0.394950
H	4.475085	−8.464574	0.314138
H	2.952667	−12.797652	−1.256573
H	4.464927	−13.726364	−1.275419
H	4.172344	−12.447788	−2.479168
H	6.033580	−13.030177	0.472181
Na	5.169003	−17.459810	−5.368330
Na	7.911380	−18.798856	0.355185
Na	13.278810	0.209061	2.301470
Na	4.931581	4.898265	0.645946

d(ACC)$^{Na+}$

E: −27513.29 kcal mol^{-1}

O	−0.120591	−0.239971	−0.353645
C	−0.066806	0.189962	1.027921
C	1.368539	0.154172	1.538273
O	1.845840	−1.219564	1.720587
C	2.862821	−1.549821	0.745091
C	2.400965	0.836482	0.623193
C	2.991471	−0.325197	−0.180339
O	3.361330	1.507939	1.497087
H	−1.057239	−0.345224	−0.595433
H	−0.681072	−0.464312	1.659198
H	−0.438041	1.220795	1.124122
H	1.378868	0.628563	2.525030
H	3.792979	−1.776315	1.275647
H	1.949367	1.590498	−0.025245
H	2.394456	−0.454842	−1.083378
H	4.018491	−0.137083	−0.481470
N	2.505709	−2.781027	0.035336
C	1.611440	−2.919866	−1.022162
N	1.530385	−4.161133	−1.471231
C	2.408371	−4.887708	−0.669392
C	2.770398	−6.256131	−0.661522
N	2.269809	−7.155871	−1.524994
N	3.661871	−6.665672	0.286715
C	4.158847	−5.763043	1.154824
N	3.893750	−4.447156	1.226461
C	3.012913	−4.056554	0.284125
H	1.032991	−2.080569	−1.380117
H	2.608164	−8.126170	−1.515309
H	1.681831	−6.838651	−2.284465
H	4.871222	−6.157799	1.876124
P	4.789781	2.105850	0.961993
O	4.793518	2.346922	−0.533035
O	5.115988	3.292402	1.849701
O	5.824831	0.867304	1.243327
C	5.991031	0.432566	2.623970
C	6.998413	−0.699820	2.695260
O	6.461233	−1.884023	2.029775
C	7.397799	−2.347213	1.025211
C	8.369861	−0.412005	2.042076
C	8.281216	−1.139006	0.699528
O	9.378998	−0.965390	2.943423
H	5.026861	0.087925	3.014137
H	6.343535	1.272322	3.234108
H	7.151125	−0.936597	3.754905
H	7.979531	−3.183234	1.416798
H	8.562349	0.656733	1.925678
H	7.794177	−0.472609	−0.016024
H	9.257049	−1.414800	0.303070
N	6.650018	−2.893811	−0.109376
C	6.645035	−4.303073	−0.325590
O	7.249020	−5.040962	0.477628
N	5.994967	−4.781068	−1.416799
C	5.311798	−3.964357	−2.241340
N	4.695642	−4.509695	−3.297266
C	5.247233	−2.550382	−1.998961
C	5.932030	−2.068222	−0.931387
H	4.749730	−5.526148	−3.475496
H	4.163716	−3.924373	−3.927678
H	4.676204	−1.892057	−2.642528
H	5.938567	−1.018327	−0.666001
P	10.947994	−1.177705	2.517863
O	11.337932	−0.338816	1.319092
O	11.762789	−0.988397	3.783607
O	10.986526	−2.741310	2.032661
C	10.744436	−3.784898	3.022880
C	11.287884	−5.117282	2.530879
O	10.474464	−5.631881	1.433806
C	11.277204	−5.809809	0.245612
C	12.741841	−5.084602	2.019587
C	12.583734	−5.036038	0.494488
O	13.377526	−6.311248	2.468667
H	9.666028	−3.868200	3.198869
H	11.243025	−3.519107	3.961566
H	11.211562	−5.825212	3.365661
H	11.449590	−6.871782	0.068654
H	13.288507	−4.217677	2.404914
H	12.482599	−3.996168	0.172395
H	13.432078	−5.483015	−0.029892
H	14.289069	−6.313496	2.123115
N	10.501889	−5.335113	−0.913459
C	10.170327	−6.226390	−1.969943
O	10.629048	−7.391584	−1.961968
N	9.364648	−5.768873	−2.965164
C	8.941673	−4.488570	−2.988102
N	8.153966	−4.102492	−4.002462
C	9.315909	−3.562538	−1.960344
C	10.071017	−4.036131	−0.937913
H	7.841947	−4.782027	−4.714645
H	7.793823	−3.157850	−4.026125
H	8.981363	−2.532855	−1.984309
H	10.358386	−3.431664	−0.084926
O	5.637585	−11.823636	−10.912385
C	6.763189	−12.717159	−11.089355
C	7.337354	−13.129046	−9.738418
O	7.953402	−11.984423	−9.061547
C	7.229999	−11.683759	−7.838793
C	6.323489	−13.705033	−8.728662
C	5.919989	−12.470957	−7.922004
O	7.044328	−14.727863	−7.956681
H	5.410594	−11.460173	−11.786084
H	7.555869	−12.223939	−11.666350

H	6.455004	−13.627078	−11.625042
H	8.125797	−13.865629	−9.927192
H	7.829141	−11.992833	−6.980618
H	5.474095	−14.193663	−9.211272
H	5.178938	−11.916863	−8.501963
H	5.498705	−12.704171	−6.945640
N	7.069075	−10.249060	−7.691242
C	6.279657	−9.370628	−8.432393
N	6.343687	−8.130875	−7.979903
C	7.211545	−8.187080	−6.884597
C	7.651796	−7.180170	−5.977524
O	7.351198	−5.960141	−5.982388
N	8.516093	−7.695906	−4.989466
C	8.924819	−9.014348	−4.904463
N	9.791504	−9.345123	−3.917694
N	8.523298	−9.954079	−5.762801
C	7.675981	−9.495278	−6.699227
H	5.716060	−9.711613	−9.290681
H	8.844977	−7.015117	−4.265244
H	10.012725	−8.669495	−3.174063
H	9.845681	−10.338181	−3.705013
P	6.759464	−15.109271	−6.395225
O	5.293466	−15.000134	−6.024513
O	7.424231	−16.451398	−6.154196
O	7.517418	−13.925808	−5.557846
C	8.949379	−13.958848	−5.298849
C	9.226699	−13.860883	−3.800305
O	9.022574	−12.509334	−3.283104
C	7.789070	−12.432967	−2.523104
C	8.329967	−14.767997	−2.939691
C	7.173782	−13.842477	−2.540436
O	9.141902	−15.231081	−1.818387
H	9.406973	−13.106759	−5.811032
H	9.374923	−14.892177	−5.680021
H	10.282468	−14.102836	−3.640515
H	8.039240	−12.096662	−1.513059
H	7.981862	−15.645078	−3.490453
H	6.393560	−13.921568	−3.297531
H	6.742221	−14.112343	−1.579308
N	6.913975	−11.399295	−3.069303
C	6.063414	−11.460212	−4.177818
N	5.450999	−10.316451	−4.409608
C	5.920453	−9.442477	−3.425744
C	5.635518	−8.067016	−3.175237
O	4.865883	−7.311264	−3.815013
N	6.347784	−7.574212	−2.065518
C	7.230136	−8.308132	−1.295057
N	7.830005	−7.671986	−0.275446
N	7.501075	−9.597610	−1.531849
C	6.834055	−10.098591	−2.586883
H	5.968687	−12.363137	−4.760526
H	6.193658	−6.567809	−1.818570
H	7.582944	−6.709237	0.001700
H	8.433755	−8.221268	0.320700
P	8.490356	−15.899634	−0.470748
O	7.117633	−16.486799	−0.725787
O	9.544844	−16.837382	0.087624
O	8.278261	−14.605284	0.509131
C	9.463920	−13.855369	0.903856
C	9.089536	−12.793795	1.920801
O	8.277123	−11.766798	1.277608
C	7.119402	−11.488417	2.098709
C	8.290899	−13.300444	3.145877
C	6.869933	−12.775274	2.897676
O	8.901212	−12.710533	4.324423
H	9.904100	−13.382397	0.018559
H	10.199280	−14.536855	1.346989
H	10.021518	−12.333283	2.274756
H	7.314200	−10.637155	2.754069
H	8.316955	−14.391802	3.221935
H	6.313091	−13.503318	2.301594
H	6.323339	−12.588070	3.825252
H	8.420902	−13.046367	5.103416
N	6.026141	−11.070675	1.214231
C	5.562563	−9.747469	1.284309
O	5.965518	−8.928085	2.115836
N	4.603889	−9.427995	0.341759
C	4.031820	−10.289082	−0.591039
O	3.146081	−9.874868	−1.368893
C	4.533910	−11.652289	−0.589541
C	3.938627	−12.644649	−1.549452
C	5.517492	−11.962946	0.291586
H	4.264910	−8.432868	0.355233
H	2.860891	−12.747484	−1.374399
H	4.400746	−13.627680	−1.433633
H	4.064208	−12.313668	−2.585983
H	5.970046	−12.947333	0.319196
Na	5.337946	−17.277105	−5.220128
Na	7.891931	−18.652588	0.010956
Na	13.104201	0.695331	2.638424
Na	5.356054	4.679121	−0.150418

d(ACG)$^{Na+}$

E: −27509.89 kcal mol^{-1}

O	0.041953	−0.253496	−0.535050
C	−0.102198	0.087111	0.865226
C	1.252375	0.047190	1.562406
O	1.728249	−1.325107	1.751708
C	2.882214	−1.597741	0.920111
C	2.381458	0.785770	0.822144
C	3.125967	−0.329973	0.079705
O	3.191174	1.452804	1.840829
H	−0.850809	−0.350712	−0.909933
H	−0.778261	−0.617458	1.365934
H	−0.510930	1.102516	0.971710
H	1.120857	0.478664	2.560179
H	3.733485	−1.840484	1.561566
H	2.002583	1.547031	0.137237
H	2.699682	−0.434795	−0.918708
H	4.187643	−0.115605	−0.018744
N	2.645527	−2.797550	0.114135
C	1.825855	−2.910966	−1.004342
N	1.806050	−4.133973	−1.508246
C	2.649663	−4.873163	−0.681024
C	3.035018	−6.234628	−0.695505
N	2.601418	−7.114402	−1.614136
N	3.874101	−6.661748	0.291641
C	4.300760	−5.782639	1.218643
N	4.001520	−4.476634	1.322241
C	3.171710	−4.068138	0.341808
H	1.250821	−2.068017	−1.361020
H	2.953109	−8.079491	−1.608577
H	2.069457	−6.780088	−2.406606
H	4.976293	−6.191225	1.967391
P	4.571534	2.248122	1.452316
O	4.560845	2.745431	0.021807
O	4.780741	3.285132	2.540477
O	5.710015	1.074544	1.533057
C	5.901089	0.402648	2.810617
C	6.884377	−0.740116	2.651888
O	6.291555	−1.791872	1.832333
C	7.292983	−2.250522	0.883038
C	8.241915	−0.369903	1.993326
C	8.144935	−1.011869	0.606713
O	9.283210	−0.922105	2.864513

H	4.941079	0.013626	3.167869
H	6.290936	1.116449	3.545760
H	7.085701	−1.140913	3.653368
H	7.887439	−3.053422	1.319809
H	8.400118	0.709016	1.939439
H	7.614973	−0.311740	−0.045144
H	9.112395	−1.236021	0.160156
N	6.626416	−2.846861	−0.268535
C	6.700536	−4.261890	−0.452807
O	7.334212	−4.946346	0.374910
N	6.077004	−4.800646	−1.530067
C	5.389963	−4.036282	−2.399887
N	4.799293	−4.639328	−3.438788
C	5.281229	−2.616197	−2.216477
C	5.916391	−2.074755	−1.147784
H	4.906233	−5.656034	−3.590944
H	4.278322	−4.088141	−4.107420
H	4.717754	−1.998268	−2.905282
H	5.889438	−1.014234	−0.934858
P	10.757550	−1.427616	2.374870
O	11.280245	−0.643475	1.188166
O	11.629299	−1.445114	3.616311
O	10.478591	−2.943882	1.833610
C	10.264207	−4.042314	2.767575
C	11.149415	−5.233241	2.408828
O	10.643342	−5.958670	1.248604
C	11.474976	−5.713214	0.088801
C	12.601444	−4.862901	2.065951
C	12.581156	−4.734862	0.534415
O	13.442381	−5.957866	2.517113
H	9.212121	−4.338851	2.710184
H	10.502029	−3.713870	3.784216
H	11.122903	−5.930127	3.254637
H	11.876391	−6.672178	−0.248526
H	12.907546	−3.931138	2.553289
H	12.328269	−3.710030	0.257452
H	13.545251	−4.987586	0.086458
H	14.359263	−5.748612	2.261179
N	10.655383	−5.225274	−1.017495
C	10.074302	−3.964179	−1.164738
N	9.304939	−3.879365	−2.231775
C	9.359319	−5.145375	−2.819473
C	8.724886	−5.661414	−3.986039
O	7.954208	−5.058567	−4.771609
N	9.051164	−7.014754	−4.210883
C	9.912302	−7.763614	−3.433376
N	10.161620	−9.030178	−3.838552
N	10.510381	−7.279679	−2.339328
C	10.193244	−5.995307	−2.077072
H	10.245038	−3.185774	−0.436755
H	8.583478	−7.470851	−5.030617
H	9.623323	−9.458146	−4.608832
H	10.593907	−9.645737	−3.160842
O	5.503387	−12.000775	−10.716260
C	6.667422	−12.827927	−10.962232
C	7.350667	−13.188346	−9.648764
O	7.960574	−12.004063	−9.039456
C	7.343794	−11.732267	−7.751141
C	6.433063	−13.788288	−8.563306
C	6.061767	−12.568671	−7.719663
O	7.237961	−14.786046	−7.849251
H	5.213530	−11.636941	−11.570971
H	7.388520	−12.294818	−11.594572
H	6.377954	−13.759820	−11.469470
H	8.154145	−13.896752	−9.878649
H	8.032182	−12.015454	−6.955806
H	5.559751	−14.295465	−8.979601
H	5.240477	−12.051607	−8.219569
H	5.742542	−12.822084	−6.710714

N	7.146272	−10.289713	−7.593176
C	7.943166	−9.581742	−6.650723
O	8.858672	−10.188275	−6.049984
N	7.676529	−8.268884	−6.438378
C	6.712330	−7.632480	−7.132932
N	6.484032	−6.341441	−6.851719
C	5.969410	−8.304446	−8.161040
C	6.219330	−9.624322	−8.354677
H	6.983182	−5.880216	−6.075318
H	5.741199	−5.847893	−7.328886
H	5.236386	−7.778710	−8.761980
H	5.724326	−10.212954	−9.119841
P	6.914409	−15.310221	−6.334216
O	5.440715	−15.227884	−5.994833
O	7.573158	−16.670378	−6.201869
O	7.664940	−14.210710	−5.381335
C	9.113834	−14.223900	−5.228718
C	9.504459	−13.990614	−3.770961
O	9.370614	−12.591545	−3.374494
C	8.231555	−12.410525	−2.498308
C	8.664362	−14.790006	−2.761751
C	7.573904	−13.795953	−2.349877
O	9.556864	−15.168452	−1.668500
H	9.540269	−13.433222	−5.854815
H	9.504911	−15.195399	−5.547508
H	10.564499	−14.247093	−3.671364
H	8.583953	−12.010588	−1.543004
H	8.246198	−15.701330	−3.195531
H	6.734137	−13.901773	−3.036957
H	7.217096	−13.970041	−1.337215
N	7.336690	−11.392020	−3.040622
C	6.509840	−11.500755	−4.160819
N	5.855713	−10.386617	−4.420209
C	6.268406	−9.484924	−3.437146
C	5.896385	−8.129125	−3.199862
O	5.100142	−7.426303	−3.865036
N	6.542722	−7.594392	−2.068889
C	7.448192	−8.275003	−1.274805
N	7.973207	−7.602601	−0.238140
N	7.800732	−9.548476	−1.499494
C	7.193436	−10.090234	−2.571472
H	6.471381	−12.416478	−4.729915
H	6.337545	−6.590231	−1.843421
H	7.732768	−6.620169	−0.031129
H	8.683665	−8.065555	0.311544
P	8.982197	−15.854609	−0.294292
O	7.655546	−16.549672	−0.520489
O	10.114022	−16.696095	0.265902
O	8.680354	−14.565891	0.667464
C	9.809823	−13.746857	1.085714
C	9.342018	−12.693442	2.071661
O	8.518070	−11.709863	1.377776
C	7.336445	−11.443142	2.168634
C	8.505170	−13.226708	3.261425
C	7.082323	−12.736865	2.954562
O	9.048803	−12.627611	4.467474
H	10.254149	−13.263669	0.207817
H	10.564956	−14.381123	1.564002
H	10.234068	−12.189812	2.467677
H	7.505892	−10.594306	2.834479
H	8.556156	−14.317208	3.334061
H	6.570200	−13.476351	2.332767
H	6.492463	−12.566752	3.858634
H	8.551957	−12.989107	5.224235
N	6.265763	−11.029858	1.256557
C	5.780288	−9.713875	1.327228
O	6.136079	−8.903229	2.188399
N	4.856672	−9.394355	0.351039
C	4.330806	−10.250552	−0.612557

O	3.474396	−9.835512	−1.422368
C	4.840695	−11.610421	−0.601292
C	4.283301	−12.600790	−1.585505
C	5.795008	−11.919332	0.311708
H	4.504709	−8.402574	0.359706
H	3.200019	−12.705793	−1.450247
H	4.742561	−13.583320	−1.455202
H	4.446396	−12.265829	−2.615459
H	6.251290	−12.901095	0.351029
Na	5.463184	−17.589436	−5.433354
Na	8.619675	−18.633875	0.265613
Na	13.266846	−0.079526	2.458333
Na	4.892361	5.022497	0.834926

d(ACT)$^{Na+}$

E: −27600.23 kcal mol^{-1}

O	−0.143682	−0.254078	−0.428144
C	−0.092945	0.167611	0.956366
C	1.333323	0.088595	1.486551
O	1.770257	−1.298906	1.671096
C	2.801008	−1.647827	0.716671
C	2.401059	0.742512	0.591828
C	2.950616	−0.434669	−0.218436
O	3.372897	1.364010	1.490832
H	−1.080333	−0.311271	−0.685903
H	−0.734580	−0.470514	1.576895
H	−0.434539	1.208546	1.052473
H	1.341969	0.559482	2.474914
H	3.723039	−1.877244	1.259978
H	1.989145	1.523524	−0.051254
H	2.327448	−0.554134	−1.105764
H	3.975728	−0.276395	−0.544156
N	2.441248	−2.883242	0.019925
C	1.486476	−3.037284	−0.979892
N	1.400567	−4.280834	−1.423608
C	2.340989	−4.990191	−0.678695
C	2.723590	−6.353050	−0.688074
N	2.169960	−7.270332	−1.498779
N	3.694581	−6.738302	0.189266
C	4.241115	−5.821991	1.012568
N	3.951306	−4.513315	1.104861
C	2.992284	−4.146164	0.231858
H	0.873775	−2.205576	−1.298975
H	2.518973	−8.236855	−1.501257
H	1.505345	−6.974879	−2.201596
H	5.017180	−6.200208	1.675407
P	4.873377	1.819041	1.021496
O	4.964511	2.078857	−0.467486
O	5.283193	2.952325	1.943099
O	5.765084	0.477376	1.318081
C	5.887037	0.024988	2.698002
C	7.002339	−1.000370	2.805301
O	6.619510	−2.238161	2.131941
C	7.570785	−2.559503	1.086475
C	8.341810	−0.557023	2.182834
C	8.361374	−1.270719	0.829727
O	9.399903	−1.001310	3.086666
H	4.938855	−0.420651	3.018895
H	6.118065	0.878899	3.344858
H	7.148238	−1.221669	3.868923
H	8.216908	−3.377340	1.408712
H	8.412196	0.527426	2.076502
H	7.860138	−0.635841	0.097161
H	9.372661	−1.470280	0.483267
N	6.838270	−3.079735	−0.077497
C	6.929065	−4.464248	−0.402821
O	7.665450	−5.201892	0.284297
N	6.217090	−4.926919	−1.462472
C	5.406182	−4.116569	−2.168142
N	4.722962	−4.647583	−3.190891
C	5.265172	−2.729308	−1.827086
C	5.987253	−2.265553	−0.776394
H	4.784620	−5.656300	−3.402678
H	4.082621	−4.071363	−3.720699
H	4.596757	−2.079339	−2.378538
H	5.921978	−1.242977	−0.423740
P	10.985253	−0.821329	2.699435
O	11.186307	0.228230	1.626114
O	11.725774	−0.619096	4.008702
O	11.370199	−2.263246	2.030968
C	11.348574	−3.439045	2.891955
C	11.878036	−4.641988	2.133799
O	10.929894	−5.014556	1.091766
C	11.627604	−5.176679	−0.163079
C	13.249398	−4.442488	1.446093
C	12.895918	−4.319272	−0.042368
O	14.043465	−5.625632	1.730016
H	10.319709	−3.631696	3.217101
H	11.974993	−3.258862	3.772913
H	11.959099	−5.472609	2.847525
H	11.866530	−6.229082	−0.328464
H	13.763852	−3.551972	1.820190
H	12.688406	−3.272352	−0.278408
H	13.697725	−4.673188	−0.695113
H	14.915121	−5.501794	1.311505
N	10.711266	−4.803211	−1.246372
C	10.229114	−5.807299	−2.102919
O	10.604601	−6.982663	−2.051632
N	9.297467	−5.372594	−3.023663
C	8.875657	−4.064636	−3.230570
O	8.056818	−3.801864	−4.137876
C	9.442974	−3.066201	−2.340695
C	9.076601	−1.622927	−2.548735
C	10.304322	−3.491177	−1.381550
H	8.922874	−6.113512	−3.675436
H	9.343150	−1.307403	−3.564805
H	9.600516	−0.976907	−1.841029
H	7.997607	−1.467125	−2.441307
H	10.734123	−2.809365	−0.657416
O	6.236925	−11.535177	−10.817595
C	7.389829	−12.409185	−10.877642
C	7.719925	−12.949417	−9.491036
O	8.231276	−11.892198	−8.615675
C	7.287167	−11.612079	−7.551268
C	6.535800	−13.580578	−8.731770
C	6.024794	−12.422557	−7.871602
O	7.061374	−14.736604	−7.994613
H	6.145259	−11.107178	−11.686740
H	8.264356	−11.865441	−11.257238
H	7.192558	−13.263292	−11.542101
H	8.519607	−13.688539	−9.607618
H	7.736328	−11.905698	−6.600066
H	5.764548	−13.960679	−9.405378
H	5.340291	−11.838209	−8.488035
H	5.490413	−12.745603	−6.980461
N	7.053249	−10.176727	−7.441717
C	6.224847	−9.373650	−8.215622
N	6.194337	−8.115901	−7.802908
C	7.049036	−8.082602	−6.701489
C	7.440236	−7.038527	−5.830491
N	7.001467	−5.772817	−5.938874
N	8.324586	−7.351548	−4.840440
C	8.785249	−8.613877	−4.732101
N	8.482939	−9.670487	−5.501385
C	7.602914	−9.348974	−6.468735

H	5.703006	−9.768424	−9.076015
H	7.302360	−5.060734	−5.261700
H	6.295427	−5.548107	−6.627003
H	9.482206	−8.781700	−3.913145
P	6.630300	−15.184232	−6.485767
O	5.156298	−14.968905	−6.200641
O	7.157573	−16.593498	−6.295013
O	7.425523	−14.124255	−5.531762
C	8.834745	−14.302810	−5.220308
C	9.042418	−14.192272	−3.714979
O	8.859918	−12.822253	−3.249107
C	7.613826	−12.686070	−2.522697
C	8.069266	−15.048908	−2.883842
C	6.940577	−14.069000	−2.541359
O	8.821294	−15.547410	−1.733761
H	9.404545	−13.521518	−5.733573
H	9.172253	−15.286528	−5.561799
H	10.077731	−14.473265	−3.494439
H	7.854594	−12.352810	−1.509168
H	7.701633	−15.914220	−3.441216
H	6.189950	−14.130145	−3.329166
H	6.458212	−14.305666	−1.595855
N	6.792510	−11.617616	−3.090119
C	5.883725	−11.659075	−4.152474
N	5.299036	−10.495728	−4.364590
C	5.842917	−9.629927	−3.412164
C	5.611979	−8.244607	−3.153294
O	4.831448	−7.465777	−3.750124
N	6.396009	−7.770452	−2.083511
C	7.309528	−8.522900	−1.370860
N	7.977944	−7.906589	−0.384352
N	7.534422	−9.819846	−1.620724
C	6.782987	−10.309250	−2.622778
H	5.714443	−12.564930	−4.714608
H	6.303462	−6.755150	−1.850107
H	7.845241	−6.909012	−0.158760
H	8.657469	−8.452005	0.127629
P	8.136084	−16.040903	−0.331723
O	6.715450	−16.533190	−0.513759
O	9.120431	−17.010289	0.296897
O	8.046829	−14.654961	0.536105
C	9.297152	−13.976266	0.853449
C	9.045161	−12.877770	1.869483
O	8.257574	−11.813812	1.257000
C	7.112800	−11.513584	2.087595
C	8.289361	−13.319064	3.143407
C	6.870866	−12.773753	2.930935
O	8.957839	−12.695443	4.272179
H	9.716407	−13.546146	−0.063256
H	10.011348	−14.696326	1.269197
H	10.020785	−12.466107	2.158956
H	7.319213	−10.642213	2.711786
H	8.297405	−14.407193	3.261621
H	6.279981	−13.508638	2.378177
H	6.361270	−12.549402	3.871269
H	8.490076	−12.972979	5.081133
N	6.003527	−11.120077	1.210154
C	5.554425	−9.790469	1.240340
O	5.977138	−8.945685	2.035984
N	4.586432	−9.493890	0.300649
C	3.976287	−10.383768	−0.578160
O	3.075351	−9.988207	−1.348606
C	4.458926	−11.753284	−0.531334
C	3.818104	−12.775369	−1.428375
C	5.462556	−12.041732	0.335352
H	4.264230	−8.491961	0.281271
H	2.742819	−12.842230	−1.223297
H	4.258159	−13.763242	−1.275630
H	3.925944	−12.497669	−2.482450
H	5.906523	−13.029495	0.388702
Na	12.653840	1.463703	3.102207
Na	5.785268	4.331059	0.006119
Na	4.948470	−17.284041	−5.555486
Na	7.354654	−18.666366	0.482807

d(AGA)$^{Na+}$

E: −27601.38 kcal mol^{-1}

O	0.044820	−0.187562	−0.543724
C	−0.102151	0.171856	0.851452
C	1.246311	0.111966	1.558314
O	1.695459	−1.267745	1.757701
C	2.854979	−1.562611	0.941211
C	2.393846	0.826258	0.822671
C	3.122689	−0.307018	0.090952
O	3.208485	1.482086	1.845380
H	−0.847092	−0.265204	−0.925207
H	−0.796072	−0.513053	1.355107
H	−0.490626	1.196577	0.943928
H	1.115554	0.551655	2.552671
H	3.697634	−1.808777	1.592205
H	2.034256	1.591871	0.132175
H	2.696890	−0.413975	−0.907780
H	4.189205	−0.112206	−0.002618
N	2.612653	−2.769924	0.148158
C	1.777941	−2.900859	−0.955714
N	1.749385	−4.132940	−1.438801
C	2.605066	−4.858657	−0.612573
C	2.990450	−6.220114	−0.607059
N	2.533859	−7.126415	−1.490475
N	3.859075	−6.621761	0.365216
C	4.297248	−5.726719	1.272600
N	3.987890	−4.423390	1.363270
C	3.142260	−4.035514	0.388817
H	1.201439	−2.061799	−1.319803
H	2.903375	−8.085711	−1.479868
H	1.994858	−6.809039	−2.285420
H	4.990459	−6.120971	2.013172
P	4.600698	2.258876	1.461604
O	4.589300	2.781357	0.039911
O	4.835758	3.272639	2.566094
O	5.717415	1.063343	1.512376
C	5.911351	0.363677	2.774267
C	6.876017	−0.790198	2.581898
O	6.252998	−1.832016	1.770294
C	7.227638	−2.292665	0.796577
C	8.218383	−0.424443	1.889598
C	8.073567	−1.053561	0.502457
O	9.286204	−0.979826	2.730189
H	4.949539	−0.015870	3.136722
H	6.322151	1.056962	3.517616
H	7.099146	−1.201025	3.574573
H	7.835825	−3.097732	1.217015
H	8.382388	0.653496	1.841910
H	7.507620	−0.357460	−0.124146
H	9.023149	−1.273000	0.017082
N	6.551090	−2.876102	−0.339906
C	5.747189	−2.254818	−1.295402
N	5.291326	−3.099428	−2.200678
C	5.822674	−4.340935	−1.840886
C	5.689725	−5.628251	−2.438202
O	4.999829	−5.937866	−3.438423
N	6.459798	−6.602077	−1.773807
C	7.247846	−6.367737	−0.661184
N	7.952708	−7.403472	−0.167601
N	7.324135	−5.171622	−0.065318

C	6.615576	−4.218795	−0.690935
H	5.547374	−1.194420	−1.265494
H	6.447416	−7.564820	−2.187919
H	8.015124	−8.293045	−0.680922
H	8.688500	−7.162059	0.490164
P	10.659460	−1.694110	2.219855
O	11.236071	−1.036819	0.979564
O	11.569219	−1.766132	3.431135
O	10.179806	−3.179737	1.745388
C	10.005969	−4.285471	2.672893
C	10.964986	−5.422903	2.331988
O	10.592593	−6.089778	1.086851
C	11.491224	−5.707232	0.013631
C	12.426861	−4.989692	2.137133
C	12.531838	−4.747797	0.621400
O	13.262212	−6.093456	2.575675
H	8.974555	−4.636881	2.573031
H	10.187998	−3.952700	3.699635
H	10.890740	−6.172825	3.127723
H	11.942824	−6.623160	−0.374295
H	12.660467	−4.089136	2.714156
H	12.289469	−3.708341	0.396683
H	13.535066	−4.957365	0.243335
H	14.190302	−5.839517	2.420199
N	10.728369	−5.150318	−1.104820
C	10.236532	−3.860188	−1.285496
N	9.460106	−3.748039	−2.350291
C	9.416899	−5.029872	−2.899172
C	8.730310	−5.576293	−4.008946
N	7.926707	−4.864758	−4.826386
N	8.905174	−6.903684	−4.258946
C	9.700464	−7.639356	−3.459279
N	10.385523	−7.225848	−2.382444
C	10.202741	−5.912952	−2.141998
H	10.480182	−3.062252	−0.600688
H	7.340497	−5.367603	−5.503072
H	7.662293	−3.926533	−4.553247
H	9.778258	−8.691411	−3.725398
O	5.609105	−11.985848	−10.585005
C	6.798613	−12.768674	−10.855137
C	7.471624	−13.175065	−9.550687
O	8.073248	−12.014004	−8.887145
C	7.443357	−11.780358	−7.604072
C	6.542359	−13.820161	−8.503920
C	6.177455	−12.647975	−7.592262
O	7.326444	−14.860043	−7.835391
H	5.306081	−11.607595	−11.428841
H	7.512384	−12.187665	−11.452297
H	6.538889	−13.682736	−11.408319
H	8.281822	−13.869193	−9.798226
H	8.134034	−12.049220	−6.803198
H	5.663575	−14.288810	−8.952300
H	5.333735	−12.116842	−8.037385
H	5.891876	−12.968660	−6.593204
N	7.198968	−10.339804	−7.435267
C	7.919991	−9.642749	−6.450255
O	8.787691	−10.166613	−5.745048
N	7.561752	−8.317314	−6.316038
C	6.635174	−7.615184	−7.078000
O	6.426225	−6.403460	−6.847184
C	5.971698	−8.377571	−8.123620
C	4.980268	−7.685945	−9.019329
C	6.291911	−9.691132	−8.253913
H	8.062552	−7.784357	−5.553540
H	5.450752	−6.850434	−9.552413
H	4.151759	−7.267161	−8.434572
H	4.569467	−8.383003	−9.755338
H	5.863714	−10.311954	−9.035212
P	6.841901	−15.587820	−6.449371

O	5.344830	−15.492682	−6.243920
O	7.449697	−16.977260	−6.458643
O	7.530841	−14.661741	−5.286803
C	8.982173	−14.669034	−5.151147
C	9.379072	−14.208983	−3.756551
O	9.161408	−12.775103	−3.595553
C	8.187436	−12.514330	−2.556886
C	8.608362	−14.896914	−2.613594
C	7.538766	−13.869167	−2.232328
O	9.572438	−15.155707	−1.547298
H	9.418822	−14.000355	−5.901374
H	9.356721	−15.685596	−5.313002
H	10.452561	−14.397529	−3.642569
H	8.679652	−12.073666	−1.688760
H	8.170095	−15.848697	−2.920560
H	6.656970	−14.043354	−2.851164
H	7.248708	−13.942486	−1.185990
N	7.245588	−11.485967	−3.031359
C	7.272063	−10.177125	−2.461205
O	8.071527	−9.927474	−1.536828
N	6.402838	−9.250496	−2.945021
C	5.556471	−9.539482	−3.951454
N	4.733838	−8.574239	−4.383520
C	5.545419	−10.836769	−4.561873
C	6.414735	−11.761334	−4.083128
H	4.808373	−7.611064	−4.012326
H	4.106490	−8.761521	−5.154403
H	4.880995	−11.062689	−5.386559
H	6.509384	−12.753791	−4.507770
P	9.098419	−15.794135	−0.112253
O	7.789673	−16.546529	−0.236801
O	10.288890	−16.565897	0.426147
O	8.797708	−14.471311	0.802155
C	9.923611	−13.615323	1.153215
C	9.468006	−12.532137	2.112221
O	8.621414	−11.580874	1.402836
C	7.464994	−11.280719	2.216183
C	8.663389	−13.027734	3.340194
C	7.233236	−12.542147	3.060322
O	9.243621	−12.396579	4.512603
H	10.330706	−13.157740	0.244060
H	10.704632	−14.217748	1.630734
H	10.364595	−12.007951	2.469968
H	7.652263	−10.405656	2.842489
H	8.713074	−14.115822	3.442800
H	6.697035	−13.301865	2.484743
H	6.674258	−12.334380	3.976094
H	8.768801	−12.735772	5.293485
N	6.369231	−10.903340	1.317981
C	5.858412	−9.595541	1.374300
O	6.208794	−8.765505	2.218291
N	4.914925	−9.312091	0.405987
C	4.358885	−10.205935	−0.504449
O	3.464732	−9.829601	−1.292229
C	4.880688	−11.561355	−0.462578
C	4.277204	−12.599966	−1.366762
C	5.875749	−11.827818	0.420435
H	4.537234	−8.329725	0.411262
H	3.207319	−12.714333	−1.153241
H	4.759676	−13.569835	−1.226130
H	4.362178	−12.309697	−2.418705
H	6.342703	−12.803753	0.477275
Na	5.217441	−17.912729	−6.097911
Na	8.867975	−18.552206	0.599767
Na	13.293579	−0.626044	2.159135
Na	4.967641	5.036336	0.877667

d(AGC)$^{Na+}$

E: −27512.91 kcal mol^{-1}

O	−0.146604	−0.398379	−0.393753
C	−0.149653	−0.014698	1.002212
C	1.269687	−0.018073	1.557141
O	1.794842	−1.378647	1.699001
C	2.849020	−1.628977	0.736596
C	2.304426	0.738378	0.705972
C	2.952534	−0.365285	−0.134317
O	3.218957	1.392447	1.641225
H	−1.070671	−0.537076	−0.665169
H	−0.758654	−0.711629	1.591643
H	−0.560915	0.998486	1.121547
H	1.230433	0.415130	2.562117
H	3.773833	−1.840113	1.282262
H	1.849063	1.511084	0.082442
H	2.378302	−0.475734	−1.054405
H	3.979716	−0.133304	−0.403975
N	2.559276	−2.844101	−0.026907
C	1.717915	−2.981819	−1.125495
N	1.682879	−4.216910	−1.598891
C	2.537956	−4.939284	−0.769327
C	2.923233	−6.300546	−0.761342
N	2.480503	−7.200211	−1.655993
N	3.783505	−6.703021	0.218806
C	4.221970	−5.804093	1.122722
N	3.924178	−4.496538	1.201333
C	3.080893	−4.111460	0.223505
H	1.140524	−2.148794	−1.498646
H	2.810263	−8.173512	−1.624210
H	1.909950	−6.887904	−2.430383
H	4.907289	−6.197404	1.871122
P	4.652683	2.033787	1.176427
O	4.680854	2.376614	−0.298402
O	4.948601	3.161239	2.148846
O	5.694374	0.790331	1.392582
C	5.874387	0.287552	2.747175
C	6.951170	−0.781950	2.765524
O	6.486487	−1.977325	2.064678
C	7.413676	−2.315495	1.002723
C	8.288806	−0.379715	2.104905
C	8.203499	−1.033642	0.725444
O	9.345161	−0.921916	2.958461
H	4.930102	−0.136567	3.107209
H	6.170617	1.110847	3.407474
H	7.132469	−1.047235	3.813647
H	8.062652	−3.135347	1.328830
H	8.418891	0.703190	2.045946
H	7.633000	−0.369147	0.072309
H	9.177979	−1.216196	0.274134
N	6.683528	−2.829646	−0.139657
C	5.916358	−2.126765	−1.070298
N	5.421390	−2.908671	−2.010577
C	5.881070	−4.191058	−1.699445
C	5.719900	−5.438472	−2.372078
O	5.045401	−5.670875	−3.402857
N	6.443723	−6.472169	−1.749202
C	7.211108	−6.329886	−0.608102
N	7.876892	−7.409390	−0.170816
N	7.322943	−5.169618	0.050598
C	6.668041	−4.158326	−0.539607
H	5.774313	−1.059488	−0.992519
H	6.375201	−7.420974	−2.188383
H	7.807131	−8.328948	−0.632004
H	8.453889	−7.292766	0.651822
P	10.900048	−1.113531	2.482084
O	11.259133	−0.229910	1.305987
O	11.747503	−0.970095	3.732439
O	10.928208	−2.657091	1.937539
C	10.743907	−3.741582	2.895594
C	11.390802	−5.016888	2.376893
O	10.629624	−5.566839	1.259676
C	11.446247	−5.615414	0.066320
C	12.839113	−4.848933	1.877076
C	12.681565	−4.746004	0.354044
O	13.569455	−6.039485	2.275123
H	9.671244	−3.904554	3.048618
H	11.207729	−3.467805	3.849493
H	11.365884	−5.750460	3.192246
H	11.711801	−6.649522	−0.153544
H	13.313722	−3.958865	2.303174
H	12.500851	−3.704435	0.077432
H	13.564526	−5.101263	−0.183136
H	14.477028	−5.956903	1.929239
N	10.633799	−5.166348	−1.076450
C	10.288330	−6.082564	−2.107556
O	10.736959	−7.251852	−2.072032
N	9.475934	−5.647984	−3.106641
C	9.026065	−4.376888	−3.138852
N	8.233967	−4.017949	−4.160576
C	9.371412	−3.438605	−2.112295
C	10.154073	−3.883579	−1.097684
H	7.923756	−4.717948	−4.852012
H	7.805483	−3.101440	−4.162572
H	8.993152	−2.423633	−2.127506
H	10.422081	−3.267406	−0.246801
O	5.652663	−11.808869	−10.832066
C	6.767388	−12.709436	−11.040467
C	7.345050	−13.165310	−9.705159
O	8.000550	−12.055886	−9.006010
C	7.279988	−11.732883	−7.790235
C	6.327756	−13.736891	−8.698638
C	5.961661	−12.512336	−7.858988
O	7.030448	−14.790684	−7.956746
H	5.416799	−11.427790	−11.695885
H	7.561869	−12.209940	−11.609393
H	6.444733	−13.600864	−11.597980
H	8.113060	−13.916128	−9.919042
H	7.882125	−12.026396	−6.926914
H	5.459853	−14.190489	−9.182715
H	5.209584	−11.940443	−8.406181
H	5.563107	−12.765943	−6.878411
N	7.122113	−10.291394	−7.671469
C	6.339949	−9.432281	−8.445269
N	6.410050	−8.179064	−8.033457
C	7.275769	−8.202198	−6.936165
C	7.716630	−7.165535	−6.060911
O	7.422179	−5.945801	−6.108161
N	8.571056	−7.651232	−5.050650
C	8.972117	−8.967732	−4.917424
N	9.816522	−9.267593	−3.907078
N	8.571678	−9.935878	−5.746355
C	7.733875	−9.506184	−6.705169
H	5.780964	−9.799661	−9.295585
H	8.906735	−6.947302	−4.353441
H	10.074152	−8.564624	−3.201364
H	9.929339	−10.254263	−3.697776
P	6.657454	−15.263697	−6.437877
O	5.181417	−15.121822	−6.128071
O	7.267724	−16.639714	−6.256065
O	7.420650	−14.162564	−5.496656
C	8.860473	−14.212858	−5.287130
C	9.183039	−13.966474	−3.817429
O	8.978929	−12.569482	−3.442293
C	7.848084	−12.435906	−2.545069
C	8.324601	−14.794879	−2.844113
C	7.206374	−13.827428	−2.439812

O	9.192267	−15.185677	−1.737397
H	9.333390	−13.442358	−5.904776
H	9.240811	−15.197853	−5.575935
H	10.244919	−14.189907	−3.668648
H	8.203657	−12.073393	−1.580298
H	7.931404	−15.702348	−3.308405
H	6.378423	−13.946192	−3.139182
H	6.835732	−14.027280	−1.437098
N	6.951665	−11.373742	−3.042238
C	6.985217	−10.093652	−2.413950
O	7.701806	−9.929553	−1.405011
N	6.226712	−9.097789	−2.940771
C	5.443158	−9.305566	−4.017219
N	4.722775	−8.274770	−4.479356
C	5.395985	−10.584270	−4.661098
C	6.181808	−11.569055	−4.158554
H	4.822194	−7.332727	−4.065358
H	4.172342	−8.387202	−5.320539
H	4.784013	−10.748723	−5.539572
H	6.260534	−12.543889	−4.622029
P	8.598492	−15.887689	−0.378541
O	7.235220	−16.506259	−0.606732
O	9.691248	−16.805027	0.139893
O	8.380527	−14.614308	0.624922
C	9.558847	−13.857805	1.028225
C	9.165783	−12.788663	2.029979
O	8.360925	−11.772099	1.363387
C	7.183744	−11.494200	2.157266
C	8.348648	−13.286260	3.246455
C	6.928683	−12.773355	2.966950
O	8.934054	−12.678591	4.428676
H	10.008797	−13.389338	0.145362
H	10.290086	−14.533895	1.486079
H	10.090633	−12.322272	2.395039
H	7.359924	−10.634610	2.807018
H	8.380194	−14.376437	3.336025
H	6.387455	−13.514048	2.372437
H	6.365448	−12.578124	3.882863
H	8.441417	−13.007793	5.202792
N	6.106268	−11.091069	1.247374
C	5.668453	−9.755690	1.264181
O	6.073588	−8.918153	2.075650
N	4.730115	−9.449948	0.297653
C	4.154651	−10.329458	−0.613479
O	3.288969	−9.927088	−1.420502
C	4.626437	−11.701961	−0.556850
C	4.012661	−12.716654	−1.481328
C	5.592513	−12.002079	0.347692
H	4.401805	−8.450985	0.284187
H	2.932046	−12.787079	−1.307838
H	4.450016	−13.705308	−1.325591
H	4.147361	−12.429431	−2.529947
H	6.022177	−12.994493	0.414642
Na	5.102542	−17.456919	−5.494369
Na	8.110713	−18.662376	0.099399
Na	13.067127	0.746464	2.620444
Na	5.204691	4.692355	0.281496

d(AGG)$^{Na+}$

E: −27512.88 kcal mol^{-1}

O	0.026790	−0.142343	−0.332068
C	−0.122239	0.153478	1.077866
C	1.228098	0.077854	1.779777
O	1.692769	−1.303461	1.919167
C	2.854088	−1.550066	1.089539
C	2.365860	0.836388	1.074139
C	3.112037	−0.255042	0.297206
O	3.172914	1.460269	2.122304
H	−0.864756	−0.216678	−0.715071
H	−0.807421	−0.560872	1.551488
H	−0.522180	1.168639	1.215670
H	1.094349	0.472854	2.792357
H	3.699021	−1.818252	1.728564
H	1.996016	1.624678	0.415523
H	2.694159	−0.322423	−0.708328
H	4.177639	−0.046063	0.220568
N	2.620947	−2.721387	0.242525
C	1.788108	−2.809964	−0.866966
N	1.763896	−4.021550	−1.399380
C	2.619643	−4.777869	−0.600774
C	3.011291	−6.136452	−0.650425
N	2.563084	−7.007346	−1.573177
N	3.876675	−6.574566	0.308559
C	4.308787	−5.716413	1.253566
N	3.996294	−4.418107	1.394962
C	3.152419	−3.994214	0.433981
H	1.210191	−1.958480	−1.198325
H	2.947918	−7.960247	−1.608884
H	2.030501	−6.657565	−2.358938
H	4.999621	−6.138962	1.980471
P	4.527512	2.307117	1.754748
O	4.464299	2.900825	0.362275
O	4.749822	3.270508	2.905602
O	5.689431	1.155961	1.718599
C	5.943812	0.404601	2.939211
C	6.902967	−0.732804	2.650361
O	6.246136	−1.732160	1.813552
C	7.200051	−2.168515	0.810227
C	8.217024	−0.328101	1.921785
C	8.034055	−0.920254	0.522547
O	9.322078	−0.888891	2.708744
H	5.001679	0.002134	3.327662
H	6.384400	1.068251	3.692408
H	7.169248	−1.193014	3.610499
H	7.821424	−2.977105	1.203723
H	8.364066	0.753105	1.899013
H	7.449214	−0.210454	−0.070597
H	8.971025	−1.126231	0.006345
N	6.506733	−2.738997	−0.321403
C	5.703997	−2.113990	−1.276629
N	5.264927	−2.953520	−2.194966
C	5.805935	−4.194236	−1.845354
C	5.707328	−5.473607	−2.466291
O	5.051935	−5.776211	−3.492680
N	6.465334	−6.449225	−1.791752
C	7.222713	−6.222571	−0.655641
N	7.908894	−7.259883	−0.144176
N	7.285440	−5.029138	−0.053465
C	6.583306	−4.077512	−0.685212
H	5.490556	−1.056293	−1.235817
H	6.469751	−7.410117	−2.209123
H	7.946629	−8.169066	−0.625541
H	8.631093	−7.023571	0.530913
P	10.685242	−1.574375	2.131801
O	11.212177	−0.885734	0.887891
O	11.635780	−1.660185	3.311213
O	10.206783	−3.059394	1.647961
C	9.977506	−4.137399	2.599622
C	10.891794	−5.323837	2.305729
O	10.474028	−6.047166	1.107665
C	11.378586	−5.779395	0.003147
C	12.363839	−4.953871	2.064724
C	12.450302	−4.808494	0.536362
O	13.168500	−6.057977	2.557795
H	8.934181	−4.450893	2.497283

H	10.161703	−3.785588	3.619516
H	10.806184	−6.024290	3.144193
H	11.801718	−6.735353	−0.314026
H	12.636730	−4.028974	2.583009
H	12.222583	−3.780190	0.251638
H	13.442870	−5.061034	0.156008
H	14.101958	−5.845938	2.374329
N	10.633967	−5.282557	−1.150508
C	10.130776	−3.997998	−1.379500
N	9.427730	−3.919535	−2.491496
C	9.442749	−5.210916	−3.025313
C	8.830317	−5.747817	−4.194965
O	8.122501	−5.146503	−5.040684
N	9.090292	−7.123378	−4.343339
C	9.868423	−7.883257	−3.491973
N	10.055970	−9.176128	−3.827432
N	10.434323	−7.383954	−2.386890
C	10.186594	−6.071370	−2.203687
H	10.305091	−3.194775	−0.679880
H	8.656159	−7.589048	−5.172759
H	9.641195	−9.592458	−4.673439
H	10.502730	−9.779450	−3.149929
O	5.425498	−12.317856	−10.661517
C	6.566535	−13.160179	−10.958536
C	7.345672	−13.461320	−9.684625
O	8.000429	−12.249386	−9.180480
C	7.496707	−11.924386	−7.861145
C	6.507679	−14.002628	−8.508693
C	6.212404	−12.744776	−7.692343
O	7.356101	−14.968210	−7.803915
H	5.045707	−12.024978	−11.508314
H	7.237236	−12.660265	−11.668649
H	6.238182	−14.113533	−11.396927
H	8.129947	−14.183164	−9.937155
H	8.245006	−12.178843	−7.110672
H	5.601362	−14.517362	−8.834762
H	5.359642	−12.235513	−8.146137
H	5.974637	−12.959324	−6.652460
N	7.312975	−10.472562	−7.744861
C	8.113239	−9.739214	−6.823791
O	9.037812	−10.323000	−6.215799
N	7.823317	−8.428523	−6.622995
C	6.850024	−7.812283	−7.324633
N	6.602252	−6.523954	−7.051724
C	6.101997	−8.511500	−8.330658
C	6.364386	−9.832495	−8.502841
H	7.127450	−6.031502	−6.312550
H	5.868140	−6.035200	−7.547436
H	5.354174	−8.006071	−8.930540
H	5.859104	−10.445157	−9.242858
P	7.028528	−15.497251	−6.289071
O	5.550861	−15.425732	−5.967630
O	7.701665	−16.848792	−6.149205
O	7.760764	−14.387371	−5.332224
C	9.210544	−14.376332	−5.183469
C	9.586091	−13.974460	−3.761635
O	9.380934	−12.546001	−3.534556
C	8.338832	−12.322199	−2.555862
C	8.777592	−14.703200	−2.672985
C	7.689788	−13.691615	−2.296776
O	9.696682	−14.997406	−1.577089
H	9.636350	−13.662682	−5.896699
H	9.605374	−15.376759	−5.387417
H	10.655251	−14.175237	−3.632572
H	8.767953	−11.889174	−1.651546
H	8.354792	−15.644315	−3.030556
H	6.829595	−13.847973	−2.949759
H	7.368297	−13.798713	−1.262517
N	7.410435	−11.297956	−3.071393

C	7.323558	−10.027068	−2.429658
O	7.977719	−9.821770	−1.387973
N	6.513206	−9.085198	−2.982389
C	5.781924	−9.345782	−4.083195
N	4.990264	−8.377758	−4.562181
C	5.853121	−10.620313	−4.734944
C	6.694655	−11.546151	−4.211919
H	4.997823	−7.429670	−4.147174
H	4.464206	−8.539243	−5.410679
H	5.275395	−10.825056	−5.627342
H	6.854693	−12.517851	−4.663638
P	9.164328	−15.742916	−0.214656
O	7.892760	−16.526426	−0.467860
O	10.348909	−16.510992	0.341722
O	8.763388	−14.494882	0.762628
C	9.834239	−13.634792	1.248044
C	9.270102	−12.601658	2.204860
O	8.473151	−11.635686	1.458768
C	7.250066	−11.379295	2.187417
C	8.363810	−13.163280	3.330554
C	6.957530	−12.686310	2.937139
O	8.818988	−12.575319	4.578346
H	10.311249	−13.130862	0.399358
H	10.582424	−14.242723	1.769211
H	10.118675	−12.079197	2.667226
H	7.384884	−10.543373	2.877307
H	8.424533	−14.253807	3.391743
H	6.497844	−13.423488	2.273134
H	6.306809	−12.536373	3.802198
H	8.281614	−12.958063	5.295904
N	6.231873	−10.945438	1.228364
C	5.771442	−9.618565	1.279864
O	6.109808	−8.816653	2.155700
N	4.889172	−9.281629	0.272797
C	4.371822	−10.128427	−0.702766
O	3.532877	−9.704356	−1.526102
C	4.866052	−11.494587	−0.683242
C	4.322893	−12.480470	−1.680423
C	5.782379	−11.821425	0.262187
H	4.534981	−8.290762	0.293667
H	3.238616	−12.591619	−1.557259
H	4.785069	−13.461956	−1.550756
H	4.493317	−12.140999	−2.707282
H	6.215985	−12.812380	0.315054
Na	5.575425	−17.792222	−5.417794
Na	8.987151	−18.553690	0.269439
Na	13.321566	−0.508645	2.015930
Na	4.766345	5.119052	1.280034

d(ATA)$^{Na+}$

E: −27693.30 kcal mol^{-1}

O	−0.199053	−0.463517	0.131146
C	−0.078041	−0.175689	1.545318
C	1.386400	−0.203940	1.965788
O	1.915259	−1.571175	2.004311
C	2.893868	−1.782596	0.961929
C	2.335041	0.591742	1.050394
C	2.953144	−0.478596	0.145534
O	3.296431	1.270580	1.915158
H	−1.144424	−0.578824	−0.068832
H	−0.631340	−0.914706	2.138170
H	−0.477846	0.824291	1.767688
H	1.444363	0.186591	2.986956
H	3.852949	−2.034066	1.425295
H	1.808017	1.352323	0.470439
H	2.347885	−0.558800	−0.757436

H	3.968513	−0.228617	−0.151721
N	2.528549	−2.950012	0.152594
C	1.529849	−3.017656	−0.814168
N	1.449972	−4.204354	−1.392652
C	2.436216	−4.968511	−0.773090
C	2.849424	−6.308665	−0.961170
N	2.307030	−7.126623	−1.878623
N	3.844124	−6.777150	−0.154294
C	4.393089	−5.955414	0.761374
N	4.095269	−4.666867	1.003858
C	3.107370	−4.217498	0.203306
H	0.883196	−2.173187	−1.006836
H	2.670418	−8.079415	−2.005382
H	1.606187	−6.766475	−2.512680
H	5.183045	−6.397109	1.365518
P	4.635284	2.008771	1.321749
O	4.507744	2.338042	−0.150518
O	4.940142	3.161328	2.260734
O	5.771876	0.837977	1.447676
C	6.056064	0.319795	2.778511
C	7.152927	−0.723626	2.702623
O	6.668202	−1.902307	1.987802
C	7.599252	−2.238282	0.927608
C	8.447533	−0.273404	1.985150
C	8.336463	−0.935746	0.610273
O	9.555672	−0.768722	2.799998
H	5.148298	−0.131131	3.194578
H	6.379244	1.139160	3.431298
H	7.400653	−1.016801	3.729750
H	8.286228	−3.016029	1.267761
H	8.533104	0.813285	1.916843
H	7.735094	−0.285523	−0.029098
H	9.300899	−1.092930	0.130413
N	6.858029	−2.834366	−0.183028
C	6.991472	−4.217782	−0.400023
O	7.692368	−4.948210	0.303050
N	6.277885	−4.697006	−1.476227
C	5.422476	−3.982326	−2.300972
O	4.816230	−4.559827	−3.229664
C	5.299925	−2.562847	−2.005993
C	4.402978	−1.716818	−2.866730
C	6.018493	−2.068057	−0.965202
H	6.409260	−5.733123	−1.667758
H	4.708374	−1.777132	−3.918154
H	4.434972	−0.669205	−2.557370
H	3.365619	−2.067999	−2.816448
H	5.963137	−1.023500	−0.682848
P	11.100954	−0.877707	2.268463
O	11.371606	0.036794	1.092086
O	11.983888	−0.702433	3.490247
O	11.187257	−2.409965	1.703459
C	11.178251	−3.515109	2.654555
C	11.936404	−4.706177	2.086587
O	11.165667	−5.367795	1.037073
C	11.825933	−5.233851	−0.241617
C	13.300292	−4.362334	1.461737
C	12.981102	−4.237568	−0.034136
O	14.193622	−5.471614	1.744056
H	10.140755	−3.801122	2.860344
H	11.658278	−3.196846	3.586061
H	12.068996	−5.429163	2.900223
H	12.179302	−6.217962	−0.563535
H	13.712544	−3.434780	1.872374
H	12.655087	−3.217996	−0.249273
H	13.839907	−4.473417	−0.667013
H	15.049000	−5.275516	1.319829
N	10.852406	−4.819873	−1.248499
C	10.236536	−3.582388	−1.373971
N	9.358170	−3.538232	−2.362641

C	9.384369	−4.817985	−2.917549
C	8.687293	−5.408082	−3.998895
N	7.784464	−4.760930	−4.758723
N	8.966616	−6.710699	−4.285428
C	9.862357	−7.383756	−3.541047
N	10.576764	−6.923574	−2.500910
C	10.301117	−5.631917	−2.232531
H	10.471227	−2.773867	−0.696455
H	7.255011	−5.275304	−5.472353
H	7.504378	−3.821865	−4.510609
H	10.007156	−8.425097	−3.823161
O	5.431974	−12.226379	−10.201039
C	6.586422	−13.075576	−10.415529
C	7.308280	−13.326095	−9.096782
O	7.933717	−12.093956	−8.610532
C	7.370672	−11.717686	−7.326431
C	6.414391	−13.835288	−7.943193
C	6.093723	−12.554418	−7.172696
O	7.183368	−14.849985	−7.206934
H	5.090637	−11.965786	−11.074233
H	7.287140	−12.600001	−11.113258
H	6.279236	−14.045573	−10.832530
H	8.105557	−14.052578	−9.287421
H	8.088373	−11.934285	−6.538670
H	5.513805	−14.333959	−8.307008
H	5.250749	−12.071606	−7.671571
H	5.816831	−12.734502	−6.136640
N	7.166488	−10.266023	−7.278521
C	7.941631	−9.517737	−6.375989
O	8.859434	−9.997738	−5.704421
N	7.584286	−8.187280	−6.290419
C	6.624479	−7.525736	−7.045789
O	6.418511	−6.303373	−6.862641
C	5.920188	−8.338631	−8.023782
C	4.898051	−7.690989	−8.918341
C	6.224600	−9.661713	−8.087960
H	8.110538	−7.625116	−5.570682
H	5.353309	−6.895328	−9.521395
H	4.098236	−7.227666	−8.327358
H	4.451311	−8.427058	−9.592981
H	5.754080	−10.326456	−8.806502
P	7.328118	−15.026128	−5.593813
O	6.017664	−14.816078	−4.852992
O	8.036823	−16.345112	−5.359878
O	8.279884	−13.760055	−5.168645
C	9.688021	−13.863152	−4.832228
C	9.886958	−13.749193	−3.318088
O	9.660468	−12.390866	−2.838484
C	8.411947	−12.293100	−2.115779
C	8.913547	−14.648322	−2.545566
C	7.794827	−13.702729	−2.085021
O	9.608796	−15.306765	−1.443257
H	10.199688	−13.040932	−5.339772
H	10.089355	−14.820331	−5.179605
H	10.926059	−14.006380	−3.085602
H	8.630324	−11.915085	−1.114602
H	8.549916	−15.437821	−3.199774
H	6.952362	−13.781892	−2.772016
H	7.450485	−13.945713	−1.082022
N	7.528197	−11.298058	−2.726688
C	6.667365	−11.467178	−3.805220
N	6.011491	−10.366227	−4.122488
C	6.459056	−9.410524	−3.213433
C	6.123974	−8.050984	−3.021822
N	5.252958	−7.378540	−3.789815
N	6.732711	−7.403503	−1.989358
C	7.617242	−8.052687	−1.212906
N	8.013358	−9.333278	−1.315215
C	7.403415	−9.968222	−2.335641

H	6.571342	−12.422736	−4.293450
H	5.054795	−6.386841	−3.610093
H	4.776507	−7.859548	−4.539698
H	8.054737	−7.455858	−0.415854
P	8.746143	−16.395354	−0.569758
O	7.598364	−16.963141	−1.395761
O	9.727978	−17.394198	0.002283
O	8.063581	−15.483810	0.599438
C	8.940872	−14.698946	1.465209
C	8.148108	−13.606450	2.153948
O	7.657339	−12.660112	1.171109
C	6.547904	−11.964066	1.783362
C	6.916229	−14.062505	2.978761
C	5.819411	−13.028190	2.630668
O	7.312597	−14.063578	4.370134
H	9.740911	−14.252792	0.866109
H	9.383847	−15.355019	2.222613
H	8.835266	−13.090998	2.843342
H	6.909678	−11.131991	2.391399
H	6.604295	−15.069856	2.689205
H	5.021133	−13.496366	2.049041
H	5.378836	−12.592670	3.530670
H	6.635603	−14.551951	4.872246
N	5.738903	−11.371902	0.713993
C	5.463716	−9.995280	0.739753
O	5.910768	−9.228702	1.599133
N	4.633293	−9.563497	−0.275231
C	4.032215	−10.347836	−1.258434
O	3.245892	−9.830935	−2.078541
C	4.381765	−11.759735	−1.245330
C	3.801180	−12.675180	−2.287810
C	5.201785	−12.195203	−0.257555
H	4.386003	−8.540275	−0.248985
H	2.713717	−12.760327	−2.169997
H	4.236526	−13.675734	−2.213104
H	3.989890	−12.285260	−3.292653
H	5.494000	−13.235257	−0.177254
Na	6.412529	−16.737169	−3.362659
Na	8.506302	−19.222656	−1.002090
Na	13.177336	1.083202	2.364041
Na	4.910430	4.681930	0.343654

d(ATC)$^{Na+}$

E: −27607.21 kcal mol^{-1}

O	−0.100983	−0.260618	−0.000776
C	0.021203	0.022772	1.414076
C	1.483200	−0.036503	1.839478
O	1.984062	−1.413791	1.881381
C	2.951606	−1.650120	0.833635
C	2.451865	0.737720	0.926861
C	3.045543	−0.346057	0.020935
O	3.426123	1.393181	1.795371
H	−1.046975	−0.367738	−0.202132
H	−0.548990	−0.705476	2.004114
H	−0.358481	1.030573	1.636662
H	1.545939	0.353315	2.860609
H	3.903832	−1.929585	1.294995
H	1.944338	1.511556	0.346975
H	2.441584	−0.406969	−0.884229
H	4.067078	−0.119773	−0.273220
N	2.550195	−2.806254	0.024892
C	1.563741	−2.843054	−0.956321
N	1.419205	−4.039807	−1.500057
C	2.346257	−4.843735	−0.840920
C	2.668587	−6.215376	−0.972626
N	2.068234	−7.033047	−1.854220
N	3.628126	−6.716585	−0.143404
C	4.222403	−5.898892	0.747372
N	4.000349	−4.587679	0.947130
C	3.049581	−4.104962	0.121807
H	0.972365	−1.968462	−1.186550
H	2.383591	−8.005459	−1.957190
H	1.405625	−6.648623	−2.514775
H	4.983225	−6.366084	1.369441
P	4.797768	2.080588	1.217383
O	4.707488	2.398462	−0.260122
O	5.125701	3.232190	2.149665
O	5.891213	0.872702	1.374988
C	6.138866	0.364610	2.717298
C	7.202437	−0.713772	2.666320
O	6.681053	−1.886095	1.968066
C	7.603428	−2.284171	0.925800
C	8.512507	−0.310474	1.948052
C	8.418906	−1.028315	0.598845
O	9.606801	−0.772645	2.796308
H	5.211368	−0.051035	3.126411
H	6.480202	1.181994	3.363148
H	7.437431	−0.999187	3.698521
H	8.238846	−3.099715	1.278995
H	8.604959	0.771167	1.830259
H	7.882838	−0.376673	−0.094590
H	9.395407	−1.250101	0.172285
N	6.838153	−2.843935	−0.191880
C	6.885827	−4.235259	−0.402734
O	7.523290	−5.005550	0.317704
N	6.175960	−4.668922	−1.500524
C	5.398739	−3.902206	−2.355951
O	4.791470	−4.440363	−3.305547
C	5.353960	−2.476939	−2.061053
C	4.537488	−1.578487	−2.947635
C	6.067047	−2.027706	−0.996464
H	6.242732	−5.710903	−1.705094
H	4.870110	−1.659366	−3.989352
H	4.623477	−0.534358	−2.637312
H	3.479712	−1.866633	−2.929344
H	6.063770	−0.982531	−0.710888
P	11.171128	−0.817931	2.308409
O	11.418999	0.033356	1.080928
O	12.013467	−0.516889	3.534260
O	11.352495	−2.377468	1.849185
C	11.218396	−3.411651	2.868673
C	11.721042	−4.737512	2.325236
O	10.814636	−5.227803	1.291928
C	11.532537	−5.439235	0.054623
C	13.128610	−4.695189	1.694715
C	12.846750	−4.652617	0.187590
O	13.809901	−5.914259	2.094342
H	10.163791	−3.502879	3.152412
H	11.805030	−3.133347	3.751359
H	11.716461	−5.456611	3.154061
H	11.705295	−6.505137	−0.095967
H	13.697210	−3.822665	2.032252
H	12.713649	−3.614579	−0.127896
H	13.651890	−5.097120	−0.402759
H	14.695787	−5.902796	1.687836
N	10.673699	−5.015681	−1.061703
C	10.205860	−5.973605	−2.003591
O	10.556507	−7.169931	−1.889263
N	9.395028	−5.547021	−3.007046
C	9.029353	−4.253905	−3.106930
N	8.218442	−3.906198	−4.117629
C	9.476557	−3.277938	−2.156150
C	10.282705	−3.707268	−1.152382
H	7.860636	−4.618394	−4.773747
H	7.921809	−2.944486	−4.216868

H	9.169480	−2.241645	−2.227744
H	10.642515	−3.056082	−0.363715
O	5.284171	−12.153559	−10.433553
C	6.419648	−13.025858	−10.649486
C	7.157158	−13.273338	−9.338329
O	7.777639	−12.035921	−8.854869
C	7.203939	−11.668551	−7.572423
C	6.285391	−13.787229	−8.171613
C	5.922858	−12.495656	−7.439842
O	7.116603	−14.727536	−7.401936
H	4.939313	−11.898398	−11.306991
H	7.120586	−12.571799	−11.361584
H	6.092525	−13.996459	−11.050638
H	7.958543	−13.992454	−9.540038
H	7.909759	−11.906843	−6.776911
H	5.408266	−14.340485	−8.513974
H	5.105348	−12.018109	−7.984932
H	5.616187	−12.649912	−6.407690
N	7.018935	−10.231250	−7.492689
C	6.157499	−9.405299	−8.212329
N	6.210856	−8.147314	−7.809459
C	7.142893	−8.137274	−6.766101
C	7.604590	−7.086325	−5.919496
O	7.278776	−5.873704	−5.952142
N	8.523177	−7.548126	−4.953685
C	8.968228	−8.852165	−4.841391
N	9.875668	−9.129720	−3.873160
N	8.560338	−9.828688	−5.653838
C	7.656185	−9.425120	−6.562882
H	5.549670	−9.795121	−9.018249
H	8.850921	−6.840110	−4.255887
H	10.055657	−8.436577	−3.132940
H	9.943734	−10.114988	−3.623782
P	7.150110	−14.894076	−5.777870
O	5.786237	−14.748725	−5.137991
O	7.927868	−16.169596	−5.501681
O	7.997662	−13.582674	−5.276062
C	9.424398	−13.556781	−5.013391
C	9.683810	−13.485514	−3.505905
O	9.352177	−12.178291	−2.947425
C	8.092861	−12.233400	−2.227078
C	8.829248	−14.498850	−2.736432
C	7.609081	−13.690957	−2.266693
O	9.604596	−15.066366	−1.636542
H	9.825161	−12.664098	−5.501659
H	9.903483	−14.452619	−5.420952
H	10.751642	−13.648928	−3.328014
H	8.281517	−11.887539	−1.209067
H	8.558959	−15.320871	−3.394967
H	6.794054	−13.823549	−2.978257
H	7.266066	−14.007787	−1.283272
N	7.136366	−11.285739	−2.793538
C	6.264383	−11.446422	−3.868109
N	5.607473	−10.339767	−4.165034
C	6.069419	−9.390578	−3.254120
C	5.781567	−8.017614	−3.068632
N	4.923443	−7.317637	−3.826680
N	6.431642	−7.382262	−2.054242
C	7.306859	−8.051156	−1.283388
N	7.660639	−9.341753	−1.382191
C	7.016724	−9.961623	−2.389866
H	6.158785	−12.396466	−4.368000
H	4.824986	−6.305955	−3.684420
H	4.466749	−7.765475	−4.609351
H	7.773899	−7.462346	−0.497539
P	8.898554	−16.266852	−0.771390
O	7.867392	−17.006068	−1.614938
O	10.007933	−17.101377	−0.170484
O	8.059124	−15.465557	0.376194
C	8.805784	−14.640603	1.322239
C	7.888685	−13.646037	2.003405
O	7.432158	−12.643245	1.061589
C	6.341334	−11.945189	1.712212
C	6.599674	−14.205001	2.638052
C	5.628500	−12.997553	2.602217
O	6.920339	−14.680865	3.961247
H	9.601854	−14.103532	0.796063
H	9.254775	−15.288088	2.082816
H	8.478768	−13.160787	2.798455
H	6.730359	−11.112569	2.300660
H	6.214314	−15.031051	2.032890
H	4.654656	−13.270380	2.190182
H	5.477089	−12.603424	3.610268
H	6.193734	−15.256216	4.259678
N	5.498512	−11.347965	0.672233
C	5.217717	−9.972993	0.723648
O	5.692633	−9.212958	1.573646
N	4.348884	−9.534063	−0.255677
C	3.709757	−10.311971	−1.219605
O	2.893789	−9.788513	−2.006110
C	4.058140	−11.724835	−1.228214
C	3.435047	−12.633109	−2.252125
C	4.921971	−12.166153	−0.280741
H	4.112438	−8.509455	−0.220666
H	2.351480	−12.709435	−2.097604
H	3.865348	−13.636918	−2.193080
H	3.592135	−12.241991	−3.262207
H	5.222588	−13.206115	−0.222119
Na	13.122830	1.274416	2.310939
Na	5.185780	4.728473	0.211369
Na	6.756366	−17.018113	−3.626025
Na	9.079302	−19.108583	−1.147337

d(ATG)$^{Na+}$

E: −27600.96 kcal mol^{-1}

O	−0.127376	−0.391970	−0.261890
C	−0.110272	−0.114701	1.159749
C	1.318346	−0.138539	1.690017
O	1.852690	−1.500916	1.767705
C	2.896085	−1.708986	0.787108
C	2.336220	0.662105	0.857930
C	3.007234	−0.401699	−0.018953
O	3.237330	1.308399	1.809076
H	−1.055335	−0.510675	−0.529713
H	−0.701174	−0.861124	1.705308
H	−0.531630	0.881201	1.359666
H	1.293654	0.249039	2.713711
H	3.824664	−1.960089	1.308708
H	1.862966	1.441147	0.256374
H	2.456035	−0.474099	−0.956977
H	4.040779	−0.153523	−0.250700
N	2.580627	−2.879694	−0.036585
C	1.637210	−2.967703	−1.055237
N	1.561317	−4.177136	−1.586121
C	2.491932	−4.935577	−0.878495
C	2.868789	−6.297067	−0.965362
N	2.335849	−7.162203	−1.845153
N	3.812710	−6.741263	−0.087877
C	4.339389	−5.887103	0.810501
N	4.056970	−4.583730	0.975099
C	3.125645	−4.155091	0.100102
H	1.023887	−2.118821	−1.322280
H	2.674224	−8.130909	−1.885729
H	1.685515	−6.826506	−2.543035
H	5.092489	−6.314873	1.468758

P	4.623756	2.048632	1.343981
O	4.583520	2.488327	−0.104329
O	4.898360	3.121601	2.381815
O	5.725816	0.843342	1.442187
C	5.937392	0.232426	2.746892
C	6.999213	−0.843205	2.642196
O	6.488789	−1.965937	1.860714
C	7.483855	−2.346926	0.874951
C	8.325814	−0.399095	1.974803
C	8.271145	−1.064790	0.597426
O	9.398166	−0.886028	2.840220
H	4.998524	−0.207682	3.101328
H	6.264654	0.996650	3.461587
H	7.218357	−1.192419	3.658595
H	8.126268	−3.136286	1.271987
H	8.414464	0.687028	1.904475
H	7.718462	−0.402790	−0.073867
H	9.254824	−1.244976	0.167962
N	6.804617	−2.937732	−0.275006
C	6.928257	−4.324765	−0.482312
O	7.585818	−5.062235	0.253151
N	6.249264	−4.797231	−1.584681
C	5.446996	−4.070855	−2.452978
O	4.856345	−4.643256	−3.393582
C	5.342663	−2.646297	−2.172798
C	4.498358	−1.786084	−3.071912
C	6.018463	−2.160738	−1.100657
H	6.358127	−5.835001	−1.764374
H	4.857149	−1.839300	−4.106912
H	4.522386	−0.741371	−2.751025
H	3.457250	−2.129306	−3.077181
H	5.965461	−1.114754	−0.823668
P	10.964023	−1.013434	2.379187
O	11.289847	−0.148222	1.179992
O	11.794744	−0.789887	3.629895
O	11.069152	−2.567371	1.882968
C	10.939198	−3.631551	2.870930
C	11.679409	−4.877920	2.405455
O	10.967873	−5.542580	1.318672
C	11.720072	−5.452303	0.084326
C	13.104044	−4.621615	1.884548
C	12.909078	−4.515155	0.365804
O	13.909247	−5.771593	2.257095
H	9.877087	−3.862329	3.009328
H	11.364671	−3.297826	3.823630
H	11.708867	−5.575599	3.250906
H	12.042871	−6.457055	−0.202958
H	13.534499	−3.708703	2.309432
H	12.658154	−3.485086	0.104916
H	13.800582	−4.811581	−0.192143
H	14.800513	−5.639593	1.885285
N	10.843006	−4.999916	−0.988587
C	10.299017	−3.728086	−1.163371
N	9.492905	−3.657786	−2.205015
C	9.489450	−4.945012	−2.747841
C	8.810468	−5.482448	−3.880551
O	8.025314	−4.889031	−4.658515
N	9.113362	−6.844368	−4.081255
C	9.957604	−7.595868	−3.290224
N	10.166716	−8.879846	−3.659752
N	10.581177	−7.098854	−2.215409
C	10.318381	−5.793854	−1.998813
H	10.529637	−2.926843	−0.476683
H	8.613904	−7.317313	−4.870849
H	9.595749	−9.323228	−4.397823
H	10.605638	−9.480767	−2.973099
O	5.445167	−11.847187	−10.498322
C	6.603057	−12.677954	−10.760901
C	7.286938	−13.065869	−9.455839

O	7.916284	−11.900194	−8.829756
C	7.304703	−11.633430	−7.538971
C	6.366582	−13.670679	−8.376059
C	6.015906	−12.460062	−7.510158
O	7.160912	−14.693904	−7.686806
H	5.149779	−11.475030	−11.347585
H	7.326050	−12.139536	−11.386507
H	6.305367	−13.599271	−11.282380
H	8.080635	−13.780328	−9.700023
H	7.992208	−11.923445	−6.745313
H	5.483569	−14.157386	−8.796290
H	5.196043	−11.926210	−7.994939
H	5.701737	−12.725056	−6.502996
N	7.116505	−10.187943	−7.376940
C	7.892749	−9.491038	−6.409704
O	8.763430	−10.113989	−5.762437
N	7.652638	−8.167439	−6.222693
C	6.738816	−7.512704	−6.967243
N	6.544591	−6.208744	−6.719358
C	6.003570	−8.182257	−8.001694
C	6.226159	−9.511191	−8.170437
H	7.052185	−5.736550	−5.955025
H	5.849640	−5.695529	−7.245608
H	5.301546	−7.647354	−8.630792
H	5.738597	−10.098390	−8.941902
P	6.847363	−15.241487	−6.178824
O	5.382118	−15.133815	−5.812161
O	7.481219	−16.616131	−6.080073
O	7.632755	−14.175597	−5.216779
C	9.082884	−14.203796	−5.092782
C	9.496588	−14.003237	−3.637464
O	9.363111	−12.615428	−3.204390
C	8.232507	−12.456977	−2.316830
C	8.678467	−14.831920	−2.633998
C	7.597839	−13.852684	−2.155128
O	9.598038	−15.247017	−1.577629
H	9.504892	−13.404439	−5.710767
H	9.461092	−15.171738	−5.437212
H	10.559417	−14.255728	−3.560531
H	8.586341	−12.049563	−1.366807
H	8.246782	−15.727615	−3.086052
H	6.720470	−13.961407	−2.793367
H	7.306025	−14.037780	−1.122848
N	7.311256	−11.452155	−2.852255
C	6.453903	−11.589022	−3.938009
N	5.810715	−10.473718	−4.234440
C	6.269995	−9.541891	−3.306437
C	5.976925	−8.171748	−3.113413
N	5.120710	−7.476541	−3.877148
N	6.610433	−7.540953	−2.085537
C	7.478621	−8.219250	−1.313275
N	7.836899	−9.509762	−1.418836
C	7.206492	−10.126345	−2.438149
H	6.378308	−12.526685	−4.465935
H	4.996734	−6.466284	−3.730502
H	4.677897	−7.930083	−4.664117
H	7.934653	−7.638421	−0.514896
P	9.065974	−16.098020	−0.281101
O	7.834385	−16.916271	−0.611784
O	10.270982	−16.848986	0.253695
O	8.586235	−14.933078	0.762373
C	9.587426	−13.997190	1.255175
C	8.911589	−12.898863	2.050301
O	8.124938	−12.068343	1.150954
C	6.981758	−11.589768	1.896827
C	7.957940	−13.369310	3.185738
C	6.593734	−12.768405	2.800649
O	8.482640	−12.834362	4.428250
H	10.131592	−13.561330	0.409730

H	10.298153	−14.526988	1.899790
H	9.701140	−12.284353	2.506713
H	7.248688	−10.705167	2.480135
H	7.913056	−14.460169	3.246335
H	6.010365	−13.505160	2.240842
H	6.016853	−12.454776	3.673987
H	7.964642	−13.221965	5.157147
N	5.962635	−11.152419	0.943015
C	5.600258	−9.795913	0.909820
O	6.064132	−8.949318	1.680727
N	4.668292	−9.478747	−0.055847
C	4.060206	−10.350385	−0.954301
O	3.207009	−9.923669	−1.761452
C	4.485102	−11.738404	−0.874139
C	3.880828	−12.749474	−1.808960
C	5.408879	−12.062473	0.064819
H	4.375451	−8.465416	−0.083999
H	2.800703	−12.839741	−1.639946
H	4.335676	−13.733218	−1.663842
H	4.018751	−12.445687	−2.852073
H	5.776196	−13.075426	0.172190
Na	13.048967	0.936487	2.506256
Na	4.998886	4.781465	0.596737
Na	5.377240	−17.503508	−5.265817
Na	8.996406	−18.939995	0.019066

d(CAA)$^{Na+}$

E: −27599.11 kcal mol^{-1}

O	0.021698	0.017601	−1.215840
C	−0.053507	0.452122	0.164323
C	1.259898	0.169187	0.882280
O	1.456496	−1.270560	1.067815
C	2.641857	−1.713457	0.352228
C	2.528970	0.673152	0.166876
C	3.012102	−0.564391	−0.590989
O	3.441188	1.120101	1.223827
H	−0.875822	0.062454	−1.589283
H	−0.859666	−0.075863	0.689081
H	−0.249161	1.532966	0.217142
H	1.196524	0.626344	1.875872
H	3.437692	−1.929171	1.064784
H	2.335075	1.520985	−0.494058
H	2.452828	−0.627359	−1.526739
H	4.072821	−0.537369	−0.828541
N	2.357039	−2.991745	−0.309511
C	2.982557	−4.178822	0.166805
O	3.743996	−4.112427	1.156714
N	2.727065	−5.346228	−0.480070
C	1.878529	−5.392175	−1.526966
N	1.685507	−6.575336	−2.128545
C	1.189171	−4.217511	−1.978847
C	1.452223	−3.049607	−1.337873
H	2.206501	−7.412916	−1.825703
H	1.075199	−6.635555	−2.933018
H	0.474237	−4.261211	−2.792412
H	0.953656	−2.117360	−1.582887
P	5.050000	1.322595	1.010177
O	5.409393	1.604480	−0.434109
O	5.492349	2.346278	2.039025
O	5.667121	−0.150927	1.358223
C	5.706597	−0.620394	2.735945
C	6.967360	−1.441617	2.982805
O	6.890618	−2.769286	2.382189
C	7.744957	−2.856497	1.217184
C	8.255457	−0.811949	2.426736
C	8.407425	−1.476868	1.053251
O	9.321791	−1.144487	3.369449
H	4.821953	−1.237483	2.926694
H	5.704891	0.238813	3.414968
H	7.054868	−1.588234	4.064670
H	8.474051	−3.654619	1.383976
H	8.192175	0.276118	2.350085
H	7.878145	−0.870672	0.317979
H	9.447066	−1.558259	0.744321
N	6.968148	−3.279955	0.053064
C	6.129421	−2.515127	−0.747480
N	5.517291	−3.224237	−1.681915
C	5.966239	−4.529236	−1.488615
C	5.683471	−5.743416	−2.157474
N	4.834989	−5.850753	−3.191268
N	6.334006	−6.857532	−1.719051
C	7.185926	−6.774580	−0.680327
N	7.502941	−5.684914	0.035625
C	6.865484	−4.586910	−0.412655
H	6.006332	−1.457079	−0.576296
H	4.643914	−6.768777	−3.614201
H	4.335391	−5.032934	−3.511440
H	7.666439	−7.709871	−0.403272
P	10.906782	−0.947418	3.005861
O	11.122680	0.128109	1.960973
O	11.624800	−0.771906	4.331332
O	11.308245	−2.369886	2.306078
C	11.249029	−3.576962	3.119254
C	11.784435	−4.754861	2.325596
O	10.852329	−5.091885	1.251313
C	11.575250	−5.185025	−0.000425
C	13.162656	−4.522926	1.664825
C	12.816947	−4.303708	0.186952
O	13.945552	−5.728327	1.874966
H	10.211066	−3.767243	3.415317
H	11.857905	−3.441608	4.020518
H	11.853879	−5.611609	3.008135
H	11.841341	−6.228451	−0.201278
H	13.680000	−3.661906	2.098948
H	12.563957	−3.251799	0.029257
H	13.632333	−4.579772	−0.485938
H	14.820382	−5.585333	1.469305
N	10.694319	−4.779781	−1.083239
C	10.231719	−3.499219	−1.358854
N	9.391977	−3.459732	−2.378711
C	9.287930	−4.783687	−2.803331
C	8.541488	−5.397026	−3.836745
N	7.729490	−4.723740	−4.674213
N	8.670710	−6.745774	−3.985117
C	9.469001	−7.436268	−3.150465
N	10.211185	−6.956696	−2.137548
C	10.085953	−5.621425	−2.008337
H	10.551439	−2.647874	−0.776348
H	7.105976	−5.249106	−5.300640
H	7.528739	−3.750589	−4.481940
H	9.496306	−8.510689	−3.316949
O	5.752713	−11.530183	−10.612794
C	7.003570	−12.222427	−10.849892
C	7.560724	−12.766869	−9.540428
O	8.073730	−11.686351	−8.694586
C	7.327295	−11.597271	−7.456632
C	6.549834	−13.543650	−8.674325
C	6.112320	−12.523070	−7.620284
O	7.263506	−14.691095	−8.116094
H	5.510797	−11.064552	−11.432240
H	7.741781	−11.542045	−11.292361
H	6.848731	−13.067613	−11.535839
H	8.406124	−13.418317	−9.786755
H	7.964181	−11.896137	−6.622111
H	5.704812	−13.918115	−9.255732

H	5.247955	−11.977547	−8.001556
H	5.832679	−12.995681	−6.681761
N	6.986308	−10.185994	−7.198767
C	7.642320	−9.509736	−6.156068
O	8.487474	−10.038334	−5.427646
N	7.252889	−8.195313	−5.994497
C	6.346665	−7.485446	−6.774077
O	6.114236	−6.282004	−6.524997
C	5.742059	−8.226724	−7.868186
C	4.765045	−7.529228	−8.774567
C	6.104704	−9.524366	−8.035339
H	7.749464	−7.669284	−5.227418
H	5.223738	−6.650372	−9.244168
H	3.895527	−7.173020	−8.207882
H	4.414875	−8.203546	−9.561429
H	5.736128	−10.117853	−8.866063
P	6.578498	−15.673772	−6.994373
O	5.066663	−15.592292	−7.012954
O	7.197409	−17.045118	−7.195504
O	7.071590	−15.013891	−5.580303
C	8.502756	−14.993301	−5.308954
C	8.764669	−14.409693	−3.933287
O	8.436572	−12.988122	−3.926451
C	7.559873	−12.698831	−2.806991
C	7.968071	−15.053613	−2.773259
C	6.869187	−14.027296	−2.488556
O	8.919574	−15.233171	−1.676585
H	9.007949	−14.383584	−6.066836
H	8.898168	−16.014833	−5.350914
H	9.836689	−14.522238	−3.730109
H	8.142795	−12.335840	−1.957864
H	7.563372	−16.033550	−3.035331
H	6.044374	−14.220299	−3.178149
H	6.485190	−14.079230	−1.471725
N	6.675717	−11.593579	−3.172201
C	6.916697	−10.328918	−2.600095
O	7.814834	−10.117614	−1.783753
N	6.055110	−9.338887	−3.023457
C	5.029520	−9.458888	−3.951758
O	4.353757	−8.462143	−4.281655
C	4.834021	−10.788921	−4.507307
C	3.722630	−11.002192	−5.496957
C	5.671409	−11.776154	−4.099165
H	6.186328	−8.397164	−2.558735
H	2.755859	−10.737650	−5.052014
H	3.679131	−12.044494	−5.822613
H	3.853865	−10.361610	−6.377101
H	5.597806	−12.780956	−4.496932
P	8.476879	−15.495062	−0.122007
O	7.084270	−16.079285	−0.009296
O	9.605084	−16.288687	0.511278
O	8.407143	−13.985372	0.501011
C	9.648221	−13.254130	0.725505
C	9.499523	−12.307088	1.909249
O	8.688833	−11.144015	1.563704
C	7.429593	−11.166159	2.278760
C	8.836814	−12.930296	3.150218
C	7.363853	−12.522837	3.005207
O	9.462088	−12.330817	4.315933
H	9.893603	−12.682906	−0.176791
H	10.454964	−13.964208	0.936133
H	10.501482	−11.940738	2.162577
H	7.409014	−10.321862	2.973534
H	8.961693	−14.017965	3.175295
H	6.843433	−13.264263	2.396254
H	6.855920	−12.442552	3.969266
H	9.011125	−12.682880	5.105128
N	6.335099	−10.933640	1.343800
C	5.828306	−11.815890	0.390190
N	4.881912	−11.275477	−0.352256
C	4.757712	−9.965898	0.116330
C	3.904001	−8.901164	−0.297841
O	3.047912	−8.916026	−1.213732
N	4.111035	−7.732605	0.462222
C	5.025570	−7.602827	1.485873
N	5.060082	−6.412855	2.133474
N	5.834517	−8.596653	1.870666
C	5.659819	−9.733900	1.165276
H	6.218389	−12.818719	0.297646
H	3.579218	−6.883499	0.157823
H	4.602544	−5.582202	1.721910
H	5.881057	−6.249338	2.704502
Na	4.956686	−17.981302	−7.442305
Na	8.024923	−17.976061	1.215265
Na	12.570399	1.322189	3.506199
Na	6.510088	3.658988	0.276981

d(CAC)$^{Na+}$

E: −27511.59 kcal mol^{-1}

O	0.122024	−0.013924	−1.158560
C	0.032007	0.383041	0.231934
C	1.340646	0.087561	0.953527
O	1.542468	−1.355385	1.104482
C	2.733710	−1.776911	0.386487
C	2.612842	0.616281	0.261577
C	3.112292	−0.600521	−0.519643
O	3.509983	1.051893	1.336232
H	−0.771990	0.037943	−1.539448
H	−0.775904	−0.162901	0.735161
H	−0.169792	1.461081	0.311793
H	1.266987	0.518628	1.957924
H	3.522781	−2.014472	1.099580
H	2.417898	1.475969	−0.383555
H	2.566709	−0.640535	−1.464606
H	4.175771	−0.562606	−0.743109
N	2.454762	−3.035719	−0.315418
C	3.073887	−4.237021	0.131932
O	3.826165	−4.201223	1.130700
N	2.823042	−5.385041	−0.550547
C	1.984818	−5.399703	−1.606758
N	1.793394	−6.564831	−2.242878
C	1.305891	−4.210075	−2.034396
C	1.563081	−3.061962	−1.356647
H	2.269841	−7.424829	−1.930666
H	1.177413	−6.603977	−3.044284
H	0.599751	−4.228731	−2.856532
H	1.068138	−2.122496	−1.580187
P	5.123574	1.245184	1.159084
O	5.519527	1.545349	−0.271598
O	5.552544	2.248016	2.213894
O	5.720746	−0.238601	1.498291
C	5.714548	−0.735939	2.866854
C	6.973984	−1.551193	3.138156
O	6.924395	−2.870159	2.516926
C	7.816794	−2.940596	1.380275
C	8.267873	−0.897236	2.627354
C	8.496780	−1.562501	1.263359
O	9.311050	−1.205557	3.603717
H	4.828762	−1.363201	3.013219
H	5.682210	0.109215	3.562406
H	7.033510	−1.712753	4.219802
H	8.536461	−3.744195	1.550603
H	8.183544	0.188376	2.544392
H	8.025692	−0.953510	0.491291
H	9.555461	−1.664662	1.031758

N	7.074720	−3.332467	0.181656
C	6.222426	−2.544900	−0.583426
N	5.632934	−3.215804	−1.558174
C	6.110198	−4.518086	−1.431706
C	5.845362	−5.700475	−2.160928
N	5.002546	−5.761523	−3.202968
N	6.499447	−6.830817	−1.770912
C	7.353192	−6.785703	−0.731371
N	7.663148	−5.726219	0.032586
C	7.007404	−4.614855	−0.356261
H	6.073403	−1.502270	−0.350618
H	4.779799	−6.667953	−3.634991
H	4.481201	−4.936756	−3.466233
H	7.835020	−7.730886	−0.490881
P	10.855282	−0.708724	3.371620
O	10.922002	0.564216	2.551926
O	11.499913	−0.680667	4.744649
O	11.499592	−1.889416	2.440271
C	11.573729	−3.239042	2.981814
C	11.971889	−4.207342	1.886210
O	10.885582	−4.327287	0.927369
C	11.485997	−4.627405	−0.355990
C	13.248841	−3.836122	1.078219
C	12.762546	−3.774699	−0.382649
O	14.218638	−4.889804	1.311376
H	10.599465	−3.525772	3.393280
H	12.320659	−3.270550	3.783458
H	12.146345	−5.186579	2.356190
H	11.710246	−5.693630	−0.431438
H	13.664108	−2.877970	1.402167
H	12.526001	−2.740860	−0.651697
H	13.508361	−4.157197	−1.083837
H	15.062664	−4.605247	0.915659
N	10.506179	−4.346337	−1.404510
C	10.020812	−5.405217	−2.226014
O	10.450747	−6.566940	−2.045865
N	9.111411	−5.106754	−3.188167
C	8.654466	−3.848504	−3.354150
N	7.763312	−3.627648	−4.329732
C	9.109403	−2.774837	−2.518993
C	10.031320	−3.071721	−1.570569
H	7.422756	−4.401646	−4.921009
H	7.360248	−2.706647	−4.440970
H	8.733411	−1.766174	−2.639195
H	10.427703	−2.327312	−0.892547
O	5.945332	−11.465849	−10.713069
C	7.173002	−12.221522	−10.852412
C	7.585710	−12.821001	−9.513488
O	8.098009	−11.802902	−8.592414
C	7.187709	−11.601969	−7.482510
C	6.461626	−13.543307	−8.749873
C	5.959895	−12.482678	−7.765560
O	7.072571	−14.707889	−8.112618
H	5.796823	−10.986278	−11.546781
H	7.983511	−11.576706	−11.214714
H	7.034693	−13.046758	−11.566226
H	8.408050	−13.517815	−9.706108
H	7.697042	−11.878731	−6.554330
H	5.665714	−13.895419	−9.409932
H	5.174854	−11.904652	−8.254701
H	5.547826	−12.919010	−6.858606
N	6.877084	−10.179706	−7.350105
C	6.083310	−9.402686	−8.197719
N	6.067478	−8.129411	−7.851312
C	6.895426	−8.046767	−6.730618
C	7.271189	−6.927790	−5.931404
O	6.909697	−5.732555	−6.065765
N	8.151963	−7.290269	−4.896820
C	8.637742	−8.564624	−4.680750

N	9.534917	−8.715520	−3.683615
N	8.275833	−9.620236	−5.419815
C	7.418810	−9.308698	−6.411543
H	5.592051	−9.839109	−9.056077
H	8.475213	−6.520049	−4.267465
H	9.767605	−7.936644	−3.051157
H	9.712378	−9.661135	−3.368906
P	6.309085	−15.592585	−6.964585
O	4.804864	−15.424174	−7.012405
O	6.846200	−17.006748	−7.089337
O	6.818972	−14.896397	−5.575041
C	8.241569	−14.966100	−5.269413
C	8.488317	−14.489615	−3.850483
O	8.260189	−13.051340	−3.758437
C	7.300172	−12.771049	−2.707373
C	7.594342	−15.148635	−2.775201
C	6.535701	−14.079825	−2.490398
O	8.469463	−15.444636	−1.642804
H	8.795813	−14.334739	−5.973375
H	8.586899	−16.001577	−5.368774
H	9.540073	−14.688858	−3.612936
H	7.823109	−12.455529	−1.802085
H	7.151569	−16.086120	−3.118620
H	5.733566	−14.199663	−3.221551
H	6.104127	−14.161280	−1.494997
N	6.491766	−11.618683	−3.108723
C	6.758572	−10.380378	−2.494940
O	7.609820	−10.232966	−1.616423
N	5.987673	−9.335734	−2.959643
C	5.022276	−9.384923	−3.957220
O	4.429939	−8.344552	−4.312737
C	4.787706	−10.693940	−4.545110
C	3.727937	−10.830187	−5.602604
C	5.547314	−11.731057	−4.108801
H	6.184265	−8.395606	−2.515494
H	2.759201	−10.488183	−5.219237
H	3.623442	−11.868996	−5.924669
H	3.961401	−10.211296	−6.476862
H	5.454174	−12.721655	−4.537950
P	7.930196	−15.833960	−0.146377
O	6.483140	−16.280928	−0.149127
O	8.943376	−16.802798	0.436116
O	7.981205	−14.399904	0.637220
C	9.280088	−13.782242	0.872715
C	9.181816	−12.756456	1.991959
O	8.460253	−11.566907	1.551810
C	7.215741	−11.426263	2.279516
C	8.462012	−13.248832	3.259886
C	7.029848	−12.730781	3.075671
O	9.128540	−12.635534	4.395597
H	9.617577	−13.292730	−0.047902
H	10.004091	−14.554452	1.156375
H	10.204315	−12.447801	2.241229
H	7.285349	−10.550164	2.930672
H	8.496882	−14.339674	3.349767
H	6.455857	−13.457405	2.497209
H	6.520463	−12.557665	4.026799
H	8.652567	−12.911547	5.200216
N	6.136559	−11.139487	1.343262
C	5.553199	−12.017341	0.430478
N	4.639417	−11.436697	−0.322366
C	4.615381	−10.105641	0.099576
C	3.840956	−8.996516	−0.350643
O	2.982606	−8.978685	−1.265498
N	4.138260	−7.820250	0.365091
C	5.064272	−7.722263	1.382863
N	5.176230	−6.523124	1.998037
N	5.812207	−8.755378	1.789584
C	5.548008	−9.901243	1.128127

H	5.864830	−13.050194	0.375363
H	3.644840	−6.950073	0.055176
H	4.716749	−5.681011	1.614391
H	5.995248	−6.391750	2.578797
Na	4.554522	−17.822340	−7.306738
Na	7.148543	−18.385701	0.872593
Na	12.124491	1.661462	4.354198
Na	6.636588	3.574570	0.518148

d(CAG)$^{Na+}$

E: −27509.49 kcal mol^{-1}

O	0.098235	−0.032799	−1.219475
C	0.009916	0.368946	0.169711
C	1.321644	0.082401	0.889463
O	1.531996	−1.358983	1.041338
C	2.725980	−1.772851	0.322357
C	2.590386	0.617712	0.195470
C	3.092732	−0.596734	−0.588061
O	3.486601	1.059825	1.269044
H	−0.797332	0.011768	−1.597605
H	−0.793962	−0.179237	0.677020
H	−0.197295	1.446231	0.246299
H	1.246889	0.514406	1.893417
H	3.518799	−1.998684	1.034476
H	2.390238	1.477171	−0.448404
H	2.540868	−0.639495	−1.529229
H	4.154905	−0.554566	−0.817615
N	2.458377	−3.038136	−0.370618
C	3.078787	−4.231587	0.096911
O	3.822425	−4.179447	1.101041
N	2.837221	−5.389193	−0.572387
C	2.006544	−5.420759	−1.634009
N	1.825058	−6.595137	−2.255866
C	1.324082	−4.239814	−2.080300
C	1.572383	−3.081761	−1.415867
H	2.317445	−7.443858	−1.937520
H	1.222190	−6.646663	−3.066418
H	0.622970	−4.272090	−2.906293
H	1.074422	−2.147648	−1.654192
P	5.107509	1.202924	1.116442
O	5.535273	1.511973	−0.303383
O	5.551193	2.176333	2.192541
O	5.653940	−0.303325	1.439720
C	5.630308	−0.818274	2.801144
C	6.896513	−1.617913	3.086918
O	6.885726	−2.925891	2.443396
C	7.789877	−2.951858	1.313241
C	8.192243	−0.936748	2.618238
C	8.450967	−1.562581	1.240141
O	9.215333	−1.262760	3.610195
H	4.752876	−1.462588	2.920597
H	5.570288	0.016156	3.507633
H	6.933373	−1.797404	4.166804
H	8.519446	−3.748805	1.472296
H	8.099617	0.150258	2.563969
H	7.980491	−0.939242	0.479248
H	9.513846	−1.643958	1.017945
N	7.067860	−3.330001	0.098097
C	6.231073	−2.547092	−0.687478
N	5.641550	−3.229632	−1.655293
C	6.102735	−4.534844	−1.502041
C	5.836564	−5.729090	−2.211746
N	5.000544	−5.812094	−3.258482
N	6.487874	−6.853091	−1.798438
C	7.325437	−6.797745	−0.745968
N	7.626407	−5.728887	0.006576
C	6.987789	−4.620781	−0.415872
H	6.092826	−1.497821	−0.479400
H	4.781621	−6.727281	−3.675341
H	4.487751	−4.990949	−3.548151
H	7.801663	−7.740899	−0.487461
P	10.775861	−0.810484	3.402870
O	10.892509	0.451367	2.571616
O	11.394680	−0.783036	4.787862
O	11.402713	−2.021706	2.499065
C	11.439381	−3.362041	3.067470
C	11.895323	−4.353308	2.014666
O	10.847001	−4.521045	1.014192
C	11.490497	−4.687934	−0.273198
C	13.192976	−3.967801	1.252019
C	12.720101	−3.772477	−0.196629
O	14.116694	−5.078804	1.393656
H	10.442081	−3.637106	3.428941
H	12.140543	−3.381445	3.909868
H	12.066025	−5.316014	2.516729
H	11.774775	−5.736386	−0.420721
H	13.644526	−3.058640	1.658491
H	12.419058	−2.731412	−0.346901
H	13.488653	−4.030204	−0.929360
H	14.962740	−4.809779	0.991234
N	10.545709	−4.371564	−1.325937
C	10.028583	−3.116150	−1.650788
N	9.213489	−3.153507	−2.685307
C	9.183418	−4.493176	−3.075112
C	8.483280	−5.137782	−4.136783
O	7.707410	−4.613345	−4.971929
N	8.743633	−6.519539	−4.179466
C	9.572546	−7.196197	−3.308758
N	9.732092	−8.521674	−3.522399
N	10.228750	−6.594520	−2.308271
C	10.001434	−5.267897	−2.239178
H	10.292445	−2.232276	−1.089116
H	8.210924	−7.076017	−4.888780
H	9.141023	−9.021195	−4.204699
H	10.097705	−9.060964	−2.747031
O	5.692679	−11.530320	−10.721540
C	6.945515	−12.230049	−10.924556
C	7.474203	−12.762189	−9.598117
O	7.990212	−11.680053	−8.757304
C	7.219932	−11.555518	−7.534608
C	6.438842	−13.513781	−8.739348
C	6.000477	−12.475219	−7.702661
O	7.126844	−14.667753	−8.163516
H	5.473611	−11.065996	−11.548249
H	7.695194	−11.558160	−11.360533
H	6.800305	−13.082712	−11.603395
H	8.313773	−13.429002	−9.822404
H	7.838475	−11.838858	−6.682020
H	5.596356	−13.880557	−9.329387
H	5.144833	−11.927741	−8.100968
H	5.703854	−12.933230	−6.762106
N	6.888124	−10.134221	−7.319835
C	7.529657	−9.403189	−6.277374
O	8.332571	−9.992471	−5.520572
N	7.235696	−8.083060	−6.138818
C	6.365565	−7.471429	−6.966774
N	6.103642	−6.172238	−6.761321
C	5.745580	−8.178713	−8.049517
C	6.050805	−9.492067	−8.197790
H	6.625136	−5.632926	−6.054264
H	5.494964	−5.679142	−7.401728
H	5.078715	−7.677878	−8.741840
H	5.677210	−10.091146	−9.021587
P	6.422923	−15.622455	−7.030971
O	4.913526	−15.501532	−7.038701

O	7.002733	−17.012139	−7.223894
O	6.944405	−14.964367	−5.627031
C	8.377641	−14.982922	−5.367132
C	8.660455	−14.453302	−3.973660
O	8.378871	−13.023180	−3.917125
C	7.477729	−12.745026	−2.813843
C	7.841178	−15.110546	−2.838321
C	6.761840	−14.068936	−2.533737
O	8.779143	−15.342487	−1.740076
H	8.889267	−14.357740	−6.107890
H	8.750654	−16.010615	−5.447513
H	9.728454	−14.605710	−3.776030
H	8.045630	−12.403550	−1.945897
H	7.417063	−16.073453	−3.131167
H	5.935975	−14.229453	−3.230193
H	6.373034	−14.138523	−1.519827
N	6.618763	−11.619978	−3.180182
C	6.876194	−10.369500	−2.584791
O	7.749437	−10.196224	−1.733497
N	6.063734	−9.348607	−3.030717
C	5.053576	−9.431824	−3.979296
O	4.413937	−8.413103	−4.314635
C	4.824700	−10.753704	−4.541296
C	3.715169	−10.930061	−5.540472
C	5.629078	−11.766757	−4.129248
H	6.237284	−8.406517	−2.582997
H	2.755627	−10.627413	−5.104165
H	3.634765	−11.971569	−5.861316
H	3.875713	−10.299487	−6.422960
H	5.535763	−12.765765	−4.537801
P	8.319644	−15.656022	−0.200289
O	6.907110	−16.195606	−0.115615
O	9.416521	−16.513590	0.404048
O	8.299953	−14.171362	0.484743
C	9.565180	−13.476263	0.687417
C	9.454335	−12.497153	1.848307
O	8.669506	−11.323367	1.480990
C	7.424756	−11.287992	2.220734
C	8.792649	−13.070552	3.113544
C	7.330005	−12.625364	2.978343
O	9.451059	−12.459728	4.255140
H	9.826328	−12.934643	−0.228780
H	10.349454	−14.207529	0.911371
H	10.468808	−12.151416	2.080277
H	7.445313	−10.428815	2.897103
H	8.888086	−14.160309	3.164680
H	6.781550	−13.365067	2.392236
H	6.836788	−12.509378	3.946399
H	9.001926	−12.778968	5.059182
N	6.319891	−11.040932	1.302295
C	5.766081	−11.928684	0.380612
N	4.824637	−11.374995	−0.358234
C	4.752105	−10.051256	0.080330
C	3.932022	−8.967431	−0.350580
O	3.066821	−8.972565	−1.258544
N	4.188667	−7.789218	0.378524
C	5.116943	−7.668177	1.391363
N	5.188253	−6.471166	2.020897
N	5.903944	−8.677430	1.782564
C	5.681355	−9.824083	1.106623
H	6.119469	−12.946589	0.306698
H	3.673718	−6.931939	0.067580
H	4.721390	−5.639583	1.622477
H	6.020841	−6.312969	2.575944
Na	4.741459	−17.898230	−7.430971
Na	7.761097	−18.169801	1.032794
Na	12.063819	1.552696	4.375811
Na	6.723583	3.478759	0.539997

d(CCA)$^{Na+}$

E: −27509.34 kcal mol^{-1}

O	−0.112000	0.256539	−0.996566
C	−0.189300	0.704476	0.379298
C	1.106783	0.384257	1.112591
O	1.250645	−1.061473	1.305702
C	2.426309	−1.546956	0.609457
C	2.397392	0.840372	0.404549
C	2.848857	−0.417348	−0.337876
O	3.324788	1.252641	1.460328
H	−1.004534	0.320274	−1.379138
H	−1.017112	0.205707	0.898707
H	−0.351495	1.791168	0.421706
H	1.050239	0.847818	2.103510
H	3.205819	−1.787341	1.332984
H	2.236731	1.688976	−0.263912
H	2.305796	−0.472420	−1.283651
H	3.915553	−0.419448	−0.551658
N	2.110476	−2.817879	−0.055953
C	2.729081	−4.016733	0.398348
O	3.475107	−3.976309	1.401650
N	2.496380	−5.163013	−0.290962
C	1.665633	−5.182323	−1.352824
N	1.509998	−6.342872	−2.005252
C	0.964765	−4.001513	−1.770832
C	1.214832	−2.850608	−1.095201
H	2.024775	−7.186910	−1.710940
H	0.905263	−6.388887	−2.814985
H	0.258029	−4.026314	−2.592221
H	0.718775	−1.911891	−1.321289
P	4.914537	1.532542	1.180042
O	5.188420	1.844466	−0.276249
O	5.355432	2.565012	2.200196
O	5.612676	0.084087	1.486192
C	5.701122	−0.393585	2.860298
C	6.860319	−1.370405	3.006945
O	6.556565	−2.649651	2.371999
C	7.463698	−2.899427	1.269091
C	8.188883	−0.894928	2.388665
C	8.197324	−1.574339	1.017687
O	9.253546	−1.362058	3.276747
H	4.763252	−0.892931	3.128164
H	5.862095	0.457456	3.530592
H	6.995371	−1.555846	4.078571
H	8.147054	−3.707164	1.533725
H	8.253297	0.193082	2.314582
H	7.647066	−0.942545	0.318249
H	9.201354	−1.718153	0.626736
N	6.696208	−3.400819	0.120685
C	6.835698	−4.759748	−0.287070
O	7.626039	−5.505326	0.325728
N	6.091457	−5.189488	−1.341583
C	5.252413	−4.360478	−1.992897
N	4.563669	−4.845295	−3.035209
C	5.094077	−2.994982	−1.587328
C	5.817859	−2.572062	−0.521161
H	4.637455	−5.842504	−3.303807
H	3.903670	−4.249509	−3.517064
H	4.403842	−2.334494	−2.097289
H	5.728566	−1.573551	−0.110895
P	10.835155	−1.302581	2.848361
O	11.089752	−0.269927	1.770173
O	11.621788	−1.158567	4.138031
O	11.095996	−2.768650	2.169069
C	11.125503	−3.946239	3.028509
C	11.940938	−5.057968	2.382186

O	11.209831	−5.676256	1.279013
C	11.893783	−5.450715	0.023261
C	13.295345	−4.613247	1.801316
C	12.990915	−4.410854	0.310951
O	14.235102	−5.696453	2.029114
H	10.099629	−4.291672	3.199679
H	11.581995	−3.681784	3.988523
H	12.098900	−5.830589	3.144264
H	12.303672	−6.399364	−0.335885
H	13.658712	−3.695369	2.274862
H	12.613330	−3.397928	0.155995
H	13.869548	−4.558768	−0.321411
H	15.086127	−5.435770	1.631647
N	10.919091	−5.034851	−0.979163
C	10.233295	−3.829183	−1.027369
N	9.322961	−3.786786	−1.985013
C	9.395765	−5.038064	−2.596117
C	8.677310	−5.618074	−3.667857
N	7.714051	−4.979278	−4.355963
N	9.001546	−6.895335	−4.019227
C	9.977443	−7.542715	−3.354438
N	10.722116	−7.086196	−2.334363
C	10.381170	−5.829952	−1.985424
H	10.450086	−3.040426	−0.322401
H	7.181373	−5.477749	−5.080588
H	7.394074	−4.076088	−4.033863
H	10.167207	−8.560813	−3.689310
O	5.822159	−11.594216	−10.620074
C	7.057162	−12.300301	−10.895581
C	7.640381	−12.865193	−9.606000
O	8.188225	−11.800820	−8.762839
C	7.472931	−11.714373	−7.504249
C	6.641090	−13.637226	−8.723146
C	6.242209	−12.623694	−7.646528
O	7.355279	−14.798301	−8.190918
H	5.565983	−11.117309	−11.428620
H	7.792997	−11.625291	−11.350165
H	6.873932	−13.136629	−11.585581
H	8.470238	−13.525273	−9.880856
H	8.126501	−12.032892	−6.690419
H	5.778417	−13.997369	−9.287255
H	5.370363	−12.064720	−7.990758
H	5.990039	−13.107745	−6.706535
N	7.163182	−10.301693	−7.222339
C	7.887417	−9.638122	−6.216597
O	8.774210	−10.175915	−5.548176
N	7.523409	−8.318707	−6.028434
C	6.594802	−7.591268	−6.763623
O	6.395421	−6.383147	−6.503850
C	5.927549	−8.317582	−7.829606
C	4.931728	−7.596459	−8.697168
C	6.254457	−9.622778	−8.012370
H	8.060024	−7.806772	−5.281599
H	5.394825	−6.733428	−9.191603
H	4.097060	−7.212644	−8.097206
H	4.529779	−8.264467	−9.464477
H	5.834830	−10.205813	−8.825741
P	6.669004	−15.800676	−7.087945
O	5.156260	−15.731951	−7.121640
O	7.300563	−17.164487	−7.300400
O	7.138229	−15.156794	−5.659390
C	8.561453	−15.141867	−5.351308
C	8.793425	−14.545599	−3.973733
O	8.486742	−13.117716	−3.985558
C	7.527338	−12.821553	−2.935223
C	7.952463	−15.164258	−2.831959
C	6.822280	−14.149351	−2.655581
O	8.852183	−15.291778	−1.680392
H	9.090856	−14.542236	−6.100844
H	8.951010	−16.166356	−5.373589
H	9.857888	−14.669861	−3.740899
H	8.050456	−12.451304	−2.049816
H	7.585580	−16.163885	−3.074650
H	6.072239	−14.350434	−3.424832
H	6.345246	−14.188656	−1.677160
N	6.662857	−11.738817	−3.350436
C	5.629318	−11.753787	−4.285168
N	5.061250	−10.571723	−4.432303
C	5.740517	−9.729521	−3.547707
C	5.558693	−8.350786	−3.236012
O	4.745822	−7.550920	−3.757452
N	6.423298	−7.922740	−2.210171
C	7.363805	−8.718245	−1.580384
N	8.095917	−8.159508	−0.598603
N	7.573944	−9.995897	−1.915514
C	6.739064	−10.439672	−2.868025
H	5.348360	−12.658778	−4.801671
H	6.313583	−6.929318	−1.893052
H	7.904476	−7.207353	−0.255282
H	8.633834	−8.800766	−0.025371
P	8.408344	−15.186722	−0.110722
O	7.008779	−15.711463	0.137894
O	9.528263	−15.834000	0.682553
O	8.353115	−13.575592	0.165434
C	9.587176	−12.811813	0.311491
C	9.536014	−11.937362	1.560138
O	8.674607	−10.773421	1.374122
C	7.448515	−10.919028	2.134842
C	9.002883	−12.652998	2.812154
C	7.507230	−12.302609	2.810346
O	9.698693	−12.093500	3.957490
H	9.705144	−12.177302	−0.572827
H	10.437716	−13.496114	0.390871
H	10.548663	−11.558482	1.739620
H	7.405839	−10.102723	2.860323
H	9.171594	−13.733943	2.762435
H	6.961322	−13.045841	2.227391
H	7.087856	−12.275377	3.819003
H	9.331137	−12.512054	4.757388
N	6.294626	−10.724155	1.262950
C	5.739157	−11.614397	0.340166
N	4.745056	−11.082654	−0.343060
C	4.640003	−9.770897	0.126813
C	3.773641	−8.703695	−0.253418
O	2.865042	−8.718845	−1.119525
N	4.041889	−7.523339	0.466695
C	4.992308	−7.395632	1.459899
N	5.052184	−6.209091	2.105596
N	5.808752	−8.393284	1.815380
C	5.598690	−9.531155	1.123340
H	6.133669	−12.612335	0.218097
H	3.474382	−6.683314	0.209314
H	4.504692	−5.395796	1.783699
H	5.888040	−6.034478	2.649487
Na	5.065363	−18.121476	−7.557982
Na	7.926727	−17.284679	1.753832
Na	12.700919	0.827941	3.238527
Na	6.176515	3.969058	0.395776

d(CGA)$^{Na+}$

E: −27507.95 kcal mol^{-1}

O	−0.213276	−0.309176	−0.978346
C	−0.265579	0.084310	0.415371
C	1.103944	−0.074963	1.064192

O	1.453380	−1.488307	1.220537
C	2.625195	−1.814955	0.421896
C	2.274585	0.560700	0.290736
C	2.848699	−0.613015	−0.504050
O	3.194423	1.092080	1.298441
H	−1.127824	−0.354504	−1.307725
H	−0.987092	−0.535859	0.962108
H	−0.568952	1.137513	0.505284
H	1.045949	0.364155	2.066241
H	3.474998	−1.986876	1.083410
H	1.957296	1.385107	−0.351715
H	2.278184	−0.711633	−1.429198
H	3.894549	−0.474122	−0.764035
N	2.400672	−3.093911	−0.264570
C	3.080882	−4.262321	0.187088
O	3.847438	−4.180745	1.171688
N	2.867449	−5.429964	−0.474962
C	2.015555	−5.495135	−1.517922
N	1.861425	−6.675910	−2.134085
C	1.270785	−4.345036	−1.942491
C	1.486222	−3.178649	−1.283022
H	2.417196	−7.497002	−1.848321
H	1.251388	−6.744532	−2.937876
H	0.549125	−4.406897	−2.748983
H	0.936528	−2.270304	−1.504097
P	4.708211	1.605605	0.941807
O	4.879601	1.908241	−0.532224
O	5.019293	2.730915	1.912486
O	5.630964	0.294845	1.262640
C	5.784826	−0.136268	2.644658
C	6.986435	−1.060930	2.783822
O	6.734610	−2.357712	2.165802
C	7.588563	−2.541227	1.006154
C	8.291318	−0.541869	2.148767
C	8.272766	−1.190374	0.763519
O	9.381776	−0.985857	3.022131
H	4.878944	−0.666463	2.960639
H	5.930425	0.740355	3.286064
H	7.138744	−1.230730	3.855746
H	8.306889	−3.336052	1.223323
H	8.331067	0.548908	2.096295
H	7.662013	−0.560628	0.114669
H	9.260307	−1.287499	0.317021
N	6.809348	−3.022614	−0.120457
C	5.965882	−2.303628	−0.964227
N	5.403404	−3.062118	−1.886555
C	5.894729	−4.348534	−1.648192
C	5.661711	−5.583288	−2.321146
O	4.908822	−5.795577	−3.300741
N	6.417455	−6.634604	−1.766972
C	7.272227	−6.516664	−0.686933
N	7.917708	−7.628986	−0.289246
N	7.458910	−5.367580	−0.029687
C	6.767409	−4.340391	−0.550459
H	5.816234	−1.242813	−0.833294
H	6.341178	−7.567219	−2.241808
H	7.908052	−8.496710	−0.846906
H	8.622603	−7.507939	0.426637
P	10.908095	−1.297287	2.526971
O	11.324856	−0.444297	1.345970
O	11.776395	−1.214609	3.768388
O	10.823004	−2.834107	1.974953
C	10.689790	−3.941494	2.911774
C	11.511297	−5.133242	2.434754
O	10.907744	−5.770728	1.268130
C	11.712332	−5.539433	0.086869
C	12.953073	−4.787580	2.025298
C	12.853221	−4.593145	0.505279
O	13.783486	−5.923974	2.381924
H	9.633155	−4.222194	2.979278
H	11.047703	−3.633138	3.899683
H	11.515334	−5.874786	3.242320
H	12.081134	−6.504313	−0.271458
H	13.314063	−3.885827	2.531566
H	12.597379	−3.554395	0.290449
H	13.787392	−4.835304	−0.007088
H	14.689439	−5.733898	2.076508
N	10.867864	−5.021509	−0.988015
C	10.341344	−3.741326	−1.121068
N	9.514481	−3.625053	−2.146137
C	9.473362	−4.897165	−2.715527
C	8.742829	−5.434564	−3.800661
N	7.897610	−4.714737	−4.566963
N	8.916430	−6.757114	−4.078545
C	9.757992	−7.493276	−3.328003
N	10.494185	−7.086227	−2.281113
C	10.307045	−5.779897	−2.009147
H	10.596521	−2.958186	−0.422619
H	7.282235	−5.208394	−5.225873
H	7.634744	−3.787822	−4.255807
H	9.827557	−8.543007	−3.605411
O	5.508368	−11.467032	−10.594799
C	6.713325	−12.215183	−10.892638
C	7.334081	−12.750777	−9.608527
O	7.929325	−11.669510	−8.819097
C	7.253158	−11.531723	−7.544311
C	6.357584	−13.474085	−8.661701
C	5.988123	−12.397950	−7.638062
O	7.092673	−14.597819	−8.081679
H	5.231279	−11.014880	−11.410644
H	7.447694	−11.575469	−11.397919
H	6.482155	−13.068963	−11.545838
H	8.142445	−13.433415	−9.891641
H	7.916937	−11.859004	−6.742127
H	5.482238	−13.868128	−9.182200
H	5.142925	−11.825708	−8.024488
H	5.703494	−12.821950	−6.678529
N	7.003538	−10.104652	−7.283038
C	7.751466	−9.459578	−6.282249
O	8.618076	−10.026363	−5.610594
N	7.426694	−8.131121	−6.096128
C	6.501983	−7.383583	−6.816160
O	6.328729	−6.174309	−6.548219
C	5.800147	−8.095882	−7.870891
C	4.798073	−7.356393	−8.714695
C	6.096373	−9.407180	−8.059848
H	7.965123	−7.633324	−5.338998
H	5.264760	−6.501986	−9.220298
H	3.985718	−6.956874	−8.094516
H	4.365136	−8.017157	−9.471238
H	5.648220	−9.984146	−8.862526
P	6.471008	−15.515959	−6.873696
O	4.962983	−15.402475	−6.792674
O	7.049149	−16.907618	−7.051628
O	7.070999	−14.806350	−5.524680
C	8.512629	−14.853515	−5.316184
C	8.868060	−14.334203	−3.932867
O	8.633481	−12.896745	−3.851540
C	7.709830	−12.601325	−2.769841
C	8.085390	−14.968339	−2.762985
C	7.006484	−13.927067	−2.460433
O	9.051295	−15.153095	−1.678911
H	9.008566	−14.236853	−6.074671
H	8.860464	−15.888286	−5.413389
H	9.939909	−14.514810	−3.787701
H	8.259376	−12.221145	−1.907601
H	7.664664	−15.943988	−3.016471
H	6.170690	−14.102394	−3.141068

H	6.631870	−13.981755	−1.440888
N	6.828811	−11.507117	−3.184268
C	7.018975	−10.204152	−2.635198
O	7.921346	−10.024676	−1.791646
N	6.197737	−9.206055	−3.053443
C	5.245172	−9.422491	−3.979343
N	4.489188	−8.385623	−4.366148
C	5.063363	−10.718656	−4.567660
C	5.881488	−11.715639	−4.146675
H	4.630961	−7.443878	−3.963052
H	3.759924	−8.527519	−5.051819
H	4.317240	−10.891068	−5.333965
H	5.840933	−12.716007	−4.559024
P	8.618424	−15.458905	−0.129670
O	7.230798	−16.057962	−0.030162
O	9.755068	−16.258101	0.480599
O	8.534182	−13.969088	0.536870
C	9.762392	−13.231488	0.804490
C	9.591906	−12.342332	2.030216
O	8.772093	−11.171607	1.735623
C	7.495120	−11.254432	2.416296
C	8.926882	−13.029677	3.235143
C	7.449295	−12.636576	3.095904
O	9.534536	−12.475998	4.432397
H	10.004548	−12.614870	−0.068277
H	10.578181	−13.938823	0.987581
H	10.587520	−11.976910	2.308729
H	7.433906	−10.432915	3.135216
H	9.066063	−14.115703	3.211943
H	6.941114	−13.364729	2.461337
H	6.936576	−12.596885	4.060020
H	9.081082	−12.868618	5.200743
N	6.419728	−11.023613	1.459399
C	5.975529	−11.889699	0.460004
N	5.071660	−11.340624	−0.326420
C	4.916037	−10.039612	0.155720
C	4.098313	−8.967017	−0.302919
O	3.289346	−8.972906	−1.261086
N	4.285486	−7.797645	0.462090
C	5.116281	−7.691144	1.558286
N	5.123534	−6.501500	2.207612
N	5.867476	−8.702937	2.004654
C	5.747992	−9.823826	1.265251
H	6.378506	−12.887230	0.372040
H	3.761348	−6.947382	0.145063
H	4.701950	−5.664760	1.772820
H	5.889585	−6.354919	2.854032
Na	4.771088	−17.807368	−7.087278
Na	8.171110	−17.961082	1.162694
Na	13.223274	0.363139	2.631570
Na	5.573717	4.180257	0.059075

d(CGG)$^{Na+}$

E: −27419.41 kcal mol^{-1}

O	−0.011528	−0.464679	−0.948498
C	−0.188300	−0.193260	0.464057
C	1.148217	−0.285920	1.190212
O	1.596945	−1.673121	1.303920
C	2.835493	−1.884098	0.573144
C	2.303118	0.483230	0.521564
C	3.082765	−0.599285	−0.232798
O	3.073063	1.107766	1.597374
H	−0.894425	−0.572513	−1.343215
H	−0.884353	−0.914515	0.910271
H	−0.589782	0.819954	0.610763
H	0.997135	0.093795	2.206763
H	3.642228	−2.085673	1.278350
H	1.948260	1.271901	−0.144736
H	2.690888	−0.678944	−1.248352
H	4.143080	−0.367340	−0.292220
N	2.708519	−3.112447	−0.231330
C	3.362977	−4.303932	0.198664
O	4.091446	−4.268954	1.214080
N	3.167228	−5.444409	−0.515521
C	2.353256	−5.464556	−1.589907
N	2.189237	−6.628545	−2.234575
C	1.658175	−4.284856	−2.016832
C	1.850690	−3.148035	−1.301420
H	2.681729	−7.479996	−1.924292
H	1.609611	−6.665885	−3.062383
H	0.977778	−4.305829	−2.860416
H	1.316784	−2.228220	−1.512952
P	4.436499	1.965068	1.289426
O	4.486803	2.458254	−0.141828
O	4.536664	3.015056	2.381706
O	5.623080	0.849620	1.445744
C	5.809365	0.231501	2.750728
C	6.905996	−0.817959	2.684541
O	6.471348	−1.941001	1.862239
C	7.450060	−2.179301	0.813187
C	8.257400	−0.343925	2.098574
C	8.202252	−0.857811	0.659085
O	9.306515	−0.941662	2.935650
H	4.874385	−0.246685	3.064390
H	6.081500	0.999429	3.484452
H	7.070319	−1.176940	3.708056
H	8.118429	−2.987269	1.116405
H	8.382308	0.739531	2.153855
H	7.610414	−0.146377	0.077164
H	9.182150	−0.969646	0.196207
N	6.788475	−2.664031	−0.376653
C	6.049496	−1.969263	−1.332471
N	5.551119	−2.763450	−2.262066
C	5.982458	−4.047117	−1.914604
C	5.777996	−5.312452	−2.537102
O	5.095510	−5.561022	−3.559418
N	6.461032	−6.347840	−1.868046
C	7.241952	−6.182349	−0.737262
N	7.855332	−7.272392	−0.237040
N	7.387077	−5.005204	−0.123493
C	6.757305	−4.000264	−0.748890
H	5.934194	−0.896612	−1.290658
H	6.392168	−7.306556	−2.288299
H	7.858313	−8.171069	−0.739932
H	8.568828	−7.091753	0.462267
P	10.747073	−1.526759	2.436792
O	11.337860	−0.738963	1.284222
O	11.600252	−1.642578	3.686218
O	10.386231	−3.002999	1.835705
C	10.078228	−4.119239	2.720752
C	10.943664	−5.329822	2.382411
O	10.507343	−5.989256	1.155122
C	11.432148	−5.715635	0.070993
C	12.430972	−5.006881	2.167714
C	12.535007	−4.805940	0.646882
O	13.190592	−6.157573	2.623407
H	9.023211	−4.378307	2.585821
H	10.258656	−3.830097	3.760825
H	10.823311	−6.058118	3.192565
H	11.822803	−6.674280	−0.278563
H	12.735209	−4.113156	2.722491
H	12.347575	−3.759072	0.404331
H	13.520543	−5.079756	0.262721
H	14.131966	−5.975942	2.447724
N	10.716655	−5.149926	−1.070132

C	10.281657	−3.836771	−1.269596
N	9.575993	−3.697825	−2.374558
C	9.514697	−4.977396	−2.932057
C	8.851695	−5.463102	−4.097692
O	8.161712	−4.813469	−4.921153
N	9.027701	−6.850454	−4.265884
C	9.779559	−7.662916	−3.441707
N	9.914474	−8.958123	−3.810179
N	10.391459	−7.212542	−2.341638
C	10.217812	−5.891721	−2.133100
H	10.504014	−3.057014	−0.556650
H	8.515345	−7.289033	−5.066094
H	9.369835	−9.345569	−4.596285
H	10.216457	−9.598970	−3.086579
O	5.075671	−11.582017	−10.701273
C	6.206304	−12.420683	−11.045573
C	6.933078	−12.875142	−9.785792
O	7.612830	−11.748357	−9.140940
C	7.056257	−11.513402	−7.821279
C	6.041364	−13.500784	−8.695761
C	5.754842	−12.322309	−7.764652
O	6.838051	−14.556676	−8.066599
H	4.754191	−11.167776	−11.521022
H	6.914098	−11.868714	−11.676643
H	5.870204	−13.313346	−11.592667
H	7.700574	−13.595683	−10.088764
H	7.770518	−11.835153	−7.063036
H	5.131122	−13.950099	−9.098709
H	4.918332	−11.754244	−8.176590
H	5.496054	−12.636578	−6.755831
N	6.896158	−10.068596	−7.610490
C	7.726354	−9.407408	−6.660885
O	8.603766	−10.063028	−6.056918
N	7.522403	−8.084488	−6.436617
C	6.584863	−7.396646	−7.119072
N	6.423184	−6.098985	−6.826692
C	5.789353	−8.029373	−8.132639
C	5.983249	−9.355982	−8.346896
H	6.998629	−5.646353	−6.101059
H	5.718353	−5.556947	−7.309002
H	5.067540	−7.468236	−8.714789
H	5.452890	−9.914455	−9.111464
P	6.401484	−15.283827	−6.664315
O	4.912210	−15.182378	−6.409544
O	7.000687	−16.676924	−6.701252
O	7.133192	−14.368199	−5.520624
C	8.584568	−14.412641	−5.392385
C	9.006048	−14.027632	−3.980377
O	8.847738	−12.596006	−3.745659
C	7.860876	−12.351557	−2.709761
C	8.219408	−14.733543	−2.859931
C	7.163952	−13.697447	−2.466153
O	9.181857	−15.028860	−1.798897
H	9.030972	−13.718777	−6.113381
H	8.935508	−15.428393	−5.603878
H	10.072612	−14.261871	−3.886584
H	8.355737	−11.973419	−1.814280
H	7.772518	−15.674239	−3.189540
H	6.301379	−13.822181	−3.123345
H	6.825813	−13.809020	−1.439925
N	6.970902	−11.266946	−3.148277
C	7.057982	−9.981612	−2.533353
O	7.839482	−9.812191	−1.575758
N	6.278548	−8.982768	−3.029279
C	5.421536	−9.199457	−4.045278
N	4.676448	−8.174807	−4.482215
C	5.314783	−10.487660	−4.664745
C	6.123754	−11.472620	−4.202789
H	4.816363	−7.217039	−4.115099
H	4.066209	−8.310859	−5.277131
H	4.640309	−10.656299	−5.495099
H	6.160136	−12.457856	−4.651323
P	8.720247	−15.525920	−0.306877
O	7.331569	−16.130411	−0.311678
O	9.846000	−16.396770	0.220728
O	8.621928	−14.130554	0.536226
C	9.844415	−13.405104	0.852402
C	9.624055	−12.517252	2.068865
O	8.785732	−11.369439	1.740272
C	7.508741	−11.456055	2.423636
C	8.941088	−13.214681	3.258513
C	7.461615	−12.845001	3.084322
O	9.510967	−12.649809	4.469293
H	10.130545	−12.789897	−0.008303
H	10.646823	−14.119125	1.068528
H	10.603974	−12.131125	2.374446
H	7.454798	−10.646036	3.156439
H	9.098304	−14.298240	3.240220
H	6.985979	−13.574447	2.426603
H	6.921920	−12.824835	4.034140
H	9.047318	−13.050531	5.227310
N	6.434310	−11.201596	1.473726
C	5.932396	−12.061035	0.496438
N	5.052699	−11.477966	−0.293395
C	4.971378	−10.161187	0.164549
C	4.219884	−9.049406	−0.314201
O	3.407498	−9.022104	−1.270338
N	4.481077	−7.879064	0.427123
C	5.324749	−7.800620	1.516340
N	5.402209	−6.606820	2.147014
N	6.023475	−8.845599	1.973358
C	5.827596	−9.970992	1.260020
H	6.264733	−13.085930	0.431354
H	4.000447	−7.007200	0.102694
H	4.986794	−5.758227	1.731457
H	6.175827	−6.488583	2.789454
Na	4.788382	−17.610772	−6.286746
Na	8.260503	−18.185637	0.633083
Na	13.323283	−0.333535	2.617361
Na	4.684671	4.744006	0.662563

d(CTA)$^{Na+}$

E: −27598.44 kcal mol^{-1}

O	−0.188254	−0.130918	−0.581688
C	−0.235023	0.119489	0.845428
C	1.144757	−0.081139	1.459774
O	1.502111	−1.500983	1.508189
C	2.662336	−1.771956	0.682111
C	2.294220	0.621793	0.712307
C	2.912105	−0.494682	−0.133162
O	3.200674	1.151990	1.728548
H	−1.104118	−0.149723	−0.910396
H	−0.942516	−0.562948	1.332601
H	−0.551871	1.153851	1.040989
H	1.103256	0.280846	2.492724
H	3.511607	−2.031496	1.315401
H	1.944334	1.454042	0.098309
H	2.392379	−0.532091	−1.091960
H	3.970843	−0.331217	−0.321308
N	2.401701	−2.973797	−0.131769
C	3.120527	−4.176154	0.126612
O	3.996196	−4.185992	1.019957
N	2.833119	−5.273842	−0.621524
C	1.885054	−5.236941	−1.579072
N	1.666759	−6.350966	−2.293285

C	1.119845	−4.048504	−1.823205
C	1.399728	−2.955605	−1.069567
H	2.141457	−7.234018	−2.052445
H	0.929185	−6.363838	−2.985679
H	0.330836	−4.028521	−2.566241
H	0.841969	−2.027841	−1.148577
P	4.622958	1.861708	1.322477
O	4.636088	2.324490	−0.119214
O	4.895921	2.911005	2.384890
O	5.692833	0.628062	1.433653
C	5.905398	0.033974	2.746427
C	7.016665	−0.997002	2.681633
O	6.571021	−2.165613	1.929914
C	7.542276	−2.480932	0.901460
C	8.331624	−0.520115	2.019444
C	8.282213	−1.167960	0.633480
O	9.414783	−1.005267	2.874984
H	4.979726	−0.446881	3.082980
H	6.181134	0.817691	3.461431
H	7.232874	−1.308072	3.710906
H	8.220831	−3.262178	1.250439
H	8.404469	0.568303	1.965612
H	7.700872	−0.513361	−0.020122
H	9.265019	−1.310065	0.188770
N	6.841044	−3.060734	−0.244497
C	6.993657	−4.439339	−0.490794
O	7.724740	−5.167491	0.180715
N	6.258966	−4.910366	−1.560837
C	5.415581	−4.176858	−2.385775
O	4.799576	−4.734156	−3.318961
C	5.315147	−2.757960	−2.087573
C	4.456065	−1.893801	−2.968975
C	6.014451	−2.282599	−1.025100
H	6.374483	−5.940661	−1.774080
H	4.782299	−1.966879	−4.013351
H	4.509305	−0.845963	−2.664499
H	3.409341	−2.217889	−2.940414
H	5.955903	−1.242687	−0.727260
P	10.974224	−1.140202	2.397489
O	11.303721	−0.241847	1.224133
O	11.817086	−0.972235	3.648613
O	11.057734	−2.677195	1.845806
C	10.970262	−3.782133	2.791256
C	11.788967	−4.966988	2.295192
O	11.137509	−5.638678	1.174854
C	11.871928	−5.419243	−0.051706
C	13.201753	−4.609554	1.804486
C	13.020006	−4.449299	0.287622
O	14.066277	−5.726358	2.141111
H	9.920557	−4.076850	2.898694
H	11.358937	−3.462204	3.764011
H	11.846507	−5.691890	3.115574
H	12.235167	−6.384931	−0.413627
H	13.575027	−3.692108	2.271664
H	12.738212	−3.418903	0.064459
H	13.929902	−4.690320	−0.267077
H	14.954143	−5.527122	1.791493
N	10.961498	−4.932171	−1.087045
C	10.403815	−3.665503	−1.203947
N	9.543564	−3.567813	−2.204307
C	9.518149	−4.839631	−2.774776
C	8.796499	−5.381651	−3.863927
N	7.930096	−4.675378	−4.613394
N	9.009617	−6.693848	−4.164870
C	9.871700	−7.415841	−3.425829
N	10.601177	−7.004557	−2.374862
C	10.386370	−5.704494	−2.089909
H	10.661967	−2.878307	−0.511002
H	7.361794	−5.152065	−5.325118
H	7.709605	−3.724538	−4.349416
H	9.970887	−8.459380	−3.718321
O	5.384030	−11.608197	−10.472419
C	6.530023	−12.464009	−10.705830
C	7.137874	−12.910890	−9.381493
O	7.786116	−11.789364	−8.696672
C	7.126147	−11.520627	−7.431308
C	6.140356	−13.504866	−8.364017
C	5.812951	−12.308841	−7.468499
O	6.824598	−14.622083	−7.698075
H	5.123840	−11.223046	−11.327347
H	7.296758	−11.929104	−11.280304
H	6.231058	−13.360381	−11.268111
H	7.912472	−13.651295	−9.608304
H	7.766205	−11.841271	−6.610982
H	5.250645	−13.917855	−8.844082
H	5.025900	−11.734169	−7.960732
H	5.460673	−12.589366	−6.479201
N	6.967049	−10.071833	−7.258397
C	7.729177	−9.429947	−6.267104
O	8.562935	−10.011692	−5.567578
N	7.461456	−8.081682	−6.131613
C	6.597746	−7.316121	−6.906882
O	6.473946	−6.090184	−6.683184
C	5.896793	−8.026834	−7.962036
C	4.976783	−7.264185	−8.876601
C	6.121684	−9.360798	−8.087996
H	8.023786	−7.578582	−5.396767
H	5.519928	−6.472278	−9.407349
H	4.174936	−6.777270	−8.307800
H	4.524100	−7.931830	−9.615373
H	5.662444	−9.947998	−8.877347
P	6.677796	−15.036285	−6.127281
O	5.270141	−14.837373	−5.594994
O	7.258896	−16.431193	−5.994848
O	7.598610	−13.936075	−5.352161
C	9.024326	−14.109817	−5.144040
C	9.339597	−14.046227	−3.653766
O	9.149235	−12.705498	−3.112630
C	7.925052	−12.629690	−2.341989
C	8.465652	−14.965222	−2.782497
C	7.322823	−14.047049	−2.322399
O	9.321626	−15.450136	−1.702374
H	9.543427	−13.300398	−5.666821
H	9.351302	−15.074721	−5.544272
H	10.398093	−14.295792	−3.523666
H	8.185156	−12.274436	−1.341744
H	8.096306	−15.832919	−3.334723
H	6.498409	−14.142573	−3.028191
H	6.954004	−14.310887	−1.333294
N	7.026220	−11.610775	−2.892450
C	6.041231	−11.737803	−3.865671
N	5.435737	−10.594213	−4.141133
C	6.046087	−9.657696	−3.310224
C	5.834586	−8.272223	−3.122067
N	4.945503	−7.549493	−3.821931
N	6.586749	−7.652644	−2.168196
C	7.510774	−8.353562	−1.485860
N	7.813149	−9.655637	−1.611053
C	7.040178	−10.266465	−2.529934
H	5.817496	−12.687216	−4.326729
H	4.848174	−6.539829	−3.653149
H	4.355339	−8.010164	−4.500618
H	8.071510	−7.782096	−0.749578
P	8.717478	−16.147680	−0.349216
O	7.391483	−16.835395	−0.603132
O	9.836033	−17.001272	0.218951
O	8.404667	−14.864493	0.615519
C	9.518293	−14.012315	1.009137

C	9.012318	−12.892713	1.898880
O	8.224603	−11.951380	1.105341
C	7.009536	−11.635924	1.831120
C	8.114938	−13.351248	3.074637
C	6.712032	−12.897147	2.651459
O	8.582873	−12.663034	4.264792
H	9.993483	−13.593752	0.114738
H	10.256862	−14.606290	1.560050
H	9.886815	−12.366150	2.303455
H	7.174204	−10.764608	2.474783
H	8.169569	−14.433273	3.225572
H	6.260136	−13.663289	2.015048
H	6.053705	−12.703083	3.501762
H	8.043333	−12.973973	5.014756
N	5.974687	−11.250976	0.890500
C	5.278311	−12.075236	0.004794
N	4.371704	−11.421485	−0.693169
C	4.461737	−10.098838	−0.256528
C	3.722924	−8.938951	−0.633964
O	2.808425	−8.856122	−1.489324
N	4.129527	−7.797296	0.081183
C	5.119484	−7.774229	1.041665
N	5.339115	−6.596800	1.666497
N	5.824709	−8.858636	1.388474
C	5.456855	−9.971881	0.724604
H	5.490099	−13.131669	−0.069343
H	3.662904	−6.894748	−0.171662
H	4.882759	−5.729275	1.344422
H	6.211441	−6.508851	2.172841
Na	13.117770	0.741169	2.580077
Na	5.096084	4.591937	0.625169
Na	5.237447	−17.152203	−4.881557
Na	8.305984	−18.934430	0.149035

d(GAA)$^{Na+}$

E: −27606.36 kcal mol^{-1}

O	0.285418	0.572895	−1.013691
C	0.412050	1.015047	0.359345
C	1.703851	0.482975	0.967427
O	1.641853	−0.976008	1.102468
C	2.712295	−1.582904	0.331185
C	2.987385	0.782435	0.158777
C	3.178410	−0.504925	−0.646579
O	4.048267	1.090991	1.131729
H	−0.600576	0.828685	−1.324404
H	−0.432935	0.651441	0.958413
H	0.432357	2.113469	0.413398
H	1.799271	0.910207	1.971886
H	3.519021	−1.880880	1.000002
H	2.879681	1.660824	−0.481223
H	2.507357	−0.458789	−1.507858
H	4.197559	−0.657661	−0.995375
N	2.260365	−2.821142	−0.277123
C	1.358071	−3.028047	−1.318807
N	1.249713	−4.303694	−1.648099
C	2.125043	−4.978229	−0.789780
C	2.466207	−6.359023	−0.676907
O	2.012089	−7.322412	−1.342901
N	3.441913	−6.584076	0.317174
C	3.991320	−5.607676	1.127270
N	4.885990	−5.994568	2.068865
N	3.656017	−4.319537	1.035119
C	2.754430	−4.069026	0.070826
H	0.814136	−2.206048	−1.764833
H	3.796687	−7.563957	0.396020
H	5.276922	−6.945794	2.038197
H	5.483383	−5.246717	2.418552
P	5.611152	0.608989	1.040838
O	6.130620	0.596870	−0.375924
O	6.337976	1.409504	2.107899
O	5.541216	−0.971990	1.470461
C	5.473750	−1.462245	2.832184
C	6.747482	−2.240854	3.159731
O	6.831560	−3.507354	2.441338
C	7.728655	−3.394178	1.304422
C	8.005983	−1.461321	2.765769
C	8.366820	−1.996760	1.369453
O	9.037009	−1.700440	3.771121
H	4.605980	−2.124594	2.903485
H	5.366323	−0.631340	3.537299
H	6.747503	−2.479517	4.227967
H	8.470796	−4.188979	1.398153
H	7.793145	−0.394843	2.752419
H	7.953387	−1.332429	0.610489
H	9.444532	−2.062264	1.227726
N	7.012738	−3.682346	0.059784
C	6.229532	−2.854876	−0.745276
N	5.589091	−3.518649	−1.692518
C	5.953198	−4.851715	−1.510905
C	5.602467	−6.045310	−2.183930
N	4.727509	−6.116218	−3.199909
N	6.202766	−7.191229	−1.758635
C	7.064977	−7.166129	−0.727667
N	7.434284	−6.102192	−0.000563
C	6.847912	−4.970598	−0.436135
H	6.186334	−1.787599	−0.592370
H	4.535560	−7.018158	−3.649770
H	4.290471	−5.273757	−3.546886
H	7.513289	−8.124285	−0.475192
P	10.432087	−0.851347	3.679912
O	10.210190	0.501680	3.017713
O	11.035082	−0.835738	5.067932
O	11.342880	−1.728661	2.645661
C	11.694552	−3.088903	3.048733
C	12.151091	−3.895954	1.850251
O	11.028518	−4.133953	0.957452
C	11.583900	−4.335629	−0.365322
C	13.286270	−3.272563	0.989781
C	12.738334	−3.322615	−0.450807
O	14.465970	−4.089925	1.190469
H	10.824816	−3.572507	3.505996
H	12.506081	−3.046053	3.783340
H	12.517546	−4.861198	2.231293
H	11.935454	−5.368598	−0.472893
H	13.499001	−2.244374	1.294552
H	12.351971	−2.340432	−0.735722
H	13.502161	−3.619469	−1.173243
H	15.222348	−3.618388	0.796532
N	10.531867	−4.154232	−1.347757
C	9.910574	−2.957780	−1.693761
N	9.018173	−3.098869	−2.656349
C	9.045844	−4.456290	−2.970263
C	8.326540	−5.222340	−3.918660
N	7.411680	−4.693945	−4.748504
N	8.591614	−6.558774	−3.980843
C	9.505261	−7.087922	−3.144676
N	10.242685	−6.454603	−2.214150
C	9.974600	−5.134142	−2.168512
H	10.155156	−2.028345	−1.199976
H	6.848157	−5.311257	−5.348010
H	7.135417	−3.728493	−4.622954
H	9.646858	−8.163508	−3.233510
O	6.033342	−11.665119	−10.576067
C	7.311150	−12.320994	−10.769739
C	7.834441	−12.851630	−9.440975

O	8.290359	−11.758290	−8.578654
C	7.505154	−11.687175	−7.365456
C	6.808082	−13.653026	−8.616144
C	6.329279	−12.659440	−7.554275
O	7.509003	−14.807857	−8.058860
H	5.808120	−11.202182	−11.401784
H	8.044724	−11.619504	−11.185926
H	7.204073	−13.169522	−11.460605
H	8.703729	−13.482845	−9.654684
H	8.128684	−11.953420	−6.510186
H	5.982398	−14.020022	−9.228755
H	5.444180	−12.144328	−7.929296
H	6.064771	−13.157944	−6.625761
N	7.098615	−10.286169	−7.131838
C	7.716575	−9.559577	−6.099800
O	8.584126	−10.033079	−5.360193
N	7.256750	−8.262728	−5.958435
C	6.305228	−7.621126	−6.747700
O	5.985770	−6.436657	−6.510569
C	5.756678	−8.407185	−7.838212
C	4.753252	−7.774126	−8.762525
C	6.191542	−9.683806	−7.985964
H	7.716969	−7.694268	−5.206268
H	5.171987	−6.881621	−9.243429
H	3.863289	−7.452178	−8.207569
H	4.443376	−8.478036	−9.540275
H	5.866045	−10.304518	−8.814387
P	6.767272	−15.841537	−7.020788
O	5.258870	−15.729881	−7.088531
O	7.370159	−17.211190	−7.273986
O	7.218704	−15.270496	−5.554347
C	8.640806	−15.275992	−5.239769
C	8.874343	−14.642542	−3.881740
O	8.534888	−13.223039	−3.939006
C	7.644258	−12.898509	−2.844905
C	8.062887	−15.245764	−2.708868
C	6.955375	−14.215436	−2.478137
O	8.995208	−15.375957	−1.590457
H	9.184870	−14.708094	−6.002787
H	9.011129	−16.307713	−5.230063
H	9.942984	−14.738442	−3.654833
H	8.214719	−12.495863	−2.005048
H	7.666193	−16.236718	−2.939805
H	6.138444	−14.442184	−3.166606
H	6.563237	−14.226618	−1.462045
N	6.751240	−11.816310	−3.259591
C	6.978055	−10.528851	−2.732058
O	7.891337	−10.274060	−1.946721
N	6.066970	−9.580500	−3.142859
C	5.021489	−9.748428	−4.039755
O	4.290145	−8.782270	−4.341466
C	4.860072	−11.091700	−4.575655
C	3.743675	−11.355802	−5.547150
C	5.731420	−12.047198	−4.159948
H	6.143141	−8.636063	−2.656879
H	2.773468	−11.110763	−5.098793
H	3.727665	−12.404381	−5.854352
H	3.847988	−10.726827	−6.439404
H	5.671847	−13.064467	−4.526551
P	8.517583	−15.607966	−0.039470
O	7.136756	−16.222798	0.050939
O	9.648248	−16.354598	0.643131
O	8.393835	−14.082828	0.539094
C	9.614070	−13.310796	0.747034
C	9.383718	−12.257754	1.818787
O	8.526845	−11.193537	1.305946
C	7.337562	−11.070491	2.118757
C	8.710814	−12.780823	3.104328
C	7.241419	−12.372279	2.929667
O	9.341426	−12.102357	4.223000
H	9.896601	−12.827001	−0.194656
H	10.419542	−13.980779	1.065828
H	10.357739	−11.818179	2.066930
H	7.416850	−10.193018	2.760948
H	8.827076	−13.864188	3.210331
H	6.714450	−13.147056	2.367189
H	6.731361	−12.222974	3.884610
H	8.903178	−12.409056	5.037877
N	6.195425	−10.806295	1.229110
C	5.526725	−9.551392	1.286047
O	5.886841	−8.700088	2.131428
N	4.513067	−9.321062	0.411779
C	4.144315	−10.252767	−0.489331
N	3.143020	−9.949999	−1.329376
C	4.805878	−11.523162	−0.561547
C	5.831382	−11.743664	0.299658
H	2.729837	−9.004484	−1.334248
H	2.860808	−10.615603	−2.036261
H	4.516119	−12.267529	−1.293277
H	6.425605	−12.650356	0.289613
Na	5.113286	−18.081772	−7.655466
Na	8.088367	−18.067692	1.348434
Na	11.279239	1.542629	5.035873
Na	8.571133	1.883447	2.216018

d(GAC)$^{Na+}$

E: −27518.41 kcal mol^{-1}

O	0.179472	0.374673	−0.610647
C	0.306372	0.788567	0.771361
C	1.643474	0.329188	1.341752
O	1.686291	−1.132989	1.441297
C	2.738216	−1.655395	0.587650
C	2.888198	0.732845	0.520245
C	3.128978	−0.506221	−0.344150
O	3.954794	1.055755	1.480800
H	−0.734684	0.557321	−0.889772
H	−0.499034	0.353014	1.376651
H	0.252910	1.884132	0.854363
H	1.727978	0.734575	2.355999
H	3.580005	−1.970747	1.203859
H	2.718174	1.630556	−0.078398
H	2.442418	−0.456641	−1.192389
H	4.147834	−0.587818	−0.716966
N	2.293968	−2.862886	−0.087280
C	1.353283	−3.011958	−1.105323
N	1.268781	−4.258294	−1.536533
C	2.201141	−4.971465	−0.775656
C	2.587464	−6.344046	−0.799115
O	2.135440	−7.256920	−1.534452
N	3.606341	−6.624643	0.134999
C	4.158365	−5.703941	1.006057
N	5.097791	−6.144942	1.877424
N	3.787321	−4.422487	1.037738
C	2.839240	−4.118038	0.134555
H	0.764387	−2.173260	−1.452271
H	3.967865	−7.606491	0.137794
H	5.510642	−7.080134	1.758453
H	5.669447	−5.416933	2.298975
P	5.536180	0.654532	1.331783
O	6.017509	0.726997	−0.096574
O	6.250690	1.446862	2.413083
O	5.552446	−0.944194	1.697183
C	5.615611	−1.487620	3.038986
C	6.948616	−2.210641	3.239764
O	7.033459	−3.446177	2.467828

C	7.862894	−3.256542	1.290658
C	8.132167	−1.351213	2.780906
C	8.407594	−1.819997	1.344106
O	9.255941	−1.566987	3.688426
H	4.787735	−2.194470	3.149103
H	5.521897	−0.688648	3.782021
H	7.043446	−2.483582	4.295763
H	8.661649	−3.999940	1.327206
H	7.865528	−0.297858	2.825054
H	7.883177	−1.166943	0.646677
H	9.469697	−1.801855	1.106789
N	7.103171	−3.563354	0.079544
C	6.223410	−2.759911	−0.645012
N	5.613200	−3.415406	−1.617005
C	6.100659	−4.718955	−1.534731
C	5.826849	−5.893463	−2.273408
N	4.942148	−5.961300	−3.281019
N	6.509355	−7.019866	−1.923309
C	7.391760	−6.987529	−0.908603
N	7.711932	−5.937223	−0.137915
C	7.032930	−4.828624	−0.491727
H	6.089534	−1.715178	−0.411369
H	4.739739	−6.865179	−3.723767
H	4.401964	−5.143878	−3.529328
H	7.887162	−7.932850	−0.699079
P	10.578615	−0.617152	3.525044
O	10.206491	0.755414	2.981478
O	11.309835	−0.646647	4.850030
O	11.442588	−1.355397	2.353255
C	11.964284	−2.691262	2.633580
C	12.318063	−3.406718	1.346164
O	11.120997	−3.721309	0.594659
C	11.574083	−4.094632	−0.732157
C	13.233444	−2.652369	0.355948
C	12.780488	−3.171538	−1.033492
O	14.602246	−2.961244	0.697473
H	11.215784	−3.273243	3.181379
H	12.864241	−2.599260	3.250592
H	12.828412	−4.342298	1.629597
H	11.856182	−5.148744	−0.743795
H	13.070809	−1.573748	0.437019
H	12.490911	−2.348429	−1.690563
H	13.584068	−3.730759	−1.519011
H	15.176177	−2.310195	0.255726
N	10.448289	−3.964478	−1.657240
C	10.006467	−5.089477	−2.412705
O	10.541252	−6.205891	−2.224410
N	9.020412	−4.899931	−3.325852
C	8.453493	−3.688676	−3.504606
N	7.511044	−3.567789	−4.446972
C	8.848346	−2.556906	−2.715695
C	9.852151	−2.739906	−1.824272
H	7.243381	−4.371661	−5.036063
H	7.036684	−2.683141	−4.571173
H	8.374373	−1.590749	−2.837718
H	10.225770	−1.942326	−1.193839
O	6.355264	−11.548648	−10.710497
C	7.607811	−12.272435	−10.775692
C	7.957310	−12.852484	−9.410447
O	8.394871	−11.815686	−8.471744
C	7.426682	−11.631045	−7.409797
C	6.804498	−13.594270	−8.710763
C	6.241432	−12.554408	−7.737538
O	7.387338	−14.764552	−8.059365
H	6.245104	−11.069678	−11.550376
H	8.420649	−11.609181	−11.097043
H	7.530978	−13.105730	−11.489491
H	8.803620	−13.533220	−9.549352
H	7.902679	−11.878668	−6.456039
H	6.045255	−13.941558	−9.415021
H	5.448791	−12.002463	−8.243377
H	5.818275	−13.011761	−6.846913
N	7.057294	−10.218684	−7.313652
C	6.249911	−9.499815	−8.199788
N	6.176168	−8.218919	−7.894876
C	6.979259	−8.068183	−6.763976
C	7.295793	−6.907349	−6.001365
O	6.883994	−5.734601	−6.184090
N	8.175340	−7.194199	−4.943798
C	8.700235	−8.440735	−4.666216
N	9.569246	−8.517524	−3.637958
N	8.393919	−9.537852	−5.372059
C	7.546544	−9.296775	−6.392834
H	5.795750	−9.979297	−9.055179
H	8.461289	−6.387488	−4.342757
H	9.819069	−7.691239	−3.078728
H	9.794498	−9.438403	−3.284796
P	6.548155	−15.683941	−6.993682
O	5.052532	−15.474537	−7.104324
O	7.057858	−17.104448	−7.154842
O	7.006143	−15.064810	−5.550310
C	8.412046	−15.181707	−5.188540
C	8.614289	−14.700770	−3.765293
O	8.411077	−13.257003	−3.697931
C	7.415590	−12.939371	−2.696981
C	7.667138	−15.334198	−2.720867
C	6.632243	−14.235734	−2.456051
O	8.491161	−15.667911	−1.563221
H	9.015660	−14.574567	−5.872620
H	8.725110	−16.229134	−5.267427
H	9.651710	−14.916929	−3.484476
H	7.905523	−12.588260	−1.786114
H	7.201878	−16.252012	−3.086357
H	5.820407	−14.354823	−3.175936
H	6.209303	−14.292208	−1.454551
N	6.620210	−11.800079	−3.169902
C	6.872961	−10.537852	−2.598610
O	7.726187	−10.351530	−1.729345
N	6.071997	−9.523422	−3.076664
C	5.099094	−9.616269	−4.061744
O	4.462446	−8.601086	−4.413578
C	4.897555	−10.941541	−4.627908
C	3.836320	−11.123100	−5.677127
C	5.678388	−11.953597	−4.168636
H	6.236352	−8.567038	−2.643401
H	2.860796	−10.799377	−5.294848
H	3.757091	−12.169150	−5.982373
H	4.048642	−10.511094	−6.561598
H	5.601804	−12.956450	−4.572051
P	7.859994	−16.160020	−0.132033
O	6.428423	−16.634436	−0.268210
O	8.854332	−17.140681	0.462110
O	7.823445	−14.771938	0.735088
C	9.100251	−14.141725	1.050137
C	8.882015	−12.960808	1.977423
O	8.200116	−11.891911	1.256194
C	7.050804	−11.445820	2.011868
C	8.044514	−13.256957	3.241996
C	6.684075	−12.618906	2.934275
O	8.716399	−12.614935	4.358397
H	9.573210	−13.799583	0.122544
H	9.756539	−14.871075	1.538481
H	9.871722	−12.597878	2.284438
H	7.295762	−10.542941	2.573162
H	7.960357	−14.332242	3.427915
H	6.054572	−13.342886	2.409574
H	6.160726	−12.289119	3.835172
H	8.196642	−12.803712	5.161220

N	5.996943	−11.056558	1.067080
C	5.571428	−9.700361	0.984940
O	6.109642	−8.840737	1.718406
N	4.575305	−9.392353	0.113529
C	4.026624	−10.332944	−0.680261
N	3.046874	−9.956422	−1.515156
C	4.482772	−11.692594	−0.651527
C	5.466004	−12.000516	0.230936
H	2.733738	−8.973372	−1.547377
H	2.610987	−10.638425	−2.121342
H	4.064705	−12.441995	−1.312670
H	5.894628	−12.992580	0.312484
Na	4.749381	−17.848908	−7.501027
Na	7.064396	−18.782993	0.683071
Na	11.378713	1.734129	4.977165
Na	8.444284	2.079903	2.385914

d(GAG)$^{Na+}$

E: −27516.99 kcal mol^{-1}

O	0.182865	0.377886	−0.909882
C	0.311940	0.796912	0.470075
C	1.634735	0.309328	1.048491
O	1.637318	−1.152912	1.160960
C	2.711294	−1.704282	0.354069
C	2.891975	0.674622	0.226323
C	3.121939	−0.588230	−0.606688
O	3.954613	1.004990	1.189400
H	−0.717875	0.602110	−1.201898
H	−0.506985	0.384960	1.074027
H	0.285118	1.893918	0.546824
H	1.728122	0.723523	2.058568
H	3.540590	−1.992452	0.999072
H	2.740195	1.560316	−0.394493
H	2.439174	−0.551697	−1.459104
H	4.141755	−0.694480	−0.970706
N	2.286614	−2.940170	−0.278321
C	1.357911	−3.144808	−1.296792
N	1.278609	−4.413005	−1.661114
C	2.201703	−5.083612	−0.851681
C	2.582632	−6.456974	−0.788235
O	2.133937	−7.411762	−1.469874
N	3.590797	−6.683720	0.172073
C	4.136360	−5.714995	0.993966
N	5.064953	−6.105346	1.901352
N	3.767541	−4.433542	0.946193
C	2.830234	−4.181107	0.016544
H	0.773683	−2.327560	−1.698291
H	3.960122	−7.660394	0.226198
H	5.483319	−7.042442	1.828977
H	5.647884	−5.352437	2.262981
P	5.533317	0.587704	1.060232
O	6.029283	0.637209	−0.364049
O	6.244255	1.388970	2.137510
O	5.533740	−1.005642	1.446115
C	5.511317	−1.532255	2.795943
C	6.824988	−2.258705	3.085785
O	6.957132	−3.503493	2.337045
C	7.834813	−3.322089	1.192568
C	8.038088	−1.410118	2.691939
C	8.402592	−1.896027	1.278796
O	9.094753	−1.620763	3.677557
H	4.675527	−2.234978	2.863563
H	5.377214	−0.724509	3.523188
H	6.853396	−2.520524	4.148353
H	8.618296	−4.078501	1.261190
H	7.773375	−0.355340	2.706896
H	7.944354	−1.236751	0.541738
H	9.479336	−1.904548	1.117616
N	7.116448	−3.621518	−0.047804
C	6.286122	−2.813756	−0.824985
N	5.650735	−3.488263	−1.767491
C	6.066332	−4.809297	−1.611147
C	5.737365	−6.007131	−2.287036
N	4.854211	−6.094314	−3.294573
N	6.369109	−7.141626	−1.874022
C	7.244673	−7.100743	−0.854634
N	7.604926	−6.028912	−0.133565
C	6.984361	−4.909920	−0.554094
H	6.205105	−1.751088	−0.655126
H	4.638531	−7.007754	−3.711037
H	4.377372	−5.263852	−3.617223
H	7.706445	−8.052955	−0.604678
P	10.448974	−0.709046	3.585043
O	10.159860	0.648450	2.958781
O	11.073333	−0.698917	4.963947
O	11.381229	−1.521489	2.516578
C	11.787934	−2.879709	2.872973
C	12.233900	−3.640548	1.640515
O	11.097560	−3.879221	0.768280
C	11.628050	−4.051826	−0.569984
C	13.333585	−2.967493	0.771904
C	12.768079	−3.022101	−0.662403
O	14.545742	−3.741734	0.950289
H	10.948251	−3.403152	3.342083
H	12.619156	−2.829116	3.584724
H	12.632618	−4.607005	1.985488
H	11.991660	−5.078168	−0.697999
H	13.512121	−1.935096	1.084810
H	12.366497	−2.044680	−0.941699
H	13.527876	−3.307575	−1.393743
H	15.279748	−3.236791	0.555571
N	10.557215	−3.870795	−1.529851
C	9.926718	−2.669636	−1.868520
N	9.034553	−2.816720	−2.825427
C	9.066341	−4.174128	−3.146685
C	8.322408	−4.917954	−4.109231
O	7.447948	−4.488279	−4.898624
N	8.661327	−6.283066	−4.099935
C	9.602176	−6.860502	−3.270630
N	9.803360	−8.186535	−3.412708
N	10.306193	−6.162882	−2.368438
C	10.001418	−4.849830	−2.348943
H	10.167940	−1.741982	−1.370116
H	8.134319	−6.904682	−4.756262
H	9.222852	−8.762172	−4.038847
H	10.357311	−8.652434	−2.705744
O	6.133130	−11.503333	−10.641523
C	7.424444	−12.148491	−10.772157
C	7.887209	−12.675902	−9.419630
O	8.303688	−11.583875	−8.537204
C	7.459126	−11.506743	−7.361163
C	6.824219	−13.478145	−8.643996
C	6.299831	−12.487955	−7.600116
O	7.497955	−14.638231	−8.065673
H	5.953781	−11.024232	−11.469313
H	8.171466	−11.441240	−11.152951
H	7.357404	−12.997868	−11.467218
H	8.764986	−13.307785	−9.593610
H	8.045081	−11.762409	−6.477348
H	6.025192	−13.840166	−9.293979
H	5.426663	−11.978844	−8.009826
H	5.998666	−12.988709	−6.684264
N	7.037223	−10.105657	−7.160066
C	7.613971	−9.325497	−6.112325
O	8.438980	−9.855668	−5.335273

N	7.232460	−8.025124	−5.989869
C	6.343649	−7.476705	−6.843038
N	5.992616	−6.195582	−6.658520
C	5.793263	−8.229344	−7.932301
C	6.182390	−9.521654	−8.062079
H	6.464424	−5.611169	−5.952049
H	5.367027	−5.752307	−7.318844
H	5.114079	−7.775445	−8.644735
H	5.868541	−10.144700	−8.892398
P	6.716347	−15.671695	−7.058504
O	5.212190	−15.519386	−7.139710
O	7.285225	−17.053071	−7.328995
O	7.166573	−15.140770	−5.578023
C	8.581623	−15.203690	−5.241267
C	8.797245	−14.676216	−3.835718
O	8.518913	−13.243908	−3.800184
C	7.550041	−12.954327	−2.764111
C	7.917970	−15.328040	−2.743175
C	6.833777	−14.279104	−2.480600
O	8.795736	−15.580612	−1.603491
H	9.153021	−14.594912	−5.951081
H	8.927525	−16.242356	−5.299771
H	9.852552	−14.831471	−3.581789
H	8.061889	−12.574644	−1.876780
H	7.494930	−16.282172	−3.064829
H	6.020352	−14.455439	−3.186799
H	6.426720	−14.334350	−1.472191
N	6.688817	−11.855697	−3.209875
C	6.927028	−10.573918	−2.673341
O	7.822110	−10.341690	−1.860282
N	6.053597	−9.602641	−3.109851
C	5.028044	−9.746062	−4.030993
O	4.323045	−8.764812	−4.348636
C	4.850961	−11.084778	−4.573174
C	3.745772	−11.322917	−5.563986
C	5.693809	−12.059837	−4.144263
H	6.181786	−8.645218	−2.665301
H	2.773117	−11.069390	−5.125255
H	3.719211	−12.367908	−5.882803
H	3.869370	−10.685734	−6.447795
H	5.626503	−13.071569	−4.525116
P	8.244972	−15.965616	−0.108848
O	6.823660	−16.488020	−0.133170
O	9.295228	−16.864555	0.516610
O	8.204044	−14.511438	0.641839
C	9.470647	−13.822857	0.861656
C	9.275411	−12.676527	1.837913
O	8.493093	−11.616715	1.210823
C	7.339734	−11.301769	2.024475
C	8.549782	−13.049318	3.148920
C	7.125293	−12.521787	2.934662
O	9.236503	−12.360871	4.227753
H	9.841878	−13.436775	−0.094561
H	10.202712	−14.527226	1.272426
H	10.268538	−12.275622	2.077441
H	7.522435	−10.390507	2.595334
H	8.566260	−14.129381	3.326498
H	6.528421	−13.285059	2.428354
H	6.630259	−12.254931	3.871711
H	8.777448	−12.584395	5.058110
N	6.212954	−10.993245	1.132550
C	5.661130	−9.681116	1.104487
O	6.127956	−8.800166	1.861898
N	4.631529	−9.434640	0.252629
C	4.150893	−10.397991	−0.557841
N	3.134393	−10.081557	−1.374252
C	4.712124	−11.718189	−0.562481
C	5.742341	−11.960826	0.286527
H	2.779523	−9.113305	−1.419763
H	2.766281	−10.775223	−2.011261
H	4.340918	−12.485953	−1.230653
H	6.258271	−12.913214	0.330383
Na	5.009693	−17.865492	−7.725330
Na	7.568721	−18.522715	0.975096
Na	11.223479	1.687131	4.979273
Na	8.450255	1.973006	2.215811

d(GCA)$^{Na+}$

E: −27511.19 kcal mol^{-1}

O	−0.278974	−0.245146	−0.570685
C	−0.244678	0.210327	0.803611
C	1.169021	0.112707	1.364501
O	1.572482	−1.280510	1.574207
C	2.626360	−1.654839	0.651759
C	2.269630	0.731162	0.484537
C	2.797105	−0.466597	−0.308492
O	3.249562	1.316664	1.399592
H	−1.212366	−0.300273	−0.840236
H	−0.911356	−0.398851	1.427050
H	−0.566026	1.260203	0.868512
H	1.165920	0.598072	2.346101
H	3.536418	−1.865028	1.222143
H	1.893982	1.524370	−0.165741
H	2.169273	−0.593167	−1.191829
H	3.825552	−0.332496	−0.636190
N	2.285291	−2.908737	−0.012838
C	1.342205	−3.112827	−1.022595
N	1.284459	−4.371868	−1.414720
C	2.225852	−5.041314	−0.630477
C	2.628227	−6.408935	−0.617917
O	2.196170	−7.342879	−1.338259
N	3.631866	−6.656500	0.336434
C	4.164553	−5.709407	1.188614
N	5.072315	−6.136899	2.092437
N	3.800882	−4.422434	1.167799
C	2.851200	−4.149658	0.253498
H	0.722939	−2.302013	−1.380152
H	3.994680	−7.636389	0.385716
H	5.441021	−7.098043	2.065875
H	5.612795	−5.422217	2.563761
P	4.749987	1.777607	0.933685
O	4.839223	2.037068	−0.555331
O	5.153591	2.913321	1.855829
O	5.651498	0.442626	1.232788
C	5.813452	0.016361	2.616882
C	6.941964	−0.995561	2.729695
O	6.566689	−2.260458	2.103543
C	7.487806	−2.578763	1.028195
C	8.273759	−0.572070	2.076379
C	8.231867	−1.275662	0.718707
O	9.334030	−1.053199	2.963280
H	4.878760	−0.435577	2.968055
H	6.046954	0.885993	3.241479
H	7.103680	−1.181650	3.797813
H	8.165345	−3.371831	1.346040
H	8.371932	0.511921	1.982811
H	7.660820	−0.647848	0.031421
H	9.218029	−1.439968	0.290070
N	6.731893	−3.141378	−0.095143
C	6.859133	−4.526458	−0.400872
O	7.630822	−5.231941	0.279469
N	6.126834	−5.025675	−1.432080
C	5.288723	−4.242864	−2.139201
N	4.603434	−4.801132	−3.146406
C	5.126819	−2.852956	−1.823925

C	5.856837	−2.356508	−0.793723
H	4.708599	−5.805406	−3.366362
H	3.937384	−4.246681	−3.667703
H	4.433761	−2.228549	−2.374365
H	5.775072	−1.329616	−0.458089
P	10.898948	−1.202475	2.505578
O	11.245956	−0.300525	1.339689
O	11.727096	−1.042874	3.766967
O	10.981789	−2.737798	1.947798
C	10.907003	−3.849212	2.887378
C	11.746048	−5.021530	2.393185
O	11.103478	−5.715812	1.281173
C	11.831651	−5.497579	0.050151
C	13.150123	−4.640864	1.894927
C	12.961898	−4.502156	0.376878
O	14.038600	−5.735270	2.242666
H	9.861253	−4.159798	2.988619
H	11.285996	−3.527002	3.863105
H	11.821189	−5.738990	3.218716
H	12.211163	−6.460186	−0.303265
H	13.505216	−3.710455	2.350388
H	12.659265	−3.480100	0.142022
H	13.875303	−4.731235	−0.177120
H	14.921289	−5.521222	1.888846
N	10.908977	−5.036123	−0.985597
C	10.304329	−3.789555	−1.082380
N	9.426540	−3.713480	−2.068416
C	9.434632	−4.981655	−2.647140
C	8.706260	−5.541379	−3.722810
N	7.800556	−4.859807	−4.446896
N	8.956903	−6.844220	−4.037043
C	9.871231	−7.536705	−3.330919
N	10.612725	−7.105266	−2.297296
C	10.348225	−5.819704	−1.988646
H	10.545549	−3.001397	−0.384698
H	7.252318	−5.340865	−5.171702
H	7.541723	−3.925529	−4.159992
H	10.005830	−8.571762	−3.639203
O	5.737683	−11.551449	−10.706354
C	6.943060	−12.315891	−10.957943
C	7.531129	−12.825521	−9.647898
O	8.100821	−11.726686	−8.862957
C	7.391121	−11.579107	−7.605509
C	6.534120	−13.534025	−8.710967
C	6.120771	−12.429126	−7.738213
O	7.271166	−14.625282	−8.071790
H	5.481640	−11.120194	−11.540194
H	7.693914	−11.693688	−11.461009
H	6.720528	−13.183183	−11.596148
H	8.348647	−13.510403	−9.897732
H	8.027965	−11.916411	−6.785896
H	5.682735	−13.960519	−9.245859
H	5.308834	−11.856638	−8.190376
H	5.773002	−12.817622	−6.784363
N	7.150626	−10.152132	−7.350528
C	7.852764	−9.523892	−6.308252
O	8.694892	−10.098336	−5.612340
N	7.519545	−8.197552	−6.117495
C	6.627012	−7.440596	−6.867095
O	6.436743	−6.235020	−6.587793
C	5.983850	−8.132861	−7.971017
C	5.035470	−7.376158	−8.860886
C	6.285853	−9.442736	−8.162358
H	8.035855	−7.710567	−5.340236
H	5.535134	−6.518546	−9.328436
H	4.190938	−6.979841	−8.283396
H	4.643465	−8.024583	−9.649881
H	5.877749	−10.008669	−8.994017
P	6.661498	−15.486077	−6.817754
O	5.155003	−15.365009	−6.722775
O	7.231936	−16.887197	−6.940499
O	7.280321	−14.723047	−5.507278
C	8.713358	−14.822773	−5.261577
C	9.041250	−14.433568	−3.827345
O	8.909189	−12.993198	−3.625432
C	7.851930	−12.705638	−2.677986
C	8.158626	−15.096953	−2.752744
C	7.100509	−14.029402	−2.468905
O	9.032195	−15.383122	−1.615739
H	9.246098	−14.158297	−5.951576
H	9.039202	−15.854718	−5.433819
H	10.091189	−14.695835	−3.654861
H	8.293148	−12.326712	−1.750315
H	7.717595	−16.036682	−3.092847
H	6.304494	−14.139308	−3.207731
H	6.662771	−14.118045	−1.476327
N	7.014172	−11.628908	−3.181814
C	6.066191	−11.699127	−4.201333
N	5.449282	−10.550360	−4.401735
C	6.010679	−9.670013	−3.473722
C	5.716565	−8.305149	−3.181920
O	4.892688	−7.558667	−3.761050
N	6.472642	−7.822590	−2.097944
C	7.418457	−8.551734	−1.401989
N	8.028929	−7.938840	−0.373145
N	7.723690	−9.820675	−1.701032
C	6.990723	−10.322155	−2.710941
H	5.900414	−12.616858	−4.745010
H	6.323676	−6.819498	−1.834280
H	7.873834	−6.943162	−0.149028
H	8.752555	−8.455472	0.108400
P	8.463102	−15.769768	−0.128423
O	7.052529	−16.319306	−0.179494
O	9.519032	−16.646966	0.517216
O	8.375969	−14.316691	0.620768
C	9.618848	−13.609359	0.906117
C	9.379066	−12.540483	1.959426
O	8.581775	−11.452221	1.402217
C	7.385992	−11.254298	2.191000
C	8.641542	−13.026645	3.224618
C	7.201571	−12.542958	3.008201
O	9.274687	−12.381746	4.361928
H	9.989812	−13.146545	−0.015529
H	10.366170	−14.319705	1.276748
H	10.358097	−12.134789	2.243943
H	7.499749	−10.376629	2.828126
H	8.698791	−14.114488	3.333489
H	6.649412	−13.291654	2.433899
H	6.673340	−12.361720	3.947691
H	8.802933	−12.671334	5.164316
N	6.276762	−10.940819	1.276995
C	5.642260	−9.670859	1.333697
O	5.980211	−8.850820	2.218717
N	4.678037	−9.392402	0.417278
C	4.309208	−10.302108	−0.507262
N	3.347516	−9.958940	−1.377313
C	4.926664	−11.594177	−0.567376
C	5.910797	−11.857517	0.327822
H	2.948362	−9.007613	−1.372655
H	3.110368	−10.587445	−2.133853
H	4.639496	−12.319985	−1.318350
H	6.472085	−12.784798	0.332451
Na	4.972535	−17.788718	−6.894053
Na	7.804865	−18.346140	0.918034
Na	13.014335	0.705889	2.687704
Na	5.656452	4.301869	−0.057421

d(GCG)$^{Na+}$

E: −27421.18 kcal mol^{-1}

O	−0.271369	−0.435701	−0.728906
C	−0.237888	0.005550	0.649967
C	1.182111	−0.059145	1.199494
O	1.625235	−1.441423	1.399854
C	2.678435	−1.785734	0.464107
C	2.259997	0.593510	0.316060
C	2.816742	−0.585293	−0.486211
O	3.224328	1.205918	1.229862
H	−1.205117	−0.510062	−0.992586
H	−0.883255	−0.627461	1.272005
H	−0.586359	1.045831	0.728332
H	1.173217	0.420651	2.183788
H	3.597389	−1.983743	1.024465
H	1.858472	1.378147	−0.329296
H	2.190794	−0.720561	−1.369766
H	3.840845	−0.423040	−0.814774
N	2.353300	−3.040342	−0.207450
C	1.389609	−3.254711	−1.195297
N	1.321466	−4.518234	−1.570888
C	2.276426	−5.180131	−0.796809
C	2.661570	−6.552132	−0.762342
O	2.198839	−7.496547	−1.449250
N	3.683025	−6.791238	0.174900
C	4.240888	−5.833590	0.999283
N	5.164300	−6.253746	1.890266
N	3.886319	−4.544287	0.964504
C	2.921860	−4.279356	0.063214
H	0.763934	−2.447349	−1.549261
H	4.024348	−7.777188	0.248210
H	5.512897	−7.222489	1.883622
H	5.724015	−5.537961	2.336637
P	4.724932	1.679214	0.779832
O	4.832107	1.936947	−0.708269
O	5.105152	2.821109	1.704211
O	5.633845	0.353262	1.093034
C	5.772849	−0.078921	2.477677
C	6.940821	−1.042997	2.614605
O	6.635879	−2.322966	1.982895
C	7.571168	−2.590479	0.903969
C	8.263788	−0.558383	1.987871
C	8.283477	−1.261131	0.628687
O	9.328215	−0.983684	2.897218
H	4.848050	−0.573135	2.796376
H	5.950727	0.793574	3.116440
H	7.088151	−1.222889	3.685825
H	8.266654	−3.373244	1.208679
H	8.310224	0.529145	1.893434
H	7.719546	−0.646823	−0.076009
H	9.286868	−1.394794	0.233181
N	6.833754	−3.149275	−0.235162
C	6.957169	−4.536566	−0.536435
O	7.729821	−5.240514	0.145553
N	6.218841	−5.041166	−1.560757
C	5.388833	−4.258085	−2.276372
N	4.689529	−4.818548	−3.272459
C	5.236014	−2.865002	−1.971522
C	5.960795	−2.366098	−0.939427
H	4.758555	−5.830478	−3.469151
H	4.027340	−4.259491	−3.793446
H	4.546769	−2.240852	−2.527164
H	5.874168	−1.338827	−0.606663
P	10.912989	−1.023571	2.485524
O	11.224554	−0.131516	1.301776
O	11.691550	−0.763550	3.762223
O	11.123800	−2.561913	1.979205
C	11.035206	−3.650540	2.942095
C	11.831014	−4.851241	2.448262
O	11.169932	−5.502587	1.323065
C	11.927798	−5.310020	0.102072
C	13.255546	−4.525506	1.968221
C	13.088625	−4.360090	0.451168
O	14.090950	−5.663908	2.309179
H	9.983636	−3.930962	3.070060
H	11.441594	−3.321488	3.904972
H	11.868578	−5.577611	3.268936
H	12.278298	−6.287221	−0.240995
H	13.647782	−3.618446	2.440194
H	12.824634	−3.324864	0.228535
H	13.998641	−4.614496	−0.097622
H	14.983589	−5.489210	1.958717
N	11.045796	−4.817366	−0.949540
C	10.516880	−3.534421	−1.090660
N	9.659226	−3.443032	−2.088036
C	9.598878	−4.727478	−2.632785
C	8.822753	−5.252161	−3.706392
O	8.006827	−4.640921	−4.438630
N	9.055491	−6.628686	−3.900864
C	9.951125	−7.392416	−3.179955
N	10.095462	−8.683465	−3.553943
N	10.676132	−6.903186	−2.168271
C	10.452598	−5.595345	−1.934161
H	10.801410	−2.739105	−0.418619
H	8.496127	−7.096219	−4.653272
H	9.468201	−9.115702	−4.251208
H	10.580056	−9.291039	−2.905189
O	5.617942	−11.188446	−10.681889
C	6.826163	−11.944313	−10.944441
C	7.406773	−12.487665	−9.644202
O	7.957897	−11.408564	−8.822140
C	7.229358	−11.303095	−7.567090
C	6.408957	−13.233120	−8.737538
C	5.969020	−12.158651	−7.742595
O	7.157767	−14.328091	−8.117364
H	5.373611	−10.726539	−11.502785
H	7.579711	−11.307790	−11.425116
H	6.610408	−12.794624	−11.607671
H	8.232247	−13.158016	−9.907787
H	7.858981	−11.664088	−6.753095
H	5.570604	−13.659700	−9.292761
H	5.158064	−11.584012	−8.193787
H	5.610073	−12.573869	−6.804615
N	6.986237	−9.885143	−7.271654
C	7.726122	−9.249882	−6.233704
O	8.571365	−9.913239	−5.592505
N	7.484895	−7.937629	−5.978414
C	6.584534	−7.240617	−6.700694
N	6.390368	−5.950406	−6.392214
C	5.870957	−7.849008	−7.786517
C	6.113670	−9.159363	−8.041798
H	6.926221	−5.498035	−5.635941
H	5.720852	−5.404592	−6.918300
H	5.180288	−7.278527	−8.396831
H	5.659864	−9.688951	−8.872467
P	6.547727	−15.256990	−6.913963
O	5.043245	−15.126732	−6.799285
O	7.101431	−16.655116	−7.121905
O	7.183580	−14.582070	−5.565162
C	8.616293	−14.704871	−5.332625
C	8.951769	−14.425803	−3.873787
O	8.830280	−13.006488	−3.555159
C	7.720269	−12.777026	−2.652014
C	8.071607	−15.162051	−2.846988
C	6.996881	−14.126124	−2.509316
O	8.943968	−15.506731	−1.724624
H	9.148340	−13.990683	−5.971687

H	8.938037	−15.721851	−5.583942
H	10.001897	−14.702447	−3.728172
H	8.114892	−12.410393	−1.699546
H	7.644404	−16.085833	−3.244362
H	6.199688	−14.216986	−3.248700
H	6.564313	−14.272440	−1.521518
N	6.873249	−11.707615	−3.159711
C	5.929597	−11.772412	−4.184854
N	5.328083	−10.617136	−4.394822
C	5.898155	−9.736664	−3.472988
C	5.642524	−8.358376	−3.212160
O	4.824606	−7.605513	−3.791035
N	6.439820	−7.864473	−2.161623
C	7.358360	−8.607943	−1.443681
N	8.014005	−7.978452	−0.453053
N	7.603477	−9.899119	−1.693414
C	6.862945	−10.397494	−2.698132
H	5.758034	−12.689882	−4.727066
H	6.334635	−6.847675	−1.928480
H	7.898994	−6.970833	−0.259001
H	8.720646	−8.506303	0.041006
P	8.387718	−15.933554	−0.244025
O	6.974722	−16.477172	−0.293211
O	9.447874	−16.832146	0.364511
O	8.313916	−14.502372	0.548023
C	9.562210	−13.799665	0.819856
C	9.333347	−12.719897	1.864159
O	8.525773	−11.642036	1.303170
C	7.346679	−11.427578	2.113592
C	8.614785	−13.192086	3.145726
C	7.173029	−12.705129	2.950084
O	9.269628	−12.540420	4.266786
H	9.930849	−13.347368	−0.107947
H	10.308153	−14.510559	1.192544
H	10.315132	−12.308699	2.131166
H	7.478981	−10.543179	2.737735
H	8.669739	−14.279164	3.263125
H	6.607596	−13.459675	2.397023
H	6.663245	−12.510045	3.896941
H	8.811452	−12.822925	5.079488
N	6.221604	−11.118083	1.219040
C	5.624454	−9.828616	1.243115
O	6.006804	−8.987105	2.088408
N	4.645466	−9.555514	0.340205
C	4.230977	−10.486132	−0.543058
N	3.251471	−10.150664	−1.396404
C	4.817031	−11.793711	−0.576109
C	5.814090	−12.053208	0.305595
H	2.885982	−9.185934	−1.422584
H	2.989024	−10.793973	−2.131860
H	4.496498	−12.535142	−1.297816
H	6.353091	−12.993287	0.325325
Na	4.829094	−17.528620	−7.122126
Na	7.736709	−18.532442	0.751466
Na	12.850072	1.067481	2.655148
Na	5.624339	4.212304	−0.193912

d(GGA)$^{\mathrm{Na^+}}$

E: −27511.92 kcal mol^{-1}

O	−0.241998	0.238198	−0.843560
C	−0.219485	0.725660	0.519935
C	1.124676	0.428150	1.174752
O	1.308160	−1.013487	1.364387
C	2.420444	−1.482900	0.556758
C	2.368226	0.899278	0.394283
C	2.745099	−0.343244	−0.411997
O	3.361657	1.293491	1.402775
H	−1.158995	0.301197	−1.162984
H	−1.007057	0.241942	1.111816
H	−0.381194	1.813362	0.544965
H	1.116539	0.894146	2.166095
H	3.267477	−1.710717	1.207089
H	2.170774	1.769933	−0.235270
H	2.092698	−0.389071	−1.286434
H	3.781044	−0.349504	−0.745846
N	2.080034	−2.743208	−0.078891
C	1.158780	−2.996267	−1.094388
N	1.138888	−4.268319	−1.452059
C	2.093335	−4.890287	−0.640568
C	2.538569	−6.242321	−0.572394
O	2.134300	−7.218894	−1.252483
N	3.554302	−6.419720	0.389984
C	4.055833	−5.422215	1.205588
N	5.000829	−5.763193	2.114365
N	3.628562	−4.159094	1.151274
C	2.680439	−3.955315	0.222135
H	0.533782	−2.208009	−1.492662
H	3.970888	−7.378083	0.446072
H	5.431913	−6.696232	2.085967
H	5.548903	−4.986254	2.476120
P	4.981801	1.195749	1.214187
O	5.413666	1.454465	−0.215948
O	5.588605	2.087826	2.280458
O	5.317483	−0.376518	1.509060
C	5.451093	−0.887260	2.865435
C	6.756619	−1.663039	3.026412
O	6.722211	−2.950569	2.340548
C	7.527172	−2.899580	1.131676
C	7.999970	−0.946628	2.469173
C	8.076788	−1.470890	1.030828
O	9.123146	−1.333638	3.325442
H	4.606881	−1.555286	3.064041
H	5.439647	−0.055824	3.577122
H	6.884289	−1.873041	4.093623
H	8.321215	−3.643641	1.229836
H	7.909558	0.141935	2.510008
H	7.432616	−0.845891	0.412029
H	9.083191	−1.437732	0.617632
N	6.743874	−3.326168	−0.018171
C	5.846927	−2.606293	−0.809078
N	5.296295	−3.347470	−1.751903
C	5.840788	−4.623901	−1.578992
C	5.640839	−5.843039	−2.290807
O	4.872444	−6.053391	−3.259952
N	6.443669	−6.885411	−1.786311
C	7.319498	−6.775267	−0.722958
N	8.007903	−7.879882	−0.369200
N	7.481405	−5.642208	−0.034785
C	6.742062	−4.623747	−0.504839
H	5.645018	−1.563025	−0.621396
H	6.408573	−7.790918	−2.313067
H	8.081440	−8.688272	−1.004694
H	8.742479	−7.741638	0.314155
P	10.677909	−1.446499	2.831139
O	11.018991	−0.442832	1.748706
O	11.518183	−1.399495	4.093655
O	10.752994	−2.924842	2.133704
C	10.689644	−4.113442	2.976073
C	11.515052	−5.236666	2.362601
O	10.860376	−5.789596	1.180408
C	11.650268	−5.529935	−0.004964
C	12.926826	−4.817869	1.913628
C	12.753521	−4.543531	0.413520
O	13.809535	−5.941992	2.169066
H	9.644289	−4.427360	3.070927

H	11.088370	−3.878988	3.968733
H	11.582356	−6.039779	3.106424
H	12.058296	−6.476628	−0.371055
H	13.279369	−3.933578	2.454856
H	12.426263	−3.511433	0.273435
H	13.676263	−4.700133	−0.150470
H	14.696850	−5.698413	1.847384
N	10.774870	−5.044462	−1.067237
C	10.172176	−3.796384	−1.172411
N	9.333077	−3.710459	−2.190770
C	9.363654	−4.972295	−2.784500
C	8.664269	−5.532839	−3.880049
N	7.776356	−4.854272	−4.633270
N	8.915799	−6.837797	−4.182137
C	9.800996	−7.535887	−3.446004
N	10.515311	−7.104346	−2.393612
C	10.251994	−5.816524	−2.098216
H	10.387231	−3.009434	−0.463796
H	7.211926	−5.366237	−5.321945
H	7.471105	−3.939349	−4.326824
H	9.929316	−8.575125	−3.739578
O	5.697606	−11.766882	−10.671455
C	6.912031	−12.510690	−10.940720
C	7.533013	−12.995852	−9.636838
O	8.101236	−11.878445	−8.877119
C	7.416482	−11.720180	−7.609790
C	6.561074	−13.706356	−8.675997
C	6.163131	−12.604870	−7.692475
O	7.310436	−14.793669	−8.047357
H	5.420596	−11.341436	−11.501577
H	7.640809	−11.879511	−11.464390
H	6.693072	−13.388193	−11.566016
H	8.355903	−13.671871	−9.892513
H	8.079121	−12.021372	−6.796342
H	5.698745	−14.133819	−9.191668
H	5.316647	−12.054524	−8.107371
H	5.869653	−13.005687	−6.725957
N	7.145151	−10.290386	−7.382400
C	7.861070	−9.615802	−6.378036
O	8.727625	−10.154636	−5.683065
N	7.500870	−8.293457	−6.211796
C	6.567262	−7.577706	−6.953125
O	6.348272	−6.374641	−6.689609
C	5.912230	−8.314825	−8.021134
C	4.916917	−7.605423	−8.898299
C	6.241442	−9.621560	−8.188417
H	8.010768	−7.772729	−5.451172
H	5.378963	−6.746001	−9.399739
H	4.080054	−7.217971	−8.303856
H	4.518869	−8.282213	−9.659768
H	5.824066	−10.218594	−8.993336
P	6.664871	−15.724516	−6.861318
O	5.157984	−15.589914	−6.792713
O	7.225092	−17.121941	−7.053148
O	7.261786	−15.048193	−5.493691
C	8.697185	−15.138216	−5.260394
C	9.054115	−14.532092	−3.913557
O	8.822332	−13.090588	−3.937336
C	7.907282	−12.722419	−2.877047
C	8.270868	−15.076236	−2.697423
C	7.191526	−14.016150	−2.477271
O	9.232678	−15.169514	−1.598036
H	9.227693	−14.594813	−6.050815
H	9.005900	−16.189640	−5.280285
H	10.125129	−14.705548	−3.755280
H	8.464504	−12.292934	−2.043461
H	7.853663	−16.070154	−2.873960
H	6.360832	−14.237837	−3.150440
H	6.811622	−13.995539	−1.456583
N	7.027037	−11.649221	−3.344069
C	7.238774	−10.322436	−2.865102
O	8.196089	−10.099166	−2.099172
N	6.358060	−9.360327	−3.241779
C	5.362727	−9.619389	−4.110358
N	4.546193	−8.612575	−4.448365
C	5.186621	−10.930358	−4.666959
C	6.038075	−11.903381	−4.252691
H	4.666451	−7.674685	−4.028486
H	3.773255	−8.780766	−5.078682
H	4.407350	−11.137257	−5.390389
H	5.988481	−12.919553	−4.623242
P	8.793028	−15.309222	−0.025006
O	7.411324	−15.909342	0.131528
O	9.935659	−16.024156	0.670899
O	8.688983	−13.753890	0.477809
C	9.914184	−12.978893	0.644428
C	9.725071	−11.917508	1.718297
O	8.862912	−10.843217	1.234974
C	7.691330	−10.718844	2.071857
C	9.095210	−12.426751	3.030248
C	7.623904	−12.009722	2.904692
O	9.769310	−11.744636	4.121087
H	10.165324	−12.500193	−0.308600
H	10.729648	−13.647060	0.940392
H	10.711374	−11.485619	1.928669
H	7.771219	−9.827160	2.694227
H	9.208273	−13.510227	3.140086
H	7.070568	−12.786284	2.370158
H	7.149189	−11.847111	3.875594
H	9.352028	−12.036303	4.952396
N	6.524634	−10.492507	1.202932
C	5.777244	−9.287850	1.307229
O	6.049994	−8.466222	2.212100
N	4.772764	−9.077732	0.415634
C	4.456775	−10.004478	−0.511378
N	3.449586	−9.733222	−1.354230
C	5.173778	−11.242133	−0.602225
C	6.209713	−11.427537	0.253892
H	2.972696	−8.818072	−1.327049
H	3.212440	−10.393826	−2.082071
H	4.918725	−11.985478	−1.347358
H	6.849765	−12.302261	0.226681
Na	4.948772	−17.999204	−7.098958
Na	8.363171	−17.688400	1.496570
Na	12.816774	0.411790	3.177330
Na	6.864669	3.227904	0.564901

d(GGC)$^{Na+}$

E: −27423.98 kcal mol^{-1}

O	−0.266198	0.011393	−0.677663
C	−0.236265	0.473955	0.694255
C	1.149874	0.275980	1.296887
O	1.449830	−1.145799	1.487389
C	2.526209	−1.558917	0.605364
C	2.321456	0.833687	0.466507
C	2.770353	−0.381267	−0.346334
O	3.314199	1.319544	1.430228
H	−1.195690	0.002518	−0.965578
H	−0.962072	−0.081487	1.301789
H	−0.483405	1.544584	0.745904
H	1.146130	0.743903	2.287140
H	3.407905	−1.792588	1.208688
H	2.028775	1.674705	−0.166403
H	2.122235	−0.457441	−1.221042
H	3.801389	−0.315336	−0.687513

N	2.176348	−2.803020	−0.064979
C	1.276278	−2.998165	−1.113297
N	1.236949	−4.254042	−1.519460
C	2.152119	−4.928003	−0.706682
C	2.566047	−6.291045	−0.691674
O	2.165697	−7.223792	−1.432229
N	3.543419	−6.537016	0.293083
C	4.030121	−5.593498	1.177210
N	4.916295	−6.015594	2.109259
N	3.642526	−4.315627	1.164641
C	2.734753	−4.042433	0.210743
H	0.674131	−2.184047	−1.492699
H	3.929063	−7.509367	0.329134
H	5.330476	−6.956755	2.053701
H	5.436971	−5.287216	2.584654
P	4.911239	1.479209	1.116991
O	5.194297	1.729782	−0.349974
O	5.433912	2.509261	2.100642
O	5.513096	−0.004265	1.455452
C	5.627777	−0.442452	2.839841
C	6.858877	−1.323429	3.017510
O	6.678659	−2.639883	2.411389
C	7.514019	−2.777455	1.235933
C	8.145516	−0.752218	2.394305
C	8.179103	−1.409696	1.012185
O	9.246292	−1.153545	3.269024
H	4.728418	−1.006002	3.111225
H	5.719464	0.431148	3.493762
H	6.997509	−1.479186	4.092818
H	8.244183	−3.571502	1.418958
H	8.134305	0.338791	2.332859
H	7.585695	−0.798241	0.331286
H	9.186542	−1.492813	0.609386
N	6.720428	−3.238295	0.104627
C	5.830151	−2.507882	−0.684966
N	5.288460	−3.235814	−1.642444
C	5.832686	−4.512589	−1.483524
C	5.650622	−5.712904	−2.232135
O	4.899698	−5.896731	−3.219240
N	6.445600	−6.766516	−1.742024
C	7.315834	−6.676736	−0.671003
N	8.019308	−7.777789	−0.355990
N	7.471576	−5.556730	0.043905
C	6.726081	−4.530760	−0.401496
H	5.628629	−1.467485	−0.479867
H	6.383621	−7.674948	−2.259777
H	7.993517	−8.635023	−0.929557
H	8.689302	−7.696919	0.396994
P	10.818211	−1.070181	2.813406
O	11.043641	−0.042853	1.723459
O	11.621130	−0.905047	4.090216
O	11.088299	−2.539146	2.140927
C	11.054069	−3.710845	3.009081
C	11.691472	−4.899024	2.307941
O	10.834709	−5.360332	1.220412
C	11.579469	−5.394530	−0.018868
C	13.082181	−4.628636	1.694396
C	12.790711	−4.473991	0.196292
O	13.906481	−5.790825	1.978470
H	10.012924	−3.943133	3.261165
H	11.606013	−3.496986	3.931043
H	11.774315	−5.708223	3.044842
H	11.876650	−6.418023	−0.250784
H	13.544268	−3.731500	2.118574
H	12.530136	−3.433805	−0.017614
H	13.640833	−4.758254	−0.428844
H	14.783413	−5.632822	1.583147
N	10.679629	−4.983789	−1.106820
C	10.332706	−5.901327	−2.135110
O	10.859722	−7.037567	−2.151397
N	9.434436	−5.504237	−3.075157
C	8.899311	−4.266604	−3.050901
N	8.024036	−3.941040	−4.013811
C	9.247786	−3.324792	−2.027902
C	10.121890	−3.733948	−1.075543
H	7.718129	−4.643705	−4.704809
H	7.538711	−3.054419	−3.968333
H	8.805614	−2.336439	−1.999639
H	10.410801	−3.114939	−0.234359
O	5.729404	−11.493960	−10.841512
C	6.874745	−12.345328	−11.087652
C	7.409175	−12.911302	−9.776930
O	8.035976	−11.868939	−8.958509
C	7.263080	−11.631244	−7.754575
C	6.355102	−13.568115	−8.868218
C	5.965406	−12.438029	−7.915277
O	7.020337	−14.689951	−8.204715
H	5.502604	−11.054704	−11.679735
H	7.676702	−11.778347	−11.577164
H	6.595086	−13.188836	−11.735540
H	8.187806	−13.639879	−10.026011
H	7.849949	−11.953254	−6.888486
H	5.499683	−13.956363	−9.425623
H	5.195257	−11.834148	−8.397701
H	5.571884	−12.804879	−6.970109
N	7.050675	−10.199528	−7.573678
C	6.175873	−9.369428	−8.278293
N	6.205988	−8.121574	−7.849419
C	7.144459	−8.114133	−6.815588
C	7.582223	−7.061390	−5.959047
O	7.201541	−5.865050	−5.958409
N	8.549316	−7.498603	−5.035497
C	9.046873	−8.783954	−4.959702
N	10.030573	−9.010838	−4.059769
N	8.623978	−9.778010	−5.748245
C	7.687704	−9.394595	−6.637033
H	5.588533	−9.747821	−9.103395
H	8.889911	−6.787343	−4.348246
H	10.257397	−8.305865	−3.343289
H	10.217113	−9.982510	−3.842191
P	6.366432	−15.490927	−6.934820
O	4.873138	−15.265910	−6.821650
O	6.840805	−16.928152	−7.039992
O	7.049849	−14.753312	−5.642213
C	8.481530	−14.918736	−5.429263
C	8.858051	−14.460805	−4.030110
O	8.722567	−13.011309	−3.913317
C	7.749847	−12.680349	−2.893018
C	8.012497	−15.064863	−2.889321
C	6.977822	−13.974113	−2.607514
O	8.930862	−15.302778	−1.776333
H	9.028716	−14.323414	−6.169291
H	8.748778	−15.974493	−5.549208
H	9.912874	−14.715383	−3.875951
H	8.262967	−12.305249	−2.006763
H	7.551272	−16.015959	−3.164926
H	6.148496	−14.104991	−3.305560
H	6.582640	−14.016616	−1.594020
N	6.919675	−11.563431	−3.358083
C	7.086152	−10.279500	−2.764022
O	7.956624	−10.126886	−1.884318
N	6.271377	−9.272871	−3.173114
C	5.357035	−9.461216	−4.144305
N	4.572865	−8.428419	−4.477573
C	5.225186	−10.729749	−4.801836
C	6.027734	−11.739742	−4.381028
H	4.687789	−7.509471	−4.018585
H	3.902666	−8.527194	−5.228663

H	4.519924	−10.872567	−5.611328
H	6.018353	−12.724820	−4.832075
P	8.420368	−15.617188	−0.250939
O	7.010502	−16.169805	−0.222405
O	9.503018	−16.461313	0.394354
O	8.356434	−14.131287	0.434335
C	9.606430	−13.422882	0.684698
C	9.404884	−12.365943	1.759082
O	8.611807	−11.256832	1.239224
C	7.422059	−11.068691	2.037498
C	8.690619	−12.857976	3.034568
C	7.250161	−12.361662	2.852446
O	9.352277	−12.228075	4.164124
H	9.941985	−12.947290	−0.243869
H	10.367674	−14.135875	1.019460
H	10.396040	−11.977380	2.024614
H	7.534735	−10.190793	2.674457
H	8.741559	−13.947249	3.132726
H	6.679583	−13.103338	2.287270
H	6.744324	−12.182441	3.804532
H	8.893069	−12.518937	4.973283
N	6.302059	−10.761669	1.132757
C	5.641814	−9.505064	1.209558
O	5.961276	−8.693801	2.108815
N	4.669986	−9.233614	0.298744
C	4.304260	−10.145126	−0.625270
N	3.328222	−9.815162	−1.483705
C	4.937416	−11.429090	−0.695270
C	5.939729	−11.679288	0.183656
H	2.915264	−8.869045	−1.474667
H	3.063405	−10.462376	−2.214286
H	4.646622	−12.158841	−1.440906
H	6.515616	−12.597582	0.176773
Na	4.516707	−17.672781	−6.905340
Na	7.812459	−18.136905	0.952514
Na	12.650555	1.098105	3.162943
Na	6.371033	3.779176	0.265886

d(GGG)$^{Na+}$

E: −27421.74 kcal mol^{-1}

O	−0.334559	0.058775	−0.642418
C	−0.317419	0.506987	0.734411
C	1.066618	0.314829	1.343789
O	1.380497	−1.106716	1.510867
C	2.473338	−1.488422	0.634076
C	2.238784	0.898393	0.532489
C	2.703007	−0.296069	−0.301395
O	3.224314	1.362480	1.513669
H	−1.262110	0.045490	−0.936382
H	−1.041576	−0.061926	1.331391
H	−0.575303	1.574556	0.796330
H	1.050425	0.765778	2.341859
H	3.354861	−1.710336	1.242437
H	1.945441	1.752185	−0.082820
H	2.057643	−0.367881	−1.178632
H	3.734907	−0.210839	−0.636151
N	2.154823	−2.733211	−0.049028
C	1.262822	−2.949299	−1.099852
N	1.249401	−4.207706	−1.501287
C	2.174040	−4.861601	−0.681931
C	2.613355	−6.216758	−0.653080
O	2.233018	−7.164219	−1.385845
N	3.592271	−6.436108	0.337347
C	4.060334	−5.475779	1.213190
N	4.955953	−5.866477	2.150341
N	3.645499	−4.207268	1.189830
C	2.735778	−3.960192	0.232571
H	0.647361	−2.148323	−1.485925
H	4.001291	−7.398816	0.378021
H	5.384838	−6.801340	2.108805
H	5.471880	−5.117328	2.599224
P	4.813160	1.585732	1.191568
O	5.070317	1.882793	−0.271442
O	5.306133	2.612306	2.194943
O	5.472341	0.119497	1.490595
C	5.589349	−0.352367	2.863253
C	6.769723	−1.307259	3.003761
O	6.521207	−2.571381	2.319760
C	7.402641	−2.698018	1.172056
C	8.103154	−0.787580	2.431690
C	8.088153	−1.338850	1.004711
O	9.159328	−1.342658	3.284787
H	4.665727	−0.872120	3.142077
H	5.738624	0.501770	3.532629
H	6.882195	−1.526320	4.071576
H	8.123476	−3.493603	1.371394
H	8.184038	0.301851	2.463506
H	7.479148	−0.670426	0.393837
H	9.078333	−1.416114	0.558364
N	6.656539	−3.149077	0.013354
C	5.767872	−2.447944	−0.800273
N	5.252388	−3.202746	−1.752859
C	5.818850	−4.467702	−1.563800
C	5.668919	−5.690886	−2.280068
O	4.932665	−5.916038	−3.270743
N	6.478232	−6.717681	−1.750421
C	7.327489	−6.583752	−0.666189
N	8.046509	−7.662198	−0.293620
N	7.440647	−5.446651	0.024085
C	6.692033	−4.447574	−0.467756
H	5.549375	−1.405441	−0.622151
H	6.468676	−7.627900	−2.269296
H	8.106084	−8.500670	−0.889929
H	8.786651	−7.482186	0.376390
P	10.677374	−1.693475	2.788170
O	11.166763	−0.743236	1.713816
O	11.516741	−1.787009	4.048435
O	10.527620	−3.158034	2.074440
C	10.389945	−4.362465	2.884534
C	11.284314	−5.475731	2.347640
O	10.743967	−6.065127	1.126134
C	11.567337	−5.714899	−0.015458
C	12.713376	−5.025316	1.998613
C	12.629224	−4.724922	0.495209
O	13.599137	−6.138797	2.288891
H	9.343984	−4.685140	2.854129
H	10.676285	−4.143805	3.918095
H	11.310444	−6.270018	3.102834
H	12.011843	−6.632614	−0.409612
H	13.014512	−4.146086	2.577802
H	12.302969	−3.693435	0.352000
H	13.587459	−4.863428	−0.011455
H	14.499131	−5.875211	2.023283
N	10.724165	−5.198922	−1.087674
C	10.116112	−3.945880	−1.187347
N	9.327922	−3.845460	−2.239503
C	9.392976	−5.093773	−2.863965
C	8.725913	−5.604927	−4.017470
O	7.911050	−5.010944	−4.764507
N	9.067264	−6.948033	−4.272679
C	9.972060	−7.691262	−3.543075
N	10.261393	−8.932822	−3.999912
N	10.586616	−7.220310	−2.453462
C	10.256398	−5.946678	−2.159402
H	10.287069	−3.180496	−0.444598

H	8.576919	−7.409484	−5.072940
H	9.672781	−9.371661	−4.725915
H	10.698728	−9.553547	−3.328962
O	5.534369	−11.828339	−10.860675
C	6.699616	−12.642364	−11.143781
C	7.368325	−13.076618	−9.845636
O	7.989946	−11.933327	−9.171492
C	7.363457	−11.706202	−7.882836
C	6.428805	−13.713718	−8.804145
C	6.076414	−12.541228	−7.889277
O	7.203038	−14.753279	−8.124767
H	5.240962	−11.429808	−11.698567
H	7.426326	−12.076538	−11.740003
H	6.411619	−13.544117	−11.703221
H	8.164794	−13.783310	−10.102617
H	8.045505	−12.011405	−7.088231
H	5.547955	−14.175238	−9.255590
H	5.248237	−11.988235	−8.336915
H	5.775789	−12.866794	−6.895844
N	7.160739	−10.263730	−7.686820
C	7.925172	−9.577175	−6.700732
O	8.802355	−10.202098	−6.064558
N	7.656365	−8.265942	−6.474024
C	6.719686	−7.611876	−7.189851
N	6.478647	−6.329258	−6.883223
C	6.006458	−8.262498	−8.251935
C	6.260743	−9.578837	−8.464701
H	6.972656	−5.869724	−6.103162
H	5.770290	−5.815366	−7.390744
H	5.292568	−7.724109	−8.864460
H	5.789050	−10.151071	−9.257100
P	6.623309	−15.571101	−6.827588
O	5.123789	−15.415083	−6.685193
O	7.162585	−16.985858	−6.929824
O	7.300560	−14.788670	−5.557452
C	8.737162	−14.920241	−5.350018
C	9.128453	−14.394081	−3.977946
O	8.988165	−12.941105	−3.927608
C	8.047046	−12.564707	−2.894578
C	8.310037	−14.944007	−2.790273
C	7.277262	−13.844360	−2.543977
O	9.253413	−15.112903	−1.684045
H	9.267547	−14.349728	−6.121223
H	9.019875	−15.975938	−5.426844
H	10.187370	−14.636408	−3.830846
H	8.582639	−12.157403	−2.036304
H	7.850941	−15.911792	−3.004508
H	6.442061	−14.001933	−3.230018
H	6.890188	−13.843228	−1.526378
N	7.204620	−11.467343	−3.381101
C	7.323600	−10.174486	−2.791669
O	8.173888	−9.988678	−1.899709
N	6.478300	−9.199936	−3.217548
C	5.575522	−9.428777	−4.190884
N	4.760136	−8.426955	−4.542255
C	5.484961	−10.707920	−4.834485
C	6.321340	−11.684833	−4.402684
H	4.828930	−7.502768	−4.081357
H	4.076015	−8.568429	−5.273601
H	4.783558	−10.882979	−5.640874
H	6.346858	−12.675552	−4.840660
P	8.773468	−15.340199	−0.132817
O	7.383089	−15.935037	−0.050368
O	9.891676	−16.106047	0.548064
O	8.666547	−13.818140	0.459496
C	9.889837	−13.063348	0.706354
C	9.656260	−12.031059	1.799318
O	8.828415	−10.937354	1.301531
C	7.621284	−10.817333	2.085613
C	8.957026	−12.569663	3.063900
C	7.498550	−12.128442	2.881022
O	9.587325	−11.929578	4.205592
H	10.192669	−12.560631	−0.218987
H	10.683021	−13.749253	1.021981
H	10.634278	−11.615029	2.071311
H	7.683874	−9.943483	2.734653
H	9.050963	−13.657312	3.146996
H	6.960360	−12.883290	2.301712
H	6.981655	−11.983751	3.833080
H	9.131432	−12.244468	5.007607
N	6.498369	−10.551149	1.170447
C	5.763024	−9.339559	1.272744
O	6.011751	−8.542555	2.206543
N	4.797142	−9.095161	0.348187
C	4.503126	−9.997010	−0.610257
N	3.532569	−9.692435	−1.484675
C	5.203915	−11.243726	−0.698469
C	6.205341	−11.460829	0.190670
H	3.069392	−8.770337	−1.458811
H	3.320594	−10.329935	−2.240520
H	4.967717	−11.967124	−1.469091
H	6.834370	−12.343313	0.169337
Na	4.876956	−17.832833	−6.788621
Na	8.266468	−17.794315	1.240184
Na	13.053027	−0.150959	3.110575
Na	6.164513	3.968486	0.389123

d(GTA)$^{Na+}$

E: −27599.51 kcal mol^{-1}

O	−0.335166	−0.427096	−0.148607
C	−0.191751	−0.115734	1.258477
C	1.274221	−0.180502	1.670092
O	1.767821	−1.560180	1.704632
C	2.735178	−1.788317	0.650102
C	2.241989	0.584962	0.749004
C	2.771046	−0.502550	−0.188591
O	3.257189	1.189150	1.611359
H	−1.285634	−0.518983	−0.336055
H	−0.761064	−0.827080	1.870023
H	−0.560132	0.899504	1.466006
H	1.347405	0.210460	2.690268
H	3.703142	−2.019203	1.105567
H	1.747271	1.390027	0.200823
H	2.090678	−0.577695	−1.037721
H	3.765095	−0.280681	−0.568316
N	2.370295	−2.977590	−0.116492
C	1.334284	−3.110016	−1.045222
N	1.247796	−4.331406	−1.536844
C	2.261779	−5.052255	−0.902896
C	2.646668	−6.419885	−1.019458
O	2.146801	−7.297033	−1.766840
N	3.713685	−6.743334	−0.162166
C	4.316153	−5.865669	0.718160
N	5.277401	−6.366660	1.519335
N	3.974378	−4.574841	0.811822
C	2.960041	−4.231200	−0.004976
H	0.677166	−2.281960	−1.270299
H	4.036638	−7.738120	−0.174068
H	5.596647	−7.342367	1.438000
H	5.847421	−5.703435	2.028132
P	4.728277	1.671857	1.079068
O	4.739249	1.997032	−0.399709
O	5.180629	2.768190	2.025759
O	5.640742	0.327473	1.269146
C	5.866841	−0.161590	2.622714

C	7.086249	−1.065953	2.646073
O	6.804057	−2.320894	1.956860
C	7.709441	−2.493146	0.837811
C	8.342997	−0.473486	1.972651
C	8.333322	−1.117841	0.583475
O	9.477397	−0.853346	2.813011
H	4.984292	−0.717851	2.958313
H	6.033914	0.686304	3.296297
H	7.308383	−1.297174	3.694122
H	8.464550	−3.239039	1.092338
H	8.311956	0.617285	1.919213
H	7.702336	−0.503134	−0.060520
H	9.321757	−1.177105	0.132939
N	6.965418	−3.070038	−0.287610
C	7.133234	−4.443761	−0.546638
O	7.899646	−5.162694	0.095970
N	6.366989	−4.926195	−1.588022
C	5.470270	−4.208114	−2.368868
O	4.813988	−4.781861	−3.264291
C	5.356438	−2.792768	−2.063198
C	4.442621	−1.946082	−2.905061
C	6.084198	−2.306249	−1.024475
H	6.466489	−5.962615	−1.785340
H	4.710116	−2.033857	−3.964713
H	4.508238	−0.893482	−2.620490
H	3.400636	−2.273439	−2.812185
H	6.001194	−1.272569	−0.710037
P	11.035459	−0.871285	2.311903
O	11.274568	0.022131	1.112572
O	11.878490	−0.601624	3.544653
O	11.232794	−2.410150	1.796098
C	11.180086	−3.490745	2.772239
C	11.941066	−4.701427	2.253130
O	11.220796	−5.346689	1.159835
C	11.945745	−5.204388	−0.083876
C	13.348124	−4.395200	1.711841
C	13.123198	−4.253940	0.199764
O	14.188882	−5.532935	2.039869
H	10.133394	−3.760374	2.952566
H	11.633988	−3.157338	3.712050
H	12.005541	−5.424780	3.074685
H	12.279248	−6.194054	−0.408890
H	13.763710	−3.482621	2.151904
H	12.852707	−3.221482	−0.029132
H	14.009309	−4.521036	−0.380793
H	15.072478	−5.363861	1.664547
N	11.036308	−4.736167	−1.126674
C	10.505277	−3.462709	−1.282207
N	9.639829	−3.380526	−2.279886
C	9.584251	−4.670018	−2.807971
C	8.836519	−5.236621	−3.867209
N	7.972775	−4.543477	−4.631966
N	9.016089	−6.564392	−4.117864
C	9.869697	−7.277624	−3.360926
N	10.619101	−6.844115	−2.333727
C	10.437822	−5.529431	−2.098016
H	10.787830	−2.656453	−0.620964
H	7.392507	−5.036878	−5.322067
H	7.781809	−3.574975	−4.412892
H	9.943920	−8.332973	−3.616865
O	5.541675	−11.550155	−10.434033
C	6.704958	−12.385834	−10.655355
C	7.300441	−12.830388	−9.324381
O	7.923901	−11.703720	−8.625724
C	7.241687	−11.445576	−7.369986
C	6.294643	−13.441890	−8.325320
C	5.941753	−12.255592	−7.426662
O	6.974433	−14.563593	−7.660639
H	5.289668	−11.161606	−11.289866
H	7.470990	−11.835277	−11.215670
H	6.428526	−13.283764	−11.226601
H	8.087912	−13.559497	−9.543013
H	7.875653	−11.756552	−6.541032
H	5.415707	−13.858062	−8.821913
H	5.151025	−11.690795	−7.924309
H	5.581628	−12.547616	−6.444023
N	7.056180	−9.998387	−7.201206
C	7.799203	−9.339613	−6.206495
O	8.637829	−9.903744	−5.497937
N	7.506613	−7.995830	−6.077475
C	6.633930	−7.247910	−6.860005
O	6.486235	−6.024070	−6.639477
C	5.953629	−7.974319	−7.917957
C	5.025321	−7.231273	−8.840192
C	6.204484	−9.304097	−8.038763
H	8.052817	−7.478044	−5.340180
H	5.556293	−6.428796	−9.367297
H	4.209406	−6.760475	−8.277760
H	4.591658	−7.908309	−9.581822
H	5.763047	−9.900802	−8.831065
P	6.862912	−14.972843	−6.087730
O	5.467194	−14.766637	−5.523302
O	7.437119	−16.370718	−5.958459
O	7.808498	−13.873901	−5.339549
C	9.209139	−14.107036	−5.037905
C	9.429311	−14.081872	−3.528881
O	9.224001	−12.748346	−2.974688
C	7.958625	−12.669215	−2.276519
C	8.489350	−15.004958	−2.733644
C	7.338747	−14.079056	−2.311599
O	9.265857	−15.537796	−1.617434
H	9.787732	−13.306688	−5.509008
H	9.527469	−15.074925	−5.437406
H	10.473613	−14.351107	−3.337196
H	8.164696	−12.332122	−1.258089
H	8.138851	−15.849245	−3.332164
H	6.544356	−14.151934	−3.053543
H	6.923129	−14.355564	−1.344984
N	7.098922	−11.636447	−2.863107
C	6.137038	−11.759976	−3.861196
N	5.528816	−10.618613	−4.136459
C	6.111953	−9.687413	−3.280637
C	5.879413	−8.307288	−3.081006
N	4.982912	−7.595010	−3.781036
N	6.615164	−7.685721	−2.116920
C	7.525757	−8.385354	−1.416736
N	7.834170	−9.687741	−1.536674
C	7.091365	−10.296813	−2.482571
H	5.927426	−12.706067	−4.336141
H	4.879750	−6.585382	−3.617412
H	4.423649	−8.055094	−4.485899
H	8.071951	−7.812733	−0.670474
P	8.552631	−16.313685	−0.361714
O	7.239782	−16.954218	−0.764029
O	9.612197	−17.224525	0.229041
O	8.186269	−15.091215	0.663714
C	9.279002	−14.266728	1.161432
C	8.723502	−13.126423	1.991317
O	7.994883	−12.207573	1.125057
C	6.814160	−11.756886	1.833503
C	7.756401	−13.537260	3.133832
C	6.402604	−12.954108	2.700519
O	8.260202	−12.932046	4.353613
H	9.849016	−13.866492	0.315498
H	9.945784	−14.875826	1.782940
H	9.575886	−12.595223	2.437109
H	7.049406	−10.880617	2.440531
H	7.711612	−14.623695	3.252625

H	5.859688	−13.695368	2.107103
H	5.781846	−12.660897	3.550685
H	7.699235	−13.241441	5.088262
N	5.823674	−11.311265	0.852593
C	5.470953	−9.932021	0.777862
O	6.028673	−9.114642	1.544355
N	4.526991	−9.558237	−0.122648
C	3.957849	−10.455201	−0.953734
N	3.019626	−10.017369	−1.803200
C	4.339358	−11.837975	−0.931109
C	5.263798	−12.213419	−0.013735
H	2.746030	−9.022713	−1.822415
H	2.615058	−10.654980	−2.476080
H	3.904651	−12.555946	−1.615733
H	5.614215	−13.233761	0.074375
Na	12.965907	1.235362	2.392496
Na	5.580148	4.234016	0.138554
Na	5.442998	−17.049740	−4.752653
Na	8.045778	−19.118429	−0.114676

d(TAA)$^{Na+}$

E: −27689.10 kcal mol^{-1}

O	−0.078833	0.022684	−0.919176
C	−0.086401	0.439619	0.468504
C	1.267405	0.168837	1.112470
O	1.490804	−1.269759	1.282522
C	2.634346	−1.699450	0.500347
C	2.489775	0.690653	0.330813
C	2.951550	−0.540539	−0.450932
O	3.448949	1.158547	1.335166
H	−0.995295	0.061146	−1.244154
H	−0.855934	−0.106115	1.028671
H	−0.294258	1.516686	0.544758
H	1.253133	0.620541	2.110265
H	3.468509	−1.923954	1.166114
H	2.245912	1.532268	−0.321448
H	2.352526	−0.607173	−1.361318
H	4.000296	−0.501958	−0.735596
N	2.316877	−2.968391	−0.167359
C	2.981859	−4.133159	0.249933
O	3.792651	−4.157169	1.180204
N	2.659097	−5.261465	−0.477877
C	1.715151	−5.362871	−1.493823
O	1.511437	−6.464714	−2.052510
C	1.007235	−4.137836	−1.822954
C	−0.067455	−4.169488	−2.876045
C	1.337974	−3.009979	−1.141878
H	3.143315	−6.147251	−0.178268
H	−0.858596	−4.879982	−2.605864
H	0.339076	−4.493510	−3.842099
H	−0.516193	−3.179807	−3.001615
H	0.824572	−2.067755	−1.308894
P	5.045716	1.363043	1.045119
O	5.340117	1.628826	−0.416834
O	5.531178	2.399087	2.041379
O	5.679700	−0.106300	1.381901
C	5.771747	−0.557838	2.762969
C	7.026200	−1.400055	2.959153
O	6.900349	−2.722814	2.354342
C	7.726837	−2.830177	1.171019
C	8.301086	−0.786609	2.355097
C	8.399991	−1.459288	0.980627
O	9.396187	−1.119158	3.263455
H	4.884046	−1.152593	3.004314
H	5.819779	0.309644	3.429747
H	7.154222	−1.552920	4.036052
H	8.451688	−3.634403	1.328104
H	8.242961	0.301392	2.272897
H	7.853400	−0.849634	0.260980
H	9.427708	−1.552134	0.637547
N	6.918825	−3.255745	0.028462
C	6.078559	−2.485480	−0.765504
N	5.454042	−3.190486	−1.694937
C	5.898703	−4.498185	−1.507495
C	5.610162	−5.708346	−2.180479
N	4.741236	−5.810136	−3.199231
N	6.264971	−6.825727	−1.756811
C	7.111130	−6.751680	−0.713537
N	7.428966	−5.667141	0.011393
C	6.802294	−4.562386	−0.435383
H	5.964307	−1.425974	−0.595039
H	4.584626	−6.715468	−3.659895
H	4.280855	−4.980243	−3.546296
H	7.589928	−7.689290	−0.441055
P	10.971063	−0.946049	2.844825
O	11.164428	0.101860	1.767993
O	11.734191	−0.745871	4.141287
O	11.333363	−2.390445	2.168256
C	11.278688	−3.574698	3.015156
C	11.798925	−4.778073	2.249858
O	10.856771	−5.138877	1.192228
C	11.559185	−5.237136	−0.070176
C	13.172706	−4.575270	1.572800
C	12.814886	−4.370779	0.095753
O	13.939707	−5.789131	1.791769
H	10.243201	−3.751033	3.327954
H	11.898623	−3.417217	3.905349
H	11.865748	−5.616237	2.955196
H	11.808211	−6.283295	−0.277803
H	13.706912	−3.715978	1.989891
H	12.575802	−3.317598	−0.073821
H	13.619684	−4.667311	−0.581148
H	14.811093	−5.666633	1.372292
N	10.664819	−4.818515	−1.137633
C	10.204261	−3.533727	−1.395485
N	9.350847	−3.481690	−2.403585
C	9.235740	−4.801484	−2.838728
C	8.481039	−5.402590	−3.873824
N	7.662943	−4.721519	−4.697368
N	8.608708	−6.749906	−4.038100
C	9.412745	−7.450608	−3.217723
N	10.162733	−6.983413	−2.205092
C	10.040687	−5.649257	−2.061390
H	10.536394	−2.689807	−0.809068
H	7.059683	−5.235237	−5.351869
H	7.477602	−3.744383	−4.511631
H	9.441163	−8.522397	−3.399355
O	5.847493	−11.483383	−10.779070
C	7.094682	−12.191104	−10.988402
C	7.619694	−12.735314	−9.665710
O	8.122275	−11.654886	−8.812870
C	7.349186	−11.557140	−7.592455
C	6.584530	−13.500628	−8.818210
C	6.127687	−12.468093	−7.785081
O	7.279040	−14.645817	−8.232171
H	5.626175	−11.020597	−11.605957
H	7.849194	−11.521272	−11.419278
H	6.943709	−13.037757	−11.673365
H	8.465305	−13.393885	−9.891287
H	7.963031	−11.864088	−6.743676
H	5.752215	−13.876374	−9.416920
H	5.283143	−11.914240	−8.197681
H	5.811708	−12.930817	−6.853310
N	7.017862	−10.142656	−7.340261
C	7.646423	−9.479260	−6.273015

O	8.472402	−10.016653	−5.529058
N	7.250911	−8.167647	−6.104404
C	6.364803	−7.448342	−6.898478
O	6.117756	−6.251069	−6.633899
C	5.797421	−8.173110	−8.023504
C	4.851701	−7.462145	−8.952440
C	6.163346	−9.469471	−8.195563
H	7.724697	−7.651423	−5.315712
H	5.327099	−6.578306	−9.395397
H	3.964423	−7.111485	−8.410315
H	4.526930	−8.125762	−9.759005
H	5.818468	−10.053302	−9.043298
P	6.571716	−15.605585	−7.104407
O	5.061354	−15.500283	−7.133202
O	7.170392	−16.988322	−7.284829
O	7.064785	−14.936457	−5.694357
C	8.492544	−14.945593	−5.405802
C	8.749257	−14.374121	−4.023864
O	8.444573	−12.946364	−4.012095
C	7.547345	−12.651730	−2.912779
C	7.928777	−15.009782	−2.876410
C	6.835029	−13.973006	−2.611305
O	8.860242	−15.195120	−1.763465
H	9.018797	−14.342344	−6.154478
H	8.868184	−15.974475	−5.448019
H	9.816964	−14.503999	−3.809796
H	8.114525	−12.294816	−2.050683
H	7.519841	−15.986557	−3.143691
H	6.022271	−14.155054	−3.317966
H	6.429294	−14.023632	−1.602876
N	6.678462	−11.537449	−3.293993
C	6.901648	−10.281890	−2.696063
O	7.776469	−10.080576	−1.853139
N	6.044415	−9.287609	−3.122887
C	5.038966	−9.400177	−4.073996
O	4.347479	−8.406385	−4.384215
C	4.865203	−10.719365	−4.658964
C	3.772331	−10.922721	−5.671679
C	5.698882	−11.709523	−4.247393
H	6.155973	−8.353553	−2.636679
H	2.797127	−10.662948	−5.243057
H	3.734824	−11.961856	−6.006883
H	3.918982	−10.274734	−6.543753
H	5.639912	−12.708469	−4.662181
P	8.389154	−15.438538	−0.214227
O	6.992372	−16.016692	−0.121725
O	9.503069	−16.226703	0.449047
O	8.309927	−13.921219	0.391211
C	9.545985	−13.196499	0.661573
C	9.372716	−12.285054	1.869560
O	8.570810	−11.109393	1.541889
C	7.294286	−11.155022	2.219527
C	8.681376	−12.943132	3.076010
C	7.211902	−12.529614	2.910019
O	9.280513	−12.379916	4.272471
H	9.807665	−12.598972	−0.218570
H	10.349018	−13.911895	0.867198
H	10.368564	−11.926801	2.155754
H	7.246913	−10.326831	2.932203
H	8.804030	−14.031296	3.070977
H	6.703132	−13.254570	2.272055
H	6.684054	−12.472914	3.864912
H	8.814488	−12.757751	5.040684
N	6.224704	−10.903693	1.256355
C	5.763340	−11.755289	0.261310
N	4.837163	−11.203653	−0.505129
C	4.683620	−9.912513	−0.000968
C	3.851864	−8.832593	−0.380206
N	2.972069	−8.886271	−1.396666
N	3.951152	−7.681645	0.343315
C	4.824274	−7.609305	1.364580
N	5.654520	−8.568306	1.808134
C	5.543320	−9.703403	1.088906
H	6.167049	−12.751321	0.152125
H	2.471282	−8.036561	−1.685935
H	2.953460	−9.702883	−1.992229
H	4.859975	−6.649477	1.876328
Na	4.896797	−17.885601	−7.516234
Na	7.885626	−17.892030	1.150158
Na	12.671992	1.320070	3.225399
Na	6.477965	3.686666	0.221332

$d(TCA)^{Na+}$

E: −27598.64 kcal mol^{-1}

O	−0.176598	0.057281	−0.731294
C	−0.275259	0.385053	0.676868
C	1.053630	0.120524	1.373590
O	1.317226	−1.317851	1.472222
C	2.505680	−1.669936	0.721551
C	2.287818	0.725949	0.678475
C	2.843852	−0.442682	−0.136742
O	3.193097	1.167657	1.739310
H	−1.073248	0.095522	−1.107502
H	−1.052116	−0.221785	1.158777
H	−0.527554	1.447297	0.806403
H	0.977385	0.511774	2.393794
H	3.312514	−1.922475	1.411958
H	2.039284	1.587728	0.055551
H	2.324917	−0.475453	−1.096467
H	3.910196	−0.344926	−0.325559
N	2.248156	−2.900791	−0.041555
C	2.911262	−4.080541	0.337164
O	3.682891	−4.147277	1.298762
N	2.644848	−5.166707	−0.470237
C	1.776120	−5.216956	−1.553587
O	1.629394	−6.285828	−2.187094
C	1.079136	−3.977957	−1.859359
C	0.095796	−3.947715	−2.997832
C	1.340199	−2.893248	−1.084741
H	3.150051	−6.058098	−0.214579
H	−0.712611	−4.672039	−2.838191
H	0.584683	−4.215659	−3.942807
H	−0.343807	−2.951796	−3.104029
H	0.824205	−1.948971	−1.229215
P	4.706564	1.711706	1.419173
O	4.860392	2.139775	−0.025197
O	5.024095	2.747097	2.482273
O	5.633110	0.376180	1.616525
C	5.772273	−0.173227	2.959289
C	6.864038	−1.230569	2.991010
O	6.439755	−2.425375	2.268063
C	7.375563	−2.719641	1.199385
C	8.215475	−0.810402	2.374290
C	8.162611	−1.424414	0.974492
O	9.253907	−1.382616	3.233642
H	4.821367	−0.623307	3.267061
H	6.029527	0.632005	3.656567
H	7.016286	−1.504486	4.041721
H	8.024150	−3.545627	1.493632
H	8.350237	0.273380	2.351304
H	7.617179	−0.735170	0.326503
H	9.146358	−1.592700	0.540743
N	6.634199	−3.203841	0.032515
C	6.698926	−4.584706	−0.318713
O	7.375580	−5.362792	0.382685

N	6.020424	−4.994886	−1.422891
C	5.263577	−4.141361	−2.137350
N	4.616675	−4.619418	−3.210647
C	5.140210	−2.762400	−1.762611
C	5.837308	−2.348411	−0.674595
H	4.688337	−5.620173	−3.463745
H	4.036694	−4.002891	−3.763742
H	4.508073	−2.080784	−2.318765
H	5.788048	−1.333354	−0.300621
P	10.811032	−1.567839	2.761581
O	11.201490	−0.586641	1.676181
O	11.644634	−1.553754	4.029039
O	10.822529	−3.053621	2.076172
C	10.757630	−4.236871	2.926490
C	11.628528	−5.352260	2.359863
O	11.002678	−5.990628	1.205286
C	11.735336	−5.691571	−0.005889
C	13.020984	−4.900771	1.887911
C	12.826836	−4.675175	0.381152
O	13.942267	−5.987582	2.167869
H	9.716929	−4.573897	2.983743
H	11.114110	−3.982232	3.929831
H	11.724354	−6.119177	3.137494
H	12.152558	−6.621523	−0.402078
H	13.348126	−3.990024	2.400513
H	12.482863	−3.653345	0.209828
H	13.747702	−4.833752	−0.185273
H	14.817674	−5.725923	1.828295
N	10.803735	−5.213531	−1.025037
C	10.121283	−4.003237	−1.037589
N	9.249435	−3.909828	−2.027035
C	9.342339	−5.129697	−2.696447
C	8.658647	−5.655885	−3.817533
N	7.718016	−4.981750	−4.503585
N	8.989455	−6.916737	−4.217821
C	9.938523	−7.600833	−3.550087
N	10.648651	−7.196585	−2.484392
C	10.302439	−5.954883	−2.089422
H	10.305044	−3.256172	−0.279246
H	7.198643	−5.446301	−5.259772
H	7.386864	−4.096814	−4.144360
H	10.134506	−8.603797	−3.924322
O	5.617802	−11.570147	−10.816817
C	6.823968	−12.319249	−11.107515
C	7.436106	−12.856843	−9.819860
O	8.026239	−11.777493	−9.024529
C	7.342376	−11.643968	−7.751831
C	6.452305	−13.581486	−8.881759
C	6.077388	−12.508335	−7.857253
O	7.181630	−14.708164	−8.298538
H	5.344955	−11.119296	−11.634810
H	7.561994	−11.679799	−11.607856
H	6.596009	−13.172312	−11.762847
H	8.245779	−13.539646	−10.099177
H	8.000781	−11.975880	−6.947116
H	5.580347	−13.973552	−9.409494
H	5.233851	−11.933454	−8.243394
H	5.791130	−12.936925	−6.900144
N	7.092696	−10.218789	−7.485098
C	7.837713	−9.579804	−6.478897
O	8.710224	−10.145178	−5.814810
N	7.511390	−8.252141	−6.284881
C	6.595895	−7.498737	−7.008914
O	6.423295	−6.289079	−6.735828
C	5.908428	−8.200753	−8.079022
C	4.928804	−7.449914	−8.939746
C	6.199052	−9.513598	−8.268952
H	8.054606	−7.764662	−5.526706
H	5.413486	−6.599985	−9.436208
H	4.109042	−7.043190	−8.334473
H	4.503770	−8.105300	−9.705526
H	5.758054	−10.084511	−9.079997
P	6.543974	−15.626721	−7.098847
O	5.032314	−15.540265	−7.060468
O	7.149466	−17.010085	−7.253126
O	7.092915	−14.898998	−5.739156
C	8.525873	−14.925625	−5.478826
C	8.829843	−14.362892	−4.099379
O	8.584526	−12.923475	−4.066010
C	7.627011	−12.613503	−3.019040
C	8.008548	−14.967020	−2.938442
C	6.902023	−13.931577	−2.736503
O	8.934464	−15.101556	−1.808142
H	9.044371	−14.327370	−6.237148
H	8.886222	−15.959454	−5.532385
H	9.896942	−14.532066	−3.912073
H	8.151653	−12.244921	−2.133960
H	7.619113	−15.961094	−3.168971
H	6.134641	−14.112716	−3.493237
H	6.440054	−13.968913	−1.751386
N	6.772719	−11.523850	−3.442699
C	5.779079	−11.536991	−4.418001
N	5.203516	−10.359685	−4.574139
C	5.838907	−9.521479	−3.653562
C	5.629792	−8.147946	−3.335200
O	4.830645	−7.350473	−3.880525
N	6.440248	−7.725035	−2.263726
C	7.365645	−8.516307	−1.607169
N	8.049025	−7.959225	−0.588912
N	7.607407	−9.786950	−1.950140
C	6.818030	−10.230089	−2.942262
H	5.536671	−12.438977	−4.958620
H	6.292642	−6.739035	−1.938174
H	7.798211	−7.030327	−0.221744
H	8.579039	−8.598461	−0.006467
P	8.515477	−15.023961	−0.230243
O	7.122463	−15.562663	0.027695
O	9.651285	−15.672493	0.537890
O	8.446400	−13.418705	0.073273
C	9.661036	−12.635797	0.261493
C	9.570897	−11.802294	1.536172
O	8.665801	−10.666318	1.384173
C	7.434314	−10.895397	2.111842
C	9.059403	−12.574132	2.763404
C	7.549347	−12.288585	2.759348
O	9.722363	−12.016185	3.928222
H	9.777516	−11.971595	−0.600866
H	10.524951	−13.304011	0.332094
H	10.566916	−11.389242	1.731943
H	7.329853	−10.097374	2.851045
H	9.274734	−13.645157	2.686070
H	7.037782	−13.043670	2.160859
H	7.125306	−12.298661	3.766293
H	9.364868	−12.467641	4.714605
N	6.292257	−10.739647	1.211525
C	5.811467	−11.627486	0.254111
N	4.847672	−11.113042	−0.490491
C	4.691611	−9.808796	−0.020946
C	3.865817	−8.735041	−0.429051
N	2.969205	−8.811267	−1.434257
N	3.992966	−7.558706	0.245054
C	4.879668	−7.453672	1.253001
N	5.708150	−8.402195	1.716192
C	5.580131	−9.559050	1.037374
H	6.228162	−12.618080	0.151777
H	2.528395	−7.947136	−1.770666
H	2.988398	−9.619094	−2.043958
H	4.926171	−6.475965	1.727549

Na	13.012486	0.212181	3.096022
Na	5.515978	4.363358	0.718015
Na	4.904943	−17.956887	−7.332723
Na	8.042860	−17.156320	1.600302

(AAA)$^{Na+}$

E: −14040.44 kcal mol^{-1}

O	−0.146995	−0.227013	−0.440670
C	−0.088174	0.230923	0.931685
C	1.331692	0.116001	1.474171
O	1.733772	−1.285490	1.618071
C	2.782313	−1.609277	0.668165
C	2.429956	0.766485	0.612414
C	2.911819	−0.394979	−0.257775
O	3.434648	1.285030	1.547082
H	−1.085425	−0.305923	−0.685542
H	−0.750853	−0.372208	1.565152
H	−0.399629	1.283578	1.001830
H	1.337312	0.560824	2.474951
H	3.704889	−1.806556	1.221401
H	2.061499	1.606848	0.019569
H	2.229456	−0.480068	−1.104456
H	3.919976	−0.257912	−0.641000
N	2.473323	−2.857369	−0.017493
C	1.642019	−3.077761	−1.108484
N	1.630127	−4.342275	−1.501677
C	2.493021	−4.997650	−0.621746
C	2.920098	−6.340954	−0.510902
N	2.490413	−7.320979	−1.339808
N	3.789639	−6.656467	0.484217
C	4.208862	−5.683223	1.316052
N	3.875041	−4.381073	1.306037
C	3.017876	−4.092867	0.313292
H	1.061035	−2.278171	−1.545711
H	2.934727	−8.230273	−1.302931
H	1.937262	−7.088430	−2.154041
H	4.907807	−5.993856	2.090749
P	5.018362	1.492975	1.199164
O	5.262707	1.770010	−0.270476
O	5.535615	2.523336	2.184937
O	5.670289	0.023743	1.496965
C	5.829747	−0.447514	2.864364
C	7.054155	−1.349433	2.966864
O	6.849824	−2.616427	2.272628
C	7.652111	−2.671463	1.068201
C	8.341727	−0.757169	2.366905
C	8.332925	−1.300573	0.936144
O	9.446922	−1.251555	3.188123
H	4.934334	−1.008008	3.155627
H	5.955990	0.407634	3.536915
H	7.203135	−1.581201	4.027022
H	8.372318	−3.488696	1.170835
H	8.357333	0.335305	2.395554
H	7.735192	−0.624950	0.324432
H	9.326187	−1.362701	0.500523
N	6.819842	−3.034606	−0.075583
C	6.002112	−2.219131	−0.846515
N	5.370562	−2.876769	−1.806358
C	5.787315	−4.200310	−1.664014
C	5.490726	−5.382277	−2.378145
N	4.648250	−5.405245	−3.440313
N	6.104684	−6.530853	−1.999552
C	6.950001	−6.501297	−0.951669
N	7.290891	−5.448203	−0.187751
C	6.686427	−4.318905	−0.591149
H	5.911721	−1.162728	−0.644087
H	4.362189	−6.303831	−3.810380
H	4.042124	−4.612278	−3.609017
H	7.413900	−7.451370	−0.692775
P	11.009316	−1.264281	2.698078
O	11.287870	−0.229424	1.627571
O	11.848700	−1.189087	3.959881
O	11.166689	−2.727788	1.982678
C	11.051215	−3.924266	2.806296
C	11.644832	−5.122402	2.080665
O	10.815317	−5.504927	0.941604
C	11.540154	−5.331230	−0.298879
C	13.065708	−4.914169	1.525983
C	12.825970	−4.561616	0.052114
O	13.771413	−6.173708	1.682786
H	9.993244	−4.110930	3.023452
H	11.588773	−3.772480	3.749111
H	11.644622	−5.961860	2.786488
H	11.748494	−6.314703	−0.730155
H	13.597122	−4.117818	2.057571
H	12.670733	−3.484755	−0.040109
H	13.661519	−4.850800	−0.589664
H	14.657500	−6.066318	1.291041
N	10.679011	−4.650862	−1.261231
C	10.283776	−3.320212	−1.238779
N	9.401636	−3.023015	−2.178157
C	9.194430	−4.222117	−2.860067
C	8.359054	−4.576706	−3.942163
N	7.535467	−3.690308	−4.560759
N	8.400577	−5.856744	−4.391876
C	9.205476	−6.738764	−3.769833
N	10.015743	−6.526309	−2.716240
C	9.977166	−5.247391	−2.301737
H	10.678487	−2.636597	−0.501381
H	6.807891	−4.069600	−5.157407
H	7.322194	−2.820842	−4.085356
H	9.192870	−7.752301	−4.167553
Na	13.037793	0.719010	3.035609
Na	6.429340	3.809483	0.323321

(AAC)$^{Na+}$

E: −13641.45 kcal mol^{-1}

O	−0.129663	−0.081852	−0.437247
C	−0.095584	0.336888	0.948520
C	1.305322	0.166179	1.525409
O	1.664218	−1.250552	1.635672
C	2.737068	−1.570715	0.712563
C	2.443148	0.810927	0.711869
C	2.914003	−0.337423	−0.179849
O	3.440778	1.266617	1.686294
H	−1.062833	−0.124068	−0.709874
H	−0.792556	−0.263489	1.546738
H	−0.378568	1.395815	1.039313
H	1.295988	0.580267	2.539234
H	3.639365	−1.796158	1.288408
H	2.114182	1.679879	0.137342
H	2.246032	−0.386256	−1.040857
H	3.931705	−0.213557	−0.541168
N	2.429887	−2.796048	−0.013276
C	1.607358	−2.972946	−1.119008
N	1.607925	−4.218343	−1.568896
C	2.472009	−4.905684	−0.714999
C	2.912941	−6.248244	−0.666432
N	2.499012	−7.191363	−1.544452
N	3.781105	−6.602207	0.317112
C	4.185590	−5.665508	1.196651
N	3.838718	−4.367455	1.246757
C	2.983183	−4.040659	0.264358
H	1.022872	−2.157657	−1.521758
H	2.952587	−8.096814	−1.548133
H	1.948349	−6.925737	−2.350275
H	4.883804	−6.005557	1.959548

P	5.028428	1.484095	1.362135
O	5.288762	1.798842	−0.096936
O	5.537532	2.486527	2.380463
O	5.678157	0.006888	1.629229
C	5.828338	−0.497436	2.986155
C	7.048667	−1.406337	3.073601
O	6.838344	−2.665080	2.364320
C	7.635207	−2.713014	1.158689
C	8.335238	−0.806383	2.479403
C	8.342650	−1.350463	1.048644
O	9.443852	−1.287318	3.301334
H	4.928581	−1.059115	3.260923
H	5.956648	0.341968	3.677820
H	7.199480	−1.653353	4.129954
H	8.339255	−3.546850	1.239816
H	8.338688	0.286149	2.504229
H	7.773224	−0.663837	0.422175
H	9.346453	−1.430653	0.639648
N	6.794226	−3.029803	0.004487
C	5.975524	−2.171623	−0.719783
N	5.358406	−2.769176	−1.725948
C	5.786826	−4.094795	−1.668085
C	5.514718	−5.224038	−2.471986
N	4.687573	−5.175140	−3.545344
N	6.133281	−6.392432	−2.167537
C	6.962154	−6.429858	−1.105940
N	7.285003	−5.430604	−0.266237
C	6.676304	−4.279112	−0.596503
H	5.873624	−1.133203	−0.444196
H	4.408112	−6.045824	−3.981664
H	4.083790	−4.371814	−3.668424
H	7.429499	−7.392431	−0.906136
P	11.009988	−1.192831	2.825050
O	11.231318	−0.107397	1.791874
O	11.835334	−1.111873	4.095467
O	11.251892	−2.620977	2.062517
C	11.140702	−3.844706	2.847370
C	11.594984	−5.032756	2.017822
O	10.653549	−5.261197	0.927666
C	11.359563	−5.306041	−0.336787
C	12.994712	−4.896084	1.379540
C	12.694124	−4.584406	−0.091546
O	13.664767	−6.173913	1.548226
H	10.097566	−3.983587	3.153413
H	11.768328	−3.764706	3.742131
H	11.586807	−5.912688	2.674178
H	11.503185	−6.341391	−0.648835
H	13.581075	−4.103889	1.856181
H	12.581583	−3.504283	−0.217808
H	13.481181	−4.934677	−0.764090
H	14.544970	−6.101098	1.135659
N	10.505052	−4.695947	−1.362838
C	9.983507	−5.493211	−2.429821
O	10.318433	−6.691334	−2.522611
N	9.135821	−4.897997	−3.314715
C	8.796638	−3.609378	−3.183419
N	7.950230	−3.084956	−4.097655
C	9.307905	−2.789342	−2.125354
C	10.147622	−3.382707	−1.238950
H	7.504621	−3.701619	−4.766896
H	7.551323	−2.165647	−3.960711
H	9.023990	−1.749135	−2.023373
H	10.560031	−2.864490	−0.381398
Na	12.894485	0.920652	3.228867
Na	6.465456	3.820214	0.582761

(AAG)$^{Na+}$

E: −14204.76 kcal mol^{-1}

O	−0.115283	−0.255167	−0.447008
C	−0.041412	0.212349	0.921485
C	1.382307	0.090845	1.451962
O	1.774683	−1.312466	1.603079
C	2.813890	−1.651207	0.647963
C	2.477951	0.726861	0.575542
C	2.944465	−0.445660	−0.288865
O	3.491547	1.253472	1.497139
H	−1.056194	−0.322609	−0.685691
H	−0.702918	−0.381251	1.565150
H	−0.344574	1.267711	0.986541
H	1.400273	0.543394	2.449147
H	3.739387	−1.849076	1.195084
H	2.109313	1.563547	−0.022178
H	2.252995	−0.532294	−1.128120
H	3.950080	−0.321212	−0.683137
N	2.492431	−2.903496	−0.023235
C	1.644211	−3.131740	−1.099208
N	1.621875	−4.400296	−1.479108
C	2.495634	−5.049881	−0.605229
C	2.919521	−6.393538	−0.485934
N	2.477580	−7.380117	−1.300318
N	3.800195	−6.702025	0.501546
C	4.235848	−5.721199	1.316158
N	3.908250	−4.417728	1.295546
C	3.037751	−4.137265	0.312148
H	1.059400	−2.334284	−1.535416
H	2.911748	−8.293684	−1.254006
H	1.907694	−7.156729	−2.105427
H	4.944304	−6.026375	2.084376
P	5.085796	1.391942	1.167202
O	5.360251	1.652286	−0.300565
O	5.635213	2.403207	2.155311
O	5.672509	−0.101989	1.473992
C	5.829431	−0.576375	2.840085
C	7.090940	−1.424468	2.953853
O	6.953556	−2.704155	2.267240
C	7.738494	−2.720371	1.050351
C	8.350783	−0.771422	2.358629
C	8.395762	−1.334805	0.934829
O	9.470002	−1.175557	3.209160
H	4.953945	−1.176580	3.110944
H	5.909361	0.277087	3.521502
H	7.244545	−1.645312	4.015538
H	8.471735	−3.528633	1.128377
H	8.298999	0.320360	2.365331
H	7.811128	−0.675291	0.293744
H	9.403806	−1.386075	0.533828
N	6.896549	−3.074758	−0.093129
C	6.068780	−2.254771	−0.851667
N	5.412294	−2.909869	−1.795137
C	5.820622	−4.235661	−1.657887
C	5.493972	−5.417656	−2.358280
N	4.631592	−5.437776	−3.405558
N	6.093474	−6.574314	−1.980214
C	6.949530	−6.552063	−0.940506
N	7.320353	−5.499174	−0.191156
C	6.736074	−4.360450	−0.598976
H	5.989659	−1.197118	−0.652368
H	4.307715	−6.336169	−3.743956
H	4.038752	−4.633980	−3.571171
H	7.396655	−7.509061	−0.678057
P	11.037497	−1.142978	2.739276
O	11.299474	−0.121365	1.651591
O	11.854581	−1.012587	4.011603
O	11.250049	−2.615180	2.058017

C	11.075253	−3.794879	2.894623
C	11.581424	−5.028831	2.165145
O	10.726088	−5.333361	1.022302
C	11.481707	−5.257113	−0.211219
C	13.016921	−4.922631	1.617506
C	12.811268	−4.568398	0.139474
O	13.636061	−6.225017	1.789854
H	10.012112	−3.915159	3.131543
H	11.636789	−3.670779	3.827917
H	11.518512	−5.870728	2.865650
H	11.628322	−6.268059	−0.602498
H	13.597931	−4.159797	2.146064
H	12.724251	−3.484856	0.038513
H	13.631070	−4.915231	−0.494078
H	14.532987	−6.177029	1.411177
N	10.689183	−4.551692	−1.210825
C	10.422811	−3.184140	−1.265842
N	9.585728	−2.866989	−2.234246
C	9.273293	−4.077579	−2.852803
C	8.415568	−4.361919	−3.958698
O	7.733759	−3.582658	−4.648644
N	8.397154	−5.755793	−4.238975
C	9.105332	−6.726963	−3.563447
N	8.925450	−8.015350	−3.954981
N	9.905183	−6.450116	−2.537541
C	9.947300	−5.136282	−2.225436
H	10.871979	−2.497808	−0.563326
H	7.782539	−6.033269	−5.001179
H	8.534379	−8.216790	−4.867167
H	9.569309	−8.700709	−3.577668
Na	12.997388	0.912157	3.053844
Na	6.611051	3.637791	0.303867

(AAT)$^{Na+}$

E: −13903.59 kcal mol^{-1}

O	0.255652	0.086917	−0.536957
C	0.373037	0.542113	0.832991
C	1.747673	0.189751	1.390228
O	1.895804	−1.259851	1.530866
C	2.922240	−1.751872	0.629954
C	2.943227	0.654822	0.531317
C	3.260155	−0.584920	−0.305921
O	4.002718	1.103839	1.447598
H	−0.672129	0.200749	−0.807302
H	−0.390433	0.066011	1.461536
H	0.245023	1.633000	0.891388
H	1.822120	0.628635	2.391008
H	3.785630	−2.061698	1.219028
H	2.693784	1.516638	−0.090925
H	2.587583	−0.578561	−1.165504
H	4.284637	−0.612263	−0.669412
N	2.470295	−2.958420	−0.048436
C	1.607038	−3.088470	−1.127778
N	1.490741	−4.339356	−1.548157
C	2.320494	−5.077997	−0.701755
C	2.654420	−6.450963	−0.635866
N	2.140021	−7.376147	−1.478632
N	3.525146	−6.852528	0.327105
C	4.029059	−5.932903	1.174620
N	3.787146	−4.611905	1.207236
C	2.929822	−4.237762	0.242081
H	1.085246	−2.233735	−1.536425
H	2.498514	−8.322851	−1.458188
H	1.574690	−7.089826	−2.267078
H	4.722820	−6.313215	1.922700
P	5.582659	0.704355	1.414385
O	6.171079	0.702788	0.017929
O	6.259100	1.581074	2.453784
O	5.587046	−0.865883	1.883601
C	5.845342	−1.329263	3.233368
C	7.227133	−1.983849	3.299721
O	7.258812	−3.258487	2.593763
C	7.945596	−3.135927	1.329142
C	8.301558	−1.104727	2.647499
C	8.500304	−1.702718	1.245320
O	9.513869	−1.114077	3.461625
H	5.070588	−2.061080	3.479388
H	5.802271	−0.493587	3.939135
H	7.468574	−2.188348	4.348145
H	8.738824	−3.887447	1.313666
H	7.956101	−0.074584	2.611573
H	7.956855	−1.103155	0.515391
H	9.552030	−1.718420	0.966219
N	7.061250	−3.480145	0.210296
C	6.215012	−2.654153	−0.526237
N	5.556826	−3.301494	−1.471712
C	5.981357	−4.623930	−1.363734
C	5.684960	−5.787084	−2.107430
N	4.819717	−5.792122	−3.146200
N	6.325847	−6.939862	−1.776129
C	7.191601	−6.928925	−0.744012
N	7.539891	−5.891113	0.039377
C	6.912735	−4.756533	−0.320824
H	6.149306	−1.594203	−0.331197
H	4.543590	−6.674601	−3.558471
H	4.252703	−4.975292	−3.331705
H	7.671680	−7.879991	−0.520497
P	10.712992	−0.088010	3.016427
O	10.151325	1.107396	2.258306
O	11.527582	0.214162	4.255069
O	11.570282	−0.955518	1.932210
C	12.236345	−2.167051	2.403318
C	12.557723	−3.083839	1.242411
O	11.340715	−3.652226	0.689829
C	11.675870	−4.212990	−0.603954
C	13.261836	−2.457241	0.028241
C	12.939622	−3.461393	−1.100634
O	14.664145	−2.316924	0.322395
H	11.591828	−2.692600	3.115809
H	13.168953	−1.888265	2.904741
H	13.195182	−3.890963	1.639088
H	11.846698	−5.285745	−0.512260
H	12.825848	−1.474894	−0.181666
H	12.770212	−2.977645	−2.064618
H	13.769815	−4.165000	−1.207232
H	15.053651	−1.718384	−0.339646
N	10.502173	−4.069830	−1.478869
C	9.999167	−5.205422	−2.121789
O	10.428923	−6.348118	−1.942773
N	8.964747	−4.932785	−3.000094
C	8.431048	−3.681784	−3.349902
O	7.562818	−3.606290	−4.230057
C	8.985060	−2.557503	−2.605714
C	8.447250	−1.176882	−2.860841
C	9.994801	−2.807538	−1.735885
H	8.593550	−5.740680	−3.496625
H	8.635928	−0.870554	−3.896877
H	8.913782	−0.448574	−2.191787
H	7.363012	−1.150718	−2.708157
H	10.467478	−2.013000	−1.170169
Na	11.238826	2.577514	3.950146
Na	8.287571	2.041860	1.306595

(ACA)$^{Na+}$

E: −13638.22 kcal mol^{-1}

O	−0.178341	−0.196601	−0.314730
C	−0.143646	0.192147	1.079896
C	1.271054	0.069168	1.633136
O	1.681776	−1.332925	1.750766
C	2.728384	−1.639654	0.797891
C	2.368294	0.747858	0.793990
C	2.890296	−0.391469	−0.083183
O	3.355774	1.264973	1.742155
H	−1.112060	−0.254093	−0.582435
H	−0.807542	−0.448903	1.673458
H	−0.467778	1.236657	1.196399
H	1.266164	0.489006	2.644494
H	3.643275	−1.885398	1.346019
H	1.990991	1.585686	0.203309
H	2.255062	−0.457664	−0.967352
H	3.916237	−0.237380	−0.408601
N	2.391195	−2.848205	0.050026
C	1.494829	−2.984119	−1.004540
N	1.460931	−4.211838	−1.498062
C	2.376290	−4.930508	−0.728974
C	2.818875	−6.272633	−0.770881
N	2.393923	−7.155336	−1.704700
N	3.735072	−6.674043	0.148222
C	4.196360	−5.775786	1.040816
N	3.868324	−4.477736	1.160153
C	2.955337	−4.105090	0.246776
H	0.884198	−2.153561	−1.330074
H	2.654697	−8.130509	−1.621254
H	1.624518	−6.914176	−2.315950
H	4.931545	−6.151532	1.750208
P	4.861370	1.731338	1.296126
O	4.936566	2.114525	−0.167067
O	5.298111	2.778832	2.303402
O	5.739455	0.361800	1.466304
C	5.918604	−0.188061	2.803604
C	7.042778	−1.212024	2.802130
O	6.654427	−2.401943	2.054333
C	7.556059	−2.603443	0.929914
C	8.368574	−0.726368	2.179549
C	8.298920	−1.276556	0.753673
O	9.443029	−1.282523	3.009224
H	4.986381	−0.665707	3.125974
H	6.166720	0.619990	3.500947
H	7.215451	−1.506255	3.843979
H	8.240619	−3.421005	1.152946
H	8.468668	0.361351	2.200695
H	7.718293	−0.568883	0.158505
H	9.270863	−1.399938	0.284608
N	6.780159	−3.057795	−0.226766
C	6.819964	−4.450484	−0.581974
O	7.546084	−5.222333	0.065037
N	6.050061	−4.859578	−1.630144
C	5.259441	−4.001798	−2.279212
N	4.541839	−4.475706	−3.325160
C	5.166083	−2.618501	−1.915093
C	5.938056	−2.201918	−0.877305
H	4.523564	−5.474956	−3.491857
H	3.816377	−3.911431	−3.747470
H	4.502028	−1.935958	−2.431550
H	5.912880	−1.185658	−0.503427
P	10.935704	−1.678986	2.480591
O	11.411314	−0.787509	1.348203
O	11.820037	−1.741722	3.711558
O	10.734172	−3.156448	1.823894
C	10.683021	−4.362196	2.632453
C	11.605827	−5.415649	2.031437
O	11.110383	−5.894370	0.746307
C	11.853682	−5.299568	−0.341051
C	13.038394	−4.930309	1.755781
C	12.993976	−4.472008	0.286221
O	13.918542	−6.067498	1.952262
H	9.652199	−4.731466	2.634119
H	10.997414	−4.143136	3.658013
H	11.618834	−6.277650	2.707910
H	12.222645	−6.120438	−0.960800
H	13.326789	−4.114374	2.427410
H	12.785458	−3.403556	0.246001
H	13.942798	−4.655134	−0.223400
H	14.822510	−5.784227	1.723242
N	10.954129	−4.529153	−1.210966
C	10.667760	−3.168676	−1.220938
N	9.749389	−2.837765	−2.114969
C	9.398769	−4.039736	−2.727431
C	8.471013	−4.362233	−3.740839
N	7.709892	−3.426338	−4.367306
N	8.361262	−5.660356	−4.121986
C	9.129872	−6.582386	−3.510446
N	10.034186	−6.396200	−2.530877
C	10.132699	−5.101696	−2.176340
H	11.152148	−2.475070	−0.549873
H	6.910305	−3.747923	−4.901731
H	7.642876	−2.503388	−3.955511
H	8.999136	−7.608031	−3.852503
Na	13.390830	−0.248233	2.610453
Na	5.804157	4.300722	0.476935

(ACC)$^{Na+}$

E: −13240.33 kcal mol^{-1}

O	−0.166835	−0.038025	−0.284829
C	−0.144293	0.286873	1.126551
C	1.259426	0.107590	1.693055
O	1.639188	−1.307624	1.745443
C	2.693905	−1.587265	0.793666
C	2.382252	0.803362	0.902478
C	2.888196	−0.301910	−0.024800
O	3.370394	1.250585	1.885860
H	−1.097792	−0.066069	−0.566793
H	−0.830459	−0.365498	1.681099
H	−0.447876	1.331864	1.285425
H	1.248226	0.476131	2.724042
H	3.596591	−1.874034	1.342449
H	2.030185	1.678217	0.351065
H	2.257565	−0.313262	−0.914801
H	3.918445	−0.150809	−0.337026
N	2.349240	−2.753381	−0.015404
C	1.462326	−2.827416	−1.084066
N	1.425254	−4.027329	−1.641463
C	2.328797	−4.791839	−0.902669
C	2.763102	−6.133504	−1.009785
N	2.338787	−6.965135	−1.988557
N	3.667910	−6.587771	−0.103666
C	4.125219	−5.740597	0.839542
N	3.804396	−4.448035	1.022532
C	2.903130	−4.022747	0.120495
H	0.861353	−1.976020	−1.371876
H	2.604210	−7.942047	−1.964129
H	1.589845	−6.682655	−2.607131
H	4.850914	−6.157521	1.535519
P	4.898961	1.687168	1.491444
O	5.024511	2.110361	0.042909
O	5.341789	2.687794	2.542063
O	5.734102	0.286680	1.640178

C	5.874106	−0.307869	2.963458
C	7.007559	−1.322377	2.963365
O	6.648088	−2.499672	2.179789
C	7.545688	−2.653637	1.049709
C	8.337482	−0.807137	2.379737
C	8.315578	−1.329422	0.942048
O	9.404019	−1.385259	3.195552
H	4.935872	−0.803312	3.237739
H	6.092664	0.477631	3.695367
H	7.160077	−1.642186	4.000573
H	8.208521	−3.502577	1.224670
H	8.421349	0.281083	2.424127
H	7.781571	−0.604733	0.324745
H	9.315097	−1.453919	0.533282
N	6.763265	−3.018154	−0.138811
C	6.796161	−4.375370	−0.610368
O	7.493783	−5.210683	−0.012159
N	6.058399	−4.681567	−1.714838
C	5.293203	−3.761546	−2.308927
N	4.601405	−4.131793	−3.409590
C	5.197030	−2.416548	−1.822108
C	5.942296	−2.101010	−0.731341
H	4.592455	−5.107299	−3.682673
H	3.915574	−3.513531	−3.821716
H	4.549373	−1.686323	−2.291486
H	5.911324	−1.123079	−0.266427
P	10.986674	−1.306072	2.774493
O	11.252141	−0.232667	1.739640
O	11.772058	−1.218347	4.070424
O	11.244221	−2.744686	2.038981
C	11.123265	−3.960231	2.835876
C	11.655371	−5.149810	2.054136
O	10.771390	−5.449999	0.934272
C	11.500869	−5.395043	−0.315796
C	13.069507	−4.973074	1.464528
C	12.808458	−4.644999	−0.011281
O	13.761458	−6.239267	1.633699
H	10.068508	−4.124031	3.084046
H	11.697167	−3.847850	3.762716
H	11.652580	−6.012618	2.732340
H	11.682909	−6.406770	−0.679364
H	13.623260	−4.175922	1.971203
H	12.679669	−3.565911	−0.125259
H	13.624800	−4.968285	−0.661938
H	14.646461	−6.146133	1.235613
N	10.644078	−4.759811	−1.327824
C	10.111026	−5.536496	−2.403195
O	10.424356	−6.741483	−2.506714
N	9.277734	−4.919934	−3.287376
C	8.945545	−3.631143	−3.134369
N	8.109442	−3.082066	−4.041536
C	9.445778	−2.838988	−2.051508
C	10.276504	−3.452706	−1.170428
H	7.681269	−3.669368	−4.746836
H	7.760863	−2.140533	−3.923011
H	9.157039	−1.802892	−1.926519
H	10.672260	−2.956516	−0.292043
Na	12.875551	0.783761	3.240030
Na	5.950470	4.247759	0.774055

(ACG)$^{Na+}$

E: −13802.26 kcal mol^{-1}

O	−0.175501	−0.266674	−0.304573
C	−0.129872	0.131851	1.087031
C	1.290429	0.020312	1.627835
O	1.709228	−1.378685	1.753488
C	2.751103	−1.687703	0.796201
C	2.376886	0.697990	0.773729
C	2.900452	−0.446174	−0.096308
O	3.366623	1.231835	1.709808
H	−1.111454	−0.332277	−0.562658
H	−0.784827	−0.508461	1.691194
H	−0.458483	1.175426	1.199447
H	1.292616	0.448656	2.635627
H	3.670881	−1.924576	1.339896
H	1.989315	1.527174	0.177528
H	2.259984	−0.524059	−0.975853
H	3.923662	−0.289952	−0.429879
N	2.414675	−2.903715	0.060254
C	1.505330	−3.052955	−0.981200
N	1.472646	−4.284455	−1.465287
C	2.403137	−4.991441	−0.703612
C	2.851284	−6.331769	−0.739805
N	2.415398	−7.225155	−1.658600
N	3.783472	−6.720395	0.168462
C	4.252554	−5.812165	1.047063
N	3.917905	−4.515531	1.161520
C	2.990273	−4.155258	0.257945
H	0.884721	−2.228666	−1.303997
H	2.678284	−8.199100	−1.567262
H	1.633693	−6.993052	−2.257609
H	5.001084	−6.177579	1.747813
P	4.868422	1.698489	1.251961
O	4.935875	2.069849	−0.214641
O	5.306265	2.756015	2.248257
O	5.750800	0.332968	1.430301
C	5.934211	−0.204717	2.772079
C	7.072699	−1.212353	2.780609
O	6.699526	−2.417508	2.050121
C	7.604770	−2.626878	0.929880
C	8.389913	−0.714254	2.148851
C	8.335331	−1.295524	0.734593
O	9.472683	−1.229371	2.993486
H	5.007693	−0.692652	3.095724
H	6.169436	0.611808	3.464071
H	7.252578	−1.490315	3.825740
H	8.296207	−3.435463	1.165423
H	8.467476	0.375591	2.145576
H	7.751644	−0.606647	0.120450
H	9.310777	−1.420551	0.272936
N	6.835398	−3.104583	−0.221639
C	6.890547	−4.500428	−0.560516
O	7.616955	−5.258984	0.102222
N	6.133529	−4.929065	−1.610322
C	5.327144	−4.088986	−2.264701
N	4.606386	−4.586466	−3.295974
C	5.209652	−2.705420	−1.908889
C	5.977110	−2.267458	−0.877676
H	4.598399	−5.587338	−3.450973
H	3.884798	−4.031251	−3.736068
H	4.531994	−2.038584	−2.428315
H	5.935344	−1.248989	−0.511111
P	10.981554	−1.581628	2.478973
O	11.440185	−0.687023	1.342786
O	11.856316	−1.604639	3.718491
O	10.831962	−3.071736	1.838130
C	10.728631	−4.256170	2.672051
C	11.598808	−5.360051	2.085689
O	11.084563	−5.825843	0.804171
C	11.854360	−5.272371	−0.285913
C	13.054666	−4.949892	1.811190
C	13.039037	−4.505098	0.336877
O	13.875534	−6.128107	2.024594
H	9.682591	−4.580204	2.689886
H	11.060212	−4.031201	3.691196

H	11.566042	−6.214501	2.771064
H	12.178410	−6.113841	−0.903151
H	13.381846	−4.142066	2.474838
H	12.892505	−3.427114	0.287022
H	13.977053	−4.746739	−0.168286
H	14.794112	−5.892550	1.799580
N	10.996775	−4.455780	−1.157544
C	10.825770	−3.071763	−1.209013
N	9.938347	−2.702119	−2.113607
C	9.485535	−3.889693	−2.686210
C	8.528656	−4.116650	−3.720505
O	7.857500	−3.292607	−4.369549
N	8.389411	−5.506087	−3.983673
C	9.065381	−6.521104	−3.342745
N	8.744368	−7.799661	−3.690915
N	9.963524	−6.298553	−2.388788
C	10.130192	−4.987558	−2.100884
H	11.357774	−2.399051	−0.553459
H	7.698268	−5.742332	−4.692144
H	8.308339	−7.973184	−4.589264
H	9.376110	−8.521875	−3.362834
Na	13.397532	−0.088380	2.611437
Na	5.804361	4.264665	0.415341

(ACT)$^{Na+}$

E: −13499.34 kcal mol^{-1}

O	−0.153371	−0.044568	−0.219800
C	−0.111023	0.268163	1.193851
C	1.301913	0.090276	1.737324
O	1.687499	−1.323617	1.775884
C	2.726287	−1.595277	0.804416
C	2.410341	0.794648	0.934078
C	2.907634	−0.303272	−0.006572
O	3.409731	1.242177	1.906058
H	−1.088209	−0.071916	−0.488665
H	−0.785765	−0.392278	1.752953
H	−0.416782	1.310323	1.366327
H	1.305347	0.452557	2.770546
H	3.637686	−1.886498	1.335872
H	2.046888	1.670901	0.392408
H	2.267318	−0.309596	−0.889626
H	3.934090	−0.147919	−0.329002
N	2.367891	−2.755541	−0.007119
C	1.460262	−2.823858	−1.058680
N	1.406447	−4.022861	−1.616573
C	2.320095	−4.792882	−0.896122
C	2.744915	−6.136771	−1.012146
N	2.296859	−6.965875	−1.983116
N	3.665178	−6.596309	−0.124576
C	4.144110	−5.752665	0.811142
N	3.832478	−4.459217	1.002131
C	2.917402	−4.028006	0.117015
H	0.858268	−1.969031	−1.333863
H	2.550047	−7.946103	−1.957187
H	1.529808	−6.681847	−2.578528
H	4.881041	−6.173855	1.492508
P	4.937727	1.669935	1.501164
O	5.057522	2.101490	0.054728
O	5.395174	2.660915	2.554728
O	5.763910	0.262008	1.636003
C	5.891987	−0.346663	2.954191
C	7.021233	−1.365065	2.951243
O	6.658457	−2.538306	2.164971
C	7.567266	−2.714414	1.048677
C	8.355188	−0.846710	2.377318
C	8.384746	−1.417627	0.957600
O	9.415558	−1.360130	3.240830
H	4.949244	−0.839115	3.218046
H	6.110408	0.430346	3.695173
H	7.171126	−1.688437	3.987924
H	8.197180	−3.588343	1.222729
H	8.411990	0.244086	2.379362
H	7.915684	−0.695937	0.287163
H	9.398547	−1.596876	0.608498
N	6.788848	−3.034918	−0.158119
C	6.786027	−4.381097	−0.659902
O	7.476981	−5.244196	−0.092560
N	6.028666	−4.648779	−1.760413
C	5.277040	−3.699705	−2.326736
N	4.555122	−4.035095	−3.417098
C	5.217540	−2.363535	−1.811061
C	5.979889	−2.087534	−0.722202
H	4.534987	−5.001013	−3.721017
H	3.892197	−3.388725	−3.822901
H	4.583561	−1.609067	−2.260179
H	5.971758	−1.120033	−0.234227
P	11.002426	−1.213561	2.851769
O	11.240128	−0.130053	1.820795
O	11.753650	−1.088440	4.165190
O	11.334326	−2.640139	2.125491
C	11.193883	−3.854703	2.918063
C	11.602016	−5.056364	2.086979
O	10.640649	−5.244420	1.006814
C	11.341829	−5.359171	−0.252231
C	12.999778	−4.965617	1.433397
C	12.693871	−4.664161	−0.039928
O	13.637965	−6.257620	1.612343
H	10.150039	−3.962993	3.233528
H	11.832281	−3.789869	3.806771
H	11.572157	−5.937422	2.741267
H	11.469672	−6.411839	−0.511057
H	13.611293	−4.182966	1.893166
H	12.613350	−3.583058	−0.174165
H	13.463428	−5.045574	−0.715462
H	14.524575	−6.205871	1.210323
N	10.496179	−4.793180	−1.310767
C	9.826254	−5.675403	−2.165900
O	9.921427	−6.905509	−2.100601
N	9.048908	−5.048469	−3.121342
C	8.825474	−3.676520	−3.288852
O	8.092951	−3.274801	−4.206478
C	9.502303	−2.830473	−2.318065
C	9.316823	−1.341441	−2.411929
C	10.286343	−3.428426	−1.385394
H	8.548970	−5.670712	−3.753291
H	9.634065	−0.977824	−3.396602
H	9.899622	−0.822940	−1.647005
H	8.260789	−1.069997	−2.296940
H	10.800595	−2.852611	−0.625806
Na	12.749069	0.989833	3.354513
Na	6.011897	4.220685	0.799888

(AGA)$^{Na+}$

E: −14206.73 kcal mol^{-1}

O	−0.129410	−0.419890	−0.229234
C	−0.114009	0.004455	1.154978
C	1.311033	0.003715	1.695541
O	1.831042	−1.358688	1.840406
C	2.889148	−1.607194	0.880694
C	2.341756	0.746731	0.827751
C	2.951594	−0.367381	−0.024211
O	3.290045	1.372216	1.750123

H	−1.058113	−0.552691	−0.487572
H	−0.721924	−0.670588	1.770469
H	−0.516040	1.023879	1.250294
H	1.283370	0.446434	2.696834
H	3.823874	−1.774969	1.424761
H	1.890737	1.532916	0.218048
H	2.328803	−0.498909	−0.909740
H	3.963801	−0.138077	−0.348260
N	2.633929	−2.852491	0.158961
C	1.785334	−3.069346	−0.920444
N	1.814261	−4.317686	−1.359449
C	2.724230	−4.967532	−0.526074
C	3.205929	−6.295421	−0.480131
N	2.788129	−7.262002	−1.334824
N	4.107976	−6.617708	0.483078
C	4.497074	−5.665404	1.353703
N	4.112127	−4.378638	1.404093
C	3.232301	−4.078395	0.434188
H	1.158450	−2.279655	−1.309328
H	3.297840	−8.137602	−1.356979
H	2.282414	−6.995879	−2.170538
H	5.217765	−5.981101	2.106016
P	4.732686	1.980616	1.268856
O	4.750203	2.326328	−0.205546
O	5.067023	3.098831	2.239758
O	5.752839	0.716505	1.467500
C	5.946656	0.199126	2.814061
C	6.988440	−0.907083	2.808068
O	6.498513	−2.055716	2.049190
C	7.435208	−2.360165	0.983869
C	8.355903	−0.540500	2.190471
C	8.229732	−1.073448	0.763912
O	9.363072	−1.232471	3.005728
H	4.997462	−0.200354	3.189382
H	6.278689	1.007751	3.475661
H	7.135704	−1.218897	3.848808
H	8.088032	−3.181216	1.289332
H	8.569279	0.529944	2.231154
H	7.642893	−0.355749	0.185078
H	9.184783	−1.233647	0.268162
N	6.719896	−2.857868	−0.173958
C	5.890399	−2.181945	−1.063344
N	5.403121	−2.972901	−2.002815
C	5.938725	−4.234056	−1.730622
C	5.802157	−5.483491	−2.410142
O	5.117738	−5.769276	−3.406098
N	6.617459	−6.483776	−1.798734
C	7.430901	−6.305969	−0.698171
N	8.163893	−7.366942	−0.251526
N	7.508471	−5.150513	−0.051120
C	6.765655	−4.172800	−0.601234
H	5.698636	−1.124387	−0.961586
H	6.587738	−7.394398	−2.251810
H	8.323233	−8.132883	−0.897651
H	8.990472	−7.086998	0.281622
P	10.827371	−1.739971	2.486775
O	11.386838	−0.859410	1.386413
O	11.681402	−1.911390	3.728176
O	10.519879	−3.183485	1.785440
C	10.365564	−4.408613	2.553981
C	11.237331	−5.515577	1.969323
O	10.722604	−6.003719	0.691512
C	11.525945	−5.511543	−0.410842
C	12.691811	−5.107858	1.686315
C	12.669044	−4.683335	0.207638
O	13.514233	−6.282097	1.909801
H	9.314315	−4.710494	2.509022
H	10.657436	−4.235720	3.594657
H	11.206347	−6.362832	2.663174
H	11.886242	−6.381102	−0.964930
H	13.017589	−4.291422	2.339674
H	12.466204	−3.614285	0.138608
H	13.620223	−4.885594	−0.290044
H	14.432101	−6.047871	1.680384
N	10.686872	−4.768704	−1.354597
C	10.269664	−3.443100	−1.294658
N	9.416304	−3.122439	−2.252242
C	9.244533	−4.296587	−2.984076
C	8.442756	−4.619860	−4.101219
N	7.630032	−3.718788	−4.711680
N	8.505290	−5.885241	−4.589796
C	9.308867	−6.779089	−3.981329
N	10.092164	−6.593792	−2.902259
C	10.026135	−5.330891	−2.442852
H	10.621484	−2.778292	−0.521280
H	6.925406	−4.082633	−5.344327
H	7.388110	−2.873845	−4.206435
H	9.318098	−7.777999	−4.414735
Na	13.371630	−0.486354	2.709948
Na	5.370999	4.617914	0.371815

(AGC)$^{Na+}$

E: −13807.32 kcal mol^{-1}

O	−0.169535	−0.384536	−0.148097
C	−0.133370	0.029082	1.238868
C	1.300264	0.021571	1.756677
O	1.823416	−1.343819	1.859430
C	2.868675	−1.563368	0.878523
C	2.314232	0.785860	0.887075
C	2.901207	−0.308059	−0.004351
O	3.284564	1.387505	1.802783
H	−1.100937	−0.528112	−0.389998
H	−0.731988	−0.650980	1.857818
H	−0.533328	1.047794	1.348783
H	1.288888	0.440947	2.768275
H	3.812856	−1.727975	1.407488
H	1.851310	1.587768	0.307426
H	2.249171	−0.423401	−0.870499
H	3.899565	−0.066529	−0.359125
N	2.620680	−2.798761	0.137411
C	1.810397	−2.997897	−0.974748
N	1.869032	−4.233864	−1.443884
C	2.759406	−4.894276	−0.597952
C	3.266720	−6.213869	−0.578461
N	2.908956	−7.154355	−1.484807
N	4.138562	−6.551074	0.407452
C	4.478309	−5.620238	1.320675
N	4.072903	−4.340764	1.393793
C	3.223151	−4.025415	0.401699
H	1.189140	−2.206506	−1.368378
H	3.412076	−8.033421	−1.502578
H	2.397675	−6.882478	−2.314673
H	5.173955	−5.948344	2.090975
P	4.734270	1.978942	1.321056
O	4.768870	2.294778	−0.159983
O	5.069298	3.114051	2.271414
O	5.744801	0.712074	1.553547
C	5.922227	0.217519	2.911053
C	6.953978	−0.898638	2.935516
O	6.470049	−2.048080	2.173067
C	7.391928	−2.337987	1.095847
C	8.338106	−0.553137	2.347372
C	8.229320	−1.069888	0.913884
O	9.315869	−1.280958	3.163019
H	4.966214	−0.168172	3.283086

H	6.254104	1.034952	3.561698
H	7.072528	−1.207262	3.980607
H	8.014253	−3.195536	1.365967
H	8.571872	0.512523	2.402045
H	7.681119	−0.330338	0.325491
H	9.194433	−1.252946	0.444607
N	6.662128	−2.757890	−0.086803
C	5.879790	−2.000197	−0.955667
N	5.408498	−2.712487	−1.963037
C	5.906421	−4.002116	−1.762895
C	5.808986	−5.183975	−2.560877
O	5.186588	−5.370380	−3.620467
N	6.583871	−6.241072	−1.999032
C	7.356899	−6.160888	−0.858196
N	8.085305	−7.251051	−0.487465
N	7.405856	−5.067920	−0.107704
C	6.691617	−4.041553	−0.602913
H	5.709058	−0.948167	−0.783907
H	6.593301	−7.100895	−2.543741
H	8.272094	−7.963223	−1.184930
H	8.872405	−7.026031	0.124460
P	10.826360	−1.668810	2.664280
O	11.358599	−0.685771	1.642047
O	11.650258	−1.870586	3.920894
O	10.620366	−3.086111	1.871769
C	10.417034	−4.332709	2.599063
C	11.175577	−5.469682	1.923796
O	10.539850	−5.865845	0.670230
C	11.390199	−5.553879	−0.465209
C	12.637501	−5.146526	1.567884
C	12.568009	−4.734127	0.089065
O	13.402075	−6.364711	1.763824
H	9.346435	−4.562364	2.611123
H	10.779490	−4.221398	3.625867
H	11.135996	−6.334882	2.595633
H	11.714450	−6.487042	−0.923900
H	13.039666	−4.346280	2.198190
H	12.377997	−3.661419	0.018975
H	13.495590	−4.955924	−0.444359
H	14.321053	−6.182480	1.494586
N	10.583832	−4.876356	−1.495546
C	10.196226	−5.597860	−2.671454
O	10.606380	−6.764107	−2.841008
N	9.373208	−4.975917	−3.561066
C	8.942797	−3.725026	−3.344738
N	8.129102	−3.171119	−4.269973
C	9.315329	−2.983738	−2.178377
C	10.115585	−3.609236	−1.277328
H	7.776776	−3.747065	−5.025660
H	7.657007	−2.296679	−4.080357
H	8.948842	−1.979869	−2.003190
H	10.397188	−3.156344	−0.334904
Na	13.278867	−0.264364	3.052937
Na	5.398251	4.581492	0.344815

(AGG)$^{Na+}$

E: −14370.49 kcal mol^{-1}

O	−0.158437	−0.438905	−0.258864
C	−0.123284	0.005397	1.118414
C	1.307512	−0.001348	1.643629
O	1.819024	−1.366625	1.791101
C	2.868746	−1.625374	0.824653
C	2.335557	0.729048	0.761456
C	2.922954	−0.395573	−0.092884
O	3.296576	1.352616	1.672763
H	−1.090209	−0.583478	−0.499269
H	−0.730402	−0.654885	1.750433
H	−0.514785	1.029911	1.203803
H	1.293059	0.448001	2.642272
H	3.807948	−1.787075	1.362908
H	1.885186	1.517221	0.153658
H	2.277613	−0.530364	−0.961768
H	3.928918	−0.177783	−0.443766
N	2.608910	−2.878082	0.119402
C	1.752017	−3.109827	−0.950241
N	1.781349	−4.363264	−1.374799
C	2.701147	−5.000282	−0.541799
C	3.185209	−6.326904	−0.481397
N	2.760184	−7.305992	−1.317380
N	4.097137	−6.633825	0.477770
C	4.493109	−5.668400	1.331003
N	4.105068	−4.382151	1.367901
C	3.214860	−4.098095	0.402690
H	1.119650	−2.326631	−1.343506
H	3.263906	−8.185127	−1.328958
H	2.231917	−7.057287	−2.144373
H	5.222396	−5.971870	2.080043
P	4.777418	1.870475	1.205628
O	4.842673	2.193823	−0.272298
O	5.161052	2.982712	2.165758
O	5.720887	0.555864	1.437284
C	5.919755	0.069833	2.794242
C	7.026428	−0.972419	2.814873
O	6.611711	−2.166352	2.084074
C	7.519290	−2.397182	0.975768
C	8.363696	−0.532313	2.182068
C	8.252265	−1.073988	0.756722
O	9.421159	−1.151409	2.992866
H	4.988827	−0.379599	3.159386
H	6.193409	0.905044	3.449113
H	7.192552	−1.253069	3.861390
H	8.215734	−3.196995	1.235913
H	8.512629	0.549377	2.215314
H	7.632355	−0.380519	0.183942
H	9.209759	−1.193867	0.254633
N	6.783862	−2.900418	−0.168480
C	5.932801	−2.239841	−1.050466
N	5.436726	−3.044087	−1.973470
C	5.984076	−4.299025	−1.696538
C	5.854813	−5.556597	−2.362291
O	5.187728	−5.856197	−3.366828
N	6.660999	−6.552018	−1.728353
C	7.461019	−6.362770	−0.619403
N	8.169830	−7.424451	−0.135135
N	7.550295	−5.194122	−0.001060
C	6.820190	−4.221123	−0.574820
H	5.732611	−1.183006	−0.954309
H	6.623743	−7.472089	−2.162558
H	8.325732	−8.207824	−0.760813
H	8.997944	−7.137402	0.391835
P	10.877401	−1.664248	2.461015
O	11.405223	−0.830255	1.308453
O	11.761918	−1.768771	3.688364
O	10.575861	−3.138090	1.827472
C	10.393977	−4.324134	2.646342
C	11.209291	−5.479371	2.074733
O	10.683775	−5.939065	0.792157
C	11.521511	−5.486870	−0.301131
C	12.687389	−5.152987	1.810323
C	12.711011	−4.736472	0.329538
O	13.441304	−6.369011	2.053517
H	9.331397	−4.588278	2.641519
H	10.720027	−4.126900	3.672877
H	11.123718	−6.321363	2.770521
H	11.829567	−6.374755	−0.857489

H	13.048611	−4.351421	2.463691
H	12.584802	−3.656499	0.253452
H	13.652093	−5.006386	−0.155142
H	14.374303	−6.185526	1.839278
N	10.742313	−4.683771	−1.249028
C	10.473777	−3.312712	−1.233828
N	9.663280	−2.942365	−2.206561
C	9.364307	−4.115050	−2.898638
C	8.538828	−4.332871	−4.043514
O	7.891161	−3.512281	−4.717772
N	8.507800	−5.713464	−4.388120
C	9.183340	−6.723497	−3.737903
N	8.954263	−7.999059	−4.158771
N	9.975990	−6.504093	−2.694453
C	10.024001	−5.206072	−2.315523
H	10.893936	−2.657730	−0.486341
H	7.905656	−5.947575	−5.174782
H	8.587928	−8.156725	−5.090668
H	9.594578	−8.705137	−3.812602
Na	13.415176	−0.377043	2.512975
Na	5.691294	4.436325	0.319667

(AGT)$^{Na+}$

E: −14065.09 kcal mol^{-1}

O	−0.215704	−0.147875	−0.105469
C	−0.113813	0.241967	1.285208
C	1.324997	0.112799	1.771306
O	1.741813	−1.290939	1.829943
C	2.762449	−1.555293	0.833103
C	2.384652	0.815820	0.902502
C	2.846044	−0.296756	−0.037992
O	3.419802	1.295852	1.823234
H	−1.161341	−0.208320	−0.326824
H	−0.750201	−0.397561	1.909879
H	−0.429664	1.287193	1.417702
H	1.364010	0.506797	2.792368
H	3.704985	−1.768209	1.346141
H	1.986533	1.679504	0.364927
H	2.134451	−0.351420	−0.862974
H	3.837165	−0.129934	−0.451695
N	2.445231	−2.772011	0.096074
C	1.565269	−2.944413	−0.965695
N	1.566839	−4.179319	−1.442462
C	2.492655	−4.864294	−0.655338
C	2.968411	−6.195673	−0.665509
N	2.541409	−7.124482	−1.552196
N	3.889848	−6.554391	0.267015
C	4.312577	−5.631481	1.153401
N	3.944141	−4.342504	1.249719
C	3.037085	−4.010359	0.316087
H	0.941039	−2.134662	−1.316243
H	2.994419	−8.029647	−1.579663
H	1.965133	−6.849861	−2.336854
H	5.050635	−5.975724	1.875844
P	4.965430	1.619401	1.400059
O	5.114207	1.957704	−0.068828
O	5.471086	2.646511	2.395624
O	5.722589	0.185063	1.615293
C	5.872768	−0.354161	2.958333
C	7.072224	−1.289935	3.011657
O	6.825268	−2.527684	2.278313
C	7.631042	−2.589535	1.081711
C	8.369041	−0.702002	2.425364
C	8.395134	−1.256298	0.996982
O	9.462224	−1.166841	3.275243
H	4.962917	−0.900125	3.232135
H	6.027903	0.465931	3.668142
H	7.227447	−1.563274	4.060875
H	8.298688	−3.453453	1.153161
H	8.372754	0.390641	2.435858
H	7.873258	−0.550443	0.350158
H	9.406297	−1.382998	0.615938
N	6.786167	−2.847345	−0.086958
C	5.953862	−1.947318	−0.754678
N	5.308153	−2.503319	−1.761567
C	5.719335	−3.835594	−1.765330
C	5.366473	−4.913103	−2.633668
O	4.598994	−4.920522	−3.610554
N	6.036137	−6.110507	−2.250429
C	6.901276	−6.246309	−1.183839
N	7.393650	−7.487671	−0.932650
N	7.237695	−5.230641	−0.396924
C	6.628373	−4.069737	−0.722158
H	5.872176	−0.920162	−0.434444
H	5.806396	−6.928680	−2.810445
H	7.364723	−8.198675	−1.653294
H	8.171536	−7.540829	−0.285174
P	11.032997	−1.121562	2.811081
O	11.295680	−0.060947	1.762308
O	11.849673	−1.040866	4.087417
O	11.234105	−2.570281	2.078831
C	11.064381	−3.770463	2.887853
C	11.545215	−4.979255	2.108947
O	10.633314	−5.237844	0.997422
C	11.401940	−5.411254	−0.213497
C	12.962534	−4.843119	1.503883
C	12.697658	−4.621290	0.008360
O	13.658021	−6.091216	1.760711
H	10.005969	−3.887430	3.146775
H	11.649309	−3.678400	3.810006
H	11.526404	−5.843930	2.784781
H	11.603449	−6.470232	−0.385313
H	13.513715	−4.010389	1.951167
H	12.545109	−3.554975	−0.178796
H	13.515286	−4.978754	−0.622191
H	14.556170	−6.009084	1.390781
N	10.574927	−4.974849	−1.344973
C	10.142746	−5.935665	−2.262888
O	10.450237	−7.130599	−2.211076
N	9.316931	−5.431536	−3.252341
C	8.907223	−4.103471	−3.442550
O	8.173016	−3.817064	−4.400222
C	9.401121	−3.167477	−2.443801
C	9.025917	−1.718322	−2.575811
C	10.189629	−3.649879	−1.450374
H	9.006684	−6.107678	−3.947816
H	9.373296	−1.320097	−3.536600
H	9.470135	−1.122640	−1.775486
H	7.938393	−1.590899	−2.551112
H	10.565564	−3.011788	−0.659203
Na	12.978863	0.930982	3.213137
Na	6.169546	4.070582	0.562532

(ATA)$^{Na+}$

E: −13898.21 kcal mol^{-1}

O	−0.244554	−0.062593	−0.091352
C	−0.138944	0.280383	1.311796
C	1.299721	0.126593	1.788983
O	1.694855	−1.283688	1.863984
C	2.688992	−1.591571	0.859779
C	2.359821	0.806532	0.903145
C	2.839282	−0.329355	−0.002919

O	3.386739	1.329484	1.803733
H	−1.190255	−0.085111	−0.319924
H	−0.781215	−0.372012	1.916535
H	−0.444355	1.323965	1.475478
H	1.354600	0.528350	2.805981
H	3.622506	−1.872386	1.357298
H	1.953947	1.643616	0.330593
H	2.177660	−0.375136	−0.868858
H	3.857747	−0.186108	−0.355897
N	2.287475	−2.773926	0.098301
C	1.280547	−2.871471	−0.856687
N	1.185819	−4.081623	−1.383792
C	2.172671	−4.829074	−0.740841
C	2.585858	−6.176877	−0.862355
N	2.021042	−7.040569	−1.733059
N	3.586687	−6.614564	−0.052248
C	4.140289	−5.750165	0.821712
N	3.847916	−4.451481	1.007563
C	2.856589	−4.039829	0.196235
H	0.645536	−2.026007	−1.084218
H	2.399589	−7.974128	−1.833629
H	1.331886	−6.718788	−2.399584
H	4.933387	−6.156623	1.447100
P	4.888652	1.745868	1.299420
O	4.924083	2.130018	−0.164644
O	5.398357	2.773447	2.292739
O	5.719890	0.342968	1.434425
C	5.918576	−0.212353	2.766402
C	7.060878	−1.212295	2.740998
O	6.668538	−2.411545	2.005013
C	7.581016	−2.631971	0.902056
C	8.356538	−0.697315	2.074855
C	8.279399	−1.291642	0.665769
O	9.469371	−1.175872	2.899892
H	4.997577	−0.707619	3.094385
H	6.160960	0.593863	3.467667
H	7.273189	−1.502395	3.776220
H	8.298245	−3.410035	1.163216
H	8.410796	0.393406	2.057550
H	7.663640	−0.619988	0.064462
H	9.245513	−1.399619	0.180486
N	6.826338	−3.172297	−0.236450
C	6.926052	−4.547867	−0.483152
O	7.628590	−5.316807	0.173464
N	6.160721	−4.980283	−1.553238
C	5.292914	−4.228957	−2.356296
O	4.664800	−4.780987	−3.271239
C	5.218965	−2.817688	−2.008737
C	4.333221	−1.927724	−2.834919
C	5.968326	−2.371389	−0.968440
H	6.223515	−5.975312	−1.759594
H	4.611797	−1.989921	−3.893657
H	4.415391	−0.885921	−2.517942
H	3.284601	−2.238501	−2.763907
H	5.931346	−1.337987	−0.645032
P	10.950519	−1.590578	2.354274
O	11.394636	−0.756230	1.166878
O	11.864890	−1.587881	3.563983
O	10.737359	−3.100493	1.778580
C	10.708031	−4.261962	2.650716
C	11.641111	−5.331748	2.097524
O	11.159200	−5.856121	0.825066
C	11.907649	−5.292318	−0.274613
C	13.073995	−4.849794	1.818108
C	13.040990	−4.441273	0.333410
O	13.956436	−5.976164	2.060648
H	9.682408	−4.644311	2.679127
H	11.025021	−3.985337	3.661432
H	11.651364	−6.169547	2.803846
H	12.283038	−6.130666	−0.866369
H	13.352917	−4.010433	2.464420
H	12.831108	−3.375274	0.255269
H	13.994261	−4.639599	−0.161780
H	14.862009	−5.696096	1.834156
N	11.009219	−4.552116	−1.173690
C	10.737674	−3.190540	−1.248185
N	9.813125	−2.894576	−2.148791
C	9.443319	−4.121652	−2.696389
C	8.503004	−4.483648	−3.686912
N	7.738062	−3.579193	−4.338393
N	8.364264	−5.801859	−3.988238
C	9.132549	−6.700039	−3.339793
N	10.055768	−6.473580	−2.387523
C	10.172314	−5.163040	−2.101096
H	11.231501	−2.467711	−0.615745
H	7.026914	−3.894796	−4.985624
H	7.787470	−2.597846	−4.101041
H	8.982048	−7.740309	−3.624436
Na	13.399581	−0.145957	2.346731
Na	5.907941	4.284044	0.470238

(ATC)$^{Na+}$

E: −13499.97 kcal mol^{-1}

O	−0.189112	−0.063522	−0.057645
C	−0.092208	0.212122	1.360767
C	1.351474	0.077216	1.828486
O	1.789126	−1.322345	1.830627
C	2.777304	−1.551939	0.799192
C	2.386557	0.833615	0.975889
C	2.898653	−0.238769	0.012318
O	3.398265	1.350733	1.897425
H	−1.133797	−0.109311	−0.286776
H	−0.713224	−0.489220	1.932204
H	−0.428522	1.236541	1.577083
H	1.397857	0.427525	2.864687
H	3.717923	−1.845654	1.275778
H	1.950609	1.683746	0.446193
H	2.244154	−0.248046	−0.859707
H	3.913521	−0.047694	−0.326685
N	2.392157	−2.693899	−0.030694
C	1.445559	−2.736932	−1.049822
N	1.350696	−3.928720	−1.616454
C	2.271638	−4.722752	−0.933541
C	2.649494	−6.080092	−1.064156
N	2.132234	−6.897703	−2.005110
N	3.581986	−6.569556	−0.203239
C	4.102108	−5.746464	0.728924
N	3.837534	−4.443909	0.930634
C	2.916037	−3.979804	0.067191
H	0.847510	−1.870634	−1.295021
H	2.398656	−7.873948	−2.029562
H	1.409315	−6.570133	−2.631674
H	4.838242	−6.194155	1.393986
P	4.906124	1.786463	1.430217
O	4.976877	2.175872	−0.031273
O	5.382141	2.814481	2.439366
O	5.748087	0.390797	1.582002
C	5.877219	−0.187362	2.912916
C	6.977383	−1.233103	2.911092
O	6.568107	−2.389844	2.117351
C	7.510305	−2.614302	1.044580
C	8.321625	−0.755810	2.323345
C	8.297998	−1.307334	0.895949
O	9.371814	−1.335636	3.156335
H	4.926363	−0.649294	3.201498

H	6.126618	0.599491	3.633646
H	7.126593	−1.564230	3.945005
H	8.164014	−3.452238	1.295873
H	8.424034	0.331298	2.344267
H	7.772733	−0.589563	0.263683
H	9.296184	−1.456188	0.492266
N	6.773713	−3.049312	−0.152881
C	6.820965	−4.409329	−0.487700
O	7.439186	−5.255957	0.156264
N	6.113579	−4.728378	−1.634963
C	5.361623	−3.876029	−2.452938
O	4.787964	−4.327585	−3.454581
C	5.331562	−2.488152	−2.012935
C	4.562356	−1.497569	−2.841623
C	6.017501	−2.154518	−0.889497
H	6.143728	−5.710224	−1.903443
H	4.917436	−1.512374	−3.878869
H	4.676341	−0.483647	−2.452270
H	3.495822	−1.749596	−2.868410
H	6.008264	−1.143741	−0.499105
P	10.959972	−1.286453	2.747603
O	11.244001	−0.247500	1.683048
O	11.735757	−1.171060	4.046380
O	11.201200	−2.749887	2.056568
C	11.055675	−3.938186	2.889473
C	11.559620	−5.161562	2.142496
O	10.672805	−5.466529	1.026412
C	11.410203	−5.470071	−0.219684
C	12.981319	−5.038018	1.556830
C	12.738187	−4.750326	0.069594
O	13.641497	−6.313990	1.771067
H	9.998313	−4.071924	3.144686
H	11.633732	−3.810992	3.811813
H	11.530758	−6.005829	2.843104
H	11.562735	−6.497027	−0.553043
H	13.550959	−4.238576	2.041925
H	12.643778	−3.672065	−0.079636
H	13.548026	−5.118704	−0.565033
H	14.533242	−6.252725	1.381965
N	10.577558	−4.838482	−1.254778
C	10.010031	−5.634136	−2.299787
O	10.259456	−6.856203	−2.348140
N	9.214316	−5.012793	−3.216558
C	8.955876	−3.701095	−3.125607
N	8.155301	−3.149096	−4.061540
C	9.474003	−2.893851	−2.061861
C	10.268744	−3.511246	−1.150020
H	7.808131	−3.714996	−4.826312
H	7.969240	−2.155721	−4.069370
H	9.235031	−1.840750	−1.980548
H	10.680102	−2.999064	−0.287988
Na	12.862410	0.807825	3.154564
Na	5.915347	4.331189	0.613185

(ATG)$^{Na+}$

E: −14061.90 kcal mol^{-1}

O	−0.250034	−0.148933	−0.032173
C	−0.135751	0.173174	1.375246
C	1.311008	0.040082	1.834073
O	1.734261	−1.362959	1.884253
C	2.721151	−1.637679	0.863258
C	2.347390	0.753734	0.946643
C	2.840767	−0.359562	0.019508
O	3.371267	1.287912	1.843970
H	−1.197431	−0.178766	−0.252822
H	−0.758209	−0.501793	1.976106
H	−0.459739	1.207667	1.559658
H	1.369773	0.427965	2.856246
H	3.664577	−1.911083	1.345886
H	1.917739	1.589395	0.389462
H	2.172994	−0.403817	−0.841805
H	3.853508	−0.192503	−0.339799
N	2.330291	−2.814439	0.087501
C	1.322600	−2.912229	−0.866588
N	1.238056	−4.118133	−1.405101
C	2.232530	−4.862679	−0.770611
C	2.657828	−6.205495	−0.905410
N	2.102748	−7.064994	−1.785771
N	3.663409	−6.641758	−0.100110
C	4.209136	−5.780935	0.782383
N	3.905303	−4.486810	0.980621
C	2.910419	−4.076206	0.173019
H	0.678776	−2.071196	−1.085113
H	2.476905	−8.000511	−1.883256
H	1.396240	−6.749618	−2.436865
H	5.005888	−6.186266	1.403738
P	4.863208	1.732450	1.334693
O	4.883397	2.132946	−0.125341
O	5.363490	2.756911	2.335863
O	5.715629	0.340930	1.449115
C	5.923699	−0.228182	2.773833
C	7.080959	−1.209922	2.733882
O	6.705099	−2.406822	1.986475
C	7.621151	−2.606280	0.881955
C	8.365826	−0.665111	2.069643
C	8.302681	−1.255230	0.657607
O	9.488725	−1.115435	2.896624
H	5.010308	−0.740699	3.096526
H	6.155313	0.572547	3.485030
H	7.302119	−1.507401	3.765209
H	8.348043	−3.377365	1.137275
H	8.394099	0.426588	2.054496
H	7.680720	−0.588303	0.057496
H	9.272256	−1.348132	0.175594
N	6.873093	−3.147313	−0.260563
C	6.973300	−4.522847	−0.503141
O	7.668740	−5.292362	0.160599
N	6.214431	−4.957379	−1.577544
C	5.340552	−4.210076	−2.380146
O	4.706828	−4.769648	−3.285901
C	5.261603	−2.799509	−2.031954
C	4.365628	−1.912822	−2.850402
C	6.010163	−2.350573	−0.992579
H	6.253442	−5.958041	−1.759712
H	4.645963	−1.963360	−3.909167
H	4.435881	−0.872860	−2.523880
H	3.320471	−2.235760	−2.780715
H	5.966510	−1.317663	−0.668499
P	10.972825	−1.524030	2.354876
O	11.414097	−0.697246	1.161636
O	11.884875	−1.504825	3.566712
O	10.770077	−3.040269	1.793666
C	10.726413	−4.189835	2.680326
C	11.645222	−5.276922	2.137634
O	11.155819	−5.805737	0.870415
C	11.912081	−5.262078	−0.234617
C	13.083902	−4.815126	1.853764
C	13.054826	−4.418358	0.366140
O	13.952394	−5.950497	2.105327
H	9.696144	−4.559259	2.715423
H	11.048497	−3.905051	3.687232
H	11.645203	−6.108189	2.851717
H	12.279087	−6.111398	−0.815787
H	13.373732	−3.974078	2.493077
H	12.855659	−3.351046	0.280090

H	14.005767	−4.630328	−0.127968
H	14.861042	−5.683919	1.874924
N	11.025814	−4.520152	−1.143304
C	10.781414	−3.149248	−1.241229
N	9.877169	−2.858149	−2.157824
C	9.490005	−4.087245	−2.690741
C	8.547906	−4.399250	−3.716086
O	7.835285	−3.635099	−4.391589
N	8.466833	−5.806751	−3.916432
C	9.209864	−6.762321	−3.252364
N	8.967048	−8.064708	−3.561243
N	10.094841	−6.456560	−2.308392
C	10.192105	−5.129073	−2.068652
H	11.277970	−2.427145	−0.610795
H	7.808317	−6.103012	−4.633976
H	8.514565	−8.296332	−4.437709
H	9.630681	−8.744098	−3.206909
Na	13.422113	−0.074874	2.366862
Na	5.836717	4.289066	0.515437

(ATT)$^{Na+}$

E: −13758.38 kcal mol^{-1}

O	−0.208907	−0.069533	0.038206
C	−0.052380	0.235079	1.445287
C	1.408707	0.100354	1.857435
O	1.839188	−1.301074	1.866105
C	2.775187	−1.554073	0.791864
C	2.412902	0.834179	0.949576
C	2.863687	−0.256625	−0.022817
O	3.471754	1.355303	1.816247
H	−1.162349	−0.123383	−0.148900
H	−0.653043	−0.450454	2.056189
H	−0.374388	1.265763	1.653798
H	1.498318	0.468093	2.884733
H	3.736005	−1.841504	1.229038
H	1.963489	1.684059	0.430807
H	2.160173	−0.272787	−0.855705
H	3.858465	−0.079371	−0.421641
N	2.350648	−2.711369	0.005151
C	1.373383	−2.775521	−0.983464
N	1.248160	−3.983847	−1.507666
C	2.180241	−4.766636	−0.826950
C	2.540373	−6.131420	−0.924615
N	1.989762	−6.972827	−1.825733
N	3.494895	−6.602683	−0.077614
C	4.052434	−5.755431	0.810443
N	3.806901	−4.444406	0.977667
C	2.863228	−3.999390	0.128346
H	0.778126	−1.910840	−1.240236
H	2.225205	−7.957288	−1.805139
H	1.227199	−6.665733	−2.414895
H	4.805075	−6.189253	1.466219
P	5.004818	1.644879	1.324235
O	5.102221	1.974984	−0.150400
O	5.571861	2.663830	2.294759
O	5.730765	0.190110	1.518518
C	5.855647	−0.343548	2.868250
C	7.003974	−1.335910	2.916699
O	6.666747	−2.542285	2.166434
C	7.579628	−2.729925	1.064221
C	8.327029	−0.809178	2.325463
C	8.366035	−1.420232	0.921010
O	9.397464	−1.276115	3.199909
H	4.919579	−0.838946	3.148850
H	6.054795	0.473160	3.570685
H	7.156028	−1.623344	3.963000
H	8.237738	−3.575186	1.274761
H	8.361056	0.282085	2.293140
H	7.881678	−0.726168	0.234022
H	9.382556	−1.590549	0.575276
N	6.811960	−3.133852	−0.131798
C	6.792764	−4.495648	−0.459009
O	7.405891	−5.363547	0.162979
N	6.028281	−4.795423	−1.574714
C	5.275861	−3.919839	−2.370102
O	4.633954	−4.358829	−3.334112
C	5.327603	−2.528457	−1.946474
C	4.590840	−1.509104	−2.769197
C	6.058618	−2.216235	−0.845981
H	5.988956	−5.783087	−1.819404
H	4.918615	−1.564157	−3.813927
H	4.775474	−0.497177	−2.402093
H	3.510942	−1.697360	−2.763082
H	6.089392	−1.205345	−0.457018
P	10.980673	−1.101445	2.802852
O	11.188085	−0.031848	1.751348
O	11.732664	−0.937791	4.110822
O	11.338354	−2.533727	2.099756
C	11.229417	−3.735082	2.917459
C	11.640525	−4.945016	2.101084
O	10.665310	−5.161342	1.037736
C	11.353418	−5.313437	−0.223819
C	13.028651	−4.848937	1.426122
C	12.700235	−4.598506	−0.052456
O	13.693265	−6.121828	1.637868
H	10.193111	−3.852141	3.254006
H	11.882458	−3.644586	3.793055
H	11.631129	−5.815973	2.769258
H	11.488453	−6.372773	−0.449543
H	13.630284	−4.040388	1.852795
H	12.607408	−3.523105	−0.220637
H	13.464984	−4.993941	−0.725225
H	14.578584	−6.062536	1.234068
N	10.484229	−4.790649	−1.287080
C	9.818897	−5.707356	−2.108977
O	9.943018	−6.932056	−2.020178
N	8.993670	−5.117678	−3.051111
C	8.754622	−3.750946	−3.259156
O	7.984499	−3.387960	−4.160688
C	9.452685	−2.868433	−2.337009
C	9.261958	−1.383878	−2.480047
C	10.262710	−3.430313	−1.403991
H	8.520411	−5.763633	−3.680421
H	9.576869	−1.052970	−3.476989
H	9.844627	−0.838098	−1.734253
H	8.205543	−1.111608	−2.374719
H	10.791404	−2.825349	−0.677853
Na	12.676149	1.152141	3.257917
Na	6.237168	4.061147	0.428406

(CAA)$^{Na+}$

E: −13638.37 kcal mol^{-1}

O	−0.137125	−0.137066	−0.511676
C	−0.055257	0.280914	0.873214
C	1.347759	0.046076	1.421745
O	1.625448	−1.384022	1.560608
C	2.719168	−1.773469	0.678619
C	2.498513	0.611801	0.564808
C	2.924814	−0.593370	−0.274490
O	3.517331	1.097929	1.506174
H	−1.077566	−0.150748	−0.761281
H	−0.767957	−0.285071	1.486113

H	−0.287119	1.352102	0.964929
H	1.385607	0.490929	2.422348
H	3.608691	−1.968084	1.275231
H	2.189677	1.461164	−0.048641
H	2.253700	−0.654290	−1.133293
H	3.944531	−0.531004	−0.642946
N	2.398194	−3.051164	0.038029
C	3.066344	−4.239647	0.490879
O	3.891781	−4.158438	1.418791
N	2.762467	−5.414719	−0.128604
C	1.840806	−5.462117	−1.098213
N	1.617975	−6.653958	−1.693948
C	1.099656	−4.305096	−1.508688
C	1.407574	−3.127676	−0.903874
H	2.133409	−7.471302	−1.390847
H	0.907793	−6.761027	−2.405439
H	0.318908	−4.363642	−2.258302
H	0.878770	−2.204553	−1.115518
P	5.124076	1.124404	1.222250
O	5.456045	1.400433	−0.233178
O	5.724144	2.071572	2.244118
O	5.592879	−0.412922	1.498907
C	5.899668	−0.906357	2.829893
C	7.286152	−1.543291	2.839853
O	7.325417	−2.779607	2.070374
C	7.907700	−2.548113	0.769168
C	8.396199	−0.662327	2.236809
C	8.436606	−1.102850	0.764769
O	9.617352	−0.890633	3.015562
H	5.149350	−1.658082	3.094897
H	5.871435	−0.084976	3.552991
H	7.527803	−1.807984	3.874463
H	8.700882	−3.287305	0.645337
H	8.170026	0.402262	2.331619
H	7.778104	−0.438843	0.207995
H	9.429162	−1.028084	0.326968
N	6.934401	−2.829045	−0.295750
C	6.176003	−1.957404	−1.068981
N	5.396749	−2.580562	−1.939268
C	5.643973	−3.936852	−1.733052
C	5.145641	−5.104558	−2.348050
N	4.251495	−5.068685	−3.372001
N	5.615076	−6.303397	−1.924846
C	6.531797	−6.328087	−0.937546
N	7.073543	−5.288708	−0.277501
C	6.595550	−4.111339	−0.715702
H	6.224091	−0.885803	−0.945785
H	3.776644	−5.930620	−3.616054
H	3.751431	−4.205516	−3.547113
H	6.877613	−7.316774	−0.639689
P	11.104421	−1.223361	2.432996
O	11.485275	−0.364670	1.241594
O	12.033524	−1.165530	3.630976
O	10.971136	−2.742278	1.856250
C	10.839758	−3.874448	2.759533
C	11.572650	−5.069444	2.166080
O	10.918617	−5.531762	0.946006
C	11.715058	−5.201444	−0.218340
C	13.034268	−4.794091	1.773757
C	12.947743	−4.427905	0.283198
O	13.773819	−6.022768	1.996968
H	9.775771	−4.105379	2.879725
H	11.272728	−3.628151	3.735063
H	11.524347	−5.889623	2.891684
H	11.982905	−6.138681	−0.712794
H	13.467715	−3.982458	2.368292
H	12.808725	−3.351246	0.186066
H	13.849583	−4.712444	−0.263813
H	14.689901	−5.874407	1.698897
N	10.889052	−4.472797	−1.183696
C	10.634804	−3.108438	−1.277148
N	9.726453	−2.811966	−2.193037
C	9.349171	−4.041329	−2.733085
C	8.408481	−4.408867	−3.721208
N	7.646572	−3.505371	−4.391100
N	8.280994	−5.724913	−4.026752
C	9.036057	−6.622909	−3.365948
N	9.939589	−6.395120	−2.395334
C	10.059874	−5.084560	−2.118179
H	11.134484	−2.391032	−0.643098
H	6.855741	−3.858271	−4.919002
H	7.558880	−2.569739	−4.012759
H	8.889580	−7.663721	−3.650722
Na	13.482995	0.348282	2.435911
Na	6.822638	3.264874	0.431230

(CAC)$^{Na+}$

E: −13240.15 kcal mol^{-1}

O	−0.061934	−0.075529	−0.580973
C	−0.061362	0.302987	0.817628
C	1.317673	0.084494	1.430096
O	1.627289	−1.342563	1.531748
C	2.775210	−1.669511	0.694082
C	2.492858	0.708352	0.652232
C	2.987767	−0.452437	−0.210065
O	3.458892	1.161997	1.660043
H	−0.987007	−0.091032	−0.882418
H	−0.790107	−0.297533	1.376602
H	−0.323874	1.365250	0.928120
H	1.293435	0.493176	2.446438
H	3.639050	−1.857695	1.331634
H	2.195026	1.576138	0.059238
H	2.357259	−0.500711	−1.099521
H	4.019402	−0.340742	−0.529914
N	2.526771	−2.935846	0.000222
C	3.181305	−4.124041	0.473212
O	3.930857	−4.052087	1.463681
N	2.951386	−5.288262	−0.196348
C	2.105198	−5.328606	−1.233131
N	1.937907	−6.512902	−1.859874
C	1.379935	−4.173311	−1.675103
C	1.616918	−3.006250	−1.020225
H	2.475230	−7.318552	−1.563780
H	1.322020	−6.602635	−2.656442
H	0.662009	−4.225837	−2.485451
H	1.087684	−2.088939	−1.252574
P	5.048488	1.411987	1.374654
O	5.338873	1.712266	−0.082806
O	5.510750	2.440575	2.389580
O	5.727559	−0.042579	1.673510
C	5.891360	−0.533166	3.031247
C	7.145188	−1.395688	3.118820
O	6.994009	−2.652584	2.394845
C	7.736447	−2.621174	1.155360
C	8.413179	−0.742926	2.539992
C	8.442669	−1.254156	1.095337
O	9.534062	−1.198135	3.360709
H	5.013968	−1.129712	3.304146
H	5.986384	0.311987	3.721580
H	7.297592	−1.651140	4.172770
H	8.440234	−3.458585	1.167786
H	8.380611	0.348527	2.586894
H	7.888609	−0.547896	0.478897
H	9.452742	−1.326189	0.699232
N	6.855907	−2.874038	0.011746

C	6.137335	−1.964985	−0.756541
N	5.472714	−2.535325	−1.748217
C	5.763055	−3.893707	−1.636386
C	5.383486	−5.017426	−2.401811
N	4.589129	−4.920665	−3.497275
N	5.865153	−6.232576	−2.040713
C	6.675389	−6.314358	−0.966460
N	7.096822	−5.321249	−0.163282
C	6.615703	−4.126003	−0.546143
H	6.134251	−0.909811	−0.531517
H	4.205281	−5.771625	−3.891363
H	4.085046	−4.058960	−3.665285
H	7.033013	−7.312933	−0.720871
P	11.095071	−1.130157	2.867567
O	11.331851	−0.038753	1.843709
O	11.933251	−1.077086	4.131299
O	11.300379	−2.554978	2.088933
C	11.153493	−3.782091	2.862002
C	11.535091	−4.975357	2.005685
O	10.555608	−5.141717	0.937442
C	11.237754	−5.263104	−0.335560
C	12.924702	−4.887206	1.333095
C	12.599910	−4.583189	−0.134806
O	13.562464	−6.180987	1.502844
H	10.113229	−3.879044	3.192680
H	11.805712	−3.741964	3.741858
H	11.507643	−5.865135	2.648248
H	11.341052	−6.314383	−0.608236
H	13.543877	−4.106725	1.786520
H	12.519031	−3.501302	−0.271186
H	13.358739	−4.967350	−0.821173
H	14.442817	−6.131430	1.087162
N	10.387274	−4.664748	−1.369646
C	9.808671	−5.490161	−2.386158
O	10.087991	−6.705115	−2.424232
N	8.970978	−4.900724	−3.283960
C	8.695530	−3.592535	−3.213632
N	7.862017	−3.074851	−4.144960
C	9.263254	−2.745056	−2.207244
C	10.092546	−3.331695	−1.306726
H	7.361178	−3.710839	−4.755193
H	7.482892	−2.144391	−4.025430
H	9.028928	−1.689044	−2.151839
H	10.545099	−2.788791	−0.485741
Na	13.029925	0.931818	3.289266
Na	6.452444	3.758468	0.572079

(CAG)$^{Na+}$

E: −13803.69 kcal mol^{-1}

O	−0.028414	−0.200034	−0.618994
C	0.040034	0.237347	0.760656
C	1.434108	−0.001224	1.329572
O	1.696750	−1.431240	1.494283
C	2.798145	−1.845126	0.634373
C	2.600785	0.541712	0.478978
C	3.033993	−0.680999	−0.331359
O	3.606287	1.041833	1.426594
H	−0.966461	−0.217657	−0.877194
H	−0.684968	−0.313642	1.372804
H	−0.183619	1.311719	0.834256
H	1.463804	0.458918	2.323554
H	3.675307	−2.044884	1.247570
H	2.305073	1.380033	−0.155825
H	2.379720	−0.749577	−1.202557
H	4.061842	−0.635712	−0.680531
N	2.470698	−3.127031	0.004549
C	3.109433	−4.319383	0.488322
O	3.912881	−4.238908	1.435424
N	2.803027	−5.497371	−0.124109
C	1.905459	−5.542795	−1.116017
N	1.686449	−6.736563	−1.709682
C	1.186455	−4.381645	−1.553350
C	1.497023	−3.201641	−0.955311
H	2.182327	−7.557570	−1.384469
H	0.986219	−6.843655	−2.430943
H	0.420692	−4.438902	−2.318368
H	0.983837	−2.274560	−1.186761
P	5.215129	1.078069	1.154158
O	5.559166	1.336112	−0.301091
O	5.798159	2.042837	2.169582
O	5.693732	−0.451685	1.456297
C	5.899951	−0.939839	2.808185
C	7.265414	−1.609745	2.913442
O	7.326040	−2.861892	2.170365
C	7.985890	−2.667653	0.899495
C	8.432318	−0.766950	2.368000
C	8.564372	−1.240380	0.911023
O	9.585691	−1.039103	3.227170
H	5.114757	−1.668648	3.034159
H	5.848692	−0.110518	3.521124
H	7.434918	−1.861429	3.965373
H	8.757153	−3.438606	0.825258
H	8.231459	0.305725	2.426622
H	7.980755	−0.565357	0.287873
H	9.590421	−1.213481	0.553838
N	7.057407	−2.926304	−0.212095
C	6.356696	−2.037107	−1.021550
N	5.582658	−2.641307	−1.907483
C	5.771354	−4.002781	−1.678964
C	5.238607	−5.158503	−2.287940
N	4.366854	−5.106882	−3.329774
N	5.647570	−6.369527	−1.835491
C	6.525846	−6.416369	−0.815148
N	7.086838	−5.389845	−0.150769
C	6.681147	−4.200219	−0.627014
H	6.439113	−0.966622	−0.910687
H	3.858586	−5.951917	−3.564983
H	3.918968	−4.224771	−3.545880
H	6.820317	−7.413861	−0.492678
P	11.148183	−1.039695	2.749205
O	11.435893	−0.027382	1.658581
O	11.967510	−0.919052	4.021412
O	11.331560	−2.515074	2.070242
C	11.114657	−3.693388	2.898229
C	11.600440	−4.928873	2.158842
O	10.754272	−5.188486	0.997287
C	11.531266	−5.092614	−0.224030
C	13.044643	−4.844317	1.630484
C	12.864942	−4.436857	0.161762
O	13.627966	−6.167156	1.767197
H	10.045326	−3.788600	3.118054
H	11.667433	−3.591515	3.839454
H	11.507191	−5.782304	2.841395
H	11.668701	−6.098938	−0.630354
H	13.638947	−4.114051	2.189402
H	12.798755	−3.349281	0.101879
H	13.686984	−4.776873	−0.472502
H	14.531688	−6.129434	1.403838
N	10.758348	−4.361521	−1.220428
C	10.619255	−2.981108	−1.371960
N	9.760095	−2.658338	−2.319257
C	9.294426	−3.875235	−2.817959
C	8.350602	−4.159014	−3.852144
O	7.718453	−3.374894	−4.581491
N	8.167763	−5.562106	−3.997633

C	8.826273	−6.544496	−3.286731
N	8.503720	−7.832162	−3.558699
N	9.716992	−6.267505	−2.337721
C	9.901749	−4.944019	−2.143266
H	11.168575	−2.285117	−0.755537
H	7.485988	−5.835117	−4.702261
H	7.996789	−8.070204	−4.401555
H	9.081258	−8.554666	−3.146559
Na	13.161859	0.960793	3.061444
Na	6.920549	3.209378	0.348640

(CAT)$^{Na+}$

E: −13499.25 kcal mol^{-1}

O	−0.074514	−0.051294	−0.474219
C	−0.007827	0.336273	0.920234
C	1.394746	0.106846	1.472940
O	1.691497	−1.322090	1.578347
C	2.791399	−1.677640	0.689946
C	2.542203	0.709514	0.637456
C	2.991607	−0.468345	−0.228058
O	3.546257	1.193628	1.595687
H	−1.012642	−0.065766	−0.732387
H	−0.717410	−0.252425	1.515115
H	−0.254025	1.402057	1.034489
H	1.420157	0.526891	2.484563
H	3.678908	−1.883727	1.286522
H	2.223651	1.567521	0.041366
H	2.329929	−0.512632	−1.094837
H	4.013040	−0.377170	−0.585545
N	2.483347	−2.940599	0.013024
C	3.136282	−4.139833	0.460693
O	3.934041	−4.078652	1.413478
N	2.850677	−5.302168	−0.191007
C	1.954756	−5.329747	−1.185686
N	1.736891	−6.512760	−1.799970
C	1.231862	−4.162098	−1.597822
C	1.523454	−2.997090	−0.960912
H	2.254597	−7.332727	−1.508420
H	1.064739	−6.600433	−2.549900
H	0.474438	−4.203482	−2.372213
H	1.001551	−2.070226	−1.171540
P	5.157611	1.246077	1.347407
O	5.525018	1.476913	−0.106925
O	5.715011	2.234284	2.354070
O	5.653811	−0.271688	1.681168
C	5.854096	−0.748000	3.036961
C	7.164028	−1.523852	3.113873
O	7.101091	−2.775573	2.368586
C	7.807183	−2.661525	1.113557
C	8.383346	−0.775094	2.546000
C	8.462251	−1.267987	1.093988
O	9.526881	−1.144050	3.377309
H	5.019758	−1.405301	3.303276
H	5.891707	0.097496	3.731818
H	7.335976	−1.787907	4.162557
H	8.540892	−3.471373	1.078003
H	8.267021	0.310454	2.599296
H	7.903787	−0.571498	0.470608
H	9.484340	−1.303605	0.724172
N	6.907637	−2.903016	−0.021907
C	6.161083	−1.996700	−0.769016
N	5.454303	−2.572736	−1.726854
C	5.738500	−3.931659	−1.613121
C	5.302291	−5.062377	−2.336984
N	4.449863	−4.975935	−3.388194
N	5.784363	−6.279267	−1.977134
C	6.633158	−6.356975	−0.932602
N	7.103954	−5.356728	−0.165989
C	6.630373	−4.159327	−0.553392
H	6.172404	−0.937669	−0.561594
H	4.021195	−5.827159	−3.732185
H	3.956582	−4.106800	−3.550111
H	6.983599	−7.356905	−0.683488
P	11.084838	−1.011840	2.890366
O	11.282351	0.086705	1.866316
O	11.911928	−0.923146	4.159939
O	11.350776	−2.427984	2.115096
C	11.211067	−3.658920	2.881391
C	11.552037	−4.845190	2.001659
O	10.539825	−4.976517	0.958003
C	11.194781	−5.181509	−0.314489
C	12.926473	−4.772776	1.291443
C	12.567773	−4.511124	−0.178535
O	13.573179	−6.057677	1.484866
H	10.179868	−3.747636	3.241729
H	11.889476	−3.636984	3.742057
H	11.528475	−5.744900	2.630277
H	11.287741	−6.249279	−0.522164
H	13.552590	−3.976597	1.705434
H	12.500691	−3.433362	−0.346213
H	13.302795	−4.929128	−0.870599
H	14.455990	−6.007547	1.074384
N	10.325455	−4.644376	−1.367581
C	9.663230	−5.544235	−2.210474
O	9.790618	−6.769780	−2.149536
N	8.839184	−4.934561	−3.140214
C	8.626983	−3.562916	−3.342304
O	7.887997	−3.181528	−4.261889
C	9.325029	−2.698233	−2.401844
C	9.153293	−1.209338	−2.520805
C	10.121262	−3.279839	−1.470299
H	8.357119	−5.568602	−3.774726
H	9.506804	−0.858126	−3.497865
H	9.714641	−0.687061	−1.742128
H	8.096734	−0.929825	−2.444078
H	10.651695	−2.690127	−0.732742
Na	12.914712	1.135514	3.340059
Na	6.901420	3.339004	0.532988

(CCA)$^{Na+}$

E: −13236.27 kcal mol^{-1}

O	−0.229965	0.033388	−0.345098
C	−0.129179	0.369668	1.060753
C	1.282293	0.104530	1.572952
O	1.562308	−1.331324	1.627467
C	2.636415	−1.674143	0.706403
C	2.417309	0.719141	0.729347
C	2.838803	−0.438784	−0.176901
O	3.448847	1.163045	1.675191
H	−1.173708	0.032641	−0.582406
H	−0.833256	−0.231492	1.649447
H	−0.359304	1.433544	1.217503
H	1.337482	0.491203	2.596513
H	3.533598	−1.918318	1.273451
H	2.093766	1.595016	0.162463
H	2.166985	−0.451688	−1.037228
H	3.859072	−0.346426	−0.539749
N	2.289530	−2.903518	−0.013702
C	2.968587	−4.123655	0.323745
O	3.826369	−4.114435	1.225294
N	2.641992	−5.246473	−0.376527
C	1.691737	−5.216169	−1.319686

N	1.452347	−6.353594	−2.007254
C	0.938298	−4.031492	−1.610927
C	1.267831	−2.905421	−0.925995
H	1.959455	−7.198901	−1.774638
H	0.717543	−6.402264	−2.700054
H	0.133734	−4.031552	−2.337334
H	0.734200	−1.968381	−1.045694
P	5.046905	1.231628	1.343795
O	5.327240	1.579237	−0.105687
O	5.660847	2.140740	2.391759
O	5.545221	−0.309495	1.539526
C	5.864840	−0.850634	2.850451
C	7.227286	−1.536711	2.808617
O	7.187651	−2.776004	2.050391
C	7.804068	−2.604692	0.749232
C	8.338368	−0.691172	2.157812
C	8.324990	−1.159173	0.693311
O	9.578121	−0.919409	2.909383
H	5.093347	−1.579454	3.119196
H	5.885625	−0.045143	3.591283
H	7.501924	−1.797733	3.836487
H	8.613931	−3.326955	0.682097
H	8.137199	0.379282	2.238641
H	7.655453	−0.492852	0.152349
H	9.301939	−1.099832	0.219129
N	6.857269	−3.001575	−0.316398
C	6.754554	−4.410177	−0.588059
O	7.445688	−5.201679	0.077494
N	5.907252	−4.817847	−1.574312
C	5.154187	−3.935438	−2.236074
N	4.356645	−4.400893	−3.226870
C	5.186993	−2.536299	−1.943053
C	6.047438	−2.122122	−0.973653
H	4.249527	−5.401341	−3.343677
H	3.678679	−3.795014	−3.669385
H	4.556283	−1.829598	−2.468558
H	6.111539	−1.084154	−0.680868
P	11.020859	−1.388595	2.316770
O	11.449453	−0.593541	1.095487
O	11.977237	−1.371971	3.494098
O	10.759888	−2.901983	1.778215
C	10.707897	−4.047369	2.669765
C	11.611946	−5.144159	2.120424
O	11.100402	−5.672267	0.860757
C	11.859701	−5.155408	−0.256624
C	13.049976	−4.692534	1.815724
C	13.005405	−4.304581	0.326114
O	13.915784	−5.830745	2.063022
H	9.671960	−4.398704	2.711810
H	11.041277	−3.763531	3.673308
H	11.616196	−5.973412	2.836779
H	12.223158	−6.017170	−0.821782
H	13.351855	−3.849181	2.446300
H	12.800677	−3.238495	0.235230
H	13.952047	−4.516360	−0.176308
H	14.823423	−5.568623	1.823546
N	10.971391	−4.431918	−1.176224
C	10.689803	−3.072668	−1.265720
N	9.755678	−2.794675	−2.161763
C	9.389577	−4.031248	−2.691747
C	8.437095	−4.414098	−3.660690
N	7.656581	−3.518231	−4.320806
N	8.321681	−5.732300	−3.961701
C	9.105968	−6.616575	−3.315168
N	10.028914	−6.372642	−2.366337
C	10.132793	−5.059677	−2.091510
H	11.185273	−2.339350	−0.646515
H	6.848682	−3.873632	−4.820150
H	7.588889	−2.575816	−3.955812
H	8.971694	−7.660180	−3.596055
Na	13.506324	−0.015589	2.200347
Na	6.690979	3.446075	0.644723

(CCC)$^{Na+}$

E: −12839.92 kcal mol^{-1}

O	−0.122540	0.193890	−0.515928
C	−0.165869	0.459799	0.908321
C	1.186593	0.162736	1.546363
O	1.448356	−1.277657	1.579052
C	2.599074	−1.606116	0.753288
C	2.399920	0.790876	0.834882
C	2.905427	−0.341563	−0.060404
O	3.347920	1.172018	1.884274
H	−1.037245	0.207827	−0.847827
H	−0.925634	−0.166698	1.391998
H	−0.411153	1.515659	1.092834
H	1.145979	0.513998	2.583196
H	3.432502	−1.894838	1.395027
H	2.138991	1.687103	0.267769
H	2.337717	−0.322426	−0.992588
H	3.960807	−0.241811	−0.300892
N	2.297621	−2.800852	−0.045405
C	2.934200	−4.044962	0.284859
O	3.720705	−4.091131	1.248433
N	2.651589	−5.129243	−0.490308
C	1.779302	−5.042511	−1.503209
N	1.569601	−6.147861	−2.248293
C	1.074696	−3.830664	−1.804862
C	1.359662	−2.743288	−1.042157
H	2.053444	−7.008306	−2.021759
H	0.902763	−6.149652	−3.008207
H	0.335581	−3.783205	−2.596369
H	0.851680	−1.792319	−1.164011
P	4.889260	1.625535	1.563320
O	5.062080	2.094333	0.133756
O	5.289565	2.596171	2.658627
O	5.727000	0.225566	1.699689
C	5.872100	−0.382291	3.016457
C	7.031792	−1.368297	3.017507
O	6.699036	−2.568213	2.257458
C	7.567971	−2.698921	1.102794
C	8.340003	−0.827952	2.409049
C	8.302293	−1.356642	0.973452
O	9.433720	−1.382537	3.204898
H	4.944916	−0.905489	3.276755
H	6.065017	0.400607	3.758008
H	7.203212	−1.669247	4.057239
H	8.256810	−3.529885	1.262841
H	8.402378	0.261896	2.447821
H	7.741114	−0.645959	0.364744
H	9.296429	−1.460526	0.546270
N	6.763942	−3.088099	−0.064106
C	6.775314	−4.457798	−0.494112
O	7.465359	−5.285237	0.128262
N	6.031264	−4.789410	−1.586295
C	5.268359	−3.879109	−2.200727
N	4.567814	−4.269808	−3.286737
C	5.180116	−2.523888	−1.743674
C	5.935356	−2.185028	−0.667203
H	4.565258	−5.246219	−3.555182
H	3.915780	−3.643638	−3.739193
H	4.528428	−1.803852	−2.222726
H	5.908566	−1.197305	−0.224225
P	11.005560	−1.255568	2.754014
O	11.216411	−0.167602	1.721425

O	11.813682	−1.152977	4.034624
O	11.291358	−2.679728	2.001769
C	11.249964	−3.903367	2.793944
C	11.805975	−5.064064	1.986181
O	10.898736	−5.393963	0.893081
C	11.587042	−5.306704	−0.378853
C	13.191641	−4.824045	1.351525
C	12.868645	−4.500450	−0.112935
O	13.940934	−6.061014	1.490248
H	10.212458	−4.114540	3.076955
H	11.850230	−3.771358	3.701157
H	11.862585	−5.930521	2.657244
H	11.802917	−6.309555	−0.748612
H	13.727553	−4.006573	1.844938
H	12.689489	−3.427384	−0.215357
H	13.676712	−4.783906	−0.791965
H	14.806281	−5.930923	1.060713
N	10.671536	−4.711379	−1.362792
C	10.127893	−5.516992	−2.412163
O	10.479405	−6.710908	−2.516754
N	9.240374	−4.938861	−3.268982
C	8.871549	−3.659829	−3.116997
N	7.987675	−3.148672	−4.000325
C	9.381452	−2.840156	−2.058946
C	10.264171	−3.416007	−1.203401
H	7.558383	−3.756779	−4.687055
H	7.612227	−2.217812	−3.879764
H	9.063057	−1.812867	−1.933385
H	10.673337	−2.897402	−0.344423
Na	12.835335	0.897757	3.189130
Na	5.946052	4.206149	0.958985

(CCG)[Na+]

E: −13400.49 kcal mol^{-1}

O	−0.225263	−0.040433	−0.335881
C	−0.124181	0.293262	1.070655
C	1.293835	0.051662	1.575532
O	1.596296	−1.379506	1.633087
C	2.671120	−1.709866	0.709021
C	2.415152	0.681370	0.724810
C	2.855194	−0.473483	−0.177279
O	3.440705	1.145525	1.666013
H	−1.169871	−0.054982	−0.569414
H	−0.814850	−0.322136	1.660672
H	−0.372735	1.352396	1.231668
H	1.348580	0.441989	2.597743
H	3.572992	−1.944337	1.273451
H	2.074496	1.548198	0.154008
H	2.184582	−0.499923	−1.038252
H	3.874426	−0.367174	−0.540039
N	2.335438	−2.943972	−0.008861
C	3.029376	−4.156484	0.325286
O	3.890321	−4.137993	1.223614
N	2.712045	−5.282943	−0.373604
C	1.755614	−5.263692	−1.310730
N	1.522432	−6.404507	−1.994867
C	0.987276	−4.087538	−1.597629
C	1.308489	−2.957647	−0.915125
H	2.040937	−7.243935	−1.766397
H	0.784514	−6.460618	−2.683691
H	0.177786	−4.096831	−2.318459
H	0.763383	−2.026866	−1.031820
P	5.027119	1.295493	1.301141
O	5.258877	1.659938	−0.151720
O	5.614529	2.230558	2.341740
O	5.604039	−0.220025	1.483960
C	5.878086	−0.769092	2.802570
C	7.217163	−1.499616	2.792193
O	7.149034	−2.753735	2.059984
C	7.819986	−2.641602	0.777242
C	8.364367	−0.702497	2.143420
C	8.366620	−1.207715	0.692101
O	9.576707	−0.959341	2.928905
H	5.077372	−1.469235	3.063161
H	5.913479	0.039192	3.540176
H	7.468391	−1.746234	3.829601
H	8.620225	−3.378846	0.762832
H	8.194898	0.375485	2.192593
H	7.714381	−0.546745	0.123262
H	9.350147	−1.178016	0.229881
N	6.906347	−3.057284	−0.306590
C	6.828682	−4.465387	−0.586249
O	7.516026	−5.252265	0.089514
N	6.005742	−4.879807	−1.590035
C	5.234571	−4.007189	−2.245869
N	4.444883	−4.481547	−3.236370
C	5.226679	−2.612243	−1.929938
C	6.073087	−2.189947	−0.953630
H	4.368432	−5.481680	−3.374240
H	3.771936	−3.882119	−3.694293
H	4.576622	−1.915713	−2.445442
H	6.107892	−1.156944	−0.638913
P	11.048990	−1.359596	2.357666
O	11.467572	−0.533825	1.154400
O	11.980960	−1.321561	3.554416
O	10.857366	−2.877549	1.801761
C	10.771432	−4.017211	2.698174
C	11.632295	−5.144560	2.143417
O	11.098831	−5.647988	0.883697
C	11.868708	−5.147452	−0.233261
C	13.085147	−4.745605	1.837286
C	13.051102	−4.348109	0.350168
O	13.908259	−5.917156	2.077180
H	9.725160	−4.335660	2.753759
H	11.124281	−3.741589	3.697551
H	11.607007	−5.975711	2.857203
H	12.195220	−6.017284	−0.808564
H	13.420963	−3.917574	2.470969
H	12.889946	−3.274147	0.267940
H	13.986488	−4.595122	−0.157226
H	14.824369	−5.688332	1.835733
N	11.005903	−4.378656	−1.141127
C	10.823333	−2.999731	−1.258735
N	9.919835	−2.682717	−2.167771
C	9.468642	−3.901160	−2.674326
C	8.496359	−4.188299	−3.679530
O	7.810316	−3.403820	−4.360610
N	8.359972	−5.590763	−3.863794
C	9.056134	−6.566988	−3.184625
N	8.738836	−7.863364	−3.458499
N	9.971675	−6.288658	−2.261911
C	10.131236	−4.963483	−2.045061
H	11.357204	−2.291083	−0.643645
H	7.656720	−5.868537	−4.544783
H	8.274758	−8.088430	−4.330875
H	9.379809	−8.564812	−3.104938
Na	13.487909	0.097616	2.298940
Na	6.535124	3.601257	0.581469

(CCT)[Na+]

E: −13098.92 kcal mol^{-1}

O	−0.078109	0.214233	−0.461996

C	−0.123718	0.456388	0.966365
C	1.226170	0.143356	1.602248
O	1.481775	−1.298508	1.611316
C	2.634908	−1.617488	0.784848
C	2.443313	0.777950	0.903123
C	2.946107	−0.341681	−0.009176
O	3.392628	1.136321	1.959555
H	−0.991636	0.240557	−0.796351
H	−0.887073	−0.175057	1.437858
H	−0.365383	1.509962	1.168154
H	1.184818	0.477840	2.644630
H	3.465877	−1.916965	1.424742
H	2.187530	1.684810	0.350767
H	2.379850	−0.307089	−0.941879
H	4.002216	−0.241944	−0.245501
N	2.333251	−2.800224	−0.031184
C	2.969235	−4.049201	0.280812
O	3.759668	−4.108677	1.240515
N	2.681559	−5.123322	−0.506612
C	1.808108	−5.021723	−1.516880
N	1.595949	−6.116830	−2.276479
C	1.104754	−3.804902	−1.801207
C	1.393069	−2.728058	−1.025039
H	2.080568	−6.980167	−2.062990
H	0.928216	−6.107987	−3.035551
H	0.364679	−3.745876	−2.590990
H	0.886845	−1.774528	−1.133430
P	4.931629	1.600371	1.641188
O	5.095055	2.106551	0.223634
O	5.335850	2.542782	2.759876
O	5.773530	0.198498	1.737179
C	5.912612	−0.445586	3.037591
C	7.073945	−1.429027	3.018748
O	6.741291	−2.619463	2.245587
C	7.615044	−2.752929	1.096333
C	8.380782	−0.872298	2.420083
C	8.391994	−1.431736	0.994485
O	9.473350	−1.366676	3.254016
H	4.984368	−0.975551	3.279500
H	6.101823	0.317028	3.800954
H	7.249485	−1.743562	4.053938
H	8.276312	−3.608735	1.241240
H	8.410353	0.219489	2.429918
H	7.889158	−0.716855	0.341941
H	9.402214	−1.580304	0.620766
N	6.806632	−3.087602	−0.087494
C	6.770696	−4.445585	−0.552201
O	7.446463	−5.308848	0.037271
N	6.005618	−4.728096	−1.642903
C	5.261471	−3.780916	−2.224734
N	4.528679	−4.126102	−3.302882
C	5.216089	−2.437028	−1.730100
C	5.991877	−2.147386	−0.654743
H	4.511095	−5.091024	−3.608941
H	3.904180	−3.465780	−3.744818
H	4.580709	−1.686821	−2.183795
H	5.992902	−1.172445	−0.182735
P	11.046705	−1.175596	2.829645
O	11.231354	−0.074683	1.806096
O	11.825224	−1.045855	4.126560
O	11.397404	−2.584195	2.078185
C	11.317534	−3.810027	2.861698
C	11.719216	−4.991400	1.999631
O	10.715730	−5.189655	0.959751
C	11.368402	−5.301346	−0.324922
C	13.085833	−4.859348	1.287377
C	12.710202	−4.571815	−0.172870
O	13.770842	−6.130108	1.442582
H	10.290223	−3.946699	3.217896
H	11.990001	−3.739335	3.724356
H	11.740263	−5.880642	2.643109
H	11.512033	−6.353205	−0.579228
H	13.691091	−4.056265	1.719276
H	12.596776	−3.493628	−0.307095
H	13.459982	−4.936352	−0.879294
H	14.638742	−6.051643	1.005736
N	10.466949	−4.766393	−1.352868
C	9.789832	−5.671937	−2.177349
O	9.913018	−6.898804	−2.102140
N	8.968341	−5.071281	−3.112648
C	8.716657	−3.705221	−3.291814
O	7.954678	−3.328533	−4.195598
C	9.401689	−2.835141	−2.348062
C	9.181816	−1.351327	−2.448804
C	10.225549	−3.407024	−1.434021
H	8.463960	−5.710365	−3.723963
H	9.474646	−0.987485	−3.440982
H	9.765900	−0.814963	−1.697257
H	8.122202	−1.102773	−2.317886
H	10.750356	−2.812727	−0.696398
Na	12.740484	1.072098	3.312412
Na	5.971901	4.209075	1.126152

(CGA)$^{Na+}$

E: −13802.48 kcal mol^{-1}

O	−0.206148	−0.228073	−0.309277
C	−0.103672	0.127620	1.091792
C	1.330205	−0.038292	1.580950
O	1.702123	−1.451549	1.657253
C	2.782843	−1.745283	0.725121
C	2.408773	0.632344	0.705686
C	2.898624	−0.514614	−0.180052
O	3.414459	1.174600	1.627123
H	−1.151791	−0.276660	−0.533834
H	−0.756428	−0.512886	1.698161
H	−0.400981	1.175534	1.243396
H	1.378595	0.371437	2.595944
H	3.700009	−1.928152	1.284543
H	2.016305	1.468216	0.122072
H	2.222909	−0.587086	−1.034329
H	3.907170	−0.368205	−0.556882
N	2.497947	−3.003174	0.027272
C	3.241673	−4.180425	0.378216
O	4.085260	−4.118421	1.290748
N	2.987372	−5.324659	−0.318212
C	2.027175	−5.360902	−1.250407
N	1.824513	−6.529166	−1.899869
C	1.200878	−4.226579	−1.544566
C	1.472309	−3.074593	−0.877406
H	2.477027	−7.291312	−1.761571
H	1.187634	−6.580683	−2.683863
H	0.389399	−4.282207	−2.261117
H	0.884708	−2.171620	−1.004523
P	4.996603	1.375328	1.274924
O	5.237699	1.660337	−0.195333
O	5.525064	2.399980	2.261594
O	5.641237	−0.095239	1.556397
C	5.941179	−0.549998	2.902863
C	7.275014	−1.289197	2.906602
O	7.198134	−2.538860	2.165107
C	7.833800	−2.398926	0.872454
C	8.442353	−0.514112	2.265777
C	8.398845	−0.973307	0.802317
O	9.657849	−0.857986	3.012094
H	5.145079	−1.227683	3.229689

H	5.995962	0.305486	3.584146
H	7.513675	−1.544452	3.944567
H	8.616215	−3.156642	0.819288
H	8.321673	0.567919	2.356356
H	7.720669	−0.305778	0.272457
H	9.369792	−0.933222	0.312254
N	6.904867	−2.735185	−0.205847
C	6.114816	−1.927364	−1.021163
N	5.456709	−2.620471	−1.934988
C	5.820272	−3.949975	−1.717496
C	5.465347	−5.146964	−2.409664
O	4.722596	−5.300016	−3.394601
N	6.126010	−6.275171	−1.839796
C	7.006200	−6.245952	−0.778631
N	7.544107	−7.429755	−0.358835
N	7.320322	−5.127692	−0.141006
C	6.714832	−4.033061	−0.644258
H	6.063864	−0.857025	−0.889511
H	5.928442	−7.162665	−2.296850
H	7.561111	−8.210445	−1.005691
H	8.380864	−7.328163	0.208666
P	11.093402	−1.335099	2.401865
O	11.527456	−0.523266	1.195775
O	12.049260	−1.358219	3.579419
O	10.813338	−2.838430	1.834993
C	10.630612	−3.973907	2.725409
C	11.348070	−5.188645	2.147628
O	10.707891	−5.656064	0.921837
C	11.528673	−5.351203	−0.230959
C	12.820560	−4.939852	1.778134
C	12.762642	−4.586717	0.283752
O	13.538247	−6.177556	2.023484
H	9.558141	−4.176606	2.815008
H	11.043311	−3.745478	3.713822
H	11.273542	−6.000462	2.880543
H	11.793272	−6.295856	−0.713053
H	13.256569	−4.129777	2.372996
H	12.635189	−3.509632	0.171642
H	13.669985	−4.885276	−0.246575
H	14.461188	−6.044457	1.739796
N	10.736576	−4.619747	−1.222923
C	10.427909	−3.264162	−1.265739
N	9.570067	−2.956826	−2.225154
C	9.284766	−4.169897	−2.850546
C	8.422988	−4.522073	−3.912461
N	7.656942	−3.615900	−4.576523
N	8.374983	−5.824743	−4.294126
C	9.131729	−6.724132	−3.635620
N	9.959226	−6.511618	−2.596246
C	10.001022	−5.214600	−2.241812
H	10.857899	−2.564098	−0.564910
H	6.900953	−3.979324	−5.147503
H	7.493480	−2.717000	−4.137132
H	9.053805	−7.752865	−3.984226
Na	13.600545	0.011209	2.336357
Na	6.422552	3.677897	0.404030

(CGC)$^{Na+}$

E: −13404.90 kcal mol^{-1}

O	−0.206400	−0.251987	−0.225375
C	−0.110792	0.069635	1.184383
C	1.329111	−0.067032	1.664965
O	1.743334	−1.470143	1.699613
C	2.823708	−1.706747	0.751728
C	2.380709	0.658139	0.802793
C	2.900492	−0.446158	−0.117251
O	3.386683	1.178423	1.731985
H	−1.150354	−0.328900	−0.448219
H	−0.742568	−0.605618	1.774998
H	−0.437940	1.104064	1.364163
H	1.370407	0.315491	2.690819
H	3.748458	−1.884291	1.303225
H	1.962548	1.501231	0.248149
H	2.231418	−0.513815	−0.976784
H	3.903331	−0.250506	−0.485369
N	2.566443	−2.952105	0.019079
C	3.303727	−4.133387	0.369939
O	4.107857	−4.089379	1.318501
N	3.090353	−5.260328	−0.365882
C	2.162047	−5.283554	−1.330819
N	2.001269	−6.433779	−2.020227
C	1.330115	−4.151834	−1.619518
C	1.568278	−3.013851	−0.916857
H	2.639090	−7.204072	−1.860457
H	1.372007	−6.483644	−2.810156
H	0.540581	−4.198235	−2.360906
H	0.972332	−2.116273	−1.040000
P	4.854307	1.739784	1.275385
O	4.939499	2.009444	−0.212890
O	5.183633	2.899180	2.198980
O	5.839972	0.469438	1.573246
C	6.004272	0.008025	2.942827
C	7.138845	−1.003647	3.025907
O	6.803345	−2.227898	2.306423
C	7.626633	−2.355888	1.122419
C	8.487797	−0.544912	2.442584
C	8.420054	−1.048245	1.000081
O	9.521736	−1.194878	3.250701
H	5.074310	−0.465646	3.277561
H	6.228269	0.860283	3.595126
H	7.262104	−1.265881	4.082395
H	8.279865	−3.224221	1.246518
H	8.633782	0.536174	2.504211
H	7.869104	−0.312214	0.413069
H	9.401464	−1.187217	0.550504
N	6.803249	−2.663974	−0.039012
C	6.049402	−1.809418	−0.841457
N	5.466491	−2.442117	−1.844587
C	5.849214	−3.776951	−1.706669
C	5.583803	−4.919344	−2.522222
O	4.898898	−5.006523	−3.556656
N	6.258236	−6.069868	−2.019569
C	7.078107	−6.104012	−0.910813
N	7.668318	−7.290214	−0.582223
N	7.291798	−5.042038	−0.146693
C	6.675654	−3.928205	−0.586668
H	5.971930	−0.754921	−0.622675
H	6.134117	−6.916757	−2.570213
H	7.779258	−7.993653	−1.304120
H	8.454100	−7.200480	0.056673
P	11.064645	−1.430635	2.755207
O	11.500474	−0.411913	1.722519
O	11.900179	−1.528872	4.017287
O	11.001607	−2.874164	1.984643
C	10.766654	−4.087278	2.759926
C	11.309767	−5.299414	2.016638
O	10.497023	−5.595313	0.841448
C	11.294407	−5.498191	−0.364251
C	12.761315	−5.156526	1.517370
C	12.597470	−4.787465	0.036717
O	13.402102	−6.447856	1.694421
H	9.689805	−4.204640	2.924686
H	11.271744	−4.004591	3.728303
H	11.239107	−6.156474	2.697625
H	11.475238	−6.497263	−0.760718

H	13.308070	−4.388687	2.074658
H	12.501623	−3.703362	−0.058021
H	13.443698	−5.116375	−0.571628
H	14.307644	−6.377912	1.340513
N	10.502725	−4.806331	−1.393995
C	10.019156	−5.534401	−2.526420
O	10.343189	−6.730133	−2.674640
N	9.206660	−4.882152	−3.405601
C	8.887884	−3.593895	−3.222969
N	8.091240	−3.005731	−4.143757
C	9.366417	−2.842262	−2.102271
C	10.149934	−3.498163	−1.208232
H	7.655307	−3.582798	−4.853868
H	7.684184	−2.097766	−3.960280
H	9.090148	−1.805530	−1.955634
H	10.514532	−3.036486	−0.297974
Na	13.338900	0.246579	3.169472
Na	5.604222	4.297095	0.248258

(CGG)$^{Na+}$

E: −13967.08 kcal mol^{-1}

O	−0.160413	−0.286924	−0.328730
C	−0.029763	0.101116	1.061229
C	1.409516	−0.074509	1.531354
O	1.761864	−1.490949	1.632412
C	2.824567	−1.819766	0.690782
C	2.483588	0.561918	0.624802
C	2.941479	−0.611513	−0.244018
O	3.509456	1.119501	1.516890
H	−1.110300	−0.328294	−0.536049
H	−0.681533	−0.514680	1.693686
H	−0.309375	1.156660	1.192119
H	1.479786	0.356717	2.536127
H	3.748051	−1.998470	1.239107
H	2.092469	1.389817	0.029275
H	2.247188	−0.692082	−1.082685
H	3.944610	−0.492272	−0.644154
N	2.516646	−3.091432	0.029956
C	3.260675	−4.264163	0.395874
O	4.127458	−4.183133	1.285136
N	2.980345	−5.425896	−0.260767
C	1.998337	−5.480175	−1.169292
N	1.770046	−6.664933	−1.778385
C	1.178154	−4.346073	−1.481338
C	1.473135	−3.178273	−0.852534
H	2.403316	−7.438210	−1.615203
H	1.102133	−6.740034	−2.533888
H	0.352565	−4.414274	−2.180440
H	0.892404	−2.272904	−0.994245
P	5.107336	1.191611	1.195024
O	5.400349	1.450889	−0.271710
O	5.697622	2.176533	2.186847
O	5.628176	−0.324317	1.488742
C	5.946009	−0.789313	2.826996
C	7.333213	−1.423649	2.830745
O	7.362801	−2.664288	2.071255
C	7.947700	−2.447167	0.767003
C	8.434209	−0.545157	2.207068
C	8.455781	−0.995638	0.737871
O	9.667980	−0.757498	2.972590
H	5.199455	−1.536183	3.116199
H	5.923741	0.046537	3.533721
H	7.587050	−1.676868	3.865475
H	8.753805	−3.173548	0.662362
H	8.208117	0.519519	2.298548
H	7.775467	−0.348050	0.187038
H	9.440921	−0.914762	0.282976
N	6.990739	−2.772808	−0.297142
C	6.197512	−1.950977	−1.098643
N	5.489592	−2.633785	−1.980537
C	5.817721	−3.970852	−1.757133
C	5.396514	−5.163146	−2.418880
O	4.623714	−5.303048	−3.383905
N	6.020639	−6.308392	−1.844021
C	6.924715	−6.297804	−0.801956
N	7.407015	−7.502696	−0.369786
N	7.311038	−5.183300	−0.200108
C	6.741730	−4.070304	−0.708825
H	6.177317	−0.877807	−0.979380
H	5.764998	−7.197069	−2.269031
H	7.393758	−8.279718	−1.022588
H	8.258804	−7.430813	0.179432
P	11.119294	−1.242094	2.409488
O	11.573681	−0.466532	1.185833
O	12.050158	−1.212700	3.606894
O	10.867512	−2.762098	1.883630
C	10.639322	−3.867862	2.799155
C	11.316475	−5.113479	2.240793
O	10.693697	−5.536564	0.991525
C	11.557238	−5.248121	−0.132613
C	12.810971	−4.937936	1.921020
C	12.822508	−4.572322	0.428365
O	13.456685	−6.212155	2.181108
H	9.559645	−4.029160	2.884993
H	11.055919	−3.634131	3.785006
H	11.177987	−5.925577	2.964065
H	11.775899	−6.195839	−0.631327
H	13.268660	−4.155134	2.535898
H	12.773301	−3.488579	0.323254
H	13.724001	−4.928374	−0.075887
H	14.393868	−6.124416	1.928113
N	10.837970	−4.444047	−1.126583
C	10.716910	−3.056529	−1.228468
N	9.909247	−2.688239	−2.205717
C	9.462285	−3.878329	−2.778094
C	8.564276	−4.108795	−3.864341
O	7.969834	−3.289007	−4.586746
N	8.378075	−5.504412	−4.076989
C	8.989373	−6.518849	−3.370391
N	8.630647	−7.798118	−3.678949
N	9.838456	−6.291267	−2.376072
C	10.028287	−4.977565	−2.117502
H	11.225562	−2.384292	−0.553455
H	7.734069	−5.743217	−4.828351
H	8.228500	−7.991261	−4.589418
H	9.230402	−8.526074	−3.305780
Na	13.619517	0.116897	2.313658
Na	6.722414	3.367088	0.335605

(CGT)$^{Na+}$

E: −13663.58 kcal mol^{-1}

O	−0.173304	−0.211124	−0.163233
C	−0.063150	0.114798	1.244565
C	1.375749	−0.049456	1.719721
O	1.761910	−1.460264	1.758175
C	2.835175	−1.719580	0.807279
C	2.441750	0.650180	0.853172
C	2.926636	−0.467341	−0.070428
O	3.459790	1.152746	1.782574
H	−1.120184	−0.269846	−0.379884
H	−0.705550	−0.544668	1.841562
H	−0.368248	1.156489	1.421429

H	1.427839	0.335777	2.744098
H	3.759934	−1.904712	1.354139
H	2.043390	1.505057	0.301917
H	2.239278	−0.524932	−0.916486
H	3.927609	−0.302869	−0.458407
N	2.558035	−2.968689	0.091218
C	3.298560	−4.148376	0.440369
O	4.146079	−4.087406	1.349423
N	3.038546	−5.293036	−0.252451
C	2.073926	−5.328256	−1.180905
N	1.871056	−6.494644	−1.829989
C	1.255143	−4.189230	−1.478705
C	1.531166	−3.037765	−0.812831
H	2.457475	−7.293504	−1.623035
H	1.174707	−6.576510	−2.558109
H	0.443471	−4.241913	−2.195176
H	0.945471	−2.133751	−0.939727
P	5.001578	1.506213	1.374961
O	5.164141	1.838808	−0.094806
O	5.475924	2.550865	2.368038
O	5.787422	0.092964	1.602571
C	5.958394	−0.447431	2.939632
C	7.217990	−1.300822	2.986810
O	7.070399	−2.541470	2.235352
C	7.803886	−2.485985	0.993651
C	8.472946	−0.614237	2.416417
C	8.546416	−1.135998	0.973521
O	9.590092	−1.001021	3.275230
H	5.085259	−1.057171	3.197208
H	6.053894	0.371849	3.661182
H	7.386804	−1.582130	4.031537
H	8.484043	−3.342132	0.973642
H	8.396940	0.475469	2.443492
H	8.042633	−0.416352	0.329437
H	9.570487	−1.239032	0.620733
N	6.907938	−2.673171	−0.155193
C	6.168863	−1.717327	−0.855750
N	5.454124	−2.242111	−1.832051
C	5.716453	−3.609494	−1.780991
C	5.228554	−4.675649	−2.595085
O	4.444835	−4.635850	−3.559342
N	5.776230	−5.920161	−2.177175
C	6.638393	−6.105655	−1.116371
N	6.999811	−7.386335	−0.822901
N	7.093405	−5.103666	−0.372955
C	6.607977	−3.896338	−0.738127
H	6.191896	−0.672743	−0.588991
H	5.454213	−6.730409	−2.702056
H	6.897850	−8.103145	−1.532303
H	7.807194	−7.488149	−0.217652
P	11.157158	−0.982405	2.804510
O	11.435650	0.054993	1.736080
O	11.975988	−0.887866	4.078608
O	11.337082	−2.446894	2.097240
C	11.101938	−3.626810	2.919796
C	11.470129	−4.870230	2.136201
O	10.514900	−5.055009	1.046900
C	11.242179	−5.345565	−0.167638
C	12.881712	−4.850708	1.499659
C	12.602859	−4.659483	0.002016
O	13.496932	−6.133141	1.788762
H	10.045035	−3.664763	3.207231
H	11.716635	−3.573096	3.825589
H	11.399329	−5.729761	2.815275
H	11.350417	−6.424352	−0.295329
H	13.496576	−4.042980	1.907727
H	12.534173	−3.591126	−0.220116
H	13.374955	−5.104032	−0.630593
H	14.398627	−6.117062	1.418601

N	10.435979	−4.887555	−1.303945
C	9.883048	−5.846155	−2.157668
O	10.052681	−7.062705	−2.034348
N	9.110491	−5.310756	−3.173745
C	8.842416	−3.958578	−3.435645
O	8.145301	−3.646894	−4.412805
C	9.430646	−3.029055	−2.482702
C	9.197826	−1.556666	−2.676396
C	10.181734	−3.538060	−1.473762
H	8.724425	−5.987096	−3.829961
H	9.611916	−1.225507	−3.636423
H	9.669607	−0.977221	−1.879202
H	8.126501	−1.329928	−2.693417
H	10.630671	−2.900156	−0.721596
Na	13.133288	1.053931	3.168308
Na	6.200180	3.940390	0.504711

(CTA)$^{Na+}$

E: −13496.28 kcal mol^{-1}

O	−0.303226	0.088535	−0.052371
C	−0.124801	0.358851	1.360361
C	1.324331	0.118044	1.767414
O	1.644472	−1.310930	1.749723
C	2.667263	−1.590446	0.754170
C	2.378638	0.797596	0.869233
C	2.784979	−0.319377	−0.092334
O	3.452663	1.268877	1.751230
H	−1.259306	0.073842	−0.232818
H	−0.770653	−0.294372	1.960175
H	−0.377718	1.405252	1.584822
H	1.440236	0.469723	2.798399
H	3.602609	−1.835414	1.255990
H	1.981665	1.670090	0.345542
H	2.070243	−0.326120	−0.917233
H	3.780958	−0.182918	−0.504920
N	2.308060	−2.797005	0.001098
C	3.051433	−4.006766	0.217909
O	3.966859	−4.017612	1.061587
N	2.724720	−5.096061	−0.531828
C	1.709735	−5.049948	−1.405330
N	1.455090	−6.157203	−2.132479
C	0.891857	−3.882728	−1.569090
C	1.225162	−2.787202	−0.839436
H	2.050794	−6.970580	−2.037003
H	0.713301	−6.174256	−2.819142
H	0.040140	−3.873219	−2.239278
H	0.651847	−1.866256	−0.865680
P	5.021964	1.389355	1.309014
O	5.196581	1.748685	−0.152870
O	5.676623	2.310640	2.321306
O	5.576844	−0.139569	1.467316
C	5.845477	−0.687152	2.788818
C	7.160572	−1.455742	2.770749
O	7.035154	−2.712639	2.046143
C	7.735481	−2.649421	0.783694
C	8.323604	−0.700911	2.098522
C	8.291091	−1.224464	0.654234
O	9.537880	−0.981210	2.871198
H	5.026882	−1.360667	3.063605
H	5.912676	0.125056	3.519753
H	7.419579	−1.703479	3.805730
H	8.537972	−3.384168	0.808549
H	8.184579	0.381758	2.134885
H	7.625698	−0.568484	0.095037
H	9.263804	−1.208026	0.168517
N	6.845375	−3.107054	−0.308764

C	6.815752	−4.491561	−0.528523
O	7.464038	−5.301286	0.137494
N	6.010850	−4.882400	−1.583354
C	5.211201	−4.076706	−2.401257
O	4.566999	−4.582941	−3.333104
C	5.230436	−2.665226	−2.054683
C	4.393229	−1.721399	−2.872628
C	6.026244	−2.259594	−1.029980
H	5.995943	−5.882291	−1.773293
H	4.641991	−1.812837	−3.936679
H	4.556492	−0.685221	−2.567904
H	3.326179	−1.954399	−2.775298
H	6.053755	−1.222564	−0.723645
P	10.985466	−1.456661	2.294321
O	11.420464	−0.678690	1.065123
O	11.933978	−1.429518	3.477248
O	10.722238	−2.978097	1.775368
C	10.671693	−4.110313	2.684215
C	11.573704	−5.215112	2.148732
O	11.068410	−5.746371	0.887887
C	11.836785	−5.236794	−0.225484
C	13.016426	−4.773050	1.853011
C	12.985961	−4.393016	0.360978
O	13.874333	−5.914037	2.114030
H	9.636313	−4.462731	2.734239
H	11.006272	−3.811991	3.682980
H	11.567436	−6.040495	2.869491
H	12.196397	−6.102046	−0.787754
H	13.317519	−3.927543	2.481059
H	12.791943	−3.325617	0.263279
H	13.934157	−4.615868	−0.133510
H	14.785668	−5.656451	1.883632
N	10.958636	−4.507036	−1.151967
C	10.712414	−3.143148	−1.267362
N	9.792812	−2.857650	−2.176475
C	9.399959	−4.093789	−2.687211
C	8.451707	−4.469110	−3.664815
N	7.710809	−3.568323	−4.353648
N	8.288189	−5.792248	−3.925625
C	9.041429	−6.684629	−3.251876
N	9.970302	−6.445947	−2.308219
C	10.110716	−5.130136	−2.061424
H	11.217870	−2.410733	−0.655052
H	6.943138	−3.896123	−4.927345
H	7.728894	−2.592959	−4.086151
H	8.871335	−7.730120	−3.504477
Na	13.470222	−0.077372	2.185012
Na	6.576410	3.635564	0.509385

(CTC)$^{Na+}$

E: −13099.11 kcal mol^{-1}

O	−0.249306	0.107110	−0.076429
C	−0.129428	0.302974	1.354400
C	1.308051	0.065563	1.800104
O	1.649135	−1.357516	1.744319
C	2.709273	−1.596473	0.780975
C	2.379161	0.793555	0.962708
C	2.873695	−0.286700	−0.000007
O	3.395706	1.271858	1.900831
H	−1.197268	0.092305	−0.296060
H	−0.785143	−0.392083	1.893571
H	−0.409153	1.331674	1.624233
H	1.386453	0.381444	2.846049
H	3.619561	−1.883117	1.309819
H	1.977088	1.661031	0.434650
H	2.234952	−0.271948	−0.884750
H	3.898656	−0.120483	−0.321191
N	2.365119	−2.757899	−0.052215
C	3.072587	−3.993526	0.128737
O	3.946654	−4.073510	1.011865
N	2.761756	−5.031667	−0.697146
C	1.780719	−4.919838	−1.602847
N	1.539608	−5.978558	−2.403042
C	0.988000	−3.731044	−1.728880
C	1.314088	−2.682932	−0.929696
H	2.105072	−6.814239	−2.317136
H	0.811502	−5.951958	−3.103897
H	0.159900	−3.669137	−2.425468
H	0.755755	−1.752579	−0.925284
P	4.902759	1.717347	1.441062
O	4.978302	2.103985	−0.020914
O	5.368067	2.751963	2.449278
O	5.755198	0.329874	1.601616
C	5.906568	−0.231271	2.937179
C	7.035671	−1.247027	2.944859
O	6.656294	−2.435309	2.186081
C	7.549746	−2.622557	1.065614
C	8.358041	−0.748326	2.329665
C	8.310907	−1.300621	0.902896
O	9.433880	−1.311739	3.141792
H	4.970389	−0.717245	3.233761
H	6.136579	0.570361	3.647931
H	7.203817	−1.549938	3.984400
H	8.227087	−3.452356	1.277630
H	8.445038	0.340195	2.348009
H	7.761835	−0.588707	0.285249
H	9.302102	−1.432289	0.476308
N	6.775370	−3.056259	−0.109541
C	6.770856	−4.425276	−0.409843
O	7.346811	−5.278136	0.267550
N	6.086078	−4.744507	−1.568840
C	5.357015	−3.890175	−2.403285
O	4.795058	−4.338685	−3.413407
C	5.336350	−2.499714	−1.972305
C	4.567667	−1.511259	−2.804171
C	6.027205	−2.161452	−0.853114
H	6.084361	−5.732563	−1.814262
H	4.901147	−1.550603	−3.847843
H	4.704657	−0.492538	−2.434927
H	3.496331	−1.745883	−2.804399
H	6.026816	−1.146729	−0.473895
P	11.011699	−1.252088	2.698836
O	11.267435	−0.209986	1.630146
O	11.814875	−1.132897	3.980816
O	11.247961	−2.713889	2.001030
C	11.122092	−3.902397	2.836968
C	11.602995	−5.126875	2.077248
O	10.688354	−5.427364	0.981667
C	11.399536	−5.446748	−0.279007
C	13.011364	−5.009053	1.458835
C	12.735222	−4.728597	−0.023826
O	13.674428	−6.284601	1.665425
H	10.071853	−4.033262	3.120892
H	11.725326	−3.777263	3.743269
H	11.587230	−5.971591	2.777751
H	11.541396	−6.477041	−0.606951
H	13.592828	−4.207785	1.926589
H	12.637382	−3.650880	−0.176414
H	13.530040	−5.100880	−0.674959
H	14.559029	−6.225299	1.260034
N	10.548325	−4.819462	−1.301364
C	9.982977	−5.612544	−2.349150
O	10.248297	−6.830684	−2.411319
N	9.170601	−4.993282	−3.252185
C	8.898850	−3.685054	−3.148825

N	8.088314	−3.133691	−4.075814
C	9.419977	−2.879559	−2.084947
C	10.229241	−3.495132	−1.185163
H	7.716586	−3.703211	−4.826078
H	7.867983	−2.147629	−4.057696
H	9.170890	−1.829629	−1.994246
H	10.646210	−2.985420	−0.324237
Na	12.914314	0.847047	3.072584
Na	5.916508	4.261265	0.631497

(CTG)$^{Na+}$

E: −13660.21 kcal mol^{-1}

O	−0.311304	−0.007636	−0.032265
C	−0.137347	0.249131	1.383433
C	1.316924	0.035833	1.786100
O	1.668468	−1.385452	1.759910
C	2.694101	−1.638470	0.760425
C	2.355124	0.743408	0.891437
C	2.789482	−0.358453	−0.075825
O	3.416529	1.230429	1.777931
H	−1.266987	−0.026844	−0.214771
H	−0.768080	−0.425239	1.976081
H	−0.412736	1.287045	1.620878
H	1.427102	0.383641	2.819040
H	3.634119	−1.873989	1.259179
H	1.938678	1.609352	0.371853
H	2.080419	−0.374376	−0.905471
H	3.785109	−0.198895	−0.481791
N	2.351897	−2.843416	−0.003734
C	3.110611	−4.045595	0.201987
O	4.022709	−4.054293	1.049000
N	2.800870	−5.130306	−0.561566
C	1.785605	−5.088912	−1.434763
N	1.547496	−6.191277	−2.175049
C	0.949383	−3.932878	−1.583617
C	1.267684	−2.840565	−0.842596
H	2.161885	−6.992310	−2.095240
H	0.811602	−6.208246	−2.868024
H	0.096356	−3.928539	−2.252169
H	0.680344	−1.928291	−0.858262
P	4.968900	1.446031	1.311923
O	5.097018	1.825862	−0.149045
O	5.583848	2.398038	2.321130
O	5.613630	−0.049231	1.453442
C	5.867098	−0.599516	2.777421
C	7.161026	−1.400947	2.765154
O	7.002573	−2.657303	2.045640
C	7.754383	−2.639208	0.809752
C	8.347315	−0.678638	2.096642
C	8.322257	−1.222118	0.660785
O	9.542995	−0.981012	2.889989
H	5.031034	−1.248632	3.058986
H	5.956865	0.214849	3.503835
H	7.410292	−1.648450	3.802803
H	8.551848	−3.376948	0.884014
H	8.231394	0.407310	2.116016
H	7.662049	−0.571355	0.088476
H	9.297326	−1.219994	0.179713
N	6.901990	−3.120047	−0.298609
C	6.897868	−4.504369	−0.514913
O	7.542529	−5.305155	0.165879
N	6.118870	−4.908665	−1.584572
C	5.305542	−4.118822	−2.407657
O	4.670080	−4.641862	−3.334843
C	5.291747	−2.707994	−2.057317
C	4.434370	−1.781578	−2.873687
C	6.068561	−2.288697	−1.024729
H	6.097607	−5.912275	−1.751942
H	4.699593	−1.854792	−3.934954
H	4.562527	−0.744088	−2.556326
H	3.373947	−2.047926	−2.789398
H	6.068038	−1.253297	−0.710303
P	11.010582	−1.411730	2.327758
O	11.439001	−0.615431	1.108600
O	11.943734	−1.367033	3.522769
O	10.795113	−2.937907	1.800305
C	10.722618	−4.065343	2.713286
C	11.592014	−5.193700	2.173462
O	11.066411	−5.711528	0.916045
C	11.842633	−5.222912	−0.201470
C	13.044536	−4.791180	1.870960
C	13.017138	−4.410436	0.379507
O	13.871480	−5.956016	2.128340
H	9.679697	−4.393773	2.775441
H	11.073919	−3.772117	3.708035
H	11.567170	−6.017915	2.895245
H	12.176961	−6.098164	−0.763802
H	13.372648	−3.954583	2.497393
H	12.850796	−3.338344	0.283401
H	13.956667	−4.657980	−0.119864
H	14.788280	−5.725146	1.891360
N	10.983560	−4.469088	−1.125755
C	10.786181	−3.092498	−1.249677
N	9.894487	−2.788340	−2.174309
C	9.467142	−4.013283	−2.685799
C	8.524855	−4.312229	−3.715506
O	7.845896	−3.537041	−4.412469
N	8.401107	−5.719058	−3.893795
C	9.105284	−6.686932	−3.206134
N	8.818132	−7.985691	−3.492024
N	9.993806	−6.394440	−2.261392
C	10.132272	−5.066822	−2.043083
H	11.302531	−2.375742	−0.628923
H	7.737525	−6.005787	−4.610507
H	8.365613	−8.217494	−4.368490
H	9.455271	−8.681080	−3.120390
Na	13.469316	0.012910	2.245880
Na	6.364485	3.797375	0.510828

(CTT)$^{Na+}$

E: −13357.64 kcal mol^{-1}

O	−0.218138	0.027986	−0.043200
C	−0.072130	0.285498	1.375407
C	1.371903	0.057973	1.806122
O	1.705082	−1.368359	1.796781
C	2.745895	−1.645677	0.821834
C	2.431094	0.747755	0.922352
C	2.893172	−0.368966	−0.012803
O	3.475112	1.246981	1.819147
H	−1.169709	0.014023	−0.246428
H	−0.722046	−0.381292	1.955699
H	−0.341518	1.326674	1.605005
H	1.469207	0.411751	2.838293
H	3.667259	−1.909426	1.343176
H	2.025098	1.601517	0.375134
H	2.228154	−0.383588	−0.877823
H	3.908600	−0.221583	−0.368003
N	2.389260	−2.840899	0.043891
C	3.099179	−4.067515	0.271248
O	3.993608	−4.102741	1.136937
N	2.769959	−5.145749	−0.493608
C	1.776706	−5.074333	−1.390174

N	1.516844	−6.172144	−2.129803
C	0.988640	−3.889081	−1.566399
C	1.327482	−2.804613	−0.823150
H	2.078441	−7.006219	−2.009649
H	0.780588	−6.175588	−2.822564
H	0.152412	−3.858187	−2.255253
H	0.771199	−1.873685	−0.856282
P	4.984650	1.638015	1.319766
O	5.053332	1.958745	−0.158446
O	5.485920	2.702676	2.277867
O	5.809371	0.239563	1.526361
C	5.948953	−0.287968	2.875782
C	7.100996	−1.277067	2.924738
O	6.769860	−2.496968	2.197861
C	7.626737	−2.662536	1.048383
C	8.420399	−0.758911	2.320905
C	8.440504	−1.367145	0.913835
O	9.494427	−1.233853	3.187536
H	5.018331	−0.787435	3.166903
H	6.149621	0.533204	3.573175
H	7.258115	−1.550082	3.974152
H	8.271530	−3.528730	1.207995
H	8.463578	0.332077	2.289778
H	7.976485	−0.656467	0.230881
H	9.452479	−1.559198	0.565806
N	6.806678	−3.015707	−0.131584
C	6.667905	−4.378440	−0.429045
O	7.207773	−5.280454	0.214476
N	5.892673	−4.636463	−1.545930
C	5.208604	−3.720186	−2.354740
O	4.534812	−4.122259	−3.313955
C	5.363056	−2.331670	−1.951123
C	4.705179	−1.270084	−2.787649
C	6.118572	−2.057995	−0.856835
H	5.752303	−5.622258	−1.757917
H	5.072831	−1.325556	−3.819413
H	4.917092	−0.271742	−2.396911
H	3.619007	−1.412063	−2.827200
H	6.220567	−1.045790	−0.486205
P	11.076119	−1.112121	2.769948
O	11.307416	−0.056254	1.709398
O	11.849258	−0.965582	4.067677
O	11.377373	−2.560382	2.071296
C	11.216199	−3.753255	2.892631
C	11.547721	−4.985628	2.073985
O	10.544385	−5.151480	1.026843
C	11.205914	−5.366502	−0.239819
C	12.928044	−4.968643	1.375234
C	12.587710	−4.715500	−0.100061
O	13.528237	−6.273152	1.588393
H	10.180696	−3.813762	3.245922
H	11.886639	−3.697595	3.758031
H	11.501804	−5.852957	2.745431
H	11.286151	−6.435901	−0.443712
H	13.578290	−4.189090	1.784220
H	12.541322	−3.638272	−0.276586
H	13.321201	−5.152201	−0.782105
H	14.405299	−6.267134	1.163104
N	10.348998	−4.823359	−1.301961
C	9.639750	−5.721540	−2.107288
O	9.712629	−6.949317	−2.003084
N	8.834647	−5.109099	−3.052028
C	8.657577	−3.736126	−3.280819
O	7.903944	−3.352484	−4.187413
C	9.396643	−2.872048	−2.372797
C	9.266458	−1.382778	−2.530855
C	10.187032	−3.455967	−1.436895
H	8.327791	−5.742013	−3.668300
H	9.596677	−1.073292	−3.529722
H	9.868601	−0.854343	−1.788053
H	8.221528	−1.068375	−2.430174
H	10.746431	−2.865442	−0.721970
Na	12.856177	1.083912	3.187785
Na	6.055308	4.110705	0.378118

(GAA)$^{Na+}$

E: −14214.44 kcal mol^{-1}

O	0.445690	0.566142	−1.250309
C	0.561105	1.046558	0.110734
C	1.815702	0.483111	0.765430
O	1.689496	−0.967879	0.939777
C	2.764810	−1.632528	0.226780
C	3.132481	0.701603	−0.016217
C	3.281642	−0.612646	−0.785007
O	4.179599	0.963110	0.983977
H	−0.429599	0.831872	−1.582328
H	−0.310095	0.735323	0.701738
H	0.623865	2.144516	0.131503
H	1.904391	0.936061	1.759192
H	3.548324	−1.914722	0.928923
H	3.088862	1.572083	−0.674262
H	2.620667	−0.569468	−1.654195
H	4.298029	−0.817608	−1.114678
N	2.299061	−2.888267	−0.331113
C	1.422985	−3.131943	−1.385658
N	1.283430	−4.422399	−1.637873
C	2.107095	−5.066610	−0.710709
C	2.382810	−6.452144	−0.494757
O	1.936294	−7.449319	−1.091409
N	3.322546	−6.623647	0.563472
C	3.901520	−5.615068	1.307022
N	4.773615	−5.959142	2.298788
N	3.618977	−4.333979	1.108357
C	2.741154	−4.124118	0.108133
H	0.920792	−2.324136	−1.901004
H	3.575346	−7.590247	0.757430
H	5.195053	−6.880942	2.256420
H	5.425317	−5.210161	2.545828
P	5.734485	0.447925	0.916683
O	6.271928	0.414252	−0.492421
O	6.453853	1.241285	1.993102
O	5.632615	−1.129254	1.358434
C	5.537665	−1.598876	2.725574
C	6.779719	−2.416370	3.073301
O	6.812126	−3.709662	2.394534
C	7.709436	−3.678954	1.254969
C	8.072778	−1.704549	2.661752
C	8.450763	−2.329622	1.306276
O	9.068139	−1.916513	3.707019
H	4.645972	−2.228170	2.800407
H	5.457936	−0.755207	3.418927
H	6.770726	−2.626851	4.147113
H	8.385318	−4.530410	1.359234
H	7.902012	−0.633887	2.575470
H	8.131547	−1.667197	0.501876
H	9.525540	−2.483181	1.222032
N	6.967188	−3.920982	0.010407
C	6.290866	−3.016493	−0.809930
N	5.565631	−3.607615	−1.742437
C	5.757931	−4.973348	−1.539178
C	5.264887	−6.117375	−2.202719
N	4.433617	−6.043102	−3.274026
N	5.670713	−7.337215	−1.767467
C	6.525353	−7.403925	−0.728768
N	7.048740	−6.388273	−0.015733

C	6.632727	−5.189851	−0.462065
H	6.379856	−1.948851	−0.679933
H	3.967722	−6.891809	−3.575173
H	3.959524	−5.167543	−3.460778
H	6.825325	−8.405820	−0.426159
P	10.433217	−1.014755	3.702913
O	10.190554	0.358888	3.095000
O	10.979521	−1.042535	5.114600
O	11.411681	−1.811452	2.670063
C	11.825790	−3.163037	3.040511
C	12.376264	−3.879934	1.824383
O	11.285214	−4.162694	0.894874
C	11.786494	−3.951747	−0.450249
C	13.458245	−3.104993	1.025693
C	12.766766	−2.785205	−0.310086
O	14.590247	−3.997335	0.867474
H	10.967808	−3.717411	3.435524
H	12.600080	−3.102851	3.813031
H	12.809355	−4.829863	2.165683
H	12.289592	−4.861764	−0.797914
H	13.772408	−2.196366	1.546678
H	12.225190	−1.840151	−0.212751
H	13.469488	−2.714815	−1.143296
H	15.305794	−3.492892	0.438936
N	10.665326	−3.736557	−1.345941
C	10.066155	−2.541008	−1.733802
N	9.084632	−2.711516	−2.601045
C	9.021330	−4.090554	−2.805047
C	8.209386	−4.906951	−3.625788
N	7.259095	−4.410399	−4.458858
N	8.416003	−6.248199	−3.597418
C	9.377221	−6.744600	−2.797154
N	10.210397	−6.070254	−1.982677
C	9.992899	−4.744729	−2.031116
H	10.398294	−1.586793	−1.351625
H	6.578387	−5.068227	−4.825734
H	6.922752	−3.469518	−4.287243
H	9.491163	−7.827311	−2.810847
Na	11.116558	1.330672	5.225421
Na	8.643707	1.772895	2.223025

(GAC)$^{Na+}$

E: −13813.66 kcal mol^{-1}

O	0.166787	0.453321	−0.669991
C	0.289546	0.849421	0.717197
C	1.606035	0.345491	1.296004
O	1.599813	−1.120423	1.370681
C	2.696132	−1.647349	0.580898
C	2.873326	0.724089	0.494070
C	3.100613	−0.519221	−0.367079
O	3.932722	1.011212	1.475002
H	−0.743066	0.651175	−0.952991
H	−0.534619	0.431208	1.309366
H	0.266882	1.945168	0.811828
H	1.692140	0.734170	2.316788
H	3.521527	−1.921224	1.237530
H	2.734865	1.628566	−0.102275
H	2.411546	−0.470464	−1.213894
H	4.118483	−0.615892	−0.738511
N	2.308922	−2.888940	−0.065362
C	1.407245	−3.113724	−1.102858
N	1.391900	−4.376833	−1.493533
C	2.332844	−5.020268	−0.685014
C	2.775508	−6.378686	−0.649319
O	2.409139	−7.347700	−1.339677
N	3.790095	−6.556754	0.336493
C	4.287745	−5.578519	1.174487
N	5.239301	−5.929471	2.086850
N	3.854158	−4.325174	1.140752
C	2.905191	−4.107229	0.209834
H	0.793347	−2.315779	−1.498848
H	4.155877	−7.503988	0.407500
H	5.765678	−6.780237	1.919229
H	5.801603	−5.140920	2.416550
P	5.515989	0.614914	1.326420
O	5.999334	0.699965	−0.100530
O	6.226736	1.396236	2.417779
O	5.537675	−0.987755	1.676172
C	5.592722	−1.547087	3.011362
C	6.923046	−2.273282	3.210692
O	7.012796	−3.503450	2.428310
C	7.831829	−3.305798	1.244664
C	8.113037	−1.418198	2.764555
C	8.403856	−1.880910	1.327359
O	9.223903	−1.641791	3.683873
H	4.763907	−2.254474	3.107902
H	5.497840	−0.757361	3.763920
H	7.012909	−2.559395	4.263170
H	8.614601	−4.066791	1.260563
H	7.849861	−0.364018	2.808857
H	7.909093	−1.209213	0.626163
H	9.470720	−1.884363	1.111391
N	7.055102	−3.571174	0.031928
C	6.210652	−2.722717	−0.683367
N	5.581586	−3.335234	−1.671283
C	6.020302	−4.657239	−1.612365
C	5.726218	−5.799157	−2.389589
N	4.871844	−5.768570	−3.439948
N	6.352575	−6.964337	−2.080995
C	7.226144	−6.980342	−1.054887
N	7.573897	−5.964954	−0.243120
C	6.941783	−4.822486	−0.566566
H	6.114051	−1.677135	−0.434810
H	4.574577	−6.644384	−3.853815
H	4.278376	−4.959295	−3.572115
H	7.705934	−7.937454	−0.857543
P	10.542391	−0.680480	3.557726
O	10.168569	0.699563	3.034445
O	11.253508	−0.730815	4.892731
O	11.426503	−1.391555	2.384785
C	11.949501	−2.731098	2.647081
C	12.322780	−3.419198	1.350789
O	11.136423	−3.722523	0.578012
C	11.614371	−4.067924	−0.748542
C	13.250889	−2.642535	0.388093
C	12.800498	−3.112328	−1.019795
O	14.614388	−2.977081	0.727492
H	11.195259	−3.326049	3.172752
H	12.840089	−2.647791	3.278670
H	12.832259	−4.359009	1.621612
H	11.924653	−5.114251	−0.768454
H	13.099734	−1.565314	0.501506
H	12.488730	−2.267301	−1.638059
H	13.611031	−3.633768	−1.535128
H	15.197615	−2.321730	0.304829
N	10.497458	−3.959645	−1.684824
C	10.109655	−5.097793	−2.466319
O	10.710365	−6.179228	−2.312395
N	9.094930	−4.938512	−3.359490
C	8.485589	−3.755098	−3.506594
N	7.508412	−3.660989	−4.435535
C	8.851347	−2.603253	−2.735049
C	9.863968	−2.756092	−1.844841
H	7.161992	−4.510226	−4.867288
H	6.907359	−2.847173	−4.455455

H	8.345006	−1.653299	−2.852950
H	10.210442	−1.948671	−1.211131
Na	11.307528	1.648940	5.063504
Na	8.417876	2.031407	2.426483

(GAG)$^{Na+}$

E: −14378.82 kcal mol^{-1}

O	0.282037	0.401100	−1.080865
C	0.423834	0.834208	0.293338
C	1.718202	0.292137	0.884962
O	1.647548	−1.168261	1.007247
C	2.738273	−1.764323	0.258296
C	2.998949	0.589394	0.069228
C	3.184944	−0.697114	−0.737519
O	4.063620	0.878308	1.042227
H	−0.603032	0.669689	−1.383468
H	−0.416901	0.470821	0.898717
H	0.450087	1.932098	0.354579
H	1.825581	0.711120	1.891799
H	3.549125	−2.025724	0.937169
H	2.894155	1.471922	−0.565557
H	2.500739	−0.660938	−1.589138
H	4.200631	−0.848799	−1.097285
N	2.321020	−3.026932	−0.321816
C	1.427517	−3.287300	−1.357678
N	1.351878	−4.575132	−1.647289
C	2.239417	−5.199883	−0.766817
C	2.600275	−6.573029	−0.605525
O	2.192465	−7.575409	−1.220947
N	3.579646	−6.723349	0.419509
C	4.120261	−5.707624	1.181855
N	5.032472	−6.033973	2.143078
N	3.762153	−4.439132	1.031674
C	2.844999	−4.248647	0.063549
H	0.865378	−2.494110	−1.832091
H	3.890118	−7.679846	0.576616
H	5.508998	−6.925250	2.053916
H	5.645964	−5.256451	2.401071
P	5.635804	0.431295	0.918263
O	6.133785	0.455146	−0.505298
O	6.352110	1.230015	1.993162
O	5.612705	−1.158246	1.323267
C	5.570630	−1.660826	2.681537
C	6.858148	−2.423169	2.985989
O	6.940720	−3.697745	2.277177
C	7.816219	−3.598983	1.123963
C	8.105145	−1.638291	2.565924
C	8.488575	−2.215029	1.191109
O	9.128648	−1.820241	3.589928
H	4.712263	−2.334230	2.760755
H	5.465178	−0.836687	3.394720
H	6.882263	−2.657421	4.054658
H	8.536710	−4.416072	1.199961
H	7.877228	−0.576452	2.506707
H	8.122123	−1.553819	0.405917
H	9.567411	−2.313187	1.083675
N	7.066926	−3.853760	−0.113029
C	6.327682	−2.969156	−0.900968
N	5.623523	−3.578600	−1.837425
C	5.897936	−4.934885	−1.672441
C	5.456120	−6.091212	−2.354672
N	4.599714	−6.045403	−3.399300
N	5.935480	−7.297477	−1.949405
C	6.797648	−7.339481	−0.914937
N	7.276505	−6.312299	−0.188698
C	6.796050	−5.127263	−0.610477
H	6.356423	−1.901672	−0.743839
H	4.198094	−6.907942	−3.746237
H	4.141220	−5.174643	−3.634088
H	7.149258	−8.330541	−0.633297
P	10.460675	−0.871437	3.565699
O	10.160510	0.495337	2.967851
O	11.032196	−0.884923	4.967814
O	11.448570	−1.631615	2.513352
C	11.894024	−2.980067	2.858937
C	12.429602	−3.676028	1.623713
O	11.326512	−3.952743	0.706552
C	11.798324	−3.703735	−0.642665
C	13.494859	−2.883813	0.820968
C	12.776355	−2.536087	−0.492359
O	14.623926	−3.771564	0.619781
H	11.054636	−3.553087	3.266733
H	12.684660	−2.915287	3.614476
H	12.873306	−4.627839	1.945723
H	12.298212	−4.602440	−1.022283
H	13.819681	−1.986548	1.355240
H	12.231937	−1.597116	−0.359527
H	13.461775	−2.440075	−1.337310
H	15.326826	−3.259144	0.179814
N	10.661430	−3.465586	−1.513639
C	10.093771	−2.249898	−1.905202
N	9.126967	−2.402584	−2.787638
C	9.035808	−3.778305	−2.996085
C	8.214044	−4.532273	−3.890391
O	7.374805	−4.137164	−4.716092
N	8.466942	−5.926556	−3.751090
C	9.404907	−6.501793	−2.920038
N	9.522529	−7.852799	−2.955135
N	10.196183	−5.784816	−2.125216
C	9.977403	−4.454780	−2.209222
H	10.438190	−1.305228	−1.511361
H	7.910269	−6.525558	−4.357004
H	8.796109	−8.419947	−3.373708
H	10.088376	−8.287687	−2.236371
Na	11.085082	1.488807	5.093815
Na	8.524572	1.847550	2.160292

(GAT)$^{Na+}$

E: −14071.85 kcal mol^{-1}

O	5.183503	−12.139204	−10.385674
C	6.315884	−13.009533	−10.622851
C	7.087715	−13.239476	−9.328982
O	7.717175	−11.991657	−8.880299
C	7.207072	−11.637996	−7.569415
C	6.246455	−13.737797	−8.132580
C	5.913263	−12.437218	−7.403928
O	7.098850	−14.669284	−7.374352
H	4.809276	−11.900860	−11.251776
H	6.995879	−12.561262	−11.358555
H	5.982251	−13.986057	−11.003870
H	7.884851	−13.960464	−9.540512
H	7.935170	−11.910445	−6.805568
H	5.358357	−14.291956	−8.443449
H	5.095769	−11.948486	−7.939430
H	5.622568	−12.577884	−6.366033
N	7.062523	−10.198043	−7.453828
C	6.209920	−9.333317	−8.135011
N	6.300477	−8.088203	−7.699671
C	7.249423	−8.128925	−6.674306
C	7.749057	−7.105382	−5.811426
O	7.470576	−5.892372	−5.779693
N	8.682632	−7.640291	−4.876039

C	9.086575	−8.958310	−4.796472
N	10.006323	−9.296322	−3.846515
N	8.633160	−9.892256	−5.622257
C	7.732657	−9.433881	−6.514320
H	5.577811	−9.685654	−8.939400
H	9.070842	−6.962868	−4.223012
H	10.121342	−8.668258	−3.058051
H	9.993030	−10.287775	−3.594803
P	7.150823	−14.841682	−5.751113
O	5.793751	−14.697388	−5.096013
O	7.932247	−16.117253	−5.487227
O	8.003752	−13.532233	−5.252943
C	9.429668	−13.508959	−4.989501
C	9.685553	−13.424090	−3.482997
O	9.355598	−12.110156	−2.935360
C	8.092366	−12.145602	−2.222898
C	8.829179	−14.426747	−2.702637
C	7.622773	−13.609686	−2.210170
O	9.615148	−15.009594	−1.620170
H	9.834730	−12.623839	−5.487425
H	9.905848	−14.411555	−5.385139
H	10.752544	−13.584733	−3.299843
H	8.275822	−11.758176	−1.218818
H	8.535439	−15.242189	−3.359110
H	6.781548	−13.767494	−2.884910
H	7.321789	−13.904190	−1.206525
N	7.125594	−11.229331	−2.830791
C	6.238072	−11.460949	−3.879721
N	5.556191	−10.384158	−4.225186
C	6.011756	−9.380896	−3.371945
C	5.674755	−8.014910	−3.233714
N	4.759775	−7.404124	−4.015740
N	6.295810	−7.298667	−2.257867
C	7.209699	−7.912528	−1.480120
N	7.620966	−9.193106	−1.532792
C	6.985672	−9.885938	−2.495709
H	6.135627	−12.438597	−4.324431
H	4.654066	−6.398192	−3.965224
H	4.379157	−7.888502	−4.818126
H	7.671545	−7.291765	−0.714488
P	8.928152	−16.238214	−0.779207
O	7.910014	−16.977961	−1.637389
O	10.051282	−17.064128	−0.192557
O	8.073968	−15.473164	0.381302
C	8.807118	−14.673874	1.359310
C	7.883115	−13.683177	2.035787
O	7.468387	−12.654035	1.101284
C	6.364224	−11.953250	1.725278
C	6.563481	−14.233898	2.607432
C	5.644056	−12.988409	2.630433
O	6.829713	−14.807903	3.902014
H	9.620740	−14.134433	0.863158
H	9.231213	−15.340062	2.117744
H	8.453654	−13.223924	2.859842
H	6.739783	−11.104939	2.298811
H	6.163140	−15.001347	1.937739
H	4.638070	−13.208840	2.267844
H	5.565336	−12.602881	3.650301
H	6.072423	−15.369515	4.145230
N	5.530507	−11.381668	0.661173
C	5.273873	−10.006199	0.670233
O	5.767697	−9.216572	1.479434
N	4.396409	−9.599483	−0.319617
C	3.716936	−10.394421	−1.256802
O	2.895400	−9.874200	−2.024280
C	4.069453	−11.808220	−1.221327
C	3.434304	−12.743820	−2.212239
C	4.935875	−12.223051	−0.264290
H	4.174069	−8.605673	−0.320689
H	2.350660	−12.802275	−2.052068
H	3.854255	−13.749345	−2.121586
H	3.591473	−12.386717	−3.235238
H	5.225925	−13.263506	−0.172737
Na	6.766010	−16.988310	−3.626077
Na	9.159696	−19.070789	−1.199349

(GCA)$^{Na+}$

E: −13804.14 kcal mol^{-1}

O	−0.291484	0.285290	−0.468576
C	−0.202782	0.745855	0.901198
C	1.155301	0.394694	1.496548
O	1.297958	−1.059033	1.635982
C	2.404697	−1.518019	0.821019
C	2.382831	0.850319	0.680561
C	2.694488	−0.383777	−0.163465
O	3.430589	1.174624	1.660135
H	−1.217138	0.383069	−0.752165
H	−0.981402	0.274299	1.514397
H	−0.330098	1.837176	0.952945
H	1.199390	0.832502	2.499729
H	3.270204	−1.720065	1.454934
H	2.191421	1.744622	0.083569
H	1.992795	−0.416112	−1.000105
H	3.711981	−0.401572	−0.548411
N	2.080668	−2.793066	0.206203
C	1.108866	−3.096393	−0.745489
N	1.162349	−4.359010	−1.133940
C	2.219502	−4.918913	−0.412817
C	2.789474	−6.229339	−0.438321
O	2.456305	−7.221149	−1.115085
N	3.896283	−6.319535	0.451005
C	4.405715	−5.293061	1.220128
N	5.501416	−5.545611	1.994523
N	3.857659	−4.084508	1.246242
C	2.796940	−3.956828	0.425393
H	0.399223	−2.351194	−1.080204
H	4.370523	−7.220239	0.455770
H	6.091989	−6.322507	1.713660
H	6.029317	−4.698737	2.227129
P	5.035981	1.075030	1.369587
O	5.376907	1.402116	−0.071795
O	5.718731	1.906438	2.438132
O	5.373708	−0.512695	1.556855
C	5.704136	−1.105447	2.843109
C	7.081067	−1.764729	2.794372
O	7.076709	−2.992927	2.009642
C	7.740229	−2.794706	0.726141
C	8.184485	−0.889489	2.171181
C	8.193794	−1.327867	0.698130
O	9.417626	−1.120663	2.931305
H	4.946325	−1.862477	3.066030
H	5.702069	−0.335712	3.620914
H	7.351669	−2.042639	3.818516
H	8.581081	−3.481288	0.688679
H	7.964624	0.175219	2.274257
H	7.491024	−0.688079	0.164888
H	9.167545	−1.214735	0.226993
N	6.851924	−3.225327	−0.367010
C	6.924688	−4.610157	−0.762917
O	7.754342	−5.351847	−0.212841
N	6.059284	−5.046524	−1.720101
C	5.162897	−4.219092	−2.267799
N	4.351012	−4.719008	−3.226932
C	5.050400	−2.847870	−1.873719
C	5.903247	−2.406411	−0.911364

H	4.371569	−5.712451	−3.423728
H	3.574720	−4.176972	−3.582215
H	4.306577	−2.190023	−2.306896
H	5.854292	−1.398970	−0.523221
P	10.885605	−1.510010	2.341151
O	11.291950	−0.659251	1.150667
O	11.826973	−1.492935	3.530190
O	10.693867	−3.014859	1.752255
C	10.675254	−4.190983	2.604046
C	11.639287	−5.228407	2.040208
O	11.183970	−5.733659	0.750634
C	11.945971	−5.144829	−0.328673
C	13.063860	−4.706475	1.790706
C	13.042752	−4.272135	0.313133
O	13.970560	−5.815129	2.026313
H	9.655759	−4.589876	2.610362
H	10.973461	−3.927031	3.623994
H	11.661138	−6.080801	2.728714
H	12.356383	−5.969693	−0.916187
H	13.312080	−3.872535	2.456305
H	12.800153	−3.211869	0.249118
H	14.009635	−4.432214	−0.169383
H	14.872690	−5.506750	1.823665
N	11.052341	−4.421869	−1.243751
C	10.704583	−3.075436	−1.276153
N	9.776369	−2.801334	−2.179545
C	9.484204	−4.027574	−2.775002
C	8.557080	−4.412629	−3.766751
N	7.730554	−3.529070	−4.385082
N	8.509809	−5.720077	−4.128259
C	9.330103	−6.593029	−3.511803
N	10.228417	−6.347868	−2.540020
C	10.265488	−5.045407	−2.206232
H	11.150371	−2.347955	−0.613960
H	6.953978	−3.896706	−4.922853
H	7.623000	−2.602659	−3.990866
H	9.250063	−7.628251	−3.840376
Na	13.314871	−0.034478	2.313367
Na	6.869154	3.137678	0.669634

(GCC)$^{Na+}$

E: −13406.97 kcal mol^{-1}

O	−0.175219	0.432218	−0.569196
C	−0.176684	0.845072	0.818782
C	1.137600	0.467417	1.492497
O	1.272194	−0.990346	1.587196
C	2.401252	−1.428863	0.793335
C	2.418521	0.946475	0.781901
C	2.767961	−0.245971	−0.106500
O	3.406247	1.191204	1.842150
H	−1.083072	0.529007	−0.905831
H	−0.995043	0.355588	1.361767
H	−0.305509	1.934466	0.898909
H	1.111845	0.864746	2.512855
H	3.228798	−1.698449	1.453358
H	2.278152	1.874951	0.223857
H	2.123198	−0.221230	−0.987520
H	3.808340	−0.258542	−0.425619
N	2.070393	−2.653607	0.084326
C	1.151509	−2.859066	−0.943763
N	1.184852	−4.096590	−1.407537
C	2.173197	−4.741219	−0.659493
C	2.703139	−6.066368	−0.739066
O	2.378714	−7.001481	−1.495377
N	3.757573	−6.249521	0.199933
C	4.245543	−5.294504	1.068711
N	5.283727	−5.631363	1.886874
N	3.730260	−4.073866	1.146638
C	2.727572	−3.856410	0.273924
H	0.492481	−2.066083	−1.272067
H	4.200485	−7.165699	0.174607
H	5.863269	−6.417818	1.612160
H	5.806490	−4.822324	2.231569
P	5.023998	1.191927	1.600444
O	5.391241	1.643810	0.201600
O	5.631003	1.965090	2.754828
O	5.436524	−0.390271	1.683681
C	5.619944	−1.074618	2.955541
C	6.928625	−1.859908	2.961530
O	6.851266	−3.066799	2.142146
C	7.651514	−2.932035	0.934770
C	8.145114	−1.084281	2.426575
C	8.212520	−1.502102	0.952419
O	9.292412	−1.511726	3.222869
H	4.782071	−1.763674	3.100997
H	5.641741	−0.342298	3.768487
H	7.111131	−2.185865	3.990732
H	8.436409	−3.688312	0.960747
H	8.033446	−0.003427	2.542214
H	7.594451	−0.813754	0.374935
H	9.225001	−1.449955	0.560440
N	6.831463	−3.277130	−0.241046
C	6.933983	−4.608851	−0.780458
O	7.743082	−5.405945	−0.277730
N	6.120677	−4.938137	−1.822865
C	5.237725	−4.060233	−2.314395
N	4.465063	−4.456839	−3.348549
C	5.092929	−2.742108	−1.774425
C	5.895666	−2.408336	−0.731000
H	4.507416	−5.419096	−3.662361
H	3.710481	−3.874735	−3.686921
H	4.356590	−2.047667	−2.160337
H	5.814459	−1.457348	−0.222037
P	10.853011	−1.274393	2.783697
O	11.004754	−0.171050	1.756976
O	11.638082	−1.121766	4.073591
O	11.238252	−2.674105	2.030963
C	11.198434	−3.908196	2.806416
C	11.802770	−5.044664	1.999724
O	10.951684	−5.352162	0.857407
C	11.695085	−5.214453	−0.380389
C	13.215442	−4.781759	1.439056
C	12.962180	−4.417471	−0.030014
O	13.962324	−6.020073	1.579314
H	10.156884	−4.144217	3.052824
H	11.765827	−3.776701	3.734929
H	11.833518	−5.926369	2.652532
H	11.930401	−6.203550	−0.774077
H	13.723769	−3.977287	1.980812
H	12.791329	−3.341502	−0.111557
H	13.801521	−4.685090	−0.676686
H	14.847389	−5.875438	1.197366
N	10.820275	−4.589903	−1.382250
C	10.282536	−5.381196	−2.446273
O	10.637167	−6.572627	−2.566223
N	9.399548	−4.793194	−3.300361
C	9.021100	−3.520070	−3.128033
N	8.112801	−3.009683	−3.986304
C	9.538196	−2.708598	−2.067152
C	10.417526	−3.294081	−1.214644
H	7.697096	−3.606449	−4.690911
H	7.751524	−2.072957	−3.869657
H	9.221421	−1.681864	−1.931981
H	10.821577	−2.781679	−0.349749
Na	12.520476	0.999795	3.246687

Na	6.807751	3.364994	1.144871

(GCG)$^{Na+}$

E: −13968.31 kcal mol^{-1}

O	−0.283820	0.275864	−0.463145
C	−0.195551	0.730780	0.908440
C	1.159001	0.369466	1.505653
O	1.292148	−1.085901	1.640159
C	2.404000	−1.546475	0.833638
C	2.391502	0.820362	0.694235
C	2.699435	−0.413778	−0.150884
O	3.438432	1.134248	1.678045
H	−1.209284	0.375413	−0.746777
H	−0.978458	0.261775	1.518047
H	−0.316366	1.822656	0.963907
H	1.203537	0.803661	2.510410
H	3.265955	−1.747304	1.472715
H	2.207328	1.717394	0.099069
H	1.998175	−0.443921	−0.988080
H	3.717269	−0.436008	−0.534736
N	2.083367	−2.822126	0.218449
C	1.106694	−3.127052	−0.727534
N	1.166524	−4.386952	−1.123801
C	2.233969	−4.942757	−0.414848
C	2.814328	−6.248199	−0.454056
O	2.482828	−7.238591	−1.133855
N	3.930343	−6.334574	0.423615
C	4.437738	−5.309394	1.196062
N	5.542337	−5.559623	1.958759
N	3.879630	−4.105921	1.236014
C	2.810954	−3.981255	0.424614
H	0.389401	−2.384932	−1.052719
H	4.411820	−7.231447	0.418823
H	6.136758	−6.328685	1.664354
H	6.067574	−4.710935	2.191696
P	5.043291	1.040639	1.381612
O	5.378897	1.387176	−0.056153
O	5.727213	1.860126	2.458866
O	5.384579	−0.548741	1.547895
C	5.719272	−1.157098	2.825918
C	7.107579	−1.791945	2.774857
O	7.126785	−3.019990	1.990030
C	7.782383	−2.810280	0.703940
C	8.193389	−0.894184	2.153029
C	8.223500	−1.338676	0.681190
O	9.425790	−1.085470	2.925109
H	4.973027	−1.930010	3.031908
H	5.700575	−0.400327	3.616045
H	7.383853	−2.066147	3.798453
H	8.628525	−3.490465	0.659180
H	7.945085	0.164836	2.248277
H	7.522461	−0.706261	0.136782
H	9.201720	−1.220083	0.220465
N	6.891467	−3.242197	−0.387474
C	6.968593	−4.624750	−0.789722
O	7.798583	−5.367312	−0.240830
N	6.104971	−5.060688	−1.749006
C	5.193480	−4.238312	−2.279973
N	4.369118	−4.742051	−3.226315
C	5.070349	−2.872190	−1.872727
C	5.929534	−2.428520	−0.917819
H	4.392273	−5.735095	−3.424271
H	3.582647	−4.205326	−3.566802
H	4.315113	−2.218864	−2.292950
H	5.874118	−1.424678	−0.521576
P	10.905307	−1.454257	2.351168
O	11.312657	−0.602006	1.162359
O	11.833316	−1.417196	3.550659
O	10.741394	−2.964446	1.768868
C	10.680668	−4.126395	2.637910
C	11.618044	−5.198067	2.095356
O	11.164012	−5.697680	0.803947
C	11.955299	−5.136442	−0.269186
C	13.061944	−4.723982	1.860948
C	13.073078	−4.297256	0.381310
O	13.928602	−5.860884	2.113618
H	9.649753	−4.495385	2.643785
H	10.979966	−3.856458	3.656150
H	11.603630	−6.045293	2.790552
H	12.345656	−5.977055	−0.847902
H	13.329917	−3.894926	2.524991
H	12.866810	−3.230002	0.310377
H	14.039765	−4.491736	−0.088937
H	14.842113	−5.585620	1.914585
N	11.096544	−4.387435	−1.196500
C	10.824859	−3.019391	−1.265879
N	9.925117	−2.726664	−2.186679
C	9.569439	−3.950827	−2.752124
C	8.613826	−4.264435	−3.764321
O	7.870750	−3.502172	−4.411652
N	8.566669	−5.665194	−3.997397
C	9.321750	−6.617965	−3.348728
N	9.090352	−7.922053	−3.671632
N	10.212051	−6.314707	−2.409881
C	10.285579	−4.991300	−2.145501
H	11.296324	−2.300949	−0.612370
H	7.877704	−5.965062	−4.683546
H	8.655733	−8.142570	−4.560330
H	9.774314	−8.591791	−3.337410
Na	13.314573	0.053556	2.340842
Na	6.863833	3.122746	0.706341

(GCT)$^{Na+}$

E: −13665.91 kcal mol^{-1}

O	−0.112976	0.518056	−0.485436
C	−0.079524	0.924607	0.904083
C	1.240097	0.518837	1.550818
O	1.348132	−0.941680	1.635206
C	2.454909	−1.397326	0.818877
C	2.516597	0.978813	0.818059
C	2.827909	−0.216299	−0.080591
O	3.528101	1.215374	1.859407
H	−1.026034	0.630553	−0.802763
H	−0.896542	0.448143	1.460580
H	−0.186278	2.015784	0.991072
H	1.241042	0.911159	2.573412
H	3.289183	−1.683420	1.462105
H	2.379387	1.910267	0.264307
H	2.167975	−0.173567	−0.949646
H	3.861713	−0.246005	−0.419005
N	2.091963	−2.613252	0.110744
C	1.156966	−2.802144	−0.905818
N	1.161535	−4.040335	−1.369245
C	2.146576	−4.703024	−0.632563
C	2.648448	−6.038853	−0.715181
O	2.297697	−6.967933	−1.467386
N	3.706246	−6.243316	0.215447
C	4.221662	−5.297664	1.078516
N	5.256177	−5.657039	1.892110
N	3.735380	−4.065262	1.156536
C	2.728771	−3.828303	0.293268
H	0.508656	−1.997515	−1.226911

H	4.127795	−7.169479	0.189972
H	5.818145	−6.454263	1.611663
H	5.800115	−4.859636	2.231660
P	5.142471	1.078645	1.639738
O	5.568145	1.466989	0.237398
O	5.798090	1.825938	2.784616
O	5.420959	−0.528546	1.757535
C	5.638032	−1.201968	3.028082
C	6.968231	−1.950167	3.012669
O	6.913937	−3.149378	2.180129
C	7.682476	−2.976479	0.957075
C	8.150726	−1.126406	2.470821
C	8.238870	−1.545409	0.996886
O	9.318321	−1.481848	3.270247
H	4.819513	−1.912175	3.178594
H	5.648513	−0.469731	3.841074
H	7.175154	−2.282597	4.035168
H	8.471128	−3.729316	0.948484
H	7.985117	−0.051754	2.579116
H	7.636469	−0.852375	0.409739
H	9.257988	−1.497337	0.622136
N	6.841950	−3.294183	−0.214615
C	6.903434	−4.628025	−0.754957
O	7.689578	−5.449359	−0.252873
N	6.086294	−4.933588	−1.800471
C	5.230407	−4.029996	−2.295283
N	4.449855	−4.403720	−3.329852
C	5.119474	−2.710719	−1.750307
C	5.929897	−2.399935	−0.706384
H	4.484137	−5.359292	−3.663739
H	3.730505	−3.791410	−3.690201
H	4.405989	−1.993922	−2.138271
H	5.873813	−1.446562	−0.199735
P	10.859537	−1.169922	2.805637
O	10.942543	−0.061747	1.776835
O	11.654987	−0.980714	4.084653
O	11.292063	−2.554065	2.048582
C	11.262522	−3.785801	2.827182
C	11.759909	−4.940440	1.978565
O	10.817562	−5.183750	0.892216
C	11.523106	−5.202304	−0.370360
C	13.147776	−4.740352	1.330019
C	12.822148	−4.419471	−0.135002
O	13.865991	−5.994359	1.474299
H	10.234561	−3.983060	3.151300
H	11.902720	−3.679127	3.710548
H	11.792751	−5.830845	2.619706
H	11.725550	−6.233371	−0.665146
H	13.707966	−3.933209	1.812550
H	12.666350	−3.343317	−0.240090
H	13.616458	−4.727450	−0.819154
H	14.742745	−5.881364	1.063593
N	10.634205	−4.663446	−1.407876
C	10.028143	−5.563966	−2.291667
O	10.231457	−6.782252	−2.282912
N	9.180941	−4.967709	−3.206903
C	8.833439	−3.615480	−3.308358
O	8.037809	−3.243223	−4.185244
C	9.464129	−2.752692	−2.320826
C	9.150481	−1.282716	−2.352338
C	10.312355	−3.318344	−1.424739
H	8.725062	−5.604807	−3.857213
H	9.405143	−0.857867	−3.330444
H	9.709968	−0.743309	−1.584401
H	8.078961	−1.107611	−2.198789
H	10.788771	−2.731955	−0.648170
Na	12.420677	1.184554	3.239063
Na	7.112648	3.075682	1.165640

(GGA)$^{Na+}$

E: −14371.61 kcal mol^{-1}

O	−0.395974	−0.171740	−0.371972
C	−0.264976	0.335124	0.977797
C	1.160689	0.144144	1.479824
O	1.464062	−1.282482	1.640129
C	2.568159	−1.644559	0.769883
C	2.272017	0.703060	0.565088
C	2.669468	−0.519900	−0.259834
O	3.329109	1.204880	1.456602
H	−1.341698	−0.156110	−0.601162
H	−0.946092	−0.196753	1.654691
H	−0.503202	1.408389	1.015124
H	1.229754	0.614717	2.466767
H	3.484698	−1.724943	1.357989
H	1.934563	1.541101	−0.048404
H	1.924544	−0.653729	−1.047598
H	3.655359	−0.442088	−0.711943
N	2.359373	−2.971456	0.217582
C	1.448601	−3.409663	−0.741348
N	1.609898	−4.686193	−1.044972
C	2.679839	−5.117005	−0.256154
C	3.329334	−6.385867	−0.153291
O	3.086480	−7.450909	−0.750067
N	4.408051	−6.330517	0.779297
C	4.787327	−5.226992	1.517088
N	5.832088	−5.353501	2.389428
N	4.162090	−4.062106	1.422862
C	3.148025	−4.062543	0.537656
H	0.697221	−2.747434	−1.150048
H	4.908157	−7.207892	0.907173
H	6.480780	−6.115748	2.219380
H	6.294251	−4.457422	2.570759
P	4.933427	1.201557	1.153867
O	5.248439	1.364742	−0.322119
O	5.549858	2.217521	2.096084
O	5.407435	−0.312262	1.535608
C	5.749384	−0.720580	2.885743
C	7.106178	−1.421648	2.897822
O	7.081365	−2.655396	2.124719
C	7.729613	−2.459295	0.838383
C	8.275959	−0.620456	2.296949
C	8.236443	−1.013232	0.814595
O	9.485108	−1.019818	3.028599
H	4.981875	−1.420179	3.232417
H	5.782126	0.149260	3.549415
H	7.328951	−1.695950	3.934068
H	8.544961	−3.177589	0.786801
H	8.166306	0.456510	2.443219
H	7.528628	−0.354982	0.310657
H	9.204497	−0.922881	0.323734
N	6.841404	−2.828246	−0.256401
C	5.947367	−2.090012	−1.030029
N	5.346593	−2.829646	−1.947688
C	5.857706	−4.119332	−1.775718
C	5.625352	−5.338821	−2.484590
O	4.874674	−5.562980	−3.448565
N	6.423004	−6.399250	−1.947645
C	7.314777	−6.290951	−0.899641
N	7.971675	−7.409383	−0.475068
N	7.519970	−5.150681	−0.259124
C	6.786935	−4.125499	−0.729722
H	5.793766	−1.032333	−0.870440
H	6.303016	−7.298335	−2.409567
H	8.062348	−8.191107	−1.114879
H	8.803740	−7.204523	0.077337

P	10.906035	−1.551782	2.428276
O	11.391109	−0.728345	1.249113
O	11.839931	−1.646369	3.618879
O	10.582002	−3.025736	1.809343
C	10.371607	−4.209726	2.625839
C	11.165703	−5.378968	2.049124
O	10.634167	−5.823311	0.762750
C	11.508749	−5.419035	−0.320494
C	12.649986	−5.070479	1.793574
C	12.683623	−4.651999	0.314150
O	13.388126	−6.296048	2.036395
H	9.302311	−4.443590	2.621136
H	10.700769	−4.022573	3.653273
H	11.066012	−6.221433	2.742831
H	11.829967	−6.324695	−0.840473
H	13.017417	−4.274863	2.450705
H	12.538911	−3.573818	0.237826
H	13.632251	−4.907371	−0.163659
H	14.324479	−6.122849	1.828245
N	10.751659	−4.650220	−1.312643
C	10.391437	−3.306390	−1.285379
N	9.558672	−2.972540	−2.257639
C	9.343042	−4.154384	−2.965033
C	8.525095	−4.472994	−4.072223
N	7.742833	−3.558882	−4.699386
N	8.538249	−5.751281	−4.531680
C	9.313029	−6.659327	−3.906811
N	10.102063	−6.479790	−2.831583
C	10.079415	−5.206655	−2.396325
H	10.767669	−2.633548	−0.529056
H	7.040008	−3.897302	−5.346924
H	7.561595	−2.673902	−4.241341
H	9.288003	−7.666308	−4.320554
Na	13.448380	−0.265928	2.418262
Na	6.632519	3.268812	0.192622

(GGC)$^{Na+}$

E: −13972.57 kcal mol^{-1}

O	−0.300325	−0.033831	−0.477145
C	−0.239748	0.423513	0.895089
C	1.152363	0.196957	1.472111
O	1.431888	−1.237629	1.602188
C	2.567797	−1.589894	0.771000
C	2.321648	0.769196	0.644187
C	2.744074	−0.427385	−0.206283
O	3.337498	1.199246	1.616205
H	−1.232368	−0.005245	−0.755506
H	−0.964336	−0.122491	1.512964
H	−0.467409	1.497894	0.956864
H	1.168158	0.632782	2.477029
H	3.452251	−1.718838	1.399592
H	2.036492	1.639462	0.049065
H	2.043798	−0.518741	−1.039480
H	3.754165	−0.349882	−0.600645
N	2.352804	−2.887307	0.154198
C	1.472299	−3.257241	−0.860605
N	1.613898	−4.522449	−1.215732
C	2.638517	−5.017194	−0.405079
C	3.258999	−6.303109	−0.343933
O	3.019316	−7.330245	−1.005140
N	4.303258	−6.318133	0.628172
C	4.677382	−5.260939	1.433357
N	5.690132	−5.452282	2.329846
N	4.077025	−4.080076	1.377809
C	3.098304	−4.013194	0.456384
H	0.755432	−2.556865	−1.267665
H	4.784676	−7.209699	0.726088
H	6.339130	−6.209063	2.138672
H	6.145585	−4.575563	2.597414
P	4.942766	1.301631	1.328595
O	5.255160	1.591246	−0.126652
O	5.499809	2.268204	2.355678
O	5.492628	−0.213877	1.600427
C	5.705815	−0.733854	2.939827
C	7.016722	−1.511426	3.006241
O	6.963689	−2.756106	2.244833
C	7.689680	−2.628005	0.997406
C	8.247284	−0.770464	2.454993
C	8.292444	−1.214298	0.987411
O	9.385376	−1.202686	3.265715
H	4.875900	−1.402395	3.191830
H	5.744085	0.091211	3.658828
H	7.179658	−1.790417	4.052090
H	8.447610	−3.414728	0.980526
H	8.161984	0.314873	2.551689
H	7.672030	−0.527484	0.412106
H	9.297744	−1.190049	0.571704
N	6.822547	−2.910242	−0.147631
C	5.971133	−2.057757	−0.853146
N	5.344784	−2.670095	−1.840886
C	5.789954	−3.990727	−1.793407
C	5.501027	−5.105527	−2.639713
O	4.752025	−5.168679	−3.630161
N	6.222165	−6.260171	−2.220507
C	7.110272	−6.317250	−1.166675
N	7.685809	−7.520620	−0.882718
N	7.380099	−5.270823	−0.398156
C	6.708993	−4.155223	−0.748397
H	5.858246	−1.019991	−0.581805
H	6.056983	−7.097043	−2.776138
H	7.738524	−8.221690	−1.613481
H	8.500088	−7.467064	−0.278456
P	10.932246	−1.307060	2.744714
O	11.267220	−0.270685	1.691531
O	11.789338	−1.311673	3.996001
O	10.984082	−2.759744	1.993760
C	10.794070	−3.970407	2.784498
C	11.367115	−5.166754	2.040931
O	10.568694	−5.455967	0.856072
C	11.387076	−5.374490	−0.337204
C	12.823046	−4.998880	1.559440
C	12.673599	−4.641006	0.074517
O	13.484751	−6.277446	1.752695
H	9.722684	−4.118493	2.960521
H	11.304685	−3.863841	3.747822
H	11.303862	−6.031289	2.713835
H	11.589110	−6.379057	−0.709518
H	13.348230	−4.218539	2.120084
H	12.563507	−3.558973	−0.027846
H	13.532664	−4.960945	−0.520506
H	14.394345	−6.192371	1.412668
N	10.602666	−4.715737	−1.392404
C	10.113992	−5.483684	−2.496581
O	10.455030	−6.677705	−2.613570
N	9.282173	−4.869788	−3.384983
C	8.944580	−3.581871	−3.234536
N	8.118778	−3.036066	−4.153103
C	9.431329	−2.788618	−2.145659
C	10.238401	−3.405956	−1.245055
H	7.708001	−3.626406	−4.866489
H	7.741633	−2.106777	−4.024214
H	9.146900	−1.750157	−2.027879
H	10.613510	−2.908114	−0.358733
Na	13.092686	0.528116	3.121519
Na	6.553955	3.515689	0.549829

(GGG)$^{Na+}$

E: –14536.08 kcal mol^{-1}

O	−0.348128	−0.268701	−0.363520
C	−0.202200	0.264336	0.974651
C	1.223128	0.064417	1.474192
O	1.507648	−1.362432	1.665597
C	2.602699	−1.757596	0.799762
C	2.338822	0.586038	0.543206
C	2.711845	−0.660267	−0.257884
O	3.408679	1.081202	1.423549
H	−1.297274	−0.268185	−0.578205
H	−0.886855	−0.243406	1.666181
H	−0.424985	1.341518	0.990368
H	1.303209	0.555897	2.450081
H	3.521997	−1.833057	1.382506
H	2.014369	1.418661	−0.084551
H	1.957852	−0.803036	−1.035595
H	3.695078	−0.614118	−0.719749
N	2.376580	−3.096446	0.285289
C	1.409161	−3.559651	−0.603463
N	1.551918	−4.844427	−0.880079
C	2.667572	−5.254466	−0.145327
C	3.321045	−6.521147	−0.045645
O	3.050830	−7.598970	−0.607457
N	4.444751	−6.445924	0.829560
C	4.874298	−5.321445	1.505372
N	5.970606	−5.428383	2.313998
N	4.250526	−4.155284	1.410114
C	3.184014	−4.178469	0.588283
H	0.634783	−2.906361	−0.983063
H	4.961070	−7.316795	0.939548
H	6.593995	−6.209603	2.135739
H	6.459761	−4.534724	2.423273
P	5.005148	1.079906	1.080803
O	5.283629	1.305178	−0.394274
O	5.651285	2.050775	2.050214
O	5.477381	−0.452878	1.383715
C	5.812510	−0.929772	2.713554
C	7.209383	−1.543499	2.711886
O	7.268526	−2.770524	1.927689
C	7.867343	−2.524989	0.629293
C	8.305348	−0.641665	2.116125
C	8.355593	−1.066731	0.638711
O	9.531464	−0.836270	2.898241
H	5.081245	−1.694760	2.991936
H	5.780223	−0.105146	3.432695
H	7.457995	−1.815908	3.742599
H	8.682816	−3.238560	0.524999
H	8.059213	0.416962	2.220346
H	7.676943	−0.419934	0.083596
H	9.348282	−0.968160	0.203243
N	6.928376	−2.850031	−0.448977
C	6.040357	−2.047011	−1.167496
N	5.324405	−2.736562	−2.037512
C	5.741512	−4.059640	−1.890542
C	5.337151	−5.253183	−2.563690
O	4.504813	−5.404770	−3.475248
N	6.045061	−6.385330	−2.057966
C	7.002476	−6.358857	−1.064066
N	7.515526	−7.553008	−0.639238
N	7.389431	−5.240276	−0.472831
C	6.736877	−4.142941	−0.907698
H	5.967781	−0.981788	−1.004375
H	5.778694	−7.278027	−2.468630
H	7.488769	−8.338537	−1.281906
H	8.388146	−7.461646	−0.125546
P	10.963424	−1.428765	2.392374
O	11.506981	−0.691862	1.180176
O	11.854941	−1.448551	3.618554
O	10.629123	−2.928744	1.861196
C	10.400038	−4.057347	2.745488
C	11.182262	−5.256390	2.221577
O	10.687537	−5.680886	0.917265
C	11.611653	−5.298291	−0.128907
C	12.686773	−5.001110	2.028601
C	12.802370	−4.594977	0.549692
O	13.370532	−6.250073	2.311132
H	9.327492	−4.275316	2.745850
H	10.726888	−3.817540	3.762791
H	11.026080	−6.090913	2.915053
H	11.906599	−6.211975	−0.650797
H	13.054427	−4.214562	2.696712
H	12.726119	−3.511211	0.464865
H	13.753445	−4.908220	0.112860
H	14.319769	−6.112215	2.138109
N	10.923915	−4.479566	−1.135153
C	10.779435	−3.092230	−1.208861
N	9.988354	−2.716736	−2.197126
C	9.578315	−3.901214	−2.807179
C	8.692552	−4.122639	−3.904458
O	8.081729	−3.296974	−4.607032
N	8.536135	−5.515989	−4.152833
C	9.162243	−6.535092	−3.465675
N	8.835857	−7.812522	−3.812795
N	9.992232	−6.315014	−2.453331
C	10.151212	−5.004526	−2.159707
H	11.257929	−2.422304	−0.509591
H	7.910399	−5.749552	−4.921136
H	8.441856	−7.991018	−4.729593
H	9.442699	−8.539291	−3.449397
Na	13.525796	−0.184948	2.363467
Na	6.671800	3.190665	0.147465

(GGT)$^{Na+}$

E: –14231.40 kcal mol^{-1}

O	−0.336393	−0.012903	−0.261092
C	−0.228659	0.424497	1.114614
C	1.179738	0.180109	1.642821
O	1.453839	−1.258223	1.738642
C	2.567617	−1.598411	0.872264
C	2.325349	0.760873	0.787289
C	2.713802	−0.423895	−0.095177
O	3.372601	1.178748	1.733779
H	−1.276341	0.028546	−0.509769
H	−0.936989	−0.125463	1.747777
H	−0.446387	1.499456	1.198723
H	1.230458	0.598395	2.653999
H	3.470159	−1.729006	1.471940
H	2.025572	1.641971	0.215953
H	1.982301	−0.500925	−0.902936
H	3.709138	−0.348089	−0.525242
N	2.340739	−2.890287	0.250246
C	1.405530	−3.264834	−0.712173
N	1.535635	−4.528279	−1.077542
C	2.608593	−5.016615	−0.327989
C	3.234290	−6.300783	−0.299143
O	2.965597	−7.327943	−0.949577
N	4.325496	−6.313990	0.618965
C	4.744049	−5.254228	1.398908
N	5.803031	−5.445862	2.239404
N	4.143476	−4.072146	1.370530

C	3.112488	−4.010008	0.506147
H	0.661080	−2.568533	−1.074692
H	4.815664	−7.203757	0.688841
H	6.423930	−6.221036	2.030772
H	6.290745	−4.574052	2.464595
P	4.979186	1.182935	1.447855
O	5.311843	1.484795	−0.002116
O	5.599882	2.089839	2.492303
O	5.432502	−0.368945	1.679691
C	5.734144	−0.923526	2.985990
C	7.111659	−1.578015	2.970768
O	7.145596	−2.782020	2.144511
C	7.782817	−2.521588	0.871368
C	8.241202	−0.687664	2.420649
C	8.319408	−1.080651	0.936810
O	9.434771	−0.964894	3.219652
H	4.974957	−1.676098	3.220470
H	5.720639	−0.134460	3.744655
H	7.342666	−1.895023	3.992466
H	8.571385	−3.268069	0.756263
H	8.019778	0.375436	2.541679
H	7.683474	−0.396367	0.376550
H	9.328452	−0.997868	0.537512
N	6.859320	−2.754043	−0.246968
C	5.990434	−1.862173	−0.883508
N	5.288271	−2.434282	−1.842228
C	5.693120	−3.767816	−1.847167
C	5.286926	−4.857903	−2.676338
O	4.466710	−4.876493	−3.609260
N	5.970517	−6.053448	−2.308894
C	6.903086	−6.169489	−1.298843
N	7.385485	−7.414042	−1.028716
N	7.297993	−5.139982	−0.560419
C	6.667123	−3.984159	−0.862487
H	5.923994	−0.823917	−0.598228
H	5.699563	−6.882784	−2.833684
H	7.306292	−8.144233	−1.727143
H	8.217564	−7.449851	−0.449932
P	10.960055	−1.120172	2.654546
O	11.289277	−0.128981	1.556357
O	11.851072	−1.095326	3.881784
O	10.954191	−2.596999	1.953841
C	10.745182	−3.765475	2.801260
C	11.335207	−4.988921	2.123519
O	10.543890	−5.322846	0.943099
C	11.404841	−5.395008	−0.215457
C	12.795431	−4.823627	1.645836
C	12.656440	−4.580804	0.135664
O	13.484025	−6.066801	1.941239
H	9.669853	−3.903910	2.959839
H	11.236874	−3.613206	3.768183
H	11.276420	−5.827754	2.828516
H	11.653596	−6.435858	−0.428830
H	13.293955	−3.990404	2.151112
H	12.505673	−3.514004	−0.044153
H	13.532726	−4.913301	−0.425977
H	14.402454	−5.977012	1.627287
N	10.638639	−4.926625	−1.379536
C	10.185077	−5.881023	−2.294641
O	10.451748	−7.085257	−2.227690
N	9.386846	−5.361820	−3.298677
C	8.995680	−4.028451	−3.487771
O	8.266573	−3.729992	−4.445961
C	9.498663	−3.101542	−2.485660
C	9.144156	−1.646993	−2.616450
C	10.272153	−3.596323	−1.486005
H	9.063721	−6.034966	−3.991252
H	9.529798	−1.241840	−3.559658
H	9.566036	−1.064109	−1.794554
H	8.057862	−1.507879	−2.629620
H	10.647307	−2.961517	−0.691547
Na	13.173687	0.673743	2.896964
Na	6.707029	3.315405	0.695166

(GTA)$^{Na+}$

E: −14064.21 kcal mol^{-1}

O	−0.470034	0.551820	0.003959
C	−0.215436	0.935828	1.376368
C	1.193920	0.527886	1.787391
O	1.311155	−0.934997	1.835907
C	2.314237	−1.369532	0.885964
C	2.322063	1.002784	0.846674
C	2.511343	−0.198056	−0.076323
O	3.489073	1.284599	1.696002
H	−1.419311	0.681041	−0.166543
H	−0.929820	0.445865	2.050236
H	−0.309012	2.024937	1.498527
H	1.371862	0.911505	2.797824
H	3.239979	−1.606584	1.412545
H	2.069933	1.920925	0.311752
H	1.720862	−0.183994	−0.830055
H	3.480276	−0.211041	−0.570936
N	1.913677	−2.616458	0.257697
C	0.865677	−2.871377	−0.625185
N	0.867909	−4.119380	−1.061187
C	1.969439	−4.719765	−0.446426
C	2.499316	−6.044407	−0.543285
O	2.089844	−7.011795	−1.210600
N	3.667877	−6.184200	0.261802
C	4.226571	−5.204413	1.059954
N	5.329870	−5.516562	1.791787
N	3.717515	−3.981165	1.145467
C	2.621162	−3.799457	0.384132
H	0.145141	−2.105626	−0.879265
H	4.092262	−7.109399	0.244447
H	5.856955	−6.350699	1.560784
H	5.877987	−4.711643	2.102813
P	5.048007	1.135654	1.223950
O	5.247180	1.451678	−0.244498
O	5.869126	1.940039	2.213599
O	5.356222	−0.465324	1.383439
C	5.625494	−1.049489	2.690907
C	6.982356	−1.746691	2.706899
O	6.953422	−3.007005	1.967129
C	7.719575	−2.900669	0.738285
C	8.128600	−0.932279	2.077931
C	8.158459	−1.436455	0.626915
O	9.330340	−1.188444	2.877122
H	4.839908	−1.781460	2.900451
H	5.618631	−0.269019	3.457747
H	7.219372	−1.993256	3.746769
H	8.575038	−3.567639	0.810695
H	7.941745	0.142287	2.131096
H	7.445216	−0.839148	0.058271
H	9.133719	−1.336913	0.156464
N	6.921077	−3.430450	−0.383280
C	7.144721	−4.772264	−0.727455
O	7.975002	−5.493615	−0.174554
N	6.333861	−5.231185	−1.751128
C	5.337518	−4.529837	−2.446471
O	4.702574	−5.095841	−3.349054
C	5.137486	−3.162639	−1.996825
C	4.076491	−2.339668	−2.673384
C	5.914790	−2.693709	−0.986224
H	6.506190	−6.190762	−2.045736

H	4.232761	−2.330973	−3.758279
H	4.094588	−1.308291	−2.316485
H	3.078535	−2.758328	−2.498565
H	5.772864	−1.700437	−0.578474
P	10.827517	−1.521369	2.325097
O	11.222818	−0.666716	1.134471
O	11.740698	−1.458269	3.533991
O	10.706916	−3.040928	1.749910
C	10.734695	−4.207250	2.615656
C	11.749940	−5.206507	2.073969
O	11.338931	−5.733950	0.778437
C	12.095044	−5.117966	−0.288557
C	13.156977	−4.629764	1.849780
C	13.150138	−4.205886	0.368972
O	14.102227	−5.699047	2.113123
H	9.735540	−4.655028	2.618524
H	11.009716	−3.917943	3.635100
H	11.792538	−6.054804	2.766525
H	12.542917	−5.927402	−0.870122
H	13.357082	−3.782174	2.514324
H	12.875414	−3.154390	0.293098
H	14.130875	−4.337291	−0.093647
H	14.995733	−5.354351	1.931742
N	11.189646	−4.425102	−1.217362
C	10.817703	−3.086136	−1.272485
N	9.895357	−2.841996	−2.190640
C	9.632554	−4.081514	−2.771103
C	8.726907	−4.495416	−3.773292
N	7.876063	−3.644984	−4.389914
N	8.692084	−5.814834	−4.099761
C	9.520537	−6.664005	−3.458895
N	10.411495	−6.386398	−2.489910
C	10.424701	−5.077080	−2.178453
H	11.238382	−2.340630	−0.613803
H	7.277456	−3.982251	−5.132979
H	7.926393	−2.650895	−4.213335
H	9.453067	−7.706881	−3.765081
Na	13.200547	0.052187	2.330721
Na	6.885366	3.130660	0.360351

(GTC)$^{Na+}$

E: −13664.70 kcal mol^{-1}

O	6.301066	−11.586921	−10.764939
C	7.532924	−12.343115	−10.854737
C	7.903268	−12.918125	−9.493184
O	8.377502	−11.877272	−8.575902
C	7.433575	−11.665581	−7.501733
C	6.756551	−13.632612	−8.755679
C	6.220611	−12.560589	−7.802775
O	7.343549	−14.787197	−8.079630
H	6.184959	−11.108711	−11.604508
H	8.353703	−11.702946	−11.201482
H	7.417948	−13.180010	−11.558932
H	8.736212	−13.612530	−9.643564
H	7.917535	−11.917502	−6.553075
H	5.983122	−13.994095	−9.436914
H	5.443400	−11.997220	−8.320244
H	5.787715	−12.987151	−6.901071
N	7.097841	−10.243946	−7.404389
C	6.291783	−9.500099	−8.268958
N	6.213959	−8.229559	−7.921422
C	7.010748	−8.115270	−6.783126
C	7.310510	−6.978502	−5.972528
O	6.909364	−5.804766	−6.081264
N	8.197772	−7.328989	−4.918255
C	8.729473	−8.581508	−4.692447
N	9.621180	−8.704684	−3.672199
N	8.425210	−9.638623	−5.439097
C	7.581011	−9.354084	−6.456039
H	5.837634	−9.955613	−9.137630
H	8.479036	−6.556769	−4.317218
H	9.654654	−7.991822	−2.952953
H	9.814266	−9.649587	−3.359763
P	6.529078	−15.652727	−6.950918
O	5.031018	−15.452742	−7.042679
O	7.040949	−17.077694	−7.051206
O	7.012576	−14.958359	−5.549791
C	8.421350	−15.069406	−5.196109
C	8.629299	−14.598204	−3.770392
O	8.432349	−13.152242	−3.691661
C	7.456071	−12.842247	−2.673996
C	7.678860	−15.232761	−2.729971
C	6.651121	−14.129790	−2.459999
O	8.498185	−15.569788	−1.570285
H	9.018117	−14.456260	−5.880802
H	8.739218	−16.114708	−5.281464
H	9.666043	−14.819767	−3.492055
H	7.955829	−12.521947	−1.757014
H	7.209811	−16.146953	−3.099315
H	5.845859	−14.225330	−3.191196
H	6.219266	−14.198495	−1.464223
N	6.675346	−11.676625	−3.117363
C	6.913904	−10.448856	−2.486540
O	7.739301	−10.284326	−1.589458
N	6.120043	−9.415027	−2.956873
C	5.147329	−9.459916	−3.965906
O	4.516162	−8.435490	−4.260287
C	4.983605	−10.766000	−4.588786
C	3.945443	−10.915968	−5.665326
C	5.759096	−11.789591	−4.148283
H	6.240822	−8.524113	−2.478688
H	2.962578	−10.604981	−5.291749
H	3.873748	−11.952306	−6.002241
H	4.174179	−10.277448	−6.526309
H	5.700404	−12.776904	−4.591292
P	7.859789	−16.091266	−0.151952
O	6.425399	−16.551902	−0.304305
O	8.845547	−17.091904	0.422795
O	7.827903	−14.725654	0.749078
C	9.103326	−14.099668	1.077434
C	8.880091	−12.924232	2.011677
O	8.174554	−11.863034	1.304403
C	6.991286	−11.473518	2.042214
C	8.064858	−13.231791	3.286652
C	6.679491	−12.647121	2.983884
O	8.723874	−12.548909	4.386190
H	9.581275	−13.749666	0.155373
H	9.756706	−14.833467	1.563124
H	9.869272	−12.548827	2.304729
H	7.182701	−10.548478	2.587768
H	8.020699	−14.306645	3.489628
H	6.069522	−13.402803	2.482382
H	6.155861	−12.320766	3.885883
H	8.207080	−12.725093	5.193764
N	5.927328	−11.153910	1.081819
C	5.503758	−9.797920	0.910694
O	6.034829	−8.896608	1.590524
N	4.515469	−9.547291	0.006032
C	3.985802	−10.537077	−0.725679
N	3.012744	−10.218655	−1.604605
C	4.442126	−11.891498	−0.621497
C	5.415541	−12.144102	0.291248
H	2.687505	−9.261862	−1.672559
H	2.548716	−10.929888	−2.152646
H	4.035742	−12.678457	−1.245145

H	5.848297	−13.128000	0.429724
Na	4.730016	−17.845323	−7.327087
Na	7.041707	−18.721424	0.614288

(GTG)$^{Na+}$

E: −14228.23 kcal mol^{-1}

O	−0.432163	0.582097	−0.050548
C	−0.181492	0.956908	1.325165
C	1.223597	0.538028	1.739552
O	1.330908	−0.925676	1.779314
C	2.338487	−1.360504	0.833830
C	2.358907	1.012033	0.806468
C	2.547753	−0.185524	−0.121352
O	3.522063	1.287603	1.662939
H	−1.380588	0.714375	−0.223596
H	−0.901710	0.467457	1.993190
H	−0.269163	2.045872	1.453166
H	1.399808	0.914577	2.752992
H	3.259244	−1.603743	1.365912
H	2.112902	1.933272	0.274088
H	1.761793	−0.162993	−0.879652
H	3.519757	−0.201306	−0.610136
N	1.937266	−2.603385	0.198528
C	0.902867	−2.849971	−0.702844
N	0.899895	−4.099132	−1.135198
C	1.983227	−4.709518	−0.498049
C	2.500623	−6.039909	−0.581011
O	2.093506	−7.005105	−1.252951
N	3.652082	−6.189846	0.246846
C	4.206087	−5.213472	1.052573
N	5.291717	−5.534326	1.806058
N	3.708732	−3.984585	1.124539
C	2.629232	−3.793615	0.341770
H	0.195049	−2.077571	−0.972185
H	4.065619	−7.120101	0.241470
H	5.812365	−6.377132	1.592989
H	5.841494	−4.734864	2.127441
P	5.084857	1.120140	1.210747
O	5.308529	1.427924	−0.255690
O	5.901308	1.919138	2.208719
O	5.373026	−0.483739	1.380189
C	5.599851	−1.067516	2.695951
C	6.950902	−1.772972	2.755016
O	6.937768	−3.036597	2.018948
C	7.741957	−2.938543	0.814023
C	8.120969	−0.969823	2.158337
C	8.187614	−1.476315	0.709983
O	9.294233	−1.253631	2.987633
H	4.803838	−1.794319	2.882920
H	5.574862	−0.286104	3.461385
H	7.153988	−2.017749	3.802422
H	8.592145	−3.610938	0.912906
H	7.945485	0.107118	2.206538
H	7.491677	−0.879073	0.120465
H	9.174969	−1.379345	0.266241
N	6.971362	−3.468126	−0.328299
C	7.186443	−4.814236	−0.656567
O	7.993257	−5.542970	−0.079374
N	6.392902	−5.271840	−1.695524
C	5.411635	−4.568152	−2.412183
O	4.780527	−5.140435	−3.312899
C	5.221444	−3.194664	−1.979307
C	4.179361	−2.365627	−2.677463
C	5.985164	−2.726173	−0.958346
H	6.534133	−6.246715	−1.953013
H	4.364135	−2.348408	−3.757823
H	4.188917	−1.337048	−2.311894
H	3.176916	−2.784667	−2.531010
H	5.843748	−1.728627	−0.561328
P	10.829608	−1.435811	2.464687
O	11.172527	−0.521761	1.303700
O	11.704667	−1.326903	3.698416
O	10.853715	−2.947079	1.854536
C	10.845982	−4.116629	2.716700
C	11.769129	−5.174887	2.126020
O	11.257122	−5.677919	0.857165
C	12.017083	−5.142448	−0.251061
C	13.195693	−4.684776	1.826249
C	13.135023	−4.266439	0.346821
O	14.086605	−5.809419	2.045265
H	9.822802	−4.503827	2.774638
H	11.190693	−3.847387	3.720714
H	11.797672	−6.022214	2.820544
H	12.407857	−5.992401	−0.815684
H	13.482551	−3.848917	2.473695
H	12.890389	−3.206686	0.281474
H	14.086896	−4.432655	−0.162929
H	14.987353	−5.523329	1.807307
N	11.127241	−4.434088	−1.179250
C	10.774496	−3.083719	−1.220117
N	9.872667	−2.821531	−2.147269
C	9.601182	−4.050038	−2.749132
C	8.694110	−4.390162	−3.797142
O	7.918646	−3.657996	−4.438523
N	8.733782	−5.787771	−4.065834
C	9.542740	−6.710784	−3.432884
N	9.412165	−8.011925	−3.807878
N	10.383686	−6.379515	−2.458082
C	10.369749	−5.061968	−2.156118
H	11.198558	−2.357835	−0.542891
H	8.109081	−6.102504	−4.805394
H	8.995905	−8.236287	−4.703892
H	10.120480	−8.653311	−3.469759
Na	13.050622	0.341464	2.560066
Na	6.968570	3.086983	0.374515

(GTT)$^{Na+}$

E: −13924.03 kcal mol^{-1}

O	6.169736	−12.188283	−11.210454
C	7.340415	−13.037399	−11.142295
C	7.726084	−13.286542	−9.689217
O	8.175008	−12.037202	−9.062601
C	7.316242	−11.728313	−7.937442
C	6.593848	−13.824140	−8.785269
C	6.045806	−12.552298	−8.145113
O	7.213555	−14.756086	−7.828612
H	6.051537	−11.924069	−12.139475
H	8.189096	−12.561360	−11.650098
H	7.145327	−14.008126	−11.621114
H	8.566629	−13.989055	−9.680771
H	7.817941	−12.006404	−7.010357
H	5.839443	−14.383465	−9.342059
H	5.383287	−12.063139	−8.862728
H	5.501828	−12.738466	−7.221484
N	7.108160	−10.296005	−7.827837
C	6.361899	−9.434890	−8.631370
N	6.325699	−8.204056	−8.151846
C	7.077523	−8.247380	−6.974651
C	7.379570	−7.236971	−6.009308
O	7.042718	−6.039319	−5.987326
N	8.173089	−7.768055	−4.949752
C	8.627939	−9.068618	−4.848424

N	9.406545	−9.399848	−3.785487
N	8.341647	−9.995019	−5.756192
C	7.578005	−9.538834	−6.767890
H	5.897584	−9.777520	−9.546150
H	8.432512	−7.095827	−4.230587
H	9.459257	−8.777809	−2.987844
H	9.477446	−10.399488	−3.590704
P	6.766400	−14.977274	−6.273213
O	5.276080	−14.825182	−6.046741
O	7.389025	−16.290679	−5.837889
O	7.459271	−13.721541	−5.479857
C	8.885044	−13.716215	−5.177167
C	9.112858	−13.642966	−3.671905
O	8.832813	−12.303954	−3.148969
C	7.635615	−12.302897	−2.336125
C	8.228114	−14.600773	−2.854517
C	7.049380	−13.718851	−2.416987
O	9.041123	−15.099869	−1.753083
H	9.324734	−12.836852	−5.656508
H	9.355644	−14.624006	−5.565899
H	10.171358	−13.843143	−3.478618
H	7.910237	−12.029672	−1.316296
H	7.899016	−15.455354	−3.449147
H	6.264710	−13.794599	−3.169691
H	6.632881	−14.040825	−1.466102
N	6.731375	−11.219821	−2.789190
C	6.776494	−10.010768	−2.080186
O	7.490046	−9.815391	−1.095539
N	5.937040	−9.031620	−2.583694
C	5.078228	−9.110075	−3.692492
O	4.387368	−8.130791	−4.006932
C	5.115103	−10.384341	−4.388897
C	4.232168	−10.567442	−5.591339
C	5.947891	−11.351620	−3.925415
H	5.951456	−8.146788	−2.079352
H	3.193996	−10.319070	−5.342548
H	4.263438	−11.600279	−5.943268
H	4.531715	−9.903625	−6.410892
H	6.058869	−12.294277	−4.446786
P	8.395332	−15.890074	−0.467628
O	7.016371	−16.439618	−0.766756
O	9.445910	−16.881505	−0.003657
O	8.204598	−14.685076	0.623052
C	9.395980	−13.947853	1.025693
C	9.020939	−12.881386	2.035960
O	8.177985	−11.880817	1.389904
C	7.007847	−11.641037	2.204432
C	8.251134	−13.382710	3.279966
C	6.809031	−12.919883	3.028319
O	8.845460	−12.733899	4.434842
H	9.845186	−13.478174	0.143569
H	10.122744	−14.635799	1.473167
H	9.949377	−12.397607	2.366428
H	7.168034	−10.771602	2.844597
H	8.319978	−14.469609	3.387503
H	6.279432	−13.683696	2.453437
H	6.260004	−12.732509	3.954247
H	8.373322	−13.050457	5.226803
N	5.895478	−11.280765	1.315457
C	5.458494	−9.951801	1.300042
O	5.918068	−9.064207	2.024105
N	4.441850	−9.707677	0.392568
C	3.841449	−10.609931	−0.497104
O	2.944158	−10.222218	−1.260878
C	4.377255	−11.961821	−0.441504
C	3.793192	−13.002036	−1.356531
C	5.372875	−12.219273	0.444921
H	4.089047	−8.752320	0.380663
H	2.716143	−13.105269	−1.180170
H	4.264267	−13.974846	−1.198449
H	3.915672	−12.714192	−2.407409
H	5.831345	−13.198616	0.514316
Na	5.250065	−17.024034	−5.003475
Na	7.761242	−18.666353	−0.233704

(TAA)$^{Na+}$

E: −13898.33 kcal mol^{-1}

O	−0.153871	−0.182564	−0.421922
C	−0.050799	0.222786	0.965330
C	1.363907	−0.004923	1.483529
O	1.654802	−1.437034	1.605715
C	2.733852	−1.808271	0.712494
C	2.495195	0.575879	0.610094
C	2.923069	−0.622660	−0.238336
O	3.521193	1.073376	1.535433
H	−1.096857	−0.175492	−0.662341
H	−0.747615	−0.354816	1.585563
H	−0.288957	1.290901	1.071465
H	1.419232	0.428223	2.487919
H	3.635880	−2.011024	1.288591
H	2.165316	1.420885	0.001790
H	2.241798	−0.691621	−1.088931
H	3.938302	−0.552872	−0.618586
N	2.401959	−3.078421	0.048808
C	3.166692	−4.208304	0.359949
O	4.093088	−4.213440	1.172750
N	2.800207	−5.338935	−0.352338
C	1.759006	−5.477379	−1.279299
O	1.574214	−6.571414	−1.839902
C	0.969522	−4.274435	−1.489333
C	−0.203598	−4.328705	−2.429923
C	1.324132	−3.148868	−0.817097
H	3.368526	−6.165981	−0.178992
H	−0.930283	−5.083437	−2.104760
H	0.118566	−4.607851	−3.440843
H	−0.705601	−3.358369	−2.479425
H	0.758741	−2.225857	−0.905146
P	5.123941	1.117612	1.227708
O	5.431729	1.404475	−0.230632
O	5.728912	2.064936	2.246090
O	5.608765	−0.417537	1.489388
C	5.936454	−0.913104	2.815047
C	7.329082	−1.535429	2.801015
O	7.370400	−2.749594	1.998104
C	7.953142	−2.483708	0.704820
C	8.425026	−0.625349	2.213701
C	8.473148	−1.035468	0.733131
O	9.653938	−0.829332	2.987832
H	5.197182	−1.673558	3.086287
H	5.908054	−0.095939	3.542828
H	7.581306	−1.824109	3.826677
H	8.750564	−3.214308	0.566501
H	8.177314	0.432210	2.327972
H	7.812167	−0.364280	0.187863
H	9.467502	−0.947087	0.300893
N	6.983723	−2.746449	−0.369788
C	6.234062	−1.863968	−1.139292
N	5.457136	−2.476201	−2.020091
C	5.695604	−3.835059	−1.822462
C	5.176302	−4.996658	−2.433109
N	4.284013	−4.952185	−3.452869
N	5.605754	−6.203703	−1.984072
C	6.526574	−6.238757	−0.998320
N	7.095735	−5.205744	−0.354208
C	6.639231	−4.022591	−0.799797

H	6.280763	−0.793493	−1.003711
H	3.830029	−5.808165	−3.748312
H	3.856221	−4.071327	−3.707257
H	6.844248	−7.232768	−0.687707
P	11.113963	−1.287375	2.423218
O	11.563166	−0.485608	1.214543
O	12.040365	−1.266991	3.623829
O	10.875271	−2.801297	1.873327
C	10.718537	−3.928291	2.778082
C	11.479081	−5.120390	2.212630
O	10.882928	−5.574369	0.960132
C	11.719857	−5.214183	−0.165448
C	12.956356	−4.842131	1.888734
C	12.938671	−4.461623	0.398725
O	13.685370	−6.072839	2.134196
H	9.651725	−4.162054	2.856699
H	11.113402	−3.675976	3.767831
H	11.397277	−5.946108	2.928740
H	11.998947	−6.139768	−0.675453
H	13.362903	−4.036078	2.509398
H	12.819578	−3.382461	0.304746
H	13.859949	−4.753539	−0.110728
H	14.613894	−5.921899	1.879032
N	10.933316	−4.453176	−1.141040
C	10.729732	−3.080673	−1.242544
N	9.852939	−2.755204	−2.179526
C	9.444862	−3.971789	−2.725812
C	8.510190	−4.308542	−3.730552
N	7.793657	−3.380910	−4.415830
N	8.340210	−5.620310	−4.034368
C	9.052431	−6.542424	−3.358724
N	9.945626	−6.343877	−2.372459
C	10.105156	−5.037592	−2.094042
H	11.236560	−2.376644	−0.598759
H	7.007570	−3.704135	−4.968787
H	7.740693	−2.437420	−4.051573
H	8.874930	−7.578080	−3.644704
Na	13.587240	0.124839	2.374990
Na	6.786922	3.284827	0.429641

(TAC)$^{Na+}$

E: −13499.88 kcal mol^{-1}

O	−0.104874	−0.125579	−0.482992
C	−0.066497	0.255069	0.914571
C	1.328893	0.040638	1.488058
O	1.640487	−1.389072	1.592403
C	2.766128	−1.720123	0.742017
C	2.484798	0.658109	0.675225
C	2.968322	−0.513374	−0.179256
O	3.468010	1.135982	1.653880
H	−1.036841	−0.127402	−0.763056
H	−0.778933	−0.345025	1.494344
H	−0.326683	1.317080	1.030044
H	1.334116	0.452964	2.502700
H	3.645839	−1.915522	1.356153
H	2.168061	1.513335	0.074036
H	2.324106	−0.580343	−1.058199
H	3.995587	−0.410335	−0.516082
N	2.492429	−2.984102	0.040694
C	3.259432	−4.106892	0.371441
O	4.131026	−4.115150	1.242442
N	2.964711	−5.224931	−0.392331
C	1.970080	−5.366681	−1.370015
O	1.829824	−6.454803	−1.952986
C	1.169003	−4.174184	−1.596901
C	0.043761	−4.232594	−2.594042
C	1.463907	−3.057106	−0.883458
H	3.525455	−6.050539	−0.190606
H	−0.684083	−5.005004	−2.316490
H	0.418951	−4.488903	−3.592498
H	−0.472659	−3.270278	−2.651656
H	0.882895	−2.144988	−0.981309
P	5.058325	1.355151	1.344748
O	5.335876	1.637990	−0.118786
O	5.550235	2.383321	2.345565
O	5.714286	−0.108964	1.643588
C	5.909548	−0.593248	2.998591
C	7.193395	−1.410990	3.067516
O	7.089823	−2.649179	2.304307
C	7.821439	−2.546870	1.062667
C	8.442142	−0.698189	2.516737
C	8.501750	−1.166222	1.057440
O	9.568769	−1.126989	3.343864
H	5.056922	−1.221893	3.277769
H	5.980569	0.252046	3.691515
H	7.350844	−1.693507	4.113756
H	8.538100	−3.372511	1.037820
H	8.368694	0.389723	2.591480
H	7.943748	−0.450576	0.455533
H	9.518151	−1.205331	0.672464
N	6.938786	−2.767211	−0.088782
C	6.232612	−1.842919	−0.851744
N	5.552138	−2.400264	−1.840591
C	5.817784	−3.763797	−1.732291
C	5.391580	−4.882797	−2.481413
N	4.571351	−4.776272	−3.551952
N	5.833357	−6.111284	−2.105722
C	6.661985	−6.205609	−1.044829
N	7.125527	−5.217657	−0.260354
C	6.672836	−4.012700	−0.647566
H	6.244528	−0.786832	−0.629211
H	4.200510	−5.614024	−3.983010
H	4.158579	−3.884118	−3.789566
H	6.991707	−7.212349	−0.794155
P	11.130728	−1.119200	2.854131
O	11.410514	−0.059216	1.808054
O	11.964393	−1.065162	4.120708
O	11.291785	−2.566911	2.107911
C	11.076408	−3.770582	2.902162
C	11.431465	−4.996570	2.081046
O	10.485389	−5.138112	0.980271
C	11.204657	−5.280811	−0.271038
C	12.845814	−4.989151	1.458453
C	12.591216	−4.670626	−0.020001
O	13.399974	−6.318386	1.650146
H	10.024555	−3.819045	3.206012
H	11.706226	−3.737577	3.798454
H	11.337118	−5.871010	2.738115
H	11.265422	−6.334116	−0.548068
H	13.492982	−4.245101	1.933855
H	12.573462	−3.586327	−0.158324
H	13.353337	−5.094939	−0.678489
H	14.289989	−6.326438	1.252617
N	10.419082	−4.636543	−1.328566
C	9.805951	−5.433773	−2.348051
O	10.003415	−6.664831	−2.367295
N	9.028354	−4.800422	−3.270008
C	8.840387	−3.475579	−3.219126
N	8.061997	−2.915431	−4.170904
C	9.439599	−2.656502	−2.206887
C	10.210160	−3.286198	−1.283468
H	7.559118	−3.516776	−4.812800
H	7.774682	−1.948409	−4.099681
H	9.274235	−1.586803	−2.166202
H	10.677900	−2.763913	−0.457300

Na	13.137066	0.880716	3.232480
Na	6.490385	3.677292	0.521770

(TAG)$^{Na+}$

E: −14063.13 kcal mol^{-1}

O	−0.105876	−0.249840	−0.457952
C	−0.007440	0.164578	0.926917
C	1.407981	−0.050322	1.448112
O	1.707497	−1.479647	1.581668
C	2.790563	−1.851313	0.694001
C	2.537174	0.531087	0.572041
C	2.976876	−0.671604	−0.264830
O	3.556199	1.045178	1.495761
H	−1.048566	−0.250702	−0.699680
H	−0.701163	−0.413852	1.549853
H	−0.252981	1.231684	1.026374
H	1.459488	0.390820	2.449251
H	3.691835	−2.046328	1.273885
H	2.202169	1.367832	−0.044884
H	2.299771	−0.751520	−1.117842
H	3.993684	−0.599663	−0.641276
N	2.466859	−3.127490	0.037912
C	3.236114	−4.251617	0.358677
O	4.158981	−4.247436	1.175479
N	2.878397	−5.388087	−0.348830
C	1.842835	−5.536549	−1.280160
O	1.668479	−6.633654	−1.838499
C	1.045256	−4.340097	−1.497079
C	−0.125296	−4.406642	−2.440033
C	1.391101	−3.209033	−0.829703
H	3.453389	−6.209820	−0.172382
H	−0.844471	−5.168997	−2.116052
H	0.201509	−4.682292	−3.450454
H	−0.637629	−3.441667	−2.490048
H	0.820235	−2.289962	−0.923659
P	5.158659	1.104910	1.187538
O	5.465070	1.389365	−0.271187
O	5.752699	2.061542	2.203711
O	5.659226	−0.424316	1.455293
C	5.947138	−0.919191	2.790136
C	7.339561	−1.539744	2.819716
O	7.408977	−2.761827	2.030117
C	8.020672	−2.504654	0.747918
C	8.451528	−0.634380	2.257164
C	8.545350	−1.057939	0.782298
O	9.651242	−0.848582	3.071474
H	5.200685	−1.679555	3.041360
H	5.897483	−0.101462	3.516252
H	7.562229	−1.819315	3.854578
H	8.817185	−3.241446	0.629619
H	8.203149	0.425017	2.354477
H	7.906927	−0.388188	0.209177
H	9.553385	−0.976875	0.382184
N	7.070860	−2.766685	−0.345497
C	6.338219	−1.882251	−1.130396
N	5.565711	−2.491946	−2.015378
C	5.789473	−3.851334	−1.808399
C	5.259615	−5.011738	−2.412473
N	4.371034	−4.969096	−3.435065
N	5.671503	−6.220817	−1.950262
C	6.575368	−6.258482	−0.948670
N	7.147321	−5.227161	−0.305397
C	6.717472	−4.041898	−0.771266
H	6.392625	−0.811574	−1.001596
H	3.910799	−5.823235	−3.725452
H	3.966917	−4.085134	−3.715335
H	6.877112	−7.253800	−0.626813
P	11.164323	−1.142628	2.535377
O	11.568116	−0.266100	1.365344
O	12.048962	−1.072365	3.766354
O	11.082267	−2.661017	1.946915
C	10.841993	−3.780600	2.845132
C	11.462220	−5.036156	2.249466
O	10.773729	−5.421871	1.022338
C	11.622444	−5.198347	−0.131011
C	12.947635	−4.903835	1.871496
C	12.908509	−4.528864	0.382238
O	13.562209	−6.199473	2.097301
H	9.760908	−3.914023	2.961695
H	11.292950	−3.578630	3.823090
H	11.329179	−5.851108	2.970706
H	11.815745	−6.168554	−0.596347
H	13.453382	−4.139945	2.471887
H	12.854280	−3.443992	0.290193
H	13.790039	−4.881842	−0.158157
H	14.491715	−6.139026	1.810301
N	10.891420	−4.421389	−1.131303
C	10.775871	−3.036293	−1.265959
N	9.943425	−2.688299	−2.229005
C	9.471371	−3.891037	−2.755478
C	8.533513	−4.146077	−3.801966
O	7.918346	−3.342543	−4.525249
N	8.328932	−5.544532	−3.966592
C	8.960462	−6.545749	−3.257900
N	8.614073	−7.825782	−3.545748
N	9.842379	−6.295160	−2.294299
C	10.047405	−4.976955	−2.081744
H	11.309739	−2.353890	−0.621547
H	7.651735	−5.798233	−4.682692
H	8.135253	−8.043402	−4.410728
H	9.182463	−8.559957	−3.140662
Na	13.508751	0.480855	2.637500
Na	6.804714	3.283447	0.385042

(TAT)$^{Na+}$

E: −13761.89 kcal mol^{-1}

O	5.359504	−12.234997	−10.137944
C	6.518981	−13.070636	−10.378010
C	7.270873	−13.319376	−9.075862
O	7.907673	−12.087624	−8.601442
C	7.360703	−11.701802	−7.315045
C	6.407995	−13.828713	−7.900171
C	6.092682	−12.545588	−7.130980
O	7.208805	−14.825094	−7.174496
H	5.003189	−11.967981	−11.003215
H	7.200589	−12.584946	−11.087498
H	6.213582	−14.042143	−10.792566
H	8.065424	−14.043321	−9.285593
H	8.091644	−11.900623	−6.534491
H	5.504535	−14.340109	−8.238295
H	5.236092	−12.071567	−7.614662
H	5.838395	−12.721857	−6.088785
N	7.142875	−10.249244	−7.288364
C	7.925141	−9.480568	−6.418616
O	8.840440	−9.933004	−5.727299
N	7.570775	−8.141411	−6.392205
C	6.612085	−7.486007	−7.177084
O	6.443921	−6.260926	−7.050941
C	5.891405	−8.347043	−8.101296
C	4.860236	−7.735002	−9.010319
C	6.194425	−9.670767	−8.112915
H	8.118210	−7.559142	−5.761560

H	5.310573	−6.966005	−9.650105
H	4.068889	−7.245928	−8.428953
H	4.404322	−8.497511	−9.648097
H	5.718296	−10.362436	−8.801619
P	7.366595	−15.001981	−5.562697
O	6.059601	−14.801197	−4.813385
O	8.085879	−16.316421	−5.336721
O	8.314817	−13.729908	−5.144441
C	9.722872	−13.833397	−4.805408
C	9.921185	−13.728769	−3.290681
O	9.692175	−12.372486	−2.804616
C	8.441169	−12.280405	−2.086337
C	8.949055	−14.632089	−2.521228
C	7.827424	−13.690749	−2.058324
O	9.643982	−15.292890	−1.420147
H	10.233958	−13.007066	−5.306846
H	10.126463	−14.787248	−5.158875
H	10.960780	−13.984236	−3.059102
H	8.655876	−11.901968	−1.084474
H	8.586404	−15.419747	−3.177876
H	6.985454	−13.772138	−2.745524
H	7.484163	−13.936515	−1.055503
N	7.558755	−11.284698	−2.700770
C	6.682359	−11.451846	−3.767201
N	6.036429	−10.345854	−4.087135
C	6.507821	−9.387325	−3.192118
C	6.188216	−8.024970	−2.995299
N	5.304272	−7.362136	−3.770329
N	6.810925	−7.361969	−1.982942
C	7.702612	−8.026090	−1.221077
N	8.086666	−9.312507	−1.318305
C	7.456715	−9.950034	−2.322415
H	6.566896	−12.410964	−4.244997
H	5.051660	−6.407853	−3.546104
H	4.752039	−7.863922	−4.452323
H	8.165734	−7.446309	−0.424598
P	8.789267	−16.403039	−0.565995
O	7.659480	−16.981582	−1.408982
O	9.780970	−17.390860	0.008025
O	8.076171	−15.515082	0.602088
C	8.923464	−14.731729	1.497672
C	8.100606	−13.653404	2.171965
O	7.632793	−12.701815	1.182919
C	6.518702	−12.004427	1.782538
C	6.844231	−14.126584	2.947719
C	5.766330	−13.067589	2.609973
O	7.203847	−14.185853	4.346916
H	9.736077	−14.272831	0.925517
H	9.350556	−15.390715	2.261448
H	8.759329	−13.138581	2.889152
H	6.877644	−11.181336	2.404096
H	6.530200	−15.118195	2.610395
H	4.959391	−13.511446	2.021692
H	5.333698	−12.635584	3.515446
H	6.512249	−14.690448	4.811595
N	5.733379	−11.392844	0.705333
C	5.500650	−10.013869	0.734560
O	5.966311	−9.252913	1.586893
N	4.681018	−9.568544	−0.287828
C	4.037746	−10.328761	−1.278753
O	3.264037	−9.775622	−2.072653
C	4.368842	−11.747656	−1.263316
C	3.773841	−12.649333	−2.309418
C	5.179448	−12.199455	−0.274858
H	4.476010	−8.571171	−0.276059
H	2.685494	−12.718335	−2.190057
H	4.194645	−13.656338	−2.241201
H	3.966755	−12.255288	−3.312005
H	5.451397	−13.245272	−0.197809
Na	6.457313	−16.744575	−3.358278
Na	8.585888	−19.231163	−1.015323

(TCA)$^{Na+}$

E: −13496.07 kcal mol^{-1}

O	−0.235286	−0.036970	−0.297329
C	−0.132735	0.319730	1.103423
C	1.282972	0.075383	1.611674
O	1.577618	−1.360608	1.667906
C	2.663356	−1.683498	0.764702
C	2.410216	0.698183	0.762517
C	2.838476	−0.459583	−0.139223
O	3.444147	1.149224	1.701953
H	−1.178319	−0.022491	−0.537334
H	−0.828392	−0.280586	1.702953
H	−0.372534	1.383059	1.247274
H	1.338182	0.463031	2.634517
H	3.566982	−1.895238	1.334976
H	2.076987	1.569750	0.194831
H	2.153860	−0.497899	−0.988852
H	3.852536	−0.357817	−0.516089
N	2.353750	−2.930376	0.050519
C	3.143647	−4.056306	0.313175
O	4.060174	−4.078773	1.136675
N	2.814832	−5.156639	−0.460789
C	1.798115	−5.267171	−1.417210
O	1.654866	−6.330588	−2.045295
C	0.979324	−4.075385	−1.575510
C	−0.171274	−4.105396	−2.544689
C	1.291492	−2.980311	−0.836029
H	3.409324	−5.972384	−0.326856
H	−0.880167	−4.901640	−2.286186
H	0.181935	−4.308181	−3.563412
H	−0.702447	−3.149381	−2.544544
H	0.704761	−2.067822	−0.884580
P	5.041904	1.220712	1.361892
O	5.312068	1.569228	−0.089359
O	5.659168	2.129558	2.407485
O	5.544436	−0.320085	1.551725
C	5.877802	−0.864327	2.858056
C	7.243075	−1.542303	2.799588
O	7.207666	−2.761460	2.010211
C	7.822724	−2.556239	0.714956
C	8.351094	−0.675568	2.171426
C	8.347283	−1.110646	0.696774
O	9.590433	−0.904543	2.923461
H	5.113092	−1.599226	3.129730
H	5.899503	−0.062614	3.603007
H	7.518775	−1.827407	3.820854
H	8.630498	−3.278491	0.629342
H	8.140970	0.391163	2.274377
H	7.683359	−0.430121	0.166697
H	9.328098	−1.042925	0.231126
N	6.876494	−2.922973	−0.363653
C	6.755215	−4.327646	−0.652245
O	7.425624	−5.135711	0.012025
N	5.914575	−4.711081	−1.655032
C	5.193910	−3.807145	−2.325478
N	4.394191	−4.251115	−3.322010
C	5.244155	−2.412090	−2.015141
C	6.094666	−2.023253	−1.025700
H	4.346313	−5.241741	−3.525137
H	3.812703	−3.616473	−3.851487
H	4.637946	−1.687938	−2.545925
H	6.171072	−0.988259	−0.724630
P	11.023628	−1.417427	2.343757

O	11.487625	−0.633137	1.128329
O	11.967654	−1.429255	3.531013
O	10.724455	−2.919971	1.797465
C	10.619543	−4.069340	2.678821
C	11.491248	−5.192621	2.130917
O	10.983262	−5.684210	0.855215
C	11.785154	−5.187097	−0.241094
C	12.951112	−4.794080	1.858090
C	12.949274	−4.385289	0.373704
O	13.764993	−5.970666	2.104754
H	9.571048	−4.383172	2.703471
H	10.949192	−3.805862	3.689266
H	11.450479	−6.030393	2.836310
H	12.128268	−6.056875	−0.806760
H	13.275516	−3.972394	2.505777
H	12.783876	−3.311440	0.293517
H	13.897098	−4.623715	−0.114323
H	14.686281	−5.744785	1.880973
N	10.944520	−4.423244	−1.172888
C	10.708234	−3.054531	−1.249037
N	9.804137	−2.734694	−2.161859
C	9.411231	−3.951647	−2.717740
C	8.469631	−4.290717	−3.713590
N	7.731255	−3.362568	−4.376055
N	8.320511	−5.600351	−4.037185
C	9.061455	−6.517712	−3.385862
N	9.969047	−6.316477	−2.412565
C	10.107672	−5.011401	−2.116018
H	11.211326	−2.345518	−0.607841
H	6.935187	−3.684110	−4.914985
H	7.687505	−2.420793	−4.006716
H	8.901033	−7.552515	−3.685013
Na	13.543014	−0.101178	2.261915
Na	6.660591	3.449292	0.644571

(TCC)$^{Na+}$

E: −13099.55 kcal mol^{-1}

O	−0.093613	0.107018	−0.476376
C	−0.178657	0.347036	0.950281
C	1.164018	0.068305	1.614478
O	1.457782	−1.369260	1.620757
C	2.642437	−1.645820	0.836835
C	2.379499	0.737405	0.945098
C	2.933915	−0.365281	0.043593
O	3.299230	1.110181	2.020887
H	−0.996849	0.143946	−0.836757
H	−0.936550	−0.303627	1.404247
H	−0.450408	1.393920	1.147279
H	1.092217	0.393104	2.657875
H	3.467361	−1.914885	1.498607
H	2.112440	1.638857	0.389725
H	2.386787	−0.354175	−0.901032
H	3.991661	−0.236059	−0.167592
N	2.404921	−2.838994	0.006839
C	3.144128	−3.996704	0.275587
O	3.973364	−4.091636	1.182461
N	2.878628	−5.040829	−0.594480
C	1.961151	−5.072975	−1.651556
O	1.857550	−6.096769	−2.348693
C	1.190491	−3.851444	−1.823051
C	0.149999	−3.798311	−2.907885
C	1.443524	−2.809530	−0.989872
H	3.435769	−5.880025	−0.445673
H	−0.603245	−4.582906	−2.764923
H	0.605101	−3.966097	−3.892011
H	−0.352682	−2.827113	−2.916763
H	0.882716	−1.881311	−1.045176
P	4.810282	1.671719	1.718948
O	4.942739	2.210892	0.310276
O	5.151269	2.617256	2.855622
O	5.737005	0.323840	1.793000
C	5.921243	−0.325523	3.084543
C	7.071525	−1.319163	3.020942
O	6.711428	−2.477331	2.211927
C	7.582463	−2.585135	1.057854
C	8.384840	−0.772262	2.425882
C	8.338198	−1.251925	0.973150
O	9.470600	−1.360312	3.208966
H	5.000095	−0.852460	3.359150
H	6.141182	0.432734	3.844161
H	7.252928	−1.667544	4.044274
H	8.257296	−3.431608	1.195005
H	8.459312	0.315048	2.500034
H	7.786798	−0.509539	0.394109
H	9.328890	−1.353904	0.535965
N	6.779945	−2.926152	−0.125315
C	6.736085	−4.294236	−0.563212
O	7.367658	−5.155140	0.071779
N	6.008365	−4.583076	−1.679546
C	5.306704	−3.632840	−2.307454
N	4.608147	−3.984636	−3.407247
C	5.260019	−2.280864	−1.835106
C	6.005502	−1.984194	−0.738843
H	4.633449	−4.941700	−3.736999
H	4.070638	−3.304797	−3.927184
H	4.652801	−1.528033	−2.322656
H	6.011768	−0.998119	−0.291941
P	11.039440	−1.339183	2.736786
O	11.315237	−0.273398	1.696565
O	11.868361	−1.287854	4.006907
O	11.217167	−2.783567	1.989034
C	11.077633	−3.997819	2.784711
C	11.588419	−5.194299	1.999319
O	10.698401	−5.477908	0.879957
C	11.424764	−5.424029	−0.371439
C	13.003975	−5.036510	1.407787
C	12.744863	−4.696610	−0.065661
O	13.676688	−6.314016	1.568938
H	10.021081	−4.143660	3.035826
H	11.656233	−3.897234	3.709779
H	11.573179	−6.058883	2.675055
H	11.588677	−6.435258	−0.744660
H	13.570594	−4.250408	1.917489
H	12.632198	−3.615197	−0.174101
H	13.554506	−5.029431	−0.719860
H	14.561231	−6.233455	1.167139
N	10.575311	−4.765721	−1.375538
C	10.043918	−5.519968	−2.467488
O	10.346115	−6.726063	−2.587787
N	9.223343	−4.881023	−3.348006
C	8.906881	−3.589899	−3.179879
N	8.087365	−3.017750	−4.086551
C	9.405320	−2.820375	−2.079597
C	10.222243	−3.456355	−1.201370
H	7.683946	−3.580024	−4.825746
H	7.772820	−2.063348	−3.977337
H	9.128627	−1.782545	−1.942371
H	10.618324	−2.977241	−0.313572
Na	13.042801	0.655890	3.135962
Na	5.647752	4.359895	1.242217

(TCG)$^{Na+}$

E: −13661.27 kcal mol^{-1}

O	−0.212077	−0.257044	−0.278757
C	−0.138972	0.073720	1.130183
C	1.286603	−0.093325	1.639033
O	1.663103	−1.509656	1.692281
C	2.765796	−1.775441	0.793663
C	2.375223	0.597529	0.793931
C	2.903157	−0.531349	−0.092687
O	3.366287	1.114836	1.738501
H	−1.152411	−0.286056	−0.527618
H	−0.799206	−0.580911	1.712635
H	−0.443535	1.117086	1.295256
H	1.319428	0.293235	2.663265
H	3.671459	−1.971793	1.369934
H	1.987289	1.436507	0.212160
H	2.263663	−0.605502	−0.974456
H	3.925389	−0.365816	−0.423108
N	2.499781	−3.019764	0.052418
C	3.309662	−4.133220	0.304589
O	4.214361	−4.153785	1.140783
N	3.016883	−5.225035	−0.495701
C	2.007812	−5.344880	−1.458918
O	1.892512	−6.401444	−2.104003
C	1.160891	−4.171065	−1.599916
C	0.011315	−4.213006	−2.569697
C	1.443038	−3.081708	−0.840458
H	3.629086	−6.028867	−0.369489
H	−0.687135	−5.019140	−2.313438
H	0.367172	−4.407984	−3.588739
H	−0.532748	−3.264331	−2.566211
H	0.831124	−2.185474	−0.875508
P	4.859342	1.611213	1.281312
O	4.917911	1.986852	−0.184515
O	5.279737	2.674539	2.279047
O	5.768150	0.263562	1.453020
C	5.986987	−0.272708	2.789207
C	7.192545	−1.201433	2.791314
O	6.915996	−2.425407	2.054010
C	7.752979	−2.509116	0.867087
C	8.472013	−0.608456	2.167599
C	8.439005	−1.144595	0.732986
O	9.592957	−1.066940	2.995102
H	5.095934	−0.826569	3.105852
H	6.165434	0.551378	3.489116
H	7.386439	−1.476661	3.834416
H	8.475613	−3.311843	1.006598
H	8.482446	0.483607	2.194790
H	7.843802	−0.445434	0.144224
H	9.421683	−1.219052	0.274651
N	6.935769	−2.931828	−0.278222
C	6.878601	−4.336127	−0.583047
O	7.528785	−5.133259	0.113279
N	6.114003	−4.730389	−1.641302
C	5.382378	−3.844529	−2.325270
N	4.635532	−4.304518	−3.352558
C	5.344544	−2.455430	−1.978872
C	6.132255	−2.053109	−0.947230
H	4.660057	−5.287694	−3.591544
H	4.091391	−3.677244	−3.928371
H	4.716695	−1.752226	−2.512707
H	6.145095	−1.028641	−0.598331
P	11.080131	−1.477297	2.466275
O	11.550424	−0.635047	1.295096
O	11.971835	−1.485844	3.693902
O	10.881168	−2.981497	1.873625
C	10.704648	−4.128624	2.746436
C	11.459724	−5.314766	2.161007
O	10.872061	−5.749623	0.900004
C	11.681872	−5.320039	−0.217494
C	12.938970	−5.036839	1.848585
C	12.929610	−4.626154	0.365491
O	13.662894	−6.274591	2.075486
H	9.636261	−4.361620	2.810303
H	11.090358	−3.905457	3.746959
H	11.369813	−6.152048	2.862434
H	11.931834	−6.214048	−0.794212
H	13.346422	−4.244388	2.486104
H	12.855679	−3.541744	0.292370
H	13.838657	−4.944079	−0.150409
H	14.594134	−6.120167	1.832595
N	10.894559	−4.477597	−1.127667
C	10.788464	−3.087216	−1.192191
N	9.947320	−2.684270	−2.126246
C	9.459696	−3.854202	−2.706964
C	8.525715	−4.044470	−3.769430
O	7.913140	−3.195273	−4.443129
N	8.330567	−5.427824	−4.028578
C	8.936303	−6.468631	−3.358510
N	8.566378	−7.732986	−3.708837
N	9.812976	−6.280295	−2.377591
C	10.033633	−4.976407	−2.094530
H	11.332224	−2.435908	−0.524744
H	7.655249	−5.637722	−4.760175
H	8.146417	−7.892576	−4.617258
H	9.150904	−8.482826	−3.356315
Na	13.555316	−0.077687	2.525046
Na	5.754590	4.187441	0.437177

(TCT)$^{Na+}$

E: −13358.59 kcal mol^{-1}

O	−0.114688	0.049055	−0.381229
C	−0.153290	0.300981	1.045754
C	1.213862	0.040320	1.666136
O	1.520192	−1.394429	1.676844
C	2.678074	−1.670471	0.853889
C	2.400006	0.713165	0.949236
C	2.934637	−0.394357	0.041481
O	3.348935	1.110830	1.990860
H	−1.030373	0.069941	−0.710109
H	−0.889321	−0.352407	1.530829
H	−0.428021	1.347082	1.242655
H	1.174633	0.374829	2.708209
H	3.526924	−1.930939	1.488330
H	2.105181	1.605141	0.392481
H	2.357476	−0.395259	−0.885176
H	3.983724	−0.259706	−0.205706
N	2.418886	−2.870666	0.040577
C	3.166951	−4.025487	0.296980
O	4.019996	−4.113200	1.182328
N	2.880537	−5.075196	−0.559560
C	1.934396	−5.115636	−1.590801
O	1.816565	−6.142633	−2.281098
C	1.151952	−3.899206	−1.744146
C	0.075891	−3.857353	−2.794242
C	1.425971	−2.851571	−0.925145
H	3.445668	−5.911105	−0.423086
H	−0.667803	−4.645160	−2.622133
H	0.498788	−4.027826	−3.792047
H	−0.432263	−2.889011	−2.791378
H	0.859275	−1.926273	−0.967944
P	4.861972	1.640714	1.646842
O	4.979887	2.148398	0.225322
O	5.240703	2.603396	2.756656

O	5.767326	0.277951	1.731826
C	5.940888	−0.362419	3.029448
C	7.097768	−1.349551	2.984856
O	6.754114	−2.521716	2.189938
C	7.628810	−2.644482	1.040883
C	8.410609	−0.794252	2.396877
C	8.419574	−1.330335	0.962431
O	9.494870	−1.311239	3.228422
H	5.018780	−0.890346	3.298314
H	6.150650	0.401686	3.786117
H	7.273894	−1.684229	4.013783
H	8.280544	−3.509459	1.174293
H	8.450846	0.296996	2.424974
H	7.926694	−0.597587	0.322318
H	9.428514	−1.481359	0.586547
N	6.821638	−2.953449	−0.151938
C	6.741935	−4.313850	−0.608760
O	7.383754	−5.194657	−0.011186
N	5.974862	−4.576256	−1.704696
C	5.273648	−3.605685	−2.301629
N	4.525106	−3.934541	−3.374612
C	5.268244	−2.259187	−1.813069
C	6.044929	−1.990322	−0.732103
H	4.543634	−4.882423	−3.730109
H	4.009183	−3.233177	−3.887888
H	4.664788	−1.489095	−2.277346
H	6.073505	−1.012236	−0.268001
P	11.072970	−1.190369	2.797841
O	11.304420	−0.111137	1.761042
O	11.859312	−1.079805	4.091933
O	11.361101	−2.623010	2.063566
C	11.216269	−3.834495	2.860247
C	11.586039	−5.044640	2.023841
O	10.599864	−5.216964	0.963053
C	11.274124	−5.351462	−0.308255
C	12.972379	−4.983029	1.342159
C	12.643128	−4.682114	−0.126178
O	13.588911	−6.286454	1.514115
H	10.177299	−3.924428	3.197079
H	11.873330	−3.779719	3.735897
H	11.552223	−5.923768	2.680513
H	11.376195	−6.407466	−0.565068
H	13.607633	−4.210235	1.786207
H	12.580663	−3.600245	−0.264157
H	13.391296	−5.081424	−0.815206
H	14.466115	−6.255452	1.090134
N	10.419139	−4.772348	−1.352391
C	9.723277	−5.643121	−2.198400
O	9.798019	−6.874534	−2.134507
N	8.941182	−5.002270	−3.141069
C	8.745715	−3.626213	−3.310986
O	8.008621	−3.212321	−4.219383
C	9.453188	−2.791664	−2.351976
C	9.298077	−1.299379	−2.446886
C	10.235996	−3.403459	−1.427435
H	8.422292	−5.615722	−3.766409
H	9.613909	−0.944149	−3.435078
H	9.899309	−0.792140	−1.688730
H	8.249431	−1.005150	−2.322619
H	10.770595	−2.836806	−0.674940
Na	12.880811	0.969944	3.262540
Na	5.775356	4.289978	1.093266

$(TGA)^{Na+}$

E: −14064.11 kcal mol^{-1}

O	−0.138742	−0.408468	−0.233023
C	−0.068987	−0.069266	1.174390
C	1.368592	−0.158531	1.670345
O	1.815254	−1.552342	1.746431
C	2.915365	−1.788456	0.834304
C	2.410101	0.570752	0.799125
C	2.999708	−0.546401	−0.063624
O	3.376272	1.169716	1.719342
H	−1.078054	−0.502473	−0.468937
H	−0.688432	−0.755384	1.765362
H	−0.426392	0.957471	1.338379
H	1.392587	0.248528	2.686903
H	3.833887	−1.949752	1.402038
H	1.968403	1.368015	0.197561
H	2.382329	−0.660657	−0.956552
H	4.016644	−0.333349	−0.380096
N	2.678019	−3.050418	0.108763
C	3.466867	−4.162126	0.424858
O	4.344297	−4.164760	1.290077
N	3.184791	−5.281839	−0.342358
C	2.168247	−5.444239	−1.294326
O	2.025770	−6.539037	−1.863855
C	1.343146	−4.265533	−1.503827
C	0.196270	−4.342138	−2.475065
C	1.629920	−3.145631	−0.791917
H	3.744088	−6.105230	−0.128915
H	−0.508527	−5.131670	−2.186351
H	0.553901	−4.583425	−3.483663
H	−0.341568	−3.390636	−2.514397
H	1.020008	−2.251192	−0.868881
P	4.764761	1.884343	1.219774
O	4.762972	2.164403	−0.268818
O	4.999459	3.069471	2.139686
O	5.886104	0.723151	1.479358
C	6.094777	0.253422	2.840959
C	7.165439	−0.827903	2.871606
O	6.733830	−1.990275	2.103046
C	7.632173	−2.191977	0.979593
C	8.544229	−0.441915	2.297981
C	8.436311	−0.899698	0.843974
O	9.532950	−1.181700	3.096185
H	5.157477	−0.164866	3.224994
H	6.400140	1.091215	3.479001
H	7.281201	−1.134640	3.917609
H	8.282181	−3.041670	1.192883
H	8.767386	0.622712	2.397666
H	7.868923	−0.144697	0.295400
H	9.399051	−1.044070	0.357504
N	6.878605	−2.584016	−0.194770
C	6.102660	−1.824547	−1.064428
N	5.541942	−2.556661	−2.011878
C	5.971619	−3.863704	−1.764994
C	5.718980	−5.088134	−2.455553
O	5.004005	−5.302783	−3.447878
N	6.440801	−6.166934	−1.853188
C	7.286340	−6.070046	−0.764060
N	7.936171	−7.191013	−0.338677
N	7.466800	−4.932552	−0.106967
C	6.806518	−3.889701	−0.641142
H	6.002816	−0.755693	−0.944828
H	6.334357	−7.065190	−2.320253
H	8.029107	−7.962853	−0.990334
H	8.778121	−6.986651	0.203900
P	10.959443	−1.780338	2.570786
O	11.582548	−0.925886	1.484165
O	11.794633	−2.025488	3.812799
O	10.562982	−3.190821	1.847173
C	10.307940	−4.412152	2.594275
C	11.104408	−5.569685	2.000341
O	10.581822	−5.989662	0.701246

C	11.445330	−5.537919	−0.372436
C	12.590613	−5.265156	1.757461
C	12.634562	−4.813138	0.287259
O	13.320767	−6.499557	1.977044
H	9.237815	−4.634655	2.533344
H	10.598778	−4.277848	3.640980
H	10.994955	−6.426780	2.673853
H	11.749786	−6.421082	−0.938566
H	12.958957	−4.486171	2.433756
H	12.517059	−3.730416	0.235579
H	13.578341	−5.079538	−0.193863
H	14.259242	−6.326438	1.778822
N	10.686705	−4.712834	−1.316885
C	10.374190	−3.359280	−1.239495
N	9.558084	−2.958470	−2.199578
C	9.301836	−4.104233	−2.951139
C	8.482179	−4.348110	−4.075464
N	7.742672	−3.378019	−4.672978
N	8.449248	−5.606950	−4.583268
C	9.180940	−6.568558	−3.986987
N	9.968514	−6.461030	−2.900748
C	9.995657	−5.204492	−2.420303
H	10.769002	−2.734126	−0.453703
H	7.016231	−3.674322	−5.315822
H	7.566597	−2.524041	−4.156095
H	9.118640	−7.557565	−4.438194
Na	13.581656	−0.703657	2.842983
Na	5.159485	4.510851	0.164686

(TGC)$^{Na+}$

E: −13664.72 kcal mol^{-1}

O	−0.235956	−0.257135	−0.164946
C	−0.129638	0.061087	1.245078
C	1.315202	−0.069565	1.709545
O	1.730782	−1.475140	1.743256
C	2.808088	−1.709047	0.804301
C	2.356132	0.654587	0.832530
C	2.882215	−0.455849	−0.077290
O	3.365848	1.191427	1.744955
H	−1.181977	−0.324231	−0.382820
H	−0.752384	−0.619079	1.839514
H	−0.459778	1.093254	1.430775
H	1.369412	0.312939	2.734347
H	3.736816	−1.884857	1.350752
H	1.924960	1.484750	0.268777
H	2.216889	−0.540308	−0.938451
H	3.885325	−0.256821	−0.442775
N	2.547152	−2.957584	0.066097
C	3.342034	−4.075497	0.342870
O	4.221297	−4.102606	1.205933
N	3.064298	−5.168887	−0.462584
C	2.041732	−5.304757	−1.412404
O	1.908888	−6.377055	−2.025906
C	1.200639	−4.128912	−1.568198
C	0.041975	−4.183835	−2.526868
C	1.487830	−3.030730	−0.823468
H	3.630379	−5.995623	−0.281949
H	−0.653310	−4.987266	−2.253733
H	0.388561	−4.391260	−3.546608
H	−0.503462	−3.235830	−2.529863
H	0.872465	−2.137633	−0.864481
P	4.785991	1.843213	1.251343
O	4.822724	2.085595	−0.243459
O	5.056030	3.040142	2.145340
O	5.856462	0.644535	1.557251
C	6.036449	0.201072	2.931668
C	7.114489	−0.871434	3.007644
O	6.706741	−2.061373	2.266027
C	7.582657	−2.256456	1.128997
C	8.495855	−0.490193	2.441509
C	8.404899	−0.971783	0.994449
O	9.476963	−1.225810	3.246441
H	5.093065	−0.216954	3.300965
H	6.321649	1.052495	3.560958
H	7.215794	−1.151737	4.062156
H	8.218239	−3.125503	1.315932
H	8.714755	0.576807	2.525033
H	7.859238	−0.220843	0.419889
H	9.377208	−1.133015	0.531993
N	6.809006	−2.604287	−0.050553
C	6.034805	−1.795171	−0.878843
N	5.506012	−2.465697	−1.887486
C	5.954439	−3.779496	−1.728733
C	5.775694	−4.942797	−2.538781
O	5.116348	−5.083376	−3.583559
N	6.505273	−6.049316	−2.006209
C	7.315454	−6.021881	−0.887195
N	8.001187	−7.149343	−0.549628
N	7.434173	−4.944027	−0.122961
C	6.762404	−3.876416	−0.588589
H	5.909095	−0.741655	−0.675904
H	6.456548	−6.901884	−2.560665
H	8.147013	−7.859973	−1.258052
H	8.793914	−6.977271	0.069988
P	10.987154	−1.612664	2.745345
O	11.525031	−0.621108	1.734548
O	11.805817	−1.828925	4.003326
O	10.783039	−3.023228	1.938592
C	10.537605	−4.266055	2.659602
C	11.208180	−5.435138	1.946297
O	10.522533	−5.765942	0.700151
C	11.379476	−5.512716	−0.443988
C	12.682044	−5.199875	1.567484
C	12.612171	−4.768019	0.094937
O	13.372772	−6.465638	1.736820
H	9.457128	−4.437634	2.712775
H	10.943429	−4.187042	3.673384
H	11.127668	−6.307563	2.605393
H	11.640129	−6.464644	−0.905321
H	13.144768	−4.433537	2.198324
H	12.480633	−3.685482	0.039608
H	13.516159	−5.035297	−0.457872
H	14.296016	−6.338096	1.451339
N	10.606421	−4.786328	−1.465953
C	10.177787	−5.474734	−2.646679
O	10.540149	−6.653406	−2.837628
N	9.370325	−4.807848	−3.518481
C	9.002199	−3.540305	−3.285224
N	8.205094	−2.938806	−4.195085
C	9.424119	−2.828732	−2.117331
C	10.202553	−3.500491	−1.230672
H	7.818708	−3.488828	−4.953489
H	7.779135	−2.043866	−3.992574
H	9.108689	−1.809877	−1.929833
H	10.517514	−3.070831	−0.287692
Na	13.445015	−0.228165	3.174078
Na	5.339142	4.415981	0.135799

(TGG)$^{Na+}$

E: −14227.10 kcal mol^{-1}

O	−0.137336	−0.305397	−0.369188
C	−0.023862	0.095601	1.018644

C	1.410867	−0.066887	1.503940
O	1.769524	−1.483577	1.621961
C	2.843381	−1.810509	0.704756
C	2.493340	0.563715	0.603364
C	2.963678	−0.617214	−0.248443
O	3.508977	1.125520	1.502237
H	−1.084337	−0.335945	−0.591267
H	−0.678559	−0.518487	1.649700
H	−0.310308	1.150508	1.137395
H	1.470121	0.370885	2.506112
H	3.764898	−1.977235	1.261543
H	2.107311	1.384902	−0.004448
H	2.274964	−0.717146	−1.089771
H	3.969135	−0.499259	−0.642847
N	2.548634	−3.093434	0.048144
C	3.346194	−4.198533	0.365325
O	4.271816	−4.172935	1.178474
N	3.010733	−5.345134	−0.337800
C	1.952446	−5.528662	−1.239252
O	1.775133	−6.641565	−1.762752
C	1.132367	−4.348056	−1.458856
C	−0.052396	−4.444771	−2.381180
C	1.463422	−3.205251	−0.804264
H	3.575531	−6.165135	−0.126257
H	−0.752210	−5.217086	−2.038853
H	0.262938	−4.722522	−3.394631
H	−0.582854	−3.489592	−2.430897
H	0.868134	−2.301616	−0.893976
P	5.106664	1.224714	1.180372
O	5.394861	1.480994	−0.287200
O	5.678079	2.223301	2.168777
O	5.653159	−0.281496	1.480520
C	5.954959	−0.742932	2.823804
C	7.329615	−1.403205	2.838812
O	7.346438	−2.633083	2.060996
C	7.971636	−2.412680	0.776186
C	8.459898	−0.539492	2.247376
C	8.496817	−0.967899	0.772636
O	9.673430	−0.797796	3.030357
H	5.193369	−1.473272	3.116655
H	5.944962	0.098190	3.524566
H	7.561327	−1.675755	3.873795
H	8.771755	−3.147610	0.685920
H	8.258454	0.528918	2.352211
H	7.830201	−0.305342	0.222459
H	9.488730	−0.891820	0.331701
N	7.039896	−2.719708	−0.315118
C	6.250329	−1.886727	−1.108813
N	5.547017	−2.557239	−2.003778
C	5.875634	−3.897567	−1.797773
C	5.446662	−5.081773	−2.467558
O	4.665516	−5.211750	−3.426898
N	6.054899	−6.238357	−1.890682
C	6.970359	−6.238566	−0.854855
N	7.451893	−7.444927	−0.434684
N	7.359593	−5.128949	−0.246091
C	6.795534	−4.010821	−0.746018
H	6.227963	−0.815241	−0.974375
H	5.808787	−7.121007	−2.334726
H	7.421114	−8.225906	−1.081626
H	8.302491	−7.382065	0.117350
P	11.131021	−1.282314	2.483067
O	11.614066	−0.486541	1.284055
O	12.039330	−1.283133	3.698092
O	10.878211	−2.790641	1.923547
C	10.600355	−3.906745	2.812829
C	11.246745	−5.163432	2.242454
O	10.631750	−5.547773	0.977021
C	11.523851	−5.274216	−0.128776
C	12.751185	−5.032894	1.949822
C	12.799979	−4.646560	0.463060
O	13.348053	−6.332588	2.201133
H	9.515017	−4.036191	2.879943
H	11.008143	−3.705368	3.809456
H	11.070774	−5.981574	2.950680
H	11.720567	−6.222215	−0.635943
H	13.224819	−4.275409	2.583857
H	12.786354	−3.560268	0.372603
H	13.698463	−5.023728	−0.031169
H	14.291708	−6.274644	1.964042
N	10.847926	−4.434862	−1.124216
C	10.752582	−3.043087	−1.191595
N	9.969206	−2.634965	−2.172696
C	9.511821	−3.801687	−2.783852
C	8.625247	−3.986777	−3.887907
O	8.055635	−3.137181	−4.595628
N	8.415840	−5.372781	−4.139538
C	8.998551	−6.416565	−3.451216
N	8.619703	−7.680087	−3.797071
N	9.837847	−6.230569	−2.439862
C	10.047186	−4.927859	−2.143214
H	11.260835	−2.398445	−0.490060
H	7.780243	−5.580078	−4.907270
H	8.219370	−7.842577	−4.714193
H	9.198307	−8.430355	−3.435151
Na	13.643073	0.058077	2.476992
Na	6.693807	3.425745	0.319713

(TGT)$^{Na+}$

E: −13922.66 kcal mol^{-1}

O	−0.213971	−0.207131	−0.111905
C	−0.091476	0.118791	1.294903
C	1.353586	−0.033130	1.753337
O	1.751416	−1.443810	1.783778
C	2.816341	−1.687011	0.830629
C	2.406006	0.675950	0.876834
C	2.886571	−0.438370	−0.053627
O	3.427853	1.194645	1.794158
H	−1.162830	−0.260242	−0.321166
H	−0.721485	−0.546474	1.898490
H	−0.402839	1.157646	1.476031
H	1.415371	0.346971	2.778597
H	3.751621	−1.858879	1.363667
H	1.994029	1.526607	0.329451
H	2.183712	−0.505380	−0.886264
H	3.879191	−0.268595	−0.460219
N	2.546436	−2.940826	0.110222
C	3.350472	−4.050365	0.393188
O	4.262262	−4.049544	1.222149
N	3.041180	−5.168816	−0.364636
C	1.998786	−5.325818	−1.289387
O	1.845647	−6.416473	−1.863891
C	1.165609	−4.147535	−1.467631
C	−0.005618	−4.220631	−2.409063
C	1.471499	−3.030943	−0.758417
H	3.606823	−5.993098	−0.173088
H	−0.699770	−5.015011	−2.107991
H	0.327136	−4.453700	−3.428025
H	−0.547593	−3.270852	−2.427606
H	0.862254	−2.134412	−0.814020
P	5.002445	1.420539	1.425359
O	5.225646	1.745533	−0.039239
O	5.536294	2.418453	2.434960
O	5.666464	−0.052354	1.651510
C	5.909344	−0.585400	2.979267

C	7.231609	−1.341335	2.983828
O	7.170515	−2.553395	2.175497
C	7.854889	−2.369613	0.918850
C	8.427024	−0.540468	2.432590
C	8.500826	−0.972606	0.959910
O	9.585842	−0.884171	3.256629
H	5.092080	−1.264960	3.244243
H	5.956191	0.230092	3.709028
H	7.432868	−1.656478	4.012805
H	8.588241	−3.175452	0.831902
H	8.278605	0.538295	2.524641
H	7.928062	−0.254582	0.374555
H	9.519125	−0.976809	0.575733
N	6.940766	−2.556519	−0.217320
C	6.162334	−1.616884	−0.898085
N	5.448743	−2.155682	−1.868117
C	5.754737	−3.515332	−1.834678
C	5.280349	−4.591774	−2.642915
O	4.471208	−4.573252	−3.585993
N	5.863476	−5.825995	−2.231475
C	6.772235	−5.986377	−1.202686
N	7.194712	−7.250391	−0.934509
N	7.207772	−4.972807	−0.463142
C	6.673617	−3.781693	−0.809802
H	6.157653	−0.571736	−0.630404
H	5.564835	−6.642285	−2.761739
H	7.080854	−7.978445	−1.629506
H	8.002308	−7.335290	−0.327690
P	11.122654	−1.038772	2.723118
O	11.473731	−0.046819	1.632342
O	11.991888	−1.017144	3.966322
O	11.126245	−2.516349	2.022815
C	10.869704	−3.677147	2.866579
C	11.349507	−4.928363	2.155870
O	10.491319	−5.190864	1.003622
C	11.310400	−5.382045	−0.171367
C	12.800591	−4.858724	1.625649
C	12.625203	−4.645396	0.114936
O	13.428477	−6.130922	1.932900
H	9.794569	−3.746602	3.067484
H	11.406962	−3.570069	3.815504
H	11.261829	−5.770107	2.854512
H	11.477147	−6.446834	−0.343125
H	13.361810	−4.043216	2.092136
H	12.534188	−3.575938	−0.090383
H	13.458711	−5.047236	−0.466224
H	14.346169	−6.092668	1.606220
N	10.551988	−4.906343	−1.335715
C	10.059798	−5.854134	−2.237120
O	10.274549	−7.067408	−2.151683
N	9.287402	−5.314788	−3.251005
C	8.973686	−3.965626	−3.473666
O	8.274484	−3.648434	−4.447838
C	9.515999	−3.046324	−2.484862
C	9.243324	−1.577374	−2.647127
C	10.255629	−3.561146	−1.470208
H	8.938547	−5.983037	−3.935760
H	9.677521	−1.207941	−3.584081
H	9.671265	−1.004901	−1.820895
H	8.166872	−1.382033	−2.694342
H	10.660197	−2.931901	−0.685922
Na	13.323803	0.767239	2.988470
Na	6.418872	3.746241	0.593432

(TTA)$^{Na+}$

E: −13755.85 kcal mol^{-1}

O	−0.290960	0.011747	−0.075567
C	−0.122257	0.310990	1.332575
C	1.323306	0.076314	1.753889
O	1.642360	−1.356165	1.756060
C	2.671457	−1.642984	0.779683
C	2.387689	0.741168	0.856147
C	2.792542	−0.388861	−0.090328
O	3.460930	1.212649	1.738179
H	−1.244938	0.020006	−0.267649
H	−0.773399	−0.328877	1.941059
H	−0.374408	1.362026	1.533753
H	1.431965	0.437448	2.782003
H	3.606752	−1.875714	1.287814
H	1.999023	1.610326	0.321011
H	2.075825	−0.410163	−0.913220
H	3.788514	−0.261135	−0.504635
N	2.320085	−2.863495	0.035478
C	3.108355	−4.004896	0.220207
O	4.073075	−4.062935	0.986035
N	2.722121	−5.078697	−0.564957
C	1.646213	−5.153629	−1.460468
O	1.440776	−6.204020	−2.091021
C	0.844080	−3.942982	−1.547790
C	−0.354215	−3.928403	−2.457351
C	1.209869	−2.875494	−0.793437
H	3.282595	−5.921314	−0.454238
H	−1.059090	−4.724631	−2.188651
H	−0.053757	−4.101995	−3.498189
H	−0.872459	−2.966954	−2.400590
H	0.631520	−1.956533	−0.781052
P	5.038641	1.291460	1.314505
O	5.238184	1.621203	−0.151496
O	5.702214	2.213976	2.318962
O	5.556173	−0.247157	1.499720
C	5.866144	−0.776915	2.818430
C	7.209921	−1.494377	2.779127
O	7.131425	−2.733873	2.019560
C	7.796268	−2.597623	0.745156
C	8.341732	−0.678131	2.125424
C	8.322269	−1.157293	0.664575
O	9.571686	−0.926964	2.883841
H	5.078313	−1.481543	3.104434
H	5.911174	0.038173	3.547574
H	7.480550	−1.761614	3.806176
H	8.611078	−3.317945	0.720129
H	8.161340	0.396687	2.195087
H	7.647552	−0.496067	0.123679
H	9.296619	−1.106652	0.183721
N	6.887177	−3.027100	−0.348127
C	6.847101	−4.408798	−0.584356
O	7.498824	−5.229785	0.061645
N	6.019404	−4.783806	−1.630256
C	5.218378	−3.960928	−2.431998
O	4.534419	−4.457891	−3.340893
C	5.273239	−2.551479	−2.087003
C	4.458332	−1.587137	−2.904196
C	6.085061	−2.163114	−1.066916
H	6.015227	−5.778725	−1.846839
H	4.719072	−1.671169	−3.966011
H	4.633121	−0.556779	−2.586068
H	3.386736	−1.805357	−2.823298
H	6.136479	−1.125425	−0.767075
P	11.011128	−1.425000	2.304902
O	11.455766	−0.653267	1.074964
O	11.960553	−1.408500	3.487111
O	10.728046	−2.941533	1.784726
C	10.652645	−4.075620	2.689452
C	11.533985	−5.195694	2.151047
O	11.023810	−5.706873	0.883833

C	11.815040	−5.214632	−0.221720
C	12.987714	−4.783962	1.866069
C	12.973761	−4.393133	0.376955
O	13.817071	−5.947122	2.123145
H	9.610071	−4.406620	2.735256
H	10.990675	−3.787741	3.690219
H	11.506152	−6.025364	2.866463
H	12.164212	−6.087349	−0.779151
H	13.305097	−3.950434	2.501982
H	12.795951	−3.322184	0.285553
H	13.921499	−4.626757	−0.113452
H	14.735195	−5.710928	1.896814
N	10.961757	−4.469526	−1.158482
C	10.729361	−3.102238	−1.265038
N	9.823293	−2.801082	−2.182692
C	9.425461	−4.029443	−2.708679
C	8.482422	−4.388358	−3.698435
N	7.746150	−3.479321	−4.375759
N	8.309791	−5.708783	−3.970813
C	9.049034	−6.612794	−3.297308
N	9.970208	−6.390038	−2.342367
C	10.119228	−5.077290	−2.083454
H	11.233815	−2.379583	−0.640289
H	7.034875	−3.790565	−5.024986
H	7.821102	−2.494216	−4.162182
H	8.872619	−7.654585	−3.560683
Na	13.512209	−0.072500	2.192182
Na	6.631896	3.504915	0.494978

(TTC)$^{Na+}$

E: −13358.39 kcal mol^{-1}

O	−0.272588	0.000407	−0.063653
C	−0.144684	0.205830	1.365084
C	1.298816	−0.009778	1.800179
O	1.658434	−1.431236	1.743009
C	2.711609	−1.652177	0.778719
C	2.355181	0.728198	0.952984
C	2.847006	−0.347885	−0.015145
O	3.380352	1.210022	1.878641
H	−1.221553	−0.002018	−0.279361
H	−0.786779	−0.494900	1.913160
H	−0.435418	1.232064	1.631801
H	1.381919	0.305486	2.845523
H	3.634073	−1.919706	1.297346
H	1.940319	1.592841	0.430381
H	2.190602	−0.349041	−0.886956
H	3.863443	−0.170520	−0.355154
N	2.379734	−2.824719	−0.053022
C	3.144426	−3.987130	0.091455
O	4.068857	−4.111644	0.897598
N	2.787756	−4.999829	−0.784104
C	1.736717	−5.011041	−1.712650
O	1.547246	−6.015729	−2.417871
C	0.940347	−3.793949	−1.739666
C	−0.240391	−3.717695	−2.668934
C	1.292354	−2.777986	−0.911043
H	3.332998	−5.855741	−0.701977
H	−0.961558	−4.514635	−2.450097
H	0.075780	−3.846935	−3.711358
H	−0.745699	−2.752451	−2.574959
H	0.715103	−1.860031	−0.855185
P	4.885104	1.653363	1.405166
O	4.952294	2.023458	−0.061401
O	5.354701	2.697391	2.400755
O	5.740392	0.268311	1.572356
C	5.898692	−0.289622	2.908123
C	7.052688	−1.277736	2.920926
O	6.718982	−2.465260	2.139436
C	7.611803	−2.595903	1.010982
C	8.374595	−0.740156	2.338684
C	8.364606	−1.263631	0.899603
O	9.446375	−1.293941	3.161669
H	4.972954	−0.798618	3.199021
H	6.104727	0.516146	3.621620
H	7.208207	−1.592579	3.958707
H	8.293018	−3.430965	1.186478
H	8.437465	0.349684	2.378925
H	7.827747	−0.541656	0.283374
H	9.366666	−1.379051	0.494213
N	6.836536	−2.985844	−0.182790
C	6.828497	−4.344722	−0.527188
O	7.417372	−5.218474	0.108822
N	6.113217	−4.629158	−1.679475
C	5.383154	−3.744631	−2.482570
O	4.791897	−4.164992	−3.488695
C	5.386226	−2.365846	−2.017856
C	4.632547	−1.344326	−2.823983
C	6.090672	−2.064974	−0.895509
H	6.135648	−5.603807	−1.973880
H	4.976966	−1.353271	−3.864885
H	4.775359	−0.339381	−2.420363
H	3.559138	−1.568816	−2.844047
H	6.100919	−1.060948	−0.489191
P	11.029152	−1.240744	2.736995
O	11.303619	−0.205087	1.666630
O	11.816219	−1.118926	4.028618
O	11.267493	−2.706533	2.049208
C	11.104214	−3.890509	2.885105
C	11.552928	−5.127711	2.126558
O	10.642729	−5.388021	1.018391
C	11.370717	−5.430413	−0.232897
C	12.973054	−5.058630	1.527723
C	12.727964	−4.762827	0.042624
O	13.585950	−6.358810	1.737809
H	10.049206	−3.992200	3.163440
H	11.706053	−3.782278	3.794517
H	11.498090	−5.974343	2.822961
H	11.480015	−6.465209	−0.558932
H	13.577508	−4.281502	2.006845
H	12.674413	−3.681751	−0.106236
H	13.516938	−5.162634	−0.599280
H	14.475862	−6.332047	1.340802
N	10.556983	−4.774125	−1.266569
C	9.934701	−5.558271	−2.289117
O	10.120292	−6.791866	−2.318007
N	9.159390	−4.912874	−3.206582
C	8.967600	−3.588668	−3.134540
N	8.178686	−3.014523	−4.067011
C	9.541218	−2.790029	−2.092772
C	10.317387	−3.431123	−1.181506
H	7.789069	−3.576435	−4.814088
H	8.034705	−2.014379	−4.085144
H	9.355598	−1.725149	−2.025306
H	10.765236	−2.924778	−0.334443
Na	12.937496	0.851513	3.122577
Na	5.892964	4.186366	0.558838

(TTG)$^{Na+}$

E: −13919.69 kcal mol^{-1}

O	−0.283701	−0.055016	−0.045358
C	−0.101283	0.234165	1.363023
C	1.353162	0.020111	1.763522

O	1.695053	−1.406905	1.756200
C	2.713323	−1.675558	0.763914
C	2.394916	0.705434	0.854540
C	2.808704	−0.414938	−0.100306
O	3.468398	1.196114	1.725154
H	−1.239931	−0.055583	−0.226017
H	−0.733568	−0.421780	1.974276
H	−0.368019	1.279001	1.577185
H	1.470042	0.378756	2.791567
H	3.657887	−1.901856	1.257571
H	1.983990	1.568236	0.325837
H	2.085951	−0.442878	−0.917821
H	3.799315	−0.271161	−0.522669
N	2.364462	−2.895389	0.016887
C	3.165178	−4.030582	0.185272
O	4.138695	−4.084970	0.940088
N	2.779339	−5.102931	−0.602201
C	1.692179	−5.182916	−1.483560
O	1.489963	−6.230648	−2.119817
C	0.874929	−3.981158	−1.549327
C	−0.338019	−3.972807	−2.439316
C	1.241552	−2.914275	−0.794451
H	3.349086	−5.940843	−0.504357
H	−1.028619	−4.780281	−2.167199
H	−0.052612	−4.132187	−3.486660
H	−0.867147	−3.018554	−2.364156
H	0.653506	−2.001811	−0.767573
P	5.041588	1.297064	1.289757
O	5.227232	1.631387	−0.176770
O	5.698218	2.227982	2.291115
O	5.581379	−0.234402	1.470862
C	5.872156	−0.766586	2.793220
C	7.217094	−1.482389	2.773965
O	7.153083	−2.723696	2.016096
C	7.841083	−2.590353	0.752792
C	8.357176	−0.665260	2.136481
C	8.362989	−1.148408	0.677009
O	9.573281	−0.913666	2.916902
H	5.080583	−1.471660	3.067709
H	5.906386	0.047496	3.524118
H	7.472885	−1.746949	3.805474
H	8.658873	−3.308110	0.744591
H	8.174425	0.409570	2.200299
H	7.693974	−0.491692	0.123491
H	9.344428	−1.095597	0.211259
N	6.952865	−3.026263	−0.354172
C	6.921418	−4.407656	−0.587312
O	7.563164	−5.226591	0.071480
N	6.112205	−4.787641	−1.646327
C	5.303918	−3.971738	−2.449965
O	4.620646	−4.478044	−3.353709
C	5.345331	−2.562684	−2.103209
C	4.519712	−1.605242	−2.917326
C	6.147234	−2.168699	−1.078030
H	6.090010	−5.786855	−1.839373
H	4.786313	−1.680521	−3.978119
H	4.679234	−0.574380	−2.592398
H	3.450765	−1.838330	−2.839664
H	6.184609	−1.131663	−0.773872
P	11.031698	−1.379538	2.358820
O	11.482298	−0.592567	1.141112
O	11.960197	−1.351704	3.557560
O	10.787966	−2.898652	1.826450
C	10.673332	−4.029208	2.731340
C	11.515257	−5.178038	2.190359
O	10.988694	−5.667478	0.921880
C	11.801319	−5.205256	−0.181557
C	12.983069	−4.817515	1.908600
C	12.985000	−4.423241	0.420659
O	13.769145	−6.010925	2.164299
H	9.620319	−4.325616	2.780718
H	11.023912	−3.753049	3.731245
H	11.457415	−6.007673	2.904118
H	12.123305	−6.090766	−0.735026
H	13.330106	−3.997393	2.546598
H	12.841724	−3.347032	0.332282
H	13.925583	−4.685812	−0.069002
H	14.694376	−5.810411	1.932718
N	10.980727	−4.432570	−1.124074
C	10.798048	−3.052536	−1.234178
N	9.924631	−2.729001	−2.169658
C	9.495118	−3.943800	−2.703843
C	8.558409	−4.222700	−3.744284
O	7.889037	−3.434506	−4.436120
N	8.423796	−5.626658	−3.939595
C	9.111309	−6.608746	−3.255083
N	8.817237	−7.901223	−3.559392
N	9.990914	−6.334652	−2.296439
C	10.139837	−5.011155	−2.062899
H	11.311268	−2.348464	−0.596314
H	7.767598	−5.899107	−4.668611
H	8.366109	−8.120962	−4.439534
H	9.442135	−8.607092	−3.186828
Na	13.516340	0.004929	2.284658
Na	6.598505	3.536668	0.468270

(TTT)$^{Na+}$

E: −13616.88 kcal mol^{-1}

O	−0.250225	0.020205	−0.052369
C	−0.096072	0.313758	1.358437
C	1.348400	0.091453	1.788321
O	1.678600	−1.338754	1.802076
C	2.713024	−1.628798	0.834739
C	2.410432	0.759826	0.891403
C	2.849723	−0.374605	−0.033833
O	3.465433	1.256858	1.777478
H	−1.202561	0.018980	−0.252539
H	−0.745871	−0.334756	1.958946
H	−0.359071	1.361551	1.562276
H	1.450221	0.459216	2.814696
H	3.641436	−1.874918	1.351983
H	2.012227	1.612739	0.337475
H	2.161724	−0.406547	−0.880454
H	3.856772	−0.239920	−0.416718
N	2.357620	−2.844442	0.079884
C	3.121400	−3.999699	0.276790
O	4.067018	−4.077812	1.064205
N	2.737955	−5.062913	−0.524020
C	1.668579	−5.123117	−1.428990
O	1.457085	−6.169312	−2.064418
C	0.881858	−3.902943	−1.518882
C	−0.313314	−3.874403	−2.432215
C	1.255526	−2.840999	−0.760497
H	3.278726	−5.916519	−0.399895
H	−1.036595	−4.650354	−2.152789
H	−0.014817	−4.071282	−3.469164
H	−0.810388	−2.901150	−2.390556
H	0.687453	−1.915711	−0.751779
P	5.009807	1.517479	1.302223
O	5.131592	1.816909	−0.177339
O	5.581419	2.544448	2.261142
O	5.715524	0.058556	1.526429
C	5.878311	−0.450938	2.879600
C	7.087244	−1.370702	2.933028
O	6.839280	−2.605460	2.197401

C	7.677730	−2.685576	1.026274
C	8.372036	−0.767245	2.334562
C	8.415283	−1.343463	0.913840
O	9.475383	−1.197075	3.187189
H	4.976381	−1.001875	3.168104
H	6.029301	0.383154	3.573399
H	7.256131	−1.641319	3.980888
H	8.372131	−3.518564	1.149208
H	8.352384	0.325085	2.326118
H	7.907228	−0.643933	0.251485
H	9.433235	−1.469633	0.553692
N	6.855870	−3.055478	−0.151384
C	6.767299	−4.420676	−0.455277
O	7.341953	−5.305606	0.179721
N	5.989746	−4.703625	−1.567151
C	5.265652	−3.808092	−2.367403
O	4.593258	−4.232933	−3.317960
C	5.374902	−2.417262	−1.959133
C	4.673745	−1.376016	−2.786683
C	6.130475	−2.120879	−0.869684
H	5.924893	−5.690233	−1.810832
H	5.018077	−1.430302	−3.826311
H	4.873483	−0.371113	−2.407180
H	3.589851	−1.540477	−2.800201
H	6.198175	−1.106781	−0.497427
P	11.048361	−1.041978	2.747971
O	11.243604	0.016028	1.681946
O	11.834481	−0.876530	4.035250
O	11.370832	−2.484295	2.046506
C	11.240429	−3.679752	2.869640
C	11.611748	−4.903319	2.054926
O	10.623046	−5.096124	0.998824
C	11.299423	−5.280382	−0.265013
C	12.997416	−4.850385	1.369286
C	12.665271	−4.599733	−0.108267
O	13.626574	−6.140794	1.583535
H	10.205122	−3.769062	3.217515
H	11.904207	−3.602892	3.738480
H	11.581920	−5.771180	2.726425
H	11.404562	−6.345177	−0.480895
H	13.625003	−4.057124	1.787101
H	12.597414	−3.523268	−0.282411
H	13.414405	−5.018355	−0.784512
H	14.507753	−6.111610	1.167839
N	10.438380	−4.743921	−1.327804
C	9.757729	−5.648795	−2.150157
O	9.862959	−6.875426	−2.063859
N	8.938906	−5.044131	−3.088493
C	8.724865	−3.673097	−3.296787
O	7.958840	−3.296712	−4.196173
C	9.442418	−2.802961	−2.377465
C	9.278684	−1.315343	−2.519576
C	10.243387	−3.379326	−1.445648
H	8.453370	−5.681195	−3.717646
H	9.600726	−0.989064	−3.515799
H	9.869947	−0.780900	−1.772327
H	8.227233	−1.024341	−2.415578
H	10.784268	−2.784453	−0.720020
Na	12.777272	1.206668	3.108149
Na	6.339727	3.857924	0.363850

d(AAA)$^{H+}$

E: −27697.96 kcal mol^{-1}

O	0.203973	−0.236509	−0.712273
C	0.027736	0.072684	0.690159
C	1.356274	−0.048947	1.427260
O	1.780493	−1.441637	1.571637
C	2.937297	−1.723731	0.746718
C	2.533521	0.671579	0.750381
C	3.237053	−0.434326	−0.038355
O	3.347140	1.241126	1.844580
H	−0.678526	−0.282528	−1.119971
H	−0.692500	−0.614362	1.151487
H	−0.338341	1.102093	0.814401
H	1.209671	0.346993	2.437064
H	3.765651	−2.020362	1.397049
H	2.217813	1.501905	0.118091
H	2.792989	−0.471027	−1.033965
H	4.306491	−0.270189	−0.152186
N	2.677287	−2.883928	−0.102496
C	1.890238	−2.949403	−1.247908
N	1.803487	−4.172735	−1.744096
C	2.563052	−4.962445	−0.882293
C	2.846529	−6.348748	−0.862658
N	2.365019	−7.223232	−1.763692
N	3.647394	−6.806484	0.141232
C	4.112952	−5.946882	1.068088
N	3.888763	−4.625750	1.153262
C	3.108622	−4.183385	0.148511
H	1.389027	−2.073426	−1.634048
H	2.666007	−8.204989	−1.740825
H	1.862431	−6.876403	−2.569893
H	4.755968	−6.387336	1.827240
P	4.816416	1.863500	1.685810
O	5.015183	2.250159	0.142509
O	5.040619	2.931796	2.697492
O	5.825155	0.614471	1.758441
C	5.945840	−0.077663	3.049919
C	7.015137	−1.143466	2.933094
O	6.533684	−2.216998	2.082255
C	7.580867	−2.625859	1.169707
C	8.355743	−0.649619	2.332601
C	8.465023	−1.382516	0.991573
O	9.398007	−1.008877	3.306935
H	4.983993	−0.532263	3.305039
H	6.226757	0.647304	3.819506
H	7.203297	−1.534391	3.940627
H	8.141687	−3.473588	1.579206
H	8.384995	0.432921	2.211300
H	8.061332	−0.735328	0.209221
H	9.489561	−1.643043	0.729484
N	6.955671	−3.105544	−0.048805
C	6.220182	−2.362324	−0.967363
N	5.671003	−3.106248	−1.910366
C	6.057672	−4.410954	−1.606590
C	5.788167	−5.655889	−2.224682
N	5.012978	−5.803578	−3.308647
N	6.362615	−6.760137	−1.668548
C	7.113382	−6.643094	−0.559533
N	7.400497	−5.521439	0.121369
C	6.853575	−4.433911	−0.450588
H	6.134600	−1.288658	−0.886206
H	4.815627	−6.741074	−3.679775
H	4.570738	−4.993934	−3.721602
H	7.526271	−7.572706	−0.177634
P	10.974747	−0.797892	3.072012
O	11.133208	0.271103	1.888055
O	11.660372	−0.507411	4.360460
O	11.467514	−2.111614	2.295473
C	11.421243	−3.392405	3.018704
C	11.873808	−4.496591	2.088102
O	10.855095	−4.706747	1.067360
C	11.524553	−4.906033	−0.202018
C	13.213722	−4.231714	1.349762
C	12.784417	−4.037003	−0.111864
O	14.040007	−5.408987	1.534241
H	10.396397	−3.575983	3.354910
H	12.088645	−3.334353	3.883612
H	11.978115	−5.410474	2.687856
H	11.771473	−5.965321	−0.335595
H	13.727747	−3.351336	1.745776
H	12.531294	−2.986299	−0.281276
H	13.558156	−4.336325	−0.822670
H	14.906773	−5.226724	1.127058
N	10.608129	−4.566777	−1.272727
C	10.156708	−3.304563	−1.640052
N	9.319420	−3.331637	−2.661825
C	9.209781	−4.680733	−2.995059
C	8.489552	−5.357092	−4.007545
N	7.710199	−4.737294	−4.913045
N	8.617481	−6.712349	−4.064150
C	9.399526	−7.349407	−3.173470
N	10.124347	−6.807125	−2.181239
C	9.996855	−5.466627	−2.139421
H	10.485825	−2.413218	−1.126477
H	7.103102	−5.300735	−5.521215
H	7.514565	−3.750967	−4.797978
H	9.435659	−8.431367	−3.276034
O	5.992918	−11.868401	−10.518627
C	7.284584	−12.478432	−10.757062
C	7.894649	−12.938774	−9.438846
O	8.348145	−11.801911	−8.637135
C	7.619310	−11.714785	−7.390927
C	6.939546	−13.741560	−8.535959
C	6.463285	−12.723693	−7.499385
O	7.739681	−14.840761	−7.958500
H	5.697620	−11.460225	−11.351211
H	7.969006	−11.763785	−11.230340
H	7.179962	−13.354092	−11.413005
H	8.774302	−13.546664	−9.674246
H	8.285917	−11.938806	−6.556241
H	6.116934	−14.195146	−9.089043
H	5.559591	−12.246292	−7.882092
H	6.229355	−13.173600	−6.536348
N	7.187483	−10.322689	−7.179400
C	7.769569	−9.583595	−6.135627
O	8.605612	−10.049723	−5.355242
N	7.315667	−8.284922	−6.035543
C	6.410794	−7.642581	−6.873497
O	6.121032	−6.441999	−6.677805
C	5.874152	−8.450015	−7.956482
C	4.899445	−7.830687	−8.920264
C	6.300548	−9.734434	−8.065018
H	7.767522	−7.706540	−5.276618
H	5.332222	−6.942539	−9.396874
H	3.990005	−7.504708	−8.399809
H	4.617307	−8.544186	−9.699858
H	5.982772	−10.375043	−8.882075
P	7.226883	−15.865008	−6.835217
O	5.627088	−15.769269	−6.828757
O	7.834422	−17.210990	−7.020115
O	7.550221	−15.152241	−5.430246
C	8.962173	−15.000451	−5.052428
C	9.029354	−14.318188	−3.703187
O	8.569130	−12.953870	−3.844999
C	7.876654	−12.563834	−2.639510

C	8.172152	−14.974830	−2.586118
C	7.235723	−13.857433	−2.108177
O	9.120385	−15.455097	−1.569064
H	9.473261	−14.395205	−5.807062
H	9.427774	−15.988803	−4.995772
H	10.078315	−14.323321	−3.378246
H	8.563250	−12.121974	−1.913399
H	7.618274	−15.841710	−2.945265
H	6.249069	−14.009373	−2.550984
H	7.129332	−13.833953	−1.024951
N	6.927955	−11.507326	−2.993576
C	7.074498	−10.235379	−2.411716
O	7.907332	−9.984280	−1.538841
N	6.188288	−9.289649	−2.882320
C	5.209137	−9.463957	−3.854068
O	4.500388	−8.503509	−4.212463
C	5.095434	−10.808995	−4.400748
C	4.015965	−11.088771	−5.407400
C	5.965516	−11.749141	−3.955139
H	6.264255	−8.331412	−2.432965
H	3.030387	−10.875715	−4.976486
H	4.035221	−12.133367	−5.727227
H	4.124516	−10.445946	−6.288332
H	5.956536	−12.763207	−4.337183
P	8.686934	−16.120662	−0.169573
O	7.184779	−16.652641	−0.339395
O	9.721693	−17.091030	0.279305
O	8.394532	−14.894707	0.823726
C	9.521648	−14.019600	1.185352
C	9.005615	−12.871838	2.025927
O	8.171148	−12.019787	1.197218
C	7.087263	−11.522614	2.018589
C	8.166916	−13.264193	3.274928
C	6.776524	−12.670868	2.988859
O	8.813409	−12.658494	4.422689
H	9.983877	−13.637558	0.270042
H	10.254844	−14.600244	1.752849
H	9.880944	−12.302250	2.368838
H	7.392641	−10.617494	2.548387
H	8.122635	−14.348940	3.407176
H	6.146663	−13.424856	2.508331
H	6.276735	−12.326435	3.897322
H	8.353314	−12.976261	5.221248
N	5.997983	−11.123595	1.128117
C	5.642040	−9.766389	1.051395
O	6.157174	−8.889290	1.751658
N	4.653653	−9.488600	0.130046
C	3.991265	−10.397859	−0.690178
O	3.108082	−10.003978	−1.480336
C	4.401827	−11.785998	−0.557938
C	3.712198	−12.837332	−1.382392
C	5.379138	−12.071389	0.338231
H	4.322687	−8.487266	0.107964
H	2.642470	−12.875893	−1.143495
H	4.141532	−13.825019	−1.194209
H	3.792622	−12.612417	−2.451179
H	5.732474	−13.084112	0.485918
H	5.198225	−16.628207	−7.009387
H	7.136433	−17.620339	−0.465387
H	11.481051	1.129923	2.197989
H	4.960944	3.212895	−0.015408

d(AAC)$^{H+}$

E: −27609.26 kcal mol^{-1}

O	0.206150	−0.227159	−0.675799
C	0.064502	0.099656	0.726541
C	1.407346	−0.030759	1.435362
O	1.820093	−1.427763	1.575733
C	2.955631	−1.723074	0.727093
C	2.576817	0.674951	0.728963
C	3.241847	−0.442250	−0.075119
O	3.421473	1.241897	1.801180
H	−0.685578	−0.266875	−1.063498
H	−0.653297	−0.573843	1.211211
H	−0.286569	1.134686	0.847694
H	1.286989	0.370763	2.446414
H	3.796606	−2.019460	1.361733
H	2.256050	1.508002	0.102790
H	2.757526	−0.479327	−1.051622
H	4.306086	−0.290504	−0.237745
N	2.674545	−2.886460	−0.110094
C	1.886533	−2.949792	−1.255011
N	1.812675	−4.169289	−1.762474
C	2.583029	−4.957950	−0.909213
C	2.885875	−6.340261	−0.906285
N	2.426996	−7.206245	−1.825755
N	3.685173	−6.800742	0.098137
C	4.138434	−5.945537	1.035168
N	3.903341	−4.626931	1.130657
C	3.119978	−4.183254	0.128907
H	1.374965	−2.076061	−1.632540
H	2.709257	−8.193352	−1.794627
H	1.904569	−6.859247	−2.619057
H	4.780232	−6.387790	1.794419
P	4.932210	1.752087	1.641533
O	5.182420	2.062537	0.088854
O	5.219756	2.841690	2.613370
O	5.848007	0.437262	1.781749
C	5.910021	−0.192516	3.110638
C	7.008543	−1.236234	3.110579
O	6.606356	−2.371703	2.299477
C	7.654433	−2.722746	1.364424
C	8.363409	−0.736264	2.558368
C	8.520561	−1.461022	1.218790
O	9.378475	−1.102665	3.560179
H	4.944376	−0.657073	3.329754
H	6.129301	0.573851	3.859917
H	7.145081	−1.567692	4.146846
H	8.229691	−3.576398	1.735797
H	8.392119	0.347310	2.445547
H	8.128447	−0.814963	0.429969
H	9.555690	−1.707292	0.985327
N	7.030502	−3.163636	0.127645
C	6.287547	−2.384158	−0.754655
N	5.748883	−3.085632	−1.734574
C	6.149290	−4.399661	−1.494814
C	5.899494	−5.612698	−2.180860
N	5.157723	−5.703186	−3.293344
N	6.456273	−6.744398	−1.663829
C	7.191238	−6.678740	−0.540694
N	7.481501	−5.587905	0.188366
C	6.939485	−4.472728	−0.336404
H	6.188458	−1.317536	−0.616265
H	4.951071	−6.623271	−3.699914
H	4.725794	−4.873162	−3.675560
H	7.586278	−7.628791	−0.189889
P	10.944743	−0.767481	3.407500
O	11.074896	0.480079	2.409179
O	11.572441	−0.647793	4.751336
O	11.542129	−1.901597	2.444341
C	11.537097	−3.290303	2.930529
C	11.848245	−4.221293	1.782051
O	10.745264	−4.224982	0.841930
C	11.312389	−4.679876	−0.412433
C	13.135829	−3.906107	0.964147

C	12.656222	−3.939575	−0.503257
O	14.100465	−4.937301	1.284538
H	10.553745	−3.522729	3.349949
H	12.299276	−3.392098	3.708425
H	11.966323	−5.230074	2.206911
H	11.449218	−5.763322	−0.391079
H	13.546575	−2.927089	1.224469
H	12.503347	−2.921176	−0.872802
H	13.370081	−4.446112	−1.157362
H	14.960665	−4.661579	0.918990
N	10.360448	−4.413757	−1.485999
C	9.879739	−5.492738	−2.283884
O	10.268089	−6.656190	−2.034680
N	9.021964	−5.210346	−3.295625
C	8.612686	−3.947414	−3.535077
N	7.760667	−3.747576	−4.548827
C	9.072719	−2.850201	−2.733173
C	9.944282	−3.131278	−1.733461
H	7.417288	−4.539455	−5.113685
H	7.404978	−2.818374	−4.731075
H	8.739522	−1.836494	−2.918207
H	10.345681	−2.368736	−1.078193
O	6.038167	−11.866572	−10.738073
C	7.285513	−12.579461	−10.913865
C	7.799263	−13.084417	−9.570310
O	8.308809	−11.996240	−8.733350
C	7.452713	−11.777174	−7.586503
C	6.747383	−13.805088	−8.711474
C	6.245356	−12.713146	−7.768840
O	7.460229	−14.907014	−8.032056
H	5.797197	−11.474452	−11.595686
H	8.047365	−11.925217	−11.355827
H	7.142095	−13.447926	−11.572504
H	8.639893	−13.756239	−9.769796
H	8.016259	−11.995813	−6.674012
H	5.948150	−14.254879	−9.301154
H	5.428012	−12.191561	−8.269396
H	5.873412	−13.093695	−6.820142
N	7.095143	−10.366040	−7.491218
C	6.281681	−9.629975	−8.358043
N	6.195524	−8.359736	−8.011209
C	6.989744	−8.237019	−6.868795
C	7.293922	−7.103466	−6.057346
O	6.887556	−5.924482	−6.201223
N	8.159182	−7.430003	−4.996033
C	8.701242	−8.681287	−4.772840
N	9.568023	−8.801652	−3.748199
N	8.410699	−9.747452	−5.526870
C	7.568012	−9.471314	−6.539235
H	5.827715	−10.094197	−9.223117
H	8.441904	−6.643752	−4.366225
H	9.749929	−8.025982	−3.094005
H	9.864197	−9.737310	−3.502188
P	6.929984	−15.746745	−6.776267
O	5.339397	−15.557964	−6.722447
O	7.453989	−17.139134	−6.808349
O	7.346909	−14.886841	−5.480115
C	8.779798	−14.819386	−5.149429
C	8.933908	−14.287035	−3.739329
O	8.583489	−12.878129	−3.710839
C	7.702594	−12.608581	−2.596621
C	8.050389	−14.998610	−2.685088
C	7.011349	−13.945693	−2.291133
O	8.948692	−15.388234	−1.587671
H	9.277159	−14.156821	−5.863416
H	9.211230	−15.822413	−5.213987
H	9.987123	−14.401211	−3.456129
H	8.265000	−12.237938	−1.736337
H	7.590994	−15.908352	−3.070869
H	6.127335	−14.084318	−2.917286
H	6.708040	−14.018874	−1.248241
N	6.809265	−11.514313	−2.986003
C	6.943092	−10.273381	−2.334452
O	7.693502	−10.093084	−1.374279
N	6.152100	−9.270920	−2.854028
C	5.248110	−9.373255	−3.904733
O	4.618468	−8.368168	−4.289491
C	5.117237	−10.694747	−4.500964
C	4.101091	−10.891910	−5.589536
C	5.924145	−11.680767	−4.036538
H	6.259391	−8.319498	−2.395866
H	3.106049	−10.597886	−5.234831
H	4.057324	−11.936367	−5.906243
H	4.326292	−10.265699	−6.460155
H	5.920034	−12.673497	−4.471300
P	8.481325	−16.101577	−0.224769
O	6.985288	−16.618708	−0.473728
O	9.489228	−17.099750	0.225603
O	8.191616	−14.900486	0.800438
C	9.342258	−14.090498	1.234578
C	8.851975	−12.965231	2.119603
O	8.068368	−12.044492	1.315134
C	6.940242	−11.593471	2.101627
C	7.974141	−13.383590	3.330549
C	6.596237	−12.777933	3.015723
O	8.586339	−12.806437	4.511716
H	9.841641	−13.681822	0.350844
H	10.037237	−14.728660	1.788517
H	9.741013	−12.446379	2.504835
H	7.208388	−10.704192	2.676267
H	7.922091	−14.471153	3.436509
H	5.985693	−13.515098	2.487108
H	6.063075	−12.466647	3.917082
H	8.092472	−13.129907	5.287372
N	5.886428	−11.174033	1.176906
C	5.563791	−9.809085	1.088026
O	6.078071	−8.941681	1.800832
N	4.606029	−9.510404	0.140996
C	3.956485	−10.403671	−0.704744
O	3.106860	−9.991922	−1.523088
C	4.343037	−11.798352	−0.571486
C	3.667658	−12.828130	−1.434372
C	5.288030	−12.104999	0.352533
H	4.305573	−8.499626	0.102863
H	2.587997	−12.843283	−1.242810
H	4.065364	−13.826891	−1.237669
H	3.798600	−12.596316	−2.497113
H	5.631942	−13.122081	0.494779
H	4.856858	−16.400194	−6.834042
H	6.900115	−17.587526	−0.382848
H	11.229113	1.324998	2.874915
H	5.202489	3.019685	−0.106521

d(AAG)$^{H+}$

E: −27608.28 kcal mol^{-1}

O	0.274208	−0.270086	−0.717208
C	0.072233	0.022932	0.685226
C	1.391339	−0.084585	1.440824
O	1.835776	−1.471228	1.579514
C	3.008765	−1.728896	0.769623
C	2.566574	0.662377	0.789128
C	3.307545	−0.424874	0.007416
O	3.345751	1.246279	1.901225
H	−0.601372	−0.328145	−1.138059
H	−0.643863	−0.679613	1.129499
H	−0.311763	1.045353	0.813543

H	1.223255	0.299934	2.451734
H	3.830130	−2.025701	1.428318
H	2.245661	1.488647	0.154213
H	2.891377	−0.456652	−1.000310
H	4.377095	−0.242982	−0.075583
N	2.777033	−2.880037	−0.100858
C	2.022840	−2.937115	−1.268802
N	1.964370	−4.153594	−1.785839
C	2.709281	−4.947752	−0.915145
C	3.008060	−6.331195	−0.907523
N	2.565002	−7.197105	−1.835548
N	3.783686	−6.795611	0.113412
C	4.210710	−5.945385	1.066822
N	3.969210	−4.628265	1.164648
C	3.215807	−4.178569	0.142862
H	1.522142	−2.061356	−1.655920
H	2.853167	−8.182156	−1.802638
H	2.072522	−6.847653	−2.646731
H	4.834014	−6.390702	1.839496
P	4.809635	1.889874	1.777334
O	5.035037	2.298935	0.243425
O	4.998914	2.948356	2.806479
O	5.832189	0.652837	1.852802
C	5.903283	−0.083873	3.123008
C	6.935109	−1.183092	2.989708
O	6.442798	−2.195959	2.077365
C	7.538256	−2.675660	1.261176
C	8.314677	−0.716824	2.454766
C	8.494562	−1.478558	1.135967
O	9.292801	−1.064603	3.498068
H	4.921074	−0.509666	3.347909
H	6.196453	0.605080	3.920670
H	7.079442	−1.624058	3.984332
H	8.023517	−3.540816	1.725997
H	8.362131	0.362394	2.312272
H	8.180784	−0.830680	0.313451
H	9.522326	−1.795631	0.961304
N	6.984055	−3.145102	0.005735
C	6.271889	−2.392730	−0.923190
N	5.774847	−3.123099	−1.904609
C	6.176233	−4.426572	−1.618091
C	5.956752	−5.659159	−2.279117
N	5.234432	−5.788847	−3.401537
N	6.523289	−6.769486	−1.725874
C	7.220277	−6.668342	−0.579560
N	7.462049	−5.559871	0.137873
C	6.924508	−4.465177	−0.431373
H	6.162945	−1.323357	−0.817519
H	5.029552	−6.723659	−3.776703
H	4.795094	−4.973942	−3.807470
H	7.623740	−7.602865	−0.198180
P	10.870408	−0.775129	3.378397
O	11.061061	0.442008	2.352182
O	11.464896	−0.636790	4.735605
O	11.461546	−1.949239	2.462026
C	11.427342	−3.320309	2.995659
C	11.804975	−4.285393	1.895327
O	10.728568	−4.349547	0.919551
C	11.351377	−4.633700	−0.358121
C	13.109664	−3.944836	1.115425
C	12.629893	−3.785726	−0.337901
O	14.012477	−5.064707	1.293778
H	10.420651	−3.540203	3.363545
H	12.143038	−3.393865	3.819528
H	11.935003	−5.274427	2.358127
H	11.580587	−5.703203	−0.434951
H	13.578252	−3.030799	1.489811
H	12.383137	−2.738042	−0.536129
H	13.375189	−4.119732	−1.063862

H	14.872480	−4.813363	0.910200
N	10.419813	−4.344787	−1.426330
C	9.944553	−3.098714	−1.841264
N	9.178640	−3.178533	−2.910741
C	9.143091	−4.536439	−3.233644
C	8.509355	−5.226788	−4.309238
O	7.798906	−4.741006	−5.222695
N	8.757821	−6.611068	−4.266413
C	9.536808	−7.250020	−3.323312
N	9.716469	−8.578036	−3.474848
N	10.128124	−6.605499	−2.309417
C	9.904874	−5.278039	−2.318242
H	10.199844	−2.189981	−1.315870
H	8.285981	−7.196077	−4.995492
H	9.232655	−9.108827	−4.215707
H	10.086910	−9.084870	−2.680947
O	5.978642	−12.016336	−10.685264
C	7.255718	−12.660212	−10.911724
C	7.878853	−13.058717	−9.580153
O	8.341326	−11.884440	−8.839687
C	7.632909	−11.746463	−7.584770
C	6.930470	−13.813363	−8.630390
C	6.463046	−12.742169	−7.646954
O	7.737455	−14.877328	−7.998995
H	5.675381	−11.647823	−11.533477
H	7.943538	−11.983468	−11.433330
H	7.126694	−13.568092	−11.517634
H	8.753997	−13.681052	−9.794338
H	8.308435	−11.952369	−6.753262
H	6.105146	−14.297913	−9.152228
H	5.569475	−12.270202	−8.059643
H	6.215827	−13.140257	−6.664673
N	7.222803	−10.342612	−7.414097
C	7.787083	−9.563143	−6.364395
O	8.585853	−10.100747	−5.565578
N	7.423583	−8.258098	−6.263026
C	6.552843	−7.705908	−7.131297
N	6.230853	−6.414183	−6.965468
C	5.989930	−8.469489	−8.207676
C	6.366293	−9.768156	−8.320956
H	6.734973	−5.830782	−6.281309
H	5.627029	−5.961475	−7.639689
H	5.311337	−8.019832	−8.923194
H	6.034808	−10.409193	−9.131891
P	7.232932	−15.842324	−6.822815
O	5.634414	−15.733475	−6.806769
O	7.827701	−17.200853	−6.947480
O	7.581169	−15.066886	−5.455737
C	8.999640	−14.943759	−5.088339
C	9.097537	−14.338137	−3.703637
O	8.695087	−12.946132	−3.759667
C	7.867460	−12.632645	−2.617023
C	8.218393	−15.025286	−2.626322
C	7.190272	−13.958346	−2.237504
O	9.129739	−15.407032	−1.536430
H	9.502653	−14.301494	−5.817238
H	9.458950	−15.936574	−5.091985
H	10.147028	−14.401115	−3.389745
H	8.467222	−12.233997	−1.795077
H	7.747048	−15.937438	−2.991323
H	6.285735	−14.118249	−2.828582
H	6.921758	−13.992128	−1.182971
N	6.958617	−11.552808	−3.006537
C	7.090490	−10.298942	−2.379955
O	7.855905	−10.095798	−1.435942
N	6.277967	−9.314692	−2.901109
C	5.343088	−9.449765	−3.921602
O	4.674958	−8.467186	−4.297631
C	5.219430	−10.783190	−4.492967

C	4.170880	−11.019462	−5.542808
C	6.050113	−11.749929	−4.030062
H	6.371775	−8.359085	−2.451405
H	3.178106	−10.774688	−5.146371
H	4.163876	−12.062503	−5.867208
H	4.331386	−10.376387	−6.415283
H	6.043649	−12.752957	−4.440123
P	8.673662	−16.074347	−0.146422
O	7.173386	−16.597837	−0.354860
O	9.688210	−17.056479	0.323579
O	8.388606	−14.842024	0.842192
C	9.537725	−14.011178	1.239772
C	9.051750	−12.877488	2.116816
O	8.248463	−11.972170	1.313170
C	7.117353	−11.536704	2.105088
C	8.194754	−13.287300	3.345083
C	6.800702	−12.722740	3.026142
O	8.800037	−12.662871	4.505780
H	10.014297	−13.611350	0.339620
H	10.251382	−14.631507	1.790219
H	9.942295	−12.346691	2.481431
H	7.375109	−10.641830	2.675782
H	8.171348	−14.372692	3.479498
H	6.211761	−13.480227	2.501639
H	6.258292	−12.419325	3.924772
H	8.316240	−12.972901	5.293276
N	6.052328	−11.134973	1.185769
C	5.715286	−9.773830	1.091908
O	6.223914	−8.898159	1.798963
N	4.752544	−9.488634	0.146325
C	4.105578	−10.392360	−0.690823
O	3.249208	−9.991259	−1.507006
C	4.502592	−11.783518	−0.548527
C	3.826568	−12.826218	−1.395264
C	5.455645	−12.076287	0.371779
H	4.436211	−8.482831	0.109363
H	2.747706	−12.840815	−1.199143
H	4.227131	−13.821363	−1.185927
H	3.952777	−12.609141	−2.461463
H	5.806288	−13.090029	0.520595
H	5.197080	−16.602950	−6.891409
H	7.104795	−17.571618	−0.316841
H	11.199848	1.298882	2.800617
H	4.946378	3.260129	0.092110

d(AAT)$^{H+}$

E: −27699.12 kcal mol^{-1}

O	0.047239	−0.141249	−0.523605
C	0.044508	0.249810	0.869568
C	1.435274	0.079483	1.469515
O	1.789997	−1.332553	1.631698
C	2.845698	−1.709863	0.716718
C	2.575808	0.696617	0.641674
C	3.098700	−0.482931	−0.175357
O	3.537156	1.252062	1.619933
H	−0.875258	−0.142168	−0.833665
H	−0.659873	−0.365481	1.443151
H	−0.245249	1.305297	0.973093
H	1.419477	0.523910	2.469563
H	3.727570	−1.999142	1.297020
H	2.249168	1.529202	0.017907
H	2.495459	−0.540142	−1.082530
H	4.138216	−0.391113	−0.478529
N	2.469798	−2.904322	−0.032657
C	1.566462	−2.997521	−1.086402
N	1.475403	−4.221892	−1.578912
C	2.359215	−4.980641	−0.812617
C	2.717622	−6.348887	−0.853986
N	2.200121	−7.226043	−1.730537
N	3.637143	−6.780451	0.056069
C	4.149469	−5.909857	0.948615
N	3.876207	−4.601217	1.075655
C	2.978007	−4.185447	0.162074
H	0.993670	−2.139040	−1.408792
H	2.532717	−8.198366	−1.750411
H	1.575336	−6.894711	−2.453598
H	4.881598	−6.329057	1.636132
P	5.095549	1.541642	1.403835
O	5.363146	1.712140	−0.166882
O	5.547471	2.639474	2.300356
O	5.834684	0.127005	1.619684
C	5.888313	−0.402074	2.993650
C	7.082852	−1.330184	3.137527
O	6.855842	−2.596422	2.460859
C	7.788712	−2.784210	1.369898
C	8.395462	−0.758530	2.571300
C	8.553691	−1.456353	1.217049
O	9.447349	−1.102239	3.541739
H	4.959644	−0.942434	3.196234
H	5.991277	0.431319	3.694546
H	7.199374	−1.537672	4.206641
H	8.454061	−3.620344	1.600268
H	8.373985	0.327581	2.478200
H	8.097144	−0.827763	0.450592
H	9.595274	−1.626711	0.948317
N	7.054637	−3.181781	0.173755
C	6.200478	−2.393961	−0.591133
N	5.605397	−3.066034	−1.559604
C	6.078030	−4.371468	−1.431275
C	5.804135	−5.563389	−2.145280
N	4.959749	−5.644231	−3.184956
N	6.444795	−6.692539	−1.732636
C	7.276694	−6.651540	−0.678082
N	7.595049	−5.583322	0.072207
C	6.970492	−4.468163	−0.350203
H	6.069196	−1.344361	−0.375336
H	4.759876	−6.555534	−3.612419
H	4.459292	−4.821559	−3.492089
H	7.736865	−7.600370	−0.415356
P	11.001214	−0.731470	3.350086
O	11.075973	0.505182	2.332613
O	11.655530	−0.579587	4.677794
O	11.606863	−1.863598	2.389346
C	11.675011	−3.238705	2.908999
C	12.012522	−4.181395	1.777241
O	10.898484	−4.245800	0.851132
C	11.463142	−4.632202	−0.426302
C	13.277885	−3.828776	0.940512
C	12.771700	−3.833310	−0.517095
O	14.261154	−4.856568	1.212828
H	10.709931	−3.506196	3.349920
H	12.453401	−3.286050	3.675971
H	12.174440	−5.175121	2.221190
H	11.647070	−5.709247	−0.447371
H	13.682660	−2.851652	1.217110
H	12.572448	−2.809978	−0.848012
H	13.492098	−4.288171	−1.201030
H	15.106300	−4.567377	0.823187
N	10.475196	−4.371393	−1.468357
C	9.994967	−5.451835	−2.228249
O	10.379672	−6.615216	−2.073004
N	9.057924	−5.106271	−3.176862
C	8.582651	−3.830972	−3.466670
O	7.755197	−3.669041	−4.388588
C	9.111840	−2.757534	−2.639068

C	8.654705	−1.345097	−2.878996
C	10.026429	−3.084099	−1.691503
H	8.712725	−5.903919	−3.777997
H	8.956281	−1.003539	−3.877113
H	9.085996	−0.665567	−2.138279
H	7.563025	−1.275837	−2.830887
H	10.461120	−2.334572	−1.041631
O	6.119750	−11.780114	−10.475061
C	7.408173	−12.388707	−10.728832
C	7.998695	−12.934878	−9.433731
O	8.492794	−11.872173	−8.557018
C	7.679306	−11.746856	−7.368489
C	7.016548	−13.755426	−8.581617
C	6.535820	−12.771253	−7.511256
O	7.790001	−14.894285	−8.046174
H	5.822489	−11.357659	−11.299926
H	8.106851	−11.657083	−11.153374
H	7.303371	−13.226126	−11.433303
H	8.861735	−13.552215	−9.701742
H	8.300235	−11.935440	−6.488322
H	6.194036	−14.168559	−9.165691
H	5.627309	−12.288701	−7.875121
H	6.309786	−13.253070	−6.561319
N	7.214942	−10.365331	−7.228948
C	6.305344	−9.698349	−8.044557
N	6.150885	−8.427691	−7.711196
C	7.005016	−8.235861	−6.627065
C	7.308584	−7.094111	−5.846845
N	6.762696	−5.882913	−6.046608
N	8.221648	−7.248722	−4.846884
C	8.800945	−8.447815	−4.649790
N	8.595472	−9.584916	−5.335287
C	7.685261	−9.423643	−6.316408
H	5.832416	−10.201658	−8.877096
H	7.025700	−5.094819	−5.445049
H	6.057979	−5.758976	−6.761131
H	9.509296	−8.487650	−3.824806
P	7.238837	−15.958721	−6.978280
O	5.637512	−15.889244	−7.014059
O	7.877113	−17.286612	−7.187287
O	7.478746	−15.285993	−5.539954
C	8.865560	−15.057862	−5.114822
C	8.849691	−14.289852	−3.812828
O	8.331985	−12.961213	−4.048585
C	7.772291	−12.486926	−2.803630
C	7.979226	−14.908167	−2.683847
C	7.149571	−13.731205	−2.144753
O	8.916264	−15.500643	−1.719484
H	9.392284	−14.487555	−5.885342
H	9.359844	−16.023087	−4.970923
H	9.885005	−14.226686	−3.448746
H	8.543560	−12.039531	−2.172130
H	7.343248	−15.712680	−3.051999
H	6.109545	−13.847969	−2.455841
H	7.183708	−13.657929	−1.058846
N	6.830231	−11.414757	−3.117191
C	7.005969	−10.159437	−2.511861
O	7.856486	−9.942449	−1.645980
N	6.134841	−9.189208	−2.955748
C	5.142748	−9.323417	−3.920366
O	4.461049	−8.334995	−4.256072
C	4.982116	−10.661933	−4.474569
C	3.890294	−10.909314	−5.475844
C	5.832753	−11.629127	−4.050428
H	6.254012	−8.232955	−2.509122
H	2.910034	−10.684150	−5.038399
H	3.891189	−11.950807	−5.808714
H	4.008442	−10.258800	−6.349893
H	5.786994	−12.641347	−4.434568
P	8.466278	−16.191356	−0.337186
O	6.943525	−16.660703	−0.510155
O	9.467609	−17.213409	0.070492
O	8.223237	−14.989778	0.697125
C	9.372683	−14.147870	1.065589
C	8.879377	−12.981963	1.895012
O	8.062215	−12.121936	1.057785
C	7.008044	−11.570725	1.884504
C	8.032595	−13.345926	3.147533
C	6.665800	−12.691821	2.875967
O	8.714702	−12.779927	4.294609
H	9.856294	−13.784358	0.154021
H	10.081960	−14.747296	1.643673
H	9.765189	−12.426428	2.234195
H	7.355200	−10.670027	2.395831
H	7.941293	−14.428601	3.271736
H	5.992714	−13.421818	2.417712
H	6.199293	−12.313171	3.788434
H	8.255442	−13.094213	5.094894
N	5.923279	−11.144539	1.001305
C	5.612384	−9.776003	0.912014
O	6.167128	−8.908138	1.592316
N	4.617667	−9.477401	0.003061
C	3.915944	−10.375071	−0.797171
O	3.033002	−9.964998	−1.579256
C	4.284202	−11.773536	−0.655932
C	3.550975	−12.808432	−1.463638
C	5.261581	−12.081162	0.232579
H	4.311843	−8.468346	−0.016669
H	2.479397	−12.791131	−1.231299
H	3.932146	−13.811398	−1.253295
H	3.647144	−12.609254	−2.536756
H	5.582012	−13.103624	0.387952
H	5.234704	−16.658513	−7.461750
H	6.859192	−17.616791	−0.693109
H	11.209826	1.361388	2.783857
H	5.475982	2.645356	−0.433546

d(ACA)$^{H+}$

E: −27609.22 kcal mol^{-1}

O	0.328741	−0.221893	−0.079231
C	0.216093	−0.062934	1.355101
C	1.575076	−0.265488	2.015101
O	1.974232	−1.672275	2.037359
C	3.096925	−1.914164	1.157236
C	2.728656	0.484092	1.330638
C	3.404164	−0.576374	0.457110
O	3.599116	0.985403	2.414320
H	−0.568760	−0.184450	−0.453897
H	−0.490374	−0.791138	1.772799
H	−0.134174	0.949586	1.601421
H	1.487232	0.058434	3.056894
H	3.942553	−2.274378	1.748705
H	2.388218	1.345297	0.755967
H	2.953591	−0.544455	−0.536259
H	4.477901	−0.421013	0.360134
N	2.772499	−3.002145	0.235286
C	1.841575	−2.978591	−0.799352
N	1.688919	−4.156355	−1.381143
C	2.553133	−5.008804	−0.695724
C	2.832076	−6.390023	−0.828938
N	2.246506	−7.186185	−1.739509
N	3.738082	−6.929675	0.034478
C	4.324169	−6.148323	0.960687
N	4.128365	−4.836889	1.176326
C	3.229204	−4.314755	0.319775

H	1.296324	−2.074999	−1.036029
H	2.518807	−8.172635	−1.813014
H	1.632489	−6.782750	−2.434255
H	5.042092	−6.653787	1.603343
P	4.891641	1.905341	2.163163
O	4.675446	2.672588	0.771607
O	5.178321	2.723263	3.372744
O	6.061312	0.913172	1.691828
C	6.615900	−0.023755	2.679617
C	7.472287	−1.040055	1.956535
O	6.627582	−1.969021	1.219245
C	7.338228	−2.260234	−0.021322
C	8.462393	−0.435097	0.914501
C	7.898729	−0.895401	−0.427738
O	9.782325	−1.077314	1.055234
H	5.800817	−0.527329	3.207895
H	7.226854	0.538260	3.391319
H	8.041236	−1.589545	2.716005
H	8.149413	−2.959150	0.189574
H	8.574428	0.645322	1.002446
H	7.103279	−0.208744	−0.728353
H	8.652723	−0.949010	−1.212849
N	6.464449	−2.954419	−0.946573
C	6.553128	−4.383357	−0.987917
O	7.307247	−4.958960	−0.177448
N	5.826900	−5.045892	−1.917934
C	5.010865	−4.390700	−2.768321
N	4.357439	−5.108259	−3.687472
C	4.851155	−2.965203	−2.694090
C	5.600703	−2.295230	−1.783491
H	4.502318	−6.129022	−3.766555
H	3.744163	−4.643584	−4.343986
H	4.173270	−2.438469	−3.354598
H	5.556692	−1.218691	−1.683124
P	10.846181	−0.575435	2.142157
O	11.658347	0.539902	1.321087
O	10.270392	−0.145253	3.447236
O	11.873020	−1.798460	2.173766
C	11.613192	−2.965777	3.036654
C	12.062670	−4.236819	2.336448
O	11.095088	−4.603747	1.308247
C	11.792240	−4.882926	0.067562
C	13.444781	−4.156843	1.643008
C	13.089144	−4.067959	0.152091
O	14.153019	−5.379903	1.968871
H	10.547440	−3.027348	3.273116
H	12.183018	−2.822213	3.958643
H	12.087857	−5.031042	3.093733
H	11.988458	−5.956855	−0.021861
H	14.021463	−3.291010	1.981292
H	12.898250	−3.025530	−0.118801
H	13.876452	−4.464228	−0.493404
H	15.035877	−5.326938	1.558904
N	10.913347	−4.537648	−1.036070
C	10.425927	−3.274702	−1.346210
N	9.572075	−3.284057	−2.355685
C	9.483055	−4.622327	−2.732763
C	8.719686	−5.278936	−3.725928
N	7.884699	−4.641974	−4.566492
N	8.860894	−6.631753	−3.828977
C	9.704019	−7.277738	−3.001258
N	10.463718	−6.755285	−2.023710
C	10.307392	−5.420822	−1.925009
H	10.711878	−2.403855	−0.774251
H	7.284185	−5.182826	−5.204015
H	7.721177	−3.651457	−4.442530
H	9.761310	−8.354913	−3.146995
O	5.856943	−11.638386	−10.385341
C	7.101946	−12.333116	−10.637225
C	7.726978	−12.786397	−9.323353
O	8.231444	−11.647513	−8.554352
C	7.517317	−11.521976	−7.296584
C	6.772572	−13.535013	−8.375208
C	6.299466	−12.448883	−7.415461
O	7.593551	−14.583232	−7.725897
H	5.568272	−11.233414	−11.221778
H	7.813668	−11.675921	−11.152093
H	6.928153	−13.221354	−11.261215
H	8.581006	−13.426827	−9.567207
H	8.174665	−11.809610	−6.474256
H	5.957985	−14.041314	−8.893577
H	5.458746	−11.935254	−7.885718
H	5.962652	−12.823727	−6.453164
N	7.195368	−10.111415	−7.059453
C	7.861534	−9.433462	−6.024081
O	8.715385	−9.962303	−5.305977
N	7.479063	−8.116428	−5.869873
C	6.568028	−7.409646	−6.649144
O	6.348349	−6.200896	−6.418528
C	5.944374	−8.159024	−7.726977
C	4.962969	−7.463086	−8.630053
C	6.299107	−9.459201	−7.886649
H	7.971802	−7.589371	−5.104725
H	5.426531	−6.598125	−9.120520
H	4.106817	−7.086420	−8.056690
H	4.592943	−8.145303	−9.400955
H	5.914461	−10.061439	−8.703705
P	7.242589	−15.443509	−6.429748
O	5.654568	−15.308849	−6.262944
O	7.813358	−16.815518	−6.508491
O	7.764935	−14.583481	−5.168171
C	9.213828	−14.518685	−4.916029
C	9.477134	−14.191446	−3.453919
O	9.191184	−12.801052	−3.145302
C	8.068956	−12.679702	−2.235956
C	8.663616	−15.034617	−2.455976
C	7.525709	−14.105687	−2.019347
O	9.592525	−15.389681	−1.368991
H	9.643977	−13.744772	−5.557831
H	9.660995	−15.488325	−5.153899
H	10.547693	−14.352490	−3.286745
H	8.414281	−12.222696	−1.305005
H	8.296208	−15.964266	−2.891392
H	6.661370	−14.296339	−2.657852
H	7.227079	−14.261677	−0.983135
N	7.087802	−11.756255	−2.790755
C	6.242330	−11.975976	−3.879552
N	5.550590	−10.905813	−4.211566
C	5.954194	−9.917913	−3.312504
C	5.569568	−8.549183	−3.195320
O	4.751937	−7.917739	−3.903376
N	6.238904	−7.906335	−2.137948
C	7.173485	−8.496057	−1.309957
N	7.701420	−7.728878	−0.341764
N	7.549082	−9.777533	−1.430052
C	6.920180	−10.424282	−2.428462
H	6.200213	−12.938348	−4.364711
H	6.046142	−6.884528	−2.020067
H	7.533351	−6.712154	−0.295620
H	8.442907	−8.132979	0.214384
P	9.175416	−16.241740	−0.070634
O	7.866560	−17.085039	−0.454506
O	10.353270	−16.985687	0.452440
O	8.493708	−15.200131	0.939208
C	9.348819	−14.167731	1.543914
C	8.476139	−13.175227	2.276402
O	7.722341	−12.393316	1.313355
C	6.513083	−11.976664	1.998286

C	7.445657	−13.779411	3.276851
C	6.092275	−13.217847	2.795861
O	7.820086	−13.316384	4.597641
H	9.918463	−13.663442	0.757304
H	10.037639	−14.647834	2.244994
H	9.142742	−12.508688	2.843245
H	6.729609	−11.134970	2.660870
H	7.455620	−14.872279	3.254410
H	5.598508	−13.946765	2.146938
H	5.425168	−12.977959	3.627212
H	7.274383	−13.799021	5.245027
N	5.566318	−11.475650	1.009832
C	5.245259	−10.106210	1.026514
O	5.695846	−9.314456	1.859487
N	4.368012	−9.715522	0.037615
C	3.751990	−10.528207	−0.909970
O	2.928624	−10.037121	−1.710560
C	4.136339	−11.931307	−0.883047
C	3.526084	−12.883041	−1.875033
C	5.021872	−12.325540	0.066017
H	4.125967	−8.688169	0.032483
H	2.446842	−12.983133	−1.703804
H	3.980665	−13.874592	−1.791389
H	3.660656	−12.516927	−2.897319
H	5.353377	−13.354305	0.135841
H	5.240155	−16.093521	−5.854067
H	8.062418	−18.019455	−0.661846
H	12.177185	1.137483	1.895199
H	4.326235	3.577905	0.885283

d(ACC)$^{H+}$

E: −27520.21 kcal mol^{-1}

O	0.110489	−0.129830	−0.169173
C	−0.028545	0.075692	1.256506
C	1.330644	−0.045518	1.935028
O	1.783680	−1.433537	2.013145
C	2.926817	−1.669189	1.161343
C	2.456753	0.731322	1.231722
C	3.235408	−0.333371	0.452340
O	3.249178	1.382649	2.295066
H	−0.782633	−0.172359	−0.553450
H	−0.708433	−0.666797	1.692279
H	−0.424643	1.080420	1.462086
H	1.220180	0.311696	2.963804
H	3.760729	−2.012061	1.778531
H	2.076177	1.520692	0.583963
H	2.880228	−0.344558	−0.579016
H	4.306175	−0.137077	0.443691
N	2.646861	−2.769525	0.235632
C	1.803749	−2.743943	−0.871539
N	1.709578	−3.916634	−1.475377
C	2.524048	−4.767735	−0.730895
C	2.827200	−6.144090	−0.862514
N	2.316748	−6.926240	−1.827721
N	3.672101	−6.689965	0.058176
C	4.172481	−5.914781	1.039009
N	3.943781	−4.608630	1.259775
C	3.109313	−4.080161	0.342617
H	1.268739	−1.844803	−1.142996
H	2.593188	−7.912298	−1.896642
H	1.746521	−6.513815	−2.553960
H	4.849035	−6.417814	1.726634
P	4.548286	2.287809	2.022325
O	4.477495	2.790370	0.501988
O	4.692620	3.317815	3.086534
O	5.773567	1.257963	1.873912
C	6.116377	0.443115	3.046959
C	7.040302	−0.673908	2.619465
O	6.318289	−1.601121	1.769245
C	7.284601	−2.179255	0.858041
C	8.314629	−0.242008	1.830740
C	8.206289	−1.005417	0.505956
O	9.454476	−0.623720	2.679844
H	5.203408	0.023816	3.479807
H	6.614615	1.076904	3.786156
H	7.369537	−1.192652	3.529695
H	7.831391	−2.990745	1.344906
H	8.379338	0.835845	1.684878
H	7.732225	−0.348442	−0.228116
H	9.167530	−1.318649	0.103116
N	6.576261	−2.792594	−0.256732
C	6.644176	−4.210920	−0.423359
O	7.289289	−4.884408	0.403634
N	6.019551	−4.757632	−1.494025
C	5.311476	−4.004802	−2.359167
N	4.724782	−4.618395	−3.391447
C	5.199565	−2.582950	−2.186506
C	5.852284	−2.028118	−1.135978
H	4.819770	−5.639805	−3.524358
H	4.180324	−4.080541	−4.052966
H	4.624164	−1.973377	−2.872530
H	5.830229	−0.964269	−0.939009
P	10.981797	−0.834862	2.263144
O	11.142663	−0.027177	0.889668
O	11.900521	−0.488562	3.380729
O	11.093313	−2.362577	1.761452
C	11.018278	−3.429872	2.777785
C	11.587310	−4.719594	2.214666
O	10.698224	−5.256720	1.193998
C	11.421866	−5.473540	−0.039463
C	12.987933	−4.601893	1.574349
C	12.696738	−4.620333	0.068499
O	13.748332	−5.759155	2.006289
H	9.968362	−3.566946	3.052670
H	11.594505	−3.126926	3.656786
H	11.631367	−5.434639	3.046078
H	11.639557	−6.534458	−0.166999
H	13.499007	−3.683943	1.882191
H	12.506447	−3.601217	−0.279770
H	13.522858	−5.036845	−0.512930
H	14.635193	−5.694562	1.606741
N	10.539796	−5.105797	−1.157271
C	10.200662	−6.060696	−2.155415
O	10.700996	−7.206873	−2.108554
N	9.343305	−5.679498	−3.139503
C	8.867507	−4.419407	−3.203772
N	8.048465	−4.104999	−4.216581
C	9.221204	−3.438111	−2.219891
C	10.040418	−3.832746	−1.214581
H	7.756162	−4.822187	−4.900202
H	7.656748	−3.174476	−4.274862
H	8.835605	−2.427397	−2.270808
H	10.333916	−3.180984	−0.400672
O	5.430189	−12.159841	−10.599101
C	6.544914	−13.050953	−10.837310
C	7.234767	−13.401750	−9.523375
O	7.896341	−12.230017	−8.945058
C	7.267058	−11.866916	−7.690532
C	6.306017	−13.933850	−8.415426
C	5.967422	−12.676884	−7.623101
O	7.098705	−14.946055	−7.668663
H	5.119045	−11.838555	−11.463391
H	7.282653	−12.577014	−11.496870
H	6.202758	−13.984558	−11.306957
H	8.008573	−14.143870	−9.744872

H	7.940310	−12.111006	−6.866737
H	5.430104	−14.456913	−8.799788
H	5.171521	−12.157789	−8.161622
H	5.628521	−12.858406	−6.605372
N	7.083757	−10.430770	−7.609518
C	6.248029	−9.608858	−8.366521
N	6.291599	−8.349153	−7.972212
C	7.190294	−8.332091	−6.901221
C	7.624248	−7.272798	−6.052813
O	7.290311	−6.063119	−6.107537
N	8.524822	−7.718914	−5.064193
C	8.974987	−9.019709	−4.930601
N	9.871891	−9.277054	−3.950737
N	8.580655	−10.009477	−5.735954
C	7.696909	−9.617000	−6.669296
H	5.670824	−10.006255	−9.191042
H	8.848496	−6.997381	−4.378314
H	10.101720	−8.560282	−3.249820
H	9.987819	−10.257276	−3.708016
P	7.088675	−15.287527	−6.107993
O	5.594494	−15.107815	−5.547824
O	7.761947	−16.593427	−5.874837
O	7.731261	−14.037845	−5.327722
C	9.187649	−13.939573	−5.136894
C	9.530056	−13.772801	−3.659690
O	9.288692	−12.421910	−3.177605
C	8.119065	−12.375493	−2.316642
C	8.733548	−14.698434	−2.726126
C	7.581555	−13.816450	−2.227700
O	9.662784	−15.112984	−1.661892
H	9.527752	−13.065990	−5.696928
H	9.665893	−14.843717	−5.520890
H	10.603538	−13.961550	−3.558776
H	8.427878	−11.989121	−1.343217
H	8.383737	−15.601492	−3.227679
H	6.723879	−13.961602	−2.885879
H	7.276386	−14.059228	−1.210497
N	7.156646	−11.411196	−2.834108
C	6.294501	−11.544221	−3.927754
N	5.649316	−10.431525	−4.204260
C	6.105420	−9.501505	−3.268116
C	5.794274	−8.120431	−3.090756
O	5.011435	−7.416816	−3.769667
N	6.498702	−7.557309	−2.010392
C	7.401256	−8.229842	−1.208170
N	7.991528	−7.525895	−0.228510
N	7.705053	−9.523082	−1.383564
C	7.045271	−10.091488	−2.408894
H	6.211335	−12.475918	−4.463261
H	6.311593	−6.546477	−1.810556
H	7.712876	−6.560412	0.000935
H	8.610217	−8.026669	0.394737
P	9.246512	−16.058922	−0.428791
O	7.961780	−16.902415	−0.886705
O	10.434462	−16.809851	0.059642
O	8.527761	−15.102729	0.639783
C	9.357457	−14.099232	1.324851
C	8.464755	−13.119076	2.049903
O	7.739546	−12.313882	1.089044
C	6.598362	−11.785295	1.809030
C	7.404984	−13.723031	3.016299
C	6.103843	−12.967045	2.660231
O	7.875984	−13.479334	4.362655
H	9.972872	−13.574678	0.587576
H	10.004435	−14.611381	2.042781
H	9.121964	−12.468365	2.647466
H	6.905872	−10.941062	2.430910
H	7.289219	−14.798654	2.859103
H	5.441522	−13.609786	2.073817
H	5.567201	−12.637134	3.552900
H	7.333445	−14.016012	4.968531
N	5.646615	−11.250275	0.840346
C	5.318351	−9.885020	0.899558
O	5.771986	−9.113958	1.749767
N	4.429368	−9.471906	−0.071731
C	3.808860	−10.264307	−1.034801
O	2.981755	−9.757202	−1.821139
C	4.190096	−11.668139	−1.039977
C	3.567666	−12.598702	−2.044552
C	5.085299	−12.082509	−0.108994
H	4.160197	−8.454267	−0.028862
H	2.490867	−12.704341	−1.861745
H	4.024252	−13.591241	−1.988547
H	3.687914	−12.209785	−3.060288
H	5.418455	−13.112385	−0.061753
H	4.974293	−15.797089	−5.856361
H	8.183225	−17.810731	−1.170171
H	12.057833	0.269146	0.716819
H	4.185141	3.719253	0.422627

d(ACG)$^{H+}$

E: −27519.82 kcal mol^{-1}

O	0.302129	−0.217399	−0.162921
C	0.160135	−0.050321	1.267852
C	1.504197	−0.255328	1.957341
O	1.901019	−1.662757	1.983231
C	3.045695	−1.900579	1.130168
C	2.673849	0.494327	1.301038
C	3.360010	−0.563231	0.433431
O	3.526962	0.980856	2.405661
H	−0.587443	−0.180115	−0.556135
H	−0.557769	−0.773751	1.674284
H	−0.191339	0.964716	1.501760
H	1.393342	0.065850	2.997826
H	3.880000	−2.253510	1.741854
H	2.348846	1.362186	0.727332
H	2.918597	−0.531275	−0.564020
H	4.434314	−0.406448	0.347366
N	2.750237	−2.993176	0.205080
C	1.841318	−2.983341	−0.849075
N	1.720239	−4.163024	−1.434568
C	2.584316	−5.001923	−0.731970
C	2.887039	−6.379014	−0.856844
N	2.334558	−7.185674	−1.778386
N	3.781962	−6.903604	0.027227
C	4.334454	−6.113254	0.966142
N	4.113532	−4.805212	1.176177
C	3.226645	−4.297690	0.298270
H	1.287771	−2.087893	−1.097420
H	2.609771	−8.172367	−1.831344
H	1.724552	−6.795759	−2.484162
H	5.044275	−6.607671	1.626118
P	4.836805	1.885307	2.188915
O	4.660526	2.670276	0.801463
O	5.109421	2.685363	3.413529
O	6.003597	0.884932	1.728124
C	6.546245	−0.054705	2.719943
C	7.419046	−1.065072	2.006622
O	6.590177	−1.994661	1.251859
C	7.314764	−2.268570	0.013911
C	8.424114	−0.453458	0.983864
C	7.873901	−0.896318	−0.369200
O	9.736470	−1.109222	1.131143
H	5.724658	−0.564479	3.232259
H	7.142277	0.506605	3.444903

H	7.977699	−1.614974	2.773768
H	8.126274	−2.966915	0.224205
H	8.542658	0.624954	1.086491
H	7.078779	−0.208362	−0.667491
H	8.635778	−0.936559	−1.147560
N	6.455738	−2.952665	−0.931683
C	6.558929	−4.379120	−1.002363
O	7.299801	−4.966395	−0.187733
N	5.854212	−5.028349	−1.958175
C	5.043707	−4.362537	−2.804438
N	4.400096	−5.067362	−3.741599
C	4.869553	−2.940801	−2.703232
C	5.599571	−2.283532	−1.768503
H	4.537762	−6.088252	−3.827025
H	3.775223	−4.596359	−4.382380
H	4.195501	−2.406596	−3.361744
H	5.544213	−1.209829	−1.646576
P	10.823443	−0.600711	2.189865
O	11.627580	0.499678	1.340558
O	10.276644	−0.151488	3.500993
O	11.843174	−1.829573	2.214727
C	11.580449	−2.997059	3.076378
C	12.026616	−4.268533	2.374312
O	11.059101	−4.632058	1.345806
C	11.755694	−4.911662	0.103348
C	13.409714	−4.190393	1.682339
C	13.055383	−4.100384	0.191604
O	14.116178	−5.414797	2.008846
H	10.514333	−3.056272	3.312154
H	12.150426	−2.855956	3.998721
H	12.050368	−5.063261	3.131272
H	11.949917	−5.985840	0.013516
H	13.987563	−3.325858	2.022181
H	12.866593	−3.057697	−0.080058
H	13.842412	−4.498512	−0.453228
H	14.999663	−5.362564	1.600041
N	10.881118	−4.561093	−0.999851
C	10.378284	−3.293382	−1.291593
N	9.554238	−3.298283	−2.320440
C	9.500996	−4.629232	−2.736557
C	8.759698	−5.247447	−3.784470
O	7.966365	−4.702050	−4.589525
N	9.000996	−6.633570	−3.853413
C	9.870126	−7.329923	−3.036719
N	10.032048	−8.643405	−3.299282
N	10.549126	−6.754950	−2.035567
C	10.320971	−5.431508	−1.927174
H	10.634068	−2.434511	−0.688686
H	8.488987	−7.158847	−4.601764
H	9.483323	−9.126037	−4.027946
H	10.520068	−9.197913	−2.607790
O	5.826686	−11.651274	−10.463894
C	7.063364	−12.357002	−10.726611
C	7.709527	−12.795924	−9.417659
O	8.223677	−11.648547	−8.668296
C	7.523987	−11.510445	−7.401185
C	6.771812	−13.535899	−8.446752
C	6.303559	−12.435278	−7.500994
O	7.608384	−14.566582	−7.788463
H	5.533628	−11.244191	−11.297830
H	7.769495	−11.710919	−11.262726
H	6.873793	−13.252729	−11.335235
H	8.560395	−13.437845	−9.669009
H	8.189889	−11.793593	−6.585198
H	5.954281	−14.055447	−8.947508
H	5.462836	−11.926122	−7.976559
H	5.969290	−12.793942	−6.531801
N	7.211679	−10.097025	−7.170805
C	7.881525	−9.389520	−6.131171
O	8.722712	−9.990732	−5.426419
N	7.577391	−8.078951	−5.945388
C	6.675713	−7.453311	−6.729199
N	6.425663	−6.158140	−6.494036
C	6.017872	−8.140974	−7.803203
C	6.326053	−9.448063	−7.993827
H	6.928600	−5.646624	−5.753165
H	5.752943	−5.665628	−7.066403
H	5.319858	−7.629344	−8.455816
H	5.918075	−10.036447	−8.809143
P	7.239871	−15.418777	−6.485121
O	5.656685	−15.295614	−6.268547
O	7.816540	−16.788367	−6.564289
O	7.740520	−14.540701	−5.230772
C	9.189741	−14.418879	−5.001068
C	9.470954	−14.137355	−3.530808
O	9.193042	−12.759457	−3.166460
C	8.038989	−12.666846	−2.293227
C	8.668458	−15.009274	−2.549820
C	7.510380	−14.103810	−2.115072
O	9.597543	−15.356374	−1.460365
H	9.572359	−13.601897	−5.618730
H	9.674416	−15.357634	−5.284740
H	10.543762	−14.301181	−3.383778
H	8.352260	−12.218812	−1.346700
H	8.322912	−15.942333	−2.995774
H	6.660888	−14.294606	−2.772743
H	7.193601	−14.285850	−1.088872
N	7.063182	−11.745551	−2.860658
C	6.235599	−11.952336	−3.965674
N	5.564193	−10.872568	−4.308836
C	5.966746	−9.889910	−3.403282
C	5.599469	−8.516419	−3.289784
O	4.787916	−7.875649	−3.996683
N	6.276371	−7.879603	−2.231974
C	7.183021	−8.488064	−1.384480
N	7.731605	−7.722770	−0.427326
N	7.520295	−9.780358	−1.484584
C	6.904990	−10.412056	−2.499010
H	6.189265	−12.913175	−4.452643
H	6.086255	−6.857520	−2.104559
H	7.547768	−6.710256	−0.352273
H	8.425415	−8.151683	0.169630
P	9.182687	−16.152892	−0.127442
O	7.834888	−16.954720	−0.460311
O	10.340630	−16.930143	0.391899
O	8.575602	−15.049939	0.866095
C	9.493398	−14.032468	1.402152
C	8.696613	−13.022024	2.195278
O	7.890379	−12.226823	1.285360
C	6.682236	−11.870609	2.005904
C	7.730753	−13.610353	3.266003
C	6.340548	−13.133405	2.805069
O	8.124693	−13.047893	4.542519
H	10.010179	−13.541178	0.572180
H	10.224482	−14.523601	2.050929
H	9.415826	−12.368322	2.709468
H	6.876776	−11.024353	2.669112
H	7.790865	−14.701112	3.309778
H	5.886085	−13.891636	2.160861
H	5.669852	−12.934328	3.644294
H	7.602082	−13.493185	5.234287
N	5.690592	−11.404650	1.044089
C	5.347508	−10.040504	1.048160
O	5.795722	−9.231777	1.866261
N	4.453164	−9.674744	0.065289
C	3.847776	−10.505883	−0.872499
O	3.018338	−10.034907	−1.679259
C	4.251176	−11.903046	−0.830388

C	3.650745	−12.873134	−1.810168
C	5.151973	−12.272739	0.114252
H	4.196856	−8.650554	0.047865
H	2.572402	−12.981674	−1.638814
H	4.115216	−13.858855	−1.715276
H	3.781452	−12.518035	−2.837006
H	5.501660	−13.294881	0.190697
H	5.193368	−16.148837	−6.377404
H	7.976518	−17.917912	−0.539695
H	12.157516	1.103924	1.897341
H	4.280638	3.563437	0.913444

d(ACT)$^{H+}$

E: −27607.26 kcal mol^{-1}

O	−0.057977	−0.224340	−0.390124
C	−0.124265	0.093635	1.020468
C	1.261649	−0.000784	1.647638
O	1.708302	−1.387681	1.793600
C	2.799923	−1.685033	0.892309
C	2.369301	0.709393	0.852827
C	3.019751	−0.415204	0.046735
O	3.262688	1.326784	1.853753
H	−0.969114	−0.256434	−0.730828
H	−0.794430	−0.599785	1.543805
H	−0.494314	1.118666	1.165162
H	1.197310	0.421000	2.655382
H	3.682221	−1.956572	1.479946
H	1.989924	1.512115	0.220101
H	2.496974	−0.490986	−0.907918
H	4.072244	−0.238178	−0.160450
N	2.483502	−2.869391	0.094142
C	1.556840	−2.963521	−0.939906
N	1.479450	−4.180575	−1.452346
C	2.394764	−4.934488	−0.720530
C	2.763475	−6.299670	−0.785390
N	2.218777	−7.173975	−1.647147
N	3.711848	−6.733359	0.093277
C	4.244999	−5.865642	0.975287
N	3.960326	−4.561334	1.127736
C	3.025218	−4.144155	0.251148
H	0.957119	−2.111402	−1.229119
H	2.550298	−8.145412	−1.677592
H	1.566105	−6.843952	−2.345674
H	5.002536	−6.283847	1.635069
P	4.696700	1.979576	1.554765
O	4.770774	2.280550	−0.017621
O	4.964009	3.110761	2.483967
O	5.742149	0.759451	1.628786
C	5.930922	0.115722	2.937360
C	6.975306	−0.971664	2.799029
O	6.438725	−2.050536	1.986445
C	7.449596	−2.490193	1.046127
C	8.301764	−0.518458	2.135564
C	8.314076	−1.249079	0.792130
O	9.382279	−0.930965	3.045522
H	4.980139	−0.313465	3.266559
H	6.266488	0.864889	3.660670
H	7.195510	−1.348835	3.805219
H	8.032899	−3.314915	1.460665
H	8.365240	0.563438	2.021568
H	7.854459	−0.599593	0.043475
H	9.316240	−1.506413	0.455505
N	6.774409	−3.034211	−0.132069
C	6.895169	−4.423013	−0.434708
O	7.637080	−5.132566	0.274244
N	6.206033	−4.911158	−1.496152
C	5.397323	−4.124198	−2.231894
N	4.732062	−4.684004	−3.248692
C	5.246330	−2.727455	−1.930266
C	5.946037	−2.237695	−0.877947
H	4.802562	−5.697964	−3.433809
H	4.098395	−4.124235	−3.803822
H	4.594582	−2.090190	−2.515536
H	5.884695	−1.203153	−0.565647
P	10.953995	−0.774163	2.740123
O	11.086002	0.224080	1.493362
O	11.700616	−0.428463	3.980396
O	11.387548	−2.147096	2.029833
C	11.388510	−3.364470	2.860949
C	11.919773	−4.525823	2.046928
O	10.958158	−4.860909	1.009775
C	11.658053	−5.057073	−0.241461
C	13.283492	−4.288843	1.348614
C	12.917349	−4.185086	−0.139494
O	14.111496	−5.444370	1.638212
H	10.363967	−3.568868	3.186141
H	12.026887	−3.197878	3.733740
H	12.021669	−5.378500	2.731748
H	11.906773	−6.111719	−0.375854
H	13.773350	−3.380907	1.713103
H	12.695701	−3.143708	−0.388065
H	13.719600	−4.536434	−0.792674
H	14.985948	−5.287654	1.236872
N	10.734703	−4.721206	−1.327607
C	10.275056	−5.746918	−2.171620
O	10.686701	−6.909195	−2.113580
N	9.320677	−5.347017	−3.085029
C	8.860729	−4.052617	−3.300774
O	8.025987	−3.821573	−4.201284
C	9.411090	−3.029216	−2.427693
C	9.009492	−1.596940	−2.645965
C	10.295433	−3.421165	−1.476463
H	8.964515	−6.104344	−3.728349
H	9.278193	−1.277761	−3.660303
H	9.510326	−0.934579	−1.936143
H	7.926140	−1.468775	−2.550809
H	10.723047	−2.717773	−0.772376
O	5.969007	−11.620208	−10.635795
C	7.156322	−12.424153	−10.830554
C	7.637939	−12.988940	−9.498681
O	8.205426	−11.948020	−8.639081
C	7.355403	−11.703713	−7.492614
C	6.548359	−13.670002	−8.652105
C	6.098682	−12.565064	−7.699049
O	7.199200	−14.827933	−7.999611
H	5.768079	−11.178259	−11.479189
H	7.964431	−11.822300	−11.264800
H	6.945180	−13.267655	−11.503610
H	8.440707	−13.700996	−9.714086
H	7.905580	−11.971829	−6.585224
H	5.729227	−14.069808	−9.250640
H	5.322536	−11.994001	−8.210388
H	5.679048	−12.926218	−6.763428
N	7.071320	−10.279225	−7.361781
C	6.182070	−9.511191	−8.105688
N	6.138680	−8.248962	−7.711531
C	7.046220	−8.173804	−6.655941
C	7.450277	−7.103199	−5.824000
N	6.977577	−5.850924	−5.939448
N	8.382679	−7.375909	−4.866465
C	8.881443	−8.623386	−4.757619
N	8.576170	−9.700538	−5.497547
C	7.645919	−9.420206	−6.429914
H	5.633183	−9.935385	−8.934806
H	7.285081	−5.117655	−5.288408

H	6.238734	−5.657903	−6.602450
H	9.614969	−8.760022	−3.965269
P	6.766734	−15.558739	−6.643784
O	5.201779	−15.300058	−6.412226
O	7.230885	−16.972473	−6.647966
O	7.345376	−14.645367	−5.450863
C	8.803740	−14.658540	−5.239942
C	9.111947	−14.270088	−3.803861
O	8.891831	−12.849215	−3.589183
C	7.864298	−12.626484	−2.589857
C	8.260897	−15.013408	−2.756156
C	7.193416	−13.991515	−2.361639
O	9.173786	−15.370619	−1.658503
H	9.263881	−13.949036	−5.932903
H	9.184664	−15.665241	−5.434066
H	10.173806	−14.480116	−3.634901
H	8.322218	−12.233379	−1.676591
H	7.831837	−15.938812	−3.141176
H	6.338254	−14.119026	−3.027828
H	6.848967	−14.107915	−1.335402
N	6.957398	−11.589806	−3.053722
C	6.027699	−11.668133	−4.093880
N	5.404278	−10.526902	−4.303596
C	5.942870	−9.637535	−3.370728
C	5.682223	−8.254289	−3.128691
O	4.874960	−7.505410	−3.725495
N	6.472161	−7.746231	−2.079501
C	7.418692	−8.463538	−1.373649
N	8.089337	−7.813875	−0.411090
N	7.675621	−9.757739	−1.611619
C	6.920934	−10.278694	−2.594155
H	5.871539	−12.588344	−4.636675
H	6.351621	−6.731346	−1.855826
H	7.908117	−6.826587	−0.176724
H	8.788110	−8.332502	0.102850
P	8.725959	−16.060948	−0.279140
O	7.266715	−16.667590	−0.543175
O	9.777639	−16.988360	0.219649
O	8.357769	−14.844513	0.703114
C	9.458470	−13.971087	1.142422
C	8.901542	−12.877073	2.026583
O	8.087497	−11.984674	1.219167
C	6.951123	−11.571855	2.014781
C	8.023474	−13.350683	3.218668
C	6.629788	−12.787287	2.895096
O	8.597541	−12.774717	4.419310
H	9.939547	−13.536049	0.260967
H	10.185433	−14.568597	1.700666
H	9.757818	−12.320061	2.432315
H	7.203409	−10.694643	2.614925
H	8.009661	−14.441160	3.303144
H	6.054316	−13.529973	2.335016
H	6.071153	−12.516793	3.794314
H	8.107563	−13.134357	5.181388
N	5.892885	−11.145037	1.100256
C	5.500512	−9.796425	1.097416
O	5.947505	−8.956620	1.884143
N	4.559535	−9.479665	0.138655
C	3.938565	−10.354628	−0.748260
O	3.062340	−9.934586	−1.533143
C	4.379275	−11.738607	−0.684255
C	3.746827	−12.751644	−1.597688
C	5.336181	−12.054665	0.223341
H	4.255574	−8.470008	0.121879
H	2.671885	−12.835620	−1.395995
H	4.199335	−13.737244	−1.460915
H	3.854367	−12.453767	−2.645777
H	5.722751	−13.062435	0.313080
H	11.507296	1.072434	1.732710
H	4.750507	3.236267	−0.219980
H	4.657072	−16.086538	−6.611483
H	7.207348	−17.617772	−0.324718

d(AGA)$^{H+}$

E: −27606.92 kcal mol^{-1}

O	0.086914	−0.287386	−0.420173
C	−0.090267	−0.020463	0.990951
C	1.250412	−0.099108	1.711865
O	1.725375	−1.475866	1.843244
C	2.892863	−1.711597	1.020351
C	2.388246	0.671637	1.022874
C	3.160899	−0.402338	0.252773
O	3.172291	1.305851	2.103878
H	−0.796027	−0.348205	−0.824951
H	−0.775070	−0.749668	1.441471
H	−0.500280	0.988408	1.142354
H	1.102282	0.286005	2.725736
H	3.729549	−1.993167	1.665015
H	2.025305	1.470005	0.375436
H	2.761537	−0.454453	−0.761055
H	4.227542	−0.192865	0.190760
N	2.667331	−2.865742	0.151459
C	1.837434	−2.940727	−0.962370
N	1.792907	−4.152661	−1.491227
C	2.628776	−4.924069	−0.685958
C	2.981805	−6.293890	−0.720638
N	2.509818	−7.162774	−1.631628
N	3.836135	−6.743141	0.243435
C	4.289934	−5.888580	1.181161
N	4.008333	−4.581992	1.311882
C	3.174142	−4.147893	0.347289
H	1.275878	−2.080221	−1.298192
H	2.847094	−8.133620	−1.642289
H	1.968133	−6.814069	−2.411344
H	4.970294	−6.322051	1.911456
P	4.536340	2.119996	1.871743
O	4.551593	2.615008	0.346970
O	4.711002	3.146887	2.934481
O	5.695505	1.011839	1.771128
C	5.980436	0.218006	2.973750
C	6.976532	−0.866141	2.625893
O	6.342172	−1.863239	1.780412
C	7.326023	−2.324267	0.818335
C	8.252393	−0.382658	1.875532
C	8.151528	−1.070603	0.513198
O	9.386999	−0.810191	2.711807
H	5.052821	−0.229383	3.343019
H	6.400971	0.874654	3.740882
H	7.289519	−1.334529	3.567791
H	7.939696	−3.123830	1.251383
H	8.314454	0.702223	1.794041
H	7.589332	−0.411302	−0.154449
H	9.109882	−1.283318	0.044126
N	6.649074	−2.910033	−0.313137
C	5.847955	−2.273758	−1.265058
N	5.377057	−3.109912	−2.167947
C	5.889269	−4.359403	−1.812079
C	5.737285	−5.640949	−2.417612
O	5.047050	−5.934160	−3.420855
N	6.487982	−6.630264	−1.754542
C	7.260770	−6.419492	−0.628607
N	7.911076	−7.480374	−0.121025
N	7.370211	−5.223405	−0.035369
C	6.685427	−4.254454	−0.663126
H	5.661505	−1.210642	−1.232464

H	6.448983	−7.593506	−2.165321
H	7.933105	−8.387386	−0.607116
H	8.561561	−7.301990	0.633377
P	10.914502	−1.030430	2.298053
O	11.133881	−0.237757	0.922812
O	11.827497	−0.701086	3.425847
O	10.998493	−2.551321	1.779105
C	10.860537	−3.633504	2.772077
C	11.564377	−4.887035	2.277227
O	10.829866	−5.503061	1.180745
C	11.606508	−5.451486	−0.041483
C	12.999998	−4.666141	1.765826
C	12.815991	−4.545959	0.246574
O	13.767308	−5.839872	2.135149
H	9.794591	−3.830200	2.916225
H	11.310104	−3.312427	3.716462
H	11.571533	−5.596211	3.113597
H	11.903610	−6.468228	−0.313507
H	13.453956	−3.768320	2.198334
H	12.594389	−3.508833	−0.013632
H	13.702291	−4.862003	−0.308267
H	14.667377	−5.728187	1.777930
N	10.754830	−4.986032	−1.129492
C	10.242141	−3.709276	−1.322412
N	9.428928	−3.628743	−2.361553
C	9.388323	−4.922750	−2.880907
C	8.697627	−5.495293	−3.975324
N	7.895453	−4.803245	−4.807533
N	8.872197	−6.829076	−4.192858
C	9.679661	−7.542711	−3.386033
N	10.387402	−7.099597	−2.335581
C	10.202449	−5.782060	−2.124473
H	10.503027	−2.898005	−0.659059
H	7.317058	−5.318603	−5.481818
H	7.641202	−3.853790	−4.565941
H	9.752451	−8.602197	−3.623687
O	5.769558	−11.898108	−10.604503
C	6.981101	−12.639637	−10.886169
C	7.649813	−13.061785	−9.584199
O	8.222058	−11.909201	−8.884596
C	7.577758	−11.713939	−7.603577
C	6.712560	−13.742373	−8.570181
C	6.331529	−12.612705	−7.618779
O	7.511070	−14.809869	−7.927790
H	5.451826	−11.518360	−11.442301
H	7.686676	−12.024514	−11.458066
H	6.752169	−13.545421	−11.465256
H	8.472864	−13.738090	−9.836493
H	8.268387	−11.978288	−6.800595
H	5.851461	−14.221621	−9.036334
H	5.475757	−12.088541	−8.049058
H	6.050519	−12.959284	−6.627276
N	7.290099	−10.285807	−7.412352
C	7.964083	−9.589673	−6.393307
O	8.808685	−10.111538	−5.659095
N	7.585417	−8.270039	−6.263130
C	6.668327	−7.576606	−7.045003
O	6.433188	−6.370682	−6.811616
C	6.043578	−8.340535	−8.113008
C	5.059929	−7.658485	−9.024110
C	6.392507	−9.645590	−8.249147
H	8.059236	−7.732403	−5.486456
H	5.523498	−6.801977	−9.528882
H	4.205186	−7.271477	−8.455449
H	4.687588	−8.352496	−9.783037
H	5.997371	−10.265207	−9.048625
P	7.148078	−15.619043	−6.601135
O	5.561071	−15.450373	−6.463801
O	7.701648	−16.999660	−6.630384
O	7.678778	−14.725674	−5.365534
C	9.122247	−14.732238	−5.072331
C	9.349084	−14.248188	−3.651621
O	9.064675	−12.826511	−3.552845
C	8.084839	−12.565911	−2.520260
C	8.480516	−14.955473	−2.589407
C	7.415954	−13.918829	−2.226536
O	9.379135	−15.291906	−1.473434
H	9.624791	−14.068958	−5.781915
H	9.505067	−15.750834	−5.181035
H	10.408096	−14.409763	−3.419651
H	8.568394	−12.144773	−1.637344
H	8.047917	−15.887197	−2.954339
H	6.549071	−14.079356	−2.870450
H	7.088609	−13.991826	−1.190840
N	7.167781	−11.524589	−3.000734
C	7.198463	−10.222092	−2.415571
O	7.966087	−10.002971	−1.456241
N	6.369709	−9.273794	−2.922912
C	5.548212	−9.537038	−3.957555
N	4.761734	−8.552571	−4.407422
C	5.528560	−10.830566	−4.578498
C	6.363867	−11.774456	−4.080802
H	4.839194	−7.595362	−4.021235
H	4.146506	−8.722873	−5.191934
H	4.883773	−11.037850	−5.423461
H	6.450079	−12.764869	−4.509902
P	8.907970	−15.968839	−0.094360
O	7.455223	−16.581180	−0.381022
O	9.952148	−16.888774	0.432893
O	8.518232	−14.744759	0.870313
C	9.612105	−13.887051	1.356958
C	9.045023	−12.830444	2.281343
O	8.255981	−11.889057	1.502427
C	7.047884	−11.579539	2.240559
C	8.135086	−13.355351	3.424122
C	6.735164	−12.855307	3.033146
O	8.615726	−12.757428	4.655017
H	10.099517	−13.414843	0.498473
H	10.334937	−14.505958	1.896736
H	9.897201	−12.302222	2.730466
H	7.215553	−10.727544	2.902879
H	8.171734	−14.445795	3.503276
H	6.246026	−13.601402	2.400919
H	6.101915	−12.662796	3.902496
H	8.088193	−13.126704	5.386859
N	6.033535	−11.147113	1.279394
C	5.627549	−9.800331	1.282079
O	6.012333	−8.978790	2.118676
N	4.747681	−9.468974	0.271507
C	4.184905	−10.331327	−0.664617
O	3.355168	−9.905677	−1.495515
C	4.624830	−11.715162	−0.596479
C	4.027959	−12.720165	−1.542496
C	5.534621	−12.041947	0.354960
H	4.427665	−8.464722	0.262004
H	2.946069	−12.802872	−1.381388
H	4.471487	−13.707924	−1.394554
H	4.172348	−12.417790	−2.584564
H	5.917580	−13.050498	0.449399
H	5.140562	−16.129581	−5.900971
H	7.393408	−17.528856	−0.152239
H	11.935229	0.321195	0.933499
H	4.315864	3.558381	0.252281

d(AGC)$^{H+}$

E: −27519.13 kcal mol^{-1}

O	−0.001250	−0.306615	−0.398738
C	−0.175281	−0.075672	1.019005
C	1.170880	−0.147127	1.730522
O	1.673176	−1.518100	1.820561
C	2.832347	−1.709150	0.974698
C	2.290680	0.662407	1.056702
C	3.061616	−0.375242	0.238084
O	3.082139	1.264752	2.150371
H	−0.885084	−0.382779	−0.798959
H	−0.843358	−0.828142	1.456194
H	−0.603493	0.921374	1.196445
H	1.021839	0.206568	2.755606
H	3.683118	−1.989720	1.601571
H	1.911619	1.480634	0.444097
H	2.635561	−0.403610	−0.765588
H	4.122007	−0.145398	0.152129
N	2.620914	−2.844404	0.078185
C	1.818207	−2.898757	−1.056969
N	1.801076	−4.096158	−1.619120
C	2.627009	−4.879634	−0.815226
C	3.000524	−6.243251	−0.879968
N	2.571278	−7.090280	−1.829998
N	3.837213	−6.707981	0.092784
C	4.254855	−5.874812	1.065580
N	3.952493	−4.576241	1.225292
C	3.137577	−4.125934	0.251595
H	1.254231	−2.036997	−1.384522
H	2.866021	−8.074399	−1.820540
H	2.004076	−6.740676	−2.590567
H	4.922030	−6.319888	1.800954
P	4.462807	2.057395	1.945235
O	4.511600	2.555207	0.422110
O	4.637284	3.080103	3.012148
O	5.605326	0.929174	1.864949
C	5.867547	0.141954	3.077186
C	6.886910	−0.931011	2.759965
O	6.286144	−1.933715	1.896142
C	7.283083	−2.366046	0.937214
C	8.175965	−0.433778	2.045977
C	8.113779	−1.103575	0.672739
O	9.295339	−0.890222	2.884643
H	4.935642	−0.317139	3.420266
H	6.257658	0.805191	3.854777
H	7.176578	−1.396733	3.710429
H	7.892224	−3.177262	1.354406
H	8.236919	0.652446	1.983387
H	7.575331	−0.438609	−0.008357
H	9.092871	−1.308867	0.243115
N	6.618647	−2.916166	−0.221939
C	5.839046	−2.238552	−1.164116
N	5.400602	−3.032011	−2.120008
C	5.909432	−4.294370	−1.810673
C	5.805124	−5.538319	−2.500984
O	5.165327	−5.777708	−3.550325
N	6.540946	−6.556195	−1.867882
C	7.279610	−6.400505	−0.710407
N	7.961586	−7.471145	−0.271701
N	7.355610	−5.238775	−0.046375
C	6.672615	−4.243394	−0.635825
H	5.641958	−1.180170	−1.082826
H	6.481188	−7.510172	−2.296231
H	7.862366	−8.400625	−0.705918
H	8.433973	−7.387202	0.618438
P	10.857045	−0.854046	2.539629
O	10.965155	0.074963	1.239613
O	11.676247	−0.479080	3.724113
O	11.188464	−2.308907	1.935636
C	11.207246	−3.448079	2.871644
C	11.819197	−4.655586	2.188179
O	10.923028	−5.139532	1.146843
C	11.638424	−5.250883	−0.106108
C	13.194256	−4.413492	1.524795
C	12.860908	−4.329813	0.029933
O	14.024684	−5.560102	1.842830
H	10.178209	−3.665141	3.173492
H	11.801076	−3.177728	3.749703
H	11.922778	−5.437257	2.951691
H	11.922292	−6.288353	−0.285378
H	13.666713	−3.497412	1.893451
H	12.600532	−3.299940	−0.230564
H	13.691527	−4.650226	−0.603480
H	14.895570	−5.416666	1.428866
N	10.720019	−4.892333	−1.194674
C	10.380650	−5.850690	−2.187612
O	10.882916	−6.996778	−2.135428
N	9.519359	−5.478424	−3.170245
C	9.009745	−4.231021	−3.219870
N	8.192185	−3.927046	−4.237660
C	9.325945	−3.258041	−2.214800
C	10.170992	−3.638159	−1.224929
H	7.914095	−4.649223	−4.920465
H	7.721996	−3.031189	−4.251671
H	8.895452	−2.264471	−2.239319
H	10.447642	−2.989240	−0.402668
O	5.910540	−11.748916	−10.735029
C	7.036733	−12.636941	−10.927685
C	7.538973	−13.156100	−9.584995
O	8.193200	−12.102806	−8.805633
C	7.411419	−11.766118	−7.634290
C	6.449133	−13.729354	−8.662831
C	6.094497	−12.549735	−7.761535
O	7.074855	−14.869094	−7.955307
H	5.704517	−11.341039	−11.594278
H	7.861283	−12.113217	−11.427348
H	6.745585	−13.501589	−11.541234
H	8.292862	−13.922653	−9.789845
H	7.977121	−12.041624	−6.738757
H	5.590329	−14.125484	−9.205369
H	5.344792	−11.951571	−8.282886
H	5.681281	−12.840075	−6.798281
N	7.232007	−10.323114	−7.544329
C	6.456710	−9.498595	−8.364896
N	6.511478	−8.231275	−8.000837
C	7.360873	−8.204598	−6.892601
C	7.778646	−7.126061	−6.058266
O	7.469938	−5.913417	−6.160330
N	8.629232	−7.556810	−5.023045
C	9.052361	−8.858884	−4.835013
N	9.905334	−9.088311	−3.817768
N	8.660150	−9.872134	−5.615411
C	7.826357	−9.494303	−6.600768
H	5.921272	−9.901958	−9.213562
H	8.963733	−6.818304	−4.361643
H	10.204294	−8.340059	−3.177000
H	10.120840	−10.052953	−3.601444
P	6.594469	−15.554412	−6.592588
O	5.046668	−15.192926	−6.391416
O	6.971492	−16.992983	−6.560604
O	7.226574	−14.655885	−5.412043
C	8.686152	−14.708858	−5.217704
C	9.026697	−14.276958	−3.801047
O	8.832860	−12.845974	−3.634547
C	7.866374	−12.568676	−2.591904
C	8.187649	−14.969647	−2.708619
C	7.169332	−13.906369	−2.292712

O	9.124907	−15.338374	−1.635608
H	9.159853	−14.040840	−5.942059
H	9.032098	−15.733955	−5.378756
H	10.088049	−14.499839	−3.645049
H	8.368548	−12.160703	−1.713521
H	7.713118	−15.885548	−3.061169
H	6.273603	−14.031607	−2.903900
H	6.881826	−13.984588	−1.245767
N	6.971169	−11.500510	−3.061055
C	7.030034	−10.209688	−2.455436
O	7.742917	−10.043665	−1.445335
N	6.293291	−9.209840	−3.005877
C	5.496166	−9.427716	−4.070177
N	4.800968	−8.394958	−4.560477
C	5.404636	−10.725516	−4.674773
C	6.175368	−11.711882	−4.156400
H	4.921699	−7.443424	−4.174338
H	4.236288	−8.520485	−5.390354
H	4.767025	−10.900192	−5.532355
H	6.216323	−12.706760	−4.582052
P	8.695465	−16.036612	−0.252049
O	7.220474	−16.620905	−0.477907
O	9.748613	−16.980291	0.211760
O	8.356262	−14.829127	0.749792
C	9.473965	−13.981591	1.197193
C	8.939324	−12.892197	2.101594
O	8.143457	−11.970409	1.309483
C	6.987421	−11.579311	2.089858
C	8.051267	−13.368182	3.284050
C	6.659037	−12.809476	2.946095
O	8.609909	−12.787627	4.489942
H	9.957162	−13.539319	0.320463
H	10.193331	−14.600954	1.741146
H	9.807123	−12.359937	2.515653
H	7.223110	−10.710658	2.708730
H	8.040927	−14.458629	3.369889
H	6.098454	−13.550250	2.369163
H	6.084995	−12.554364	3.840034
H	8.108185	−13.141895	5.246892
N	5.947032	−11.140329	1.160504
C	5.602280	−9.777526	1.124879
O	6.055953	−8.943216	1.913437
N	4.699387	−9.443424	0.136291
C	4.089641	−10.307051	−0.767351
O	3.269398	−9.869974	−1.602415
C	4.471591	−11.705593	−0.664205
C	3.822196	−12.708173	−1.577708
C	5.385182	−12.042091	0.280476
H	4.410999	−8.429652	0.109721
H	2.736413	−12.721737	−1.423993
H	4.206991	−13.714236	−1.392664
H	3.990344	−12.450300	−2.628986
H	5.726463	−13.062867	0.399490
H	4.469152	−15.980619	−6.419175
H	7.185071	−17.594223	−0.401652
H	11.811696	0.559469	1.180557
H	4.331410	3.511014	0.329628

d(AGG)$^{H+}$

E: −27519.63 kcal mol^{-1}

O	0.122583	−0.170228	−0.175978
C	−0.027145	0.066124	1.243824
C	1.327368	−0.030350	1.935984
O	1.797297	−1.411339	2.033509
C	2.947094	−1.638949	1.185263
C	2.453807	0.748549	1.236700
C	3.218965	−0.314894	0.443966
O	3.255382	1.375153	2.308851
H	−0.768023	−0.225706	−0.564431
H	−0.704215	−0.672057	1.691501
H	−0.432820	1.071839	1.424842
H	1.201790	0.337295	2.959407
H	3.791237	−1.944165	1.808386
H	2.077763	1.550780	0.601942
H	2.820305	−0.345512	−0.571132
H	4.287315	−0.110600	0.389145
N	2.695065	−2.770591	0.293170
C	1.844865	−2.813869	−0.807005
N	1.781441	−4.012851	−1.362563
C	2.625011	−4.809190	−0.590030
C	2.974176	−6.178161	−0.666539
N	2.487346	−7.022963	−1.593723
N	3.840177	−6.654981	0.272823
C	4.313950	−5.826704	1.223872
N	4.042557	−4.521949	1.390291
C	3.194144	−4.060543	0.451233
H	1.284717	−1.941387	−1.112905
H	2.852322	−7.982015	−1.655707
H	1.950178	−6.647776	−2.364632
H	5.003181	−6.281927	1.932163
P	4.574826	2.253645	2.050680
O	4.514815	2.783625	0.538660
O	4.741321	3.263326	3.131274
O	5.777198	1.201955	1.882941
C	6.128128	0.370253	3.042104
C	7.081066	−0.716097	2.591365
O	6.372910	−1.657106	1.742437
C	7.307361	−2.118235	0.731764
C	8.319686	−0.218641	1.783516
C	8.142593	−0.874361	0.411753
O	9.524154	−0.591056	2.557305
H	5.220905	−0.076941	3.458695
H	6.607879	0.999395	3.797447
H	7.442031	−1.231513	3.491223
H	7.927298	−2.925619	1.131452
H	8.366297	0.867211	1.717160
H	7.558668	−0.188032	−0.208747
H	9.074266	−1.090614	−0.108528
N	6.587008	−2.698987	−0.372265
C	5.775206	−2.084030	−1.329604
N	5.301606	−2.941713	−2.212003
C	5.823772	−4.182705	−1.836479
C	5.696163	−5.475826	−2.423624
O	5.020630	−5.792888	−3.431015
N	6.451088	−6.446051	−1.736075
C	7.224664	−6.204462	−0.614691
N	7.890061	−7.241631	−0.076337
N	7.319692	−4.995355	−0.048279
C	6.625500	−4.049712	−0.696495
H	5.583214	−1.021119	−1.317375
H	6.436580	−7.414915	−2.134261
H	7.902829	−8.165065	−0.530169
H	8.617419	−7.003068	0.591494
P	10.790850	−1.484932	2.170907
O	11.339636	−1.002510	0.739282
O	11.753512	−1.483216	3.304683
O	10.268680	−2.924117	1.696815
C	10.101361	−4.031621	2.655283
C	10.979418	−5.218236	2.278088
O	10.474494	−5.901469	1.095668
C	11.363914	−5.698423	−0.037074
C	12.447799	−4.877243	1.970366
C	12.466047	−4.735704	0.440099
O	13.245444	−5.998435	2.428145
H	9.049054	−4.320596	2.610423

H	10.356007	−3.687228	3.660703
H	10.925051	−5.927048	3.112753
H	11.759271	−6.673929	−0.329849
H	12.768297	−3.959448	2.473892
H	12.235360	−3.706381	0.160524
H	13.438035	−4.994687	0.014302
H	14.175785	−5.807112	2.208854
N	10.603086	−5.222047	−1.185158
C	10.105882	−3.938419	−1.426649
N	9.381237	−3.871514	−2.524701
C	9.376377	−5.171187	−3.037428
C	8.747220	−5.720791	−4.193161
O	8.032555	−5.128313	−5.038457
N	9.001376	−7.098690	−4.326631
C	9.785283	−7.851857	−3.474048
N	9.944591	−9.153255	−3.783186
N	10.367518	−7.340491	−2.382635
C	10.129838	−6.024440	−2.216384
H	10.308512	−3.121268	−0.752309
H	8.576266	−7.567820	−5.158912
H	9.593966	−9.560539	−4.661797
H	10.480007	−9.730337	−3.148758
O	5.392024	−12.347632	−10.600665
C	6.540365	−13.153885	−10.958159
C	7.389980	−13.429809	−9.723963
O	8.025003	−12.198697	−9.245246
C	7.578716	−11.894814	−7.902802
C	6.617796	−13.996737	−8.517068
C	6.331604	−12.759010	−7.674988
O	7.532243	−14.960967	−7.860381
H	4.956231	−12.069625	−11.425159
H	7.161296	−12.633693	−11.698077
H	6.221080	−14.117038	−11.381038
H	8.184004	−14.126148	−10.012931
H	8.372163	−12.122737	−7.190497
H	5.720427	−14.546756	−8.801808
H	5.443162	−12.276323	−8.088178
H	6.146229	−12.972943	−6.624892
N	7.344388	−10.453038	−7.768083
C	8.120998	−9.705202	−6.838877
O	9.059956	−10.268772	−6.233314
N	7.791303	−8.406296	−6.627100
C	6.796764	−7.814281	−7.320491
N	6.514030	−6.535499	−7.038483
C	6.060198	−8.530604	−8.323246
C	6.364587	−9.840362	−8.509806
H	7.035904	−6.030333	−6.305730
H	5.766694	−6.062996	−7.530213
H	5.289483	−8.045595	−8.910816
H	5.871144	−10.465151	−9.248087
P	7.408209	−15.515325	−6.361486
O	5.869925	−15.418299	−5.918057
O	8.086850	−16.833750	−6.246645
O	7.974541	−14.366784	−5.386711
C	9.426803	−14.259767	−5.157895
C	9.696286	−13.863333	−3.712928
O	9.410642	−12.455563	−3.487712
C	8.364000	−12.283123	−2.502935
C	8.871002	−14.650995	−2.676780
C	7.760179	−13.679163	−2.271535
O	9.793794	−14.985934	−1.579148
H	9.821063	−13.499521	−5.836421
H	9.893868	−15.225988	−5.364223
H	10.765113	−14.021922	−3.533174
H	8.777306	−11.853825	−1.589362
H	8.482200	−15.588919	−3.073467
H	6.901465	−13.848892	−2.924152
H	7.437872	−13.813752	−1.240072
N	7.408996	−11.283615	−3.006947
C	7.281208	−10.024097	−2.346650
O	7.897033	−9.829848	−1.280544
N	6.473834	−9.087582	−2.910066
C	5.782899	−9.340535	−4.038820
N	4.995639	−8.376734	−4.528962
C	5.892793	−10.604621	−4.708123
C	6.732833	−11.523598	−4.173691
H	4.978341	−7.435116	−4.099147
H	4.494683	−8.532756	−5.393536
H	5.343985	−10.804585	−5.619647
H	6.922207	−12.483347	−4.636976
P	9.381337	−15.711124	−0.212671
O	8.050331	−16.506559	−0.614944
O	10.523251	−16.483646	0.347541
O	8.809629	−14.570197	0.764271
C	9.773382	−13.682034	1.433886
C	9.017003	−12.724322	2.328243
O	8.247026	−11.812211	1.500225
C	6.988745	−11.556604	2.171169
C	8.028881	−13.380477	3.332460
C	6.649200	−12.878414	2.873917
O	8.390318	−12.903286	4.653294
H	10.331721	−13.128298	0.672615
H	10.462907	−14.287226	2.029253
H	9.761512	−12.155415	2.902785
H	7.100739	−10.740019	2.888041
H	8.091408	−14.472202	3.305158
H	6.214582	−13.595337	2.171816
H	5.957411	−12.742672	3.708590
H	7.832461	−13.374398	5.298998
N	6.029022	−11.086758	1.174715
C	5.617460	−9.742928	1.215649
O	5.973190	−8.952400	2.094590
N	4.762482	−9.376861	0.196873
C	4.248692	−10.198331	−0.801384
O	3.433928	−9.745366	−1.632939
C	4.720266	−11.573689	−0.794049
C	4.201602	−12.532677	−1.829795
C	5.590814	−11.937287	0.180568
H	4.434603	−8.376029	0.222949
H	3.114624	−12.647838	−1.737675
H	4.661033	−13.517893	−1.713727
H	4.398554	−12.166391	−2.842418
H	5.989099	−12.942641	0.235588
H	5.322625	−16.155880	−6.251854
H	7.719829	−17.108406	0.080428
H	11.680234	−0.086222	0.740511
H	4.229816	3.716025	0.474870

d(ATA)$^{H+}$

E: −27697.50 kcal mol^{-1}

O	−0.035096	−0.222043	−0.143007
C	−0.107959	−0.016450	1.288124
C	1.278149	−0.144878	1.908533
O	1.729086	−1.536024	1.973235
C	2.824860	−1.778225	1.063159
C	2.380777	0.617783	1.155852
C	3.064410	−0.456352	0.306164
O	3.254516	1.209780	2.187467
H	−0.944625	−0.225712	−0.489716
H	−0.772385	−0.754893	1.753789
H	−0.488251	0.990547	1.510639
H	1.213226	0.215690	2.939726
H	3.701654	−2.096513	1.634515
H	1.989931	1.437160	0.552543
H	2.580366	−0.477441	−0.671268

H	4.126043	−0.267410	0.158480
N	2.503794	−2.902692	0.182657
C	1.544084	−2.933273	−0.825023
N	1.456476	−4.113090	−1.416378
C	2.396068	−4.909985	−0.765290
C	2.771488	−6.264722	−0.931452
N	2.233897	−7.082715	−1.851676
N	3.729857	−6.751666	−0.093986
C	4.274356	−5.948228	0.839044
N	3.998322	−4.654896	1.075074
C	3.048868	−4.183097	0.242613
H	0.934013	−2.065359	−1.036861
H	2.553034	−8.055140	−1.928074
H	1.551022	−6.723108	−2.504966
H	5.036216	−6.413481	1.460988
P	4.629710	1.981639	1.887084
O	4.599311	2.436673	0.350469
O	4.872926	3.030599	2.913900
O	5.750364	0.834594	1.781032
C	6.034274	0.060069	2.997306
C	7.040117	−1.021468	2.671412
O	6.423686	−2.024604	1.817140
C	7.400176	−2.418093	0.815454
C	8.319770	−0.535284	1.935908
C	8.179201	−1.130113	0.535211
O	9.444930	−1.114924	2.685563
H	5.106746	−0.389249	3.364255
H	6.444046	0.730102	3.758832
H	7.338190	−1.486042	3.619349
H	8.052734	−3.201494	1.209610
H	8.430861	0.548764	1.937904
H	7.589561	−0.438283	−0.072299
H	9.130886	−1.296336	0.034345
N	6.702009	−3.016774	−0.311752
C	6.845591	−4.402828	−0.520491
O	7.553915	−5.121841	0.185813
N	6.130870	−4.891263	−1.591472
C	5.278290	−4.182691	−2.429391
O	4.671404	−4.770321	−3.348603
C	5.165144	−2.757356	−2.155367
C	4.276593	−1.912262	−3.025552
C	5.879411	−2.255183	−1.116737
H	6.249562	−5.929552	−1.775259
H	4.604186	−1.952824	−4.071439
H	4.289291	−0.868520	−2.700086
H	3.244326	−2.278714	−2.999652
H	5.833850	−1.205134	−0.856794
P	11.010271	−0.961514	2.395079
O	11.088479	−0.082693	1.058202
O	11.771693	−0.471232	3.576990
O	11.460388	−2.411272	1.867081
C	11.545606	−3.517808	2.834438
C	12.185378	−4.724250	2.170546
O	11.280044	−5.298916	1.181407
C	11.887101	−5.270577	−0.133670
C	13.513129	−4.444251	1.438621
C	13.086472	−4.313960	−0.028620
O	14.369445	−5.593188	1.662314
H	10.534094	−3.766813	3.169051
H	12.150977	−3.198502	3.688147
H	12.350075	−5.471259	2.956432
H	12.189304	−6.284052	−0.414626
H	13.998724	−3.535838	1.809714
H	12.776101	−3.285720	−0.227912
H	13.885431	−4.579547	−0.724732
H	15.199816	−5.441731	1.174593
N	10.885020	−4.868581	−1.111825
C	10.303023	−3.616726	−1.255403
N	9.415028	−3.566167	−2.233804

C	9.402934	−4.854965	−2.766684
C	8.690211	−5.438008	−3.841588
N	7.807347	−4.773952	−4.607112
N	8.932631	−6.752251	−4.109298
C	9.813534	−7.438512	−3.359785
N	10.545031	−6.983963	−2.328602
C	10.303519	−5.682452	−2.076804
H	10.577427	−2.798882	−0.605131
H	7.286191	−5.270020	−5.340507
H	7.595995	−3.806636	−4.403726
H	9.932481	−8.486142	−3.629697
O	5.659856	−11.889947	−10.431665
C	6.852995	−12.667054	−10.694810
C	7.531443	−13.048795	−9.385345
O	8.121966	−11.876861	−8.734045
C	7.494451	−11.638858	−7.450058
C	6.602907	−13.683497	−8.333265
C	6.229869	−12.507060	−7.437547
O	7.414672	−14.717582	−7.651678
H	5.338153	−11.543272	−11.282226
H	7.563485	−12.090552	−11.299982
H	6.600013	−13.591466	−11.233224
H	8.345164	−13.741490	−9.623472
H	8.186263	−11.905073	−6.649004
H	5.739438	−14.187212	−8.768574
H	5.394378	−11.984708	−7.908167
H	5.921044	−12.790727	−6.434733
N	7.245341	−10.203221	−7.283663
C	7.936447	−9.512369	−6.274363
O	8.784071	−10.039766	−5.547752
N	7.578464	−8.185707	−6.150826
C	6.685336	−7.478634	−6.947769
O	6.478170	−6.263424	−6.729086
C	6.051576	−8.237090	−8.013482
C	5.098875	−7.539577	−8.946019
C	6.366025	−9.553126	−8.129933
H	8.076584	−7.650785	−5.389618
H	5.597697	−6.714101	−9.468841
H	4.256486	−7.106802	−8.392401
H	4.704589	−8.236828	−9.690803
H	5.957427	−10.173569	−8.922102
P	7.087171	−15.435272	−6.259311
O	5.527654	−15.219734	−5.959973
O	7.604675	−16.830073	−6.248073
O	7.693049	−14.474685	−5.115701
C	9.154613	−14.451740	−4.930299
C	9.495139	−14.131387	−3.481497
O	9.317894	−12.720265	−3.178829
C	8.229075	−12.512309	−2.249892
C	8.658054	−14.903490	−2.446961
C	7.593674	−13.895142	−2.002231
O	9.585854	−15.295362	−1.372229
H	9.576008	−13.691380	−5.593082
H	9.561190	−15.434110	−5.186082
H	10.557302	−14.362653	−3.349732
H	8.621103	−12.066238	−1.332620
H	8.221367	−15.815289	−2.855236
H	6.705243	−14.035056	−2.620173
H	7.309129	−14.017214	−0.958115
N	7.297167	−11.530125	−2.798894
C	6.436798	−11.704075	−3.876149
N	5.775993	−10.606636	−4.195866
C	6.223725	−9.646335	−3.290172
C	5.900623	−8.279509	−3.113724
N	5.024968	−7.610964	−3.878870
N	6.530328	−7.620480	−2.101157
C	7.427629	−8.263352	−1.334222
N	7.813805	−9.547573	−1.423466
C	7.179118	−10.195514	−2.418847

H	6.361308	−12.657750	−4.375182
H	4.849703	−6.612497	−3.714820
H	4.535597	−8.094555	−4.619015
H	7.883552	−7.659319	−0.553284
P	9.139935	−16.098824	−0.050621
O	7.784496	−16.879616	−0.401956
O	10.284871	−16.891267	0.473725
O	8.523862	−15.004319	0.945950
C	9.436708	−13.996209	1.506399
C	8.624442	−12.926950	2.199536
O	7.884256	−12.166768	1.209540
C	6.719527	−11.641793	1.893941
C	7.592592	−13.422784	3.256036
C	6.260710	−12.796347	2.795324
O	8.042385	−12.934757	4.543368
H	10.029676	−13.556970	0.698271
H	10.101824	−14.484232	2.224705
H	9.333938	−12.261193	2.713172
H	6.992474	−10.760090	2.478828
H	7.532082	−14.514161	3.278390
H	5.684593	−13.526778	2.219901
H	5.653026	−12.454765	3.636468
H	7.497203	−13.362786	5.228384
N	5.761282	−11.182183	0.893740
C	5.438123	−9.815133	0.849292
O	5.913513	−8.979493	1.625180
N	4.533172	−9.476277	−0.132180
C	3.914953	−10.331260	−1.038905
O	3.081947	−9.880970	−1.853616
C	4.308161	−11.729687	−0.956244
C	3.694657	−12.725812	−1.901216
C	5.201157	−12.078336	0.003770
H	4.256877	−8.456198	−0.152790
H	2.615100	−12.814307	−1.726655
H	4.145797	−13.713765	−1.771750
H	3.827716	−12.407660	−2.940328
H	5.532586	−13.102421	0.122733
H	5.007707	−16.042809	−6.041438
H	7.929873	−17.822842	−0.611055
H	11.901563	0.456599	1.001153
H	4.448056	3.395778	0.242107

d(ATC)$^{H+}$

E: −27609.92 kcal mol^{-1}

O	0.021531	−0.108173	−0.198480
C	−0.075721	0.071436	1.234422
C	1.299415	−0.069376	1.876382
O	1.747312	−1.461752	1.929836
C	2.854720	−1.695164	1.030942
C	2.416617	0.701334	1.154057
C	3.115526	−0.361478	0.301901
O	3.272205	1.277393	2.209604
H	−0.881931	−0.108560	−0.560553
H	−0.748711	−0.674388	1.675379
H	−0.458970	1.074736	1.468383
H	1.217373	0.276597	2.911364
H	3.719852	−2.029690	1.610344
H	2.037944	1.529498	0.555082
H	2.655659	−0.363498	−0.687419
H	4.181233	−0.173909	0.183292
N	2.537466	−2.801951	0.126778
C	1.599709	−2.803446	−0.901874
N	1.487295	−3.980436	−1.494310
C	2.386242	−4.806000	−0.821934
C	2.710230	−6.175917	−0.970932
N	2.147694	−6.979829	−1.888182
N	3.638078	−6.692961	−0.116659
C	4.195263	−5.903357	0.820786
N	3.958011	−4.600688	1.049653
C	3.040964	−4.099059	0.198363
H	1.020249	−1.918254	−1.126587
H	2.427848	−7.964962	−1.953564
H	1.487036	−6.598358	−2.551946
H	4.933063	−6.389746	1.455429
P	4.644687	2.066534	1.943327
O	4.636161	2.540308	0.412177
O	4.860044	3.104471	2.987597
O	5.779015	0.932101	1.841922
C	6.057381	0.147962	3.052600
C	7.038627	−0.950277	2.710177
O	6.396657	−1.915240	1.836967
C	7.397707	−2.421772	0.921243
C	8.338698	−0.485419	1.991445
C	8.300689	−1.212451	0.643649
O	9.447841	−0.892510	2.867454
H	5.124989	−0.285327	3.426118
H	6.487120	0.805939	3.813421
H	7.324487	−1.440660	3.649759
H	7.954880	−3.247155	1.372807
H	8.396576	0.596889	1.879357
H	7.844264	−0.548027	−0.094589
H	9.286869	−1.493599	0.280451
N	6.716709	−2.985698	−0.237856
C	6.816454	−4.372643	−0.468966
O	7.489217	−5.125998	0.235618
N	6.111153	−4.815047	−1.565854
C	5.320044	−4.059078	−2.421670
O	4.727379	−4.605545	−3.373729
C	5.250504	−2.634262	−2.124193
C	4.421741	−1.742486	−3.006125
C	5.947491	−2.177894	−1.053751
H	6.203360	−5.852221	−1.779780
H	4.777881	−1.780168	−4.042705
H	4.463792	−0.705073	−2.663687
H	3.375972	−2.070320	−3.016368
H	5.930888	−1.132528	−0.771134
P	11.010813	−0.872802	2.519834
O	11.126293	0.049999	1.215809
O	11.829857	−0.498897	3.704595
O	11.331735	−2.331484	1.919463
C	11.311094	−3.467972	2.858533
C	11.833063	−4.709793	2.164072
O	10.883561	−5.132288	1.145082
C	11.571676	−5.336466	−0.112730
C	13.207364	−4.559317	1.470354
C	12.854369	−4.495724	−0.021918
O	13.980729	−5.739630	1.807561
H	10.279950	−3.626788	3.188599
H	11.942419	−3.229750	3.719765
H	11.905314	−5.495822	2.927110
H	11.785315	−6.396381	−0.255732
H	13.734882	−3.660741	1.805487
H	12.662999	−3.457707	−0.307437
H	13.651333	−4.889116	−0.657478
H	14.855950	−5.648114	1.388155
N	10.659067	−4.959715	−1.198010
C	10.168355	−5.947496	−2.098620
O	10.532931	−7.137022	−1.964479
N	9.323003	−5.551746	−3.085711
C	8.931543	−4.267022	−3.198779
N	8.092672	−3.949978	−4.195128
C	9.382171	−3.266404	−2.274577
C	10.234536	−3.662654	−1.297225
H	7.741081	−4.678465	−4.837513
H	7.784065	−2.993609	−4.308459

H	9.052880	−2.237328	−2.352476
H	10.614270	−2.988337	−0.539635
O	5.448568	−11.955751	−10.540479
C	6.583206	−12.821896	−10.777193
C	7.222864	−13.230890	−9.454901
O	7.858830	−12.088145	−8.797238
C	7.164596	−11.761617	−7.566837
C	6.252464	−13.814021	−8.410719
C	5.878700	−12.596539	−7.572006
O	7.009088	−14.870106	−7.688962
H	5.173100	−11.588285	−11.398407
H	7.341200	−12.307671	−11.381216
H	6.271828	−13.734197	−11.306472
H	8.007371	−13.960656	−9.679256
H	7.808612	−12.008616	−6.720240
H	5.391014	−14.313318	−8.854721
H	5.086295	−12.068843	−8.106676
H	5.515665	−12.829172	−6.573420
N	6.951042	−10.329359	−7.467321
C	6.081004	−9.514889	−8.193726
N	6.124389	−8.255260	−7.799647
C	7.061707	−8.228305	−6.762387
C	7.519925	−7.163164	−5.933222
O	7.176070	−5.956511	−5.975883
N	8.458467	−7.602352	−4.976702
C	8.927270	−8.897234	−4.862638
N	9.868798	−9.143782	−3.920356
N	8.512486	−9.891286	−5.651831
C	7.590145	−9.508116	−6.551298
H	5.481333	−9.915041	−9.000189
H	8.790434	−6.879326	−4.295847
H	10.056084	−8.445274	−3.187538
H	10.001266	−10.124650	−3.690798
P	6.983270	−15.269853	−6.143248
O	5.494043	−15.071441	−5.576566
O	7.620392	−16.601291	−5.957887
O	7.655867	−14.067177	−5.315388
C	9.115940	−13.998471	−5.146720
C	9.475063	−13.797689	−3.678991
O	9.217967	−12.440886	−3.221988
C	8.071065	−12.399111	−2.336506
C	8.708290	−14.716257	−2.713602
C	7.549763	−13.842936	−2.216081
O	9.662478	−15.087325	−1.655676
H	9.474098	−13.152462	−5.737356
H	9.567807	−14.925836	−5.507325
H	10.552624	−13.968447	−3.589749
H	8.392976	−11.994335	−1.374949
H	8.365988	−15.636779	−3.188090
H	6.688193	−14.010695	−2.863648
H	7.256932	−14.071276	−1.191915
N	7.083133	−11.452293	−2.846924
C	6.195468	−11.613276	−3.907641
N	5.533828	−10.509391	−4.200033
C	6.012454	−9.556755	−3.301941
C	5.736290	−8.178346	−3.130617
N	4.869011	−7.485830	−3.883049
N	6.413433	−7.531201	−2.141614
C	7.306557	−8.194269	−1.385829
N	7.651565	−9.488432	−1.474424
C	6.978079	−10.121427	−2.453434
H	6.096138	−12.559079	−4.415273
H	4.767447	−6.473888	−3.745718
H	4.384443	−7.944933	−4.642111
H	7.796569	−7.598160	−0.620006
P	9.283008	−15.992178	−0.381222
O	8.002959	−16.867972	−0.788459
O	10.489539	−16.709166	0.112355
O	8.570139	−15.006911	0.664514
C	9.391796	−13.966872	1.301940
C	8.490369	−12.977452	2.003423
O	7.738909	−12.219843	1.024395
C	6.605447	−11.675454	1.745611
C	7.451791	−13.565959	3.001676
C	6.138100	−12.828092	2.650123
O	7.943127	−13.289383	4.333828
H	9.989298	−13.458683	0.538953
H	10.055996	−14.442251	2.029283
H	9.141813	−12.294006	2.570222
H	6.917573	−10.806493	2.329964
H	7.341065	−14.645411	2.869799
H	5.463495	−13.494499	2.105851
H	5.623439	−12.466878	3.543602
H	7.418313	−13.820171	4.960054
N	5.631175	−11.183267	0.776703
C	5.310513	−9.814684	0.778293
O	5.805966	−9.002287	1.565771
N	4.383928	−9.447086	−0.173069
C	3.734455	−10.278950	−1.080578
O	2.883982	−9.805021	−1.863073
C	4.118877	−11.681660	−1.039009
C	3.472174	−12.653554	−1.987176
C	5.041274	−12.056206	−0.117398
H	4.121842	−8.424060	−0.172714
H	2.398982	−12.745340	−1.778104
H	3.926157	−13.644724	−1.896547
H	3.571399	−12.311048	−3.022194
H	5.376492	−13.083336	−0.036106
H	11.966327	0.547021	1.168959
H	4.479681	3.499579	0.313055
H	4.857068	−15.735640	−5.905259
H	8.228196	−17.792976	−1.008078

d(ATG)$^{H+}$

E: −27607.79 kcal mol^{-1}

O	−0.011246	−0.216337	−0.181113
C	−0.111644	−0.035682	1.251485
C	1.265479	−0.153646	1.893996
O	1.736540	−1.538454	1.945332
C	2.849088	−1.751120	1.047426
C	2.369848	0.637000	1.173572
C	3.087406	−0.412212	0.320327
O	3.215591	1.225507	2.230544
H	−0.914602	−0.233404	−0.543075
H	−0.772567	−0.791590	1.693559
H	−0.511207	0.961620	1.483782
H	1.176854	0.189463	2.929434
H	3.719369	−2.071170	1.627327
H	1.977507	1.459949	0.576167
H	2.628076	−0.421256	−0.669183
H	4.149964	−0.206929	0.202388
N	2.553794	−2.861682	0.141082
C	1.622787	−2.878557	−0.893327
N	1.545542	−4.052723	−1.497063
C	2.462302	−4.860077	−0.826772
C	2.830513	−6.216799	−0.991088
N	2.306483	−7.024476	−1.928207
N	3.762356	−6.717234	−0.131892
C	4.288477	−5.923384	0.819841
N	4.014264	−4.630018	1.058729
C	3.090772	−4.145491	0.204702
H	1.021863	−2.006788	−1.113968
H	2.624599	−7.996929	−2.009177
H	1.650513	−6.651056	−2.601062
H	5.031071	−6.397480	1.458085

P	4.574340	2.038167	1.964481
O	4.551485	2.522175	0.436656
O	4.776193	3.073241	3.014401
O	5.726539	0.923399	1.852521
C	6.016292	0.133700	3.057358
C	7.023925	−0.939823	2.712504
O	6.408593	−1.920642	1.836904
C	7.416890	−2.368622	0.893394
C	8.313505	−0.441855	1.996791
C	8.256130	−1.115453	0.624348
O	9.432039	−0.885714	2.845517
H	5.091590	−0.323678	3.421408
H	6.426340	0.794498	3.826716
H	7.319376	−1.423883	3.652283
H	8.019126	−3.171189	1.327811
H	8.372323	0.644348	1.931034
H	7.738961	−0.441701	−0.064224
H	9.235579	−1.335708	0.205365
N	6.748228	−2.952186	−0.259379
C	6.882928	−4.336920	−0.479406
O	7.565055	−5.070061	0.238494
N	6.187272	−4.807210	−1.569437
C	5.369354	−4.082392	−2.425437
O	4.774010	−4.656094	−3.360805
C	5.260587	−2.658244	−2.137844
C	4.397942	−1.796072	−3.017404
C	5.951254	−2.173665	−1.075932
H	6.305331	−5.843710	−1.759052
H	4.758896	−1.813333	−4.052944
H	4.397475	−0.759467	−2.669326
H	3.366008	−2.164489	−3.031577
H	5.903801	−1.127269	−0.800930
P	10.989662	−0.922219	2.492090
O	11.114694	0.009617	1.194663
O	11.828041	−0.578958	3.673199
O	11.270490	−2.386547	1.887292
C	11.365318	−3.518252	2.827387
C	12.043373	−4.695232	2.149364
O	11.163364	−5.274132	1.142651
C	11.800277	−5.242900	−0.160043
C	13.372880	−4.366391	1.439027
C	12.964954	−4.246863	−0.034344
O	14.266589	−5.484705	1.674123
H	10.352666	−3.793777	3.136834
H	11.948292	−3.207987	3.699312
H	12.221520	−5.449903	2.925546
H	12.145111	−6.248906	−0.418372
H	13.820152	−3.442617	1.820257
H	12.623261	−3.228820	−0.235559
H	13.783292	−4.483216	−0.718445
H	15.102883	−5.296164	1.210175
N	10.811728	−4.887425	−1.164219
C	10.209521	−3.644937	−1.353141
N	9.349185	−3.640629	−2.351564
C	9.371077	−4.944521	−2.851982
C	8.681996	−5.535672	−3.951742
O	7.857240	−4.989793	−4.722092
N	9.033040	−6.888664	−4.129113
C	9.929327	−7.587376	−3.345653
N	10.164057	−8.873609	−3.680165
N	10.573094	−7.035974	−2.308498
C	10.268813	−5.736064	−2.119979
H	10.452652	−2.806182	−0.718320
H	8.549168	−7.392543	−4.909315
H	9.612012	−9.354040	−4.408689
H	10.686756	−9.433029	−3.018353
O	5.544276	−12.113329	−10.487140
C	6.715017	−12.928785	−10.734246
C	7.412007	−13.259726	−9.420087
O	8.023334	−12.064326	−8.836174
C	7.424998	−11.768781	−7.548329
C	6.496678	−13.837088	−8.322137
C	6.147775	−12.613735	−7.481373
O	7.305821	−14.860038	−7.614317
H	5.219236	−11.792564	−11.346542
H	7.425720	−12.397385	−11.379235
H	6.433265	−13.872090	−11.223447
H	8.214280	−13.971600	−9.640361
H	8.126789	−12.021256	−6.753942
H	5.622152	−14.353015	−8.719215
H	5.318408	−12.104500	−7.977014
H	5.838177	−12.840998	−6.464089
N	7.207324	−10.325999	−7.421747
C	7.933992	−9.597552	−6.439119
O	8.798291	−10.191221	−5.756351
N	7.651192	−8.280128	−6.276485
C	6.744274	−7.659796	−7.058596
N	6.502767	−6.361002	−6.829742
C	6.063171	−8.360304	−8.110155
C	6.324062	−9.684444	−8.253171
H	6.970222	−5.868183	−6.052515
H	5.817916	−5.870483	−7.389982
H	5.367592	−7.851756	−8.767585
H	5.874069	−10.296309	−9.028664
P	7.157086	−15.367362	−6.104554
O	5.634834	−15.167784	−5.639683
O	7.753469	−16.723090	−5.964309
O	7.801999	−14.235665	−5.160877
C	9.264976	−14.175080	−4.999493
C	9.631304	−13.926001	−3.540924
O	9.440973	−12.539085	−3.148983
C	8.313660	−12.398583	−2.253135
C	8.825617	−14.763041	−2.534613
C	7.721077	−13.809627	−2.065524
O	9.768278	−15.141076	−1.467820
H	9.636461	−13.358005	−5.622240
H	9.701736	−15.122789	−5.324699
H	10.699749	−14.143788	−3.443190
H	8.663410	−11.967740	−1.312270
H	8.429850	−15.682124	−2.968228
H	6.845247	−13.956716	−2.699340
H	7.428957	−13.980194	−1.030399
N	7.365085	−11.429725	−2.798004
C	6.532258	−11.589486	−3.900291
N	5.876256	−10.489569	−4.218903
C	6.301442	−9.540818	−3.290981
C	5.980832	−8.174024	−3.110872
N	5.130751	−7.492643	−3.892092
N	6.585707	−7.529409	−2.074259
C	7.450987	−8.186355	−1.281669
N	7.831443	−9.471443	−1.372164
C	7.230287	−10.102056	−2.399229
H	6.476330	−12.532482	−4.419972
H	4.971702	−6.489584	−3.734555
H	4.692715	−7.955994	−4.675965
H	7.882901	−7.591950	−0.480029
P	9.357045	−16.001478	−0.172058
O	8.057749	−16.856605	−0.562479
O	10.543140	−16.733365	0.348597
O	8.659153	−14.974062	0.841948
C	9.497800	−13.938831	1.464317
C	8.608163	−12.928687	2.151485
O	7.878423	−12.167987	1.156292
C	6.703636	−11.657468	1.834834
C	7.552393	−13.499463	3.143950
C	6.230726	−12.830632	2.706586
O	7.980443	−13.125595	4.475302
H	10.101692	−13.449709	0.693848

H	10.155539	−14.414169	2.197436
H	9.264167	−12.250677	2.718272
H	6.969004	−10.787733	2.440807
H	7.485102	−14.588214	3.073222
H	5.633302	−13.531118	2.116195
H	5.636661	−12.499936	3.561700
H	7.433255	−13.620605	5.111800
N	5.759516	−11.177942	0.830592
C	5.427715	−9.812279	0.814863
O	5.879073	−8.994905	1.623784
N	4.543025	−9.453284	−0.178105
C	3.950006	−10.289160	−1.119408
O	3.134472	−9.822994	−1.942748
C	4.347012	−11.687627	−1.060918
C	3.760324	−12.663703	−2.043273
C	5.223394	−12.054847	−0.092597
H	4.270935	−8.432028	−0.187773
H	2.677523	−12.761369	−1.895702
H	4.213376	−13.652492	−1.926287
H	3.915841	−12.320726	−3.071216
H	5.560407	−13.079310	0.006500
H	12.022537	0.330345	1.027465
H	4.388338	3.481150	0.345973
H	5.037548	−15.876506	−5.949028
H	8.265295	−17.784015	−0.789513

d(CAA)$^{H+}$

E: −27606.14 kcal mol^{-1}

O	0.008708	−0.016894	−0.942037
C	−0.036577	0.409837	0.440772
C	1.296697	0.125998	1.121728
O	1.502428	−1.311886	1.300311
C	2.664077	−1.757847	0.552925
C	2.536001	0.631422	0.359367
C	3.016074	−0.604292	−0.395137
O	3.482015	1.098638	1.398193
H	−0.894944	0.036651	−1.299275
H	−0.825553	−0.124475	0.984242
H	−0.233503	1.489420	0.505435
H	1.265017	0.584689	2.115603
H	3.476417	−1.987261	1.243545
H	2.326060	1.481312	−0.290454
H	2.441076	−0.669968	−1.321270
H	4.070501	−0.584794	−0.658859
N	2.356187	−3.025214	−0.117227
C	3.015363	−4.216613	0.300058
O	3.827124	−4.163699	1.250110
N	2.737343	−5.370438	−0.360534
C	1.837729	−5.400362	−1.365061
N	1.627589	−6.569121	−1.987272
C	1.114737	−4.223002	−1.752652
C	1.400856	−3.067407	−1.100996
H	2.172819	−7.408536	−1.735350
H	0.973218	−6.616090	−2.757285
H	0.360416	−4.255132	−2.530479
H	0.884920	−2.133691	−1.301214
P	5.038229	1.416539	1.217612
O	5.297615	1.578222	−0.355891
O	5.466426	2.535145	2.100572
O	5.799940	0.020324	1.478635
C	5.874120	−0.482093	2.860597
C	7.101921	−1.365087	3.034137
O	6.931883	−2.671072	2.418448
C	7.797827	−2.825060	1.267376
C	8.396086	−0.780187	2.442788
C	8.524068	−1.478911	1.086225

O	9.468771	−1.117398	3.395379
H	4.966788	−1.054775	3.070782
H	5.943816	0.367535	3.546047
H	7.222338	−1.525226	4.110829
H	8.489907	−3.650836	1.455850
H	8.369543	0.306364	2.352331
H	8.028339	−0.861113	0.336206
H	9.557652	−1.619754	0.776265
N	7.013124	−3.232128	0.106776
C	6.163667	−2.452009	−0.668826
N	5.520261	−3.146682	−1.591090
C	5.957820	−4.458380	−1.418286
C	5.646170	−5.664575	−2.090363
N	4.771891	−5.756940	−3.103318
N	6.294228	−6.788786	−1.674075
C	7.167749	−6.724429	−0.652704
N	7.513750	−5.643456	0.063733
C	6.881556	−4.535651	−0.364307
H	6.065839	−1.391401	−0.496508
H	4.588647	−6.664116	−3.551465
H	4.287715	−4.929075	−3.421724
H	7.641827	−7.667738	−0.391657
P	11.040284	−0.932232	3.115251
O	11.184441	0.156645	1.947367
O	11.767845	−0.677935	4.388152
O	11.489592	−2.238841	2.299175
C	11.461446	−3.530565	3.003978
C	11.912105	−4.619053	2.052900
O	10.882086	−4.830059	1.043451
C	11.532643	−4.989773	−0.242806
C	13.237971	−4.330965	1.298939
C	12.783456	−4.107898	−0.150887
O	14.073040	−5.507439	1.443540
H	10.442280	−3.725876	3.350672
H	12.138774	−3.480524	3.861718
H	12.033811	−5.538591	2.640778
H	11.788624	−6.042458	−0.406823
H	13.753692	−3.456351	1.705440
H	12.515295	−3.056781	−0.291813
H	13.549304	−4.381135	−0.880360
H	14.930179	−5.313626	1.021649
N	10.593261	−4.633311	−1.287846
C	10.102500	−3.368871	−1.590740
N	9.227320	−3.376781	−2.580345
C	9.130263	−4.715271	−2.957132
C	8.373629	−5.370361	−3.957403
N	7.535709	−4.735733	−4.795978
N	8.524288	−6.720343	−4.069241
C	9.357355	−7.371924	−3.237528
N	10.116265	−6.850244	−2.258719
C	9.967273	−5.514518	−2.163862
H	10.433717	−2.490724	−1.056555
H	6.941384	−5.281614	−5.431931
H	7.344633	−3.752436	−4.654364
H	9.405956	−8.449140	−3.379450
O	5.923177	−11.689052	−10.688781
C	7.205406	−12.321927	−10.918085
C	7.786784	−12.813874	−9.598211
O	8.247358	−11.697548	−8.771637
C	7.502531	−11.619994	−7.534323
C	6.803551	−13.613722	−8.723225
C	6.328819	−12.603457	−7.678727
O	7.574436	−14.737293	−8.152555
H	5.646249	−11.266253	−11.520426
H	7.910969	−11.614681	−11.371127
H	7.092625	−13.185715	−11.588304
H	8.659216	−13.433704	−9.829661
H	8.152382	−11.873082	−6.694471
H	5.981708	−14.042689	−9.296728

H	5.441988	−12.101516	−8.069778
H	6.068939	−13.062992	−6.727192
N	7.093031	−10.225053	−7.300780
C	7.666130	−9.521009	−6.228082
O	8.492410	−10.013982	−5.454731
N	7.214880	−8.224362	−6.092401
C	6.323562	−7.552773	−6.921613
O	6.027972	−6.360443	−6.687575
C	5.806755	−8.320388	−8.042891
C	4.853836	−7.664909	−9.004632
C	6.226629	−9.603850	−8.183754
H	7.660187	−7.673901	−5.309396
H	5.303224	−6.769461	−9.451330
H	3.940229	−7.342466	−8.489387
H	4.575936	−8.354499	−9.806823
H	5.918348	−10.217784	−9.024640
P	7.026214	−15.760058	−7.042586
O	5.427435	−15.646325	−7.043385
O	7.621963	−17.110624	−7.231854
O	7.330034	−15.060581	−5.627311
C	8.739538	−14.923027	−5.233389
C	8.809308	−14.287837	−3.860996
O	8.392240	−12.899097	−3.947635
C	7.505631	−12.606509	−2.835939
C	7.920767	−14.955800	−2.778820
C	6.804378	−13.938543	−2.549335
O	8.794351	−15.134642	−1.607062
H	9.255954	−14.292375	−5.963334
H	9.202816	−15.913901	−5.209808
H	9.855209	−14.333648	−3.533534
H	8.080338	−12.260860	−1.972832
H	7.553420	−15.938847	−3.073009
H	6.015863	−14.135008	−3.280229
H	6.362366	−13.988776	−1.556526
N	6.641075	−11.494498	−3.205941
C	6.861053	−10.243937	−2.594219
O	7.728825	−10.055241	−1.740116
N	6.014951	−9.245233	−3.023964
C	5.020946	−9.342204	−3.990092
O	4.344279	−8.341822	−4.303223
C	4.858205	−10.654504	−4.598904
C	3.796788	−10.845446	−5.646568
C	5.677826	−11.651384	−4.181313
H	6.133516	−8.313225	−2.536013
H	2.806612	−10.605301	−5.241452
H	3.781188	−11.877910	−6.005157
H	3.963237	−10.177619	−6.499641
H	5.617713	−12.645782	−4.605712
P	8.391254	−15.650620	−0.148076
O	6.850091	−16.068529	−0.277512
O	9.336732	−16.679186	0.367081
O	8.300272	−14.322007	0.752644
C	9.555961	−13.665466	1.155110
C	9.271757	−12.643488	2.242041
O	8.539564	−11.508732	1.694007
C	7.255039	−11.370069	2.354503
C	8.444153	−13.167345	3.432689
C	7.022825	−12.688929	3.111026
O	8.978142	−12.541670	4.627650
H	9.985700	−13.172586	0.277950
H	10.249164	−14.423617	1.531751
H	10.243231	−12.281895	2.600857
H	7.289263	−10.508730	3.028159
H	8.503438	−14.257146	3.521473
H	6.529937	−13.420434	2.466635
H	6.414135	−12.545497	4.006904
H	8.440956	−12.843434	5.383160
N	6.239252	−11.062096	1.360632
C	5.723722	−11.916533	0.387054

N	4.820709	−11.334738	−0.376535
C	4.730447	−10.026759	0.103849
C	3.909439	−8.934771	−0.305695
O	3.068820	−8.914268	−1.235310
N	4.128240	−7.786828	0.480784
C	5.028548	−7.695796	1.521023
N	5.082170	−6.518516	2.186428
N	5.806500	−8.716112	1.903131
C	5.612303	−9.836918	1.178481
H	6.065591	−12.937588	0.305636
H	3.605497	−6.924282	0.198472
H	4.645580	−5.669270	1.791568
H	5.878639	−6.391869	2.799660
H	4.992521	−16.446941	−7.396173
H	6.593201	−16.784842	0.335432
H	11.548660	1.005408	2.265932
H	5.689308	2.443191	−0.586042

d(CAC)$^{H+}$

E: −27518.05 kcal mol^{-1}

O	0.117500	−0.010590	−1.025996
C	0.014417	0.364799	0.368378
C	1.324032	0.069495	1.088941
O	1.540108	−1.371711	1.222105
C	2.732594	−1.778315	0.500911
C	2.585192	0.617363	0.394552
C	3.110147	−0.584610	−0.385189
O	3.478281	1.074740	1.484925
H	−0.771402	0.052022	−1.417232
H	−0.788515	−0.197042	0.861513
H	−0.197162	1.439309	0.464238
H	1.249020	0.489419	2.097410
H	3.520068	−2.029547	1.212153
H	2.386394	1.484690	−0.235676
H	2.573005	−0.617051	−1.335416
H	4.173423	−0.543705	−0.606941
N	2.462063	−3.021588	−0.228168
C	3.101809	−4.225865	0.182803
O	3.875082	−4.200490	1.165655
N	2.847730	−5.361358	−0.517552
C	1.992126	−5.361630	−1.560402
N	1.796275	−6.515278	−2.214147
C	1.300137	−4.168377	−1.956178
C	1.557094	−3.032647	−1.259341
H	2.288327	−7.376726	−1.929986
H	1.166612	−6.542278	−3.005501
H	0.583989	−4.176007	−2.769835
H	1.051855	−2.092884	−1.457642
P	5.058565	1.316063	1.410992
O	5.466494	1.539120	−0.123844
O	5.466967	2.357959	2.391466
O	5.746577	−0.124381	1.614501
C	5.775706	−0.694544	2.972702
C	7.029615	−1.537077	3.165128
O	6.928045	−2.829748	2.509920
C	7.828977	−2.917647	1.380293
C	8.318713	−0.887590	2.634177
C	8.530485	−1.549762	1.269298
O	9.372615	−1.202883	3.614764
H	4.884588	−1.313633	3.104477
H	5.774555	0.120791	3.701440
H	7.119705	−1.723973	4.240408
H	8.534569	−3.732788	1.555203
H	8.248054	0.198305	2.564968
H	8.062731	−0.925552	0.506922
H	9.582873	−1.665761	1.015821

N	7.087289	−3.293226	0.180791
C	6.242293	−2.493368	−0.580139
N	5.640665	−3.153963	−1.553158
C	6.100254	−4.463369	−1.430270
C	5.823020	−5.640847	−2.164485
N	4.986838	−5.689831	−3.211088
N	6.460435	−6.779948	−1.772238
C	7.311165	−6.749224	−0.730467
N	7.636147	−5.694437	0.033882
C	6.997323	−4.574971	−0.356676
H	6.113362	−1.446522	−0.354844
H	4.773936	−6.589559	−3.660824
H	4.488148	−4.855982	−3.489168
H	7.779115	−7.701427	−0.489939
P	10.922761	−0.817112	3.425834
O	10.988296	0.428086	2.417711
O	11.573686	−0.668629	4.755670
O	11.542388	−1.934283	2.455745
C	11.609473	−3.318034	2.951604
C	11.969242	−4.241294	1.809453
O	10.868659	−4.298606	0.867841
C	11.450270	−4.649289	−0.414116
C	13.243761	−3.870342	0.994644
C	12.751775	−3.835688	−0.467388
O	14.220854	−4.909099	1.248708
H	10.638641	−3.597454	3.371923
H	12.375118	−3.375388	3.730748
H	12.131170	−5.240180	2.241798
H	11.642717	−5.723326	−0.459496
H	13.649600	−2.902349	1.300752
H	12.545034	−2.804218	−0.768128
H	13.483800	−4.261402	−1.157942
H	15.069380	−4.615694	0.869520
N	10.471707	−4.369582	−1.460504
C	9.977734	−5.435101	−2.268861
O	10.402059	−6.596366	−2.074948
N	9.068303	−5.142130	−3.231509
C	8.618858	−3.883037	−3.411129
N	7.722027	−3.670742	−4.382768
C	9.086450	−2.801105	−2.592758
C	10.009847	−3.092241	−1.643910
H	7.380420	−4.450080	−4.966184
H	7.334547	−2.745943	−4.515936
H	8.720389	−1.790705	−2.727665
H	10.419019	−2.341161	−0.980651
O	5.917526	−11.565894	−10.736507
C	7.161174	−12.280974	−10.927274
C	7.645274	−12.861944	−9.603645
O	8.165050	−11.831258	−8.704003
C	7.294556	−11.645651	−7.561165
C	6.565997	−13.604439	−8.799747
C	6.079961	−12.563397	−7.791957
O	7.242592	−14.764568	−8.184380
H	5.698749	−11.121394	−11.574195
H	7.937111	−11.612807	−11.321248
H	7.021024	−13.112722	−11.632523
H	8.474910	−13.540196	−9.826154
H	7.844909	−11.902631	−6.650608
H	5.761755	−13.995015	−9.423603
H	5.259941	−12.009795	−8.251631
H	5.716073	−13.000083	−6.864053
N	6.949145	−10.234030	−7.423462
C	6.134284	−9.470958	−8.265129
N	6.086212	−8.202046	−7.907418
C	6.911634	−8.106935	−6.785380
C	7.259058	−6.986070	−5.974954
O	6.868751	−5.799031	−6.097501
N	8.148560	−7.336607	−4.942978
C	8.667264	−8.600261	−4.739494

N	9.565592	−8.740821	−3.743683
N	8.330944	−9.657357	−5.488435
C	7.468016	−9.357080	−6.477400
H	5.653115	−9.914832	−9.125751
H	8.456093	−6.562560	−4.310233
H	9.781451	−7.966098	−3.099939
H	9.793060	−9.686048	−3.462574
P	6.658531	−15.713922	−7.039917
O	5.072970	−15.498228	−7.105381
O	7.173466	−17.104417	−7.168274
O	7.028824	−14.993819	−5.646883
C	8.446595	−14.984124	−5.256869
C	8.575356	−14.398638	−3.866774
O	8.240842	−12.984255	−3.897748
C	7.341811	−12.683529	−2.798247
C	7.665455	−15.050715	−2.795063
C	6.592713	−13.994381	−2.538440
O	8.538182	−15.296276	−1.633910
H	9.007399	−14.375993	−5.973170
H	8.829132	−16.009117	−5.265285
H	9.621665	−14.515662	−3.559753
H	7.909553	−12.363763	−1.921035
H	7.256279	−16.011146	−3.108625
H	5.792780	−14.144694	−3.267624
H	6.154695	−14.046186	−1.544220
N	6.519952	−11.540278	−3.173265
C	6.761377	−10.309811	−2.531896
O	7.587150	−10.172448	−1.628302
N	5.995720	−9.265683	−3.002959
C	5.042699	−9.310516	−4.013575
O	4.448922	−8.270718	−4.364671
C	4.821838	−10.616125	−4.617091
C	3.777849	−10.750981	−5.689835
C	5.582677	−11.650928	−4.181332
H	6.171424	−8.329023	−2.540791
H	2.797125	−10.437563	−5.312482
H	3.698966	−11.784924	−6.034785
H	4.010272	−10.108333	−6.546778
H	5.495955	−12.639960	−4.614626
P	8.120864	−15.784946	−0.170573
O	6.572543	−16.174543	−0.303918
O	9.046788	−16.825463	0.355403
O	8.052177	−14.448369	0.720658
C	9.319582	−13.819220	1.131435
C	9.056276	−12.811160	2.236497
O	8.346552	−11.652476	1.711120
C	7.056293	−11.511177	2.360789
C	8.220204	−13.338461	3.419185
C	6.805999	−12.834782	3.103371
O	8.762553	−12.737280	4.622791
H	9.754214	−13.318192	0.261317
H	10.001490	−14.595027	1.492250
H	10.035403	−12.474866	2.599311
H	7.094078	−10.656613	3.042584
H	8.262736	−14.430115	3.493523
H	6.301696	−13.553703	2.454101
H	6.200852	−12.691486	4.001607
H	8.221975	−13.043539	5.373983
N	6.051262	−11.182977	1.362040
C	5.498971	−12.032471	0.404143
N	4.632833	−11.423269	−0.380456
C	4.608437	−10.100903	0.067149
C	3.864361	−8.973556	−0.389451
O	3.042002	−8.928762	−1.335108
N	4.145636	−7.816411	0.362588
C	5.030356	−7.750413	1.418850
N	5.137019	−6.564256	2.057320
N	5.744780	−8.802888	1.837719
C	5.492891	−9.930789	1.143054

H	5.785814	−13.071955	0.349261
H	3.666297	−6.936268	0.058322
H	4.709794	−5.706808	1.671072
H	5.917261	−6.463067	2.694715
H	4.563232	−16.282519	−6.822583
H	6.287160	−16.852717	0.339238
H	11.107416	1.282586	2.876298
H	5.481033	2.481364	−0.382869

d(CAG)$^{H+}$

E: −27516.53 kcal mol^{-1}

O	0.041023	−0.018653	−0.924638
C	−0.031557	0.356235	0.471955
C	1.297981	0.073653	1.160751
O	1.530155	−1.365572	1.288889
C	2.708693	−1.761068	0.538993
C	2.536772	0.632056	0.435582
C	3.052042	−0.565313	−0.357891
O	3.455768	1.089405	1.503367
H	−0.859017	0.028106	−1.291891
H	−0.816665	−0.213356	0.984581
H	−0.251612	1.428656	0.573012
H	1.243689	0.493604	2.170588
H	3.515928	−2.002655	1.231203
H	2.316499	1.499288	−0.187548
H	2.491632	−0.603565	−1.294250
H	4.109403	−0.515908	−0.605822
N	2.434252	−3.008289	−0.181098
C	3.091988	−4.205916	0.221773
O	3.878525	−4.171407	1.193436
N	2.839096	−5.344479	−0.473723
C	1.965085	−5.355200	−1.500955
N	1.772386	−6.511447	−2.151052
C	1.249393	−4.171186	−1.882301
C	1.508208	−3.031217	−1.193058
H	2.297339	−7.360230	−1.888306
H	1.134236	−6.545751	−2.935221
H	0.517262	−4.188207	−2.681479
H	0.989081	−2.097273	−1.382729
P	5.024048	1.376705	1.372970
O	5.350295	1.600310	−0.180438
O	5.444542	2.443259	2.321507
O	5.750502	−0.045930	1.578872
C	5.768014	−0.618562	2.935938
C	6.999984	−1.492636	3.124532
O	6.872309	−2.775278	2.454795
C	7.787982	−2.875703	1.336623
C	8.308482	−0.870227	2.608944
C	8.503554	−1.515714	1.233769
O	9.344324	−1.235562	3.591728
H	4.861425	−1.214091	3.072156
H	5.790724	0.195660	3.666012
H	7.080132	−1.691771	4.198428
H	8.483938	−3.697549	1.521803
H	8.271319	0.218530	2.558947
H	8.031064	−0.876785	0.486203
H	9.551930	−1.637548	0.965428
N	7.056168	−3.250474	0.132172
C	6.207417	−2.459368	−0.632321
N	5.600759	−3.131634	−1.595293
C	6.062246	−4.439360	−1.461281
C	5.787847	−5.626378	−2.181994
N	4.936109	−5.699875	−3.214953
N	6.444733	−6.754439	−1.787938
C	7.290316	−6.711667	−0.741708
N	7.601132	−5.650702	0.017987
C	6.962114	−4.538111	−0.389074
H	6.081617	−1.408917	−0.420339
H	4.752695	−6.602656	−3.672456
H	4.443625	−4.870201	−3.515196
H	7.766740	−7.658376	−0.496207
P	10.914353	−0.944160	3.407714
O	11.057503	0.285743	2.388498
O	11.567904	−0.820396	4.738924
O	11.470056	−2.106522	2.453268
C	11.494837	−3.479320	2.982987
C	11.907553	−4.428798	1.879892
O	10.828641	−4.540251	0.910637
C	11.442248	−4.722224	−0.391508
C	13.189807	−4.031692	1.092912
C	12.681027	−3.818706	−0.342415
O	14.113333	−5.144016	1.201620
H	10.499214	−3.740005	3.354786
H	12.216312	−3.526242	3.803938
H	12.081175	−5.410269	2.343507
H	11.716248	−5.773970	−0.534016
H	13.650970	−3.127500	1.499229
H	12.385843	−2.773955	−0.479639
H	13.428590	−4.079213	−1.095362
H	14.954699	−4.868648	0.793699
N	10.476439	−4.411761	−1.423233
C	9.936585	−3.164292	−1.742515
N	9.105780	−3.216590	−2.763864
C	9.088518	−4.557780	−3.150624
C	8.396011	−5.215049	−4.210470
O	7.608508	−4.705821	−5.043674
N	8.685215	−6.591315	−4.256836
C	9.542363	−7.250956	−3.399951
N	9.743226	−8.565852	−3.626794
N	10.187667	−6.637713	−2.398670
C	9.931340	−5.317535	−2.326228
H	10.199381	−2.274384	−1.189773
H	8.182287	−7.150927	−4.985037
H	9.186141	−9.080609	−4.326330
H	10.179283	−9.095252	−2.882294
O	5.786872	−11.738987	−10.830004
C	7.051532	−12.405034	−11.062582
C	7.647613	−12.867117	−9.738479
O	8.139005	−11.734556	−8.953592
C	7.417300	−11.611237	−7.703924
C	6.667553	−13.624235	−8.823504
C	6.221679	−12.573298	−7.807900
O	7.436501	−14.732775	−8.221507
H	5.504516	−11.332894	−11.668207
H	7.762485	−11.726722	−11.550166
H	6.909180	−13.286532	−11.703567
H	8.505468	−13.508451	−9.966202
H	8.075988	−11.858552	−6.870255
H	5.833580	−14.065810	−9.369327
H	5.343172	−12.064570	−8.209117
H	5.958401	−12.997786	−6.840937
N	7.043745	−10.201989	−7.499528
C	7.625876	−9.462491	−6.429209
O	8.417013	−10.036853	−5.649325
N	7.286725	−8.154429	−6.290250
C	6.429018	−7.560605	−7.144004
N	6.125578	−6.271277	−6.936972
C	5.862350	−8.278551	−8.249871
C	6.209028	−9.581917	−8.396509
H	6.604572	−5.728303	−6.202898
H	5.521597	−5.788676	−7.589739
H	5.198792	−7.793723	−8.956472
H	5.868343	−10.191983	−9.227282
P	6.893053	−15.729818	−7.089969
O	5.297356	−15.586349	−7.099333

O	7.461668	−17.095145	−7.257164
O	7.236240	−15.015475	−5.689399
C	8.652712	−14.932113	−5.305012
C	8.757233	−14.329231	−3.919702
O	8.392726	−12.924341	−3.970846
C	7.498997	−12.623300	−2.867906
C	7.854399	−14.987931	−2.844783
C	6.768973	−13.942707	−2.593343
O	8.730009	−15.220452	−1.682955
H	9.182145	−14.302684	−6.026670
H	9.083860	−15.937659	−5.306584
H	9.803722	−14.420690	−3.603948
H	8.068695	−12.289007	−1.997195
H	7.455926	−15.954107	−3.154259
H	5.969066	−14.109603	−3.319072
H	6.333710	−13.992886	−1.597777
N	6.659212	−11.493911	−3.245495
C	6.883510	−10.254762	−2.612668
O	7.712775	−10.098375	−1.715427
N	6.091266	−9.228523	−3.079344
C	5.118877	−9.298710	−4.069479
O	4.479463	−8.279838	−4.400775
C	4.926878	−10.608993	−4.673626
C	3.869041	−10.772664	−5.729308
C	5.715316	−11.625547	−4.243673
H	6.232558	−8.294225	−2.602959
H	2.885387	−10.493417	−5.333277
H	3.817891	−11.807192	−6.077967
H	4.063528	−10.118930	−6.587221
H	5.642670	−12.617671	−4.672000
P	8.319564	−15.697986	−0.214307
O	6.774908	−16.103298	−0.342433
O	9.255823	−16.724675	0.320496
O	8.240601	−14.353427	0.664389
C	9.503152	−13.710175	1.068605
C	9.234272	−12.701759	2.172058
O	8.512264	−11.550520	1.646690
C	7.224612	−11.417435	2.302870
C	8.407121	−13.234714	3.358570
C	6.987778	−12.742360	3.047784
O	8.949625	−12.628705	4.559649
H	9.929615	−13.207963	0.195147
H	10.194104	−14.477964	1.429496
H	10.211445	−12.355803	2.531034
H	7.258548	−10.561860	2.983686
H	8.458622	−14.325943	3.433337
H	6.486345	−13.465004	2.400297
H	6.384933	−12.603134	3.948240
H	8.415351	−12.939835	5.313387
N	6.212488	−11.098407	1.308426
C	5.681023	−11.948134	0.339165
N	4.797300	−11.350874	−0.434796
C	4.738202	−10.036520	0.032580
C	3.956546	−8.925042	−0.399939
O	3.129169	−8.889131	−1.341200
N	4.201362	−7.774789	0.375669
C	5.087872	−7.700414	1.429488
N	5.157363	−6.523648	2.092577
N	5.837057	−8.737001	1.825947
C	5.618820	−9.859086	1.110417
H	5.996957	−12.978151	0.268495
H	3.696313	−6.904036	0.086124
H	4.722624	−5.669936	1.705934
H	5.945505	−6.409295	2.718403
H	4.842016	−16.445716	−7.191527
H	6.490654	−16.762163	0.320865
H	11.224900	1.135844	2.839987
H	5.580611	2.526950	−0.387848

d(CCA)$^{H+}$

E: −27514.85 kcal mol^{-1}

O	−0.205278	0.025428	−0.835153
C	−0.293431	0.415643	0.556424
C	1.039807	0.168864	1.251782
O	1.293048	−1.263555	1.414287
C	2.473089	−1.665420	0.674722
C	2.265839	0.728896	0.508521
C	2.817338	−0.480075	−0.240794
O	3.181164	1.237337	1.554286
H	−1.102373	0.058090	−1.211157
H	−1.068995	−0.163732	1.072369
H	−0.535643	1.484338	0.642478
H	0.981275	0.611786	2.251429
H	3.280242	−1.898508	1.370773
H	2.024639	1.567219	−0.145212
H	2.289294	−0.552896	−1.193864
H	3.881984	−0.406582	−0.449691
N	2.198655	−2.921746	−0.036484
C	2.876551	−4.114481	0.351006
O	3.685213	−4.074651	1.304128
N	2.627147	−5.252202	−0.346847
C	1.746375	−5.266556	−1.367900
N	1.573291	−6.416790	−2.033089
C	1.010274	−4.090018	−1.732939
C	1.260895	−2.951493	−1.038778
H	2.098539	−7.265672	−1.771805
H	0.918370	−6.458221	−2.803165
H	0.271986	−4.110512	−2.526328
H	0.729916	−2.022101	−1.219197
P	4.699155	1.693762	1.365159
O	4.822590	1.972498	−0.207444
O	5.062781	2.794005	2.298667
O	5.599384	0.365838	1.536918
C	5.833726	−0.139484	2.899076
C	6.962203	−1.154445	2.882986
O	6.545513	−2.365290	2.193494
C	7.470450	−2.675890	1.119500
C	8.262219	−0.682984	2.195374
C	8.213109	−1.365156	0.831443
O	9.361151	−1.156593	3.059636
H	4.915376	−0.612020	3.259356
H	6.099215	0.697591	3.550867
H	7.178710	−1.403474	3.928388
H	8.148541	−3.473176	1.429038
H	8.346543	0.402270	2.131426
H	7.638416	−0.723958	0.159178
H	9.191244	−1.521437	0.383054
N	6.716338	−3.218989	−0.009577
C	6.855070	−4.596900	−0.350203
O	7.646851	−5.305836	0.302134
N	6.116588	−5.076965	−1.384418
C	5.276037	−4.282585	−2.075876
N	4.589356	−4.819028	−3.091859
C	5.115101	−2.897414	−1.736789
C	5.841632	−2.422393	−0.695574
H	4.692693	−5.820081	−3.333063
H	3.944940	−4.245391	−3.619440
H	4.427823	−2.261100	−2.280708
H	5.761005	−1.401652	−0.344702
P	10.913845	−1.271049	2.708916
O	11.094130	−0.292287	1.452004
O	11.762877	−1.025901	3.906060
O	11.127319	−2.718105	2.036423
C	11.247623	−3.899263	2.911318
C	12.056235	−4.983237	2.217219

O	11.290340	−5.589171	1.136458
C	11.934049	−5.353154	−0.140031
C	13.387897	−4.516910	1.598376
C	13.038397	−4.313513	0.117941
O	14.344624	−5.588756	1.795612
H	10.239536	−4.262467	3.129829
H	11.746760	−3.605477	3.838882
H	12.250467	−5.760134	2.966654
H	12.332963	−6.299581	−0.516000
H	13.754515	−3.596410	2.064707
H	12.659901	−3.299996	−0.030294
H	13.898449	−4.461281	−0.539070
H	15.182703	−5.316013	1.379057
N	10.928807	−4.932236	−1.107699
C	10.271408	−3.710870	−1.162444
N	9.342310	−3.661679	−2.101001
C	9.372774	−4.923637	−2.692652
C	8.627179	−5.498697	−3.748435
N	7.681234	−4.842176	−4.441539
N	8.907779	−6.791616	−4.077053
C	9.874044	−7.456360	−3.415679
N	10.649319	−7.002143	−2.417797
C	10.349215	−5.730546	−2.087854
H	10.523317	−2.912646	−0.480858
H	7.133837	−5.332539	−5.160839
H	7.411920	−3.911527	−4.152916
H	10.026319	−8.486152	−3.733609
O	5.702991	−11.534039	−10.657616
C	6.935981	−12.235624	−10.947772
C	7.561372	−12.754359	−9.658684
O	8.105997	−11.661059	−8.853050
C	7.414678	−11.561618	−7.581803
C	6.595161	−13.514392	−8.731855
C	6.181188	−12.468867	−7.700375
O	7.379213	−14.631448	−8.157839
H	5.411210	−11.095315	−11.475735
H	7.654245	−11.568282	−11.439944
H	6.743904	−13.094102	−11.606911
H	8.394831	−13.408218	−9.935747
H	8.082023	−11.876313	−6.777226
H	5.750039	−13.954663	−9.261430
H	5.325504	−11.921155	−8.100023
H	5.890236	−12.895494	−6.743803
N	7.108887	−10.152811	−7.304946
C	7.791571	−9.507369	−6.259420
O	8.638454	−10.064463	−5.555505
N	7.433479	−8.186988	−6.078801
C	6.531069	−7.450065	−6.836211
O	6.325462	−6.244202	−6.570182
C	5.896338	−8.162517	−7.932287
C	4.932143	−7.429544	−8.825003
C	6.226158	−9.466228	−8.118428
H	7.956051	−7.680370	−5.316773
H	5.413776	−6.561837	−9.292786
H	4.077652	−7.051360	−8.250019
H	4.556683	−8.087698	−9.613945
H	5.830237	−10.043282	−8.948346
P	6.970713	−15.559178	−6.925841
O	5.376663	−15.413356	−6.852574
O	7.539488	−16.927884	−7.061128
O	7.436578	−14.785241	−5.589448
C	8.861439	−14.830501	−5.225291
C	9.043265	−14.398301	−3.780806
O	8.787890	−12.973997	−3.632264
C	7.724798	−12.744382	−2.669678
C	8.132471	−15.116533	−2.762140
C	7.024563	−14.098649	−2.500551
O	8.988131	−15.407168	−1.596307
H	9.412016	−14.154092	−5.885804
H	9.230259	−15.852466	−5.351998
H	10.091923	−14.589506	−3.525332
H	8.157792	−12.381096	−1.731229
H	7.752660	−16.071939	−3.125881
H	6.265699	−14.237458	−3.273540
H	6.537606	−14.206466	−1.533180
N	6.861141	−11.681557	−3.141294
C	5.891310	−11.735985	−4.142964
N	5.304904	−10.572983	−4.345706
C	5.908475	−9.698174	−3.439257
C	5.678167	−8.316658	−3.173619
O	4.873767	−7.547813	−3.750509
N	6.485396	−7.841672	−2.122232
C	7.428267	−8.589484	−1.442552
N	8.125762	−7.968791	−0.476088
N	7.668963	−9.877370	−1.716604
C	6.884370	−10.368889	−2.689144
H	5.672262	−12.654059	−4.666515
H	6.354856	−6.837315	−1.852723
H	7.939151	−6.993691	−0.197279
H	8.758410	−8.536513	0.071532
P	8.530729	−15.723311	−0.094422
O	6.999403	−16.191833	−0.164838
O	9.492411	−16.646391	0.567726
O	8.358505	−14.289488	0.611870
C	9.584153	−13.531205	0.926984
C	9.285849	−12.493831	1.995243
O	8.483861	−11.413574	1.439445
C	7.247156	−11.275452	2.183765
C	8.524401	−13.027162	3.227001
C	7.077895	−12.590102	2.961769
O	9.092617	−12.375349	4.392156
H	9.934072	−13.043905	0.012291
H	10.347641	−14.224683	1.291816
H	10.250537	−12.079256	2.314000
H	7.318622	−10.410320	2.850280
H	8.616303	−14.113657	3.324210
H	6.578853	−13.340627	2.343813
H	6.503864	−12.454335	3.881501
H	8.607262	−12.695652	5.174572
N	6.169801	−10.978209	1.254741
C	5.609751	−11.834790	0.306298
N	4.684153	−11.249504	−0.426038
C	4.628454	−9.935462	0.043112
C	3.819042	−8.831965	−0.357750
O	2.936717	−8.808777	−1.249569
N	4.111958	−7.667570	0.377936
C	5.031156	−7.586499	1.405155
N	5.128244	−6.403072	2.048637
N	5.783532	−8.623696	1.791553
C	5.550584	−9.747206	1.085294
H	5.935147	−12.860780	0.216213
H	3.580635	−6.804504	0.118268
H	4.635902	−5.563301	1.707532
H	5.936625	−6.275222	2.644299
H	4.942558	−16.088505	−6.295305
H	6.861932	−17.078936	0.220876
H	12.021749	−0.036209	1.282075
H	5.598106	2.515015	−0.451083

d(CGA)$^{H+}$

E: −27517.60 kcal mol^{-1}

O	−0.033535	−0.736874	−1.051582
C	−0.160864	−0.398798	0.350803
C	1.196781	−0.487755	1.038239
O	1.628624	−1.875927	1.195797

C	2.834224	−2.138145	0.427373
C	2.338368	0.225274	0.291694
C	3.045501	−0.900757	−0.458195
O	3.173863	0.862795	1.334027
H	−0.930132	−0.819458	−1.420899
H	−0.853605	−1.085371	0.852980
H	−0.536971	0.627879	0.465042
H	1.087629	−0.060616	2.040607
H	3.668681	−2.307416	1.109654
H	1.986663	1.020420	−0.365903
H	2.556373	−1.018206	−1.426784
H	4.098223	−0.709411	−0.641582
N	2.672720	−3.400161	−0.308693
C	3.382920	−4.563204	0.114027
O	4.178439	−4.479960	1.075009
N	3.165184	−5.728249	−0.552251
C	2.289769	−5.794260	−1.576185
N	2.115333	−6.975296	−2.183430
C	1.543803	−4.643215	−1.994030
C	1.752960	−3.482991	−1.324640
H	2.627497	−7.816098	−1.873775
H	1.473627	−7.047591	−2.961709
H	0.817660	−4.703199	−2.796622
H	1.189610	−2.579560	−1.531003
P	4.620583	1.513011	1.142657
O	4.775624	1.744946	−0.434985
O	4.816302	2.690254	2.032126
O	5.669679	0.313227	1.385201
C	5.793441	−0.217853	2.750159
C	7.019895	−1.109347	2.849867
O	6.841683	−2.344810	2.107144
C	7.778446	−2.428514	1.000313
C	8.326260	−0.477412	2.332716
C	8.478347	−1.062123	0.928338
O	9.373254	−0.880365	3.291890
H	4.894817	−0.796136	2.984584
H	5.889288	0.615694	3.452911
H	7.132707	−1.366519	3.909030
H	8.477050	−3.247787	1.197321
H	8.303536	0.612975	2.333403
H	7.959757	−0.393352	0.239690
H	9.511944	−1.142175	0.597936
N	7.071915	−2.798068	−0.215583
C	6.310785	−1.987945	−1.060980
N	5.708494	−2.672023	−2.013597
C	6.079002	−4.000737	−1.799815
C	5.731739	−5.193950	−2.498681
O	4.967102	−5.314845	−3.484458
N	6.374368	−6.322766	−1.957084
C	7.230309	−6.307205	−0.872844
N	7.763267	−7.485410	−0.499053
N	7.518956	−5.195029	−0.188127
C	6.928774	−4.097203	−0.687341
H	6.250153	−0.919647	−0.918751
H	6.215505	−7.230940	−2.455840
H	7.685970	−8.327247	−1.088790
H	8.469937	−7.457511	0.223844
P	10.950251	−0.955462	3.059958
O	11.220957	0.145401	1.926854
O	11.687717	−0.822576	4.345814
O	11.232760	−2.327333	2.270017
C	11.212260	−3.590519	3.026417
C	11.849834	−4.680317	2.187460
O	10.982584	−4.998789	1.060341
C	11.752791	−4.951996	−0.167379
C	13.236851	−4.325350	1.602612
C	12.933303	−4.019687	0.129892
O	14.076126	−5.497538	1.760411
H	10.171974	−3.843342	3.252837
H	11.771518	−3.460184	3.957373
H	11.943911	−5.569079	2.824744
H	12.086536	−5.961184	−0.431157
H	13.687997	−3.470663	2.116311
H	12.631854	−2.973546	0.032558
H	13.787474	−4.204990	−0.525400
H	14.955633	−5.280857	1.400003
N	10.882296	−4.522834	−1.247227
C	10.389174	−3.247011	−1.493859
N	9.552271	−3.203257	−2.515380
C	9.473610	−4.520043	−2.967066
C	8.738724	−5.132265	−4.009549
N	7.917753	−4.467559	−4.846636
N	8.887532	−6.477466	−4.168703
C	9.707004	−7.165391	−3.352571
N	10.448517	−6.685157	−2.341039
C	10.290547	−5.356664	−2.188869
H	10.688135	−2.400295	−0.893519
H	7.297233	−5.005179	−5.464755
H	7.679845	−3.506980	−4.633302
H	9.753951	−8.236179	−3.540591
O	5.374222	−11.634741	−10.253144
C	6.619617	−12.214991	−10.712129
C	7.463001	−12.650731	−9.519836
O	8.025808	−11.498929	−8.817022
C	7.533946	−11.423098	−7.458386
C	6.699724	−13.463503	−8.458925
C	6.395574	−12.452036	−7.353078
O	7.620659	−14.537069	−8.021530
H	4.923952	−11.248326	−11.024535
H	7.192244	−11.487220	−11.300143
H	6.424048	−13.097820	−11.336881
H	8.299498	−13.243757	−9.904105
H	8.343675	−11.635556	−6.758983
H	5.805202	−13.941945	−8.856667
H	5.427633	−11.990704	−7.559784
H	6.363266	−12.907419	−6.364768
N	7.131350	−10.035162	−7.177310
C	7.858405	−9.297323	−6.228792
O	8.765764	−9.778858	−5.543355
N	7.467330	−7.979843	−6.112177
C	6.497991	−7.319625	−6.857444
O	6.302660	−6.096522	−6.685509
C	5.773101	−8.141936	−7.812957
C	4.683012	−7.517733	−8.640275
C	6.136194	−9.444301	−7.936617
H	7.989616	−7.416676	−5.388580
H	5.074600	−6.690947	−9.245671
H	3.897425	−7.098752	−7.998739
H	4.230301	−8.256362	−9.307817
H	5.677573	−10.098425	−8.672136
P	7.277524	−15.647292	−6.923761
O	5.695146	−15.836198	−7.087982
O	8.153426	−16.839539	−7.082018
O	7.373845	−14.941028	−5.481539
C	8.692743	−14.714026	−4.875434
C	8.499322	−13.996501	−3.552987
O	8.063853	−12.628911	−3.792980
C	6.957374	−12.351462	−2.880654
C	7.428844	−14.628970	−2.627391
C	6.229846	−13.693656	−2.772751
O	7.915600	−14.511057	−1.241265
H	9.299684	−14.100299	−5.547914
H	9.181639	−15.679102	−4.714269
H	9.465957	−13.988749	−3.033970
H	7.356213	−12.054358	−1.909362
H	7.219416	−15.672818	−2.862908
H	5.702953	−13.948144	−3.695282
H	5.541534	−13.741169	−1.929966

N	6.215054	−11.200939	−3.365872
C	6.599373	−9.909951	−2.879733
O	7.545974	−9.827669	−2.070227
N	5.907148	−8.829088	−3.312608
C	4.898576	−8.950635	−4.197170
N	4.267598	−7.836307	−4.585993
C	4.518477	−10.230485	−4.721574
C	5.196423	−11.316649	−4.274220
H	4.528502	−6.914767	−4.195717
H	3.493171	−7.898303	−5.233094
H	3.711060	−10.331871	−5.436684
H	4.957725	−12.317283	−4.607616
P	7.937726	−15.740070	−0.221558
O	6.381035	−16.094638	−0.043034
O	8.816648	−16.884023	−0.594624
O	8.266669	−14.988391	1.148380
C	9.572038	−14.346173	1.374665
C	9.401399	−13.198249	2.358082
O	8.699199	−12.087546	1.724393
C	7.482524	−11.774421	2.455544
C	8.612900	−13.540684	3.639374
C	7.209225	−12.994090	3.349174
O	9.259293	−12.840005	4.733013
H	9.962335	−13.965598	0.427098
H	10.254456	−15.096732	1.783846
H	10.408440	−12.857949	2.629403
H	7.634589	−10.860715	3.038087
H	8.604496	−14.617251	3.837880
H	6.629285	−13.743646	2.805267
H	6.667690	−12.724164	4.258969
H	8.755459	−13.032990	5.544972
N	6.427085	−11.474221	1.501129
C	5.846089	−12.360705	0.593211
N	4.967901	−11.778122	−0.197023
C	4.961648	−10.438632	0.193494
C	4.223141	−9.328905	−0.308611
O	3.388214	−9.326428	−1.245223
N	4.529064	−8.137836	0.377353
C	5.408874	−8.037522	1.436561
N	5.520220	−6.825897	2.022514
N	6.110403	−9.077871	1.905654
C	5.861297	−10.228601	1.250372
H	6.127541	−13.402210	0.567036
H	4.039609	−7.270918	0.050979
H	5.086036	−5.985724	1.610797
H	6.288035	−6.699582	2.669981
H	5.316330	−16.541046	−6.526991
H	6.226541	−17.030409	0.192387
H	12.166206	0.360679	1.802598
H	5.275778	2.554795	−0.655608

d(CGG)$^{H+}$

E: −27425.44 kcal mol^{-1}

O	−0.162073	−0.491650	−0.991717
C	−0.265267	−0.146852	0.410957
C	1.093687	−0.284259	1.087022
O	1.483833	−1.687264	1.218335
C	2.681089	−1.965988	0.442082
C	2.253859	0.403966	0.344914
C	2.904216	−0.730399	−0.441085
O	3.125822	0.973853	1.396450
H	−1.064628	−0.549324	−1.351042
H	−0.976990	−0.808438	0.920339
H	−0.604685	0.892351	0.527684
H	1.003946	0.131261	2.096289
H	3.517048	−2.146031	1.119547
H	1.928439	1.232426	−0.284573
H	2.375110	−0.821175	−1.391442
H	3.955124	−0.565494	−0.661074
N	2.500661	−3.222048	−0.295005
C	3.204939	−4.389713	0.121718
O	3.974560	−4.319954	1.104865
N	3.009933	−5.541354	−0.572147
C	2.157479	−5.591865	−1.616183
N	2.017400	−6.757331	−2.261798
C	1.398836	−4.441145	−2.013959
C	1.591210	−3.291743	−1.320489
H	2.546706	−7.593704	−1.969817
H	1.389611	−6.819110	−3.052471
H	0.680782	−4.492245	−2.824351
H	1.024668	−2.387721	−1.515857
P	4.607279	1.543234	1.236025
O	4.779102	1.782263	−0.339111
O	4.855037	2.699954	2.139517
O	5.593998	0.292220	1.485054
C	5.751024	−0.196189	2.863423
C	6.972752	−1.094915	2.961747
O	6.763738	−2.350555	2.261225
C	7.678246	−2.475387	1.138560
C	8.271817	−0.494625	2.389894
C	8.360899	−1.107610	0.994024
O	9.346095	−0.908779	3.315332
H	4.854556	−0.760239	3.137693
H	5.869658	0.658520	3.536253
H	7.109540	−1.319825	4.025423
H	8.391188	−3.276736	1.355893
H	8.271673	0.595966	2.375533
H	7.798196	−0.461869	0.317580
H	9.377024	−1.185834	0.612423
N	6.956534	−2.905874	−0.044395
C	6.143633	−2.154569	−0.894195
N	5.586833	−2.884972	−1.840513
C	6.045305	−4.185064	−1.616517
C	5.794730	−5.403836	−2.312353
O	5.063206	−5.584466	−3.313643
N	6.499682	−6.485501	−1.746634
C	7.328070	−6.405997	−0.643184
N	7.914520	−7.543776	−0.231757
N	7.542209	−5.266851	0.022915
C	6.893419	−4.215743	−0.500557
H	6.014018	−1.092154	−0.755545
H	6.394307	−7.415895	−2.218144
H	7.850800	−8.426316	−0.762379
H	8.585933	−7.464816	0.520382
P	10.910147	−1.035668	3.025494
O	11.166465	0.040497	1.865140
O	11.697982	−0.901369	4.280983
O	11.130808	−2.428001	2.250435
C	11.152499	−3.675608	3.034833
C	11.782087	−4.779851	2.206930
O	10.889543	−5.149143	1.117560
C	11.616219	−5.122602	−0.136609
C	13.147617	−4.423689	1.573923
C	12.800937	−4.175048	0.101157
O	14.012432	−5.574951	1.751212
H	10.122846	−3.936935	3.297179
H	11.736178	−3.514647	3.945679
H	11.908655	−5.646086	2.869506
H	11.947078	−6.134672	−0.392984
H	13.597095	−3.544020	2.045636
H	12.489492	−3.135099	−0.026833
H	13.638389	−4.377493	−0.570650
H	14.877563	−5.355556	1.359113
N	10.715694	−4.712407	−1.198257
C	10.196407	−3.437347	−1.424277

N	9.388928	−3.391902	−2.464215
C	9.354918	−4.699098	−2.953255
C	8.665128	−5.262940	−4.066548
O	7.917702	−4.674863	−4.884967
N	8.900726	−6.645841	−4.188726
C	9.740270	−7.382724	−3.377496
N	9.906123	−8.686439	−3.691953
N	10.400672	−6.853094	−2.341096
C	10.169912	−5.536158	−2.174630
H	10.454244	−2.603365	−0.788495
H	8.398666	−7.140416	−4.962608
H	9.328561	−9.139216	−4.416756
H	10.320242	−9.274464	−2.979282
O	5.519655	−11.490481	−10.833479
C	6.678667	−12.325466	−11.071163
C	7.250717	−12.813288	−9.745432
O	7.888829	−11.720654	−9.009439
C	7.212050	−11.478541	−7.751313
C	6.212668	−13.419662	−8.782438
C	5.899805	−12.275961	−7.821046
O	6.876433	−14.570585	−8.134317
H	5.266543	−11.083517	−11.680494
H	7.456874	−11.764829	−11.603439
H	6.403138	−13.203954	−11.671488
H	8.022703	−13.556517	−9.970003
H	7.849378	−11.796716	−6.924781
H	5.329087	−13.802468	−9.293476
H	5.101253	−11.676511	−8.262981
H	5.563184	−12.612790	−6.843217
N	7.027805	−10.031965	−7.564624
C	7.728911	−9.355993	−6.523268
O	8.514378	−10.001931	−5.795473
N	7.502076	−8.027536	−6.353321
C	6.659144	−7.353364	−7.161553
N	6.456546	−6.051748	−6.915438
C	6.000790	−8.002315	−8.259079
C	6.219448	−9.330786	−8.425460
H	6.949693	−5.573015	−6.145751
H	5.842201	−5.517503	−7.516074
H	5.361178	−7.450965	−8.938571
H	5.793468	−9.899822	−9.245707
P	6.343204	−15.397306	−6.872869
O	4.792109	−15.023275	−6.732348
O	6.693808	−16.839868	−6.974905
O	6.954950	−14.647856	−5.582822
C	8.406088	−14.753588	−5.359005
C	8.745727	−14.267365	−3.961262
O	8.570220	−12.827095	−3.866650
C	7.650940	−12.498549	−2.790361
C	7.896260	−14.887822	−2.831969
C	6.883217	−13.795475	−2.503516
O	8.837040	−15.161844	−1.729113
H	8.921779	−14.138866	−6.102585
H	8.710112	−15.798771	−5.468911
H	9.802798	−14.502963	−3.791550
H	8.205899	−12.147151	−1.918938
H	7.434215	−15.834421	−3.113427
H	6.033129	−13.909077	−3.180276
H	6.506180	−13.841150	−1.485682
N	6.822403	−11.368265	−3.210191
C	6.982210	−10.099864	−2.576887
O	7.786194	−9.992746	−1.628554
N	6.243538	−9.057150	−3.037512
C	5.367902	−9.209632	−4.049905
N	4.665737	−8.142475	−4.450132
C	5.198559	−10.477954	−4.700519
C	5.954887	−11.511982	−4.258419
H	4.806616	−7.213400	−4.017782
H	4.015331	−8.233197	−5.219100
H	4.509210	−10.597973	−5.527394
H	5.926982	−12.493995	−4.713539
P	8.483309	−15.640060	−0.245423
O	6.944098	−16.078507	−0.332959
O	9.459918	−16.643783	0.259366
O	8.393942	−14.302088	0.642601
C	9.642351	−13.633676	1.048128
C	9.377524	−12.711505	2.226966
O	8.597894	−11.552506	1.817273
C	7.301217	−11.549263	2.472314
C	8.618149	−13.350101	3.404981
C	7.165614	−12.914603	3.169024
O	9.171845	−12.784047	4.620716
H	10.014209	−13.056764	0.196350
H	10.377588	−14.392797	1.331404
H	10.355874	−12.353332	2.570458
H	7.271289	−10.715951	3.179673
H	8.724676	−14.439989	3.416968
H	6.671448	−13.637469	2.516949
H	6.596286	−12.841647	4.098538
H	8.672151	−13.153221	5.371846
N	6.268543	−11.274038	1.484827
C	5.787061	−12.136245	0.500064
N	4.941336	−11.551204	−0.323438
C	4.860166	−10.230670	0.121222
C	4.119804	−9.122054	−0.380735
O	3.331223	−9.103673	−1.356082
N	4.361272	−7.948676	0.361143
C	5.175093	−7.865614	1.472835
N	5.239381	−6.667328	2.095148
N	5.860975	−8.908869	1.954068
C	5.681969	−10.038315	1.242546
H	6.104758	−13.166644	0.458841
H	3.875949	−7.080687	0.030783
H	4.829863	−5.820100	1.671003
H	5.977407	−6.549992	2.778160
H	4.230249	−15.801226	−6.548503
H	6.682268	−16.718646	0.357399
H	12.111651	0.204535	1.678476
H	5.449522	2.458947	−0.557537

d(CTA)$^{H+}$

E: −27606.21 kcal mol^{-1}

O	−0.201258	0.102976	−0.404136
C	−0.254157	0.339028	1.024111
C	1.107934	0.059428	1.648324
O	1.395145	−1.375639	1.677045
C	2.554420	−1.691898	0.869512
C	2.291840	0.713465	0.913550
C	2.872811	−0.418682	0.068879
O	3.218242	1.194266	1.958519
H	−1.109063	0.162047	−0.750188
H	−0.998644	−0.313207	1.497042
H	−0.519561	1.385492	1.230151
H	1.077841	0.410733	2.684912
H	3.382513	−1.995509	1.511621
H	1.994998	1.576707	0.318042
H	2.358047	−0.423297	−0.893659
H	3.940086	−0.310436	−0.114935
N	2.255355	−2.868227	0.035496
C	2.972461	−4.082068	0.235389
O	3.874906	−4.123751	1.100822
N	2.655554	−5.152354	−0.538817
C	1.680117	−5.078775	−1.466722
N	1.434532	−6.166462	−2.210787
C	0.918878	−3.876952	−1.653207

C	1.230037	−2.809574	−0.876186
H	1.920861	−7.056619	−2.024422
H	0.680485	−6.150819	−2.885296
H	0.112153	−3.827026	−2.375330
H	0.686160	−1.870890	−0.917919
P	4.658842	1.842568	1.682875
O	4.682256	2.235939	0.129868
O	4.965387	2.914171	2.669123
O	5.690252	0.606599	1.675931
C	5.947673	−0.062165	2.959479
C	7.030792	−1.104862	2.774412
O	6.517124	−2.208990	1.980509
C	7.483661	−2.550605	0.954886
C	8.316727	−0.606835	2.066675
C	8.234854	−1.243319	0.680768
O	9.434885	−1.114039	2.882177
H	5.025972	−0.540798	3.303873
H	6.272034	0.682915	3.692069
H	7.298082	−1.472925	3.772350
H	8.155790	−3.335331	1.311147
H	8.397768	0.479743	2.036965
H	7.648540	−0.578693	0.041194
H	9.203680	−1.394213	0.209589
N	6.772008	−3.128672	−0.179890
C	6.939329	−4.503085	−0.441769
O	7.705348	−5.222456	0.200558
N	6.177713	−4.977455	−1.488685
C	5.304313	−4.251394	−2.289525
O	4.675993	−4.813623	−3.210108
C	5.194096	−2.833877	−1.981422
C	4.300553	−1.975048	−2.832880
C	5.916825	−2.355476	−0.937239
H	6.309404	−6.004025	−1.718183
H	4.601655	−2.033101	−3.885598
H	4.346282	−0.929236	−2.520211
H	3.259981	−2.314903	−2.781551
H	5.858620	−1.316336	−0.637162
P	10.996508	−1.099200	2.546091
O	11.114341	−0.145432	1.264005
O	11.818469	−0.755260	3.738550
O	11.319665	−2.544944	1.917877
C	11.444932	−3.693532	2.832017
C	12.163719	−4.832763	2.130233
O	11.313856	−5.412174	1.097643
C	11.933460	−5.275070	−0.204740
C	13.489382	−4.449627	1.443092
C	13.089041	−4.275940	−0.027733
O	14.404015	−5.558183	1.637359
H	10.439985	−4.008473	3.128506
H	12.012870	−3.385921	3.714809
H	12.352090	−5.604123	2.886889
H	12.281709	−6.256578	−0.539902
H	13.915733	−3.533260	1.864211
H	12.744471	−3.252691	−0.191870
H	13.914071	−4.479894	−0.713951
H	15.238136	−5.334541	1.185213
N	10.925052	−4.859285	−1.170635
C	10.322982	−3.613562	−1.279824
N	9.413862	−3.557977	−2.238163
C	9.405602	−4.838131	−2.790936
C	8.671790	−5.413453	−3.855347
N	7.762632	−4.747026	−4.587135
N	8.920996	−6.720984	−4.148391
C	9.827690	−7.407920	−3.430853
N	10.579796	−6.960302	−2.411303
C	10.330718	−5.665148	−2.134523
H	10.598215	−2.802891	−0.621575
H	7.230531	−5.236825	−5.317016
H	7.542530	−3.787277	−4.358665

H	9.949633	−8.450866	−3.716869
O	5.572960	−11.724514	−10.498549
C	6.753813	−12.523165	−10.753713
C	7.387466	−12.960302	−9.439011
O	8.002335	−11.827594	−8.743315
C	7.350981	−11.593223	−7.470769
C	6.412693	−13.591936	−8.426842
C	6.067085	−12.432969	−7.496644
O	7.162495	−14.688306	−7.774049
H	5.286680	−11.339379	−11.345195
H	7.493712	−11.947320	−11.323108
H	6.492321	−13.424609	−11.325888
H	8.183257	−13.673704	−9.676560
H	8.019673	−11.882520	−6.658125
H	5.537029	−14.040478	−8.896870
H	5.247815	−11.875570	−7.955706
H	5.744503	−12.737533	−6.504310
N	7.125794	−10.154048	−7.291367
C	7.823747	−9.483238	−6.272688
O	8.657225	−10.030989	−5.545464
N	7.489479	−8.150456	−6.142335
C	6.617119	−7.421122	−6.942156
O	6.427101	−6.204604	−6.714523
C	5.986867	−8.156963	−8.025268
C	5.068045	−7.431542	−8.970641
C	6.275457	−9.478227	−8.147202
H	8.005832	−7.624810	−5.387150
H	5.594967	−6.612363	−9.475666
H	4.223487	−6.985613	−8.430962
H	4.675349	−8.113914	−9.729943
H	5.868680	−10.082449	−8.952556
P	6.804853	−15.426301	−6.400629
O	5.258749	−15.141049	−6.090252
O	7.246213	−16.846845	−6.430831
O	7.470884	−14.529310	−5.240240
C	8.938263	−14.546204	−5.115826
C	9.343407	−14.216411	−3.687157
O	9.170227	−12.805046	−3.388300
C	8.128423	−12.601933	−2.402140
C	8.558420	−14.985381	−2.610217
C	7.501132	−13.983267	−2.138411
O	9.538268	−15.348857	−1.570549
H	9.354389	−13.806600	−5.805156
H	9.307415	−15.542856	−5.374659
H	10.411500	−14.443639	−3.602350
H	8.568950	−12.169166	−1.500837
H	8.119602	−15.909977	−2.986450
H	6.601048	−14.126891	−2.738476
H	7.237997	−14.104384	−1.088377
N	7.178418	−11.607399	−2.888322
C	6.236097	−11.756987	−3.898541
N	5.590728	−10.637399	−4.174581
C	6.132361	−9.691708	−3.306075
C	5.862593	−8.317134	−3.105022
N	4.969600	−7.614602	−3.818623
N	6.567873	−7.684981	−2.124418
C	7.502579	−8.356150	−1.428404
N	7.855705	−9.644556	−1.560021
C	7.129107	−10.270222	−2.505047
H	6.085978	−12.708980	−4.384011
H	4.817371	−6.617665	−3.619717
H	4.403419	−8.084827	−4.511194
H	8.024810	−7.770733	−0.674990
P	9.154439	−16.087599	−0.194155
O	7.784243	−16.882073	−0.445500
O	10.321965	−16.858182	0.312997
O	8.585493	−14.945848	0.776355
C	9.518580	−13.909931	1.245481
C	8.737507	−12.829902	1.960116

O	7.959644	−12.074099	0.991978
C	6.772458	−11.614558	1.685407
C	7.747093	−13.323199	3.056227
C	6.381442	−12.807350	2.568423
O	8.166529	−12.720358	4.306187
H	10.051750	−13.488751	0.387705
H	10.235366	−14.368376	1.932917
H	9.467978	−12.161783	2.438890
H	7.008005	−10.728725	2.287164
H	7.758015	−14.412230	3.150938
H	5.888816	−13.572281	1.960169
H	5.721101	−12.528697	3.392952
H	7.635077	−13.120382	5.018575
N	5.781043	−11.192949	0.719392
C	5.082117	−11.982454	−0.196194
N	4.192989	−11.294720	−0.884247
C	4.294152	−9.986912	−0.406137
C	3.562427	−8.808801	−0.740782
O	2.645177	−8.690177	−1.588845
N	3.977892	−7.696256	0.014262
C	4.961066	−7.716323	0.982491
N	5.191399	−6.565064	1.646507
N	5.652371	−8.819781	1.295938
C	5.279631	−9.903256	0.588763
H	5.277524	−13.039847	−0.298686
H	3.505058	−6.785245	−0.192509
H	4.733991	−5.681445	1.372665
H	6.038446	−6.516669	2.198306
H	12.002890	0.245380	1.150339
H	4.900073	3.176623	−0.018211
H	4.712664	−15.950670	−6.116297
H	7.921544	−17.834808	−0.612275

d(GAA)$^{H+}$

E: −27608.60 kcal mol^{-1}

O	0.115780	0.171711	−0.986297
C	0.074777	0.640253	0.382132
C	1.394701	0.344219	1.085561
O	1.581706	−1.094080	1.282770
C	2.695656	−1.573298	0.487284
C	2.656297	0.819883	0.342117
C	3.077550	−0.417059	−0.444675
O	3.610486	1.251705	1.396205
H	−0.784934	0.238238	−1.348540
H	−0.729474	0.140614	0.936716
H	−0.096905	1.725953	0.416047
H	1.351826	0.809974	2.075432
H	3.516614	−1.848342	1.152879
H	2.483892	1.694017	−0.286253
H	2.469982	−0.444170	−1.351570
H	4.124927	−0.433813	−0.737521
N	2.337981	−2.804739	−0.192791
C	1.441328	−3.001627	−1.243540
N	1.379694	−4.265490	−1.622031
C	2.276070	−4.941133	−0.788476
C	2.654585	−6.314234	−0.726733
O	2.227783	−7.260088	−1.433196
N	3.630424	−6.550513	0.263841
C	4.144201	−5.591652	1.115644
N	5.036849	−5.996260	2.052136
N	3.777779	−4.308605	1.069262
C	2.872378	−4.047412	0.110499
H	0.863634	−2.181104	−1.647610
H	4.005056	−7.526249	0.311836
H	5.476811	−6.923089	1.968636
H	5.586025	−5.249647	2.469198
P	5.201375	1.115649	1.443943
O	5.779117	1.275821	−0.046055
O	5.749321	2.011919	2.497315
O	5.557027	−0.442036	1.617994
C	5.579122	−1.049520	2.958737
C	6.864418	−1.840812	3.169062
O	6.868170	−3.103985	2.448952
C	7.765962	−3.050247	1.311413
C	8.140823	−1.106310	2.726809
C	8.404263	−1.649643	1.316637
O	9.175328	−1.463945	3.711377
H	4.717202	−1.716517	3.030566
H	5.509396	−0.263491	3.714826
H	6.916121	−2.080362	4.236028
H	8.506416	−3.845241	1.426392
H	8.034879	−0.020693	2.744468
H	7.920730	−0.987148	0.598185
H	9.464053	−1.697437	1.070265
N	7.040840	−3.373321	0.085643
C	6.211437	−2.568797	−0.690763
N	5.599748	−3.235704	−1.654082
C	6.030103	−4.553653	−1.507251
C	5.720205	−5.750295	−2.198407
N	4.852216	−5.834486	−3.218278
N	6.352653	−6.883280	−1.783039
C	7.205026	−6.843764	−0.743582
N	7.542208	−5.776295	−0.005847
C	6.926798	−4.659006	−0.432375
H	6.104733	−1.514136	−0.493934
H	4.656522	−6.744958	−3.649949
H	4.359322	−5.009061	−3.530092
H	7.667551	−7.793641	−0.488042
P	10.738734	−1.121409	3.568388
O	10.862948	0.120092	2.561696
O	11.358539	−0.984180	4.914229
O	11.349204	−2.268329	2.626051
C	11.388303	−3.640016	3.159644
C	11.869339	−4.584422	2.080077
O	10.839334	−4.718366	1.059850
C	11.511640	−4.866071	−0.216673
C	13.180468	−4.169895	1.353598
C	12.734440	−3.947751	−0.101113
O	14.106788	−5.276099	1.492794
H	10.385313	−3.924273	3.491764
H	12.077203	−3.667596	4.008898
H	12.035652	−5.561095	2.555667
H	11.802188	−5.911654	−0.370341
H	13.615738	−3.265345	1.787099
H	12.433999	−2.904779	−0.239003
H	13.518382	−4.190214	−0.822170
H	14.962248	−4.992537	1.121299
N	10.580833	−4.542548	−1.278413
C	10.058069	−3.294089	−1.597666
N	9.216499	−3.331378	−2.615246
C	9.176805	−4.671942	−2.994602
C	8.462837	−5.351678	−4.009622
N	7.626234	−4.740548	−4.867340
N	8.651587	−6.697787	−4.112715
C	9.483551	−7.321293	−3.257905
N	10.203489	−6.774739	−2.263040
C	10.013528	−5.443782	−2.175179
H	10.336902	−2.405256	−1.050455
H	7.036237	−5.307108	−5.490083
H	7.399751	−3.765132	−4.721009
H	9.563964	−8.397836	−3.390058
O	5.887232	−11.769063	−10.604904
C	7.170840	−12.385610	−10.869109
C	7.791397	−12.875413	−9.566213
O	8.264249	−11.757292	−8.749179

C	7.547529	−11.683854	−7.495190
C	6.837173	−13.683935	−8.668181
C	6.380451	−12.679992	−7.609950
O	7.630258	−14.798960	−8.111524
H	5.586835	−11.341679	−11.425933
H	7.856065	−11.668201	−11.336986
H	7.051079	−13.248172	−11.539636
H	8.662604	−13.487207	−9.821953
H	8.219414	−11.929172	−6.670720
H	6.005594	−14.120558	−9.221334
H	5.476525	−12.188221	−7.973057
H	6.154522	−13.147403	−6.653612
N	7.132182	−10.290605	−7.256380
C	7.741927	−9.570092	−6.215194
O	8.588595	−10.054174	−5.457972
N	7.301361	−8.268282	−6.088310
C	6.379138	−7.610459	−6.895969
O	6.097324	−6.412464	−6.677429
C	5.814229	−8.399490	−7.977860
C	4.819758	−7.760853	−8.908043
C	6.230630	−9.684178	−8.114537
H	7.771727	−7.704085	−5.332276
H	5.252554	−6.881107	−9.399757
H	3.935898	−7.417723	−8.356054
H	4.497144	−8.468428	−9.677192
H	5.892231	−10.310530	−8.934237
P	7.100010	−15.856966	−7.028367
O	5.502214	−15.742026	−7.041172
O	7.696262	−17.201982	−7.252249
O	7.421172	−15.197855	−5.595542
C	8.833151	−15.086891	−5.204651
C	8.915351	−14.417538	−3.849652
O	8.497066	−13.033023	−3.977356
C	7.682831	−12.680880	−2.834406
C	8.038830	−15.056092	−2.737078
C	6.984231	−13.988479	−2.439303
O	8.950327	−15.333004	−1.614910
H	9.366527	−14.487692	−5.948782
H	9.271561	−16.087965	−5.154984
H	9.963621	−14.454177	−3.527855
H	8.303760	−12.290956	−2.023343
H	7.598365	−16.006640	−3.037520
H	6.120101	−14.180212	−3.079944
H	6.642639	−13.992836	−1.406099
N	6.796771	−11.585102	−3.215674
C	7.022698	−10.310579	−2.655556
O	7.910286	−10.085557	−1.832510
N	6.144663	−9.339292	−3.084917
C	5.116077	−9.481482	−4.007423
O	4.404403	−8.503752	−4.312623
C	4.949402	−10.815690	−4.567435
C	3.851308	−11.051357	−5.566552
C	5.798087	−11.787678	−4.147454
H	6.243733	−8.390180	−2.615659
H	2.875422	−10.796285	−5.136539
H	3.826040	−12.096500	−5.884666
H	3.984689	−10.414957	−6.449185
H	5.734773	−12.797736	−4.532825
P	8.542640	−15.743413	−0.119988
O	7.007067	−16.192031	−0.197481
O	9.511837	−16.712508	0.459276
O	8.416230	−14.351215	0.676488
C	9.658841	−13.600633	0.934362
C	9.372966	−12.466847	1.899427
O	8.553890	−11.459970	1.240710
C	7.404145	−11.147606	2.064651
C	8.636444	−12.871712	3.197715
C	7.202761	−12.373662	2.968168
O	9.294033	−12.180207	4.290245
H	10.029197	−13.204198	−0.015803
H	10.401368	−14.277928	1.366687
H	10.339629	−12.018122	2.162629
H	7.589335	−10.239751	2.640246
H	8.674201	−13.952802	3.364798
H	6.625819	−13.146846	2.453101
H	6.692380	−12.121128	3.900686
H	8.843501	−12.438189	5.115305
N	6.278415	−10.837519	1.176057
C	5.719404	−9.527664	1.156389
O	6.172734	−8.654100	1.929635
N	4.699694	−9.278177	0.294328
C	4.232513	−10.234868	−0.532209
N	3.230251	−9.913785	−1.362654
C	4.795455	−11.554572	−0.539096
C	5.815094	−11.799843	0.320675
H	2.865803	−8.947975	−1.397189
H	2.860915	−10.608388	−1.998058
H	4.428694	−12.320423	−1.211811
H	6.325806	−12.753626	0.370127
H	5.063244	−16.604082	−7.177244
H	6.826502	−17.012357	0.301532
H	11.083448	0.955542	3.017649
H	5.710687	2.185620	−0.396308

d(GAC)$^{H+}$

E: −27519.67 kcal mol^{-1}

O	0.027178	−0.053964	−0.724590
C	0.026614	0.374281	0.657519
C	1.404285	0.168477	1.278183
O	1.711876	−1.252098	1.449257
C	2.778955	−1.656780	0.554469
C	2.578423	0.744275	0.466103
C	3.052132	−0.451500	−0.356023
O	3.552736	1.261210	1.459889
H	−0.894905	−0.052074	−1.035738
H	−0.703596	−0.201457	1.239923
H	−0.227876	1.441496	0.732038
H	1.386422	0.620307	2.275090
H	3.651675	−1.930800	1.152597
H	2.296293	1.599149	−0.148914
H	2.421048	−0.496710	−1.245364
H	4.087594	−0.392518	−0.683663
N	2.414706	−2.870664	−0.158625
C	1.517765	−3.030166	−1.216763
N	1.453963	−4.279074	−1.639739
C	2.346406	−4.985520	−0.828984
C	2.719931	−6.361185	−0.819053
O	2.296954	−7.276870	−1.566595
N	3.684631	−6.639853	0.169709
C	4.191389	−5.717766	1.064124
N	5.062283	−6.169754	1.998140
N	3.837537	−4.429909	1.060375
C	2.941968	−4.126624	0.104190
H	0.937105	−2.197535	−1.588966
H	4.041822	−7.623669	0.196935
H	5.496012	−7.097906	1.890082
H	5.594596	−5.451307	2.477614
P	5.149526	1.252134	1.420715
O	5.638044	1.405601	−0.100553
O	5.679551	2.221879	2.416644
O	5.625718	−0.270847	1.623214
C	5.668181	−0.835163	2.982862
C	6.932953	−1.662655	3.170267
O	6.863310	−2.940092	2.479994
C	7.772232	−2.975863	1.353067

C	8.213814	−0.973864	2.668817
C	8.452700	−1.595117	1.288608
O	9.263223	−1.288226	3.653082
H	4.784191	−1.463921	3.113276
H	5.657412	−0.021076	3.712415
H	7.015330	−1.875726	4.241142
H	8.489782	−3.784436	1.508950
H	8.119984	0.111949	2.626298
H	7.986041	−0.957409	0.536881
H	9.509900	−1.688377	1.045634
N	7.046349	−3.330121	0.136716
C	6.203437	−2.527275	−0.626668
N	5.620718	−3.182538	−1.614581
C	6.087928	−4.490526	−1.499913
C	5.815604	−5.669394	−2.234692
N	4.971300	−5.725931	−3.275184
N	6.453369	−6.807281	−1.839711
C	7.292846	−6.779221	−0.788905
N	7.609097	−5.724546	−0.021290
C	6.974823	−4.605070	−0.417626
H	6.061990	−1.483507	−0.394423
H	4.766007	−6.628450	−3.719630
H	4.463509	−4.897519	−3.553948
H	7.756725	−7.731440	−0.543164
P	10.811591	−0.887642	3.481128
O	10.876036	0.355248	2.470381
O	11.447109	−0.729285	4.817214
O	11.449826	−2.004475	2.522422
C	11.509704	−3.386838	3.023255
C	11.906183	−4.309987	1.893422
O	10.831600	−4.372181	0.921835
C	11.449340	−4.695565	−0.350367
C	13.201477	−3.933145	1.114509
C	12.743088	−3.869057	−0.356890
O	14.164704	−4.984593	1.370126
H	10.528313	−3.670040	3.415578
H	12.252632	−3.439343	3.824459
H	12.060069	−5.307602	2.331068
H	11.654114	−5.766630	−0.408578
H	13.607708	−2.974355	1.447856
H	12.533567	−2.832536	−0.637634
H	13.494341	−4.271845	−1.040406
H	15.022096	−4.694029	1.009000
N	10.493959	−4.408373	−1.415983
C	10.017907	−5.468235	−2.242138
O	10.439356	−6.630516	−2.048343
N	9.127379	−5.169183	−3.220609
C	8.680157	−3.909254	−3.400005
N	7.802233	−3.690773	−4.387549
C	9.129116	−2.833193	−2.563735
C	10.032751	−3.130586	−1.597956
H	7.467199	−4.467971	−4.977786
H	7.416491	−2.765355	−4.521673
H	8.763678	−1.822408	−2.697630
H	10.425442	−2.384356	−0.919257
O	6.077117	−11.563161	−10.783638
C	7.315919	−12.290760	−10.958126
C	7.775876	−12.874983	−9.627587
O	8.285345	−11.845086	−8.720696
C	7.399145	−11.657609	−7.590891
C	6.679173	−13.611842	−8.841789
C	6.183540	−12.568076	−7.841915
O	7.335851	−14.776534	−8.214756
H	5.874697	−11.114797	−11.623359
H	8.103634	−11.630440	−11.341700
H	7.177152	−13.121449	−11.664943
H	8.605799	−13.557361	−9.836030
H	7.935559	−11.919487	−6.673384
H	5.882454	−13.995880	−9.479267
H	5.374320	−12.010054	−8.314604
H	5.801086	−13.002194	−6.920585
N	7.055664	−10.245510	−7.452688
C	6.231755	−9.482552	−8.285940
N	6.176036	−8.216624	−7.919664
C	7.005023	−8.123017	−6.800231
C	7.349512	−7.004101	−5.985498
O	6.951662	−5.819118	−6.100318
N	8.245880	−7.354920	−4.959387
C	8.774981	−8.615811	−4.766444
N	9.680094	−8.755224	−3.776455
N	8.442527	−9.670950	−5.520208
C	7.572623	−9.370792	−6.503357
H	5.749902	−9.923198	−9.147706
H	8.547226	−6.582720	−4.320889
H	9.876619	−7.988187	−3.116868
H	9.920522	−9.700744	−3.507183
P	6.715186	−15.718353	−7.080519
O	5.134740	−15.460418	−7.121842
O	7.186136	−17.123001	−7.221241
O	7.104847	−15.013911	−5.685097
C	8.528184	−15.014893	−5.315298
C	8.677969	−14.429383	−3.927754
O	8.352139	−13.012934	−3.960747
C	7.483670	−12.692866	−2.847472
C	7.776431	−15.074972	−2.845181
C	6.736540	−13.997216	−2.541114
O	8.664255	−15.374701	−1.710457
H	9.084727	−14.412597	−6.039545
H	8.902047	−16.043015	−5.326587
H	9.726670	−14.551552	−3.631512
H	8.068198	−12.349768	−1.990107
H	7.329736	−16.014682	−3.169679
H	5.893273	−14.138148	−3.221614
H	6.357756	−14.040445	−1.522180
N	6.644870	−11.560420	−3.231916
C	6.861988	−10.320790	−2.597215
O	7.673889	−10.165610	−1.684946
N	6.075366	−9.294426	−3.074537
C	5.112111	−9.368632	−4.073020
O	4.474318	−8.349654	−4.406941
C	4.920154	−10.681070	−4.673347
C	3.867803	−10.843091	−5.734030
C	5.707246	−11.696498	−4.237786
H	6.215569	−8.352354	−2.604260
H	2.885942	−10.542132	−5.349808
H	3.802055	−11.880896	−6.069097
H	4.080151	−10.203407	−6.598319
H	5.641591	−12.689814	−4.665332
P	8.236981	−15.886252	−0.254921
O	6.694840	−16.292527	−0.402213
O	9.173896	−16.920349	0.263010
O	8.142283	−14.559060	0.648895
C	9.404730	−13.885979	1.002485
C	9.119248	−12.764548	1.981511
O	8.366162	−11.715606	1.310749
C	7.185667	−11.385756	2.084334
C	8.314377	−13.167867	3.238748
C	6.909312	−12.616361	2.961204
O	8.949627	−12.521280	4.371629
H	9.851358	−13.482307	0.088698
H	10.083127	−14.615125	1.455507
H	10.089545	−12.358665	2.295849
H	7.370371	−10.492055	2.681568
H	8.307639	−14.252633	3.384885
H	6.326292	−13.364840	2.418102
H	6.373343	−12.356847	3.877396
H	8.456583	−12.778910	5.172221
N	6.110220	−11.029093	1.152922

C	5.657901	−9.680915	1.068569
O	6.172570	−8.813005	1.808728
N	4.664962	−9.390406	0.188245
C	4.141574	−10.338789	−0.613132
N	3.168737	−9.978073	−1.462674
C	4.619423	−11.690630	−0.578036
C	5.602917	−11.979864	0.309987
H	2.855039	−8.995246	−1.514836
H	2.754708	−10.667408	−2.075988
H	4.217167	−12.447724	−1.240251
H	6.047493	−12.964061	0.391665
H	4.613622	−16.284080	−7.053899
H	6.432487	−17.026878	0.186817
H	11.001648	1.210239	2.926320
H	5.547071	2.313549	−0.450165

d(GAG)$^{H+}$

E: −27518.57 kcal mol^{-1}

O	0.025169	−0.009917	−0.779028
C	0.014634	0.420827	0.602246
C	1.382962	0.199651	1.237894
O	1.673667	−1.225216	1.403152
C	2.755598	−1.630355	0.526178
C	2.572368	0.768256	0.442775
C	3.040497	−0.427289	−0.382319
O	3.542825	1.265026	1.451214
H	−0.893493	0.001989	−1.099874
H	−0.727994	−0.146028	1.177647
H	−0.228675	1.490939	0.672875
H	1.357940	0.646488	2.236958
H	3.620261	−1.899525	1.137222
H	2.307294	1.631739	−0.167850
H	2.410648	−0.466295	−1.272950
H	4.077684	−0.374809	−0.706013
N	2.406954	−2.848631	−0.185295
C	1.521218	−3.024534	−1.249782
N	1.475282	−4.277080	−1.665314
C	2.369138	−4.968367	−0.842531
C	2.758858	−6.339394	−0.818341
O	2.352281	−7.265948	−1.561465
N	3.719561	−6.598808	0.180483
C	4.208959	−5.663323	1.070994
N	5.079496	−6.094294	2.016102
N	3.838442	−4.380580	1.054276
C	2.946864	−4.096290	0.089236
H	0.935639	−2.200469	−1.633599
H	4.091202	−7.576776	0.215476
H	5.526722	−7.016509	1.916399
H	5.608129	−5.361089	2.478410
P	5.139591	1.219128	1.439522
O	5.657199	1.384697	−0.071139
O	5.672570	2.162656	2.458788
O	5.582131	−0.315641	1.625769
C	5.610445	−0.902114	2.976203
C	6.879326	−1.721718	3.172088
O	6.838471	−2.987025	2.458265
C	7.754398	−2.980664	1.334458
C	8.163983	−1.014615	2.708219
C	8.414164	−1.589954	1.309528
O	9.199899	−1.362955	3.695678
H	4.731311	−1.542320	3.080766
H	5.579033	−0.100997	3.719421
H	6.942472	−1.953933	4.240347
H	8.482685	−3.782120	1.477182
H	8.071323	0.072278	2.702575
H	7.935498	−0.936029	0.579776
H	9.472015	−1.659396	1.060055
N	7.043252	−3.321571	0.106405
C	6.207524	−2.523688	−0.668931
N	5.630086	−3.186888	−1.655525
C	6.093796	−4.494792	−1.527259
C	5.831575	−5.681127	−2.254474
N	4.991584	−5.758174	−3.297637
N	6.478169	−6.810618	−1.848056
C	7.300651	−6.774203	−0.783701
N	7.598386	−5.714849	−0.017836
C	6.970668	−4.600669	−0.436306
H	6.068638	−1.476704	−0.449899
H	4.809537	−6.665777	−3.742695
H	4.491727	−4.933863	−3.601270
H	7.767280	−7.722477	−0.528380
P	10.761809	−1.015785	3.537146
O	10.877605	0.231997	2.536937
O	11.393373	−0.889162	4.878564
O	11.365917	−2.148805	2.575504
C	11.397042	−3.531149	3.080693
C	11.842092	−4.458384	1.971485
O	10.786944	−4.564119	0.975423
C	11.434170	−4.735027	−0.312115
C	13.139544	−4.041709	1.220090
C	12.665334	−3.823686	−0.226550
O	14.071201	−5.145444	1.345590
H	10.397471	−3.808965	3.428529
H	12.102891	−3.584930	3.914607
H	12.012674	−5.445585	2.424047
H	11.718374	−5.784323	−0.452701
H	13.580644	−3.135490	1.644058
H	12.366562	−2.780149	−0.365551
H	13.433324	−4.075037	−0.961816
H	14.921053	−4.858156	0.964336
N	10.493113	−4.425528	−1.366737
C	9.950875	−3.180563	−1.692706
N	9.147141	−3.233967	−2.735475
C	9.152077	−4.572847	−3.130780
C	8.484780	−5.232046	−4.205636
O	7.707496	−4.727554	−5.051058
N	8.785413	−6.606158	−4.250473
C	9.630238	−7.262205	−3.378828
N	9.846965	−8.574497	−3.607532
N	10.248577	−6.648160	−2.361469
C	9.980270	−5.330246	−2.289435
H	10.190647	−2.291822	−1.127303
H	8.293787	−7.169559	−4.983034
H	9.300999	−9.092489	−4.313401
H	10.272652	−9.102692	−2.855981
O	5.925876	−11.789912	−10.810825
C	7.193144	−12.453776	−11.035779
C	7.781807	−12.913881	−9.707664
O	8.267043	−11.779509	−8.921165
C	7.536648	−11.654369	−7.676916
C	6.795593	−13.670056	−8.798850
C	6.343143	−12.618369	−7.787350
O	7.558258	−14.778674	−8.189015
H	5.647977	−11.383851	−11.650540
H	7.905714	−11.774533	−11.519600
H	7.056016	−13.335945	−11.676985
H	8.641735	−13.554689	−9.928809
H	8.190183	−11.899220	−6.838507
H	5.965114	−14.111229	−9.350197
H	5.465871	−12.111749	−8.193606
H	6.074896	−13.043337	−6.822254
N	7.159378	−10.245006	−7.477610
C	7.739294	−9.499165	−6.410562
O	8.531233	−10.068245	−5.627000
N	7.396262	−8.191529	−6.276656

C	6.535569	−7.603955	−7.132310
N	6.227952	−6.314868	−6.930949
C	5.970177	−8.328563	−8.234371
C	6.322000	−9.630940	−8.376623
H	6.706357	−5.765174	−6.201601
H	5.619582	−5.838351	−7.584181
H	5.304624	−7.848875	−8.942585
H	5.984281	−10.244635	−9.205853
P	7.000972	−15.777364	−7.065598
O	5.407260	−15.615347	−7.081648
O	7.555993	−17.147668	−7.236977
O	7.348639	−15.074934	−5.659388
C	8.764850	−15.021206	−5.268974
C	8.873671	−14.408457	−3.888826
O	8.519979	−13.001463	−3.957120
C	7.651745	−12.669748	−2.847932
C	7.965685	−15.051270	−2.807855
C	6.916877	−13.976653	−2.521102
O	8.847571	−15.343621	−1.665755
H	9.312600	−14.410451	−5.992898
H	9.172618	−16.036337	−5.259181
H	9.918796	−14.504429	−3.570006
H	8.234642	−12.308287	−1.996852
H	7.525835	−15.995361	−3.128997
H	6.076117	−14.135735	−3.200828
H	6.535387	−14.007336	−1.502596
N	6.801916	−11.550767	−3.247068
C	7.008835	−10.298376	−2.632569
O	7.822887	−10.121974	−1.725848
N	6.205082	−9.289352	−3.117306
C	5.225689	−9.391489	−4.096917
O	4.553948	−8.392406	−4.423597
C	5.051810	−10.712979	−4.683192
C	3.986504	−10.906107	−5.726173
C	5.858103	−11.711267	−4.242381
H	6.317180	−8.345399	−2.644264
H	3.003740	−10.627608	−5.327388
H	3.941164	−11.946830	−6.055477
H	4.167327	−10.265262	−6.596728
H	5.799061	−12.711445	−4.654006
P	8.418595	−15.803408	−0.194416
O	6.880280	−16.226812	−0.333499
O	9.362005	−16.810479	0.363061
O	8.310278	−14.445355	0.662247
C	9.565448	−13.754294	1.006689
C	9.272389	−12.634859	1.986287
O	8.507098	−11.591709	1.319838
C	7.321831	−11.279283	2.093121
C	8.475759	−13.046728	3.245679
C	7.063102	−12.514170	2.969702
O	9.104098	−12.389485	4.376266
H	10.000748	−13.347444	0.089020
H	10.256588	−14.472821	1.457288
H	10.239791	−12.219547	2.296908
H	7.491939	−10.382396	2.689818
H	8.483832	−14.131306	3.393730
H	6.489352	−13.269071	2.425469
H	6.524461	−12.262703	3.886540
H	8.614122	−12.649943	5.177808
N	6.240957	−10.940848	1.160597
C	5.744075	−9.607480	1.095757
O	6.222210	−8.735831	1.856070
N	4.751573	−9.334413	0.209478
C	4.266245	−10.286215	−0.611818
N	3.293013	−9.942215	−1.467736
C	4.782399	−11.624269	−0.588888
C	5.768293	−11.895534	0.301943
H	2.956211	−8.966872	−1.515841
H	2.911053	−10.633347	−2.099521
H	4.407370	−12.384240	−1.263661
H	6.242979	−12.866339	0.374150
H	4.940709	−16.469767	−7.162718
H	6.612259	−16.918622	0.302830
H	11.024526	1.078635	3.002177
H	5.569408	2.294377	−0.417018

d(GCA)$^{H+}$

E: −27516.94 kcal mol^{-1}

O	−0.268613	−0.328874	−0.583383
C	−0.256901	0.102081	0.797937
C	1.153755	0.014884	1.368780
O	1.576295	−1.371199	1.576105
C	2.632497	−1.737478	0.654644
C	2.244592	0.649037	0.490162
C	2.811790	−0.534503	−0.289537
O	3.206787	1.270649	1.424772
H	−1.197097	−0.381025	−0.870168
H	−0.918020	−0.527778	1.406016
H	−0.593247	1.145775	0.878358
H	1.141081	0.498295	2.350718
H	3.538439	−1.960478	1.227199
H	1.868633	1.443036	−0.155511
H	2.206076	−0.658907	−1.188609
H	3.844797	−0.394327	−0.599269
N	2.291029	−2.976330	−0.035033
C	1.332391	−3.156128	−1.036542
N	1.262513	−4.406599	−1.450263
C	2.208914	−5.095841	−0.690300
C	2.600788	−6.465758	−0.711591
O	2.152355	−7.379951	−1.447092
N	3.613498	−6.740846	0.224232
C	4.166567	−5.815079	1.087454
N	5.088010	−6.268433	1.960340
N	3.814824	−4.523286	1.096202
C	2.852914	−4.226200	0.201959
H	0.712121	−2.335262	−1.368552
H	3.957458	−7.728292	0.260016
H	5.406802	−7.246968	1.953883
H	5.613973	−5.578726	2.480978
P	4.692664	1.764491	1.120509
O	4.747329	1.938192	−0.470796
O	5.062359	2.938847	1.956311
O	5.637977	0.474820	1.338661
C	5.854812	0.015233	2.719405
C	6.974073	−1.008829	2.746494
O	6.558238	−2.230311	2.073622
C	7.504818	−2.570192	1.026231
C	8.288989	−0.561897	2.070899
C	8.254449	−1.268859	0.719155
O	9.369194	−1.032646	2.960002
H	4.929029	−0.436023	3.088400
H	6.122917	0.871255	3.345650
H	7.170277	−1.237740	3.800336
H	8.176336	−3.358603	1.370545
H	8.384606	0.521149	1.989554
H	7.688730	−0.640067	0.027623
H	9.237288	−1.437650	0.286052
N	6.770447	−3.144562	−0.099539
C	6.920532	−4.529999	−0.398573
O	7.715844	−5.212369	0.277636
N	6.186056	−5.048376	−1.416780
C	5.332931	−4.284048	−2.127028
N	4.639942	−4.863340	−3.114708
C	5.163048	−2.889628	−1.831897
C	5.889864	−2.375245	−0.809579

H	4.764124	−5.866438	−3.332150
H	3.974891	−4.319170	−3.648168
H	4.467623	−2.276790	−2.392202
H	5.803966	−1.343762	−0.491840
P	10.930037	−1.146851	2.644915
O	11.134363	−0.202579	1.365788
O	11.753686	−0.865927	3.852074
O	11.162035	−2.611319	2.017040
C	11.240418	−3.768393	2.927810
C	12.028425	−4.893534	2.277454
O	11.262808	−5.507912	1.201286
C	11.928653	−5.321583	−0.071322
C	13.383824	−4.485121	1.669537
C	13.068067	−4.317104	0.177133
O	14.305487	−5.576856	1.918322
H	10.220857	−4.099798	3.143899
H	11.735300	−3.460023	3.852997
H	12.187132	−5.654016	3.051712
H	12.295745	−6.290087	−0.422674
H	13.768528	−3.561300	2.114148
H	12.733052	−3.294996	−0.010466
H	13.933603	−4.518744	−0.457979
H	15.159732	−5.339187	1.513239
N	10.952813	−4.882879	−1.062076
C	10.359864	−3.632159	−1.164489
N	9.451292	−3.563784	−2.122062
C	9.429402	−4.841692	−2.678101
C	8.676923	−5.406904	−3.734369
N	7.778246	−4.722369	−4.462588
N	8.900520	−6.719779	−4.025526
C	9.816743	−7.414004	−3.324248
N	10.589015	−6.974134	−2.317411
C	10.350616	−5.679194	−2.029947
H	10.639717	−2.827700	−0.501410
H	7.216811	−5.206443	−5.175264
H	7.553750	−3.770626	−4.205646
H	9.927910	−8.456935	−3.615209
O	5.647588	−11.449926	−10.651253
C	6.841296	−12.221289	−10.929468
C	7.464077	−12.714584	−9.629140
O	8.051727	−11.609401	−8.869538
C	7.378431	−11.449662	−7.595059
C	6.485222	−13.407502	−8.663303
C	6.104292	−12.300383	−7.687253
O	7.250723	−14.510453	−8.038067
H	5.364050	−11.031817	−11.482972
H	7.581658	−11.609388	−11.459545
H	6.598525	−13.096286	−11.549002
H	8.272737	−13.404854	−9.890734
H	8.037594	−11.778822	−6.789663
H	5.628450	−13.856605	−9.166119
H	5.285987	−11.731686	−8.133438
H	5.768386	−12.662249	−6.719104
N	7.136772	−10.024994	−7.343170
C	7.829094	−9.397150	−6.293966
O	8.675450	−9.969288	−5.600582
N	7.478626	−8.077564	−6.094916
C	6.584107	−7.324004	−6.846147
O	6.376455	−6.123865	−6.557565
C	5.958448	−8.012733	−7.962685
C	5.015517	−7.256268	−8.858343
C	6.270442	−9.319193	−8.158082
H	7.986973	−7.586927	−5.313363
H	5.516085	−6.392667	−9.313815
H	4.161971	−6.868688	−8.288416
H	4.637385	−7.901477	−9.656565
H	5.871369	−9.885489	−8.994128
P	6.876434	−15.356583	−6.740216
O	5.305455	−15.105115	−6.545872
O	7.344766	−16.765219	−6.844543
O	7.492666	−14.556148	−5.482249
C	8.935189	−14.678242	−5.215343
C	9.226336	−14.372422	−3.754638
O	9.078542	−12.954558	−3.463675
C	7.972367	−12.722113	−2.554740
C	8.325813	−15.111211	−2.748157
C	7.257891	−14.076253	−2.398214
O	9.200405	−15.482751	−1.619927
H	9.466116	−13.969623	−5.857101
H	9.255411	−15.698601	−5.444310
H	10.273928	−14.641232	−3.581064
H	8.368477	−12.346705	−1.605948
H	7.905499	−16.035052	−3.147349
H	6.448977	−14.174819	−3.123879
H	6.832691	−14.211556	−1.406141
N	7.117572	−11.665356	−3.069672
C	6.188738	−11.745772	−4.108344
N	5.566063	−10.604262	−4.322655
C	6.101968	−9.716065	−3.388131
C	5.798429	−8.349268	−3.115244
O	4.983124	−7.611217	−3.715235
N	6.537477	−7.853654	−2.025181
C	7.472469	−8.571988	−1.305109
N	8.069830	−7.943810	−0.278258
N	7.781302	−9.846260	−1.581424
C	7.070585	−10.356869	−2.602263
H	6.039224	−12.664671	−4.653266
H	6.388200	−6.845610	−1.781842
H	7.930662	−6.938730	−0.089689
H	8.797237	−8.447500	0.211202
P	8.749726	−15.929126	−0.153390
O	7.281009	−16.526907	−0.381370
O	9.771986	−16.806921	0.477948
O	8.441153	−14.577850	0.665863
C	9.585050	−13.794603	1.166985
C	9.090612	−12.764859	2.163090
O	8.306583	−11.755539	1.466942
C	7.068210	−11.530174	2.184009
C	8.206543	−13.319966	3.307350
C	6.795776	−12.840175	2.937741
O	8.688250	−12.727064	4.540562
H	10.064712	−13.300257	0.316597
H	10.296710	−14.470103	1.650156
H	9.977557	−12.287992	2.601015
H	7.172871	−10.682595	2.862888
H	8.265404	−14.410872	3.372202
H	6.320950	−13.575326	2.281877
H	6.160039	−12.691565	3.813912
H	8.154837	−13.092228	5.270224
N	6.043648	−11.142349	1.209957
C	5.489299	−9.831994	1.236425
O	5.847470	−9.029206	2.127627
N	4.576939	−9.502122	0.285111
C	4.196777	−10.387094	−0.658940
N	3.273421	−9.999607	−1.549081
C	4.762577	−11.703838	−0.714642
C	5.679583	−12.026248	0.229445
H	2.892968	−9.040518	−1.530961
H	3.028748	−10.612350	−2.315993
H	4.477428	−12.407751	−1.486988
H	6.181827	−12.984783	0.254960
H	4.862391	−15.789679	−6.007352
H	6.922128	−17.007872	0.390003
H	12.061131	0.073695	1.225272
H	5.410931	2.591782	−0.766811

d(GCG)$^{H+}$

E: −27427.04 kcal mol−1

O	−0.264601	−0.384113	−0.606999
C	−0.279493	0.010709	0.785080
C	1.124736	−0.065966	1.373383
O	1.571371	−1.449095	1.545292
C	2.644346	−1.767357	0.624066
C	2.215848	0.614116	0.530085
C	2.808619	−0.534972	−0.282884
O	3.157566	1.216058	1.497878
H	−1.188055	−0.447322	−0.907369
H	−0.936694	−0.647301	1.367183
H	−0.636811	1.045481	0.888142
H	1.089006	0.387539	2.369036
H	3.549004	−1.987797	1.199676
H	1.835654	1.424177	−0.092838
H	2.209815	−0.641360	−1.188823
H	3.841700	−0.371294	−0.581566
N	2.336449	−2.994033	−0.101032
C	1.401380	−3.172532	−1.124582
N	1.353062	−4.419445	−1.552768
C	2.290561	−5.107448	−0.780492
C	2.685963	−6.476732	−0.794246
O	2.250497	−7.392678	−1.534795
N	3.681982	−6.749425	0.160824
C	4.211532	−5.823966	1.038576
N	5.116253	−6.273566	1.932423
N	3.849059	−4.535866	1.047355
C	2.906271	−4.240401	0.133468
H	0.779861	−2.355016	−1.462089
H	4.023725	−7.737053	0.207342
H	5.466867	−7.241203	1.905470
H	5.637149	−5.575566	2.447895
P	4.640656	1.735946	1.206631
O	4.728789	1.968883	−0.376411
O	4.993677	2.882889	2.086553
O	5.588133	0.442067	1.369734
C	5.766552	−0.098605	2.727085
C	6.893805	−1.113943	2.722439
O	6.493579	−2.304272	1.990744
C	7.473776	−2.613490	0.964132
C	8.210562	−0.620367	2.081684
C	8.223681	−1.298992	0.714379
O	9.287364	−1.080634	2.978511
H	4.834024	−0.575562	3.042535
H	6.010632	0.720541	3.410034
H	7.082334	−1.387852	3.767086
H	8.140466	−3.406442	1.307765
H	8.276665	0.466268	2.022281
H	7.679711	−0.659299	0.015353
H	9.222924	−1.453960	0.315937
N	6.772284	−3.162872	−0.194772
C	6.918784	−4.544916	−0.515822
O	7.702377	−5.243790	0.157964
N	6.191017	−5.044077	−1.548449
C	5.358590	−4.261463	−2.262684
N	4.671738	−4.818433	−3.267126
C	5.192678	−2.870548	−1.947114
C	5.904175	−2.377806	−0.904362
H	4.769654	−5.823118	−3.487104
H	4.015694	−4.259003	−3.795613
H	4.508352	−2.244177	−2.506466
H	5.814935	−1.351935	−0.569749
P	10.863174	−1.035121	2.716797
O	11.015767	−0.048533	1.463073
O	11.618334	−0.701192	3.955483
O	11.249521	−2.458253	2.073643
C	11.362648	−3.623728	2.968105
C	12.121964	−4.738040	2.268314
O	11.317195	−5.310035	1.198308
C	11.979676	−5.140619	−0.082189
C	13.464799	−4.322665	1.635038
C	13.114180	−4.129806	0.154514
O	14.389385	−5.420951	1.843712
H	10.353364	−3.959658	3.224192
H	11.895594	−3.325528	3.875773
H	12.297551	−5.519902	3.017628
H	12.354124	−6.112214	−0.417555
H	13.862476	−3.407781	2.086521
H	12.763050	−3.108059	−0.003731
H	13.965921	−4.310479	−0.505167
H	15.234533	−5.177832	1.423132
N	11.002310	−4.723499	−1.074366
C	10.412113	−3.467767	−1.208754
N	9.512005	−3.425994	−2.170380
C	9.488650	−4.718157	−2.698618
C	8.719229	−5.280045	−3.758721
O	7.872173	−4.704340	−4.482535
N	9.007800	−6.645867	−3.951723
C	9.946341	−7.367968	−3.242293
N	10.126963	−8.657214	−3.603692
N	10.676644	−6.839756	−2.253493
C	10.404782	−5.540375	−2.024977
H	10.697459	−2.646472	−0.569143
H	8.470740	−7.136666	−4.706016
H	9.498967	−9.124310	−4.277233
H	10.672209	−9.233880	−2.975361
O	5.868162	−11.298438	−10.792570
C	7.050162	−12.113886	−10.979462
C	7.532806	−12.654875	−9.638666
O	8.108969	−11.592852	−8.814112
C	7.336405	−11.403907	−7.598952
C	6.439416	−13.310071	−8.773469
C	6.042098	−12.206469	−7.798374
O	7.081116	−14.477169	−8.127347
H	5.683451	−10.844813	−11.633493
H	7.859155	−11.524605	−11.428630
H	6.829033	−12.967181	−11.636422
H	8.324910	−13.382958	−9.842473
H	7.915526	−11.753781	−6.742996
H	5.600827	−13.690274	−9.357326
H	5.274850	−11.598757	−8.282167
H	5.629720	−12.573896	−6.862343
N	7.129391	−9.969092	−7.367776
C	7.796060	−9.325220	−6.285516
O	8.585398	−9.985837	−5.574676
N	7.544034	−8.008384	−6.067182
C	6.716058	−7.313160	−6.873346
N	6.490035	−6.023447	−6.587117
C	6.107348	−7.924129	−8.020138
C	6.346472	−9.242461	−8.229853
H	6.946585	−5.568354	−5.781034
H	5.886041	−5.476641	−7.186667
H	5.488873	−7.351713	−8.701893
H	5.960174	−9.780697	−9.089098
P	6.558641	−15.344669	−6.894945
O	5.007414	−14.972085	−6.748533
O	6.907558	−16.783841	−7.043742
O	7.181346	−14.649208	−5.578130
C	8.626007	−14.797094	−5.340250
C	8.956581	−14.457480	−3.895621
O	8.827498	−13.031201	−3.640653
C	7.764289	−12.767469	−2.689241
C	8.076882	−15.162955	−2.847072
C	7.033639	−14.106631	−2.489539
O	8.979423	−15.517062	−1.735158
H	9.160952	−14.119769	−6.012305

H	8.919152	−15.830788	−5.546774
H	10.005881	−14.732621	−3.742036
H	8.204036	−12.387965	−1.761330
H	7.634858	−16.090397	−3.212779
H	6.206374	−14.204006	−3.194950
H	6.630166	−14.219026	−1.485478
N	6.910701	−11.701587	−3.183799
C	5.978196	−11.764365	−4.220138
N	5.385234	−10.607614	−4.439610
C	5.947390	−9.727685	−3.512839
C	5.694555	−8.346702	−3.260390
O	4.894870	−7.589794	−3.857722
N	6.471848	−7.857093	−2.192830
C	7.379095	−8.601543	−1.461872
N	8.024713	−7.972049	−0.465795
N	7.625894	−9.893907	−1.708142
C	6.898951	−10.389912	−2.723424
H	5.802007	−12.683187	−4.758011
H	6.353404	−6.844069	−1.951815
H	7.891142	−6.969917	−0.258020
H	8.702862	−8.507469	0.058965
P	8.570198	−15.959017	−0.254620
O	7.066387	−16.487413	−0.414207
O	9.575240	−16.887231	0.330970
O	8.361100	−14.597632	0.580455
C	9.564736	−13.854049	0.995829
C	9.179383	−12.791621	2.006470
O	8.389279	−11.759320	1.352156
C	7.187429	−11.504602	2.120535
C	8.358681	−13.294161	3.218162
C	6.939008	−12.787309	2.928071
O	8.934759	−12.682753	4.400920
H	10.008081	−13.390364	0.109327
H	10.278654	−14.551465	1.443495
H	10.112923	−12.344336	2.372327
H	7.329540	−10.636170	2.765069
H	8.394630	−14.384409	3.308343
H	6.402468	−13.527960	2.328463
H	6.368011	−12.597630	3.840225
H	8.437175	−13.009275	5.173041
N	6.118834	−11.140647	1.184014
C	5.572717	−9.827222	1.195333
O	5.967807	−9.004142	2.051683
N	4.627801	−9.516432	0.269285
C	4.205924	−10.425778	−0.632664
N	3.257647	−10.054699	−1.504321
C	4.754053	−11.750502	−0.665826
C	5.709556	−12.051051	0.246983
H	2.910959	−9.082291	−1.523389
H	2.985026	−10.687454	−2.245225
H	4.431844	−12.474732	−1.404094
H	6.210486	−13.010375	0.278752
H	4.469617	−15.700147	−6.380361
H	6.766179	−17.051063	0.325450
H	11.916190	0.316281	1.358479
H	5.123862	2.832242	−0.607616

d(GGA)$^{H+}$

E: −27517.16 kcal mol^{-1}

O	−0.132984	−0.131929	−0.642986
C	−0.206387	0.268475	0.745672
C	1.161016	0.138881	1.406772
O	1.546647	−1.260832	1.591032
C	2.659042	−1.609293	0.727744
C	2.317885	0.782344	0.624871
C	2.903890	−0.383869	−0.167939
O	3.233766	1.342151	1.643388
H	−1.041277	−0.162598	−0.991214
H	−0.918656	−0.361587	1.293028
H	−0.528160	1.316678	0.828338
H	1.089538	0.589034	2.402077
H	3.525755	−1.851677	1.350604
H	1.999096	1.610284	−0.009105
H	2.340409	−0.468405	−1.098464
H	3.953932	−0.258457	−0.421326
N	2.359884	−2.829369	−0.009970
C	1.472896	−2.994024	−1.076318
N	1.422551	−4.241133	−1.504468
C	2.312561	−4.942851	−0.688494
C	2.681864	−6.318491	−0.674952
O	2.262533	−7.234815	−1.425136
N	3.632804	−6.600729	0.324688
C	4.129101	−5.681222	1.227660
N	4.982366	−6.141490	2.169504
N	3.780462	−4.390959	1.218753
C	2.895508	−4.083244	0.253552
H	0.884902	−2.165938	−1.446691
H	3.981814	−7.586482	0.367298
H	5.347274	−7.103687	2.134979
H	5.501560	−5.443273	2.687385
P	4.750603	1.799954	1.405308
O	4.933469	2.073502	−0.163903
O	5.117911	2.897564	2.340530
O	5.635135	0.461449	1.528406
C	5.789767	−0.122980	2.870269
C	6.916574	−1.139976	2.859832
O	6.519150	−2.339253	2.138115
C	7.458692	−2.611866	1.065595
C	8.226716	−0.648928	2.206983
C	8.185424	−1.285155	0.819970
O	9.311536	−1.152666	3.070547
H	4.851476	−0.607530	3.155336
H	6.023798	0.674671	3.581473
H	7.109769	−1.410619	3.904122
H	8.146236	−3.408437	1.370731
H	8.314534	0.437548	2.179021
H	7.587407	−0.635064	0.176846
H	9.161685	−1.410702	0.356444
N	6.742015	−3.129594	−0.080122
C	5.884659	−2.444835	−0.941971
N	5.349797	−3.230019	−1.855223
C	5.870093	−4.499838	−1.593334
C	5.673062	−5.749598	−2.249863
O	4.929199	−5.993339	−3.228235
N	6.447574	−6.776009	−1.672972
C	7.296976	−6.621571	−0.593467
N	7.965209	−7.713503	−0.171905
N	7.455729	−5.455716	0.042872
C	6.739373	−4.454677	−0.493211
H	5.710792	−1.384491	−0.838572
H	6.410597	−7.706093	−2.154308
H	8.013861	−8.564092	−0.750766
H	8.689637	−7.553641	0.517412
P	10.859909	−1.316170	2.711824
O	11.104554	−0.357701	1.450837
O	11.718544	−1.103754	3.908366
O	11.000226	−2.769528	2.034653
C	10.957019	−3.954804	2.912547
C	11.705014	−5.108961	2.265903
O	10.951439	−5.653276	1.144729
C	11.688041	−5.480134	−0.090661
C	13.102429	−4.755184	1.721654
C	12.848624	−4.523105	0.226344
O	13.954211	−5.903674	1.963696
H	9.908977	−4.225020	3.069542

H	11.424414	−3.705983	3.869652
H	11.791860	−5.895531	3.025353
H	12.038975	−6.457778	−0.433719
H	13.519184	−3.868765	2.211110
H	12.551118	−3.484697	0.065740
H	13.729084	−4.731964	−0.385611
H	14.833042	−5.702277	1.592987
N	10.777942	−5.002426	−1.123585
C	10.218294	−3.737011	−1.248645
N	9.357745	−3.646752	−2.247819
C	9.331399	−4.923108	−2.809282
C	8.606749	−5.480269	−3.889778
N	7.742676	−4.787543	−4.656042
N	8.812876	−6.798893	−4.163949
C	9.679149	−7.513067	−3.421480
N	10.419396	−7.084101	−2.387103
C	10.204307	−5.781564	−2.120432
H	10.484745	−2.938404	−0.572010
H	7.162232	−5.293451	−5.335511
H	7.473134	−3.854043	−4.372715
H	9.772603	−8.560705	−3.699127
O	5.691668	−11.676175	−10.699234
C	6.917268	−12.378499	−11.017852
C	7.594359	−12.855472	−9.739014
O	8.152812	−11.731932	−8.983816
C	7.506675	−11.605019	−7.694692
C	6.669316	−13.598233	−8.758403
C	6.276543	−12.525869	−7.747025
O	7.484911	−14.682266	−8.168634
H	5.362926	−11.265296	−11.517846
H	7.611159	−11.720867	−11.555761
H	6.705953	−13.256511	−11.644420
H	8.426073	−13.506930	−10.026073
H	8.203607	−11.891771	−6.904692
H	5.813211	−14.064304	−9.246468
H	5.405040	−11.996102	−8.137129
H	6.016457	−12.932628	−6.772863
N	7.194006	−10.190829	−7.443188
C	7.868930	−9.521307	−6.407481
O	8.726081	−10.057346	−5.698015
N	7.476998	−8.211281	−6.229392
C	6.539718	−7.504637	−6.974877
O	6.288921	−6.311960	−6.694741
C	5.914282	−8.239404	−8.062553
C	4.911710	−7.540199	−8.939613
C	6.279710	−9.533763	−8.248110
H	7.960372	−7.690921	−5.447337
H	5.359783	−6.663288	−9.422993
H	4.060059	−7.180593	−8.348871
H	4.537503	−8.214338	−9.715455
H	5.885529	−10.129483	−9.065953
P	7.087944	−15.622525	−6.941087
O	5.496735	−15.467783	−6.843157
O	7.649866	−16.991967	−7.093380
O	7.577577	−14.861142	−5.604672
C	9.009173	−14.914979	−5.267960
C	9.220736	−14.382055	−3.863518
O	8.943405	−12.954598	−3.828602
C	8.000696	−12.650739	−2.773182
C	8.342606	−15.035407	−2.771355
C	7.285761	−13.975487	−2.467937
O	9.245425	−15.319575	−1.640313
H	9.561722	−14.300226	−5.984600
H	9.352709	−15.951655	−5.327874
H	10.276286	−14.543099	−3.615130
H	8.525361	−12.254807	−1.901265
H	7.910987	−15.985920	−3.085861
H	6.445738	−14.137624	−3.147152
H	6.904580	−14.023788	−1.449694
N	7.117649	−11.572852	−3.223944
C	7.233312	−10.272926	−2.643509
O	8.096101	−10.073824	−1.765802
N	6.371784	−9.309867	−3.059929
C	5.465875	−9.546433	−4.027993
N	4.652307	−8.547280	−4.389114
C	5.375453	−10.830761	−4.662874
C	6.223050	−11.798398	−4.234185
H	4.739345	−7.611993	−3.954118
H	3.958776	−8.698267	−5.109684
H	4.667382	−11.016799	−5.461096
H	6.244438	−12.789122	−4.669704
P	8.825156	−15.723861	−0.151573
O	7.344283	−16.308067	−0.329857
O	9.851067	−16.596593	0.481011
O	8.553374	−14.347089	0.639254
C	9.719492	−13.577163	1.111627
C	9.270325	−12.547355	2.129598
O	8.489699	−11.512074	1.467012
C	7.256732	−11.292147	2.192631
C	8.406366	−13.094011	3.291631
C	6.992944	−12.602132	2.950855
O	8.918724	−12.503756	4.513673
H	10.177636	−13.083182	0.249586
H	10.437647	−14.262943	1.569726
H	10.178386	−12.092295	2.546541
H	7.359137	−10.440352	2.866354
H	8.457330	−14.185414	3.356250
H	6.498640	−13.330894	2.302053
H	6.375471	−12.450683	3.839511
H	8.392826	−12.857730	5.254256
N	6.220804	−10.917876	1.222993
C	5.591791	−9.643750	1.297959
O	5.891666	−8.863062	2.228707
N	4.669603	−9.328143	0.350944
C	4.323684	−10.207927	−0.610429
N	3.379854	−9.843066	−1.488669
C	4.944067	−11.498416	−0.697328
C	5.889774	−11.796613	0.226703
H	2.976346	−8.892535	−1.468173
H	3.126275	−10.469647	−2.240964
H	4.677371	−12.199893	−1.478352
H	6.440732	−12.728903	0.226102
H	5.064653	−16.159925	−6.305345
H	6.994909	−16.757782	0.464352
H	11.965257	0.103392	1.483068
H	4.979795	3.025952	−0.377272

d(GGC)$^{H+}$

E: −27428.90 kcal mol^{-1}

O	−0.296096	−0.185191	−0.355825
C	−0.267271	0.212812	1.034887
C	1.146334	0.091235	1.592002
O	1.557900	−1.307214	1.721775
C	2.610730	−1.625178	0.776649
C	2.239447	0.762629	0.742622
C	2.776422	−0.382565	−0.112044
O	3.217398	1.317880	1.705180
H	−1.228357	−0.240611	−0.629079
H	−0.930959	−0.424027	1.632878
H	−0.590303	1.258136	1.144804
H	1.141529	0.523270	2.597889
H	3.520971	−1.864951	1.335313
H	1.869793	1.599389	0.149173
H	2.140829	−0.455561	−0.995755
H	3.800632	−0.242224	−0.449743

N	2.283238	−2.836375	0.037373
C	1.376090	−2.992330	−1.013628
N	1.341187	−4.229027	−1.472033
C	2.262371	−4.932904	−0.693176
C	2.669383	−6.297479	−0.731216
O	2.263498	−7.199232	−1.505808
N	3.644833	−6.585389	0.243026
C	4.135504	−5.680106	1.163687
N	5.014605	−6.145949	2.077925
N	3.758716	−4.397936	1.196671
C	2.847390	−4.085742	0.258086
H	0.763950	−2.168557	−1.352455
H	4.010414	−7.566370	0.256823
H	5.408852	−7.094973	2.003457
H	5.521358	−5.449535	2.610556
P	4.741033	1.715435	1.421242
O	4.894299	1.952706	−0.156420
O	5.173136	2.817886	2.322349
O	5.578485	0.346095	1.560399
C	5.743971	−0.206613	2.915473
C	6.917511	−1.171139	2.939507
O	6.586084	−2.411986	2.255102
C	7.486812	−2.639143	1.142505
C	8.201771	−0.633666	2.275855
C	8.192667	−1.296014	0.899616
O	9.314155	−1.068549	3.137268
H	4.824054	−0.726448	3.197602
H	5.933428	0.613410	3.614233
H	7.116784	−1.403673	3.991455
H	8.190660	−3.438690	1.398303
H	8.232463	0.455100	2.222986
H	7.601538	−0.667568	0.230141
H	9.183403	−1.410510	0.464319
N	6.732315	−3.125926	0.002637
C	5.838928	−2.409102	−0.796638
N	5.299114	−3.150082	−1.742936
C	5.847193	−4.422347	−1.567909
C	5.665479	−5.630377	−2.304712
O	4.922003	−5.819879	−3.295198
N	6.450595	−6.681813	−1.795516
C	7.318216	−6.581035	−0.723215
N	8.012025	−7.681920	−0.392714
N	7.485527	−5.448166	−0.028576
C	6.741398	−4.427185	−0.486252
H	5.640700	−1.363015	−0.618063
H	6.375594	−7.602027	−2.289434
H	7.926998	−8.569509	−0.909985
H	8.648765	−7.614357	0.389621
P	10.877104	−0.989518	2.788092
O	11.001211	−0.004128	1.530858
O	11.684451	−0.663599	3.995101
O	11.224322	−2.398214	2.095608
C	11.241317	−3.594275	2.958139
C	11.856180	−4.751303	2.195439
O	10.949777	−5.173431	1.137509
C	11.662661	−5.231941	−0.121821
C	13.220746	−4.451348	1.531129
C	12.869894	−4.297844	0.045142
O	14.075084	−5.596388	1.782669
H	10.212502	−3.832136	3.244852
H	11.833892	−3.379669	3.852354
H	11.978329	−5.578819	2.906055
H	11.965144	−6.257838	−0.334919
H	13.680500	−3.546426	1.940550
H	12.591326	−3.260313	−0.159096
H	13.698580	−4.572874	−0.611638
H	14.942009	−5.411029	1.377259
N	10.730631	−4.857877	−1.191044
C	10.351628	−5.813942	−2.172524
O	10.863681	−6.956185	−2.146927
N	9.443964	−5.443943	−3.113052
C	8.921337	−4.200791	−3.130700
N	8.045993	−3.901223	−4.100229
C	9.284259	−3.227312	−2.142073
C	10.176303	−3.606193	−1.194378
H	7.750898	−4.617591	−4.782070
H	7.583708	−3.001335	−4.098932
H	8.849413	−2.235257	−2.143104
H	10.488362	−2.956149	−0.386394
O	6.077679	−11.492889	−10.874283
C	7.234882	−12.342521	−11.055102
C	7.664503	−12.939900	−9.720128
O	8.257542	−11.931228	−8.840496
C	7.398988	−11.663524	−7.704968
C	6.527969	−13.578711	−8.904164
C	6.109390	−12.468391	−7.943382
O	7.117602	−14.758196	−8.236468
H	5.927213	−11.014577	−11.708271
H	8.075675	−11.769484	−11.465302
H	7.003435	−13.169162	−11.742264
H	8.439287	−13.685201	−9.925609
H	7.921894	−11.968264	−6.792814
H	5.705247	−13.937922	−9.523033
H	5.362113	−11.856762	−8.450984
H	5.667868	−12.835939	−7.019775
N	7.175564	−10.229639	−7.562922
C	6.339678	−9.407042	−8.324137
N	6.355405	−8.153249	−7.914210
C	7.242883	−8.131710	−6.836893
C	7.648039	−7.065276	−5.981569
O	7.275652	−5.866832	−6.021545
N	8.568885	−7.490133	−5.007141
C	9.053134	−8.777535	−4.882701
N	9.981281	−8.994918	−3.928720
N	8.659417	−9.784609	−5.671366
C	7.770442	−9.410765	−6.610034
H	5.793520	−9.795521	−9.172080
H	8.898356	−6.761484	−4.332076
H	10.232926	−8.263526	−3.248161
H	10.197779	−9.959705	−3.712858
P	6.501368	−15.597623	−7.023111
O	4.947966	−15.212144	−6.956486
O	6.842596	−17.041968	−7.130621
O	7.047838	−14.873466	−5.689012
C	8.489366	−14.982209	−5.409396
C	8.778573	−14.457459	−4.015331
O	8.583066	−13.015935	−3.974281
C	7.674792	−12.663281	−2.901882
C	7.899700	−15.057073	−2.894887
C	6.894657	−13.948517	−2.590967
O	8.810384	−15.340268	−1.772002
H	9.037463	−14.392933	−6.150405
H	8.789567	−16.032016	−5.478185
H	9.831387	−14.676220	−3.802555
H	8.232757	−12.299907	−2.036834
H	7.425719	−15.996120	−3.181348
H	6.044440	−14.066531	−3.267141
H	6.518114	−13.977356	−1.570587
N	6.849845	−11.536066	−3.340445
C	7.000903	−10.262127	−2.714696
O	7.810995	−10.140238	−1.775125
N	6.236640	−9.234100	−3.165773
C	5.362118	−9.399048	−4.177533
N	4.625997	−8.346595	−4.552428
C	5.219390	−10.666463	−4.837240
C	5.986338	−11.691436	−4.392065
H	4.737290	−7.428598	−4.090041
H	3.986662	−8.432650	−5.331732

H	4.535414	−10.794838	−5.667172
H	5.968024	−12.675399	−4.844162
P	8.381126	−15.818942	−0.302410
O	6.845326	−16.257888	−0.426801
O	9.337979	−16.818653	0.245242
O	8.243718	−14.469501	0.562255
C	9.485604	−13.766298	0.932647
C	9.172273	−12.683223	1.947093
O	8.420120	−11.614436	1.306430
C	7.226237	−11.323286	2.073339
C	8.353096	−13.139508	3.176738
C	6.947684	−12.589658	2.897231
O	8.964924	−12.528600	4.341821
H	9.917069	−13.323808	0.029808
H	10.186947	−14.487044	1.363434
H	10.134031	−12.280191	2.289907
H	7.388721	−10.449449	2.705470
H	8.354043	−14.228872	3.283878
H	6.379995	−13.317995	2.311236
H	6.393686	−12.372360	3.813641
H	8.462315	−12.820022	5.124742
N	6.163061	−10.947418	1.133730
C	5.616442	−9.633920	1.156737
O	6.002777	−8.823951	2.029248
N	4.678329	−9.312477	0.227689
C	4.249616	−10.217099	−0.675709
N	3.306704	−9.838311	−1.549246
C	4.786106	−11.546859	−0.709740
C	5.744475	−11.854315	0.198485
H	2.944003	−8.871410	−1.549821
H	2.981175	−10.489394	−2.251396
H	4.449750	−12.270557	−1.442247
H	6.238411	−12.817470	0.230376
H	4.368237	−15.994439	−6.875334
H	6.660243	−17.114859	0.004373
H	11.660179	0.703614	1.669876
H	5.075693	2.886666	−0.379558

d(GGG)$^{H+}$

E: −27427.17 kcal mol^{-1}

O	−0.185855	−0.164338	−0.604420
C	−0.281690	0.193471	0.794559
C	1.078742	0.061455	1.469747
O	1.482616	−1.337878	1.610227
C	2.611224	−1.638399	0.749298
C	2.237309	0.747808	0.727281
C	2.845313	−0.380764	−0.102233
O	3.135643	1.275112	1.778176
H	−1.089777	−0.203365	−0.962912
H	−0.991989	−0.462343	1.313556
H	−0.618908	1.234345	0.904378
H	0.986790	0.475395	2.479054
H	3.474667	−1.885870	1.374943
H	1.917103	1.597033	0.122739
H	2.288130	−0.440145	−1.038563
H	3.895137	−0.233114	−0.345015
N	2.342570	−2.838544	−0.030203
C	1.479717	−2.986775	−1.118480
N	1.459028	−4.222017	−1.582381
C	2.344066	−4.932174	−0.768129
C	2.735050	−6.301569	−0.783656
O	2.346594	−7.203280	−1.567583
N	3.667481	−6.596420	0.229483
C	4.131060	−5.693838	1.166591
N	4.971865	−6.165543	2.113439
N	3.762602	−4.409107	1.184487
C	2.893206	−4.090199	0.209100
H	0.885870	−2.158231	−1.478202
H	4.028631	−7.578740	0.255661
H	5.350022	−7.122368	2.063575
H	5.468725	−5.473519	2.660690
P	4.650467	1.756826	1.578846
O	4.852635	2.080177	0.021425
O	4.990106	2.830759	2.551544
O	5.550005	0.426325	1.678498
C	5.706459	−0.181524	3.009959
C	6.865038	−1.162717	2.994604
O	6.514674	−2.360037	2.247203
C	7.467234	−2.582714	1.174804
C	8.167818	−0.617409	2.369138
C	8.176005	−1.237726	0.974073
O	9.257831	−1.077584	3.251729
H	4.779877	−0.700505	3.272059
H	5.906259	0.607892	3.740399
H	7.053480	−1.446399	4.036362
H	8.164081	−3.378386	1.460097
H	8.212841	0.471866	2.351598
H	7.587055	−0.588371	0.322075
H	9.168938	−1.339601	0.540223
N	6.767365	−3.074305	0.006251
C	5.907278	−2.374907	−0.842140
N	5.391904	−3.138945	−1.783875
C	5.926629	−4.409257	−1.556221
C	5.757735	−5.639036	−2.256092
O	5.036269	−5.859872	−3.256945
N	6.528441	−6.677073	−1.693930
C	7.358909	−6.546163	−0.595895
N	8.029000	−7.642287	−0.194314
N	7.499898	−5.396191	0.072412
C	6.782682	−4.387138	−0.445518
H	5.714271	−1.321489	−0.705274
H	6.497509	−7.599933	−2.188451
H	8.042158	−8.508160	−0.752402
H	8.730445	−7.505440	0.522320
P	10.808901	−1.236113	2.913210
O	11.046975	−0.189928	1.722246
O	11.642797	−1.092899	4.137410
O	10.975574	−2.649122	2.160982
C	11.017115	−3.879734	2.973106
C	11.732756	−4.976912	2.205074
O	10.901690	−5.446736	1.105686
C	11.617956	−5.316469	−0.148007
C	13.090032	−4.564479	1.595641
C	12.751541	−4.315122	0.120990
O	13.994057	−5.685121	1.771496
H	9.988369	−4.179821	3.192145
H	11.549742	−3.672827	3.905487
H	11.884980	−5.809053	2.904175
H	12.000545	−6.296255	−0.451264
H	13.500678	−3.673378	2.081402
H	12.395527	−3.289214	−0.000909
H	13.606750	−4.470946	−0.540780
H	14.849625	−5.437615	1.375156
N	10.687721	−4.912313	−1.187979
C	10.089079	−3.664056	−1.359006
N	9.270723	−3.627832	−2.391155
C	9.311489	−4.915431	−2.930737
C	8.650675	−5.479215	−4.062324
O	7.861648	−4.910351	−4.854588
N	8.974185	−6.838326	−4.239411
C	9.868927	−7.550048	−3.465618
N	10.126677	−8.822627	−3.839270
N	10.497478	−7.021478	−2.409381
C	10.184064	−5.729249	−2.191173
H	10.299097	−2.843699	−0.688787

H	8.508822	−7.330564	−5.036724
H	9.572289	−9.288793	−4.574028
H	10.601914	−9.404543	−3.160625
O	5.718910	−11.774246	−10.904913
C	6.876403	−12.611247	−11.142729
C	7.477072	−13.060548	−9.816132
O	8.116848	−11.941569	−9.121303
C	7.461091	−11.677471	−7.857519
C	6.463745	−13.650779	−8.817105
C	6.151490	−12.480843	−7.889579
O	7.156947	−14.769316	−8.141104
H	5.454159	−11.383109	−11.755876
H	7.641996	−12.061822	−11.704221
H	6.593445	−13.506329	−11.714530
H	8.252498	−13.801574	−10.035667
H	8.113488	−11.977602	−7.036016
H	5.578419	−14.065109	−9.299671
H	5.348188	−11.898451	−8.345789
H	5.821207	−12.789020	−6.900156
N	7.270785	−10.229537	−7.692888
C	7.959714	−9.538506	−6.654855
O	8.771613	−10.164648	−5.938024
N	7.688591	−8.220684	−6.471208
C	6.826362	−7.565841	−7.275428
N	6.573415	−6.276990	−7.010194
C	6.198479	−8.223237	−8.385855
C	6.448704	−9.545748	−8.555142
H	7.020064	−5.800149	−6.211094
H	5.931955	−5.760387	−7.597839
H	5.548234	−7.684761	−9.065529
H	6.035465	−10.125162	−9.374819
P	6.687420	−15.547848	−6.827849
O	5.134582	−15.189138	−6.667718
O	7.060863	−16.987273	−6.873666
O	7.329870	−14.737922	−5.588482
C	8.779002	−14.864172	−5.361008
C	9.119199	−14.379728	−3.962640
O	8.961642	−12.936089	−3.871776
C	8.024704	−12.589057	−2.823314
C	8.254312	−14.988604	−2.839456
C	7.253888	−13.882328	−2.514673
O	9.178462	−15.292067	−1.730716
H	9.303674	−14.257791	−6.104853
H	9.068454	−15.913487	−5.467646
H	10.172364	−14.625752	−3.785910
H	8.560436	−12.207500	−1.952416
H	7.776063	−15.924343	−3.129908
H	6.396047	−13.997485	−3.181035
H	6.890264	−13.921361	−1.490477
N	7.185626	−11.478557	−3.284815
C	7.280597	−10.203097	−2.649689
O	8.064525	−10.058390	−1.691375
N	6.489246	−9.200284	−3.111694
C	5.632354	−9.393399	−4.133683
N	4.867405	−8.366908	−4.521360
C	5.535315	−10.665482	−4.791126
C	6.337792	−11.661999	−4.343690
H	4.935704	−7.445540	−4.054351
H	4.219991	−8.485243	−5.289453
H	4.859974	−10.816915	−5.624140
H	6.361510	−12.644317	−4.798565
P	8.772053	−15.713816	−0.242220
O	7.286182	−16.286537	−0.415874
O	9.799608	−16.600560	0.367489
O	8.513371	−14.348485	0.571491
C	9.681389	−13.596985	1.067389
C	9.230003	−12.580565	2.098380
O	8.467432	−11.526210	1.445582
C	7.215211	−11.322699	2.143743
C	8.346683	−13.138458	3.239705
C	6.939134	−12.642889	2.879920
O	8.837350	−12.559743	4.476186
H	10.151180	−13.091280	0.218402
H	10.389682	−14.296877	1.519674
H	10.137344	−12.141460	2.533210
H	7.298650	−10.479485	2.830592
H	8.396791	−14.230522	3.294762
H	6.456405	−13.365222	2.215706
H	6.306399	−12.502066	3.759594
H	8.294056	−12.915658	5.203160
N	6.201458	−10.937460	1.155155
C	5.604427	−9.646879	1.203854
O	5.901560	−8.867408	2.136691
N	4.715185	−9.314765	0.231376
C	4.375784	−10.190239	−0.736376
N	3.470089	−9.805736	−1.645855
C	4.965262	−11.496268	−0.798201
C	5.876481	−11.811914	0.154196
H	3.074722	−8.851716	−1.629316
H	3.219913	−10.431289	−2.400133
H	4.703245	−12.195706	−1.582592
H	6.403896	−12.756897	0.173763
H	4.612266	−15.910670	−6.265696
H	6.944367	−16.752187	0.372447
H	11.989469	−0.013750	1.533246
H	4.913287	3.038372	−0.159709

d(GTA)$^{H+}$

E: −27607.69 kcal mol^{-1}

O	−0.245689	−0.216331	−0.058067
C	−0.167360	0.053111	1.361780
C	1.274807	−0.065831	1.839866
O	1.724434	−1.458040	1.882924
C	2.731462	−1.716018	0.876054
C	2.298808	0.677904	0.965212
C	2.873781	−0.415417	0.064345
O	3.279472	1.276323	1.893040
H	−1.186903	−0.244582	−0.304076
H	−0.783579	−0.657731	1.926290
H	−0.516229	1.072719	1.579013
H	1.315557	0.315679	2.864953
H	3.662878	−2.003406	1.374000
H	1.855348	1.495850	0.396938
H	2.265770	−0.457916	−0.840262
H	3.904055	−0.231211	−0.232572
N	2.350057	−2.869410	0.064486
C	1.328652	−2.940666	−0.888302
N	1.220657	−4.140685	−1.424838
C	2.202316	−4.911225	−0.798762
C	2.561934	−6.281026	−0.967663
O	2.064247	−7.114077	−1.764701
N	3.602751	−6.663721	−0.102610
C	4.207924	−5.836711	0.824047
N	5.143199	−6.391093	1.618258
N	3.895466	−4.541731	0.961373
C	2.904038	−4.142620	0.142052
H	0.698951	−2.086366	−1.094615
H	3.907790	−7.662787	−0.156819
H	5.435175	−7.373167	1.511191
H	5.698023	−5.768032	2.190122
P	4.690609	1.921285	1.488368
O	4.654675	2.198411	−0.089308
O	5.025071	3.067224	2.377032
O	5.734004	0.696225	1.519076
C	5.981928	0.072998	2.828345

C	7.061730	−0.978756	2.687163
O	6.554975	−2.103004	1.915205
C	7.530591	−2.464995	0.904067
C	8.357749	−0.502883	1.983984
C	8.285759	−1.165130	0.610026
O	9.464374	−1.002566	2.818872
H	5.055861	−0.387705	3.184566
H	6.305852	0.842377	3.535752
H	7.312926	−1.321687	3.697958
H	8.198181	−3.243961	1.280394
H	8.445541	0.582434	1.934812
H	7.705663	−0.513029	−0.047873
H	9.257907	−1.328097	0.150356
N	6.824850	−3.061502	−0.224196
C	7.002783	−4.436957	−0.469343
O	7.774610	−5.142842	0.180461
N	6.240157	−4.929962	−1.506944
C	5.343434	−4.223622	−2.299760
O	4.696420	−4.809869	−3.191994
C	5.225462	−2.802698	−2.011858
C	4.304049	−1.967081	−2.855518
C	5.956919	−2.303568	−0.983799
H	6.355154	−5.965367	−1.706430
H	4.584547	−2.033812	−3.913346
H	4.340241	−0.917003	−2.555786
H	3.270249	−2.322273	−2.777072
H	5.892374	−1.261377	−0.694824
P	11.029782	−0.991240	2.498186
O	11.158493	−0.054001	1.204936
O	11.839735	−0.631966	3.694184
O	11.359516	−2.444353	1.891188
C	11.471921	−3.582934	2.819417
C	12.188560	−4.734198	2.135390
O	11.343951	−5.317894	1.101107
C	11.972751	−5.192138	−0.198088
C	13.523097	−4.368016	1.456090
C	13.136282	−4.202642	−0.019236
O	14.426215	−5.482984	1.666739
H	10.463209	−3.888903	3.112608
H	12.035626	−3.268363	3.702479
H	12.364450	−5.499113	2.901549
H	12.313886	−6.178674	−0.525629
H	13.954074	−3.452028	1.873380
H	12.803771	−3.177260	−0.193799
H	13.964776	−4.419954	−0.697072
H	15.265519	−5.270943	1.218588
N	10.974800	−4.772369	−1.173585
C	10.400031	−3.516385	−1.308819
N	9.489227	−3.461753	−2.265750
C	9.450841	−4.753315	−2.790129
C	8.696421	−5.337171	−3.835299
N	7.797078	−4.668541	−4.577723
N	8.911608	−6.657546	−4.095328
C	9.806824	−7.347860	−3.366446
N	10.574784	−6.893403	−2.362127
C	10.358867	−5.586034	−2.117079
H	10.695679	−2.696789	−0.670909
H	7.245503	−5.165311	−5.288225
H	7.605678	−3.696471	−4.376859
H	9.904014	−8.399577	−3.629210
O	5.592905	−11.683827	−10.444640
C	6.786204	−12.457207	−10.718723
C	7.448516	−12.882785	−9.414637
O	8.044115	−11.737328	−8.722739
C	7.404567	−11.525925	−7.440183
C	6.504559	−13.541785	−8.391294
C	6.139098	−12.393165	−7.456088
O	7.295602	−14.615147	−7.748176
H	5.285673	−11.304054	−11.286332

H	7.505353	−11.865189	−11.298060
H	6.535353	−13.363279	−11.288362
H	8.258511	−13.575272	−9.665735
H	8.089520	−11.806935	−6.638253
H	5.636278	−14.015750	−8.850098
H	5.304108	−11.853448	−7.907975
H	5.830976	−12.706893	−6.461991
N	7.152353	−10.093248	−7.247495
C	7.843054	−9.417921	−6.227117
O	8.687992	−9.957012	−5.506278
N	7.487523	−8.092018	−6.086480
C	6.600703	−7.371257	−6.878445
O	6.395243	−6.158709	−6.643751
C	5.973714	−8.111610	−7.960449
C	5.034691	−7.396277	−8.893470
C	6.284064	−9.427074	−8.092765
H	7.999747	−7.562424	−5.330829
H	5.540609	−6.559512	−9.390946
H	4.182449	−6.976075	−8.345128
H	4.654252	−8.078558	−9.659109
H	5.881393	−10.033800	−8.898417
P	6.970466	−15.367256	−6.374004
O	5.419236	−15.132843	−6.046124
O	7.459731	−16.771796	−6.412456
O	7.610376	−14.452253	−5.213280
C	9.077301	−14.430321	−5.082679
C	9.468344	−14.126017	−3.644021
O	9.270242	−12.725236	−3.312229
C	8.211713	−12.561877	−2.337418
C	8.688284	−14.931620	−2.591631
C	7.608752	−13.960246	−2.106460
O	9.662751	−15.293690	−1.546665
H	9.473784	−13.661644	−5.751320
H	9.475609	−15.409110	−5.364983
H	10.539493	−14.336648	−3.557027
H	8.631628	−12.136578	−1.422911
H	8.270888	−15.856234	−2.991361
H	6.713825	−14.107997	−2.713071
H	7.345689	−14.108394	−1.060100
N	7.248663	−11.578006	−2.821960
C	6.331574	−11.733695	−3.855123
N	5.669134	−10.624826	−4.131425
C	6.173646	−9.678328	−3.240906
C	5.877328	−8.309661	−3.036791
N	4.973995	−7.624796	−3.753545
N	6.561843	−7.667736	−2.048582
C	7.484121	−8.330921	−1.330223
N	7.852111	−9.617424	−1.452534
C	7.162794	−10.247251	−2.423057
H	6.215228	−12.681743	−4.357019
H	4.825037	−6.623568	−3.577363
H	4.450595	−8.095205	−4.478779
H	7.985832	−7.740930	−0.566430
P	9.290711	−16.158508	−0.242284
O	8.015336	−17.057448	−0.613174
O	10.505533	−16.849126	0.268571
O	8.569344	−15.150208	0.773875
C	9.381369	−14.087342	1.385217
C	8.467726	−13.076853	2.038931
O	7.743356	−12.339316	1.023460
C	6.585062	−11.792202	1.704638
C	7.402686	−13.641097	3.025082
C	6.092192	−12.947714	2.589836
O	7.835408	−13.287890	4.360479
H	9.989100	−13.604570	0.613729
H	10.035797	−14.537122	2.137122
H	9.106836	−12.381915	2.605335
H	6.879309	−10.925685	2.300699
H	7.319084	−14.727840	2.943192

H	5.475605	−13.638664	2.007523
H	5.510798	−12.597456	3.446006
H	7.287976	−13.789677	4.991360
N	5.640399	−11.296094	0.709602
C	5.292934	−9.913337	0.699208
O	5.829567	−9.142798	1.527010
N	4.378878	−9.487800	−0.207174
C	3.829388	−10.336946	−1.099720
N	2.927233	−9.845259	−1.958652
C	4.194691	−11.724094	−1.131016
C	5.088238	−12.153772	−0.206447
H	2.655122	−8.851078	−1.925321
H	2.515517	−10.449682	−2.657372
H	3.768178	−12.404608	−1.857656
H	5.416143	−13.183834	−0.149145
H	12.046916	0.338047	1.095057
H	4.677894	3.151424	−0.303756
H	4.885156	−15.945366	−6.141699
H	8.253306	−17.969189	−0.871454

d(TAA)$^{H+}$

E: −27695.53 kcal mol^{-1}

O	−0.034818	0.054911	−0.672783
C	−0.064227	0.427424	0.726158
C	1.284247	0.139009	1.374540
O	1.510140	−1.301760	1.507802
C	2.657336	−1.714782	0.725688
C	2.503065	0.682787	0.605610
C	2.993727	−0.526591	−0.185727
O	3.456314	1.151077	1.636229
H	−0.942552	0.121932	−1.017092
H	−0.836485	−0.139283	1.260811
H	−0.275925	1.500579	0.834745
H	1.265148	0.565726	2.382708
H	3.482230	−1.971925	1.392698
H	2.264695	1.540161	−0.024418
H	2.419245	−0.571661	−1.113217
H	4.047682	−0.486927	−0.449358
N	2.339361	−2.954372	0.007688
C	3.015541	−4.133417	0.364153
O	3.845095	−4.192133	1.276137
N	2.681970	−5.230409	−0.404138
C	1.723892	−5.290975	−1.410122
O	1.512468	−6.369087	−2.010288
C	1.013039	−4.053614	−1.682138
C	−0.072172	−4.041620	−2.724457
C	1.350439	−2.955207	−0.958786
H	3.173471	−6.127522	−0.149488
H	−0.871990	−4.745940	−2.463799
H	0.319989	−4.348103	−3.701964
H	−0.505949	−3.042130	−2.820541
H	0.835961	−2.006941	−1.082676
P	5.009968	1.476160	1.426070
O	5.247083	1.697882	−0.143383
O	5.452207	2.557805	2.346984
O	5.776746	0.070538	1.603041
C	5.878261	−0.480934	2.965076
C	7.082689	−1.403692	3.066158
O	6.844605	−2.674755	2.401695
C	7.725707	−2.846411	1.265102
C	8.376086	−0.831693	2.457591
C	8.464516	−1.507904	1.087063
O	9.461580	−1.202732	3.381401
H	4.960940	−1.032930	3.187024
H	5.995640	0.342807	3.675090
H	7.232968	−1.609311	4.131459
H	8.410147	−3.676725	1.463694
H	8.365844	0.256382	2.385591
H	7.955383	−0.872415	0.360603
H	9.488577	−1.653991	0.748904
N	6.946473	−3.251338	0.101250
C	6.092594	−2.467371	−0.665122
N	5.457866	−3.153572	−1.599711
C	5.908432	−4.464043	−1.446336
C	5.614960	−5.662162	−2.140170
N	4.728924	−5.751897	−3.144732
N	6.282334	−6.783821	−1.748931
C	7.140273	−6.729679	−0.714871
N	7.462110	−5.659179	0.029186
C	6.826446	−4.549263	−0.387601
H	5.986087	−1.409394	−0.478932
H	4.589194	−6.644776	−3.633595
H	4.265472	−4.917928	−3.478072
H	7.625091	−7.671307	−0.468068
P	11.029550	−1.020568	3.079855
O	11.161108	0.082596	1.924301
O	11.778375	−0.786607	4.344269
O	11.458362	−2.319951	2.241718
C	11.442419	−3.617562	2.936130
C	11.896297	−4.695455	1.974524
O	10.861032	−4.909327	0.970764
C	11.499810	−5.031437	−0.325160
C	13.213054	−4.388323	1.213659
C	12.742837	−4.139009	−0.226822
O	14.053445	−5.564599	1.325782
H	10.425935	−3.822618	3.285281
H	12.122943	−3.569730	3.791463
H	12.030266	−5.617879	2.555003
H	11.764687	−6.077152	−0.517194
H	13.730238	−3.520213	1.632192
H	12.463470	−3.087733	−0.342737
H	13.504150	−4.389738	−0.969092
H	14.903014	−5.361217	0.893378
N	10.545651	−4.660877	−1.352094
C	10.042463	−3.395153	−1.627466
N	9.162376	−3.391371	−2.613011
C	9.074856	−4.723109	−3.015585
C	8.327858	−5.364753	−4.032176
N	7.485903	−4.722871	−4.860053
N	8.492186	−6.710933	−4.170867
C	9.330268	−7.371212	−3.351285
N	10.083792	−6.862177	−2.362169
C	9.922165	−5.530034	−2.241229
H	10.368900	−2.524751	−1.077794
H	6.921063	−5.257649	−5.530969
H	7.298967	−3.739205	−4.716820
H	9.391048	−8.444547	−3.516056
O	5.954856	−11.655105	−10.851102
C	7.231455	−12.302577	−11.071237
C	7.802359	−12.791808	−9.745777
O	8.267035	−11.674115	−8.922854
C	7.514419	−11.584167	−7.691271
C	6.808522	−13.577740	−8.870980
C	6.330009	−12.553446	−7.843243
O	7.571564	−14.693968	−8.275977
H	5.683328	−11.238983	−11.687869
H	7.946033	−11.605335	−11.525552
H	7.112698	−13.169366	−11.736449
H	8.671045	−13.419761	−9.969013
H	8.154815	−11.843723	−6.846036
H	5.991137	−14.013262	−9.445818
H	5.456156	−12.043992	−8.253095
H	6.048772	−13.001606	−6.892353
N	7.119837	−10.184154	−7.463628
C	7.682350	−9.489010	−6.379804

O	8.492801	−9.991741	−5.595788
N	7.237636	−8.190049	−6.244782
C	6.358736	−7.510563	−7.080739
O	6.060762	−6.319977	−6.840088
C	5.856238	−8.268067	−8.215312
C	4.921109	−7.601977	−9.187170
C	6.270615	−9.553355	−8.356570
H	7.667402	−7.647492	−5.447358
H	5.382937	−6.708450	−9.624917
H	4.003318	−7.274686	−8.682741
H	4.649067	−8.286638	−9.995527
H	5.969583	−10.162384	−9.203695
P	7.023560	−15.700220	−7.155075
O	5.428042	−15.561259	−7.189451
O	7.600964	−17.061808	−7.321352
O	7.351121	−14.991392	−5.747005
C	8.763384	−14.896866	−5.351284
C	8.849719	−14.276508	−3.972795
O	8.455465	−12.879632	−4.044640
C	7.565514	−12.581656	−2.940067
C	7.954694	−14.940722	−2.894460
C	6.855141	−13.908702	−2.648970
O	8.830315	−15.159058	−1.729970
H	9.296791	−14.273697	−6.075385
H	9.200051	−15.899864	−5.336297
H	9.895954	−14.341788	−3.650079
H	8.135096	−12.230693	−2.075739
H	7.567568	−15.912575	−3.200232
H	6.054151	−14.093495	−3.369065
H	6.425827	−13.957546	−1.650527
N	6.702600	−11.468761	−3.321877
C	6.905544	−10.220821	−2.700092
O	7.751117	−10.033244	−1.824239
N	6.064619	−9.221779	−3.142397
C	5.088687	−9.320087	−4.126284
O	4.404078	−8.323494	−4.437641
C	4.938077	−10.630992	−4.739665
C	3.885678	−10.821466	−5.796748
C	5.755573	−11.626570	−4.312191
H	6.165804	−8.292215	−2.643688
H	2.894348	−10.565496	−5.404631
H	3.860894	−11.857214	−6.144214
H	4.066997	−10.164437	−6.655137
H	5.706218	−12.620507	−4.739394
P	8.409388	−15.628764	−0.261065
O	6.888528	−16.105521	−0.423516
O	9.379955	−16.601761	0.310944
O	8.246559	−14.276290	0.595569
C	9.466978	−13.592541	1.059386
C	9.113300	−12.594537	2.147469
O	8.381451	−11.467348	1.581603
C	7.087025	−11.338881	2.219213
C	8.245697	−13.153190	3.294101
C	6.833047	−12.677323	2.931724
O	8.729600	−12.553689	4.522866
H	9.918777	−13.076806	0.206799
H	10.164186	−14.337453	1.454107
H	10.059673	−12.218156	2.554835
H	7.106886	−10.496744	2.917861
H	8.309981	−14.244238	3.358424
H	6.371248	−13.394786	2.249379
H	6.189339	−12.561936	3.806785
H	8.174775	−12.887099	5.251743
N	6.096769	−10.997188	1.208321
C	5.615540	−11.814729	0.195274
N	4.739633	−11.210227	−0.588546
C	4.636221	−9.919148	−0.071510
C	3.850623	−8.802654	−0.444571
N	2.987840	−8.805580	−1.474272
N	3.982409	−7.669454	0.301368
C	4.842204	−7.646049	1.335484
N	5.626287	−8.644181	1.777747
C	5.479505	−9.762697	1.039759
H	5.956501	−12.834469	0.094151
H	2.480804	−7.947653	−1.725552
H	2.913483	−9.625015	−2.061126
H	4.906370	−6.696053	1.862598
H	4.972328	−16.425133	−7.208347
H	6.602251	−16.736504	0.265569
H	11.547631	0.920928	2.244119
H	5.457539	2.625879	−0.365292

d(TCA)$^{H+}$

E: −27604.48 kcal mol^{-1}

O	−0.169423	0.003455	−0.719824
C	−0.307003	0.307690	0.689214
C	1.012712	0.054748	1.407522
O	1.297896	−1.377738	1.505308
C	2.502651	−1.715746	0.777412
C	2.241938	0.681225	0.725687
C	2.852335	−0.475291	−0.060611
O	3.114716	1.167652	1.815848
H	−1.053999	0.052530	−1.122551
H	−1.081461	−0.319998	1.147130
H	−0.579135	1.363257	0.829786
H	0.917674	0.442857	2.426763
H	3.296754	−1.975588	1.480094
H	1.992447	1.539443	0.101792
H	2.370085	−0.511720	−1.039377
H	3.923224	−0.367132	−0.213891
N	2.266604	−2.933942	−0.011214
C	2.953955	−4.109980	0.336468
O	3.747759	−4.178883	1.279561
N	2.684344	−5.187553	−0.481279
C	1.799691	−5.229989	−1.552619
O	1.654667	−6.289016	−2.202206
C	1.086028	−3.994257	−1.830776
C	0.086752	−3.958571	−2.954853
C	1.345339	−2.919037	−1.043384
H	3.199228	−6.078709	−0.245478
H	−0.711710	−4.693179	−2.792832
H	0.564759	−4.209199	−3.910027
H	−0.364350	−2.965960	−3.041256
H	0.818117	−1.977912	−1.167528
P	4.603554	1.725763	1.666496
O	4.702877	2.123789	0.118148
O	4.896278	2.775769	2.679767
O	5.584715	0.448435	1.746520
C	5.843654	−0.134064	3.071836
C	6.930224	−1.185920	2.960705
O	6.445616	−2.320328	2.190719
C	7.390383	−2.641422	1.137249
C	8.240596	−0.717833	2.284694
C	8.173552	−1.345851	0.895476
O	9.331190	−1.244861	3.128123
H	4.921769	−0.592631	3.441575
H	6.161565	0.658086	3.755691
H	7.156973	−1.518163	3.980726
H	8.040959	−3.459282	1.453588
H	8.348170	0.366889	2.260570
H	7.618663	−0.660899	0.250099
H	9.147489	−1.513436	0.440653
N	6.654782	−3.148583	−0.018710
C	6.727373	−4.536561	−0.343821
O	7.421200	−5.291100	0.366723

N	6.041604	−4.974825	−1.430524
C	5.271627	−4.142852	−2.157189
N	4.614046	−4.648832	−3.208914
C	5.146952	−2.754778	−1.814701
C	5.852218	−2.312808	−0.744235
H	4.710702	−5.648507	−3.458147
H	4.037663	−4.045071	−3.779770
H	4.513243	−2.086638	−2.385289
H	5.808836	−1.286543	−0.404247
P	10.879088	−1.390885	2.768892
O	11.079544	−0.382121	1.539462
O	11.738073	−1.201577	3.969129
O	11.052197	−2.825243	2.057411
C	11.153711	−4.029513	2.904270
C	11.941394	−5.111458	2.183958
O	11.155760	−5.687489	1.100241
C	11.807013	−5.464121	−0.174145
C	13.273533	−4.650671	1.560929
C	12.915213	−4.429362	0.085413
O	14.222146	−5.732620	1.740298
H	10.139737	−4.379346	3.117229
H	11.660400	−3.765914	3.836738
H	12.133019	−5.903819	2.917745
H	12.203467	−6.413372	−0.546262
H	13.651188	−3.738311	2.034309
H	12.534462	−3.414374	−0.049045
H	13.770418	−4.570356	−0.579411
H	15.060548	−5.461636	1.323190
N	10.807333	−5.040525	−1.145425
C	10.124970	−3.831477	−1.173403
N	9.217754	−3.769217	−2.132161
C	9.290825	−5.008503	−2.767064
C	8.586485	−5.560349	−3.862927
N	7.642353	−4.900210	−4.554182
N	8.904771	−6.833923	−4.231628
C	9.866109	−7.502715	−3.566999
N	10.605454	−7.068671	−2.533401
C	10.269959	−5.815737	−2.166898
H	10.341916	−3.054032	−0.456145
H	7.125405	−5.372884	−5.307219
H	7.345962	−3.986543	−4.239769
H	10.048626	−8.517358	−3.915985
O	5.622476	−11.554922	−10.811983
C	6.843178	−12.269436	−11.121774
C	7.491734	−12.781078	−9.841333
O	8.057971	−11.683724	−9.056010
C	7.391086	−11.569429	−7.773421
C	6.539908	−13.526736	−8.888916
C	6.147972	−12.467024	−7.863549
O	7.330675	−14.637880	−8.312647
H	5.313785	−11.126112	−11.629178
H	7.555952	−11.612618	−11.635562
H	6.630138	−13.132554	−11.768387
H	8.315919	−13.441939	−10.129154
H	8.069806	−11.884846	−6.978723
H	5.684616	−13.971302	−9.397947
H	5.291114	−11.916616	−8.256754
H	5.867857	−12.883063	−6.898947
N	7.103133	−10.156280	−7.500391
C	7.803733	−9.513311	−6.465331
O	8.653774	−10.075155	−5.769140
N	7.459464	−8.189507	−6.284528
C	6.553219	−7.447805	−7.031996
O	6.360755	−6.239572	−6.766615
C	5.897086	−8.158783	−8.116372
C	4.922917	−7.421865	−8.994783
C	6.213266	−9.465808	−8.302696
H	7.982746	−7.692359	−5.517111
H	5.402556	−6.560263	−9.475641
H	4.082637	−7.033972	−8.405493
H	4.527071	−8.080762	−9.773038
H	5.798897	−10.043477	−9.123264
P	6.932147	−15.546858	−7.063293
O	5.336569	−15.415563	−6.994133
O	7.514760	−16.911939	−7.173508
O	7.387769	−14.743286	−5.740451
C	8.806678	−14.785218	−5.354277
C	8.967426	−14.321485	−3.917024
O	8.726390	−12.890152	−3.809464
C	7.674566	−12.625541	−2.845005
C	8.026906	−15.004618	−2.899305
C	6.937729	−13.958996	−2.674512
O	8.860402	−15.310809	−1.719115
H	9.370412	−14.126332	−6.021301
H	9.173059	−15.811194	−5.451956
H	10.008189	−14.519866	−3.636026
H	8.117613	−12.273714	−1.907480
H	7.632043	−15.956491	−3.255928
H	6.199294	−14.088523	−3.469111
H	6.419601	−14.044943	−1.721843
N	6.841608	−11.538809	−3.315438
C	5.888252	−11.563558	−4.333017
N	5.319558	−10.390259	−4.525746
C	5.918581	−9.539094	−3.593781
C	5.695129	−8.161157	−3.304174
O	4.909681	−7.374982	−3.882960
N	6.475576	−7.718268	−2.218901
C	7.392828	−8.490452	−1.531556
N	8.068702	−7.899617	−0.529326
N	7.634383	−9.772469	−1.832939
C	6.871494	−10.235308	−2.836844
H	5.667131	−12.469990	−4.875269
H	6.322417	−6.726016	−1.917419
H	7.827104	−6.955054	−0.196124
H	8.641242	−8.500916	0.049341
P	8.415623	−15.437679	−0.185353
O	6.862191	−15.833443	−0.134636
O	9.370621	−16.315284	0.543752
O	8.285070	−13.936896	0.371102
C	9.524400	−13.198086	0.681719
C	9.294892	−12.264356	1.859726
O	8.478014	−11.122949	1.473012
C	7.207869	−11.147070	2.170382
C	8.594556	−12.906193	3.072228
C	7.123926	−12.510213	2.878685
O	9.173304	−12.308899	4.259931
H	9.807671	−12.625347	−0.205255
H	10.314327	−13.912244	0.930620
H	10.279544	−11.883864	2.156883
H	7.178864	−10.310487	2.874021
H	8.728608	−13.992792	3.094635
H	6.628237	−13.248994	2.245840
H	6.581852	−12.444683	3.824805
H	8.707664	−12.678248	5.032602
N	6.134900	−10.896110	1.215434
C	5.640894	−11.754778	0.240442
N	4.737002	−11.188283	−0.539562
C	4.634637	−9.878964	−0.070984
C	3.861332	−8.770317	−0.489945
N	2.978647	−8.809854	−1.505945
N	4.028512	−7.601076	0.189115
C	4.897432	−7.539085	1.215761
N	5.666537	−8.527081	1.700120
C	5.500058	−9.676065	1.016141
H	5.992602	−12.772628	0.160365
H	2.543686	−7.936948	−1.827311
H	2.949304	−9.627472	−2.101252
H	4.981341	−6.564495	1.692199

H	12.014408	−0.172229	1.347581
H	5.477201	2.679090	−0.098587
H	4.907954	−16.092200	−6.434391
H	6.715981	−16.798528	−0.086271

(AAA)$^{H+}$

E: −14043.60 kcal mol^{-1}

O	−0.074506	−0.443907	−0.625245
C	−0.199132	0.008575	0.743547
C	1.157700	−0.025549	1.438357
O	1.614573	−1.397347	1.665053
C	2.759267	−1.701245	0.828588
C	2.297837	0.657401	0.666067
C	2.944461	−0.491960	−0.100456
O	3.178883	1.259373	1.693650
H	−0.972256	−0.568034	−0.979456
H	−0.889387	−0.635774	1.302246
H	−0.576431	1.040914	0.776956
H	1.038182	0.443852	2.419972
H	3.628354	−1.868572	1.472639
H	1.958754	1.468692	0.021574
H	2.386939	−0.625544	−1.028572
H	3.986650	−0.316372	−0.353846
N	2.551815	−2.962115	0.128884
C	1.764468	−3.218651	−0.988340
N	1.796106	−4.488167	−1.360298
C	2.640272	−5.110389	−0.439546
C	3.081782	−6.444921	−0.286216
N	2.689578	−7.449330	−1.104145
N	3.922932	−6.726629	0.742836
C	4.296443	−5.731878	1.570244
N	3.938891	−4.436218	1.525854
C	3.112260	−4.180453	0.499058
H	1.178690	−2.438302	−1.453533
H	3.150149	−8.348757	−1.035158
H	2.170825	−7.240212	−1.947111
H	4.973677	−6.015881	2.373814
P	4.691296	1.734543	1.524236
O	4.810253	2.072421	−0.037951
O	5.034425	2.807099	2.497012
O	5.613965	0.417039	1.649254
C	5.882332	−0.119602	2.992133
C	7.035268	−1.105516	2.930264
O	6.645634	−2.303648	2.200694
C	7.538063	−2.521179	1.078605
C	8.313203	−0.576950	2.246242
C	8.233090	−1.174508	0.845422
O	9.436219	−1.089866	3.054697
H	4.981699	−0.624400	3.353949
H	6.137508	0.704611	3.664283
H	7.266571	−1.388057	3.963540
H	8.249422	−3.317186	1.324945
H	8.385682	0.511102	2.247119
H	7.603387	−0.515123	0.244499
H	9.194229	−1.266610	0.346632
N	6.777328	−3.010936	−0.055751
C	5.959413	−2.290031	−0.915965
N	5.383272	−3.043005	−1.836608
C	5.843000	−4.335109	−1.577738
C	5.618511	−5.579588	−2.210746
N	4.799327	−5.721012	−3.276017
N	6.276213	−6.666985	−1.733780
C	7.089160	−6.521563	−0.670885
N	7.356728	−5.401497	0.025036
C	6.711817	−4.336353	−0.475278
H	5.839972	−1.221832	−0.817677
H	4.605219	−6.648492	−3.631926
H	4.196938	−4.958918	−3.557683
H	7.591032	−7.426411	−0.333194
P	10.977451	−1.215894	2.659402
O	11.148011	−0.191210	1.438858
O	11.857479	−1.034527	3.845612
O	11.146565	−2.637468	1.923654
C	11.198616	−3.858773	2.748086
C	11.827701	−4.988678	1.951828
O	10.944990	−5.400241	0.868687
C	11.589070	−5.204599	−0.413881
C	13.191260	−4.665448	1.309176
C	12.830999	−4.338061	−0.145541
O	14.004421	−5.861240	1.419604
H	10.176022	−4.123402	3.033088
H	11.792796	−3.660100	3.644672
H	11.939586	−5.835962	2.639440
H	11.845946	−6.180280	−0.836566
H	13.688223	−3.826813	1.808172
H	12.589186	−3.276449	−0.228995
H	13.643845	−4.563548	−0.839505
H	14.858289	−5.678678	0.986343
N	10.633953	−4.607738	−1.339489
C	10.179936	−3.295764	−1.353963
N	9.259917	−3.072049	−2.276206
C	9.088383	−4.303137	−2.908773
C	8.257833	−4.726448	−3.970169
N	7.402030	−3.891499	−4.613499
N	8.343144	−6.019672	−4.374079
C	9.191314	−6.846357	−3.733592
N	10.013110	−6.563260	−2.705694
C	9.928297	−5.273148	−2.335447
H	10.567764	−2.564054	−0.661233
H	6.693534	−4.311884	−5.204936
H	7.162038	−3.012161	−4.170406
H	9.210889	−7.874058	−4.092638
H	12.076569	0.055415	1.259517
H	5.611973	2.579617	−0.272811

(AAC)$^{H+}$

E: −13644.17 kcal mol^{-1}

O	−0.201811	−0.421054	−0.390513
C	−0.195725	0.055463	0.975552
C	1.215783	0.003763	1.549783
O	1.669460	−1.375697	1.734360
C	2.741147	−1.688643	0.809717
C	2.297186	0.673011	0.684675
C	2.854353	−0.484323	−0.135930
O	3.267729	1.265913	1.635690
H	−1.129654	−0.536653	−0.659959
H	−0.845407	−0.566415	1.603850
H	−0.549537	1.095486	1.025321
H	1.187235	0.473478	2.538123
H	3.658192	−1.856000	1.383617
H	1.918042	1.491888	0.072932
H	2.201616	−0.610511	−1.000829
H	3.865391	−0.324800	−0.500823
N	2.480217	−2.951246	0.133488
C	1.655597	−3.206950	−0.956609
N	1.699086	−4.469565	−1.350097
C	2.592949	−5.087408	−0.473876
C	3.082476	−6.410072	−0.370247
N	2.698507	−7.404313	−1.203189
N	3.968235	−6.688732	0.621868
C	4.344930	−5.700643	1.455710
N	3.953451	−4.413826	1.449861
C	3.079957	−4.162567	0.461428
H	1.037353	−2.432961	−1.388925
H	3.184363	−8.292278	−1.170501
H	2.132396	−7.194754	−2.014738
H	5.058738	−5.981667	2.228058

P	4.802561	1.626042	1.409175
O	4.929870	1.859476	−0.171325
O	5.242510	2.725745	2.309081
O	5.631230	0.252215	1.598725
C	5.846749	−0.247096	2.967163
C	7.050715	−1.174343	3.003500
O	6.771474	−2.436090	2.334938
C	7.611677	−2.605302	1.169255
C	8.323605	−0.610385	2.345239
C	8.330405	−1.259836	0.963093
O	9.443080	−1.022467	3.211705
H	4.950612	−0.788401	3.283560
H	6.020750	0.601330	3.635047
H	7.246969	−1.391598	4.059019
H	8.309111	−3.431024	1.343526
H	8.335974	0.479224	2.299574
H	7.762872	−0.615038	0.290121
H	9.327689	−1.377979	0.547344
N	6.798530	−3.011538	0.030421
C	5.945638	−2.216001	−0.725537
N	5.335549	−2.880571	−1.691027
C	5.804220	−4.188621	−1.572470
C	5.555961	−5.364826	−2.316028
N	4.717464	−5.395130	−3.378426
N	6.210188	−6.498259	−1.958690
C	7.050570	−6.458857	−0.907195
N	7.355583	−5.408785	−0.123939
C	6.710778	−4.294361	−0.504867
H	5.820780	−1.166486	−0.506995
H	4.473849	−6.291670	−3.782086
H	4.099970	−4.612110	−3.551534
H	7.546137	−7.396838	−0.663878
P	10.998820	−1.035400	2.844267
O	11.123170	0.052129	1.674595
O	11.838028	−0.850520	4.059107
O	11.268632	−2.410142	2.051723
C	11.260575	−3.664787	2.824478
C	11.716911	−4.803022	1.934016
O	10.730372	−5.017603	0.887583
C	11.400501	−5.128739	−0.394406
C	13.086370	−4.602586	1.238998
C	12.717279	−4.355274	−0.230091
O	13.835656	−5.831159	1.425995
H	10.241611	−3.847528	3.179015
H	11.935553	−3.563786	3.679251
H	11.772649	−5.700547	2.564409
H	11.569553	−6.177927	−0.641626
H	13.639923	−3.761536	1.668602
H	12.563579	−3.284627	−0.392080
H	13.490294	−4.703659	−0.919485
H	14.708952	−5.710084	1.009991
N	10.495314	−4.615289	−1.425425
C	9.960499	−5.501014	−2.415453
O	10.326125	−6.692831	−2.433471
N	9.067312	−4.992372	−3.308590
C	8.694145	−3.707356	−3.255207
N	7.802838	−3.272753	−4.171601
C	9.214291	−2.800668	−2.273945
C	10.103894	−3.306595	−1.382777
H	7.362823	−3.942809	−4.791277
H	7.396930	−2.349262	−4.100595
H	8.905768	−1.763093	−2.235880
H	10.536133	−2.714284	−0.586220
H	12.040219	0.341778	1.500246
H	5.707035	2.393525	−0.428062

(AAG)$^{H+}$

E: −14207.66 kcal mol^{-1}

O	−0.176242	−0.491601	−0.241650
C	−0.145101	−0.069884	1.141837
C	1.284605	−0.094320	1.669809
O	1.788801	−1.461470	1.807107
C	2.823600	−1.729348	0.828796
C	2.314557	0.632865	0.789199
C	2.900493	−0.485626	−0.069464
O	3.278925	1.255148	1.722191
H	−1.107932	−0.616579	−0.493207
H	−0.754886	−0.737313	1.763466
H	−0.530540	0.955103	1.243455
H	1.273864	0.348606	2.670824
H	3.760988	−1.921934	1.360323
H	1.881387	1.439638	0.197216
H	2.255699	−0.596715	−0.942048
H	3.909698	−0.290170	−0.423774
N	2.535593	−2.962816	0.105922
C	1.667710	−3.169591	−0.961099
N	1.652219	−4.425581	−1.377309
C	2.548010	−5.091298	−0.539660
C	2.971749	−6.438009	−0.459147
N	2.513388	−7.405258	−1.286413
N	3.866777	−6.770383	0.507575
C	4.312558	−5.810725	1.340498
N	3.982752	−4.507145	1.359003
C	3.098408	−4.201863	0.395447
H	1.061769	−2.367546	−1.357558
H	2.936495	−8.324707	−1.258314
H	1.934266	−7.163627	−2.079631
H	5.031495	−6.134606	2.090962
P	4.769415	1.736427	1.398377
O	4.853837	1.981361	−0.183377
O	5.164658	2.864591	2.284677
O	5.685669	0.416792	1.518698
C	5.886426	−0.144754	2.864086
C	7.040626	−1.130473	2.839581
O	6.668568	−2.336227	2.114839
C	7.580109	−2.568542	1.011455
C	8.331608	−0.601896	2.180111
C	8.305975	−1.234688	0.790568
O	9.437004	−1.072756	3.032155
H	4.968392	−0.651094	3.175871
H	6.113454	0.667243	3.561099
H	7.248355	−1.403345	3.880351
H	8.270074	−3.380263	1.267125
H	8.381998	0.487002	2.149836
H	7.722300	−0.581614	0.138367
H	9.289501	−1.360236	0.345057
N	6.827318	−3.037526	−0.138564
C	5.980624	−2.301414	−0.959037
N	5.387655	−3.036022	−1.883397
C	5.862250	−4.330047	−1.668187
C	5.616271	−5.563238	−2.315299
N	4.777122	−5.685660	−3.366372
N	6.267445	−6.664940	−1.860856
C	7.093173	−6.543286	−0.804723
N	7.388110	−5.434608	−0.102249
C	6.751969	−4.354330	−0.582491
H	5.850106	−1.237958	−0.824282
H	4.557799	−6.606507	−3.724452
H	4.211185	−4.901404	−3.661400
H	7.584311	−7.459493	−0.482462
P	11.001754	−1.042688	2.683518
O	11.166200	−0.067826	1.422137
O	11.814064	−0.735810	3.892474
O	11.307290	−2.461085	1.994798

C	11.236613	−3.661535	2.846818
C	11.773738	−4.855585	2.080397
O	10.867762	−5.194312	0.991690
C	11.575729	−5.168465	−0.273302
C	13.171081	−4.663114	1.454536
C	12.862836	−4.369293	−0.018915
O	13.887597	−5.912052	1.630143
H	10.192256	−3.830679	3.125203
H	11.836688	−3.496360	3.746748
H	11.804041	−5.698474	2.782281
H	11.784688	−6.194130	−0.592018
H	13.721520	−3.845460	1.931004
H	12.685198	−3.298707	−0.144359
H	13.673131	−4.670601	−0.686620
H	14.768381	−5.806170	1.225999
N	10.701394	−4.602155	−1.288657
C	10.325953	−3.268106	−1.437216
N	9.461256	−3.087502	−2.415978
C	9.242927	−4.360120	−2.943782
C	8.423278	−4.787178	−4.032737
O	7.694238	−4.113278	−4.781999
N	8.517562	−6.194263	−4.214516
C	9.280581	−7.059409	−3.460014
N	9.187665	−8.384274	−3.744249
N	10.054010	−6.648168	−2.458218
C	9.999487	−5.315062	−2.248583
H	10.733152	−2.496490	−0.801251
H	7.937255	−6.571273	−4.960704
H	8.812795	−8.685619	−4.635498
H	9.876182	−8.991973	−3.315486
H	11.802729	0.654987	1.586027
H	5.081366	2.904587	−0.407901

(AAT)$^{H+}$

E: −13904.21 kcal mol^{-1}

O	0.012674	−0.229318	−0.671219
C	−0.023928	0.304500	0.673380
C	1.336819	0.147776	1.344235
O	1.646018	−1.261075	1.600087
C	2.756892	−1.692958	0.774801
C	2.529971	0.681066	0.532972
C	3.013802	−0.552089	−0.218659
O	3.502058	1.187570	1.531818
H	−0.901663	−0.258677	−1.003127
H	−0.768776	−0.226931	1.278878
H	−0.280505	1.373653	0.659427
H	1.288580	0.654315	2.313307
H	3.620608	−1.885318	1.419170
H	2.275159	1.517933	−0.117810
H	2.382627	−0.664616	−1.101563
H	4.047257	−0.498868	−0.549980
N	2.458256	−2.970648	0.148498
C	1.636213	−3.235412	−0.940656
N	1.648700	−4.510271	−1.296239
C	2.521400	−5.124751	−0.395962
C	2.981796	−6.454254	−0.253233
N	2.579608	−7.462865	−1.059759
N	3.859844	−6.723781	0.748494
C	4.255662	−5.720995	1.556008
N	3.891683	−4.427189	1.512827
C	3.026232	−4.185388	0.515118
H	1.044395	−2.455424	−1.399895
H	3.045025	−8.360257	−0.999522
H	2.019559	−7.264574	−1.878313
H	4.962545	−5.994988	2.337212
P	5.064845	1.450392	1.359069
O	5.290571	1.601279	−0.220884
O	5.533530	2.560346	2.231676
O	5.796903	0.038403	1.640064
C	5.893204	−0.440993	3.028552
C	7.063605	−1.402103	3.174858
O	6.799591	−2.678575	2.531347
C	7.684724	−2.895351	1.409141
C	8.395099	−0.894435	2.594528
C	8.502864	−1.600086	1.240718
O	9.437552	−1.302510	3.552542
H	4.959290	−0.948849	3.284693
H	6.042816	0.414721	3.693164
H	7.177661	−1.590345	4.247920
H	8.317948	−3.763290	1.609693
H	8.432741	0.191838	2.505459
H	8.062106	−0.955004	0.478957
H	9.531619	−1.814806	0.956634
N	6.901062	−3.244423	0.226901
C	6.060425	−2.404805	−0.494106
N	5.413515	−3.024084	−1.464355
C	5.838707	−4.349319	−1.383987
C	5.521753	−5.502163	−2.138971
N	4.653858	−5.484127	−3.173409
N	6.134762	−6.669420	−1.811014
C	6.988581	−6.685068	−0.769928
N	7.355617	−5.661173	0.022886
C	6.756160	−4.511466	−0.331455
H	5.972176	−1.359899	−0.239679
H	4.376755	−6.357519	−3.604131
H	4.098845	−4.658225	−3.355015
H	7.442663	−7.648485	−0.546896
P	11.010765	−1.037478	3.345656
O	11.158192	0.212251	2.351663
O	11.688494	−0.957344	4.667624
O	11.529202	−2.184948	2.353056
C	11.523295	−3.574475	2.837045
C	11.804747	−4.503469	1.678167
O	10.685037	−4.477931	0.757419
C	11.215320	−4.854618	−0.535145
C	13.084279	−4.200267	0.844370
C	12.575250	−4.143974	−0.612085
O	14.012705	−5.283002	1.091606
H	10.546124	−3.799812	3.274930
H	12.299869	−3.685161	3.599163
H	11.911014	−5.516944	2.093156
H	11.321671	−5.940089	−0.601004
H	13.540150	−3.251726	1.140131
H	12.445195	−3.103803	−0.924166
H	13.262209	−4.633688	−1.306134
H	14.872877	−5.028063	0.711045
N	10.236869	−4.478226	−1.555093
C	9.679084	−5.482085	−2.354782
O	9.985253	−6.674506	−2.278349
N	8.740557	−5.015580	−3.257282
C	8.328743	−3.691557	−3.474704
O	7.504157	−3.441277	−4.365451
C	8.946252	−2.713045	−2.591198
C	8.569071	−1.264754	−2.732492
C	9.861984	−3.153825	−1.693432
H	8.328211	−5.725114	−3.860201
H	8.854807	−0.884350	−3.720869
H	9.066650	−0.656378	−1.972146
H	7.485353	−1.135855	−2.639488
H	10.360893	−2.474080	−1.014314
H	11.344303	1.049686	2.819350
H	6.011785	2.218791	−0.451179

(ACA)$^{H+}$

E: −13644.08 kcal mol^{-1}

O	0.325533	−0.251197	−0.138607
C	0.205714	−0.054830	1.290346
C	1.557462	−0.259102	1.964574
O	1.944897	−1.668556	2.001652
C	3.071190	−1.923467	1.129014
C	2.724792	0.472631	1.284200
C	3.378689	−0.598055	0.408350
O	3.605101	0.947676	2.372729
H	−0.570581	−0.228538	−0.517694
H	−0.511719	−0.764465	1.721118
H	−0.134446	0.967302	1.509818
H	1.462653	0.075390	3.002468
H	3.915017	−2.271926	1.730564
H	2.402416	1.344339	0.714743
H	2.908356	−0.572087	−0.576013
H	4.451516	−0.452344	0.288822
N	2.756557	−3.027794	0.226519
C	1.850387	−3.034893	−0.829952
N	1.731996	−4.222891	−1.399010
C	2.594844	−5.051486	−0.680808
C	2.927826	−6.420131	−0.794918
N	2.433396	−7.218416	−1.771050
N	3.811030	−6.941166	0.095625
C	4.343611	−6.133085	1.031973
N	4.115190	−4.822127	1.226917
C	3.232118	−4.329938	0.341475
H	1.297032	−2.143884	−1.093717
H	2.602339	−8.216057	−1.723721
H	1.669973	−6.891035	−2.349039
H	5.048013	−6.603424	1.715705
P	4.938276	1.809781	2.128181
O	4.762271	2.586682	0.735687
O	5.252293	2.613996	3.340271
O	6.069185	0.773600	1.659417
C	6.623788	−0.155005	2.655573
C	7.515481	−1.151188	1.945007
O	6.704277	−2.096254	1.193517
C	7.418296	−2.356007	−0.053963
C	8.507124	−0.519789	0.921695
C	7.969462	−0.977954	−0.430395
O	9.840034	−1.129961	1.079681
H	5.807727	−0.676234	3.164984
H	7.209062	0.416538	3.381041
H	8.084290	−1.688322	2.713483
H	8.231224	−3.059917	0.139524
H	8.591676	0.562523	1.018000
H	7.169740	−0.297129	−0.732430
H	8.732822	−1.011650	−1.206833
N	6.538381	−3.024754	−0.991376
C	6.586919	−4.470111	−1.039987
O	7.336485	−5.070365	−0.255159
N	5.812168	−5.096238	−1.963884
C	4.999274	−4.402253	−2.766491
N	4.290995	−5.090178	−3.689036
C	4.878859	−2.974852	−2.691947
C	5.670300	−2.334650	−1.793918
H	4.273677	−6.102429	−3.636058
H	3.559937	−4.632220	−4.217546
H	4.196638	−2.425092	−3.328707
H	5.652988	−1.258552	−1.680582
P	10.870190	−0.578797	2.174084
O	11.676690	0.536697	1.346315
O	10.256761	−0.128994	3.455786
O	11.929274	−1.770462	2.261351
C	11.643856	−2.957503	3.088069
C	12.035035	−4.215821	2.334436
O	11.059049	−4.479478	1.284154
C	11.761486	−4.782143	0.051076
C	13.427369	−4.169954	1.655010
C	13.092168	−4.029723	0.163085
O	14.088145	−5.424690	1.957874
H	10.581954	−2.989109	3.346331
H	12.236969	−2.864659	4.001608
H	12.017716	−5.045388	3.053699
H	11.912296	−5.863716	−0.035722
H	14.031584	−3.334168	2.019925
H	12.954361	−2.972708	−0.081517
H	13.867037	−4.446871	−0.484248
H	14.978741	−5.391460	1.562811
N	10.920357	−4.401760	−1.068790
C	10.606559	−3.118693	−1.499147
N	9.759620	−3.105410	−2.514111
C	9.491207	−4.450043	−2.771400
C	8.642182	−5.096016	−3.697304
N	7.899327	−4.422251	−4.609748
N	8.587307	−6.452315	−3.677144
C	9.327351	−7.119526	−2.771471
N	10.153302	−6.614468	−1.835659
C	10.197117	−5.271978	−1.879084
H	11.011589	−2.244018	−1.011434
H	7.172771	−4.923261	−5.107715
H	7.806915	−3.417395	−4.529424
H	9.243127	−8.204958	−2.800277
H	12.162123	1.162691	1.919119
H	4.402255	3.488435	0.844237

(ACC)$^{H+}$

E: −13243.15 kcal mol^{-1}

O	−0.215018	−0.356767	−0.240207
C	−0.223283	0.045446	1.149832
C	1.185860	−0.020330	1.727770
O	1.647011	−1.401933	1.876962
C	2.711432	−1.694776	0.940868
C	2.262571	0.680622	0.883501
C	2.851545	−0.450852	0.045878
O	3.219972	1.266986	1.845730
H	−1.139966	−0.442566	−0.529957
H	−0.871300	−0.614850	1.739404
H	−0.587189	1.078039	1.250892
H	1.153084	0.419602	2.729483
H	3.625616	−1.911446	1.503092
H	1.870092	1.498859	0.279185
H	2.241066	−0.549868	−0.852588
H	3.878726	−0.274224	−0.263451
N	2.417345	−2.917205	0.199717
C	1.535065	−3.088619	−0.863099
N	1.543781	−4.320090	−1.345980
C	2.472652	−5.005000	−0.562619
C	2.953680	−6.334536	−0.588434
N	2.562800	−7.236814	−1.516930
N	3.870951	−6.701753	0.344041
C	4.297009	−5.783698	1.233156
N	3.931486	−4.493685	1.337340
C	3.017404	−4.155642	0.411936
H	0.901936	−2.279841	−1.200334
H	2.855042	−8.202410	−1.426523
H	1.797118	−7.021894	−2.142468
H	5.034331	−6.132148	1.954226
P	4.680560	1.830814	1.515134
O	4.706986	2.098581	−0.064376
O	5.035923	2.966663	2.408372
O	5.665214	0.558985	1.612493

C	5.892544	−0.021480	2.945162
C	6.982565	−1.071845	2.854621
O	6.508274	−2.209119	2.084577
C	7.459301	−2.534477	1.036718
C	8.294724	−0.597172	2.189047
C	8.254774	−1.241903	0.806154
O	9.385001	−1.101759	3.044646
H	4.962385	−0.478196	3.295831
H	6.197006	0.771233	3.634899
H	7.199709	−1.399349	3.878164
H	8.100325	−3.361141	1.350207
H	8.386571	0.488556	2.151579
H	7.722213	−0.564276	0.134289
H	9.242658	−1.420973	0.389610
N	6.720997	−3.030951	−0.123248
C	6.814631	−4.423081	−0.478207
O	7.566844	−5.164709	0.172737
N	6.070253	−4.857249	−1.532863
C	5.264012	−4.025621	−2.199528
N	4.562879	−4.526231	−3.238727
C	5.136259	−2.638931	−1.851667
C	5.878238	−2.197721	−0.804681
H	4.583295	−5.523731	−3.414930
H	3.854374	−3.972467	−3.701236
H	4.471654	−1.974218	−2.390189
H	5.833099	−1.175880	−0.449188
P	10.951421	−1.105488	2.723799
O	11.097728	−0.055703	1.522042
O	11.760107	−0.873329	3.951610
O	11.257703	−2.502566	1.986204
C	11.292059	−3.728700	2.803960
C	11.853243	−4.871778	1.979292
O	10.921735	−5.212306	0.915525
C	11.598366	−5.188515	−0.367597
C	13.220925	−4.604145	1.311372
C	12.861164	−4.339318	−0.156558
O	14.012715	−5.808827	1.478168
H	10.270957	−3.960413	3.121668
H	11.922572	−3.555590	3.680889
H	11.948256	−5.732860	2.653635
H	11.837119	−6.206696	−0.677042
H	13.736777	−3.753343	1.768323
H	12.651121	−3.275400	−0.292058
H	13.664278	−4.623017	−0.841075
H	14.873543	−5.658177	1.046258
N	10.660721	−4.677218	−1.373476
C	10.112275	−5.563757	−2.354301
O	10.469620	−6.759797	−2.368732
N	9.217381	−5.056039	−3.246729
C	8.833648	−3.773951	−3.182087
N	7.941904	−3.334873	−4.093899
C	9.335168	−2.877158	−2.183301
C	10.235202	−3.380306	−1.301288
H	7.535333	−3.985979	−4.754409
H	7.577035	−2.393046	−4.055553
H	9.005540	−1.847155	−2.127710
H	10.650374	−2.794523	−0.490919
H	12.012785	0.258692	1.384330
H	5.054569	2.982905	−0.291980

(ACG)$^{H+}$

E: −13809.92 kcal mol^{-1}

O	0.425868	−0.380231	−0.834321
C	0.146486	0.030341	0.525267
C	1.404114	−0.082033	1.379562
O	1.748859	−1.474165	1.664678
C	2.959710	−1.864683	0.974479
C	2.663873	0.536065	0.751413
C	3.393598	−0.654238	0.126234
O	3.411801	1.164891	1.861699
H	−0.422916	−0.440892	−1.306589
H	−0.633470	−0.601378	0.968337
H	−0.191696	1.076286	0.548953
H	1.193538	0.399730	2.339818
H	3.714391	−2.143239	1.713931
H	2.435329	1.318158	0.027155
H	3.057039	−0.759379	−0.906109
H	4.476272	−0.538416	0.126169
N	2.722385	−3.075438	0.191211
C	2.007302	−3.201018	−0.996227
N	1.941489	−4.449038	−1.428805
C	2.640462	−5.194075	−0.478955
C	2.943666	−6.570385	−0.374106
N	2.604085	−7.471199	−1.327616
N	3.638936	−6.992824	0.712901
C	4.031800	−6.084214	1.625974
N	3.825263	−4.755245	1.619533
C	3.123071	−4.360996	0.542805
H	1.537917	−2.343152	−1.457273
H	2.724092	−8.458356	−1.133157
H	1.950136	−7.207709	−2.053927
H	4.589397	−6.477722	2.473850
P	4.840015	1.881156	1.698234
O	4.990518	2.317863	0.162559
O	5.005739	2.932856	2.738197
O	5.934158	0.706613	1.707930
C	6.105551	−0.064476	2.945649
C	6.978208	−1.264849	2.656782
O	6.284546	−2.168466	1.772871
C	7.300007	−2.984639	1.136275
C	8.347400	−0.964982	1.983376
C	8.463915	−2.014793	0.866062
O	9.353201	−1.078043	3.052075
H	5.126906	−0.390072	3.310697
H	6.583206	0.570672	3.697212
H	7.180802	−1.762914	3.616712
H	7.599636	−3.807131	1.789717
H	8.403194	0.051876	1.595777
H	8.339011	−1.529667	−0.104497
H	9.420011	−2.536748	0.866702
N	6.710981	−3.601114	−0.046513
C	6.688928	−5.034076	−0.163849
O	7.261964	−5.722059	0.696137
N	6.031881	−5.566344	−1.230506
C	5.421404	−4.784838	−2.126751
N	4.777003	−5.391187	−3.150164
C	5.458489	−3.353811	−2.050914
C	6.113985	−2.813093	−0.993332
H	4.619858	−6.391486	−3.095029
H	4.170704	−4.854008	−3.756485
H	4.988903	−2.728930	−2.799997
H	6.185782	−1.744120	−0.836724
P	10.931583	−0.902300	2.857528
O	11.050232	0.155373	1.660559
O	11.590352	−0.571364	4.150176
O	11.463748	−2.245288	2.154123
C	11.364334	−3.520223	2.878147
C	11.739475	−4.643995	1.930261
O	10.756404	−4.711638	0.856177
C	11.440706	−4.719906	−0.423993
C	13.128000	−4.510689	1.262435
C	12.797741	−4.056251	−0.165817
O	13.741193	−5.824274	1.297651
H	10.335088	−3.652529	3.225414
H	12.042473	−3.501756	3.736627

H	11.707495	−5.579358	2.503563
H	11.550210	−5.754201	−0.766017
H	13.762943	−3.788764	1.785624
H	12.706540	−2.967725	−0.179685
H	13.556046	−4.358498	−0.891651
H	14.624672	−5.748803	0.892453
N	10.604043	−4.052635	−1.408407
C	10.507965	−2.688839	−1.690775
N	9.621956	−2.425130	−2.630756
C	9.096541	−3.665317	−2.993595
C	8.139968	−4.012638	−3.996367
O	7.520931	−3.275430	−4.785341
N	7.930236	−5.416723	−4.027992
C	8.552773	−6.348703	−3.224792
N	8.178165	−7.644745	−3.371322
N	9.461437	−6.016000	−2.311003
C	9.690962	−4.687964	−2.240484
H	11.106137	−1.954681	−1.171702
H	7.219673	−5.728913	−4.686384
H	7.696286	−7.942647	−4.210563
H	8.742985	−8.341830	−2.900639
H	11.931171	0.570881	1.584134
H	4.777999	3.259621	0.012367

(ACT)$^{H+}$

E: −13502.58 kcal mol^{-1}

O	−0.135291	−0.359658	−0.192244
C	−0.152865	0.017182	1.204913
C	1.249088	−0.077936	1.796027
O	1.689107	−1.467973	1.929011
C	2.758835	−1.762312	0.999297
C	2.343561	0.619686	0.972421
C	2.925357	−0.507622	0.124007
O	3.300751	1.177250	1.951845
H	−1.058109	−0.421635	−0.494757
H	−0.816059	−0.644769	1.775437
H	−0.503883	1.052687	1.320729
H	1.212387	0.347330	2.803916
H	3.664675	−1.999858	1.566236
H	1.967992	1.451829	0.376415
H	2.324169	−0.586075	−0.782797
H	3.958561	−0.341211	−0.170161
N	2.453173	−2.969670	0.238578
C	1.556892	−3.116544	−0.816162
N	1.547422	−4.340877	−1.316747
C	2.478574	−5.046172	−0.554555
C	2.949819	−6.378378	−0.609086
N	2.543320	−7.260488	−1.550356
N	3.873051	−6.769175	0.307764
C	4.315744	−5.869861	1.207883
N	3.963635	−4.578324	1.336263
C	3.042762	−4.217047	0.426269
H	0.928713	−2.295748	−1.133567
H	2.831210	−8.229226	−1.483343
H	1.775846	−7.027115	−2.166882
H	5.056755	−6.236837	1.915706
P	4.761672	1.748226	1.629240
O	4.794875	2.046423	0.055103
O	5.115761	2.866468	2.545103
O	5.746128	0.475035	1.696940
C	5.976314	−0.131255	3.017964
C	7.053372	−1.190873	2.894372
O	6.548729	−2.301236	2.110746
C	7.513412	−2.694732	1.103714
C	8.364989	−0.712645	2.224814
C	8.411291	−1.464010	0.891740
O	9.453744	−1.062504	3.152227
H	5.043388	−0.582010	3.368854
H	6.296888	0.645602	3.718455
H	7.283674	−1.539321	3.908878
H	8.081467	−3.567613	1.430922
H	8.392589	0.368503	2.088310
H	8.006103	−0.813042	0.113884
H	9.421396	−1.750154	0.604712
N	6.775720	−3.125545	−0.086507
C	6.812471	−4.504435	−0.492138
O	7.548428	−5.296868	0.118008
N	6.039272	−4.873447	−1.550366
C	5.258098	−3.988845	−2.178773
N	4.513673	−4.429326	−3.212256
C	5.192326	−2.610074	−1.784275
C	5.959254	−2.235978	−0.730059
H	4.507822	−5.416740	−3.437276
H	3.842307	−3.825774	−3.666927
H	4.552077	−1.901008	−2.294584
H	5.955843	−1.227543	−0.334188
P	11.019797	−0.857972	2.847832
O	11.133282	0.178695	1.630396
O	11.754296	−0.532090	4.100698
O	11.488068	−2.191633	2.088826
C	11.432051	−3.457532	2.838518
C	11.790267	−4.596986	1.910081
O	10.743299	−4.733388	0.913186
C	11.362350	−5.067808	−0.351051
C	13.137745	−4.459385	1.148073
C	12.718193	−4.347750	−0.328168
O	13.900864	−5.660537	1.426331
H	10.418954	−3.595097	3.227935
H	12.142770	−3.410675	3.668913
H	11.834813	−5.510319	2.519538
H	11.483183	−6.149473	−0.441145
H	13.700515	−3.580389	1.475623
H	12.607113	−3.294009	−0.598067
H	13.444872	−4.806235	−1.002892
H	14.780976	−5.551207	1.021753
N	10.444359	−4.672660	−1.420496
C	9.790931	−5.673437	−2.149617
O	9.973260	−6.882551	−1.977908
N	8.923264	−5.187333	−3.109358
C	8.602525	−3.852808	−3.391288
O	7.801701	−3.586133	−4.300029
C	9.273117	−2.877731	−2.544254
C	8.982625	−1.417117	−2.749355
C	10.150320	−3.336152	−1.617158
H	8.436048	−5.896047	−3.654336
H	9.251401	−1.107818	−3.766635
H	9.545454	−0.802937	−2.041632
H	7.913115	−1.211713	−2.625135
H	10.683006	−2.658519	−0.962024
H	11.483562	1.047840	1.906346
H	5.054414	2.966153	−0.148257

(AGA)$^{H+}$

E: −14209.93 kcal mol^{-1}

O	−0.063271	−0.568845	−0.238802
C	−0.130279	−0.161107	1.147793
C	1.270263	−0.104568	1.747134
O	1.833428	−1.440417	1.945919
C	2.918941	−1.691627	1.021369
C	2.300627	0.654356	0.895343
C	3.016120	−0.449188	0.116440
O	3.175151	1.374098	1.846342

H	−0.971960	−0.737641	−0.543480
H	−0.730323	−0.869859	1.731868
H	−0.581579	0.837871	1.234504
H	1.188958	0.359803	2.735053
H	3.835036	−1.862683	1.593584
H	1.848171	1.404063	0.245845
H	2.480932	−0.603458	−0.821296
H	4.048587	−0.201482	−0.120008
N	2.688225	−2.935169	0.287976
C	1.836580	−3.158833	−0.788929
N	1.866290	−4.409039	−1.220433
C	2.778884	−5.053439	−0.386136
C	3.256565	−6.382615	−0.333178
N	2.827629	−7.354287	−1.175599
N	4.165500	−6.699388	0.625588
C	4.561565	−5.742079	1.487037
N	4.177276	−4.454317	1.533107
C	3.290605	−4.159310	0.567385
H	1.206544	−2.371107	−1.176711
H	3.335732	−8.230853	−1.197467
H	2.315603	−7.092594	−2.008964
H	5.287483	−6.053570	2.236069
P	4.565648	2.085519	1.491721
O	4.528185	2.368478	−0.084720
O	4.820541	3.244296	2.390266
O	5.674455	0.917930	1.554305
C	5.924124	0.294897	2.860813
C	6.935754	−0.822841	2.708388
O	6.364234	−1.906174	1.918065
C	7.336393	−2.315000	0.922108
C	8.269928	−0.434459	2.021202
C	8.131875	−1.042716	0.628726
O	9.327632	−1.052730	2.850664
H	4.984911	−0.109165	3.250532
H	6.310807	1.050887	3.550969
H	7.154553	−1.192941	3.717080
H	7.978930	−3.105299	1.323708
H	8.459035	0.638953	2.013347
H	7.534393	−0.356967	0.020749
H	9.073944	−1.222776	0.115662
N	6.659229	−2.910984	−0.206482
C	5.805889	−2.323200	−1.138890
N	5.359113	−3.187611	−2.030197
C	5.947485	−4.405698	−1.685207
C	5.862956	−5.696387	−2.294071
O	5.187114	−6.065701	−3.266518
N	6.725207	−6.622581	−1.633321
C	7.531157	−6.349065	−0.547258
N	8.314178	−7.349213	−0.052374
N	7.554643	−5.157275	0.039355
C	6.767622	−4.246268	−0.561286
H	5.568707	−1.270771	−1.106497
H	6.737376	−7.557151	−2.035610
H	8.493762	−8.148297	−0.651106
H	9.132166	−7.009420	0.457665
P	10.805577	−1.498576	2.437303
O	11.217127	−0.600961	1.172383
O	11.712696	−1.475896	3.615847
O	10.662283	−2.927415	1.714284
C	10.536362	−4.158745	2.515449
C	11.392063	−5.270757	1.922155
O	10.805557	−5.818507	0.706171
C	11.561335	−5.411678	−0.464534
C	12.825528	−4.866283	1.539929
C	12.715287	−4.526825	0.043889
O	13.667625	−6.020130	1.782179
H	9.484109	−4.453050	2.505002
H	10.861528	−3.957192	3.539507
H	11.411437	−6.081618	2.658681
H	11.910438	−6.318162	−0.963465
H	13.179339	−4.012341	2.127179
H	12.483474	−3.467793	−0.074894
H	13.643670	−4.734895	−0.492335
H	14.571902	−5.791152	1.499360
N	10.673607	−4.752134	−1.421799
C	10.205915	−3.442916	−1.408309
N	9.316724	−3.196439	−2.354346
C	9.173607	−4.407345	−3.030479
C	8.361246	−4.808136	−4.115424
N	7.505951	−3.965077	−4.746879
N	8.458982	−6.092328	−4.546925
C	9.305278	−6.929222	−3.916202
N	10.106201	−6.667538	−2.866082
C	10.005968	−5.387596	−2.464742
H	10.555194	−2.731481	−0.677136
H	6.804429	−4.377651	−5.352248
H	7.250340	−3.101934	−4.280770
H	9.338792	−7.946770	−4.302232
H	12.028259	−0.079039	1.327415
H	4.788720	3.282203	−0.311736

(AGC)$^{H+}$

E: −13810.37 kcal mol^{-1}

O	−0.091913	−0.581688	−0.228475
C	−0.180232	−0.160111	1.152673
C	1.212169	−0.086502	1.769283
O	1.789880	−1.416276	1.967519
C	2.886282	−1.648177	1.050318
C	2.243239	0.687935	0.932556
C	2.969500	−0.402869	0.148860
O	3.107708	1.404913	1.897494
H	−0.993437	−0.777826	−0.537919
H	−0.781123	−0.868396	1.736268
H	−0.641436	0.835610	1.223874
H	1.112869	0.372493	2.758079
H	3.799968	−1.803801	1.631169
H	1.791427	1.443545	0.289449
H	2.432771	−0.558510	−0.787557
H	3.997049	−0.140264	−0.089800
N	2.685378	−2.893820	0.311828
C	1.880246	−3.125135	−0.799162
N	1.953198	−4.370085	−1.239833
C	2.846292	−5.003522	−0.376601
C	3.362163	−6.319048	−0.325386
N	3.010455	−7.283586	−1.208800
N	4.234872	−6.627223	0.669194
C	4.567001	−5.673223	1.560690
N	4.151504	−4.395241	1.604306
C	3.299912	−4.108994	0.604899
H	1.249884	−2.347697	−1.206119
H	3.525346	−8.156109	−1.207865
H	2.508592	−7.031689	−2.050791
H	5.264341	−5.978091	2.338941
P	4.525399	2.078371	1.600927
O	4.474411	2.409579	0.035032
O	4.796840	3.198620	2.542330
O	5.616514	0.891607	1.655582
C	5.904860	0.300597	2.969053
C	6.918145	−0.815647	2.814307
O	6.343260	−1.895570	2.021877
C	7.293800	−2.287708	1.003541
C	8.251881	−0.428699	2.128032
C	8.109124	−1.021631	0.729929
O	9.306799	−1.071041	2.938763
H	4.977791	−0.102727	3.387914

H	6.301138	1.073275	3.634560
H	7.137071	−1.188489	3.821853
H	7.925738	−3.103617	1.370897
H	8.446871	0.643826	2.130747
H	7.528093	−0.321184	0.123229
H	9.055581	−1.210898	0.226717
N	6.595226	−2.832266	−0.140607
C	5.784492	−2.184857	−1.074030
N	5.354565	−2.998108	−2.019249
C	5.908916	−4.242192	−1.712667
C	5.865592	−5.488974	−2.411473
O	5.259162	−5.784585	−3.454291
N	6.676787	−6.463434	−1.759674
C	7.446312	−6.254467	−0.633309
N	8.223208	−7.275918	−0.177977
N	7.447776	−5.099965	0.023209
C	6.688817	−4.152439	−0.552856
H	5.565090	−1.130933	−0.999770
H	6.727074	−7.363593	−2.232391
H	8.430785	−8.038541	−0.813750
H	9.010610	−6.964047	0.393446
P	10.820181	−1.389300	2.522216
O	11.168561	−0.431096	1.285467
O	11.715948	−1.333213	3.708223
O	10.784128	−2.807128	1.762324
C	10.645445	−4.043753	2.556144
C	11.354371	−5.197723	1.862569
O	10.617232	−5.631396	0.685296
C	11.403318	−5.431218	−0.521463
C	12.789496	−4.902876	1.385103
C	12.612202	−4.577517	−0.105820
O	13.557776	−6.114567	1.595563
H	9.579384	−4.264633	2.655093
H	11.087490	−3.884887	3.543554
H	11.366575	−6.031516	2.574453
H	11.698282	−6.403452	−0.914377
H	13.240540	−4.071279	1.936656
H	12.410215	−3.510925	−0.225773
H	13.500351	−4.826281	−0.691411
H	14.456312	−5.958468	1.251226
N	10.539178	−4.838903	−1.552786
C	10.094227	−5.647827	−2.649813
O	10.497160	−6.823772	−2.748537
N	9.228619	−5.092404	−3.542462
C	8.802921	−3.830167	−3.398092
N	7.951792	−3.343672	−4.326338
C	9.220608	−3.006383	−2.302841
C	10.070853	−3.561691	−1.402659
H	7.568419	−3.974226	−5.020939
H	7.482007	−2.459971	−4.178739
H	8.858027	−1.992627	−2.185176
H	10.402256	−3.038162	−0.515211
H	11.909701	0.176353	1.475779
H	5.174554	3.022394	−0.264009

(AGG)$^{H+}$

E: −14374.20 kcal mol^{-1}

O	−0.065684	−0.589908	−0.253077
C	−0.130072	−0.184307	1.134315
C	1.271417	−0.130800	1.731619
O	1.832173	−1.467557	1.929733
C	2.916752	−1.720932	1.004696
C	2.302492	0.626822	0.879275
C	3.018990	−0.477443	0.101680
O	3.175687	1.347900	1.830176
H	−0.975122	−0.756275	−0.556781
H	−0.730245	−0.893159	1.718155
H	−0.579747	0.815224	1.223329
H	1.192185	0.333614	2.719696
H	3.832145	−1.896100	1.576708
H	1.850203	1.375439	0.228395
H	2.486824	−0.629595	−0.838067
H	4.053172	−0.231892	−0.130808
N	2.681301	−2.962377	0.268999
C	1.830405	−3.180264	−0.809723
N	1.854232	−4.430079	−1.242646
C	2.761942	−5.080174	−0.407424
C	3.231602	−6.412140	−0.355025
N	2.798087	−7.380063	−1.199384
N	4.136248	−6.735520	0.605714
C	4.536012	−5.781343	1.468997
N	4.159682	−4.491213	1.515273
C	3.276617	−4.190006	0.548084
H	1.204898	−2.388973	−1.197540
H	3.300878	−8.259616	−1.222881
H	2.285014	−7.114874	−2.030975
H	5.258234	−6.097916	2.219460
P	4.560747	2.068495	1.471518
O	4.524426	2.349507	−0.105411
O	4.807180	3.230041	2.368603
O	5.676600	0.908351	1.532159
C	5.928677	0.285065	2.838057
C	6.935985	−0.835168	2.682048
O	6.357148	−1.917741	1.896796
C	7.335850	−2.346283	0.915898
C	8.268377	−0.451817	1.987003
C	8.132983	−1.079894	0.603077
O	9.324129	−1.067890	2.818505
H	4.989771	−0.115322	3.231831
H	6.321027	1.040164	3.525895
H	7.160294	−1.203670	3.690236
H	7.977135	−3.127740	1.338382
H	8.455253	0.621865	1.965992
H	7.538728	−0.404724	−0.020023
H	9.076736	−1.273371	0.098215
N	6.666452	−2.967221	−0.203678
C	5.807609	−2.408915	−1.150828
N	5.378015	−3.295614	−2.027613
C	5.983369	−4.498037	−1.660051
C	5.930213	−5.799237	−2.249383
O	5.286778	−6.195442	−3.234158
N	6.789465	−6.704356	−1.554425
C	7.565658	−6.403698	−0.453398
N	8.341656	−7.387714	0.084205
N	7.570314	−5.199417	0.104577
C	6.789655	−4.307609	−0.530473
H	5.553232	−1.359922	−1.137765
H	6.809400	−7.649755	−1.931494
H	8.551400	−8.192606	−0.496571
H	9.141462	−7.025475	0.607630
P	10.852146	−1.362568	2.446620
O	11.192121	−0.465507	1.161752
O	11.728167	−1.210759	3.639707
O	10.872508	−2.821455	1.770502
C	10.710217	−4.014911	2.618739
C	11.440066	−5.203801	2.008720
O	10.761485	−5.706629	0.822708
C	11.527323	−5.416328	−0.376070
C	12.892123	−4.939597	1.574759
C	12.766397	−4.621637	0.076621
O	13.631304	−6.162824	1.816585
H	9.641857	−4.233417	2.696842
H	11.122707	−3.808060	3.610540
H	11.412743	−6.003073	2.758051
H	11.784381	−6.368361	−0.845656

H	13.341737	−4.111115	2.132399
H	12.618057	−3.549241	−0.057118
H	13.656942	−4.915946	−0.482989
H	14.540864	−6.027508	1.493227
N	10.686815	−4.712834	−1.342492
C	10.342140	−3.361208	−1.365698
N	9.490910	−3.067680	−2.328805
C	9.243334	−4.277903	−2.974492
C	8.415455	−4.578107	−4.099939
O	7.720064	−3.815703	−4.792649
N	8.451168	−5.970113	−4.394176
C	9.186344	−6.921257	−3.719615
N	9.020120	−8.219425	−4.092944
N	9.984628	−6.623744	−2.698710
C	9.972341	−5.312491	−2.369990
H	10.749140	−2.665352	−0.649479
H	7.848925	−6.263373	−5.160669
H	8.636728	−8.432617	−5.006526
H	9.694524	−8.882689	−3.727947
H	11.939920	0.144640	1.313125
H	4.731740	3.278108	−0.327185

(AGT)$^{H+}$

E: −14067.79 kcal mol^{-1}

O	−0.256564	−0.403054	−0.176125
C	−0.208699	0.051954	1.196485
C	1.219064	−0.010371	1.728191
O	1.676447	−1.392796	1.879334
C	2.722819	−1.691077	0.920660
C	2.275790	0.669350	0.841534
C	2.801404	−0.476146	−0.014928
O	3.280366	1.235113	1.774284
H	−1.192498	−0.503058	−0.422906
H	−0.840839	−0.578488	1.834028
H	−0.558801	1.091621	1.272649
H	1.219770	0.445090	2.723610
H	3.658166	−1.859831	1.463695
H	1.883312	1.502241	0.257753
H	2.116630	−0.593640	−0.856151
H	3.797515	−0.313442	−0.416341
N	2.446570	−2.948168	0.241427
C	1.556055	−3.202080	−0.796076
N	1.596266	−4.457198	−1.212815
C	2.559922	−5.070017	−0.411664
C	3.085653	−6.382476	−0.368859
N	2.683669	−7.364609	−1.205151
N	4.039014	−6.660827	0.559606
C	4.442943	−5.681748	1.392301
N	4.026551	−4.404223	1.436419
C	3.088985	−4.152229	0.507928
H	0.896047	−2.432061	−1.170716
H	3.137154	−8.269034	−1.173320
H	2.043366	−7.165485	−1.962063
H	5.207786	−5.962987	2.114111
P	4.800110	1.624301	1.499014
O	4.877832	1.877265	−0.081630
O	5.254981	2.719094	2.397693
O	5.654992	0.260447	1.642082
C	5.887138	−0.277687	2.992163
C	7.092482	−1.204151	2.987934
O	6.809343	−2.448430	2.290259
C	7.627432	−2.588981	1.108548
C	8.361248	−0.618125	2.338876
C	8.395812	−1.263214	0.951868
O	9.480472	−0.998544	3.216432
H	4.995274	−0.828018	3.304979
H	6.069674	0.551431	3.681857
H	7.298421	−1.451195	4.035106
H	8.292267	−3.449415	1.232465
H	8.346078	0.471322	2.288207
H	7.881718	−0.597187	0.257133
H	9.404654	−1.420014	0.575695
N	6.785206	−2.901672	−0.043298
C	5.910686	−2.038233	−0.704678
N	5.247684	−2.631391	−1.676654
C	5.690644	−3.953273	−1.662958
C	5.335079	−5.058220	−2.495877
O	4.537858	−5.104371	−3.446145
N	6.043631	−6.230943	−2.106432
C	6.946777	−6.322781	−1.067080
N	7.478651	−7.543529	−0.808040
N	7.286115	−5.280345	−0.314452
C	6.638033	−4.143398	−0.645134
H	5.819332	−1.004708	−0.409420
H	5.815537	−7.066600	−2.640899
H	7.427136	−8.280064	−1.500900
H	8.274239	−7.570182	−0.181210
P	11.040957	−0.905037	2.843706
O	11.183123	0.115266	1.616062
O	11.847561	−0.630269	4.063501
O	11.377571	−2.273176	2.074494
C	11.308396	−3.518670	2.858986
C	11.769034	−4.673229	1.996783
O	10.781663	−4.913396	0.953896
C	11.482738	−5.177345	−0.283612
C	13.139288	−4.478068	1.294000
C	12.770410	−4.350041	−0.192579
O	13.927301	−5.662944	1.572321
H	10.275298	−3.673849	3.183873
H	11.959184	−3.425635	3.733279
H	11.831605	−5.558437	2.643642
H	11.696612	−6.243678	−0.381023
H	13.660569	−3.589842	1.662474
H	12.577371	−3.300921	−0.434457
H	13.556130	−4.724986	−0.852483
H	14.811957	−5.526189	1.186300
N	10.583025	−4.843475	−1.389837
C	10.144131	−5.871654	−2.228987
O	10.497546	−7.049270	−2.121388
N	9.258565	−5.453364	−3.206578
C	8.787566	−4.153624	−3.448972
O	8.003538	−3.947676	−4.386856
C	9.286740	−3.145500	−2.525004
C	8.848111	−1.720101	−2.709665
C	10.144001	−3.541619	−1.551618
H	8.944725	−6.178254	−3.849348
H	9.150591	−1.350986	−3.696923
H	9.291264	−1.071816	−1.950088
H	7.757498	−1.635696	−2.656940
H	10.541219	−2.843728	−0.824597
H	11.557088	0.977627	1.882790
H	5.600330	2.480134	−0.345589

(ATA)$^{H+}$

E: −13902.26 kcal mol^{-1}

O	−0.141593	−0.366352	−0.098031
C	−0.113792	−0.003382	1.302580
C	1.311761	−0.074318	1.836833
O	1.775931	−1.455682	1.975486
C	2.807975	−1.760162	1.010679
C	2.361040	0.630384	0.960390
C	2.970358	−0.503320	0.134884

O	3.309852	1.276112	1.890025
H	−1.073652	−0.433819	−0.369668
H	−0.741829	−0.683031	1.891744
H	−0.479992	1.023960	1.440620
H	1.310415	0.364732	2.839452
H	3.727517	−2.019511	1.544174
H	1.934244	1.417422	0.338271
H	2.392844	−0.595091	−0.785793
H	4.010158	−0.329473	−0.133828
N	2.454274	−2.957230	0.247526
C	1.492351	−3.080383	−0.751619
N	1.417858	−4.304263	−1.246935
C	2.368986	−5.036157	−0.536813
C	2.782167	−6.387736	−0.603999
N	2.258686	−7.272151	−1.478748
N	3.743688	−6.806054	0.262775
C	4.258641	−5.921095	1.138986
N	3.963288	−4.616046	1.275461
C	3.012199	−4.223070	0.408735
H	0.869369	−2.241905	−1.030978
H	2.632398	−8.211577	−1.530514
H	1.599041	−6.969154	−2.182867
H	5.019284	−6.312823	1.811581
P	4.722851	1.920892	1.486884
O	4.692946	2.200103	−0.090639
O	5.050963	3.063838	2.381435
O	5.769157	0.699041	1.507390
C	6.012256	0.051754	2.804521
C	7.014800	−1.065761	2.618464
O	6.415151	−2.125109	1.824771
C	7.403077	−2.622269	0.891753
C	8.335069	−0.661132	1.908013
C	8.285419	−1.409896	0.575833
O	9.420649	−1.103545	2.798596
H	5.069420	−0.349223	3.187685
H	6.408429	0.791481	3.506604
H	7.260564	−1.453762	3.614766
H	7.979171	−3.437727	1.336431
H	8.435349	0.416602	1.782075
H	7.810534	−0.762915	−0.166173
H	9.262857	−1.695731	0.195439
N	6.700065	−3.207405	−0.249444
C	6.777863	−4.594100	−0.439497
O	7.468811	−5.344110	0.247762
N	6.005362	−5.052921	−1.492957
C	5.168910	−4.315997	−2.344377
O	4.537549	−4.892225	−3.239089
C	5.137280	−2.885187	−2.068815
C	4.288989	−2.005416	−2.943053
C	5.891666	−2.413919	−1.045869
H	6.052808	−6.055833	−1.661712
H	4.599543	−2.091450	−3.991091
H	4.370028	−0.957830	−2.643314
H	3.236644	−2.308006	−2.896621
H	5.900605	−1.362663	−0.785937
P	10.989299	−1.096673	2.471812
O	11.147358	−0.186667	1.162034
O	11.793205	−0.724561	3.667621
O	11.306255	−2.552831	1.871343
C	11.239551	−3.711452	2.776652
C	11.853555	−4.921905	2.094839
O	11.033610	−5.343444	0.969101
C	11.756643	−5.179793	−0.276781
C	13.275966	−4.719279	1.540834
C	13.039702	−4.402365	0.057037
O	13.984395	−5.970115	1.730515
H	10.189058	−3.907258	3.011326
H	11.787663	−3.482132	3.695604
H	11.857807	−5.733732	2.832145
H	11.967301	−6.172939	−0.683056
H	13.802678	−3.908128	2.054331
H	12.894563	−3.327351	−0.058305
H	13.876741	−4.709731	−0.573599
H	14.875331	−5.865628	1.348843
N	10.885047	−4.528995	−1.251519
C	10.657394	−3.172886	−1.453046
N	9.718944	−2.931773	−2.354009
C	9.292933	−4.192728	−2.768572
C	8.322858	−4.616678	−3.706842
N	7.569153	−3.754853	−4.421266
N	8.134858	−5.952779	−3.872758
C	8.880958	−6.809844	−3.147856
N	9.831872	−6.524312	−2.239752
C	9.999759	−5.197665	−2.090080
H	11.210086	−2.416983	−0.915018
H	6.894539	−4.107082	−5.088402
H	7.718193	−2.757606	−4.349724
H	8.686208	−7.866891	−3.321778
H	11.921816	0.407650	1.203067
H	4.643656	3.152361	−0.303999

(ATC)$^{H+}$

E: −13502.99 kcal mol^{-1}

O	−0.134177	−0.358385	−0.084991
C	−0.127818	−0.007379	1.318989
C	1.291364	−0.072239	1.870595
O	1.767426	−1.451679	1.989766
C	2.814647	−1.726454	1.032328
C	2.343636	0.657439	1.018625
C	2.965920	−0.455447	0.176561
O	3.282517	1.287298	1.968665
H	−1.061976	−0.434850	−0.368453
H	−0.757708	−0.697619	1.893776
H	−0.504189	1.015705	1.461908
H	1.273446	0.349073	2.880604
H	3.731935	−1.978042	1.573794
H	1.918188	1.455434	0.409697
H	2.390369	−0.537644	−0.745952
H	4.003293	−0.265529	−0.089189
N	2.491039	−2.918161	0.248229
C	1.563184	−3.040469	−0.782828
N	1.520853	−4.258503	−1.296031
C	2.458363	−4.987582	−0.565093
C	2.890334	−6.333529	−0.634113
N	2.415207	−7.210346	−1.542073
N	3.827017	−6.750818	0.260152
C	4.301662	−5.870668	1.163160
N	3.985859	−4.570837	1.304913
C	3.059694	−4.179018	0.411344
H	0.938088	−2.207144	−1.072254
H	2.766653	−8.159358	−1.563506
H	1.739083	−6.920358	−2.235677
H	5.043552	−6.261604	1.856815
P	4.702225	1.934017	1.597464
O	4.696245	2.210022	0.019401
O	5.012663	3.080866	2.493251
O	5.749454	0.712799	1.650515
C	5.971951	0.077775	2.957848
C	6.998061	−1.023961	2.801193
O	6.433010	−2.095153	1.996766
C	7.426501	−2.552343	1.050769
C	8.324012	−0.597155	2.119097
C	8.301009	−1.322669	0.773045
O	9.405462	−1.046911	3.010695
H	5.026923	−0.337614	3.319704

H	6.338075	0.828309	3.664705
H	7.226521	−1.405406	3.803838
H	8.008758	−3.377683	1.467770
H	8.415133	0.483467	2.011999
H	7.834753	−0.665918	0.034532
H	9.292261	−1.589297	0.414910
N	6.730482	−3.104780	−0.112592
C	6.819902	−4.484138	−0.350479
O	7.494521	−5.256331	0.327333
N	6.082291	−4.907598	−1.443109
C	5.283235	−4.141208	−2.302380
O	4.696543	−4.682421	−3.248395
C	5.231978	−2.721796	−1.973765
C	4.415562	−1.814455	−2.850215
C	5.945576	−2.285929	−0.906497
H	6.146143	−5.903245	−1.647101
H	4.763037	−1.870104	−3.888724
H	4.485745	−0.776244	−2.517635
H	3.362278	−2.117278	−2.852022
H	5.939823	−1.244818	−0.607906
P	10.977236	−0.969557	2.690287
O	11.111539	0.033725	1.448319
O	11.763315	−0.657111	3.914984
O	11.337934	−2.368226	1.986544
C	11.296940	−3.584006	2.818491
C	11.751338	−4.770185	1.990943
O	10.766698	−5.034463	0.955192
C	11.431933	−5.168954	−0.327404
C	13.121309	−4.611890	1.288270
C	12.751583	−4.396949	−0.186696
O	13.849085	−5.848041	1.504304
H	10.269712	−3.738329	3.162473
H	11.958926	−3.449635	3.679241
H	11.800740	−5.633336	2.668072
H	11.600982	−6.223993	−0.547695
H	13.691212	−3.769827	1.693393
H	12.612192	−3.328552	−0.370413
H	13.520677	−4.768653	−0.868079
H	14.721381	−5.754819	1.079233
N	10.516744	−4.688569	−1.366666
C	9.841423	−5.635375	−2.206266
O	10.076072	−6.851916	−2.069745
N	8.968953	−5.155281	−3.136193
C	8.718101	−3.842633	−3.232534
N	7.838018	−3.435906	−4.170110
C	9.329344	−2.882550	−2.361492
C	10.216985	−3.357832	−1.450954
H	7.422703	−4.111877	−4.799678
H	7.651592	−2.453877	−4.319735
H	9.100906	−1.825664	−2.427003
H	10.724527	−2.712832	−0.745335
H	11.809088	0.705417	1.577421
H	4.768412	3.160025	−0.197083

(ATG)$^{H+}$

E: −14066.25 kcal mol^{-1}

O	−0.150900	−0.395788	−0.098469
C	−0.113581	−0.038613	1.303396
C	1.315872	−0.109455	1.826748
O	1.784317	−1.490869	1.953375
C	2.812895	−1.785259	0.982143
C	2.357407	0.603335	0.947663
C	2.964038	−0.523884	0.111486
O	3.309182	1.246971	1.875576
H	−1.084812	−0.458478	−0.364905
H	−0.736297	−0.721662	1.894356
H	−0.479995	0.987656	1.448707
H	1.320711	0.323113	2.832150
H	3.736324	−2.042800	1.509946
H	1.924668	1.392759	0.332797
H	2.378292	−0.613350	−0.804202
H	4.000499	−0.345163	−0.166998
N	2.461702	−2.980428	0.215235
C	1.492096	−3.105032	−0.776230
N	1.423470	−4.326543	−1.278638
C	2.386946	−5.054797	−0.581396
C	2.810058	−6.402922	−0.659511
N	2.287091	−7.285467	−1.535677
N	3.782888	−6.818128	0.196345
C	4.297947	−5.933778	1.073301
N	3.992991	−4.632233	1.220223
C	3.031519	−4.242214	0.363587
H	0.860196	−2.269764	−1.045188
H	2.655177	−8.227108	−1.586114
H	1.605243	−6.987910	−2.220542
H	5.068100	−6.322857	1.736678
P	4.727393	1.880157	1.474051
O	4.702055	2.154717	−0.104225
O	5.062262	3.024039	2.364774
O	5.763897	0.650194	1.503258
C	6.002359	0.010680	2.805483
C	7.038289	−1.079694	2.639779
O	6.479682	−2.169561	1.854070
C	7.466822	−2.596650	0.882488
C	8.346942	−0.642444	1.931421
C	8.274712	−1.333712	0.571450
O	9.441628	−1.140795	2.780238
H	5.064010	−0.414716	3.173000
H	6.366461	0.761737	3.512975
H	7.286284	−1.448845	3.642217
H	8.097929	−3.385915	1.298974
H	8.450567	0.439735	1.856230
H	7.732499	−0.678442	−0.115377
H	9.245093	−1.552534	0.133894
N	6.768331	−3.204721	−0.248322
C	6.871394	−4.591897	−0.419034
O	7.565085	−5.323271	0.285660
N	6.119204	−5.077689	−1.475648
C	5.267105	−4.368186	−2.337023
O	4.653608	−4.970786	−3.226485
C	5.195534	−2.937847	−2.069558
C	4.322702	−2.086503	−2.947708
C	5.935902	−2.439967	−1.048968
H	6.157603	−6.086521	−1.606369
H	4.647311	−2.158073	−3.992492
H	4.363422	−1.037819	−2.643624
H	3.281596	−2.426541	−2.910759
H	5.913880	−1.387221	−0.795946
P	11.013071	−1.079898	2.478759
O	11.158265	−0.183126	1.158781
O	11.790766	−0.662570	3.677500
O	11.385087	−2.534250	1.907661
C	11.310338	−3.687833	2.817832
C	11.896682	−4.910118	2.132099
O	11.061069	−5.316980	1.012311
C	11.775176	−5.161069	−0.240062
C	13.319024	−4.735801	1.568827
C	13.080253	−4.416008	0.086429
O	14.004271	−6.000226	1.756192
H	10.259787	−3.867234	3.065080
H	11.872438	−3.463710	3.729814
H	11.890366	−5.721503	2.869956
H	11.958118	−6.155690	−0.655555
H	13.864879	−3.934451	2.078043
H	12.961313	−3.338060	−0.031793

H	13.905330	−4.745333	−0.549056
H	14.894556	−5.913673	1.368559
N	10.914350	−4.479308	−1.202636
C	10.705059	−3.109998	−1.367134
N	9.786530	−2.839969	−2.274773
C	9.352946	−4.083571	−2.732106
C	8.386124	−4.421192	−3.728218
O	7.681853	−3.672433	−4.427250
N	8.269802	−5.833564	−3.860511
C	8.993604	−6.775472	−3.156026
N	8.708911	−8.082489	−3.391712
N	9.900986	−6.446904	−2.240292
C	10.039731	−5.113393	−2.072674
H	11.254106	−2.377928	−0.794300
H	7.591570	−6.146828	−4.552161
H	8.226304	−8.349794	−4.241400
H	9.350417	−8.764800	−3.003874
H	11.927980	0.417972	1.188316
H	4.688252	3.107549	−0.320168

(ATT)^H+^

E: −13762.92 kcal mol^{-1}

O	0.146221	−0.224881	−0.259336
C	−0.000086	0.082960	1.147355
C	1.340524	−0.060523	1.857773
O	1.737222	−1.460959	2.002826
C	2.877942	−1.771208	1.170153
C	2.515328	0.632719	1.148773
C	3.197127	−0.494025	0.369779
O	3.358768	1.209035	2.215425
H	−0.743660	−0.249845	−0.652642
H	−0.721885	−0.594630	1.620065
H	−0.348101	1.117436	1.279845
H	1.224568	0.345607	2.867513
H	3.710610	−2.080321	1.807608
H	2.196617	1.452838	0.505280
H	2.751716	−0.536871	−0.625266
H	4.270729	−0.348409	0.261350
N	2.584241	−2.930230	0.328160
C	1.726086	−2.992923	−0.766382
N	1.648887	−4.204041	−1.291454
C	2.488372	−4.990097	−0.502594
C	2.839947	−6.360014	−0.548795
N	2.369969	−7.206705	−1.487923
N	3.686813	−6.835315	0.404065
C	4.152334	−5.987571	1.342159
N	3.905994	−4.671291	1.471052
C	3.066519	−4.221744	0.519836
H	1.172378	−2.121839	−1.089099
H	2.682647	−8.169439	−1.502987
H	1.788203	−6.867904	−2.242320
H	4.821115	−6.424054	2.081498
P	4.765955	1.941470	1.969011
O	4.807740	2.393554	0.431379
O	4.996522	2.984611	3.004880
O	5.862249	0.768795	1.901102
C	6.107711	−0.013266	3.119289
C	7.011500	−1.176298	2.777053
O	6.309367	−2.082868	1.896713
C	7.305374	−2.822828	1.160907
C	8.342196	−0.814053	2.060350
C	8.440185	−1.816255	0.897268
O	9.400646	−0.935961	3.071401
H	5.155630	−0.380155	3.513751
H	6.590792	0.625859	3.863991
H	7.262429	−1.690002	3.716130
H	7.652778	−3.687584	1.731092
H	8.348757	0.216102	1.704923
H	8.274390	−1.293751	−0.046838
H	9.405355	−2.318376	0.852876
N	6.663905	−3.355399	−0.042973
C	6.689995	−4.735937	−0.269941
O	7.307024	−5.536682	0.430886
N	5.963278	−5.126252	−1.383166
C	5.239026	−4.320241	−2.278697
O	4.634081	−4.839994	−3.222792
C	5.291769	−2.893504	−1.982059
C	4.589819	−1.939222	−2.904845
C	5.989665	−2.492779	−0.891812
H	5.947967	−6.129167	−1.559222
H	5.003331	−2.016370	−3.917416
H	4.700759	−0.907621	−2.560249
H	3.522260	−2.177612	−2.972003
H	6.055783	−1.449014	−0.609423
P	10.965335	−0.703791	2.769032
O	11.071999	0.285849	1.512762
O	11.667493	−0.315938	4.021970
O	11.483880	−2.039602	2.049795
C	11.431158	−3.304347	2.800155
C	11.738852	−4.444654	1.854126
O	10.666184	−4.550768	0.878408
C	11.256496	−4.958403	−0.379938
C	13.071560	−4.330236	1.060403
C	12.623662	−4.263130	−0.411359
O	13.842831	−5.519063	1.362473
H	10.431496	−3.426889	3.227353
H	12.171843	−3.272056	3.604327
H	11.777666	−5.364854	2.453390
H	11.357199	−6.045375	−0.416786
H	13.637798	−3.440087	1.348608
H	12.524038	−3.218114	−0.717256
H	13.328670	−4.758646	−1.082672
H	14.723267	−5.410611	0.958335
N	10.323330	−4.601613	−1.450603
C	9.667765	−5.630963	−2.139296
O	9.854894	−6.831437	−1.927459
N	8.775384	−5.179404	−3.096365
C	8.467227	−3.852781	−3.440899
O	7.652307	−3.622311	−4.344963
C	9.161881	−2.847560	−2.650531
C	8.888954	−1.394300	−2.922622
C	10.047669	−3.271463	−1.714993
H	8.311316	−5.908204	−3.635841
H	9.168184	−1.132872	−3.950376
H	9.453970	−0.755434	−2.238489
H	7.820980	−1.171729	−2.816202
H	10.600063	−2.567270	−1.106036
H	11.227044	1.214818	1.773105
H	4.660762	3.352094	0.312780

(CAA)^H+^

E: −13642.65 kcal mol^{-1}

O	−0.168835	−0.383968	−0.557544
C	−0.172992	0.033378	0.828967
C	1.220638	−0.112220	1.428986
O	1.581809	−1.518418	1.597866
C	2.726945	−1.859284	0.766922
C	2.354413	0.509986	0.593793
C	2.917256	−0.672728	−0.187293
O	3.309302	1.077993	1.574096
H	−1.094334	−0.447371	−0.851319
H	−0.868835	−0.580098	1.414512

H	−0.476349	1.086812	0.913065
H	1.201051	0.349098	2.422231
H	3.598152	−2.019970	1.403959
H	2.021530	1.330971	−0.041641
H	2.317314	−0.789835	−1.091773
H	3.953575	−0.548730	−0.489301
N	2.487067	−3.147737	0.112473
C	3.201700	−4.307436	0.570270
O	4.022958	−4.188679	1.497397
N	2.945830	−5.495276	−0.045403
C	2.030596	−5.582609	−1.018837
N	1.841718	−6.788105	−1.596022
C	1.256656	−4.453569	−1.447147
C	1.512020	−3.264033	−0.843007
H	2.389240	−7.583429	−1.290883
H	1.155212	−6.919309	−2.326453
H	0.486837	−4.543472	−2.204946
H	0.948122	−2.363544	−1.062368
P	4.803779	1.568905	1.309328
O	4.904641	1.765982	−0.278334
O	5.161676	2.729598	2.169527
O	5.741919	0.274351	1.520769
C	5.962461	−0.211923	2.890655
C	7.178523	−1.123914	2.930633
O	6.926413	−2.381430	2.245542
C	7.736993	−2.488985	1.047802
C	8.452039	−0.543417	2.288800
C	8.436780	−1.132041	0.879833
O	9.571208	−1.007475	3.130653
H	5.075477	−0.766168	3.210861
H	6.124342	0.642590	3.554638
H	7.365188	−1.350976	3.985732
H	8.445785	−3.312077	1.183866
H	8.479507	0.546916	2.293516
H	7.848527	−0.460571	0.253655
H	9.422654	−1.221225	0.431729
N	6.903243	−2.872251	−0.082789
C	6.128374	−2.065216	−0.906792
N	5.445463	−2.749564	−1.808740
C	5.778758	−4.083001	−1.571375
C	5.388009	−5.292467	−2.187217
N	4.540570	−5.335844	−3.243201
N	5.918107	−6.449439	−1.717313
C	6.778828	−6.397521	−0.682467
N	7.210369	−5.313398	−0.011986
C	6.679596	−4.180729	−0.500025
H	6.111775	−0.991686	−0.796049
H	4.168316	−6.233289	−3.530067
H	4.014067	−4.507957	−3.491415
H	7.174133	−7.355567	−0.348905
P	11.121044	−1.110595	2.761887
O	11.302774	−0.073068	1.553913
O	11.974512	−0.926217	3.967015
O	11.321051	−2.524610	2.019037
C	11.317806	−3.747852	2.840826
C	11.854048	−4.910687	2.026218
O	10.927019	−5.237598	0.950403
C	11.594265	−5.142979	−0.333662
C	13.231576	−4.682493	1.369963
C	12.882911	−4.344200	−0.085100
O	13.964187	−5.929001	1.485396
H	10.289057	−3.950048	3.154143
H	11.947404	−3.589879	3.721470
H	11.914373	−5.771652	2.703600
H	11.798305	−6.150669	−0.707757
H	13.786646	−3.874686	1.858190
H	12.698796	−3.271104	−0.172258
H	13.677385	−4.619208	−0.782613
H	14.828030	−5.805575	1.050751
N	10.680361	−4.536264	−1.291173
C	10.305400	−3.201593	−1.374131
N	9.381411	−2.975578	−2.291624
C	9.124145	−4.227736	−2.849427
C	8.242281	−4.661228	−3.864385
N	7.416975	−3.817174	−4.534918
N	8.242935	−5.977851	−4.194032
C	9.060267	−6.815293	−3.527989
N	9.920184	−6.523500	−2.534344
C	9.917925	−5.212405	−2.237158
H	10.753178	−2.453810	−0.736720
H	6.666575	−4.230897	−5.077779
H	7.240086	−2.900488	−4.140557
H	9.014001	−7.860354	−3.829988
H	12.231577	0.185737	1.394077
H	5.501473	2.495232	−0.536783

(CAC)$^{H+}$

E: −13244.21 kcal mol^{-1}

O	−7.010300	0.077969	−0.920839
C	−7.185023	1.268418	−0.113941
C	−5.828314	1.828505	0.300014
O	−5.168821	0.955448	1.270876
C	−3.943202	0.403865	0.708753
C	−4.826323	1.993789	−0.855639
C	−3.998371	0.717611	−0.791386
O	−4.041052	3.215169	−0.551124
H	−7.880523	−0.347455	−1.014547
H	−7.757494	1.038498	0.793352
H	−7.720249	2.043149	−0.682094
H	−6.002262	2.796107	0.783418
H	−3.085924	0.871575	1.195993
H	−5.305199	2.155371	−1.821925
H	−4.535255	−0.055875	−1.343130
H	−3.010215	0.812964	−1.229239
N	−3.840014	−1.018216	1.040498
C	−2.919501	−1.429621	2.064835
O	−2.278618	−0.564973	2.689217
N	−2.783680	−2.765156	2.298185
C	−3.553203	−3.654391	1.657196
N	−3.353981	−4.963419	1.923681
C	−4.571990	−3.258336	0.728273
C	−4.676150	−1.932565	0.452587
H	−2.556489	−5.238762	2.484521
H	−3.857697	−5.679129	1.417107
H	−5.228313	−3.982289	0.258738
H	−5.427196	−1.530764	−0.219058
P	−2.673359	3.672739	−1.232745
O	−2.635617	2.884628	−2.627547
O	−2.552881	5.155289	−1.274638
O	−1.487120	2.938781	−0.424079
C	−1.172512	3.415623	0.930711
C	0.171328	2.863450	1.376545
O	0.110784	1.427006	1.582232
C	0.947949	0.727350	0.631260
C	1.337979	3.112423	0.403622
C	1.484251	1.787311	−0.347085
O	2.503202	3.448408	1.244118
H	−1.956100	3.071389	1.612227
H	−1.138819	4.509542	0.928949

H	0.394691	3.331440	2.342211
H	1.750514	0.221080	1.173212
H	1.161316	3.956167	−0.264917
H	0.874776	1.841500	−1.250225
H	2.510874	1.574242	−0.641706
N	0.187637	−0.337925	−0.017549
C	−0.689937	−0.243341	−1.093169
N	−1.250916	−1.397515	−1.409532
C	−0.720988	−2.312452	−0.499573
C	−0.887123	−3.707067	−0.336957
N	−1.680873	−4.448310	−1.146148
N	−0.193880	−4.324437	0.652261
C	0.614446	−3.585106	1.435581
N	0.857195	−2.262444	1.372195
C	0.169360	−1.673336	0.379512
H	−0.869386	0.695783	−1.594376
H	−1.870108	−5.411623	−0.896754
H	−2.319350	−3.986356	−1.780831
H	1.140793	−4.130139	2.217661
P	3.996705	3.650380	0.684238
O	3.879546	4.044471	−0.865371
O	4.752313	4.569243	1.577836
O	4.608337	2.180858	0.501746
C	4.826645	1.371605	1.711027
C	5.188589	−0.039193	1.306849
O	4.042027	−0.675587	0.690469
C	4.572634	−1.716379	−0.168991
C	6.378775	−0.188720	0.314146
C	5.809732	−1.073172	−0.814763
O	7.462270	−0.809863	1.048823
H	3.912837	1.371635	2.313401
H	5.644335	1.813660	2.288150
H	5.455216	−0.579959	2.227942
H	4.833842	−2.598843	0.419878
H	6.706438	0.781926	−0.069005
H	5.512026	−0.451622	−1.665098
H	6.534050	−1.814809	−1.161015
H	8.260216	−0.765459	0.490979
N	3.519781	−2.138689	−1.087676
C	3.111263	−3.513201	−1.116993
O	3.686837	−4.337748	−0.379738
N	2.104646	−3.857947	−1.966428
C	1.526592	−2.943357	−2.756902
N	0.534902	−3.358469	−3.577068
C	1.937369	−1.569830	−2.768344
C	2.932578	−1.219576	−1.914396
H	0.151586	−4.287369	−3.441357
H	−0.040917	−2.678674	−4.057281
H	1.468689	−0.839047	−3.416097
H	3.301097	−0.204503	−1.832475
H	3.960360	5.005487	−1.021417
H	−2.101585	3.332556	−3.312439

(CAG)$^{H+}$

E: −13807.79 kcal mol^{-1}

O	−0.014135	−0.499116	−0.707671
C	−0.059939	−0.036249	0.663689
C	1.314886	−0.162563	1.310144
O	1.667417	−1.562796	1.536177
C	2.833025	−1.932431	0.746610
C	2.474616	0.429462	0.488505
C	3.051042	−0.779185	−0.241082
O	3.405202	1.025048	1.476655
H	−0.930458	−0.575539	−1.026031
H	−0.773482	−0.630571	1.247813
H	−0.365120	1.019279	0.704110
H	1.266580	0.331522	2.286539
H	3.686412	−2.071064	1.411985
H	2.163593	1.232401	−0.180202
H	2.470162	−0.921781	−1.154366
H	4.094761	−0.672498	−0.523795
N	2.611401	−3.243478	0.133571
C	3.293685	−4.387118	0.674284
O	4.064730	−4.234231	1.638861
N	3.064095	−5.596937	0.093579
C	2.200909	−5.719142	−0.922570
N	2.045859	−6.943211	−1.469340
C	1.448165	−4.607419	−1.427498
C	1.678967	−3.395705	−0.859498
H	2.590354	−7.722344	−1.120284
H	1.397020	−7.101140	−2.228335
H	0.715896	−4.726024	−2.217740
H	1.129177	−2.503755	−1.139702
P	4.921609	1.463899	1.243734
O	5.072558	1.639832	−0.342321
O	5.292018	2.622700	2.101065
O	5.812779	0.142886	1.491265
C	5.967348	−0.345047	2.869627
C	7.204317	−1.223418	2.977261
O	7.026518	−2.501058	2.309393
C	7.857820	−2.593805	1.125798
C	8.486458	−0.611131	2.385423
C	8.586357	−1.244867	0.996484
O	9.575484	−0.983931	3.309000
H	5.077771	−0.922662	3.136326
H	6.069927	0.509553	3.545206
H	7.348933	−1.427926	4.043474
H	8.548852	−3.431162	1.257921
H	8.462710	0.478571	2.347073
H	8.080654	−0.583034	0.293431
H	9.610378	−1.370677	0.654405
N	7.040883	−2.940521	−0.034564
C	6.327928	−2.103658	−0.887529
N	5.620467	−2.762059	−1.788528
C	5.867956	−4.108215	−1.523326
C	5.422868	−5.303337	−2.132098
N	4.601097	−5.318283	−3.206407
N	5.868180	−6.482406	−1.629402
C	6.696771	−6.463894	−0.568894
N	7.178733	−5.396767	0.094200
C	6.739537	−4.241336	−0.430906
H	6.370158	−1.029205	−0.796019
H	4.191461	−6.196772	−3.498801
H	4.169109	−4.459880	−3.521613
H	7.020013	−7.438534	−0.206733
P	11.145563	−0.988411	3.011106
O	11.326232	0.124358	1.872940
O	11.927557	−0.828613	4.267226
O	11.449071	−2.345025	2.202412
C	11.336892	−3.620886	2.927520
C	11.706443	−4.754922	1.992546
O	10.707044	−4.855405	0.936390
C	11.376170	−4.930189	−0.349480
C	13.084948	−4.619408	1.302056
C	12.727749	−4.236002	−0.139943
O	13.730113	−5.915452	1.387918
H	10.306139	−3.737915	3.275591

H	12.015118	−3.607117	3.786053
H	11.695220	−5.679849	2.583747
H	11.495975	−5.978968	−0.640229
H	13.708106	−3.860680	1.785705
H	12.617179	−3.150709	−0.206456
H	13.480700	−4.560185	−0.862127
H	14.611558	−5.833805	0.979411
N	10.522846	−4.324672	−1.355659
C	10.301826	−2.965997	−1.586319
N	9.430430	−2.749138	−2.551066
C	9.047896	−4.017672	−2.985876
C	8.147819	−4.412447	−4.022949
O	7.475077	−3.705498	−4.793306
N	8.073430	−5.829047	−4.115006
C	8.780186	−6.729849	−3.345187
N	8.557479	−8.048933	−3.567807
N	9.622946	−6.349924	−2.388208
C	9.715098	−5.009568	−2.252506
H	10.819621	−2.203990	−1.023464
H	7.429741	−6.180229	−4.820676
H	8.113871	−8.353287	−4.425428
H	9.188305	−8.704709	−3.122413
H	12.245562	0.438765	1.769965
H	5.671414	2.370649	−0.591131

(CAT)$^{H+}$

E: −13503.05 kcal mol^{-1}

O	−0.117548	−0.313692	−0.488320
C	−0.113543	0.101665	0.898721
C	1.277396	−0.069601	1.499106
O	1.616799	−1.483296	1.655268
C	2.757209	−1.830946	0.819795
C	2.422126	0.540370	0.670256
C	2.950256	−0.643123	−0.131507
O	3.396196	1.064967	1.657634
H	−1.044197	−0.359175	−0.781850
H	−0.819607	−0.500884	1.483458
H	−0.398715	1.160019	0.984883
H	1.263281	0.383107	2.496319
H	3.629457	−1.996998	1.453865
H	2.108672	1.381762	0.051978
H	2.327045	−0.743627	−1.022142
H	3.980051	−0.532872	−0.457856
N	2.509484	−3.117233	0.165818
C	3.229457	−4.277205	0.613547
O	4.050084	−4.162553	1.541893
N	2.982070	−5.460272	−0.014401
C	2.068151	−5.543342	−0.989601
N	1.888998	−6.743805	−1.580111
C	1.285581	−4.415346	−1.405048
C	1.535513	−3.229554	−0.791103
H	2.453616	−7.534232	−1.293900
H	1.214054	−6.869089	−2.322237
H	0.516703	−4.502691	−2.164034
H	0.969818	−2.328882	−1.004676
P	4.908792	1.507233	1.415982
O	5.039708	1.702822	−0.169852
O	5.293848	2.652836	2.284324
O	5.804184	0.182538	1.638347
C	5.986804	−0.315231	3.010283
C	7.200844	−1.228464	3.081268
O	6.961504	−2.502190	2.423256
C	7.770494	−2.640728	1.233005
C	8.484510	−0.654784	2.453098
C	8.552809	−1.322427	1.076820
O	9.584843	−1.028173	3.359440
H	5.089525	−0.868150	3.302311
H	6.133224	0.534761	3.683275
H	7.371154	−1.435516	4.143255
H	8.429489	−3.504318	1.355973
H	8.474081	0.433773	2.386302
H	8.074302	−0.660605	0.354350
H	9.572355	−1.501971	0.741815
N	6.926704	−2.952378	0.081347
C	6.139889	−2.080239	−0.662880
N	5.448052	−2.685557	−1.611347
C	5.784469	−4.032662	−1.491799
C	5.379367	−5.184438	−2.203362
N	4.521427	−5.135660	−3.247405
N	5.897616	−6.382413	−1.828987
C	6.751487	−6.423568	−0.787609
N	7.201137	−5.400634	−0.037264
C	6.689248	−4.223478	−0.435483
H	6.119356	−1.022849	−0.449397
H	4.142575	−6.000653	−3.613257
H	4.030555	−4.275232	−3.453264
H	7.129712	−7.410176	−0.526843
P	11.151436	−0.890031	3.023107
O	11.299559	0.193855	1.851502
O	11.926209	−0.665469	4.273539
O	11.532601	−2.204349	2.185111
C	11.426064	−3.502813	2.870097
C	11.716988	−4.609352	1.880625
O	10.647061	−4.658896	0.898349
C	11.232938	−5.024294	−0.374085
C	13.055078	−4.490004	1.097132
C	12.613950	−4.355182	−0.371744
O	13.800691	−5.705919	1.353268
H	10.414359	−3.611510	3.272399
H	12.151348	−3.533737	3.688574
H	11.735805	−5.552693	2.443948
H	11.310936	−6.110261	−0.459713
H	13.638086	−3.624163	1.423399
H	12.533608	−3.297172	−0.636594
H	13.312280	−4.835890	−1.060707
H	14.682639	−5.601438	0.951536
N	10.314426	−4.598060	−1.430481
C	9.672090	−5.576090	−2.198857
O	9.859440	−6.788191	−2.069705
N	8.796069	−5.059605	−3.136845
C	8.508591	−3.712972	−3.407172
O	7.725670	−3.420802	−4.322487
C	9.191891	−2.763321	−2.540339
C	8.941632	−1.293186	−2.729431
C	10.049146	−3.252576	−1.610782
H	8.326816	−5.750930	−3.718988
H	9.265158	−0.970382	−3.726536
H	9.484358	−0.705806	−1.984047
H	7.872669	−1.067015	−2.650632
H	10.586917	−2.596211	−0.938170
H	11.602635	1.064135	2.176059
H	5.675801	2.401165	−0.420193

(CCA)$^{H+}$

E: −13241.42 kcal mol^{-1}

O	−0.264134	−0.195382	−0.347858
C	−0.233490	0.149310	1.058410
C	1.171175	−0.046700	1.616671
O	1.509414	−1.465315	1.716965
C	2.623960	−1.796971	0.846062
C	2.293005	0.593476	0.779047
C	2.837768	−0.566931	−0.050174

O	3.272527	1.128334	1.749417
H	−1.196023	−0.228621	−0.626209
H	−0.926047	−0.483913	1.626523
H	−0.518741	1.201168	1.202438
H	1.185455	0.370590	2.629146
H	3.501827	−2.025322	1.452857
H	1.948550	1.430078	0.170907
H	2.245578	−0.634936	−0.964748
H	3.879904	−0.440826	−0.333965
N	2.324736	−3.036642	0.120654
C	3.026139	−4.241492	0.467837
O	3.876368	−4.211062	1.375952
N	2.729928	−5.372570	−0.231235
C	1.789894	−5.366003	−1.185236
N	1.572432	−6.512869	−1.862307
C	1.021536	−4.195350	−1.494941
C	1.318297	−3.061365	−0.809580
H	2.099705	−7.345169	−1.627809
H	0.857248	−6.573546	−2.574366
H	0.228159	−4.212714	−2.233341
H	0.767402	−2.135975	−0.941965
P	4.725430	1.705806	1.407218
O	4.732876	1.967037	−0.174062
O	5.078970	2.850034	2.291281
O	5.723986	0.445946	1.503527
C	5.987323	−0.114376	2.838193
C	7.106908	−1.135113	2.750577
O	6.661890	−2.310240	2.021946
C	7.557966	−2.578979	0.906913
C	8.390089	−0.643539	2.046253
C	8.292082	−1.257252	0.652619
O	9.506059	−1.187060	2.842966
H	5.075715	−0.595137	3.204969
H	6.276930	0.694954	3.514953
H	7.350150	−1.428014	3.778718
H	8.247464	−3.382182	1.174812
H	8.487381	0.442432	2.036004
H	7.700726	−0.580295	0.032155
H	9.253101	−1.399842	0.166444
N	6.769971	−3.098489	−0.208423
C	6.776116	−4.514645	−0.465045
O	7.502643	−5.251208	0.223892
N	5.984814	−4.981831	−1.469573
C	5.190821	−4.158074	−2.160068
N	4.443243	−4.685297	−3.152860
C	5.119501	−2.752272	−1.884056
C	5.918572	−2.278607	−0.894610
H	4.434448	−5.688332	−3.291093
H	3.765695	−4.123581	−3.650223
H	4.452880	−2.096501	−2.430853
H	5.914747	−1.238310	−0.595416
P	11.070497	−1.134961	2.521246
O	11.192765	−0.198404	1.226058
O	11.875760	−0.757747	3.715545
O	11.426137	−2.578269	1.909680
C	11.418819	−3.744170	2.807752
C	12.021497	−4.941572	2.092383
O	11.160222	−5.376753	1.001882
C	11.820198	−5.190917	−0.275815
C	13.414716	−4.714466	1.477312
C	13.109393	−4.400471	0.006310
O	14.150899	−5.954174	1.635271
H	10.383355	−3.959097	3.088101
H	12.004065	−3.511236	3.702682
H	12.071468	−5.755592	2.825549
H	12.022096	−6.175948	−0.705457
H	13.949744	−3.895204	1.969129
H	12.946747	−3.327230	−0.106026
H	13.921353	−4.698511	−0.660753
H	15.021951	−5.836460	1.213752
N	10.901190	−4.535862	−1.201374
C	10.585869	−3.186001	−1.290061
N	9.637257	−2.929886	−2.175165
C	9.297771	−4.175899	−2.700437
C	8.357921	−4.579257	−3.674679
N	7.570918	−3.700783	−4.343430
N	8.265117	−5.901366	−3.967086
C	9.060989	−6.768458	−3.312024
N	9.983181	−6.503444	−2.367606
C	10.064884	−5.187667	−2.101695
H	11.085766	−2.449084	−0.679436
H	6.787033	−4.063503	−4.873632
H	7.509792	−2.745265	−4.014731
H	8.941134	−7.816328	−3.582932
H	12.030836	0.302956	1.191975
H	5.084023	2.848077	−0.408835

(CCC)$^{H+}$

E: −12842.74 kcal mol^{-1}

O	−0.221367	−0.070280	−0.496315
C	−0.264521	0.290966	0.905283
C	1.104149	0.084196	1.543568
O	1.420932	−1.337458	1.672339
C	2.576153	−1.685109	0.862339
C	2.278460	0.703275	0.765008
C	2.836524	−0.467558	−0.037760
O	3.217815	1.216529	1.788751
H	−1.136754	−0.094771	−0.825938
H	−0.995390	−0.326800	1.441474
H	−0.543643	1.347873	1.021403
H	1.066520	0.509193	2.552123
H	3.422663	−1.905670	1.514644
H	1.984862	1.550979	0.145671
H	2.269265	−0.534887	−0.968350
H	3.887351	−0.353041	−0.291836
N	2.309634	−2.934601	0.142378
C	3.010601	−4.129423	0.524305
O	3.833323	−4.080966	1.456679
N	2.744959	−5.270801	−0.169733
C	1.836626	−5.282682	−1.154375
N	1.643731	−6.441595	−1.817701
C	1.075789	−4.119396	−1.508199
C	1.340425	−2.975496	−0.825941
H	2.170795	−7.266024	−1.556474
H	0.955228	−6.516275	−2.554256
H	0.311458	−4.150074	−2.276294
H	0.791842	−2.053753	−0.990744
P	4.726923	1.685744	1.575698
O	4.814713	2.005635	0.008016
O	5.105034	2.765576	2.527299
O	5.642442	0.362353	1.692545
C	5.912836	−0.176343	3.035275
C	7.072918	−1.153516	2.971511
O	6.685992	−2.355577	2.253650
C	7.572292	−2.589250	1.128236
C	8.343438	−0.616758	2.278938
C	8.299780	−1.256778	0.893383
O	9.473855	−1.069453	3.111698
H	5.014890	−0.688928	3.392327
H	6.161159	0.648635	3.709050
H	7.314485	−1.426575	4.005320
H	8.265339	−3.398466	1.366698
H	8.385340	0.472353	2.243659
H	7.727419	−0.595566	0.239535
H	9.284492	−1.388861	0.452594

N	6.781635	−3.077923	−0.003161
C	6.829928	−4.475112	−0.340901
O	7.563571	−5.232627	0.317482
N	6.070783	−4.902878	−1.387425
C	5.266406	−4.060910	−2.045107
N	4.543202	−4.548931	−3.073105
C	5.154624	−2.675948	−1.689113
C	5.921205	−2.241162	−0.657661
H	4.574295	−5.538711	−3.284375
H	3.881638	−3.968206	−3.569902
H	4.480438	−2.007403	−2.210179
H	5.882464	−1.221739	−0.295906
P	11.026542	−1.076624	2.737913
O	11.133279	−0.014056	1.542995
O	11.872505	−0.857832	3.942490
O	11.310972	−2.465609	1.975883
C	11.394909	−3.699165	2.778971
C	11.962682	−4.819014	1.928126
O	11.012840	−5.170548	0.884052
C	11.658217	−5.121127	−0.414731
C	13.306326	−4.509016	1.229625
C	12.904400	−4.242579	−0.227266
O	14.135724	−5.691490	1.366921
H	10.387479	−3.958097	3.118389
H	12.042314	−3.520455	3.642300
H	12.095886	−5.684841	2.589794
H	11.914150	−6.131111	−0.737469
H	13.809035	−3.647422	1.680797
H	12.665369	−3.183151	−0.349448
H	13.697551	−4.501447	−0.932854
H	14.984151	−5.509590	0.922598
N	10.683350	−4.625334	−1.392333
C	10.120586	−5.520880	−2.357274
O	10.500259	−6.709500	−2.385991
N	9.186678	−5.028754	−3.218137
C	8.782428	−3.753755	−3.139294
N	7.855576	−3.329325	−4.022317
C	9.298344	−2.849249	−2.154690
C	10.236134	−3.336540	−1.303912
H	7.442378	−3.985965	−4.673177
H	7.475509	−2.394144	−3.970857
H	8.951934	−1.825611	−2.086253
H	10.665786	−2.743899	−0.506346
H	12.050622	0.238216	1.319520
H	5.589536	2.547314	−0.239504

(CCG)$^{H+}$

E: −13405.69 kcal mol^{-1}

O	−0.245932	−0.236752	−0.326394
C	−0.210633	0.093369	1.083260
C	1.197183	−0.103568	1.633283
O	1.540961	−1.521793	1.716921
C	2.655869	−1.839569	0.841202
C	2.313297	0.550501	0.798259
C	2.863649	−0.599295	−0.042019
O	3.290151	1.085649	1.771361
H	−1.178660	−0.265191	−0.602492
H	−0.898748	−0.547994	1.647700
H	−0.498661	1.142757	1.239166
H	1.214133	0.302896	2.650131
H	3.535403	−2.071626	1.444103
H	1.961913	1.389682	0.197730
H	2.271212	−0.661384	−0.956762
H	3.905210	−0.466703	−0.325644
N	2.360059	−3.071652	0.101843
C	3.070592	−4.276692	0.428717
O	3.924152	−4.253997	1.333996
N	2.777683	−5.399594	−0.284664
C	1.831668	−5.385806	−1.232622
N	1.616423	−6.524823	−1.923415
C	1.053078	−4.216307	−1.520495
C	1.347598	−3.089888	−0.822060
H	2.152773	−7.355840	−1.705850
H	0.897803	−6.580319	−2.632406
H	0.254583	−4.228808	−2.253458
H	0.790132	−2.166164	−0.938023
P	4.744005	1.662668	1.432816
O	4.752071	1.940995	−0.145293
O	5.100739	2.796781	2.328649
O	5.739099	0.399150	1.514540
C	6.001191	−0.174193	2.844001
C	7.132813	−1.180546	2.749192
O	6.700214	−2.356474	2.015742
C	7.604281	−2.621687	0.906293
C	8.408788	−0.665842	2.046614
C	8.339984	−1.298574	0.659114
O	9.533565	−1.161979	2.859998
H	5.092619	−0.668288	3.200743
H	6.278670	0.630279	3.531724
H	7.382158	−1.475678	3.775377
H	8.291863	−3.426331	1.175596
H	8.474541	0.422241	2.019940
H	7.762778	−0.631109	0.015457
H	9.312061	−1.446691	0.196535
N	6.824131	−3.134744	−0.217874
C	6.831646	−4.548283	−0.485903
O	7.542129	−5.293059	0.211660
N	6.059114	−5.006142	−1.509018
C	5.265624	−4.177397	−2.196359
N	4.518147	−4.698376	−3.189699
C	5.181946	−2.776332	−1.899681
C	5.972165	−2.310523	−0.900541
H	4.532484	−5.696069	−3.359078
H	3.879114	−4.124413	−3.722032
H	4.514032	−2.117712	−2.441664
H	5.959675	−1.274073	−0.588096
P	11.098163	−1.049069	2.547120
O	11.207348	−0.127004	1.240606
O	11.875708	−0.624956	3.744281
O	11.508158	−2.485164	1.955508
C	11.462098	−3.646255	2.858465
C	12.003887	−4.867576	2.136969
O	11.111906	−5.252098	1.052794
C	11.784290	−5.128884	−0.226708
C	13.402208	−4.700999	1.512955
C	13.101460	−4.382428	0.042612
O	14.089460	−5.968432	1.673624
H	10.422410	−3.817936	3.152587
H	12.068055	−3.435034	3.745175
H	12.024065	−5.685664	2.867481
H	11.952285	−6.132765	−0.626435
H	13.972615	−3.901744	1.997833
H	12.974074	−3.304724	−0.071465
H	13.899017	−4.710769	−0.627672
H	14.965271	−5.884193	1.253726
N	10.898245	−4.465024	−1.175766
C	10.665401	−3.099259	−1.334872
N	9.751688	−2.841616	−2.251087
C	9.347460	−4.090589	−2.720734
C	8.402855	−4.441938	−3.733287
O	7.702888	−3.701943	−4.447380
N	8.322701	−5.853084	−3.881167
C	9.044208	−6.783868	−3.164399
N	8.786514	−8.097134	−3.410251
N	9.932729	−6.445328	−2.234514

C	10.044084	−5.110555	−2.057789
H	11.196683	−2.361933	−0.751781
H	7.652657	−6.176243	−4.575631
H	8.339014	−8.365095	−4.278951
H	9.445292	−8.764317	−3.024723
H	11.969535	0.483555	1.269617
H	5.095180	2.827973	−0.369354

(CCT)$^{H+}$

E: −13101.95 kcal mol^{-1}

O	−0.195780	−0.087986	−0.394511
C	−0.213572	0.237998	1.016566
C	1.167094	0.019020	1.624888
O	1.488335	−1.404415	1.716017
C	2.627253	−1.732696	0.875064
C	2.326232	0.657637	0.839034
C	2.871046	−0.493803	−0.000040
O	3.282986	1.151752	1.856423
H	−1.117314	−0.109386	−0.706632
H	−0.933118	−0.395141	1.550113
H	−0.493055	1.290885	1.163901
H	1.147055	0.420861	2.643374
H	3.486000	−1.969598	1.505198
H	2.020067	1.518390	0.244235
H	2.287435	−0.539981	−0.921690
H	3.917044	−0.373363	−0.270173
N	2.345629	−2.964154	0.130463
C	3.049207	−4.169384	0.473153
O	3.888479	−4.143808	1.391581
N	2.767152	−5.293883	−0.241564
C	1.841011	−5.280456	−1.209253
N	1.636597	−6.421231	−1.900178
C	1.072842	−4.109288	−1.518132
C	1.354621	−2.981621	−0.816489
H	2.168111	−7.252324	−1.670874
H	0.933738	−6.476871	−2.624846
H	0.292041	−4.121603	−2.269996
H	0.804423	−2.055612	−0.948493
P	4.795754	1.602909	1.633120
O	4.871762	1.949677	0.070624
O	5.201329	2.659715	2.599227
O	5.693098	0.263868	1.716479
C	5.963388	−0.301750	3.048987
C	7.138924	−1.258632	2.968515
O	6.767071	−2.458598	2.243173
C	7.651054	−2.689405	1.117906
C	8.396648	−0.684697	2.280115
C	8.432099	−1.379440	0.916254
O	9.530978	−1.016397	3.158750
H	5.070173	−0.833760	3.388698
H	6.196712	0.511526	3.741894
H	7.391498	−1.538275	3.998186
H	8.310656	−3.532261	1.331202
H	8.366964	0.401547	2.189260
H	7.943375	−0.727758	0.189986
H	9.444603	−1.568796	0.565859
N	6.845555	−3.108495	−0.035541
C	6.829798	−4.494664	−0.417120
O	7.543466	−5.302769	0.203427
N	6.042022	−4.858262	−1.465641
C	5.263255	−3.964767	−2.085649
N	4.500389	−4.393009	−3.109627
C	5.208764	−2.589970	−1.680826
C	6.003316	−2.219647	−0.645954
H	4.512248	−5.370761	−3.371609
H	3.881146	−3.764552	−3.602504
H	4.554110	−1.879792	−2.170268
H	6.001422	−1.214117	−0.244351
P	11.084884	−0.818070	2.802717
O	11.154410	0.197463	1.564692
O	11.862176	−0.465154	4.022086
O	11.525896	−2.175173	2.066448
C	11.489701	−3.413839	2.862886
C	11.854825	−4.584024	1.975948
O	10.804381	−4.768427	0.988937
C	11.417575	−5.091754	−0.281112
C	13.196187	−4.459652	1.203639
C	12.766491	−4.358524	−0.269599
O	13.955303	−5.662597	1.484684
H	10.480556	−3.547268	3.263821
H	12.204922	−3.328546	3.686371
H	11.909873	−5.473136	2.619042
H	11.547622	−6.171808	−0.375240
H	13.765533	−3.581016	1.520658
H	12.645050	−3.306949	−0.542901
H	13.492525	−4.813566	−0.947324
H	14.828073	−5.566150	1.061322
N	10.487359	−4.702487	−1.342825
C	9.834644	−5.707741	−2.066320
O	10.020839	−6.915648	−1.890448
N	8.960926	−5.227787	−3.023810
C	8.636832	−3.895078	−3.310667
O	7.831696	−3.633813	−4.217021
C	9.308775	−2.914732	−2.470249
C	9.015418	−1.455986	−2.683865
C	10.188143	−3.367176	−1.542248
H	8.474322	−5.939999	−3.564858
H	9.275698	−1.155542	−3.705910
H	9.583929	−0.835340	−1.986682
H	7.946954	−1.249813	−2.552605
H	10.718272	−2.686699	−0.887714
H	11.610168	1.031907	1.789062
H	5.644684	2.495337	−0.174220

(CGA)$^{H+}$

E: −13807.36 kcal mol^{-1}

O	−0.229202	−0.468322	−0.309637
C	−0.206036	−0.086473	1.086997
C	1.214904	−0.171064	1.632007
O	1.650325	−1.559478	1.767035
C	2.785258	−1.837204	0.902017
C	2.280064	0.523372	0.763147
C	2.912024	−0.618679	−0.026913
O	3.215361	1.174788	1.707160
H	−1.160054	−0.560984	−0.577745
H	−0.847134	−0.750668	1.679541
H	−0.560870	0.946939	1.208686
H	1.210840	0.275357	2.632200
H	3.677469	−1.988477	1.511866
H	1.867661	1.307369	0.127784
H	2.334661	−0.758137	−0.943137
H	3.943489	−0.424258	−0.308743
N	2.570230	−3.111703	0.207197
C	3.365910	−4.253637	0.561767
O	4.206156	−4.151779	1.474089
N	3.164372	−5.409058	−0.131922
C	2.212661	−5.489990	−1.069846
N	2.066082	−6.667538	−1.718139
C	1.338263	−4.393941	−1.370475
C	1.554316	−3.229780	−0.705202
H	2.775761	−7.381227	−1.602352
H	1.450177	−6.737870	−2.517708

H	0.535051	−4.486510	−2.092501
H	0.929150	−2.353094	−0.839847
P	4.616330	1.861093	1.366300
O	4.585447	2.062765	−0.222765
O	4.862016	3.062767	2.210407
O	5.727341	0.704973	1.528913
C	6.039208	0.241633	2.887879
C	7.154642	−0.788313	2.840246
O	6.710084	−1.980455	2.133072
C	7.586396	−2.234161	1.002639
C	8.456081	−0.340433	2.142168
C	8.300570	−0.908633	0.735765
O	9.549056	−0.959927	2.926293
H	5.142961	−0.216424	3.317247
H	6.345547	1.095725	3.499542
H	7.376563	−1.059302	3.878805
H	8.293288	−3.026061	1.266603
H	8.617466	0.737523	2.167111
H	7.654377	−0.227816	0.176252
H	9.232951	−1.022698	0.186720
N	6.823132	−2.764671	−0.104173
C	5.978710	−2.119847	−1.003594
N	5.441004	−2.952212	−1.877123
C	5.955643	−4.208482	−1.549788
C	5.766313	−5.493436	−2.147468
O	5.045120	−5.822601	−3.101556
N	6.587427	−6.473569	−1.507117
C	7.447078	−6.247794	−0.451603
N	8.191600	−7.289906	0.020521
N	7.561177	−5.060791	0.129452
C	6.817242	−4.103344	−0.453145
H	5.823058	−1.052190	−0.967797
H	6.529213	−7.407280	−1.907670
H	8.328747	−8.089333	−0.588640
H	9.027397	−6.990924	0.524929
P	11.023539	−1.380058	2.477490
O	11.381863	−0.474404	1.202806
O	11.961834	−1.335391	3.631115
O	10.892210	−2.819319	1.768656
C	10.740818	−4.030028	2.597195
C	11.465566	−5.208094	1.959218
O	10.768573	−5.703660	0.779999
C	11.513604	−5.397772	−0.427121
C	12.906560	−4.923888	1.501522
C	12.747368	−4.587150	0.010567
O	13.662799	−6.142327	1.711995
H	9.673142	−4.248544	2.681765
H	11.163005	−3.840740	3.588024
H	11.458997	−6.016153	2.699553
H	11.778373	−6.342862	−0.906751
H	13.358435	−4.098376	2.061770
H	12.576523	−3.515792	−0.105656
H	13.631834	−4.857892	−0.570121
H	14.565333	−5.992027	1.375691
N	10.644440	−4.704631	−1.375678
C	10.241249	−3.374548	−1.359452
N	9.345947	−3.089786	−2.289206
C	9.130578	−4.295683	−2.955191
C	8.276413	−4.663644	−4.019406
N	7.445950	−3.785023	−4.636170
N	8.306121	−5.952127	−4.447284
C	9.125782	−6.825081	−3.830446
N	9.958361	−6.597082	−2.797527
C	9.926093	−5.312197	−2.400411
H	10.638697	−2.678086	−0.637535
H	6.712938	−4.170238	−5.222159
H	7.233165	−2.914096	−4.162814
H	9.104973	−7.844366	−4.212822
H	12.236820	−0.010920	1.296279
H	5.180788	2.771598	−0.535846

(CGC)$^{H+}$

E: −13407.70 kcal mol^{-1}

O	−0.279540	−0.455878	−0.278040
C	−0.227243	−0.049296	1.110602
C	1.201210	−0.142251	1.634314
O	1.629300	−1.534369	1.763344
C	2.746204	−1.813695	0.872797
C	2.260330	0.538534	0.748163
C	2.829030	−0.609779	−0.076024
O	3.242484	1.136112	1.682823
H	−1.216291	−0.536207	−0.529252
H	−0.865734	−0.695329	1.725762
H	−0.567786	0.990209	1.220527
H	1.215214	0.305821	2.633576
H	3.655801	−1.938684	1.462972
H	1.858719	1.350941	0.142204
H	2.182671	−0.747270	−0.945194
H	3.837893	−0.433326	−0.436695
N	2.535692	−3.103449	0.210837
C	3.351434	−4.225670	0.583022
O	4.201943	−4.088375	1.480786
N	3.156677	−5.400568	−0.078997
C	2.190772	−5.518264	−0.999415
N	2.051992	−6.712062	−1.614040
C	1.301619	−4.439176	−1.320073
C	1.510276	−3.256498	−0.686009
H	2.718915	−7.450921	−1.426997
H	1.383948	−6.840334	−2.362247
H	0.490487	−4.560264	−2.028827
H	0.872328	−2.391335	−0.833651
P	4.689083	1.723105	1.357908
O	4.720434	1.883326	−0.236585
O	4.995225	2.928759	2.176046
O	5.721064	0.502686	1.574697
C	5.998822	0.063987	2.949043
C	7.185976	−0.885630	2.962232
O	6.867093	−2.133766	2.287645
C	7.659513	−2.282306	1.084223
C	8.466710	−0.357993	2.288013
C	8.387827	−0.945576	0.882026
O	9.586401	−0.867461	3.106646
H	5.115216	−0.452010	3.336365
H	6.218166	0.938087	3.569858
H	7.391921	−1.114169	4.013505
H	8.352619	−3.117141	1.225124
H	8.543619	0.729800	2.293666
H	7.787384	−0.262073	0.279431
H	9.354577	−1.057441	0.395683
N	6.815683	−2.670157	−0.034445
C	6.010115	−1.876663	−0.850979
N	5.402748	−2.573764	−1.792724
C	5.821475	−3.890110	−1.600586
C	5.545075	−5.078805	−2.343641
O	4.817471	−5.233297	−3.339321
N	6.265324	−6.186593	−1.811766
C	7.129532	−6.147538	−0.737413
N	7.750997	−7.304207	−0.373587
N	7.355116	−5.041291	−0.039931
C	6.696435	−3.965714	−0.510567
H	5.922661	−0.812335	−0.696055
H	6.134137	−7.064573	−2.309882
H	7.830617	−8.053881	−1.051246
H	8.551880	−7.182224	0.239244
P	11.089832	−1.187565	2.684256

O	11.369981	−0.179212	1.469914
O	11.998958	−1.125980	3.860135
O	11.076588	−2.614086	1.935027
C	10.992657	−3.839853	2.754031
C	11.541524	−5.019222	1.971106
O	10.658970	−5.338321	0.859482
C	11.393946	−5.307462	−0.390487
C	12.953839	−4.819131	1.379165
C	12.685752	−4.526139	−0.102946
O	13.672514	−6.064057	1.575118
H	9.941781	−4.007490	3.006542
H	11.575309	−3.698619	3.668550
H	11.558032	−5.874589	2.658416
H	11.595562	−6.326143	−0.722794
H	13.488169	−3.998840	1.870044
H	12.537436	−3.452123	−0.241681
H	13.508410	−4.846423	−0.746791
H	14.559944	−5.956885	1.186094
N	10.526274	−4.720803	−1.419659
C	10.015766	−5.543415	−2.474550
O	10.374338	−6.734806	−2.553459
N	9.143407	−4.980075	−3.357064
C	8.788193	−3.692822	−3.249114
N	7.936047	−3.195333	−4.171346
C	9.284801	−2.849955	−2.202142
C	10.132196	−3.416196	−1.306190
H	7.504432	−3.827368	−4.835449
H	7.518807	−2.282508	−4.045238
H	8.980600	−1.814285	−2.115093
H	10.524117	−2.878987	−0.451501
H	12.313123	−0.105439	1.224846
H	5.326176	2.586996	−0.541440

$(CGG)^{H+}$

E: −13971.82 kcal mol^{-1}

O	−0.179730	−0.599873	−0.376898
C	−0.176294	−0.176919	1.008012
C	1.239253	−0.230013	1.570983
O	1.686919	−1.608748	1.757101
C	2.835387	−1.903343	0.915891
C	2.306513	0.446230	0.690780
C	2.962238	−0.714797	−0.051029
O	3.222424	1.142881	1.621893
H	−1.106643	−0.706012	−0.653662
H	−0.817648	−0.829690	1.612883
H	−0.542580	0.856006	1.094965
H	1.219881	0.249363	2.555616
H	3.721696	−2.026573	1.540427
H	1.892404	1.201873	0.023049
H	2.401342	−0.892541	−0.970956
H	3.995996	−0.519198	−0.322437
N	2.640131	−3.201960	0.260113
C	3.454986	−4.319982	0.645380
O	4.304448	−4.175284	1.542949
N	3.260190	−5.502576	−0.004082
C	2.300183	−5.628423	−0.928694
N	2.157139	−6.831761	−1.526796
C	1.416347	−4.551085	−1.267504
C	1.622853	−3.362214	−0.644231
H	2.854150	−7.548714	−1.365434
H	1.521222	−6.949351	−2.304475
H	0.611009	−4.677272	−1.981987
H	0.988519	−2.496821	−0.807074
P	4.617925	1.833976	1.267343
O	4.584827	2.004143	−0.325301
O	4.852174	3.053732	2.088532
O	5.739890	0.691749	1.451440
C	6.048040	0.253156	2.819202
C	7.176899	−0.762873	2.795435
O	6.756573	−1.971530	2.103542
C	7.638383	−2.226826	0.976969
C	8.478233	−0.307100	2.103238
C	8.349679	−0.900283	0.704988
O	9.572961	−0.886827	2.916503
H	5.154576	−0.209081	3.249790
H	6.339676	1.120398	3.419507
H	7.392951	−1.014847	3.840021
H	8.346293	−3.015477	1.246577
H	8.622934	0.773428	2.110884
H	7.710022	−0.230827	0.124553
H	9.291054	−1.018988	0.173336
N	6.878688	−2.765438	−0.129388
C	6.032055	−2.127902	−1.033744
N	5.479861	−2.968978	−1.888128
C	5.985945	−4.223945	−1.545260
C	5.788635	−5.515028	−2.125854
O	5.079394	−5.850932	−3.088071
N	6.588708	−6.496067	−1.461563
C	7.440245	−6.265325	−0.399680
N	8.159950	−7.311430	0.102108
N	7.573634	−5.068936	0.155199
C	6.851964	−4.110250	−0.451840
H	5.887393	−1.058182	−1.014888
H	6.521652	−7.435405	−1.848408
H	8.278881	−8.129044	−0.486505
H	9.007136	−7.012548	0.587661
P	11.035209	−1.372649	2.516135
O	11.428503	−0.468428	1.249672
O	11.948891	−1.345165	3.689798
O	10.886112	−2.821193	1.828572
C	10.710019	−4.024602	2.660510
C	11.400645	−5.221123	2.019285
O	10.714606	−5.666876	0.815075
C	11.491296	−5.351877	−0.369304
C	12.863928	−4.993309	1.603219
C	12.760770	−4.626068	0.114395
O	13.561772	−6.246127	1.813533
H	9.637390	−4.216543	2.748537
H	11.139328	−3.844363	3.649960
H	11.340838	−6.040099	2.745301
H	11.711099	−6.292593	−0.878953
H	13.336333	−4.197401	2.189025
H	12.661790	−3.544956	0.011553
H	13.641508	−4.941887	−0.448904
H	14.478655	−6.129129	1.504232
N	10.681443	−4.575001	−1.307759
C	10.434890	−3.201328	−1.317294
N	9.584555	−2.841377	−2.258426
C	9.234390	−4.026642	−2.902710
C	8.350119	−4.261230	−4.000090
O	7.686817	−3.448264	−4.666778
N	8.276184	−5.650668	−4.299016
C	8.968186	−6.654550	−3.656224
N	8.704442	−7.935221	−4.034750
N	9.814146	−6.420721	−2.658055
C	9.902134	−5.114167	−2.321269
H	10.905738	−2.537707	−0.610240
H	7.630218	−5.895734	−5.046606
H	8.286550	−8.112270	−4.940933
H	9.345790	−8.643950	−3.696692
H	12.391958	−0.348076	1.140550
H	5.170661	2.714257	−0.652790

$(CGT)^{H+}$

E: −13666.89 kcal mol^{-1}

C	−5.864723	−1.825463	−0.681781
O	−5.096828	−0.887204	−1.504185
C	−3.931445	−0.415701	−0.782318
C	−4.989330	−2.105082	0.552105
C	−4.138619	−0.846824	0.675996
O	−4.197339	−3.314476	0.227583
H	−8.029176	0.250418	0.618149
H	−7.720959	−0.957279	−1.306231
H	−7.851733	−2.085720	0.071141
H	−5.995062	−2.744866	−1.262176
H	−3.030563	−0.854319	−1.216155
H	−5.567965	−2.328824	1.448778
H	−4.716078	−0.103785	1.229369
H	−3.201236	−1.000973	1.200742
N	−3.790052	1.034158	−0.980210
C	−2.728571	1.496734	−1.768083
O	−1.897912	0.762703	−2.306172
N	−2.679504	2.877226	−1.880124
C	−3.584288	3.820527	−1.371640
O	−3.406371	5.028673	−1.598548
C	−4.688412	3.251636	−0.614456
C	−5.731860	4.174150	−0.045714
C	−4.742854	1.902606	−0.470529
H	−1.919516	3.238741	−2.452959
H	−6.211965	4.758760	−0.840446
H	−5.279186	4.889555	0.652216
H	−6.501646	3.607318	0.486118
H	−5.567360	1.415833	0.041215
P	−2.951477	−3.906848	1.029199
O	−3.038381	−3.202293	2.465279
O	−2.925994	−5.393924	0.985569
O	−1.636551	−3.211183	0.406103
C	−1.175652	−3.661467	−0.915010
C	0.214296	−3.116775	−1.202319
O	0.191191	−1.670743	−1.357773
C	1.282709	−3.411576	−0.133309
C	1.308166	−2.134134	0.702291
O	2.529164	−3.683953	−0.877640
H	−1.873245	−3.290616	−1.672253
H	−1.156129	−4.755419	−0.936556
H	0.525423	−3.552766	−2.158318
H	1.812814	−0.538943	−0.730157
H	1.067900	−4.302089	0.458910
H	0.544449	−2.239265	1.474673
H	2.258918	−1.952420	1.199481
N	0.148059	0.048349	0.295707
C	−0.830352	−0.027379	1.288767
N	−1.332852	1.151952	1.598378
C	−0.662549	2.060168	0.777796
C	−0.752706	3.483138	0.695234
O	−1.482145	4.263648	1.330871
N	0.156634	3.991884	−0.275183
C	1.046507	3.242015	−1.023834
N	1.870593	3.899702	−1.865025
N	1.112045	1.914178	−0.940477
C	0.260534	1.390154	−0.039800
H	−1.117675	−0.969600	1.729841
H	0.165208	5.005835	−0.366773
H	1.862932	4.906136	−1.954757
H	2.549743	3.363515	−2.389147
P	4.036868	−3.536136	−0.377745
O	3.938291	−3.777452	1.202814
O	4.945335	−4.417669	−1.159221
O	4.401538	−1.970234	−0.458283
C	4.645065	−1.390100	−1.792332
C	5.292272	−0.027335	−1.643502
O	4.344869	0.898646	−1.044522
C	4.979751	1.598876	0.054206
C	6.576316	0.010518	−0.780343
C	6.114749	0.676090	0.522783
O	7.543758	0.816849	−1.502581
H	3.684236	−1.298226	−2.308408
H	5.303994	−2.055738	−2.357611
H	5.538223	0.319988	−2.656168
H	5.351429	2.568662	−0.280684
H	6.981796	−0.992193	−0.609504
H	5.736524	−0.087891	1.208706
H	6.917524	1.225419	1.021265
H	8.358617	0.847557	−0.968243
N	3.954553	1.890848	1.060610
C	3.602858	3.248037	1.351874
O	4.237141	4.176023	0.811536
N	2.575508	3.467433	2.220550
C	1.943847	2.444153	2.813964
N	0.948586	2.735355	3.678421
C	2.296822	1.078000	2.559288
C	3.292720	0.857313	1.663724
H	0.638588	3.695615	3.770853
H	0.350667	2.004055	4.040380
H	1.775523	0.257280	3.037038
H	3.603924	−0.136945	1.366473
H	−2.554743	−3.682145	3.165921
H	4.803708	−3.895807	1.641461
C	0.934890	−1.026188	−0.294344
O	−7.150444	−0.163107	0.555129
C	−7.251141	−1.275676	−0.367178

(CTA)$^{H+}$

E: −13501.39 kcal mol^{-1}

O	−0.239612	−0.154085	−0.055032
C	−0.151800	0.090066	1.369784
C	1.282880	−0.106491	1.844832
O	1.647416	−1.521535	1.866773
C	2.726690	−1.804065	0.940562
C	2.345365	0.597085	0.978401
C	2.923945	−0.522560	0.112446
O	3.318283	1.192161	1.917614
H	−1.182165	−0.186987	−0.295340
H	−0.802360	−0.599447	1.921526
H	−0.453597	1.121438	1.601059
H	1.345390	0.265617	2.872887
H	3.623315	−2.081390	1.497307
H	1.932789	1.413112	0.385108
H	2.351902	−0.561853	−0.816325
H	3.969840	−0.366184	−0.144362
N	2.387087	−2.990472	0.141075
C	3.105178	−4.216066	0.349544
O	4.011603	−4.255716	1.202405
N	2.769397	−5.288522	−0.419274
C	1.769443	−5.213019	−1.308383
N	1.501445	−6.307696	−2.047984
C	0.982528	−4.025461	−1.474801
C	1.323083	−2.947515	−0.723550
H	2.083679	−7.131325	−1.957033
H	0.777771	−6.298578	−2.753978
H	0.146022	−3.987630	−2.162987
H	0.767855	−2.015354	−0.750419
P	4.718503	1.859352	1.509535
O	4.658283	2.155434	−0.064201
O	5.047355	2.999090	2.408624
O	5.778664	0.649036	1.514661
C	6.048346	0.009142	2.810844

C	7.078471	−1.083361	2.622657
O	6.496540	−2.174843	1.859056
C	7.443408	−2.600906	0.848632
C	8.368367	−0.654041	1.877776
C	8.252402	−1.341895	0.518909
O	9.482787	−1.171425	2.687626
H	5.118904	−0.417764	3.199094
H	6.428342	0.760932	3.509109
H	7.352461	−1.446779	3.620590
H	8.081463	−3.399053	1.236681
H	8.478650	0.427508	1.803065
H	7.698575	−0.680084	−0.151701
H	9.210306	−1.565457	0.056160
N	6.702814	−3.191876	−0.264327
C	6.745281	−4.584922	−0.423666
O	7.409953	−5.336547	0.290487
N	5.992816	−5.046293	−1.488228
C	5.171523	−4.311253	−2.353415
O	4.564957	−4.882777	−3.268870
C	5.123182	−2.882509	−2.070548
C	4.255981	−2.009529	−2.933184
C	5.879857	−2.405772	−1.051746
H	6.027992	−6.051766	−1.643171
H	4.544341	−2.101189	−3.986942
H	4.340856	−0.960115	−2.641325
H	3.204877	−2.312884	−2.862944
H	5.880832	−1.354267	−0.793071
P	11.049341	−1.036683	2.382416
O	11.149095	−0.197700	1.020784
O	11.810711	−0.520647	3.553367
O	11.488417	−2.497265	1.881278
C	11.422916	−3.617917	2.832257
C	11.971627	−4.870400	2.171405
O	11.093794	−5.300825	1.091786
C	11.779495	−5.221263	−0.183127
C	13.377957	−4.734581	1.559037
C	13.097488	−4.472736	0.073090
O	14.057167	−5.996888	1.777992
H	10.377916	−3.774137	3.115780
H	12.014936	−3.371409	3.719229
H	11.978102	−5.656769	2.935874
H	11.944058	−6.238170	−0.550046
H	13.945815	−3.918804	2.018541
H	12.979317	−3.399454	−0.084512
H	13.901043	−4.832032	−0.573571
H	14.938405	−5.933191	1.366082
N	10.901684	−4.584461	−1.160375
C	10.679032	−3.229524	−1.373704
N	9.749678	−2.993283	−2.285279
C	9.325869	−4.256429	−2.695471
C	8.363813	−4.684736	−3.639500
N	7.628120	−3.826423	−4.377113
N	8.173665	−6.021443	−3.797336
C	8.910236	−6.875000	−3.058796
N	9.853129	−6.585212	−2.143592
C	10.023124	−5.258022	−2.002009
H	11.225273	−2.472009	−0.831110
H	6.909627	−4.180378	−4.995966
H	7.732687	−2.827324	−4.263033
H	8.713951	−7.932804	−3.226318
H	11.867690	0.464254	1.035182
H	4.698550	3.110145	−0.267816

(CTC)$^{\mathrm{H+}}$

E: −13102.18 kcal mol^{-1}

O	−0.224281	−0.125789	−0.089963
C	−0.158541	0.143502	1.331741
C	1.268984	−0.041342	1.832179
O	1.637894	−1.455158	1.873452
C	2.735204	−1.740448	0.969454
C	2.343578	0.657133	0.976352
C	2.933133	−0.468543	0.127708
O	3.305194	1.256832	1.924387
H	−1.162989	−0.169324	−0.342987
H	−0.815307	−0.538621	1.885331
H	−0.466845	1.177687	1.540552
H	1.312839	0.341208	2.857373
H	3.624539	−2.001730	1.545620
H	1.939976	1.470137	0.372699
H	2.366534	−0.521111	−0.803686
H	3.978951	−0.309547	−0.126015
N	2.421711	−2.939081	0.178331
C	3.151856	−4.153520	0.409097
O	4.033711	−4.178796	1.287786
N	2.854595	−5.231307	−0.368241
C	1.873655	−5.173808	−1.279593
N	1.643628	−6.272233	−2.026330
C	1.068098	−4.001133	−1.463055
C	1.375344	−2.915637	−0.708545
H	2.234658	−7.087401	−1.918112
H	0.932159	−6.277880	−2.744609
H	0.244189	−3.979950	−2.166993
H	0.805901	−1.992611	−0.749492
P	4.723024	1.900081	1.541802
O	4.712606	2.144588	−0.041799
O	5.034376	3.067075	2.411701
O	5.772771	0.682184	1.624912
C	6.004918	0.091603	2.951952
C	7.049610	−0.998401	2.834827
O	6.502363	−2.112427	2.078187
C	7.473028	−2.552489	1.100168
C	8.363344	−0.576630	2.130458
C	8.312988	−1.306816	0.788652
O	9.458486	−1.030689	3.003633
H	5.066362	−0.329063	3.324291
H	6.356475	0.870939	3.634706
H	7.286329	−1.334264	3.851710
H	8.085807	−3.361849	1.504700
H	8.457095	0.503429	2.019702
H	7.810695	−0.659875	0.065715
H	9.297201	−1.553165	0.398446
N	6.759122	−3.129372	−0.039414
C	6.812528	−4.518799	−0.224083
O	7.440496	−5.284104	0.507769
N	6.119532	−4.959403	−1.337131
C	5.332432	−4.210352	−2.220364
O	4.768519	−4.763983	−3.173589
C	5.263271	−2.787688	−1.909405
C	4.429675	−1.903703	−2.793607
C	5.969727	−2.329310	−0.846912
H	6.161062	−5.962845	−1.504817
H	4.753666	−1.988096	−3.837587
H	4.506438	−0.857101	−2.490359
H	3.375804	−2.204906	−2.763534
H	5.952667	−1.283848	−0.564172
P	11.024364	−0.967350	2.653197
O	11.152824	0.016785	1.395077
O	11.832437	−0.647645	3.861386
O	11.361800	−2.375287	1.956436
C	11.331656	−3.581535	2.802681
C	11.806969	−4.772130	1.992984
O	10.829636	−5.071597	0.958987
C	11.493325	−5.182670	−0.326549
C	13.173980	−4.598014	1.289685
C	12.800302	−4.389056	−0.185056

O	13.916997	−5.825237	1.505632
H	10.304854	−3.744265	3.143520
H	11.987679	−3.428448	3.664734
H	11.869749	−5.624418	2.682283
H	11.678923	−6.232462	−0.557694
H	13.733675	−3.748673	1.693935
H	12.643582	−3.323314	−0.369786
H	13.575151	−4.748777	−0.866282
H	14.786318	−5.722975	1.076439
N	10.565193	−4.707250	−1.357213
C	9.913928	−5.653976	−2.214902
O	10.182178	−6.866642	−2.104427
N	9.024187	−5.178953	−3.130692
C	8.739678	−3.871428	−3.200733
N	7.851573	−3.468379	−4.131460
C	9.329307	−2.912935	−2.312828
C	10.230883	−3.382877	−1.413582
H	7.433333	−4.145231	−4.758039
H	7.622927	−2.490770	−4.247308
H	9.073219	−1.861320	−2.357576
H	10.722458	−2.741122	−0.693566
H	11.806474	0.728667	1.537105
H	4.831834	3.085292	−0.277052

(CTG)$^{H+}$

E: −13665.46 kcal mol^{-1}

O	−0.211646	−0.209004	−0.088961
C	−0.133381	0.036797	1.336070
C	1.299257	−0.151740	1.820042
O	1.671913	−1.564518	1.845186
C	2.760350	−1.841089	0.927965
C	2.364119	0.557799	0.961112
C	2.956850	−0.558969	0.100437
O	3.324115	1.160280	1.908714
H	−1.152568	−0.244085	−0.335209
H	−0.783610	−0.655618	1.884664
H	−0.441876	1.066776	1.564823
H	1.352717	0.221265	2.848300
H	3.654062	−2.113255	1.491933
H	1.951056	1.370762	0.363840
H	2.392235	−0.602619	−0.832896
H	4.003950	−0.396992	−0.148701
N	2.433291	−3.029357	0.126143
C	3.158453	−4.250180	0.337202
O	4.059655	−4.285191	1.195770
N	2.834831	−5.323560	−0.435693
C	1.837361	−5.254197	−1.328059
N	1.580047	−6.350086	−2.070181
C	1.041572	−4.072731	−1.495157
C	1.372067	−2.992996	−0.741932
H	2.174340	−7.165611	−1.984626
H	0.863962	−6.342322	−2.783964
H	0.206628	−4.040543	−2.185494
H	0.810489	−2.064676	−0.769937
P	4.727331	1.830078	1.515838
O	4.685701	2.129490	−0.057886
O	5.044245	2.968306	2.421093
O	5.788723	0.621475	1.528085
C	6.050463	−0.020675	2.824757
C	7.096499	−1.100004	2.645427
O	6.535713	−2.202740	1.880373
C	7.487730	−2.597808	0.860113
C	8.382230	−0.653841	1.904708
C	8.257863	−1.314621	0.534059
O	9.500359	−1.197253	2.692807
H	5.121550	−0.460320	3.199986
H	6.412692	0.732980	3.530367
H	7.369386	−1.457312	3.645751
H	8.152225	−3.376486	1.243644
H	8.494179	0.429054	1.854658
H	7.672840	−0.652954	−0.109629
H	9.211185	−1.505070	0.048132
N	6.761093	−3.211460	−0.248596
C	6.815252	−4.606659	−0.378417
O	7.466423	−5.340800	0.365765
N	6.089752	−5.094459	−1.450607
C	5.270086	−4.383512	−2.340229
O	4.684031	−4.981247	−3.251616
C	5.197382	−2.952380	−2.077949
C	4.324968	−2.103532	−2.958848
C	5.935927	−2.449845	−1.058420
H	6.104987	−6.106319	−1.559836
H	4.635523	−2.192943	−4.006371
H	4.380603	−1.051299	−2.668844
H	3.279879	−2.430198	−2.903752
H	5.919376	−1.394205	−0.818277
P	11.067416	−1.023989	2.412959
O	11.165229	−0.182977	1.052495
O	11.802566	−0.488046	3.592005
O	11.545768	−2.475091	1.921431
C	11.469171	−3.603306	2.861549
C	11.983313	−4.859786	2.180269
O	11.088510	−5.253326	1.100709
C	11.767281	−5.172335	−0.177877
C	13.389264	−4.750995	1.561651
C	13.107781	−4.464220	0.080825
O	14.038745	−6.032300	1.761878
H	10.424947	−3.742730	3.156399
H	12.076925	−3.377522	3.743567
H	11.974920	−5.656381	2.934064
H	11.901376	−6.187526	−0.561429
H	13.979567	−3.955319	2.028196
H	13.018677	−3.386443	−0.064456
H	13.897827	−4.838135	−0.574318
H	14.919917	−5.984120	1.347774
N	10.904966	−4.493325	−1.140637
C	10.728646	−3.122668	−1.330375
N	9.824519	−2.847785	−2.250856
C	9.367955	−4.088945	−2.692051
C	8.410660	−4.421161	−3.699352
O	7.736163	−3.668350	−4.422767
N	8.268621	−5.832474	−3.814594
C	8.960368	−6.778419	−3.084323
N	8.650975	−8.082933	−3.305041
N	9.860319	−6.455592	−2.159253
C	10.024471	−5.122796	−2.008493
H	11.286471	−2.393545	−0.762148
H	7.593715	−6.141495	−4.511386
H	8.180344	−8.351226	−4.161230
H	9.274558	−8.772022	−2.900179
H	11.882646	0.480199	1.066568
H	4.700873	3.085725	−0.258060

(CTT)$^{H+}$

E: −13362.28 kcal mol^{-1}

O	0.084982	−0.036208	−0.321417
C	−0.031916	0.201515	1.102296
C	1.303428	−0.066270	1.786247
O	1.593932	−1.496384	1.847399
C	2.788176	−1.825692	1.092499
C	2.515250	0.586732	1.096396
C	3.165125	−0.554216	0.311333

O	3.371235	1.116222	2.177508
H	−0.810619	−0.017986	−0.701865
H	−0.791247	−0.455421	1.544117
H	−0.314768	1.246307	1.294285
H	1.227748	0.299998	2.815564
H	3.578919	−2.139075	1.774987
H	2.234632	1.426783	0.461266
H	2.736884	−0.566372	−0.693034
H	4.245367	−0.444511	0.222618
N	2.514257	−2.999830	0.250589
C	3.142247	−4.253216	0.557274
O	3.954505	−4.316729	1.499544
N	2.831665	−5.327840	−0.219118
C	1.944035	−5.223501	−1.217927
N	1.688766	−6.327181	−1.947866
C	1.260533	−3.996554	−1.507532
C	1.568644	−2.920346	−0.739763
H	2.204839	−7.179587	−1.767218
H	1.054959	−6.299358	−2.735123
H	0.517503	−3.930078	−2.293918
H	1.073380	−1.960875	−0.850546
P	4.767049	1.873721	1.945001
O	4.813942	2.311217	0.403054
O	4.966933	2.933676	2.970965
O	5.887724	0.723387	1.911590
C	6.173479	0.009095	3.162485
C	7.108416	−1.140872	2.860679
O	6.411588	−2.107454	2.043380
C	7.399011	−2.820693	1.270076
C	8.411523	−0.770629	2.096536
C	8.479267	−1.776646	0.936659
O	9.509351	−0.879605	3.068178
H	5.238917	−0.370835	3.585667
H	6.643813	0.699299	3.868687
H	7.395255	−1.598571	3.818125
H	7.807994	−3.660853	1.836021
H	8.398659	0.258252	1.737801
H	8.246229	−1.263953	0.001647
H	9.456292	−2.245713	0.840692
N	6.730542	−3.398081	0.104890
C	6.667665	−4.791742	−0.021581
O	7.190708	−5.578123	0.768253
N	5.992237	−5.212974	−1.153765
C	5.310495	−4.432299	−2.100748
O	4.734944	−4.975836	−3.050840
C	5.365081	−2.996831	−1.852422
C	4.661136	−2.072376	−2.803851
C	6.065502	−2.562519	−0.777124
H	5.921408	−6.222632	−1.265755
H	5.068303	−2.185734	−3.815085
H	4.777175	−1.030312	−2.494545
H	3.591795	−2.308052	−2.854119
H	6.139673	−1.508728	−0.536719
P	11.062870	−0.679940	2.694068
O	11.131972	0.264278	1.400522
O	11.821592	−0.259762	3.902609
O	11.534291	−2.045708	1.999562
C	11.454713	−3.294610	2.772872
C	11.710284	−4.458431	1.839672
O	10.621274	−4.544083	0.878828
C	11.183527	−4.949493	−0.393584
C	13.034464	−4.393664	1.027639
C	12.568295	−4.291869	−0.435851
O	13.757621	−5.617642	1.305329
H	10.459898	−3.377437	3.220067
H	12.210934	−3.273234	3.562983
H	11.727755	−5.370759	2.451542
H	11.254211	−6.038141	−0.446283
H	13.642735	−3.531774	1.316493
H	12.494454	−3.239715	−0.723785
H	13.249144	−4.796552	−1.125022
H	14.633735	−5.545177	0.884218
N	10.246512	−4.553018	−1.447629
C	9.542927	−5.553605	−2.131722
O	9.672487	−6.760524	−1.912784
N	8.672912	−5.065650	−3.091384
C	8.421748	−3.727612	−3.437559
O	7.630716	−3.464026	−4.353282
C	9.145893	−2.752145	−2.636003
C	8.914717	−1.288933	−2.890529
C	10.018771	−3.211853	−1.705790
H	8.176462	−5.774689	−3.628205
H	9.197320	−1.023118	−3.916161
H	9.499225	−0.674309	−2.200552
H	7.853413	−1.038107	−2.777811
H	10.597929	−2.531003	−1.095390
H	11.311907	1.199039	1.620659
H	4.724254	3.276012	0.279922

(GAA)$^{H+}$

E: −14210.67 kcal mol^{-1}

O	−0.109089	−0.196987	−0.812808
C	−0.141311	0.384875	0.511573
C	1.199759	0.190030	1.211275
O	1.448816	−1.228894	1.479381
C	2.600693	−1.679275	0.724293
C	2.432519	0.673977	0.424998
C	2.879503	−0.583939	−0.308114
O	3.397332	1.157248	1.449150
H	−1.020147	−0.202215	−1.154864
H	−0.917947	−0.092299	1.122399
H	−0.349930	1.463341	0.458180
H	1.148998	0.707377	2.174845
H	3.446181	−1.814991	1.402074
H	2.226020	1.519354	−0.231836
H	2.229574	−0.706638	−1.177278
H	3.911619	−0.570264	−0.648318
N	2.355328	−2.996901	0.169390
C	1.450362	−3.395259	−0.813093
N	1.534248	−4.687295	−1.078533
C	2.541767	−5.169375	−0.239172
C	3.087241	−6.480127	−0.073833
O	2.778208	−7.542043	−0.643649
N	4.135665	−6.474749	0.892911
C	4.578638	−5.379128	1.604850
N	5.584197	−5.557181	2.509793
N	4.044681	−4.172880	1.457817
C	3.056766	−4.129185	0.543696
H	0.761080	−2.691048	−1.259945
H	4.576533	−7.378542	1.050533
H	6.178783	−6.371198	2.394864
H	6.090136	−4.695472	2.723916
P	4.995833	1.155544	1.415219
O	5.474651	1.328679	−0.108371
O	5.520481	2.115499	2.422997
O	5.495964	−0.362085	1.594693
C	5.698201	−0.934766	2.933909
C	7.045257	−1.643034	3.029411
O	7.070514	−2.920511	2.332363
C	7.810921	−2.823868	1.086941
C	8.235128	−0.855062	2.459113
C	8.366869	−1.389478	1.029177
O	9.376449	−1.152188	3.343711
H	4.893227	−1.652190	3.108503
H	5.659588	−0.136115	3.678984

H	7.206789	−1.860898	4.089585
H	8.596170	−3.584650	1.111050
H	8.089349	0.225524	2.490157
H	7.765915	−0.753505	0.379773
H	9.386765	−1.363045	0.655353
N	6.956587	−3.177946	−0.045043
C	6.079881	−2.376604	−0.769402
N	5.393844	−3.045980	−1.678796
C	5.826445	−4.365225	−1.552770
C	5.478333	−5.557487	−2.228776
N	4.566787	−5.595026	−3.227425
N	6.113630	−6.702635	−1.870603
C	7.035514	−6.657389	−0.890103
N	7.433128	−5.592178	−0.170183
C	6.798600	−4.469458	−0.544668
H	5.996621	−1.318562	−0.582747
H	4.253222	−6.495644	−3.569193
H	3.969382	−4.793982	−3.387654
H	7.516555	−7.603927	−0.650331
P	10.934008	−1.143309	2.996168
O	11.068140	0.011350	1.892944
O	11.749463	−1.019953	4.234391
O	11.228789	−2.467952	2.131982
C	11.269904	−3.762647	2.832900
C	11.806855	−4.822310	1.890705
O	10.853012	−5.040808	0.810492
C	11.538171	−4.952986	−0.466129
C	13.166690	−4.492953	1.231811
C	12.784050	−4.098866	−0.200978
O	13.961678	−5.705179	1.276139
H	10.254431	−4.017703	3.151702
H	11.919960	−3.676562	3.708316
H	11.904909	−5.749423	2.470082
H	11.796252	−5.958046	−0.815414
H	13.687239	−3.685130	1.755958
H	12.534761	−3.035034	−0.229903
H	13.583143	−4.293810	−0.919797
H	14.823351	−5.508532	0.864828
N	10.615249	−4.407866	−1.446640
C	10.155715	−3.100040	−1.537956
N	9.227308	−2.937967	−2.463917
C	9.058707	−4.205656	−3.020614
C	8.202669	−4.701014	−4.029992
N	7.306695	−3.920186	−4.681547
N	8.299384	−6.013471	−4.364020
C	9.176001	−6.790471	−3.700070
N	10.008203	−6.441065	−2.701247
C	9.912113	−5.133952	−2.402181
H	10.550705	−2.319587	−0.904913
H	6.592991	−4.375771	−5.239118
H	7.087109	−3.006672	−4.303231
H	9.208339	−7.834589	−4.007167
H	11.991363	0.245683	1.673680
H	5.320712	2.223544	−0.469625

(GAC)$^{H+}$

E: −13811.96 kcal mol^{-1}

O	0.051618	−0.085460	−0.901426
C	−0.018997	0.489570	0.424488
C	1.288488	0.259092	1.174889
O	1.494787	−1.166557	1.443528
C	2.660238	−1.641900	0.725330
C	2.561202	0.718389	0.440456
C	3.005498	−0.544540	−0.285432
O	3.497057	1.164831	1.506117
H	−0.846197	−0.073037	−1.276682
H	−0.830369	0.027868	1.000958
H	−0.199320	1.572968	0.369557
H	1.211996	0.772416	2.138935
H	3.476167	−1.808964	1.433168
H	2.400853	1.573297	−0.216958
H	2.387780	−0.645090	−1.180446
H	4.049567	−0.551405	−0.586589
N	2.398564	−2.946120	0.145408
C	1.538127	−3.301948	−0.892233
N	1.609802	−4.588564	−1.185471
C	2.563344	−5.112015	−0.308312
C	3.081257	−6.435421	−0.155170
O	2.785098	−7.475743	−0.769757
N	4.083227	−6.474010	0.859448
C	4.502402	−5.407055	1.627303
N	5.456020	−5.626501	2.577053
N	3.992348	−4.189238	1.490126
C	3.055847	−4.102422	0.527203
H	0.887491	−2.572783	−1.356436
H	4.500402	−7.389229	1.014523
H	6.046852	−6.444919	2.474557
H	5.949218	−4.778336	2.860666
P	5.096318	1.201352	1.490714
O	5.588163	1.372108	−0.028103
O	5.588240	2.179668	2.497577
O	5.623593	−0.305368	1.692202
C	5.728868	−0.880209	3.042620
C	7.009422	−1.694865	3.189649
O	6.943987	−2.978876	2.509142
C	7.763729	−2.983278	1.312413
C	8.277818	−1.013113	2.652536
C	8.445535	−1.602835	1.247825
O	9.358286	−1.380028	3.583937
H	4.862390	−1.527113	3.199211
H	5.732017	−0.071516	3.777970
H	7.119102	−1.908147	4.257607
H	8.481820	−3.801386	1.393835
H	8.205241	0.075339	2.644155
H	7.948090	−0.944051	0.535577
H	9.488368	−1.693817	0.950436
N	6.952151	−3.293660	0.137731
C	6.107834	−2.448077	−0.574573
N	5.451097	−3.062017	−1.541637
C	5.872420	−4.388790	−1.470626
C	5.553047	−5.534915	−2.233786
N	4.694800	−5.500351	−3.279865
N	6.160149	−6.705842	−1.913191
C	7.034949	−6.725745	−0.889264
N	7.411454	−5.706375	−0.095277
C	6.803723	−4.556413	−0.432621
H	6.022837	−1.403723	−0.324062
H	4.383219	−6.376279	−3.683011
H	4.107916	−4.686119	−3.412019
H	7.493991	−7.689935	−0.678986
P	10.921402	−1.086980	3.342099
O	11.026324	0.158997	2.338249
O	11.624885	−0.984148	4.649003
O	11.438817	−2.233274	2.347617
C	11.434166	−3.624541	2.828980
C	11.769328	−4.547083	1.677781
O	10.681533	−4.530065	0.720330
C	11.260630	−4.806145	−0.582012
C	13.073741	−4.221262	0.892161
C	12.596749	−4.049044	−0.563559
O	13.964451	−5.349814	1.072406
H	10.443930	−3.862143	3.229466
H	12.182260	−3.727192	3.620415
H	11.873682	−5.559814	2.094343
H	11.404819	−5.880835	−0.712365

H	13.553137	−3.312393	1.266325
H	12.438065	−2.988046	−0.779427
H	13.314964	−4.449048	−1.283382
H	14.828684	−5.107142	0.692737
N	10.300015	−4.400887	−1.603524
C	9.752366	−5.384063	−2.494346
O	10.164485	−6.558383	−2.436369
N	8.798908	−4.979367	−3.376788
C	8.396857	−3.702488	−3.421814
N	7.446001	−3.374372	−4.322839
C	8.950403	−2.695007	−2.565024
C	9.893089	−3.096795	−1.675346
H	6.972114	−4.113493	−4.829289
H	7.010691	−2.461616	−4.297794
H	8.627426	−1.662317	−2.613688
H	10.356847	−2.415598	−0.973459
H	11.219944	1.000179	2.796012
H	5.467218	2.275642	−0.379923

(GAG)$^{H+}$

E: −14375.47 kcal mol^{-1}

O	−0.092255	−0.268802	−0.800998
C	−0.124794	0.325932	0.517527
C	1.217724	0.142177	1.217670
O	1.474240	−1.273546	1.495279
C	2.627319	−1.723179	0.741264
C	2.447836	0.626463	0.427496
C	2.898943	−0.633629	−0.299037
O	3.412243	1.114565	1.449244
H	−1.003094	−0.275878	−1.143740
H	−0.899542	−0.147309	1.133791
H	−0.336533	1.403142	0.453605
H	1.164954	0.665801	2.177691
H	3.474619	−1.849927	1.418719
H	2.237997	1.468195	−0.232927
H	2.248336	−0.765302	−1.166374
H	3.930609	−0.617723	−0.640525
N	2.387225	−3.045598	0.195847
C	1.477702	−3.455898	−0.777582
N	1.568319	−4.748861	−1.035712
C	2.585462	−5.219063	−0.201325
C	3.142012	−6.524699	−0.032997
O	2.836441	−7.592161	−0.594511
N	4.197497	−6.506088	0.925603
C	4.638548	−5.403060	1.627396
N	5.652840	−5.568865	2.525146
N	4.094806	−4.201653	1.477701
C	3.099216	−4.170637	0.571407
H	0.780430	−2.758871	−1.223327
H	4.645813	−7.405805	1.085443
H	6.254378	−6.377122	2.405671
H	6.153722	−4.701734	2.729982
P	5.010475	1.142129	1.397322
O	5.467738	1.322776	−0.131703
O	5.528071	2.112927	2.398681
O	5.540513	−0.365941	1.573084
C	5.751810	−0.935448	2.912662
C	7.099360	−1.643977	3.001692
O	7.116135	−2.927716	2.315921
C	7.853949	−2.847633	1.067378
C	8.285809	−0.860931	2.416217
C	8.414544	−1.415861	0.993185
O	9.430904	−1.142033	3.299139
H	4.947723	−1.651889	3.095737
H	5.718788	−0.134311	3.655376
H	7.269293	−1.853098	4.062403
H	8.636336	−3.610693	1.096000
H	8.133265	0.219267	2.429359
H	7.816964	−0.786344	0.334207
H	9.433398	−1.401722	0.616566
N	6.995520	−3.208042	−0.060215
C	6.110623	−2.408573	−0.777924
N	5.416092	−3.079648	−1.678738
C	5.851058	−4.397828	−1.555285
C	5.496112	−5.590903	−2.226351
N	4.576996	−5.630264	−3.217182
N	6.128906	−6.738018	−1.867536
C	7.050573	−6.693277	−0.886410
N	7.455799	−5.627270	−0.172826
C	6.829663	−4.501460	−0.553032
H	6.027635	−1.350523	−0.590799
H	4.253272	−6.530429	−3.550175
H	3.992899	−4.822029	−3.389314
H	7.526354	−7.641638	−0.643310
P	10.989659	−1.028150	2.933696
O	11.135840	0.023362	1.732548
O	11.783631	−0.772851	4.166128
O	11.346718	−2.371777	2.132048
C	11.253502	−3.648473	2.860057
C	11.714393	−4.772163	1.952751
O	10.762150	−4.936707	0.863162
C	11.472777	−4.954289	−0.403053
C	13.107327	−4.577586	1.309599
C	12.781851	−4.198371	−0.141194
O	13.795646	−5.850737	1.406656
H	10.213958	−3.806628	3.162576
H	11.890890	−3.600954	3.748060
H	11.725325	−5.688902	2.556399
H	11.651321	−5.989641	−0.709877
H	13.685502	−3.800124	1.818748
H	12.622416	−3.118879	−0.206545
H	13.573360	−4.481465	−0.838911
H	14.678635	−5.740560	1.008574
N	10.615665	−4.371601	−1.419933
C	10.260198	−3.030346	−1.563383
N	9.371838	−2.838339	−2.517970
C	9.118525	−4.107977	−3.036870
C	8.242267	−4.525755	−4.085076
O	7.481618	−3.844126	−4.794464
N	8.309990	−5.934006	−4.268511
C	9.107309	−6.806735	−3.557762
N	9.004318	−8.126931	−3.853424
N	9.920032	−6.405359	−2.583474
C	9.882065	−5.073878	−2.365190
H	10.700577	−2.260983	−0.947103
H	7.692126	−6.304638	−4.987392
H	8.589155	−8.421615	−4.728795
H	9.700830	−8.742564	−3.450409
H	11.513287	0.876696	2.022433
H	5.294309	2.215012	−0.490893

(GAT)$^{H+}$

E: −14071.00 kcal mol^{-1}

O	−0.086927	−0.107074	−0.696637
C	−0.115830	0.439220	0.642930
C	1.224020	0.217411	1.336451
O	1.460805	−1.209312	1.574246
C	2.609983	−1.653637	0.812310
C	2.458769	0.707358	0.557470
C	2.897286	−0.539890	−0.198440
O	3.428338	1.161661	1.588892
H	−0.998094	−0.098890	−1.038358

H	−0.895788	−0.048800	1.240715
H	−0.316800	1.520083	0.618351
H	1.179645	0.715197	2.310594
H	3.453079	−1.810739	1.489754
H	2.256620	1.565275	−0.084282
H	2.247797	−0.642478	−1.070428
H	3.929146	−0.524472	−0.538953
N	2.354709	−2.957086	0.228081
C	1.461763	−3.319952	−0.779296
N	1.545728	−4.602261	−1.087088
C	2.541795	−5.115280	−0.252285
C	3.088782	−6.430370	−0.131629
O	2.787042	−7.470746	−0.743162
N	4.131176	−6.459060	0.841820
C	4.560839	−5.391250	1.602799
N	5.554948	−5.601940	2.512222
N	4.024665	−4.181558	1.496426
C	3.047995	−4.103976	0.572885
H	0.780629	−2.599216	−1.211980
H	4.568647	−7.368478	0.973656
H	6.158435	−6.406054	2.376244
H	6.041208	−4.747240	2.787707
P	5.024853	1.221024	1.511569
O	5.456837	1.409952	−0.022726
O	5.541921	2.195366	2.509447
O	5.579873	−0.281056	1.676981
C	5.739828	−0.871716	3.014830
C	7.045960	−1.651832	3.113452
O	6.996161	−2.933023	2.423961
C	7.772914	−2.905173	1.201129
C	8.277967	−0.930664	2.543416
C	8.416811	−1.507108	1.129005
O	9.392698	−1.263604	3.444255
H	4.896295	−1.544408	3.186528
H	5.742692	−0.074100	3.762201
H	7.195376	−1.872129	4.174940
H	8.512840	−3.707555	1.249614
H	8.172624	0.155172	2.541193
H	7.877875	−0.854573	0.441754
H	9.450242	−1.563347	0.793336
N	6.927613	−3.222423	0.050203
C	6.033278	−2.391916	−0.618258
N	5.355195	−3.012082	−1.565961
C	5.810320	−4.328814	−1.525295
C	5.477125	−5.479172	−2.278650
N	4.565227	−5.462877	−3.273903
N	6.121115	−6.640453	−1.987755
C	7.028113	−6.650607	−0.991693
N	7.418124	−5.626883	−0.209693
C	6.781877	−4.484848	−0.523210
H	5.929490	−1.353207	−0.350793
H	4.262967	−6.339707	−3.681126
H	3.980861	−4.647394	−3.406382
H	7.508488	−7.608109	−0.800114
P	10.950877	−1.018543	3.132252
O	11.042284	0.143215	2.031605
O	11.701586	−0.829149	4.402489
O	11.410981	−2.254148	2.218382
C	11.394887	−3.595026	2.825497
C	11.776387	−4.613604	1.774726
O	10.721768	−4.681023	0.777950
C	11.344570	−4.949947	−0.501038
C	13.108007	−4.344804	1.016258
C	12.673974	−4.184972	−0.453063
O	13.954962	−5.499734	1.234279
H	10.390781	−3.801292	3.208181
H	12.113725	−3.621539	3.649640
H	11.862284	−5.586383	2.278826
H	11.501190	−6.023143	−0.629382
H	13.608326	−3.445590	1.386364
H	12.521335	−3.126060	−0.679953
H	13.412793	−4.588857	−1.149087
H	14.830672	−5.299187	0.856069
N	10.406993	−4.547981	−1.550196
C	9.854347	−5.537279	−2.372039
O	10.151502	−6.732496	−2.308138
N	8.930284	−5.053588	−3.281040
C	8.513503	−3.727760	−3.471851
O	7.689342	−3.461445	−4.358475
C	9.121491	−2.765655	−2.563547
C	8.739315	−1.316612	−2.680189
C	10.025923	−3.222966	−1.662815
H	8.523916	−5.752130	−3.900726
H	9.024642	−0.918954	−3.661601
H	9.233096	−0.718410	−1.909853
H	7.654876	−1.191861	−2.588084
H	10.509058	−2.556903	−0.959015
H	11.309526	1.003900	2.408847
H	5.319048	2.316403	−0.360893

(GCA)$^{H+}$

E: −13808.50 kcal mol^{-1}

O	−0.187102	−0.033529	−0.726413
C	−0.224355	0.545515	0.599255
C	1.102019	0.315890	1.314993
O	1.307496	−1.108764	1.592191
C	2.446684	−1.599521	0.849175
C	2.354949	0.761558	0.538801
C	2.779100	−0.512844	−0.179633
O	3.323618	1.217082	1.567593
H	−1.091439	−0.007891	−1.085138
H	−1.021067	0.085965	1.197684
H	−0.405080	1.628850	0.545910
H	1.056521	0.837816	2.276243
H	3.279984	−1.775324	1.534514
H	2.176122	1.605428	−0.128087
H	2.147452	−0.621916	−1.063847
H	3.820911	−0.525664	−0.491619
N	2.153329	−2.901372	0.275221
C	1.223261	−3.244850	−0.706409
N	1.293906	−4.521761	−1.038822
C	2.316932	−5.051105	−0.248593
C	2.888701	−6.360284	−0.196741
O	2.585712	−7.379160	−0.845537
N	3.958685	−6.411112	0.740731
C	4.429008	−5.354877	1.493025
N	5.484987	−5.573203	2.328150
N	3.878897	−4.147254	1.446279
C	2.856313	−4.055650	0.575031
H	0.529600	−2.513260	−1.099764
H	4.430411	−7.310902	0.805297
H	6.087367	−6.365071	2.128667
H	5.982904	−4.717297	2.580577
P	4.919436	1.281169	1.448821
O	5.302230	1.518600	−0.091530
O	5.460971	2.229529	2.458415
O	5.474844	−0.224926	1.557249
C	5.700410	−0.834977	2.878354
C	7.002816	−1.626048	2.894677
O	6.889215	−2.889875	2.185179
C	7.695468	−2.885973	0.969434
C	8.206830	−0.907381	2.262938
C	8.243712	−1.458920	0.836464
O	9.367927	−1.275742	3.094103
H	4.862508	−1.505156	3.083650

H	5.745933	−0.047470	3.634800
H	7.212789	−1.858262	3.943838
H	8.488228	−3.626255	1.080946
H	8.125252	0.179886	2.295320
H	7.599796	−0.827682	0.222658
H	9.233376	−1.441254	0.389585
N	6.879967	−3.367778	−0.150914
C	6.984826	−4.757487	−0.521710
O	7.817206	−5.474489	0.055545
N	6.146784	−5.222145	−1.488605
C	5.246188	−4.420459	−2.067642
N	4.456489	−4.949803	−3.025082
C	5.103295	−3.042478	−1.699599
C	5.926720	−2.574270	−0.727457
H	4.511269	−5.941429	−3.224056
H	3.691483	−4.420909	−3.422122
H	4.357335	−2.405623	−2.158953
H	5.855050	−1.562748	−0.352385
P	10.919047	−1.255099	2.721299
O	11.025939	−0.181405	1.535624
O	11.758184	−1.035023	3.930239
O	11.221171	−2.631252	1.943708
C	11.299886	−3.878968	2.723391
C	11.992201	−4.953687	1.902578
O	11.164046	−5.344720	0.771703
C	11.824708	−5.027386	−0.479147
C	13.365262	−4.565431	1.322053
C	13.040092	−4.156443	−0.121433
O	14.198913	−5.750711	1.383580
H	10.281707	−4.194133	2.970133
H	11.861900	−3.692687	3.643237
H	12.104663	−5.826365	2.557524
H	12.112456	−5.963094	−0.966809
H	13.831269	−3.749724	1.884939
H	12.785210	−3.095536	−0.144349
H	13.877084	−4.325786	−0.802515
H	15.058434	−5.528538	0.980953
N	10.867777	−4.388413	−1.376072
C	10.458520	−3.061319	−1.388860
N	9.485203	−2.824134	−2.251367
C	9.226777	−4.061036	−2.841371
C	8.295186	−4.479099	−3.817606
N	7.428392	−3.627653	−4.417895
N	8.289469	−5.787533	−4.179215
C	9.155969	−6.629194	−3.583465
N	10.073478	−6.350071	−2.638778
C	10.068928	−5.047651	−2.304463
H	10.916227	−2.324401	−0.746455
H	6.666992	−4.015402	−4.962387
H	7.310406	−2.694925	−4.042876
H	9.104341	−7.667197	−3.908656
H	11.926625	0.177970	1.413885
H	5.160603	2.438153	−0.390966

(GCC)$^{H+}$

E: −13409.67 kcal mol^{-1}

O	−0.199013	0.110785	−0.649533
C	−0.241494	0.643335	0.695383
C	1.073656	0.368469	1.415439
O	1.252147	−1.067942	1.647154
C	2.397160	−1.550423	0.908956
C	2.340356	0.818842	0.664910
C	2.753788	−0.439924	−0.085874
O	3.306584	1.231051	1.714506
H	−1.100030	0.157595	−1.014223
H	−1.051212	0.175588	1.269139
H	−0.404945	1.730401	0.678392
H	1.027558	0.860156	2.392465
H	3.219097	−1.753009	1.600825
H	2.179316	1.683981	0.021172
H	2.127962	−0.516020	−0.977650
H	3.797567	−0.456740	−0.390314
N	2.099679	−2.832277	0.293390
C	1.178109	−3.134812	−0.709788
N	1.252004	−4.396765	−1.093859
C	2.269863	−4.957559	−0.318999
C	2.846194	−6.265788	−0.323731
O	2.549094	−7.255610	−1.018225
N	3.915077	−6.353325	0.612571
C	4.375680	−5.331119	1.416684
N	5.428452	−5.583413	2.244950
N	3.820140	−4.124617	1.422932
C	2.802045	−3.997339	0.550285
H	0.487836	−2.387345	−1.078627
H	4.388621	−7.254069	0.639838
H	6.025695	−6.374470	2.028816
H	5.924318	−4.742113	2.544237
P	4.903882	1.273430	1.609735
O	5.301764	1.544354	0.078531
O	5.454056	2.185801	2.647328
O	5.436064	−0.243386	1.682552
C	5.668398	−0.888548	2.986013
C	6.977318	−1.669382	2.975333
O	6.865927	−2.918247	2.238074
C	7.665662	−2.889653	1.021620
C	8.168826	−0.922854	2.351314
C	8.222358	−1.460609	0.919568
O	9.339215	−1.271565	3.175270
H	4.834973	−1.569413	3.172874
H	5.710762	−0.121955	3.763479
H	7.198401	−1.924495	4.016824
H	8.453224	−3.639448	1.106334
H	8.061565	0.161872	2.390492
H	7.592704	−0.821609	0.298716
H	9.223331	−1.439881	0.497213
N	6.841938	−3.328103	−0.112912
C	6.977319	−4.685016	−0.582229
O	7.825728	−5.423559	−0.057578
N	6.151862	−5.095398	−1.584431
C	5.234244	−4.272582	−2.106381
N	4.455139	−4.745474	−3.100065
C	5.058781	−2.929585	−1.636733
C	5.869899	−2.516075	−0.631028
H	4.532021	−5.716501	−3.378319
H	3.687772	−4.198531	−3.466983
H	4.297762	−2.279050	−2.049590
H	5.775044	−1.538360	−0.179106
P	10.887404	−1.127350	2.807327
O	10.907694	−0.012303	1.657172
O	11.707485	−0.884728	4.025077
O	11.285695	−2.458073	1.994528
C	11.404236	−3.717756	2.751640
C	12.001293	−4.789437	1.860368
O	11.068542	−5.107250	0.791569
C	11.732971	−5.005057	−0.495865
C	13.348748	−4.430348	1.193007
C	12.962826	−4.116320	−0.258747
O	14.196041	−5.603394	1.298748
H	10.404586	−4.017514	3.080806
H	12.046117	−3.553692	3.621962
H	12.140906	−5.680782	2.485936
H	12.009727	−6.000332	−0.845650
H	13.830681	−3.578274	1.683347
H	12.711428	−3.056385	−0.345341
H	13.769147	−4.338286	−0.961913

H	15.047165	−5.391611	0.873359
N	10.765540	−4.494212	−1.471611
C	10.206245	−5.378465	−2.449328
O	10.604360	−6.559841	−2.506972
N	9.256836	−4.883739	−3.291116
C	8.831606	−3.618418	−3.181294
N	7.868914	−3.200197	−4.027991
C	9.355278	−2.720713	−2.194101
C	10.307577	−3.210956	−1.361346
H	7.468314	−3.848198	−4.695053
H	7.490921	−2.264449	−3.971996
H	8.999594	−1.701497	−2.105902
H	10.739199	−2.623233	−0.561263
H	11.795205	0.357671	1.481267
H	5.169710	2.471749	−0.200062

$(GCG)^{H+}$

E: −13972.78 kcal mol^{-1}

O	−0.222770	−0.084620	−0.662386
C	−0.244578	0.479749	0.669886
C	1.093673	0.251639	1.363444
O	1.312370	−1.175200	1.618812
C	2.451553	−1.644411	0.862321
C	2.332014	0.716588	0.574847
C	2.758588	−0.546487	−0.161695
O	3.311734	1.169699	1.594712
H	−1.133460	−0.067172	−1.005060
H	−1.028985	0.008139	1.275051
H	−0.433779	1.562376	0.631249
H	1.058328	0.761773	2.331561
H	3.293865	−1.812572	1.538615
H	2.135897	1.565622	−0.080575
H	2.115109	−0.654421	−1.037565
H	3.796073	−0.546713	−0.488197
N	2.172414	−2.946017	0.281430
C	1.243239	−3.296561	−0.698520
N	1.333548	−4.569075	−1.042402
C	2.369597	−5.087935	−0.262211
C	2.964176	−6.387366	−0.224904
O	2.676182	−7.405312	−0.882218
N	4.038362	−6.428320	0.708069
C	4.493899	−5.371148	1.467913
N	5.555899	−5.580173	2.299113
N	3.924077	−4.172523	1.433453
C	2.896127	−4.090704	0.567451
H	0.535295	−2.573789	−1.082579
H	4.525099	−7.320663	0.763396
H	6.177685	−6.350820	2.075583
H	6.040899	−4.716276	2.551079
P	4.904106	1.260452	1.444689
O	5.251691	1.510071	−0.102008
O	5.450215	2.213675	2.447659
O	5.486999	−0.236456	1.535803
C	5.731966	−0.849172	2.852525
C	7.043260	−1.625732	2.855355
O	6.936328	−2.891189	2.148613
C	7.737989	−2.886070	0.929389
C	8.234825	−0.893162	2.214378
C	8.281468	−1.456987	0.792895
O	9.402128	−1.231319	3.048949
H	4.903188	−1.528945	3.063629
H	5.776443	−0.063574	3.611034
H	7.265341	−1.854603	3.902893
H	8.532922	−3.624749	1.038494
H	8.133275	0.192741	2.235567
H	7.639640	−0.832693	0.169622
H	9.273173	−1.441497	0.349793
N	6.919589	−3.367794	−0.189242
C	7.016586	−4.758927	−0.556129
O	7.833678	−5.482827	0.034380
N	6.187124	−5.218554	−1.533116
C	5.285857	−4.414938	−2.109600
N	4.494675	−4.942569	−3.065715
C	5.136712	−3.040545	−1.731035
C	5.961890	−2.573861	−0.760530
H	4.547997	−5.933621	−3.266689
H	3.739010	−4.408306	−3.472746
H	4.387435	−2.403824	−2.185383
H	5.887692	−1.564077	−0.380965
P	10.954377	−1.162614	2.681546
O	11.043343	−0.134087	1.455381
O	11.779214	−0.867043	3.884715
O	11.301104	−2.557450	1.959673
C	11.320222	−3.778454	2.783342
C	11.950256	−4.913344	1.995010
O	11.106400	−5.273786	0.866150
C	11.799722	−5.034897	−0.385359
C	13.350654	−4.629009	1.419222
C	13.065890	−4.235604	−0.036751
O	14.103293	−5.865434	1.518862
H	10.288975	−4.033711	3.045997
H	11.896559	−3.589537	3.694179
H	12.002587	−5.775912	2.670837
H	12.032805	−6.001253	−0.841213
H	13.864401	−3.832363	1.967608
H	12.881279	−3.161190	−0.087826
H	13.895894	−4.476489	−0.704771
H	14.979402	−5.711320	1.120211
N	10.897064	−4.363145	−1.312961
C	10.587250	−3.005485	−1.388840
N	9.649004	−2.745439	−2.278711
C	9.307749	−3.985489	−2.817645
C	8.350374	−4.332142	−3.819537
O	7.584654	−3.594011	−4.465697
N	8.339553	−5.734789	−4.043757
C	9.120559	−6.664121	−3.390282
N	8.913554	−7.974566	−3.691737
N	10.017614	−6.329353	−2.467433
C	10.067305	−5.002517	−2.222276
H	11.086232	−2.274837	−0.770407
H	7.661001	−6.055814	−4.730777
H	8.451417	−8.222315	−4.558856
H	9.609590	−8.629985	−3.354753
H	11.896450	0.340587	1.411064
H	5.077880	2.425148	−0.397826

$(GCT)^{H+}$

E: −13669.35 kcal mol^{-1}

O	−0.128670	0.162075	−0.579800
C	−0.153583	0.683964	0.769636
C	1.163689	0.385934	1.477104
O	1.323285	−1.054613	1.698184
C	2.452826	−1.549895	0.944445
C	2.430626	0.822781	0.718623
C	2.819079	−0.436658	−0.043966
O	3.412429	1.216134	1.761540
H	−1.031170	0.226423	−0.938144
H	−0.964519	0.221491	1.346292
H	−0.303556	1.773239	0.763255
H	1.132511	0.871710	2.457685
H	3.276991	−1.772751	1.626864
H	2.276856	1.694410	0.081902

H	2.185917	−0.495960	−0.931785
H	3.860144	−0.466446	−0.356739
N	2.129510	−2.821090	0.319865
C	1.200109	−3.098238	−0.683335
N	1.251862	−4.357342	−1.080173
C	2.262715	−4.942491	−0.314228
C	2.818648	−6.259382	−0.335856
O	2.504869	−7.235946	−1.041675
N	3.887601	−6.374810	0.597092
C	4.365645	−5.369483	1.412540
N	5.413401	−5.649858	2.237541
N	3.830828	−4.153727	1.432298
C	2.813062	−4.000316	0.563309
H	0.521332	−2.335771	−1.042645
H	4.346633	−7.283314	0.613710
H	5.996676	−6.449247	2.014767
H	5.921022	−4.821934	2.553663
P	5.009720	1.210289	1.654479
O	5.416499	1.470556	0.124103
O	5.588212	2.102718	2.693990
O	5.497134	−0.321968	1.723457
C	5.703585	−0.979809	3.024789
C	7.005621	−1.771120	3.023789
O	6.889098	−3.017023	2.283253
C	7.707109	−3.006334	1.081246
C	8.208053	−1.027167	2.416410
C	8.320666	−1.598075	0.997444
O	9.356198	−1.333203	3.282332
H	4.860902	−1.653959	3.193257
H	5.742134	−0.220714	3.809823
H	7.216270	−2.031251	4.066188
H	8.465225	−3.785205	1.166987
H	8.079177	0.055893	2.418230
H	7.760241	−0.949001	0.323236
H	9.348306	−1.635819	0.641897
N	6.883929	−3.400085	−0.073427
C	6.986311	−4.749329	−0.571238
O	7.822074	−5.518162	−0.068292
N	6.152142	−5.120726	−1.580323
C	5.252500	−4.267062	−2.085807
N	4.458198	−4.705069	−3.080924
C	5.108554	−2.930288	−1.587779
C	5.929053	−2.556492	−0.574491
H	4.528266	−5.665778	−3.393789
H	3.720351	−4.125332	−3.457414
H	4.363818	−2.253539	−1.987756
H	5.856525	−1.586376	−0.101868
P	10.889493	−0.970779	2.964222
O	10.876031	0.148248	1.816745
O	11.624564	−0.658590	4.219915
O	11.448916	−2.213796	2.117476
C	11.519619	−3.518174	2.798021
C	11.983182	−4.565004	1.808985
O	10.944968	−4.758189	0.810183
C	11.581593	−4.922331	−0.480327
C	13.298322	−4.241734	1.048467
C	12.843708	−4.053984	−0.408671
O	14.174538	−5.383801	1.218845
H	10.526178	−3.773784	3.178679
H	12.225996	−3.447567	3.630479
H	12.128706	−5.499906	2.367362
H	11.827130	−5.973006	−0.649207
H	13.781965	−3.342519	1.440752
H	12.607397	−3.000828	−0.582870
H	13.605844	−4.365672	−1.126764
H	15.029442	−5.163878	0.805560
N	10.610249	−4.563162	−1.514613
C	10.052676	−5.585294	−2.292704
O	10.368633	−6.774634	−2.195918
N	9.115896	−5.142852	−3.207897
C	8.644570	−3.837984	−3.401951
O	7.790491	−3.609703	−4.271864
C	9.232429	−2.843926	−2.515650
C	8.786884	−1.413448	−2.636663
C	10.173250	−3.256478	−1.630239
H	8.698232	−5.867802	−3.788275
H	8.996621	−1.027102	−3.641097
H	9.300298	−0.779872	−1.908963
H	7.705112	−1.327515	−2.482010
H	10.644207	−2.563186	−0.944851
H	11.219820	1.008990	2.126072
H	5.302979	2.399512	−0.157536

(GGA)$^{H+}$

E: −14375.10 kcal mol^{-1}

O	−0.276201	−0.487243	−0.574342
C	−0.319736	0.118543	0.739060
C	1.051466	0.047342	1.403016
O	1.422293	−1.340908	1.693115
C	2.586615	−1.713239	0.916346
C	2.215882	0.609981	0.567221
C	2.744702	−0.624815	−0.148459
O	3.173895	1.186481	1.546281
H	−1.191801	−0.560007	−0.895944
H	−1.039551	−0.405453	1.380408
H	−0.615453	1.175465	0.670635
H	0.986816	0.580965	2.356830
H	3.459821	−1.764683	1.572425
H	1.919943	1.418490	−0.101903
H	2.089466	−0.824576	−0.998924
H	3.764275	−0.532392	−0.510965
N	2.434933	−3.058793	0.391039
C	1.534851	−3.547929	−0.554859
N	1.734957	−4.826070	−0.825304
C	2.820253	−5.203562	−0.030492
C	3.507246	−6.449803	0.103190
O	3.292801	−7.537190	−0.462063
N	4.590177	−6.336156	1.026703
C	4.939659	−5.202227	1.731503
N	5.993497	−5.273840	2.598305
N	4.278671	−4.059424	1.611183
C	3.258796	−4.115533	0.734618
H	0.762437	−2.915861	−0.972806
H	5.115853	−7.194806	1.177250
H	6.666893	−6.017867	2.446197
H	6.417947	−4.359994	2.770997
P	4.751856	1.402968	1.384778
O	5.081528	1.579566	−0.177407
O	5.210985	2.466870	2.318134
O	5.469350	−0.023047	1.570148
C	5.838249	−0.500279	2.910596
C	7.166775	−1.247677	2.873699
O	7.070790	−2.499842	2.145014
C	7.740438	−2.396501	0.857483
C	8.341768	−0.503459	2.216062
C	8.269140	−0.961670	0.758241
O	9.559175	−0.900336	2.960456
H	5.052072	−1.179106	3.250320
H	5.916490	0.351365	3.591507
H	7.411582	−1.492398	3.912329
H	8.545026	−3.129065	0.856872
H	8.282744	0.579683	2.323574
H	7.556023	−0.311010	0.250459
H	9.216986	−0.899846	0.226382
N	6.860309	−2.819354	−0.217134

C	5.943910	−2.125616	−1.004149
N	5.340185	−2.905648	−1.883114
C	5.875419	−4.179757	−1.673186
C	5.654430	−5.427560	−2.337500
O	4.887436	−5.698879	−3.273821
N	6.491451	−6.450399	−1.786952
C	7.400999	−6.290369	−0.761068
N	8.104687	−7.373705	−0.328190
N	7.583716	−5.126702	−0.152553
C	6.821012	−4.134925	−0.643508
H	5.786837	−1.064525	−0.888988
H	6.385177	−7.365651	−2.219833
H	8.194563	−8.172182	−0.946934
H	8.949637	−7.128246	0.188968
P	10.925711	−1.596598	2.510090
O	11.458693	−0.856523	1.185780
O	11.844464	−1.659842	3.677886
O	10.600032	−2.999848	1.808593
C	10.473446	−4.250485	2.574939
C	11.285610	−5.360555	1.917931
O	10.694045	−5.794284	0.658849
C	11.516921	−5.387097	−0.466662
C	12.744656	−4.999528	1.590086
C	12.687778	−4.574282	0.113603
O	13.529403	−6.201588	1.786240
H	9.413225	−4.514166	2.582658
H	10.826975	−4.089799	3.596439
H	11.255296	−6.216576	2.601784
H	11.846147	−6.292228	−0.981956
H	13.121255	−4.196364	2.232424
H	12.497740	−3.502193	0.048468
H	13.622088	−4.786256	−0.410780
H	14.449985	−5.997299	1.538811
N	10.697507	−4.657866	−1.434549
C	10.302381	−3.324728	−1.405421
N	9.427996	−3.019476	−2.348756
C	9.217473	−4.212992	−3.037874
C	8.376092	−4.558972	−4.120224
N	7.562417	−3.667577	−4.735688
N	8.401235	−5.842584	−4.564517
C	9.211188	−6.728074	−3.952498
N	10.032073	−6.520038	−2.906198
C	9.997684	−5.242325	−2.486240
H	10.694297	−2.634431	−0.674656
H	6.856783	−4.020864	−5.371900
H	7.385424	−2.773927	−4.293433
H	9.191233	−7.740949	−4.351776
H	11.687978	0.082797	1.328521
H	4.806192	2.443254	−0.542072

(GGC)$^{H+}$

E: −13975.21 kcal mol^{-1}

O	−0.395599	−0.247799	−0.348922
C	−0.327880	0.257455	1.004957
C	1.081357	0.093219	1.561560
O	1.414159	−1.324536	1.727960
C	2.530914	−1.672557	0.872422
C	2.209219	0.674311	0.688174
C	2.655323	−0.527559	−0.135727
O	3.241525	1.151551	1.643469
H	−1.333826	−0.281203	−0.605136
H	−1.020003	−0.291150	1.656146
H	−0.589315	1.325176	1.036655
H	1.103410	0.561708	2.551077
H	3.434100	−1.773280	1.481135
H	1.902370	1.533481	0.091360
H	1.938822	−0.656243	−0.950092
H	3.650661	−0.440937	−0.564685
N	2.329316	−2.985270	0.288245
C	1.427691	−3.398606	−0.692147
N	1.587083	−4.668727	−1.018775
C	2.643984	−5.121930	−0.225331
C	3.291958	−6.393675	−0.147910
O	3.054797	−7.442565	−0.773492
N	4.363869	−6.361568	0.794339
C	4.734306	−5.277905	1.564781
N	5.765025	−5.429327	2.446305
N	4.111258	−4.109411	1.492061
C	3.106652	−4.087727	0.596821
H	0.684162	−2.723632	−1.094365
H	4.858530	−7.243748	0.909809
H	6.424753	−6.181042	2.276015
H	6.193127	−4.542668	2.717580
P	4.796119	1.414871	1.387420
O	4.980122	1.616067	−0.193167
O	5.309369	2.487190	2.281594
O	5.533935	−0.009889	1.551688
C	5.751851	−0.565512	2.895692
C	7.041992	−1.375327	2.942449
O	6.934101	−2.648905	2.245689
C	7.678894	−2.629927	1.002165
C	8.272690	−0.685632	2.329991
C	8.305796	−1.229009	0.899322
O	9.413050	−1.087097	3.170975
H	4.904442	−1.209936	3.143100
H	5.819467	0.253967	3.616824
H	7.224785	−1.604437	3.996837
H	8.424259	−3.429106	1.044704
H	8.217877	0.403324	2.364265
H	7.692473	−0.570025	0.283767
H	9.301359	−1.249417	0.461749
N	6.811487	−2.970129	−0.121307
C	5.899527	−2.160087	−0.800610
N	5.251599	−2.808577	−1.748327
C	5.742673	−4.112420	−1.698963
C	5.452836	−5.249400	−2.516158
O	4.666261	−5.350594	−3.472032
N	6.226859	−6.373512	−2.109977
C	7.158858	−6.388139	−1.093354
N	7.780424	−7.567555	−0.821017
N	7.430302	−5.319579	−0.352583
C	6.711616	−4.231140	−0.693201
H	5.765860	−1.123253	−0.538328
H	6.067008	−7.223409	−2.647158
H	7.802444	−8.290238	−1.531501
H	8.613103	−7.496914	−0.245706
P	10.962099	−1.160307	2.774548
O	11.166492	−0.130930	1.563263
O	11.820057	−0.978993	3.976300
O	11.148959	−2.558336	2.002322
C	11.054888	−3.800187	2.792878
C	11.613959	−4.953905	1.982339
O	10.733676	−5.233111	0.858866
C	11.496032	−5.239172	−0.376903
C	13.029834	−4.731319	1.402514
C	12.769890	−4.434742	−0.080723
O	13.763269	−5.967659	1.598435
H	10.002650	−3.978555	3.033863
H	11.629042	−3.681132	3.716329
H	11.632510	−5.830118	2.643387
H	11.720305	−6.265538	−0.670009
H	13.548587	−3.906738	1.902047
H	12.600081	−3.362944	−0.213619
H	13.603674	−4.736162	−0.719376
H	14.654789	−5.844183	1.223980

N	10.639819	−4.700689	−1.437476
C	10.160413	−5.566306	−2.472999
O	10.565134	−6.744223	−2.523028
N	9.268340	−5.057205	−3.368162
C	8.862335	−3.782671	−3.286734
N	7.972694	−3.346419	−4.201672
C	9.336180	−2.893562	−2.266542
C	10.207845	−3.405748	−1.361593
H	7.597817	−3.992067	−4.886133
H	7.578150	−2.417245	−4.146401
H	8.999600	−1.865818	−2.206697
H	10.591882	−2.829350	−0.529045
H	11.906076	0.488918	1.716331
H	5.349292	2.490723	−0.423688

(GGG)$^{H+}$

E: −14539.57 kcal mol^{-1}

O	−0.200455	−0.511750	−0.672272
C	−0.227245	0.121799	0.628021
C	1.138139	0.015606	1.298829
O	1.459978	−1.379763	1.613000
C	2.622454	−1.797894	0.857395
C	2.325139	0.525106	0.460039
C	2.817153	−0.739098	−0.230483
O	3.293929	1.091158	1.436549
H	−1.116538	−0.556154	−0.997648
H	−0.967649	−0.362713	1.276841
H	−0.484623	1.187051	0.536988
H	1.088005	0.567244	2.243238
H	3.487865	−1.853292	1.521759
H	2.060337	1.331441	−0.224384
H	2.154214	−0.935582	−1.075935
H	3.839088	−0.690914	−0.595606
N	2.444177	−3.151766	0.365696
C	1.515226	−3.648645	−0.547193
N	1.682368	−4.938298	−0.782016
C	2.773983	−5.315406	0.003933
C	3.434546	−6.572542	0.162266
O	3.191375	−7.668375	−0.374725
N	4.528557	−6.459897	1.071308
C	4.922196	−5.313290	1.731380
N	5.988045	−5.389349	2.581685
N	4.291817	−4.155655	1.581444
C	3.252559	−4.214745	0.726410
H	0.749206	−3.011685	−0.969612
H	5.041056	−7.324563	1.233688
H	6.627326	−6.166917	2.450884
H	6.457033	−4.489150	2.706011
P	4.884645	1.203292	1.310610
O	5.259129	1.376594	−0.242152
O	5.393463	2.222304	2.267562
O	5.504835	−0.268729	1.487155
C	5.835553	−0.793927	2.819835
C	7.219301	−1.435131	2.826380
O	7.258878	−2.704592	2.118454
C	7.890815	−2.556029	0.818817
C	8.335578	−0.593406	2.186692
C	8.375448	−1.099116	0.739905
O	9.551779	−0.812369	2.996628
H	5.084335	−1.546242	3.070474
H	5.812316	0.020827	3.547986
H	7.457401	−1.649821	3.872692
H	8.707026	−3.278398	0.780584
H	8.146391	0.478807	2.244052
H	7.683393	−0.482441	0.166974
H	9.351407	−1.012028	0.269392
N	6.977964	−2.950990	−0.251663
C	6.083278	−2.197052	−1.014434
N	5.399920	−2.932325	−1.870013
C	5.847791	−4.238258	−1.671074
C	5.505739	−5.461089	−2.328123
O	4.707081	−5.663074	−3.257525
N	6.248595	−6.552878	−1.783857
C	7.183539	−6.469415	−0.771594
N	7.751375	−7.626186	−0.327089
N	7.503906	−5.324127	−0.188253
C	6.825762	−4.264703	−0.667768
H	5.995989	−1.128597	−0.898941
H	6.045442	−7.459654	−2.200073
H	7.740028	−8.441453	−0.930267
H	8.605053	−7.495209	0.208199
P	11.018957	−1.295643	2.582708
O	11.472112	−0.504993	1.259403
O	11.908205	−1.204523	3.771820
O	10.897188	−2.740361	1.902984
C	10.735238	−3.941278	2.739573
C	11.381181	−5.130385	2.043783
O	10.670241	−5.464170	0.820285
C	11.504495	−5.217443	−0.339769
C	12.856603	−4.937026	1.645228
C	12.777493	−4.520814	0.169006
O	13.511520	−6.218895	1.820588
H	9.663499	−4.112641	2.871751
H	11.208230	−3.774645	3.711242
H	11.291659	−5.983175	2.727638
H	11.716184	−6.178271	−0.816084
H	13.350535	−4.176789	2.259854
H	12.688094	−3.435556	0.104522
H	13.660162	−4.827426	−0.396629
H	14.435047	−6.121719	1.524211
N	10.749195	−4.445577	−1.324893
C	10.546205	−3.065965	−1.382907
N	9.701963	−2.714454	−2.333693
C	9.314451	−3.911162	−2.935034
C	8.406139	−4.161084	−4.009415
O	7.748182	−3.355489	−4.690254
N	8.293710	−5.558418	−4.259943
C	8.971333	−6.557254	−3.591989
N	8.675355	−7.843261	−3.925178
N	9.827789	−6.310122	−2.607131
C	9.953662	−4.996135	−2.318228
H	11.045637	−2.388701	−0.707563
H	7.648724	−5.811606	−5.005932
H	8.242339	−8.040578	−4.820003
H	9.309384	−8.553101	−3.575503
H	11.671254	0.438205	1.419669
H	5.036902	2.256475	−0.604579

(GGT)$^{H+}$

E: −14234.58 kcal mol^{-1}

O	−0.245694	−0.215306	−0.456675
C	−0.253765	0.293158	0.897893
C	1.115706	0.111106	1.543665
O	1.417836	−1.309768	1.739887
C	2.564277	−1.685798	0.938062
C	2.304297	0.668385	0.738786
C	2.769992	−0.542168	−0.058663
O	3.289485	1.133991	1.751353
H	−1.167088	−0.237585	−0.769003
H	−0.993035	−0.243385	1.505802
H	−0.501154	1.364614	0.910323
H	1.082200	0.584740	2.530265

H	3.433970	−1.815051	1.587431
H	2.048198	1.532867	0.125740
H	2.097841	−0.656403	−0.911857
H	3.788809	−0.478234	−0.429780
N	2.352046	−2.989383	0.336613
C	1.431542	−3.378820	−0.635735
N	1.565513	−4.648501	−0.974708
C	2.624321	−5.125538	−0.198293
C	3.247855	−6.409783	−0.137825
O	2.988365	−7.447430	−0.773751
N	4.325930	−6.407622	0.796988
C	4.731505	−5.335850	1.566803
N	5.770272	−5.517141	2.431656
N	4.134851	−4.152147	1.506174
C	3.116684	−4.105508	0.625902
H	0.695044	−2.685702	−1.020453
H	4.807962	−7.298974	0.896114
H	6.397607	−6.295673	2.259715
H	6.236968	−4.646112	2.690647
P	4.876873	1.273811	1.613953
O	5.235649	1.530007	0.070094
O	5.385499	2.243447	2.620911
O	5.515783	−0.200009	1.707292
C	5.776574	−0.820647	3.013708
C	7.114474	−1.551400	3.011266
O	7.075956	−2.806510	2.275022
C	7.780729	−2.706002	1.015564
C	8.291854	−0.760378	2.413491
C	8.406116	−1.297574	0.980137
O	9.448010	−1.035190	3.280472
H	4.970503	−1.530288	3.214639
H	5.795024	−0.045742	3.784483
H	7.327410	−1.807662	4.053636
H	8.525969	−3.505595	0.989498
H	8.131379	0.318385	2.435225
H	7.844963	−0.630177	0.326244
H	9.429561	−1.323611	0.612943
N	6.880425	−2.967727	−0.110238
C	5.990506	−2.088134	−0.732441
N	5.278958	−2.665671	−1.678063
C	5.698064	−3.994046	−1.688367
C	5.289203	−5.086301	−2.514884
O	4.461511	−5.107898	−3.438822
N	5.982595	−6.277090	−2.152270
C	6.926291	−6.389839	−1.152105
N	7.418642	−7.629704	−0.894369
N	7.326797	−5.355628	−0.418203
C	6.687055	−4.203828	−0.716424
H	5.922975	−1.054092	−0.438751
H	5.711355	−7.108248	−2.674168
H	7.315199	−8.364367	−1.584594
H	8.259879	−7.670394	−0.329828
P	11.001674	−0.915066	2.893895
O	11.129169	0.125749	1.682098
O	11.810008	−0.647311	4.113821
O	11.348184	−2.267132	2.100759
C	11.254500	−3.529239	2.855550
C	11.698477	−4.670101	1.967258
O	10.717874	−4.856758	0.907487
C	11.430388	−5.133071	−0.322102
C	13.081989	−4.491092	1.285022
C	12.737668	−4.340771	−0.205616
O	13.844992	−5.692175	1.562125
H	10.217614	−3.673872	3.173604
H	11.903165	−3.468602	3.734366
H	11.732355	−5.574240	2.589675
H	11.617399	−6.204302	−0.420321
H	13.613154	−3.615970	1.670238
H	12.576831	−3.284864	−0.441835
H	13.522643	−4.731689	−0.857086
H	14.737770	−5.566800	1.191426
N	10.556656	−4.771147	−1.438870
C	10.081757	−5.788347	−2.272420
O	10.390327	−6.977137	−2.154758
N	9.217115	−5.344388	−3.257531
C	8.776275	−4.033465	−3.495232
O	7.995035	−3.806198	−4.430418
C	9.301283	−3.039716	−2.569384
C	8.890111	−1.604722	−2.742480
C	10.152548	−3.458028	−1.600130
H	8.881296	−6.061006	−3.898496
H	9.209318	−1.228225	−3.721764
H	9.335300	−0.973258	−1.969563
H	7.800340	−1.503269	−2.700219
H	10.569120	−2.770170	−0.874361
H	11.492684	0.988277	1.962940
H	5.019764	2.431025	−0.240313

(GTA)$^{H+}$

E: −14067.52 kcal mol^{-1}

O	−0.392582	0.259790	−0.273122
C	−0.259369	0.782792	1.069610
C	1.134207	0.488775	1.613480
O	1.325112	−0.952009	1.803882
C	2.359126	−1.436402	0.917873
C	2.298671	0.938953	0.710751
C	2.607977	−0.314844	−0.096099
O	3.393949	1.352291	1.625427
H	−1.332151	0.315164	−0.520295
H	−0.994459	0.318023	1.738501
H	−0.412625	1.871547	1.080867
H	1.217933	0.968461	2.594022
H	3.257241	−1.662251	1.497132
H	2.059531	1.808302	0.097789
H	1.885679	−0.372506	−0.913189
H	3.610849	−0.337949	−0.516072
N	1.973330	−2.705690	0.324933
C	0.943507	−2.989560	−0.572376
N	0.949390	−4.253552	−0.957675
C	2.031685	−4.835410	−0.292609
C	2.556879	−6.165147	−0.325730
O	2.159275	−7.155712	−0.964369
N	3.706056	−6.277996	0.512115
C	4.247961	−5.271576	1.287568
N	5.328833	−5.559912	2.058649
N	3.743408	−4.043024	1.312985
C	2.668378	−3.887180	0.517420
H	0.234885	−2.227851	−0.869750
H	4.124937	−7.205461	0.541794
H	5.853940	−6.409947	1.892908
H	5.858081	−4.749730	2.380114
P	4.974400	1.263657	1.385879
O	5.269054	1.447534	−0.180919
O	5.674838	2.169457	2.335461
O	5.398535	−0.286508	1.482933
C	5.656496	−0.881436	2.805600
C	6.960066	−1.666019	2.800079
O	6.820470	−2.926664	2.081390
C	7.690294	−2.955989	0.919226
C	8.152639	−0.945247	2.144516
C	8.175309	−1.516337	0.725614
O	9.324809	−1.305284	2.960828
H	4.825054	−1.552268	3.032776
H	5.715008	−0.088820	3.555556
H	7.194263	−1.902518	3.842967

H	8.517970	−3.641119	1.109146
H	8.063338	0.141558	2.166382
H	7.479062	−0.930475	0.123290
H	9.148173	−1.467776	0.244655
N	6.953917	−3.549802	−0.204915
C	7.205004	−4.902007	−0.488681
O	8.045084	−5.580527	0.099948
N	6.410506	−5.417838	−1.497557
C	5.402938	−4.766912	−2.227394
O	4.773947	−5.385941	−3.095369
C	5.188637	−3.377367	−1.852508
C	4.133557	−2.600879	−2.588123
C	5.948657	−2.853239	−0.858617
H	6.590605	−6.390668	−1.739183
H	4.322895	−2.627918	−3.667528
H	4.119407	−1.557173	−2.269192
H	3.140179	−3.035236	−2.428580
H	5.799378	−1.839118	−0.509882
P	10.876739	−1.217470	2.590055
O	10.952770	−0.203690	1.351231
O	11.701614	−0.902576	3.787636
O	11.235556	−2.613820	1.877190
C	11.303939	−3.829826	2.706006
C	12.056386	−4.917676	1.957650
O	11.297998	−5.369256	0.801645
C	11.982352	−5.022405	−0.427647
C	13.447154	−4.520836	1.430401
C	13.179704	−4.140857	−0.033568
O	14.291051	−5.693901	1.549595
H	10.282455	−4.159087	2.918036
H	11.819208	−3.598555	3.643117
H	12.151760	−5.765233	2.647124
H	12.289217	−5.948825	−0.920257
H	13.878031	−3.689081	1.997869
H	12.926680	−3.081059	−0.088682
H	14.044645	−4.322476	−0.675318
H	15.163271	−5.470188	1.176282
N	11.037031	−4.382737	−1.340465
C	10.629823	−3.054937	−1.372726
N	9.661988	−2.828282	−2.245037
C	9.405438	−4.072205	−2.820215
C	8.481044	−4.501519	−3.800528
N	7.596422	−3.668165	−4.388946
N	8.468445	−5.819051	−4.134264
C	9.331507	−6.653425	−3.521207
N	10.247086	−6.360629	−2.579498
C	10.243536	−5.052474	−2.265356
H	11.078847	−2.309551	−0.734010
H	6.986525	−4.015887	−5.118039
H	7.630745	−2.673718	−4.210235
H	9.275872	−7.696603	−3.828324
H	11.768554	0.334045	1.343075
H	5.161485	2.367528	−0.492422

(GTC)$^{H+}$

E: −13668.25 kcal mol^{-1}

O	−0.380174	0.367919	−0.207801
C	−0.251583	0.843546	1.152681
C	1.136766	0.520821	1.693988
O	1.315439	−0.927044	1.836658
C	2.354037	−1.387765	0.943069
C	2.307902	0.992275	0.811083
C	2.611890	−0.236446	−0.034256
O	3.404295	1.370466	1.740451
H	−1.317869	0.437341	−0.458459
H	−0.994146	0.361629	1.800871
H	−0.396991	1.932424	1.200504
H	1.219500	0.967175	2.690172
H	3.247549	−1.632839	1.521348
H	2.076798	1.882701	0.225979
H	1.891706	−0.264005	−0.854717
H	3.615475	−0.250809	−0.451808
N	1.971019	−2.637273	0.308627
C	0.955565	−2.889328	−0.614407
N	0.964243	−4.140502	−1.039113
C	2.033580	−4.746608	−0.374783
C	2.554481	−6.076859	−0.440476
O	2.166042	−7.044291	−1.119134
N	3.685940	−6.221920	0.415885
C	4.218175	−5.242574	1.231910
N	5.281155	−5.561836	2.014774
N	3.720508	−4.011752	1.284052
C	2.659625	−3.826624	0.475268
H	0.253536	−2.116763	−0.898966
H	4.098619	−7.152587	0.426712
H	5.801435	−6.412649	1.838592
H	5.807801	−4.768188	2.378189
P	4.984767	1.254221	1.519264
O	5.306515	1.456608	−0.039853
O	5.692246	2.127145	2.493946
O	5.378170	−0.306074	1.593457
C	5.622525	−0.928240	2.906605
C	6.937216	−1.694720	2.902657
O	6.819615	−2.948103	2.167902
C	7.682556	−2.952514	1.004118
C	8.121437	−0.944121	2.265857
C	8.184104	−1.511022	0.845305
O	9.294268	−1.266438	3.093279
H	4.796070	−1.614744	3.102089
H	5.657709	−0.153169	3.675773
H	7.167843	−1.942596	3.943622
H	8.502156	−3.654315	1.168162
H	7.998110	0.139346	2.283844
H	7.519402	−0.915316	0.217900
H	9.178936	−1.464956	0.410013
N	6.935202	−3.498371	−0.141004
C	7.191201	−4.832246	−0.499714
O	8.020348	−5.545744	0.062030
N	6.418282	−5.285749	−1.553995
C	5.447101	−4.582822	−2.281761
O	4.854902	−5.137953	−3.217460
C	5.221041	−3.218743	−1.827422
C	4.196200	−2.392671	−2.552224
C	5.949885	−2.759939	−0.778915
H	6.611648	−6.240127	−1.853305
H	4.413500	−2.373455	−3.626336
H	4.189775	−1.364537	−2.187386
H	3.192115	−2.817172	−2.438295
H	5.791243	−1.769036	−0.372152
P	10.839800	−1.044506	2.741491
O	10.824762	0.027295	1.551394
O	11.630824	−0.716605	3.958260
O	11.309685	−2.381778	1.980284
C	11.438815	−3.613989	2.780054
C	12.052589	−4.706414	1.926715
O	11.130909	−5.060388	0.859663
C	11.810817	−5.002160	−0.421635
C	13.403711	−4.357494	1.260910
C	13.029236	−4.093694	−0.203918
O	14.259483	−5.519160	1.412928
H	10.441262	−3.915534	3.114412
H	12.073942	−3.413274	3.647917
H	12.192834	−5.578395	2.578966
H	12.101378	−6.007424	−0.729293
H	13.873509	−3.485744	1.727810

H	12.768359	−3.039529	−0.325339
H	13.843987	−4.330035	−0.892434
H	15.113455	−5.313728	0.990182
N	10.849588	−4.540715	−1.428643
C	10.301039	−5.477118	−2.365221
O	10.702949	−6.656950	−2.359719
N	9.354678	−5.028465	−3.237564
C	8.932959	−3.757516	−3.201854
N	7.971818	−3.386992	−4.073732
C	9.440295	−2.810983	−2.252746
C	10.386547	−3.255716	−1.386835
H	7.643808	−4.045084	−4.770434
H	7.665822	−2.425381	−4.134044
H	9.081825	−1.789389	−2.220081
H	10.808749	−2.625589	−0.614215
H	11.652197	0.542874	1.482463
H	5.196952	2.377655	−0.347250

(GTG)$^{H+}$

E: −14231.56 kcal mol^{-1}

O	−0.376135	0.234132	−0.280840
C	−0.231944	0.753738	1.062105
C	1.168096	0.463141	1.590700
O	1.365278	−0.977475	1.774414
C	2.395211	−1.454467	0.879109
C	2.321620	0.920026	0.677149
C	2.628200	−0.330765	−0.135635
O	3.423451	1.339387	1.581589
H	−1.319867	0.275797	−0.514480
H	−0.958459	0.283984	1.736712
H	−0.389333	1.841895	1.078246
H	1.260831	0.940373	2.571580
H	3.299482	−1.673621	1.450929
H	2.072412	1.789496	0.068424
H	1.896913	−0.389268	−0.944713
H	3.626423	−0.348736	−0.567165
N	2.014430	−2.726796	0.290538
C	0.991283	−3.019358	−0.611582
N	0.998887	−4.287354	−0.983247
C	2.074525	−4.863382	−0.302624
C	2.595229	−6.195226	−0.313066
O	2.200179	−7.192911	−0.942264
N	3.735449	−6.301109	0.537997
C	4.273010	−5.286410	1.305867
N	5.344478	−5.568592	2.092888
N	3.773027	−4.055936	1.309494
C	2.705794	−3.907177	0.501946
H	0.285078	−2.260917	−0.922694
H	4.148255	−7.230488	0.586983
H	5.872826	−6.418442	1.936524
H	5.873537	−4.755495	2.408006
P	5.003182	1.233302	1.348897
O	5.306439	1.398323	−0.218298
O	5.708727	2.141845	2.292101
O	5.413880	−0.319294	1.463656
C	5.664197	−0.900611	2.794094
C	6.978545	−1.667306	2.812491
O	6.865543	−2.940166	2.110751
C	7.736205	−2.968494	0.949161
C	8.166872	−0.937959	2.159379
C	8.212884	−1.526618	0.748321
O	9.336777	−1.265476	2.992576
H	4.838893	−1.580711	3.015292
H	5.702472	−0.101643	3.538467
H	7.204692	−1.886522	3.860942
H	8.567262	−3.648163	1.143538
H	8.059827	0.147468	2.164722
H	7.522800	−0.951490	0.129155
H	9.192006	−1.477425	0.280713
N	7.005009	−3.572460	−0.174290
C	7.236718	−4.934131	−0.424303
O	8.050275	−5.617169	0.195034
N	6.451410	−5.457412	−1.437738
C	5.460949	−4.807425	−2.193496
O	4.837978	−5.436762	−3.058334
C	5.258283	−3.410145	−1.844151
C	4.219926	−2.632773	−2.602048
C	6.014391	−2.877539	−0.851892
H	6.598999	−6.445396	−1.634989
H	4.426098	−2.673162	−3.677848
H	4.210997	−1.585454	−2.293757
H	3.220463	−3.056408	−2.450997
H	5.871328	−1.855908	−0.523479
P	10.889630	−1.164250	2.627787
O	10.958021	−0.150719	1.388316
O	11.705561	−0.837140	3.828405
O	11.266417	−2.559007	1.921566
C	11.327064	−3.771539	2.755962
C	12.036285	−4.878816	1.994696
O	11.245103	−5.299274	0.849195
C	11.933558	−4.992157	−0.388697
C	13.431723	−4.524210	1.449039
C	13.157449	−4.140343	−0.012330
O	14.242000	−5.721765	1.561454
H	10.303883	−4.077802	2.993651
H	11.868126	−3.546824	3.680114
H	12.116481	−5.730988	2.680619
H	12.213426	−5.934188	−0.867839
H	13.894188	−3.704455	2.009005
H	12.930061	−3.074709	−0.065637
H	14.009933	−4.344642	−0.663909
H	15.118041	−5.522932	1.182926
N	11.003602	−4.339184	−1.307060
C	10.648457	−2.991843	−1.369116
N	9.695820	−2.754858	−2.249807
C	9.391934	−4.000543	−2.797922
C	8.443577	−4.367376	−3.801440
O	7.647494	−3.650656	−4.432687
N	8.471221	−5.771428	−4.032939
C	9.299424	−6.679936	−3.403658
N	9.144459	−7.989911	−3.726180
N	10.185508	−6.322669	−2.477831
C	10.190984	−4.997401	−2.218373
H	11.124995	−2.250554	−0.745999
H	7.814096	−6.105434	−4.735138
H	8.668602	−8.249161	−4.582028
H	9.854812	−8.630203	−3.390918
H	11.782118	0.373827	1.364335
H	5.200468	2.314440	−0.541879

(GTT)$^{H+}$

E: −13927.29 kcal mol^{-1}

O	−0.306419	0.545086	−0.064505
C	−0.082413	0.988215	1.294347
C	1.317012	0.591583	1.750641
O	1.430815	−0.866072	1.858081
C	2.415529	−1.353210	0.917637
C	2.457389	1.025016	0.808183
C	2.661934	−0.204953	−0.064792
O	3.618910	1.354325	1.677093
H	−1.252828	0.657793	−0.260733
H	−0.809798	0.528264	1.974755

H	−0.174148	2.081419	1.368204
H	1.478114	1.014988	2.747305
H	3.326189	−1.622379	1.455467
H	2.231399	1.930942	0.245793
H	1.892369	−0.193821	−0.839829
H	3.636764	−0.254006	−0.543186
N	1.975098	−2.591791	0.301988
C	0.946608	−2.815058	−0.614109
N	0.903485	−4.070502	−1.022838
C	1.949876	−4.710883	−0.354017
C	2.415613	−6.062035	−0.404723
O	1.984876	−7.021065	−1.069241
N	3.543628	−6.242408	0.449563
C	4.116932	−5.276157	1.253291
N	5.167208	−5.629654	2.036304
N	3.670827	−4.024731	1.290799
C	2.616090	−3.806505	0.481719
H	0.273668	−2.020794	−0.908292
H	3.918637	−7.188602	0.469975
H	5.635872	−6.515687	1.894265
H	5.723080	−4.859996	2.406840
P	5.180163	1.129073	1.411960
O	5.473818	1.259007	−0.159460
O	5.972559	1.980859	2.338653
O	5.469998	−0.452435	1.526539
C	5.624154	−1.058697	2.861220
C	6.928964	−1.836608	2.952292
O	6.843840	−3.109062	2.247098
C	7.746122	−3.147349	1.115882
C	8.152452	−1.109948	2.366760
C	8.319011	−1.729189	0.972840
O	9.268387	−1.394081	3.278327
H	4.780430	−1.735686	3.010209
H	5.616638	−0.274961	3.622501
H	7.097229	−2.060354	4.010337
H	8.528941	−3.882899	1.304296
H	8.021651	−0.028096	2.328586
H	7.752594	−1.127247	0.261086
H	9.356845	−1.751843	0.645872
N	7.011818	−3.661984	−0.057011
C	7.203752	−5.011219	−0.395815
O	8.013219	−5.751768	0.161164
N	6.394168	−5.449993	−1.429741
C	5.435767	−4.721016	−2.153314
O	4.785030	−5.275531	−3.047470
C	5.296564	−3.333749	−1.737022
C	4.316223	−2.468694	−2.476684
C	6.056029	−2.891136	−0.703907
H	6.516661	−6.427077	−1.690969
H	4.521787	−2.501161	−3.552693
H	4.381787	−1.430136	−2.147479
H	3.288200	−2.822125	−2.337176
H	5.945221	−1.886684	−0.314360
P	10.794660	−0.943363	3.039437
O	10.771557	0.208763	1.925595
O	11.455671	−0.639738	4.337170
O	11.454528	−2.121867	2.174407
C	11.590410	−3.440429	2.816086
C	12.087657	−4.437015	1.792611
O	11.050969	−4.641811	0.795182
C	11.699197	−4.809567	−0.488504
C	13.384814	−4.043040	1.031235
C	12.923082	−3.887836	−0.428916
O	14.323279	−5.132527	1.209835
H	10.614712	−3.751650	3.201239
H	12.303252	−3.361506	3.642072
H	12.275512	−5.381625	2.321698
H	11.990356	−5.852317	−0.632863
H	13.816104	−3.115653	1.418679
H	12.641715	−2.847623	−0.613555
H	13.698192	−4.174036	−1.143545
H	15.168851	−4.862950	0.806682
N	10.712943	−4.517472	−1.530050
C	10.204141	−5.582283	−2.285087
O	10.586982	−6.749482	−2.177074
N	9.217250	−5.208034	−3.180702
C	8.682422	−3.928509	−3.398903
O	7.790369	−3.767029	−4.243992
C	9.247226	−2.885373	−2.555678
C	8.746630	−1.476652	−2.713311
C	10.219826	−3.233599	−1.676370
H	8.849889	−5.959511	−3.761995
H	8.945455	−1.107837	−3.726524
H	9.232387	−0.805807	−2.000084
H	7.661515	−1.429062	−2.566522
H	10.673925	−2.500885	−1.021444
H	10.991961	1.089495	2.286464
H	5.490560	2.181841	−0.480640

(TAA)$^{H+}$

E: −13902.74 kcal mol^{-1}

O	−0.094236	−0.416712	−0.610688
C	−0.123103	0.018733	0.770094
C	1.255968	−0.135521	1.398820
O	1.592840	−1.547100	1.598218
C	2.748517	−1.911751	0.807966
C	2.415161	0.454096	0.574739
C	2.975958	−0.751971	−0.170883
O	3.362359	1.022238	1.560556
H	−1.012283	−0.444598	−0.932482
H	−0.838632	−0.578496	1.348607
H	−0.414308	1.076676	0.834231
H	1.225861	0.339381	2.384851
H	3.606719	−2.072403	1.463141
H	2.105627	1.266018	−0.083511
H	2.388838	−0.887793	−1.081690
H	4.019754	−0.653576	−0.456850
N	2.505472	−3.209690	0.161257
C	3.282145	−4.306028	0.553173
O	4.167336	−4.252807	1.409188
N	2.980714	−5.471508	−0.131633
C	1.991848	−5.672979	−1.104836
O	1.857415	−6.793499	−1.623769
C	1.190865	−4.497200	−1.408292
C	0.074090	−4.615909	−2.409244
C	1.479590	−3.339405	−0.761243
H	3.554066	−6.278196	0.107692
H	−0.655004	−5.371581	−2.092097
H	0.458176	−4.930277	−3.387534
H	−0.442478	−3.659389	−2.528459
H	0.897029	−2.436089	−0.915821
P	4.854261	1.523490	1.286323
O	4.958745	1.696296	−0.303553
O	5.201689	2.699341	2.129805
O	5.800858	0.238443	1.512955
C	6.003811	−0.248014	2.884857
C	7.235675	−1.137621	2.944984
O	7.031673	−2.390252	2.236570
C	7.847134	−2.448171	1.039714
C	8.515762	−0.522688	2.350261
C	8.559589	−1.090948	0.931937
O	9.619255	−0.961970	3.225559
H	5.121024	−0.818932	3.186317
H	6.137613	0.606235	3.555399
H	7.395777	−1.377789	4.001535

H	8.545722	−3.283906	1.145195
H	8.516468	0.567887	2.366760
H	8.008541	−0.403243	0.290370
H	9.565342	−1.180986	0.529807
N	7.015308	−2.775176	−0.114452
C	6.299212	−1.927140	−0.951924
N	5.584038	−2.575012	−1.856197
C	5.830666	−3.924552	−1.605996
C	5.360885	−5.113343	−2.209134
N	4.500871	−5.116370	−3.250544
N	5.802252	−6.298487	−1.713295
C	6.666908	−6.290387	−0.679065
N	7.170437	−5.229230	−0.024361
C	6.716465	−4.069793	−0.527092
H	6.342620	−0.853545	−0.850345
H	4.132784	−5.993607	−3.596182
H	4.106924	−4.250826	−3.593568
H	6.994108	−7.268943	−0.331954
P	11.173070	−1.092244	2.881924
O	11.404106	−0.024921	1.709152
O	12.003343	−0.961694	4.109788
O	11.353857	−2.488163	2.101829
C	11.272575	−3.733386	2.885344
C	11.734308	−4.897669	2.029902
O	10.785389	−5.116059	0.945683
C	11.479446	−5.104819	−0.327759
C	13.126338	−4.732591	1.381279
C	12.803196	−4.369700	−0.073744
O	13.798905	−6.012480	1.494870
H	10.233279	−3.880919	3.194375
H	11.910991	−3.643526	3.769202
H	11.741647	−5.785815	2.674523
H	11.638218	−6.133733	−0.665661
H	13.716069	−3.954723	1.876827
H	12.667511	−3.288598	−0.154149
H	13.587327	−4.677765	−0.769125
H	14.678909	−5.921022	1.085328
N	10.615565	−4.483004	−1.318936
C	10.311308	−3.133743	−1.450280
N	9.412900	−2.891715	−2.388733
C	9.098704	−4.146926	−2.909489
C	8.204857	−4.568740	−3.919486
N	7.423358	−3.708349	−4.617614
N	8.143671	−5.893588	−4.208103
C	8.912430	−6.748617	−3.507264
N	9.773569	−6.468520	−2.511482
C	9.833656	−5.149941	−2.255720
H	10.789591	−2.387106	−0.833694
H	6.676649	−4.098065	−5.181932
H	7.292675	−2.768349	−4.263240
H	8.818257	−7.798933	−3.778229
H	12.343321	0.203912	1.564565
H	5.485069	2.475615	−0.568956

(TAC)$^{H+}$

E: −13503.70 kcal mol^{-1}

O	−0.061638	−0.328863	−0.567175
C	−0.086986	0.092898	0.817880
C	1.290523	−0.079700	1.445612
O	1.617882	−1.497066	1.621010
C	2.773056	−1.853833	0.825922
C	2.455033	0.515293	0.632713
C	3.000771	−0.681437	−0.137396
O	3.409525	1.051376	1.630013
H	−0.979907	−0.346218	−0.889068
H	−0.807453	−0.504431	1.390185
H	−0.369516	1.152439	0.893053
H	1.262039	0.378824	2.439403
H	3.631311	−2.022908	1.479012
H	2.154195	1.344821	−0.007421
H	2.402681	−0.798247	−1.043530
H	4.041360	−0.584197	−0.433611
N	2.530177	−3.142901	0.162691
C	3.305237	−4.244344	0.543879
O	4.174504	−4.206172	1.416572
N	3.022738	−5.395405	−0.172835
C	2.043357	−5.582645	−1.158681
O	1.920039	−6.692976	−1.701293
C	1.236717	−4.405918	−1.443700
C	0.126301	−4.512615	−2.453121
C	1.513137	−3.259687	−0.771210
H	3.590890	−6.206628	0.063621
H	−0.598263	−5.280630	−2.155686
H	0.517915	−4.803579	−3.435556
H	−0.397584	−3.558096	−2.554876
H	0.926638	−2.356737	−0.912447
P	4.911552	1.534634	1.386211
O	5.045009	1.706808	−0.201617
O	5.258666	2.705643	2.236496
O	5.839734	0.238880	1.633680
C	6.003779	−0.248226	3.010889
C	7.204338	−1.177209	3.098120
O	6.969139	−2.426984	2.395448
C	7.815328	−2.537355	1.226516
C	8.519131	−0.609970	2.531070
C	8.609174	−1.221828	1.130815
O	9.579893	−1.053760	3.453740
H	5.097675	−0.789169	3.298805
H	6.152699	0.604688	3.679911
H	7.335068	−1.414993	4.159437
H	8.465526	−3.407463	1.348525
H	8.540698	0.480436	2.512223
H	8.145785	−0.526415	0.430359
H	9.632440	−1.393946	0.802525
N	7.004502	−2.820035	0.043991
C	6.302428	−1.929225	−0.761255
N	5.619686	−2.527159	−1.722456
C	5.876255	−3.886156	−1.549003
C	5.440555	−5.037616	−2.243287
N	4.622217	−4.978708	−3.316612
N	5.870153	−6.249347	−1.805209
C	6.696212	−6.299614	−0.741112
N	7.171401	−5.278091	−0.006778
C	6.729379	−4.090545	−0.452957
H	6.329941	−0.864314	−0.588453
H	4.268385	−5.833966	−3.726741
H	4.236523	−4.094546	−3.619588
H	7.015331	−7.295727	−0.440124
P	11.160913	−0.969361	3.176193
O	11.393277	0.165807	2.067902
O	11.900365	−0.837211	4.460832
O	11.515831	−2.258013	2.289330
C	11.354365	−3.582202	2.912422
C	11.628264	−4.651998	1.878122
O	10.572808	−4.629897	0.882050
C	11.166052	−4.999215	−0.390281
C	12.980620	−4.535192	1.117254
C	12.562275	−4.363055	−0.354385
O	13.705906	−5.766471	1.359989
H	10.332290	−3.675362	3.292041
H	12.062690	−3.673082	3.741249
H	11.616440	−5.619179	2.400860
H	11.217530	−6.085981	−0.483661
H	13.570537	−3.684865	1.470625
H	12.505584	−3.298372	−0.600494

H	13.260334	−4.845051	−1.042981
H	14.596116	−5.664865	0.976299
N	10.278693	−4.544360	−1.457193
C	9.648504	−5.505696	−2.315753
O	9.904425	−6.716657	−2.170076
N	8.794773	−5.042416	−3.268836
C	8.549029	−3.731913	−3.397503
N	7.694444	−3.346435	−4.369094
C	9.167628	−2.750708	−2.554699
C	10.018820	−3.209299	−1.602444
H	7.181351	−4.052988	−4.883202
H	7.373190	−2.388923	−4.422333
H	8.961444	−1.692838	−2.662024
H	10.521634	−2.550181	−0.906162
H	11.776154	0.983971	2.440065
H	5.635205	2.441459	−0.459576

(TAG)$^{H+}$

E: −14067.62 kcal mol^{-1}

O	−0.037000	−0.475323	−0.613120
C	−0.053524	−0.032176	0.765308
C	1.331345	−0.181779	1.382231
O	1.670069	−1.592007	1.588005
C	2.819861	−1.962127	0.792341
C	2.483609	0.404193	0.544958
C	3.044151	−0.807335	−0.193098
O	3.431833	0.990781	1.518012
H	−0.957932	−0.505996	−0.926312
H	−0.763335	−0.626545	1.353762
H	−0.345070	1.025881	0.826272
H	1.309571	0.299221	2.365525
H	3.681374	−2.122763	1.443101
H	2.165235	1.206520	−0.120862
H	2.454206	−0.949327	−1.101169
H	4.087305	−0.712132	−0.483718
N	2.571297	−3.261813	0.151082
C	3.350457	−4.357279	0.540211
O	4.236426	−4.303614	1.395332
N	3.049755	−5.522317	−0.145495
C	2.057777	−5.724880	−1.114879
O	1.924020	−6.845210	−1.634721
C	1.250587	−4.551537	−1.411282
C	0.127124	−4.672347	−2.404430
C	1.540316	−3.393395	−0.765528
H	3.628710	−6.326861	0.087330
H	−0.593372	−5.436133	−2.086995
H	0.505927	−4.976444	−3.388051
H	−0.398227	−3.719284	−2.512385
H	0.954829	−2.491345	−0.916342
P	4.927520	1.477977	1.227908
O	5.059306	1.619338	−0.362944
O	5.277169	2.666786	2.052163
O	5.859973	0.187522	1.473368
C	6.009323	−0.305365	2.850131
C	7.241404	−1.190279	2.954924
O	7.066630	−2.449098	2.251527
C	7.916093	−2.513276	1.078313
C	8.536298	−0.571911	2.398102
C	8.642575	−1.161061	0.989856
O	9.609039	−0.990537	3.318837
H	5.116759	−0.879414	3.114549
H	6.114832	0.546053	3.529527
H	7.368878	−1.421562	4.017934
H	8.606109	−3.352789	1.201689
H	8.524810	0.518768	2.396002
H	8.134409	−0.478278	0.308791
H	9.667265	−1.272594	0.643429
N	7.113914	−2.836265	−0.099252
C	6.415149	−1.986447	−0.951419
N	5.703548	−2.633167	−1.858405
C	5.932186	−3.983093	−1.596226
C	5.458257	−5.171548	−2.197559
N	4.614758	−5.176510	−3.250823
N	5.867471	−6.358411	−1.676186
C	6.705857	−6.351528	−0.620517
N	7.216799	−5.291385	0.028772
C	6.798923	−4.130526	−0.501624
H	6.468475	−0.912567	−0.856536
H	4.291432	−6.054908	−3.635150
H	4.322152	−4.310887	−3.683016
H	7.004394	−7.331442	−0.251797
P	11.184080	−0.997941	3.034760
O	11.409496	0.090052	1.880228
O	11.955210	−0.827722	4.296163
O	11.475517	−2.363247	2.237988
C	11.300736	−3.633628	2.959934
C	11.656069	−4.780552	2.035539
O	10.672740	−4.859015	0.962652
C	11.362725	−4.968539	−0.309675
C	13.049408	−4.683651	1.368307
C	12.726637	−4.304854	−0.082994
O	13.662311	−5.993862	1.476095
H	10.258528	−3.716281	3.282936
H	11.957985	−3.641976	3.834689
H	11.610343	−5.702513	2.629753
H	11.461989	−6.023685	−0.584775
H	13.681936	−3.935664	1.856486
H	12.642138	−3.217883	−0.161670
H	13.484146	−4.653713	−0.788717
H	14.552893	−5.935817	1.083863
N	10.543300	−4.355516	−1.339220
C	10.347023	−2.994497	−1.577864
N	9.503869	−2.767827	−2.565099
C	9.115756	−4.031946	−3.007616
C	8.229697	−4.416724	−4.060448
O	7.578485	−3.702561	−4.842695
N	8.136906	−5.832402	−4.150761
C	8.811101	−6.741133	−3.361115
N	8.568596	−8.057478	−3.579948
N	9.638808	−6.370590	−2.387528
C	9.749810	−5.031324	−2.255113
H	10.859757	−2.238704	−1.002018
H	7.508678	−6.176627	−4.873663
H	8.141370	−8.360394	−4.446556
H	9.179548	−8.720308	−3.117481
H	12.232018	0.604801	1.996011
H	5.401861	2.492262	−0.636964

(TAT)$^{H+}$

E: −13762.84 kcal mol^{-1}

O	−0.124175	−0.352689	−0.431382
C	−0.110650	0.061893	0.955917
C	1.284071	−0.111617	1.544692
O	1.620550	−1.529404	1.699232
C	2.755604	−1.874796	0.870201
C	2.425056	0.493099	0.706421
C	2.948485	−0.694989	−0.091545
O	3.408091	1.014511	1.683866
H	−1.051520	−0.373405	−0.725803
H	−0.813875	−0.539600	1.545088
H	−0.392498	1.120640	1.044527
H	1.280572	0.338618	2.542633

H	3.632802	−2.041722	1.498236
H	2.107583	1.330758	0.085223
H	2.320555	−0.806915	−0.977835
H	3.977717	−0.591666	−0.422376
N	2.501997	−3.162070	0.208142
C	3.294815	−4.259940	0.561363
O	4.192944	−4.218488	1.404478
N	2.994327	−5.411793	−0.146533
C	1.985018	−5.602344	−1.101252
O	1.846436	−6.713956	−1.637431
C	1.167916	−4.427454	−1.362724
C	0.031139	−4.534926	−2.342138
C	1.459231	−3.281222	−0.696611
H	3.570902	−6.221599	0.073641
H	−0.683419	−5.305390	−2.027334
H	0.397217	−4.822346	−3.335551
H	−0.497536	−3.581447	−2.427596
H	0.865514	−2.380338	−0.819547
P	4.899288	1.511790	1.401989
O	5.006494	1.681345	−0.188131
O	5.256860	2.686703	2.242475
O	5.841303	0.221631	1.628342
C	6.018287	−0.279688	2.998896
C	7.224169	−1.203400	3.067848
O	6.985112	−2.456624	2.371726
C	7.813974	−2.568850	1.192308
C	8.526874	−0.629038	2.480054
C	8.610066	−1.254800	1.084950
O	9.602581	−1.049300	3.395055
H	5.116886	−0.826830	3.289398
H	6.169876	0.567048	3.675216
H	7.372522	−1.439340	4.127155
H	8.462834	−3.441549	1.303313
H	8.534959	0.461132	2.447670
H	8.147306	−0.566021	0.377488
H	9.632545	−1.433303	0.757772
N	6.984999	−2.843614	0.019635
C	6.231844	−1.946761	−0.730955
N	5.528266	−2.531275	−1.684174
C	5.820025	−3.888319	−1.560622
C	5.372414	−5.029347	−2.266023
N	4.500101	−4.959796	−3.291964
N	5.836006	−6.245561	−1.871674
C	6.691623	−6.309817	−0.831214
N	7.180932	−5.298411	−0.091637
C	6.712218	−4.106229	−0.498727
H	6.241782	−0.888181	−0.522012
H	4.189971	−5.806237	−3.752083
H	4.152237	−4.067085	−3.614608
H	7.029266	−7.308529	−0.561207
P	11.179068	−0.941395	3.098489
O	11.377601	0.168757	1.959516
O	11.930003	−0.766673	4.371100
O	11.546975	−2.244478	2.237435
C	11.405769	−3.556183	2.889876
C	11.682659	−4.644289	1.876243
O	10.619862	−4.649003	0.885313
C	11.207139	−5.022996	−0.384049
C	13.029068	−4.533462	1.104847
C	12.602581	−4.385295	−0.367543
O	13.761296	−5.756963	1.361535
H	10.388157	−3.653076	3.280201
H	12.122266	−3.622528	3.713871
H	11.679120	−5.601355	2.416444
H	11.260995	−6.110332	−0.472902
H	13.616643	−3.674516	1.440689
H	12.547881	−3.325182	−0.630570
H	13.295818	−4.879740	−1.051770
H	14.649299	−5.656650	0.972370
N	10.308001	−4.572213	−1.446784
C	9.651225	−5.532531	−2.225277
O	9.804962	−6.749085	−2.095216
N	8.801248	−4.992172	−3.174198
C	8.555464	−3.637902	−3.448727
O	7.792931	−3.324783	−4.374341
C	9.250966	−2.707306	−2.570880
C	9.042661	−1.230490	−2.759794
C	10.080446	−3.220044	−1.628834
H	8.325171	−5.670297	−3.766262
H	9.402608	−0.911374	−3.745597
H	9.578205	−0.660382	−1.996009
H	7.978022	−0.977703	−2.710509
H	10.623208	−2.578610	−0.945901
H	11.734627	1.009107	2.307333
H	5.503689	2.479987	−0.452530

(TCA)$^{H+}$

E: −13501.08 kcal mol^{-1}

O	−0.248935	−0.308911	−0.271996
C	−0.209751	0.043259	1.132297
C	1.207095	−0.110724	1.669653
O	1.582845	−1.522344	1.770487
C	2.699711	−1.823377	0.904460
C	2.299797	0.555341	0.812524
C	2.876854	−0.597308	−0.005903
O	3.268496	1.140122	1.763346
H	−1.182512	−0.340016	−0.544952
H	−0.876508	−0.606505	1.712445
H	−0.521528	1.087368	1.275725
H	1.226812	0.308717	2.680771
H	3.589328	−2.024910	1.504285
H	1.919340	1.368657	0.194363
H	2.285282	−0.696967	−0.918161
H	3.914679	−0.446132	−0.292188
N	2.429833	−3.075125	0.177541
C	3.225180	−4.194108	0.452594
O	4.132178	−4.207749	1.286558
N	2.916519	−5.298612	−0.323122
C	1.907188	−5.425138	−1.286044
O	1.774516	−6.494680	−1.904203
C	1.082990	−4.239029	−1.460137
C	−0.060213	−4.282502	−2.437187
C	1.377622	−3.138879	−0.722015
H	3.512923	−6.110589	−0.174861
H	−0.769843	−5.076077	−2.173022
H	0.300403	−4.497310	−3.450710
H	−0.592490	−3.327355	−2.452451
H	0.779912	−2.234176	−0.780397
P	4.698304	1.761040	1.393544
O	4.677318	2.009195	−0.189250
O	5.026154	2.922239	2.264803
O	5.734289	0.531469	1.482737
C	5.996818	−0.041787	2.811763
C	7.102424	−1.076462	2.712106
O	6.646407	−2.226075	1.950732
C	7.558594	−2.486884	0.846156
C	8.402119	−0.591791	2.034118
C	8.305933	−1.167259	0.624168
O	9.496336	−1.184753	2.824892
H	5.081346	−0.514796	3.179207
H	6.298138	0.758470	3.494206
H	7.329186	−1.396027	3.736139
H	8.238455	−3.297840	1.115733
H	8.522965	0.491629	2.055685
H	7.722563	−0.468125	0.020945

H	9.267743	−1.305126	0.138057
N	6.785694	−2.989033	−0.287736
C	6.760358	−4.408317	−0.533879
O	7.454258	−5.156818	0.172618
N	5.975716	−4.862687	−1.550888
C	5.224719	−4.021938	−2.269572
N	4.473301	−4.539952	−3.263612
C	5.184671	−2.612643	−2.005132
C	5.974940	−2.152712	−1.001298
H	4.495221	−5.535911	−3.444863
H	3.884393	−3.953060	−3.838243
H	4.552034	−1.942412	−2.574624
H	5.995290	−1.108588	−0.716402
P	11.068193	−1.145414	2.539971
O	11.232462	−0.220805	1.240833
O	11.850884	−0.765632	3.748360
O	11.420218	−2.598103	1.948784
C	11.348612	−3.759643	2.849673
C	11.896551	−4.987665	2.142378
O	11.022541	−5.382175	1.046649
C	11.704705	−5.247079	−0.225599
C	13.304750	−4.832822	1.539249
C	13.029587	−4.519323	0.062529
O	13.980919	−6.103768	1.716542
H	10.301844	−3.922999	3.122877
H	11.937939	−3.552860	3.748492
H	11.900316	−5.800475	2.878734
H	11.860456	−6.245676	−0.642848
H	13.872831	−4.034940	2.029020
H	12.920469	−3.440752	−0.063152
H	13.832052	−4.864121	−0.593407
H	14.861450	−6.029402	1.304922
N	10.832984	−4.554622	−1.169233
C	10.570827	−3.193027	−1.250930
N	9.663882	−2.891370	−2.164807
C	9.298125	−4.117561	−2.718170
C	8.380836	−4.473537	−3.731845
N	7.648886	−3.559172	−4.412849
N	8.251855	−5.787570	−4.047671
C	8.992043	−6.691362	−3.377129
N	9.887540	−6.472813	−2.395802
C	10.006138	−5.164507	−2.106883
H	11.075321	−2.483461	−0.612274
H	6.889303	−3.882877	−4.999882
H	7.618766	−2.601928	−4.085893
H	8.845260	−7.730378	−3.668140
H	12.064412	0.291739	1.238430
H	4.966774	2.909695	−0.433749

(TCC)$^{H+}$

E: −13102.26 kcal mol^{-1}

O	−0.256674	−0.211111	−0.369136
C	−0.270668	0.150705	1.032955
C	1.121501	−0.013628	1.629575
O	1.484094	−1.428449	1.743180
C	2.629870	−1.731643	0.915541
C	2.253513	0.642105	0.818210
C	2.827072	−0.511595	0.002706
O	3.207523	1.183680	1.811516
H	−1.178547	−0.231027	−0.680308
H	−0.966728	−0.488464	1.590023
H	−0.577492	1.198716	1.157065
H	1.101981	0.404610	2.641075
H	3.501055	−1.920693	1.545661
H	1.914371	1.477484	0.205718
H	2.236767	−0.606468	−0.910893
H	3.866239	−0.366262	−0.279350
N	2.392060	−2.990725	0.192156
C	3.203807	−4.093680	0.484054
O	4.094037	−4.088661	1.336109
N	2.933481	−5.203706	−0.297794
C	1.948029	−5.349058	−1.282931
O	1.850334	−6.420464	−1.904188
C	1.104670	−4.179204	−1.475005
C	−0.016241	−4.245952	−2.476021
C	1.362041	−3.073383	−0.731560
H	3.539812	−6.005495	−0.134806
H	−0.719330	−5.048762	−2.221956
H	0.369365	−4.462260	−3.479921
H	−0.562445	−3.299175	−2.508744
H	0.751289	−2.178487	−0.805707
P	4.684428	1.726910	1.539594
O	4.716733	2.010775	−0.036789
O	5.034820	2.847615	2.453702
O	5.663818	0.450491	1.659231
C	5.942479	−0.081192	3.002256
C	7.090644	−1.072755	2.934602
O	6.695088	−2.256027	2.190651
C	7.585862	−2.475354	1.065226
C	8.380171	−0.547446	2.269533
C	8.334985	−1.149555	0.867295
O	9.489963	−1.051667	3.100980
H	5.043647	−0.582755	3.372750
H	6.205382	0.745286	3.669040
H	7.313279	−1.366963	3.966823
H	8.264644	−3.299821	1.291986
H	8.449530	0.540789	2.265098
H	7.776880	−0.460426	0.230351
H	9.319442	−1.284411	0.426215
N	6.799300	−2.929127	−0.084145
C	6.800013	−4.329173	−0.419353
O	7.490895	−5.111991	0.252547
N	6.045531	−4.729032	−1.481331
C	5.292095	−3.856429	−2.159183
N	4.563129	−4.320615	−3.194449
C	5.222301	−2.468795	−1.803174
C	5.985539	−2.061871	−0.756851
H	4.609466	−5.300752	−3.444933
H	3.991023	−3.702318	−3.753091
H	4.587465	−1.774479	−2.339973
H	5.978194	−1.039896	−0.400254
P	11.046531	−1.099863	2.743825
O	11.195990	−0.048478	1.543832
O	11.887564	−0.897393	3.954814
O	11.297017	−2.501565	1.993334
C	11.306567	−3.732465	2.804393
C	11.830408	−4.885149	1.969148
O	10.886550	−5.188128	0.904674
C	11.562640	−5.178733	−0.378938
C	13.204535	−4.654382	1.300665
C	12.851171	−4.369514	−0.164953
O	13.959375	−5.883672	1.458871
H	10.282852	−3.938374	3.130668
H	11.949507	−3.581470	3.676260
H	11.900719	−5.754254	2.636002
H	11.769894	−6.201885	−0.694639
H	13.746608	−3.822480	1.762106
H	12.672861	−3.298863	−0.294286
H	13.644276	−4.673318	−0.852465
H	14.822891	−5.757688	1.024444
N	10.638990	−4.633022	−1.379547
C	10.075974	−5.492344	−2.376386
O	10.414470	−6.693236	−2.414211
N	9.187528	−4.953640	−3.256992
C	8.829457	−3.665263	−3.172371

N	7.941812	−3.196959	−4.073162
C	9.351898	−2.793204	−2.161935
C	10.241603	−3.327955	−1.288116
H	7.536177	−3.825787	−4.755370
H	7.608161	−2.243763	−4.032749
H	9.044668	−1.757188	−2.091330
H	10.669172	−2.763202	−0.469083
H	12.118000	0.224249	1.369936
H	5.393730	2.663670	−0.302141

(TCG)$^{H+}$

E: −13665.71 kcal mol^{-1}

O	−0.201759	−0.386466	−0.331239
C	−0.168674	−0.015102	1.068039
C	1.245329	−0.163224	1.614382
O	1.620841	−1.573998	1.729742
C	2.746042	−1.879358	0.875832
C	2.343467	0.495066	0.758011
C	2.921659	−0.664474	−0.048922
O	3.308754	1.082206	1.711055
H	−1.134091	−0.418908	−0.608312
H	−0.838756	−0.656165	1.653997
H	−0.479996	1.031145	1.196286
H	1.259138	0.265570	2.621697
H	3.632309	−2.067257	1.484824
H	1.968575	1.306335	0.133832
H	2.327188	−0.775172	−0.958134
H	3.958716	−0.516300	−0.340290
N	2.489868	−3.141001	0.162414
C	3.300196	−4.246880	0.446281
O	4.204780	−4.242494	1.282906
N	3.008371	−5.360296	−0.323182
C	2.003147	−5.506083	−1.287792
O	1.887838	−6.580524	−1.901076
C	1.161859	−4.333323	−1.469711
C	0.020916	−4.398638	−2.448154
C	1.440318	−3.224370	−0.738553
H	3.617126	−6.162209	−0.170535
H	−0.679368	−5.198808	−2.178851
H	0.385866	−4.617361	−3.459212
H	−0.523149	−3.450313	−2.471526
H	0.831272	−2.327722	−0.804280
P	4.748927	1.683526	1.352897
O	4.750942	1.908000	−0.233473
O	5.076822	2.854868	2.210588
O	5.772954	0.446252	1.477611
C	6.024572	−0.094066	2.823007
C	7.160039	−1.099004	2.763565
O	6.744745	−2.282685	2.033486
C	7.666296	−2.556708	0.941964
C	8.452732	−0.591496	2.087347
C	8.421566	−1.242612	0.706824
O	9.557830	−1.058168	2.945843
H	5.114505	−0.583652	3.182155
H	6.291500	0.726855	3.495348
H	7.385366	−1.383174	3.798313
H	8.338219	−3.370096	1.224124
H	8.515287	0.496334	2.043958
H	7.872997	−0.574785	0.039358
H	9.404995	−1.404027	0.274461
N	6.903844	−3.062051	−0.200066
C	6.865341	−4.482092	−0.434309
O	7.520116	−5.234792	0.303923
N	6.112329	−4.934740	−1.476623
C	5.378850	−4.091673	−2.211600
N	4.644894	−4.608396	−3.218594
C	5.329647	−2.683941	−1.941873
C	6.102835	−2.224625	−0.925588
H	4.671765	−5.603405	−3.403063
H	4.101123	−4.015139	−3.830083
H	4.705877	−2.013682	−2.520944
H	6.114121	−1.181437	−0.636349
P	11.122290	−1.072471	2.617540
O	11.290359	−0.130322	1.331659
O	11.935686	−0.739878	3.819361
O	11.417138	−2.522606	1.989926
C	11.300556	−3.694217	2.873054
C	11.790307	−4.929667	2.138724
O	10.905961	−5.239020	1.025271
C	11.616911	−5.130788	−0.233656
C	13.211772	−4.831335	1.553961
C	12.968628	−4.469498	0.082677
O	13.822596	−6.138107	1.707579
H	10.249266	−3.816759	3.150099
H	11.903177	−3.528941	3.771594
H	11.744737	−5.762181	2.851759
H	11.734720	−6.134896	−0.650669
H	13.813267	−4.074489	2.068363
H	12.908651	−3.384290	−0.013234
H	13.762829	−4.832509	−0.573532
H	14.712549	−6.096126	1.311887
N	10.797188	−4.395679	−1.190851
C	10.669956	−3.015446	−1.343662
N	9.800215	−2.683866	−2.278360
C	9.316962	−3.896173	−2.769123
C	8.376925	−4.169026	−3.809628
O	7.746642	−3.373645	−4.529605
N	8.202074	−5.568702	−3.978083
C	8.830769	−6.555872	−3.249026
N	8.481207	−7.844164	−3.513750
N	9.715554	−6.291316	−2.292408
C	9.920098	−4.970152	−2.099348
H	11.240237	−2.322978	−0.742837
H	7.531476	−5.835197	−4.695616
H	8.044326	−8.070935	−4.399499
H	9.077374	−8.561800	−3.116816
H	12.130547	0.368315	1.329548
H	5.079089	2.792290	−0.488297

(TCT)$^{H+}$

E: −13361.70 kcal mol^{-1}

O	−0.177989	−0.219867	−0.333166
C	−0.211390	0.117023	1.075006
C	1.169518	−0.071211	1.690799
O	1.516175	−1.491187	1.787857
C	2.668572	−1.794046	0.969139
C	2.319980	0.584668	0.906138
C	2.891263	−0.561853	0.079073
O	3.266505	1.100549	1.920890
H	−1.095159	−0.228057	−0.658647
H	−0.922264	−0.525338	1.609231
H	−0.510504	1.165565	1.212679
H	1.138760	0.331270	2.708367
H	3.529846	−2.002813	1.606536
H	1.998449	1.434094	0.303339
H	2.311782	−0.634474	−0.843537
H	3.935746	−0.424414	−0.186083
N	2.425332	−3.039475	0.224330
C	3.218601	−4.156518	0.512662
O	4.102914	−4.171537	1.371014
N	2.937577	−5.256051	−0.279803
C	1.963669	−5.375824	−1.279252

O	1.857994	−6.439250	−1.913139
C	1.140906	−4.191316	−1.470312
C	0.035311	−4.230344	−2.489489
C	1.405264	−3.097192	−0.712418
H	3.532262	−6.067204	−0.120492
H	−0.676867	−5.033168	−2.262324
H	0.435621	−4.430110	−3.491114
H	−0.504098	−3.279336	−2.512666
H	0.809573	−2.192157	−0.784986
P	4.759766	1.610435	1.679494
O	4.798488	1.958982	0.116156
O	5.134275	2.681574	2.642153
O	5.709745	0.307682	1.749518
C	6.009528	−0.255120	3.076209
C	7.166004	−1.231302	2.964753
O	6.760199	−2.405535	2.217020
C	7.652167	−2.655154	1.103248
C	8.433343	−0.670696	2.280860
C	8.473326	−1.367624	0.918090
O	9.559422	−1.006310	3.169244
H	5.118871	−0.771819	3.445298
H	6.275411	0.557240	3.758485
H	7.421405	−1.536552	3.986738
H	8.284237	−3.518313	1.318676
H	8.412732	0.415445	2.186294
H	8.019923	−0.702399	0.181505
H	9.486022	−1.588212	0.587855
N	6.849856	−3.042711	−0.064804
C	6.781398	−4.428547	−0.443901
O	7.452169	−5.263842	0.184983
N	5.991252	−4.760602	−1.503480
C	5.263438	−3.834766	−2.138392
N	4.486704	−4.234413	−3.164815
C	5.258861	−2.459185	−1.734026
C	6.054301	−2.121302	−0.688219
H	4.511455	−5.200662	−3.467116
H	3.961338	−3.567792	−3.713909
H	4.644292	−1.721627	−2.235095
H	6.088376	−1.115230	−0.289470
P	11.117621	−0.839629	2.810055
O	11.221794	0.176214	1.574707
O	11.897716	−0.514006	4.035090
O	11.530185	−2.194612	2.056474
C	11.448222	−3.447493	2.826528
C	11.770475	−4.609440	1.913124
O	10.717123	−4.729113	0.919917
C	11.322002	−5.062788	−0.351655
C	13.118600	−4.517935	1.147213
C	12.698568	−4.384073	−0.326571
O	13.838528	−5.746807	1.416752
H	10.435119	−3.552435	3.225536
H	12.166106	−3.406572	3.651145
H	11.789401	−5.515360	2.534472
H	11.408473	−6.146077	−0.458450
H	13.713348	−3.661336	1.477407
H	12.619283	−3.325695	−0.589070
H	13.409060	−4.859734	−1.006555
H	14.719776	−5.668260	1.007562
N	10.411550	−4.622295	−1.410513
C	9.724843	−5.590006	−2.153502
O	9.871399	−6.806424	−2.003754
N	8.864132	−5.060272	−3.096764
C	8.586045	−3.711171	−3.356247
O	7.781325	−3.404990	−4.248786
C	9.300591	−2.771339	−2.505030
C	9.066418	−1.298223	−2.692720
C	10.165158	−3.273285	−1.588789
H	8.350650	−5.743954	−3.649655
H	9.343635	−0.988946	−3.707614
H	9.655700	−0.714536	−1.980879
H	8.006401	−1.051485	−2.562354
H	10.724641	−2.624314	−0.926961
H	11.555426	1.056232	1.836553
H	5.553511	2.522135	−0.144012

(TGA)$^{H+}$

E: −14067.00 kcal mol^{-1}

O	−0.211897	−0.362886	−0.276442
C	−0.186425	−0.003391	1.126120
C	1.233859	−0.106961	1.666937
O	1.655318	−1.503330	1.790700
C	2.784451	−1.784975	0.932373
C	2.305211	0.582957	0.801647
C	2.937055	−0.561724	0.012947
O	3.236924	1.234601	1.746156
H	−1.143348	−0.422186	−0.551850
H	−0.832878	−0.671294	1.708529
H	−0.531932	1.030901	1.263845
H	1.238408	0.328297	2.671592
H	3.674804	−1.961233	1.539137
H	1.896218	1.365066	0.161955
H	2.372320	−0.692836	−0.912335
H	3.974815	−0.375819	−0.251507
N	2.544534	−3.048553	0.213735
C	3.349435	−4.154707	0.509229
O	4.252703	−4.148327	1.347804
N	3.048894	−5.277848	−0.244272
C	2.015869	−5.443159	−1.177769
O	1.860237	−6.540573	−1.738489
C	1.191925	−4.262505	−1.383578
C	0.036592	−4.337740	−2.344555
C	1.488500	−3.141517	−0.678519
H	3.618044	−6.097965	−0.045482
H	−0.667878	−5.124374	−2.047720
H	0.385037	−4.582111	−3.355610
H	−0.498527	−3.384551	−2.381242
H	0.882800	−2.243642	−0.754990
P	4.648177	1.900950	1.400491
O	4.613841	2.118638	−0.185743
O	4.919054	3.088323	2.256223
O	5.735263	0.719501	1.542755
C	6.021577	0.216094	2.893623
C	7.124508	−0.824777	2.828829
O	6.666858	−1.994169	2.092109
C	7.578659	−2.264266	0.994980
C	8.433108	−0.368503	2.146060
C	8.303170	−0.942161	0.738827
O	9.529274	−0.946284	2.958912
H	5.113359	−0.239062	3.300120
H	6.333197	1.049070	3.531210
H	7.341706	−1.121364	3.861394
H	8.276698	−3.053157	1.285492
H	8.575592	0.712152	2.164657
H	7.667916	−0.261846	0.165716
H	9.245775	−1.058497	0.208727
N	6.846351	−2.804488	−0.128143
C	5.969925	−2.175116	−1.007096
N	5.450552	−3.010080	−1.888618
C	6.012875	−4.253151	−1.588106
C	5.853644	−5.533905	−2.201951
O	5.124407	−5.870391	−3.148107
N	6.702511	−6.501119	−1.578649
C	7.570768	−6.265467	−0.530841
N	8.344607	−7.292987	−0.081847
N	7.661179	−5.080763	0.062696

C	6.884836	−4.137071	−0.498719
H	5.777263	−1.114458	−0.950189
H	6.664790	−7.431397	−1.990085
H	8.475419	−8.091872	−0.692960
H	9.188444	−6.983311	0.404443
P	10.948386	−1.540847	2.520546
O	11.461642	−0.736577	1.227871
O	11.847870	−1.594372	3.704081
O	10.691906	−2.943403	1.784991
C	10.613561	−4.198459	2.555096
C	11.473944	−5.280413	1.914806
O	10.883018	−5.788849	0.683765
C	11.633953	−5.342837	−0.475894
C	12.902984	−4.853846	1.539816
C	12.786097	−4.469489	0.055132
O	13.754241	−6.008367	1.744090
H	9.566274	−4.509259	2.557120
H	10.955008	−4.017108	3.577179
H	11.503859	−6.116163	2.622844
H	11.985527	−6.232780	−1.002383
H	13.253147	−4.016111	2.152212
H	12.552256	−3.407876	−0.031267
H	13.713504	−4.658496	−0.489820
H	14.656443	−5.763316	1.468122
N	10.741410	−4.658353	−1.411178
C	10.294844	−3.342032	−1.379740
N	9.392690	−3.074642	−2.307817
C	9.216603	−4.278396	−2.988640
C	8.374454	−4.659963	−4.057941
N	7.519584	−3.800004	−4.666092
N	8.442117	−5.942892	−4.498692
C	9.290403	−6.796245	−3.892829
N	10.117527	−6.553552	−2.858825
C	10.044361	−5.275244	−2.446090
H	10.669396	−2.638735	−0.653202
H	6.802917	−4.195417	−5.265098
H	7.289554	−2.933368	−4.193696
H	9.300684	−7.811384	−4.286541
H	11.722612	0.185649	1.419069
H	5.176323	2.858135	−0.488526

(TGC)$^{H+}$

E: −13667.39 kcal mol^{-1}

O	−0.250374	−0.479702	−0.233722
C	−0.217334	−0.106971	1.165333
C	1.207690	−0.191093	1.698025
O	1.652484	−1.582602	1.802081
C	2.778454	−1.827459	0.926127
C	2.264376	0.520273	0.833295
C	2.865273	−0.605739	−0.000848
O	3.233415	1.112104	1.780826
H	−1.183560	−0.554610	−0.499399
H	−0.851473	−0.777384	1.758326
H	−0.573836	0.924413	1.297197
H	1.210434	0.233619	2.707141
H	3.685050	−1.959405	1.519921
H	1.852381	1.331301	0.232804
H	2.245557	−0.740503	−0.889562
H	3.881876	−0.406723	−0.327332
N	2.581182	−3.106106	0.225499
C	3.429090	−4.175909	0.535231
O	4.328038	−4.123399	1.376555
N	3.179753	−5.316434	−0.211150
C	2.152222	−5.534086	−1.141276
O	2.049400	−6.639580	−1.697837
C	1.273385	−4.394472	−1.350622
C	0.118032	−4.531209	−2.304508
C	1.524050	−3.254106	−0.658465
H	3.779810	−6.111854	−0.001792
H	−0.549855	−5.344160	−1.994213
H	0.471916	−4.772549	−3.314410
H	−0.458144	−3.602706	−2.349836
H	0.880458	−2.383532	−0.740233
P	4.599981	1.863288	1.427711
O	4.583119	2.031301	−0.165467
O	4.790488	3.092148	2.246251
O	5.756042	0.756617	1.615067
C	6.052511	0.298640	2.978888
C	7.171041	−0.729357	2.945478
O	6.748287	−1.912063	2.209989
C	7.609193	−2.116779	1.061956
C	8.490040	−0.271664	2.290139
C	8.369939	−0.802362	0.866493
O	9.566311	−0.903365	3.086923
H	5.153506	−0.161953	3.399170
H	6.349094	1.155496	3.591463
H	7.364078	−1.017738	3.984702
H	8.285314	−2.951127	1.268336
H	8.652867	0.804886	2.343000
H	7.769840	−0.086837	0.299796
H	9.322973	−0.929237	0.355832
N	6.826743	−2.540692	−0.081214
C	6.027437	−1.796615	−0.948666
N	5.483218	−2.539363	−1.894556
C	5.944932	−3.835941	−1.654020
C	5.755190	−5.055462	−2.375264
O	5.073439	−5.272428	−3.390591
N	6.502916	−6.117834	−1.780537
C	7.346757	−6.004241	−0.692294
N	8.055284	−7.096223	−0.297301
N	7.476582	−4.872332	−0.010970
C	6.780296	−3.848393	−0.531047
H	5.905082	−0.730365	−0.830563
H	6.448708	−7.007549	−2.272867
H	8.180160	−7.859139	−0.953212
H	8.867310	−6.872648	0.278991
P	11.018656	−1.397855	2.658314
O	11.425483	−0.408038	1.464638
O	11.925425	−1.474162	3.834673
O	10.842339	−2.796427	1.874285
C	10.679315	−4.049394	2.638913
C	11.333672	−5.211318	1.905929
O	10.581950	−5.567404	0.711471
C	11.392155	−5.383969	−0.481753
C	12.785664	−4.971071	1.447341
C	12.638887	−4.602770	−0.036799
O	13.497562	−6.220058	1.635569
H	9.607711	−4.233539	2.751736
H	11.142455	−3.930312	3.621904
H	11.302431	−6.067879	2.590382
H	11.642664	−6.361866	−0.891531
H	13.268240	−4.174175	2.023302
H	12.492805	−3.524851	−0.134521
H	13.518741	−4.882443	−0.620857
H	14.406322	−6.095073	1.305924
N	10.571370	−4.727978	−1.509668
C	10.084201	−5.496050	−2.618147
O	10.431536	−6.687324	−2.738963
N	9.242400	−4.885199	−3.497648
C	8.889689	−3.602060	−3.338681
N	8.060348	−3.059005	−4.254410
C	9.361869	−2.813809	−2.239630
C	10.178114	−3.427481	−1.346038
H	7.647462	−3.653446	−4.963626
H	7.657226	−2.143323	−4.106079

H	9.059055	−1.782110	−2.110468
H	10.538237	−2.932465	−0.453400
H	12.377331	−0.423714	1.243548
H	4.993707	2.865219	−0.466579

(TGG)$^{H+}$

E: −14231.24 kcal mol^{-1}

O	−0.212694	−0.440333	−0.297717
C	−0.172370	−0.038596	1.092885
C	1.254790	−0.115959	1.619714
O	1.689073	−1.504870	1.780803
C	2.811388	−1.802657	0.918788
C	2.312022	0.555500	0.723315
C	2.940493	−0.607717	−0.040402
O	3.248550	1.239061	1.640719
H	−1.147118	−0.513509	−0.559388
H	−0.807078	−0.692623	1.703289
H	−0.523354	0.997120	1.203805
H	1.266815	0.349307	2.610730
H	3.710630	−1.951118	1.519981
H	1.892094	1.317896	0.067080
H	2.359931	−0.771382	−0.950659
H	3.971947	−0.423382	−0.329584
N	2.576117	−3.090442	0.242985
C	3.405232	−4.174851	0.552342
O	4.324730	−4.130541	1.371400
N	3.108182	−5.323538	−0.163674
C	2.057386	−5.532055	−1.068294
O	1.910235	−6.646583	−1.597097
C	1.207416	−4.372398	−1.285983
C	0.031113	−4.495066	−2.215705
C	1.501728	−3.226084	−0.621386
H	3.694644	−6.128531	0.046253
H	−0.653337	−5.282649	−1.877167
H	0.359102	−4.766001	−3.226821
H	−0.520044	−3.551879	−2.268781
H	0.880519	−2.339930	−0.710797
P	4.667982	1.876971	1.280102
O	4.630454	2.060857	−0.310258
O	4.954315	3.079059	2.109916
O	5.741640	0.686449	1.447875
C	6.026102	0.214402	2.810337
C	7.142405	−0.813733	2.775503
O	6.708683	−2.003503	2.058691
C	7.621630	−2.273277	0.961894
C	8.452452	−0.355366	2.097940
C	8.341446	−0.949993	0.697705
O	9.546009	−0.906852	2.933149
H	5.120551	−0.243622	3.219546
H	6.322637	1.064023	3.432920
H	7.352069	−1.087020	3.816009
H	8.323188	−3.057395	1.255388
H	8.585061	0.726727	2.101653
H	7.709836	−0.280219	0.108253
H	9.289197	−1.071854	0.178728
N	6.891221	−2.824386	−0.157913
C	6.004112	−2.208587	−1.037353
N	5.478930	−3.055878	−1.902406
C	6.045636	−4.293643	−1.590632
C	5.890959	−5.581510	−2.189850
O	5.182146	−5.929122	−3.148670
N	6.721859	−6.545966	−1.537518
C	7.578380	−6.300198	−0.481368
N	8.328965	−7.329315	0.003534
N	7.681251	−5.103526	0.083000
C	6.920563	−4.163085	−0.504314
H	5.808990	−1.147703	−0.990837
H	6.681103	−7.483069	−1.933974
H	8.447607	−8.147073	−0.584789
H	9.181648	−7.015172	0.471758
P	10.958122	−1.539859	2.530475
O	11.505209	−0.777059	1.225854
O	11.839339	−1.577791	3.728400
O	10.691849	−2.953931	1.824744
C	10.579271	−4.192678	2.613367
C	11.411173	−5.303235	1.985794
O	10.839517	−5.768376	0.730131
C	11.634741	−5.323174	−0.399883
C	12.869581	−4.939479	1.662917
C	12.823121	−4.532603	0.179968
O	13.658230	−6.135561	1.881955
H	9.524395	−4.476805	2.618203
H	10.923952	−4.007351	3.633856
H	11.380554	−6.147806	2.683509
H	11.946263	−6.215750	−0.946248
H	13.238144	−4.125871	2.296826
H	12.664335	−3.457022	0.101643
H	13.753234	−4.775279	−0.338417
H	14.579366	−5.931379	1.637113
N	10.803654	−4.558187	−1.331758
C	10.512874	−3.192615	−1.333877
N	9.650230	−2.855195	−2.272116
C	9.336768	−4.047347	−2.922445
C	8.459525	−4.303362	−4.020973
O	7.768062	−3.508118	−4.680346
N	8.432190	−5.692302	−4.330421
C	9.158030	−6.677047	−3.694841
N	8.936377	−7.963335	−4.081584
N	9.995721	−6.422004	−2.694816
C	10.041353	−5.115521	−2.349316
H	10.968563	−2.513247	−0.632268
H	7.798045	−5.953212	−5.082849
H	8.525364	−8.147948	−4.989512
H	9.600307	−8.652957	−3.747575
H	11.764061	0.150301	1.393202
H	5.244491	2.746396	−0.638598

(TGT)$^{H+}$

E: −13926.11 kcal mol^{-1}

O	−0.249182	−0.352618	−0.195340
C	−0.186641	−0.001046	1.207722
C	1.237749	−0.154692	1.726178
O	1.627637	−1.565106	1.792983
C	2.734816	−1.829180	0.897471
C	2.319420	0.531415	0.871842
C	2.846451	−0.595652	−0.007971
O	3.324682	1.040368	1.833038
H	−1.187331	−0.381901	−0.452474
H	−0.843753	−0.651381	1.798105
H	−0.496819	1.042743	1.357526
H	1.264023	0.244660	2.745229
H	3.644226	−1.996624	1.477703
H	1.947245	1.386892	0.307965
H	2.185377	−0.682440	−0.872407
H	3.856050	−0.439005	−0.375488
N	2.492981	−3.090823	0.184222
C	3.309117	−4.188063	0.480055
O	4.220450	−4.167101	1.309735
N	3.014334	−5.316392	−0.267388
C	1.982457	−5.490969	−1.201857
O	1.844193	−6.587942	−1.766576
C	1.142447	−4.320606	−1.402029

C	−0.015849	−4.410174	−2.357788
C	1.429887	−3.195949	−0.699096
H	3.586128	−6.133651	−0.064432
H	−0.712997	−5.200645	−2.053853
H	0.331122	−4.657787	−3.368549
H	−0.558665	−3.461517	−2.397662
H	0.814858	−2.304303	−0.771222
P	4.791088	1.591232	1.526305
O	4.840065	1.830313	−0.057544
O	5.144589	2.739908	2.404045
O	5.774579	0.320046	1.670205
C	6.003269	−0.234556	3.011905
C	7.218144	−1.148340	2.999577
O	6.962052	−2.376247	2.264154
C	7.764715	−2.450325	1.064722
C	8.493411	−0.535796	2.388529
C	8.549523	−1.127821	0.977574
O	9.600708	−0.944471	3.268492
H	5.116855	−0.800367	3.312806
H	6.173442	0.586094	3.715506
H	7.408742	−1.423802	4.042359
H	8.418455	−3.324589	1.136542
H	8.476770	0.554761	2.378459
H	8.063046	−0.426290	0.299059
H	9.564451	−1.283779	0.616818
N	6.910289	−2.688591	−0.097440
C	6.103687	−1.766525	−0.766954
N	5.394445	−2.317255	−1.731233
C	5.732911	−3.668790	−1.704277
C	5.271036	−4.752905	−2.510896
O	4.457673	−4.746412	−3.448316
N	5.874138	−5.977313	−2.099339
C	6.791852	−6.124230	−1.076314
N	7.235689	−7.378513	−0.814245
N	7.220297	−5.101531	−0.341419
C	6.663732	−3.920936	−0.685663
H	6.088639	−0.724689	−0.488192
H	5.586316	−6.798346	−2.628260
H	7.100824	−8.119033	−1.491508
H	8.042964	−7.461826	−0.207608
P	11.166801	−0.880657	2.921668
O	11.339553	0.154395	1.710342
O	11.965176	−0.633375	4.152884
O	11.484154	−2.252282	2.148659
C	11.364850	−3.502653	2.918101
C	11.762928	−4.666053	2.037474
O	10.759973	−4.839690	0.996525
C	11.444289	−5.172036	−0.234328
C	13.139823	−4.534521	1.330160
C	12.776614	−4.416402	−0.159282
O	13.883339	−5.742469	1.629113
H	10.329105	−3.614998	3.252198
H	12.027668	−3.450912	3.786780
H	11.783745	−5.562882	2.671313
H	11.598276	−6.250971	−0.305449
H	13.694395	−3.660558	1.683464
H	12.643383	−3.363416	−0.423377
H	13.539694	−4.848739	−0.810545
H	14.775971	−5.640291	1.250916
N	10.565017	−4.814705	−1.348474
C	10.051234	−5.838412	−2.150044
O	10.316410	−7.033997	−1.997550
N	9.200589	−5.392559	−3.146409
C	8.819261	−4.072523	−3.432496
O	8.054468	−3.844240	−4.381018
C	9.379591	−3.071997	−2.535681
C	9.026723	−1.627973	−2.755191
C	10.210209	−3.492826	−1.549823
H	8.840793	−6.114953	−3.767614
H	9.370031	−1.293221	−3.741578
H	9.488899	−0.991920	−1.996049
H	7.941819	−1.483001	−2.727669
H	10.649489	−2.799660	−0.842751
H	11.855965	0.944762	1.961745
H	5.333756	2.636288	−0.305838

(TTA)$^{H+}$

E: −13760.81 kcal mol^{-1}

O	−0.181330	−0.179618	−0.162927
C	−0.145759	0.084242	1.261206
C	1.269031	−0.112152	1.790795
O	1.625331	−1.531689	1.839373
C	2.736869	−1.823115	0.966861
C	2.367927	0.575093	0.957612
C	2.967392	−0.557610	0.123705
O	3.314663	1.166168	1.923689
H	−1.113661	−0.185327	−0.442022
H	−0.819903	−0.594602	1.797701
H	−0.449947	1.120008	1.466709
H	1.296267	0.266794	2.817457
H	3.613884	−2.092778	1.558178
H	1.983058	1.388106	0.342222
H	2.421211	−0.610471	−0.819728
H	4.021496	−0.410528	−0.102648
N	2.423458	−3.022535	0.166041
C	3.169104	−4.187392	0.376370
O	4.094804	−4.280769	1.185468
N	2.792541	−5.242271	−0.438046
C	1.752121	−5.284321	−1.378066
O	1.541081	−6.326540	−2.018034
C	0.993782	−4.048370	−1.495718
C	−0.163921	−3.993393	−2.455079
C	1.354974	−2.997272	−0.717042
H	3.321338	−6.102205	−0.305868
H	−0.907348	−4.762722	−2.213506
H	0.172353	−4.182260	−3.482063
H	−0.649348	−3.014020	−2.421770
H	0.800967	−2.063157	−0.726144
P	4.720207	1.843843	1.545212
O	4.686917	2.151349	−0.026719
O	5.026108	2.975864	2.461466
O	5.786789	0.639357	1.554239
C	6.050159	−0.006360	2.848574
C	7.080145	−1.098428	2.658259
O	6.502679	−2.172461	1.869012
C	7.482313	−2.632015	0.909277
C	8.387047	−0.663867	1.943407
C	8.342285	−1.400507	0.603713
O	9.485596	−1.094643	2.823599
H	5.118813	−0.433708	3.231537
H	6.428322	0.741268	3.552067
H	7.338415	−1.478882	3.654340
H	8.077640	−3.448768	1.325703
H	8.467345	0.416550	1.826036
H	7.854992	−0.751827	−0.128484
H	9.322519	−1.665220	0.215452
N	6.769051	−3.202979	−0.234678
C	6.812016	−4.592371	−0.419719
O	7.474661	−5.358139	0.278447
N	6.049065	−5.034090	−1.488762
C	5.227420	−4.279527	−2.339986
O	4.592050	−4.838612	−3.242993
C	5.201430	−2.853135	−2.045175
C	4.348192	−1.961110	−2.902486
C	5.961513	−2.397715	−1.018507

H	6.099492	−6.033784	−1.675898
H	4.639884	−2.046895	−3.955761
H	4.443985	−0.915803	−2.600123
H	3.292807	−2.252277	−2.840257
H	5.974452	−1.349287	−0.747171
P	11.052314	−1.053967	2.484951
O	11.191748	−0.153479	1.166638
O	11.855089	−0.656939	3.673630
O	11.390326	−2.507356	1.889998
C	11.297325	−3.664629	2.795238
C	11.869685	−4.891405	2.107375
O	11.025084	−5.285498	0.989210
C	11.750090	−5.165936	−0.260852
C	13.292471	−4.729754	1.540594
C	13.052181	−4.414588	0.057384
O	13.968655	−5.998148	1.731293
H	10.243844	−3.830594	3.039370
H	11.859812	−3.451941	3.709579
H	11.857796	−5.703771	2.844199
H	11.936151	−6.171386	−0.648783
H	13.844872	−3.930332	2.045336
H	12.927624	−3.337264	−0.061956
H	13.877358	−4.742243	−0.578700
H	14.859385	−5.919294	1.343138
N	10.893395	−4.511914	−1.245361
C	10.683826	−3.153860	−1.453101
N	9.756236	−2.904537	−2.362907
C	9.319655	−4.161813	−2.777908
C	8.354218	−4.577398	−3.724894
N	7.614480	−3.708942	−4.445825
N	8.155640	−5.911747	−3.892146
C	8.885350	−6.775430	−3.158355
N	9.828542	−6.498249	−2.239813
C	10.008638	−5.173098	−2.090353
H	11.241514	−2.403336	−0.912548
H	6.943157	−4.055359	−5.119303
H	7.774122	−2.713367	−4.374962
H	8.682257	−7.830678	−3.333727
H	11.895469	0.520054	1.241102
H	4.685847	3.108501	−0.223213

(TTC)H+

E: −13361.46 kcal mol^{-1}

O	−0.203405	−0.186802	−0.111636
C	−0.165398	0.071488	1.313305
C	1.254564	−0.104910	1.835586
O	1.635381	−1.518906	1.866448
C	2.747633	−1.779540	0.984990
C	2.337918	0.609710	1.004812
C	2.943500	−0.503072	0.150573
O	3.286483	1.196715	1.972037
H	−1.136865	−0.211133	−0.385718
H	−0.825765	−0.620616	1.850021
H	−0.485353	1.101244	1.525190
H	1.279548	0.263274	2.866191
H	3.633695	−2.033029	1.570391
H	1.938878	1.428212	0.405783
H	2.382742	−0.555503	−0.784136
H	3.989810	−0.334354	−0.093433
N	2.459311	−2.979292	0.176090
C	3.222419	−4.132844	0.387703
O	4.133152	−4.218591	1.214333
N	2.883219	−5.184323	−0.447358
C	1.857800	−5.236508	−1.403343
O	1.679729	−6.274184	−2.060581
C	1.072483	−4.016766	−1.514638
C	−0.077838	−3.977997	−2.483526
C	1.400895	−2.967015	−0.719704
H	3.424617	−6.036234	−0.313468
H	−0.810202	−4.760234	−2.249687
H	0.269515	−4.158046	−3.508243
H	−0.580008	−3.007146	−2.450726
H	0.825637	−2.045923	−0.723694
P	4.699532	1.863805	1.608133
O	4.686694	2.141439	0.030485
O	4.997115	3.013119	2.505413
O	5.761493	0.654804	1.665952
C	6.000778	0.037775	2.979304
C	7.058171	−1.036840	2.838101
O	6.527122	−2.133465	2.044916
C	7.508472	−2.539322	1.064041
C	8.374996	−0.584617	2.158308
C	8.350234	−1.284339	0.798855
O	9.463490	−1.043691	3.036652
H	5.067370	−0.402599	3.341912
H	6.342432	0.805404	3.680069
H	7.289730	−1.400223	3.846580
H	8.117819	−3.362366	1.445361
H	8.457318	0.498700	2.071896
H	7.860095	−0.621488	0.081931
H	9.341737	−1.519761	0.420106
N	6.801033	−3.077651	−0.100712
C	6.861467	−4.459475	−0.335276
O	7.511648	−5.245197	0.351345
N	6.135591	−4.868015	−1.442415
C	5.341313	−4.088888	−2.294629
O	4.747816	−4.616751	−3.244703
C	5.288924	−2.674840	−1.946760
C	4.458933	−1.760721	−2.803694
C	6.009601	−2.251220	−0.878377
H	6.207257	−5.859344	−1.664881
H	4.781291	−1.818799	−3.849852
H	4.543774	−0.723328	−2.472714
H	3.402797	−2.055073	−2.781288
H	6.002327	−1.213629	−0.567552
P	11.033407	−0.973118	2.705879
O	11.171572	0.027755	1.462239
O	11.827172	−0.666208	3.926874
O	11.380664	−2.372449	1.997668
C	11.326910	−3.590075	2.826036
C	11.771064	−4.777529	1.994993
O	10.787018	−5.027183	0.954787
C	11.454382	−5.157234	−0.327130
C	13.144411	−4.628855	1.297329
C	12.781068	−4.399337	−0.176943
O	13.859366	−5.873555	1.506314
H	10.298203	−3.735252	3.169247
H	11.989938	−3.464619	3.687129
H	11.809601	−5.644175	2.668311
H	11.613820	−6.211981	−0.555729
H	13.721594	−3.795524	1.710267
H	12.652723	−3.328315	−0.353032
H	13.548301	−4.773630	−0.858907
H	14.734119	−5.785069	1.085164
N	10.546620	−4.659734	−1.364908
C	9.865260	−5.593975	−2.213658
O	10.088095	−6.813693	−2.085662
N	8.999151	−5.098846	−3.141716
C	8.762771	−3.782971	−3.230691
N	7.886865	−3.361919	−4.165911
C	9.383846	−2.834499	−2.353737
C	10.262693	−3.324997	−1.442691
H	7.465348	−4.030094	−4.799725
H	7.709947	−2.377169	−4.309661
H	9.167883	−1.774650	−2.414149

H	10.774515	−2.689725	−0.731242
H	11.841249	0.723584	1.609180
H	4.806842	3.086436	−0.186436

(TTG)$^{H+}$

E: −13924.83 kcal mol^{-1}

O	−0.175727	−0.226768	−0.169546
C	−0.136851	0.032816	1.255106
C	1.281195	−0.153792	1.778891
O	1.649819	−1.570398	1.821405
C	2.761936	−1.849152	0.945957
C	2.372153	0.545954	0.945343
C	2.979861	−0.579024	0.106386
O	3.313088	1.143917	1.912790
H	−1.108931	−0.238099	−0.445386
H	−0.803503	−0.652881	1.792254
H	−0.448330	1.065510	1.465252
H	1.308649	0.221607	2.806852
H	3.642358	−2.113867	1.534697
H	1.978880	1.357306	0.333029
H	2.431606	−0.633794	−0.835848
H	4.031925	−0.422533	−0.123216
N	2.456987	−3.047997	0.141061
C	3.213536	−4.207066	0.344069
O	4.141138	−4.296560	1.151366
N	2.844847	−5.261043	−0.475360
C	1.801928	−5.307834	−1.412566
O	1.598829	−6.348638	−2.057544
C	1.031928	−4.078364	−1.521698
C	−0.130137	−4.029190	−2.476101
C	1.385977	−3.027896	−0.738883
H	3.379974	−6.117694	−0.347215
H	−0.869040	−4.801545	−2.230448
H	0.202262	−4.217183	−3.504344
H	−0.619915	−3.052056	−2.440877
H	0.823578	−2.098733	−0.741981
P	4.724679	1.812775	1.543089
O	4.705782	2.122571	−0.028589
O	5.031510	2.941048	2.463409
O	5.782307	0.600861	1.556122
C	6.036913	−0.048552	2.850552
C	7.097878	−1.113579	2.672874
O	6.559180	−2.212240	1.885629
C	7.528362	−2.586206	0.875089
C	8.389942	−0.644940	1.956799
C	8.297467	−1.295698	0.578472
O	9.501486	−1.171167	2.765421
H	5.109179	−0.501710	3.212194
H	6.382577	0.703036	3.566624
H	7.359917	−1.481602	3.672040
H	8.189612	−3.369932	1.253560
H	8.487246	0.439750	1.914818
H	7.722847	−0.631103	−0.071526
H	9.261106	−1.476465	0.109893
N	6.815772	−3.183498	−0.253830
C	6.883832	−4.575139	−0.411471
O	7.549950	−5.317552	0.308118
N	6.140635	−5.050240	−1.480591
C	5.309603	−4.326811	−2.351576
O	4.699397	−4.916550	−3.252344
C	5.243336	−2.899108	−2.072900
C	4.370349	−2.038279	−2.942029
C	5.987817	−2.411404	−1.049610
H	6.185525	−6.056800	−1.626579
H	4.675028	−2.122443	−3.991567
H	4.434079	−0.988538	−2.645457
H	3.323190	−2.358776	−2.885349
H	5.972895	−1.359154	−0.794810
P	11.071113	−1.047265	2.473041
O	11.188673	−0.172739	1.135444
O	11.829308	−0.571661	3.663041
O	11.498972	−2.498868	1.936464
C	11.413988	−3.643493	2.856413
C	11.937686	−4.887399	2.159704
O	11.058336	−5.261030	1.061040
C	11.756052	−5.159245	−0.205946
C	13.352293	−4.767083	1.563175
C	13.091523	−4.453947	0.083779
O	13.999107	−6.051857	1.749320
H	10.366666	−3.788111	3.137614
H	12.011620	−3.432579	3.748875
H	11.919427	−5.697464	2.898910
H	11.897014	−6.167891	−0.603889
H	13.935666	−3.980000	2.052435
H	13.002761	−3.373979	−0.043409
H	13.891143	−4.815157	−0.566769
H	14.884710	−5.997522	1.345571
N	10.906472	−4.465626	−1.169428
C	10.725262	−3.092302	−1.332905
N	9.825194	−2.802832	−2.252761
C	9.376378	−4.036963	−2.720984
C	8.420886	−4.353354	−3.734731
O	7.742017	−3.589492	−4.442500
N	8.282537	−5.762786	−3.875641
C	8.976748	−6.720267	−3.162336
N	8.672184	−8.020718	−3.408377
N	9.873526	−6.411616	−2.229331
C	10.033734	−5.081499	−2.054318
H	11.278302	−2.372705	−0.747986
H	7.612572	−6.060987	−4.581856
H	8.197873	−8.275179	−4.266600
H	9.293375	−8.716954	−3.012488
H	11.929238	0.463908	1.162066
H	4.696837	3.079752	−0.224126

(TTT)$^{H+}$

E: −13621.18 kcal mol^{-1}

O	0.073231	−0.047526	−0.309754
C	−0.060046	0.168834	1.116037
C	1.270212	−0.099276	1.808800
O	1.569058	−1.531375	1.856598
C	2.767495	−1.842502	1.114981
C	2.485583	0.565955	1.137650
C	3.146221	−0.564415	0.345753
O	3.331433	1.084842	2.230336
H	−0.814825	0.007387	−0.704151
H	−0.819999	−0.498682	1.540573
H	−0.350035	1.209036	1.319741
H	1.183956	0.251128	2.842276
H	3.556829	−2.161034	1.797427
H	2.206620	1.410533	0.507967
H	2.723056	−0.572024	−0.660675
H	4.226390	−0.449970	0.265847
N	2.501525	−3.009536	0.250660
C	3.172040	−4.209095	0.509916
O	4.030567	−4.348817	1.384476
N	2.806544	−5.244100	−0.334847
C	1.842589	−5.233452	−1.354571
O	1.623531	−6.266363	−2.006406
C	1.169291	−3.956916	−1.534805
C	0.103020	−3.838484	−2.589296
C	1.517503	−2.926988	−0.722799

H	3.275124	−6.131174	−0.160741
H	−0.708315	−4.553101	−2.404469
H	0.510550	−4.063948	−3.582333
H	−0.317458	−2.828980	−2.604275
H	1.015491	−1.965278	−0.775194
P	4.725756	1.851949	2.010402
O	4.767278	2.329315	0.480536
O	4.925982	2.881984	3.065556
O	5.848211	0.705518	1.934910
C	6.141468	−0.050368	3.159377
C	7.087047	−1.180250	2.817203
O	6.403074	−2.120913	1.959392
C	7.401032	−2.805710	1.175145
C	8.391638	−0.773154	2.075863
C	8.487789	−1.752827	0.894045
O	9.477994	−0.883508	3.059566
H	5.210621	−0.453205	3.569365
H	6.605917	0.617898	3.889967
H	7.371708	−1.672621	3.757880
H	7.801197	−3.668708	1.712439
H	8.365808	0.262696	1.738227
H	8.277875	−1.220120	−0.035223
H	9.467985	−2.218838	0.812931
N	6.740308	−3.336627	−0.019393
C	6.730791	−4.720179	−0.231548
O	7.326107	−5.529878	0.478018
N	6.004560	−5.102963	−1.349109
C	5.269556	−4.290931	−2.230271
O	4.644950	−4.802908	−3.166391
C	5.320321	−2.867098	−1.922553
C	4.584111	−1.912702	−2.818663
C	6.043053	−2.471753	−0.846179
H	5.990296	−6.104383	−1.534573
H	4.957901	−1.990091	−3.846240
H	4.706729	−0.881243	−2.478945
H	3.514437	−2.151313	−2.846056
H	6.110693	−1.429335	−0.558829
P	11.036892	−0.682472	2.709577
O	11.123020	0.256529	1.413497
O	11.777282	−0.255945	3.927157
O	11.519335	−2.052332	2.030048
C	11.430515	−3.294761	2.813319
C	11.733566	−4.464102	1.901928
O	10.673607	−4.576244	0.911910
C	11.277558	−4.975585	−0.342844
C	13.079143	−4.387473	1.126731
C	12.651644	−4.294100	−0.349172
O	13.805632	−5.604535	1.426014
H	10.420992	−3.387246	3.224554
H	12.156987	−3.254601	3.630156
H	11.747153	−5.369250	2.524341
H	11.368343	−6.062898	−0.390890
H	13.670978	−3.519385	1.430634
H	12.567322	−3.243300	−0.639411
H	13.359557	−4.787092	−1.019348
H	14.689175	−5.528329	1.021362
N	10.360764	−4.598067	−1.420909
C	9.690040	−5.612983	−2.116506
O	9.858605	−6.817440	−1.912260
N	8.804211	−5.141780	−3.070133
C	8.512140	−3.808540	−3.402522
O	7.693796	−3.560292	−4.298866
C	9.226251	−2.818952	−2.609895
C	8.975390	−1.360084	−2.872743
C	10.106587	−3.262331	−1.678291
H	8.328130	−5.859845	−3.613485
H	9.249644	−1.098362	−3.901737
H	9.556910	−0.733880	−2.190795
H	7.912057	−1.120197	−2.756229
H	10.671311	−2.570301	−1.066711
H	11.315795	1.189236	1.632071
H	4.612529	3.288407	0.377199
